# Global, Regional, and National Prevalence, Incidence, and Disability-Adjusted Life Years for Oral Conditions for 195 Countries, 1990–2015: A Systematic Analysis for the Global Burden of Diseases, Injuries, and Risk Factors

**DOI:** 10.1177/0022034517693566

**Published:** 2017-02-01

**Authors:** N.J. Kassebaum, A.G.C. Smith, E. Bernabé, T.D. Fleming, A.E. Reynolds, T. Vos, C.J.L. Murray, W. Marcenes, GBD 2015 Oral Health Collaborators, G.Y. Abyu

**Affiliations:** 1 Department of Anesthesiology and Pain Medicine, University of Washington, Seattle, WA, USA; 2 Institute for Health Metrics and Evaluation, University of Washington, Seattle, WA, USA; 3 Division of Population and Patient Health, King’s College London Dental Institute, London, UK; * Collaborators are listed in the Contributing Authors section at the end of this article.

**Keywords:** caries, periodontal diseases, tooth loss, dental public health, epidemiology, global health

## Abstract

The Global Burden of Disease 2015 study aims to use all available data of sufficient quality to generate reliable and valid prevalence, incidence, and disability-adjusted life year (DALY) estimates of oral conditions for the period of 1990 to 2015. Since death as a direct result of oral diseases is rare, DALY estimates were based on years lived with disability, which are estimated only on those persons with unmet need for dental care. We used our data to assess progress toward the Federation Dental International, World Health Organization, and International Association for Dental Research’s oral health goals of reducing the level of oral diseases and minimizing their impact by 2020. Oral health has not improved in the last 25 y, and oral conditions remained a major public health challenge all over the world in 2015. Due to demographic changes, including population growth and aging, the cumulative burden of oral conditions dramatically increased between 1990 and 2015. The number of people with untreated oral conditions rose from 2.5 billion in 1990 to 3.5 billion in 2015, with a 64% increase in DALYs due to oral conditions throughout the world. Clearly, oral diseases are highly prevalent in the globe, posing a very serious public health challenge to policy makers. Greater efforts and potentially different approaches are needed if the oral health goal of reducing the level of oral diseases and minimizing their impact is to be achieved by 2020. Despite some challenges with current measurement methodologies for oral diseases, measurable specific oral health goals should be developed to advance global public health.

## Introduction

Governments and nongovernmental agencies have made great efforts to improve oral health in recent decades. Subsequent World Health Organization (WHO) findings suggest that prevalence of caries experience has declined in many locations in the world. The largest decline—a 90% reduction in the number of decayed, missing, and filled (DMF) teeth for 12-y-olds—occurred in early 1970s to mid-1990s in the United States and Western and Nordic European high-income countries. The decline was less obvious in low-income countries (Lagerweij and van Loveren 2015). Trends in periodontal health and tooth loss are less well documented than trends in dental caries. Available evidence suggests that the prevalence of periodontal disease (Dye et al. 2007; Bergström 2014) and tooth loss (Sanders et al. 2004; Slade et al. 2014; Bernabe and Sheiham 2014b) has declined in selected high-income countries, but there are recent suggestions of a higher prevalence of periodontitis in the adult US population than previously reported (Thornton-Evans et al. 2013; Eke et al. 2015), coincident with changes in examination criteria from partial- to full-mouth assessment. Also, although the lifetime prevalence of dental caries experience in children, as measured by the DMF index, may have declined in the last 40 y in many high-income countries (Marthaler 2004; Bernabe and Sheiham 2014a), information is scarce on the population-level epidemiology of untreated dental caries. Since treated diseases do not cause burden, it is more relevant to assess the decayed component of the DMF index rather than a composite measure of caries experience. Furthermore, individuals are susceptible to caries throughout life (Thomson 2004; Griffin et al. 2005; Broadbent et al. 2013), and a current assumption that caries have declined needs to be reviewed.

Given the prominent role attached to quantification of disease burden for health research and policy nationally and globally (GBD 2010 Country Collaboration 2013; Murray et al. 2013; Gilmour et al. 2014; Yu et al. 2014), up-to-date comparable estimates reflecting the latest evidence for descriptive epidemiology constitute an essential responsibility. For this reason, the Global Burden of Disease (GBD) study provides information on disease prevalence, incidence, and severity by age, sex, geography, and time. The aim of this component of the GBD 2015 study was to use all available data of sufficient quality to generate reliable estimates of prevalence, incidence, years lived with disability (YLDs), and disability-adjusted life years (DALYs) for oral conditions spanning the period of 1990 to 2015. This article reports on the global burden of untreated dental caries, severe chronic periodontitis (SCP), and total tooth loss. This report supersedes our previous GBD report (Marcenes et al. 2013).

## Methods

The GBD 2015 study included untreated caries, SCP, total tooth loss, and other oral disorders that encompass a variety of mouth disorders and malformations but not mouth cancer. Detailed methods for each component of the GBD study are described elsewhere (GBD 2015 DALYs and HALE Collaborators 2016; GBD 2015 Disease and Injury Incidence and Prevalence Collaborators 2016), including a complete list of *International Classification of Diseases* (ninth and tenth revisions) codes assigned to each cause. We provide only a brief description here, with emphasis on oral conditions; further details on the oral conditions models are available in Appendix 1, available online. The present report complies with the STROBE (Strengthening the Reporting of Observational Studies; Vandenbroucke et al. 2007) and GATHER (Guidelines for Accurate and Transparent Health Estimates Reporting; Stevens et al. 2016) statements.

### Data Input

Oral condition data on prevalence and incidence to inform models came from 3 updated independent systematic reviews of observational studies on untreated dental caries (Kassebaum et al. 2015), SCP (Kassebaum et al. 2014a), and total tooth loss (Kassebaum et al. 2014b). Full case definitions and inclusion/exclusion criteria for literature review and definition of disability associated with oral conditions were consistent with previous analyses (Marcenes et al. 2013) and are presented in Appendix 1. Case definitions followed the WHO definition of caries, which includes “a lesion in a pit or fissure, or on a smooth tooth surface, has an unmistakable cavity, undermined enamel, or a detectably softened floor or wall (coronal caries), or feel soft or leathery to probing (root caries)”; “a gingival pocket depth equal or more than 6 mm, or Community Periodontal Index of Treatment Needs (CPITN) also referred as Community Periodontal Index (CPI) score of 4, or a clinical attachment loss (CAL) more than 6 mm” for periodontal disease; and “complete loss of natural teeth” for total tooth loss. Clinical examination was required for diagnosis of caries and periodontal disease, while either examination or self-report data were included for tooth loss. Surveys were excluded if they reported on nonrepresentative samples of the general populations or representative samples of specific high-risk groups, such as people with chronic illnesses, disabilities, or emotional or behavioral health problems. The GBD 2015 study analyzed 195 countries and territories, along with subnational locations in Brazil, China, India, Japan, Kenya, Mexico, Saudi Arabia, South Africa, Sweden, the United Kingdom, and the United States. Data availability for main oral conditions is presented in Appendix 2. A full set of input sources for each condition is available on the Global Health Data Exchange (http://ghdx.healthdata.org).

### Data Analysis

Internally consistent estimates of prevalence, incidence, and remission were generated with DisMod-MR 2.1, a Bayesian meta-regression framework developed for GBD 2010 (Flaxman et al. 2014). DisMod-MR 2.1 consolidated code in a single language, Python, which made the code more transparent, computationally efficient, and better able to ensure internal consistency at the subnational level, even given sparse data. The entire time series from 1990 to 2015 was reestimated via the same methods to facilitate direct calculation of trends. DisMod-MR 2.1 details are provided in Appendix 1.

YLDs were calculated as prevalence of each cause-specific sequela—in each age group, sex, geography, and year—times a corresponding GBD disability weight derived from a population survey of >60,000 respondents (Salomon et al. 2012; Salomon et al. 2015). Their empirical basis was thus derived from judgments of the general public about health severity rather than researchers themselves or health professionals. The GBD study definition of disability associated with untreated symptomatic caries was “a toothache, which causes some difficulty eating.” Untreated caries was considered asymptomatic, mild, and severe. We approximated those with mild disability to have periodic pain lasting 1 h per day for the duration of the episode of caries. Those with severe symptoms were modeled as having 2 phases: an “initial” phase with periodic pain and a “terminal” phase with constant pain, the length of which was determined by performing log-normal distribution of symptom duration from casualty ward studies (Kassebaum et al. 2015). We estimated the distribution of sequelae for caries by completing meta-analyses on the proportion symptomatic and the duration of symptoms (details are presented in Appendix 1). The disability definitions of SCP and total tooth loss were “bad breath, a bad taste in the mouth, and gums that bleed a little from time to time, but this does not interfere with daily activities” and “great difficulty in eating meat, fruits, and vegetables,” respectively. Our case definition of SCP included only severe disease, so all cases were assumed to be symptomatic. Total tooth loss definition of disability was “great difficulty in eating meat, fruits, and vegetables”; a meta-analysis of the proportion of persons with this level of disability was performed for GBD 2010 (Kassebaum et al. 2014b). A microsimulation framework was used to estimate the occurrence of comorbidity in each age group, sex, geography, and year; YLDs for each comorbid condition were then adjusted proportionally.

Since death as a direct result of oral diseases is rare, we assumed no mortality for any of them. DALY estimates, which are computed as the sum of years of life lost and YLDs, were thus based on YLDs only. One DALY can be interpreted as a year of “healthy life” lost due to either premature mortality or disability and the sum of DALYs as the gap between the population’s current health status and an ideal situation where the entire population lives to an advanced age, free of disease (GBD 2015 DALYs and HALE Collaborators 2016).

Uncertainty from all data inputs into the calculations of DALYs were propagated through Monte Carlo simulation techniques by taking 1,000 draws for each age, sex, country, year, and cause. Aggregations were made at the level of the 1,000 draws for all estimates. The 95% uncertainty interval (UI) around each quantity of interest was computed as the ordinal 25th and 975th draws of the quantity of interest.

We report total cause-specific DALYs and DALY rates (per 100,000 population) in addition to prevalence and incidence estimates for each geography in 1990 and 2015. Age standardization accounts for differences in population size and age structure (GBD 2015 DALYs and HALE Collaborators 2016). Interactive data visualizations and downloads are available online (http://vizhub.healthdata.org/gbd-compare).

## Results

Oral conditions remained highly prevalent in 2015 (Table 1). Nearly half of the world population suffered disability from oral conditions (age-standardized prevalence: 48.0%). Untreated caries in permanent teeth was the most prevalent condition in all of GBD 2015 (age-standardized prevalence: 34.1%), affecting 2.5 billion people worldwide (95% UI: 2.4 to 2.7 billion). The age-standardized prevalence rates of untreated caries in deciduous teeth, SCP, and total tooth loss were 7.8% (573 million; 95% UI: 475 to 687), 7.4% (538 million; 95% UI: 465 to 626), and 4.1% (276 million; 95% UI: 264 to 288), respectively. The prevalence of other oral disorders was 1.8%. The number of incident cases of caries in permanent and in deciduous teeth, SCP and total tooth loss in 2015 were 616 million worldwide (95% UI: 577 to 656), 126 (95% UI: 94 to 167), 6 (95% UI: 5.0 to 6.6), and 3 (95% UI: 3 to 3), respectively. Sex differences were not significant in 2015 at the global level. Total tooth loss remained the leading cause of DALYs due to oral conditions, contributing 7.6 million (95% UI: 5.1 to 10.5). SCP and untreated caries in permanent and deciduous teeth accounted for 3.5 million DALYs (95% UI: 1.4 to 7.2), 1.7 (95% UI: 0.8 to 3.3), and 0.1 (95% UI: 0.1 to 0.3), respectively. Age-standardized DALY rates in 2015 were 113, 49, 24, 2, and 53 per 100,000 person-years for total tooth loss, SCP, untreated caries in permanent, deciduous teeth, and other oral conditions, respectively.

**Table 1.** Change Rates in Oral Conditions from 1990 to 2015.

	1990		2015	
	*n*^a^	95% UI	*n*^a^	95% UI
No. of prevalent cases, millions				
Untreated caries in permanent teeth	1,739	1,623 to 1,845	2,521	2,361 to 2,680
Untreated caries in deciduous teeth	555	469 to 655	573	475 to 687
Severe periodontitis	307	267 to 357	538	465 to 626
Total tooth loss	157	151 to 164	276	264 to 288
Other oral conditions	89	85 to 93	134	128 to 140
All oral conditions	2,513	2,472 to 2,551	3,522	3,467 to 3,575
Prevalence, %				
Untreated caries in permanent teeth	34.3	32.2 to 36.2	34.1	32.0 to 36.2
Untreated caries in deciduous teeth	8.2	6.9 to 9.7	7.8	6.4 to 9.3
Severe periodontitis	7.4	6.4 to 8.5	7.4	6.4 to 8.6
Total tooth loss	4.3	4.1 to 4.5	4.1	3.9 to 4.3
Other oral conditions	1.8	1.7 to 1.9	1.8	1.7 to 1.9
All oral conditions	48.4	47.6 to 49.0	48.0	47.3 to 48.7
No. of incident cases, millions				
Untreated caries in permanent teeth	627	589 to 665	616	577 to 656
Untreated caries in deciduous teeth	129	98 to 169	126	94 to 167
Severe periodontitis	6	5 to 7	6	5 to 6.6
Total tooth loss	3	3 to 3	3	3 to 3
Other oral conditions	Not estimated		Not estimated	
All oral conditions	764	713 to 820	750	700 to 808
DALYs, thousands				
Untreated caries in permanent teeth	1,239	551 to 2,361	1,743	777 to 3,315
Untreated caries in deciduous teeth	144	62 to 285	147	63 to 292
Severe periodontitis	2,010	780 to 4,174	3,518	1,357 to 7,247
Total tooth loss	4,334	2,898 to 5,985	7,625	5,088 to 10,540
Other oral conditions	2,609	1,616 to 3,923	3,916	2,421 to 5,886
All oral conditions	10,342	6,228 to 15,800	16,949	10,278 to 26,002
Age-standardized DALY rates, per 1,000 person-years				
Untreated caries in permanent teeth	0.25	0.11 to 0.47	0.24	0.11 to 0.45
Untreated caries in deciduous teeth	0.02	0.01 to 0.04	0.02	0.01 to 0.04
Severe periodontitis	0.48	0.19 to 0.99	0.49	0.19 to 1.00
Total tooth loss	1.17	0.79 to 1.62	1.13	0.76 to 1.57
Other oral conditions	0.53	0.33 to 0.79	0.53	0.33 to 0.79
All oral conditions	2.45	1.50 to 3.73	2.41	1.47 to 3.67

Changes in number of cases, age-standardized prevalence and incidence, DALYs, and age-standardized DALY rates in 1990 and 2015 for untreated caries, severe periodontitis, total tooth loss, other oral conditions, and all oral conditions combined globally.

DALY, disability-adjusted life year; UI, uncertainty interval.

a Values are presented in *n* unless noted otherwise.

**Figure 1.**
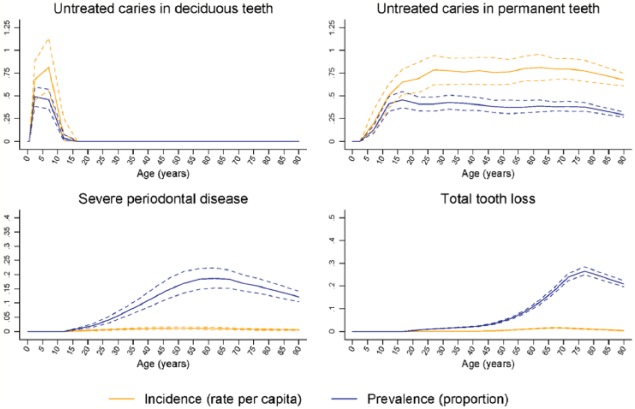
Global age patterns of prevalence and incidence of untreated caries in deciduous teeth, untreated caries in permanent teeth, severe periodontitis, and total tooth loss in 2015 for both sexes combined.

**Figure 2.**
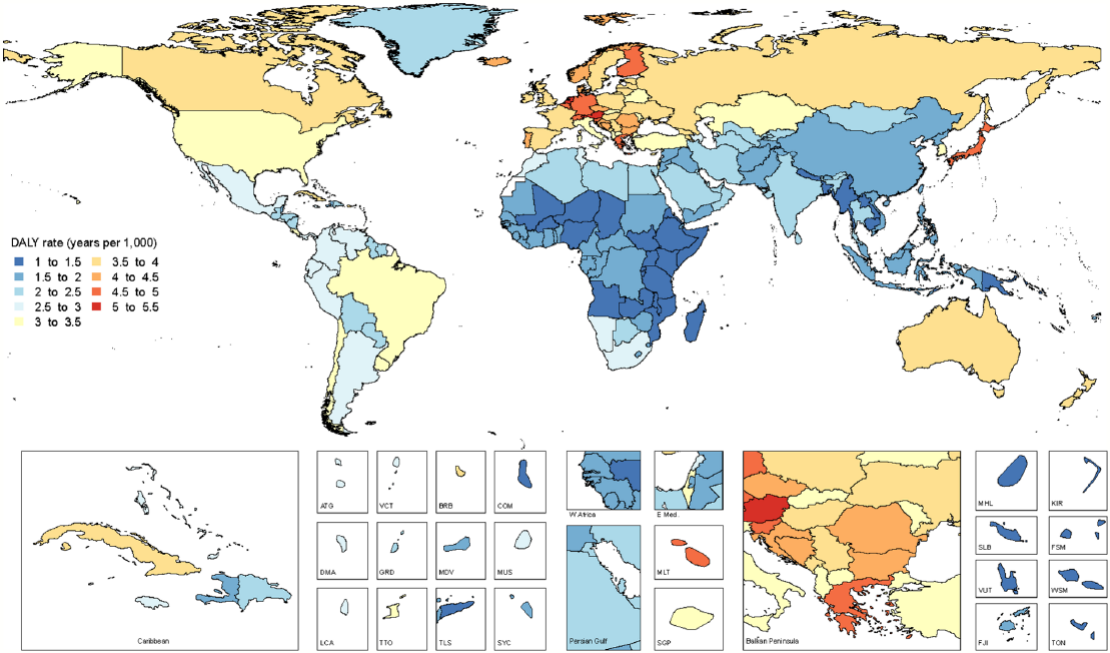
World map of age-standardized disability-adjusted life year (DALY) rates (per 1,000 population) for all oral conditions combined in 2015, both sexes.

While 2015 age-standardized prevalence rates were comparable to 1990 estimates, the number of people with oral conditions increased by 40% in this period. Total DALYs due to oral conditions increased by 64% from 1990 to 16.9 million (95% UI: 10.3 to 26.0). Increases were mainly due to population growth and aging, offsetting 0.9% and 0.4% decreases due to changes in age-specific prevalence and DALY rates, respectively (Table 2). Global age patterns in the prevalence and incidence of oral conditions in 2015 are shown in Figures 1 and 2. The prevalence of total tooth loss peaked at 75 to 79 y, while that of severe periodontal disease peaked nearly 2 decades earlier. Untreated deciduous caries peaked in the 1- to 4-y-old age group globally, while that of permanent caries was highest in the 15- to 19-y-old group, decreasing gradually with increasing age.

**Table 2.** Changes in Global Prevalence and DALYs between 1990 and 2015 Decomposed into Changes due to Population Growth, Aging, and Change in Disease Rates.

	Percentage Change (95% UI), 1990 to 2015	
	Prevalence	DALYs
Total percentage change		
Untreated caries in permanent teeth	44.96 (43.23 to 46.85)	40.62 (38.17 to 43)
Untreated caries in deciduous teeth	3.2 (0.78 to 5.3)	2.08 (−0.46 to 4.45)
Severe periodontitis	74.97 (72.72 to 77.33)	74.97 (72.68 to 77.35)
Total tooth loss	75.77 (75.25 to 76.3)	75.67 (75.1 to 76.25)
Other oral conditions	50.12 (48.85 to 51.33)	50.12 (48.85 to 51.33)
All oral conditions	40.15 (39.31 to 40.96)	63.94 (62.06 to 65.75)
Percentage change due to population growth		
Untreated caries in permanent teeth	38.9 (38.55 to 39.24)	35.33 (33.75 to 36.92)
Untreated caries in deciduous teeth	39.59 (38.63 to 40.65)	37.58 (36.13 to 39.03)
Severe periodontitis	40.88 (40.12 to 41.86)	40.85 (40.08 to 41.84)
Total tooth loss	33.37 (33.06 to 33.68)	33.38 (33.08 to 33.71)
Other oral conditions	38.41 (38.24 to 38.59)	38.41 (38.24 to 38.59)
All oral conditions	39.13 (38.97 to 39.28)	36.35 (35.69 to 37.11)
Percentage change due to population aging		
Untreated caries in permanent teeth	6.41 (4.74 to 8.18)	5.37 (3.82 to 7.14)
Untreated caries in deciduous teeth	−33.22 (−33.8 to −32.58)	−32.85 (−33.43 to −32.22)
Severe periodontitis	35.42 (33.48 to 37.55)	35.18 (33.22 to 37.38)
Total tooth loss	43.18 (42.62 to 43.72)	42.83 (42.29 to 43.37)
Other oral conditions	11.53 (10.33 to 12.68)	11.53 (10.33 to 12.68)
All oral conditions	1.91 (1.46 to 2.38)	28.02 (25.87 to 30.09)
Percentage change due to change in disease rate		
Untreated caries in permanent teeth	−0.4 (−1.01 to 0.31)	−0.1 (−0.71 to 0.49)
Untreated caries in deciduous teeth	−3.2 (−4.49 to −1.66)	−2.7 (−4.26 to −0.96)
Severe periodontitis	−1.3 (−1.97 to −0.7)	−1.1 (−1.73 to −0.43)
Total tooth loss	−0.8 (−0.94 to −0.63)	−0.5 (−0.8 to −0.29)
Other oral conditions	0.2 (−0.19 to 0.57)	0.2 (−0.19 to 0.57)
All oral conditions	−0.9 (−1.52 to −0.25)	−0.4 (−0.65 to −0.22)

DALY, disability-adjusted life year; UI, uncertainty interval.

**Figure 3.**
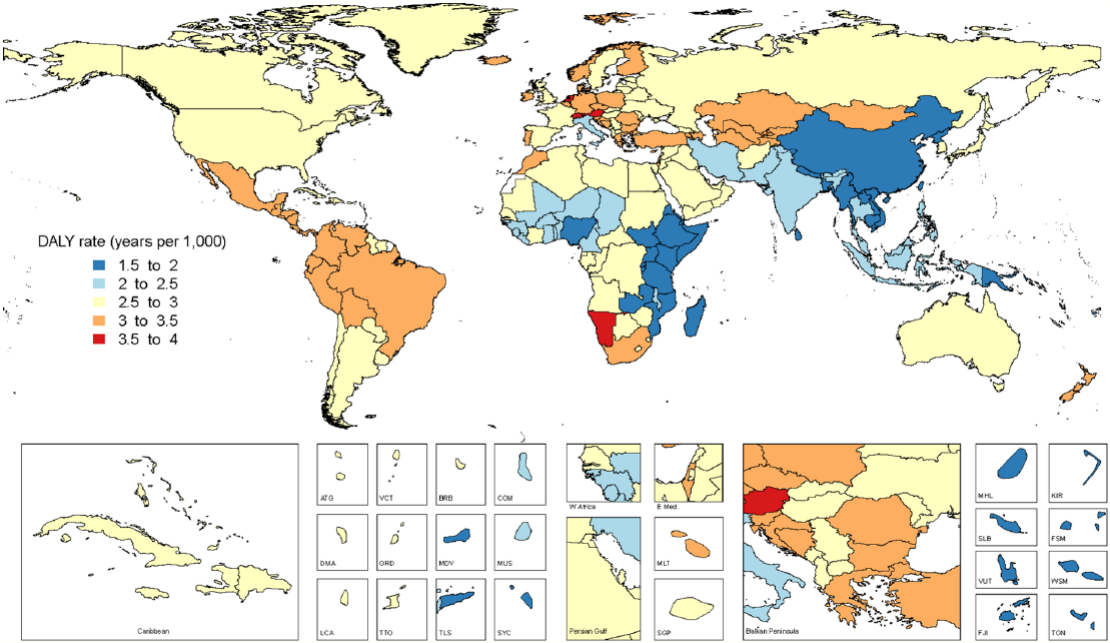
World map of disability-adjusted life year (DALY) for all oral conditions combined in 2015, both sexes.

Age-standardized DALY rates due to all oral conditions combined are mapped in Figure 3. Maps of all-age and age-standardized DALY rates in 2015 for the oral conditions are shown in Appendix 2, Figures 1–12. The highest prevalence (7.4%) and incidence (5.9 million new cases) of total tooth loss was observed in Tropical Latin America. The highest prevalence of caries in permanent teeth was observed in Andean Latin America (54.9%), while SCP (10.5%) was most prevalent in West Sub-Saharan Africa. Of note, Tropical Latin America was the only region with significantly higher prevalence and incidence of untreated caries in permanent teeth, severe periodontal disease, and total tooth loss, as compared with global averages in 2015.

Prevalence, incidence, and DALYs for each oral condition by country and age in 1990 and 2015 are shown in Appendix 2, Tables 1–5; there was an increase in prevalence and DALYs from oral conditions in 19 of the 21 regions. DALY increases ranged from 15.8% for Eastern Europe to 114.5% for Central Sub-Saharan Africa. An >100% increase in DALYs due to oral conditions was observed in 4 regions in Africa and 3 regions in South America between 1990 and 2015. Total tooth loss was the leading cause of DALYs associated with oral conditions in 19 of 21 regions. The only exceptions were Western and Eastern Sub-Saharan Africa, where SCP was the leading cause of oral DALYs.

## Discussion

In 2003, the World Dental Federation, WHO, and International Association for Dental Research set 2 general oral health goals for the year 2020 (Hobdell et al. 2003). The first of those goals was “to minimise the impact of diseases of oral and craniofacial origin on health and psychosocial development, giving emphasis to promoting oral health and reducing oral disease amongst populations with the greatest burden of such conditions and diseases” (Hobdell et al. 2003). The GBD 2015 study provides robust data to measure the progress toward achieving this general goal. We used 1) DALY estimates to assess whether the burden of oral conditions was minimized and 2) prevalence rates to assess whether oral conditions have reduced over time. Our data analysis demonstrated that the burden of oral conditions (as measured by age-standardized DALY rates) and the prevalence of oral conditions remained relatively stable between 1990 and 2015. Overall, oral health has not improved during the last 25 y, which suggests that greater efforts and maybe a different strategy are needed if this goal is to be achieved by 2020.

The inclusion of dental care as part of universal health coverage initiatives has been proposed to address this challenge (Hosseinpoor et al. 2012; Masood et al. 2015; Mathur et al. 2015). The number of people with untreated oral conditions and the DALY estimates are relevant measures to identify the overall population with unmet normative demand for dental care. The number of people with untreated oral conditions reached 3.5 billion in 2015; untreated caries in permanent teeth affected 2.5 billion people; untreated caries in deciduous teeth affected 573 million children; severe periodontal disease affected 538 million people; and total tooth loss affected 276 million people worldwide. These numbers are likely to continue increasing as many populations continue growing and aging. In 2010, the direct treatment costs due to oral conditions worldwide were estimated at US$298 billion worldwide, corresponding to an average of 4.6% of global health expenditure (Listl et al. 2015).

At first glance, these figures suggest that universal coverage for oral health care based on conventional dental care may be too expensive to tackle the high prevalence of oral conditions, and palliative measures have limited value to minimize the impact of oral conditions among populations. The value of the GBD study is thus in identifying which populations and countries have the highest population with unmet normative demand for dental care. Minimal-intervention dentistry may help address this public health challenge—in particular, where extraction would be the norm to alleviate dental pain and mouth infection. Universal health coverage also has the potential to widen social inequalities in oral health because services may be underused among lower socioeconomic groups (Veugelers and Yip 2003). Therefore, it is imperative to adopt proportionate universalism (Marmot 2010) in conjunction with universal health coverage. A primary focus on prevention of oral diseases—including implementation of population-level interventions that tackle the social determinants of oral health over the life course, along with targeted treatment-focused interventions—may be the way forward to reduce the high prevalence of oral diseases and avoid a major financial burden in addressing current overall population with unmet normative demand for dental care.

Oral health goals and policies development may be more effective and efficient if based on up-to-date descriptive epidemiology of the best quality. The GBD study provides an opportunity to independently monitor oral health goals and specific objectives across all geographic areas in the world and over time in a comparable manner. The DALY metric provides a standardized measure to compare the effects of all fatal (i.e., cancer, circulatory diseases) and nonfatal (i.e., oral conditions) diseases, injuries, and risk factors on population health to facilitate the identification of priorities.

Some methodological issues need to be mentioned. First, although the data representativeness index—which is the fraction of countries for which any incidence, prevalence, remission, or excess mortality data were available—increased over that represented by the GBD 2010 study (Marcenes et al. 2013), data were still scarce in some geographies (GBD 2015 Disease and Injury Incidence and Prevalence Collaborators 2016). Second, the level of aggregation of causes creates some challenges. We do recognize that the clinical considerations for root and coronal caries differ. Despite some differences, there is no evidence that policy and preventive measures are categorically different between coronal and root caries. Coronal and root caries are caused by high sugar consumption, which tends to be related to socioeconomic position. Because of this and because a large proportion of data sources do not make the distinction (instead stating that both have been combined), we opted for including both in the same model. This maximizes the geographic and temporal coverage of the data set, and it seems to be the best representation of contemporary data on caries. We will continue revising this issue in future GBD iterations. The major challenge remained inherent to the availability and reporting of oral conditions (Marcenes et al. 2013; Kassebaum et al. 2014a, 2014b, 2015). As incidence data are scarce in the dental literature, we extrapolated incidence data from 2 types of studies. First, for longitudinal or cohort studies, we calculated the disease increment over successive ages and time periods as the difference in the level of disease between each time point. Narrow age and time intervals were preferred, and most studies adopted a ≤3-y follow-up interval. We did not extrapolate incidence data if the age or time interval was >10 y. Second, if a study performed only a single cross-sectional examination but reported data in age intervals ≤3 y, we extrapolated incidence data in the same manner. This is described in detail in Appendix 1. Third, while this study reports on levels and trends in oral diseases, it does not estimate the contribution of specific risk factors, which may aid in interpreting geographic and temporal patterns of burden. Finally, we assumed that there is no mortality burden from oral conditions. While this assumption reflects the reality that death is rare from oral diseases, life-threatening events do occur, and avoiding them requires timely access to appropriate care.

Policy makers must acknowledge that oral conditions pose a very serious public health problem and that oral health goals must be included in the health agenda. International oral health organizations such as the WHO, World Dental Federation, and International Association for Dental Research may propose oral health goals that include clear and measurable targets to allow measuring achievement. Despite its relevance, oral health seems to be neglected, and currently 3.5 billion people worldwide are suffering the consequences of untreated oral conditions. Oral conditions accounted for more health loss than 35 of 39 categories of cancer. Only the combined category of trachea, bronchus, and lung cancers ranked higher than oral conditions, and estimates for liver, stomach, and colon and rectum cancer combined were comparable. The total health loss associated with oral conditions was also comparable to those for hypertensive heart disease, schizophrenia, and all maternal conditions combined. Indirect costs due to oral conditions worldwide amounted to US$144 billion yearly, corresponding to economic losses within the range of the 10 most frequent global causes of death (Listl et al. 2015). The direct and indirect global economic impact of oral conditions may amount to more than US$442 billion (Listl et al. 2015), and these estimates will keep rising with increasing DALYs.

## Conclusion

Oral health has not improved in the last 25 y. Oral conditions remain a major and growing global public health challenge in 2015. While the age-standardized prevalence of oral conditions remained relatively stable between 1990 and 2015, population growth and aging have led to a dramatic increase in the burden of untreated oral conditions throughout the world. Greater efforts and potentially different approaches are needed if international oral health goals are to be achieved by 2020.

## Author Contributions

N.J. Kassebaum, A.G.C. Smith, E. Bernabé, C.J.L. Murray, W. Marcenes, contributed to conception, design, data acquisition, analysis, and interpretation, drafted and critically revised the manuscript; T.D. Fleming, contributed to conception, design, data acquisition, analysis, and interpretation, critically revised the manuscript; A.E. Reynolds, contributed to design, data acquisition, and analysis, critically revised the manuscript; T. Vos, contributed to conception, data acquisition, analysis, and interpretation, drafted and critically revised the manuscript. All authors gave final approval and agree to be accountable for all aspects of the work.

## Supplementary Material

Supplementary material

Supplementary material
