# Supplementary material for: Global, Regional, and National Prevalence, Incidence, and Disability-Adjusted Life Years for Oral Conditions for 195 Countries, 1990–2015: A Systematic Analysis for the Global Burden of Diseases, Injuries, and Risk Factors
Source: J Dent Res. 2017 Apr;96(4):380–7. doi: 10.1177/0022034517693566 (PMC5912207; doi:10.1177/0022034517693566)
Supplement: Supplementary material [file DS_10.1177_0022034517693566.zip › DS_10.1177_0022034517693566_Appendix1.pdf]

Global, Regional, and National Prevalence, Incidence, and Disability-Adjusted Life Years for Oral Conditions for 195 Countries, 1990–2015: A Systematic Analysis for the Global Burden of Diseases, Injuries, and Risk Factors

N.J. Kassebaum, A.G.C. Smith, E. Bernabé, T.D. Fleming, A.E. Reynolds, T. Vos, C.J.L. Murray, W. Marcenes, and GBD 2015 Oral Health Collaborators

Appendix 1: Background and GBD 2015 methods for estimating the burden of oral conditions

DisMod-MR 2.1

Until GBD 2010, non-fatal estimates were based on a single data source on prevalence, incidence, remission, or a mortality risk selected by the researcher as most relevant to a particular geography and time. For GBD 2010, we set a more ambitious goal: to evaluate all available information on a disease that passes a minimum quality standard. That required a different analytical tool that would be able to pool disparate information presented in varying age groupings and from data sources using different methods. The DisMod-MR 1.0 tool used in GBD 2010 evaluated and pooled all available data, adjusted data for systematic bias associated with methods that varied from the reference and produced estimates by world regions with uncertainty intervals. For GBD 2013, the improved DisMod-MR 2.0 had increased computational speed allowing computations that were consistent between all disease parameters at the country rather than region level. The hundred-fold increase in speed of DisMod-MR 2.0 was partly due to a more efficient re-write of the code in C++ but also by changing to a model specification using log rates rather than a negative binomial model used in DisMod-MR 1.0. In cross-validation tests, the log rates specification worked as well or better than the negative binomial specification [1]. For GBD 2015, the computational engine (DisMod-MR 2.1) remained substantively unchanged but we re-wrote the 'wrapper' code that organized the flow of data and settings at each level of the analytical cascade. The sequence of estimation occurred at five levels: global, super-region, region, country, and, where applicable, subnational geographical units (see flow diagram of DisMod-MR 2.1 cascade, below). The super-region priors were generated at the global level with mixed-effects, non-linear regression using all available data; the super-region fit, in turn, informed the region fit, and so on down the cascade. The wrapper gave analysts the choice to branch the cascade in terms of time and sex at different levels depending on data density. The default used in most models was to branch by sex after the global fit but to retain all years of data until the lowest level in the cascade. For GBD 2015, we generated fits for the years 1990, 1995, 2000, 2005, 2010, and 2015.

In updating the 'wrapper,' we consolidated the code base into a single language, Python, to make the code more transparent and efficient and to better deal with subnational estimation. The computational engine is limited to three levels of random effects; we differentiated estimates at the super-region, region, and country level. In GBD 2013, the subnational units of China, Mexico, and the UK were treated as 'countries' such that a random effect was estimated for every geography with contributing data. However, the lack of a hierarchy between country

and subnational units meant that the fit to country data contributed as much to the estimation of a subnational unit as the fits for all other countries in the region. We found inconsistency between the country fit and the aggregation of subnational estimates when the country's epidemiology varied from the average of the region. Adding an additional level of random effects required a prohibitively comprehensive rewrite of the underlying DisMod-MR engine. Instead, we added a fifth layer to the cascade, with subnational estimation informed by the country fit and country covariates, plus an adjustment based on the average of the residuals between the subnational unit's available data and its prior. This mimicked the impact of a random effect on estimates between subnationals.

For GBD 2015 we improved how country covariates differentiate non-fatal estimates for diseases with sparse data. The coefficients for country covariates were re-estimated at each level of the cascade. For a given geography, country coefficients were calculated using both data and prior information available for that geography. In the absence of data, the coefficient of its parent geography was used, in order to utilize the predictive power of our covariates in data sparse situations.

Appendix Figure - GBD 2015 DisMod-MR 2.1 analytical cascade

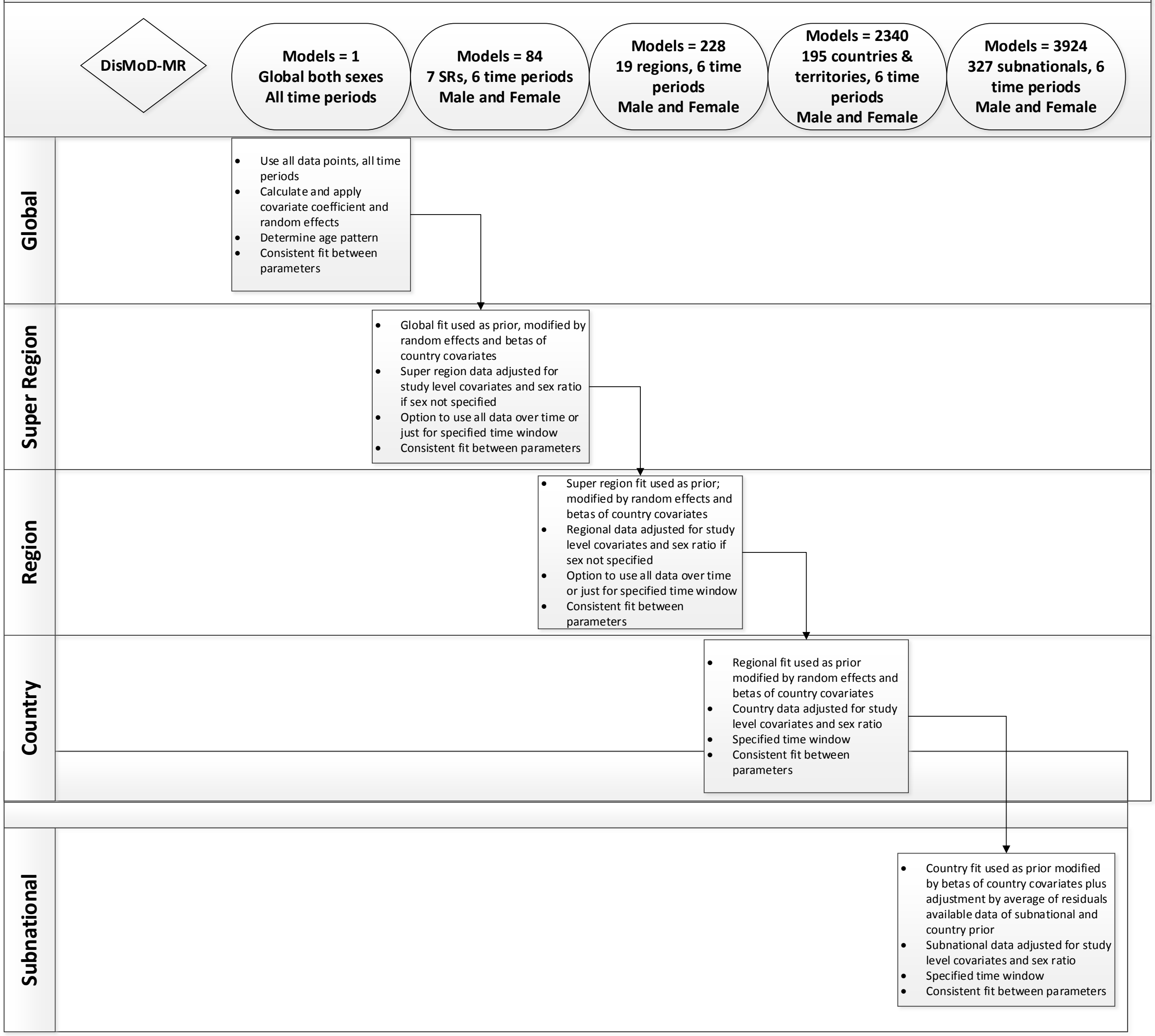

DisMod-MR 2.1 likelihood estimation

Analysts have the choice of using a Gaussian, log-Guassian, Laplace, or log-Laplace likelihood function in DisMod-MR 2.1. The default log-Gaussian equation for the data likelihood is:

$$-log\left[p\left(y_j|\Phi\right)\right] = \log\left(\sqrt{2\pi}\right) + \log\left(\delta_j + s_j\right) + \frac{1}{2}\left(\frac{\log\left(a_j + \eta_j\right) - \log\left(m_j + \eta_j\right)}{\delta_j + s_j}\right)^2$$

where, yj is a 'measurement value' (i.e. data point); Φ denotes all model random variables; ηj is the offset value, eta, for a particular 'integrand' (prevalence, incidence, remission, excess mortality rate, with-condition mortality rate, cause-specific mortality rate, relative risk, or standardised mortality ratio), and aj is the adjusted measurement for data point j, defined by:

$$a_j = e^{(-u_j - c_j)} y_j$$

where uj is the total 'area effect' (i.e. the sum of the random effects at three levels of the cascade: super-region, region, and country) and cj is the total covariate effect (i.e. the mean combined fixed effects for sex, study level, and country level covariates), defined by:

$$c_j = \sum_{k=0}^{K[I(j)]-1} \beta_{I(j),k} \hat{X}_{k,j}$$

with standard deviation

$$s_j = \sum_{l=0}^{L[I(j)]-1} \zeta_{I(j),l} \hat{Z}_{k,j}$$

where k denotes the mean value of each data point in relation to a covariate (also called x-covariate); I(j) denotes a data point for a particular integrand, j; βI(j),k is the multiplier of the kth x-covariate for the ith integrand; $\hat{X}_{k,j}$ is the covariate value corresponding to the data point j for covariate k; l denotes the standard deviation of each data point in relation to a covariate (also called z-covariate); ζI(j),k is the multiplier of the lth z-covariate for the ith integrand; and δj is the standard deviation for adjusted measurement j, defined by:

$$\delta_j = log\left[y_j + e^{(-u_j - c_j)} \eta_j + c_j\right] - log\left[y_j + e^{(-u_j - c_j)} \eta_j\right]$$

Where mj denotes the model for the jth measurement, not counting effects or measurement noise and defined by:

$$m_j = \frac{1}{B(j) - A(j)} \int_{A(j)}^{B(j)} I_j(\text{a})\ \text{da}$$

where A(j) is the lower bound of the age range for a data point; B(j) is the upper bound of the age range for a data point; and I(j) denotes the function of age corresponding to the integrand for data point j.

Caries in the deciduous dentition

Flowchart

Deciduous caries

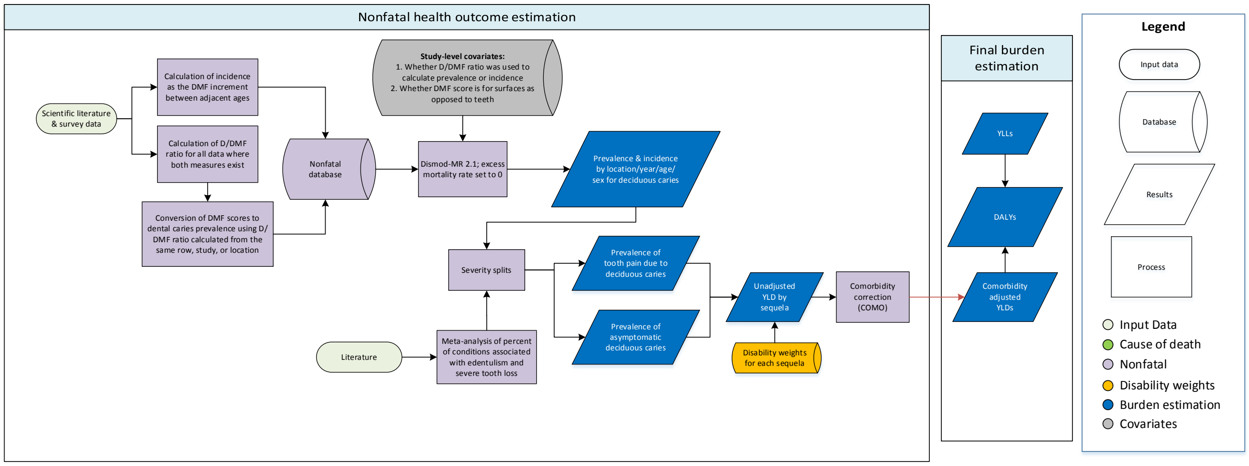

Case definition

The case definition for dental caries in the deciduous dentition is “teeth with unmistakable coronal cavity at dentin level.” This definition corresponds to an ICD-9 code of 521.0 and an ICD-10 code of K02.3 – K02.9. Most caries may be subclinical in the sense that they do not cause symptoms a majority of the time. Once a carious lesion develops, it will occasionally recede without intervention. Generally, however, it worsens with time and eventually requires either filling or extraction. The major sequela associated with the condition is symptomatic caries, which is defined as “a toothache which causes some difficulty eating.”

Deciduous teeth are colloquially known by several names throughout the world, including “baby,” “milk,” or “fall” teeth. They start erupting in infants around 6 months. Exfoliation of deciduous teeth begins around age 5-6 and usually is complete by age 12-14, when only permanent teeth remain.

Dental caries and the dmft index

Public health dentists commonly measure dental caries in deciduous teeth using the dmft index, which is an incremental measure of the proportion of unhealthy teeth and is also a measure of an individual’s lifetime prevalence of caries. “d” is for decayed, “m” for missing, “f” for filled, and “t” for teeth. The maximum dmft score is 20. Furthermore, dental caries can be measured in terms of tooth surfaces, rather than number of teeth, and results are reported using an analogous dmfs index. The maximum dmfs score is 88.

The dmft index is easy to measure and inter-rater reliability is high. However, the primary shortcoming of the dmft is that it does not discriminate well between current and past disease (treated or untreated caries). Strategies were employed to maximally utilize dmf data for estimating the prevalence of burden due to deciduous caries are described below.

Input data

Literature reviews

A literature review was conducted by the expert group for GBD 2010 and updated for GBD 2013. The search terms used in the GBD 2013 literature review for deciduous caries were (Deciduous caries[Title/Abstract]) OR (milk caries[Title/Abstract]) OR (baby caries[Title/Abstract]) OR (caries[Title/Abstract]) OR (dental health[Title/Abstract]) OR (oral health[Title/Abstract]) AND (prevalence[Title/Abstract]) AND ("2010"[Date - Publication] : "2013"[Date - Publication]). Updates to systematic reviews are performed on an ongoing schedule across all GBD causes and an update for caries in the deciduous dentition will be performed for GBD 2016.

We eliminated many data points to avoid repetition in the dataset, while striving to maintain as much data detail as possible. Redundancy tended to arise in three data descriptors: age, gender, and urbanicity. Our order of preference for maintaining detail was age, followed by gender, then urbanicity. Additionally, many of the studies presented dmft scores, which represent lifetime prevalence and were often described as "caries experience." For the purposes of measuring the burden of disability from dental caries, we considered only data on current prevalence to be relevant (d component of the dmf index), and thus converted lifetime prevalence data to current prevalence and incidence where possible (see below).

Conversion of dmf scores to prevalence and incidence

Caries on the deciduous dentition

Many of the studies that reported lifetime prevalence of deciduous caries (dmf scores) provided detail on the component breakdown of these scores. We used these data to calculate a d/dmf ratio and convert the lifetime prevalence value into one that reflected current prevalence. For example, if the lifetime prevalence was reported as 0.8 with d = 1.5 and dmft = 2 (d/dmf ratio = 0.75), we adjusted the prevalence value to 0.6. When possible, we used within-study d/dmf ratios to convert lifetime prevalence to current prevalence. Otherwise, we converted to current prevalence using a weighted average d/dmf ratio at the country, region, super-region, or global level, in that order of preference.

For studies reporting dmf scores for successive age intervals, the increment in the dmf values between examinations was considered to be equivalent to the caries incidence over the study duration. We extrapolated incidence data from two types of studies. First, for longitudinal or cohort studies, we calculated the caries increment over successive ages and time periods as the difference between the dmf scores at each time point. Narrow age and time intervals were preferred; most were of three years or less. We did not extrapolate incidence data if the age or time interval was greater than 10 years. Secondly, if a study only performed a single cross-sectional examination, but reported data in age intervals of three years or less, we extrapolated incidence data in the same manner. Narrow age ranges were considered necessary for this incidence extrapolation because the dental health of population cohorts has been observed to change in just a few years when preventive measures are instituted.

Data availability for caries in deciduous teeth:

	Prevalence	Incidence	Continuous DMF score converted to prevalence	Continuous DMF score converted to incidence
Studies	69	0	124	59
Countries/subnationals	42/41	0	55/26	32/19
GBD world regions	17	0	19	16

Modeling strategy

Separate estimates of deciduous and permanent caries

The natural histories of deciduous and permanent caries share many similarities, but they also share some important differences. Age patterns of decay in permanent and deciduous dentition are distinct, and duration of a carious lesion in deciduous teeth also tends to be shorter than an untreated episode of caries in the permanent dentition. Finally, it is unclear whether the gender patterns and regional differences are the same for both deciduous and permanent caries. For all of these reasons, we elected to model deciduous caries and permanent caries as separate entities and then add the estimates together for an overall estimation of the global burden of dental caries. This modeling approach was also taken in GBD 2010 and GBD 2013.

DisMod model development: caries in the deciduous dentition

Serious health consequences of caries in deciduous teeth were assumed to be uncommon and death very rare. For purposes of modeling, we therefore assigned excess mortality to be zero from age 0 to 100. We fixed incidence and prevalence at zero after age 12 when exfoliation is presumed to be complete. This age was chosen because 11 was the oldest age of a non-zero prevalence data point. We additionally assigned incidence and prevalence to be zero before age 6 months to indicate that this condition never begins at birth and is absent in the neonatal and post-neonatal periods. Incidence bounds of 0 to 4.0 for ages 1 to 10 years were chosen based on examining the dataset and adding a comfortable margin to the highest reported value. An upper remission bound of 1.0 for ages 0 to 5 years was chosen in order to fit the sharp increase in prevalence over this age range. As prevalence was assigned to be zero after age 14 anyway, we elected to not include a lower remission bounds.

No country-level covariates were included. We used a study-level covariate to indicate whether a given prevalence data point was of “true” current prevalence or calculated from lifetime prevalence using the d/dmf ratio. For both incidence and prevalence, we also used study-level covariates to indicate whether the dmf scores were for surfaces as opposed to teeth.

Because no “true” unconverted incidence values were present in the dataset, we could not crosswalk the extrapolated incidence values to reference incidence values for deciduous caries. Instead, we used higher heterogeneity settings for incidence values than for prevalence (0.7 for incidence, 0.2 for prevalence). We calculated age mesh points at ages 0, 0.5, 1, 2, 3, 4, 5, 6, 7, 8, 9, 10, 11, 12, 13, 14, 15, and 100 years. High smoothness settings were used for both incidence and prevalence to allow for dynamic age trends.

Study-level covariate	Parameter	beta	Exponentiated beta
Whether d/dmf ratio was used to convert lifetime prevalence to current prevalence	Prevalence	-0.08	0.92
Whether dmf score is for surfaces	Prevalence	0.18	1.19
Whether dmf score is for surfaces	Incidence	1.99	7.30

Models were vetted based on the biological plausibility of the results, the extent to which estimates fit the data, and the plausibility of the range of estimates across location hierarchies.

Disability weights

The GBD definition of disability associated with symptomatic dental caries is “this person has a toothache, which causes some difficulty eating.” The disability weight associated with this condition is 0.01 (0.005 – 0.019), as derived from the GBD Disability Weights Study. Those with deciduous caries who had undergone exfoliation or had their cavities filled were considered to have no disability. Also, not all those with untreated dental caries had this disability all the time. We considered only those with untreated decay at dentine level to experience symptomatic tooth pain. Two additional pieces of information are required to complete the calculation of years of life lived with disability (YLDs): proportion with symptoms and duration of disability.

To determine duration, we adapted the method employed by the Australian Burden of Disease (AusBoD) Study in 1996. For total duration, we used the posterior estimates of duration from final DisMod-MR 2.1 models. For those with symptoms, we split this total duration into two distinct phases of caries disability. The “initial” phase is characterized by *periodic* pain that we assigned to occur an average of one hour per day. The “terminal” phase is a period of *constant* symptoms at the end of an episode. The length of the terminal phase was determined by literature review as described by the AusBoD group. Based on the distribution of time courses, a log-normal distribution was plotted that approximated the average duration of *constant* symptoms at 27.6 days leading up to seeking care. For deciduous caries we used a study by Mason, et al. of children in the UK presenting to a casualty ward with tooth pain [2]. For those with severe disease, the length of time spent in the terminal phase was subtracted from the total duration to determine the amount of time spent in the initial phase. For those with mild disease, we considered the entire duration to be spent in the initial phase.

To determine proportion with symptoms, we completed a supplemental literature review of tooth pain and caries. We identified a total of 21 studies with data about the prevalence of pain. The studies were grouped according to the type of dentition studied (deciduous or permanent) and the location of the study group (high-income or low- and middle-income countries). We extracted data on the proportion in each group that described symptoms of pain related to their caries as well as a subset who described their symptoms as being severe. The proportions in

each group were weighted according to sample size to give estimates of the relative sizes of three groups: asymptomatic, mild, and severe.

We considered asymptomatic individuals to experience no disability. Those with mild disease spent the entire duration in the initial phase of disease (one hour of pain per day). Those with severe disease spent a majority of the duration in the initial phase followed by a period of time in the terminal phase (constant pain). YLDs were calculated by multiplying the prevalence, duration, proportion, and disability weight for each age, country, sex, and year.

Caries in the permanent dentition

Flowchart

Permanent Caries

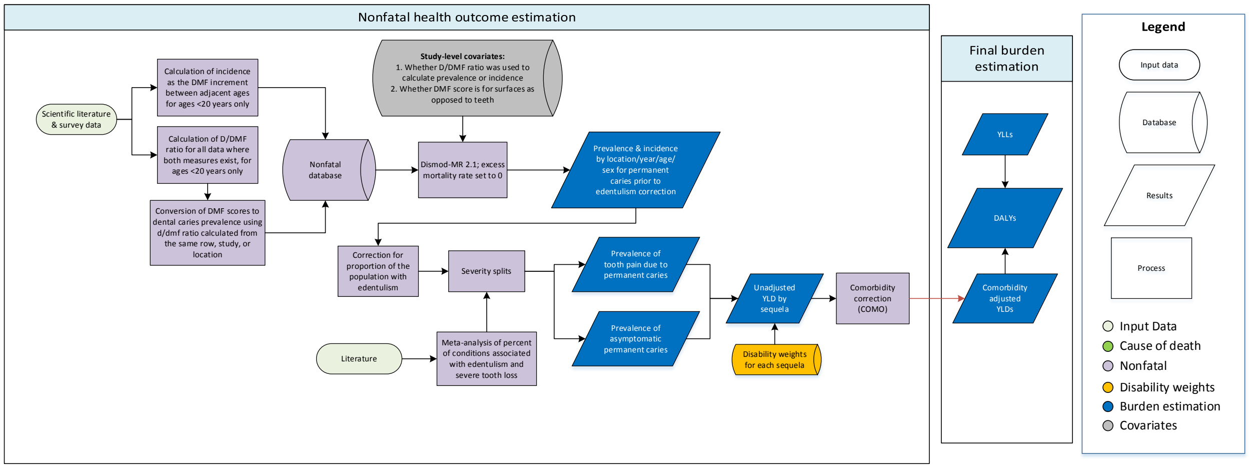

Case definition

The case definition for dental caries in the permanent dentition is "teeth with unmistakable coronal cavity at dentin level or root cavity in cementum that feel soft or leathery to probing" This definition corresponds to an ICD-9 code of 521.0 and an ICD-10 code of K02.3 – K02.9. Most caries may be subclinical in the sense that they do not cause symptoms a majority of the time. Once a carious lesion develops, it will occasionally recede without intervention. Generally, however, it worsens with time and eventually requires either filling or extraction. The major sequela associated with the condition is symptomatic caries and the disability definition is "a toothache, which causes some difficulty eating."

Dental caries and the DMFT index

Public health dentists commonly measure dental caries in permanent teeth using the DMFT index, which is an incremental measure of the proportion of unhealthy teeth and is also a measure of an individual's lifetime prevalence of caries. "D" is for decayed, "M" for missing, "F" for filled, and "T" for teeth. The maximum DMFT score is 32. Furthermore, some dentists prefer to measure dental caries in terms of tooth surfaces, rather than number of teeth, and report their results using an analogous DMFS index. The maximum DMFT score is 88, and the maximum DMFS score is 128 or 148 depending on whether the third molars are counted.

The DMFT index is easy to measure and inter-rater reliability is high. However, the primary shortcoming of the DMFT is that it does not discriminate well between current and past disease (treated or untreated caries). Strategies were employed to maximally utilize DMF data for estimating the prevalence of burden due to permanent caries are described below.

Input data

Literature reviews

A literature review was conducted by the expert group for GBD 2010 and updated for GBD 2013. The search terms used in the GBD 2013 literature review for permanent caries were (Permanent caries[Title/Abstract]) OR (caries prevalence[Title/Abstract]) OR (dental health[Title/Abstract]) OR (oral health[Title/Abstract]) AND (prevalence[Title/Abstract]) AND (“2010”[Date - Publication] : “2013”[Date - Publication]). Updates to systematic reviews are performed on an ongoing schedule across all GBD causes and an update for deciduous and permanent caries will be performed for GBD 2016.

We eliminated many data points to avoid repetition in the dataset, while striving to maintain as much data detail as possible. Redundancy tended to arise in three data descriptors: age, gender, and urbanicity. Our order of preference for maintaining detail was age, followed by gender, then urbanicity. Additionally, many of the studies presented DMFT scores, which represent lifetime prevalence and were often described as “caries experience.” For the purposes of measuring the burden of disability from dental caries, we considered only data on current prevalence to be relevant (D component of the DMF index), and thus converted lifetime prevalence data to current prevalence and incidence where possible (see below).

Conversion of DMF scores to prevalence and incidence

Caries in the permanent dentition

Whereas in the deciduous dentition, a vast majority of the dmf index is accounted for by caries, tooth loss is a major contributor to the DMF index for the permanent dentition. Permanent caries may not necessarily be the primary driver of this tooth loss, as other factors such as periodontal disease and trauma may contribute significantly. Thus, we performed the conversions of DMF scores to prevalence and incidence values as described above for caries in deciduous teeth only in individuals ages 20 years or less.

Data availability for caries in permanent teeth:

	Prevalence	Incidence	Continuous DMF score converted to prevalence	Continuous DMF score converted to incidence
Studies	70	4	90	31
Countries/subnationals	45/22	4/2	47/20	24/11
GBD world regions	16	3	19	14

Modeling strategy

Separate estimates of caries in the deciduous and permanent dentition

The natural history of caries in the deciduous and permanent dentitions share many similarities, but they also share some important differences. Age patterns of decay in permanent and deciduous dentition are distinct, and duration of a carious lesion in deciduous teeth also tends to be shorter than an untreated episode of permanent caries. Also, it is unclear whether the gender

patterns and regional differences are the same for both deciduous and permanent dentitions. We elected to model caries in the deciduous and permanent dentitions as separate entities and then add the estimates together for an overall estimation of the global burden of dental caries. This is the modeling approach which was also taken in GBD 2010.

DisMod model development: caries in the permanent dentition

Serious health consequences of caries in the permanent dentition were also assumed to be uncommon and death very rare. We therefore assigned excess mortality to be zero from age 0 to 100. The dataset suggested that permanent caries are sometimes incident in 5-year-olds, so we fixed incidence and prevalence at 0 for ages 0 to 4. Incidence bounds were again chosen based on examining the dataset and adding a margin to the highest reported value. In this case, incidence bounds were 0 – 2. Lower bounds for remission were set at 0.2 and upper bounds were set at 3.

As with caries in the deciduous dentition, no country-level covariates were included. We used a study-level covariate to indicate whether a given prevalence data point was of “true” current prevalence or calculated from lifetime prevalence using the D/DMF ratio, and another study-level covariate to indicate whether a given incidence values was extrapolated from DMF scores. For both incidence and prevalence, we also used study-level covariates to indicate whether the DMF scores were for surfaces as opposed to teeth.

We calculated age mesh points at ages 0, 4, 5, 8, 10, 12, 15, 19, 20, 25, 29, 30, 35, 40, 45, 50, 55, 60, 70, 80, 90, and 100 years. Heterogeneity was set to 0.5 for both incidence and prevalence. High smoothness settings were used for both incidence and prevalence to allow for dynamic age trends.

**Study-level covariate**	**Parameter**	**beta**	**Exponentiated beta**
Whether D/DMF ratio was used to convert lifetime prevalence to current prevalence	Prevalence	-0.27	0.77
Whether DMF score was used to calculate incidence	Incidence	0.25	1.28

Although studies were screened carefully during data extraction to ensure that they specified whether they were measuring caries in the permanent or deciduous dentition, some data points were marked as outliers during modeling due to their high prevalence values in young ages, as it was deemed likely that some of these studies were reporting deciduous in addition to permanent caries.

As with caries in the deciduous dentition, models for caries in the permanent dentition were vetted based on the biological plausibility of the results, the extent to which estimates fit the data, and the plausibility of the range of estimates across location hierarchies.

Correction for total tooth loss

One systematic source of bias in the literature was the exclusion of edentate individuals from the study populations, which leads to systematic overestimation of caries prevalence when modeled over the entire population. To account for this bias, we used our GBD estimates of total tooth loss prevalence to adjust YLD estimates for dental caries. Using the final DisMod-MR 2.1 estimates for prevalence of total tooth loss we calculated the mean prevalence for each age and sex and averaged the 1990 and 2015 values. We then calculated a population-weighted mean prevalence for each region and each GBD super-region. The resulting super-regional averages were used to adjust the DisMod-MR 2.1 estimates for prevalence of permanent caries in calculating years lost due to disability (YLDs).

Disability weights

As described above, the GBD definition of disability associated with symptomatic dental caries is “this person has a toothache, which causes some difficulty eating.” The disability weight associated with this condition is 0.01 (0.005 – 0.019), as derived from the GBD Disability Weights study. Those with caries in the permanent dentition who had received fillings or had their teeth extracted due to a carious lesion were considered to have no disability. Also, not all those with untreated dental caries had this disability all the time. We considered only those with untreated decay at dentine level to experience symptomatic tooth pain. Likewise, those with permanent caries who had received fillings, had their cavities extracted, or lost a carious tooth altogether were considered to have no disability. Thus, two additional pieces of information are required to complete the calculation of years of life lived with disability (YLDs): proportion with symptoms and duration of disability.

To determine duration, we adapted the method employed by the Australian Burden of Disease (AusBoD) Study in 1996. For total duration, we used the posterior estimates of duration from final DisMod-MR 2.1 models. For those with symptoms, we split this total duration into two distinct phases of caries disability. The “initial” phase is characterized by *periodic* pain that we assigned to occur an average of one hour per day. The “terminal” phase is a period of *constant* symptoms at the end of an episode. The length of the terminal phase was determined by literature review as described by the AusBoD group. The length of time each person had been experiencing tooth pain was recorded. Based on the distribution of time courses, a log-normal distribution was plotted that approximated the average duration of *constant* symptoms at 27.6 days leading up to seeking care. For permanent caries, a similar study of the tooth pain experience of adults in New Zealand who presented to hospital dental departments and an emergency clinic [3] resulted in an estimated 55.2 days spent in the terminal phase of caries. For those with severe disease, the length of time spent in the terminal phase was subtracted from the total duration to determine the amount of time spent in the initial phase. For those with mild disease, we considered the entire duration to be spent in the initial phase.

To determine proportion with symptoms, we completed a supplemental literature review of tooth pain and caries. We identified a total of 21 studies with data about the prevalence of pain. The studies were grouped according to the type of dentition studied (deciduous or permanent) and the location of the study group (high-income or low- and middle-income countries). We

extracted data on the proportion in each group that described symptoms of pain related to their caries as well as a subset who described their symptoms as being severe. The proportions in each group were weighted according to sample size to give estimates of the relative sizes of three groups: asymptomatic, mild, and severe.

We considered asymptomatic individuals to experience no disability. Those with mild disease spent the entire duration in the initial phase of disease (one hour of pain per day). Those with severe disease spent a majority of the duration in the initial phase followed by a period of time in the terminal phase (constant pain). YLDs were calculated by multiplying the prevalence, duration, proportion, and disability weight for each age, country, sex, and year.

Severe Chronic Periodontitis

Flowchart

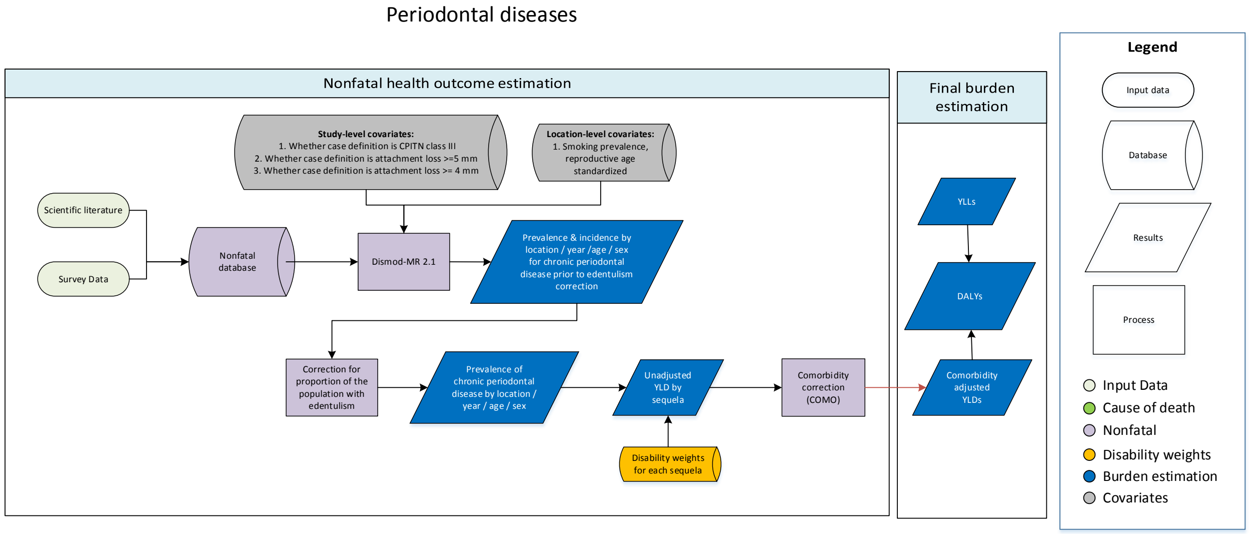

Case definition

The case definition of severe chronic periodontitis (SCP) for literature review was in order of preference, “a Community Periodontal Index of Treatment Needs (CPITN) score of 4, a gingival pocket (GP) depth equal or more than 6mm or a clinical attachment loss (AL) more than 6mm”, depending on which was used in the publication. The 1999 World Workshop for the Classification of Periodontal Diseases and Conditions identified three periodontitis types based on specific aetiological formulation. The three groups were: chronic periodontitis, aggressive periodontitis and periodontitis as a manifestation of systemic disease [4]. The cause list of the GBD study included chronic periodontitis.

Quantifying periodontal diseases in a meaningful and reproducible manner has been an ongoing challenge for oral epidemiologists and clinicians. Several classification systems have been developed to describe clinical manifestations of periodontitis, most of which have their own case definitions and scales for quantifying severity. Unification under a single case definition have been a challenge. The World Health Organization (WHO) introduced the CPITN in 1987, recommending the use of PD as criteria for identifying cases of chronic periodontitis. Influential oral health surveys such the Adult Dental Health Survey in the United Kingdom and the National

Health and Nutrition Examination Surveys (NHANES) in the United States have measured AL in addition to PD. AL and PD have emerged as the most common metrics of periodontal health measurement. In 2007, a new CDC proposal for gold standard diagnosis of severe, chronic periodontitis was published. This standard specified that a more strictly definition of the condition should be implemented. This more exclusive definition of chronic periodontal disease includes ≥ 2 interproximal sites with AL ≥ 6 mm ***AND*** ≥ 1 interproximal site with PD ≥ 5 mm [2].

PD is measured as the distance between the gingival margin and the base of the pocket and AL is the distance from the cemento-enamel junction to the base of the pocket. The CPITN is a classification system that was developed by the WHO as a standardized method of periodontal health treatment need measurement [1]. CPITN classification scores range from 0 to 4 and includes quantifying pocket depth. The mouth is divided into 6 sections, called sextants. Sextants with fewer than two teeth are excluded. Multiple teeth in each sextant are examined. A standard-sized probe is used with depth markings from 3.5 to 5.5 mm. The probe is inserted into the sulcus between a tooth and the gingiva until it meets resistance. The surrounding area is then explored with the probe to determine the maximum depth of the pocket. Multiple areas around each tooth are probed. The examiner moves the probe gently, with short upward and downward movements, along the buccal sulcus or pocket, to the mesial surface of the teeth. A similar procedure is carried out for lingual surfaces, starting on the disto-lingual aspect of the teeth. When the CPITN method was employed, we considered those with Class 4 only (PD ≥ 6mm). We excluded studies in which the study population was reported as the number of sextants rather than the number of individuals surveyed. If more than one type of data was included in a study, our first preference was for CPITN = 4, followed by AL >6 mm and PD ≥ 6mm, which was considered a satisfactory representation of the GBD case definition. All definitions were extracted for each datum as available this time, and a series of study covariates were used to crosswalk non-standard definitions to the reference standard of CPITN stage 4 (see below).

The GBD definition of disability associated with symptomatic SCP is “bad breath, a bad taste in the mouth, and gums that bleed a little from time to time, but which does not interfere with daily activities.” The ICD-10 codes for periodontal disease are K05.0 – K05.6, and the ICD-9 codes are 523.0 – 523.9.

Input data

Literature reviews

For GBD 2010, a review of the literature on periodontal disease prevalence was conducted by the Expert Group and updated for GBD 2013. The GBD 2013 literature review used the following search terms: (Periodontal disease[Title/Abstract]) OR (periodontitis[Title/Abstract]) OR (periodontal[Title/Abstract]) AND (prevalence[Title/Abstract]) AND (“2010”[Date - Publication] : “2013”[Date - Publication]). Updates to systematic reviews are performed on an ongoing schedule across all GBD causes and an update for chronic periodontal disease will be performed in GBD 2016.

Data availability for severe chronic periodontitis:

	Prevalence	Incidence	Mortality risk
Studies	100	-	4
Countries/subnationals	49/16	-	3/1
GBD world regions	18	-	2

Modeling strategy

Overview

Evidence for chronic periodontal disease being a direct, proximate cause of death is lacking. As such, it was not included in overall causes of death analysis. However, there is a developing body of literature to suggest that those with chronic periodontal disease may be at increased risk of death from other causes. Relative risk data were, therefore, included in modeling of morbidity, but overall years of life lost (YLLs) were estimated to be zero. Models of disease burden due to chronic periodontal disease instead focused on estimating morbidity (YLDs) associated with the condition, and chronic periodontal disease was not included in risk factor analysis of any other condition.

Correction for total tooth loss

Bias in the dataset was felt to be limited, but some systematic bias was present in the definition of the study populations. In virtually all studies, edentate persons were excluded from evaluation. This exclusion is justified in the context of periodontal disease surveillance because advanced periodontal disease is not common in those who are toothless. To account for the systematic bias inherent in excluding those with total tooth loss from the denominator, we discounted the prevalence numbers estimated by DisMod MR 2.1. For example, if 40% of 70-74 years old females were estimated to be edentate in a certain region, the corresponding estimates for severe chronic periodontitis prevalence were reduced to 60% of the original value.

DisMod model development

Mortality was fixed to zero and relative risk was fixed to 1.0 before age 30, as any excess cardiovascular events that occur in those with severe tooth loss would not be expected at young ages. Incidence and prevalence were assigned to be zero until age 8 as periodontal disease is largely considered to be a disease of adulthood. Incidence was allowed to rise beginning at age 9, based on the youngest age at which there was a non-zero point estimate for prevalence in the dataset.

Bounds were assigned for remission and excess mortality to improve plausibility in the DisMod-MR 2.1 estimates. Remission was bounded 0 to 0.05 and excess mortality rate was bounded to 0.0001. We considered both bounds to be within reasonable ranges for the observed natural history of the disease. Reproductive age-standardized smoking prevalence was used as a country-level covariate.

Models were vetted based on the biological plausibility of the results, the extent to which estimates fit the data, and the plausibility of the range of estimates across location hierarchies.

Study covariate	Parameter	beta	Exponentiated beta
Data correspond to those with CPITN class III periodontal disease	Prevalence	0.21 (0.0062 – 0.58)	1.23 (1.01 – 1.79)
Data correspond to those with attachment loss >= 5 mm	Prevalence	0.89 (0.64 – 1.13)	2.43 (1.90 – 3.11)
**Country covariate**	**Parameter**	**beta**	**Exponentiated beta**
Smoking prevalence (reproductive age-standardized)	Prevalence	0.11 (0.0054 – 0.20)	1.11 (1.01 – 1.22)

Disability calculation and YLDs

Because those who are edentate can rarely have severe chronic periodontitis, we corrected for total tooth loss as described above. Using the DisMod-MR 2.1 estimates for prevalence of total tooth loss, we calculated the mean prevalence for each age and sex and averaged the 1990 and 2015 values. We then calculated a population-weighted mean prevalence for each region followed by the same for each GBD super-region. The resulting super-regional averages were used to adjust the estimates for prevalence of severe chronic periodontitis in calculating years lost due to disability (YLDs).

We considered all estimated prevalent cases of chronic periodontal disease to experience the disability described by "bad breath, a bad taste in the mouth, and gums that bleed a little from time to time, but this does not interfere with daily activities." The GBD Disability Survey differentiated between those who experience pain and those who do not, but the calculated disability weight was the same for both forms of the condition, 0.007 (0.003 – 0.014).

Total tooth loss

Flowchart

Edentulism and severe tooth loss

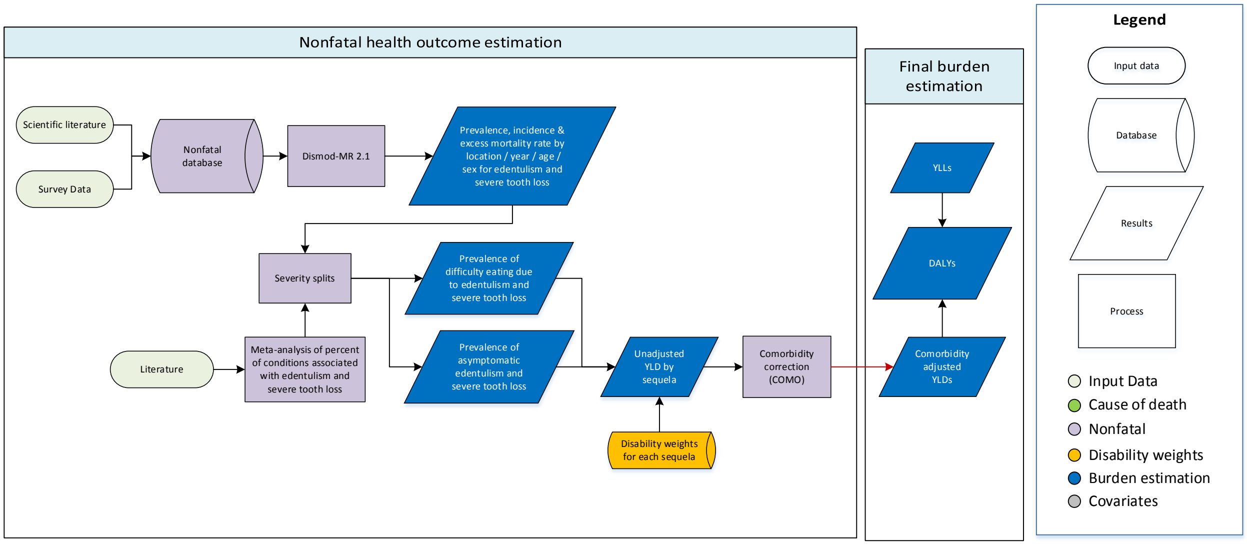

Case definition

The case definition of total tooth loss includes any individual with no remaining natural teeth; tooth loss in the deciduous dentition is not included. The GBD definition of disability associated with total tooth loss is “great difficulty in eating meat, fruits, and vegetables.” A small body of evidence has begun to emerge that implicates tooth loss as predisposing individuals to increased risk for systemic diseases. These data are sparse but have been included in models estimating burden of total tooth loss. However, this oral condition was not included in the risk factor analysis for ischemic cardiovascular diseases or other systemic diseases.

Input data

Literature reviews

An initial literature review was done by the Expert Group for GBD 2010, including published articles as well as the results of national and subnational reports, and updated for GBD 2013. The search terms for this systematic review included: (Edentulism[Title/Abstract]) OR (edentulous[Title/Abstract]) OR (endentulousness[Title/Abstract]) OR (severe tooth loss[Title/Abstract]) OR (total tooth loss[Title/Abstract]) OR (complete tooth loss[Title/Abstract]) AND (prevalence[Title/Abstract]) AND (“2010”[Date - Publication] : “2013”[Date - Publication]). New World Health Survey data were added for 47 countries. Updates to systematic reviews are performed on an ongoing schedule across all GBD causes and an update for total tooth loss will be performed in GBD 2016.

Bias in the dataset was considered to be negligible. Diagnostic criteria for this condition are very clear (no natural teeth). Additionally, all included studies are considered representative of the study population. Thus, covariates to account for excess variability were not deemed necessary. Few data points were marked as outliers during the modeling process.

Data availability for total tooth loss:

	Prevalence	Incidence	Mortality risk
Studies	157	11	11
Countries/subnationals	76/11	5/4	4/5
GBD world regions	20	4	3

Modeling strategy

First, estimates for the prevalence of total tooth loss were calculated for each location/year/sex/age using DisMod-MR 2.1. Then, estimates of the proportion of the population with access to dentures were generated for each location, and the disability weight for "great difficulty in eating meat, fruits, and vegetables" was applied to the proportion of the population with total tooth loss and no access to dentures.

DisMod model development

As would be expected for an irreversible condition, remission was fixed at zero for all ages. Mortality and relative risk were both fixed at zero before age 30, as any excess cardiovascular events resulting from severe tooth loss would not be expected at younger ages. We also assigned incidence and prevalence to be zero during childhood. Incidence was allowed to rise beginning at age 15, which was chosen based on the age at which the permanent dentition is expected to have fully formed in all individuals. The random effect limits for all locations were bounded at +/- 1.

As mentioned above, the criteria for diagnosis of total tooth loss are straightforward, and bias in the dataset was considered negligible. Thus, no study-level covariates were used in modeling the prevalence of this oral condition. We included lnLDI as a country-level covariate, with a minimum beta value of 0.02; see results in the table below.

Country-level covariate	Parameter	beta	Exponentiated beta
lnLDI ($ per capita)	Prevalence	0.026 (0.020 — 0.042)	1.03 (1.02 — 1.04)

Models were vetted based on the biological plausibility of the results, the extent to which estimates fit the data, and the plausibility of the range of estimates across location hierarchies. We have made no substantive changes in the modeling strategy from GBD 2013.

Disability weights

The disability weight used for total tooth loss is 0.067 (0.045 – 0.095) as determined by the GBD Disability Survey. We considered all those with total tooth loss and no access to dentures to experience this disability. However, the proportion of those with total tooth loss who have dentures has not been studied extensively.

In order to estimate the proportion of individuals with total tooth loss and no access to dentures, we completed a supplemental literature review of dentures prevalence. Only six systematic surveys of dentures prevalence were identified, all in high- and middle-income countries. All were completed since 2000. After extracting the data from the studies, we performed linear

regressions of denture presence and denture absence against health system access (HSA), a standardized covariate of treatment availability used in many disease estimation models. From the results of the regression, the prevalence of no dentures was calculated for all super-regions. We then completed a population-weighted average of all countries in the super-region based on 2013 populations, the average year of the dentures studies. Uncertainties for the prevalence of dentures were calculated by finding the standard deviation and standard error of the calculated prevalence values.

The estimated prevalence of dentures in each location was used to calculate the proportion of individuals with total tooth loss who have access to dentures and those with difficulty eating due to total tooth loss without access to dentures. This latter sequela was included as a cause of years lost due to disability (YLDs).

Other Oral Disorders

Other oral disorders encompass a wide variety of dental, tongue, and jaw disorders and malformations, including all oral disorders that are not included in the case definitions of dental caries in the permanent or deciduous dentitions, severe chronic periodontitis, or total tooth loss. Mouth cancers were not included in this category. All data on the prevalence of other oral disorders were obtained from the United States Medical Expenditure Panel Surveys, a nationally representative survey conducted yearly from 1996 to 2011 by the US Agency for Healthcare Research and Quality. Updates to systematic reviews are performed on an ongoing schedule across all GBD causes and an update for deciduous and permanent caries will be performed for GBD 2016.

These data were modeled in DisMod-MR 2.1 using a prevalence-only model with age mesh points set at 0, 0.5, 1, 5, 10, 20, 30, 40, 50, 60, 70, 80, 90, and 100 years of age. Heterogeneity for prevalence was set to the default of 0.5, and smoothness for prevalence was set to the default of 0.3. No study-level or country-level covariates were used in this model other than the study-level covariate for sex, which was fixed at the super-region level. This model provided us with estimates of the prevalence of other oral disorders for every location/age/sex combination. We plan to further improve the comprehensiveness of estimates for other oral conditions during GBD 2016.

References

1. Flaxman A, Vos T, Murray C. Integrated Meta-Regression Framework for Descriptive Epidemiology. University of Washington Press, 2014.
2. Mason MC, Porter SR, Madland G, Parry J. Early management of dental pain in children and adolescents. J Dent. Jan 1997; 25(1): 31-4.
3. Whyman RA, Treasure ET, Ayers KM. Dental disease levels and reasons for emergency clinic attendance in patients seeking relief of pain in Auckland. NZ Dent J. Dec 1996; 92(410): 114-7.
4. Armitage GC. Periodontal diagnoses and classification of periodontal diseases. *Periodontology 2000*. 2004: 34:9-21.
