# Supplementary material for: Global, Regional, and National Prevalence, Incidence, and Disability-Adjusted Life Years for Oral Conditions for 195 Countries, 1990–2015: A Systematic Analysis for the Global Burden of Diseases, Injuries, and Risk Factors
Source: J Dent Res. 2017 Apr;96(4):380–7. doi: 10.1177/0022034517693566 (PMC5912207; doi:10.1177/0022034517693566)
Supplement: Supplementary material [file DS_10.1177_0022034517693566.zip › DS_10.1177_0022034517693566_Appendix2.pdf]

Appendix 2, Figure 1. World map of all-ages DALY rate (per 1,000 population) due to untreated permanent caries in 2015, both sexes

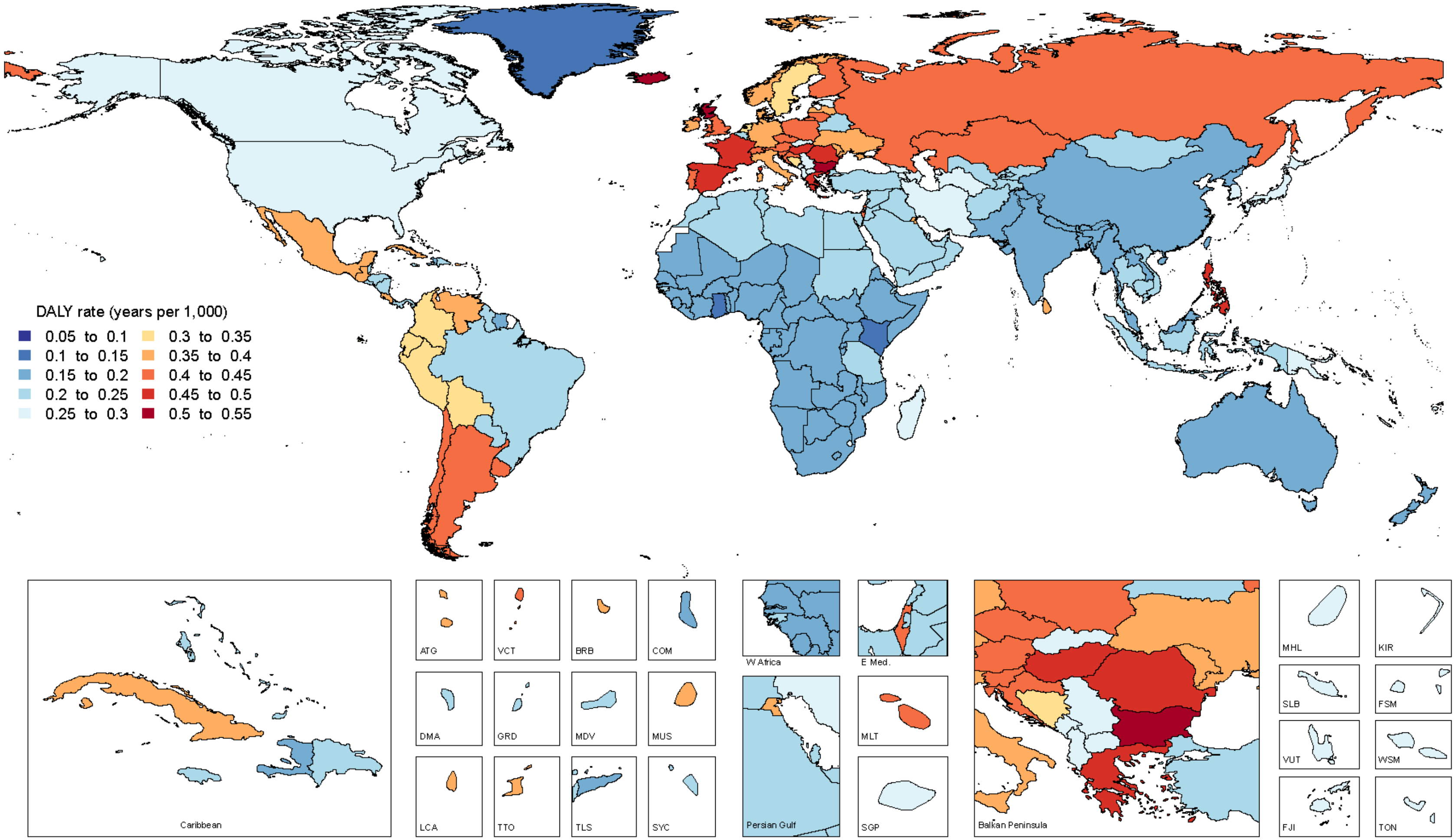

Appendix 2, Figure 2. World Map of age-standardised DALY rate (per 1,000 population) due to untreated permanent caries in 2015, both sexes

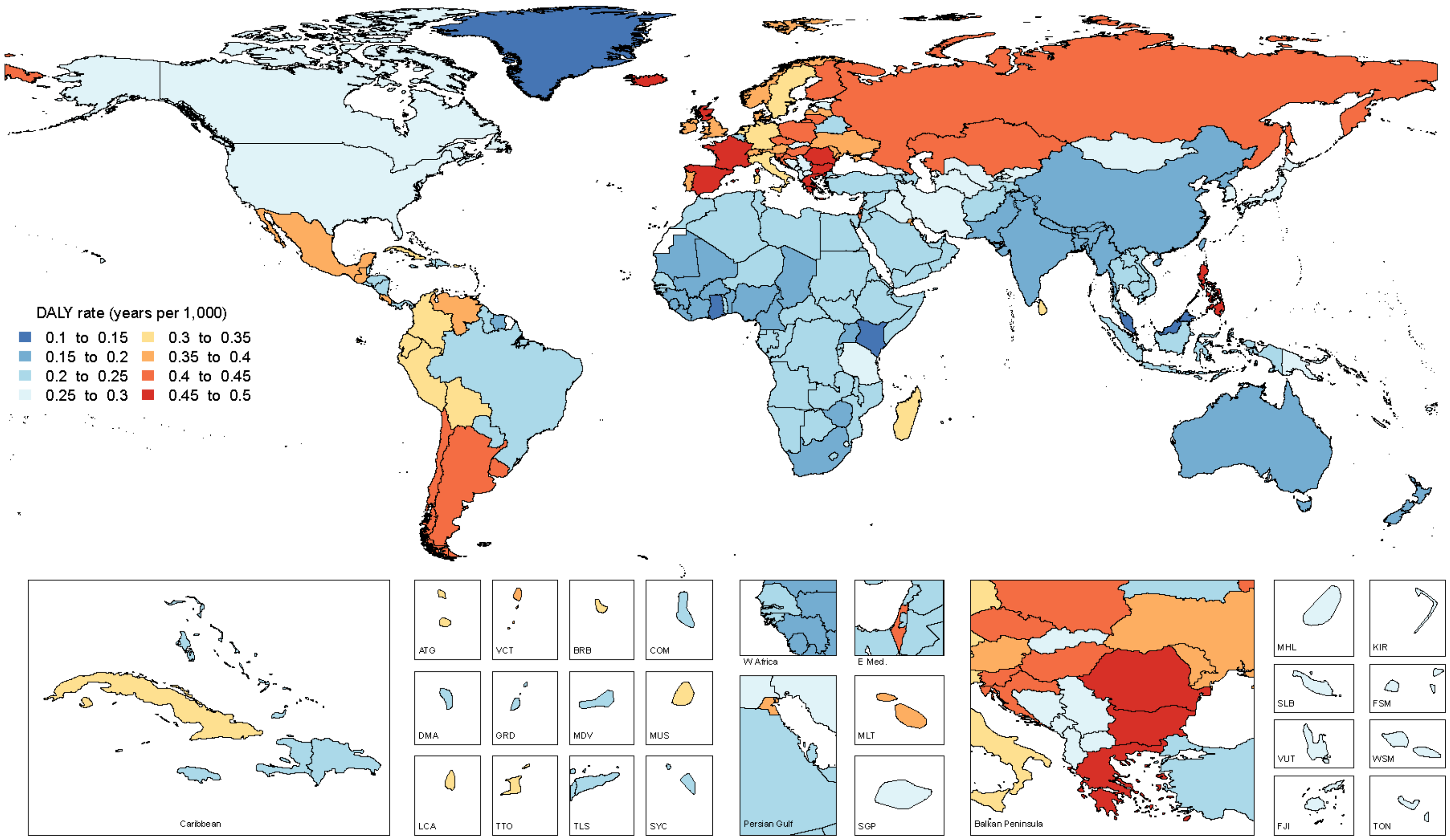

Appendix 2, Figure 3. World map of all-ages DALY rate (per 100,000 population) due to untreated deciduous caries in 2015, both sexes

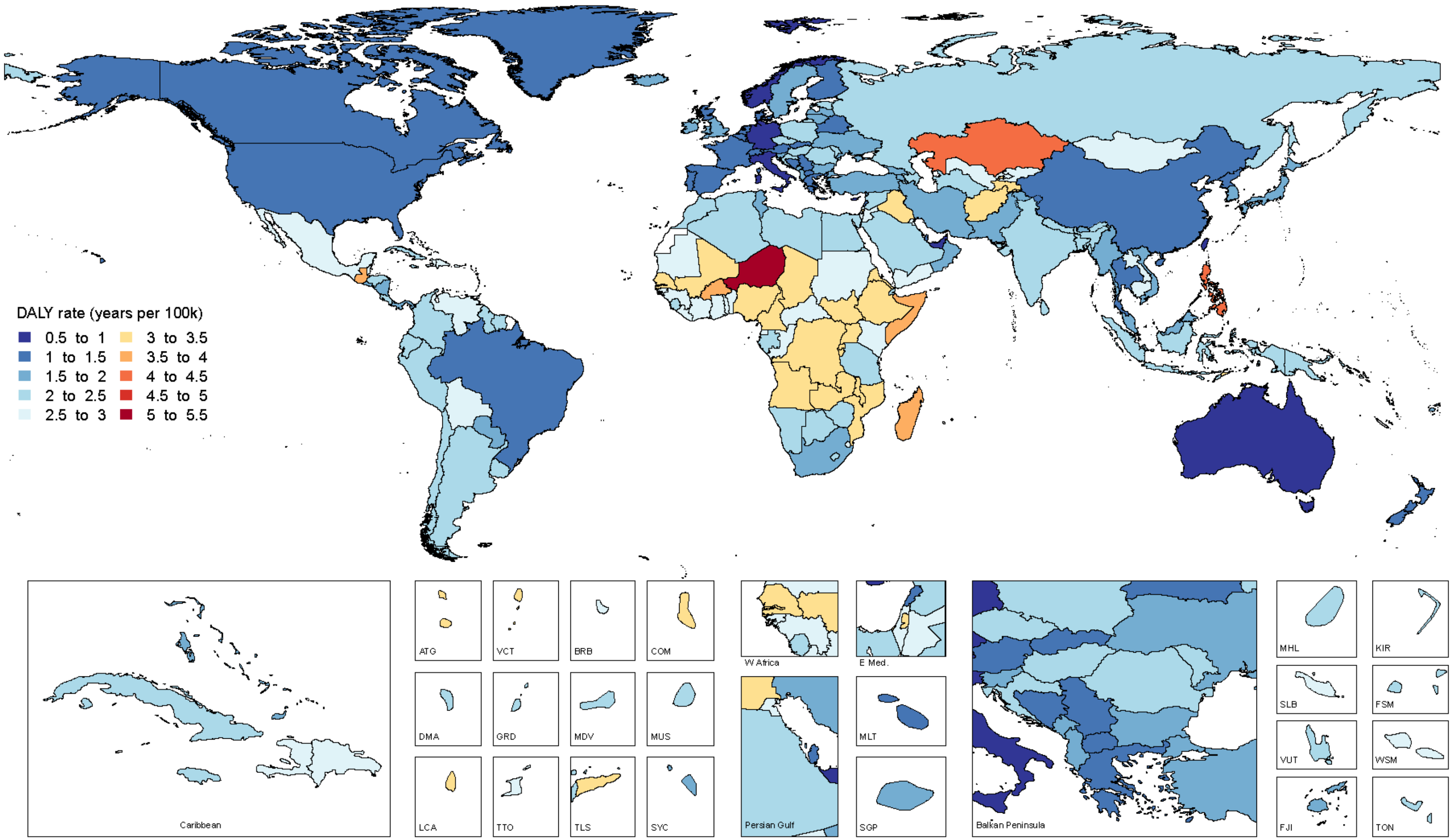

Appendix 2, Figure 4. World Map of age-standardised DALY rate (per 100,000 population) due to untreated deciduous caries in 2015, both sexes

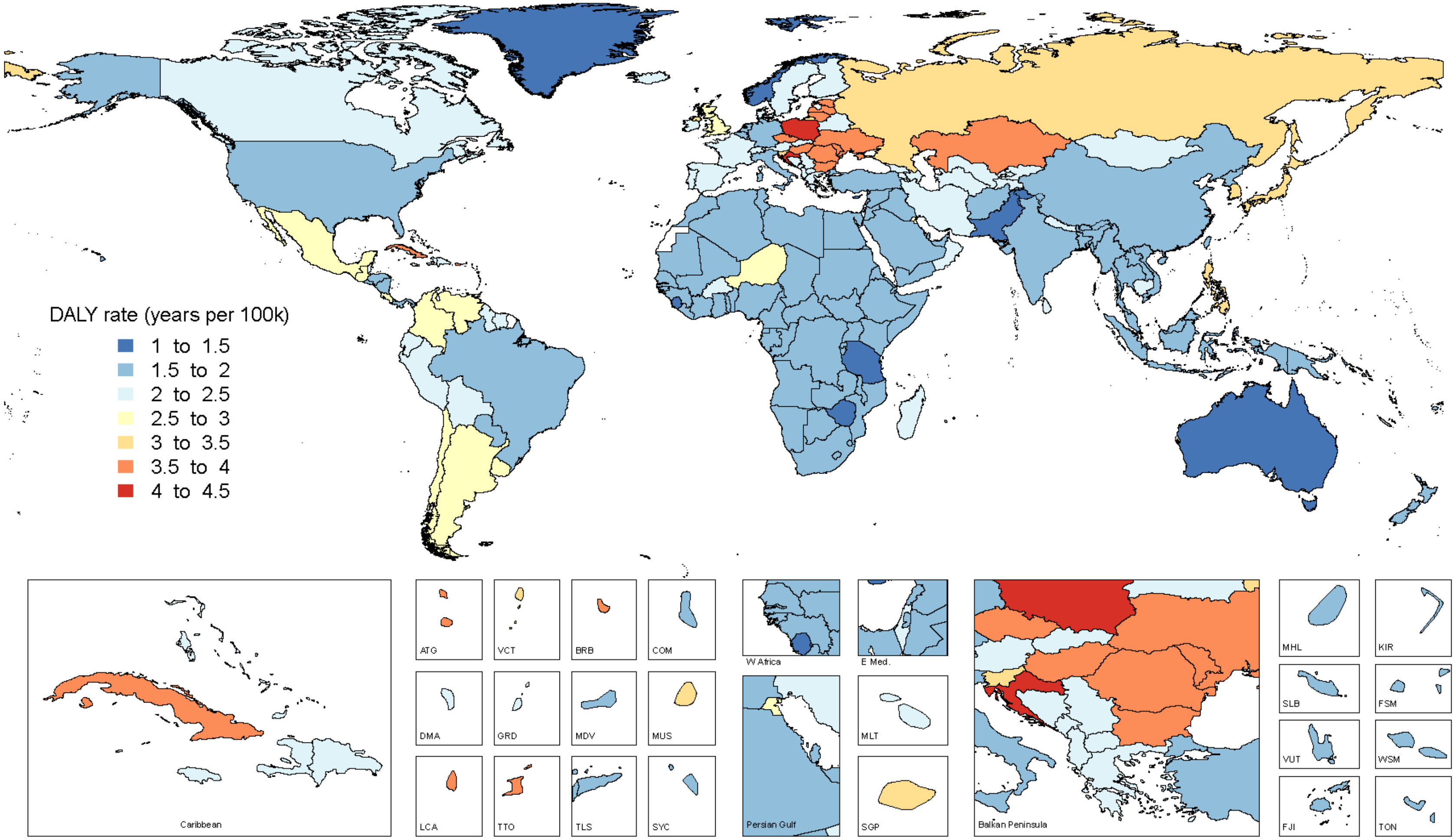

Appendix 2, Figure 5. World map of all-ages DALY rate (per 1,000 population) due to severe periodontal disease in 2015, both sexes

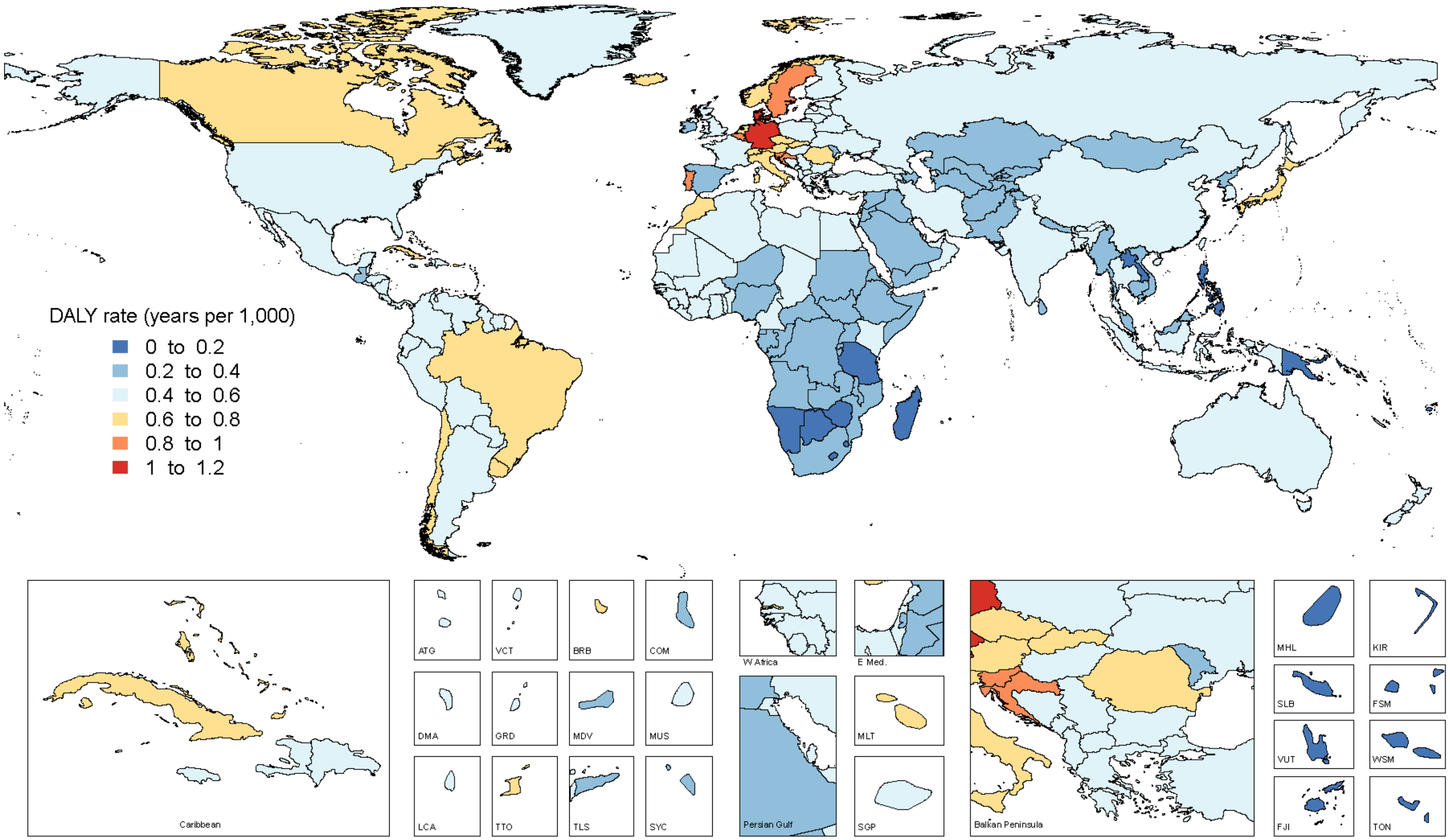

Appendix 2, Figure 6. World Map of age-standardised DALY rate (per 1,000 population) due to severe periodontal disease in 2015, both sexes

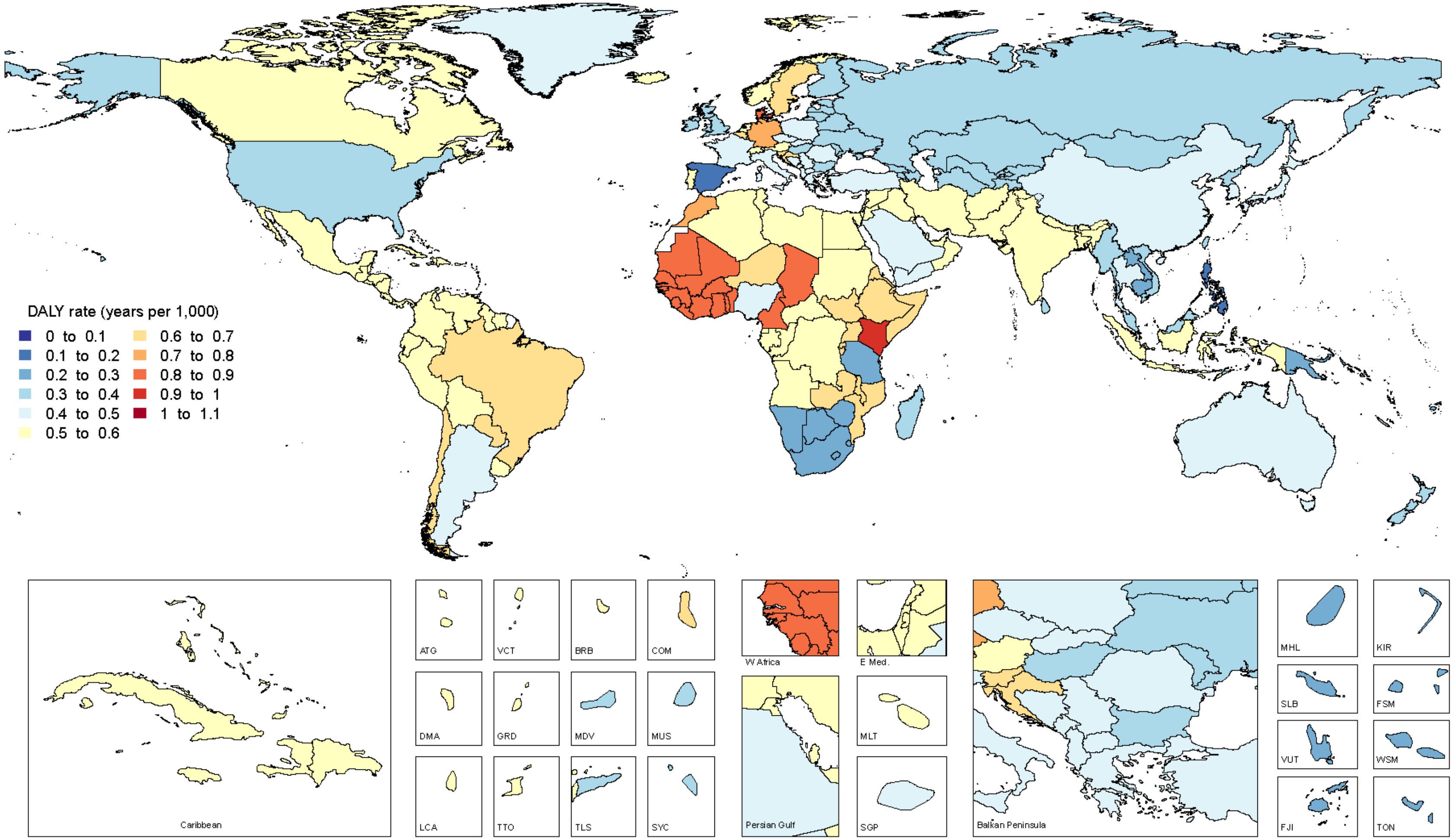

Appendix 2, Figure 7. World map of all-ages DALY rate (per 100,000 population) due to edentulism and severe tooth loss in 2015, both sexes

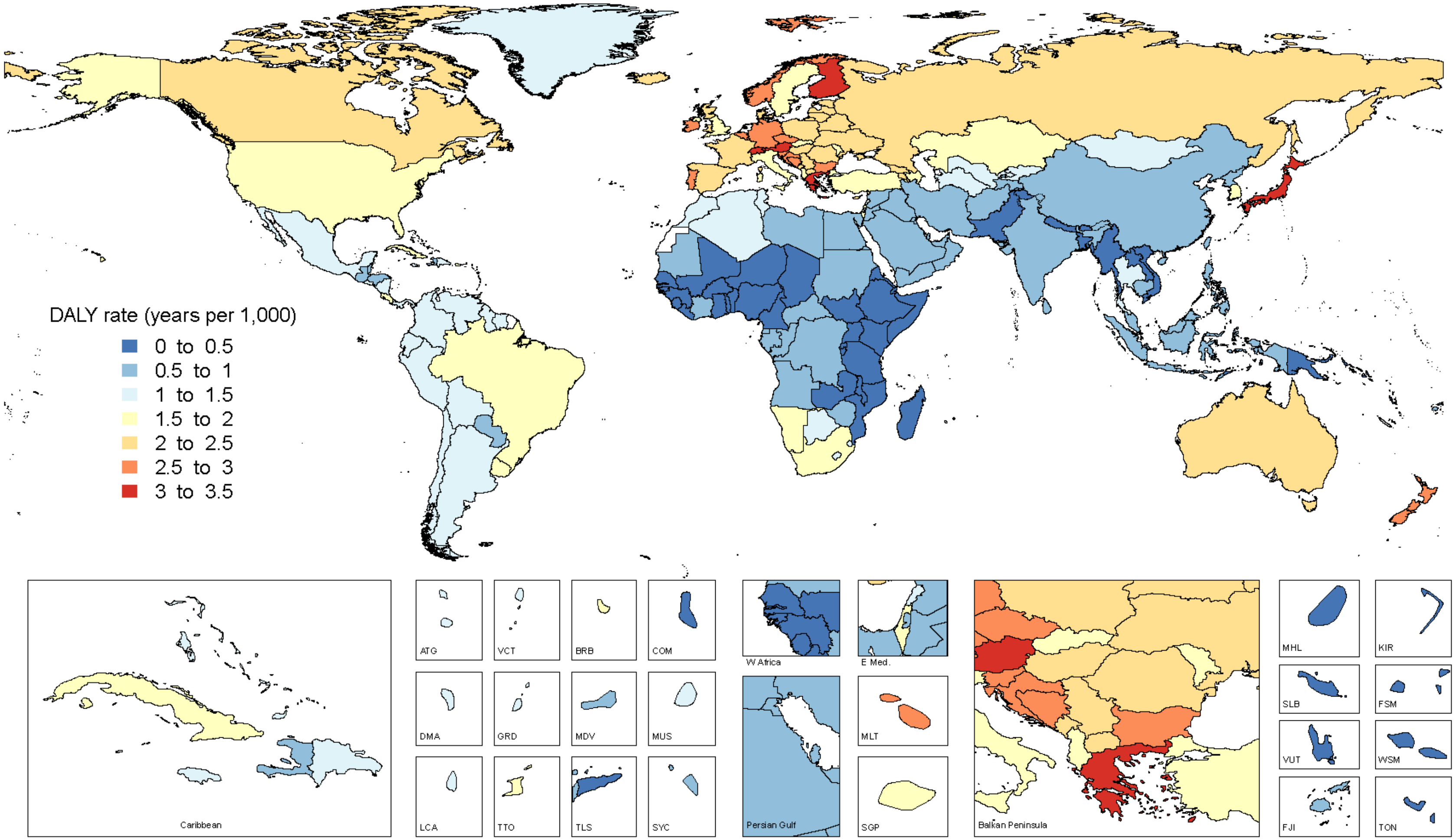

Appendix 2, Figure 8. World Map of age-standardised DALY rate (per 100,000 population) due to edentulism and severe tooth loss in 2015, both sexes

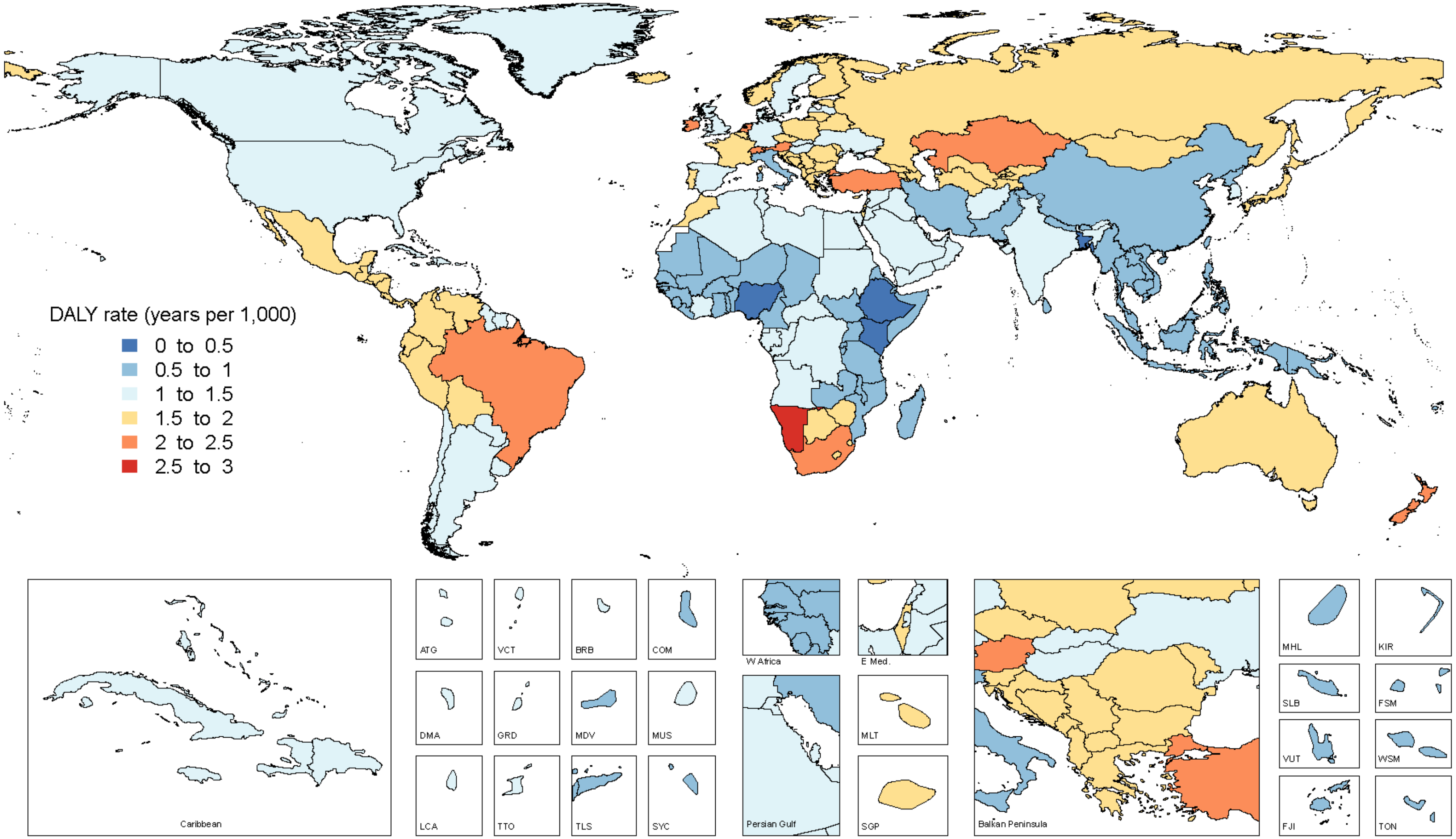

Appendix 2, Figure 9. World map of all-ages DALY rate (per 100,000 population) due to other oral conditions in 2015, both sexes

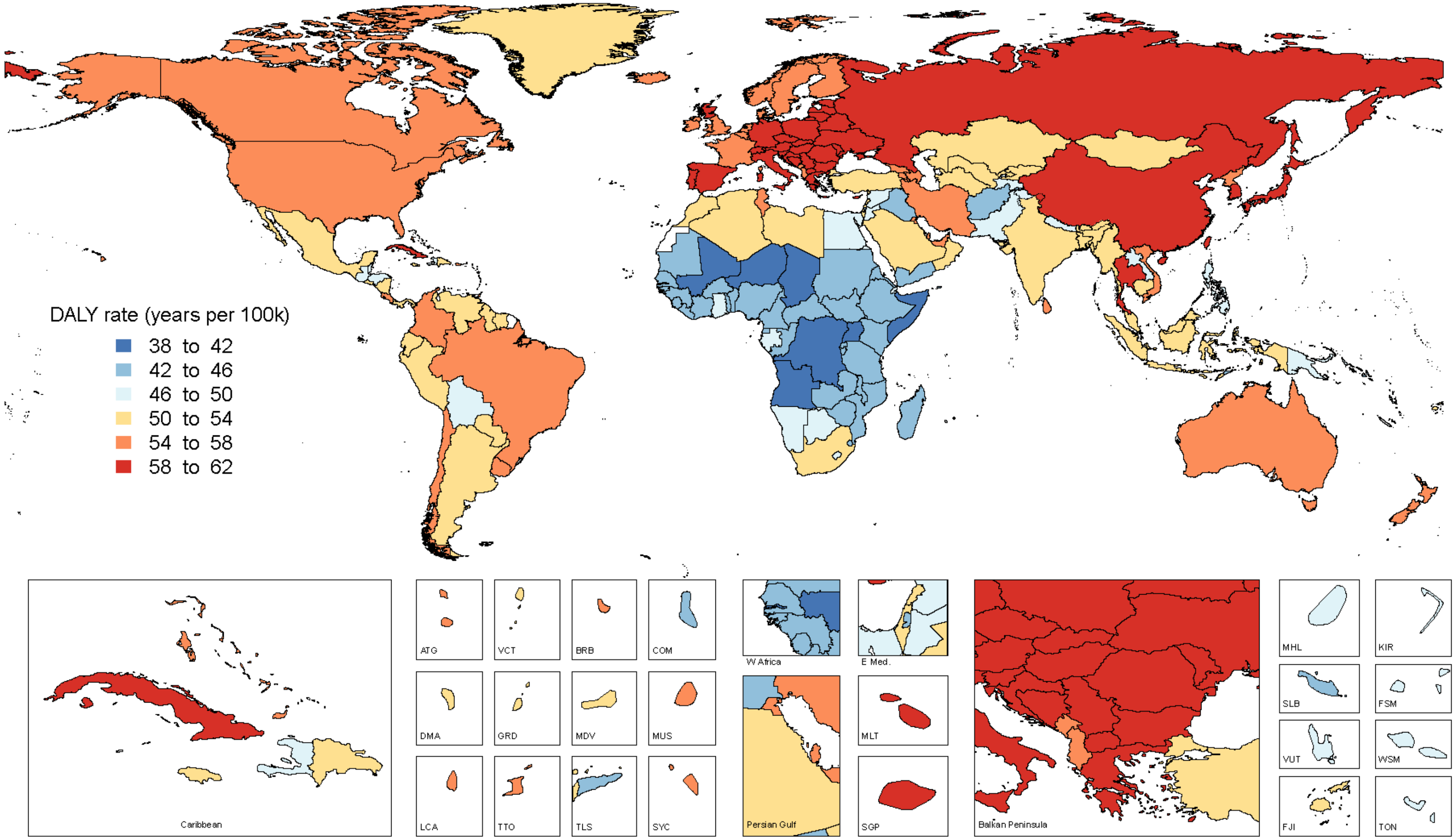

Appendix 2, Figure 10. World Map of age-standardised DALY rate (per 100,000 population) due to other oral conditions in 2015, both sexes

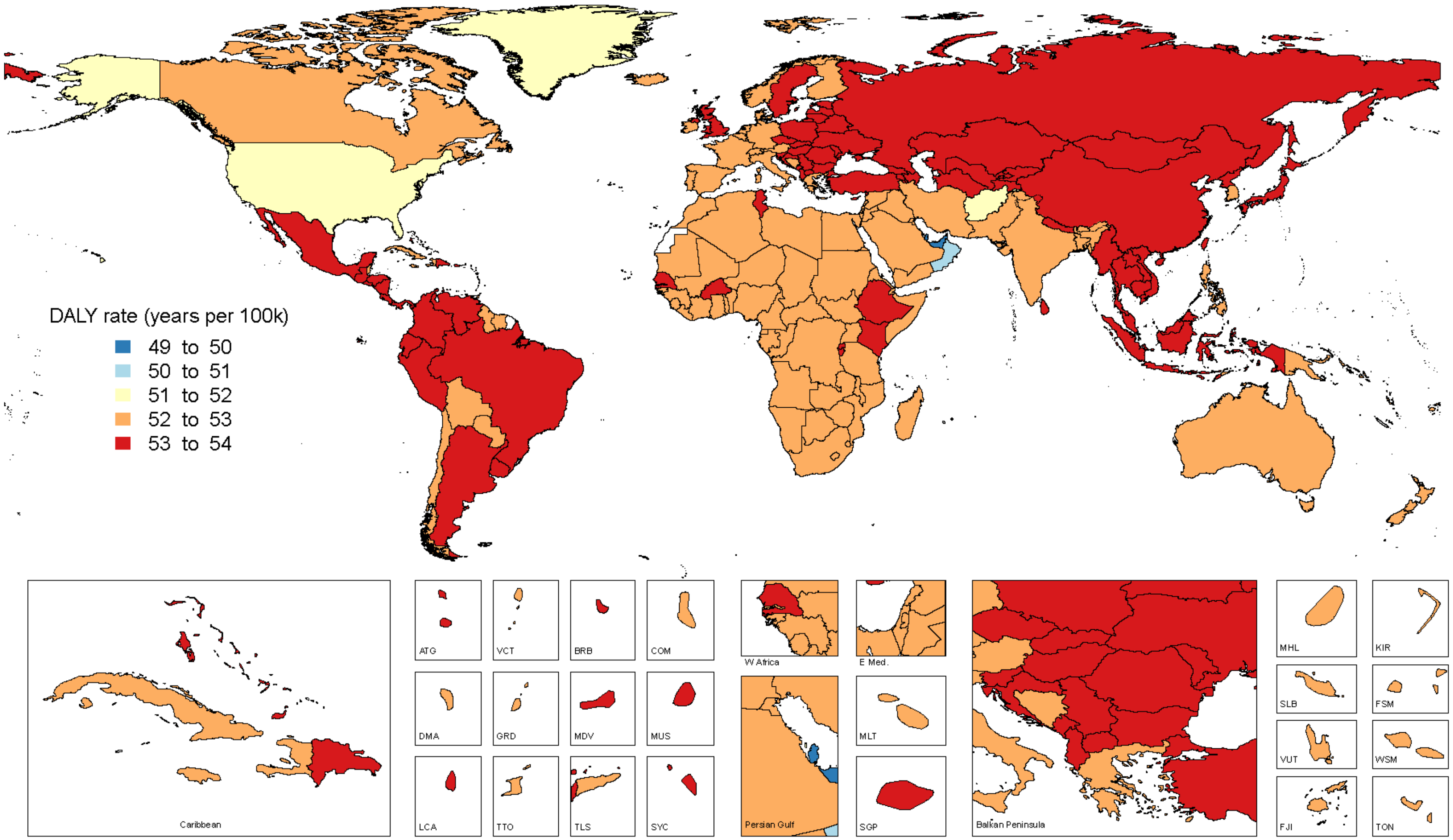

Appendix 2, Table 1. Prevalence, incidence, and YLD rates per 1,000 population (with 95% uncertainty intervals) for all oral conditions in 1990 and 2015, by country and by age

	Prevalence 1990	Prevalence 2005	Prevalence 2015	Incidence 1990	Incidence 2005	Incidence 2015	YLD rate 1990	YLD rate 2005	YLD rate 2015
**Global**									
Under-5 years (rate per capita)	0.394 (0.371 - 0.418)	0.382 (0.359 - 0.406)	0.387 (0.363 - 0.413)	0.554 (0.375 - 0.711)	0.526 (0.357 - 0.679)	0.542 (0.362 - 0.705)	0.101 (0.043 - 0.197)	0.097 (0.042 - 0.191)	0.099 (0.042 - 0.194)
5-14 years (rate per capita)	0.497 (0.476 - 0.513)	0.479 (0.457 - 0.498)	0.479 (0.456 - 0.498)	0.827 (0.656 - 1.045)	0.794 (0.629 - 1.007)	0.800 (0.623 - 1.023)	0.570 (0.321 - 0.926)	0.558 (0.315 - 0.901)	0.558 (0.315 - 0.899)
15 - 49 years (rate per capita)	0.457 (0.447 - 0.466)	0.457 (0.448 - 0.467)	0.463 (0.453 - 0.471)	0.752 (0.683 - 0.822)	0.745 (0.678 - 0.812)	0.749 (0.679 - 0.815)	1.669 (0.971 - 2.639)	1.721 (1.002 - 2.720)	1.772 (1.030 - 2.805)
50 - 69 years (rate per capita)	0.548 (0.536 - 0.559)	0.542 (0.532 - 0.552)	0.541 (0.530 - 0.552)	0.827 (0.739 - 0.912)	0.812 (0.726 - 0.902)	0.811 (0.723 - 0.901)	5.130 (3.193 - 7.659)	4.968 (3.090 - 7.419)	4.963 (3.087 - 7.410)
70+ years (rate per capita)	0.580 (0.571 - 0.589)	0.576 (0.567 - 0.584)	0.570 (0.561 - 0.578)	0.788 (0.708 - 0.866)	0.776 (0.698 - 0.852)	0.767 (0.692 - 0.842)	8.413 (5.513 - 11.934)	8.216 (5.376 - 11.669)	8.101 (5.313 - 11.512)
All ages (rate per capita)	0.474 (0.466 - 0.481)	0.472 (0.465 - 0.479)	0.478 (0.470 - 0.485)	0.754 (0.698 - 0.815)	0.744 (0.692 - 0.801)	0.750 (0.700 - 0.806)	1.950 (1.174 - 2.979)	2.125 (1.285 - 3.256)	2.299 (1.394 - 3.528)
Age-standardised (rate per capita)	0.484 (0.476 - 0.490)	0.478 (0.471 - 0.485)	0.480 (0.473 - 0.487)	0.764 (0.713 - 0.820)	0.750 (0.701 - 0.804)	0.750 (0.700 - 0.808)	2.450 (1.498 - 3.730)	2.410 (1.472 - 3.670)	2.405 (1.468 - 3.668)
Total number of cases (thousands)	2,512,817 (2,472,307 - 2,551,015)	3,076,695 (3,029,569 - 3,125,086)	3,521,907 (3,467,172 - 3,575,410)	4,000,869 (3,701,208 - 4,321,302)	4,853,261 (4,514,911 - 5,220,839)	5,529,726 (5,159,972 - 5,942,455)	10,342 (6,228 - 15,800)	13,854 (8,379 - 21,233)	16,949 (10,278 - 26,002)
**High-income**									
Under-5 years (rate per capita)	0.198 (0.183 - 0.218)	0.184 (0.167 - 0.204)	0.208 (0.185 - 0.234)	0.359 (0.184 - 0.586)	0.333 (0.166 - 0.565)	0.404 (0.188 - 0.700)	0.075 (0.030 - 0.154)	0.070 (0.028 - 0.140)	0.079 (0.031 - 0.159)
5-14 years (rate per capita)	0.423 (0.403 - 0.438)	0.391 (0.374 - 0.406)	0.411 (0.389 - 0.428)	0.624 (0.504 - 0.776)	0.586 (0.470 - 0.739)	0.613 (0.478 - 0.782)	0.611 (0.337 - 1.001)	0.583 (0.325 - 0.955)	0.597 (0.331 - 0.987)
15 - 49 years (rate per capita)	0.455 (0.447 - 0.462)	0.454 (0.446 - 0.461)	0.458 (0.450 - 0.465)	0.804 (0.741 - 0.867)	0.802 (0.740 - 0.863)	0.807 (0.745 - 0.869)	2.048 (1.193 - 3.253)	2.109 (1.230 - 3.347)	2.121 (1.237 - 3.373)
50 - 69 years (rate per capita)	0.559 (0.550 - 0.568)	0.549 (0.541 - 0.558)	0.548 (0.539 - 0.557)	0.902 (0.818 - 0.983)	0.891 (0.806 - 0.974)	0.890 (0.808 - 0.974)	6.101 (3.855 - 9.057)	5.911 (3.720 - 8.813)	5.974 (3.775 - 8.849)
70+ years (rate per capita)	0.586 (0.577 - 0.594)	0.580 (0.572 - 0.587)	0.574 (0.566 - 0.580)	0.828 (0.754 - 0.902)	0.819 (0.750 - 0.891)	0.811 (0.741 - 0.882)	9.673 (6.403 - 13.553)	9.468 (6.265 - 13.250)	9.340 (6.189 - 13.118)
All ages (rate per capita)	0.464 (0.459 - 0.470)	0.465 (0.461 - 0.470)	0.475 (0.471 - 0.481)	0.770 (0.729 - 0.815)	0.770 (0.728 - 0.811)	0.784 (0.741 - 0.828)	3.145 (1.952 - 4.730)	3.429 (2.141 - 5.140)	3.679 (2.312 - 5.474)
Age-standardised (rate per capita)	0.451 (0.445 - 0.457)	0.440 (0.435 - 0.445)	0.446 (0.440 - 0.452)	0.749 (0.705 - 0.797)	0.733 (0.689 - 0.780)	0.744 (0.696 - 0.798)	2.804 (1.733 - 4.231)	2.767 (1.710 - 4.174)	2.764 (1.710 - 4.162)
Total number of cases (thousands)	416,533 (411,993 - 421,685)	463,084 (458,635 - 468,208)	508,560 (503,596 - 514,346)	691,073 (654,194 - 731,144)	766,325 (725,460 - 807,544)	839,021 (793,229 - 885,636)	2,822 (1,752 - 4,244)	3,415 (2,132 - 5,119)	3,937 (2,474 - 5,858)
**High-income North America**									
Under-5 years (rate per capita)	0.185 (0.165 - 0.203)	0.165 (0.143 - 0.183)	0.195 (0.169 - 0.216)	0.367 (0.172 - 0.620)	0.311 (0.144 - 0.589)	0.398 (0.177 - 0.719)	0.070 (0.027 - 0.143)	0.062 (0.024 - 0.127)	0.073 (0.028 - 0.150)
5-14 years (rate per capita)	0.331 (0.309 - 0.351)	0.291 (0.273 - 0.310)	0.328 (0.308 - 0.352)	0.560 (0.422 - 0.738)	0.509 (0.383 - 0.684)	0.576 (0.436 - 0.762)	0.532 (0.298 - 0.881)	0.498 (0.283 - 0.812)	0.518 (0.295 - 0.853)
15 - 49 years (rate per capita)	0.390 (0.382 - 0.398)	0.373 (0.365 - 0.380)	0.382 (0.374 - 0.390)	0.794 (0.724 - 0.866)	0.763 (0.696 - 0.832)	0.779 (0.711 - 0.847)	1.963 (1.160 - 3.081)	1.952 (1.169 - 3.052)	1.939 (1.157 - 3.045)
50 - 69 years (rate per capita)	0.500 (0.491 - 0.508)	0.484 (0.475 - 0.493)	0.491 (0.481 - 0.499)	0.812 (0.726 - 0.901)	0.800 (0.709 - 0.898)	0.811 (0.724 - 0.908)	5.973 (3.851 - 8.712)	5.361 (3.415 - 7.906)	5.503 (3.526 - 8.083)
70+ years (rate per capita)	0.526 (0.517 - 0.534)	0.513 (0.505 - 0.520)	0.509 (0.501 - 0.516)	0.713 (0.635 - 0.797)	0.701 (0.628 - 0.779)	0.696 (0.627 - 0.774)	8.821 (5.846 - 12.285)	8.213 (5.433 - 11.477)	8.146 (5.412 - 11.373)
All ages (rate per capita)	0.396 (0.390 - 0.402)	0.383 (0.379 - 0.388)	0.403 (0.399 - 0.409)	0.725 (0.677 - 0.779)	0.700 (0.655 - 0.749)	0.730 (0.684 - 0.781)	2.873 (1.811 - 4.262)	2.892 (1.816 - 4.286)	3.137 (1.975 - 4.640)
Age-standardised (rate per capita)	0.384 (0.378 - 0.390)	0.366 (0.361 - 0.372)	0.381 (0.375 - 0.387)	0.703 (0.653 - 0.759)	0.678 (0.631 - 0.733)	0.706 (0.653 - 0.765)	2.649 (1.659 - 3.956)	2.528 (1.582 - 3.767)	2.546 (1.596 - 3.797)
Total number of cases (thousands)	109,992 (108,417 - 111,710)	125,718 (124,386 - 127,405)	145,145 (143,457 - 147,002)	201,287 (188,058 - 216,381)	229,824 (214,894 - 245,953)	262,775 (246,022 - 281,185)	798 (503 - 1,184)	949 (596 - 1,407)	1,129 (711 - 1,670)
**Canada**									
Under-5 years (rate per capita)	0.209 (0.161 - 0.258)	0.165 (0.113 - 0.226)	0.211 (0.162 - 0.260)	0.432 (0.181 - 0.738)	0.252 (0.111 - 0.620)	0.434 (0.181 - 0.740)	0.079 (0.030 - 0.164)	0.061 (0.022 - 0.142)	0.079 (0.029 - 0.165)
5-14 years (rate per capita)	0.347 (0.301 - 0.405)	0.269 (0.225 - 0.318)	0.344 (0.297 - 0.401)	0.572 (0.418 - 0.777)	0.468 (0.340 - 0.661)	0.570 (0.417 - 0.773)	0.525 (0.294 - 0.848)	0.492 (0.279 - 0.789)	0.525 (0.296 - 0.862)
15 - 49 years (rate per capita)	0.399 (0.382 - 0.418)	0.398 (0.383 - 0.417)	0.397 (0.380 - 0.416)	0.772 (0.703 - 0.848)	0.765 (0.696 - 0.839)	0.763 (0.697 - 0.836)	2.120 (1.232 - 3.371)	2.206 (1.297 - 3.492)	2.175 (1.279 - 3.467)
50 - 69 years (rate per capita)	0.524 (0.501 - 0.547)	0.518 (0.494 - 0.545)	0.520 (0.497 - 0.544)	0.794 (0.703 - 0.893)	0.794 (0.701 - 0.896)	0.795 (0.703 - 0.894)	6.204 (3.879 - 9.244)	5.905 (3.673 - 8.868)	6.020 (3.745 - 9.020)
70+ years (rate per capita)	0.542 (0.523 - 0.562)	0.534 (0.516 - 0.555)	0.528 (0.511 - 0.547)	0.696 (0.614 - 0.778)	0.685 (0.608 - 0.764)	0.675 (0.602 - 0.754)	9.029 (5.942 - 12.804)	8.822 (5.809 - 12.529)	8.662 (5.733 - 12.322)
All ages (rate per capita)	0.411 (0.397 - 0.426)	0.409 (0.396 - 0.422)	0.428 (0.414 - 0.441)	0.720 (0.667 - 0.776)	0.700 (0.652 - 0.752)	0.726 (0.677 - 0.778)	2.980 (1.836 - 4.500)	3.315 (2.062 - 4.945)	3.587 (2.229 - 5.356)
Age-standardised (rate per capita)	0.398 (0.382 - 0.413)	0.380 (0.365 - 0.394)	0.396 (0.381 - 0.412)	0.694 (0.638 - 0.757)	0.661 (0.610 - 0.724)	0.694 (0.637 - 0.756)	2.779 (1.710 - 4.209)	2.756 (1.706 - 4.134)	2.765 (1.706 - 4.151)
Total number of cases (thousands)	11,357 (10,948 - 11,765)	13,180 (12,742 - 13,593)	15,469 (14,972 - 15,950)	19,873 (18,414 - 21,429)	22,563 (20,996 - 24,207)	26,248 (24,464 - 28,116)	82 (51 - 124)	107 (66 - 159)	130 (81 - 194)
**Greenland**									
Under-5 years (rate per capita)	0.199 (0.148 - 0.250)	0.202 (0.151 - 0.252)	0.202 (0.150 - 0.253)	0.409 (0.167 - 0.695)	0.420 (0.172 - 0.713)	0.419 (0.171 - 0.711)	0.046 (0.018 - 0.094)	0.047 (0.019 - 0.099)	0.047 (0.018 - 0.098)
5-14 years (rate per capita)	0.343 (0.290 - 0.399)	0.328 (0.277 - 0.385)	0.332 (0.285 - 0.392)	0.582 (0.418 - 0.796)	0.555 (0.402 - 0.757)	0.563 (0.406 - 0.766)	0.434 (0.252 - 0.692)	0.431 (0.249 - 0.677)	0.431 (0.249 - 0.676)
15 - 49 years (rate per capita)	0.389 (0.371 - 0.408)	0.389 (0.371 - 0.407)	0.386 (0.370 - 0.404)	0.742 (0.676 - 0.817)	0.734 (0.671 - 0.802)	0.728 (0.665 - 0.794)	1.801 (1.068 - 2.787)	1.960 (1.169 - 3.036)	1.903 (1.131 - 2.948)
50 - 69 years (rate per capita)	0.495 (0.470 - 0.522)	0.495 (0.469 - 0.523)	0.494 (0.470 - 0.521)	0.769 (0.680 - 0.867)	0.765 (0.677 - 0.862)	0.766 (0.676 - 0.863)	5.297 (3.332 - 7.849)	5.332 (3.375 - 7.852)	5.260 (3.295 - 7.792)
70+ years (rate per capita)	0.548 (0.527 - 0.569)	0.551 (0.531 - 0.576)	0.551 (0.530 - 0.575)	0.705 (0.611 - 0.794)	0.708 (0.611 - 0.800)	0.706 (0.608 - 0.801)	9.128 (6.059 - 12.848)	9.236 (6.130 - 12.932)	9.269 (6.155 - 13.076)
All ages (rate per capita)	0.360 (0.341 - 0.377)	0.370 (0.354 - 0.386)	0.382 (0.366 - 0.397)	0.658 (0.590 - 0.741)	0.659 (0.596 - 0.735)	0.669 (0.607 - 0.738)	1.771 (1.085 - 2.667)	2.126 (1.317 - 3.188)	2.437 (1.519 - 3.656)
Age-standardised (rate per capita)	0.386 (0.371 - 0.401)	0.386 (0.370 - 0.400)	0.387 (0.370 - 0.401)	0.672 (0.617 - 0.737)	0.672 (0.617 - 0.736)	0.672 (0.617 - 0.736)	2.524 (1.590 - 3.743)	2.522 (1.583 - 3.757)	2.525 (1.586 - 3.732)
Total number of cases (thousands)	20 (19 - 21)	20 (19 - 21)	21 (20 - 21)	36 (33 - 41)	36 (33 - 40)	36 (33 - 40)	0 (0 - 0)	0 (0 - 0)	0 (0 - 0)
**United States**									
Under-5 years (rate per capita)	0.182 (0.162 - 0.200)	0.165 (0.143 - 0.184)	0.193 (0.168 - 0.214)	0.360 (0.172 - 0.609)	0.316 (0.145 - 0.587)	0.395 (0.177 - 0.718)	0.069 (0.026 - 0.140)	0.062 (0.023 - 0.127)	0.072 (0.027 - 0.149)
5-14 years (rate per capita)	0.329 (0.309 - 0.349)	0.293 (0.275 - 0.313)	0.326 (0.307 - 0.350)	0.559 (0.420 - 0.734)	0.512 (0.387 - 0.685)	0.576 (0.438 - 0.761)	0.532 (0.299 - 0.879)	0.499 (0.284 - 0.811)	0.517 (0.294 - 0.852)
15 - 49 years (rate per capita)	0.389 (0.380 - 0.397)	0.370 (0.362 - 0.377)	0.380 (0.372 - 0.389)	0.796 (0.726 - 0.869)	0.763 (0.695 - 0.832)	0.780 (0.712 - 0.849)	1.945 (1.155 - 3.052)	1.924 (1.158 - 3.011)	1.912 (1.143 - 3.006)
50 - 69 years (rate per capita)	0.497 (0.488 - 0.505)	0.480 (0.471 - 0.489)	0.487 (0.477 - 0.496)	0.814 (0.726 - 0.903)	0.801 (0.710 - 0.897)	0.813 (0.725 - 0.912)	5.947 (3.849 - 8.673)	5.298 (3.382 - 7.794)	5.443 (3.494 - 7.984)
70+ years (rate per capita)	0.524 (0.515 - 0.533)	0.511 (0.502 - 0.518)	0.507 (0.499 - 0.514)	0.714 (0.637 - 0.799)	0.703 (0.630 - 0.781)	0.698 (0.629 - 0.776)	8.800 (5.839 - 12.232)	8.142 (5.404 - 11.354)	8.083 (5.386 - 11.252)
All ages (rate per capita)	0.394 (0.389 - 0.400)	0.380 (0.376 - 0.386)	0.401 (0.396 - 0.406)	0.725 (0.678 - 0.779)	0.700 (0.655 - 0.749)	0.731 (0.684 - 0.783)	2.862 (1.808 - 4.242)	2.846 (1.789 - 4.219)	3.087 (1.949 - 4.570)
Age-standardised (rate per capita)	0.383 (0.376 - 0.389)	0.365 (0.359 - 0.370)	0.379 (0.373 - 0.384)	0.705 (0.654 - 0.761)	0.680 (0.632 - 0.735)	0.707 (0.655 - 0.768)	2.634 (1.650 - 3.920)	2.502 (1.567 - 3.724)	2.519 (1.582 - 3.752)
Total number of cases (thousands)	98,567 (97,136 - 99,998)	112,470 (111,237 - 114,176)	129,606 (128,063 - 131,310)	181,289 (169,497 - 194,791)	207,135 (193,692 - 221,534)	236,399 (221,186 - 253,251)	715 (452 - 1,060)	842 (529 - 1,248)	999 (631 - 1,478)
**Australasia**									
Under-5 years (rate per capita)	0.140 (0.099 - 0.191)	0.108 (0.091 - 0.127)	0.116 (0.089 - 0.146)	0.283 (0.109 - 0.686)	0.176 (0.113 - 0.275)	0.217 (0.101 - 0.449)	0.051 (0.018 - 0.113)	0.041 (0.017 - 0.082)	0.043 (0.016 - 0.094)
5-14 years (rate per capita)	0.248 (0.206 - 0.285)	0.232 (0.214 - 0.253)	0.237 (0.197 - 0.273)	0.313 (0.218 - 0.443)	0.300 (0.229 - 0.396)	0.298 (0.211 - 0.417)	0.448 (0.260 - 0.711)	0.440 (0.258 - 0.702)	0.444 (0.261 - 0.709)
15 - 49 years (rate per capita)	0.316 (0.303 - 0.329)	0.321 (0.309 - 0.334)	0.315 (0.303 - 0.329)	0.409 (0.366 - 0.450)	0.416 (0.377 - 0.455)	0.411 (0.370 - 0.452)	2.059 (1.242 - 3.190)	2.121 (1.290 - 3.251)	2.095 (1.272 - 3.204)
50 - 69 years (rate per capita)	0.450 (0.434 - 0.467)	0.445 (0.427 - 0.463)	0.444 (0.427 - 0.460)	0.444 (0.395 - 0.503)	0.450 (0.397 - 0.516)	0.444 (0.395 - 0.503)	6.904 (4.460 - 10.103)	6.432 (4.178 - 9.472)	6.693 (4.339 - 9.815)
70+ years (rate per capita)	0.522 (0.509 - 0.536)	0.513 (0.501 - 0.527)	0.507 (0.495 - 0.520)	0.382 (0.338 - 0.429)	0.378 (0.338 - 0.420)	0.368 (0.329 - 0.411)	11.542 (7.701 - 15.985)	11.091 (7.372 - 15.371)	10.974 (7.313 - 15.177)
All ages (rate per capita)	0.331 (0.318 - 0.343)	0.339 (0.329 - 0.347)	0.341 (0.330 - 0.351)	0.390 (0.354 - 0.435)	0.389 (0.362 - 0.418)	0.389 (0.359 - 0.423)	3.195 (2.044 - 4.714)	3.471 (2.224 - 5.075)	3.667 (2.359 - 5.332)
Age-standardised (rate per capita)	0.324 (0.309 - 0.337)	0.319 (0.310 - 0.326)	0.317 (0.305 - 0.329)	0.384 (0.346 - 0.436)	0.376 (0.348 - 0.408)	0.375 (0.342 - 0.417)	3.035 (1.940 - 4.481)	2.965 (1.894 - 4.346)	2.990 (1.915 - 4.369)
Total number of cases (thousands)	6,751 (6,478 - 6,996)	8,306 (8,076 - 8,510)	9,845 (9,544 - 10,142)	7,948 (7,227 - 8,872)	9,532 (8,876 - 10,249)	11,232 (10,372 - 12,220)	65 (42 - 96)	85 (55 - 124)	106 (68 - 154)
**Australia**									
Under-5 years (rate per capita)	0.139 (0.091 - 0.198)	0.104 (0.087 - 0.123)	0.110 (0.079 - 0.142)	0.280 (0.107 - 0.685)	0.165 (0.115 - 0.224)	0.201 (0.097 - 0.412)	0.051 (0.018 - 0.112)	0.040 (0.017 - 0.081)	0.041 (0.015 - 0.088)
5-14 years (rate per capita)	0.247 (0.199 - 0.288)	0.230 (0.210 - 0.254)	0.234 (0.189 - 0.273)	0.312 (0.218 - 0.443)	0.290 (0.226 - 0.379)	0.294 (0.208 - 0.412)	0.448 (0.259 - 0.711)	0.441 (0.258 - 0.704)	0.443 (0.259 - 0.706)
15 - 49 years (rate per capita)	0.318 (0.303 - 0.334)	0.324 (0.309 - 0.339)	0.316 (0.303 - 0.332)	0.410 (0.368 - 0.452)	0.418 (0.378 - 0.456)	0.412 (0.370 - 0.454)	2.066 (1.239 - 3.231)	2.118 (1.281 - 3.253)	2.086 (1.262 - 3.196)
50 - 69 years (rate per capita)	0.452 (0.433 - 0.472)	0.448 (0.427 - 0.469)	0.446 (0.427 - 0.463)	0.444 (0.395 - 0.504)	0.452 (0.396 - 0.518)	0.445 (0.396 - 0.505)	6.839 (4.417 - 10.033)	6.361 (4.118 - 9.388)	6.627 (4.289 - 9.690)
70+ years (rate per capita)	0.520 (0.505 - 0.535)	0.511 (0.497 - 0.527)	0.504 (0.491 - 0.520)	0.382 (0.337 - 0.431)	0.379 (0.339 - 0.422)	0.368 (0.327 - 0.411)	11.346 (7.564 - 15.685)	10.901 (7.248 - 15.127)	10.797 (7.202 - 14.951)
All ages (rate per capita)	0.333 (0.318 - 0.346)	0.341 (0.330 - 0.350)	0.341 (0.329 - 0.353)	0.390 (0.355 - 0.436)	0.388 (0.362 - 0.415)	0.388 (0.359 - 0.421)	3.180 (2.025 - 4.701)	3.462 (2.217 - 5.068)	3.631 (2.341 - 5.285)
Age-standardised (rate per capita)	0.324 (0.309 - 0.339)	0.319 (0.310 - 0.328)	0.317 (0.303 - 0.330)	0.384 (0.346 - 0.437)	0.374 (0.347 - 0.402)	0.373 (0.341 - 0.413)	3.012 (1.916 - 4.455)	2.937 (1.873 - 4.310)	2.964 (1.896 - 4.334)

	Prevalence 1990	Prevalence 2005	Prevalence 2015	Incidence 1990	Incidence 2005	Incidence 2015	YLD rate 1990	YLD rate 2005	YLD rate 2015
Total number of cases (thousands)	5,663 (5,407 - 5,899)	6,948 (6,732 - 7,141)	8,298 (7,997 - 8,584)	6,642 (6,041 - 7,419)	7,918 (7,382 - 8,463)	9,440 (8,725 - 10,244)	54 (34 - 80)	71 (45 - 103)	88 (57 - 129)
**New Zealand**									
Under-5 years (rate per capita)	0.145 (0.101 - 0.201)	0.122 (0.084 - 0.177)	0.148 (0.101 - 0.206)	0.295 (0.114 - 0.693)	0.225 (0.087 - 0.581)	0.299 (0.116 - 0.703)	0.053 (0.019 - 0.119)	0.046 (0.016 - 0.106)	0.054 (0.019 - 0.117)
5-14 years (rate per capita)	0.251 (0.209 - 0.291)	0.241 (0.210 - 0.272)	0.255 (0.208 - 0.295)	0.315 (0.221 - 0.442)	0.344 (0.244 - 0.518)	0.319 (0.222 - 0.451)	0.448 (0.258 - 0.711)	0.434 (0.255 - 0.690)	0.451 (0.261 - 0.719)
15 - 49 years (rate per capita)	0.303 (0.289 - 0.319)	0.309 (0.295 - 0.325)	0.306 (0.293 - 0.320)	0.404 (0.364 - 0.446)	0.409 (0.369 - 0.450)	0.405 (0.366 - 0.444)	2.022 (1.251 - 3.064)	2.139 (1.311 - 3.246)	2.144 (1.309 - 3.254)
50 - 69 years (rate per capita)	0.440 (0.422 - 0.459)	0.430 (0.410 - 0.449)	0.435 (0.417 - 0.453)	0.444 (0.396 - 0.502)	0.443 (0.390 - 0.505)	0.443 (0.394 - 0.503)	7.240 (4.692 - 10.468)	6.804 (4.439 - 9.924)	7.036 (4.595 - 10.188)
70+ years (rate per capita)	0.534 (0.520 - 0.549)	0.523 (0.509 - 0.538)	0.519 (0.504 - 0.535)	0.381 (0.334 - 0.430)	0.372 (0.333 - 0.416)	0.368 (0.328 - 0.414)	12.517 (8.332 - 17.377)	12.107 (8.059 - 16.864)	11.943 (7.997 - 16.548)
All ages (rate per capita)	0.322 (0.309 - 0.335)	0.328 (0.317 - 0.339)	0.339 (0.326 - 0.351)	0.387 (0.351 - 0.434)	0.390 (0.356 - 0.444)	0.393 (0.359 - 0.433)	3.273 (2.121 - 4.757)	3.515 (2.272 - 5.163)	3.857 (2.502 - 5.601)
Age-standardised (rate per capita)	0.319 (0.305 - 0.332)	0.315 (0.303 - 0.327)	0.319 (0.305 - 0.333)	0.385 (0.346 - 0.437)	0.384 (0.348 - 0.450)	0.385 (0.346 - 0.439)	3.153 (2.038 - 4.603)	3.108 (2.018 - 4.558)	3.131 (2.023 - 4.570)
Total number of cases (thousands)	1,088 (1,043 - 1,130)	1,358 (1,312 - 1,403)	1,547 (1,489 - 1,601)	1,306 (1,184 - 1,464)	1,615 (1,473 - 1,838)	1,792 (1,639 - 1,977)	11 (7 - 16)	15 (9 - 21)	18 (11 - 26)
**High-income Asia Pacific**									
Under-5 years (rate per capita)	0.341 (0.318 - 0.366)	0.324 (0.301 - 0.348)	0.351 (0.328 - 0.379)	0.570 (0.336 - 0.784)	0.545 (0.314 - 0.761)	0.570 (0.344 - 0.779)	0.131 (0.056 - 0.265)	0.125 (0.053 - 0.250)	0.135 (0.058 - 0.274)
5-14 years (rate per capita)	0.417 (0.392 - 0.439)	0.417 (0.392 - 0.439)	0.418 (0.396 - 0.439)	0.749 (0.601 - 0.967)	0.739 (0.597 - 0.938)	0.748 (0.597 - 0.964)	0.558 (0.312 - 0.907)	0.551 (0.308 - 0.886)	0.559 (0.313 - 0.908)
15 - 49 years (rate per capita)	0.397 (0.388 - 0.407)	0.410 (0.400 - 0.419)	0.415 (0.406 - 0.425)	0.807 (0.735 - 0.881)	0.829 (0.757 - 0.903)	0.836 (0.761 - 0.912)	1.968 (1.163 - 3.079)	2.067 (1.217 - 3.243)	2.144 (1.263 - 3.351)
50 - 69 years (rate per capita)	0.518 (0.509 - 0.527)	0.520 (0.511 - 0.529)	0.523 (0.513 - 0.533)	0.860 (0.763 - 0.967)	0.859 (0.757 - 0.965)	0.858 (0.763 - 0.962)	5.958 (3.788 - 8.850)	6.154 (3.926 - 9.091)	6.386 (4.087 - 9.390)
70+ years (rate per capita)	0.553 (0.544 - 0.561)	0.550 (0.542 - 0.557)	0.542 (0.535 - 0.548)	0.748 (0.668 - 0.831)	0.740 (0.662 - 0.820)	0.729 (0.654 - 0.806)	9.870 (6.530 - 13.760)	9.859 (6.515 - 13.711)	9.631 (6.373 - 13.472)
All ages (rate per capita)	0.431 (0.425 - 0.437)	0.451 (0.445 - 0.457)	0.462 (0.456 - 0.467)	0.791 (0.738 - 0.844)	0.803 (0.751 - 0.853)	0.806 (0.755 - 0.856)	2.960 (1.840 - 4.429)	3.767 (2.387 - 5.605)	4.229 (2.698 - 6.221)
Age-standardised (rate per capita)	0.428 (0.422 - 0.435)	0.426 (0.419 - 0.433)	0.430 (0.423 - 0.436)	0.783 (0.729 - 0.842)	0.776 (0.724 - 0.835)	0.782 (0.727 - 0.839)	2.769 (1.724 - 4.147)	2.794 (1.734 - 4.199)	2.790 (1.734 - 4.175)
Total number of cases (thousands)	72,740 (71,717 - 73,788)	79,949 (78,923 - 81,002)	84,431 (83,386 - 85,375)	133,590 (124,612 - 142,622)	142,416 (133,206 - 151,268)	147,400 (138,045 - 156,564)	500 (311 - 748)	668 (423 - 994)	774 (494 - 1,138)
**Brunei**									
Under-5 years (rate per capita)	0.326 (0.275 - 0.373)	0.333 (0.285 - 0.377)	0.333 (0.281 - 0.380)	0.544 (0.315 - 0.753)	0.554 (0.321 - 0.766)	0.554 (0.321 - 0.766)	0.077 (0.033 - 0.157)	0.079 (0.033 - 0.155)	0.079 (0.034 - 0.156)
5-14 years (rate per capita)	0.453 (0.403 - 0.509)	0.439 (0.389 - 0.496)	0.427 (0.379 - 0.479)	0.748 (0.589 - 0.966)	0.730 (0.574 - 0.947)	0.714 (0.564 - 0.924)	0.463 (0.267 - 0.736)	0.460 (0.266 - 0.733)	0.458 (0.266 - 0.724)
15 - 49 years (rate per capita)	0.397 (0.378 - 0.417)	0.402 (0.384 - 0.421)	0.405 (0.388 - 0.425)	0.765 (0.697 - 0.842)	0.772 (0.703 - 0.847)	0.776 (0.707 - 0.850)	1.690 (1.016 - 2.620)	1.780 (1.054 - 2.749)	1.842 (1.104 - 2.844)
50 - 69 years (rate per capita)	0.510 (0.482 - 0.538)	0.506 (0.478 - 0.534)	0.508 (0.480 - 0.536)	0.818 (0.730 - 0.925)	0.818 (0.725 - 0.927)	0.820 (0.729 - 0.930)	5.467 (3.503 - 8.070)	5.303 (3.366 - 7.845)	5.400 (3.406 - 7.985)
70+ years (rate per capita)	0.549 (0.530 - 0.569)	0.551 (0.532 - 0.570)	0.551 (0.533 - 0.572)	0.712 (0.632 - 0.797)	0.717 (0.638 - 0.804)	0.719 (0.640 - 0.806)	9.646 (6.368 - 13.434)	9.704 (6.429 - 13.482)	9.714 (6.440 - 13.495)
All ages (rate per capita)	0.409 (0.390 - 0.425)	0.416 (0.399 - 0.430)	0.422 (0.408 - 0.437)	0.732 (0.669 - 0.803)	0.747 (0.690 - 0.810)	0.754 (0.699 - 0.810)	1.585 (0.971 - 2.412)	1.866 (1.136 - 2.817)	2.240 (1.386 - 3.366)
Age-standardised (rate per capita)	0.430 (0.414 - 0.443)	0.430 (0.415 - 0.443)	0.429 (0.415 - 0.443)	0.745 (0.693 - 0.802)	0.746 (0.694 - 0.803)	0.746 (0.693 - 0.803)	2.628 (1.656 - 3.870)	2.624 (1.644 - 3.855)	2.623 (1.657 - 3.856)
Total number of cases (thousands)	107 (102 - 111)	151 (145 - 157)	179 (172 - 185)	191 (175 - 210)	272 (251 - 295)	319 (296 - 343)	0 (0 - 1)	1 (0 - 1)	1 (1 - 1)
**Japan**									
Under-5 years (rate per capita)	0.346 (0.327 - 0.371)	0.323 (0.305 - 0.345)	0.361 (0.341 - 0.388)	0.573 (0.340 - 0.787)	0.543 (0.309 - 0.764)	0.573 (0.352 - 0.780)	0.134 (0.056 - 0.269)	0.124 (0.052 - 0.251)	0.140 (0.059 - 0.281)
5-14 years (rate per capita)	0.410 (0.388 - 0.426)	0.422 (0.396 - 0.444)	0.415 (0.396 - 0.434)	0.752 (0.600 - 0.965)	0.764 (0.611 - 0.980)	0.754 (0.601 - 0.971)	0.558 (0.311 - 0.903)	0.562 (0.312 - 0.909)	0.561 (0.313 - 0.905)
15 - 49 years (rate per capita)	0.401 (0.391 - 0.410)	0.413 (0.404 - 0.422)	0.419 (0.409 - 0.428)	0.817 (0.743 - 0.891)	0.838 (0.764 - 0.915)	0.849 (0.771 - 0.927)	2.026 (1.197 - 3.171)	2.083 (1.226 - 3.271)	2.176 (1.282 - 3.409)
50 - 69 years (rate per capita)	0.520 (0.511 - 0.530)	0.524 (0.514 - 0.533)	0.529 (0.520 - 0.538)	0.863 (0.764 - 0.973)	0.863 (0.765 - 0.970)	0.863 (0.765 - 0.972)	6.029 (3.838 - 8.950)	6.265 (4.000 - 9.308)	6.632 (4.259 - 9.746)
70+ years (rate per capita)	0.553 (0.544 - 0.561)	0.550 (0.542 - 0.557)	0.542 (0.535 - 0.547)	0.748 (0.665 - 0.831)	0.740 (0.661 - 0.820)	0.728 (0.652 - 0.805)	9.878 (6.526 - 13.769)	9.871 (6.521 - 13.751)	9.629 (6.378 - 13.436)
All ages (rate per capita)	0.438 (0.431 - 0.443)	0.460 (0.455 - 0.465)	0.469 (0.464 - 0.474)	0.800 (0.746 - 0.852)	0.811 (0.757 - 0.862)	0.811 (0.757 - 0.862)	3.243 (2.027 - 4.848)	4.119 (2.629 - 6.114)	4.567 (2.934 - 6.708)
Age-standardised (rate per capita)	0.428 (0.422 - 0.435)	0.428 (0.422 - 0.434)	0.431 (0.425 - 0.437)	0.788 (0.732 - 0.850)	0.785 (0.730 - 0.846)	0.788 (0.733 - 0.847)	2.777 (1.730 - 4.170)	2.812 (1.746 - 4.236)	2.807 (1.747 - 4.209)
Total number of cases (thousands)	53,762 (52,954 - 54,465)	57,953 (57,366 - 58,616)	60,239 (59,526 - 60,836)	98,272 (91,624 - 104,646)	102,187 (95,443 - 108,638)	103,993 (97,164 - 110,545)	398 (249 - 595)	519 (331 - 771)	586 (376 - 861)
**Singapore**									
Under-5 years (rate per capita)	0.319 (0.269 - 0.361)	0.236 (0.202 - 0.270)	0.326 (0.281 - 0.372)	0.550 (0.313 - 0.769)	0.278 (0.210 - 0.394)	0.563 (0.321 - 0.788)	0.123 (0.052 - 0.244)	0.091 (0.039 - 0.185)	0.125 (0.055 - 0.250)
5-14 years (rate per capita)	0.438 (0.392 - 0.492)	0.389 (0.341 - 0.434)	0.417 (0.375 - 0.465)	0.755 (0.601 - 0.971)	0.761 (0.595 - 0.999)	0.724 (0.577 - 0.935)	0.560 (0.316 - 0.910)	0.540 (0.308 - 0.866)	0.554 (0.309 - 0.905)
15 - 49 years (rate per capita)	0.402 (0.385 - 0.422)	0.412 (0.395 - 0.431)	0.407 (0.390 - 0.426)	0.809 (0.734 - 0.887)	0.820 (0.747 - 0.897)	0.812 (0.740 - 0.888)	1.929 (1.149 - 3.024)	2.122 (1.256 - 3.334)	2.084 (1.230 - 3.269)
50 - 69 years (rate per capita)	0.510 (0.485 - 0.534)	0.507 (0.480 - 0.533)	0.508 (0.479 - 0.532)	0.846 (0.753 - 0.955)	0.844 (0.748 - 0.956)	0.848 (0.752 - 0.961)	5.815 (3.707 - 8.637)	5.638 (3.575 - 8.388)	5.698 (3.603 - 8.511)
70+ years (rate per capita)	0.550 (0.531 - 0.570)	0.541 (0.522 - 0.561)	0.534 (0.516 - 0.552)	0.740 (0.659 - 0.833)	0.728 (0.650 - 0.815)	0.717 (0.642 - 0.799)	9.900 (6.522 - 13.946)	9.701 (6.373 - 13.598)	9.478 (6.254 - 13.300)
All ages (rate per capita)	0.421 (0.407 - 0.433)	0.425 (0.412 - 0.437)	0.439 (0.425 - 0.451)	0.782 (0.723 - 0.841)	0.781 (0.727 - 0.837)	0.791 (0.738 - 0.843)	2.395 (1.475 - 3.628)	2.896 (1.790 - 4.379)	3.283 (2.035 - 4.915)
Age-standardised (rate per capita)	0.426 (0.412 - 0.439)	0.411 (0.399 - 0.423)	0.426 (0.412 - 0.439)	0.770 (0.714 - 0.828)	0.748 (0.695 - 0.806)	0.770 (0.713 - 0.828)	2.757 (1.719 - 4.130)	2.759 (1.713 - 4.134)	2.768 (1.712 - 4.158)
Total number of cases (thousands)	1,152 (1,114 - 1,188)	1,511 (1,467 - 1,555)	1,722 (1,668 - 1,771)	2,142 (1,982 - 2,305)	2,778 (2,588 - 2,976)	3,105 (2,896 - 3,307)	7 (4 - 10)	10 (6 - 16)	13 (8 - 19)
**South Korea**									
Under-5 years (rate per capita)	0.332 (0.283 - 0.376)	0.334 (0.283 - 0.376)	0.331 (0.285 - 0.377)	0.565 (0.327 - 0.783)	0.571 (0.330 - 0.791)	0.563 (0.325 - 0.781)	0.127 (0.053 - 0.261)	0.129 (0.054 - 0.259)	0.127 (0.054 - 0.260)
5-14 years (rate per capita)	0.429 (0.382 - 0.482)	0.408 (0.361 - 0.458)	0.425 (0.380 - 0.477)	0.744 (0.586 - 0.961)	0.691 (0.563 - 0.875)	0.737 (0.581 - 0.953)	0.557 (0.313 - 0.909)	0.532 (0.300 - 0.878)	0.557 (0.310 - 0.902)
15 - 49 years (rate per capita)	0.388 (0.371 - 0.404)	0.404 (0.387 - 0.424)	0.407 (0.391 - 0.427)	0.782 (0.707 - 0.857)	0.810 (0.737 - 0.888)	0.812 (0.739 - 0.889)	1.825 (1.073 - 2.870)	2.031 (1.200 - 3.179)	2.087 (1.231 - 3.263)
50 - 69 years (rate per capita)	0.507 (0.482 - 0.531)	0.508 (0.484 - 0.533)	0.508 (0.485 - 0.533)	0.847 (0.750 - 0.961)	0.845 (0.744 - 0.954)	0.846 (0.750 - 0.963)	5.628 (3.578 - 8.380)	5.774 (3.649 - 8.566)	5.760 (3.642 - 8.548)
70+ years (rate per capita)	0.552 (0.533 - 0.573)	0.550 (0.530 - 0.574)	0.545 (0.527 - 0.564)	0.750 (0.663 - 0.850)	0.743 (0.657 - 0.839)	0.733 (0.653 - 0.825)	9.808 (6.459 - 13.753)	9.792 (6.483 - 13.710)	9.655 (6.394 - 13.629)
All ages (rate per capita)	0.411 (0.397 - 0.425)	0.429 (0.415 - 0.444)	0.443 (0.430 - 0.456)	0.766 (0.707 - 0.827)	0.785 (0.735 - 0.838)	0.795 (0.745 - 0.845)	2.197 (1.346 - 3.333)	2.911 (1.801 - 4.386)	3.457 (2.156 - 5.168)
Age-standardised (rate per capita)	0.426 (0.413 - 0.439)	0.422 (0.407 - 0.437)	0.426 (0.412 - 0.439)	0.771 (0.715 - 0.831)	0.761 (0.709 - 0.819)	0.770 (0.714 - 0.830)	2.722 (1.693 - 4.074)	2.729 (1.697 - 4.074)	2.741 (1.707 - 4.113)
Total number of cases (thousands)	17,719 (17,113 - 18,306)	20,335 (19,655 - 21,022)	22,291 (21,611 - 22,917)	32,984 (30,445 - 35,641)	37,179 (34,814 - 39,664)	39,983 (37,447 - 42,495)	95 (58 - 144)	138 (85 - 208)	174 (108 - 260)
**Western Europe**									
Under-5 years (rate per capita)	0.136 (0.121 - 0.154)	0.142 (0.127 - 0.156)	0.172 (0.146 - 0.195)	0.237 (0.120 - 0.443)	0.261 (0.126 - 0.461)	0.358 (0.146 - 0.690)	0.052 (0.020 - 0.104)	0.053 (0.021 - 0.108)	0.064 (0.023 - 0.135)
5-14 years (rate per capita)	0.493 (0.477 - 0.509)	0.465 (0.448 - 0.482)	0.481 (0.457 - 0.504)	0.632 (0.528 - 0.755)	0.614 (0.493 - 0.751)	0.623 (0.485 - 0.787)	0.696 (0.373 - 1.155)	0.665 (0.363 - 1.103)	0.679 (0.368 - 1.136)
15 - 49 years (rate per capita)	0.528 (0.520 - 0.537)	0.535 (0.526 - 0.544)	0.534 (0.526 - 0.544)	0.838 (0.778 - 0.895)	0.852 (0.794 - 0.908)	0.854 (0.795 - 0.911)	2.151 (1.235 - 3.472)	2.259 (1.301 - 3.645)	2.269 (1.308 - 3.673)
50 - 69 years (rate per capita)	0.609 (0.598 - 0.620)	0.610 (0.599 - 0.620)	0.607 (0.596 - 0.617)	0.987 (0.901 - 1.065)	0.990 (0.904 - 1.069)	0.992 (0.906 - 1.073)	6.232 (3.890 - 9.298)	6.191 (3.854 - 9.244)	6.172 (3.842 - 9.212)
70+ years (rate per capita)	0.632 (0.624 - 0.642)	0.631 (0.623 - 0.640)	0.624 (0.616 - 0.632)	0.938 (0.861 - 1.009)	0.938 (0.870 - 1.007)	0.930 (0.862 - 0.999)	10.130 (6.709 - 14.210)	10.028 (6.635 - 14.084)	9.655 (6.394 - 13.629)
All ages (rate per capita)	0.528 (0.522 - 0.535)	0.535 (0.529 - 0.542)	0.541 (0.535 - 0.548)	0.819 (0.780 - 0.858)	0.837 (0.798 - 0.876)	0.850 (0.807 - 0.891)	3.503 (2.162 - 5.288)	3.813 (2.369 - 5.735)	4.015 (2.506 - 6.006)
Age-standardised (rate per capita)	0.507 (0.500 - 0.513)	0.503 (0.496 - 0.510)	0.506 (0.500 - 0.515)	0.779 (0.740 - 0.823)	0.781 (0.740 - 0.828)	0.790 (0.741 - 0.844)	2.918 (1.781 - 4.456)	2.910 (1.778 - 4.464)	2.898 (1.773 - 4.424)
Total number of cases (thousands)	201,454 (199,168 - 204,114)	218,071 (215,507 - 220,691)	234,402 (231,880 - 237,437)	312,129 (297,406 - 327,265)	340,781 (325,055 - 356,821)	368,432 (349,762 - 386,418)	1,336 (824 - 2,016)	1,553 (965 - 2,336)	1,741 (1,087 - 2,604)
**Andorra**									
Under-5 years (rate per capita)	0.229 (0.162 - 0.293)	0.226 (0.161 - 0.290)	0.235 (0.171 - 0.299)	0.426 (0.185 - 0.694)	0.423 (0.183 - 0.690)	0.439 (0.190 - 0.715)	0.053 (0.020 - 0.114)	0.053 (0.020 - 0.114)	0.055 (0.021 - 0.118)
5-14 years (rate per capita)	0.445 (0.378 - 0.521)	0.445 (0.380 - 0.514)	0.447 (0.380 - 0.519)	0.744 (0.531 - 0.988)	0.744 (0.531 - 0.988)	0.747 (0.533 - 0.991)	0.509 (0.287 - 0.833)	0.509 (0.290 - 0.840)	0.509 (0.290 - 0.831)
15 - 49 years (rate per capita)	0.521 (0.477 - 0.566)	0.527 (0.485 - 0.576)	0.530 (0.486 - 0.570)	0.816 (0.707 - 0.930)	0.823 (0.717 - 0.936)	0.826 (0.715 - 0.939)	2.115 (1.224 - 3.446)	2.251 (1.307 - 3.672)	2.311 (1.320 - 3.814)
50 - 69 years (rate per capita)	0.643 (0.598 - 0.693)	0.638 (0.593 - 0.689)	0.641 (0.600 - 0.694)	0.942 (0.771 - 1.114)	0.941 (0.765 - 1.114)	0.941 (0.770 - 1.115)	7.483 (4.616 - 11.183)	7.385 (4.555 - 11.098)	7.370 (4.537 - 10.992)
70+ years (rate per capita)	0.674 (0.639 - 0.713)	0.671 (0.635 - 0.709)	0.666 (0.633 - 0.701)	0.908 (0.753 - 1.056)	0.907 (0.754 - 1.055)	0.901 (0.753 - 1.043)	12.115 (7.881 - 17.223)	12.017 (7.811 - 17.179)	11.786 (7.660 - 16.709)
All ages (rate per capita)	0.536 (0.506 - 0.570)	0.545 (0.518 - 0.577)	0.555 (0.527 - 0.584)	0.820 (0.750 - 0.898)	0.830 (0.761 - 0.907)	0.839 (0.768 - 0.912)	3.924 (2.441 - 5.798)	4.308 (2.671 - 6.346)	4.667 (2.893 - 6.806)
Age-standardised (rate per capita)	0.512 (0.482 - 0.547)	0.510 (0.481 - 0.541)	0.511 (0.481 - 0.542)	0.796 (0.726 - 0.877)	0.796 (0.726 - 0.877)	0.796 (0.726 - 0.877)	3.208 (1.994 - 4.819)	3.202 (1.977 - 4.791)	3.201 (1.983 - 4.783)
Total number of cases (thousands)	27 (25 - 29)	39 (37 - 42)	44 (42 - 46)	41 (38 - 45)	60 (55 - 65)	67 (61 - 72)	0 (0 - 0)	0 (0 - 0)	0 (0 - 1)

	Prevalence 1990	Prevalence 2005	Prevalence 2015	Incidence 1990	Incidence 2005	Incidence 2015	YLD rate 1990	YLD rate 2005	YLD rate 2015
**Austria**									
Under-5 years (rate per capita)	0.229 (0.162 - 0.293)	0.231 (0.163 - 0.295)	0.229 (0.160 - 0.298)	0.434 (0.187 - 0.711)	0.437 (0.188 - 0.715)	0.433 (0.187 - 0.710)	0.086 (0.031 - 0.185)	0.087 (0.031 - 0.190)	0.086 (0.031 - 0.184)
5-14 years (rate per capita)	0.445 (0.377 - 0.516)	0.439 (0.373 - 0.514)	0.441 (0.372 - 0.511)	0.769 (0.549 - 1.035)	0.758 (0.539 - 1.013)	0.766 (0.544 - 1.025)	0.639 (0.347 - 1.079)	0.642 (0.351 - 1.088)	0.640 (0.347 - 1.085)
15 - 49 years (rate per capita)	0.516 (0.475 - 0.560)	0.523 (0.479 - 0.568)	0.525 (0.485 - 0.568)	0.861 (0.738 - 0.980)	0.871 (0.747 - 0.986)	0.872 (0.751 - 0.987)	2.416 (1.415 - 3.990)	2.580 (1.502 - 4.265)	2.609 (1.528 - 4.378)
50 - 69 years (rate per capita)	0.642 (0.600 - 0.691)	0.639 (0.597 - 0.687)	0.638 (0.597 - 0.686)	0.991 (0.819 - 1.162)	0.990 (0.818 - 1.164)	0.987 (0.812 - 1.162)	8.094 (5.134 - 12.087)	7.937 (5.020 - 12.030)	7.765 (4.936 - 11.797)
70+ years (rate per capita)	0.682 (0.652 - 0.713)	0.679 (0.649 - 0.710)	0.678 (0.648 - 0.709)	0.947 (0.786 - 1.098)	0.947 (0.790 - 1.095)	0.947 (0.797 - 1.095)	13.165 (8.822 - 18.348)	13.018 (8.645 - 18.141)	12.916 (8.576 - 18.104)
All ages (rate per capita)	0.534 (0.507 - 0.564)	0.544 (0.516 - 0.572)	0.553 (0.526 - 0.584)	0.862 (0.789 - 0.943)	0.873 (0.796 - 0.951)	0.881 (0.804 - 0.958)	4.337 (2.718 - 6.498)	4.646 (2.922 - 6.971)	5.035 (3.187 - 7.512)
Age-standardised (rate per capita)	0.508 (0.479 - 0.539)	0.508 (0.479 - 0.538)	0.508 (0.481 - 0.540)	0.833 (0.759 - 0.915)	0.833 (0.759 - 0.915)	0.833 (0.759 - 0.915)	3.535 (2.198 - 5.354)	3.531 (2.199 - 5.377)	3.530 (2.198 - 5.355)
Total number of cases (thousands)	4,097 (3,883 - 4,326)	4,475 (4,242 - 4,707)	4,795 (4,559 - 5,067)	6,610 (6,045 - 7,232)	7,182 (6,548 - 7,824)	7,640 (6,968 - 8,307)	33 (21 - 50)	38 (24 - 57)	44 (28 - 65)
**Belgium**									
Under-5 years (rate per capita)	0.091 (0.066 - 0.120)	0.072 (0.056 - 0.088)	0.140 (0.094 - 0.194)	0.136 (0.075 - 0.271)	0.109 (0.064 - 0.184)	0.283 (0.100 - 0.671)	0.034 (0.013 - 0.071)	0.028 (0.011 - 0.056)	0.052 (0.018 - 0.118)
5-14 years (rate per capita)	0.286 (0.245 - 0.327)	0.285 (0.244 - 0.330)	0.331 (0.274 - 0.380)	0.296 (0.225 - 0.395)	0.295 (0.216 - 0.404)	0.348 (0.249 - 0.495)	0.496 (0.277 - 0.799)	0.497 (0.282 - 0.789)	0.516 (0.290 - 0.833)
15 - 49 years (rate per capita)	0.386 (0.367 - 0.408)	0.393 (0.372 - 0.414)	0.391 (0.374 - 0.412)	0.490 (0.440 - 0.542)	0.495 (0.446 - 0.546)	0.494 (0.445 - 0.545)	2.329 (1.348 - 3.728)	2.476 (1.446 - 3.983)	2.454 (1.428 - 3.919)
50 - 69 years (rate per capita)	0.544 (0.521 - 0.570)	0.539 (0.514 - 0.564)	0.539 (0.512 - 0.565)	0.583 (0.514 - 0.660)	0.582 (0.513 - 0.662)	0.582 (0.511 - 0.666)	7.732 (4.906 - 11.388)	7.453 (4.694 - 11.031)	7.378 (4.698 - 10.923)
70+ years (rate per capita)	0.586 (0.568 - 0.607)	0.586 (0.569 - 0.605)	0.579 (0.561 - 0.597)	0.520 (0.459 - 0.582)	0.522 (0.459 - 0.589)	0.512 (0.457 - 0.575)	12.442 (8.329 - 17.282)	12.400 (8.293 - 17.285)	12.146 (8.129 - 16.918)
All ages (rate per capita)	0.411 (0.397 - 0.425)	0.420 (0.406 - 0.433)	0.432 (0.416 - 0.447)	0.469 (0.436 - 0.503)	0.473 (0.442 - 0.507)	0.491 (0.454 - 0.533)	4.158 (2.609 - 6.171)	4.476 (2.817 - 6.581)	4.620 (2.910 - 6.809)
Age-standardised (rate per capita)	0.379 (0.365 - 0.393)	0.377 (0.363 - 0.390)	0.391 (0.373 - 0.408)	0.440 (0.407 - 0.475)	0.437 (0.406 - 0.471)	0.462 (0.419 - 0.519)	3.350 (2.092 - 4.997)	3.349 (2.086 - 4.988)	3.350 (2.088 - 4.988)
Total number of cases (thousands)	4,089 (3,949 - 4,227)	4,398 (4,252 - 4,534)	4,897 (4,714 - 5,060)	4,667 (4,334 - 5,003)	4,964 (4,638 - 5,320)	5,560 (5,147 - 6,036)	41 (26 - 61)	47 (30 - 69)	52 (33 - 77)
**Cyprus**									
Under-5 years (rate per capita)	0.233 (0.163 - 0.306)	0.233 (0.163 - 0.302)	0.235 (0.163 - 0.300)	0.433 (0.187 - 0.705)	0.435 (0.188 - 0.708)	0.437 (0.189 - 0.712)	0.054 (0.020 - 0.118)	0.054 (0.021 - 0.119)	0.054 (0.021 - 0.116)
5-14 years (rate per capita)	0.445 (0.378 - 0.516)	0.440 (0.374 - 0.513)	0.445 (0.382 - 0.517)	0.743 (0.531 - 0.988)	0.735 (0.527 - 0.972)	0.745 (0.531 - 0.989)	0.509 (0.281 - 0.817)	0.510 (0.286 - 0.839)	0.509 (0.285 - 0.834)
15 - 49 years (rate per capita)	0.515 (0.470 - 0.560)	0.519 (0.478 - 0.562)	0.522 (0.476 - 0.566)	0.811 (0.697 - 0.925)	0.816 (0.706 - 0.930)	0.815 (0.705 - 0.931)	2.053 (1.188 - 3.364)	2.133 (1.227 - 3.490)	2.121 (1.231 - 3.473)
50 - 69 years (rate per capita)	0.636 (0.593 - 0.687)	0.636 (0.591 - 0.685)	0.637 (0.589 - 0.692)	0.938 (0.764 - 1.110)	0.939 (0.763 - 1.110)	0.939 (0.764 - 1.111)	7.038 (4.307 - 10.664)	7.078 (4.293 - 10.663)	7.131 (4.343 - 10.775)
70+ years (rate per capita)	0.669 (0.633 - 0.705)	0.668 (0.629 - 0.707)	0.667 (0.632 - 0.707)	0.909 (0.757 - 1.055)	0.906 (0.755 - 1.052)	0.906 (0.757 - 1.050)	11.738 (7.555 - 16.879)	11.716 (7.539 - 16.745)	11.661 (7.499 - 16.733)
All ages (rate per capita)	0.509 (0.478 - 0.541)	0.527 (0.499 - 0.557)	0.542 (0.511 - 0.576)	0.794 (0.724 - 0.874)	0.814 (0.744 - 0.892)	0.829 (0.756 - 0.908)	3.112 (1.908 - 4.702)	3.618 (2.236 - 5.421)	3.910 (2.414 - 5.847)
Age-standardised (rate per capita)	0.511 (0.480 - 0.542)	0.510 (0.482 - 0.540)	0.511 (0.481 - 0.543)	0.796 (0.726 - 0.876)	0.795 (0.726 - 0.876)	0.795 (0.726 - 0.876)	3.158 (1.936 - 4.765)	3.153 (1.944 - 4.731)	3.157 (1.943 - 4.765)
Total number of cases (thousands)	340 (319 - 361)	397 (376 - 419)	483 (456 - 514)	531 (483 - 584)	613 (560 - 672)	739 (675 - 810)	2 (1 - 3)	3 (2 - 4)	3 (2 - 5)
**Denmark**									
Under-5 years (rate per capita)	0.227 (0.158 - 0.294)	0.230 (0.163 - 0.296)	0.231 (0.159 - 0.299)	0.428 (0.184 - 0.702)	0.436 (0.188 - 0.714)	0.436 (0.188 - 0.715)	0.085 (0.032 - 0.182)	0.086 (0.031 - 0.190)	0.087 (0.032 - 0.190)
5-14 years (rate per capita)	0.435 (0.370 - 0.505)	0.441 (0.380 - 0.513)	0.441 (0.371 - 0.512)	0.755 (0.538 - 1.010)	0.764 (0.541 - 1.022)	0.765 (0.542 - 1.023)	0.643 (0.346 - 1.069)	0.641 (0.350 - 1.091)	0.641 (0.352 - 1.092)
15 - 49 years (rate per capita)	0.539 (0.500 - 0.576)	0.544 (0.506 - 0.584)	0.541 (0.503 - 0.580)	0.867 (0.746 - 0.982)	0.872 (0.750 - 0.988)	0.868 (0.745 - 0.982)	2.505 (1.389 - 4.160)	2.586 (1.439 - 4.313)	2.571 (1.426 - 4.316)
50 - 69 years (rate per capita)	0.662 (0.627 - 0.702)	0.658 (0.621 - 0.698)	0.664 (0.629 - 0.703)	0.984 (0.808 - 1.154)	0.982 (0.803 - 1.159)	0.986 (0.814 - 1.155)	6.824 (4.112 - 10.465)	6.610 (4.018 - 10.212)	6.977 (4.241 - 10.741)
70+ years (rate per capita)	0.661 (0.631 - 0.691)	0.652 (0.623 - 0.682)	0.658 (0.629 - 0.688)	0.945 (0.787 - 1.097)	0.939 (0.786 - 1.086)	0.944 (0.789 - 1.093)	10.323 (6.723 - 14.791)	10.049 (6.529 - 14.324)	10.241 (6.726 - 14.641)
All ages (rate per capita)	0.548 (0.523 - 0.576)	0.551 (0.527 - 0.578)	0.559 (0.533 - 0.586)	0.862 (0.788 - 0.941)	0.866 (0.792 - 0.942)	0.873 (0.795 - 0.952)	3.873 (2.325 - 5.968)	3.957 (2.383 - 6.074)	4.252 (2.587 - 6.485)
Age-standardised (rate per capita)	0.521 (0.493 - 0.549)	0.519 (0.492 - 0.548)	0.521 (0.490 - 0.550)	0.833 (0.759 - 0.916)	0.833 (0.759 - 0.915)	0.833 (0.759 - 0.915)	3.166 (1.870 - 4.896)	3.139 (1.873 - 4.848)	3.170 (1.878 - 4.907)
Total number of cases (thousands)	2,813 (2,684 - 2,958)	2,987 (2,857 - 3,131)	3,190 (3,043 - 3,349)	4,426 (4,047 - 4,831)	4,693 (4,292 - 5,105)	4,986 (4,542 - 5,437)	20 (12 - 31)	21 (13 - 33)	24 (15 - 37)
**Finland**									
Under-5 years (rate per capita)	0.114 (0.095 - 0.133)	0.172 (0.122 - 0.224)	0.173 (0.123 - 0.223)	0.175 (0.110 - 0.282)	0.365 (0.145 - 0.710)	0.365 (0.146 - 0.712)	0.043 (0.018 - 0.087)	0.064 (0.023 - 0.138)	0.064 (0.023 - 0.137)
5-14 years (rate per capita)	0.445 (0.396 - 0.498)	0.488 (0.431 - 0.551)	0.493 (0.432 - 0.546)	0.654 (0.509 - 0.825)	0.697 (0.547 - 0.878)	0.698 (0.547 - 0.880)	0.667 (0.358 - 1.136)	0.690 (0.374 - 1.184)	0.687 (0.372 - 1.156)
15 - 49 years (rate per capita)	0.548 (0.528 - 0.571)	0.544 (0.526 - 0.565)	0.546 (0.526 - 0.567)	1.011 (0.948 - 1.071)	1.014 (0.951 - 1.075)	1.007 (0.945 - 1.068)	2.301 (1.350 - 3.657)	2.310 (1.358 - 3.667)	2.281 (1.344 - 3.613)
50 - 69 years (rate per capita)	0.616 (0.596 - 0.640)	0.610 (0.589 - 0.630)	0.616 (0.596 - 0.639)	1.190 (1.108 - 1.261)	1.187 (1.100 - 1.259)	1.192 (1.108 - 1.262)	7.135 (4.612 - 10.461)	6.737 (4.323 - 9.862)	7.126 (4.613 - 10.474)
70+ years (rate per capita)	0.671 (0.654 - 0.688)	0.662 (0.645 - 0.680)	0.657 (0.641 - 0.674)	1.154 (1.085 - 1.215)	1.150 (1.085 - 1.210)	1.142 (1.076 - 1.200)	12.490 (8.267 - 17.254)	12.002 (8.012 - 16.584)	11.849 (7.943 - 16.276)
All ages (rate per capita)	0.532 (0.519 - 0.546)	0.547 (0.534 - 0.561)	0.555 (0.541 - 0.569)	0.961 (0.915 - 1.002)	1.000 (0.955 - 1.048)	1.011 (0.964 - 1.057)	3.831 (2.441 - 5.634)	4.218 (2.693 - 6.159)	4.607 (2.974 - 6.702)
Age-standardised (rate per capita)	0.507 (0.494 - 0.523)	0.520 (0.503 - 0.536)	0.521 (0.503 - 0.538)	0.909 (0.861 - 0.954)	0.934 (0.882 - 0.992)	0.933 (0.878 - 0.995)	3.244 (2.056 - 4.820)	3.206 (2.025 - 4.761)	3.220 (2.039 - 4.788)
Total number of cases (thousands)	2,655 (2,590 - 2,725)	2,872 (2,800 - 2,943)	3,081 (3,005 - 3,159)	4,793 (4,564 - 5,002)	5,249 (5,013 - 5,500)	5,613 (5,353 - 5,866)	19 (12 - 28)	22 (14 - 32)	26 (17 - 37)
**France**									
Under-5 years (rate per capita)	0.123 (0.084 - 0.188)	0.097 (0.074 - 0.123)	0.153 (0.103 - 0.208)	0.216 (0.073 - 0.635)	0.138 (0.085 - 0.223)	0.334 (0.111 - 0.701)	0.047 (0.015 - 0.112)	0.037 (0.014 - 0.074)	0.058 (0.020 - 0.131)
5-14 years (rate per capita)	0.581 (0.552 - 0.608)	0.451 (0.408 - 0.490)	0.554 (0.495 - 0.613)	0.718 (0.574 - 0.861)	0.602 (0.495 - 0.736)	0.738 (0.577 - 0.928)	0.803 (0.424 - 1.376)	0.709 (0.377 - 1.220)	0.779 (0.413 - 1.340)
15 - 49 years (rate per capita)	0.605 (0.588 - 0.624)	0.604 (0.588 - 0.623)	0.604 (0.586 - 0.624)	0.954 (0.898 - 1.010)	0.960 (0.903 - 1.016)	0.962 (0.906 - 1.017)	2.149 (1.245 - 3.477)	2.236 (1.298 - 3.631)	2.248 (1.291 - 3.634)
50 - 69 years (rate per capita)	0.650 (0.630 - 0.670)	0.648 (0.627 - 0.670)	0.650 (0.630 - 0.671)	1.103 (1.030 - 1.166)	1.101 (1.029 - 1.165)	1.102 (1.028 - 1.168)	6.285 (3.984 - 9.299)	6.022 (3.841 - 8.913)	6.194 (3.883 - 9.214)
70+ years (rate per capita)	0.671 (0.658 - 0.687)	0.675 (0.659 - 0.692)	0.664 (0.650 - 0.681)	1.069 (1.014 - 1.123)	1.075 (1.021 - 1.128)	1.065 (1.014 - 1.117)	10.283 (6.782 - 14.498)	10.405 (6.923 - 14.635)	10.057 (6.701 - 14.088)
All ages (rate per capita)	0.585 (0.573 - 0.598)	0.572 (0.560 - 0.586)	0.590 (0.577 - 0.604)	0.914 (0.871 - 0.962)	0.911 (0.870 - 0.948)	0.945 (0.898 - 0.991)	3.422 (2.134 - 5.147)	3.751 (2.363 - 5.618)	3.949 (2.471 - 5.878)
Age-standardised (rate per capita)	0.569 (0.556 - 0.583)	0.544 (0.530 - 0.559)	0.566 (0.551 - 0.584)	0.880 (0.835 - 0.939)	0.854 (0.811 - 0.893)	0.894 (0.838 - 0.952)	2.954 (1.813 - 4.484)	2.956 (1.824 - 4.481)	2.963 (1.826 - 4.508)
Total number of cases (thousands)	33,172 (32,493 - 33,915)	35,031 (34,294 - 35,874)	38,512 (37,667 - 39,400)	51,811 (49,359 - 54,552)	55,732 (53,219 - 58,004)	61,655 (58,547 - 64,614)	194 (121 - 292)	230 (145 - 344)	258 (161 - 383)
**Germany**									
Under-5 years (rate per capita)	0.108 (0.089 - 0.128)	0.106 (0.076 - 0.141)	0.150 (0.106 - 0.202)	0.159 (0.107 - 0.213)	0.174 (0.076 - 0.393)	0.305 (0.121 - 0.665)	0.041 (0.017 - 0.084)	0.040 (0.015 - 0.085)	0.056 (0.020 - 0.117)
5-14 years (rate per capita)	0.434 (0.409 - 0.461)	0.449 (0.408 - 0.486)	0.404 (0.353 - 0.455)	0.543 (0.394 - 0.713)	0.638 (0.468 - 0.873)	0.503 (0.376 - 0.659)	0.606 (0.331 - 1.012)	0.628 (0.340 - 1.025)	0.604 (0.338 - 1.006)
15 - 49 years (rate per capita)	0.519 (0.500 - 0.542)	0.526 (0.507 - 0.548)	0.518 (0.500 - 0.542)	0.856 (0.785 - 0.930)	0.869 (0.795 - 0.940)	0.871 (0.797 - 0.951)	2.442 (1.373 - 4.053)	2.603 (1.467 - 4.331)	2.546 (1.445 - 4.239)
50 - 69 years (rate per capita)	0.624 (0.600 - 0.651)	0.635 (0.616 - 0.657)	0.616 (0.589 - 0.642)	1.019 (0.912 - 1.122)	1.024 (0.918 - 1.131)	1.019 (0.908 - 1.127)	6.677 (4.092 - 10.212)	6.995 (4.278 - 10.672)	6.494 (3.980 - 9.940)
70+ years (rate per capita)	0.630 (0.612 - 0.653)	0.639 (0.622 - 0.657)	0.629 (0.611 - 0.651)	0.945 (0.846 - 1.050)	0.948 (0.852 - 1.051)	0.950 (0.852 - 1.051)	10.587 (6.970 - 14.899)	10.650 (7.020 - 15.161)	10.471 (6.894 - 14.826)
All ages (rate per capita)	0.524 (0.511 - 0.538)	0.541 (0.530 - 0.554)	0.538 (0.524 - 0.553)	0.834 (0.782 - 0.883)	0.864 (0.811 - 0.919)	0.869 (0.815 - 0.921)	3.947 (2.416 - 6.045)	4.392 (2.683 - 6.629)	4.677 (2.907 - 7.026)
Age-standardised (rate per capita)	0.488 (0.476 - 0.501)	0.493 (0.480 - 0.507)	0.483 (0.467 - 0.500)	0.768 (0.714 - 0.820)	0.786 (0.733 - 0.850)	0.774 (0.720 - 0.834)	3.154 (1.903 - 4.848)	3.172 (1.917 - 4.837)	3.087 (1.874 - 4.706)
Total number of cases (thousands)	41,510 (40,485 - 42,617)	44,575 (43,600 - 45,632)	45,001 (43,815 - 46,271)	66,002 (61,874 - 69,892)	71,177 (66,794 - 75,663)	72,672 (68,144 - 77,050)	313 (191 - 479)	362 (221 - 546)	391 (243 - 588)
**Greece**									
Under-5 years (rate per capita)	0.118 (0.096 - 0.149)	0.187 (0.145 - 0.231)	0.176 (0.127 - 0.228)	0.174 (0.104 - 0.287)	0.357 (0.184 - 0.631)	0.372 (0.153 - 0.735)	0.046 (0.019 - 0.094)	0.070 (0.028 - 0.148)	0.066 (0.024 - 0.144)
5-14 years (rate per capita)	0.585 (0.557 - 0.608)	0.533 (0.502 - 0.560)	0.546 (0.487 - 0.601)	0.702 (0.555 - 0.864)	0.510 (0.412 - 0.626)	0.671 (0.530 - 0.829)	0.819 (0.427 - 1.399)	0.701 (0.374 - 1.182)	0.756 (0.403 - 1.279)
15 - 49 years (rate per capita)	0.606 (0.587 - 0.626)	0.603 (0.586 - 0.623)	0.605 (0.589 - 0.624)	0.902 (0.839 - 0.962)	0.913 (0.852 - 0.972)	0.921 (0.859 - 0.978)	2.312 (1.344 - 3.741)	2.376 (1.379 - 3.812)	2.479 (1.447 - 3.972)
50 - 69 years (rate per capita)	0.669 (0.648 - 0.688)	0.665 (0.645 - 0.685)	0.665 (0.644 - 0.685)	1.077 (0.999 - 1.150)	1.080 (1.005 - 1.154)	1.078 (0.999 - 1.150)	7.124 (4.526 - 10.543)	7.230 (4.644 - 10.624)	7.190 (4.584 - 10.579)
70+ years (rate per capita)	0.702 (0.687 - 0.719)	0.704 (0.688 - 0.720)	0.690 (0.675 - 0.706)	1.053 (0.988 - 1.117)	1.059 (0.994 - 1.120)	1.042 (0.980 - 1.098)	12.076 (8.045 - 16.719)	12.238 (8.140 - 17.090)	11.737 (7.798 - 16.317)
All ages (rate per capita)	0.600 (0.588 - 0.613)	0.603 (0.592 - 0.616)	0.608 (0.595 - 0.620)	0.891 (0.845 - 0.932)	0.903 (0.859 - 0.944)	0.930 (0.887 - 0.973)	4.025 (2.519 - 6.017)	4.472 (2.823 - 6.538)	4.812 (3.066 - 7.101)
Age-standardised (rate per capita)	0.575 (0.562 - 0.588)	0.566 (0.553 - 0.580)	0.567 (0.551 - 0.583)	0.841 (0.794 - 0.888)	0.827 (0.780 - 0.874)	0.855 (0.803 - 0.914)	3.321 (2.054 - 5.006)	3.274 (2.035 - 4.921)	3.280 (2.046 - 4.937)
Total number of cases (thousands)	6,081 (5,954 - 6,215)	6,676 (6,549 - 6,825)	6,640 (6,498 - 6,769)	9,022 (8,558 - 9,442)	9,998 (9,513 - 10,447)	10,153 (9,686 - 10,627)	41 (26 - 61)	50 (31 - 72)	53 (33 - 78)
**Iceland**									

	Prevalence 1990	Prevalence 2005	Prevalence 2015	Incidence 1990	Incidence 2005	Incidence 2015	YLD rate 1990	YLD rate 2005	YLD rate 2015
Under-5 years (rate per capita)	0.230 (0.164 - 0.290)	0.227 (0.161 - 0.294)	0.232 (0.166 - 0.306)	0.433 (0.187 - 0.710)	0.432 (0.186 - 0.708)	0.437 (0.188 - 0.716)	0.086 (0.032 - 0.188)	0.086 (0.032 - 0.187)	0.087 (0.032 - 0.187)
5-14 years (rate per capita)	0.551 (0.497 - 0.611)	0.563 (0.506 - 0.620)	0.552 (0.494 - 0.614)	0.785 (0.593 - 1.015)	0.791 (0.609 - 1.013)	0.784 (0.594 - 1.007)	0.779 (0.414 - 1.301)	0.803 (0.418 - 1.355)	0.780 (0.410 - 1.321)
15 - 49 years (rate per capita)	0.620 (0.603 - 0.642)	0.625 (0.609 - 0.647)	0.622 (0.605 - 0.645)	0.841 (0.782 - 0.902)	0.851 (0.793 - 0.909)	0.847 (0.788 - 0.910)	2.362 (1.346 - 3.940)	2.489 (1.420 - 4.173)	2.443 (1.392 - 4.084)
50 - 69 years (rate per capita)	0.708 (0.681 - 0.736)	0.703 (0.675 - 0.735)	0.705 (0.678 - 0.735)	1.021 (0.941 - 1.096)	1.014 (0.934 - 1.087)	1.017 (0.932 - 1.092)	7.678 (4.681 - 11.636)	7.235 (4.412 - 11.094)	7.447 (4.517 - 11.386)
70+ years (rate per capita)	0.727 (0.704 - 0.749)	0.725 (0.701 - 0.749)	0.717 (0.693 - 0.740)	0.997 (0.924 - 1.060)	0.994 (0.921 - 1.056)	0.987 (0.919 - 1.046)	12.101 (7.785 - 17.240)	12.047 (7.688 - 17.226)	11.793 (7.550 - 16.816)
All ages (rate per capita)	0.597 (0.580 - 0.615)	0.611 (0.594 - 0.630)	0.614 (0.598 - 0.634)	0.837 (0.784 - 0.898)	0.855 (0.806 - 0.910)	0.863 (0.815 - 0.917)	3.442 (2.075 - 5.290)	3.781 (2.282 - 5.819)	4.066 (2.460 - 6.230)
Age-standardised (rate per capita)	0.595 (0.578 - 0.612)	0.598 (0.581 - 0.617)	0.595 (0.578 - 0.615)	0.838 (0.784 - 0.902)	0.840 (0.786 - 0.900)	0.837 (0.784 - 0.901)	3.447 (2.074 - 5.337)	3.445 (2.066 - 5.300)	3.444 (2.077 - 5.315)
Total number of cases (thousands)	152 (148 - 157)	181 (176 - 187)	200 (195 - 207)	213 (200 - 229)	254 (239 - 270)	282 (266 - 299)	1 (1 - 1)	1 (1 - 2)	1 (1 - 2)
**Ireland**									
Under-5 years (rate per capita)	0.138 (0.100 - 0.183)	0.168 (0.124 - 0.220)	0.171 (0.126 - 0.222)	0.241 (0.116 - 0.500)	0.350 (0.150 - 0.697)	0.359 (0.154 - 0.713)	0.052 (0.020 - 0.107)	0.063 (0.023 - 0.137)	0.064 (0.024 - 0.138)
5-14 years (rate per capita)	0.433 (0.380 - 0.485)	0.459 (0.402 - 0.514)	0.462 (0.402 - 0.516)	0.528 (0.407 - 0.684)	0.554 (0.419 - 0.715)	0.555 (0.419 - 0.720)	0.633 (0.347 - 1.061)	0.643 (0.350 - 1.081)	0.643 (0.353 - 1.079)
15 - 49 years (rate per capita)	0.505 (0.484 - 0.529)	0.515 (0.493 - 0.541)	0.528 (0.508 - 0.552)	0.844 (0.770 - 0.916)	0.860 (0.787 - 0.939)	0.875 (0.799 - 0.952)	2.165 (1.287 - 3.393)	2.267 (1.355 - 3.508)	2.396 (1.442 - 3.741)
50 - 69 years (rate per capita)	0.604 (0.582 - 0.630)	0.596 (0.571 - 0.621)	0.599 (0.576 - 0.623)	1.050 (0.944 - 1.151)	1.046 (0.938 - 1.145)	1.048 (0.937 - 1.150)	7.749 (5.067 - 11.307)	7.335 (4.752 - 10.656)	7.497 (4.854 - 11.022)
70+ years (rate per capita)	0.671 (0.651 - 0.691)	0.663 (0.646 - 0.684)	0.661 (0.643 - 0.681)	1.000 (0.898 - 1.102)	0.991 (0.889 - 1.084)	0.987 (0.887 - 1.079)	13.520 (9.006 - 18.777)	13.282 (8.846 - 18.473)	13.143 (8.696 - 18.115)
All ages (rate per capita)	0.491 (0.474 - 0.508)	0.508 (0.493 - 0.525)	0.518 (0.501 - 0.533)	0.781 (0.734 - 0.831)	0.826 (0.773 - 0.882)	0.837 (0.783 - 0.893)	3.469 (2.229 - 5.089)	3.683 (2.367 - 5.379)	3.882 (2.490 - 5.646)
Age-standardised (rate per capita)	0.490 (0.473 - 0.507)	0.496 (0.479 - 0.514)	0.497 (0.479 - 0.513)	0.785 (0.738 - 0.837)	0.800 (0.745 - 0.860)	0.800 (0.745 - 0.860)	3.415 (2.183 - 5.035)	3.408 (2.184 - 4.981)	3.412 (2.182 - 4.972)
Total number of cases (thousands)	1,733 (1,672 - 1,794)	2,120 (2,056 - 2,191)	2,480 (2,401 - 2,554)	2,757 (2,592 - 2,935)	3,448 (3,228 - 3,681)	4,008 (3,752 - 4,278)	12 (8 - 18)	15 (10 - 22)	19 (12 - 27)
**Israel**									
Under-5 years (rate per capita)	0.158 (0.119 - 0.202)	0.184 (0.133 - 0.232)	0.185 (0.137 - 0.235)	0.287 (0.144 - 0.565)	0.390 (0.158 - 0.711)	0.392 (0.159 - 0.715)	0.060 (0.023 - 0.121)	0.069 (0.026 - 0.143)	0.070 (0.027 - 0.143)
5-14 years (rate per capita)	0.520 (0.469 - 0.572)	0.534 (0.478 - 0.590)	0.534 (0.478 - 0.592)	0.612 (0.474 - 0.766)	0.629 (0.484 - 0.803)	0.629 (0.484 - 0.801)	0.728 (0.391 - 1.234)	0.721 (0.389 - 1.220)	0.720 (0.384 - 1.219)
15 - 49 years (rate per capita)	0.567 (0.548 - 0.589)	0.570 (0.553 - 0.593)	0.573 (0.554 - 0.597)	0.849 (0.783 - 0.917)	0.855 (0.791 - 0.921)	0.860 (0.797 - 0.924)	2.250 (1.296 - 3.726)	2.305 (1.331 - 3.838)	2.352 (1.352 - 3.951)
50 - 69 years (rate per capita)	0.672 (0.643 - 0.707)	0.666 (0.633 - 0.700)	0.672 (0.642 - 0.704)	1.057 (0.968 - 1.140)	1.050 (0.953 - 1.139)	1.056 (0.960 - 1.142)	7.433 (4.683 - 11.388)	7.010 (4.340 - 10.843)	7.432 (4.627 - 11.250)
70+ years (rate per capita)	0.704 (0.683 - 0.724)	0.699 (0.678 - 0.720)	0.692 (0.671 - 0.712)	1.022 (0.932 - 1.101)	1.014 (0.934 - 1.089)	1.006 (0.927 - 1.078)	12.279 (8.058 - 17.231)	12.073 (7.962 - 17.128)	11.826 (7.828 - 16.635)
All ages (rate per capita)	0.536 (0.518 - 0.554)	0.550 (0.533 - 0.568)	0.553 (0.535 - 0.572)	0.779 (0.726 - 0.836)	0.812 (0.758 - 0.870)	0.817 (0.764 - 0.877)	3.002 (1.830 - 4.647)	3.276 (2.016 - 5.060)	3.439 (2.124 - 5.277)
Age-standardised (rate per capita)	0.552 (0.535 - 0.569)	0.556 (0.539 - 0.573)	0.556 (0.538 - 0.575)	0.811 (0.762 - 0.863)	0.822 (0.769 - 0.878)	0.822 (0.769 - 0.878)	3.376 (2.076 - 5.200)	3.372 (2.071 - 5.204)	3.370 (2.076 - 5.188)
Total number of cases (thousands)	2,409 (2,331 - 2,490)	3,632 (3,515 - 3,747)	4,453 (4,305 - 4,603)	3,503 (3,264 - 3,759)	5,359 (5,001 - 5,746)	6,579 (6,148 - 7,057)	13 (8 - 21)	22 (13 - 33)	28 (17 - 42)
**Italy**									
Under-5 years (rate per capita)	0.124 (0.090 - 0.168)	0.097 (0.087 - 0.107)	0.150 (0.101 - 0.204)	0.217 (0.103 - 0.497)	0.140 (0.112 - 0.170)	0.306 (0.118 - 0.688)	0.046 (0.017 - 0.096)	0.037 (0.016 - 0.072)	0.056 (0.019 - 0.124)
5-14 years (rate per capita)	0.437 (0.378 - 0.490)	0.388 (0.366 - 0.411)	0.429 (0.366 - 0.480)	0.463 (0.341 - 0.612)	0.316 (0.263 - 0.378)	0.461 (0.337 - 0.624)	0.660 (0.357 - 1.116)	0.584 (0.324 - 0.958)	0.624 (0.346 - 1.043)
15 - 49 years (rate per capita)	0.467 (0.447 - 0.490)	0.480 (0.462 - 0.504)	0.479 (0.460 - 0.503)	0.638 (0.579 - 0.698)	0.658 (0.596 - 0.725)	0.663 (0.598 - 0.728)	1.789 (1.018 - 2.918)	1.920 (1.092 - 3.146)	1.975 (1.124 - 3.216)
50 - 69 years (rate per capita)	0.546 (0.520 - 0.575)	0.542 (0.518 - 0.569)	0.540 (0.514 - 0.567)	0.766 (0.675 - 0.866)	0.767 (0.680 - 0.863)	0.767 (0.678 - 0.876)	4.596 (2.803 - 7.001)	4.588 (2.823 - 7.013)	4.532 (2.752 - 6.909)
70+ years (rate per capita)	0.553 (0.534 - 0.573)	0.549 (0.528 - 0.572)	0.541 (0.523 - 0.563)	0.702 (0.620 - 0.785)	0.701 (0.626 - 0.782)	0.692 (0.617 - 0.770)	7.055 (4.624 - 10.134)	7.016 (4.578 - 10.101)	6.821 (4.468 - 9.833)
All ages (rate per capita)	0.473 (0.457 - 0.489)	0.478 (0.464 - 0.492)	0.486 (0.472 - 0.500)	0.633 (0.591 - 0.681)	0.634 (0.594 - 0.675)	0.660 (0.616 - 0.705)	2.736 (1.658 - 4.169)	3.061 (1.874 - 4.688)	3.191 (1.949 - 4.873)
Age-standardised (rate per capita)	0.450 (0.434 - 0.468)	0.437 (0.424 - 0.449)	0.448 (0.431 - 0.462)	0.597 (0.553 - 0.651)	0.563 (0.527 - 0.599)	0.603 (0.552 - 0.661)	2.257 (1.357 - 3.469)	2.241 (1.345 - 3.475)	2.245 (1.352 - 3.470)
Total number of cases (thousands)	26,910 (26,017 - 27,821)	28,005 (27,188 - 28,800)	30,509 (29,656 - 31,421)	36,008 (33,628 - 38,727)	37,142 (34,796 - 39,549)	41,436 (38,685 - 44,276)	156 (94 - 237)	179 (110 - 275)	200 (122 - 306)
**Luxembourg**									
Under-5 years (rate per capita)	0.227 (0.160 - 0.289)	0.231 (0.162 - 0.293)	0.227 (0.160 - 0.290)	0.430 (0.185 - 0.704)	0.436 (0.188 - 0.713)	0.430 (0.185 - 0.705)	0.085 (0.030 - 0.186)	0.087 (0.032 - 0.187)	0.086 (0.031 - 0.183)
5-14 years (rate per capita)	0.445 (0.380 - 0.515)	0.443 (0.378 - 0.513)	0.439 (0.371 - 0.511)	0.768 (0.547 - 1.031)	0.766 (0.544 - 1.026)	0.763 (0.541 - 1.021)	0.639 (0.344 - 1.076)	0.639 (0.345 - 1.083)	0.641 (0.350 - 1.090)
15 - 49 years (rate per capita)	0.519 (0.481 - 0.563)	0.526 (0.484 - 0.571)	0.525 (0.481 - 0.570)	0.866 (0.742 - 0.983)	0.872 (0.750 - 0.987)	0.871 (0.750 - 0.985)	2.448 (1.428 - 4.068)	2.585 (1.511 - 4.320)	2.568 (1.506 - 4.273)
50 - 69 years (rate per capita)	0.638 (0.595 - 0.689)	0.636 (0.591 - 0.690)	0.635 (0.589 - 0.688)	0.989 (0.815 - 1.165)	0.986 (0.812 - 1.162)	0.986 (0.808 - 1.161)	7.769 (4.941 - 11.698)	7.569 (4.714 - 11.388)	7.483 (4.689 - 11.430)
70+ years (rate per capita)	0.682 (0.653 - 0.713)	0.683 (0.649 - 0.716)	0.674 (0.644 - 0.706)	0.953 (0.790 - 1.107)	0.955 (0.794 - 1.109)	0.942 (0.789 - 1.087)	13.037 (8.700 - 18.244)	12.978 (8.628 - 18.092)	12.708 (8.450 - 17.817)
All ages (rate per capita)	0.534 (0.506 - 0.564)	0.538 (0.510 - 0.569)	0.539 (0.509 - 0.571)	0.864 (0.789 - 0.945)	0.866 (0.791 - 0.942)	0.868 (0.792 - 0.946)	4.216 (2.627 - 6.356)	4.301 (2.699 - 6.513)	4.349 (2.716 - 6.588)
Age-standardised (rate per capita)	0.508 (0.478 - 0.539)	0.508 (0.479 - 0.540)	0.508 (0.478 - 0.542)	0.833 (0.759 - 0.915)	0.833 (0.759 - 0.915)	0.833 (0.759 - 0.915)	3.486 (2.165 - 5.297)	3.482 (2.165 - 5.306)	3.489 (2.168 - 5.312)
Total number of cases (thousands)	203 (192 - 215)	245 (233 - 260)	300 (283 - 318)	328 (300 - 359)	395 (361 - 430)	483 (441 - 527)	2 (1 - 2)	2 (1 - 3)	2 (2 - 4)
**Malta**									
Under-5 years (rate per capita)	0.230 (0.161 - 0.300)	0.234 (0.166 - 0.297)	0.228 (0.165 - 0.294)	0.436 (0.188 - 0.714)	0.442 (0.190 - 0.724)	0.434 (0.187 - 0.711)	0.087 (0.032 - 0.185)	0.088 (0.031 - 0.191)	0.086 (0.032 - 0.185)
5-14 years (rate per capita)	0.442 (0.375 - 0.515)	0.438 (0.374 - 0.511)	0.439 (0.370 - 0.510)	0.762 (0.541 - 1.020)	0.760 (0.541 - 1.017)	0.759 (0.540 - 1.013)	0.641 (0.347 - 1.094)	0.640 (0.350 - 1.083)	0.644 (0.350 - 1.091)
15 - 49 years (rate per capita)	0.516 (0.472 - 0.565)	0.517 (0.473 - 0.562)	0.516 (0.470 - 0.558)	0.862 (0.739 - 0.981)	0.865 (0.741 - 0.979)	0.865 (0.742 - 0.981)	2.296 (1.314 - 3.783)	2.367 (1.361 - 3.921)	2.367 (1.343 - 3.989)
50 - 69 years (rate per capita)	0.634 (0.591 - 0.688)	0.632 (0.589 - 0.683)	0.637 (0.594 - 0.695)	0.985 (0.809 - 1.161)	0.985 (0.807 - 1.162)	0.989 (0.815 - 1.159)	7.310 (4.478 - 11.089)	7.320 (4.486 - 11.134)	7.661 (4.684 - 11.484)
70+ years (rate per capita)	0.674 (0.638 - 0.712)	0.672 (0.635 - 0.710)	0.674 (0.640 - 0.713)	0.956 (0.793 - 1.114)	0.954 (0.794 - 1.111)	0.958 (0.793 - 1.116)	12.136 (7.788 - 17.204)	12.139 (7.816 - 17.330)	12.180 (7.867 - 17.391)
All ages (rate per capita)	0.512 (0.483 - 0.545)	0.534 (0.505 - 0.564)	0.548 (0.519 - 0.582)	0.840 (0.764 - 0.922)	0.867 (0.792 - 0.946)	0.880 (0.805 - 0.960)	3.376 (2.046 - 5.166)	4.101 (2.511 - 6.182)	4.723 (2.915 - 7.069)
Age-standardised (rate per capita)	0.506 (0.477 - 0.539)	0.505 (0.476 - 0.538)	0.505 (0.475 - 0.536)	0.832 (0.758 - 0.914)	0.832 (0.758 - 0.914)	0.833 (0.759 - 0.914)	3.315 (2.016 - 5.071)	3.318 (2.015 - 5.057)	3.316 (2.010 - 5.088)
Total number of cases (thousands)	182 (172 - 194)	212 (200 - 224)	229 (217 - 243)	299 (272 - 328)	344 (314 - 376)	368 (337 - 402)	1 (1 - 2)	2 (1 - 2)	2 (1 - 3)
**Netherlands**									
Under-5 years (rate per capita)	0.158 (0.109 - 0.210)	0.159 (0.120 - 0.205)	0.158 (0.108 - 0.208)	0.319 (0.128 - 0.690)	0.331 (0.145 - 0.678)	0.321 (0.128 - 0.695)	0.058 (0.021 - 0.126)	0.060 (0.021 - 0.127)	0.058 (0.021 - 0.126)
5-14 years (rate per capita)	0.382 (0.329 - 0.434)	0.385 (0.335 - 0.438)	0.384 (0.330 - 0.435)	0.475 (0.355 - 0.626)	0.486 (0.368 - 0.638)	0.476 (0.355 - 0.629)	0.563 (0.317 - 0.936)	0.566 (0.320 - 0.926)	0.564 (0.317 - 0.923)
15 - 49 years (rate per capita)	0.463 (0.440 - 0.488)	0.471 (0.446 - 0.497)	0.464 (0.440 - 0.491)	0.828 (0.756 - 0.908)	0.841 (0.767 - 0.922)	0.836 (0.762 - 0.917)	2.485 (1.474 - 4.009)	2.646 (1.568 - 4.277)	2.631 (1.556 - 4.276)
50 - 69 years (rate per capita)	0.603 (0.568 - 0.639)	0.598 (0.564 - 0.634)	0.601 (0.569 - 0.636)	0.992 (0.884 - 1.102)	0.990 (0.880 - 1.104)	0.993 (0.883 - 1.107)	8.742 (5.648 - 13.009)	8.432 (5.378 - 12.604)	8.720 (5.511 - 12.827)
70+ years (rate per capita)	0.663 (0.643 - 0.684)	0.660 (0.640 - 0.681)	0.658 (0.638 - 0.680)	0.913 (0.817 - 1.012)	0.909 (0.812 - 1.005)	0.905 (0.811 - 1.001)	14.621 (9.799 - 20.204)	14.480 (9.632 - 19.938)	14.367 (9.590 - 19.809)
All ages (rate per capita)	0.478 (0.458 - 0.498)	0.490 (0.472 - 0.510)	0.497 (0.479 - 0.518)	0.793 (0.739 - 0.847)	0.808 (0.755 - 0.860)	0.817 (0.762 - 0.869)	4.334 (2.763 - 6.483)	4.751 (3.033 - 7.050)	5.200 (3.313 - 7.648)
Age-standardised (rate per capita)	0.456 (0.435 - 0.476)	0.456 (0.437 - 0.477)	0.455 (0.435 - 0.474)	0.751 (0.694 - 0.812)	0.754 (0.699 - 0.811)	0.751 (0.694 - 0.812)	3.772 (2.391 - 5.670)	3.770 (2.391 - 5.606)	3.761 (2.361 - 5.603)
Total number of cases (thousands)	7,141 (6,837 - 7,432)	7,982 (7,693 - 8,318)	8,552 (8,240 - 8,902)	11,839 (11,039 - 12,649)	13,173 (12,300 - 14,022)	14,051 (13,104 - 14,934)	65 (41 - 97)	77 (49 - 115)	89 (57 - 131)
**Norway**									
Under-5 years (rate per capita)	0.061 (0.043 - 0.081)	0.102 (0.054 - 0.159)	0.100 (0.054 - 0.158)	0.103 (0.054 - 0.161)	0.187 (0.052 - 0.487)	0.187 (0.052 - 0.486)	0.023 (0.009 - 0.049)	0.036 (0.010 - 0.089)	0.036 (0.010 - 0.090)
5-14 years (rate per capita)	0.323 (0.278 - 0.379)	0.353 (0.300 - 0.406)	0.370 (0.315 - 0.429)	0.480 (0.369 - 0.649)	0.509 (0.370 - 0.685)	0.524 (0.382 - 0.711)	0.559 (0.315 - 0.916)	0.578 (0.322 - 0.955)	0.602 (0.338 - 1.001)
15 - 49 years (rate per capita)	0.523 (0.503 - 0.544)	0.527 (0.508 - 0.548)	0.521 (0.504 - 0.541)	0.864 (0.794 - 0.936)	0.871 (0.798 - 0.942)	0.868 (0.798 - 0.938)	2.334 (1.351 - 3.759)	2.432 (1.414 - 3.911)	2.416 (1.403 - 3.874)
50 - 69 years (rate per capita)	0.626 (0.603 - 0.650)	0.621 (0.597 - 0.647)	0.625 (0.602 - 0.650)	1.050 (0.943 - 1.154)	1.045 (0.939 - 1.153)	1.048 (0.938 - 1.152)	7.547 (4.832 - 11.068)	7.011 (4.458 - 10.431)	7.359 (4.724 - 10.931)
70+ years (rate per capita)	0.660 (0.643 - 0.677)	0.649 (0.633 - 0.668)	0.648 (0.632 - 0.666)	0.990 (0.894 - 1.092)	0.972 (0.881 - 1.067)	0.970 (0.877 - 1.062)	12.174 (8.139 - 16.937)	11.819 (7.894 - 16.356)	11.798 (7.860 - 16.501)
All ages (rate per capita)	0.503 (0.488 - 0.517)	0.511 (0.497 - 0.525)	0.517 (0.502 - 0.532)	0.816 (0.767 - 0.863)	0.829 (0.777 - 0.878)	0.842 (0.791 - 0.889)	4.053 (2.553 - 5.981)	4.060 (2.564 - 6.045)	4.222 (2.675 - 6.276)
Age-standardised (rate per capita)	0.472 (0.457 - 0.487)	0.479 (0.463 - 0.494)	0.481 (0.463 - 0.498)	0.767 (0.719 - 0.817)	0.777 (0.726 - 0.834)	0.779 (0.726 - 0.837)	3.292 (2.049 - 4.949)	3.291 (2.044 - 4.954)	3.309 (2.067 - 4.973)
Total number of cases (thousands)	2,130 (2,068 - 2,191)	2,362 (2,296 - 2,427)	2,668 (2,590 - 2,749)	3,456 (3,248 - 3,655)	3,830 (3,592 - 4,058)	4,346 (4,085 - 4,594)	17 (11 - 25)	19 (12 - 28)	22 (14 - 32)
**Portugal**									
Under-5 years (rate per capita)	0.173 (0.124 - 0.227)	0.171 (0.123 - 0.226)	0.173 (0.125 - 0.231)	0.359 (0.144 - 0.715)	0.356 (0.143 - 0.710)	0.360 (0.145 - 0.717)	0.064 (0.023 - 0.139)	0.064 (0.023 - 0.136)	0.064 (0.024 - 0.140)

	Prevalence 1990	Prevalence 2005	Prevalence 2015	Incidence 1990	Incidence 2005	Incidence 2015	YLD rate 1990	YLD rate 2005	YLD rate 2015
5-14 years (rate per capita)	0.459 (0.402 - 0.516)	0.465 (0.402 - 0.522)	0.469 (0.411 - 0.524)	0.558 (0.435 - 0.712)	0.562 (0.434 - 0.722)	0.565 (0.434 - 0.728)	0.656 (0.355 - 1.108)	0.656 (0.356 - 1.110)	0.654 (0.358 - 1.114)
15 - 49 years (rate per capita)	0.527 (0.505 - 0.555)	0.541 (0.519 - 0.567)	0.547 (0.524 - 0.573)	0.854 (0.786 - 0.925)	0.875 (0.806 - 0.947)	0.885 (0.814 - 0.957)	2.201 (1.266 - 3.677)	2.355 (1.340 - 4.007)	2.458 (1.410 - 4.174)
50 - 69 years (rate per capita)	0.635 (0.601 - 0.675)	0.633 (0.599 - 0.674)	0.632 (0.598 - 0.670)	1.054 (0.948 - 1.154)	1.054 (0.947 - 1.152)	1.053 (0.947 - 1.151)	6.882 (4.370 - 10.655)	6.861 (4.287 - 10.575)	6.799 (4.258 - 10.557)
70+ years (rate per capita)	0.668 (0.641 - 0.692)	0.667 (0.643 - 0.692)	0.662 (0.636 - 0.686)	1.005 (0.907 - 1.099)	1.003 (0.907 - 1.098)	0.994 (0.902 - 1.088)	11.334 (7.510 - 15.846)	11.336 (7.509 - 15.903)	11.194 (7.407 - 15.813)
All ages (rate per capita)	0.531 (0.512 - 0.553)	0.550 (0.532 - 0.572)	0.561 (0.541 - 0.580)	0.837 (0.784 - 0.892)	0.871 (0.820 - 0.924)	0.886 (0.835 - 0.938)	3.656 (2.266 - 5.607)	4.160 (2.607 - 6.358)	4.463 (2.785 - 6.766)
Age-standardised (rate per capita)	0.513 (0.493 - 0.534)	0.513 (0.494 - 0.535)	0.513 (0.493 - 0.532)	0.805 (0.750 - 0.869)	0.805 (0.750 - 0.869)	0.805 (0.750 - 0.869)	3.144 (1.924 - 4.874)	3.151 (1.935 - 4.860)	3.153 (1.933 - 4.904)
Total number of cases (thousands)	5,289 (5,098 - 5,502)	5,800 (5,610 - 6,031)	6,054 (5,845 - 6,261)	8,328 (7,804 - 8,872)	9,191 (8,645 - 9,742)	9,574 (9,021 - 10,129)	36 (23 - 56)	44 (27 - 67)	48 (30 - 73)
**Spain**									
Under-5 years (rate per capita)	0.107 (0.079 - 0.146)	0.176 (0.118 - 0.245)	0.185 (0.130 - 0.240)	0.141 (0.073 - 0.303)	0.346 (0.105 - 0.702)	0.393 (0.153 - 0.736)	0.041 (0.016 - 0.088)	0.066 (0.022 - 0.144)	0.069 (0.025 - 0.145)
5-14 years (rate per capita)	0.621 (0.585 - 0.656)	0.628 (0.577 - 0.674)	0.575 (0.510 - 0.636)	0.913 (0.769 - 1.040)	0.963 (0.767 - 1.203)	0.790 (0.625 - 0.975)	0.845 (0.437 - 1.450)	0.818 (0.438 - 1.399)	0.773 (0.411 - 1.296)
15 - 49 years (rate per capita)	0.578 (0.560 - 0.597)	0.580 (0.562 - 0.601)	0.584 (0.566 - 0.603)	0.978 (0.919 - 1.035)	0.986 (0.929 - 1.041)	0.989 (0.931 - 1.043)	1.881 (1.114 - 2.974)	1.996 (1.178 - 3.161)	2.077 (1.238 - 3.298)
50 - 69 years (rate per capita)	0.604 (0.583 - 0.627)	0.599 (0.576 - 0.622)	0.601 (0.579 - 0.624)	1.129 (1.059 - 1.195)	1.128 (1.057 - 1.191)	1.126 (1.054 - 1.191)	5.656 (3.698 - 8.249)	5.442 (3.532 - 7.975)	5.448 (3.532 - 7.954)
70+ years (rate per capita)	0.648 (0.630 - 0.665)	0.640 (0.623 - 0.658)	0.634 (0.619 - 0.651)	1.105 (1.047 - 1.166)	1.106 (1.052 - 1.161)	1.092 (1.041 - 1.143)	9.873 (6.643 - 13.816)	9.674 (6.462 - 13.411)	9.501 (6.365 - 13.218)
All ages (rate per capita)	0.570 (0.556 - 0.583)	0.576 (0.563 - 0.590)	0.575 (0.562 - 0.589)	0.966 (0.923 - 1.007)	0.997 (0.947 - 1.045)	0.987 (0.942 - 1.028)	3.138 (2.017 - 4.629)	3.460 (2.218 - 5.084)	3.582 (2.314 - 5.256)
Age-standardised (rate per capita)	0.551 (0.537 - 0.564)	0.556 (0.541 - 0.572)	0.548 (0.531 - 0.565)	0.924 (0.880 - 0.967)	0.956 (0.890 - 1.027)	0.928 (0.873 - 0.984)	2.702 (1.711 - 4.021)	2.677 (1.694 - 3.981)	2.689 (1.703 - 3.999)
Total number of cases (thousands)	22,211 (21,658 - 22,706)	24,970 (24,421 - 25,556)	28,049 (27,399 - 28,698)	37,620 (35,951 - 39,210)	43,199 (41,028 - 45,310)	48,139 (45,946 - 50,113)	122 (79 - 180)	150 (96 - 220)	175 (113 - 256)
**Sweden**									
Under-5 years (rate per capita)	0.111 (0.098 - 0.125)	0.133 (0.113 - 0.156)	0.191 (0.150 - 0.229)	0.139 (0.098 - 0.195)	0.208 (0.123 - 0.368)	0.396 (0.162 - 0.705)	0.042 (0.017 - 0.085)	0.050 (0.021 - 0.100)	0.072 (0.027 - 0.153)
5-14 years (rate per capita)	0.387 (0.339 - 0.438)	0.453 (0.400 - 0.505)	0.468 (0.424 - 0.513)	0.416 (0.310 - 0.560)	0.520 (0.391 - 0.710)	0.514 (0.388 - 0.668)	0.599 (0.331 - 0.985)	0.647 (0.355 - 1.100)	0.634 (0.348 - 1.041)
15 - 49 years (rate per capita)	0.474 (0.457 - 0.494)	0.479 (0.462 - 0.497)	0.478 (0.462 - 0.497)	0.578 (0.521 - 0.635)	0.589 (0.534 - 0.646)	0.592 (0.535 - 0.649)	2.097 (1.196 - 3.434)	2.117 (1.207 - 3.481)	2.125 (1.207 - 3.470)
50 - 69 years (rate per capita)	0.583 (0.560 - 0.607)	0.592 (0.568 - 0.614)	0.596 (0.574 - 0.616)	0.681 (0.605 - 0.767)	0.690 (0.613 - 0.784)	0.689 (0.612 - 0.775)	5.959 (3.606 - 9.080)	5.754 (3.487 - 8.824)	6.070 (3.725 - 9.255)
70+ years (rate per capita)	0.595 (0.576 - 0.617)	0.596 (0.578 - 0.615)	0.594 (0.576 - 0.614)	0.616 (0.544 - 0.691)	0.618 (0.556 - 0.681)	0.619 (0.556 - 0.685)	8.696 (5.656 - 12.786)	8.358 (5.390 - 12.186)	8.409 (5.454 - 12.207)
All ages (rate per capita)	0.478 (0.465 - 0.491)	0.499 (0.488 - 0.512)	0.504 (0.492 - 0.516)	0.557 (0.520 - 0.597)	0.589 (0.550 - 0.632)	0.599 (0.557 - 0.645)	3.414 (2.081 - 5.229)	3.505 (2.122 - 5.350)	3.626 (2.210 - 5.526)
Age-standardised (rate per capita)	0.450 (0.436 - 0.463)	0.468 (0.456 - 0.481)	0.473 (0.459 - 0.486)	0.526 (0.488 - 0.569)	0.561 (0.517 - 0.613)	0.573 (0.525 - 0.626)	2.678 (1.597 - 4.129)	2.679 (1.593 - 4.137)	2.690 (1.608 - 4.116)
Total number of cases (thousands)	4,090 (3,979 - 4,198)	4,504 (4,398 - 4,613)	4,943 (4,823 - 5,058)	4,766 (4,449 - 5,106)	5,311 (4,961 - 5,702)	5,880 (5,462 - 6,328)	29 (18 - 45)	32 (19 - 48)	36 (22 - 54)
**Switzerland**									
Under-5 years (rate per capita)	0.230 (0.163 - 0.292)	0.230 (0.160 - 0.294)	0.228 (0.166 - 0.290)	0.432 (0.186 - 0.708)	0.436 (0.188 - 0.714)	0.432 (0.186 - 0.707)	0.086 (0.032 - 0.186)	0.087 (0.031 - 0.191)	0.086 (0.031 - 0.190)
5-14 years (rate per capita)	0.443 (0.373 - 0.512)	0.439 (0.376 - 0.516)	0.443 (0.375 - 0.515)	0.767 (0.545 - 1.027)	0.760 (0.541 - 1.017)	0.763 (0.541 - 1.021)	0.640 (0.346 - 1.081)	0.641 (0.344 - 1.081)	0.641 (0.350 - 1.092)
15 - 49 years (rate per capita)	0.520 (0.478 - 0.561)	0.525 (0.485 - 0.565)	0.525 (0.482 - 0.567)	0.866 (0.743 - 0.982)	0.871 (0.749 - 0.987)	0.871 (0.749 - 0.985)	2.452 (1.432 - 4.107)	2.555 (1.494 - 4.284)	2.570 (1.501 - 4.299)
50 - 69 years (rate per capita)	0.639 (0.595 - 0.688)	0.636 (0.593 - 0.689)	0.639 (0.596 - 0.692)	0.989 (0.817 - 1.164)	0.988 (0.812 - 1.165)	0.988 (0.814 - 1.162)	7.812 (4.889 - 11.745)	7.725 (4.867 - 11.716)	7.832 (4.932 - 11.836)
70+ years (rate per capita)	0.679 (0.646 - 0.709)	0.676 (0.643 - 0.711)	0.675 (0.643 - 0.708)	0.948 (0.790 - 1.098)	0.945 (0.790 - 1.094)	0.941 (0.790 - 1.086)	12.832 (8.505 - 17.924)	12.871 (8.533 - 18.103)	12.760 (8.436 - 17.902)
All ages (rate per capita)	0.535 (0.506 - 0.564)	0.543 (0.516 - 0.573)	0.549 (0.519 - 0.579)	0.863 (0.789 - 0.943)	0.872 (0.796 - 0.949)	0.877 (0.799 - 0.954)	4.267 (2.683 - 6.412)	4.589 (2.877 - 6.930)	4.830 (3.021 - 7.280)
Age-standardised (rate per capita)	0.508 (0.480 - 0.538)	0.508 (0.479 - 0.539)	0.509 (0.479 - 0.538)	0.833 (0.758 - 0.915)	0.833 (0.759 - 0.914)	0.833 (0.759 - 0.915)	3.483 (2.169 - 5.298)	3.499 (2.178 - 5.318)	3.502 (2.160 - 5.349)
Total number of cases (thousands)	3,589 (3,396 - 3,781)	4,040 (3,836 - 4,264)	4,546 (4,297 - 4,794)	5,790 (5,290 - 6,324)	6,487 (5,919 - 7,054)	7,259 (6,618 - 7,896)	29 (18 - 43)	34 (21 - 52)	40 (25 - 60)
**United Kingdom**									
Under-5 years (rate per capita)	0.182 (0.166 - 0.197)	0.197 (0.175 - 0.219)	0.205 (0.182 - 0.226)	0.354 (0.178 - 0.570)	0.412 (0.179 - 0.684)	0.436 (0.183 - 0.723)	0.069 (0.028 - 0.141)	0.075 (0.030 - 0.154)	0.078 (0.030 - 0.160)
5-14 years (rate per capita)	0.522 (0.501 - 0.544)	0.516 (0.493 - 0.538)	0.520 (0.498 - 0.544)	0.683 (0.542 - 0.848)	0.704 (0.553 - 0.876)	0.708 (0.558 - 0.880)	0.703 (0.379 - 1.176)	0.699 (0.378 - 1.162)	0.699 (0.379 - 1.161)
15 - 49 years (rate per capita)	0.530 (0.521 - 0.539)	0.534 (0.524 - 0.543)	0.533 (0.523 - 0.543)	0.861 (0.792 - 0.926)	0.868 (0.804 - 0.937)	0.871 (0.805 - 0.938)	1.997 (1.153 - 3.246)	2.047 (1.186 - 3.306)	2.068 (1.192 - 3.343)
50 - 69 years (rate per capita)	0.602 (0.592 - 0.614)	0.597 (0.588 - 0.607)	0.599 (0.590 - 0.610)	1.035 (0.934 - 1.128)	1.032 (0.932 - 1.127)	1.034 (0.936 - 1.128)	5.743 (3.629 - 8.540)	5.458 (3.422 - 8.176)	5.649 (3.555 - 8.418)
70+ years (rate per capita)	0.632 (0.623 - 0.640)	0.624 (0.616 - 0.631)	0.622 (0.613 - 0.631)	1.004 (0.915 - 1.086)	0.998 (0.911 - 1.080)	0.995 (0.909 - 1.076)	9.481 (6.253 - 13.284)	9.228 (6.083 - 12.952)	9.193 (6.060 - 12.876)
All ages (rate per capita)	0.532 (0.526 - 0.538)	0.537 (0.530 - 0.544)	0.538 (0.531 - 0.545)	0.856 (0.808 - 0.904)	0.873 (0.823 - 0.923)	0.881 (0.829 - 0.929)	3.282 (2.052 - 4.936)	3.353 (2.085 - 5.050)	3.524 (2.203 - 5.289)
Age-standardised (rate per capita)	0.514 (0.507 - 0.521)	0.516 (0.508 - 0.523)	0.516 (0.509 - 0.524)	0.821 (0.771 - 0.874)	0.832 (0.777 - 0.890)	0.834 (0.778 - 0.893)	2.699 (1.670 - 4.099)	2.666 (1.647 - 4.054)	2.689 (1.664 - 4.086)
Total number of cases (thousands)	30,433 (30,092 - 30,799)	32,344 (31,950 - 32,773)	34,544 (34,106 - 34,988)	49,015 (46,267 - 51,766)	52,631 (49,574 - 55,594)	56,577 (53,250 - 59,679)	188 (117 - 283)	202 (126 - 304)	226 (142 - 340)
**England**									
Under-5 years (rate per capita)	0.176 (0.157 - 0.194)	0.201 (0.177 - 0.224)	0.204 (0.178 - 0.227)	0.352 (0.165 - 0.574)	0.437 (0.181 - 0.733)	0.444 (0.182 - 0.747)	0.066 (0.027 - 0.137)	0.076 (0.029 - 0.156)	0.077 (0.029 - 0.159)
5-14 years (rate per capita)	0.512 (0.487 - 0.536)	0.508 (0.486 - 0.535)	0.511 (0.487 - 0.536)	0.679 (0.532 - 0.853)	0.692 (0.543 - 0.864)	0.696 (0.545 - 0.867)	0.692 (0.374 - 1.154)	0.689 (0.373 - 1.148)	0.689 (0.373 - 1.149)
15 - 49 years (rate per capita)	0.524 (0.514 - 0.533)	0.527 (0.517 - 0.537)	0.526 (0.516 - 0.537)	0.862 (0.792 - 0.929)	0.870 (0.804 - 0.941)	0.872 (0.806 - 0.943)	1.991 (1.148 - 3.236)	2.039 (1.180 - 3.293)	2.061 (1.189 - 3.328)
50 - 69 years (rate per capita)	0.597 (0.586 - 0.609)	0.591 (0.580 - 0.602)	0.593 (0.582 - 0.605)	1.037 (0.934 - 1.135)	1.035 (0.930 - 1.135)	1.037 (0.934 - 1.134)	5.742 (3.628 - 8.536)	5.453 (3.415 - 8.163)	5.652 (3.560 - 8.424)
70+ years (rate per capita)	0.627 (0.618 - 0.637)	0.619 (0.610 - 0.628)	0.616 (0.608 - 0.626)	1.004 (0.911 - 1.091)	0.998 (0.905 - 1.084)	0.995 (0.905 - 1.079)	9.479 (6.257 - 13.304)	9.224 (6.079 - 12.959)	9.188 (6.068 - 12.864)
All ages (rate per capita)	0.525 (0.519 - 0.532)	0.530 (0.523 - 0.538)	0.531 (0.524 - 0.538)	0.857 (0.806 - 0.906)	0.874 (0.822 - 0.924)	0.880 (0.826 - 0.929)	3.284 (2.055 - 4.942)	3.340 (2.075 - 5.026)	3.501 (2.190 - 5.257)
Age-standardised (rate per capita)	0.507 (0.500 - 0.514)	0.510 (0.502 - 0.518)	0.510 (0.502 - 0.518)	0.821 (0.769 - 0.875)	0.833 (0.776 - 0.893)	0.833 (0.777 - 0.896)	2.692 (1.665 - 4.088)	2.661 (1.644 - 4.044)	2.684 (1.660 - 4.076)
Total number of cases (thousands)	25,051 (24,746 - 25,378)	26,783 (26,419 - 27,173)	28,665 (28,275 - 29,062)	40,879 (38,428 - 43,238)	44,134 (41,488 - 46,651)	47,530 (44,627 - 50,183)	157 (98 - 236)	169 (105 - 254)	189 (118 - 284)
**Northern Ireland**									
Under-5 years (rate per capita)	0.240 (0.222 - 0.259)	0.293 (0.254 - 0.329)	0.291 (0.252 - 0.324)	0.391 (0.330 - 0.441)	0.547 (0.293 - 0.793)	0.546 (0.289 - 0.784)	0.092 (0.040 - 0.180)	0.112 (0.047 - 0.228)	0.112 (0.046 - 0.229)
5-14 years (rate per capita)	0.561 (0.512 - 0.612)	0.553 (0.505 - 0.607)	0.554 (0.503 - 0.609)	0.836 (0.680 - 1.053)	0.832 (0.677 - 1.053)	0.835 (0.673 - 1.055)	0.723 (0.386 - 1.208)	0.716 (0.384 - 1.191)	0.717 (0.387 - 1.201)
15 - 49 years (rate per capita)	0.522 (0.502 - 0.542)	0.527 (0.508 - 0.548)	0.529 (0.510 - 0.550)	0.855 (0.782 - 0.924)	0.865 (0.800 - 0.935)	0.872 (0.806 - 0.941)	1.937 (1.119 - 3.125)	2.003 (1.152 - 3.227)	2.056 (1.187 - 3.348)
50 - 69 years (rate per capita)	0.600 (0.578 - 0.624)	0.593 (0.569 - 0.617)	0.594 (0.571 - 0.617)	1.038 (0.934 - 1.138)	1.036 (0.932 - 1.136)	1.037 (0.932 - 1.135)	5.696 (3.574 - 8.494)	5.425 (3.422 - 8.137)	5.536 (3.487 - 8.286)
70+ years (rate per capita)	0.631 (0.612 - 0.650)	0.624 (0.608 - 0.644)	0.621 (0.605 - 0.642)	1.009 (0.914 - 1.096)	1.005 (0.911 - 1.093)	1.002 (0.909 - 1.087)	9.493 (6.274 - 13.374)	9.268 (6.116 - 13.091)	9.227 (6.079 - 12.963)
All ages (rate per capita)	0.528 (0.515 - 0.541)	0.538 (0.525 - 0.552)	0.540 (0.527 - 0.555)	0.859 (0.808 - 0.908)	0.888 (0.832 - 0.944)	0.897 (0.842 - 0.951)	2.914 (1.811 - 4.397)	3.096 (1.915 - 4.674)	3.344 (2.080 - 5.039)
Age-standardised (rate per capita)	0.524 (0.511 - 0.538)	0.528 (0.514 - 0.542)	0.528 (0.513 - 0.544)	0.852 (0.801 - 0.903)	0.868 (0.807 - 0.932)	0.868 (0.808 - 0.934)	2.700 (1.667 - 4.106)	2.665 (1.639 - 4.077)	2.687 (1.664 - 4.091)
Total number of cases (thousands)	843 (823 - 865)	928 (904 - 951)	990 (965 - 1,017)	1,372 (1,291 - 1,451)	1,531 (1,434 - 1,627)	1,644 (1,542 - 1,742)	5 (3 - 7)	5 (3 - 8)	6 (4 - 9)
**Scotland**									
Under-5 years (rate per capita)	0.193 (0.164 - 0.228)	0.125 (0.108 - 0.146)	0.162 (0.133 - 0.189)	0.286 (0.183 - 0.469)	0.170 (0.112 - 0.282)	0.273 (0.144 - 0.444)	0.075 (0.030 - 0.150)	0.048 (0.020 - 0.098)	0.062 (0.025 - 0.124)
5-14 years (rate per capita)	0.589 (0.550 - 0.623)	0.583 (0.531 - 0.633)	0.600 (0.550 - 0.652)	0.645 (0.533 - 0.765)	0.790 (0.634 - 0.982)	0.790 (0.638 - 0.959)	0.791 (0.421 - 1.355)	0.793 (0.421 - 1.341)	0.799 (0.423 - 1.362)
15 - 49 years (rate per capita)	0.597 (0.580 - 0.616)	0.610 (0.594 - 0.628)	0.609 (0.594 - 0.627)	0.853 (0.792 - 0.914)	0.856 (0.796 - 0.914)	0.857 (0.798 - 0.916)	2.061 (1.180 - 3.346)	2.153 (1.242 - 3.526)	2.166 (1.246 - 3.541)
50 - 69 years (rate per capita)	0.655 (0.637 - 0.673)	0.660 (0.643 - 0.680)	0.661 (0.643 - 0.680)	1.015 (0.932 - 1.092)	1.003 (0.925 - 1.073)	1.003 (0.927 - 1.073)	5.750 (3.619 - 8.528)	5.512 (3.435 - 8.276)	5.633 (3.537 - 8.433)
70+ years (rate per capita)	0.676 (0.662 - 0.692)	0.676 (0.661 - 0.692)	0.674 (0.659 - 0.689)	1.000 (0.925 - 1.067)	0.997 (0.931 - 1.057)	0.994 (0.929 - 1.053)	9.518 (6.279 - 13.339)	9.296 (6.173 - 13.047)	9.267 (6.110 - 13.053)
All ages (rate per capita)	0.590 (0.579 - 0.602)	0.601 (0.589 - 0.613)	0.606 (0.593 - 0.619)	0.840 (0.797 - 0.882)	0.863 (0.818 - 0.907)	0.875 (0.830 - 0.918)	3.309 (2.052 - 5.002)	3.499 (2.180 - 5.303)	3.713 (2.307 - 5.587)
Age-standardised (rate per capita)	0.573 (0.561 - 0.585)	0.573 (0.561 - 0.587)	0.579 (0.566 - 0.594)	0.802 (0.755 - 0.846)	0.814 (0.766 - 0.861)	0.822 (0.773 - 0.871)	2.761 (1.691 - 4.207)	2.733 (1.680 - 4.177)	2.756 (1.693 - 4.204)
Total number of cases (thousands)	3,000 (2,942 - 3,060)	3,053 (2,994 - 3,117)	3,226 (3,158 - 3,292)	4,267 (4,047 - 4,480)	4,385 (4,159 - 4,611)	4,656 (4,416 - 4,887)	17 (10 - 25)	18 (11 - 27)	20 (12 - 30)
**Wales**									
Under-5 years (rate per capita)	0.232 (0.187 - 0.273)	0.181 (0.146 - 0.219)	0.231 (0.182 - 0.276)	0.474 (0.222 - 0.753)	0.275 (0.158 - 0.512)	0.470 (0.211 - 0.756)	0.088 (0.035 - 0.183)	0.069 (0.027 - 0.138)	0.088 (0.034 - 0.180)
5-14 years (rate per capita)	0.552 (0.498 - 0.603)	0.506 (0.455 - 0.561)	0.533 (0.483 - 0.590)	0.706 (0.559 - 0.873)	0.681 (0.540 - 0.849)	0.705 (0.560 - 0.870)	0.713 (0.385 - 1.206)	0.690 (0.372 - 1.153)	0.702 (0.381 - 1.161)

	Prevalence 1990	Prevalence 2005	Prevalence 2015	Incidence 1990	Incidence 2005	Incidence 2015	YLD rate 1990	YLD rate 2005	YLD rate 2015
15 - 49 years (rate per capita)	0.527 (0.508 - 0.548)	0.527 (0.508 - 0.548)	0.524 (0.506 - 0.547)	0.864 (0.793 - 0.931)	0.869 (0.803 - 0.939)	0.870 (0.803 - 0.939)	2.001 (1.153 - 3.221)	2.029 (1.172 - 3.265)	2.035 (1.182 - 3.296)
50 - 69 years (rate per capita)	0.600 (0.577 - 0.624)	0.594 (0.570 - 0.619)	0.597 (0.574 - 0.620)	1.040 (0.935 - 1.137)	1.038 (0.931 - 1.138)	1.040 (0.934 - 1.138)	5.774 (3.656 - 8.585)	5.458 (3.423 - 8.168)	5.688 (3.605 - 8.457)
70+ years (rate per capita)	0.630 (0.611 - 0.651)	0.621 (0.604 - 0.642)	0.619 (0.602 - 0.640)	1.009 (0.914 - 1.096)	1.001 (0.908 - 1.088)	1.000 (0.907 - 1.085)	9.442 (6.264 - 13.262)	9.158 (6.036 - 12.912)	9.141 (6.039 - 12.894)
All ages (rate per capita)	0.538 (0.525 - 0.552)	0.534 (0.520 - 0.549)	0.540 (0.527 - 0.554)	0.874 (0.820 - 0.924)	0.872 (0.823 - 0.919)	0.893 (0.839 - 0.941)	3.391 (2.131 - 5.093)	3.488 (2.176 - 5.233)	3.706 (2.340 - 5.537)
Age-standardised (rate per capita)	0.521 (0.507 - 0.536)	0.510 (0.494 - 0.526)	0.518 (0.503 - 0.534)	0.837 (0.781 - 0.896)	0.818 (0.767 - 0.870)	0.839 (0.780 - 0.898)	2.691 (1.672 - 4.075)	2.650 (1.633 - 4.031)	2.675 (1.648 - 4.059)
Total number of cases (thousands)	1,539 (1,501 - 1,577)	1,580 (1,539 - 1,624)	1,663 (1,621 - 1,704)	2,497 (2,343 - 2,642)	2,580 (2,436 - 2,720)	2,747 (2,582 - 2,896)	10 (6 - 15)	10 (6 - 15)	11 (7 - 17)
**Southern Latin America**									
Under-5 years (rate per capita)	0.255 (0.211 - 0.296)	0.243 (0.197 - 0.287)	0.241 (0.192 - 0.285)	0.470 (0.224 - 0.721)	0.435 (0.189 - 0.721)	0.437 (0.193 - 0.720)	0.098 (0.039 - 0.199)	0.092 (0.035 - 0.191)	0.092 (0.035 - 0.193)
5-14 years (rate per capita)	0.516 (0.470 - 0.560)	0.515 (0.470 - 0.558)	0.514 (0.468 - 0.561)	0.624 (0.462 - 0.828)	0.620 (0.462 - 0.817)	0.620 (0.459 - 0.822)	0.699 (0.376 - 1.169)	0.700 (0.377 - 1.174)	0.698 (0.376 - 1.186)
15 - 49 years (rate per capita)	0.540 (0.525 - 0.557)	0.543 (0.528 - 0.560)	0.546 (0.531 - 0.563)	0.757 (0.694 - 0.824)	0.763 (0.699 - 0.829)	0.768 (0.705 - 0.836)	2.042 (1.163 - 3.372)	2.092 (1.186 - 3.454)	2.111 (1.197 - 3.472)
50 - 69 years (rate per capita)	0.618 (0.598 - 0.638)	0.619 (0.600 - 0.640)	0.619 (0.602 - 0.639)	0.949 (0.846 - 1.052)	0.947 (0.847 - 1.052)	0.948 (0.846 - 1.053)	5.747 (3.522 - 8.664)	5.689 (3.495 - 8.549)	5.732 (3.512 - 8.749)
70+ years (rate per capita)	0.629 (0.612 - 0.648)	0.624 (0.608 - 0.640)	0.618 (0.604 - 0.632)	0.876 (0.776 - 0.975)	0.869 (0.774 - 0.965)	0.863 (0.772 - 0.957)	8.850 (5.750 - 12.749)	8.730 (5.659 - 12.584)	8.604 (5.587 - 12.431)
All ages (rate per capita)	0.523 (0.509 - 0.536)	0.530 (0.517 - 0.543)	0.536 (0.524 - 0.548)	0.737 (0.680 - 0.802)	0.747 (0.693 - 0.807)	0.759 (0.704 - 0.816)	2.520 (1.505 - 3.894)	2.723 (1.632 - 4.190)	2.889 (1.740 - 4.460)
Age-standardised (rate per capita)	0.532 (0.519 - 0.544)	0.531 (0.518 - 0.544)	0.530 (0.517 - 0.543)	0.754 (0.698 - 0.811)	0.750 (0.696 - 0.810)	0.750 (0.695 - 0.810)	2.749 (1.649 - 4.238)	2.763 (1.657 - 4.253)	2.753 (1.656 - 4.255)
Total number of cases (thousands)	25,596 (24,916 - 26,276)	31,040 (30,251 - 31,783)	34,737 (33,925 - 35,511)	36,118 (33,316 - 39,271)	43,771 (40,606 - 47,280)	49,182 (45,626 - 52,845)	123 (74 - 191)	159 (96 - 245)	187 (113 - 289)
**Argentina**									
Under-5 years (rate per capita)	0.228 (0.168 - 0.285)	0.230 (0.172 - 0.285)	0.229 (0.167 - 0.284)	0.427 (0.179 - 0.720)	0.426 (0.173 - 0.720)	0.429 (0.180 - 0.723)	0.087 (0.033 - 0.180)	0.086 (0.033 - 0.179)	0.087 (0.033 - 0.184)
5-14 years (rate per capita)	0.509 (0.451 - 0.565)	0.511 (0.456 - 0.565)	0.510 (0.450 - 0.570)	0.610 (0.452 - 0.808)	0.611 (0.454 - 0.811)	0.612 (0.453 - 0.811)	0.696 (0.375 - 1.165)	0.699 (0.374 - 1.161)	0.696 (0.373 - 1.181)
15 - 49 years (rate per capita)	0.535 (0.515 - 0.558)	0.537 (0.517 - 0.559)	0.541 (0.522 - 0.564)	0.758 (0.695 - 0.825)	0.759 (0.692 - 0.824)	0.765 (0.702 - 0.833)	1.994 (1.138 - 3.248)	2.009 (1.143 - 3.305)	2.031 (1.158 - 3.326)
50 - 69 years (rate per capita)	0.609 (0.584 - 0.635)	0.610 (0.583 - 0.638)	0.609 (0.585 - 0.635)	0.948 (0.846 - 1.054)	0.946 (0.841 - 1.054)	0.947 (0.846 - 1.053)	5.646 (3.504 - 8.418)	5.573 (3.448 - 8.363)	5.615 (3.472 - 8.448)
70+ years (rate per capita)	0.625 (0.602 - 0.649)	0.619 (0.600 - 0.640)	0.613 (0.593 - 0.632)	0.878 (0.773 - 0.980)	0.869 (0.777 - 0.966)	0.864 (0.767 - 0.958)	8.808 (5.748 - 12.637)	8.664 (5.637 - 12.426)	8.534 (5.584 - 12.305)
All ages (rate per capita)	0.515 (0.496 - 0.534)	0.522 (0.504 - 0.539)	0.526 (0.509 - 0.544)	0.731 (0.674 - 0.796)	0.741 (0.683 - 0.800)	0.750 (0.695 - 0.809)	2.515 (1.517 - 3.872)	2.649 (1.603 - 4.049)	2.750 (1.674 - 4.218)
Age-standardised (rate per capita)	0.524 (0.506 - 0.541)	0.525 (0.508 - 0.541)	0.524 (0.506 - 0.541)	0.747 (0.691 - 0.807)	0.747 (0.690 - 0.805)	0.747 (0.691 - 0.807)	2.690 (1.621 - 4.145)	2.704 (1.636 - 4.138)	2.690 (1.634 - 4.140)
Total number of cases (thousands)	16,855 (16,227 - 17,463)	20,417 (19,715 - 21,087)	22,852 (22,087 - 23,597)	23,919 (22,040 - 26,032)	28,978 (26,738 - 31,325)	32,538 (30,183 - 35,104)	82 (50 - 127)	104 (63 - 158)	119 (73 - 183)
**Chile**									
Under-5 years (rate per capita)	0.323 (0.283 - 0.361)	0.277 (0.222 - 0.330)	0.277 (0.218 - 0.334)	0.582 (0.334 - 0.763)	0.463 (0.226 - 0.723)	0.461 (0.225 - 0.722)	0.125 (0.053 - 0.252)	0.106 (0.042 - 0.215)	0.106 (0.042 - 0.215)
5-14 years (rate per capita)	0.538 (0.481 - 0.592)	0.526 (0.472 - 0.579)	0.528 (0.474 - 0.582)	0.659 (0.487 - 0.888)	0.641 (0.477 - 0.860)	0.642 (0.478 - 0.861)	0.708 (0.382 - 1.201)	0.705 (0.378 - 1.197)	0.706 (0.381 - 1.196)
15 - 49 years (rate per capita)	0.549 (0.530 - 0.572)	0.555 (0.537 - 0.578)	0.558 (0.539 - 0.580)	0.756 (0.693 - 0.828)	0.770 (0.708 - 0.839)	0.776 (0.712 - 0.845)	2.138 (1.205 - 3.585)	2.269 (1.282 - 3.784)	2.292 (1.293 - 3.831)
50 - 69 years (rate per capita)	0.643 (0.618 - 0.670)	0.641 (0.613 - 0.668)	0.641 (0.615 - 0.667)	0.951 (0.855 - 1.055)	0.949 (0.850 - 1.053)	0.950 (0.853 - 1.055)	6.013 (3.639 - 9.289)	5.929 (3.594 - 9.178)	5.956 (3.605 - 9.114)
70+ years (rate per capita)	0.642 (0.623 - 0.666)	0.638 (0.619 - 0.660)	0.632 (0.613 - 0.655)	0.873 (0.776 - 0.971)	0.869 (0.774 - 0.966)	0.864 (0.772 - 0.959)	9.001 (5.768 - 12.942)	8.922 (5.743 - 12.887)	8.799 (5.647 - 12.698)
All ages (rate per capita)	0.539 (0.523 - 0.555)	0.548 (0.533 - 0.565)	0.558 (0.543 - 0.574)	0.749 (0.689 - 0.812)	0.760 (0.705 - 0.820)	0.779 (0.727 - 0.834)	2.435 (1.436 - 3.836)	2.833 (1.682 - 4.426)	3.169 (1.878 - 4.907)
Age-standardised (rate per capita)	0.552 (0.537 - 0.567)	0.546 (0.530 - 0.563)	0.546 (0.529 - 0.562)	0.770 (0.715 - 0.830)	0.757 (0.700 - 0.818)	0.757 (0.700 - 0.818)	2.900 (1.726 - 4.522)	2.896 (1.726 - 4.499)	2.887 (1.715 - 4.469)
Total number of cases (thousands)	7,079 (6,873 - 7,295)	8,823 (8,574 - 9,091)	10,019 (9,739 - 10,308)	9,846 (9,060 - 10,671)	12,242 (11,350 - 13,196)	13,986 (13,046 - 14,971)	32 (19 - 50)	46 (27 - 71)	57 (34 - 88)
**Uruguay**									
Under-5 years (rate per capita)	0.253 (0.190 - 0.314)	0.256 (0.190 - 0.323)	0.254 (0.190 - 0.317)	0.438 (0.208 - 0.709)	0.442 (0.210 - 0.715)	0.441 (0.210 - 0.714)	0.097 (0.039 - 0.205)	0.098 (0.039 - 0.210)	0.098 (0.037 - 0.211)
5-14 years (rate per capita)	0.505 (0.448 - 0.562)	0.508 (0.451 - 0.567)	0.508 (0.448 - 0.567)	0.640 (0.469 - 0.869)	0.642 (0.470 - 0.872)	0.643 (0.470 - 0.872)	0.696 (0.375 - 1.168)	0.695 (0.378 - 1.176)	0.695 (0.378 - 1.153)
15 - 49 years (rate per capita)	0.543 (0.524 - 0.565)	0.547 (0.529 - 0.569)	0.547 (0.527 - 0.569)	0.759 (0.697 - 0.828)	0.767 (0.704 - 0.835)	0.769 (0.707 - 0.838)	2.090 (1.187 - 3.447)	2.138 (1.210 - 3.518)	2.142 (1.214 - 3.497)
50 - 69 years (rate per capita)	0.625 (0.600 - 0.650)	0.624 (0.599 - 0.651)	0.624 (0.599 - 0.651)	0.952 (0.856 - 1.052)	0.950 (0.855 - 1.052)	0.950 (0.855 - 1.052)	5.890 (3.607 - 8.860)	5.854 (3.615 - 8.863)	5.816 (3.585 - 8.758)
70+ years (rate per capita)	0.631 (0.612 - 0.651)	0.627 (0.609 - 0.647)	0.616 (0.599 - 0.635)	0.873 (0.775 - 0.973)	0.868 (0.773 - 0.965)	0.857 (0.769 - 0.952)	8.825 (5.784 - 12.636)	8.739 (5.705 - 12.505)	8.503 (5.574 - 12.161)
All ages (rate per capita)	0.534 (0.517 - 0.551)	0.540 (0.524 - 0.557)	0.543 (0.527 - 0.559)	0.756 (0.699 - 0.818)	0.765 (0.709 - 0.824)	0.773 (0.719 - 0.829)	2.919 (1.776 - 4.483)	3.065 (1.863 - 4.715)	3.186 (1.947 - 4.856)
Age-standardised (rate per capita)	0.533 (0.515 - 0.550)	0.532 (0.516 - 0.550)	0.532 (0.514 - 0.549)	0.754 (0.697 - 0.818)	0.754 (0.697 - 0.818)	0.754 (0.697 - 0.818)	2.775 (1.671 - 4.280)	2.778 (1.673 - 4.297)	2.775 (1.680 - 4.272)
Total number of cases (thousands)	1,660 (1,607 - 1,714)	1,798 (1,745 - 1,854)	1,865 (1,809 - 1,919)	2,353 (2,174 - 2,546)	2,548 (2,363 - 2,745)	2,655 (2,469 - 2,846)	9 (6 - 14)	10 (6 - 16)	11 (7 - 17)
**Central Europe, Eastern Europe, and Central Asia**									
Under-5 years (rate per capita)	0.419 (0.392 - 0.448)	0.419 (0.394 - 0.448)	0.420 (0.393 - 0.449)	0.608 (0.422 - 0.778)	0.619 (0.429 - 0.786)	0.618 (0.427 - 0.791)	0.141 (0.061 - 0.278)	0.139 (0.060 - 0.272)	0.140 (0.060 - 0.273)
5-14 years (rate per capita)	0.620 (0.596 - 0.647)	0.606 (0.584 - 0.627)	0.621 (0.598 - 0.646)	1.094 (0.922 - 1.309)	1.053 (0.876 - 1.259)	1.099 (0.920 - 1.324)	0.768 (0.409 - 1.294)	0.750 (0.398 - 1.258)	0.746 (0.398 - 1.240)
15 - 49 years (rate per capita)	0.535 (0.523 - 0.549)	0.536 (0.526 - 0.548)	0.534 (0.523 - 0.546)	0.853 (0.777 - 0.930)	0.852 (0.777 - 0.924)	0.856 (0.779 - 0.932)	2.000 (1.169 - 3.199)	2.047 (1.198 - 3.250)	2.079 (1.224 - 3.292)
50 - 69 years (rate per capita)	0.602 (0.587 - 0.617)	0.603 (0.589 - 0.617)	0.600 (0.584 - 0.615)	0.963 (0.853 - 1.074)	0.960 (0.852 - 1.065)	0.958 (0.841 - 1.069)	6.142 (3.935 - 9.010)	6.155 (3.942 - 9.008)	5.992 (3.818 - 8.780)
70+ years (rate per capita)	0.653 (0.643 - 0.665)	0.659 (0.648 - 0.671)	0.649 (0.638 - 0.661)	0.945 (0.843 - 1.041)	0.948 (0.842 - 1.049)	0.945 (0.845 - 1.041)	10.460 (6.935 - 14.668)	10.548 (6.973 - 14.764)	10.305 (6.828 - 14.465)
All ages (rate per capita)	0.560 (0.551 - 0.569)	0.562 (0.554 - 0.569)	0.563 (0.554 - 0.571)	0.900 (0.842 - 0.959)	0.894 (0.838 - 0.946)	0.899 (0.843 - 0.951)	2.983 (1.853 - 4.426)	3.302 (2.065 - 4.890)	3.548 (2.227 - 5.242)
Age-standardised (rate per capita)	0.558 (0.549 - 0.567)	0.559 (0.550 - 0.567)	0.558 (0.549 - 0.566)	0.896 (0.838 - 0.954)	0.895 (0.837 - 0.953)	0.894 (0.835 - 0.954)	2.912 (1.812 - 4.323)	2.910 (1.811 - 4.314)	2.914 (1.814 - 4.323)
Total number of cases (thousands)	231,327 (227,606 - 234,980)	228,248 (225,001 - 231,252)	235,622 (232,158 - 239,042)	371,826 (347,780 - 396,251)	363,091 (340,262 - 384,162)	376,443 (353,184 - 398,199)	1,232 (766 - 1,829)	1,341 (839 - 1,986)	1,486 (932 - 2,195)
**Eastern Europe**									
Under-5 years (rate per capita)	0.361 (0.321 - 0.407)	0.358 (0.318 - 0.406)	0.363 (0.320 - 0.408)	0.568 (0.358 - 0.774)	0.590 (0.363 - 0.780)	0.592 (0.366 - 0.791)	0.137 (0.058 - 0.275)	0.137 (0.058 - 0.275)	0.138 (0.057 - 0.280)
5-14 years (rate per capita)	0.610 (0.573 - 0.645)	0.598 (0.568 - 0.631)	0.620 (0.584 - 0.656)	1.099 (0.927 - 1.311)	1.053 (0.887 - 1.250)	1.118 (0.936 - 1.332)	0.802 (0.424 - 1.364)	0.803 (0.427 - 1.364)	0.798 (0.420 - 1.337)
15 - 49 years (rate per capita)	0.523 (0.508 - 0.542)	0.526 (0.512 - 0.544)	0.523 (0.507 - 0.541)	0.885 (0.809 - 0.960)	0.885 (0.811 - 0.957)	0.891 (0.814 - 0.967)	1.989 (1.172 - 3.200)	2.040 (1.209 - 3.241)	2.066 (1.224 - 3.305)
50 - 69 years (rate per capita)	0.585 (0.565 - 0.605)	0.587 (0.569 - 0.606)	0.582 (0.561 - 0.602)	0.990 (0.882 - 1.098)	0.992 (0.884 - 1.090)	0.990 (0.874 - 1.098)	5.859 (3.774 - 8.558)	5.948 (3.832 - 8.691)	5.621 (3.603 - 8.278)
70+ years (rate per capita)	0.640 (0.626 - 0.656)	0.646 (0.631 - 0.662)	0.638 (0.622 - 0.654)	0.973 (0.869 - 1.068)	0.980 (0.873 - 1.078)	0.975 (0.873 - 1.072)	10.269 (6.844 - 14.259)	10.356 (6.887 - 14.303)	10.103 (6.699 - 14.082)
All ages (rate per capita)	0.546 (0.535 - 0.558)	0.550 (0.539 - 0.561)	0.551 (0.540 - 0.562)	0.923 (0.867 - 0.981)	0.920 (0.865 - 0.972)	0.929 (0.872 - 0.981)	3.095 (1.962 - 4.600)	3.421 (2.171 - 5.062)	3.687 (2.344 - 5.422)
Age-standardised (rate per capita)	0.541 (0.529 - 0.553)	0.544 (0.533 - 0.555)	0.542 (0.530 - 0.553)	0.914 (0.854 - 0.973)	0.917 (0.856 - 0.974)	0.917 (0.854 - 0.976)	2.841 (1.787 - 4.242)	2.841 (1.795 - 4.240)	2.844 (1.789 - 4.246)
Total number of cases (thousands)	120,814 (118,312 - 123,406)	115,991 (113,759 - 118,405)	118,439 (116,046 - 120,807)	204,253 (191,726 - 216,930)	194,182 (182,587 - 204,972)	199,676 (187,568 - 210,886)	685 (434 - 1,018)	722 (458 - 1,068)	793 (504 - 1,166)
**Belarus**									
Under-5 years (rate per capita)	0.400 (0.329 - 0.472)	0.392 (0.322 - 0.468)	0.395 (0.329 - 0.472)	0.594 (0.381 - 0.769)	0.580 (0.372 - 0.750)	0.584 (0.375 - 0.756)	0.095 (0.040 - 0.190)	0.093 (0.039 - 0.188)	0.094 (0.040 - 0.187)
5-14 years (rate per capita)	0.588 (0.544 - 0.642)	0.563 (0.515 - 0.618)	0.585 (0.539 - 0.634)	1.033 (0.855 - 1.242)	0.991 (0.821 - 1.193)	1.028 (0.852 - 1.239)	0.585 (0.323 - 0.956)	0.582 (0.319 - 0.954)	0.584 (0.323 - 0.967)
15 - 49 years (rate per capita)	0.487 (0.460 - 0.519)	0.490 (0.465 - 0.518)	0.490 (0.463 - 0.519)	0.837 (0.747 - 0.933)	0.838 (0.752 - 0.928)	0.841 (0.752 - 0.939)	1.733 (1.040 - 2.683)	1.818 (1.089 - 2.814)	1.822 (1.092 - 2.796)
50 - 69 years (rate per capita)	0.558 (0.523 - 0.594)	0.560 (0.526 - 0.591)	0.550 (0.513 - 0.588)	0.931 (0.799 - 1.057)	0.930 (0.799 - 1.054)	0.926 (0.789 - 1.061)	5.625 (3.612 - 8.230)	5.661 (3.652 - 8.262)	5.249 (3.354 - 7.600)
70+ years (rate per capita)	0.610 (0.584 - 0.637)	0.619 (0.594 - 0.649)	0.609 (0.586 - 0.637)	0.906 (0.785 - 1.023)	0.918 (0.790 - 1.043)	0.907 (0.788 - 1.019)	9.714 (6.482 - 13.670)	9.895 (6.599 - 13.883)	9.625 (6.391 - 13.516)
All ages (rate per capita)	0.520 (0.501 - 0.540)	0.521 (0.502 - 0.541)	0.522 (0.503 - 0.541)	0.874 (0.814 - 0.939)	0.870 (0.811 - 0.930)	0.872 (0.812 - 0.932)	2.839 (1.794 - 4.160)	3.193 (2.030 - 4.697)	3.389 (2.155 - 4.918)
Age-standardised (rate per capita)	0.516 (0.497 - 0.536)	0.517 (0.497 - 0.536)	0.517 (0.497 - 0.537)	0.866 (0.806 - 0.931)	0.867 (0.807 - 0.931)	0.867 (0.807 - 0.931)	2.600 (1.644 - 3.826)	2.601 (1.644 - 3.831)	2.596 (1.632 - 3.807)
Total number of cases (thousands)	5,275 (5,080 - 5,472)	5,038 (4,857 - 5,228)	5,014 (4,835 - 5,203)	8,868 (8,258 - 9,519)	8,413 (7,841 - 8,995)	8,381 (7,807 - 8,962)	29 (18 - 42)	31 (20 - 45)	33 (21 - 47)
**Estonia**									
Under-5 years (rate per capita)	0.399 (0.333 - 0.472)	0.388 (0.320 - 0.461)	0.396 (0.328 - 0.468)	0.612 (0.388 - 0.801)	0.592 (0.375 - 0.775)	0.605 (0.384 - 0.792)	0.155 (0.064 - 0.320)	0.150 (0.062 - 0.309)	0.153 (0.062 - 0.313)
5-14 years (rate per capita)	0.512 (0.471 - 0.560)	0.475 (0.431 - 0.522)	0.529 (0.487 - 0.576)	0.959 (0.788 - 1.177)	0.898 (0.743 - 1.091)	0.982 (0.807 - 1.211)	0.667 (0.360 - 1.111)	0.645 (0.350 - 1.072)	0.671 (0.361 - 1.113)
15 - 49 years (rate per capita)	0.383 (0.364 - 0.406)	0.382 (0.363 - 0.404)	0.382 (0.363 - 0.406)	0.787 (0.710 - 0.866)	0.786 (0.712 - 0.860)	0.788 (0.710 - 0.866)	1.858 (1.120 - 2.901)	1.868 (1.114 - 2.926)	1.920 (1.154 - 2.994)

	Prevalence 1990	Prevalence 2005	Prevalence 2015	Incidence 1990	Incidence 2005	Incidence 2015	YLD rate 1990	YLD rate 2005	YLD rate 2015
50 - 69 years (rate per capita)	0.476 (0.454 - 0.499)	0.477 (0.457 - 0.499)	0.474 (0.451 - 0.497)	0.850 (0.752 - 0.960)	0.852 (0.762 - 0.956)	0.850 (0.750 - 0.965)	5.781 (3.702 - 8.447)	5.887 (3.827 - 8.609)	5.741 (3.695 - 8.418)
70+ years (rate per capita)	0.545 (0.525 - 0.566)	0.547 (0.527 - 0.570)	0.540 (0.522 - 0.560)	0.812 (0.726 - 0.904)	0.820 (0.729 - 0.917)	0.808 (0.726 - 0.898)	9.952 (6.625 - 13.959)	10.038 (6.671 - 13.965)	9.852 (6.525 - 13.748)
All ages (rate per capita)	0.436 (0.422 - 0.450)	0.433 (0.420 - 0.446)	0.441 (0.428 - 0.454)	0.815 (0.758 - 0.875)	0.807 (0.754 - 0.860)	0.815 (0.762 - 0.865)	3.033 (1.930 - 4.448)	3.505 (2.247 - 5.105)	3.733 (2.398 - 5.462)
Age-standardised (rate per capita)	0.432 (0.418 - 0.447)	0.429 (0.414 - 0.444)	0.432 (0.417 - 0.446)	0.811 (0.752 - 0.873)	0.806 (0.749 - 0.867)	0.811 (0.752 - 0.873)	2.702 (1.713 - 3.979)	2.698 (1.704 - 3.975)	2.710 (1.711 - 4.005)
Total number of cases (thousands)	681 (660 - 703)	583 (566 - 601)	597 (580 - 614)	1,274 (1,185 - 1,368)	1,087 (1,016 - 1,159)	1,103 (1,032 - 1,172)	5 (3 - 7)	5 (3 - 7)	5 (3 - 7)
**Latvia**									
Under-5 years (rate per capita)	0.364 (0.320 - 0.418)	0.377 (0.316 - 0.444)	0.385 (0.322 - 0.455)	0.565 (0.493 - 0.675)	0.592 (0.374 - 0.786)	0.604 (0.382 - 0.803)	0.141 (0.060 - 0.275)	0.146 (0.060 - 0.291)	0.149 (0.062 - 0.301)
5-14 years (rate per capita)	0.584 (0.539 - 0.632)	0.553 (0.505 - 0.604)	0.597 (0.551 - 0.641)	1.077 (0.893 - 1.298)	1.021 (0.844 - 1.238)	1.102 (0.914 - 1.333)	0.762 (0.404 - 1.284)	0.761 (0.402 - 1.301)	0.763 (0.409 - 1.295)
15 - 49 years (rate per capita)	0.481 (0.454 - 0.509)	0.483 (0.458 - 0.511)	0.482 (0.456 - 0.509)	0.881 (0.785 - 0.980)	0.880 (0.783 - 0.977)	0.884 (0.786 - 0.985)	1.909 (1.117 - 3.039)	1.921 (1.122 - 3.053)	1.961 (1.147 - 3.127)
50 - 69 years (rate per capita)	0.549 (0.513 - 0.584)	0.553 (0.520 - 0.584)	0.547 (0.512 - 0.581)	0.971 (0.828 - 1.107)	0.973 (0.835 - 1.107)	0.969 (0.827 - 1.108)	5.421 (3.443 - 7.915)	5.619 (3.655 - 8.232)	5.348 (3.376 - 7.817)
70+ years (rate per capita)	0.597 (0.574 - 0.625)	0.604 (0.579 - 0.633)	0.598 (0.574 - 0.625)	0.942 (0.815 - 1.066)	0.951 (0.822 - 1.080)	0.943 (0.819 - 1.066)	9.186 (6.099 - 12.835)	9.319 (6.211 - 13.039)	9.145 (6.091 - 12.852)
All ages (rate per capita)	0.511 (0.493 - 0.528)	0.515 (0.498 - 0.533)	0.519 (0.501 - 0.536)	0.909 (0.845 - 0.972)	0.911 (0.845 - 0.973)	0.919 (0.855 - 0.981)	2.985 (1.878 - 4.437)	3.406 (2.179 - 5.017)	3.606 (2.304 - 5.311)
Age-standardised (rate per capita)	0.507 (0.488 - 0.525)	0.509 (0.490 - 0.528)	0.509 (0.491 - 0.528)	0.903 (0.837 - 0.969)	0.906 (0.839 - 0.977)	0.906 (0.839 - 0.976)	2.637 (1.648 - 3.935)	2.636 (1.656 - 3.948)	2.642 (1.648 - 3.963)
Total number of cases (thousands)	1,354 (1,306 - 1,400)	1,186 (1,144 - 1,226)	1,149 (1,107 - 1,185)	2,409 (2,239 - 2,575)	2,096 (1,943 - 2,238)	2,033 (1,892 - 2,170)	8 (5 - 12)	8 (5 - 12)	8 (5 - 12)
**Lithuania**									
Under-5 years (rate per capita)	0.390 (0.327 - 0.450)	0.356 (0.313 - 0.403)	0.397 (0.346 - 0.458)	0.604 (0.386 - 0.800)	0.578 (0.438 - 0.733)	0.641 (0.480 - 0.801)	0.152 (0.064 - 0.302)	0.139 (0.060 - 0.270)	0.155 (0.066 - 0.301)
5-14 years (rate per capita)	0.637 (0.591 - 0.680)	0.641 (0.606 - 0.675)	0.613 (0.569 - 0.655)	1.115 (0.938 - 1.326)	1.061 (0.895 - 1.264)	1.066 (0.897 - 1.270)	0.839 (0.443 - 1.438)	0.851 (0.447 - 1.438)	0.813 (0.432 - 1.373)
15 - 49 years (rate per capita)	0.527 (0.505 - 0.551)	0.531 (0.511 - 0.553)	0.524 (0.507 - 0.545)	0.887 (0.813 - 0.956)	0.889 (0.813 - 0.956)	0.892 (0.821 - 0.965)	1.974 (1.157 - 3.167)	2.041 (1.197 - 3.277)	2.052 (1.217 - 3.263)
50 - 69 years (rate per capita)	0.586 (0.563 - 0.610)	0.590 (0.566 - 0.611)	0.583 (0.559 - 0.606)	1.002 (0.897 - 1.109)	1.002 (0.896 - 1.100)	0.999 (0.891 - 1.109)	5.846 (3.717 - 8.607)	5.968 (3.801 - 8.780)	5.715 (3.592 - 8.382)
70+ years (rate per capita)	0.629 (0.607 - 0.649)	0.639 (0.618 - 0.666)	0.630 (0.606 - 0.652)	0.970 (0.875 - 1.058)	0.985 (0.885 - 1.077)	0.973 (0.878 - 1.062)	9.771 (6.562 - 13.662)	9.985 (6.660 - 13.896)	9.798 (6.522 - 13.730)
All ages (rate per capita)	0.552 (0.537 - 0.567)	0.560 (0.547 - 0.573)	0.554 (0.541 - 0.568)	0.929 (0.872 - 0.987)	0.930 (0.878 - 0.982)	0.932 (0.881 - 0.982)	3.016 (1.891 - 4.506)	3.466 (2.189 - 5.145)	3.774 (2.398 - 5.590)
Age-standardised (rate per capita)	0.551 (0.534 - 0.565)	0.552 (0.539 - 0.565)	0.547 (0.532 - 0.562)	0.925 (0.867 - 0.986)	0.919 (0.862 - 0.975)	0.924 (0.869 - 0.980)	2.809 (1.756 - 4.216)	2.812 (1.749 - 4.210)	2.807 (1.752 - 4.200)
Total number of cases (thousands)	2,035 (1,977 - 2,087)	1,913 (1,870 - 1,957)	1,748 (1,705 - 1,790)	3,423 (3,213 - 3,637)	3,177 (3,000 - 3,354)	2,940 (2,777 - 3,095)	11 (7 - 17)	12 (7 - 18)	12 (8 - 18)
**Moldova**									
Under-5 years (rate per capita)	0.397 (0.330 - 0.471)	0.396 (0.328 - 0.468)	0.395 (0.329 - 0.467)	0.610 (0.387 - 0.798)	0.605 (0.384 - 0.792)	0.605 (0.384 - 0.792)	0.154 (0.064 - 0.316)	0.153 (0.063 - 0.314)	0.153 (0.063 - 0.315)
5-14 years (rate per capita)	0.582 (0.537 - 0.629)	0.556 (0.510 - 0.608)	0.581 (0.538 - 0.630)	1.074 (0.890 - 1.295)	1.027 (0.849 - 1.240)	1.071 (0.888 - 1.292)	0.761 (0.404 - 1.288)	0.760 (0.400 - 1.302)	0.761 (0.401 - 1.287)
15 - 49 years (rate per capita)	0.481 (0.453 - 0.511)	0.482 (0.456 - 0.512)	0.481 (0.453 - 0.512)	0.879 (0.778 - 0.981)	0.876 (0.780 - 0.971)	0.882 (0.781 - 0.983)	1.901 (1.118 - 3.004)	1.904 (1.116 - 3.012)	1.942 (1.148 - 3.087)
50 - 69 years (rate per capita)	0.553 (0.522 - 0.584)	0.550 (0.515 - 0.585)	0.549 (0.513 - 0.584)	0.972 (0.832 - 1.107)	0.968 (0.831 - 1.100)	0.970 (0.826 - 1.109)	5.762 (3.691 - 8.445)	5.569 (3.547 - 8.205)	5.556 (3.531 - 8.154)
70+ years (rate per capita)	0.614 (0.590 - 0.644)	0.616 (0.591 - 0.644)	0.609 (0.585 - 0.637)	0.956 (0.823 - 1.088)	0.958 (0.823 - 1.088)	0.948 (0.821 - 1.073)	9.976 (6.590 - 13.917)	10.019 (6.654 - 13.987)	9.863 (6.487 - 13.765)
All ages (rate per capita)	0.510 (0.491 - 0.530)	0.509 (0.490 - 0.528)	0.512 (0.492 - 0.532)	0.909 (0.839 - 0.978)	0.905 (0.838 - 0.970)	0.912 (0.845 - 0.976)	2.609 (1.624 - 3.902)	2.860 (1.776 - 4.267)	3.145 (1.970 - 4.647)
Age-standardised (rate per capita)	0.511 (0.492 - 0.529)	0.511 (0.491 - 0.530)	0.511 (0.492 - 0.531)	0.906 (0.837 - 0.974)	0.906 (0.837 - 0.974)	0.906 (0.837 - 0.974)	2.749 (1.722 - 4.085)	2.754 (1.720 - 4.097)	2.757 (1.722 - 4.084)
Total number of cases (thousands)	2,228 (2,144 - 2,312)	2,123 (2,041 - 2,200)	2,081 (1,999 - 2,162)	3,965 (3,662 - 4,269)	3,771 (3,492 - 4,042)	3,709 (3,436 - 3,970)	11 (7 - 17)	12 (7 - 18)	13 (8 - 19)
**Russia**									
Under-5 years (rate per capita)	0.345 (0.294 - 0.403)	0.346 (0.298 - 0.405)	0.350 (0.300 - 0.405)	0.550 (0.344 - 0.770)	0.589 (0.351 - 0.788)	0.589 (0.355 - 0.797)	0.133 (0.056 - 0.266)	0.135 (0.057 - 0.273)	0.136 (0.056 - 0.274)
5-14 years (rate per capita)	0.624 (0.577 - 0.668)	0.616 (0.578 - 0.660)	0.634 (0.587 - 0.677)	1.116 (0.942 - 1.328)	1.068 (0.906 - 1.259)	1.138 (0.953 - 1.357)	0.833 (0.438 - 1.427)	0.836 (0.442 - 1.406)	0.824 (0.431 - 1.381)
15 - 49 years (rate per capita)	0.543 (0.523 - 0.568)	0.545 (0.527 - 0.566)	0.542 (0.521 - 0.564)	0.891 (0.819 - 0.962)	0.891 (0.819 - 0.961)	0.897 (0.826 - 0.969)	2.035 (1.201 - 3.284)	2.086 (1.237 - 3.301)	2.113 (1.251 - 3.387)
50 - 69 years (rate per capita)	0.602 (0.579 - 0.627)	0.603 (0.577 - 0.627)	0.597 (0.573 - 0.621)	1.004 (0.900 - 1.109)	1.004 (0.904 - 1.102)	1.002 (0.889 - 1.109)	5.957 (3.844 - 8.670)	6.013 (3.872 - 8.797)	5.701 (3.673 - 8.336)
70+ years (rate per capita)	0.658 (0.640 - 0.681)	0.662 (0.645 - 0.683)	0.654 (0.635 - 0.675)	0.991 (0.889 - 1.082)	0.997 (0.894 - 1.085)	0.992 (0.897 - 1.076)	10.534 (7.015 - 14.590)	10.607 (7.052 - 14.706)	10.339 (6.860 - 14.393)
All ages (rate per capita)	0.561 (0.546 - 0.575)	0.565 (0.552 - 0.580)	0.565 (0.551 - 0.579)	0.931 (0.875 - 0.987)	0.928 (0.874 - 0.979)	0.938 (0.881 - 0.989)	3.113 (1.971 - 4.639)	3.442 (2.185 - 5.107)	3.716 (2.369 - 5.465)
Age-standardised (rate per capita)	0.556 (0.541 - 0.570)	0.559 (0.544 - 0.574)	0.556 (0.541 - 0.571)	0.921 (0.862 - 0.980)	0.926 (0.866 - 0.982)	0.925 (0.865 - 0.985)	2.909 (1.835 - 4.350)	2.905 (1.838 - 4.336)	2.907 (1.828 - 4.339)
Total number of cases (thousands)	82,731 (80,580 - 84,778)	80,876 (79,015 - 83,030)	83,751 (81,667 - 85,779)	137,299 (129,102 - 145,608)	132,793 (125,092 - 140,047)	138,955 (130,539 - 146,541)	459 (291 - 684)	493 (313 - 731)	550 (351 - 809)
**Ukraine**									
Under-5 years (rate per capita)	0.397 (0.330 - 0.471)	0.387 (0.321 - 0.459)	0.394 (0.329 - 0.466)	0.610 (0.387 - 0.799)	0.594 (0.377 - 0.778)	0.602 (0.382 - 0.789)	0.154 (0.063 - 0.312)	0.150 (0.062 - 0.308)	0.152 (0.063 - 0.308)
5-14 years (rate per capita)	0.579 (0.530 - 0.627)	0.558 (0.513 - 0.608)	0.587 (0.543 - 0.637)	1.068 (0.886 - 1.285)	1.030 (0.853 - 1.244)	1.082 (0.895 - 1.301)	0.761 (0.403 - 1.306)	0.760 (0.398 - 1.292)	0.761 (0.402 - 1.279)
15 - 49 years (rate per capita)	0.482 (0.455 - 0.513)	0.484 (0.459 - 0.514)	0.482 (0.455 - 0.510)	0.880 (0.781 - 0.981)	0.880 (0.784 - 0.977)	0.884 (0.785 - 0.985)	1.925 (1.126 - 3.059)	1.963 (1.160 - 3.098)	1.991 (1.179 - 3.161)
50 - 69 years (rate per capita)	0.552 (0.519 - 0.582)	0.556 (0.526 - 0.588)	0.546 (0.510 - 0.580)	0.971 (0.833 - 1.104)	0.973 (0.836 - 1.103)	0.970 (0.825 - 1.109)	5.673 (3.646 - 8.356)	5.859 (3.776 - 8.542)	5.449 (3.475 - 8.013)
70+ years (rate per capita)	0.609 (0.585 - 0.637)	0.614 (0.590 - 0.641)	0.606 (0.583 - 0.635)	0.948 (0.818 - 1.078)	0.954 (0.822 - 1.085)	0.950 (0.822 - 1.077)	9.843 (6.552 - 13.787)	9.900 (6.570 - 13.800)	9.643 (6.357 - 13.605)
All ages (rate per capita)	0.517 (0.498 - 0.536)	0.517 (0.499 - 0.537)	0.518 (0.499 - 0.536)	0.917 (0.849 - 0.982)	0.913 (0.847 - 0.975)	0.915 (0.850 - 0.979)	3.150 (1.984 - 4.655)	3.450 (2.192 - 5.080)	3.701 (2.348 - 5.458)
Age-standardised (rate per capita)	0.511 (0.490 - 0.530)	0.511 (0.492 - 0.530)	0.510 (0.491 - 0.529)	0.906 (0.837 - 0.974)	0.906 (0.837 - 0.974)	0.906 (0.837 - 0.974)	2.731 (1.709 - 4.082)	2.728 (1.714 - 4.068)	2.731 (1.711 - 4.078)
Total number of cases (thousands)	26,511 (25,532 - 27,514)	24,272 (23,425 - 25,208)	24,099 (23,199 - 24,934)	47,015 (43,525 - 50,376)	42,844 (39,763 - 45,760)	42,556 (39,526 - 45,520)	162 (102 - 239)	162 (103 - 239)	172 (109 - 254)
**Central Europe**									
Under-5 years (rate per capita)	0.476 (0.450 - 0.505)	0.475 (0.449 - 0.501)	0.473 (0.445 - 0.501)	0.663 (0.485 - 0.820)	0.666 (0.492 - 0.812)	0.662 (0.483 - 0.817)	0.168 (0.073 - 0.333)	0.167 (0.073 - 0.329)	0.168 (0.073 - 0.332)
5-14 years (rate per capita)	0.648 (0.623 - 0.676)	0.632 (0.602 - 0.663)	0.640 (0.612 - 0.669)	1.090 (0.913 - 1.304)	1.058 (0.874 - 1.277)	1.078 (0.893 - 1.303)	0.805 (0.425 - 1.359)	0.790 (0.415 - 1.340)	0.791 (0.418 - 1.344)
15 - 49 years (rate per capita)	0.557 (0.538 - 0.575)	0.556 (0.539 - 0.573)	0.554 (0.536 - 0.575)	0.818 (0.729 - 0.900)	0.820 (0.732 - 0.905)	0.825 (0.737 - 0.912)	2.097 (1.226 - 3.353)	2.133 (1.242 - 3.398)	2.185 (1.275 - 3.482)
50 - 69 years (rate per capita)	0.633 (0.614 - 0.653)	0.627 (0.606 - 0.648)	0.631 (0.608 - 0.652)	0.923 (0.799 - 1.042)	0.918 (0.791 - 1.047)	0.919 (0.788 - 1.049)	6.475 (4.023 - 9.497)	6.276 (3.900 - 9.260)	6.438 (4.005 - 9.427)
70+ years (rate per capita)	0.671 (0.655 - 0.687)	0.674 (0.658 - 0.691)	0.665 (0.649 - 0.680)	0.903 (0.789 - 1.014)	0.905 (0.789 - 1.018)	0.897 (0.785 - 1.003)	10.343 (6.858 - 14.665)	10.417 (6.864 - 14.803)	10.225 (6.748 - 14.482)
All ages (rate per capita)	0.588 (0.577 - 0.601)	0.589 (0.577 - 0.601)	0.591 (0.578 - 0.604)	0.878 (0.815 - 0.940)	0.871 (0.807 - 0.929)	0.873 (0.811 - 0.935)	3.162 (1.937 - 4.716)	3.630 (2.238 - 5.364)	3.971 (2.468 - 5.854)
Age-standardised (rate per capita)	0.585 (0.573 - 0.597)	0.583 (0.571 - 0.595)	0.582 (0.569 - 0.594)	0.874 (0.812 - 0.939)	0.872 (0.809 - 0.937)	0.871 (0.806 - 0.937)	2.981 (1.827 - 4.463)	2.978 (1.818 - 4.449)	2.978 (1.821 - 4.461)
Total number of cases (thousands)	72,428 (71,030 - 73,946)	69,579 (68,175 - 70,999)	68,922 (67,443 - 70,461)	108,062 (100,366 - 115,804)	102,939 (95,463 - 109,868)	101,918 (94,632 - 109,106)	389 (239 - 581)	429 (265 - 634)	463 (288 - 683)
**Albania**									
Under-5 years (rate per capita)	0.469 (0.400 - 0.524)	0.483 (0.415 - 0.539)	0.463 (0.396 - 0.518)	0.633 (0.459 - 0.784)	0.652 (0.474 - 0.808)	0.627 (0.455 - 0.776)	0.111 (0.046 - 0.216)	0.115 (0.048 - 0.224)	0.110 (0.047 - 0.215)
5-14 years (rate per capita)	0.629 (0.572 - 0.681)	0.615 (0.554 - 0.671)	0.612 (0.540 - 0.672)	1.058 (0.848 - 1.311)	1.031 (0.827 - 1.276)	1.024 (0.820 - 1.268)	0.616 (0.341 - 1.035)	0.618 (0.338 - 1.033)	0.618 (0.341 - 1.034)
15 - 49 years (rate per capita)	0.540 (0.494 - 0.587)	0.547 (0.504 - 0.590)	0.544 (0.500 - 0.586)	0.775 (0.662 - 0.884)	0.777 (0.668 - 0.881)	0.776 (0.665 - 0.883)	1.771 (1.050 - 2.779)	1.901 (1.124 - 2.997)	1.862 (1.110 - 2.938)
50 - 69 years (rate per capita)	0.626 (0.580 - 0.675)	0.627 (0.581 - 0.675)	0.627 (0.580 - 0.677)	0.864 (0.693 - 1.036)	0.865 (0.698 - 1.032)	0.865 (0.697 - 1.036)	6.127 (3.817 - 8.995)	6.233 (3.881 - 9.142)	6.246 (3.884 - 9.169)
70+ years (rate per capita)	0.676 (0.634 - 0.714)	0.677 (0.633 - 0.717)	0.672 (0.632 - 0.709)	0.858 (0.696 - 1.012)	0.859 (0.697 - 1.013)	0.856 (0.698 - 1.007)	10.381 (6.764 - 14.652)	10.396 (6.735 - 14.751)	10.327 (6.723 - 14.603)
All ages (rate per capita)	0.566 (0.537 - 0.596)	0.576 (0.549 - 0.606)	0.577 (0.550 - 0.607)	0.831 (0.755 - 0.916)	0.836 (0.759 - 0.921)	0.824 (0.749 - 0.904)	2.164 (1.323 - 3.244)	2.716 (1.673 - 4.051)	3.318 (2.058 - 4.876)
Age-standardised (rate per capita)	0.575 (0.549 - 0.602)	0.575 (0.548 - 0.603)	0.574 (0.549 - 0.601)	0.834 (0.758 - 0.914)	0.833 (0.758 - 0.913)	0.834 (0.758 - 0.914)	2.842 (1.761 - 4.202)	2.845 (1.754 - 4.215)	2.851 (1.755 - 4.205)
Total number of cases (thousands)	1,854 (1,760 - 1,953)	1,779 (1,694 - 1,869)	1,672 (1,594 - 1,759)	2,725 (2,474 - 3,004)	2,582 (2,342 - 2,844)	2,387 (2,169 - 2,619)	7 (4 - 11)	8 (5 - 13)	10 (6 - 14)
**Bosnia and Herzegovina**									
Under-5 years (rate per capita)	0.475 (0.413 - 0.531)	0.469 (0.407 - 0.526)	0.469 (0.409 - 0.525)	0.651 (0.474 - 0.804)	0.645 (0.470 - 0.796)	0.643 (0.468 - 0.793)	0.113 (0.048 - 0.222)	0.112 (0.048 - 0.220)	0.111 (0.047 - 0.221)
5-14 years (rate per capita)	0.694 (0.648 - 0.732)	0.690 (0.640 - 0.732)	0.692 (0.644 - 0.731)	1.042 (0.877 - 1.240)	1.028 (0.866 - 1.221)	1.041 (0.876 - 1.238)	0.668 (0.363 - 1.104)	0.668 (0.360 - 1.105)	0.670 (0.362 - 1.101)
15 - 49 years (rate per capita)	0.620 (0.600 - 0.643)	0.623 (0.603 - 0.643)	0.622 (0.601 - 0.644)	0.759 (0.703 - 0.818)	0.766 (0.710 - 0.825)	0.770 (0.714 - 0.830)	1.987 (1.168 - 3.123)	2.141 (1.272 - 3.317)	2.172 (1.284 - 3.391)
50 - 69 years (rate per capita)	0.675 (0.653 - 0.700)	0.678 (0.655 - 0.701)	0.679 (0.656 - 0.702)	0.892 (0.804 - 0.978)	0.892 (0.807 - 0.974)	0.893 (0.806 - 0.976)	6.626 (4.218 - 9.726)	6.774 (4.330 - 10.009)	6.790 (4.328 - 9.985)

	Prevalence 1990	Prevalence 2005	Prevalence 2015	Incidence 1990	Incidence 2005	Incidence 2015	YLD rate 1990	YLD rate 2005	YLD rate 2015
70+ years (rate per capita)	0.720 (0.701 - 0.736)	0.732 (0.713 - 0.750)	0.716 (0.699 - 0.732)	0.877 (0.796 - 0.947)	0.882 (0.792 - 0.958)	0.877 (0.796 - 0.946)	11.540 (7.699 - 16.129)	11.670 (7.802 - 16.309)	11.231 (7.506 - 15.647)
All ages (rate per capita)	0.635 (0.619 - 0.651)	0.645 (0.632 - 0.660)	0.647 (0.633 - 0.662)	0.826 (0.775 - 0.877)	0.829 (0.782 - 0.874)	0.834 (0.787 - 0.877)	2.832 (1.750 - 4.236)	3.706 (2.352 - 5.475)	4.140 (2.612 - 6.072)
Age-standardised (rate per capita)	0.636 (0.620 - 0.651)	0.636 (0.621 - 0.651)	0.636 (0.620 - 0.651)	0.827 (0.778 - 0.879)	0.827 (0.778 - 0.879)	0.827 (0.778 - 0.879)	3.107 (1.937 - 4.617)	3.091 (1.942 - 4.574)	3.062 (1.902 - 4.541)
Total number of cases (thousands)	2,871 (2,798 - 2,943)	2,477 (2,426 - 2,535)	2,464 (2,412 - 2,521)	3,734 (3,505 - 3,964)	3,185 (3,005 - 3,357)	3,178 (3,000 - 3,340)	13 (8 - 19)	14 (9 - 21)	16 (10 - 23)
**Bulgaria**									
Under-5 years (rate per capita)	0.469 (0.412 - 0.524)	0.458 (0.399 - 0.513)	0.466 (0.405 - 0.520)	0.666 (0.496 - 0.814)	0.657 (0.474 - 0.812)	0.669 (0.483 - 0.827)	0.181 (0.078 - 0.363)	0.177 (0.075 - 0.350)	0.180 (0.077 - 0.365)
5-14 years (rate per capita)	0.681 (0.643 - 0.717)	0.669 (0.625 - 0.709)	0.680 (0.638 - 0.720)	1.178 (1.024 - 1.380)	1.149 (0.979 - 1.367)	1.191 (1.012 - 1.419)	0.918 (0.475 - 1.572)	0.910 (0.467 - 1.556)	0.902 (0.466 - 1.549)
15 - 49 years (rate per capita)	0.604 (0.586 - 0.623)	0.603 (0.584 - 0.622)	0.602 (0.584 - 0.620)	0.939 (0.881 - 0.997)	0.941 (0.884 - 1.001)	0.948 (0.889 - 1.006)	2.166 (1.271 - 3.470)	2.189 (1.282 - 3.529)	2.262 (1.325 - 3.678)
50 - 69 years (rate per capita)	0.652 (0.631 - 0.674)	0.647 (0.625 - 0.670)	0.651 (0.629 - 0.673)	1.070 (0.992 - 1.143)	1.069 (0.991 - 1.144)	1.070 (0.992 - 1.144)	6.519 (4.070 - 9.493)	6.351 (3.955 - 9.296)	6.567 (4.116 - 9.593)
70+ years (rate per capita)	0.691 (0.671 - 0.711)	0.693 (0.671 - 0.714)	0.685 (0.664 - 0.704)	1.044 (0.979 - 1.103)	1.045 (0.981 - 1.109)	1.040 (0.980 - 1.099)	10.361 (6.794 - 14.766)	10.402 (6.801 - 14.771)	10.275 (6.692 - 14.545)
All ages (rate per capita)	0.624 (0.610 - 0.637)	0.625 (0.612 - 0.640)	0.627 (0.613 - 0.640)	0.994 (0.947 - 1.042)	0.994 (0.951 - 1.036)	1.002 (0.960 - 1.045)	3.537 (2.174 - 5.209)	4.041 (2.509 - 5.900)	4.333 (2.707 - 6.291)
Age-standardised (rate per capita)	0.618 (0.603 - 0.631)	0.616 (0.603 - 0.631)	0.616 (0.602 - 0.630)	0.985 (0.935 - 1.038)	0.983 (0.932 - 1.036)	0.983 (0.932 - 1.037)	2.999 (1.832 - 4.482)	3.003 (1.843 - 4.489)	3.003 (1.834 - 4.477)
Total number of cases (thousands)	5,508 (5,385 - 5,621)	4,804 (4,705 - 4,915)	4,556 (4,457 - 4,654)	8,777 (8,361 - 9,194)	7,636 (7,306 - 7,961)	7,283 (6,974 - 7,593)	31 (19 - 46)	31 (19 - 45)	31 (20 - 46)
**Croatia**									
Under-5 years (rate per capita)	0.476 (0.421 - 0.529)	0.472 (0.416 - 0.522)	0.474 (0.417 - 0.524)	0.679 (0.488 - 0.837)	0.665 (0.481 - 0.822)	0.667 (0.482 - 0.824)	0.185 (0.079 - 0.365)	0.183 (0.078 - 0.355)	0.183 (0.078 - 0.375)
5-14 years (rate per capita)	0.648 (0.598 - 0.694)	0.647 (0.599 - 0.684)	0.650 (0.603 - 0.686)	0.970 (0.799 - 1.182)	0.926 (0.769 - 1.136)	0.930 (0.772 - 1.141)	0.851 (0.449 - 1.445)	0.820 (0.428 - 1.400)	0.819 (0.428 - 1.390)
15 - 49 years (rate per capita)	0.560 (0.540 - 0.582)	0.562 (0.542 - 0.585)	0.560 (0.540 - 0.584)	0.647 (0.587 - 0.709)	0.649 (0.589 - 0.708)	0.649 (0.589 - 0.710)	2.359 (1.348 - 3.865)	2.440 (1.396 - 3.990)	2.427 (1.391 - 3.946)
50 - 69 years (rate per capita)	0.644 (0.619 - 0.669)	0.644 (0.620 - 0.671)	0.641 (0.616 - 0.667)	0.734 (0.651 - 0.824)	0.732 (0.648 - 0.820)	0.734 (0.648 - 0.828)	6.821 (4.243 - 10.247)	6.850 (4.270 - 10.269)	6.885 (4.321 - 10.293)
70+ years (rate per capita)	0.674 (0.656 - 0.694)	0.679 (0.661 - 0.700)	0.664 (0.646 - 0.683)	0.696 (0.618 - 0.773)	0.701 (0.619 - 0.790)	0.692 (0.618 - 0.773)	10.594 (7.030 - 15.146)	10.721 (7.090 - 15.270)	10.417 (6.895 - 14.868)
All ages (rate per capita)	0.595 (0.580 - 0.610)	0.601 (0.586 - 0.614)	0.601 (0.587 - 0.615)	0.717 (0.669 - 0.767)	0.706 (0.662 - 0.752)	0.707 (0.665 - 0.753)	3.660 (2.226 - 5.545)	4.174 (2.572 - 6.284)	4.421 (2.745 - 6.646)
Age-standardised (rate per capita)	0.589 (0.573 - 0.604)	0.589 (0.573 - 0.603)	0.587 (0.573 - 0.602)	0.722 (0.670 - 0.778)	0.713 (0.666 - 0.767)	0.713 (0.665 - 0.767)	3.195 (1.943 - 4.864)	3.188 (1.931 - 4.869)	3.162 (1.921 - 4.831)
Total number of cases (thousands)	2,839 (2,771 - 2,912)	2,633 (2,568 - 2,694)	2,547 (2,491 - 2,609)	3,423 (3,195 - 3,662)	3,097 (2,905 - 3,299)	2,998 (2,818 - 3,195)	17 (11 - 26)	18 (11 - 28)	19 (12 - 28)
**Czech Republic**									
Under-5 years (rate per capita)	0.469 (0.405 - 0.528)	0.458 (0.397 - 0.511)	0.469 (0.404 - 0.523)	0.662 (0.478 - 0.825)	0.647 (0.467 - 0.806)	0.662 (0.478 - 0.824)	0.182 (0.079 - 0.365)	0.178 (0.077 - 0.365)	0.182 (0.077 - 0.366)
5-14 years (rate per capita)	0.606 (0.538 - 0.665)	0.604 (0.550 - 0.663)	0.628 (0.571 - 0.682)	1.072 (0.857 - 1.320)	1.067 (0.853 - 1.316)	1.120 (0.902 - 1.380)	0.818 (0.426 - 1.401)	0.819 (0.434 - 1.404)	0.814 (0.429 - 1.397)
15 - 49 years (rate per capita)	0.541 (0.501 - 0.585)	0.539 (0.498 - 0.581)	0.542 (0.499 - 0.586)	0.833 (0.718 - 0.942)	0.834 (0.719 - 0.947)	0.839 (0.725 - 0.957)	2.207 (1.277 - 3.499)	2.198 (1.270 - 3.488)	2.323 (1.352 - 3.678)
50 - 69 years (rate per capita)	0.631 (0.588 - 0.679)	0.623 (0.577 - 0.669)	0.633 (0.591 - 0.682)	0.917 (0.749 - 1.089)	0.915 (0.740 - 1.087)	0.919 (0.750 - 1.100)	6.813 (4.368 - 9.984)	6.387 (4.042 - 9.479)	6.912 (4.418 - 10.086)
70+ years (rate per capita)	0.669 (0.637 - 0.706)	0.670 (0.636 - 0.705)	0.661 (0.627 - 0.695)	0.898 (0.735 - 1.048)	0.900 (0.735 - 1.052)	0.895 (0.741 - 1.040)	10.624 (7.073 - 14.936)	10.623 (7.060 - 14.930)	10.387 (6.861 - 14.596)
All ages (rate per capita)	0.574 (0.548 - 0.604)	0.576 (0.549 - 0.606)	0.584 (0.558 - 0.613)	0.880 (0.802 - 0.961)	0.876 (0.794 - 0.957)	0.883 (0.800 - 0.967)	3.448 (2.136 - 5.189)	3.849 (2.403 - 5.773)	4.173 (2.620 - 6.187)
Age-standardised (rate per capita)	0.569 (0.543 - 0.598)	0.569 (0.542 - 0.597)	0.570 (0.543 - 0.597)	0.880 (0.800 - 0.963)	0.880 (0.800 - 0.963)	0.880 (0.800 - 0.964)	3.057 (1.888 - 4.616)	3.054 (1.880 - 4.633)	3.054 (1.874 - 4.630)
Total number of cases (thousands)	5,916 (5,646 - 6,223)	5,917 (5,648 - 6,232)	6,242 (5,971 - 6,555)	9,066 (8,255 - 9,899)	9,002 (8,164 - 9,838)	9,444 (8,555 - 10,348)	36 (22 - 53)	40 (25 - 59)	45 (28 - 66)
**Hungary**									
Under-5 years (rate per capita)	0.449 (0.389 - 0.506)	0.450 (0.392 - 0.508)	0.452 (0.389 - 0.509)	0.652 (0.460 - 0.817)	0.656 (0.460 - 0.824)	0.657 (0.464 - 0.822)	0.174 (0.075 - 0.341)	0.174 (0.074 - 0.347)	0.175 (0.074 - 0.354)
5-14 years (rate per capita)	0.628 (0.587 - 0.667)	0.644 (0.600 - 0.684)	0.649 (0.599 - 0.692)	1.023 (0.847 - 1.227)	1.049 (0.861 - 1.268)	1.075 (0.890 - 1.307)	0.839 (0.442 - 1.439)	0.845 (0.439 - 1.462)	0.839 (0.436 - 1.422)
15 - 49 years (rate per capita)	0.545 (0.522 - 0.570)	0.541 (0.520 - 0.565)	0.541 (0.520 - 0.565)	0.825 (0.752 - 0.897)	0.826 (0.755 - 0.895)	0.827 (0.756 - 0.899)	2.025 (1.184 - 3.250)	2.011 (1.187 - 3.238)	2.046 (1.196 - 3.296)
50 - 69 years (rate per capita)	0.595 (0.570 - 0.619)	0.588 (0.562 - 0.613)	0.594 (0.569 - 0.619)	0.942 (0.840 - 1.045)	0.940 (0.839 - 1.038)	0.942 (0.843 - 1.047)	5.988 (3.882 - 8.777)	5.729 (3.685 - 8.414)	5.918 (3.838 - 8.647)
70+ years (rate per capita)	0.644 (0.624 - 0.666)	0.643 (0.622 - 0.665)	0.636 (0.616 - 0.657)	0.913 (0.820 - 1.006)	0.915 (0.818 - 1.011)	0.907 (0.819 - 0.996)	9.699 (6.472 - 13.483)	9.659 (6.398 - 13.385)	9.542 (6.350 - 13.321)
All ages (rate per capita)	0.570 (0.556 - 0.586)	0.571 (0.556 - 0.585)	0.572 (0.558 - 0.586)	0.876 (0.823 - 0.932)	0.880 (0.827 - 0.932)	0.882 (0.828 - 0.931)	3.260 (2.058 - 4.838)	3.554 (2.263 - 5.252)	3.768 (2.395 - 5.528)
Age-standardised (rate per capita)	0.566 (0.550 - 0.583)	0.568 (0.553 - 0.583)	0.567 (0.551 - 0.582)	0.873 (0.815 - 0.931)	0.875 (0.820 - 0.932)	0.874 (0.817 - 0.932)	2.784 (1.742 - 4.173)	2.774 (1.738 - 4.155)	2.788 (1.730 - 4.175)
Total number of cases (thousands)	5,921 (5,772 - 6,087)	5,752 (5,610 - 5,901)	5,814 (5,669 - 5,955)	9,098 (8,551 - 9,677)	8,870 (8,337 - 9,392)	8,963 (8,421 - 9,464)	34 (21 - 50)	36 (23 - 53)	38 (24 - 56)
**Macedonia**									
Under-5 years (rate per capita)	0.473 (0.406 - 0.528)	0.475 (0.409 - 0.530)	0.470 (0.404 - 0.522)	0.639 (0.464 - 0.792)	0.641 (0.465 - 0.794)	0.633 (0.459 - 0.784)	0.113 (0.047 - 0.221)	0.113 (0.048 - 0.221)	0.111 (0.047 - 0.217)
5-14 years (rate per capita)	0.627 (0.565 - 0.682)	0.621 (0.561 - 0.678)	0.619 (0.560 - 0.676)	1.056 (0.847 - 1.308)	1.039 (0.834 - 1.288)	1.039 (0.834 - 1.287)	0.617 (0.338 - 1.018)	0.618 (0.341 - 1.015)	0.618 (0.340 - 1.039)
15 - 49 years (rate per capita)	0.544 (0.503 - 0.588)	0.546 (0.507 - 0.588)	0.546 (0.505 - 0.594)	0.779 (0.666 - 0.887)	0.782 (0.671 - 0.889)	0.785 (0.678 - 0.893)	1.878 (1.115 - 2.959)	1.963 (1.168 - 3.098)	2.009 (1.191 - 3.149)
50 - 69 years (rate per capita)	0.628 (0.580 - 0.677)	0.629 (0.580 - 0.679)	0.629 (0.583 - 0.681)	0.865 (0.697 - 1.035)	0.864 (0.698 - 1.029)	0.866 (0.698 - 1.035)	6.216 (3.883 - 9.197)	6.173 (3.860 - 9.070)	6.285 (3.942 - 9.234)
70+ years (rate per capita)	0.673 (0.636 - 0.710)	0.679 (0.636 - 0.719)	0.673 (0.634 - 0.711)	0.857 (0.698 - 1.011)	0.860 (0.697 - 1.019)	0.856 (0.699 - 1.007)	10.303 (6.721 - 14.648)	10.414 (6.785 - 14.760)	10.284 (6.718 - 14.568)
All ages (rate per capita)	0.572 (0.545 - 0.600)	0.578 (0.550 - 0.607)	0.580 (0.553 - 0.613)	0.833 (0.757 - 0.916)	0.832 (0.757 - 0.912)	0.831 (0.756 - 0.909)	2.593 (1.592 - 3.856)	3.063 (1.897 - 4.511)	3.428 (2.128 - 5.045)
Age-standardised (rate per capita)	0.575 (0.548 - 0.601)	0.575 (0.548 - 0.603)	0.574 (0.548 - 0.606)	0.834 (0.758 - 0.914)	0.834 (0.758 - 0.914)	0.834 (0.758 - 0.914)	2.836 (1.748 - 4.182)	2.840 (1.758 - 4.176)	2.838 (1.750 - 4.185)
Total number of cases (thousands)	1,145 (1,091 - 1,201)	1,180 (1,124 - 1,240)	1,205 (1,148 - 1,273)	1,668 (1,516 - 1,834)	1,698 (1,546 - 1,863)	1,726 (1,570 - 1,889)	5 (3 - 8)	6 (4 - 9)	7 (4 - 10)
**Montenegro**									
Under-5 years (rate per capita)	0.474 (0.409 - 0.529)	0.471 (0.408 - 0.526)	0.474 (0.412 - 0.529)	0.639 (0.464 - 0.791)	0.636 (0.461 - 0.787)	0.640 (0.464 - 0.792)	0.112 (0.048 - 0.216)	0.112 (0.048 - 0.218)	0.113 (0.048 - 0.219)
5-14 years (rate per capita)	0.627 (0.572 - 0.685)	0.624 (0.564 - 0.680)	0.624 (0.563 - 0.679)	1.057 (0.848 - 1.308)	1.044 (0.838 - 1.293)	1.050 (0.843 - 1.298)	0.616 (0.343 - 1.018)	0.617 (0.342 - 1.018)	0.617 (0.339 - 1.031)
15 - 49 years (rate per capita)	0.543 (0.500 - 0.589)	0.546 (0.503 - 0.587)	0.546 (0.504 - 0.586)	0.779 (0.668 - 0.886)	0.782 (0.669 - 0.890)	0.784 (0.676 - 0.892)	1.860 (1.103 - 2.922)	1.959 (1.156 - 3.070)	1.983 (1.177 - 3.126)
50 - 69 years (rate per capita)	0.626 (0.575 - 0.677)	0.630 (0.578 - 0.684)	0.630 (0.585 - 0.682)	0.865 (0.695 - 1.035)	0.864 (0.699 - 1.028)	0.866 (0.699 - 1.036)	6.133 (3.831 - 9.004)	6.226 (3.889 - 9.166)	6.336 (3.965 - 9.334)
70+ years (rate per capita)	0.664 (0.624 - 0.700)	0.679 (0.635 - 0.720)	0.671 (0.630 - 0.708)	0.850 (0.700 - 0.990)	0.859 (0.696 - 1.017)	0.854 (0.697 - 1.002)	10.221 (6.738 - 14.468)	10.449 (6.830 - 14.830)	10.309 (6.708 - 14.567)
All ages (rate per capita)	0.573 (0.545 - 0.603)	0.581 (0.554 - 0.611)	0.584 (0.559 - 0.613)	0.834 (0.759 - 0.916)	0.832 (0.757 - 0.912)	0.836 (0.760 - 0.915)	2.728 (1.681 - 4.042)	3.282 (2.037 - 4.855)	3.526 (2.198 - 5.203)
Age-standardised (rate per capita)	0.575 (0.548 - 0.604)	0.576 (0.550 - 0.604)	0.575 (0.549 - 0.603)	0.834 (0.758 - 0.914)	0.834 (0.758 - 0.914)	0.834 (0.758 - 0.914)	2.849 (1.759 - 4.221)	2.847 (1.756 - 4.220)	2.847 (1.763 - 4.226)
Total number of cases (thousands)	353 (335 - 371)	358 (342 - 377)	365 (350 - 384)	513 (467 - 564)	513 (467 - 562)	523 (476 - 573)	2 (1 - 2)	2 (1 - 3)	2 (1 - 3)
**Poland**									
Under-5 years (rate per capita)	0.495 (0.445 - 0.539)	0.500 (0.457 - 0.537)	0.493 (0.443 - 0.535)	0.685 (0.510 - 0.842)	0.693 (0.533 - 0.823)	0.679 (0.505 - 0.835)	0.192 (0.084 - 0.384)	0.194 (0.084 - 0.379)	0.191 (0.083 - 0.375)
5-14 years (rate per capita)	0.629 (0.575 - 0.684)	0.616 (0.559 - 0.672)	0.627 (0.573 - 0.682)	1.103 (0.894 - 1.350)	1.073 (0.866 - 1.301)	1.097 (0.888 - 1.344)	0.818 (0.429 - 1.391)	0.822 (0.429 - 1.407)	0.819 (0.427 - 1.403)
15 - 49 years (rate per capita)	0.537 (0.493 - 0.581)	0.540 (0.496 - 0.581)	0.536 (0.491 - 0.580)	0.832 (0.715 - 0.943)	0.831 (0.713 - 0.942)	0.834 (0.718 - 0.948)	2.137 (1.239 - 3.430)	2.160 (1.261 - 3.453)	2.152 (1.249 - 3.457)
50 - 69 years (rate per capita)	0.629 (0.582 - 0.683)	0.621 (0.573 - 0.672)	0.622 (0.576 - 0.674)	0.916 (0.743 - 1.088)	0.912 (0.736 - 1.084)	0.915 (0.733 - 1.094)	6.555 (4.072 - 9.564)	6.246 (3.916 - 9.197)	6.275 (3.916 - 9.259)
70+ years (rate per capita)	0.665 (0.625 - 0.700)	0.668 (0.627 - 0.708)	0.658 (0.619 - 0.695)	0.898 (0.735 - 1.047)	0.901 (0.736 - 1.057)	0.894 (0.741 - 1.037)	10.397 (6.816 - 14.717)	10.468 (6.804 - 14.875)	10.257 (6.724 - 14.524)
All ages (rate per capita)	0.575 (0.549 - 0.604)	0.577 (0.550 - 0.607)	0.578 (0.551 - 0.607)	0.887 (0.807 - 0.968)	0.877 (0.795 - 0.957)	0.876 (0.793 - 0.958)	3.104 (1.906 - 4.653)	3.567 (2.203 - 5.279)	3.926 (2.437 - 5.777)
Age-standardised (rate per capita)	0.572 (0.547 - 0.602)	0.574 (0.549 - 0.602)	0.572 (0.546 - 0.600)	0.881 (0.802 - 0.961)	0.883 (0.804 - 0.962)	0.881 (0.802 - 0.961)	3.022 (1.853 - 4.531)	3.023 (1.853 - 4.522)	3.023 (1.850 - 4.556)
Total number of cases (thousands)	21,975 (20,971 - 23,098)	22,021 (20,993 - 23,172)	22,489 (21,433 - 23,639)	33,911 (30,846 - 37,001)	33,498 (30,371 - 36,558)	34,096 (30,840 - 37,281)	119 (73 - 178)	136 (84 - 202)	153 (95 - 225)
**Romania**									
Under-5 years (rate per capita)	0.459 (0.396 - 0.516)	0.446 (0.383 - 0.506)	0.452 (0.391 - 0.512)	0.653 (0.459 - 0.828)	0.655 (0.456 - 0.818)	0.662 (0.461 - 0.827)	0.178 (0.075 - 0.357)	0.173 (0.074 - 0.346)	0.175 (0.074 - 0.344)
5-14 years (rate per capita)	0.714 (0.680 - 0.745)	0.656 (0.610 - 0.703)	0.660 (0.614 - 0.702)	1.152 (0.984 - 1.343)	1.066 (0.890 - 1.270)	1.079 (0.904 - 1.287)	0.927 (0.485 - 1.597)	0.873 (0.460 - 1.477)	0.870 (0.458 - 1.470)
15 - 49 years (rate per capita)	0.583 (0.563 - 0.606)	0.581 (0.561 - 0.604)	0.583 (0.564 - 0.605)	0.824 (0.759 - 0.893)	0.831 (0.765 - 0.894)	0.842 (0.774 - 0.907)	2.139 (1.239 - 3.470)	2.197 (1.269 - 3.565)	2.382 (1.389 - 3.841)
50 - 69 years (rate per capita)	0.643 (0.617 - 0.668)	0.642 (0.619 - 0.667)	0.647 (0.621 - 0.671)	0.955 (0.852 - 1.050)	0.953 (0.855 - 1.050)	0.957 (0.856 - 1.053)	6.518 (4.076 - 9.605)	6.511 (4.020 - 9.557)	6.750 (4.196 - 9.914)
70+ years (rate per capita)	0.685 (0.662 - 0.709)	0.688 (0.664 - 0.709)	0.679 (0.656 - 0.700)	0.933 (0.840 - 1.020)	0.937 (0.845 - 1.029)	0.929 (0.841 - 1.014)	10.569 (6.873 - 14.988)	10.609 (6.899 - 15.039)	10.428 (6.793 - 14.796)

	Prevalence 1990	Prevalence 2005	Prevalence 2015	Incidence 1990	Incidence 2005	Incidence 2015	YLD rate 1990	YLD rate 2005	YLD rate 2015
All ages (rate per capita)	0.614 (0.599 - 0.629)	0.607 (0.593 - 0.623)	0.612 (0.598 - 0.627)	0.900 (0.847 - 0.955)	0.886 (0.833 - 0.936)	0.898 (0.845 - 0.946)	3.267 (1.989 - 4.895)	3.776 (2.329 - 5.626)	4.169 (2.571 - 6.145)
Age-standardised (rate per capita)	0.610 (0.595 - 0.625)	0.600 (0.585 - 0.616)	0.600 (0.585 - 0.616)	0.897 (0.844 - 0.955)	0.885 (0.826 - 0.943)	0.885 (0.826 - 0.943)	3.073 (1.871 - 4.607)	3.068 (1.872 - 4.620)	3.069 (1.867 - 4.618)
Total number of cases (thousands)	14,387 (14,020 - 14,729)	12,999 (12,698 - 13,337)	11,958 (11,687 - 12,248)	21,073 (19,837 - 22,356)	18,978 (17,837 - 20,057)	17,536 (16,503 - 18,475)	77 (47 - 115)	81 (50 - 121)	81 (50 - 120)
**Serbia**									
Under-5 years (rate per capita)	0.495 (0.440 - 0.542)	0.515 (0.468 - 0.558)	0.491 (0.434 - 0.536)	0.652 (0.487 - 0.794)	0.677 (0.521 - 0.802)	0.649 (0.484 - 0.790)	0.118 (0.051 - 0.227)	0.123 (0.053 - 0.235)	0.117 (0.050 - 0.227)
5-14 years (rate per capita)	0.631 (0.571 - 0.688)	0.636 (0.577 - 0.686)	0.627 (0.570 - 0.686)	1.042 (0.843 - 1.283)	1.039 (0.835 - 1.280)	1.031 (0.830 - 1.271)	0.619 (0.342 - 1.033)	0.621 (0.343 - 1.019)	0.621 (0.344 - 1.027)
15 - 49 years (rate per capita)	0.546 (0.504 - 0.587)	0.547 (0.506 - 0.588)	0.547 (0.504 - 0.588)	0.782 (0.672 - 0.889)	0.782 (0.673 - 0.889)	0.785 (0.675 - 0.893)	1.935 (1.152 - 3.041)	1.956 (1.154 - 3.080)	2.020 (1.199 - 3.176)
50 - 69 years (rate per capita)	0.631 (0.584 - 0.682)	0.629 (0.579 - 0.678)	0.636 (0.590 - 0.685)	0.867 (0.699 - 1.035)	0.865 (0.698 - 1.030)	0.868 (0.702 - 1.036)	6.348 (3.970 - 9.303)	6.173 (3.866 - 9.031)	6.572 (4.127 - 9.593)
70+ years (rate per capita)	0.670 (0.636 - 0.706)	0.680 (0.636 - 0.721)	0.667 (0.629 - 0.702)	0.853 (0.699 - 0.998)	0.859 (0.698 - 1.018)	0.852 (0.699 - 0.995)	10.229 (6.684 - 14.462)	10.412 (6.794 - 14.716)	10.170 (6.631 - 14.417)
All ages (rate per capita)	0.581 (0.554 - 0.609)	0.589 (0.563 - 0.618)	0.590 (0.564 - 0.618)	0.836 (0.762 - 0.915)	0.836 (0.760 - 0.913)	0.835 (0.759 - 0.916)	2.993 (1.855 - 4.415)	3.502 (2.184 - 5.117)	3.842 (2.413 - 5.612)
Age-standardised (rate per capita)	0.578 (0.552 - 0.606)	0.581 (0.555 - 0.610)	0.578 (0.552 - 0.606)	0.833 (0.760 - 0.912)	0.835 (0.761 - 0.911)	0.834 (0.760 - 0.912)	2.841 (1.757 - 4.183)	2.839 (1.757 - 4.176)	2.837 (1.753 - 4.178)
Total number of cases (thousands)	5,524 (5,266 - 5,794)	5,425 (5,183 - 5,690)	5,221 (4,995 - 5,472)	7,952 (7,246 - 8,707)	7,700 (7,002 - 8,411)	7,393 (6,724 - 8,110)	28 (18 - 42)	32 (20 - 47)	34 (21 - 50)
**Slovakia**									
Under-5 years (rate per capita)	0.475 (0.408 - 0.529)	0.468 (0.403 - 0.520)	0.470 (0.408 - 0.525)	0.641 (0.465 - 0.794)	0.633 (0.459 - 0.784)	0.635 (0.460 - 0.786)	0.113 (0.048 - 0.225)	0.111 (0.047 - 0.219)	0.112 (0.047 - 0.218)
5-14 years (rate per capita)	0.623 (0.562 - 0.679)	0.615 (0.554 - 0.673)	0.626 (0.568 - 0.684)	1.043 (0.837 - 1.292)	1.025 (0.821 - 1.270)	1.052 (0.846 - 1.302)	0.617 (0.342 - 1.027)	0.618 (0.345 - 1.015)	0.617 (0.342 - 1.021)
15 - 49 years (rate per capita)	0.544 (0.503 - 0.587)	0.544 (0.503 - 0.584)	0.543 (0.500 - 0.588)	0.781 (0.671 - 0.889)	0.782 (0.670 - 0.890)	0.786 (0.673 - 0.902)	1.786 (1.040 - 2.826)	1.819 (1.054 - 2.870)	1.866 (1.090 - 2.958)
50 - 69 years (rate per capita)	0.622 (0.575 - 0.674)	0.616 (0.568 - 0.667)	0.618 (0.569 - 0.673)	0.864 (0.698 - 1.033)	0.860 (0.691 - 1.034)	0.863 (0.693 - 1.036)	5.523 (3.486 - 8.216)	5.193 (3.242 - 7.752)	5.324 (3.330 - 7.942)
70+ years (rate per capita)	0.650 (0.614 - 0.689)	0.651 (0.615 - 0.691)	0.643 (0.606 - 0.680)	0.851 (0.695 - 0.998)	0.851 (0.697 - 1.001)	0.848 (0.699 - 0.988)	8.561 (5.631 - 12.188)	8.551 (5.611 - 12.178)	8.415 (5.499 - 12.033)
All ages (rate per capita)	0.573 (0.547 - 0.600)	0.573 (0.546 - 0.601)	0.575 (0.548 - 0.608)	0.835 (0.758 - 0.917)	0.826 (0.753 - 0.906)	0.826 (0.751 - 0.907)	2.533 (1.566 - 3.834)	2.851 (1.748 - 4.307)	3.126 (1.936 - 4.716)
Age-standardised (rate per capita)	0.572 (0.545 - 0.599)	0.571 (0.544 - 0.598)	0.571 (0.544 - 0.600)	0.832 (0.757 - 0.913)	0.832 (0.757 - 0.913)	0.833 (0.757 - 0.913)	2.502 (1.548 - 3.785)	2.499 (1.530 - 3.780)	2.504 (1.540 - 3.788)
Total number of cases (thousands)	3,021 (2,882 - 3,164)	3,087 (2,942 - 3,240)	3,192 (3,042 - 3,375)	4,400 (3,996 - 4,831)	4,453 (4,056 - 4,882)	4,589 (4,171 - 5,038)	13 (8 - 20)	15 (9 - 23)	17 (11 - 26)
**Slovenia**									
Under-5 years (rate per capita)	0.383 (0.326 - 0.443)	0.375 (0.319 - 0.438)	0.377 (0.324 - 0.435)	0.604 (0.378 - 0.811)	0.611 (0.375 - 0.816)	0.615 (0.377 - 0.821)	0.148 (0.063 - 0.296)	0.145 (0.061 - 0.296)	0.146 (0.062 - 0.295)
5-14 years (rate per capita)	0.541 (0.495 - 0.582)	0.604 (0.557 - 0.655)	0.614 (0.562 - 0.662)	0.970 (0.794 - 1.202)	1.022 (0.846 - 1.250)	1.050 (0.874 - 1.286)	0.729 (0.390 - 1.232)	0.806 (0.427 - 1.367)	0.801 (0.430 - 1.348)
15 - 49 years (rate per capita)	0.533 (0.512 - 0.557)	0.539 (0.519 - 0.563)	0.540 (0.517 - 0.565)	0.822 (0.751 - 0.897)	0.822 (0.749 - 0.899)	0.827 (0.754 - 0.904)	2.454 (1.417 - 3.954)	2.536 (1.465 - 4.081)	2.621 (1.521 - 4.237)
50 - 69 years (rate per capita)	0.635 (0.610 - 0.660)	0.629 (0.604 - 0.655)	0.632 (0.608 - 0.657)	0.931 (0.824 - 1.039)	0.928 (0.827 - 1.034)	0.931 (0.828 - 1.039)	7.539 (4.743 - 11.156)	7.379 (4.648 - 10.927)	7.478 (4.726 - 11.071)
70+ years (rate per capita)	0.674 (0.654 - 0.696)	0.676 (0.658 - 0.699)	0.666 (0.648 - 0.687)	0.889 (0.790 - 0.991)	0.894 (0.793 - 0.999)	0.885 (0.791 - 0.983)	11.848 (7.899 - 16.648)	11.911 (7.880 - 16.857)	11.615 (7.708 - 16.270)
All ages (rate per capita)	0.556 (0.541 - 0.571)	0.574 (0.558 - 0.589)	0.580 (0.564 - 0.594)	0.857 (0.798 - 0.919)	0.865 (0.812 - 0.921)	0.873 (0.820 - 0.929)	3.735 (2.297 - 5.639)	4.425 (2.751 - 6.620)	4.803 (3.013 - 7.136)
Age-standardised (rate per capita)	0.548 (0.532 - 0.563)	0.560 (0.543 - 0.576)	0.560 (0.544 - 0.575)	0.850 (0.789 - 0.914)	0.860 (0.799 - 0.925)	0.860 (0.799 - 0.925)	3.421 (2.101 - 5.170)	3.417 (2.095 - 5.152)	3.408 (2.082 - 5.176)
Total number of cases (thousands)	1,115 (1,086 - 1,145)	1,147 (1,115 - 1,177)	1,196 (1,164 - 1,226)	1,720 (1,602 - 1,844)	1,727 (1,622 - 1,840)	1,801 (1,691 - 1,917)	7 (5 - 11)	9 (5 - 13)	10 (6 - 15)
**Central Asia**									
Under-5 years (rate per capita)	0.462 (0.427 - 0.497)	0.457 (0.423 - 0.493)	0.463 (0.424 - 0.497)	0.624 (0.452 - 0.777)	0.619 (0.448 - 0.770)	0.626 (0.453 - 0.779)	0.124 (0.053 - 0.242)	0.121 (0.052 - 0.237)	0.124 (0.053 - 0.241)
5-14 years (rate per capita)	0.607 (0.578 - 0.637)	0.594 (0.568 - 0.620)	0.608 (0.580 - 0.639)	1.087 (0.897 - 1.315)	1.048 (0.862 - 1.270)	1.090 (0.899 - 1.317)	0.648 (0.358 - 1.064)	0.640 (0.352 - 1.056)	0.642 (0.352 - 1.060)
15 - 49 years (rate per capita)	0.533 (0.521 - 0.546)	0.536 (0.524 - 0.548)	0.534 (0.524 - 0.547)	0.811 (0.744 - 0.882)	0.811 (0.744 - 0.874)	0.815 (0.750 - 0.883)	1.853 (1.101 - 2.883)	1.943 (1.154 - 3.006)	1.979 (1.177 - 3.065)
50 - 69 years (rate per capita)	0.608 (0.595 - 0.623)	0.610 (0.596 - 0.623)	0.603 (0.587 - 0.620)	0.926 (0.830 - 1.031)	0.923 (0.833 - 1.028)	0.921 (0.822 - 1.029)	6.780 (4.363 - 9.902)	6.882 (4.423 - 10.039)	6.479 (4.181 - 9.443)
70+ years (rate per capita)	0.675 (0.665 - 0.687)	0.682 (0.671 - 0.695)	0.675 (0.663 - 0.687)	0.910 (0.818 - 1.000)	0.915 (0.816 - 1.009)	0.909 (0.815 - 1.002)	12.218 (8.125 - 17.017)	12.274 (8.145 - 17.159)	12.140 (8.061 - 16.944)
All ages (rate per capita)	0.554 (0.543 - 0.564)	0.554 (0.545 - 0.564)	0.555 (0.546 - 0.564)	0.865 (0.799 - 0.931)	0.857 (0.799 - 0.919)	0.860 (0.804 - 0.919)	2.301 (1.438 - 3.412)	2.473 (1.544 - 3.665)	2.639 (1.651 - 3.918)
Age-standardised (rate per capita)	0.563 (0.555 - 0.571)	0.563 (0.555 - 0.572)	0.563 (0.555 - 0.572)	0.870 (0.816 - 0.928)	0.868 (0.814 - 0.925)	0.868 (0.814 - 0.925)	3.175 (2.015 - 4.655)	3.157 (1.994 - 4.634)	3.156 (2.003 - 4.633)
Total number of cases (thousands)	38,085 (37,365 - 38,789)	42,678 (41,927 - 43,428)	48,261 (47,475 - 49,084)	59,511 (54,987 - 64,065)	65,970 (61,475 - 70,762)	74,849 (69,977 - 79,947)	158 (99 - 235)	190 (119 - 282)	230 (144 - 341)
**Armenia**									
Under-5 years (rate per capita)	0.466 (0.395 - 0.531)	0.461 (0.395 - 0.522)	0.467 (0.399 - 0.529)	0.626 (0.454 - 0.778)	0.619 (0.450 - 0.770)	0.626 (0.455 - 0.779)	0.111 (0.046 - 0.219)	0.110 (0.046 - 0.213)	0.111 (0.047 - 0.217)
5-14 years (rate per capita)	0.608 (0.559 - 0.654)	0.588 (0.534 - 0.637)	0.609 (0.558 - 0.655)	1.079 (0.889 - 1.303)	1.026 (0.843 - 1.244)	1.083 (0.893 - 1.308)	0.606 (0.334 - 0.991)	0.606 (0.333 - 1.006)	0.606 (0.332 - 0.998)
15 - 49 years (rate per capita)	0.535 (0.515 - 0.559)	0.540 (0.521 - 0.564)	0.536 (0.517 - 0.560)	0.804 (0.738 - 0.876)	0.804 (0.740 - 0.869)	0.809 (0.741 - 0.878)	1.865 (1.116 - 2.885)	1.981 (1.183 - 3.051)	1.996 (1.194 - 3.078)
50 - 69 years (rate per capita)	0.610 (0.586 - 0.635)	0.615 (0.590 - 0.639)	0.608 (0.583 - 0.633)	0.913 (0.819 - 1.018)	0.912 (0.823 - 1.010)	0.913 (0.816 - 1.019)	6.668 (4.224 - 9.715)	7.003 (4.487 - 10.185)	6.595 (4.193 - 9.588)
70+ years (rate per capita)	0.672 (0.651 - 0.693)	0.681 (0.660 - 0.705)	0.668 (0.648 - 0.690)	0.895 (0.806 - 0.985)	0.904 (0.805 - 1.000)	0.892 (0.802 - 0.980)	11.862 (7.845 - 16.567)	12.063 (7.990 - 16.938)	11.830 (7.808 - 16.582)
All ages (rate per capita)	0.559 (0.543 - 0.576)	0.564 (0.548 - 0.581)	0.567 (0.552 - 0.582)	0.860 (0.800 - 0.922)	0.850 (0.798 - 0.904)	0.860 (0.807 - 0.910)	2.573 (1.611 - 3.804)	3.188 (2.013 - 4.674)	3.562 (2.250 - 5.227)
Age-standardised (rate per capita)	0.565 (0.550 - 0.581)	0.564 (0.548 - 0.580)	0.565 (0.548 - 0.580)	0.859 (0.805 - 0.915)	0.859 (0.805 - 0.915)	0.859 (0.805 - 0.915)	3.096 (1.962 - 4.536)	3.091 (1.959 - 4.538)	3.098 (1.960 - 4.545)
Total number of cases (thousands)	1,973 (1,915 - 2,035)	1,703 (1,655 - 1,753)	1,706 (1,662 - 1,750)	3,037 (2,825 - 3,254)	2,567 (2,409 - 2,729)	2,586 (2,427 - 2,737)	9 (6 - 13)	10 (6 - 14)	11 (7 - 16)
**Azerbaijan**									
Under-5 years (rate per capita)	0.458 (0.395 - 0.514)	0.455 (0.392 - 0.512)	0.466 (0.403 - 0.527)	0.613 (0.445 - 0.762)	0.609 (0.443 - 0.758)	0.624 (0.453 - 0.776)	0.109 (0.046 - 0.214)	0.108 (0.046 - 0.217)	0.111 (0.046 - 0.216)
5-14 years (rate per capita)	0.609 (0.554 - 0.659)	0.592 (0.539 - 0.642)	0.604 (0.547 - 0.652)	1.079 (0.889 - 1.303)	1.031 (0.847 - 1.251)	1.067 (0.876 - 1.292)	0.606 (0.333 - 0.991)	0.607 (0.337 - 1.004)	0.605 (0.331 - 0.987)
15 - 49 years (rate per capita)	0.534 (0.513 - 0.560)	0.539 (0.518 - 0.560)	0.536 (0.517 - 0.560)	0.798 (0.731 - 0.871)	0.804 (0.741 - 0.867)	0.809 (0.742 - 0.877)	1.789 (1.074 - 2.796)	1.966 (1.173 - 3.044)	1.983 (1.185 - 3.068)
50 - 69 years (rate per capita)	0.606 (0.580 - 0.633)	0.610 (0.584 - 0.635)	0.600 (0.568 - 0.627)	0.912 (0.816 - 1.019)	0.910 (0.821 - 1.009)	0.909 (0.810 - 1.019)	6.549 (4.204 - 9.521)	6.796 (4.339 - 9.920)	6.184 (3.925 - 8.974)
70+ years (rate per capita)	0.686 (0.664 - 0.709)	0.686 (0.664 - 0.711)	0.676 (0.655 - 0.698)	0.906 (0.806 - 1.003)	0.910 (0.804 - 1.010)	0.899 (0.806 - 0.990)	12.361 (8.186 - 17.250)	12.229 (8.115 - 17.127)	12.031 (8.010 - 16.867)
All ages (rate per capita)	0.554 (0.536 - 0.573)	0.555 (0.539 - 0.572)	0.555 (0.540 - 0.573)	0.853 (0.790 - 0.918)	0.846 (0.790 - 0.906)	0.845 (0.790 - 0.898)	2.227 (1.383 - 3.298)	2.498 (1.553 - 3.707)	2.852 (1.790 - 4.207)
Age-standardised (rate per capita)	0.565 (0.550 - 0.582)	0.564 (0.548 - 0.579)	0.564 (0.548 - 0.582)	0.859 (0.805 - 0.915)	0.859 (0.805 - 0.915)	0.859 (0.805 - 0.916)	3.111 (1.972 - 4.566)	3.101 (1.963 - 4.553)	3.098 (1.960 - 4.533)
Total number of cases (thousands)	3,963 (3,836 - 4,096)	4,809 (4,663 - 4,951)	5,434 (5,285 - 5,604)	6,102 (5,652 - 6,569)	7,328 (6,841 - 7,847)	8,269 (7,730 - 8,781)	16 (10 - 24)	22 (13 - 32)	28 (18 - 41)
**Georgia**									
Under-5 years (rate per capita)	0.468 (0.406 - 0.530)	0.463 (0.397 - 0.520)	0.469 (0.404 - 0.530)	0.627 (0.455 - 0.780)	0.620 (0.450 - 0.771)	0.629 (0.457 - 0.782)	0.112 (0.047 - 0.218)	0.110 (0.046 - 0.218)	0.112 (0.047 - 0.219)
5-14 years (rate per capita)	0.604 (0.553 - 0.649)	0.587 (0.535 - 0.634)	0.610 (0.561 - 0.659)	1.066 (0.875 - 1.292)	1.014 (0.834 - 1.234)	1.085 (0.895 - 1.311)	0.607 (0.336 - 0.996)	0.610 (0.334 - 1.001)	0.604 (0.333 - 0.991)
15 - 49 years (rate per capita)	0.535 (0.516 - 0.558)	0.539 (0.517 - 0.562)	0.537 (0.516 - 0.559)	0.807 (0.742 - 0.872)	0.808 (0.744 - 0.871)	0.812 (0.748 - 0.877)	1.898 (1.139 - 2.938)	1.987 (1.187 - 3.065)	2.009 (1.208 - 3.108)
50 - 69 years (rate per capita)	0.607 (0.583 - 0.630)	0.612 (0.589 - 0.636)	0.606 (0.580 - 0.634)	0.913 (0.820 - 1.015)	0.913 (0.826 - 1.010)	0.912 (0.818 - 1.016)	6.533 (4.222 - 9.595)	6.823 (4.418 - 10.000)	6.408 (4.125 - 9.369)
70+ years (rate per capita)	0.667 (0.647 - 0.688)	0.674 (0.655 - 0.696)	0.661 (0.643 - 0.680)	0.896 (0.804 - 0.989)	0.902 (0.805 - 0.997)	0.893 (0.804 - 0.982)	11.442 (7.648 - 15.999)	11.549 (7.685 - 16.120)	11.213 (7.492 - 15.696)
All ages (rate per capita)	0.564 (0.549 - 0.579)	0.567 (0.552 - 0.582)	0.568 (0.554 - 0.582)	0.861 (0.807 - 0.917)	0.853 (0.801 - 0.902)	0.859 (0.809 - 0.906)	3.078 (1.948 - 4.544)	3.533 (2.250 - 5.153)	3.681 (2.347 - 5.411)
Age-standardised (rate per capita)	0.563 (0.548 - 0.577)	0.563 (0.547 - 0.578)	0.562 (0.547 - 0.577)	0.858 (0.804 - 0.915)	0.858 (0.804 - 0.915)	0.859 (0.805 - 0.915)	2.988 (1.891 - 4.395)	2.985 (1.886 - 4.383)	2.973 (1.882 - 4.392)
Total number of cases (thousands)	3,078 (2,998 - 3,160)	2,560 (2,490 - 2,626)	2,275 (2,217 - 2,331)	4,704 (4,407 - 5,011)	3,849 (3,617 - 4,073)	3,439 (3,239 - 3,630)	17 (11 - 25)	16 (10 - 23)	15 (9 - 22)
**Kazakhstan**									
Under-5 years (rate per capita)	0.465 (0.402 - 0.524)	0.447 (0.381 - 0.506)	0.461 (0.399 - 0.518)	0.651 (0.466 - 0.814)	0.629 (0.450 - 0.787)	0.646 (0.463 - 0.809)	0.180 (0.076 - 0.365)	0.174 (0.074 - 0.342)	0.179 (0.075 - 0.365)
5-14 years (rate per capita)	0.600 (0.550 - 0.645)	0.587 (0.535 - 0.635)	0.611 (0.563 - 0.659)	1.113 (0.917 - 1.352)	1.078 (0.887 - 1.313)	1.147 (0.944 - 1.387)	0.797 (0.428 - 1.335)	0.802 (0.421 - 1.348)	0.795 (0.424 - 1.325)
15 - 49 years (rate per capita)	0.528 (0.509 - 0.552)	0.531 (0.512 - 0.554)	0.529 (0.512 - 0.552)	0.853 (0.781 - 0.924)	0.854 (0.783 - 0.922)	0.859 (0.787 - 0.930)	2.120 (1.262 - 3.361)	2.210 (1.313 - 3.493)	2.251 (1.336 - 3.568)
50 - 69 years (rate per capita)	0.607 (0.582 - 0.633)	0.610 (0.587 - 0.635)	0.604 (0.578 - 0.630)	0.960 (0.859 - 1.067)	0.960 (0.862 - 1.066)	0.960 (0.855 - 1.071)	7.140 (4.627 - 10.421)	7.306 (4.692 - 10.675)	6.951 (4.499 - 10.147)
70+ years (rate per capita)	0.683 (0.664 - 0.704)	0.689 (0.669 - 0.708)	0.689 (0.669 - 0.709)	0.943 (0.844 - 1.043)	0.950 (0.847 - 1.053)	0.950 (0.845 - 1.054)	13.044 (8.725 - 18.153)	13.090 (8.693 - 18.227)	13.120 (8.792 - 18.251)
All ages (rate per capita)	0.552 (0.535 - 0.569)	0.552 (0.536 - 0.569)	0.555 (0.540 - 0.572)	0.901 (0.837 - 0.966)	0.892 (0.835 - 0.951)	0.903 (0.844 - 0.963)	2.768 (1.737 - 4.141)	3.038 (1.901 - 4.512)	3.125 (1.968 - 4.616)

	Prevalence 1990	Prevalence 2005	Prevalence 2015	Incidence 1990	Incidence 2005	Incidence 2015	YLD rate 1990	YLD rate 2005	YLD rate 2015
Age-standardised (rate per capita)	0.560 (0.545 - 0.575)	0.560 (0.544 - 0.575)	0.560 (0.545 - 0.576)	0.905 (0.846 - 0.964)	0.904 (0.846 - 0.964)	0.905 (0.846 - 0.965)	3.429 (2.182 - 5.020)	3.421 (2.166 - 5.033)	3.416 (2.178 - 5.003)
Total number of cases (thousands)	9,167 (8,884 - 9,448)	8,546 (8,296 - 8,808)	9,741 (9,469 - 10,024)	14,949 (13,896 - 16,041)	13,810 (12,926 - 14,722)	15,844 (14,807 - 16,891)	46 (29 - 69)	47 (29 - 70)	55 (35 - 81)
**Kyrgyzstan**									
Under-5 years (rate per capita)	0.461 (0.403 - 0.519)	0.460 (0.394 - 0.517)	0.462 (0.399 - 0.519)	0.618 (0.449 - 0.769)	0.617 (0.447 - 0.767)	0.618 (0.449 - 0.769)	0.110 (0.046 - 0.212)	0.110 (0.046 - 0.224)	0.110 (0.046 - 0.215)
5-14 years (rate per capita)	0.609 (0.560 - 0.655)	0.597 (0.543 - 0.644)	0.611 (0.560 - 0.657)	1.079 (0.889 - 1.304)	1.044 (0.858 - 1.266)	1.088 (0.897 - 1.316)	0.605 (0.331 - 0.989)	0.609 (0.335 - 0.993)	0.606 (0.331 - 0.988)
15 - 49 years (rate per capita)	0.534 (0.512 - 0.558)	0.537 (0.516 - 0.562)	0.535 (0.516 - 0.558)	0.797 (0.731 - 0.868)	0.800 (0.736 - 0.865)	0.803 (0.738 - 0.870)	1.765 (1.054 - 2.735)	1.868 (1.119 - 2.885)	1.885 (1.124 - 2.931)
50 - 69 years (rate per capita)	0.610 (0.585 - 0.636)	0.607 (0.582 - 0.632)	0.602 (0.575 - 0.631)	0.913 (0.820 - 1.017)	0.909 (0.819 - 1.013)	0.910 (0.811 - 1.018)	6.763 (4.353 - 9.808)	6.639 (4.294 - 9.602)	6.323 (4.034 - 9.194)
70+ years (rate per capita)	0.673 (0.651 - 0.695)	0.680 (0.658 - 0.702)	0.672 (0.651 - 0.693)	0.896 (0.805 - 0.987)	0.903 (0.805 - 0.998)	0.896 (0.805 - 0.987)	12.015 (7.994 - 16.812)	12.130 (8.088 - 17.091)	11.955 (7.972 - 16.823)
All ages (rate per capita)	0.555 (0.537 - 0.572)	0.554 (0.537 - 0.571)	0.553 (0.537 - 0.569)	0.854 (0.790 - 0.921)	0.848 (0.787 - 0.911)	0.850 (0.790 - 0.911)	2.195 (1.367 - 3.248)	2.249 (1.397 - 3.335)	2.314 (1.437 - 3.427)
Age-standardised (rate per capita)	0.564 (0.549 - 0.579)	0.564 (0.549 - 0.579)	0.564 (0.550 - 0.579)	0.859 (0.805 - 0.915)	0.859 (0.804 - 0.915)	0.859 (0.805 - 0.915)	3.104 (1.957 - 4.568)	3.097 (1.959 - 4.540)	3.101 (1.957 - 4.551)
Total number of cases (thousands)	2,441 (2,364 - 2,519)	2,849 (2,762 - 2,937)	3,259 (3,164 - 3,356)	3,760 (3,475 - 4,051)	4,361 (4,048 - 4,688)	5,007 (4,658 - 5,367)	10 (6 - 14)	12 (7 - 17)	14 (8 - 20)
**Mongolia**									
Under-5 years (rate per capita)	0.464 (0.396 - 0.526)	0.455 (0.386 - 0.516)	0.462 (0.398 - 0.524)	0.622 (0.451 - 0.774)	0.612 (0.444 - 0.761)	0.619 (0.449 - 0.770)	0.110 (0.046 - 0.217)	0.109 (0.046 - 0.212)	0.110 (0.046 - 0.215)
5-14 years (rate per capita)	0.606 (0.550 - 0.654)	0.595 (0.540 - 0.640)	0.611 (0.559 - 0.660)	1.074 (0.884 - 1.297)	1.037 (0.852 - 1.259)	1.090 (0.899 - 1.319)	0.609 (0.336 - 1.010)	0.610 (0.333 - 0.993)	0.607 (0.331 - 0.988)
15 - 49 years (rate per capita)	0.535 (0.512 - 0.559)	0.536 (0.515 - 0.560)	0.536 (0.517 - 0.560)	0.792 (0.725 - 0.862)	0.799 (0.735 - 0.866)	0.809 (0.743 - 0.876)	1.723 (1.036 - 2.683)	1.840 (1.103 - 2.841)	1.976 (1.191 - 3.038)
50 - 69 years (rate per capita)	0.610 (0.586 - 0.635)	0.608 (0.583 - 0.634)	0.600 (0.573 - 0.628)	0.913 (0.821 - 1.016)	0.911 (0.820 - 1.015)	0.908 (0.809 - 1.016)	6.764 (4.313 - 9.930)	6.628 (4.259 - 9.654)	6.254 (4.015 - 9.087)
70+ years (rate per capita)	0.681 (0.659 - 0.705)	0.683 (0.661 - 0.707)	0.678 (0.656 - 0.701)	0.906 (0.805 - 1.001)	0.907 (0.805 - 1.003)	0.902 (0.805 - 0.994)	12.100 (8.014 - 17.149)	12.163 (8.098 - 17.170)	12.005 (8.015 - 16.933)
All ages (rate per capita)	0.552 (0.533 - 0.572)	0.550 (0.534 - 0.569)	0.552 (0.536 - 0.568)	0.851 (0.784 - 0.921)	0.842 (0.782 - 0.905)	0.850 (0.794 - 0.909)	1.923 (1.192 - 2.843)	2.090 (1.286 - 3.105)	2.339 (1.443 - 3.462)
Age-standardised (rate per capita)	0.564 (0.549 - 0.579)	0.564 (0.549 - 0.580)	0.564 (0.549 - 0.579)	0.859 (0.804 - 0.915)	0.859 (0.804 - 0.915)	0.859 (0.805 - 0.915)	3.097 (1.953 - 4.545)	3.094 (1.954 - 4.535)	3.093 (1.954 - 4.539)
Total number of cases (thousands)	1,208 (1,165 - 1,251)	1,394 (1,352 - 1,441)	1,630 (1,582 - 1,678)	1,862 (1,714 - 2,014)	2,134 (1,981 - 2,293)	2,510 (2,343 - 2,684)	4 (3 - 6)	5 (3 - 8)	7 (4 - 10)
**Tajikistan**									
Under-5 years (rate per capita)	0.458 (0.394 - 0.516)	0.459 (0.400 - 0.520)	0.459 (0.391 - 0.517)	0.612 (0.444 - 0.761)	0.616 (0.447 - 0.766)	0.616 (0.447 - 0.766)	0.108 (0.046 - 0.209)	0.109 (0.046 - 0.213)	0.109 (0.046 - 0.213)
5-14 years (rate per capita)	0.612 (0.564 - 0.661)	0.602 (0.550 - 0.649)	0.606 (0.553 - 0.654)	1.088 (0.898 - 1.316)	1.060 (0.870 - 1.287)	1.075 (0.886 - 1.298)	0.606 (0.333 - 0.992)	0.607 (0.334 - 0.994)	0.605 (0.332 - 0.983)
15 - 49 years (rate per capita)	0.535 (0.513 - 0.560)	0.537 (0.516 - 0.562)	0.535 (0.515 - 0.561)	0.793 (0.724 - 0.865)	0.795 (0.729 - 0.862)	0.799 (0.733 - 0.868)	1.702 (1.020 - 2.656)	1.785 (1.066 - 2.769)	1.822 (1.092 - 2.840)
50 - 69 years (rate per capita)	0.609 (0.583 - 0.634)	0.609 (0.583 - 0.637)	0.599 (0.572 - 0.626)	0.913 (0.819 - 1.017)	0.911 (0.821 - 1.014)	0.908 (0.808 - 1.017)	6.697 (4.277 - 9.803)	6.691 (4.256 - 9.771)	6.169 (3.878 - 8.975)
70+ years (rate per capita)	0.671 (0.651 - 0.693)	0.681 (0.659 - 0.702)	0.673 (0.653 - 0.695)	0.895 (0.807 - 0.984)	0.906 (0.805 - 1.001)	0.899 (0.807 - 0.990)	11.917 (7.931 - 16.749)	12.078 (8.047 - 16.955)	11.876 (7.925 - 16.614)
All ages (rate per capita)	0.551 (0.531 - 0.572)	0.551 (0.532 - 0.572)	0.549 (0.532 - 0.567)	0.851 (0.780 - 0.921)	0.849 (0.782 - 0.919)	0.845 (0.782 - 0.909)	1.869 (1.154 - 2.766)	1.844 (1.137 - 2.755)	1.984 (1.225 - 2.975)
Age-standardised (rate per capita)	0.565 (0.550 - 0.580)	0.564 (0.549 - 0.580)	0.564 (0.549 - 0.580)	0.859 (0.804 - 0.915)	0.859 (0.804 - 0.915)	0.859 (0.805 - 0.915)	3.103 (1.950 - 4.554)	3.091 (1.942 - 4.531)	3.090 (1.947 - 4.544)
Total number of cases (thousands)	2,912 (2,806 - 3,023)	3,779 (3,649 - 3,922)	4,665 (4,519 - 4,824)	4,495 (4,120 - 4,866)	5,822 (5,358 - 6,296)	7,180 (6,651 - 7,731)	10 (6 - 15)	13 (8 - 19)	17 (10 - 25)
**Turkmenistan**									
Under-5 years (rate per capita)	0.456 (0.388 - 0.514)	0.461 (0.399 - 0.523)	0.461 (0.393 - 0.528)	0.611 (0.444 - 0.760)	0.619 (0.449 - 0.769)	0.620 (0.450 - 0.771)	0.108 (0.046 - 0.216)	0.110 (0.047 - 0.215)	0.110 (0.046 - 0.224)
5-14 years (rate per capita)	0.608 (0.556 - 0.654)	0.595 (0.542 - 0.645)	0.603 (0.549 - 0.649)	1.078 (0.888 - 1.302)	1.039 (0.853 - 1.260)	1.062 (0.872 - 1.289)	0.606 (0.331 - 0.986)	0.609 (0.331 - 1.008)	0.608 (0.337 - 1.001)
15 - 49 years (rate per capita)	0.533 (0.511 - 0.558)	0.536 (0.517 - 0.559)	0.536 (0.516 - 0.558)	0.795 (0.728 - 0.866)	0.799 (0.735 - 0.864)	0.804 (0.739 - 0.871)	1.731 (1.036 - 2.687)	1.860 (1.112 - 2.875)	1.911 (1.151 - 2.956)
50 - 69 years (rate per capita)	0.608 (0.582 - 0.634)	0.607 (0.579 - 0.633)	0.603 (0.575 - 0.628)	0.913 (0.820 - 1.017)	0.909 (0.819 - 1.012)	0.910 (0.812 - 1.019)	6.699 (4.333 - 9.755)	6.602 (4.246 - 9.642)	6.375 (4.104 - 9.306)
70+ years (rate per capita)	0.677 (0.654 - 0.698)	0.682 (0.661 - 0.704)	0.675 (0.653 - 0.696)	0.899 (0.806 - 0.989)	0.905 (0.806 - 1.000)	0.898 (0.807 - 0.989)	12.143 (8.112 - 17.031)	12.207 (8.168 - 17.094)	12.030 (8.057 - 16.845)
All ages (rate per capita)	0.550 (0.531 - 0.570)	0.552 (0.535 - 0.569)	0.553 (0.538 - 0.570)	0.850 (0.781 - 0.919)	0.846 (0.783 - 0.910)	0.850 (0.793 - 0.909)	1.926 (1.195 - 2.866)	2.124 (1.319 - 3.163)	2.349 (1.458 - 3.490)
Age-standardised (rate per capita)	0.564 (0.549 - 0.579)	0.564 (0.549 - 0.579)	0.564 (0.549 - 0.580)	0.859 (0.804 - 0.915)	0.859 (0.804 - 0.915)	0.859 (0.805 - 0.915)	3.111 (1.970 - 4.572)	3.101 (1.963 - 4.546)	3.100 (1.961 - 4.547)
Total number of cases (thousands)	2,016 (1,946 - 2,086)	2,619 (2,540 - 2,702)	2,977 (2,893 - 3,066)	3,114 (2,862 - 3,366)	4,016 (3,719 - 4,321)	4,573 (4,266 - 4,894)	7 (4 - 10)	10 (6 - 15)	13 (8 - 19)
**Uzbekistan**									
Under-5 years (rate per capita)	0.461 (0.396 - 0.521)	0.460 (0.394 - 0.523)	0.463 (0.397 - 0.522)	0.618 (0.448 - 0.768)	0.619 (0.449 - 0.769)	0.621 (0.451 - 0.772)	0.110 (0.046 - 0.212)	0.110 (0.046 - 0.212)	0.110 (0.047 - 0.214)
5-14 years (rate per capita)	0.609 (0.552 - 0.657)	0.596 (0.542 - 0.645)	0.607 (0.556 - 0.658)	1.082 (0.892 - 1.307)	1.044 (0.858 - 1.267)	1.077 (0.887 - 1.300)	0.607 (0.341 - 0.995)	0.608 (0.332 - 0.997)	0.607 (0.333 - 1.000)
15 - 49 years (rate per capita)	0.534 (0.511 - 0.557)	0.537 (0.516 - 0.562)	0.535 (0.516 - 0.559)	0.795 (0.728 - 0.866)	0.798 (0.733 - 0.864)	0.804 (0.738 - 0.870)	1.725 (1.030 - 2.677)	1.843 (1.092 - 2.859)	1.895 (1.125 - 2.926)
50 - 69 years (rate per capita)	0.609 (0.583 - 0.635)	0.609 (0.583 - 0.634)	0.603 (0.575 - 0.630)	0.912 (0.819 - 1.017)	0.910 (0.820 - 1.011)	0.910 (0.813 - 1.018)	6.669 (4.269 - 9.712)	6.683 (4.305 - 9.721)	6.391 (4.131 - 9.309)
70+ years (rate per capita)	0.670 (0.650 - 0.690)	0.680 (0.658 - 0.702)	0.671 (0.649 - 0.691)	0.893 (0.806 - 0.981)	0.903 (0.805 - 0.996)	0.895 (0.806 - 0.986)	11.910 (7.923 - 16.734)	12.124 (8.065 - 17.033)	11.926 (7.969 - 16.752)
All ages (rate per capita)	0.552 (0.531 - 0.571)	0.554 (0.535 - 0.571)	0.554 (0.536 - 0.569)	0.852 (0.782 - 0.921)	0.848 (0.786 - 0.912)	0.850 (0.793 - 0.909)	1.938 (1.205 - 2.854)	2.175 (1.355 - 3.220)	2.384 (1.485 - 3.536)
Age-standardised (rate per capita)	0.565 (0.548 - 0.580)	0.564 (0.548 - 0.579)	0.564 (0.548 - 0.579)	0.859 (0.804 - 0.915)	0.859 (0.804 - 0.915)	0.859 (0.805 - 0.915)	3.106 (1.964 - 4.560)	3.101 (1.963 - 4.550)	3.099 (1.963 - 4.541)
Total number of cases (thousands)	11,327 (10,894 - 11,725)	14,418 (13,921 - 14,863)	16,573 (16,054 - 17,050)	17,489 (16,054 - 18,904)	22,084 (20,476 - 23,760)	25,440 (23,736 - 27,207)	40 (25 - 59)	57 (35 - 84)	71 (44 - 106)
**Latin America and Caribbean**									
Under-5 years (rate per capita)	0.338 (0.323 - 0.358)	0.317 (0.301 - 0.336)	0.342 (0.325 - 0.362)	0.552 (0.328 - 0.763)	0.506 (0.308 - 0.702)	0.531 (0.330 - 0.728)	0.101 (0.045 - 0.201)	0.097 (0.042 - 0.190)	0.104 (0.045 - 0.208)
5-14 years (rate per capita)	0.566 (0.547 - 0.582)	0.559 (0.538 - 0.575)	0.566 (0.547 - 0.585)	0.825 (0.668 - 1.014)	0.793 (0.639 - 0.975)	0.811 (0.655 - 0.994)	0.665 (0.362 - 1.085)	0.667 (0.364 - 1.088)	0.673 (0.367 - 1.107)
15 - 49 years (rate per capita)	0.512 (0.502 - 0.521)	0.516 (0.507 - 0.527)	0.517 (0.508 - 0.528)	0.758 (0.693 - 0.823)	0.760 (0.698 - 0.821)	0.757 (0.695 - 0.819)	1.887 (1.079 - 3.040)	1.999 (1.144 - 3.236)	2.048 (1.171 - 3.312)
50 - 69 years (rate per capita)	0.626 (0.615 - 0.635)	0.624 (0.614 - 0.632)	0.624 (0.613 - 0.632)	0.861 (0.778 - 0.943)	0.862 (0.778 - 0.944)	0.860 (0.779 - 0.941)	7.292 (4.642 - 10.778)	7.247 (4.613 - 10.692)	7.258 (4.602 - 10.727)
70+ years (rate per capita)	0.669 (0.661 - 0.678)	0.670 (0.661 - 0.678)	0.667 (0.659 - 0.674)	0.827 (0.748 - 0.898)	0.832 (0.757 - 0.900)	0.833 (0.758 - 0.899)	12.063 (8.001 - 16.792)	12.172 (8.095 - 16.896)	12.040 (7.997 - 16.718)
All ages (rate per capita)	0.518 (0.511 - 0.525)	0.523 (0.517 - 0.530)	0.534 (0.528 - 0.541)	0.759 (0.697 - 0.825)	0.756 (0.703 - 0.812)	0.767 (0.719 - 0.817)	2.154 (1.301 - 3.280)	2.549 (1.551 - 3.887)	2.923 (1.798 - 4.439)
Age-standardised (rate per capita)	0.538 (0.533 - 0.545)	0.536 (0.530 - 0.542)	0.539 (0.533 - 0.546)	0.777 (0.726 - 0.829)	0.767 (0.719 - 0.817)	0.770 (0.722 - 0.819)	3.249 (2.016 - 4.862)	3.269 (2.030 - 4.892)	3.261 (2.025 - 4.880)
Total number of cases (thousands)	206,273 (203,659 - 209,229)	264,500 (261,413 - 267,962)	304,586 (301,096 - 308,225)	302,499 (277,541 - 328,767)	382,142 (355,317 - 410,229)	436,954 (409,720 - 465,570)	858 (519 - 1,307)	1,289 (784 - 1,965)	1,666 (1,025 - 2,530)
**Central Latin America**									
Under-5 years (rate per capita)	0.323 (0.307 - 0.343)	0.321 (0.305 - 0.342)	0.324 (0.308 - 0.345)	0.551 (0.320 - 0.774)	0.550 (0.318 - 0.777)	0.555 (0.320 - 0.785)	0.120 (0.051 - 0.239)	0.119 (0.052 - 0.236)	0.121 (0.052 - 0.243)
5-14 years (rate per capita)	0.547 (0.527 - 0.568)	0.548 (0.525 - 0.568)	0.550 (0.525 - 0.573)	0.721 (0.563 - 0.922)	0.716 (0.560 - 0.913)	0.719 (0.561 - 0.914)	0.730 (0.394 - 1.212)	0.734 (0.395 - 1.219)	0.741 (0.397 - 1.229)
15 - 49 years (rate per capita)	0.486 (0.474 - 0.497)	0.487 (0.476 - 0.500)	0.488 (0.477 - 0.501)	0.573 (0.510 - 0.641)	0.576 (0.515 - 0.640)	0.578 (0.518 - 0.644)	1.895 (1.086 - 3.097)	2.016 (1.151 - 3.273)	2.069 (1.185 - 3.355)
50 - 69 years (rate per capita)	0.588 (0.576 - 0.600)	0.584 (0.572 - 0.595)	0.584 (0.572 - 0.595)	0.628 (0.549 - 0.720)	0.628 (0.545 - 0.723)	0.626 (0.546 - 0.718)	6.866 (4.362 - 10.220)	6.704 (4.255 - 9.974)	6.767 (4.303 - 10.051)
70+ years (rate per capita)	0.635 (0.627 - 0.644)	0.633 (0.624 - 0.642)	0.629 (0.620 - 0.638)	0.582 (0.513 - 0.652)	0.581 (0.512 - 0.654)	0.577 (0.510 - 0.646)	11.250 (7.451 - 15.730)	11.183 (7.398 - 15.666)	11.086 (7.332 - 15.481)
All ages (rate per capita)	0.491 (0.483 - 0.499)	0.498 (0.490 - 0.505)	0.504 (0.496 - 0.512)	0.612 (0.546 - 0.690)	0.609 (0.550 - 0.674)	0.609 (0.555 - 0.667)	2.035 (1.220 - 3.119)	2.367 (1.423 - 3.624)	2.692 (1.631 - 4.096)
Age-standardised (rate per capita)	0.511 (0.505 - 0.518)	0.511 (0.503 - 0.517)	0.511 (0.504 - 0.518)	0.609 (0.557 - 0.667)	0.608 (0.555 - 0.662)	0.609 (0.558 - 0.667)	3.147 (1.929 - 4.769)	3.144 (1.928 - 4.774)	3.147 (1.931 - 4.771)
Total number of cases (thousands)	83,040 (81,556 - 84,395)	108,971 (107,183 - 110,637)	126,959 (124,925 - 128,885)	103,494 (92,186 - 116,642)	133,336 (120,284 - 147,606)	153,306 (139,744 - 168,033)	344 (206 - 527)	518 (311 - 793)	678 (411 - 1,031)
**Colombia**									
Under-5 years (rate per capita)	0.310 (0.264 - 0.354)	0.311 (0.261 - 0.354)	0.311 (0.266 - 0.352)	0.548 (0.307 - 0.784)	0.550 (0.308 - 0.787)	0.551 (0.309 - 0.789)	0.120 (0.051 - 0.249)	0.120 (0.050 - 0.242)	0.120 (0.051 - 0.243)
5-14 years (rate per capita)	0.516 (0.465 - 0.574)	0.521 (0.469 - 0.578)	0.520 (0.471 - 0.576)	0.667 (0.509 - 0.855)	0.663 (0.506 - 0.864)	0.663 (0.506 - 0.864)	0.701 (0.380 - 1.174)	0.716 (0.384 - 1.210)	0.715 (0.384 - 1.205)
15 - 49 years (rate per capita)	0.441 (0.418 - 0.467)	0.446 (0.426 - 0.470)	0.446 (0.425 - 0.470)	0.531 (0.475 - 0.592)	0.533 (0.478 - 0.593)	0.535 (0.480 - 0.594)	1.893 (1.092 - 3.076)	2.029 (1.162 - 3.302)	2.085 (1.203 - 3.365)
50 - 69 years (rate per capita)	0.556 (0.531 - 0.583)	0.552 (0.527 - 0.579)	0.554 (0.528 - 0.582)	0.589 (0.516 - 0.672)	0.588 (0.514 - 0.670)	0.588 (0.514 - 0.669)	6.928 (4.415 - 10.285)	6.703 (4.238 - 9.978)	6.826 (4.330 - 10.112)
70+ years (rate per capita)	0.612 (0.593 - 0.634)	0.609 (0.588 - 0.632)	0.607 (0.588 - 0.629)	0.547 (0.482 - 0.612)	0.542 (0.481 - 0.607)	0.541 (0.481 - 0.605)	11.486 (7.614 - 16.135)	11.402 (7.568 - 15.997)	11.333 (7.493 - 15.956)
All ages (rate per capita)	0.457 (0.435 - 0.475)	0.466 (0.448 - 0.483)	0.473 (0.457 - 0.490)	0.571 (0.507 - 0.643)	0.567 (0.513 - 0.627)	0.567 (0.517 - 0.622)	2.073 (1.254 - 3.177)	2.485 (1.495 - 3.808)	2.912 (1.788 - 4.405)
Age-standardised (rate per capita)	0.476 (0.459 - 0.491)	0.477 (0.460 - 0.494)	0.477 (0.460 - 0.494)	0.568 (0.518 - 0.625)	0.568 (0.518 - 0.624)	0.568 (0.518 - 0.624)	3.148 (1.954 - 4.750)	3.154 (1.956 - 4.729)	3.149 (1.956 - 4.745)

	Prevalence 1990	Prevalence 2005	Prevalence 2015	Incidence 1990	Incidence 2005	Incidence 2015	YLD rate 1990	YLD rate 2005	YLD rate 2015
Total number of cases (thousands)	15,676 (14,939 - 16,300)	20,181 (19,410 - 20,916)	22,844 (22,051 - 23,644)	19,608 (17,398 - 22,090)	24,547 (22,190 - 27,154)	27,356 (24,960 - 30,029)	71 (43 - 109)	108 (65 - 165)	141 (86 - 213)
**Costa Rica**									
Under-5 years (rate per capita)	0.308 (0.264 - 0.350)	0.311 (0.266 - 0.353)	0.311 (0.264 - 0.353)	0.546 (0.306 - 0.782)	0.551 (0.309 - 0.788)	0.551 (0.309 - 0.788)	0.119 (0.050 - 0.240)	0.120 (0.049 - 0.240)	0.120 (0.051 - 0.242)
5-14 years (rate per capita)	0.546 (0.490 - 0.604)	0.548 (0.494 - 0.605)	0.547 (0.488 - 0.605)	0.722 (0.551 - 0.926)	0.710 (0.545 - 0.906)	0.712 (0.546 - 0.909)	0.741 (0.399 - 1.238)	0.752 (0.404 - 1.271)	0.750 (0.398 - 1.262)
15 - 49 years (rate per capita)	0.487 (0.452 - 0.521)	0.492 (0.458 - 0.521)	0.491 (0.457 - 0.523)	0.596 (0.509 - 0.692)	0.596 (0.510 - 0.687)	0.598 (0.514 - 0.689)	2.018 (1.156 - 3.276)	2.108 (1.206 - 3.421)	2.137 (1.227 - 3.454)
50 - 69 years (rate per capita)	0.587 (0.553 - 0.627)	0.587 (0.554 - 0.626)	0.587 (0.553 - 0.624)	0.642 (0.530 - 0.782)	0.642 (0.531 - 0.785)	0.642 (0.531 - 0.781)	6.956 (4.402 - 10.344)	6.921 (4.385 - 10.296)	6.947 (4.381 - 10.314)
70+ years (rate per capita)	0.637 (0.611 - 0.666)	0.634 (0.610 - 0.661)	0.632 (0.606 - 0.660)	0.590 (0.497 - 0.695)	0.589 (0.497 - 0.691)	0.587 (0.497 - 0.688)	11.543 (7.663 - 16.211)	11.453 (7.583 - 16.152)	11.351 (7.513 - 15.929)
All ages (rate per capita)	0.492 (0.467 - 0.515)	0.506 (0.481 - 0.527)	0.512 (0.491 - 0.533)	0.622 (0.549 - 0.700)	0.620 (0.553 - 0.688)	0.619 (0.555 - 0.684)	2.291 (1.372 - 3.497)	2.739 (1.655 - 4.172)	3.190 (1.950 - 4.824)
Age-standardised (rate per capita)	0.510 (0.488 - 0.532)	0.511 (0.489 - 0.532)	0.511 (0.490 - 0.532)	0.620 (0.556 - 0.686)	0.620 (0.556 - 0.686)	0.620 (0.556 - 0.686)	3.206 (1.975 - 4.849)	3.205 (1.968 - 4.855)	3.201 (1.965 - 4.836)
Total number of cases (thousands)	1,523 (1,445 - 1,593)	2,152 (2,047 - 2,243)	2,461 (2,360 - 2,564)	1,926 (1,699 - 2,166)	2,635 (2,352 - 2,927)	2,978 (2,668 - 3,288)	7 (4 - 11)	12 (7 - 18)	15 (9 - 23)
**El Salvador**									
Under-5 years (rate per capita)	0.311 (0.267 - 0.353)	0.315 (0.268 - 0.358)	0.313 (0.266 - 0.356)	0.538 (0.303 - 0.767)	0.546 (0.307 - 0.779)	0.541 (0.305 - 0.772)	0.074 (0.031 - 0.148)	0.075 (0.031 - 0.150)	0.074 (0.032 - 0.149)
5-14 years (rate per capita)	0.555 (0.498 - 0.610)	0.555 (0.497 - 0.615)	0.552 (0.498 - 0.611)	0.690 (0.531 - 0.879)	0.685 (0.528 - 0.875)	0.679 (0.526 - 0.866)	0.574 (0.319 - 0.944)	0.577 (0.319 - 0.933)	0.580 (0.321 - 0.951)
15 - 49 years (rate per capita)	0.496 (0.457 - 0.527)	0.497 (0.460 - 0.527)	0.498 (0.463 - 0.529)	0.563 (0.481 - 0.653)	0.566 (0.484 - 0.655)	0.566 (0.485 - 0.657)	1.766 (1.028 - 2.817)	1.840 (1.063 - 2.923)	1.854 (1.076 - 2.951)
50 - 69 years (rate per capita)	0.589 (0.556 - 0.626)	0.592 (0.556 - 0.628)	0.591 (0.557 - 0.633)	0.618 (0.510 - 0.753)	0.618 (0.512 - 0.754)	0.618 (0.512 - 0.756)	6.692 (4.242 - 9.883)	6.869 (4.354 - 10.058)	6.866 (4.364 - 10.098)
70+ years (rate per capita)	0.644 (0.617 - 0.674)	0.640 (0.612 - 0.668)	0.634 (0.608 - 0.662)	0.573 (0.481 - 0.677)	0.571 (0.480 - 0.672)	0.567 (0.480 - 0.665)	11.402 (7.597 - 15.915)	11.277 (7.546 - 15.705)	11.176 (7.432 - 15.619)
All ages (rate per capita)	0.497 (0.472 - 0.518)	0.508 (0.484 - 0.531)	0.513 (0.489 - 0.535)	0.598 (0.523 - 0.676)	0.598 (0.531 - 0.668)	0.592 (0.531 - 0.657)	1.966 (1.195 - 2.977)	2.360 (1.450 - 3.538)	2.704 (1.684 - 4.051)
Age-standardised (rate per capita)	0.516 (0.493 - 0.536)	0.516 (0.494 - 0.538)	0.516 (0.494 - 0.538)	0.595 (0.534 - 0.656)	0.595 (0.534 - 0.656)	0.595 (0.534 - 0.656)	3.022 (1.891 - 4.493)	3.031 (1.905 - 4.497)	3.040 (1.909 - 4.533)
Total number of cases (thousands)	2,628 (2,494 - 2,739)	3,031 (2,883 - 3,166)	3,147 (2,999 - 3,285)	3,161 (2,768 - 3,576)	3,566 (3,166 - 3,982)	3,635 (3,256 - 4,031)	10 (6 - 16)	14 (9 - 21)	17 (10 - 25)
**Guatemala**									
Under-5 years (rate per capita)	0.303 (0.257 - 0.345)	0.309 (0.263 - 0.351)	0.309 (0.263 - 0.352)	0.540 (0.303 - 0.773)	0.546 (0.306 - 0.781)	0.546 (0.306 - 0.781)	0.118 (0.050 - 0.239)	0.119 (0.050 - 0.240)	0.119 (0.050 - 0.242)
5-14 years (rate per capita)	0.548 (0.493 - 0.605)	0.548 (0.495 - 0.602)	0.547 (0.492 - 0.603)	0.723 (0.552 - 0.928)	0.720 (0.550 - 0.922)	0.714 (0.547 - 0.913)	0.736 (0.397 - 1.243)	0.743 (0.396 - 1.258)	0.749 (0.400 - 1.273)
15 - 49 years (rate per capita)	0.489 (0.452 - 0.526)	0.488 (0.454 - 0.521)	0.489 (0.451 - 0.521)	0.590 (0.501 - 0.684)	0.591 (0.502 - 0.686)	0.593 (0.503 - 0.686)	1.914 (1.094 - 3.114)	1.915 (1.102 - 3.109)	1.957 (1.117 - 3.163)
50 - 69 years (rate per capita)	0.586 (0.553 - 0.629)	0.588 (0.555 - 0.624)	0.590 (0.557 - 0.630)	0.643 (0.531 - 0.785)	0.643 (0.532 - 0.783)	0.643 (0.531 - 0.783)	6.963 (4.451 - 10.269)	6.989 (4.456 - 10.377)	7.108 (4.558 - 10.461)
70+ years (rate per capita)	0.643 (0.617 - 0.670)	0.642 (0.617 - 0.673)	0.636 (0.610 - 0.662)	0.594 (0.498 - 0.702)	0.593 (0.497 - 0.700)	0.588 (0.498 - 0.690)	11.635 (7.709 - 16.299)	11.620 (7.768 - 16.263)	11.474 (7.615 - 15.992)
All ages (rate per capita)	0.485 (0.460 - 0.510)	0.490 (0.464 - 0.513)	0.494 (0.469 - 0.518)	0.624 (0.539 - 0.716)	0.623 (0.543 - 0.709)	0.620 (0.546 - 0.700)	1.887 (1.134 - 2.889)	2.047 (1.242 - 3.117)	2.214 (1.333 - 3.347)
Age-standardised (rate per capita)	0.511 (0.488 - 0.533)	0.512 (0.490 - 0.533)	0.512 (0.490 - 0.533)	0.620 (0.556 - 0.686)	0.620 (0.556 - 0.686)	0.620 (0.556 - 0.686)	3.207 (1.988 - 4.810)	3.223 (2.001 - 4.843)	3.226 (2.003 - 4.851)
Total number of cases (thousands)	4,461 (4,230 - 4,694)	6,469 (6,130 - 6,771)	8,078 (7,665 - 8,469)	5,741 (4,960 - 6,590)	8,224 (7,165 - 9,362)	10,142 (8,932 - 11,448)	17 (10 - 27)	27 (16 - 41)	36 (22 - 55)
**Honduras**									
Under-5 years (rate per capita)	0.307 (0.265 - 0.348)	0.312 (0.266 - 0.359)	0.311 (0.261 - 0.354)	0.532 (0.299 - 0.759)	0.541 (0.304 - 0.772)	0.540 (0.304 - 0.771)	0.073 (0.031 - 0.147)	0.074 (0.032 - 0.147)	0.074 (0.032 - 0.147)
5-14 years (rate per capita)	0.555 (0.499 - 0.611)	0.554 (0.496 - 0.610)	0.554 (0.498 - 0.611)	0.696 (0.534 - 0.887)	0.689 (0.530 - 0.878)	0.682 (0.527 - 0.870)	0.569 (0.312 - 0.936)	0.574 (0.318 - 0.933)	0.578 (0.322 - 0.942)
15 - 49 years (rate per capita)	0.494 (0.456 - 0.527)	0.495 (0.459 - 0.526)	0.494 (0.457 - 0.529)	0.561 (0.478 - 0.653)	0.563 (0.480 - 0.654)	0.565 (0.483 - 0.656)	1.686 (0.975 - 2.704)	1.739 (1.006 - 2.764)	1.786 (1.037 - 2.855)
50 - 69 years (rate per capita)	0.590 (0.556 - 0.630)	0.587 (0.551 - 0.623)	0.588 (0.552 - 0.625)	0.618 (0.512 - 0.755)	0.618 (0.509 - 0.750)	0.618 (0.509 - 0.752)	6.747 (4.276 - 9.951)	6.609 (4.196 - 9.757)	6.629 (4.230 - 9.765)
70+ years (rate per capita)	0.641 (0.616 - 0.671)	0.636 (0.607 - 0.663)	0.630 (0.604 - 0.658)	0.571 (0.481 - 0.672)	0.569 (0.482 - 0.667)	0.566 (0.482 - 0.661)	11.330 (7.550 - 15.826)	11.181 (7.428 - 15.563)	11.043 (7.316 - 15.432)
All ages (rate per capita)	0.489 (0.463 - 0.511)	0.497 (0.470 - 0.520)	0.502 (0.476 - 0.525)	0.599 (0.521 - 0.686)	0.598 (0.524 - 0.676)	0.593 (0.528 - 0.664)	1.671 (1.016 - 2.535)	1.873 (1.137 - 2.845)	2.151 (1.309 - 3.245)
Age-standardised (rate per capita)	0.516 (0.494 - 0.537)	0.515 (0.493 - 0.536)	0.515 (0.493 - 0.536)	0.595 (0.534 - 0.657)	0.595 (0.534 - 0.657)	0.595 (0.534 - 0.657)	3.016 (1.883 - 4.497)	3.012 (1.878 - 4.518)	3.012 (1.877 - 4.514)
Total number of cases (thousands)	2,404 (2,276 - 2,516)	3,425 (3,240 - 3,584)	4,066 (3,855 - 4,250)	2,944 (2,563 - 3,374)	4,121 (3,616 - 4,661)	4,803 (4,271 - 5,374)	8 (5 - 12)	13 (8 - 20)	17 (11 - 26)
**Mexico**									
Under-5 years (rate per capita)	0.338 (0.322 - 0.358)	0.332 (0.315 - 0.352)	0.338 (0.321 - 0.358)	0.559 (0.335 - 0.776)	0.553 (0.328 - 0.772)	0.563 (0.330 - 0.784)	0.131 (0.056 - 0.260)	0.128 (0.055 - 0.257)	0.131 (0.056 - 0.266)
5-14 years (rate per capita)	0.555 (0.536 - 0.576)	0.555 (0.534 - 0.575)	0.560 (0.537 - 0.579)	0.747 (0.587 - 0.959)	0.741 (0.583 - 0.941)	0.746 (0.591 - 0.950)	0.768 (0.411 - 1.294)	0.768 (0.414 - 1.284)	0.778 (0.417 - 1.301)
15 - 49 years (rate per capita)	0.501 (0.490 - 0.511)	0.501 (0.490 - 0.512)	0.501 (0.492 - 0.513)	0.586 (0.527 - 0.648)	0.590 (0.533 - 0.647)	0.590 (0.536 - 0.650)	1.905 (1.082 - 3.118)	2.048 (1.169 - 3.356)	2.108 (1.200 - 3.431)
50 - 69 years (rate per capita)	0.600 (0.590 - 0.611)	0.595 (0.585 - 0.606)	0.596 (0.585 - 0.605)	0.641 (0.568 - 0.727)	0.642 (0.563 - 0.726)	0.640 (0.562 - 0.722)	6.849 (4.312 - 10.223)	6.623 (4.155 - 9.894)	6.702 (4.208 - 10.017)
70+ years (rate per capita)	0.641 (0.632 - 0.649)	0.638 (0.628 - 0.647)	0.634 (0.624 - 0.642)	0.593 (0.529 - 0.660)	0.594 (0.530 - 0.659)	0.588 (0.525 - 0.655)	11.042 (7.288 - 15.463)	10.990 (7.256 - 15.420)	10.866 (7.168 - 15.193)
All ages (rate per capita)	0.505 (0.498 - 0.513)	0.509 (0.502 - 0.516)	0.516 (0.508 - 0.524)	0.628 (0.561 - 0.704)	0.624 (0.566 - 0.686)	0.623 (0.570 - 0.679)	2.060 (1.227 - 3.172)	2.381 (1.423 - 3.663)	2.693 (1.627 - 4.127)
Age-standardised (rate per capita)	0.524 (0.517 - 0.530)	0.522 (0.516 - 0.528)	0.523 (0.516 - 0.530)	0.625 (0.574 - 0.683)	0.622 (0.572 - 0.676)	0.624 (0.572 - 0.678)	3.152 (1.927 - 4.785)	3.140 (1.921 - 4.776)	3.146 (1.924 - 4.778)
Total number of cases (thousands)	43,279 (42,618 - 43,940)	55,891 (55,106 - 56,677)	65,499 (64,517 - 66,516)	53,825 (48,025 - 60,333)	68,502 (62,152 - 75,362)	79,169 (72,455 - 86,322)	176 (105 - 272)	261 (156 - 402)	342 (207 - 524)
**Nicaragua**									
Under-5 years (rate per capita)	0.309 (0.265 - 0.356)	0.311 (0.268 - 0.357)	0.313 (0.267 - 0.356)	0.537 (0.302 - 0.766)	0.540 (0.304 - 0.770)	0.542 (0.305 - 0.773)	0.074 (0.032 - 0.150)	0.074 (0.032 - 0.147)	0.074 (0.032 - 0.146)
5-14 years (rate per capita)	0.556 (0.500 - 0.613)	0.554 (0.496 - 0.613)	0.554 (0.496 - 0.609)	0.696 (0.534 - 0.887)	0.685 (0.528 - 0.875)	0.687 (0.529 - 0.877)	0.571 (0.320 - 0.938)	0.577 (0.320 - 0.939)	0.577 (0.324 - 0.954)
15 - 49 years (rate per capita)	0.494 (0.454 - 0.527)	0.494 (0.458 - 0.526)	0.494 (0.459 - 0.528)	0.561 (0.478 - 0.651)	0.564 (0.481 - 0.654)	0.567 (0.486 - 0.659)	1.696 (0.975 - 2.719)	1.767 (1.022 - 2.810)	1.848 (1.071 - 2.946)
50 - 69 years (rate per capita)	0.591 (0.557 - 0.633)	0.589 (0.556 - 0.629)	0.588 (0.551 - 0.624)	0.618 (0.511 - 0.756)	0.618 (0.510 - 0.750)	0.619 (0.508 - 0.761)	6.853 (4.358 - 10.112)	6.730 (4.283 - 9.959)	6.696 (4.266 - 9.877)
70+ years (rate per capita)	0.646 (0.617 - 0.676)	0.638 (0.612 - 0.667)	0.633 (0.605 - 0.660)	0.574 (0.481 - 0.678)	0.569 (0.481 - 0.669)	0.566 (0.481 - 0.663)	11.585 (7.666 - 16.267)	11.363 (7.533 - 15.925)	11.209 (7.418 - 15.662)
All ages (rate per capita)	0.490 (0.463 - 0.514)	0.499 (0.474 - 0.522)	0.504 (0.477 - 0.526)	0.601 (0.523 - 0.689)	0.595 (0.526 - 0.669)	0.595 (0.530 - 0.663)	1.674 (1.012 - 2.545)	1.977 (1.199 - 2.999)	2.309 (1.415 - 3.509)
Age-standardised (rate per capita)	0.516 (0.493 - 0.537)	0.516 (0.494 - 0.538)	0.516 (0.492 - 0.537)	0.595 (0.534 - 0.657)	0.595 (0.534 - 0.657)	0.595 (0.534 - 0.657)	3.052 (1.913 - 4.565)	3.051 (1.899 - 4.567)	3.051 (1.908 - 4.565)
Total number of cases (thousands)	2,039 (1,923 - 2,137)	2,688 (2,551 - 2,810)	3,068 (2,905 - 3,204)	2,497 (2,172 - 2,866)	3,205 (2,834 - 3,605)	3,621 (3,229 - 4,039)	7 (4 - 11)	11 (6 - 16)	14 (9 - 21)
**Panama**									
Under-5 years (rate per capita)	0.310 (0.264 - 0.349)	0.309 (0.262 - 0.354)	0.310 (0.264 - 0.354)	0.537 (0.302 - 0.766)	0.538 (0.303 - 0.767)	0.538 (0.303 - 0.768)	0.074 (0.031 - 0.144)	0.074 (0.032 - 0.147)	0.074 (0.032 - 0.149)
5-14 years (rate per capita)	0.604 (0.550 - 0.661)	0.595 (0.538 - 0.653)	0.595 (0.539 - 0.654)	0.702 (0.555 - 0.884)	0.697 (0.545 - 0.887)	0.695 (0.544 - 0.882)	0.617 (0.338 - 1.025)	0.611 (0.337 - 1.012)	0.612 (0.338 - 1.008)
15 - 49 years (rate per capita)	0.534 (0.512 - 0.562)	0.532 (0.510 - 0.557)	0.534 (0.513 - 0.559)	0.540 (0.487 - 0.598)	0.545 (0.494 - 0.600)	0.546 (0.496 - 0.600)	1.801 (1.042 - 2.903)	1.924 (1.116 - 3.072)	1.978 (1.144 - 3.183)
50 - 69 years (rate per capita)	0.617 (0.590 - 0.643)	0.619 (0.590 - 0.647)	0.618 (0.590 - 0.647)	0.615 (0.546 - 0.699)	0.616 (0.546 - 0.697)	0.616 (0.546 - 0.697)	6.800 (4.284 - 10.079)	6.783 (4.299 - 10.079)	6.765 (4.274 - 10.013)
70+ years (rate per capita)	0.662 (0.643 - 0.684)	0.659 (0.640 - 0.681)	0.657 (0.639 - 0.678)	0.577 (0.514 - 0.643)	0.575 (0.513 - 0.641)	0.574 (0.512 - 0.639)	11.319 (7.521 - 15.830)	11.163 (7.410 - 15.713)	11.104 (7.389 - 15.605)
All ages (rate per capita)	0.534 (0.515 - 0.554)	0.537 (0.517 - 0.554)	0.543 (0.524 - 0.559)	0.586 (0.526 - 0.652)	0.584 (0.532 - 0.641)	0.584 (0.534 - 0.635)	2.089 (1.269 - 3.169)	2.440 (1.492 - 3.688)	2.752 (1.690 - 4.150)
Age-standardised (rate per capita)	0.550 (0.533 - 0.567)	0.547 (0.528 - 0.563)	0.547 (0.530 - 0.563)	0.586 (0.538 - 0.637)	0.585 (0.537 - 0.635)	0.585 (0.537 - 0.634)	3.062 (1.901 - 4.606)	3.059 (1.904 - 4.576)	3.056 (1.893 - 4.554)
Total number of cases (thousands)	1,320 (1,273 - 1,371)	1,784 (1,718 - 1,839)	2,132 (2,060 - 2,197)	1,448 (1,302 - 1,613)	1,942 (1,767 - 2,130)	2,294 (2,097 - 2,496)	5 (3 - 8)	8 (5 - 12)	11 (7 - 16)
**Venezuela**									
Under-5 years (rate per capita)	0.308 (0.264 - 0.348)	0.308 (0.266 - 0.352)	0.310 (0.260 - 0.356)	0.546 (0.306 - 0.781)	0.547 (0.307 - 0.783)	0.549 (0.308 - 0.785)	0.119 (0.050 - 0.248)	0.119 (0.051 - 0.243)	0.120 (0.050 - 0.244)
5-14 years (rate per capita)	0.548 (0.496 - 0.602)	0.548 (0.495 - 0.604)	0.547 (0.487 - 0.610)	0.719 (0.549 - 0.921)	0.713 (0.546 - 0.910)	0.714 (0.547 - 0.912)	0.745 (0.399 - 1.248)	0.751 (0.405 - 1.275)	0.751 (0.400 - 1.272)
15 - 49 years (rate per capita)	0.487 (0.450 - 0.518)	0.491 (0.456 - 0.521)	0.490 (0.457 - 0.519)	0.594 (0.505 - 0.689)	0.595 (0.508 - 0.687)	0.597 (0.511 - 0.689)	1.953 (1.112 - 3.191)	2.055 (1.170 - 3.335)	2.098 (1.200 - 3.404)
50 - 69 years (rate per capita)	0.586 (0.554 - 0.625)	0.585 (0.549 - 0.625)	0.584 (0.550 - 0.623)	0.642 (0.531 - 0.783)	0.642 (0.531 - 0.783)	0.642 (0.530 - 0.784)	6.863 (4.367 - 10.223)	6.833 (4.355 - 10.178)	6.780 (4.283 - 10.102)
70+ years (rate per capita)	0.638 (0.610 - 0.666)	0.635 (0.609 - 0.661)	0.635 (0.610 - 0.661)	0.592 (0.496 - 0.700)	0.590 (0.496 - 0.694)	0.589 (0.496 - 0.692)	11.503 (7.587 - 16.075)	11.418 (7.570 - 16.006)	11.388 (7.510 - 15.932)
All ages (rate per capita)	0.489 (0.462 - 0.511)	0.499 (0.474 - 0.520)	0.504 (0.480 - 0.527)	0.622 (0.546 - 0.703)	0.620 (0.548 - 0.693)	0.621 (0.551 - 0.689)	2.068 (1.239 - 3.179)	2.410 (1.445 - 3.681)	2.729 (1.655 - 4.169)
Age-standardised (rate per capita)	0.511 (0.488 - 0.532)	0.511 (0.489 - 0.532)	0.511 (0.489 - 0.533)	0.620 (0.556 - 0.686)	0.620 (0.556 - 0.686)	0.620 (0.556 - 0.686)	3.191 (1.966 - 4.821)	3.190 (1.966 - 4.825)	3.189 (1.969 - 4.809)
Total number of cases (thousands)	9,707 (9,177 - 10,145)	13,350 (12,699 - 13,925)	15,664 (14,934 - 16,383)	12,344 (10,838 - 13,965)	16,595 (14,680 - 18,546)	19,307 (17,151 - 21,425)	41 (25 - 63)	65 (39 - 99)	85 (51 - 130)

	Prevalence 1990	Prevalence 2005	Prevalence 2015	Incidence 1990	Incidence 2005	Incidence 2015	YLD rate 1990	YLD rate 2005	YLD rate 2015
**Andean Latin America**									
**Under-5 years (rate per capita)**	0.409<br>(0.366 - 0.459)	0.412<br>(0.370 - 0.456)	0.413<br>(0.371 - 0.454)	0.569<br>(0.382 - 0.741)	0.574<br>(0.386 - 0.748)	0.574<br>(0.386 - 0.748)	0.097<br>(0.041 - 0.192)	0.098<br>(0.041 - 0.194)	0.098<br>(0.041 - 0.191)
**5-14 years (rate per capita)**	0.693<br>(0.662 - 0.723)	0.693<br>(0.663 - 0.722)	0.694<br>(0.666 - 0.724)	0.812<br>(0.662 - 1.002)	0.808<br>(0.659 - 0.995)	0.808<br>(0.660 - 0.996)	0.678<br>(0.369 - 1.125)	0.681<br>(0.367 - 1.126)	0.681<br>(0.365 - 1.128)
**15 - 49 years (rate per capita)**	0.665<br>(0.649 - 0.680)	0.666<br>(0.650 - 0.682)	0.666<br>(0.652 - 0.681)	0.677<br>(0.624 - 0.737)	0.682<br>(0.629 - 0.740)	0.686<br>(0.633 - 0.744)	1.883<br>(1.081 - 3.040)	1.963<br>(1.125 - 3.161)	2.023<br>(1.160 - 3.257)
**50 - 69 years (rate per capita)**	0.723<br>(0.706 - 0.738)	0.723<br>(0.705 - 0.740)	0.723<br>(0.706 - 0.740)	0.821<br>(0.741 - 0.903)	0.821<br>(0.740 - 0.902)	0.821<br>(0.741 - 0.902)	7.051<br>(4.460 - 10.424)	7.033<br>(4.433 - 10.439)	7.045<br>(4.442 - 10.399)
**70+ years (rate per capita)**	0.759<br>(0.746 - 0.771)	0.753<br>(0.742 - 0.766)	0.748<br>(0.737 - 0.760)	0.817<br>(0.738 - 0.885)	0.816<br>(0.741 - 0.882)	0.816<br>(0.742 - 0.880)	11.844<br>(7.905 - 16.716)	11.713<br>(7.786 - 16.525)	11.593<br>(7.730 - 16.394)
**All ages (rate per capita)**	0.643<br>(0.630 - 0.656)	0.652<br>(0.640 - 0.664)	0.657<br>(0.647 - 0.668)	0.712<br>(0.656 - 0.777)	0.717<br>(0.666 - 0.776)	0.722<br>(0.675 - 0.779)	2.068<br>(1.241 - 3.164)	2.399<br>(1.453 - 3.646)	2.686<br>(1.640 - 4.058)
**Age-standardised (rate per capita)**	0.664<br>(0.653 - 0.675)	0.664<br>(0.654 - 0.675)	0.664<br>(0.655 - 0.674)	0.731<br>(0.686 - 0.782)	0.731<br>(0.686 - 0.782)	0.731<br>(0.686 - 0.782)	3.195<br>(1.982 - 4.797)	3.198<br>(1.986 - 4.794)	3.198<br>(1.978 - 4.798)
**Total number of cases (thousands)**	25,069<br>(24,544 - 25,574)	32,994<br>(32,390 - 33,578)	38,325<br>(37,709 - 38,956)	27,760<br>(25,554 - 30,297)	36,270<br>(33,672 - 39,245)	42,107<br>(39,376 - 45,404)	81<br>(48 - 123)	121<br>(73 - 184)	157<br>(96 - 237)
**Bolivia**									
**Under-5 years (rate per capita)**	0.408<br>(0.339 - 0.473)	0.413<br>(0.349 - 0.479)	0.414<br>(0.349 - 0.483)	0.567<br>(0.382 - 0.740)	0.574<br>(0.387 - 0.749)	0.575<br>(0.388 - 0.750)	0.096<br>(0.041 - 0.187)	0.098<br>(0.041 - 0.192)	0.098<br>(0.041 - 0.193)
**5-14 years (rate per capita)**	0.690<br>(0.649 - 0.729)	0.693<br>(0.649 - 0.734)	0.693<br>(0.651 - 0.734)	0.818<br>(0.664 - 1.013)	0.814<br>(0.661 - 1.007)	0.809<br>(0.658 - 1.000)	0.678<br>(0.364 - 1.130)	0.679<br>(0.368 - 1.126)	0.681<br>(0.370 - 1.144)
**15 - 49 years (rate per capita)**	0.665<br>(0.644 - 0.687)	0.665<br>(0.644 - 0.690)	0.665<br>(0.645 - 0.689)	0.676<br>(0.624 - 0.737)	0.678<br>(0.626 - 0.739)	0.681<br>(0.629 - 0.742)	1.888<br>(1.085 - 3.077)	1.913<br>(1.093 - 3.095)	1.956<br>(1.119 - 3.166)
**50 - 69 years (rate per capita)**	0.725<br>(0.702 - 0.748)	0.723<br>(0.697 - 0.747)	0.724<br>(0.701 - 0.746)	0.822<br>(0.741 - 0.902)	0.821<br>(0.739 - 0.904)	0.822<br>(0.739 - 0.904)	7.153<br>(4.523 - 10.585)	7.043<br>(4.453 - 10.459)	7.081<br>(4.475 - 10.464)
**70+ years (rate per capita)**	0.764<br>(0.744 - 0.781)	0.755<br>(0.736 - 0.773)	0.746<br>(0.728 - 0.763)	0.817<br>(0.738 - 0.889)	0.816<br>(0.741 - 0.884)	0.816<br>(0.746 - 0.879)	11.873<br>(7.909 - 16.677)	11.692<br>(7.792 - 16.374)	11.483<br>(7.653 - 16.152)
**All ages (rate per capita)**	0.640<br>(0.620 - 0.656)	0.647<br>(0.628 - 0.665)	0.653<br>(0.636 - 0.669)	0.713<br>(0.652 - 0.783)	0.716<br>(0.660 - 0.780)	0.719<br>(0.668 - 0.779)	2.052<br>(1.230 - 3.132)	2.260<br>(1.362 - 3.433)	2.489<br>(1.517 - 3.769)
**Age-standardised (rate per capita)**	0.664<br>(0.649 - 0.678)	0.664<br>(0.648 - 0.679)	0.664<br>(0.649 - 0.679)	0.731<br>(0.685 - 0.783)	0.731<br>(0.685 - 0.783)	0.731<br>(0.685 - 0.782)	3.182<br>(1.969 - 4.756)	3.188<br>(1.973 - 4.779)	3.184<br>(1.975 - 4.759)
**Total number of cases (thousands)**	4,407<br>(4,273 - 4,521)	5,932<br>(5,760 - 6,093)	7,031<br>(6,851 - 7,201)	4,914<br>(4,494 - 5,392)	6,567<br>(6,056 - 7,148)	7,738<br>(7,191 - 8,386)	14<br>(8 - 22)	21<br>(12 - 31)	27<br>(16 - 41)
**Ecuador**									
**Under-5 years (rate per capita)**	0.406<br>(0.348 - 0.469)	0.409<br>(0.346 - 0.472)	0.408<br>(0.350 - 0.472)	0.568<br>(0.379 - 0.737)	0.571<br>(0.381 - 0.742)	0.572<br>(0.381 - 0.743)	0.096<br>(0.041 - 0.192)	0.097<br>(0.041 - 0.195)	0.097<br>(0.041 - 0.193)
**5-14 years (rate per capita)**	0.697<br>(0.654 - 0.737)	0.696<br>(0.655 - 0.737)	0.698<br>(0.657 - 0.737)	0.810<br>(0.668 - 0.992)	0.805<br>(0.665 - 0.986)	0.806<br>(0.665 - 0.987)	0.679<br>(0.367 - 1.136)	0.682<br>(0.367 - 1.141)	0.682<br>(0.365 - 1.132)
**15 - 49 years (rate per capita)**	0.665<br>(0.643 - 0.687)	0.667<br>(0.647 - 0.688)	0.666<br>(0.647 - 0.686)	0.677<br>(0.624 - 0.737)	0.682<br>(0.630 - 0.739)	0.685<br>(0.633 - 0.742)	1.879<br>(1.073 - 3.030)	1.973<br>(1.125 - 3.190)	2.018<br>(1.153 - 3.256)
**50 - 69 years (rate per capita)**	0.723<br>(0.702 - 0.744)	0.722<br>(0.698 - 0.743)	0.722<br>(0.697 - 0.746)	0.820<br>(0.736 - 0.901)	0.819<br>(0.734 - 0.901)	0.820<br>(0.736 - 0.901)	7.019<br>(4.437 - 10.400)	6.951<br>(4.405 - 10.325)	7.002<br>(4.439 - 10.369)
**70+ years (rate per capita)**	0.755<br>(0.738 - 0.773)	0.748<br>(0.732 - 0.764)	0.748<br>(0.732 - 0.766)	0.818<br>(0.738 - 0.888)	0.817<br>(0.742 - 0.884)	0.817<br>(0.741 - 0.884)	11.730<br>(7.772 - 16.470)	11.522<br>(7.671 - 16.154)	11.537<br>(7.691 - 16.195)
**All ages (rate per capita)**	0.644<br>(0.625 - 0.660)	0.653<br>(0.637 - 0.669)	0.658<br>(0.642 - 0.672)	0.711<br>(0.654 - 0.775)	0.717<br>(0.665 - 0.776)	0.722<br>(0.673 - 0.776)	2.070<br>(1.247 - 3.170)	2.399<br>(1.448 - 3.642)	2.692<br>(1.650 - 4.088)
**Age-standardised (rate per capita)**	0.665<br>(0.650 - 0.678)	0.665<br>(0.650 - 0.679)	0.664<br>(0.650 - 0.678)	0.730<br>(0.683 - 0.781)	0.730<br>(0.683 - 0.781)	0.730<br>(0.683 - 0.781)	3.185<br>(1.970 - 4.761)	3.185<br>(1.969 - 4.741)	3.185<br>(1.975 - 4.758)
**Total number of cases (thousands)**	6,595<br>(6,403 - 6,757)	8,987<br>(8,756 - 9,204)	10,622<br>(10,368 - 10,860)	7,283<br>(6,700 - 7,934)	9,861<br>(9,146 - 10,673)	11,660<br>(10,869 - 12,540)	21<br>(13 - 32)	33<br>(20 - 50)	43<br>(27 - 66)
**Peru**									
**Under-5 years (rate per capita)**	0.411<br>(0.347 - 0.478)	0.414<br>(0.350 - 0.478)	0.415<br>(0.350 - 0.479)	0.570<br>(0.384 - 0.744)	0.576<br>(0.388 - 0.752)	0.576<br>(0.388 - 0.751)	0.097<br>(0.041 - 0.195)	0.099<br>(0.041 - 0.196)	0.098<br>(0.042 - 0.194)
**5-14 years (rate per capita)**	0.692<br>(0.647 - 0.733)	0.692<br>(0.648 - 0.731)	0.692<br>(0.649 - 0.734)	0.812<br>(0.659 - 1.004)	0.806<br>(0.656 - 0.996)	0.810<br>(0.658 - 1.001)	0.678<br>(0.367 - 1.124)	0.682<br>(0.364 - 1.124)	0.681<br>(0.365 - 1.135)
**15 - 49 years (rate per capita)**	0.664<br>(0.642 - 0.687)	0.666<br>(0.644 - 0.689)	0.667<br>(0.646 - 0.688)	0.677<br>(0.624 - 0.738)	0.682<br>(0.630 - 0.742)	0.687<br>(0.635 - 0.746)	1.883<br>(1.084 - 3.039)	1.974<br>(1.134 - 3.171)	2.047<br>(1.177 - 3.305)
**50 - 69 years (rate per capita)**	0.722<br>(0.698 - 0.745)	0.723<br>(0.697 - 0.745)	0.722<br>(0.699 - 0.746)	0.821<br>(0.739 - 0.906)	0.821<br>(0.739 - 0.905)	0.821<br>(0.738 - 0.905)	7.033<br>(4.459 - 10.385)	7.070<br>(4.437 - 10.481)	7.056<br>(4.438 - 10.400)
**70+ years (rate per capita)**	0.759<br>(0.742 - 0.777)	0.756<br>(0.739 - 0.774)	0.749<br>(0.731 - 0.766)	0.816<br>(0.740 - 0.885)	0.816<br>(0.740 - 0.883)	0.816<br>(0.744 - 0.879)	11.893<br>(7.915 - 16.840)	11.814<br>(7.842 - 16.711)	11.659<br>(7.762 - 16.497)
**All ages (rate per capita)**	0.644<br>(0.624 - 0.662)	0.654<br>(0.636 - 0.670)	0.658<br>(0.643 - 0.674)	0.712<br>(0.655 - 0.778)	0.718<br>(0.667 - 0.776)	0.723<br>(0.676 - 0.778)	2.072<br>(1.245 - 3.175)	2.446<br>(1.480 - 3.711)	2.750<br>(1.676 - 4.163)
**Age-standardised (rate per capita)**	0.664<br>(0.648 - 0.678)	0.664<br>(0.648 - 0.679)	0.664<br>(0.650 - 0.679)	0.731<br>(0.685 - 0.782)	0.731<br>(0.685 - 0.782)	0.731<br>(0.684 - 0.783)	3.203<br>(1.994 - 4.814)	3.206<br>(1.992 - 4.818)	3.208<br>(1.982 - 4.813)
**Total number of cases (thousands)**	14,066<br>(13,632 - 14,454)	18,075<br>(17,583 - 18,518)	20,672<br>(20,180 - 21,159)	15,563<br>(14,307 - 16,987)	19,842<br>(18,448 - 21,469)	22,709<br>(21,209 - 24,424)	45<br>(27 - 69)	68<br>(41 - 103)	86<br>(53 - 131)
**Caribbean**									
**Under-5 years (rate per capita)**	0.435<br>(0.399 - 0.472)	0.438<br>(0.401 - 0.475)	0.439<br>(0.404 - 0.475)	0.586<br>(0.403 - 0.753)	0.588<br>(0.405 - 0.756)	0.588<br>(0.405 - 0.755)	0.125<br>(0.054 - 0.243)	0.122<br>(0.052 - 0.241)	0.121<br>(0.052 - 0.237)
**5-14 years (rate per capita)**	0.567<br>(0.539 - 0.594)	0.563<br>(0.535 - 0.593)	0.562<br>(0.532 - 0.592)	0.837<br>(0.667 - 1.045)	0.831<br>(0.661 - 1.033)	0.829<br>(0.660 - 1.031)	0.623<br>(0.342 - 1.021)	0.616<br>(0.337 - 1.015)	0.609<br>(0.335 - 1.003)
**15 - 49 years (rate per capita)**	0.470<br>(0.453 - 0.485)	0.475<br>(0.459 - 0.490)	0.476<br>(0.459 - 0.491)	0.842<br>(0.737 - 0.947)	0.843<br>(0.743 - 0.945)	0.842<br>(0.742 - 0.948)	1.762<br>(1.003 - 2.851)	1.865<br>(1.068 - 3.021)	1.885<br>(1.076 - 3.047)
**50 - 69 years (rate per capita)**	0.589<br>(0.570 - 0.605)	0.588<br>(0.572 - 0.605)	0.587<br>(0.570 - 0.604)	0.925<br>(0.787 - 1.064)	0.924<br>(0.787 - 1.063)	0.923<br>(0.787 - 1.060)	6.322<br>(3.940 - 9.432)	6.294<br>(3.937 - 9.393)	6.214<br>(3.879 - 9.287)
**70+ years (rate per capita)**	0.627<br>(0.613 - 0.642)	0.625<br>(0.612 - 0.639)	0.623<br>(0.608 - 0.637)	0.905<br>(0.769 - 1.035)	0.900<br>(0.767 - 1.025)	0.897<br>(0.766 - 1.021)	10.273<br>(6.782 - 14.466)	10.185<br>(6.720 - 14.385)	10.115<br>(6.692 - 14.218)
**All ages (rate per capita)**	0.507<br>(0.495 - 0.519)	0.512<br>(0.502 - 0.523)	0.516<br>(0.505 - 0.526)	0.823<br>(0.752 - 0.896)	0.831<br>(0.763 - 0.900)	0.836<br>(0.772 - 0.901)	2.237<br>(1.352 - 3.393)	2.516<br>(1.530 - 3.825)	2.785<br>(1.705 - 4.242)
**Age-standardised (rate per capita)**	0.517<br>(0.507 - 0.527)	0.517<br>(0.507 - 0.528)	0.517<br>(0.507 - 0.528)	0.839<br>(0.774 - 0.904)	0.837<br>(0.772 - 0.902)	0.836<br>(0.771 - 0.901)	2.862<br>(1.753 - 4.327)	2.855<br>(1.757 - 4.319)	2.843<br>(1.749 - 4.308)
**Total number of cases (thousands)**	18,123<br>(17,712 - 18,556)	21,416<br>(20,998 - 21,896)	23,336<br>(22,861 - 23,813)	29,419<br>(26,896 - 32,019)	34,764<br>(31,923 - 37,642)	37,828<br>(34,923 - 40,773)	80<br>(48 - 121)	105<br>(64 - 160)	126<br>(77 - 192)
**Antigua and Barbuda**									
**Under-5 years (rate per capita)**	0.437<br>(0.374 - 0.502)	0.436<br>(0.367 - 0.503)	0.435<br>(0.363 - 0.498)	0.603<br>(0.413 - 0.777)	0.603<br>(0.412 - 0.776)	0.601<br>(0.411 - 0.774)	0.169<br>(0.071 - 0.344)	0.169<br>(0.070 - 0.340)	0.168<br>(0.069 - 0.341)
**5-14 years (rate per capita)**	0.577<br>(0.526 - 0.631)	0.561<br>(0.511 - 0.615)	0.556<br>(0.501 - 0.610)	0.868<br>(0.687 - 1.088)	0.853<br>(0.680 - 1.069)	0.849<br>(0.677 - 1.064)	0.730<br>(0.391 - 1.216)	0.732<br>(0.390 - 1.231)	0.731<br>(0.391 - 1.234)
**15 - 49 years (rate per capita)**	0.467<br>(0.437 - 0.495)	0.475<br>(0.447 - 0.506)	0.476<br>(0.450 - 0.504)	0.863<br>(0.754 - 0.973)	0.868<br>(0.762 - 0.973)	0.867<br>(0.762 - 0.968)	1.860<br>(1.054 - 3.098)	2.037<br>(1.166 - 3.319)	2.073<br>(1.186 - 3.398)
**50 - 69 years (rate per capita)**	0.593<br>(0.566 - 0.623)	0.586<br>(0.560 - 0.617)	0.583<br>(0.552 - 0.615)	0.947<br>(0.803 - 1.090)	0.940<br>(0.803 - 1.083)	0.937<br>(0.790 - 1.082)	6.725<br>(4.242 - 10.008)	6.337<br>(3.965 - 9.463)	6.060<br>(3.811 - 9.150)
**70+ years (rate per capita)**	0.630<br>(0.605 - 0.656)	0.617<br>(0.595 - 0.641)	0.625<br>(0.600 - 0.651)	0.923<br>(0.782 - 1.058)	0.904<br>(0.774 - 1.028)	0.915<br>(0.778 - 1.044)	10.452<br>(6.880 - 14.758)	10.087<br>(6.671 - 14.188)	10.279<br>(6.828 - 14.435)
**All ages (rate per capita)**	0.509<br>(0.487 - 0.528)	0.508<br>(0.489 - 0.527)	0.512<br>(0.496 - 0.532)	0.852<br>(0.778 - 0.927)	0.851<br>(0.781 - 0.926)	0.857<br>(0.788 - 0.928)	2.521<br>(1.525 - 3.828)	2.539<br>(1.533 - 3.880)	2.800<br>(1.690 - 4.284)
**Age-standardised (rate per capita)**	0.515<br>(0.496 - 0.533)	0.515<br>(0.497 - 0.533)	0.515<br>(0.498 - 0.535)	0.857<br>(0.790 - 0.927)	0.857<br>(0.790 - 0.927)	0.857<br>(0.790 - 0.927)	2.953<br>(1.795 - 4.496)	2.939<br>(1.793 - 4.451)	2.942<br>(1.798 - 4.478)
**Total number of cases (thousands)**	31<br>(30 - 32)	42<br>(40 - 44)	47<br>(46 - 49)	52<br>(48 - 57)	70<br>(64 - 76)	79<br>(72 - 85)	0<br>(0 - 0)	0<br>(0 - 0)	0<br>(0 - 0)
**The Bahamas**									
**Under-5 years (rate per capita)**	0.437<br>(0.368 - 0.501)	0.440<br>(0.370 - 0.506)	0.438<br>(0.370 - 0.505)	0.580<br>(0.400 - 0.745)	0.583<br>(0.403 - 0.749)	0.581<br>(0.401 - 0.746)	0.104<br>(0.043 - 0.206)	0.104<br>(0.045 - 0.201)	0.104<br>(0.044 - 0.206)
**5-14 years (rate per capita)**	0.572<br>(0.518 - 0.628)	0.562<br>(0.511 - 0.617)	0.569<br>(0.517 - 0.624)	0.829<br>(0.661 - 1.037)	0.818<br>(0.653 - 1.016)	0.827<br>(0.658 - 1.033)	0.564<br>(0.315 - 0.921)	0.566<br>(0.316 - 0.922)	0.565<br>(0.313 - 0.921)
**15 - 49 years (rate per capita)**	0.471<br>(0.441 - 0.499)	0.479<br>(0.449 - 0.507)	0.480<br>(0.453 - 0.508)	0.824<br>(0.722 - 0.930)	0.829<br>(0.732 - 0.932)	0.830<br>(0.732 - 0.936)	1.675<br>(0.961 - 2.711)	1.835<br>(1.052 - 2.989)	1.850<br>(1.054 - 2.979)
**50 - 69 years (rate per capita)**	0.587<br>(0.557 - 0.623)	0.588<br>(0.557 - 0.621)	0.588<br>(0.559 - 0.620)	0.902<br>(0.769 - 1.035)	0.902<br>(0.768 - 1.035)	0.903<br>(0.767 - 1.035)	6.030<br>(3.781 - 9.027)	6.029<br>(3.795 - 8.991)	6.058<br>(3.821 - 8.985)
**70+ years (rate per capita)**	0.635<br>(0.609 - 0.663)	0.631<br>(0.606 - 0.661)	0.630<br>(0.604 - 0.656)	0.891<br>(0.755 - 1.025)	0.888<br>(0.755 - 1.018)	0.885<br>(0.755 - 1.014)	10.307<br>(6.819 - 14.461)	10.192<br>(6.764 - 14.340)	10.147<br>(6.765 - 14.270)
**All ages (rate per capita)**	0.505<br>(0.483 - 0.526)	0.513<br>(0.492 - 0.532)	0.518<br>(0.500 - 0.536)	0.807<br>(0.739 - 0.878)	0.822<br>(0.757 - 0.889)	0.828<br>(0.764 - 0.893)	1.940<br>(1.167 - 2.950)	2.444<br>(1.483 - 3.719)	2.807<br>(1.728 - 4.248)
**Age-standardised (rate per capita)**	0.518<br>(0.500 - 0.537)	0.520<br>(0.499 - 0.539)	0.520<br>(0.502 - 0.538)	0.822<br>(0.759 - 0.887)	0.822<br>(0.759 - 0.887)	0.822<br>(0.759 - 0.887)	2.782<br>(1.726 - 4.167)	2.783<br>(1.722 - 4.186)	2.779<br>(1.720 - 4.169)
**Total number of cases (thousands)**	130<br>(124 - 135)	167<br>(161 - 174)	200<br>(193 - 208)	208<br>(190 - 226)	268<br>(247 - 290)	321<br>(296 - 346)	0<br>(0 - 1)	1<br>(0 - 1)	1<br>(1 - 2)
**Barbados**									
**Under-5 years (rate per capita)**	0.438<br>(0.376 - 0.499)	0.437<br>(0.372 - 0.502)	0.437<br>(0.370 - 0.502)	0.604<br>(0.413 - 0.777)	0.604<br>(0.413 - 0.777)	0.603<br>(0.412 - 0.776)	0.169<br>(0.071 - 0.341)	0.169<br>(0.070 - 0.341)	0.169<br>(0.070 - 0.343)
**5-14 years (rate per capita)**	0.560<br>(0.510 - 0.616)	0.556<br>(0.504 - 0.610)	0.558<br>(0.509 - 0.612)	0.851<br>(0.679 - 1.067)	0.850<br>(0.677 - 1.064)	0.850<br>(0.678 - 1.065)	0.730<br>(0.389 - 1.234)	0.731<br>(0.392 - 1.243)	0.730<br>(0.389 - 1.234)
**15 - 49 years (rate per capita)**	0.468<br>(0.439 - 0.497)	0.478<br>(0.450 - 0.504)	0.476<br>(0.449 - 0.504)	0.864<br>(0.757 - 0.970)	0.870<br>(0.768 - 0.974)	0.870<br>(0.768 - 0.974)	1.876<br>(1.065 - 3.088)	2.106<br>(1.201 - 3.453)	2.102<br>(1.203 - 3.424)
**50 - 69 years (rate per capita)**	0.592<br>(0.565 - 0.624)	0.586<br>(0.558 - 0.617)	0.588<br>(0.560 - 0.621)	0.946<br>(0.806 - 1.087)	0.940<br>(0.801 - 1.083)	0.943<br>(0.798 - 1.088)	6.650<br>(4.197 - 9.911)	6.282<br>(3.955 - 9.384)	6.417<br>(4.058 - 9.580)
**70+ years (rate per capita)**	0.627<br>(0.602 - 0.654)	0.621<br>(0.597 - 0.647)	0.621<br>(0.596 - 0.645)	0.917<br>(0.779 - 1.046)	0.909<br>(0.775 - 1.033)	0.909<br>(0.775 - 1.034)	10.361<br>(6.852 - 14.597)	10.169<br>(6.752 - 14.383)	10.133<br>(6.725 - 14.204)
**All ages (rate per capita)**	0.508<br>(0.489 - 0.529)	0.518<br>(0.501 - 0.537)	0.526<br>(0.510 - 0.545)	0.856<br>(0.783 - 0.927)	0.866<br>(0.800 - 0.936)	0.873<br>(0.807 - 0.939)	2.788<br>(1.703 - 4.230)	3.240<br>(1.991 - 4.956)	3.625<br>(2.248 - 5.484)
**Age-standardised (rate per capita)**	0.515<br>(0.497 - 0.535)	0.515<br>(0.497 - 0.534)	0.515<br>(0.497 - 0.535)	0.857<br>(0.790 - 0.928)	0.857<br>(0.790 - 0.928)	0.857<br>(0.790 - 0.928)	2.951<br>(1.806 - 4.506)	2.939<br>(1.800 - 4.510)	2.934<br>(1.794 - 4.465)
**Total number of cases (thousands)**	132<br>(127 - 138)	142<br>(137 - 147)	149<br>(145 - 155)	223<br>(204 - 242)	237<br>(219 - 256)	248<br>(229 - 266)	1<br>(0 - 1)	1<br>(1 - 1)	1<br>(1 - 2)
**Belize**									

	Prevalence 1990	Prevalence 2005	Prevalence 2015	Incidence 1990	Incidence 2005	Incidence 2015	YLD rate 1990	YLD rate 2005	YLD rate 2015
Under-5 years (rate per capita)	0.436 (0.369 - 0.501)	0.438 (0.369 - 0.504)	0.435 (0.367 - 0.501)	0.578 (0.399 - 0.743)	0.581 (0.401 - 0.746)	0.577 (0.399 - 0.742)	0.103 (0.044 - 0.205)	0.104 (0.045 - 0.206)	0.103 (0.043 - 0.200)
5-14 years (rate per capita)	0.574 (0.517 - 0.630)	0.572 (0.518 - 0.628)	0.563 (0.509 - 0.620)	0.831 (0.662 - 1.038)	0.828 (0.659 - 1.035)	0.820 (0.654 - 1.018)	0.564 (0.314 - 0.921)	0.565 (0.314 - 0.918)	0.565 (0.317 - 0.922)
15 - 49 years (rate per capita)	0.469 (0.437 - 0.498)	0.472 (0.441 - 0.500)	0.474 (0.445 - 0.504)	0.818 (0.716 - 0.926)	0.823 (0.724 - 0.929)	0.824 (0.726 - 0.928)	1.550 (0.882 - 2.500)	1.665 (0.951 - 2.713)	1.712 (0.982 - 2.757)
50 - 69 years (rate per capita)	0.591 (0.562 - 0.624)	0.589 (0.559 - 0.623)	0.586 (0.556 - 0.618)	0.905 (0.769 - 1.038)	0.902 (0.770 - 1.035)	0.900 (0.763 - 1.033)	6.207 (3.883 - 9.268)	6.052 (3.773 - 9.072)	5.881 (3.685 - 8.797)
70+ years (rate per capita)	0.628 (0.602 - 0.654)	0.635 (0.610 - 0.662)	0.631 (0.605 - 0.658)	0.882 (0.753 - 1.008)	0.892 (0.756 - 1.028)	0.887 (0.752 - 1.019)	10.127 (6.682 - 14.204)	10.255 (6.773 - 14.405)	10.128 (6.714 - 14.261)
All ages (rate per capita)	0.505 (0.483 - 0.529)	0.505 (0.482 - 0.527)	0.504 (0.484 - 0.527)	0.790 (0.715 - 0.871)	0.800 (0.728 - 0.878)	0.806 (0.737 - 0.876)	1.641 (0.984 - 2.513)	1.668 (0.991 - 2.581)	1.936 (1.160 - 2.972)
Age-standardised (rate per capita)	0.519 (0.501 - 0.538)	0.520 (0.501 - 0.538)	0.519 (0.501 - 0.540)	0.822 (0.759 - 0.887)	0.822 (0.759 - 0.887)	0.822 (0.759 - 0.887)	2.779 (1.719 - 4.216)	2.774 (1.715 - 4.186)	2.769 (1.715 - 4.177)
Total number of cases (thousands)	95 (91 - 100)	142 (136 - 148)	181 (174 - 189)	149 (135 - 164)	225 (205 - 247)	289 (264 - 314)	0 (0 - 0)	0 (0 - 1)	1 (0 - 1)
**Bermuda**									
Under-5 years (rate per capita)	0.438 (0.370 - 0.505)	0.435 (0.366 - 0.501)	0.435 (0.366 - 0.497)	0.603 (0.413 - 0.776)	0.602 (0.412 - 0.775)	0.601 (0.411 - 0.773)	0.169 (0.071 - 0.344)	0.169 (0.070 - 0.335)	0.168 (0.070 - 0.342)
5-14 years (rate per capita)	0.564 (0.515 - 0.620)	0.560 (0.513 - 0.615)	0.557 (0.506 - 0.614)	0.857 (0.678 - 1.072)	0.852 (0.679 - 1.068)	0.851 (0.679 - 1.066)	0.730 (0.392 - 1.220)	0.730 (0.388 - 1.228)	0.729 (0.393 - 1.234)
15 - 49 years (rate per capita)	0.468 (0.438 - 0.497)	0.472 (0.444 - 0.501)	0.473 (0.444 - 0.501)	0.861 (0.751 - 0.967)	0.865 (0.758 - 0.967)	0.866 (0.762 - 0.968)	1.848 (1.050 - 3.036)	1.963 (1.116 - 3.215)	1.995 (1.138 - 3.277)
50 - 69 years (rate per capita)	0.587 (0.560 - 0.618)	0.587 (0.559 - 0.617)	0.586 (0.556 - 0.616)	0.942 (0.799 - 1.086)	0.941 (0.800 - 1.085)	0.940 (0.800 - 1.084)	6.388 (4.022 - 9.613)	6.364 (4.030 - 9.578)	6.294 (3.944 - 9.474)
70+ years (rate per capita)	0.628 (0.602 - 0.654)	0.624 (0.600 - 0.648)	0.623 (0.597 - 0.649)	0.918 (0.779 - 1.050)	0.914 (0.777 - 1.043)	0.912 (0.776 - 1.040)	10.341 (6.881 - 14.636)	10.252 (6.848 - 14.486)	10.221 (6.797 - 14.482)
All ages (rate per capita)	0.508 (0.488 - 0.529)	0.512 (0.493 - 0.531)	0.515 (0.496 - 0.534)	0.852 (0.779 - 0.928)	0.859 (0.788 - 0.933)	0.864 (0.796 - 0.935)	2.414 (1.462 - 3.682)	2.676 (1.615 - 4.087)	2.950 (1.792 - 4.490)
Age-standardised (rate per capita)	0.515 (0.496 - 0.534)	0.515 (0.498 - 0.534)	0.514 (0.495 - 0.534)	0.857 (0.790 - 0.927)	0.857 (0.790 - 0.927)	0.857 (0.790 - 0.927)	2.942 (1.802 - 4.475)	2.944 (1.790 - 4.476)	2.942 (1.792 - 4.458)
Total number of cases (thousands)	28 (27 - 29)	32 (31 - 33)	34 (33 - 36)	47 (43 - 51)	54 (50 - 59)	58 (53 - 63)	0 (0 - 0)	0 (0 - 0)	0 (0 - 0)
**Cuba**									
Under-5 years (rate per capita)	0.435 (0.365 - 0.501)	0.441 (0.367 - 0.509)	0.440 (0.372 - 0.504)	0.602 (0.412 - 0.775)	0.608 (0.416 - 0.782)	0.606 (0.415 - 0.780)	0.168 (0.071 - 0.343)	0.170 (0.069 - 0.345)	0.170 (0.070 - 0.342)
5-14 years (rate per capita)	0.559 (0.509 - 0.615)	0.555 (0.504 - 0.610)	0.555 (0.505 - 0.612)	0.852 (0.679 - 1.068)	0.849 (0.677 - 1.064)	0.848 (0.675 - 1.062)	0.729 (0.393 - 1.236)	0.729 (0.390 - 1.227)	0.730 (0.387 - 1.236)
15 - 49 years (rate per capita)	0.467 (0.436 - 0.495)	0.477 (0.451 - 0.505)	0.480 (0.453 - 0.506)	0.862 (0.755 - 0.971)	0.872 (0.767 - 0.973)	0.872 (0.765 - 0.974)	1.872 (1.061 - 3.078)	2.103 (1.200 - 3.427)	2.187 (1.240 - 3.557)
50 - 69 years (rate per capita)	0.588 (0.561 - 0.618)	0.588 (0.561 - 0.619)	0.585 (0.557 - 0.617)	0.942 (0.800 - 1.086)	0.942 (0.800 - 1.086)	0.939 (0.801 - 1.082)	6.439 (4.027 - 9.600)	6.442 (4.012 - 9.624)	6.294 (3.911 - 9.462)
70+ years (rate per capita)	0.624 (0.601 - 0.652)	0.622 (0.599 - 0.647)	0.620 (0.596 - 0.645)	0.914 (0.777 - 1.043)	0.911 (0.775 - 1.038)	0.908 (0.774 - 1.034)	10.309 (6.760 - 14.471)	10.233 (6.732 - 14.390)	10.178 (6.721 - 14.293)
All ages (rate per capita)	0.505 (0.485 - 0.524)	0.517 (0.499 - 0.535)	0.526 (0.509 - 0.544)	0.854 (0.782 - 0.928)	0.868 (0.801 - 0.939)	0.876 (0.809 - 0.944)	2.734 (1.652 - 4.167)	3.204 (1.960 - 4.884)	3.704 (2.281 - 5.598)
Age-standardised (rate per capita)	0.514 (0.497 - 0.532)	0.515 (0.497 - 0.533)	0.515 (0.497 - 0.534)	0.857 (0.790 - 0.927)	0.857 (0.790 - 0.927)	0.857 (0.790 - 0.927)	2.951 (1.792 - 4.470)	2.949 (1.799 - 4.482)	2.947 (1.795 - 4.466)
Total number of cases (thousands)	5,339 (5,130 - 5,542)	5,825 (5,625 - 6,028)	5,991 (5,803 - 6,193)	9,020 (8,261 - 9,801)	9,787 (9,032 - 10,580)	9,977 (9,214 - 10,749)	29 (17 - 44)	36 (22 - 55)	42 (26 - 64)
**Dominica**									
Under-5 years (rate per capita)	0.439 (0.370 - 0.507)	0.441 (0.375 - 0.504)	0.438 (0.369 - 0.503)	0.583 (0.403 - 0.749)	0.584 (0.404 - 0.751)	0.582 (0.402 - 0.748)	0.104 (0.044 - 0.205)	0.104 (0.044 - 0.202)	0.104 (0.044 - 0.205)
5-14 years (rate per capita)	0.568 (0.517 - 0.626)	0.564 (0.513 - 0.620)	0.563 (0.512 - 0.620)	0.827 (0.659 - 1.033)	0.822 (0.654 - 1.020)	0.821 (0.655 - 1.019)	0.565 (0.313 - 0.925)	0.564 (0.312 - 0.935)	0.565 (0.311 - 0.919)
15 - 49 years (rate per capita)	0.472 (0.441 - 0.502)	0.476 (0.447 - 0.503)	0.478 (0.451 - 0.507)	0.823 (0.723 - 0.928)	0.827 (0.729 - 0.931)	0.828 (0.731 - 0.932)	1.677 (0.962 - 2.713)	1.792 (1.023 - 2.873)	1.822 (1.047 - 2.923)
50 - 69 years (rate per capita)	0.591 (0.563 - 0.621)	0.590 (0.560 - 0.622)	0.589 (0.559 - 0.621)	0.904 (0.770 - 1.038)	0.904 (0.771 - 1.037)	0.903 (0.769 - 1.036)	6.213 (3.947 - 9.198)	6.176 (3.898 - 9.134)	6.094 (3.855 - 9.042)
70+ years (rate per capita)	0.629 (0.606 - 0.655)	0.626 (0.601 - 0.651)	0.625 (0.601 - 0.651)	0.884 (0.753 - 1.013)	0.880 (0.754 - 1.005)	0.879 (0.752 - 1.002)	10.142 (6.694 - 14.321)	10.051 (6.675 - 14.122)	9.988 (6.629 - 14.011)
All ages (rate per capita)	0.509 (0.489 - 0.530)	0.515 (0.496 - 0.534)	0.518 (0.500 - 0.537)	0.808 (0.739 - 0.879)	0.820 (0.756 - 0.888)	0.824 (0.760 - 0.889)	2.158 (1.317 - 3.267)	2.469 (1.513 - 3.750)	2.719 (1.686 - 4.113)
Age-standardised (rate per capita)	0.518 (0.500 - 0.538)	0.519 (0.501 - 0.537)	0.519 (0.501 - 0.537)	0.822 (0.759 - 0.887)	0.822 (0.759 - 0.887)	0.822 (0.759 - 0.887)	2.780 (1.729 - 4.193)	2.778 (1.728 - 4.175)	2.772 (1.724 - 4.177)
Total number of cases (thousands)	35 (34 - 37)	36 (35 - 37)	37 (36 - 38)	56 (51 - 61)	57 (53 - 62)	59 (55 - 64)	0 (0 - 0)	0 (0 - 0)	0 (0 - 0)
**Dominican Republic**									
Under-5 years (rate per capita)	0.433 (0.365 - 0.499)	0.439 (0.372 - 0.503)	0.439 (0.369 - 0.509)	0.578 (0.399 - 0.743)	0.580 (0.401 - 0.746)	0.583 (0.402 - 0.749)	0.103 (0.044 - 0.202)	0.104 (0.044 - 0.201)	0.104 (0.045 - 0.205)
5-14 years (rate per capita)	0.569 (0.519 - 0.622)	0.565 (0.513 - 0.624)	0.566 (0.517 - 0.625)	0.827 (0.659 - 1.033)	0.822 (0.654 - 1.022)	0.823 (0.654 - 1.024)	0.565 (0.318 - 0.922)	0.565 (0.316 - 0.920)	0.565 (0.315 - 0.925)
15 - 49 years (rate per capita)	0.471 (0.440 - 0.501)	0.475 (0.447 - 0.501)	0.476 (0.446 - 0.505)	0.821 (0.721 - 0.927)	0.825 (0.726 - 0.929)	0.826 (0.728 - 0.930)	1.627 (0.928 - 2.620)	1.724 (0.982 - 2.786)	1.761 (1.013 - 2.820)
50 - 69 years (rate per capita)	0.589 (0.560 - 0.620)	0.590 (0.562 - 0.624)	0.589 (0.559 - 0.624)	0.903 (0.766 - 1.036)	0.903 (0.769 - 1.034)	0.903 (0.766 - 1.036)	6.145 (3.847 - 9.167)	6.171 (3.887 - 9.147)	6.130 (3.845 - 9.113)
70+ years (rate per capita)	0.634 (0.610 - 0.661)	0.629 (0.606 - 0.655)	0.624 (0.600 - 0.649)	0.892 (0.753 - 1.029)	0.883 (0.753 - 1.009)	0.876 (0.751 - 0.997)	10.299 (6.801 - 14.434)	10.152 (6.691 - 14.260)	10.041 (6.674 - 14.103)
All ages (rate per capita)	0.505 (0.482 - 0.528)	0.509 (0.489 - 0.531)	0.513 (0.493 - 0.533)	0.798 (0.727 - 0.875)	0.807 (0.738 - 0.879)	0.814 (0.748 - 0.882)	1.789 (1.069 - 2.721)	2.093 (1.274 - 3.189)	2.331 (1.421 - 3.555)
Age-standardised (rate per capita)	0.519 (0.501 - 0.538)	0.520 (0.503 - 0.540)	0.520 (0.501 - 0.540)	0.822 (0.759 - 0.886)	0.822 (0.759 - 0.887)	0.822 (0.759 - 0.887)	2.790 (1.716 - 4.211)	2.794 (1.730 - 4.204)	2.792 (1.724 - 4.209)
Total number of cases (thousands)	3,636 (3,471 - 3,797)	4,703 (4,522 - 4,907)	5,397 (5,188 - 5,617)	5,744 (5,229 - 6,298)	7,457 (6,816 - 8,119)	8,570 (7,873 - 9,289)	13 (8 - 20)	19 (12 - 29)	25 (15 - 37)
**Grenada**									
Under-5 years (rate per capita)	0.445 (0.374 - 0.511)	0.439 (0.370 - 0.505)	0.440 (0.370 - 0.503)	0.590 (0.407 - 0.758)	0.582 (0.402 - 0.748)	0.582 (0.402 - 0.749)	0.105 (0.045 - 0.207)	0.104 (0.044 - 0.205)	0.104 (0.043 - 0.205)
5-14 years (rate per capita)	0.581 (0.528 - 0.638)	0.557 (0.505 - 0.612)	0.567 (0.509 - 0.627)	0.837 (0.668 - 1.046)	0.815 (0.650 - 1.011)	0.825 (0.657 - 1.030)	0.561 (0.316 - 0.918)	0.566 (0.312 - 0.937)	0.564 (0.311 - 0.914)
15 - 49 years (rate per capita)	0.470 (0.435 - 0.501)	0.473 (0.440 - 0.502)	0.473 (0.441 - 0.506)	0.821 (0.720 - 0.926)	0.820 (0.720 - 0.924)	0.826 (0.725 - 0.931)	1.605 (0.919 - 2.573)	1.646 (0.943 - 2.638)	1.699 (0.977 - 2.740)
50 - 69 years (rate per capita)	0.596 (0.569 - 0.628)	0.591 (0.564 - 0.624)	0.587 (0.557 - 0.618)	0.908 (0.774 - 1.041)	0.905 (0.772 - 1.036)	0.901 (0.766 - 1.034)	6.460 (4.124 - 9.606)	6.246 (3.993 - 9.228)	5.938 (3.739 - 8.896)
70+ years (rate per capita)	0.634 (0.609 - 0.664)	0.627 (0.602 - 0.654)	0.624 (0.600 - 0.649)	0.894 (0.757 - 1.031)	0.880 (0.748 - 1.005)	0.877 (0.750 - 0.998)	10.308 (6.842 - 14.494)	10.144 (6.754 - 14.278)	10.016 (6.643 - 14.075)
All ages (rate per capita)	0.516 (0.493 - 0.538)	0.508 (0.487 - 0.530)	0.510 (0.489 - 0.533)	0.804 (0.730 - 0.878)	0.809 (0.740 - 0.879)	0.816 (0.752 - 0.886)	2.166 (1.341 - 3.264)	2.224 (1.365 - 3.359)	2.384 (1.461 - 3.630)
Age-standardised (rate per capita)	0.519 (0.499 - 0.538)	0.519 (0.501 - 0.540)	0.520 (0.500 - 0.540)	0.822 (0.759 - 0.887)	0.822 (0.759 - 0.887)	0.822 (0.759 - 0.887)	2.779 (1.731 - 4.191)	2.777 (1.726 - 4.177)	2.769 (1.719 - 4.160)
Total number of cases (thousands)	50 (47 - 52)	52 (50 - 55)	55 (52 - 57)	77 (70 - 84)	84 (76 - 91)	87 (80 - 95)	0 (0 - 0)	0 (0 - 0)	0 (0 - 0)
**Guyana**									
Under-5 years (rate per capita)	0.440 (0.373 - 0.505)	0.445 (0.374 - 0.516)	0.435 (0.367 - 0.499)	0.584 (0.403 - 0.750)	0.590 (0.408 - 0.758)	0.578 (0.399 - 0.743)	0.104 (0.045 - 0.204)	0.105 (0.045 - 0.204)	0.103 (0.044 - 0.200)
5-14 years (rate per capita)	0.559 (0.508 - 0.613)	0.568 (0.513 - 0.623)	0.545 (0.493 - 0.599)	0.815 (0.650 - 1.012)	0.826 (0.659 - 1.033)	0.803 (0.641 - 1.001)	0.566 (0.319 - 0.936)	0.565 (0.318 - 0.933)	0.566 (0.314 - 0.921)
15 - 49 years (rate per capita)	0.472 (0.443 - 0.502)	0.480 (0.451 - 0.509)	0.477 (0.447 - 0.506)	0.822 (0.722 - 0.928)	0.830 (0.734 - 0.933)	0.821 (0.723 - 0.921)	1.635 (0.938 - 2.644)	1.848 (1.057 - 2.966)	1.747 (1.004 - 2.795)
50 - 69 years (rate per capita)	0.591 (0.561 - 0.621)	0.585 (0.555 - 0.617)	0.585 (0.554 - 0.620)	0.904 (0.771 - 1.037)	0.900 (0.765 - 1.033)	0.900 (0.762 - 1.035)	6.177 (3.925 - 9.219)	5.913 (3.747 - 8.842)	5.845 (3.697 - 8.756)
70+ years (rate per capita)	0.620 (0.596 - 0.644)	0.629 (0.605 - 0.655)	0.640 (0.615 - 0.667)	0.874 (0.751 - 0.990)	0.883 (0.753 - 1.012)	0.898 (0.757 - 1.042)	9.814 (6.485 - 13.804)	10.093 (6.685 - 14.248)	10.297 (6.831 - 14.505)
All ages (rate per capita)	0.500 (0.479 - 0.522)	0.514 (0.492 - 0.535)	0.510 (0.490 - 0.532)	0.792 (0.722 - 0.865)	0.811 (0.738 - 0.883)	0.811 (0.744 - 0.880)	1.735 (1.045 - 2.649)	1.972 (1.185 - 3.025)	2.289 (1.389 - 3.511)
Age-standardised (rate per capita)	0.520 (0.502 - 0.538)	0.519 (0.501 - 0.539)	0.519 (0.500 - 0.539)	0.822 (0.759 - 0.887)	0.822 (0.759 - 0.887)	0.822 (0.759 - 0.887)	2.763 (1.715 - 4.163)	2.762 (1.716 - 4.157)	2.759 (1.715 - 4.154)
Total number of cases (thousands)	374 (358 - 391)	382 (366 - 397)	393 (377 - 410)	593 (541 - 648)	602 (548 - 656)	624 (573 - 678)	1 (1 - 2)	1 (1 - 2)	2 (1 - 3)
**Haiti**									
Under-5 years (rate per capita)	0.433 (0.363 - 0.495)	0.434 (0.365 - 0.499)	0.436 (0.368 - 0.504)	0.573 (0.396 - 0.736)	0.578 (0.399 - 0.742)	0.580 (0.400 - 0.745)	0.102 (0.042 - 0.199)	0.103 (0.044 - 0.204)	0.103 (0.043 - 0.203)
5-14 years (rate per capita)	0.577 (0.525 - 0.634)	0.567 (0.517 - 0.619)	0.566 (0.512 - 0.627)	0.834 (0.666 - 1.042)	0.825 (0.656 - 1.028)	0.824 (0.655 - 1.026)	0.560 (0.314 - 0.906)	0.563 (0.312 - 0.920)	0.562 (0.313 - 0.925)
15 - 49 years (rate per capita)	0.472 (0.441 - 0.503)	0.472 (0.441 - 0.501)	0.472 (0.442 - 0.504)	0.821 (0.723 - 0.927)	0.820 (0.720 - 0.925)	0.823 (0.722 - 0.929)	1.639 (0.945 - 2.645)	1.626 (0.934 - 2.603)	1.663 (0.952 - 2.677)
50 - 69 years (rate per capita)	0.589 (0.559 - 0.624)	0.590 (0.563 - 0.621)	0.588 (0.559 - 0.623)	0.903 (0.769 - 1.035)	0.903 (0.771 - 1.036)	0.902 (0.767 - 1.035)	6.032 (3.789 - 9.018)	6.103 (3.825 - 9.090)	5.962 (3.743 - 8.866)
70+ years (rate per capita)	0.638 (0.612 - 0.665)	0.635 (0.609 - 0.664)	0.634 (0.608 - 0.663)	0.896 (0.757 - 1.037)	0.894 (0.756 - 1.032)	0.892 (0.755 - 1.028)	10.200 (6.774 - 14.301)	10.197 (6.736 - 14.331)	10.057 (6.631 - 14.129)
All ages (rate per capita)	0.509 (0.487 - 0.534)	0.506 (0.485 - 0.528)	0.506 (0.485 - 0.528)	0.794 (0.721 - 0.871)	0.799 (0.727 - 0.876)	0.806 (0.737 - 0.878)	1.722 (1.040 - 2.617)	1.806 (1.087 - 2.749)	1.949 (1.172 - 2.985)
Age-standardised (rate per capita)	0.519 (0.502 - 0.540)	0.519 (0.501 - 0.537)	0.519 (0.501 - 0.539)	0.822 (0.759 - 0.887)	0.822 (0.759 - 0.887)	0.822 (0.759 - 0.887)	2.749 (1.696 - 4.147)	2.761 (1.708 - 4.158)	2.740 (1.686 - 4.131)
Total number of cases (thousands)	3,613 (3,456 - 3,788)	4,689 (4,493 - 4,892)	5,426 (5,201 - 5,662)	5,637 (5,120 - 6,187)	7,403 (6,742 - 8,121)	8,639 (7,898 - 9,414)	12 (7 - 19)	17 (10 - 25)	21 (13 - 32)
**Jamaica**									
Under-5 years (rate per capita)	0.440 (0.369 - 0.505)	0.440 (0.377 - 0.504)	0.441 (0.368 - 0.508)	0.584 (0.403 - 0.751)	0.586 (0.405 - 0.753)	0.584 (0.403 - 0.750)	0.104 (0.045 - 0.203)	0.104 (0.044 - 0.205)	0.104 (0.045 - 0.201)

	Prevalence 1990	Prevalence 2005	Prevalence 2015	Incidence 1990	Incidence 2005	Incidence 2015	YLD rate 1990	YLD rate 2005	YLD rate 2015
5-14 years (rate per capita)	0.571 (0.518 - 0.628)	0.565 (0.514 - 0.621)	0.558 (0.504 - 0.615)	0.827 (0.659 - 1.033)	0.822 (0.654 - 1.022)	0.817 (0.651 - 1.013)	0.565 (0.314 - 0.934)	0.564 (0.315 - 0.920)	0.563 (0.313 - 0.916)
15 - 49 years (rate per capita)	0.470 (0.440 - 0.501)	0.477 (0.447 - 0.505)	0.476 (0.444 - 0.504)	0.820 (0.720 - 0.927)	0.826 (0.727 - 0.930)	0.826 (0.726 - 0.930)	1.617 (0.929 - 2.611)	1.773 (1.021 - 2.854)	1.759 (1.011 - 2.831)
50 - 69 years (rate per capita)	0.594 (0.567 - 0.626)	0.590 (0.560 - 0.621)	0.590 (0.561 - 0.624)	0.907 (0.771 - 1.040)	0.903 (0.770 - 1.033)	0.903 (0.768 - 1.036)	6.410 (4.004 - 9.463)	6.141 (3.818 - 9.168)	6.120 (3.848 - 9.078)
70+ years (rate per capita)	0.625 (0.602 - 0.650)	0.623 (0.599 - 0.648)	0.620 (0.597 - 0.644)	0.877 (0.751 - 0.999)	0.875 (0.749 - 0.995)	0.871 (0.749 - 0.988)	10.124 (6.685 - 14.216)	10.025 (6.649 - 14.131)	9.908 (6.561 - 13.905)
All ages (rate per capita)	0.510 (0.490 - 0.532)	0.514 (0.495 - 0.536)	0.514 (0.493 - 0.535)	0.804 (0.735 - 0.878)	0.814 (0.746 - 0.883)	0.819 (0.757 - 0.883)	2.128 (1.303 - 3.195)	2.374 (1.451 - 3.576)	2.640 (1.623 - 3.974)
Age-standardised (rate per capita)	0.519 (0.502 - 0.539)	0.519 (0.501 - 0.540)	0.519 (0.500 - 0.540)	0.822 (0.759 - 0.887)	0.822 (0.759 - 0.887)	0.822 (0.759 - 0.887)	2.793 (1.731 - 4.206)	2.784 (1.721 - 4.190)	2.774 (1.714 - 4.153)
Total number of cases (thousands)	1,224 (1,177 - 1,276)	1,382 (1,331 - 1,442)	1,453 (1,396 - 1,513)	1,929 (1,765 - 2,106)	2,189 (2,006 - 2,374)	2,318 (2,142 - 2,498)	5 (3 - 8)	6 (4 - 10)	7 (5 - 11)
**Puerto Rico**									
Under-5 years (rate per capita)	0.437 (0.370 - 0.500)	0.439 (0.372 - 0.502)	0.439 (0.370 - 0.503)	0.603 (0.413 - 0.776)	0.608 (0.416 - 0.782)	0.606 (0.414 - 0.780)	0.169 (0.071 - 0.341)	0.170 (0.071 - 0.343)	0.170 (0.070 - 0.348)
5-14 years (rate per capita)	0.553 (0.502 - 0.606)	0.555 (0.502 - 0.611)	0.553 (0.503 - 0.607)	0.848 (0.675 - 1.062)	0.849 (0.676 - 1.063)	0.848 (0.674 - 1.061)	0.730 (0.391 - 1.245)	0.730 (0.389 - 1.217)	0.730 (0.388 - 1.247)
15 - 49 years (rate per capita)	0.472 (0.445 - 0.501)	0.473 (0.446 - 0.501)	0.474 (0.446 - 0.502)	0.865 (0.759 - 0.967)	0.867 (0.763 - 0.970)	0.868 (0.764 - 0.973)	1.958 (1.116 - 3.198)	2.015 (1.150 - 3.295)	2.039 (1.156 - 3.336)
50 - 69 years (rate per capita)	0.590 (0.561 - 0.619)	0.589 (0.560 - 0.623)	0.591 (0.563 - 0.623)	0.944 (0.800 - 1.088)	0.943 (0.800 - 1.087)	0.944 (0.800 - 1.087)	6.537 (4.062 - 9.732)	6.472 (4.056 - 9.658)	6.574 (4.143 - 9.815)
70+ years (rate per capita)	0.624 (0.601 - 0.650)	0.620 (0.597 - 0.644)	0.620 (0.597 - 0.646)	0.914 (0.777 - 1.043)	0.907 (0.775 - 1.031)	0.906 (0.775 - 1.029)	10.281 (6.833 - 14.552)	10.158 (6.761 - 14.393)	10.122 (6.747 - 14.225)
All ages (rate per capita)	0.512 (0.494 - 0.531)	0.518 (0.500 - 0.538)	0.522 (0.506 - 0.539)	0.853 (0.785 - 0.925)	0.865 (0.800 - 0.932)	0.870 (0.805 - 0.937)	2.812 (1.713 - 4.262)	3.251 (2.002 - 4.917)	3.538 (2.194 - 5.335)
Age-standardised (rate per capita)	0.515 (0.497 - 0.533)	0.514 (0.496 - 0.533)	0.515 (0.497 - 0.533)	0.857 (0.790 - 0.927)	0.857 (0.790 - 0.928)	0.857 (0.790 - 0.928)	2.951 (1.806 - 4.471)	2.950 (1.805 - 4.475)	2.948 (1.801 - 4.472)
Total number of cases (thousands)	1,808 (1,747 - 1,875)	1,951 (1,883 - 2,023)	1,923 (1,863 - 1,987)	3,015 (2,773 - 3,267)	3,253 (3,009 - 3,509)	3,205 (2,966 - 3,452)	10 (6 - 15)	12 (8 - 19)	13 (8 - 20)
**Saint Lucia**									
Under-5 years (rate per capita)	0.436 (0.373 - 0.501)	0.436 (0.367 - 0.501)	0.437 (0.369 - 0.501)	0.603 (0.413 - 0.776)	0.604 (0.413 - 0.777)	0.604 (0.413 - 0.777)	0.169 (0.069 - 0.338)	0.169 (0.070 - 0.345)	0.169 (0.072 - 0.334)
5-14 years (rate per capita)	0.564 (0.512 - 0.620)	0.552 (0.500 - 0.607)	0.556 (0.506 - 0.608)	0.856 (0.678 - 1.072)	0.846 (0.674 - 1.059)	0.848 (0.676 - 1.062)	0.725 (0.390 - 1.200)	0.732 (0.395 - 1.238)	0.731 (0.389 - 1.219)
15 - 49 years (rate per capita)	0.465 (0.437 - 0.495)	0.470 (0.439 - 0.498)	0.474 (0.446 - 0.500)	0.858 (0.748 - 0.968)	0.863 (0.755 - 0.967)	0.866 (0.761 - 0.969)	1.772 (1.003 - 2.924)	1.915 (1.082 - 3.144)	2.004 (1.148 - 3.297)
50 - 69 years (rate per capita)	0.590 (0.562 - 0.620)	0.586 (0.557 - 0.619)	0.583 (0.555 - 0.615)	0.944 (0.798 - 1.089)	0.941 (0.802 - 1.085)	0.939 (0.798 - 1.081)	6.486 (4.087 - 9.667)	6.311 (3.944 - 9.459)	6.192 (3.869 - 9.342)
70+ years (rate per capita)	0.634 (0.609 - 0.661)	0.614 (0.592 - 0.636)	0.619 (0.595 - 0.645)	0.929 (0.782 - 1.071)	0.900 (0.774 - 1.018)	0.907 (0.774 - 1.031)	10.413 (6.915 - 14.694)	9.940 (6.583 - 14.023)	10.085 (6.674 - 14.195)
All ages (rate per capita)	0.507 (0.487 - 0.530)	0.504 (0.484 - 0.523)	0.512 (0.494 - 0.531)	0.836 (0.759 - 0.914)	0.848 (0.775 - 0.920)	0.859 (0.792 - 0.930)	2.254 (1.363 - 3.437)	2.443 (1.468 - 3.733)	2.890 (1.747 - 4.429)
Age-standardised (rate per capita)	0.515 (0.497 - 0.534)	0.514 (0.496 - 0.533)	0.515 (0.497 - 0.534)	0.857 (0.790 - 0.927)	0.857 (0.790 - 0.927)	0.857 (0.790 - 0.927)	2.917 (1.783 - 4.436)	2.931 (1.787 - 4.454)	2.929 (1.789 - 4.484)
Total number of cases (thousands)	71 (68 - 74)	83 (80 - 87)	95 (91 - 98)	117 (106 - 128)	140 (128 - 152)	159 (147 - 172)	0 (0 - 0)	0 (0 - 1)	1 (0 - 1)
**Saint Vincent and the Grenadines**									
Under-5 years (rate per capita)	0.396 (0.334 - 0.462)	0.393 (0.335 - 0.456)	0.392 (0.331 - 0.454)	0.590 (0.373 - 0.780)	0.587 (0.379 - 0.784)	0.586 (0.379 - 0.784)	0.154 (0.064 - 0.311)	0.152 (0.064 - 0.310)	0.152 (0.064 - 0.303)
5-14 years (rate per capita)	0.666 (0.630 - 0.695)	0.589 (0.534 - 0.640)	0.589 (0.536 - 0.642)	0.958 (0.775 - 1.200)	0.850 (0.681 - 1.068)	0.851 (0.682 - 1.070)	0.855 (0.451 - 1.449)	0.780 (0.419 - 1.303)	0.779 (0.417 - 1.294)
15 - 49 years (rate per capita)	0.523 (0.500 - 0.546)	0.517 (0.496 - 0.542)	0.517 (0.497 - 0.540)	0.869 (0.792 - 0.949)	0.879 (0.806 - 0.953)	0.880 (0.809 - 0.954)	1.803 (1.016 - 3.031)	1.994 (1.128 - 3.315)	2.020 (1.150 - 3.361)
50 - 69 years (rate per capita)	0.622 (0.597 - 0.647)	0.621 (0.596 - 0.645)	0.617 (0.592 - 0.642)	0.982 (0.883 - 1.087)	0.980 (0.882 - 1.085)	0.978 (0.876 - 1.083)	6.511 (4.073 - 9.680)	6.337 (3.975 - 9.441)	6.100 (3.785 - 9.175)
70+ years (rate per capita)	0.655 (0.638 - 0.676)	0.653 (0.635 - 0.673)	0.652 (0.633 - 0.672)	0.956 (0.854 - 1.055)	0.951 (0.854 - 1.052)	0.948 (0.853 - 1.048)	10.386 (6.872 - 14.762)	10.266 (6.768 - 14.537)	10.223 (6.749 - 14.473)
All ages (rate per capita)	0.559 (0.541 - 0.574)	0.539 (0.522 - 0.556)	0.543 (0.528 - 0.559)	0.872 (0.804 - 0.954)	0.862 (0.802 - 0.931)	0.872 (0.814 - 0.936)	2.186 (1.303 - 3.370)	2.495 (1.492 - 3.858)	2.775 (1.669 - 4.264)
Age-standardised (rate per capita)	0.564 (0.549 - 0.578)	0.546 (0.530 - 0.562)	0.546 (0.531 - 0.561)	0.890 (0.833 - 0.953)	0.872 (0.813 - 0.936)	0.872 (0.812 - 0.937)	2.998 (1.813 - 4.581)	2.967 (1.797 - 4.514)	2.962 (1.800 - 4.527)
Total number of cases (thousands)	60 (58 - 62)	59 (57 - 61)	60 (58 - 61)	94 (86 - 102)	94 (87 - 101)	96 (89 - 103)	0 (0 - 0)	0 (0 - 0)	0 (0 - 0)
**Suriname**									
Under-5 years (rate per capita)	0.466 (0.413 - 0.516)	0.477 (0.416 - 0.524)	0.469 (0.414 - 0.521)	0.582 (0.422 - 0.729)	0.601 (0.444 - 0.743)	0.586 (0.424 - 0.733)	0.110 (0.048 - 0.211)	0.113 (0.049 - 0.214)	0.111 (0.048 - 0.215)
5-14 years (rate per capita)	0.553 (0.502 - 0.604)	0.583 (0.536 - 0.622)	0.538 (0.490 - 0.588)	0.774 (0.615 - 0.978)	0.793 (0.618 - 1.019)	0.764 (0.609 - 0.965)	0.539 (0.304 - 0.866)	0.548 (0.306 - 0.892)	0.540 (0.303 - 0.867)
15 - 49 years (rate per capita)	0.421 (0.401 - 0.444)	0.428 (0.409 - 0.451)	0.429 (0.410 - 0.451)	0.781 (0.705 - 0.861)	0.786 (0.712 - 0.861)	0.787 (0.714 - 0.859)	1.613 (0.934 - 2.603)	1.757 (1.019 - 2.849)	1.791 (1.029 - 2.887)
50 - 69 years (rate per capita)	0.552 (0.525 - 0.581)	0.554 (0.529 - 0.579)	0.549 (0.524 - 0.577)	0.863 (0.765 - 0.972)	0.862 (0.766 - 0.963)	0.860 (0.762 - 0.970)	6.102 (3.811 - 9.175)	6.151 (3.882 - 9.115)	5.926 (3.725 - 8.899)
70+ years (rate per capita)	0.596 (0.577 - 0.618)	0.603 (0.584 - 0.624)	0.599 (0.579 - 0.622)	0.827 (0.737 - 0.919)	0.840 (0.744 - 0.940)	0.830 (0.739 - 0.923)	10.133 (6.717 - 14.170)	10.291 (6.811 - 14.439)	10.196 (6.744 - 14.261)
All ages (rate per capita)	0.475 (0.458 - 0.491)	0.485 (0.471 - 0.500)	0.480 (0.463 - 0.495)	0.764 (0.703 - 0.829)	0.780 (0.722 - 0.848)	0.779 (0.724 - 0.836)	1.946 (1.185 - 2.968)	2.237 (1.364 - 3.418)	2.479 (1.522 - 3.776)
Age-standardised (rate per capita)	0.485 (0.470 - 0.499)	0.491 (0.477 - 0.505)	0.485 (0.469 - 0.500)	0.781 (0.728 - 0.837)	0.787 (0.732 - 0.850)	0.781 (0.728 - 0.837)	2.755 (1.712 - 4.154)	2.759 (1.708 - 4.146)	2.756 (1.713 - 4.129)
Total number of cases (thousands)	194 (187 - 201)	238 (231 - 246)	260 (251 - 269)	312 (287 - 338)	383 (355 - 416)	423 (393 - 453)	1 (0 - 1)	1 (1 - 2)	1 (1 - 2)
**Trinidad and Tobago**									
Under-5 years (rate per capita)	0.445 (0.378 - 0.510)	0.433 (0.364 - 0.496)	0.438 (0.370 - 0.503)	0.614 (0.420 - 0.791)	0.598 (0.409 - 0.770)	0.606 (0.414 - 0.780)	0.172 (0.071 - 0.352)	0.167 (0.070 - 0.339)	0.170 (0.072 - 0.342)
5-14 years (rate per capita)	0.569 (0.517 - 0.628)	0.549 (0.497 - 0.605)	0.562 (0.510 - 0.618)	0.861 (0.682 - 1.079)	0.843 (0.672 - 1.055)	0.856 (0.678 - 1.071)	0.728 (0.392 - 1.233)	0.731 (0.387 - 1.239)	0.729 (0.388 - 1.213)
15 - 49 years (rate per capita)	0.469 (0.441 - 0.498)	0.473 (0.446 - 0.501)	0.474 (0.447 - 0.504)	0.864 (0.755 - 0.969)	0.864 (0.758 - 0.969)	0.871 (0.767 - 0.977)	1.890 (1.069 - 3.106)	1.968 (1.121 - 3.211)	2.049 (1.161 - 3.349)
50 - 69 years (rate per capita)	0.588 (0.559 - 0.621)	0.585 (0.557 - 0.616)	0.585 (0.556 - 0.617)	0.942 (0.801 - 1.085)	0.940 (0.798 - 1.083)	0.940 (0.800 - 1.085)	6.330 (3.973 - 9.467)	6.177 (3.845 - 9.318)	6.221 (3.884 - 9.375)
70+ years (rate per capita)	0.631 (0.605 - 0.657)	0.626 (0.603 - 0.652)	0.629 (0.604 - 0.656)	0.924 (0.781 - 1.060)	0.919 (0.780 - 1.050)	0.922 (0.783 - 1.055)	10.383 (6.857 - 14.672)	10.295 (6.825 - 14.526)	10.303 (6.818 - 14.535)
All ages (rate per capita)	0.508 (0.487 - 0.531)	0.507 (0.488 - 0.526)	0.516 (0.499 - 0.535)	0.846 (0.772 - 0.921)	0.857 (0.786 - 0.929)	0.868 (0.801 - 0.938)	2.239 (1.338 - 3.434)	2.730 (1.645 - 4.158)	3.109 (1.900 - 4.720)
Age-standardised (rate per capita)	0.515 (0.496 - 0.535)	0.515 (0.497 - 0.534)	0.514 (0.497 - 0.534)	0.857 (0.790 - 0.927)	0.857 (0.790 - 0.928)	0.857 (0.790 - 0.927)	2.927 (1.789 - 4.443)	2.924 (1.784 - 4.431)	2.921 (1.787 - 4.436)
Total number of cases (thousands)	623 (597 - 652)	658 (634 - 684)	702 (679 - 729)	1,038 (947 - 1,130)	1,113 (1,020 - 1,207)	1,181 (1,090 - 1,276)	3 (2 - 4)	4 (2 - 5)	4 (3 - 6)
**Virgin Islands, U.S.**									
Under-5 years (rate per capita)	0.442 (0.372 - 0.503)	0.435 (0.368 - 0.501)	0.444 (0.374 - 0.514)	0.585 (0.404 - 0.752)	0.578 (0.399 - 0.743)	0.590 (0.407 - 0.758)	0.105 (0.045 - 0.209)	0.103 (0.043 - 0.204)	0.105 (0.045 - 0.207)
5-14 years (rate per capita)	0.564 (0.512 - 0.618)	0.557 (0.502 - 0.611)	0.566 (0.514 - 0.623)	0.821 (0.654 - 1.020)	0.816 (0.651 - 1.011)	0.824 (0.655 - 1.026)	0.565 (0.316 - 0.931)	0.566 (0.315 - 0.926)	0.566 (0.316 - 0.917)
15 - 49 years (rate per capita)	0.480 (0.451 - 0.509)	0.483 (0.456 - 0.508)	0.482 (0.454 - 0.510)	0.829 (0.732 - 0.928)	0.831 (0.736 - 0.930)	0.830 (0.733 - 0.929)	1.861 (1.062 - 3.006)	1.939 (1.108 - 3.122)	1.928 (1.107 - 3.114)
50 - 69 years (rate per capita)	0.588 (0.558 - 0.620)	0.591 (0.563 - 0.625)	0.596 (0.568 - 0.627)	0.901 (0.770 - 1.034)	0.906 (0.766 - 1.040)	0.909 (0.771 - 1.042)	6.047 (3.796 - 9.052)	6.231 (3.896 - 9.267)	6.500 (4.106 - 9.622)
70+ years (rate per capita)	0.640 (0.614 - 0.668)	0.628 (0.603 - 0.653)	0.631 (0.606 - 0.657)	0.898 (0.758 - 1.041)	0.883 (0.753 - 1.008)	0.888 (0.754 - 1.019)	10.432 (6.850 - 14.775)	10.101 (6.655 - 14.185)	10.145 (6.699 - 14.358)
All ages (rate per capita)	0.513 (0.494 - 0.534)	0.526 (0.510 - 0.545)	0.538 (0.523 - 0.556)	0.813 (0.747 - 0.881)	0.832 (0.767 - 0.896)	0.840 (0.774 - 0.904)	2.326 (1.415 - 3.563)	3.154 (1.950 - 4.766)	3.767 (2.363 - 5.584)
Age-standardised (rate per capita)	0.519 (0.501 - 0.540)	0.519 (0.502 - 0.538)	0.519 (0.502 - 0.537)	0.822 (0.759 - 0.887)	0.822 (0.759 - 0.887)	0.822 (0.759 - 0.887)	2.791 (1.728 - 4.214)	2.785 (1.725 - 4.202)	2.785 (1.724 - 4.200)
Total number of cases (thousands)	54 (52 - 57)	57 (55 - 59)	57 (56 - 59)	86 (79 - 93)	90 (83 - 97)	90 (82 - 96)	0 (0 - 0)	0 (0 - 1)	0 (0 - 1)
**Tropical Latin America**									
Under-5 years (rate per capita)	0.314 (0.297 - 0.332)	0.256 (0.243 - 0.270)	0.318 (0.299 - 0.337)	0.539 (0.302 - 0.757)	0.409 (0.243 - 0.595)	0.464 (0.294 - 0.636)	0.074 (0.032 - 0.148)	0.060 (0.026 - 0.121)	0.076 (0.032 - 0.152)
5-14 years (rate per capita)	0.555 (0.535 - 0.575)	0.532 (0.510 - 0.551)	0.547 (0.527 - 0.570)	0.947 (0.775 - 1.140)	0.881 (0.715 - 1.060)	0.934 (0.775 - 1.117)	0.594 (0.330 - 0.975)	0.585 (0.324 - 0.951)	0.593 (0.328 - 0.962)
15 - 49 years (rate per capita)	0.512 (0.504 - 0.521)	0.520 (0.511 - 0.528)	0.520 (0.512 - 0.528)	0.951 (0.888 - 1.016)	0.962 (0.902 - 1.026)	0.967 (0.904 - 1.026)	1.907 (1.090 - 3.060)	2.015 (1.153 - 3.253)	2.064 (1.175 - 3.326)
50 - 69 years (rate per capita)	0.649 (0.638 - 0.659)	0.647 (0.638 - 0.656)	0.649 (0.639 - 0.658)	1.073 (0.983 - 1.157)	1.077 (0.988 - 1.157)	1.080 (0.995 - 1.164)	8.004 (5.096 - 11.741)	8.024 (5.115 - 11.756)	7.996 (5.099 - 11.737)
70+ years (rate per capita)	0.703 (0.695 - 0.711)	0.701 (0.693 - 0.708)	0.696 (0.689 - 0.703)	1.083 (0.996 - 1.155)	1.081 (1.002 - 1.150)	1.077 (0.997 - 1.141)	13.838 (9.194 - 19.186)	13.956 (9.308 - 19.269)	13.610 (9.044 - 18.847)
All ages (rate per capita)	0.517 (0.510 - 0.525)	0.521 (0.514 - 0.529)	0.541 (0.534 - 0.547)	0.916 (0.852 - 0.982)	0.916 (0.866 - 0.969)	0.950 (0.903 - 1.000)	2.288 (1.395 - 3.464)	2.802 (1.722 - 4.207)	3.287 (2.040 - 4.915)
Age-standardised (rate per capita)	0.541 (0.535 - 0.549)	0.533 (0.527 - 0.540)	0.541 (0.535 - 0.548)	0.946 (0.894 - 0.999)	0.928 (0.880 - 0.979)	0.943 (0.895 - 0.995)	3.483 (2.175 - 5.159)	3.506 (2.193 - 5.189)	3.469 (2.170 - 5.149)
Total number of cases (thousands)	80,042 (78,873 - 81,253)	101,120 (99,895 - 102,640)	115,965 (114,545 - 117,388)	141,825 (131,908 - 151,898)	177,771 (168,099 - 188,193)	203,714 (193,619 - 214,507)	354 (216 - 536)	544 (334 - 817)	705 (438 - 1,054)
**Brazil**									
Under-5 years (rate per capita)	0.315 (0.297 - 0.333)	0.255 (0.242 - 0.268)	0.319 (0.300 - 0.339)	0.540 (0.302 - 0.757)	0.405 (0.241 - 0.589)	0.462 (0.293 - 0.631)	0.075 (0.032 - 0.148)	0.060 (0.026 - 0.120)	0.076 (0.033 - 0.153)
5-14 years (rate per capita)	0.555 (0.534 - 0.575)	0.530 (0.508 - 0.549)	0.546 (0.526 - 0.569)	0.949 (0.776 - 1.141)	0.881 (0.714 - 1.059)	0.935 (0.776 - 1.119)	0.594 (0.330 - 0.974)	0.585 (0.324 - 0.950)	0.593 (0.328 - 0.961)

	Prevalence 1990	Prevalence 2005	Prevalence 2015	Incidence 1990	Incidence 2005	Incidence 2015	YLD rate 1990	YLD rate 2005	YLD rate 2015
15 - 49 years (rate per capita)	0.512 (0.503 - 0.521)	0.520 (0.511 - 0.528)	0.520 (0.512 - 0.528)	0.952 (0.888 - 1.017)	0.963 (0.903 - 1.027)	0.967 (0.905 - 1.026)	1.913 (1.093 - 3.068)	2.023 (1.158 - 3.266)	2.074 (1.181 - 3.343)
50 - 69 years (rate per capita)	0.650 (0.639 - 0.660)	0.648 (0.637 - 0.657)	0.649 (0.639 - 0.659)	1.073 (0.983 - 1.157)	1.078 (0.989 - 1.157)	1.080 (0.996 - 1.165)	8.046 (5.123 - 11.801)	8.071 (5.150 - 11.824)	8.037 (5.130 - 11.797)
70+ years (rate per capita)	0.704 (0.696 - 0.712)	0.702 (0.694 - 0.709)	0.697 (0.690 - 0.704)	1.083 (0.996 - 1.154)	1.082 (1.003 - 1.151)	1.077 (0.997 - 1.141)	13.943 (9.268 - 19.322)	14.056 (9.380 - 19.409)	13.697 (9.103 - 18.970)
All ages (rate per capita)	0.517 (0.510 - 0.525)	0.521 (0.514 - 0.529)	0.541 (0.534 - 0.548)	0.917 (0.854 - 0.982)	0.916 (0.867 - 0.969)	0.951 (0.904 - 1.001)	2.302 (1.404 - 3.484)	2.826 (1.739 - 4.242)	3.321 (2.062 - 4.961)
Age-standardised (rate per capita)	0.541 (0.535 - 0.549)	0.533 (0.526 - 0.540)	0.541 (0.535 - 0.548)	0.947 (0.895 - 0.999)	0.927 (0.880 - 0.978)	0.943 (0.896 - 0.995)	3.501 (2.187 - 5.183)	3.523 (2.204 - 5.212)	3.485 (2.181 - 5.174)
Total number of cases (thousands)	77,893 (76,759 - 79,087)	98,099 (96,881 - 99,565)	112,481 (111,091 - 113,852)	138,106 (128,513 - 147,859)	172,548 (163,231 - 182,555)	197,652 (187,930 - 208,011)	347 (211 - 524)	532 (327 - 799)	690 (429 - 1,031)
**Paraguay**									
Under-5 years (rate per capita)	0.293 (0.245 - 0.336)	0.296 (0.250 - 0.337)	0.296 (0.248 - 0.334)	0.524 (0.287 - 0.759)	0.529 (0.290 - 0.766)	0.527 (0.289 - 0.764)	0.069 (0.029 - 0.138)	0.070 (0.030 - 0.137)	0.069 (0.030 - 0.137)
5-14 years (rate per capita)	0.569 (0.515 - 0.622)	0.567 (0.511 - 0.621)	0.567 (0.513 - 0.622)	0.910 (0.735 - 1.122)	0.902 (0.730 - 1.106)	0.901 (0.729 - 1.104)	0.594 (0.327 - 0.977)	0.596 (0.327 - 0.983)	0.595 (0.331 - 0.974)
15 - 49 years (rate per capita)	0.517 (0.498 - 0.538)	0.519 (0.500 - 0.542)	0.517 (0.499 - 0.541)	0.938 (0.867 - 1.008)	0.938 (0.869 - 1.008)	0.941 (0.872 - 1.012)	1.699 (0.959 - 2.772)	1.727 (0.970 - 2.805)	1.739 (0.983 - 2.845)
50 - 69 years (rate per capita)	0.628 (0.604 - 0.650)	0.626 (0.602 - 0.650)	0.628 (0.605 - 0.650)	1.067 (0.970 - 1.159)	1.065 (0.968 - 1.157)	1.067 (0.969 - 1.159)	6.282 (3.969 - 9.378)	6.107 (3.823 - 9.164)	6.229 (3.905 - 9.326)
70+ years (rate per capita)	0.662 (0.644 - 0.684)	0.655 (0.637 - 0.675)	0.654 (0.637 - 0.673)	1.070 (0.978 - 1.147)	1.062 (0.973 - 1.138)	1.061 (0.971 - 1.137)	10.340 (6.823 - 14.534)	10.119 (6.671 - 14.203)	10.081 (6.659 - 14.148)
All ages (rate per capita)	0.508 (0.487 - 0.527)	0.520 (0.499 - 0.538)	0.524 (0.507 - 0.539)	0.880 (0.804 - 0.958)	0.898 (0.830 - 0.969)	0.911 (0.849 - 0.976)	1.781 (1.062 - 2.751)	1.993 (1.193 - 3.089)	2.230 (1.345 - 3.452)
Age-standardised (rate per capita)	0.537 (0.520 - 0.551)	0.537 (0.521 - 0.553)	0.537 (0.522 - 0.551)	0.932 (0.874 - 0.989)	0.932 (0.874 - 0.989)	0.932 (0.875 - 0.989)	2.827 (1.744 - 4.303)	2.815 (1.722 - 4.280)	2.811 (1.736 - 4.284)
Total number of cases (thousands)	2,149 (2,057 - 2,226)	3,021 (2,902 - 3,128)	3,484 (3,374 - 3,589)	3,719 (3,399 - 4,049)	5,223 (4,824 - 5,631)	6,062 (5,649 - 6,495)	8 (4 - 12)	12 (7 - 18)	15 (9 - 23)
**Southeast Asia, East Asia, and Oceania**									
Under-5 years (rate per capita)	0.440 (0.420 - 0.464)	0.433 (0.410 - 0.458)	0.436 (0.411 - 0.457)	0.596 (0.436 - 0.739)	0.575 (0.406 - 0.728)	0.594 (0.423 - 0.745)	0.108 (0.047 - 0.209)	0.109 (0.048 - 0.211)	0.109 (0.048 - 0.211)
5-14 years (rate per capita)	0.495 (0.475 - 0.513)	0.465 (0.446 - 0.481)	0.466 (0.444 - 0.487)	0.819 (0.649 - 1.041)	0.772 (0.620 - 0.979)	0.813 (0.641 - 1.045)	0.544 (0.308 - 0.882)	0.538 (0.304 - 0.864)	0.543 (0.306 - 0.876)
15 - 49 years (rate per capita)	0.407 (0.397 - 0.416)	0.406 (0.397 - 0.414)	0.413 (0.404 - 0.421)	0.726 (0.660 - 0.797)	0.718 (0.654 - 0.779)	0.731 (0.664 - 0.794)	1.321 (0.762 - 2.096)	1.412 (0.813 - 2.249)	1.492 (0.860 - 2.366)
50 - 69 years (rate per capita)	0.478 (0.467 - 0.487)	0.477 (0.466 - 0.485)	0.480 (0.468 - 0.491)	0.748 (0.664 - 0.837)	0.740 (0.657 - 0.832)	0.750 (0.668 - 0.837)	3.672 (2.206 - 5.657)	3.582 (2.157 - 5.528)	3.672 (2.208 - 5.673)
70+ years (rate per capita)	0.511 (0.503 - 0.520)	0.506 (0.497 - 0.516)	0.502 (0.492 - 0.511)	0.717 (0.641 - 0.801)	0.704 (0.627 - 0.787)	0.698 (0.622 - 0.774)	5.727 (3.659 - 8.401)	5.629 (3.604 - 8.232)	5.653 (3.625 - 8.268)
All ages (rate per capita)	0.439 (0.431 - 0.447)	0.433 (0.425 - 0.439)	0.440 (0.433 - 0.446)	0.732 (0.675 - 0.796)	0.720 (0.670 - 0.772)	0.734 (0.687 - 0.786)	1.445 (0.857 - 2.245)	1.696 (1.010 - 2.636)	1.928 (1.151 - 2.981)
Age-standardised (rate per capita)	0.444 (0.437 - 0.451)	0.439 (0.432 - 0.446)	0.442 (0.434 - 0.449)	0.733 (0.681 - 0.789)	0.722 (0.672 - 0.777)	0.732 (0.681 - 0.791)	1.834 (1.098 - 2.835)	1.821 (1.089 - 2.818)	1.839 (1.098 - 2.844)
Total number of cases (thousands)	730,664 (716,880 - 742,749)	843,113 (827,976 - 855,712)	921,371 (907,383 - 934,800)	1,216,758 (1,122,976 - 1,324,317)	1,402,654 (1,306,258 - 1,503,745)	1,537,684 (1,439,522 - 1,645,946)	2,404 (1,425 - 3,734)	3,305 (1,969 - 5,137)	4,038 (2,410 - 6,245)
**East Asia**									
Under-5 years (rate per capita)	0.454 (0.434 - 0.481)	0.455 (0.436 - 0.480)	0.457 (0.437 - 0.481)	0.581 (0.432 - 0.724)	0.564 (0.407 - 0.711)	0.587 (0.432 - 0.732)	0.108 (0.046 - 0.209)	0.108 (0.047 - 0.210)	0.109 (0.048 - 0.211)
5-14 years (rate per capita)	0.474 (0.453 - 0.493)	0.413 (0.394 - 0.430)	0.416 (0.392 - 0.437)	0.811 (0.643 - 1.026)	0.744 (0.588 - 0.954)	0.800 (0.625 - 1.041)	0.514 (0.293 - 0.819)	0.484 (0.277 - 0.767)	0.491 (0.280 - 0.776)
15 - 49 years (rate per capita)	0.381 (0.373 - 0.390)	0.376 (0.367 - 0.384)	0.382 (0.373 - 0.390)	0.718 (0.652 - 0.787)	0.704 (0.640 - 0.765)	0.718 (0.650 - 0.785)	1.287 (0.743 - 2.051)	1.379 (0.792 - 2.194)	1.470 (0.842 - 2.349)
50 - 69 years (rate per capita)	0.462 (0.452 - 0.471)	0.461 (0.452 - 0.468)	0.464 (0.455 - 0.474)	0.726 (0.645 - 0.812)	0.716 (0.638 - 0.808)	0.727 (0.646 - 0.810)	3.584 (2.151 - 5.520)	3.473 (2.076 - 5.355)	3.596 (2.146 - 5.559)
70+ years (rate per capita)	0.495 (0.487 - 0.506)	0.490 (0.481 - 0.499)	0.485 (0.475 - 0.493)	0.700 (0.621 - 0.786)	0.685 (0.612 - 0.765)	0.678 (0.606 - 0.751)	5.622 (3.593 - 8.285)	5.496 (3.529 - 8.107)	5.509 (3.529 - 8.115)
All ages (rate per capita)	0.419 (0.412 - 0.426)	0.406 (0.400 - 0.412)	0.415 (0.408 - 0.421)	0.720 (0.666 - 0.780)	0.703 (0.654 - 0.754)	0.719 (0.674 - 0.768)	1.452 (0.859 - 2.259)	1.728 (1.025 - 2.681)	1.992 (1.185 - 3.087)
Age-standardised (rate per capita)	0.426 (0.420 - 0.432)	0.415 (0.408 - 0.421)	0.414 (0.407 - 0.422)	0.720 (0.668 - 0.776)	0.706 (0.654 - 0.764)	0.717 (0.666 - 0.777)	1.783 (1.066 - 2.758)	1.759 (1.053 - 2.715)	1.778 (1.062 - 2.747)
Total number of cases (thousands)	500,901 (492,391 - 509,396)	551,671 (543,524 - 560,046)	594,452 (585,233 - 603,868)	860,030 (795,235 - 931,881)	955,302 (888,733 - 1,024,679)	1,029,976 (965,986 - 1,100,998)	1,735 (1,027 - 2,700)	2,349 (1,393 - 3,644)	2,855 (1,697 - 4,423)
**China**									
Under-5 years (rate per capita)	0.455 (0.436 - 0.481)	0.456 (0.437 - 0.480)	0.458 (0.438 - 0.482)	0.582 (0.433 - 0.723)	0.563 (0.407 - 0.709)	0.588 (0.433 - 0.732)	0.108 (0.046 - 0.209)	0.108 (0.047 - 0.211)	0.109 (0.048 - 0.211)
5-14 years (rate per capita)	0.477 (0.456 - 0.495)	0.413 (0.395 - 0.431)	0.417 (0.393 - 0.437)	0.813 (0.645 - 1.025)	0.744 (0.589 - 0.952)	0.802 (0.628 - 1.044)	0.515 (0.294 - 0.822)	0.484 (0.278 - 0.767)	0.492 (0.280 - 0.777)
15 - 49 years (rate per capita)	0.382 (0.373 - 0.391)	0.377 (0.368 - 0.385)	0.383 (0.374 - 0.391)	0.719 (0.653 - 0.788)	0.704 (0.640 - 0.766)	0.719 (0.650 - 0.786)	1.288 (0.743 - 2.054)	1.380 (0.792 - 2.197)	1.474 (0.843 - 2.358)
50 - 69 years (rate per capita)	0.463 (0.453 - 0.472)	0.462 (0.454 - 0.469)	0.466 (0.456 - 0.475)	0.726 (0.645 - 0.812)	0.716 (0.638 - 0.808)	0.727 (0.646 - 0.811)	3.595 (2.157 - 5.540)	3.475 (2.077 - 5.363)	3.607 (2.150 - 5.578)
70+ years (rate per capita)	0.496 (0.487 - 0.506)	0.491 (0.483 - 0.500)	0.486 (0.476 - 0.494)	0.701 (0.622 - 0.786)	0.685 (0.612 - 0.765)	0.679 (0.606 - 0.752)	5.631 (3.599 - 8.299)	5.502 (3.531 - 8.119)	5.519 (3.533 - 8.132)
All ages (rate per capita)	0.420 (0.413 - 0.427)	0.407 (0.400 - 0.413)	0.416 (0.409 - 0.422)	0.720 (0.666 - 0.780)	0.703 (0.654 - 0.754)	0.720 (0.675 - 0.769)	1.453 (0.860 - 2.263)	1.729 (1.025 - 2.685)	1.996 (1.187 - 3.094)
Age-standardised (rate per capita)	0.427 (0.421 - 0.434)	0.416 (0.409 - 0.422)	0.415 (0.408 - 0.422)	0.721 (0.669 - 0.777)	0.706 (0.655 - 0.764)	0.718 (0.667 - 0.777)	1.787 (1.068 - 2.765)	1.762 (1.054 - 2.721)	1.782 (1.063 - 2.754)
Total number of cases (thousands)	485,418 (477,162 - 493,483)	533,729 (525,361 - 541,879)	575,344 (566,366 - 584,370)	831,680 (769,218 - 901,044)	922,760 (858,235 - 990,147)	995,647 (934,046 - 1,064,162)	1,678 (993 - 2,613)	2,270 (1,346 - 3,525)	2,762 (1,642 - 4,281)
**North Korea**									
Under-5 years (rate per capita)	0.415 (0.344 - 0.480)	0.417 (0.344 - 0.486)	0.418 (0.346 - 0.483)	0.575 (0.395 - 0.741)	0.582 (0.400 - 0.750)	0.579 (0.398 - 0.747)	0.098 (0.041 - 0.197)	0.099 (0.042 - 0.197)	0.099 (0.042 - 0.197)
5-14 years (rate per capita)	0.409 (0.355 - 0.464)	0.398 (0.347 - 0.458)	0.395 (0.347 - 0.447)	0.787 (0.593 - 1.060)	0.760 (0.577 - 1.027)	0.753 (0.571 - 1.017)	0.477 (0.274 - 0.755)	0.478 (0.273 - 0.762)	0.478 (0.274 - 0.758)
15 - 49 years (rate per capita)	0.359 (0.340 - 0.380)	0.360 (0.341 - 0.380)	0.362 (0.345 - 0.382)	0.695 (0.628 - 0.766)	0.693 (0.632 - 0.760)	0.696 (0.633 - 0.762)	1.247 (0.733 - 1.992)	1.351 (0.789 - 2.141)	1.373 (0.802 - 2.184)
50 - 69 years (rate per capita)	0.426 (0.403 - 0.453)	0.439 (0.418 - 0.463)	0.430 (0.407 - 0.455)	0.709 (0.622 - 0.803)	0.724 (0.635 - 0.816)	0.712 (0.626 - 0.802)	3.271 (1.975 - 5.044)	3.563 (2.189 - 5.383)	3.365 (2.050 - 5.140)
70+ years (rate per capita)	0.470 (0.448 - 0.495)	0.471 (0.450 - 0.500)	0.470 (0.449 - 0.498)	0.692 (0.608 - 0.784)	0.695 (0.609 - 0.789)	0.692 (0.608 - 0.783)	5.426 (3.532 - 7.907)	5.431 (3.522 - 7.966)	5.428 (3.503 - 7.887)
All ages (rate per capita)	0.385 (0.369 - 0.402)	0.388 (0.373 - 0.404)	0.390 (0.376 - 0.404)	0.701 (0.643 - 0.770)	0.701 (0.646 - 0.761)	0.699 (0.647 - 0.757)	1.390 (0.829 - 2.149)	1.630 (0.978 - 2.507)	1.778 (1.071 - 2.727)
Age-standardised (rate per capita)	0.391 (0.376 - 0.407)	0.391 (0.377 - 0.407)	0.391 (0.377 - 0.407)	0.700 (0.645 - 0.762)	0.699 (0.645 - 0.761)	0.699 (0.644 - 0.761)	1.702 (1.032 - 2.602)	1.702 (1.027 - 2.607)	1.698 (1.028 - 2.598)
Total number of cases (thousands)	7,771 (7,440 - 8,113)	9,213 (8,867 - 9,593)	9,808 (9,456 - 10,176)	14,152 (12,984 - 15,540)	16,652 (15,339 - 18,070)	17,577 (16,286 - 19,050)	28 (17 - 43)	39 (23 - 60)	45 (27 - 69)
**Taiwan**									
Under-5 years (rate per capita)	0.418 (0.341 - 0.484)	0.430 (0.358 - 0.499)	0.415 (0.347 - 0.484)	0.580 (0.399 - 0.748)	0.599 (0.412 - 0.773)	0.576 (0.396 - 0.744)	0.099 (0.041 - 0.197)	0.102 (0.043 - 0.203)	0.098 (0.041 - 0.192)
5-14 years (rate per capita)	0.398 (0.349 - 0.448)	0.397 (0.347 - 0.453)	0.377 (0.331 - 0.428)	0.758 (0.576 - 1.025)	0.755 (0.573 - 1.021)	0.707 (0.531 - 0.962)	0.476 (0.273 - 0.757)	0.478 (0.273 - 0.767)	0.477 (0.271 - 0.759)
15 - 49 years (rate per capita)	0.353 (0.334 - 0.373)	0.357 (0.340 - 0.377)	0.357 (0.339 - 0.378)	0.696 (0.628 - 0.765)	0.696 (0.634 - 0.762)	0.696 (0.633 - 0.763)	1.230 (0.729 - 1.946)	1.347 (0.790 - 2.132)	1.354 (0.794 - 2.126)
50 - 69 years (rate per capita)	0.424 (0.403 - 0.449)	0.421 (0.399 - 0.449)	0.419 (0.397 - 0.444)	0.714 (0.629 - 0.803)	0.709 (0.625 - 0.799)	0.710 (0.622 - 0.802)	3.337 (2.049 - 5.040)	3.257 (1.990 - 4.909)	3.223 (1.981 - 4.908)
70+ years (rate per capita)	0.461 (0.440 - 0.484)	0.453 (0.434 - 0.477)	0.443 (0.424 - 0.466)	0.681 (0.598 - 0.771)	0.662 (0.580 - 0.749)	0.644 (0.572 - 0.722)	5.300 (3.436 - 7.728)	5.254 (3.412 - 7.642)	5.158 (3.348 - 7.456)
All ages (rate per capita)	0.381 (0.366 - 0.396)	0.384 (0.371 - 0.398)	0.384 (0.371 - 0.397)	0.701 (0.642 - 0.768)	0.699 (0.648 - 0.755)	0.691 (0.643 - 0.742)	1.428 (0.862 - 2.190)	1.757 (1.060 - 2.676)	1.989 (1.208 - 3.024)
Age-standardised (rate per capita)	0.387 (0.373 - 0.403)	0.388 (0.374 - 0.403)	0.387 (0.373 - 0.402)	0.697 (0.643 - 0.759)	0.698 (0.643 - 0.760)	0.698 (0.643 - 0.760)	1.654 (1.000 - 2.518)	1.669 (1.008 - 2.535)	1.663 (1.007 - 2.519)
Total number of cases (thousands)	7,712 (7,409 - 8,029)	8,729 (8,430 - 9,044)	9,300 (8,983 - 9,632)	14,197 (13,011 - 15,564)	15,889 (14,726 - 17,163)	16,752 (15,585 - 17,982)	29 (17 - 44)	40 (24 - 61)	48 (29 - 73)
**Southeast Asia**									
Under-5 years (rate per capita)	0.410 (0.375 - 0.446)	0.405 (0.371 - 0.445)	0.408 (0.371 - 0.446)	0.628 (0.448 - 0.784)	0.590 (0.404 - 0.755)	0.606 (0.413 - 0.777)	0.109 (0.048 - 0.214)	0.111 (0.048 - 0.216)	0.111 (0.048 - 0.216)
5-14 years (rate per capita)	0.534 (0.506 - 0.562)	0.549 (0.523 - 0.578)	0.534 (0.507 - 0.566)	0.832 (0.663 - 1.070)	0.818 (0.661 - 1.023)	0.830 (0.666 - 1.060)	0.601 (0.333 - 0.983)	0.626 (0.344 - 1.021)	0.615 (0.338 - 0.998)
15 - 49 years (rate per capita)	0.478 (0.463 - 0.495)	0.478 (0.464 - 0.491)	0.479 (0.465 - 0.493)	0.751 (0.677 - 0.823)	0.754 (0.688 - 0.822)	0.759 (0.693 - 0.826)	1.419 (0.821 - 2.269)	1.497 (0.866 - 2.390)	1.545 (0.900 - 2.475)
50 - 69 years (rate per capita)	0.528 (0.509 - 0.546)	0.528 (0.510 - 0.545)	0.527 (0.508 - 0.545)	0.818 (0.721 - 0.920)	0.819 (0.721 - 0.919)	0.820 (0.722 - 0.923)	3.954 (2.381 - 6.041)	3.949 (2.387 - 6.045)	3.916 (2.368 - 5.996)
70+ years (rate per capita)	0.565 (0.549 - 0.579)	0.562 (0.546 - 0.579)	0.561 (0.547 - 0.578)	0.773 (0.678 - 0.868)	0.768 (0.671 - 0.866)	0.768 (0.672 - 0.862)	6.082 (3.888 - 9.005)	6.085 (3.870 - 9.009)	6.160 (3.923 - 9.102)
All ages (rate per capita)	0.490 (0.479 - 0.502)	0.494 (0.482 - 0.504)	0.493 (0.481 - 0.503)	0.762 (0.700 - 0.833)	0.759 (0.705 - 0.819)	0.767 (0.715 - 0.825)	1.434 (0.844 - 2.215)	1.628 (0.959 - 2.523)	1.796 (1.064 - 2.774)
Age-standardised (rate per capita)	0.495 (0.485 - 0.505)	0.498 (0.487 - 0.508)	0.497 (0.486 - 0.507)	0.770 (0.717 - 0.826)	0.765 (0.713 - 0.820)	0.769 (0.717 - 0.825)	1.984 (1.184 - 3.027)	1.994 (1.192 - 3.050)	1.997 (1.195 - 3.048)
Total number of cases (thousands)	226,176 (220,885 - 231,527)	286,489 (280,065 - 292,582)	320,904 (313,468 - 327,612)	351,876 (323,295 - 384,351)	440,606 (409,523 - 475,337)	499,550 (465,377 - 536,973)	662 (389 - 1,022)	945 (557 - 1,464)	1,169 (693 - 1,806)
**Cambodia**									
Under-5 years (rate per capita)	0.448 (0.388 - 0.500)	0.451 (0.392 - 0.502)	0.453 (0.395 - 0.502)	0.614 (0.450 - 0.758)	0.624 (0.460 - 0.768)	0.619 (0.454 - 0.764)	0.106 (0.046 - 0.206)	0.107 (0.045 - 0.204)	0.107 (0.046 - 0.206)
5-14 years (rate per capita)	0.567 (0.512 - 0.624)	0.563 (0.517 - 0.618)	0.553 (0.501 - 0.611)	0.879 (0.690 - 1.136)	0.791 (0.632 - 1.009)	0.814 (0.643 - 1.047)	0.560 (0.314 - 0.920)	0.581 (0.323 - 0.951)	0.569 (0.317 - 0.920)

	Prevalence 1990	Prevalence 2005	Prevalence 2015	Incidence 1990	Incidence 2005	Incidence 2015	YLD rate 1990	YLD rate 2005	YLD rate 2015
15 - 49 years (rate per capita)	0.484 (0.460 - 0.512)	0.485 (0.460 - 0.511)	0.481 (0.458 - 0.509)	0.728 (0.661 - 0.803)	0.723 (0.658 - 0.795)	0.734 (0.667 - 0.811)	1.327 (0.777 - 2.114)	1.322 (0.772 - 2.097)	1.366 (0.797 - 2.177)
50 - 69 years (rate per capita)	0.514 (0.483 - 0.543)	0.515 (0.480 - 0.546)	0.515 (0.481 - 0.545)	0.812 (0.725 - 0.908)	0.811 (0.718 - 0.910)	0.813 (0.724 - 0.910)	3.801 (2.291 - 5.700)	3.814 (2.305 - 5.782)	3.831 (2.296 - 5.844)
70+ years (rate per capita)	0.579 (0.550 - 0.609)	0.575 (0.547 - 0.610)	0.572 (0.541 - 0.604)	0.778 (0.682 - 0.881)	0.776 (0.679 - 0.870)	0.768 (0.678 - 0.866)	6.398 (3.900 - 9.477)	6.357 (3.890 - 9.377)	6.396 (3.922 - 9.496)
All ages (rate per capita)	0.502 (0.481 - 0.523)	0.505 (0.486 - 0.525)	0.499 (0.480 - 0.517)	0.752 (0.681 - 0.829)	0.738 (0.677 - 0.807)	0.747 (0.689 - 0.810)	1.191 (0.702 - 1.837)	1.319 (0.784 - 2.027)	1.474 (0.882 - 2.253)
Age-standardised (rate per capita)	0.502 (0.485 - 0.519)	0.504 (0.487 - 0.520)	0.501 (0.484 - 0.519)	0.753 (0.701 - 0.810)	0.756 (0.705 - 0.810)	0.753 (0.700 - 0.811)	1.932 (1.188 - 2.904)	1.937 (1.188 - 2.906)	1.946 (1.189 - 2.929)
Total number of cases (thousands)	4,524 (4,333 - 4,715)	6,761 (6,504 - 7,023)	7,775 (7,487 - 8,067)	6,775 (6,135 - 7,469)	9,873 (9,059 - 10,794)	11,650 (10,745 - 12,622)	11 (6 - 17)	18 (10 - 27)	23 (14 - 35)
**Indonesia**									
Under-5 years (rate per capita)	0.417 (0.357 - 0.480)	0.401 (0.333 - 0.470)	0.407 (0.341 - 0.476)	0.667 (0.496 - 0.796)	0.598 (0.398 - 0.770)	0.603 (0.401 - 0.777)	0.099 (0.042 - 0.196)	0.095 (0.039 - 0.190)	0.096 (0.041 - 0.190)
5-14 years (rate per capita)	0.548 (0.498 - 0.601)	0.535 (0.487 - 0.589)	0.533 (0.482 - 0.589)	0.792 (0.624 - 1.026)	0.793 (0.631 - 1.017)	0.789 (0.627 - 1.012)	0.568 (0.314 - 0.942)	0.564 (0.314 - 0.912)	0.564 (0.311 - 0.913)
15 - 49 years (rate per capita)	0.488 (0.466 - 0.510)	0.488 (0.465 - 0.513)	0.488 (0.466 - 0.514)	0.731 (0.665 - 0.807)	0.737 (0.673 - 0.806)	0.740 (0.674 - 0.808)	1.473 (0.850 - 2.392)	1.557 (0.893 - 2.520)	1.623 (0.937 - 2.649)
50 - 69 years (rate per capita)	0.553 (0.522 - 0.583)	0.554 (0.524 - 0.583)	0.551 (0.521 - 0.585)	0.812 (0.719 - 0.918)	0.811 (0.717 - 0.913)	0.809 (0.714 - 0.915)	4.325 (2.568 - 6.709)	4.347 (2.597 - 6.763)	4.272 (2.525 - 6.633)
70+ years (rate per capita)	0.599 (0.572 - 0.630)	0.597 (0.568 - 0.627)	0.596 (0.570 - 0.627)	0.768 (0.674 - 0.869)	0.765 (0.671 - 0.861)	0.764 (0.670 - 0.860)	6.787 (4.093 - 10.279)	6.777 (4.099 - 10.156)	6.792 (4.103 - 10.222)
All ages (rate per capita)	0.503 (0.486 - 0.520)	0.500 (0.482 - 0.517)	0.501 (0.483 - 0.518)	0.747 (0.686 - 0.814)	0.744 (0.687 - 0.809)	0.746 (0.691 - 0.807)	1.505 (0.872 - 2.349)	1.690 (0.983 - 2.653)	1.838 (1.072 - 2.858)
Age-standardised (rate per capita)	0.510 (0.494 - 0.525)	0.507 (0.491 - 0.523)	0.507 (0.490 - 0.523)	0.755 (0.702 - 0.812)	0.750 (0.696 - 0.807)	0.750 (0.696 - 0.807)	2.136 (1.268 - 3.296)	2.130 (1.263 - 3.292)	2.131 (1.265 - 3.289)
Total number of cases (thousands)	91,203 (88,164 - 94,338)	113,215 (109,165 - 117,130)	129,155 (124,509 - 133,382)	135,418 (124,356 - 147,535)	168,604 (155,694 - 183,273)	192,298 (177,894 - 207,853)	273 (158 - 426)	383 (223 - 601)	473 (276 - 736)
**Laos**									
Under-5 years (rate per capita)	0.411 (0.348 - 0.476)	0.445 (0.388 - 0.499)	0.422 (0.357 - 0.487)	0.592 (0.410 - 0.746)	0.647 (0.483 - 0.774)	0.608 (0.421 - 0.766)	0.097 (0.041 - 0.191)	0.105 (0.044 - 0.202)	0.100 (0.042 - 0.194)
5-14 years (rate per capita)	0.510 (0.437 - 0.577)	0.508 (0.431 - 0.571)	0.509 (0.441 - 0.572)	0.891 (0.668 - 1.157)	0.872 (0.647 - 1.144)	0.884 (0.662 - 1.147)	0.540 (0.305 - 0.894)	0.544 (0.304 - 0.907)	0.543 (0.306 - 0.887)
15 - 49 years (rate per capita)	0.451 (0.399 - 0.506)	0.450 (0.398 - 0.502)	0.450 (0.400 - 0.496)	0.767 (0.617 - 0.894)	0.767 (0.619 - 0.894)	0.770 (0.623 - 0.895)	1.188 (0.696 - 1.871)	1.197 (0.700 - 1.874)	1.213 (0.707 - 1.907)
50 - 69 years (rate per capita)	0.488 (0.436 - 0.542)	0.489 (0.435 - 0.542)	0.487 (0.436 - 0.549)	0.808 (0.620 - 0.998)	0.807 (0.621 - 0.994)	0.807 (0.616 - 0.995)	3.108 (1.922 - 4.736)	3.070 (1.878 - 4.642)	3.068 (1.888 - 4.654)
70+ years (rate per capita)	0.527 (0.472 - 0.586)	0.523 (0.470 - 0.581)	0.522 (0.471 - 0.575)	0.783 (0.588 - 0.982)	0.778 (0.588 - 0.975)	0.775 (0.587 - 0.967)	4.915 (3.185 - 7.099)	4.879 (3.182 - 7.042)	4.890 (3.166 - 7.124)
All ages (rate per capita)	0.465 (0.432 - 0.500)	0.470 (0.438 - 0.504)	0.465 (0.434 - 0.497)	0.774 (0.685 - 0.875)	0.784 (0.694 - 0.884)	0.779 (0.691 - 0.873)	1.074 (0.639 - 1.653)	1.118 (0.662 - 1.718)	1.194 (0.710 - 1.845)
Age-standardised (rate per capita)	0.468 (0.439 - 0.499)	0.471 (0.443 - 0.499)	0.468 (0.439 - 0.496)	0.783 (0.705 - 0.863)	0.787 (0.708 - 0.867)	0.783 (0.705 - 0.863)	1.618 (0.985 - 2.455)	1.617 (0.978 - 2.437)	1.623 (0.986 - 2.466)
Total number of cases (thousands)	1,964 (1,826 - 2,110)	2,697 (2,511 - 2,891)	3,161 (2,949 - 3,377)	3,271 (2,893 - 3,695)	4,497 (3,979 - 5,070)	5,299 (4,700 - 5,936)	5 (3 - 7)	6 (4 - 10)	8 (5 - 13)
**Malaysia**									
Under-5 years (rate per capita)	0.379 (0.321 - 0.449)	0.392 (0.331 - 0.461)	0.380 (0.323 - 0.452)	0.587 (0.369 - 0.769)	0.601 (0.378 - 0.787)	0.590 (0.371 - 0.772)	0.090 (0.038 - 0.180)	0.092 (0.040 - 0.187)	0.091 (0.039 - 0.182)
5-14 years (rate per capita)	0.455 (0.404 - 0.514)	0.433 (0.385 - 0.482)	0.444 (0.395 - 0.500)	0.686 (0.520 - 0.920)	0.642 (0.487 - 0.864)	0.647 (0.495 - 0.877)	0.502 (0.286 - 0.809)	0.491 (0.279 - 0.784)	0.507 (0.287 - 0.821)
15 - 49 years (rate per capita)	0.355 (0.332 - 0.382)	0.357 (0.335 - 0.381)	0.355 (0.333 - 0.379)	0.482 (0.424 - 0.545)	0.482 (0.425 - 0.547)	0.484 (0.428 - 0.546)	1.355 (0.807 - 2.109)	1.412 (0.839 - 2.206)	1.425 (0.850 - 2.209)
50 - 69 years (rate per capita)	0.431 (0.399 - 0.463)	0.430 (0.400 - 0.467)	0.431 (0.401 - 0.461)	0.516 (0.446 - 0.601)	0.515 (0.442 - 0.601)	0.517 (0.447 - 0.601)	4.165 (2.595 - 6.324)	4.163 (2.625 - 6.370)	4.199 (2.631 - 6.423)
70+ years (rate per capita)	0.499 (0.472 - 0.528)	0.497 (0.471 - 0.527)	0.495 (0.469 - 0.523)	0.479 (0.418 - 0.545)	0.481 (0.418 - 0.551)	0.477 (0.416 - 0.542)	7.011 (4.627 - 9.995)	7.012 (4.653 - 10.034)	6.960 (4.606 - 9.888)
All ages (rate per capita)	0.392 (0.371 - 0.411)	0.388 (0.371 - 0.406)	0.388 (0.370 - 0.403)	0.548 (0.486 - 0.621)	0.530 (0.476 - 0.589)	0.524 (0.474 - 0.578)	1.373 (0.837 - 2.101)	1.582 (0.971 - 2.438)	1.781 (1.088 - 2.729)
Age-standardised (rate per capita)	0.397 (0.378 - 0.413)	0.395 (0.378 - 0.412)	0.397 (0.380 - 0.412)	0.531 (0.480 - 0.587)	0.525 (0.477 - 0.578)	0.531 (0.480 - 0.587)	2.033 (1.260 - 3.072)	2.036 (1.266 - 3.074)	2.030 (1.259 - 3.061)
Total number of cases (thousands)	7,125 (6,732 - 7,468)	9,981 (9,531 - 10,435)	11,754 (11,214 - 12,219)	9,947 (8,824 - 11,279)	13,621 (12,246 - 15,148)	15,883 (14,360 - 17,526)	25 (15 - 38)	41 (25 - 63)	54 (33 - 83)
**Maldives**									
Under-5 years (rate per capita)	0.417 (0.342 - 0.492)	0.418 (0.347 - 0.490)	0.418 (0.345 - 0.494)	0.602 (0.391 - 0.777)	0.604 (0.392 - 0.780)	0.603 (0.392 - 0.778)	0.098 (0.041 - 0.195)	0.099 (0.043 - 0.195)	0.099 (0.041 - 0.197)
5-14 years (rate per capita)	0.498 (0.428 - 0.561)	0.486 (0.415 - 0.550)	0.492 (0.426 - 0.553)	0.920 (0.680 - 1.219)	0.859 (0.626 - 1.141)	0.894 (0.659 - 1.182)	0.535 (0.302 - 0.876)	0.541 (0.305 - 0.897)	0.539 (0.304 - 0.879)
15 - 49 years (rate per capita)	0.458 (0.407 - 0.506)	0.457 (0.404 - 0.506)	0.456 (0.401 - 0.503)	0.768 (0.617 - 0.896)	0.768 (0.622 - 0.894)	0.777 (0.639 - 0.907)	1.301 (0.757 - 2.070)	1.331 (0.778 - 2.105)	1.414 (0.825 - 2.258)
50 - 69 years (rate per capita)	0.522 (0.468 - 0.577)	0.528 (0.480 - 0.583)	0.518 (0.469 - 0.577)	0.809 (0.617 - 0.999)	0.813 (0.624 - 0.993)	0.809 (0.617 - 1.000)	3.883 (2.313 - 6.067)	4.180 (2.485 - 6.456)	3.915 (2.331 - 6.062)
70+ years (rate per capita)	0.575 (0.515 - 0.634)	0.570 (0.514 - 0.630)	0.565 (0.517 - 0.618)	0.792 (0.584 - 1.005)	0.785 (0.587 - 0.985)	0.773 (0.585 - 0.960)	6.324 (3.909 - 9.447)	6.361 (3.890 - 9.494)	6.471 (3.984 - 9.580)
All ages (rate per capita)	0.469 (0.435 - 0.503)	0.469 (0.435 - 0.505)	0.469 (0.433 - 0.500)	0.783 (0.686 - 0.890)	0.778 (0.686 - 0.875)	0.783 (0.697 - 0.868)	1.172 (0.701 - 1.824)	1.393 (0.831 - 2.147)	1.577 (0.940 - 2.430)
Age-standardised (rate per capita)	0.479 (0.449 - 0.509)	0.478 (0.450 - 0.510)	0.478 (0.448 - 0.508)	0.785 (0.706 - 0.867)	0.785 (0.706 - 0.867)	0.786 (0.706 - 0.867)	1.954 (1.168 - 2.967)	1.976 (1.174 - 3.026)	1.987 (1.186 - 3.021)
Total number of cases (thousands)	105 (97 - 113)	143 (133 - 154)	170 (157 - 182)	175 (153 - 199)	238 (209 - 267)	284 (253 - 315)	0 (0 - 0)	0 (0 - 1)	1 (0 - 1)
**Mauritius**									
Under-5 years (rate per capita)	0.412 (0.341 - 0.483)	0.420 (0.347 - 0.497)	0.419 (0.341 - 0.494)	0.619 (0.396 - 0.800)	0.632 (0.404 - 0.817)	0.629 (0.402 - 0.813)	0.159 (0.067 - 0.323)	0.162 (0.069 - 0.329)	0.162 (0.067 - 0.334)
5-14 years (rate per capita)	0.485 (0.419 - 0.545)	0.482 (0.412 - 0.541)	0.480 (0.408 - 0.546)	0.904 (0.660 - 1.202)	0.895 (0.653 - 1.191)	0.888 (0.643 - 1.181)	0.690 (0.372 - 1.176)	0.692 (0.374 - 1.183)	0.693 (0.370 - 1.194)
15 - 49 years (rate per capita)	0.452 (0.403 - 0.499)	0.454 (0.409 - 0.500)	0.455 (0.412 - 0.500)	0.811 (0.666 - 0.943)	0.816 (0.677 - 0.951)	0.815 (0.674 - 0.948)	1.711 (1.003 - 2.752)	1.863 (1.105 - 2.963)	1.840 (1.089 - 2.936)
50 - 69 years (rate per capita)	0.532 (0.486 - 0.585)	0.527 (0.477 - 0.578)	0.533 (0.487 - 0.584)	0.849 (0.652 - 1.045)	0.846 (0.648 - 1.045)	0.849 (0.652 - 1.046)	5.025 (3.179 - 7.558)	4.899 (3.042 - 7.367)	4.988 (3.150 - 7.537)
70+ years (rate per capita)	0.593 (0.546 - 0.640)	0.584 (0.541 - 0.630)	0.580 (0.535 - 0.622)	0.819 (0.620 - 1.029)	0.803 (0.616 - 1.003)	0.801 (0.616 - 0.998)	8.619 (5.663 - 12.172)	8.423 (5.551 - 11.845)	8.307 (5.502 - 11.765)
All ages (rate per capita)	0.467 (0.436 - 0.498)	0.473 (0.446 - 0.504)	0.481 (0.454 - 0.512)	0.815 (0.722 - 0.909)	0.820 (0.728 - 0.907)	0.821 (0.731 - 0.911)	1.923 (1.162 - 2.979)	2.299 (1.404 - 3.518)	2.670 (1.644 - 4.032)
Age-standardised (rate per capita)	0.478 (0.451 - 0.507)	0.478 (0.451 - 0.508)	0.478 (0.452 - 0.509)	0.819 (0.732 - 0.905)	0.819 (0.732 - 0.905)	0.819 (0.732 - 0.905)	2.487 (1.537 - 3.760)	2.491 (1.533 - 3.768)	2.467 (1.521 - 3.731)
Total number of cases (thousands)	497 (464 - 529)	578 (544 - 615)	613 (578 - 652)	866 (768 - 966)	1,001 (889 - 1,108)	1,046 (931 - 1,161)	2 (1 - 3)	3 (2 - 4)	3 (2 - 5)
**Myanmar**									
Under-5 years (rate per capita)	0.306 (0.254 - 0.362)	0.282 (0.226 - 0.344)	0.309 (0.254 - 0.366)	0.558 (0.308 - 0.789)	0.439 (0.242 - 0.715)	0.562 (0.310 - 0.794)	0.072 (0.030 - 0.149)	0.066 (0.027 - 0.138)	0.073 (0.031 - 0.149)
5-14 years (rate per capita)	0.434 (0.386 - 0.485)	0.404 (0.355 - 0.450)	0.431 (0.381 - 0.481)	0.742 (0.579 - 0.968)	0.719 (0.546 - 0.944)	0.725 (0.566 - 0.944)	0.499 (0.282 - 0.802)	0.491 (0.279 - 0.781)	0.503 (0.284 - 0.804)
15 - 49 years (rate per capita)	0.404 (0.379 - 0.430)	0.402 (0.378 - 0.426)	0.400 (0.377 - 0.425)	0.689 (0.618 - 0.763)	0.693 (0.623 - 0.765)	0.693 (0.625 - 0.763)	1.214 (0.698 - 1.940)	1.272 (0.735 - 2.039)	1.309 (0.751 - 2.098)
50 - 69 years (rate per capita)	0.450 (0.417 - 0.487)	0.446 (0.414 - 0.484)	0.446 (0.413 - 0.486)	0.739 (0.652 - 0.836)	0.736 (0.646 - 0.830)	0.736 (0.648 - 0.840)	3.218 (1.928 - 5.151)	3.127 (1.893 - 5.058)	3.147 (1.879 - 5.093)
70+ years (rate per capita)	0.492 (0.459 - 0.531)	0.490 (0.456 - 0.527)	0.486 (0.454 - 0.522)	0.703 (0.618 - 0.795)	0.697 (0.614 - 0.792)	0.693 (0.611 - 0.781)	4.842 (3.137 - 7.137)	4.780 (3.081 - 7.029)	4.800 (3.090 - 7.059)
All ages (rate per capita)	0.406 (0.386 - 0.424)	0.397 (0.379 - 0.414)	0.408 (0.390 - 0.426)	0.691 (0.620 - 0.774)	0.676 (0.613 - 0.750)	0.694 (0.634 - 0.763)	1.183 (0.696 - 1.891)	1.306 (0.767 - 2.081)	1.439 (0.849 - 2.296)
Age-standardised (rate per capita)	0.411 (0.394 - 0.429)	0.404 (0.388 - 0.420)	0.411 (0.394 - 0.429)	0.696 (0.637 - 0.761)	0.682 (0.626 - 0.748)	0.696 (0.638 - 0.761)	1.628 (0.975 - 2.575)	1.620 (0.973 - 2.556)	1.628 (0.976 - 2.582)
Total number of cases (thousands)	17,073 (16,241 - 17,840)	19,840 (18,938 - 20,681)	22,033 (21,078 - 22,997)	29,052 (26,052 - 32,550)	33,748 (30,631 - 37,447)	37,514 (34,276 - 41,241)	50 (29 - 80)	65 (38 - 104)	78 (46 - 124)
**Philippines**									
Under-5 years (rate per capita)	0.457 (0.399 - 0.504)	0.476 (0.412 - 0.523)	0.461 (0.403 - 0.507)	0.643 (0.472 - 0.798)	0.638 (0.470 - 0.791)	0.648 (0.476 - 0.805)	0.176 (0.077 - 0.348)	0.183 (0.078 - 0.359)	0.178 (0.077 - 0.348)
5-14 years (rate per capita)	0.652 (0.608 - 0.696)	0.765 (0.739 - 0.790)	0.653 (0.610 - 0.695)	1.014 (0.821 - 1.258)	0.972 (0.818 - 1.155)	1.004 (0.817 - 1.247)	0.878 (0.463 - 1.493)	0.967 (0.502 - 1.667)	0.884 (0.465 - 1.513)
15 - 49 years (rate per capita)	0.586 (0.567 - 0.608)	0.585 (0.568 - 0.606)	0.584 (0.564 - 0.604)	0.929 (0.868 - 0.991)	0.932 (0.872 - 0.997)	0.937 (0.877 - 0.998)	1.615 (0.940 - 2.616)	1.662 (0.972 - 2.660)	1.674 (0.972 - 2.710)
50 - 69 years (rate per capita)	0.595 (0.571 - 0.620)	0.595 (0.572 - 0.617)	0.595 (0.569 - 0.617)	1.054 (0.969 - 1.128)	1.053 (0.965 - 1.126)	1.054 (0.969 - 1.128)	4.054 (2.581 - 5.980)	3.993 (2.540 - 5.924)	4.031 (2.565 - 5.947)
70+ years (rate per capita)	0.643 (0.624 - 0.663)	0.642 (0.623 - 0.662)	0.641 (0.622 - 0.663)	1.020 (0.955 - 1.082)	1.019 (0.953 - 1.087)	1.019 (0.955 - 1.081)	6.869 (4.548 - 9.763)	6.843 (4.511 - 9.687)	6.856 (4.528 - 9.789)
All ages (rate per capita)	0.585 (0.566 - 0.602)	0.616 (0.601 - 0.630)	0.587 (0.570 - 0.602)	0.919 (0.851 - 0.993)	0.916 (0.858 - 0.979)	0.935 (0.876 - 0.998)	1.498 (0.895 - 2.366)	1.633 (0.978 - 2.569)	1.778 (1.070 - 2.783)
Age-standardised (rate per capita)	0.589 (0.574 - 0.603)	0.610 (0.598 - 0.623)	0.589 (0.573 - 0.602)	0.952 (0.900 - 1.007)	0.944 (0.893 - 0.995)	0.952 (0.900 - 1.007)	2.201 (1.355 - 3.353)	2.223 (1.371 - 3.386)	2.201 (1.353 - 3.360)
Total number of cases (thousands)	36,217 (35,030 - 37,256)	53,025 (51,725 - 54,278)	59,180 (57,443 - 60,655)	56,866 (52,688 - 61,434)	78,908 (73,905 - 84,311)	94,293 (88,294 - 100,561)	93 (55 - 146)	141 (84 - 221)	179 (108 - 281)
**Sri Lanka**									
Under-5 years (rate per capita)	0.311 (0.266 - 0.354)	0.257 (0.229 - 0.287)	0.259 (0.230 - 0.289)	0.583 (0.327 - 0.814)	0.472 (0.310 - 0.663)	0.478 (0.314 - 0.672)	0.119 (0.050 - 0.244)	0.099 (0.042 - 0.197)	0.100 (0.042 - 0.197)
5-14 years (rate per capita)	0.480 (0.410 - 0.541)	0.477 (0.406 - 0.535)	0.478 (0.410 - 0.540)	0.886 (0.658 - 1.164)	0.883 (0.651 - 1.141)	0.888 (0.657 - 1.146)	0.681 (0.365 - 1.154)	0.684 (0.368 - 1.173)	0.684 (0.372 - 1.187)
15 - 49 years (rate per capita)	0.449 (0.405 - 0.494)	0.450 (0.402 - 0.496)	0.451 (0.405 - 0.495)	0.811 (0.664 - 0.942)	0.813 (0.671 - 0.947)	0.814 (0.673 - 0.948)	1.524 (0.873 - 2.499)	1.590 (0.912 - 2.584)	1.623 (0.934 - 2.653)

	Prevalence 1990	Prevalence 2005	Prevalence 2015	Incidence 1990	Incidence 2005	Incidence 2015	YLD rate 1990	YLD rate 2005	YLD rate 2015
50 - 69 years (rate per capita)	0.515 (0.469 - 0.574)	0.513 (0.464 - 0.568)	0.516 (0.467 - 0.573)	0.845 (0.648 - 1.043)	0.845 (0.647 - 1.042)	0.847 (0.648 - 1.044)	3.845 (2.344 - 6.020)	3.784 (2.287 - 5.945)	3.877 (2.353 - 6.069)
70+ years (rate per capita)	0.549 (0.499 - 0.599)	0.543 (0.494 - 0.595)	0.545 (0.498 - 0.595)	0.805 (0.609 - 1.008)	0.798 (0.609 - 0.998)	0.801 (0.613 - 0.998)	5.830 (3.775 - 8.478)	5.785 (3.769 - 8.378)	5.783 (3.771 - 8.354)
All ages (rate per capita)	0.453 (0.425 - 0.483)	0.451 (0.422 - 0.483)	0.458 (0.430 - 0.489)	0.808 (0.715 - 0.905)	0.799 (0.707 - 0.884)	0.805 (0.717 - 0.892)	1.614 (0.950 - 2.585)	1.840 (1.094 - 2.938)	2.021 (1.207 - 3.197)
Age-standardised (rate per capita)	0.460 (0.434 - 0.489)	0.455 (0.426 - 0.486)	0.455 (0.428 - 0.486)	0.809 (0.719 - 0.895)	0.800 (0.714 - 0.884)	0.800 (0.714 - 0.884)	1.980 (1.186 - 3.126)	1.978 (1.190 - 3.129)	1.981 (1.190 - 3.142)
Total number of cases (thousands)	7,846 (7,363 - 8,373)	8,815 (8,240 - 9,445)	9,510 (8,927 - 10,147)	14,005 (12,394 - 15,695)	15,604 (13,819 - 17,266)	16,706 (14,880 - 18,508)	28 (16 - 45)	36 (21 - 57)	42 (25 - 66)
**Seychelles**									
Under-5 years (rate per capita)	0.420 (0.344 - 0.493)	0.414 (0.334 - 0.486)	0.424 (0.343 - 0.498)	0.608 (0.395 - 0.785)	0.599 (0.389 - 0.773)	0.611 (0.396 - 0.788)	0.100 (0.042 - 0.198)	0.098 (0.042 - 0.195)	0.100 (0.043 - 0.206)
5-14 years (rate per capita)	0.490 (0.422 - 0.554)	0.491 (0.418 - 0.552)	0.496 (0.426 - 0.560)	0.886 (0.649 - 1.171)	0.875 (0.640 - 1.158)	0.903 (0.663 - 1.195)	0.541 (0.302 - 0.892)	0.542 (0.304 - 0.893)	0.539 (0.301 - 0.867)
15 - 49 years (rate per capita)	0.455 (0.402 - 0.509)	0.456 (0.413 - 0.502)	0.457 (0.413 - 0.499)	0.771 (0.620 - 0.901)	0.779 (0.648 - 0.907)	0.781 (0.650 - 0.911)	1.310 (0.758 - 2.077)	1.484 (0.867 - 2.366)	1.536 (0.901 - 2.461)
50 - 69 years (rate per capita)	0.527 (0.480 - 0.587)	0.522 (0.475 - 0.578)	0.521 (0.468 - 0.578)	0.814 (0.619 - 1.003)	0.809 (0.626 - 0.987)	0.809 (0.614 - 1.002)	4.192 (2.496 - 6.475)	3.970 (2.348 - 6.159)	3.912 (2.307 - 6.009)
70+ years (rate per capita)	0.559 (0.513 - 0.607)	0.561 (0.514 - 0.608)	0.556 (0.512 - 0.606)	0.764 (0.589 - 0.944)	0.768 (0.590 - 0.953)	0.760 (0.588 - 0.937)	6.641 (4.049 - 9.740)	6.627 (4.055 - 9.855)	6.584 (4.027 - 9.726)
All ages (rate per capita)	0.472 (0.440 - 0.506)	0.471 (0.441 - 0.503)	0.476 (0.449 - 0.508)	0.784 (0.695 - 0.880)	0.781 (0.694 - 0.866)	0.788 (0.707 - 0.872)	1.544 (0.921 - 2.335)	1.748 (1.056 - 2.695)	1.944 (1.171 - 2.970)
Age-standardised (rate per capita)	0.478 (0.450 - 0.509)	0.478 (0.451 - 0.510)	0.479 (0.452 - 0.509)	0.786 (0.706 - 0.868)	0.786 (0.706 - 0.868)	0.786 (0.706 - 0.867)	2.003 (1.192 - 3.046)	1.997 (1.196 - 3.054)	1.993 (1.187 - 3.031)
Total number of cases (thousands)	33 (31 - 36)	42 (39 - 45)	46 (43 - 49)	56 (49 - 62)	69 (62 - 77)	76 (68 - 84)	0 (0 - 0)	0 (0 - 0)	0 (0 - 0)
**Thailand**									
Under-5 years (rate per capita)	0.422 (0.345 - 0.493)	0.422 (0.340 - 0.495)	0.422 (0.345 - 0.493)	0.609 (0.395 - 0.785)	0.610 (0.396 - 0.787)	0.612 (0.397 - 0.789)	0.100 (0.043 - 0.200)	0.100 (0.043 - 0.198)	0.100 (0.042 - 0.196)
5-14 years (rate per capita)	0.488 (0.417 - 0.552)	0.487 (0.416 - 0.552)	0.491 (0.423 - 0.555)	0.875 (0.639 - 1.157)	0.875 (0.640 - 1.158)	0.877 (0.643 - 1.160)	0.540 (0.302 - 0.881)	0.541 (0.303 - 0.884)	0.540 (0.302 - 0.896)
15 - 49 years (rate per capita)	0.462 (0.415 - 0.514)	0.464 (0.417 - 0.509)	0.465 (0.422 - 0.511)	0.774 (0.632 - 0.899)	0.780 (0.649 - 0.909)	0.782 (0.653 - 0.916)	1.438 (0.823 - 2.294)	1.599 (0.921 - 2.575)	1.656 (0.964 - 2.699)
50 - 69 years (rate per capita)	0.534 (0.484 - 0.586)	0.535 (0.491 - 0.582)	0.536 (0.487 - 0.589)	0.812 (0.621 - 1.000)	0.811 (0.626 - 0.996)	0.813 (0.621 - 1.002)	4.148 (2.471 - 6.436)	4.160 (2.490 - 6.449)	4.179 (2.503 - 6.453)
70+ years (rate per capita)	0.570 (0.525 - 0.620)	0.571 (0.524 - 0.621)	0.563 (0.520 - 0.610)	0.773 (0.590 - 0.959)	0.775 (0.590 - 0.964)	0.766 (0.587 - 0.948)	6.620 (4.053 - 9.969)	6.615 (4.039 - 9.949)	6.586 (4.008 - 9.876)
All ages (rate per capita)	0.475 (0.446 - 0.507)	0.482 (0.452 - 0.513)	0.489 (0.460 - 0.520)	0.783 (0.694 - 0.875)	0.789 (0.705 - 0.874)	0.790 (0.707 - 0.875)	1.588 (0.940 - 2.456)	2.008 (1.198 - 3.108)	2.358 (1.415 - 3.635)
Age-standardised (rate per capita)	0.484 (0.458 - 0.514)	0.484 (0.457 - 0.513)	0.484 (0.457 - 0.514)	0.786 (0.707 - 0.867)	0.786 (0.707 - 0.867)	0.786 (0.706 - 0.867)	2.053 (1.223 - 3.140)	2.055 (1.227 - 3.158)	2.049 (1.231 - 3.143)
Total number of cases (thousands)	26,911 (25,268 - 28,747)	31,658 (29,711 - 33,668)	33,206 (31,245 - 35,293)	44,379 (39,285 - 49,546)	51,820 (46,293 - 57,437)	53,659 (47,979 - 59,411)	90 (53 - 139)	132 (79 - 204)	160 (96 - 247)
**Timor-Leste**									
Under-5 years (rate per capita)	0.404 (0.335 - 0.474)	0.413 (0.336 - 0.481)	0.414 (0.340 - 0.487)	0.586 (0.380 - 0.756)	0.599 (0.388 - 0.773)	0.602 (0.390 - 0.777)	0.095 (0.040 - 0.194)	0.098 (0.041 - 0.194)	0.098 (0.042 - 0.194)
5-14 years (rate per capita)	0.500 (0.428 - 0.566)	0.495 (0.424 - 0.557)	0.490 (0.422 - 0.551)	0.926 (0.684 - 1.226)	0.907 (0.669 - 1.201)	0.882 (0.646 - 1.167)	0.534 (0.303 - 0.859)	0.536 (0.300 - 0.878)	0.539 (0.303 - 0.890)
15 - 49 years (rate per capita)	0.456 (0.404 - 0.511)	0.458 (0.406 - 0.510)	0.457 (0.406 - 0.508)	0.771 (0.623 - 0.897)	0.766 (0.620 - 0.894)	0.769 (0.621 - 0.895)	1.338 (0.780 - 2.125)	1.328 (0.770 - 2.104)	1.333 (0.774 - 2.104)
50 - 69 years (rate per capita)	0.519 (0.470 - 0.576)	0.523 (0.474 - 0.584)	0.530 (0.487 - 0.587)	0.809 (0.616 - 1.000)	0.811 (0.619 - 1.001)	0.814 (0.630 - 0.987)	3.895 (2.304 - 6.002)	3.988 (2.395 - 6.252)	4.220 (2.527 - 6.485)
70+ years (rate per capita)	0.576 (0.522 - 0.631)	0.576 (0.522 - 0.636)	0.574 (0.514 - 0.628)	0.792 (0.589 - 0.993)	0.790 (0.591 - 0.992)	0.789 (0.590 - 0.986)	6.549 (3.923 - 9.784)	6.588 (4.004 - 9.813)	6.521 (3.948 - 9.731)
All ages (rate per capita)	0.462 (0.429 - 0.497)	0.469 (0.435 - 0.504)	0.468 (0.435 - 0.503)	0.776 (0.685 - 0.876)	0.785 (0.687 - 0.895)	0.773 (0.682 - 0.871)	1.162 (0.689 - 1.811)	1.163 (0.693 - 1.794)	1.338 (0.796 - 2.053)
Age-standardised (rate per capita)	0.478 (0.449 - 0.509)	0.478 (0.449 - 0.510)	0.478 (0.450 - 0.510)	0.786 (0.706 - 0.867)	0.786 (0.706 - 0.867)	0.786 (0.706 - 0.867)	1.986 (1.180 - 3.020)	1.985 (1.188 - 3.017)	1.982 (1.184 - 3.009)
Total number of cases (thousands)	346 (321 - 372)	467 (433 - 502)	557 (518 - 598)	581 (513 - 656)	782 (684 - 891)	920 (811 - 1,036)	1 (1 - 1)	1 (1 - 2)	2 (1 - 2)
**Vietnam**									
Under-5 years (rate per capita)	0.416 (0.341 - 0.489)	0.418 (0.341 - 0.489)	0.418 (0.337 - 0.491)	0.601 (0.390 - 0.776)	0.603 (0.391 - 0.778)	0.605 (0.393 - 0.780)	0.098 (0.042 - 0.194)	0.099 (0.042 - 0.198)	0.099 (0.042 - 0.197)
5-14 years (rate per capita)	0.508 (0.458 - 0.561)	0.499 (0.450 - 0.560)	0.508 (0.457 - 0.562)	0.805 (0.616 - 1.072)	0.756 (0.577 - 0.997)	0.805 (0.616 - 1.071)	0.545 (0.306 - 0.886)	0.551 (0.306 - 0.903)	0.547 (0.308 - 0.887)
15 - 49 years (rate per capita)	0.459 (0.434 - 0.486)	0.448 (0.426 - 0.473)	0.451 (0.430 - 0.477)	0.721 (0.652 - 0.797)	0.723 (0.655 - 0.795)	0.732 (0.663 - 0.804)	1.212 (0.706 - 1.943)	1.284 (0.744 - 2.056)	1.332 (0.769 - 2.113)
50 - 69 years (rate per capita)	0.485 (0.460 - 0.511)	0.475 (0.449 - 0.507)	0.478 (0.452 - 0.505)	0.795 (0.710 - 0.895)	0.786 (0.697 - 0.884)	0.791 (0.701 - 0.897)	3.159 (1.925 - 4.783)	3.031 (1.845 - 4.681)	3.020 (1.836 - 4.585)
70+ years (rate per capita)	0.514 (0.491 - 0.542)	0.506 (0.483 - 0.531)	0.497 (0.475 - 0.524)	0.736 (0.653 - 0.827)	0.726 (0.644 - 0.809)	0.713 (0.638 - 0.796)	4.677 (3.021 - 6.827)	4.608 (2.995 - 6.697)	4.580 (2.953 - 6.630)
All ages (rate per capita)	0.469 (0.447 - 0.490)	0.461 (0.442 - 0.480)	0.463 (0.446 - 0.481)	0.733 (0.667 - 0.813)	0.726 (0.667 - 0.790)	0.741 (0.688 - 0.798)	1.230 (0.730 - 1.901)	1.394 (0.829 - 2.156)	1.543 (0.919 - 2.378)
Age-standardised (rate per capita)	0.468 (0.450 - 0.486)	0.464 (0.446 - 0.481)	0.468 (0.451 - 0.486)	0.741 (0.688 - 0.801)	0.738 (0.680 - 0.798)	0.741 (0.688 - 0.801)	1.618 (0.973 - 2.486)	1.613 (0.968 - 2.475)	1.623 (0.976 - 2.496)
Total number of cases (thousands)	32,033 (30,520 - 33,433)	38,877 (37,245 - 40,451)	43,314 (41,727 - 44,978)	50,021 (45,500 - 55,504)	61,240 (56,247 - 66,592)	69,257 (64,305 - 74,579)	84 (50 - 130)	118 (70 - 182)	144 (86 - 222)
**Oceania**									
Under-5 years (rate per capita)	0.328 (0.288 - 0.373)	0.329 (0.286 - 0.378)	0.330 (0.284 - 0.378)	0.548 (0.318 - 0.754)	0.552 (0.320 - 0.757)	0.552 (0.320 - 0.759)	0.077 (0.033 - 0.155)	0.078 (0.034 - 0.154)	0.078 (0.033 - 0.157)
5-14 years (rate per capita)	0.598 (0.559 - 0.638)	0.601 (0.562 - 0.642)	0.598 (0.559 - 0.639)	0.855 (0.680 - 1.075)	0.862 (0.687 - 1.079)	0.851 (0.676 - 1.071)	0.611 (0.335 - 0.997)	0.613 (0.336 - 1.014)	0.613 (0.333 - 1.012)
15 - 49 years (rate per capita)	0.577 (0.559 - 0.595)	0.573 (0.554 - 0.591)	0.573 (0.553 - 0.594)	0.710 (0.650 - 0.776)	0.718 (0.657 - 0.782)	0.719 (0.660 - 0.784)	1.278 (0.751 - 2.017)	1.310 (0.769 - 2.070)	1.320 (0.775 - 2.092)
50 - 69 years (rate per capita)	0.563 (0.540 - 0.585)	0.563 (0.540 - 0.587)	0.563 (0.539 - 0.588)	0.841 (0.750 - 0.933)	0.841 (0.753 - 0.931)	0.841 (0.750 - 0.933)	3.191 (1.997 - 4.753)	3.173 (1.986 - 4.734)	3.194 (2.001 - 4.752)
70+ years (rate per capita)	0.614 (0.595 - 0.634)	0.610 (0.592 - 0.631)	0.609 (0.590 - 0.629)	0.798 (0.710 - 0.893)	0.797 (0.708 - 0.891)	0.796 (0.710 - 0.890)	5.346 (3.540 - 7.674)	5.318 (3.503 - 7.637)	5.313 (3.500 - 7.598)
All ages (rate per capita)	0.544 (0.527 - 0.558)	0.545 (0.529 - 0.560)	0.548 (0.532 - 0.563)	0.735 (0.667 - 0.811)	0.742 (0.677 - 0.813)	0.743 (0.681 - 0.813)	1.140 (0.682 - 1.755)	1.219 (0.731 - 1.883)	1.297 (0.779 - 1.994)
Age-standardised (rate per capita)	0.554 (0.542 - 0.566)	0.554 (0.542 - 0.568)	0.554 (0.540 - 0.567)	0.756 (0.705 - 0.812)	0.757 (0.705 - 0.811)	0.757 (0.705 - 0.813)	1.720 (1.054 - 2.601)	1.720 (1.054 - 2.598)	1.720 (1.052 - 2.595)
Total number of cases (thousands)	3,588 (3,479 - 3,685)	4,952 (4,808 - 5,093)	6,015 (5,837 - 6,180)	4,852 (4,401 - 5,355)	6,746 (6,156 - 7,392)	8,158 (7,474 - 8,925)	8 (5 - 12)	11 (7 - 17)	14 (9 - 22)
**American Samoa**									
Under-5 years (rate per capita)	0.327 (0.272 - 0.385)	0.330 (0.270 - 0.391)	0.329 (0.274 - 0.390)	0.543 (0.315 - 0.745)	0.548 (0.318 - 0.752)	0.549 (0.319 - 0.754)	0.077 (0.033 - 0.155)	0.078 (0.033 - 0.155)	0.078 (0.033 - 0.156)
5-14 years (rate per capita)	0.597 (0.544 - 0.647)	0.598 (0.549 - 0.651)	0.597 (0.548 - 0.649)	0.853 (0.678 - 1.072)	0.873 (0.693 - 1.098)	0.886 (0.703 - 1.115)	0.615 (0.337 - 1.013)	0.612 (0.336 - 1.009)	0.611 (0.337 - 1.005)
15 - 49 years (rate per capita)	0.574 (0.550 - 0.599)	0.569 (0.546 - 0.592)	0.566 (0.541 - 0.590)	0.715 (0.654 - 0.782)	0.727 (0.668 - 0.791)	0.736 (0.673 - 0.800)	1.299 (0.758 - 2.054)	1.371 (0.801 - 2.174)	1.424 (0.839 - 2.243)
50 - 69 years (rate per capita)	0.565 (0.534 - 0.592)	0.562 (0.532 - 0.589)	0.561 (0.530 - 0.590)	0.841 (0.750 - 0.930)	0.841 (0.751 - 0.932)	0.841 (0.751 - 0.935)	3.232 (2.012 - 4.801)	3.169 (1.979 - 4.727)	3.136 (1.961 - 4.703)
70+ years (rate per capita)	0.601 (0.578 - 0.626)	0.609 (0.583 - 0.636)	0.612 (0.586 - 0.641)	0.792 (0.708 - 0.882)	0.796 (0.710 - 0.887)	0.799 (0.709 - 0.895)	5.283 (3.490 - 7.560)	5.328 (3.508 - 7.617)	5.363 (3.519 - 7.703)
All ages (rate per capita)	0.537 (0.517 - 0.556)	0.544 (0.521 - 0.564)	0.541 (0.520 - 0.561)	0.730 (0.663 - 0.804)	0.752 (0.684 - 0.830)	0.762 (0.691 - 0.842)	1.187 (0.708 - 1.826)	1.249 (0.747 - 1.924)	1.267 (0.760 - 1.949)
Age-standardised (rate per capita)	0.554 (0.538 - 0.570)	0.554 (0.536 - 0.571)	0.554 (0.536 - 0.570)	0.757 (0.704 - 0.813)	0.757 (0.705 - 0.813)	0.757 (0.705 - 0.813)	1.729 (1.054 - 2.606)	1.729 (1.055 - 2.608)	1.730 (1.064 - 2.609)
Total number of cases (thousands)	26 (25 - 27)	35 (34 - 36)	45 (43 - 46)	35 (32 - 39)	49 (44 - 54)	63 (57 - 70)	0 (0 - 0)	0 (0 - 0)	0 (0 - 0)
**Federated States of Micronesia**									
Under-5 years (rate per capita)	0.333 (0.273 - 0.396)	0.340 (0.281 - 0.405)	0.333 (0.271 - 0.394)	0.553 (0.321 - 0.759)	0.564 (0.327 - 0.775)	0.556 (0.322 - 0.763)	0.079 (0.033 - 0.162)	0.080 (0.034 - 0.165)	0.079 (0.033 - 0.161)
5-14 years (rate per capita)	0.598 (0.546 - 0.653)	0.596 (0.545 - 0.650)	0.598 (0.549 - 0.650)	0.866 (0.688 - 1.089)	0.840 (0.668 - 1.053)	0.829 (0.661 - 1.040)	0.611 (0.337 - 1.012)	0.618 (0.340 - 1.036)	0.619 (0.339 - 1.024)
15 - 49 years (rate per capita)	0.578 (0.555 - 0.603)	0.578 (0.553 - 0.603)	0.578 (0.552 - 0.602)	0.706 (0.644 - 0.771)	0.704 (0.641 - 0.770)	0.704 (0.642 - 0.771)	1.263 (0.739 - 2.015)	1.273 (0.745 - 2.023)	1.242 (0.730 - 1.971)
50 - 69 years (rate per capita)	0.569 (0.541 - 0.598)	0.558 (0.525 - 0.590)	0.566 (0.537 - 0.594)	0.843 (0.753 - 0.933)	0.839 (0.747 - 0.935)	0.843 (0.750 - 0.935)	3.349 (2.086 - 4.964)	3.115 (1.933 - 4.636)	3.290 (2.055 - 4.872)
70+ years (rate per capita)	0.613 (0.589 - 0.638)	0.596 (0.573 - 0.623)	0.599 (0.575 - 0.622)	0.798 (0.709 - 0.893)	0.790 (0.708 - 0.879)	0.791 (0.708 - 0.881)	5.381 (3.528 - 7.707)	5.256 (3.480 - 7.496)	5.283 (3.484 - 7.516)
All ages (rate per capita)	0.544 (0.522 - 0.566)	0.549 (0.528 - 0.569)	0.553 (0.533 - 0.572)	0.738 (0.664 - 0.820)	0.735 (0.668 - 0.810)	0.735 (0.674 - 0.803)	1.129 (0.675 - 1.752)	1.217 (0.730 - 1.873)	1.313 (0.793 - 2.022)
Age-standardised (rate per capita)	0.554 (0.537 - 0.572)	0.554 (0.537 - 0.569)	0.554 (0.536 - 0.570)	0.757 (0.705 - 0.813)	0.757 (0.705 - 0.813)	0.757 (0.705 - 0.813)	1.731 (1.057 - 2.610)	1.729 (1.059 - 2.617)	1.728 (1.059 - 2.620)
Total number of cases (thousands)	53 (50 - 55)	59 (57 - 61)	58 (56 - 60)	71 (64 - 79)	79 (72 - 87)	77 (71 - 84)	0 (0 - 0)	0 (0 - 0)	0 (0 - 0)
**Fiji**									
Under-5 years (rate per capita)	0.321 (0.267 - 0.371)	0.327 (0.276 - 0.377)	0.321 (0.272 - 0.372)	0.550 (0.318 - 0.769)	0.568 (0.330 - 0.771)	0.552 (0.319 - 0.771)	0.076 (0.032 - 0.155)	0.077 (0.032 - 0.152)	0.076 (0.032 - 0.151)
5-14 years (rate per capita)	0.598 (0.551 - 0.651)	0.645 (0.602 - 0.687)	0.598 (0.544 - 0.645)	0.855 (0.687 - 1.082)	0.880 (0.717 - 1.098)	0.850 (0.684 - 1.075)	0.612 (0.337 - 1.004)	0.637 (0.351 - 1.051)	0.614 (0.338 - 1.011)
15 - 49 years (rate per capita)	0.572 (0.549 - 0.596)	0.570 (0.548 - 0.594)	0.570 (0.548 - 0.591)	0.717 (0.655 - 0.786)	0.724 (0.664 - 0.787)	0.725 (0.664 - 0.793)	1.297 (0.763 - 2.048)	1.355 (0.800 - 2.142)	1.352 (0.794 - 2.139)
50 - 69 years (rate per capita)	0.562 (0.532 - 0.593)	0.564 (0.531 - 0.595)	0.565 (0.536 - 0.594)	0.842 (0.749 - 0.935)	0.841 (0.753 - 0.932)	0.843 (0.749 - 0.933)	3.144 (1.972 - 4.684)	3.179 (1.988 - 4.686)	3.198 (1.997 - 4.735)

	Prevalence 1990	Prevalence 2005	Prevalence 2015	Incidence 1990	Incidence 2005	Incidence 2015	YLD rate 1990	YLD rate 2005	YLD rate 2015
**70+ years (rate per capita)**	0.612 (0.587 - 0.638)	0.613 (0.586 - 0.641)	0.614 (0.586 - 0.642)	0.797 (0.708 - 0.895)	0.798 (0.710 - 0.894)	0.798 (0.708 - 0.897)	5.317 (3.493 - 7.582)	5.320 (3.480 - 7.595)	5.329 (3.484 - 7.606)
**All ages (rate per capita)**	0.542 (0.523 - 0.562)	0.557 (0.541 - 0.574)	0.551 (0.534 - 0.568)	0.739 (0.671 - 0.812)	0.753 (0.693 - 0.820)	0.753 (0.698 - 0.815)	1.183 (0.705 - 1.820)	1.388 (0.838 - 2.133)	1.519 (0.919 - 2.315)
**Age-standardised (rate per capita)**	0.552 (0.536 - 0.568)	0.562 (0.547 - 0.578)	0.553 (0.537 - 0.569)	0.755 (0.703 - 0.811)	0.763 (0.709 - 0.823)	0.755 (0.703 - 0.811)	1.712 (1.050 - 2.573)	1.720 (1.055 - 2.587)	1.717 (1.051 - 2.584)
**Total number of cases (thousands)**	398 (384 - 412)	461 (448 - 476)	492 (476 - 506)	543 (493 - 596)	623 (574 - 678)	672 (623 - 727)	1 (1 - 1)	1 (1 - 2)	1 (1 - 2)
**Guam**									
**Under-5 years (rate per capita)**	0.332 (0.274 - 0.393)	0.336 (0.282 - 0.396)	0.332 (0.276 - 0.392)	0.554 (0.321 - 0.760)	0.562 (0.326 - 0.772)	0.555 (0.322 - 0.762)	0.079 (0.034 - 0.154)	0.080 (0.034 - 0.165)	0.079 (0.034 - 0.154)
**5-14 years (rate per capita)**	0.598 (0.548 - 0.647)	0.598 (0.546 - 0.649)	0.598 (0.550 - 0.649)	0.857 (0.681 - 1.077)	0.848 (0.674 - 1.065)	0.834 (0.665 - 1.046)	0.615 (0.337 - 1.017)	0.616 (0.338 - 1.014)	0.619 (0.338 - 1.028)
**15 - 49 years (rate per capita)**	0.569 (0.545 - 0.593)	0.569 (0.546 - 0.593)	0.570 (0.547 - 0.595)	0.724 (0.662 - 0.791)	0.730 (0.669 - 0.795)	0.726 (0.666 - 0.788)	1.324 (0.776 - 2.087)	1.391 (0.819 - 2.199)	1.375 (0.813 - 2.165)
**50 - 69 years (rate per capita)**	0.566 (0.533 - 0.593)	0.564 (0.534 - 0.595)	0.567 (0.536 - 0.596)	0.842 (0.750 - 0.931)	0.841 (0.750 - 0.932)	0.841 (0.751 - 0.931)	3.308 (2.063 - 4.922)	3.264 (2.036 - 4.834)	3.308 (2.066 - 4.938)
**70+ years (rate per capita)**	0.609 (0.586 - 0.634)	0.604 (0.581 - 0.630)	0.598 (0.575 - 0.622)	0.796 (0.709 - 0.889)	0.794 (0.709 - 0.884)	0.791 (0.709 - 0.880)	5.432 (3.591 - 7.860)	5.372 (3.563 - 7.683)	5.344 (3.540 - 7.691)
**All ages (rate per capita)**	0.546 (0.526 - 0.564)	0.551 (0.533 - 0.569)	0.556 (0.538 - 0.573)	0.742 (0.684 - 0.806)	0.755 (0.699 - 0.816)	0.756 (0.704 - 0.810)	1.351 (0.814 - 2.081)	1.543 (0.934 - 2.360)	1.721 (1.048 - 2.625)
**Age-standardised (rate per capita)**	0.554 (0.536 - 0.570)	0.554 (0.536 - 0.571)	0.554 (0.538 - 0.572)	0.757 (0.705 - 0.813)	0.757 (0.704 - 0.813)	0.757 (0.704 - 0.813)	1.740 (1.066 - 2.627)	1.741 (1.061 - 2.633)	1.738 (1.061 - 2.633)
**Total number of cases (thousands)**	72 (69 - 74)	87 (85 - 90)	94 (91 - 97)	98 (90 - 106)	120 (111 - 129)	128 (120 - 138)	0 (0 - 0)	0 (0 - 0)	0 (0 - 0)
**Kiribati**									
**Under-5 years (rate per capita)**	0.328 (0.267 - 0.393)	0.330 (0.272 - 0.390)	0.329 (0.273 - 0.390)	0.547 (0.317 - 0.751)	0.550 (0.319 - 0.755)	0.551 (0.319 - 0.756)	0.078 (0.034 - 0.154)	0.078 (0.033 - 0.158)	0.078 (0.033 - 0.156)
**5-14 years (rate per capita)**	0.599 (0.549 - 0.648)	0.598 (0.547 - 0.653)	0.597 (0.548 - 0.647)	0.871 (0.692 - 1.096)	0.864 (0.686 - 1.086)	0.878 (0.697 - 1.104)	0.605 (0.338 - 0.991)	0.607 (0.335 - 1.004)	0.605 (0.334 - 0.996)
**15 - 49 years (rate per capita)**	0.573 (0.551 - 0.601)	0.576 (0.550 - 0.600)	0.573 (0.549 - 0.598)	0.717 (0.655 - 0.784)	0.713 (0.653 - 0.778)	0.716 (0.655 - 0.783)	1.301 (0.765 - 2.059)	1.309 (0.769 - 2.079)	1.315 (0.774 - 2.078)
**50 - 69 years (rate per capita)**	0.566 (0.536 - 0.595)	0.564 (0.531 - 0.594)	0.561 (0.531 - 0.593)	0.841 (0.750 - 0.931)	0.841 (0.751 - 0.934)	0.840 (0.751 - 0.934)	3.238 (2.019 - 4.823)	3.183 (1.997 - 4.763)	3.149 (1.954 - 4.670)
**70+ years (rate per capita)**	0.613 (0.589 - 0.640)	0.617 (0.588 - 0.644)	0.612 (0.586 - 0.640)	0.798 (0.708 - 0.893)	0.800 (0.709 - 0.895)	0.798 (0.706 - 0.891)	5.372 (3.557 - 7.662)	5.362 (3.527 - 7.675)	5.366 (3.532 - 7.668)
**All ages (rate per capita)**	0.540 (0.520 - 0.560)	0.548 (0.529 - 0.570)	0.546 (0.526 - 0.565)	0.739 (0.671 - 0.816)	0.742 (0.675 - 0.816)	0.746 (0.681 - 0.815)	1.193 (0.714 - 1.842)	1.230 (0.739 - 1.891)	1.296 (0.779 - 1.990)
**Age-standardised (rate per capita)**	0.555 (0.538 - 0.573)	0.554 (0.538 - 0.573)	0.554 (0.537 - 0.571)	0.756 (0.704 - 0.813)	0.756 (0.704 - 0.813)	0.757 (0.704 - 0.813)	1.726 (1.060 - 2.590)	1.724 (1.056 - 2.607)	1.725 (1.055 - 2.599)
**Total number of cases (thousands)**	39 (38 - 41)	52 (50 - 54)	61 (59 - 64)	54 (49 - 59)	70 (63 - 77)	84 (77 - 92)	0 (0 - 0)	0 (0 - 0)	0 (0 - 0)
**Marshall Islands**									
**Under-5 years (rate per capita)**	0.329 (0.267 - 0.391)	0.330 (0.268 - 0.393)	0.336 (0.275 - 0.399)	0.549 (0.319 - 0.754)	0.551 (0.320 - 0.757)	0.560 (0.325 - 0.768)	0.078 (0.033 - 0.156)	0.078 (0.033 - 0.155)	0.079 (0.034 - 0.162)
**5-14 years (rate per capita)**	0.597 (0.545 - 0.647)	0.598 (0.547 - 0.650)	0.597 (0.549 - 0.645)	0.855 (0.680 - 1.075)	0.861 (0.684 - 1.082)	0.851 (0.676 - 1.069)	0.611 (0.335 - 1.016)	0.611 (0.338 - 1.003)	0.612 (0.335 - 1.015)
**15 - 49 years (rate per capita)**	0.576 (0.552 - 0.602)	0.573 (0.549 - 0.599)	0.572 (0.549 - 0.597)	0.710 (0.649 - 0.776)	0.718 (0.656 - 0.782)	0.718 (0.658 - 0.784)	1.277 (0.756 - 2.027)	1.306 (0.769 - 2.062)	1.316 (0.778 - 2.091)
**50 - 69 years (rate per capita)**	0.563 (0.532 - 0.592)	0.562 (0.529 - 0.595)	0.563 (0.530 - 0.591)	0.841 (0.750 - 0.932)	0.841 (0.751 - 0.933)	0.841 (0.750 - 0.932)	3.175 (1.991 - 4.672)	3.152 (1.974 - 4.636)	3.167 (1.993 - 4.686)
**70+ years (rate per capita)**	0.614 (0.589 - 0.641)	0.611 (0.585 - 0.637)	0.609 (0.584 - 0.637)	0.798 (0.709 - 0.893)	0.797 (0.709 - 0.890)	0.796 (0.708 - 0.889)	5.323 (3.506 - 7.621)	5.285 (3.487 - 7.554)	5.272 (3.503 - 7.499)
**All ages (rate per capita)**	0.536 (0.513 - 0.558)	0.541 (0.519 - 0.561)	0.547 (0.527 - 0.566)	0.729 (0.657 - 0.809)	0.739 (0.672 - 0.813)	0.743 (0.680 - 0.814)	1.099 (0.657 - 1.707)	1.192 (0.714 - 1.834)	1.279 (0.773 - 1.974)
**Age-standardised (rate per capita)**	0.554 (0.536 - 0.571)	0.554 (0.536 - 0.572)	0.554 (0.536 - 0.571)	0.757 (0.704 - 0.813)	0.757 (0.704 - 0.813)	0.757 (0.704 - 0.813)	1.716 (1.059 - 2.591)	1.711 (1.054 - 2.577)	1.710 (1.058 - 2.575)
**Total number of cases (thousands)**	25 (24 - 27)	32 (31 - 34)	40 (38 - 41)	35 (31 - 38)	44 (40 - 49)	54 (49 - 59)	0 (0 - 0)	0 (0 - 0)	0 (0 - 0)
**Northern Mariana Islands**									
**Under-5 years (rate per capita)**	0.323 (0.267 - 0.381)	0.330 (0.271 - 0.393)	0.331 (0.273 - 0.392)	0.541 (0.314 - 0.743)	0.551 (0.320 - 0.756)	0.551 (0.320 - 0.757)	0.077 (0.032 - 0.158)	0.079 (0.034 - 0.160)	0.078 (0.033 - 0.158)
**5-14 years (rate per capita)**	0.597 (0.548 - 0.646)	0.597 (0.547 - 0.647)	0.597 (0.545 - 0.650)	0.860 (0.683 - 1.081)	0.868 (0.690 - 1.093)	0.857 (0.681 - 1.077)	0.614 (0.338 - 1.015)	0.614 (0.337 - 1.014)	0.617 (0.338 - 1.024)
**15 - 49 years (rate per capita)**	0.566 (0.544 - 0.591)	0.563 (0.539 - 0.589)	0.562 (0.538 - 0.589)	0.733 (0.669 - 0.808)	0.740 (0.675 - 0.810)	0.741 (0.676 - 0.810)	1.350 (0.789 - 2.145)	1.390 (0.809 - 2.212)	1.401 (0.828 - 2.224)
**50 - 69 years (rate per capita)**	0.557 (0.523 - 0.590)	0.555 (0.521 - 0.590)	0.553 (0.517 - 0.588)	0.839 (0.744 - 0.935)	0.837 (0.741 - 0.938)	0.836 (0.738 - 0.939)	3.079 (1.918 - 4.596)	3.010 (1.868 - 4.510)	2.960 (1.835 - 4.493)
**70+ years (rate per capita)**	0.607 (0.583 - 0.632)	0.603 (0.579 - 0.629)	0.602 (0.578 - 0.628)	0.795 (0.706 - 0.888)	0.794 (0.709 - 0.882)	0.794 (0.708 - 0.882)	5.415 (3.557 - 7.786)	5.321 (3.499 - 7.624)	5.281 (3.444 - 7.583)
**All ages (rate per capita)**	0.548 (0.529 - 0.567)	0.549 (0.530 - 0.568)	0.551 (0.530 - 0.571)	0.741 (0.687 - 0.801)	0.750 (0.696 - 0.810)	0.752 (0.698 - 0.811)	1.282 (0.760 - 2.010)	1.319 (0.776 - 2.058)	1.346 (0.802 - 2.096)
**Age-standardised (rate per capita)**	0.554 (0.537 - 0.571)	0.554 (0.537 - 0.573)	0.554 (0.536 - 0.572)	0.756 (0.705 - 0.813)	0.756 (0.704 - 0.812)	0.756 (0.704 - 0.812)	1.731 (1.058 - 2.627)	1.734 (1.057 - 2.627)	1.734 (1.059 - 2.630)
**Total number of cases (thousands)**	24 (23 - 24)	45 (44 - 47)	64 (61 - 66)	32 (30 - 35)	62 (57 - 67)	87 (81 - 94)	0 (0 - 0)	0 (0 - 0)	0 (0 - 0)
**Papua New Guinea**									
**Under-5 years (rate per capita)**	0.328 (0.271 - 0.389)	0.328 (0.270 - 0.388)	0.331 (0.274 - 0.390)	0.546 (0.317 - 0.750)	0.550 (0.319 - 0.755)	0.552 (0.320 - 0.758)	0.077 (0.033 - 0.157)	0.078 (0.034 - 0.154)	0.078 (0.033 - 0.159)
**5-14 years (rate per capita)**	0.598 (0.546 - 0.649)	0.597 (0.548 - 0.647)	0.599 (0.550 - 0.648)	0.854 (0.679 - 1.073)	0.865 (0.687 - 1.088)	0.851 (0.677 - 1.070)	0.610 (0.334 - 0.995)	0.610 (0.336 - 1.016)	0.612 (0.332 - 1.008)
**15 - 49 years (rate per capita)**	0.578 (0.556 - 0.603)	0.573 (0.549 - 0.597)	0.573 (0.548 - 0.600)	0.708 (0.647 - 0.773)	0.716 (0.656 - 0.781)	0.717 (0.657 - 0.783)	1.269 (0.748 - 2.005)	1.296 (0.761 - 2.054)	1.311 (0.767 - 2.082)
**50 - 69 years (rate per capita)**	0.562 (0.534 - 0.591)	0.562 (0.531 - 0.593)	0.563 (0.529 - 0.593)	0.841 (0.750 - 0.933)	0.841 (0.751 - 0.934)	0.841 (0.750 - 0.933)	3.170 (1.983 - 4.716)	3.156 (1.975 - 4.736)	3.177 (1.989 - 4.750)
**70+ years (rate per capita)**	0.618 (0.591 - 0.645)	0.614 (0.589 - 0.641)	0.612 (0.586 - 0.639)	0.800 (0.708 - 0.895)	0.799 (0.708 - 0.893)	0.798 (0.708 - 0.892)	5.357 (3.547 - 7.687)	5.327 (3.514 - 7.651)	5.325 (3.514 - 7.623)
**All ages (rate per capita)**	0.544 (0.522 - 0.565)	0.542 (0.522 - 0.562)	0.547 (0.526 - 0.567)	0.734 (0.664 - 0.812)	0.740 (0.672 - 0.816)	0.741 (0.676 - 0.814)	1.106 (0.660 - 1.708)	1.159 (0.692 - 1.796)	1.225 (0.733 - 1.892)
**Age-standardised (rate per capita)**	0.555 (0.539 - 0.571)	0.554 (0.537 - 0.572)	0.554 (0.536 - 0.571)	0.757 (0.704 - 0.813)	0.757 (0.705 - 0.813)	0.757 (0.705 - 0.813)	1.719 (1.054 - 2.603)	1.717 (1.051 - 2.598)	1.718 (1.055 - 2.594)
**Total number of cases (thousands)**	2,267 (2,177 - 2,353)	3,304 (3,180 - 3,428)	4,175 (4,016 - 4,326)	3,058 (2,766 - 3,382)	4,512 (4,097 - 4,975)	5,655 (5,161 - 6,213)	5 (3 - 7)	7 (4 - 11)	9 (6 - 14)
**Samoa**									
**Under-5 years (rate per capita)**	0.335 (0.273 - 0.398)	0.334 (0.271 - 0.394)	0.335 (0.278 - 0.400)	0.558 (0.324 - 0.766)	0.556 (0.323 - 0.764)	0.560 (0.325 - 0.769)	0.079 (0.034 - 0.164)	0.079 (0.034 - 0.157)	0.080 (0.033 - 0.162)
**5-14 years (rate per capita)**	0.598 (0.548 - 0.650)	0.597 (0.551 - 0.648)	0.598 (0.547 - 0.653)	0.844 (0.671 - 1.059)	0.850 (0.676 - 1.068)	0.857 (0.681 - 1.078)	0.616 (0.338 - 1.015)	0.616 (0.334 - 1.010)	0.615 (0.340 - 1.005)
**15 - 49 years (rate per capita)**	0.579 (0.553 - 0.605)	0.573 (0.550 - 0.598)	0.575 (0.552 - 0.599)	0.702 (0.640 - 0.769)	0.718 (0.658 - 0.783)	0.714 (0.653 - 0.778)	1.249 (0.727 - 1.985)	1.324 (0.778 - 2.088)	1.316 (0.773 - 2.076)
**50 - 69 years (rate per capita)**	0.568 (0.537 - 0.596)	0.565 (0.536 - 0.593)	0.563 (0.531 - 0.593)	0.842 (0.752 - 0.933)	0.841 (0.751 - 0.931)	0.842 (0.749 - 0.932)	3.330 (2.079 - 4.926)	3.267 (2.037 - 4.863)	3.217 (1.998 - 4.759)
**70+ years (rate per capita)**	0.610 (0.586 - 0.637)	0.603 (0.579 - 0.630)	0.598 (0.575 - 0.624)	0.797 (0.708 - 0.892)	0.793 (0.706 - 0.885)	0.791 (0.708 - 0.879)	5.380 (3.527 - 7.742)	5.344 (3.528 - 7.651)	5.288 (3.496 - 7.565)
**All ages (rate per capita)**	0.544 (0.522 - 0.566)	0.545 (0.523 - 0.564)	0.549 (0.530 - 0.570)	0.729 (0.659 - 0.807)	0.743 (0.675 - 0.818)	0.748 (0.683 - 0.819)	1.171 (0.701 - 1.806)	1.280 (0.769 - 1.966)	1.353 (0.814 - 2.073)
**Age-standardised (rate per capita)**	0.554 (0.536 - 0.572)	0.554 (0.536 - 0.571)	0.554 (0.538 - 0.572)	0.757 (0.705 - 0.813)	0.757 (0.705 - 0.813)	0.757 (0.705 - 0.813)	1.736 (1.064 - 2.623)	1.731 (1.064 - 2.622)	1.730 (1.058 - 2.627)
**Total number of cases (thousands)**	91 (87 - 94)	99 (95 - 102)	107 (103 - 110)	122 (110 - 135)	134 (122 - 148)	145 (132 - 159)	0 (0 - 0)	0 (0 - 0)	0 (0 - 0)
**Solomon Islands**									
**Under-5 years (rate per capita)**	0.327 (0.267 - 0.391)	0.330 (0.275 - 0.393)	0.335 (0.273 - 0.396)	0.549 (0.318 - 0.753)	0.550 (0.319 - 0.755)	0.556 (0.323 - 0.763)	0.078 (0.033 - 0.159)	0.078 (0.033 - 0.156)	0.079 (0.034 - 0.159)
**5-14 years (rate per capita)**	0.597 (0.547 - 0.650)	0.599 (0.548 - 0.652)	0.596 (0.547 - 0.646)	0.853 (0.678 - 1.072)	0.856 (0.681 - 1.076)	0.856 (0.680 - 1.076)	0.613 (0.337 - 1.020)	0.613 (0.338 - 1.016)	0.614 (0.337 - 1.014)
**15 - 49 years (rate per capita)**	0.578 (0.553 - 0.602)	0.575 (0.551 - 0.601)	0.574 (0.549 - 0.600)	0.706 (0.645 - 0.773)	0.713 (0.652 - 0.779)	0.715 (0.654 - 0.780)	1.272 (0.748 - 2.016)	1.289 (0.758 - 2.039)	1.309 (0.767 - 2.072)
**50 - 69 years (rate per capita)**	0.564 (0.530 - 0.592)	0.564 (0.532 - 0.592)	0.563 (0.533 - 0.591)	0.842 (0.749 - 0.932)	0.842 (0.749 - 0.934)	0.841 (0.750 - 0.931)	3.223 (2.013 - 4.774)	3.219 (2.007 - 4.798)	3.207 (1.995 - 4.774)
**70+ years (rate per capita)**	0.609 (0.584 - 0.637)	0.610 (0.584 - 0.638)	0.608 (0.582 - 0.637)	0.796 (0.709 - 0.889)	0.797 (0.709 - 0.891)	0.796 (0.709 - 0.890)	5.325 (3.483 - 7.655)	5.333 (3.515 - 7.674)	5.312 (3.468 - 7.641)
**All ages (rate per capita)**	0.537 (0.515 - 0.560)	0.542 (0.520 - 0.563)	0.545 (0.524 - 0.566)	0.730 (0.655 - 0.815)	0.736 (0.666 - 0.813)	0.740 (0.673 - 0.817)	1.078 (0.640 - 1.675)	1.150 (0.681 - 1.779)	1.202 (0.715 - 1.856)
**Age-standardised (rate per capita)**	0.554 (0.537 - 0.571)	0.554 (0.538 - 0.572)	0.553 (0.536 - 0.571)	0.757 (0.704 - 0.813)	0.757 (0.704 - 0.813)	0.757 (0.704 - 0.813)	1.726 (1.054 - 2.605)	1.726 (1.056 - 2.602)	1.726 (1.058 - 2.616)
**Total number of cases (thousands)**	170 (163 - 177)	256 (246 - 266)	319 (307 - 331)	231 (207 - 258)	348 (315 - 384)	434 (394 - 479)	0 (0 - 1)	1 (0 - 1)	1 (0 - 1)
**Tonga**									
**Under-5 years (rate per capita)**	0.334 (0.272 - 0.400)	0.333 (0.276 - 0.392)	0.335 (0.279 - 0.395)	0.558 (0.324 - 0.767)	0.553 (0.321 - 0.760)	0.560 (0.325 - 0.770)	0.079 (0.034 - 0.160)	0.079 (0.033 - 0.159)	0.080 (0.034 - 0.162)
**5-14 years (rate per capita)**	0.596 (0.545 - 0.647)	0.596 (0.548 - 0.650)	0.597 (0.544 - 0.649)	0.867 (0.689 - 1.091)	0.852 (0.677 - 1.070)	0.847 (0.674 - 1.063)	0.611 (0.337 - 1.018)	0.615 (0.338 - 1.020)	0.616 (0.337 - 1.029)
**15 - 49 years (rate per capita)**	0.579 (0.553 - 0.605)	0.574 (0.551 - 0.599)	0.575 (0.552 - 0.598)	0.703 (0.641 - 0.772)	0.715 (0.653 - 0.781)	0.713 (0.653 - 0.778)	1.252 (0.733 - 1.992)	1.316 (0.770 - 2.082)	1.312 (0.773 - 2.085)
**50 - 69 years (rate per capita)**	0.569 (0.540 - 0.596)	0.569 (0.537 - 0.598)	0.565 (0.533 - 0.594)	0.843 (0.752 - 0.932)	0.841 (0.754 - 0.931)	0.842 (0.749 - 0.935)	3.331 (2.097 - 4.955)	3.352 (2.105 - 4.985)	3.251 (2.029 - 4.848)
**70+ years (rate per capita)**	0.605 (0.579 - 0.633)	0.604 (0.580 - 0.630)	0.592 (0.569 - 0.616)	0.794 (0.709 - 0.886)	0.793 (0.706 - 0.885)	0.788 (0.707 - 0.874)	5.304 (3.498 - 7.574)	5.322 (3.532 - 7.588)	5.243 (3.441 - 7.496)

	Prevalence 1990	Prevalence 2005	Prevalence 2015	Incidence 1990	Incidence 2005	Incidence 2015	YLD rate 1990	YLD rate 2005	YLD rate 2015
All ages (rate per capita)	0.545 (0.523 - 0.564)	0.547 (0.527 - 0.566)	0.550 (0.531 - 0.569)	0.738 (0.669 - 0.816)	0.742 (0.676 - 0.815)	0.744 (0.681 - 0.815)	1.226 (0.735 - 1.885)	1.335 (0.805 - 2.035)	1.365 (0.822 - 2.086)
Age-standardised (rate per capita)	0.554 (0.537 - 0.570)	0.554 (0.537 - 0.571)	0.554 (0.537 - 0.570)	0.757 (0.704 - 0.813)	0.757 (0.704 - 0.813)	0.757 (0.705 - 0.813)	1.730 (1.056 - 2.612)	1.732 (1.054 - 2.625)	1.731 (1.061 - 2.633)
Total number of cases (thousands)	53 (50 - 54)	55 (53 - 57)	59 (57 - 61)	71 (65 - 79)	75 (68 - 82)	79 (73 - 87)	0 (0 - 0)	0 (0 - 0)	0 (0 - 0)
**Vanuatu**									
Under-5 years (rate per capita)	0.328 (0.268 - 0.386)	0.332 (0.270 - 0.393)	0.330 (0.272 - 0.395)	0.550 (0.319 - 0.755)	0.554 (0.321 - 0.761)	0.552 (0.320 - 0.758)	0.078 (0.034 - 0.156)	0.079 (0.033 - 0.155)	0.078 (0.034 - 0.158)
5-14 years (rate per capita)	0.596 (0.544 - 0.648)	0.597 (0.548 - 0.648)	0.597 (0.546 - 0.647)	0.865 (0.687 - 1.088)	0.856 (0.681 - 1.077)	0.866 (0.688 - 1.090)	0.611 (0.335 - 1.006)	0.613 (0.338 - 1.007)	0.611 (0.334 - 1.001)
15 - 49 years (rate per capita)	0.574 (0.551 - 0.599)	0.575 (0.550 - 0.602)	0.572 (0.549 - 0.597)	0.713 (0.653 - 0.779)	0.713 (0.652 - 0.778)	0.719 (0.658 - 0.783)	1.296 (0.755 - 2.049)	1.301 (0.763 - 2.062)	1.321 (0.772 - 2.095)
50 - 69 years (rate per capita)	0.565 (0.535 - 0.595)	0.564 (0.533 - 0.594)	0.562 (0.530 - 0.592)	0.841 (0.751 - 0.931)	0.841 (0.750 - 0.932)	0.840 (0.751 - 0.932)	3.250 (2.041 - 4.831)	3.216 (2.013 - 4.813)	3.200 (2.007 - 4.733)
70+ years (rate per capita)	0.601 (0.578 - 0.627)	0.604 (0.581 - 0.631)	0.615 (0.589 - 0.641)	0.792 (0.707 - 0.883)	0.794 (0.709 - 0.886)	0.799 (0.709 - 0.895)	5.302 (3.509 - 7.624)	5.308 (3.483 - 7.658)	5.374 (3.534 - 7.707)
All ages (rate per capita)	0.539 (0.516 - 0.559)	0.546 (0.524 - 0.567)	0.548 (0.529 - 0.566)	0.739 (0.667 - 0.820)	0.740 (0.672 - 0.815)	0.748 (0.683 - 0.819)	1.145 (0.688 - 1.762)	1.200 (0.716 - 1.856)	1.307 (0.793 - 2.003)
Age-standardised (rate per capita)	0.553 (0.535 - 0.570)	0.554 (0.537 - 0.571)	0.553 (0.537 - 0.570)	0.757 (0.705 - 0.813)	0.757 (0.704 - 0.813)	0.757 (0.704 - 0.813)	1.726 (1.067 - 2.609)	1.727 (1.055 - 2.612)	1.728 (1.067 - 2.598)
Total number of cases (thousands)	80 (76 - 82)	114 (110 - 119)	144 (139 - 149)	109 (98 - 121)	155 (140 - 171)	196 (179 - 215)	0 (0 - 0)	0 (0 - 0)	0 (0 - 1)
**North Africa and Middle East**									
Under-5 years (rate per capita)	0.373 (0.339 - 0.406)	0.373 (0.343 - 0.405)	0.380 (0.343 - 0.415)	0.562 (0.348 - 0.751)	0.574 (0.395 - 0.745)	0.568 (0.356 - 0.757)	0.088 (0.038 - 0.173)	0.088 (0.038 - 0.171)	0.090 (0.038 - 0.176)
5-14 years (rate per capita)	0.567 (0.540 - 0.594)	0.558 (0.533 - 0.583)	0.558 (0.531 - 0.584)	1.041 (0.838 - 1.307)	1.017 (0.815 - 1.272)	1.039 (0.828 - 1.306)	0.592 (0.332 - 0.984)	0.590 (0.329 - 0.975)	0.589 (0.331 - 0.976)
15 - 49 years (rate per capita)	0.509 (0.496 - 0.523)	0.510 (0.497 - 0.523)	0.514 (0.501 - 0.527)	0.806 (0.727 - 0.891)	0.807 (0.728 - 0.889)	0.812 (0.732 - 0.895)	1.820 (1.073 - 2.866)	1.865 (1.103 - 2.925)	1.970 (1.168 - 3.085)
50 - 69 years (rate per capita)	0.618 (0.604 - 0.632)	0.615 (0.601 - 0.630)	0.614 (0.599 - 0.629)	0.906 (0.795 - 1.006)	0.903 (0.791 - 1.006)	0.899 (0.786 - 1.004)	6.093 (3.804 - 9.007)	6.024 (3.771 - 8.902)	5.955 (3.715 - 8.812)
70+ years (rate per capita)	0.659 (0.647 - 0.672)	0.660 (0.647 - 0.673)	0.654 (0.641 - 0.666)	0.903 (0.796 - 1.006)	0.901 (0.792 - 1.003)	0.896 (0.788 - 0.997)	9.659 (6.403 - 13.634)	9.619 (6.319 - 13.486)	9.446 (6.237 - 13.298)
All ages (rate per capita)	0.517 (0.505 - 0.529)	0.519 (0.510 - 0.529)	0.524 (0.514 - 0.534)	0.842 (0.768 - 0.924)	0.838 (0.775 - 0.911)	0.842 (0.777 - 0.914)	1.795 (1.091 - 2.704)	2.022 (1.231 - 3.047)	2.195 (1.340 - 3.312)
Age-standardised (rate per capita)	0.540 (0.531 - 0.549)	0.539 (0.530 - 0.549)	0.537 (0.528 - 0.548)	0.848 (0.792 - 0.910)	0.848 (0.791 - 0.912)	0.849 (0.790 - 0.913)	2.846 (1.761 - 4.241)	2.837 (1.752 - 4.223)	2.820 (1.744 - 4.206)
Total number of cases (thousands)	173,610 (169,576 - 177,884)	241,437 (237,216 - 246,077)	296,526 (291,207 - 302,632)	282,891 (258,011 - 310,671)	389,538 (360,153 - 423,267)	476,815 (440,153 - 517,644)	603 (367 - 909)	940 (572 - 1,416)	1,243 (759 - 1,875)
**North Africa and Middle East**									
Under-5 years (rate per capita)	0.373 (0.339 - 0.406)	0.373 (0.343 - 0.405)	0.380 (0.343 - 0.415)	0.562 (0.348 - 0.751)	0.574 (0.395 - 0.745)	0.568 (0.356 - 0.757)	0.088 (0.038 - 0.173)	0.088 (0.038 - 0.171)	0.090 (0.038 - 0.176)
5-14 years (rate per capita)	0.567 (0.540 - 0.594)	0.558 (0.533 - 0.583)	0.558 (0.531 - 0.584)	1.041 (0.838 - 1.307)	1.017 (0.815 - 1.272)	1.039 (0.828 - 1.306)	0.592 (0.332 - 0.984)	0.590 (0.329 - 0.975)	0.589 (0.331 - 0.976)
15 - 49 years (rate per capita)	0.509 (0.496 - 0.523)	0.510 (0.497 - 0.523)	0.514 (0.501 - 0.527)	0.806 (0.727 - 0.891)	0.807 (0.728 - 0.889)	0.812 (0.732 - 0.895)	1.820 (1.073 - 2.866)	1.865 (1.103 - 2.925)	1.970 (1.168 - 3.085)
50 - 69 years (rate per capita)	0.618 (0.604 - 0.632)	0.615 (0.601 - 0.630)	0.614 (0.599 - 0.629)	0.906 (0.795 - 1.006)	0.903 (0.791 - 1.006)	0.899 (0.786 - 1.004)	6.093 (3.804 - 9.007)	6.024 (3.771 - 8.902)	5.955 (3.715 - 8.812)
70+ years (rate per capita)	0.659 (0.647 - 0.672)	0.660 (0.647 - 0.673)	0.654 (0.641 - 0.666)	0.903 (0.796 - 1.006)	0.901 (0.792 - 1.003)	0.896 (0.788 - 0.997)	9.659 (6.403 - 13.634)	9.619 (6.319 - 13.486)	9.446 (6.237 - 13.298)
All ages (rate per capita)	0.517 (0.505 - 0.529)	0.519 (0.510 - 0.529)	0.524 (0.514 - 0.534)	0.842 (0.768 - 0.924)	0.838 (0.775 - 0.911)	0.842 (0.777 - 0.914)	1.795 (1.091 - 2.704)	2.022 (1.231 - 3.047)	2.195 (1.340 - 3.312)
Age-standardised (rate per capita)	0.540 (0.531 - 0.549)	0.539 (0.530 - 0.549)	0.537 (0.528 - 0.548)	0.848 (0.792 - 0.910)	0.848 (0.791 - 0.912)	0.849 (0.790 - 0.913)	2.846 (1.761 - 4.241)	2.837 (1.752 - 4.223)	2.820 (1.744 - 4.206)
Total number of cases (thousands)	173,610 (169,576 - 177,884)	241,437 (237,216 - 246,077)	296,526 (291,207 - 302,632)	282,891 (258,011 - 310,671)	389,538 (360,153 - 423,267)	476,815 (440,153 - 517,644)	603 (367 - 909)	940 (572 - 1,416)	1,243 (759 - 1,875)
**Afghanistan**									
Under-5 years (rate per capita)	0.421 (0.327 - 0.499)	0.422 (0.331 - 0.500)	0.426 (0.330 - 0.507)	0.579 (0.350 - 0.773)	0.582 (0.352 - 0.778)	0.591 (0.357 - 0.790)	0.098 (0.041 - 0.196)	0.099 (0.042 - 0.200)	0.100 (0.043 - 0.204)
5-14 years (rate per capita)	0.531 (0.466 - 0.603)	0.530 (0.457 - 0.598)	0.529 (0.464 - 0.598)	1.101 (0.839 - 1.448)	1.105 (0.842 - 1.453)	1.087 (0.830 - 1.424)	0.565 (0.318 - 0.923)	0.569 (0.322 - 0.940)	0.572 (0.323 - 0.923)
15 - 49 years (rate per capita)	0.496 (0.452 - 0.536)	0.493 (0.450 - 0.530)	0.493 (0.451 - 0.534)	0.816 (0.700 - 0.928)	0.815 (0.700 - 0.931)	0.815 (0.699 - 0.931)	1.708 (1.012 - 2.703)	1.681 (0.996 - 2.654)	1.709 (1.012 - 2.710)
50 - 69 years (rate per capita)	0.609 (0.569 - 0.653)	0.607 (0.565 - 0.649)	0.609 (0.570 - 0.653)	0.896 (0.728 - 1.051)	0.896 (0.723 - 1.053)	0.896 (0.727 - 1.053)	5.739 (3.560 - 8.467)	5.578 (3.450 - 8.335)	5.537 (3.461 - 8.216)
70+ years (rate per capita)	0.657 (0.615 - 0.699)	0.650 (0.608 - 0.690)	0.652 (0.611 - 0.690)	0.905 (0.744 - 1.062)	0.900 (0.744 - 1.049)	0.900 (0.743 - 1.050)	9.007 (5.816 - 12.930)	8.927 (5.764 - 12.833)	8.875 (5.741 - 12.840)
All ages (rate per capita)	0.499 (0.463 - 0.531)	0.500 (0.463 - 0.532)	0.503 (0.470 - 0.535)	0.853 (0.753 - 0.959)	0.860 (0.760 - 0.969)	0.866 (0.770 - 0.973)	1.388 (0.839 - 2.124)	1.417 (0.856 - 2.173)	1.502 (0.910 - 2.297)
Age-standardised (rate per capita)	0.531 (0.502 - 0.559)	0.530 (0.503 - 0.555)	0.530 (0.504 - 0.557)	0.863 (0.790 - 0.944)	0.863 (0.790 - 0.944)	0.863 (0.790 - 0.944)	2.680 (1.663 - 3.967)	2.668 (1.654 - 3.951)	2.665 (1.659 - 3.948)
Total number of cases (thousands)	6,141 (5,700 - 6,537)	12,206 (11,312 - 12,991)	16,417 (15,314 - 17,446)	10,491 (9,271 - 11,800)	20,999 (18,569 - 23,670)	28,234 (25,119 - 31,719)	17 (10 - 26)	35 (21 - 53)	49 (30 - 75)
**Algeria**									
Under-5 years (rate per capita)	0.430 (0.332 - 0.509)	0.421 (0.329 - 0.502)	0.429 (0.336 - 0.505)	0.595 (0.359 - 0.795)	0.583 (0.352 - 0.780)	0.593 (0.358 - 0.792)	0.101 (0.043 - 0.206)	0.100 (0.041 - 0.202)	0.101 (0.042 - 0.200)
5-14 years (rate per capita)	0.530 (0.463 - 0.600)	0.523 (0.453 - 0.590)	0.531 (0.462 - 0.604)	1.091 (0.833 - 1.428)	1.049 (0.808 - 1.373)	1.112 (0.846 - 1.465)	0.575 (0.322 - 0.952)	0.578 (0.320 - 0.962)	0.574 (0.322 - 0.949)
15 - 49 years (rate per capita)	0.491 (0.448 - 0.534)	0.499 (0.457 - 0.540)	0.505 (0.465 - 0.545)	0.815 (0.698 - 0.933)	0.818 (0.705 - 0.932)	0.823 (0.713 - 0.935)	1.718 (1.011 - 2.703)	1.829 (1.076 - 2.880)	1.987 (1.176 - 3.136)
50 - 69 years (rate per capita)	0.610 (0.570 - 0.654)	0.611 (0.570 - 0.655)	0.610 (0.568 - 0.654)	0.898 (0.733 - 1.053)	0.896 (0.729 - 1.052)	0.897 (0.732 - 1.051)	5.968 (3.658 - 8.843)	5.945 (3.676 - 8.855)	5.929 (3.685 - 8.768)
70+ years (rate per capita)	0.649 (0.607 - 0.687)	0.647 (0.606 - 0.685)	0.640 (0.603 - 0.676)	0.897 (0.743 - 1.042)	0.895 (0.743 - 1.037)	0.887 (0.740 - 1.021)	9.176 (5.877 - 13.130)	9.170 (5.902 - 13.199)	9.041 (5.818 - 12.934)
All ages (rate per capita)	0.505 (0.472 - 0.538)	0.512 (0.482 - 0.544)	0.520 (0.491 - 0.548)	0.866 (0.772 - 0.971)	0.853 (0.773 - 0.942)	0.858 (0.781 - 0.944)	1.641 (0.990 - 2.501)	2.070 (1.260 - 3.155)	2.329 (1.429 - 3.512)
Age-standardised (rate per capita)	0.529 (0.502 - 0.558)	0.531 (0.503 - 0.559)	0.530 (0.503 - 0.557)	0.863 (0.790 - 0.944)	0.863 (0.790 - 0.944)	0.863 (0.790 - 0.944)	2.771 (1.697 - 4.116)	2.778 (1.700 - 4.137)	2.775 (1.695 - 4.111)
Total number of cases (thousands)	13,111 (12,243 - 13,969)	17,113 (16,093 - 18,171)	20,609 (19,445 - 21,701)	22,483 (20,037 - 25,212)	28,494 (25,807 - 31,462)	34,008 (30,958 - 37,427)	43 (26 - 65)	69 (42 - 105)	92 (57 - 139)
**Bahrain**									
Under-5 years (rate per capita)	0.427 (0.333 - 0.505)	0.422 (0.332 - 0.502)	0.428 (0.335 - 0.509)	0.593 (0.358 - 0.792)	0.585 (0.354 - 0.782)	0.595 (0.360 - 0.795)	0.101 (0.043 - 0.204)	0.100 (0.042 - 0.201)	0.102 (0.043 - 0.207)
5-14 years (rate per capita)	0.531 (0.462 - 0.597)	0.531 (0.465 - 0.597)	0.530 (0.460 - 0.596)	1.103 (0.840 - 1.451)	1.099 (0.838 - 1.443)	1.094 (0.835 - 1.432)	0.574 (0.322 - 0.942)	0.574 (0.319 - 0.941)	0.575 (0.320 - 0.946)
15 - 49 years (rate per capita)	0.502 (0.456 - 0.549)	0.508 (0.465 - 0.548)	0.510 (0.465 - 0.551)	0.824 (0.707 - 0.943)	0.827 (0.717 - 0.941)	0.828 (0.718 - 0.946)	1.916 (1.128 - 3.017)	2.051 (1.209 - 3.235)	2.083 (1.230 - 3.293)
50 - 69 years (rate per capita)	0.609 (0.570 - 0.655)	0.604 (0.561 - 0.651)	0.603 (0.561 - 0.647)	0.895 (0.721 - 1.051)	0.891 (0.718 - 1.045)	0.891 (0.712 - 1.054)	5.795 (3.581 - 8.630)	5.596 (3.490 - 8.355)	5.494 (3.417 - 8.191)
70+ years (rate per capita)	0.642 (0.604 - 0.679)	0.649 (0.609 - 0.688)	0.649 (0.606 - 0.685)	0.889 (0.743 - 1.025)	0.896 (0.742 - 1.039)	0.896 (0.742 - 1.040)	9.013 (5.833 - 12.888)	9.106 (5.881 - 13.071)	9.091 (5.891 - 13.040)
All ages (rate per capita)	0.506 (0.473 - 0.539)	0.514 (0.480 - 0.543)	0.519 (0.486 - 0.548)	0.851 (0.769 - 0.945)	0.859 (0.777 - 0.951)	0.856 (0.775 - 0.945)	1.759 (1.054 - 2.695)	1.985 (1.192 - 3.052)	2.220 (1.332 - 3.416)
Age-standardised (rate per capita)	0.530 (0.502 - 0.558)	0.530 (0.502 - 0.558)	0.530 (0.502 - 0.557)	0.864 (0.789 - 0.946)	0.864 (0.789 - 0.946)	0.864 (0.789 - 0.947)	2.763 (1.704 - 4.097)	2.756 (1.708 - 4.116)	2.759 (1.707 - 4.109)
Total number of cases (thousands)	253 (236 - 269)	443 (414 - 468)	710 (665 - 749)	426 (385 - 472)	741 (670 - 821)	1,170 (1,060 - 1,292)	1 (1 - 1)	2 (1 - 3)	3 (2 - 5)
**Egypt**									
Under-5 years (rate per capita)	0.426 (0.334 - 0.507)	0.424 (0.330 - 0.504)	0.426 (0.327 - 0.507)	0.592 (0.357 - 0.791)	0.588 (0.355 - 0.786)	0.589 (0.355 - 0.787)	0.101 (0.042 - 0.202)	0.100 (0.042 - 0.204)	0.100 (0.043 - 0.198)
5-14 years (rate per capita)	0.536 (0.473 - 0.601)	0.523 (0.464 - 0.585)	0.528 (0.472 - 0.592)	1.045 (0.788 - 1.383)	1.010 (0.768 - 1.328)	1.028 (0.775 - 1.360)	0.573 (0.321 - 0.945)	0.572 (0.315 - 0.935)	0.571 (0.315 - 0.940)
15 - 49 years (rate per capita)	0.496 (0.472 - 0.525)	0.492 (0.468 - 0.520)	0.497 (0.474 - 0.523)	0.805 (0.736 - 0.881)	0.804 (0.730 - 0.877)	0.809 (0.737 - 0.884)	1.849 (1.101 - 2.910)	1.827 (1.079 - 2.876)	1.918 (1.140 - 3.019)
50 - 69 years (rate per capita)	0.600 (0.570 - 0.631)	0.597 (0.564 - 0.629)	0.597 (0.567 - 0.628)	0.910 (0.806 - 1.014)	0.908 (0.805 - 1.017)	0.909 (0.808 - 1.016)	5.964 (3.665 - 8.820)	5.847 (3.601 - 8.718)	5.878 (3.620 - 8.715)
70+ years (rate per capita)	0.640 (0.614 - 0.668)	0.638 (0.610 - 0.666)	0.635 (0.608 - 0.663)	0.903 (0.800 - 1.008)	0.902 (0.801 - 1.007)	0.898 (0.801 - 0.999)	9.060 (5.869 - 13.125)	9.030 (5.838 - 13.007)	8.951 (5.846 - 12.897)
All ages (rate per capita)	0.510 (0.483 - 0.535)	0.507 (0.485 - 0.530)	0.511 (0.487 - 0.533)	0.846 (0.761 - 0.934)	0.838 (0.769 - 0.913)	0.840 (0.771 - 0.914)	1.898 (1.158 - 2.866)	2.040 (1.242 - 3.096)	2.129 (1.299 - 3.216)
Age-standardised (rate per capita)	0.527 (0.506 - 0.546)	0.524 (0.504 - 0.545)	0.525 (0.503 - 0.546)	0.850 (0.788 - 0.915)	0.849 (0.789 - 0.915)	0.849 (0.789 - 0.915)	2.752 (1.698 - 4.100)	2.748 (1.684 - 4.105)	2.743 (1.694 - 4.094)
Total number of cases (thousands)	28,739 (27,222 - 30,150)	37,959 (36,274 - 39,643)	46,596 (44,425 - 48,586)	47,665 (42,846 - 52,572)	62,711 (57,570 - 68,360)	76,537 (70,312 - 83,292)	107 (65 - 161)	153 (93 - 232)	194 (118 - 293)
**Iran**									
Under-5 years (rate per capita)	0.368 (0.306 - 0.431)	0.362 (0.329 - 0.403)	0.365 (0.308 - 0.426)	0.623 (0.377 - 0.822)	0.731 (0.566 - 0.852)	0.621 (0.376 - 0.820)	0.087 (0.037 - 0.173)	0.086 (0.036 - 0.171)	0.086 (0.037 - 0.174)
5-14 years (rate per capita)	0.638 (0.586 - 0.685)	0.637 (0.596 - 0.674)	0.637 (0.595 - 0.683)	1.071 (0.892 - 1.288)	0.987 (0.824 - 1.212)	1.064 (0.887 - 1.282)	0.628 (0.349 - 1.055)	0.628 (0.343 - 1.022)	0.630 (0.347 - 1.052)
15 - 49 years (rate per capita)	0.536 (0.512 - 0.564)	0.535 (0.510 - 0.562)	0.545 (0.522 - 0.570)	0.779 (0.714 - 0.851)	0.779 (0.717 - 0.851)	0.789 (0.726 - 0.860)	1.639 (0.942 - 2.663)	1.644 (0.939 - 2.636)	1.843 (1.063 - 2.983)
50 - 69 years (rate per capita)	0.625 (0.599 - 0.651)	0.619 (0.592 - 0.648)	0.620 (0.591 - 0.648)	0.884 (0.794 - 0.974)	0.881 (0.788 - 0.976)	0.880 (0.787 - 0.971)	4.760 (2.924 - 7.200)	4.604 (2.801 - 7.114)	4.593 (2.766 - 7.030)
70+ years (rate per capita)	0.647 (0.623 - 0.671)	0.644 (0.620 - 0.671)	0.637 (0.614 - 0.661)	0.883 (0.788 - 0.973)	0.880 (0.784 - 0.966)	0.874 (0.786 - 0.957)	6.874 (4.432 - 9.943)	6.792 (4.369 - 9.872)	6.691 (4.322 - 9.781)
All ages (rate per capita)	0.548 (0.525 - 0.568)	0.551 (0.535 - 0.568)	0.556 (0.540 - 0.575)	0.849 (0.777 - 0.930)	0.826 (0.773 - 0.885)	0.831 (0.779 - 0.890)	1.462 (0.862 - 2.299)	1.789 (1.056 - 2.784)	2.046 (1.213 - 3.179)

	Prevalence 1990	Prevalence 2005	Prevalence 2015	Incidence 1990	Incidence 2005	Incidence 2015	YLD rate 1990	YLD rate 2005	YLD rate 2015
Age-standardised (rate per capita)	0.563 (0.547 - 0.580)	0.566 (0.552 - 0.581)	0.563 (0.547 - 0.579)	0.838 (0.785 - 0.894)	0.845 (0.796 - 0.898)	0.838 (0.785 - 0.894)	2.315 (1.388 - 3.557)	2.292 (1.372 - 3.558)	2.303 (1.384 - 3.543)
Total number of cases (thousands)	30,645 (29,351 - 31,780)	38,884 (37,729 - 40,091)	43,973 (42,655 - 45,410)	47,488 (43,480 - 52,018)	58,306 (54,531 - 62,478)	65,694 (61,599 - 70,307)	82 (48 - 129)	126 (75 - 196)	162 (96 - 251)
**Iraq**									
Under-5 years (rate per capita)	0.424 (0.337 - 0.504)	0.422 (0.332 - 0.498)	0.425 (0.332 - 0.506)	0.586 (0.354 - 0.783)	0.586 (0.354 - 0.783)	0.587 (0.354 - 0.784)	0.099 (0.042 - 0.197)	0.100 (0.042 - 0.201)	0.100 (0.043 - 0.203)
5-14 years (rate per capita)	0.583 (0.526 - 0.641)	0.585 (0.525 - 0.641)	0.583 (0.526 - 0.644)	1.144 (0.907 - 1.466)	1.149 (0.909 - 1.472)	1.152 (0.910 - 1.477)	0.613 (0.336 - 1.022)	0.616 (0.339 - 1.028)	0.616 (0.337 - 1.025)
15 - 49 years (rate per capita)	0.550 (0.527 - 0.577)	0.553 (0.530 - 0.576)	0.555 (0.534 - 0.577)	0.933 (0.866 - 1.004)	0.935 (0.870 - 1.003)	0.938 (0.875 - 1.005)	1.715 (1.013 - 2.722)	1.767 (1.047 - 2.797)	1.860 (1.107 - 2.952)
50 - 69 years (rate per capita)	0.644 (0.619 - 0.668)	0.642 (0.617 - 0.668)	0.641 (0.615 - 0.665)	1.053 (0.972 - 1.134)	1.052 (0.971 - 1.133)	1.052 (0.971 - 1.134)	5.852 (3.604 - 8.659)	5.754 (3.591 - 8.531)	5.735 (3.577 - 8.530)
70+ years (rate per capita)	0.675 (0.653 - 0.697)	0.673 (0.650 - 0.695)	0.674 (0.652 - 0.698)	1.042 (0.968 - 1.114)	1.040 (0.966 - 1.110)	1.041 (0.967 - 1.113)	8.966 (5.821 - 12.958)	8.895 (5.774 - 12.706)	8.913 (5.782 - 12.861)
All ages (rate per capita)	0.548 (0.523 - 0.572)	0.550 (0.522 - 0.574)	0.551 (0.525 - 0.573)	0.946 (0.855 - 1.046)	0.947 (0.863 - 1.039)	0.948 (0.867 - 1.038)	1.624 (0.983 - 2.473)	1.657 (1.006 - 2.532)	1.706 (1.024 - 2.617)
Age-standardised (rate per capita)	0.575 (0.556 - 0.592)	0.575 (0.555 - 0.593)	0.574 (0.555 - 0.590)	0.970 (0.915 - 1.034)	0.970 (0.915 - 1.034)	0.970 (0.915 - 1.034)	2.745 (1.696 - 4.095)	2.742 (1.698 - 4.085)	2.740 (1.697 - 4.083)
Total number of cases (thousands)	9,576 (9,133 - 9,994)	14,879 (14,130 - 15,542)	20,053 (19,116 - 20,871)	16,525 (14,927 - 18,274)	25,631 (23,348 - 28,127)	34,509 (31,568 - 37,796)	28 (17 - 43)	45 (27 - 69)	62 (37 - 95)
**Jordan**									
Under-5 years (rate per capita)	0.317 (0.264 - 0.371)	0.302 (0.266 - 0.341)	0.319 (0.266 - 0.376)	0.536 (0.295 - 0.751)	0.342 (0.278 - 0.434)	0.542 (0.298 - 0.759)	0.075 (0.032 - 0.150)	0.072 (0.030 - 0.139)	0.076 (0.032 - 0.157)
5-14 years (rate per capita)	0.572 (0.510 - 0.632)	0.561 (0.497 - 0.618)	0.568 (0.502 - 0.627)	1.002 (0.806 - 1.243)	0.977 (0.777 - 1.229)	0.994 (0.799 - 1.236)	0.585 (0.328 - 0.965)	0.583 (0.330 - 0.974)	0.587 (0.327 - 0.974)
15 - 49 years (rate per capita)	0.490 (0.447 - 0.534)	0.496 (0.455 - 0.535)	0.501 (0.459 - 0.543)	0.813 (0.693 - 0.931)	0.818 (0.703 - 0.935)	0.820 (0.708 - 0.931)	1.650 (0.972 - 2.614)	1.790 (1.060 - 2.843)	1.914 (1.137 - 3.036)
50 - 69 years (rate per capita)	0.610 (0.569 - 0.657)	0.612 (0.574 - 0.655)	0.608 (0.566 - 0.651)	0.897 (0.729 - 1.053)	0.900 (0.740 - 1.054)	0.894 (0.721 - 1.052)	5.877 (3.665 - 8.751)	6.043 (3.767 - 8.967)	5.778 (3.580 - 8.605)
70+ years (rate per capita)	0.643 (0.608 - 0.680)	0.650 (0.610 - 0.688)	0.648 (0.611 - 0.686)	0.889 (0.741 - 1.024)	0.898 (0.742 - 1.044)	0.896 (0.743 - 1.039)	9.029 (5.880 - 12.911)	9.130 (5.948 - 13.112)	9.114 (5.905 - 13.064)
All ages (rate per capita)	0.499 (0.467 - 0.528)	0.496 (0.469 - 0.524)	0.506 (0.477 - 0.536)	0.833 (0.741 - 0.930)	0.797 (0.718 - 0.877)	0.833 (0.751 - 0.917)	1.557 (0.940 - 2.364)	1.732 (1.048 - 2.636)	1.904 (1.153 - 2.898)
Age-standardised (rate per capita)	0.528 (0.501 - 0.555)	0.524 (0.501 - 0.550)	0.527 (0.501 - 0.554)	0.844 (0.770 - 0.919)	0.822 (0.753 - 0.895)	0.843 (0.770 - 0.919)	2.758 (1.699 - 4.118)	2.761 (1.711 - 4.112)	2.763 (1.703 - 4.120)
Total number of cases (thousands)	1,664 (1,557 - 1,760)	2,659 (2,514 - 2,807)	3,835 (3,612 - 4,056)	2,776 (2,469 - 3,098)	4,267 (3,847 - 4,700)	6,307 (5,689 - 6,940)	5 (3 - 8)	9 (6 - 14)	14 (9 - 22)
**Kuwait**									
Under-5 years (rate per capita)	0.261 (0.230 - 0.299)	0.282 (0.247 - 0.318)	0.288 (0.238 - 0.335)	0.435 (0.365 - 0.523)	0.451 (0.339 - 0.610)	0.540 (0.287 - 0.784)	0.101 (0.043 - 0.200)	0.109 (0.047 - 0.211)	0.111 (0.046 - 0.226)
5-14 years (rate per capita)	0.548 (0.492 - 0.603)	0.558 (0.496 - 0.612)	0.569 (0.508 - 0.626)	1.020 (0.813 - 1.282)	0.995 (0.793 - 1.236)	1.065 (0.853 - 1.320)	0.757 (0.400 - 1.266)	0.759 (0.404 - 1.303)	0.761 (0.408 - 1.301)
15 - 49 years (rate per capita)	0.498 (0.458 - 0.536)	0.503 (0.459 - 0.545)	0.507 (0.465 - 0.549)	0.864 (0.744 - 0.976)	0.870 (0.749 - 0.988)	0.872 (0.753 - 0.996)	2.146 (1.253 - 3.421)	2.229 (1.300 - 3.562)	2.331 (1.367 - 3.739)
50 - 69 years (rate per capita)	0.600 (0.558 - 0.640)	0.603 (0.561 - 0.646)	0.600 (0.556 - 0.646)	0.936 (0.750 - 1.108)	0.937 (0.754 - 1.104)	0.935 (0.748 - 1.102)	5.847 (3.623 - 8.742)	5.919 (3.679 - 8.863)	5.788 (3.604 - 8.657)
70+ years (rate per capita)	0.637 (0.601 - 0.673)	0.654 (0.612 - 0.694)	0.648 (0.609 - 0.689)	0.930 (0.778 - 1.075)	0.950 (0.773 - 1.117)	0.940 (0.778 - 1.094)	9.256 (5.984 - 13.198)	9.553 (6.183 - 13.717)	9.460 (6.134 - 13.605)
All ages (rate per capita)	0.491 (0.465 - 0.517)	0.499 (0.469 - 0.527)	0.505 (0.477 - 0.537)	0.857 (0.769 - 0.944)	0.854 (0.767 - 0.943)	0.874 (0.786 - 0.964)	1.920 (1.130 - 2.983)	2.150 (1.264 - 3.350)	2.328 (1.376 - 3.628)
Age-standardised (rate per capita)	0.513 (0.489 - 0.538)	0.519 (0.495 - 0.543)	0.519 (0.495 - 0.546)	0.868 (0.790 - 0.941)	0.867 (0.792 - 0.943)	0.884 (0.806 - 0.963)	2.960 (1.811 - 4.475)	2.954 (1.802 - 4.487)	2.954 (1.811 - 4.467)
Total number of cases (thousands)	1,009 (954 - 1,062)	1,135 (1,067 - 1,199)	1,972 (1,862 - 2,094)	1,761 (1,580 - 1,938)	1,942 (1,745 - 2,144)	3,410 (3,065 - 3,760)	4 (2 - 6)	5 (3 - 8)	9 (5 - 14)
**Lebanon**									
Under-5 years (rate per capita)	0.432 (0.341 - 0.514)	0.436 (0.340 - 0.520)	0.417 (0.326 - 0.496)	0.597 (0.361 - 0.798)	0.603 (0.364 - 0.806)	0.577 (0.348 - 0.771)	0.101 (0.043 - 0.206)	0.103 (0.044 - 0.207)	0.098 (0.041 - 0.195)
5-14 years (rate per capita)	0.527 (0.458 - 0.596)	0.524 (0.459 - 0.592)	0.524 (0.458 - 0.591)	1.079 (0.825 - 1.413)	1.061 (0.813 - 1.385)	1.055 (0.811 - 1.381)	0.565 (0.317 - 0.935)	0.579 (0.324 - 0.941)	0.579 (0.322 - 0.960)
15 - 49 years (rate per capita)	0.498 (0.456 - 0.539)	0.502 (0.463 - 0.540)	0.502 (0.462 - 0.541)	0.818 (0.705 - 0.931)	0.820 (0.710 - 0.930)	0.821 (0.708 - 0.932)	1.800 (1.066 - 2.834)	1.898 (1.131 - 2.986)	1.915 (1.131 - 3.016)
50 - 69 years (rate per capita)	0.613 (0.572 - 0.656)	0.614 (0.575 - 0.657)	0.611 (0.571 - 0.654)	0.899 (0.739 - 1.053)	0.900 (0.740 - 1.054)	0.898 (0.731 - 1.053)	5.923 (3.687 - 8.757)	5.961 (3.701 - 8.820)	5.806 (3.600 - 8.611)
70+ years (rate per capita)	0.645 (0.607 - 0.683)	0.649 (0.609 - 0.687)	0.643 (0.609 - 0.680)	0.891 (0.743 - 1.029)	0.896 (0.742 - 1.039)	0.890 (0.743 - 1.025)	8.902 (5.695 - 12.799)	8.979 (5.718 - 12.956)	8.916 (5.724 - 12.838)
All ages (rate per capita)	0.517 (0.484 - 0.548)	0.525 (0.495 - 0.552)	0.525 (0.498 - 0.555)	0.863 (0.780 - 0.955)	0.868 (0.789 - 0.953)	0.859 (0.784 - 0.940)	2.109 (1.295 - 3.181)	2.413 (1.483 - 3.629)	2.577 (1.579 - 3.857)
Age-standardised (rate per capita)	0.531 (0.501 - 0.560)	0.530 (0.503 - 0.558)	0.531 (0.504 - 0.560)	0.863 (0.790 - 0.944)	0.863 (0.790 - 0.945)	0.863 (0.790 - 0.944)	2.709 (1.673 - 4.021)	2.719 (1.679 - 4.052)	2.728 (1.678 - 4.064)
Total number of cases (thousands)	1,413 (1,323 - 1,498)	2,071 (1,955 - 2,181)	3,026 (2,867 - 3,195)	2,357 (2,130 - 2,608)	3,425 (3,113 - 3,762)	4,951 (4,515 - 5,416)	6 (4 - 9)	10 (6 - 14)	15 (9 - 22)
**Libya**									
Under-5 years (rate per capita)	0.432 (0.341 - 0.512)	0.427 (0.332 - 0.505)	0.433 (0.341 - 0.516)	0.597 (0.361 - 0.798)	0.587 (0.354 - 0.784)	0.599 (0.362 - 0.801)	0.102 (0.042 - 0.207)	0.100 (0.042 - 0.204)	0.102 (0.044 - 0.206)
5-14 years (rate per capita)	0.529 (0.463 - 0.593)	0.527 (0.459 - 0.596)	0.529 (0.460 - 0.600)	1.081 (0.826 - 1.416)	1.071 (0.820 - 1.399)	1.092 (0.834 - 1.430)	0.576 (0.317 - 0.940)	0.576 (0.322 - 0.953)	0.575 (0.322 - 0.947)
15 - 49 years (rate per capita)	0.494 (0.450 - 0.536)	0.498 (0.454 - 0.540)	0.508 (0.469 - 0.548)	0.815 (0.699 - 0.931)	0.819 (0.705 - 0.935)	0.824 (0.717 - 0.934)	1.748 (1.034 - 2.749)	1.843 (1.091 - 2.928)	2.064 (1.219 - 3.246)
50 - 69 years (rate per capita)	0.611 (0.572 - 0.655)	0.609 (0.570 - 0.653)	0.607 (0.568 - 0.649)	0.898 (0.732 - 1.054)	0.897 (0.728 - 1.053)	0.895 (0.720 - 1.051)	5.960 (3.651 - 8.874)	5.922 (3.627 - 8.851)	5.807 (3.561 - 8.682)
70+ years (rate per capita)	0.653 (0.611 - 0.692)	0.648 (0.609 - 0.686)	0.645 (0.607 - 0.683)	0.900 (0.743 - 1.048)	0.897 (0.744 - 1.041)	0.893 (0.743 - 1.034)	9.266 (5.971 - 13.334)	9.200 (5.897 - 13.290)	9.104 (5.836 - 13.073)
All ages (rate per capita)	0.507 (0.474 - 0.539)	0.510 (0.477 - 0.541)	0.520 (0.491 - 0.549)	0.865 (0.772 - 0.970)	0.853 (0.772 - 0.942)	0.863 (0.785 - 0.952)	1.679 (1.010 - 2.556)	1.977 (1.193 - 3.014)	2.212 (1.343 - 3.362)
Age-standardised (rate per capita)	0.531 (0.504 - 0.558)	0.530 (0.502 - 0.559)	0.530 (0.503 - 0.558)	0.864 (0.790 - 0.945)	0.863 (0.790 - 0.945)	0.863 (0.790 - 0.944)	2.779 (1.708 - 4.132)	2.774 (1.706 - 4.133)	2.768 (1.702 - 4.135)
Total number of cases (thousands)	2,226 (2,080 - 2,365)	2,966 (2,774 - 3,144)	3,273 (3,089 - 3,455)	3,797 (3,387 - 4,257)	4,956 (4,489 - 5,472)	5,430 (4,939 - 5,993)	7 (4 - 11)	11 (7 - 18)	14 (8 - 21)
**Morocco**									
Under-5 years (rate per capita)	0.430 (0.337 - 0.508)	0.428 (0.334 - 0.511)	0.428 (0.331 - 0.511)	0.594 (0.359 - 0.794)	0.593 (0.358 - 0.792)	0.593 (0.358 - 0.793)	0.101 (0.043 - 0.203)	0.101 (0.043 - 0.205)	0.101 (0.043 - 0.199)
5-14 years (rate per capita)	0.529 (0.462 - 0.595)	0.526 (0.455 - 0.594)	0.528 (0.455 - 0.597)	1.091 (0.833 - 1.428)	1.064 (0.815 - 1.389)	1.086 (0.828 - 1.422)	0.573 (0.323 - 0.938)	0.576 (0.325 - 0.950)	0.575 (0.320 - 0.933)
15 - 49 years (rate per capita)	0.511 (0.468 - 0.552)	0.517 (0.479 - 0.554)	0.521 (0.480 - 0.558)	0.819 (0.701 - 0.939)	0.822 (0.712 - 0.933)	0.825 (0.714 - 0.936)	1.973 (1.134 - 3.200)	2.155 (1.250 - 3.424)	2.214 (1.282 - 3.517)
50 - 69 years (rate per capita)	0.650 (0.610 - 0.687)	0.646 (0.610 - 0.685)	0.643 (0.604 - 0.683)	0.902 (0.740 - 1.055)	0.900 (0.736 - 1.052)	0.899 (0.731 - 1.056)	6.565 (4.069 - 9.829)	6.473 (4.008 - 9.784)	6.341 (3.929 - 9.607)
70+ years (rate per capita)	0.676 (0.640 - 0.711)	0.674 (0.641 - 0.708)	0.667 (0.637 - 0.698)	0.898 (0.741 - 1.044)	0.898 (0.743 - 1.044)	0.891 (0.743 - 1.028)	9.744 (6.395 - 13.944)	9.781 (6.393 - 13.924)	9.597 (6.380 - 13.703)
All ages (rate per capita)	0.522 (0.489 - 0.553)	0.531 (0.502 - 0.558)	0.538 (0.509 - 0.566)	0.868 (0.775 - 0.970)	0.862 (0.781 - 0.947)	0.861 (0.785 - 0.946)	1.987 (1.186 - 3.065)	2.400 (1.442 - 3.678)	2.665 (1.611 - 4.121)
Age-standardised (rate per capita)	0.549 (0.521 - 0.575)	0.547 (0.521 - 0.572)	0.546 (0.519 - 0.574)	0.865 (0.792 - 0.946)	0.865 (0.792 - 0.946)	0.865 (0.792 - 0.946)	3.040 (1.852 - 4.642)	3.025 (1.854 - 4.612)	3.014 (1.845 - 4.624)
Total number of cases (thousands)	13,067 (12,234 - 13,846)	16,165 (15,288 - 16,984)	18,490 (17,498 - 19,472)	21,727 (19,409 - 24,283)	26,245 (23,770 - 28,839)	29,598 (26,983 - 32,513)	50 (30 - 77)	73 (44 - 112)	92 (55 - 142)
**Palestine**									
Under-5 years (rate per capita)	0.420 (0.321 - 0.500)	0.427 (0.332 - 0.505)	0.425 (0.337 - 0.503)	0.580 (0.351 - 0.776)	0.592 (0.358 - 0.791)	0.588 (0.355 - 0.786)	0.099 (0.042 - 0.199)	0.101 (0.043 - 0.204)	0.101 (0.042 - 0.203)
5-14 years (rate per capita)	0.530 (0.465 - 0.597)	0.529 (0.456 - 0.599)	0.527 (0.461 - 0.599)	1.098 (0.837 - 1.441)	1.087 (0.830 - 1.424)	1.090 (0.833 - 1.427)	0.572 (0.318 - 0.938)	0.576 (0.321 - 0.954)	0.576 (0.321 - 0.954)
15 - 49 years (rate per capita)	0.493 (0.451 - 0.535)	0.495 (0.453 - 0.536)	0.496 (0.455 - 0.538)	0.815 (0.700 - 0.929)	0.816 (0.700 - 0.931)	0.816 (0.700 - 0.931)	1.702 (1.008 - 2.695)	1.737 (1.029 - 2.743)	1.770 (1.047 - 2.787)
50 - 69 years (rate per capita)	0.607 (0.567 - 0.653)	0.610 (0.569 - 0.656)	0.607 (0.567 - 0.651)	0.895 (0.719 - 1.052)	0.897 (0.731 - 1.053)	0.895 (0.723 - 1.051)	5.764 (3.553 - 8.573)	5.864 (3.630 - 8.733)	5.774 (3.580 - 8.523)
70+ years (rate per capita)	0.648 (0.611 - 0.688)	0.650 (0.609 - 0.690)	0.650 (0.609 - 0.690)	0.896 (0.742 - 1.041)	0.898 (0.742 - 1.045)	0.898 (0.744 - 1.044)	9.113 (5.822 - 13.052)	9.145 (5.850 - 13.195)	9.132 (5.835 - 13.056)
All ages (rate per capita)	0.498 (0.462 - 0.531)	0.503 (0.470 - 0.536)	0.504 (0.471 - 0.537)	0.856 (0.756 - 0.965)	0.865 (0.766 - 0.972)	0.858 (0.766 - 0.960)	1.405 (0.842 - 2.152)	1.511 (0.907 - 2.314)	1.655 (0.993 - 2.524)
Age-standardised (rate per capita)	0.530 (0.504 - 0.558)	0.530 (0.503 - 0.559)	0.530 (0.502 - 0.560)	0.863 (0.790 - 0.944)	0.863 (0.790 - 0.944)	0.863 (0.790 - 0.944)	2.746 (1.682 - 4.080)	2.751 (1.700 - 4.093)	2.751 (1.683 - 4.087)
Total number of cases (thousands)	1,053 (977 - 1,122)	1,813 (1,692 - 1,932)	2,357 (2,202 - 2,509)	1,807 (1,596 - 2,039)	3,114 (2,761 - 3,499)	4,009 (3,581 - 4,486)	3 (2 - 5)	5 (3 - 8)	8 (5 - 12)
**Oman**									
Under-5 years (rate per capita)	0.377 (0.313 - 0.451)	0.377 (0.312 - 0.450)	0.372 (0.307 - 0.439)	0.581 (0.366 - 0.760)	0.569 (0.363 - 0.754)	0.560 (0.357 - 0.741)	0.090 (0.038 - 0.181)	0.090 (0.038 - 0.179)	0.088 (0.037 - 0.176)
5-14 years (rate per capita)	0.597 (0.534 - 0.652)	0.575 (0.509 - 0.633)	0.589 (0.530 - 0.647)	1.036 (0.852 - 1.259)	1.004 (0.811 - 1.236)	1.041 (0.846 - 1.272)	0.593 (0.330 - 0.985)	0.592 (0.327 - 0.984)	0.592 (0.332 - 0.972)
15 - 49 years (rate per capita)	0.502 (0.460 - 0.541)	0.500 (0.457 - 0.542)	0.505 (0.458 - 0.554)	0.821 (0.711 - 0.932)	0.820 (0.709 - 0.932)	0.828 (0.710 - 0.950)	1.913 (1.128 - 3.013)	1.854 (1.097 - 2.939)	1.976 (1.171 - 3.138)
50 - 69 years (rate per capita)	0.607 (0.563 - 0.652)	0.608 (0.569 - 0.651)	0.609 (0.565 - 0.655)	0.894 (0.718 - 1.050)	0.894 (0.720 - 1.052)	0.896 (0.719 - 1.053)	5.736 (3.516 - 8.579)	5.774 (3.582 - 8.588)	5.764 (3.566 - 8.575)
70+ years (rate per capita)	0.651 (0.614 - 0.688)	0.644 (0.608 - 0.681)	0.647 (0.609 - 0.684)	0.898 (0.743 - 1.044)	0.891 (0.742 - 1.029)	0.895 (0.744 - 1.038)	9.102 (5.840 - 12.982)	8.995 (5.802 - 12.889)	9.049 (5.833 - 12.984)
All ages (rate per capita)	0.514 (0.483 - 0.541)	0.515 (0.486 - 0.544)	0.515 (0.479 - 0.552)	0.841 (0.757 - 0.929)	0.843 (0.763 - 0.929)	0.838 (0.755 - 0.929)	1.566 (0.939 - 2.398)	1.776 (1.064 - 2.739)	2.085 (1.253 - 3.202)
Age-standardised (rate per capita)	0.536 (0.510 - 0.562)	0.535 (0.510 - 0.563)	0.534 (0.507 - 0.562)	0.851 (0.781 - 0.926)	0.850 (0.779 - 0.925)	0.851 (0.780 - 0.926)	2.757 (1.709 - 4.119)	2.768 (1.727 - 4.145)	2.764 (1.711 - 4.138)

	Prevalence 1990	Prevalence 2005	Prevalence 2015	Incidence 1990	Incidence 2005	Incidence 2015	YLD rate 1990	YLD rate 2005	YLD rate 2015
Total number of cases (thousands)	938 (882 - 987)	1,290 (1,217 - 1,364)	2,308 (2,148 - 2,472)	1,535 (1,381 - 1,695)	2,113 (1,911 - 2,328)	3,755 (3,384 - 4,164)	3 (2 - 4)	4 (3 - 7)	9 (6 - 14)
**Qatar**									
Under-5 years (rate per capita)	0.429 (0.336 - 0.512)	0.421 (0.329 - 0.498)	0.421 (0.332 - 0.501)	0.594 (0.359 - 0.794)	0.581 (0.351 - 0.776)	0.582 (0.351 - 0.777)	0.101 (0.042 - 0.207)	0.099 (0.042 - 0.197)	0.099 (0.042 - 0.199)
5-14 years (rate per capita)	0.534 (0.466 - 0.604)	0.531 (0.465 - 0.599)	0.528 (0.461 - 0.595)	1.123 (0.851 - 1.476)	1.107 (0.843 - 1.457)	1.087 (0.829 - 1.424)	0.574 (0.319 - 0.949)	0.575 (0.321 - 0.936)	0.574 (0.321 - 0.930)
15 - 49 years (rate per capita)	0.510 (0.462 - 0.557)	0.514 (0.471 - 0.557)	0.510 (0.458 - 0.557)	0.828 (0.715 - 0.950)	0.830 (0.722 - 0.949)	0.831 (0.715 - 0.956)	2.090 (1.239 - 3.308)	2.175 (1.290 - 3.438)	2.059 (1.217 - 3.265)
50 - 69 years (rate per capita)	0.603 (0.559 - 0.650)	0.602 (0.552 - 0.648)	0.602 (0.553 - 0.647)	0.891 (0.716 - 1.057)	0.889 (0.707 - 1.054)	0.891 (0.714 - 1.058)	5.495 (3.442 - 8.240)	5.387 (3.370 - 8.105)	5.441 (3.408 - 8.154)
70+ years (rate per capita)	0.639 (0.601 - 0.674)	0.648 (0.606 - 0.686)	0.659 (0.616 - 0.700)	0.886 (0.742 - 1.017)	0.894 (0.742 - 1.035)	0.904 (0.741 - 1.062)	8.988 (5.826 - 12.874)	9.100 (5.887 - 13.132)	9.390 (6.111 - 13.463)
All ages (rate per capita)	0.512 (0.478 - 0.546)	0.519 (0.485 - 0.549)	0.515 (0.474 - 0.552)	0.859 (0.775 - 0.953)	0.859 (0.777 - 0.953)	0.847 (0.757 - 0.949)	1.885 (1.121 - 2.958)	2.141 (1.282 - 3.321)	2.121 (1.275 - 3.295)
Age-standardised (rate per capita)	0.530 (0.502 - 0.559)	0.531 (0.502 - 0.561)	0.530 (0.501 - 0.559)	0.865 (0.790 - 0.947)	0.865 (0.790 - 0.947)	0.865 (0.790 - 0.948)	2.772 (1.713 - 4.133)	2.777 (1.710 - 4.154)	2.775 (1.717 - 4.146)
Total number of cases (thousands)	244 (228 - 260)	430 (402 - 456)	1,144 (1,052 - 1,226)	409 (369 - 454)	713 (644 - 790)	1,882 (1,681 - 2,108)	1 (1 - 1)	2 (1 - 3)	5 (3 - 7)
**Saudi Arabia**									
Under-5 years (rate per capita)	0.305 (0.279 - 0.332)	0.323 (0.294 - 0.351)	0.341 (0.310 - 0.369)	0.461 (0.262 - 0.678)	0.463 (0.275 - 0.667)	0.493 (0.292 - 0.698)	0.072 (0.031 - 0.145)	0.077 (0.032 - 0.156)	0.081 (0.035 - 0.164)
5-14 years (rate per capita)	0.537 (0.514 - 0.564)	0.531 (0.506 - 0.558)	0.538 (0.514 - 0.567)	0.771 (0.616 - 0.948)	0.793 (0.632 - 0.988)	0.830 (0.656 - 1.039)	0.570 (0.320 - 0.921)	0.570 (0.317 - 0.922)	0.574 (0.322 - 0.935)
15 - 49 years (rate per capita)	0.454 (0.443 - 0.465)	0.457 (0.446 - 0.468)	0.461 (0.450 - 0.472)	0.672 (0.608 - 0.742)	0.672 (0.611 - 0.740)	0.673 (0.611 - 0.735)	1.702 (1.006 - 2.641)	1.835 (1.090 - 2.849)	1.991 (1.190 - 3.090)
50 - 69 years (rate per capita)	0.560 (0.547 - 0.574)	0.553 (0.538 - 0.567)	0.547 (0.531 - 0.564)	0.733 (0.649 - 0.826)	0.720 (0.638 - 0.813)	0.714 (0.625 - 0.815)	5.863 (3.695 - 8.657)	5.579 (3.519 - 8.206)	5.511 (3.456 - 8.102)
70+ years (rate per capita)	0.610 (0.600 - 0.623)	0.607 (0.596 - 0.619)	0.602 (0.590 - 0.614)	0.727 (0.648 - 0.812)	0.712 (0.633 - 0.797)	0.709 (0.631 - 0.790)	9.071 (6.027 - 12.851)	9.054 (6.025 - 12.809)	9.006 (5.999 - 12.725)
All ages (rate per capita)	0.460 (0.448 - 0.471)	0.468 (0.458 - 0.478)	0.476 (0.467 - 0.485)	0.667 (0.603 - 0.744)	0.679 (0.621 - 0.743)	0.690 (0.634 - 0.749)	1.532 (0.928 - 2.326)	1.762 (1.068 - 2.674)	2.059 (1.257 - 3.120)
Age-standardised (rate per capita)	0.486 (0.477 - 0.494)	0.486 (0.478 - 0.494)	0.486 (0.478 - 0.495)	0.682 (0.632 - 0.732)	0.685 (0.634 - 0.739)	0.691 (0.639 - 0.748)	2.675 (1.671 - 3.970)	2.684 (1.673 - 3.987)	2.688 (1.677 - 4.000)
Total number of cases (thousands)	7,507 (7,315 - 7,679)	11,596 (11,339 - 11,831)	14,956 (14,666 - 15,241)	10,890 (9,840 - 12,139)	16,824 (15,388 - 18,401)	21,683 (19,925 - 23,541)	25 (15 - 38)	44 (26 - 66)	65 (40 - 98)
**Sudan**									
Under-5 years (rate per capita)	0.219 (0.178 - 0.263)	0.273 (0.227 - 0.315)	0.275 (0.222 - 0.318)	0.325 (0.201 - 0.574)	0.529 (0.271 - 0.770)	0.531 (0.272 - 0.773)	0.051 (0.021 - 0.108)	0.064 (0.027 - 0.128)	0.064 (0.027 - 0.130)
5-14 years (rate per capita)	0.545 (0.482 - 0.602)	0.564 (0.501 - 0.623)	0.561 (0.502 - 0.621)	1.021 (0.817 - 1.251)	1.019 (0.824 - 1.249)	1.012 (0.819 - 1.243)	0.574 (0.320 - 0.939)	0.580 (0.326 - 0.960)	0.581 (0.325 - 0.952)
15 - 49 years (rate per capita)	0.493 (0.450 - 0.535)	0.496 (0.456 - 0.536)	0.498 (0.457 - 0.537)	0.815 (0.700 - 0.930)	0.817 (0.702 - 0.930)	0.817 (0.703 - 0.930)	1.737 (1.035 - 2.724)	1.769 (1.053 - 2.794)	1.807 (1.072 - 2.838)
50 - 69 years (rate per capita)	0.608 (0.571 - 0.652)	0.609 (0.568 - 0.653)	0.609 (0.567 - 0.652)	0.897 (0.729 - 1.053)	0.897 (0.727 - 1.053)	0.896 (0.727 - 1.052)	5.838 (3.605 - 8.679)	5.807 (3.587 - 8.588)	5.805 (3.568 - 8.588)
70+ years (rate per capita)	0.649 (0.610 - 0.687)	0.648 (0.605 - 0.687)	0.648 (0.611 - 0.683)	0.897 (0.743 - 1.043)	0.896 (0.743 - 1.041)	0.895 (0.743 - 1.038)	9.047 (5.858 - 12.959)	9.007 (5.852 - 12.987)	8.986 (5.829 - 12.888)
All ages (rate per capita)	0.471 (0.442 - 0.500)	0.488 (0.460 - 0.516)	0.493 (0.463 - 0.520)	0.795 (0.714 - 0.892)	0.829 (0.736 - 0.927)	0.832 (0.744 - 0.926)	1.556 (0.945 - 2.365)	1.612 (0.978 - 2.456)	1.713 (1.036 - 2.605)
Age-standardised (rate per capita)	0.513 (0.488 - 0.541)	0.522 (0.497 - 0.547)	0.522 (0.496 - 0.548)	0.828 (0.757 - 0.902)	0.845 (0.773 - 0.923)	0.845 (0.773 - 0.923)	2.733 (1.681 - 4.063)	2.731 (1.677 - 4.053)	2.733 (1.673 - 4.078)
Total number of cases (thousands)	9,423 (8,849 - 10,003)	15,653 (14,754 - 16,544)	19,909 (18,707 - 21,003)	15,898 (14,276 - 17,850)	26,605 (23,623 - 29,739)	33,612 (30,044 - 37,383)	31 (19 - 47)	52 (31 - 79)	69 (42 - 105)
**Syria**									
Under-5 years (rate per capita)	0.282 (0.229 - 0.333)	0.276 (0.244 - 0.313)	0.281 (0.249 - 0.314)	0.531 (0.267 - 0.777)	0.516 (0.430 - 0.618)	0.525 (0.437 - 0.628)	0.066 (0.027 - 0.131)	0.065 (0.028 - 0.126)	0.066 (0.028 - 0.131)
5-14 years (rate per capita)	0.560 (0.500 - 0.622)	0.553 (0.489 - 0.612)	0.550 (0.485 - 0.610)	1.011 (0.810 - 1.247)	0.990 (0.784 - 1.229)	0.980 (0.779 - 1.214)	0.582 (0.326 - 0.963)	0.582 (0.323 - 0.964)	0.581 (0.324 - 0.969)
15 - 49 years (rate per capita)	0.490 (0.447 - 0.535)	0.495 (0.451 - 0.536)	0.497 (0.459 - 0.536)	0.814 (0.697 - 0.933)	0.816 (0.700 - 0.931)	0.817 (0.704 - 0.926)	1.686 (0.994 - 2.651)	1.771 (1.046 - 2.811)	1.830 (1.091 - 2.876)
50 - 69 years (rate per capita)	0.612 (0.576 - 0.654)	0.609 (0.569 - 0.651)	0.608 (0.568 - 0.652)	0.900 (0.738 - 1.054)	0.895 (0.725 - 1.051)	0.896 (0.727 - 1.051)	6.072 (3.742 - 9.005)	5.907 (3.650 - 8.791)	5.859 (3.621 - 8.766)
70+ years (rate per capita)	0.650 (0.611 - 0.686)	0.649 (0.610 - 0.687)	0.643 (0.605 - 0.679)	0.897 (0.743 - 1.042)	0.897 (0.743 - 1.042)	0.891 (0.742 - 1.030)	9.247 (5.964 - 13.266)	9.287 (5.959 - 13.382)	9.102 (5.839 - 13.067)
All ages (rate per capita)	0.487 (0.455 - 0.516)	0.492 (0.464 - 0.520)	0.499 (0.474 - 0.526)	0.832 (0.739 - 0.931)	0.827 (0.749 - 0.912)	0.832 (0.755 - 0.913)	1.527 (0.924 - 2.330)	1.720 (1.038 - 2.621)	1.909 (1.161 - 2.885)
Age-standardised (rate per capita)	0.522 (0.495 - 0.547)	0.520 (0.496 - 0.547)	0.520 (0.498 - 0.545)	0.844 (0.771 - 0.923)	0.840 (0.771 - 0.912)	0.840 (0.771 - 0.912)	2.782 (1.722 - 4.147)	2.788 (1.716 - 4.177)	2.768 (1.705 - 4.122)
Total number of cases (thousands)	6,058 (5,658 - 6,421)	8,938 (8,432 - 9,456)	9,291 (8,834 - 9,786)	10,346 (9,189 - 11,577)	15,036 (13,609 - 16,582)	15,495 (14,051 - 17,001)	19 (11 - 29)	31 (19 - 48)	36 (22 - 54)
**Tunisia**									
Under-5 years (rate per capita)	0.431 (0.340 - 0.513)	0.428 (0.330 - 0.508)	0.430 (0.336 - 0.511)	0.597 (0.361 - 0.798)	0.593 (0.358 - 0.793)	0.592 (0.357 - 0.791)	0.102 (0.042 - 0.207)	0.101 (0.042 - 0.207)	0.101 (0.043 - 0.206)
5-14 years (rate per capita)	0.544 (0.484 - 0.605)	0.551 (0.492 - 0.614)	0.553 (0.490 - 0.619)	1.038 (0.789 - 1.359)	1.011 (0.774 - 1.326)	1.042 (0.787 - 1.366)	0.585 (0.322 - 0.974)	0.596 (0.331 - 0.984)	0.593 (0.326 - 0.977)
15 - 49 years (rate per capita)	0.510 (0.484 - 0.540)	0.516 (0.491 - 0.542)	0.522 (0.498 - 0.549)	0.808 (0.736 - 0.885)	0.812 (0.744 - 0.884)	0.817 (0.749 - 0.891)	1.797 (1.058 - 2.815)	1.946 (1.141 - 3.058)	2.063 (1.216 - 3.271)
50 - 69 years (rate per capita)	0.612 (0.582 - 0.644)	0.611 (0.581 - 0.642)	0.609 (0.579 - 0.641)	0.917 (0.816 - 1.018)	0.915 (0.815 - 1.015)	0.915 (0.813 - 1.019)	5.905 (3.716 - 8.773)	5.864 (3.691 - 8.705)	5.782 (3.626 - 8.593)
70+ years (rate per capita)	0.656 (0.628 - 0.681)	0.651 (0.627 - 0.676)	0.641 (0.620 - 0.665)	0.915 (0.817 - 1.016)	0.911 (0.817 - 1.011)	0.900 (0.812 - 0.996)	9.187 (6.062 - 13.052)	9.110 (6.016 - 12.912)	8.893 (5.939 - 12.637)
All ages (rate per capita)	0.524 (0.499 - 0.547)	0.534 (0.513 - 0.555)	0.540 (0.518 - 0.560)	0.852 (0.776 - 0.935)	0.848 (0.788 - 0.913)	0.852 (0.796 - 0.913)	1.973 (1.196 - 2.993)	2.424 (1.487 - 3.687)	2.680 (1.655 - 4.077)
Age-standardised (rate per capita)	0.540 (0.520 - 0.559)	0.542 (0.520 - 0.562)	0.542 (0.520 - 0.562)	0.855 (0.794 - 0.920)	0.856 (0.798 - 0.920)	0.856 (0.798 - 0.920)	2.753 (1.707 - 4.140)	2.759 (1.711 - 4.140)	2.750 (1.709 - 4.140)
Total number of cases (thousands)	4,313 (4,109 - 4,502)	5,403 (5,186 - 5,618)	6,071 (5,828 - 6,303)	7,015 (6,393 - 7,701)	8,577 (7,974 - 9,234)	9,584 (8,952 - 10,267)	16 (10 - 25)	25 (15 - 37)	30 (19 - 46)
**Turkey**									
Under-5 years (rate per capita)	0.317 (0.262 - 0.365)	0.306 (0.257 - 0.360)	0.302 (0.250 - 0.354)	0.550 (0.309 - 0.770)	0.519 (0.318 - 0.738)	0.491 (0.284 - 0.724)	0.075 (0.032 - 0.149)	0.073 (0.030 - 0.146)	0.071 (0.031 - 0.146)
5-14 years (rate per capita)	0.598 (0.543 - 0.653)	0.591 (0.539 - 0.640)	0.590 (0.538 - 0.638)	0.970 (0.805 - 1.174)	0.966 (0.794 - 1.188)	0.961 (0.798 - 1.171)	0.606 (0.335 - 1.011)	0.605 (0.336 - 1.002)	0.605 (0.335 - 1.003)
15 - 49 years (rate per capita)	0.528 (0.505 - 0.553)	0.533 (0.510 - 0.559)	0.536 (0.514 - 0.559)	0.812 (0.744 - 0.883)	0.817 (0.750 - 0.886)	0.819 (0.753 - 0.888)	2.111 (1.277 - 3.220)	2.249 (1.354 - 3.448)	2.347 (1.428 - 3.590)
50 - 69 years (rate per capita)	0.634 (0.606 - 0.660)	0.638 (0.614 - 0.662)	0.635 (0.608 - 0.660)	0.926 (0.830 - 1.026)	0.927 (0.832 - 1.021)	0.926 (0.831 - 1.025)	7.489 (4.784 - 10.886)	7.698 (4.951 - 11.200)	7.560 (4.868 - 11.034)
70+ years (rate per capita)	0.689 (0.667 - 0.710)	0.702 (0.683 - 0.722)	0.688 (0.667 - 0.709)	0.911 (0.817 - 1.001)	0.921 (0.823 - 1.016)	0.913 (0.824 - 1.002)	12.207 (8.152 - 17.025)	12.727 (8.498 - 17.615)	12.183 (8.108 - 16.918)
All ages (rate per capita)	0.536 (0.515 - 0.556)	0.544 (0.528 - 0.562)	0.550 (0.532 - 0.566)	0.833 (0.769 - 0.901)	0.836 (0.778 - 0.899)	0.839 (0.785 - 0.897)	2.488 (1.560 - 3.659)	2.936 (1.852 - 4.296)	3.215 (2.042 - 4.699)
Age-standardised (rate per capita)	0.554 (0.537 - 0.571)	0.554 (0.539 - 0.570)	0.552 (0.534 - 0.567)	0.845 (0.790 - 0.901)	0.842 (0.788 - 0.902)	0.839 (0.786 - 0.898)	3.419 (2.173 - 4.967)	3.479 (2.221 - 5.075)	3.432 (2.190 - 5.015)
Total number of cases (thousands)	29,308 (28,163 - 30,382)	37,143 (36,033 - 38,351)	43,121 (41,749 - 44,387)	45,544 (41,997 - 49,238)	57,076 (53,130 - 61,358)	65,757 (61,557 - 70,313)	136 (85 - 200)	200 (126 - 293)	252 (160 - 368)
**United Arab Emirates**									
Under-5 years (rate per capita)	0.302 (0.252 - 0.345)	0.283 (0.234 - 0.336)	0.267 (0.212 - 0.325)	0.549 (0.336 - 0.764)	0.459 (0.262 - 0.705)	0.376 (0.222 - 0.685)	0.071 (0.030 - 0.140)	0.066 (0.028 - 0.132)	0.062 (0.026 - 0.130)
5-14 years (rate per capita)	0.577 (0.511 - 0.634)	0.550 (0.492 - 0.608)	0.534 (0.467 - 0.592)	1.040 (0.838 - 1.290)	0.995 (0.788 - 1.234)	1.021 (0.807 - 1.289)	0.585 (0.328 - 0.968)	0.581 (0.327 - 0.965)	0.575 (0.322 - 0.960)
15 - 49 years (rate per capita)	0.506 (0.458 - 0.552)	0.506 (0.455 - 0.555)	0.513 (0.466 - 0.561)	0.826 (0.713 - 0.944)	0.828 (0.711 - 0.948)	0.832 (0.722 - 0.955)	1.959 (1.140 - 3.128)	1.943 (1.127 - 3.088)	2.123 (1.238 - 3.405)
50 - 69 years (rate per capita)	0.597 (0.552 - 0.638)	0.596 (0.549 - 0.642)	0.597 (0.542 - 0.644)	0.889 (0.715 - 1.054)	0.888 (0.707 - 1.055)	0.888 (0.707 - 1.052)	5.034 (3.139 - 7.688)	4.974 (3.071 - 7.577)	5.007 (3.098 - 7.657)
70+ years (rate per capita)	0.641 (0.601 - 0.681)	0.636 (0.597 - 0.675)	0.641 (0.597 - 0.680)	0.896 (0.736 - 1.046)	0.894 (0.741 - 1.039)	0.896 (0.739 - 1.044)	8.318 (5.473 - 11.891)	8.196 (5.379 - 11.684)	8.402 (5.529 - 11.982)
All ages (rate per capita)	0.500 (0.468 - 0.531)	0.503 (0.464 - 0.540)	0.511 (0.474 - 0.549)	0.836 (0.753 - 0.925)	0.828 (0.739 - 0.923)	0.830 (0.745 - 0.929)	1.682 (0.993 - 2.645)	1.870 (1.102 - 2.946)	2.194 (1.294 - 3.472)
Age-standardised (rate per capita)	0.523 (0.496 - 0.550)	0.519 (0.493 - 0.547)	0.514 (0.486 - 0.541)	0.849 (0.775 - 0.924)	0.840 (0.768 - 0.915)	0.832 (0.757 - 0.910)	2.567 (1.583 - 3.931)	2.582 (1.574 - 3.950)	2.605 (1.617 - 3.959)
Total number of cases (thousands)	902 (844 - 958)	2,255 (2,080 - 2,423)	4,673 (4,331 - 5,021)	1,508 (1,359 - 1,668)	3,714 (3,315 - 4,142)	7,591 (6,815 - 8,497)	3 (2 - 5)	8 (5 - 13)	20 (12 - 32)
**Yemen**									
Under-5 years (rate per capita)	0.414 (0.321 - 0.490)	0.423 (0.329 - 0.502)	0.430 (0.340 - 0.506)	0.576 (0.348 - 0.769)	0.586 (0.354 - 0.784)	0.590 (0.356 - 0.788)	0.098 (0.042 - 0.193)	0.099 (0.043 - 0.201)	0.100 (0.042 - 0.200)
5-14 years (rate per capita)	0.533 (0.469 - 0.600)	0.526 (0.459 - 0.593)	0.529 (0.462 - 0.597)	1.109 (0.844 - 1.460)	1.079 (0.825 - 1.413)	1.089 (0.832 - 1.426)	0.569 (0.319 - 0.936)	0.572 (0.318 - 0.932)	0.572 (0.318 - 0.941)
15 - 49 years (rate per capita)	0.487 (0.447 - 0.526)	0.485 (0.440 - 0.530)	0.487 (0.441 - 0.527)	0.813 (0.698 - 0.928)	0.812 (0.696 - 0.928)	0.814 (0.697 - 0.935)	1.640 (0.977 - 2.568)	1.607 (0.952 - 2.542)	1.632 (0.967 - 2.576)
50 - 69 years (rate per capita)	0.590 (0.546 - 0.631)	0.586 (0.538 - 0.631)	0.589 (0.546 - 0.635)	0.894 (0.728 - 1.049)	0.892 (0.717 - 1.048)	0.896 (0.732 - 1.051)	5.573 (3.451 - 8.199)	5.417 (3.364 - 8.006)	5.615 (3.475 - 8.212)
70+ years (rate per capita)	0.638 (0.598 - 0.679)	0.634 (0.598 - 0.670)	0.634 (0.595 - 0.676)	0.898 (0.742 - 1.046)	0.893 (0.743 - 1.035)	0.897 (0.739 - 1.042)	8.868 (5.719 - 12.743)	8.844 (5.741 - 12.741)	8.857 (5.718 - 12.752)
All ages (rate per capita)	0.494 (0.458 - 0.528)	0.497 (0.461 - 0.530)	0.499 (0.463 - 0.532)	0.858 (0.752 - 0.970)	0.860 (0.762 - 0.971)	0.859 (0.767 - 0.961)	1.341 (0.811 - 2.032)	1.450 (0.877 - 2.202)	1.558 (0.954 - 2.356)
Age-standardised (rate per capita)	0.522 (0.496 - 0.549)	0.521 (0.493 - 0.550)	0.522 (0.493 - 0.549)	0.862 (0.788 - 0.943)	0.862 (0.789 - 0.943)	0.862 (0.789 - 0.943)	2.632 (1.629 - 3.864)	2.633 (1.630 - 3.873)	2.633 (1.624 - 3.871)
Total number of cases (thousands)	5,907 (5,483 - 6,313)	10,213 (9,481 - 10,896)	13,440 (12,466 - 14,316)	10,261 (8,996 - 11,609)	17,695 (15,681 - 19,979)	23,114 (20,642 - 25,855)	16 (10 - 24)	30 (18 - 45)	42 (26 - 63)

	Prevalence 1990	Prevalence 2005	Prevalence 2015	Incidence 1990	Incidence 2005	Incidence 2015	YLD rate 1990	YLD rate 2005	YLD rate 2015
**South Asia**									
**Under-5 years (rate per capita)**	0.458<br>(0.424 - 0.489)	0.452<br>(0.418 - 0.482)	0.450<br>(0.416 - 0.480)	0.587<br>(0.403 - 0.748)	0.557<br>(0.392 - 0.701)	0.554<br>(0.394 - 0.695)	0.108<br>(0.045 - 0.210)	0.107<br>(0.046 - 0.208)	0.106<br>(0.045 - 0.207)
**5-14 years (rate per capita)**	0.473<br>(0.448 - 0.494)	0.463<br>(0.438 - 0.486)	0.452<br>(0.428 - 0.472)	0.844<br>(0.637 - 1.106)	0.825<br>(0.625 - 1.089)	0.791<br>(0.588 - 1.053)	0.525<br>(0.297 - 0.842)	0.524<br>(0.296 - 0.845)	0.521<br>(0.293 - 0.837)
**15 - 49 years (rate per capita)**	0.463<br>(0.453 - 0.473)	0.459<br>(0.449 - 0.469)	0.462<br>(0.453 - 0.472)	0.716<br>(0.645 - 0.789)	0.715<br>(0.647 - 0.784)	0.719<br>(0.649 - 0.788)	1.631<br>(0.933 - 2.619)	1.664<br>(0.952 - 2.665)	1.700<br>(0.970 - 2.731)
**50 - 69 years (rate per capita)**	0.568<br>(0.557 - 0.579)	0.569<br>(0.558 - 0.579)	0.569<br>(0.557 - 0.579)	0.755<br>(0.666 - 0.851)	0.755<br>(0.671 - 0.852)	0.755<br>(0.668 - 0.852)	4.803<br>(2.938 - 7.351)	4.819<br>(2.940 - 7.381)	4.830<br>(2.957 - 7.393)
**70+ years (rate per capita)**	0.577<br>(0.567 - 0.588)	0.575<br>(0.563 - 0.585)	0.571<br>(0.560 - 0.581)	0.650<br>(0.573 - 0.735)	0.647<br>(0.574 - 0.726)	0.643<br>(0.568 - 0.718)	7.089<br>(4.590 - 10.252)	7.037<br>(4.571 - 10.175)	7.001<br>(4.530 - 10.133)
**All ages (rate per capita)**	0.478<br>(0.469 - 0.489)	0.475<br>(0.465 - 0.484)	0.477<br>(0.468 - 0.485)	0.731<br>(0.661 - 0.805)	0.724<br>(0.662 - 0.789)	0.719<br>(0.662 - 0.783)	1.583<br>(0.933 - 2.447)	1.740<br>(1.030 - 2.685)	1.909<br>(1.132 - 2.945)
**Age-standardised (rate per capita)**	0.492<br>(0.485 - 0.500)	0.487<br>(0.479 - 0.495)	0.486<br>(0.478 - 0.494)	0.730<br>(0.675 - 0.789)	0.724<br>(0.671 - 0.780)	0.718<br>(0.665 - 0.775)	2.304<br>(1.400 - 3.538)	2.299<br>(1.398 - 3.521)	2.301<br>(1.397 - 3.541)
**Total number of cases (thousands)**	527,716<br>(517,297 - 539,437)	696,896<br>(682,453 - 710,394)	806,744<br>(791,113 - 819,881)	807,275<br>(729,990 - 888,492)	1,061,593<br>(971,487 - 1,157,650)	1,216,148<br>(1,119,223 - 1,324,569)	1,747<br>(1,030 - 2,701)	2,553<br>(1,511 - 3,939)	3,227<br>(1,913 - 4,980)
**South Asia**									
**Under-5 years (rate per capita)**	0.458<br>(0.424 - 0.489)	0.452<br>(0.418 - 0.482)	0.450<br>(0.416 - 0.480)	0.587<br>(0.403 - 0.748)	0.557<br>(0.392 - 0.701)	0.554<br>(0.394 - 0.695)	0.108<br>(0.045 - 0.210)	0.107<br>(0.046 - 0.208)	0.106<br>(0.045 - 0.207)
**5-14 years (rate per capita)**	0.473<br>(0.448 - 0.494)	0.463<br>(0.438 - 0.486)	0.452<br>(0.428 - 0.472)	0.844<br>(0.637 - 1.106)	0.825<br>(0.625 - 1.089)	0.791<br>(0.588 - 1.053)	0.525<br>(0.297 - 0.842)	0.524<br>(0.296 - 0.845)	0.521<br>(0.293 - 0.837)
**15 - 49 years (rate per capita)**	0.463<br>(0.453 - 0.473)	0.459<br>(0.449 - 0.469)	0.462<br>(0.453 - 0.472)	0.716<br>(0.645 - 0.789)	0.715<br>(0.647 - 0.784)	0.719<br>(0.649 - 0.788)	1.631<br>(0.933 - 2.619)	1.664<br>(0.952 - 2.665)	1.700<br>(0.970 - 2.731)
**50 - 69 years (rate per capita)**	0.568<br>(0.557 - 0.579)	0.569<br>(0.558 - 0.579)	0.569<br>(0.557 - 0.579)	0.755<br>(0.666 - 0.851)	0.755<br>(0.671 - 0.852)	0.755<br>(0.668 - 0.852)	4.803<br>(2.938 - 7.351)	4.819<br>(2.940 - 7.381)	4.830<br>(2.957 - 7.393)
**70+ years (rate per capita)**	0.577<br>(0.567 - 0.588)	0.575<br>(0.563 - 0.585)	0.571<br>(0.560 - 0.581)	0.650<br>(0.573 - 0.735)	0.647<br>(0.574 - 0.726)	0.643<br>(0.568 - 0.718)	7.089<br>(4.590 - 10.252)	7.037<br>(4.571 - 10.175)	7.001<br>(4.530 - 10.133)
**All ages (rate per capita)**	0.478<br>(0.469 - 0.489)	0.475<br>(0.465 - 0.484)	0.477<br>(0.468 - 0.485)	0.731<br>(0.661 - 0.805)	0.724<br>(0.662 - 0.789)	0.719<br>(0.662 - 0.783)	1.583<br>(0.933 - 2.447)	1.740<br>(1.030 - 2.685)	1.909<br>(1.132 - 2.945)
**Age-standardised (rate per capita)**	0.492<br>(0.485 - 0.500)	0.487<br>(0.479 - 0.495)	0.486<br>(0.478 - 0.494)	0.730<br>(0.675 - 0.789)	0.724<br>(0.671 - 0.780)	0.718<br>(0.665 - 0.775)	2.304<br>(1.400 - 3.538)	2.299<br>(1.398 - 3.521)	2.301<br>(1.397 - 3.541)
**Total number of cases (thousands)**	527,716<br>(517,297 - 539,437)	696,896<br>(682,453 - 710,394)	806,744<br>(791,113 - 819,881)	807,275<br>(729,990 - 888,492)	1,061,593<br>(971,487 - 1,157,650)	1,216,148<br>(1,119,223 - 1,324,569)	1,747<br>(1,030 - 2,701)	2,553<br>(1,511 - 3,939)	3,227<br>(1,913 - 4,980)
**Bangladesh**									
**Under-5 years (rate per capita)**	0.473<br>(0.403 - 0.541)	0.478<br>(0.406 - 0.545)	0.478<br>(0.401 - 0.541)	0.570<br>(0.399 - 0.730)	0.578<br>(0.405 - 0.740)	0.577<br>(0.405 - 0.740)	0.111<br>(0.047 - 0.222)	0.113<br>(0.048 - 0.220)	0.113<br>(0.047 - 0.228)
**5-14 years (rate per capita)**	0.440<br>(0.381 - 0.515)	0.440<br>(0.383 - 0.510)	0.439<br>(0.385 - 0.506)	0.865<br>(0.590 - 1.169)	0.853<br>(0.585 - 1.149)	0.843<br>(0.583 - 1.134)	0.507<br>(0.287 - 0.819)	0.512<br>(0.289 - 0.836)	0.514<br>(0.288 - 0.839)
**15 - 49 years (rate per capita)**	0.439<br>(0.414 - 0.471)	0.442<br>(0.417 - 0.470)	0.444<br>(0.419 - 0.470)	0.688<br>(0.608 - 0.777)	0.694<br>(0.617 - 0.778)	0.699<br>(0.623 - 0.781)	1.353<br>(0.755 - 2.204)	1.411<br>(0.788 - 2.313)	1.470<br>(0.821 - 2.448)
**50 - 69 years (rate per capita)**	0.529<br>(0.499 - 0.561)	0.530<br>(0.501 - 0.561)	0.528<br>(0.495 - 0.562)	0.732<br>(0.632 - 0.837)	0.730<br>(0.633 - 0.836)	0.733<br>(0.631 - 0.838)	3.340<br>(1.917 - 5.341)	3.404<br>(1.958 - 5.443)	3.321<br>(1.905 - 5.341)
**70+ years (rate per capita)**	0.528<br>(0.498 - 0.561)	0.518<br>(0.492 - 0.549)	0.511<br>(0.485 - 0.541)	0.639<br>(0.551 - 0.736)	0.629<br>(0.545 - 0.716)	0.621<br>(0.539 - 0.702)	4.387<br>(2.739 - 6.701)	4.306<br>(2.675 - 6.538)	4.269<br>(2.667 - 6.440)
**All ages (rate per capita)**	0.454<br>(0.431 - 0.480)	0.456<br>(0.432 - 0.479)	0.458<br>(0.437 - 0.478)	0.720<br>(0.634 - 0.814)	0.719<br>(0.645 - 0.801)	0.718<br>(0.650 - 0.794)	1.165<br>(0.667 - 1.853)	1.317<br>(0.749 - 2.115)	1.452<br>(0.832 - 2.330)
**Age-standardised (rate per capita)**	0.466<br>(0.449 - 0.487)	0.466<br>(0.446 - 0.486)	0.466<br>(0.447 - 0.485)	0.715<br>(0.653 - 0.783)	0.715<br>(0.653 - 0.783)	0.715<br>(0.653 - 0.783)	1.720<br>(0.989 - 2.738)	1.726<br>(0.989 - 2.746)	1.734<br>(0.999 - 2.757)
**Total number of cases (thousands)**	48,169<br>(45,688 - 50,828)	65,127<br>(61,768 - 68,362)	73,690<br>(70,331 - 76,908)	76,341<br>(67,215 - 86,252)	102,660<br>(92,134 - 114,419)	115,501<br>(104,638 - 127,747)	124<br>(71 - 196)	188<br>(107 - 302)	234<br>(134 - 375)
**Bhutan**									
**Under-5 years (rate per capita)**	0.475<br>(0.400 - 0.539)	0.480<br>(0.411 - 0.545)	0.479<br>(0.407 - 0.547)	0.573<br>(0.402 - 0.734)	0.576<br>(0.404 - 0.738)	0.579<br>(0.406 - 0.741)	0.112<br>(0.048 - 0.226)	0.113<br>(0.047 - 0.222)	0.113<br>(0.048 - 0.226)
**5-14 years (rate per capita)**	0.438<br>(0.380 - 0.515)	0.438<br>(0.384 - 0.505)	0.439<br>(0.378 - 0.506)	0.867<br>(0.590 - 1.173)	0.846<br>(0.583 - 1.138)	0.851<br>(0.585 - 1.146)	0.510<br>(0.285 - 0.823)	0.515<br>(0.291 - 0.829)	0.514<br>(0.290 - 0.840)
**15 - 49 years (rate per capita)**	0.445<br>(0.419 - 0.473)	0.442<br>(0.416 - 0.470)	0.444<br>(0.420 - 0.473)	0.691<br>(0.613 - 0.772)	0.690<br>(0.609 - 0.779)	0.702<br>(0.625 - 0.786)	1.509<br>(0.863 - 2.471)	1.457<br>(0.825 - 2.372)	1.557<br>(0.884 - 2.545)
**50 - 69 years (rate per capita)**	0.537<br>(0.504 - 0.566)	0.537<br>(0.506 - 0.568)	0.538<br>(0.506 - 0.569)	0.734<br>(0.633 - 0.839)	0.732<br>(0.633 - 0.836)	0.733<br>(0.631 - 0.836)	4.037<br>(2.401 - 6.303)	4.115<br>(2.443 - 6.422)	4.081<br>(2.419 - 6.374)
**70+ years (rate per capita)**	0.542<br>(0.515 - 0.572)	0.537<br>(0.511 - 0.567)	0.531<br>(0.506 - 0.559)	0.637<br>(0.551 - 0.730)	0.631<br>(0.547 - 0.719)	0.621<br>(0.539 - 0.702)	5.759<br>(3.529 - 8.598)	5.746<br>(3.512 - 8.635)	5.674<br>(3.477 - 8.496)
**All ages (rate per capita)**	0.458<br>(0.434 - 0.484)	0.456<br>(0.434 - 0.478)	0.459<br>(0.438 - 0.481)	0.722<br>(0.635 - 0.814)	0.716<br>(0.641 - 0.799)	0.720<br>(0.654 - 0.795)	1.313<br>(0.763 - 2.061)	1.445<br>(0.839 - 2.266)	1.656<br>(0.967 - 2.577)
**Age-standardised (rate per capita)**	0.470<br>(0.451 - 0.488)	0.470<br>(0.451 - 0.489)	0.470<br>(0.450 - 0.490)	0.716<br>(0.653 - 0.784)	0.716<br>(0.653 - 0.783)	0.715<br>(0.653 - 0.783)	2.008<br>(1.181 - 3.109)	2.011<br>(1.185 - 3.106)	2.009<br>(1.184 - 3.098)
**Total number of cases (thousands)**	245<br>(232 - 259)	297<br>(283 - 311)	356<br>(339 - 372)	386<br>(339 - 435)	466<br>(417 - 521)	558<br>(506 - 616)	1<br>(0 - 1)	1<br>(1 - 1)	1<br>(1 - 2)
**India**									
**Under-5 years (rate per capita)**	0.465<br>(0.428 - 0.501)	0.470<br>(0.435 - 0.503)	0.472<br>(0.436 - 0.507)	0.597<br>(0.409 - 0.756)	0.595<br>(0.411 - 0.757)	0.601<br>(0.417 - 0.764)	0.110<br>(0.046 - 0.214)	0.111<br>(0.047 - 0.218)	0.112<br>(0.047 - 0.216)
**5-14 years (rate per capita)**	0.479<br>(0.456 - 0.501)	0.477<br>(0.453 - 0.503)	0.465<br>(0.442 - 0.485)	0.842<br>(0.640 - 1.101)	0.813<br>(0.617 - 1.067)	0.769<br>(0.576 - 1.016)	0.528<br>(0.300 - 0.848)	0.530<br>(0.299 - 0.851)	0.527<br>(0.297 - 0.845)
**15 - 49 years (rate per capita)**	0.468<br>(0.457 - 0.478)	0.464<br>(0.454 - 0.473)	0.467<br>(0.459 - 0.477)	0.724<br>(0.653 - 0.796)	0.722<br>(0.655 - 0.790)	0.726<br>(0.660 - 0.794)	1.682<br>(0.962 - 2.694)	1.719<br>(0.986 - 2.762)	1.756<br>(1.004 - 2.819)
**50 - 69 years (rate per capita)**	0.576<br>(0.566 - 0.587)	0.576<br>(0.565 - 0.585)	0.576<br>(0.565 - 0.587)	0.760<br>(0.672 - 0.855)	0.760<br>(0.674 - 0.857)	0.760<br>(0.675 - 0.855)	5.028<br>(3.094 - 7.666)	5.025<br>(3.084 - 7.667)	5.056<br>(3.103 - 7.713)
**70+ years (rate per capita)**	0.587<br>(0.576 - 0.597)	0.585<br>(0.573 - 0.595)	0.582<br>(0.571 - 0.592)	0.654<br>(0.576 - 0.737)	0.652<br>(0.579 - 0.732)	0.647<br>(0.572 - 0.720)	7.474<br>(4.858 - 10.731)	7.471<br>(4.876 - 10.712)	7.442<br>(4.840 - 10.683)
**All ages (rate per capita)**	0.484<br>(0.475 - 0.495)	0.484<br>(0.474 - 0.492)	0.487<br>(0.479 - 0.495)	0.737<br>(0.671 - 0.809)	0.730<br>(0.670 - 0.795)	0.725<br>(0.669 - 0.788)	1.665<br>(0.985 - 2.566)	1.839<br>(1.092 - 2.827)	2.027<br>(1.214 - 3.122)
**Age-standardised (rate per capita)**	0.498<br>(0.490 - 0.506)	0.495<br>(0.487 - 0.503)	0.495<br>(0.487 - 0.503)	0.735<br>(0.681 - 0.793)	0.730<br>(0.676 - 0.784)	0.723<br>(0.671 - 0.781)	2.394<br>(1.460 - 3.673)	2.393<br>(1.459 - 3.663)	2.395<br>(1.460 - 3.681)
**Total number of cases (thousands)**	421,556<br>(413,692 - 430,956)	553,809<br>(542,126 - 563,408)	638,384<br>(627,720 - 648,668)	641,456<br>(584,070 - 704,167)	835,018<br>(766,758 - 909,331)	950,658<br>(877,829 - 1,032,917)	1,449<br>(858 - 2,234)	2,104<br>(1,250 - 3,235)	2,658<br>(1,592 - 4,094)
**Nepal**									
**Under-5 years (rate per capita)**	0.501<br>(0.448 - 0.544)	0.514<br>(0.461 - 0.559)	0.512<br>(0.460 - 0.560)	0.541<br>(0.395 - 0.670)	0.567<br>(0.431 - 0.704)	0.556<br>(0.406 - 0.687)	0.118<br>(0.051 - 0.227)	0.122<br>(0.053 - 0.235)	0.122<br>(0.053 - 0.232)
**5-14 years (rate per capita)**	0.459<br>(0.413 - 0.516)	0.470<br>(0.431 - 0.520)	0.454<br>(0.406 - 0.509)	0.818<br>(0.617 - 1.065)	0.829<br>(0.637 - 1.072)	0.793<br>(0.599 - 1.039)	0.506<br>(0.288 - 0.826)	0.515<br>(0.286 - 0.824)	0.513<br>(0.288 - 0.831)
**15 - 49 years (rate per capita)**	0.425<br>(0.401 - 0.450)	0.428<br>(0.406 - 0.455)	0.425<br>(0.401 - 0.450)	0.674<br>(0.602 - 0.751)	0.675<br>(0.606 - 0.747)	0.674<br>(0.603 - 0.749)	1.397<br>(0.796 - 2.265)	1.438<br>(0.810 - 2.340)	1.419<br>(0.807 - 2.298)
**50 - 69 years (rate per capita)**	0.513<br>(0.484 - 0.542)	0.516<br>(0.489 - 0.547)	0.514<br>(0.487 - 0.543)	0.720<br>(0.632 - 0.820)	0.716<br>(0.636 - 0.814)	0.718<br>(0.632 - 0.818)	3.576<br>(2.109 - 5.551)	3.639<br>(2.151 - 5.715)	3.639<br>(2.158 - 5.719)
**70+ years (rate per capita)**	0.515<br>(0.490 - 0.546)	0.513<br>(0.488 - 0.542)	0.510<br>(0.485 - 0.538)	0.627<br>(0.548 - 0.713)	0.620<br>(0.543 - 0.703)	0.620<br>(0.546 - 0.702)	4.873<br>(3.088 - 7.377)	4.863<br>(3.072 - 7.287)	4.887<br>(3.104 - 7.389)
**All ages (rate per capita)**	0.456<br>(0.436 - 0.476)	0.462<br>(0.444 - 0.483)	0.454<br>(0.436 - 0.475)	0.693<br>(0.621 - 0.769)	0.704<br>(0.634 - 0.782)	0.693<br>(0.629 - 0.760)	1.231<br>(0.714 - 1.936)	1.334<br>(0.766 - 2.118)	1.472<br>(0.850 - 2.305)
**Age-standardised (rate per capita)**	0.460<br>(0.444 - 0.476)	0.464<br>(0.449 - 0.482)	0.460<br>(0.443 - 0.477)	0.693<br>(0.638 - 0.750)	0.696<br>(0.641 - 0.756)	0.693<br>(0.639 - 0.750)	1.801<br>(1.057 - 2.822)	1.826<br>(1.063 - 2.857)	1.813<br>(1.062 - 2.838)
**Total number of cases (thousands)**	8,545<br>(8,174 - 8,916)	11,789<br>(11,326 - 12,330)	12,976<br>(12,442 - 13,559)	12,989<br>(11,639 - 14,405)	17,956<br>(16,183 - 19,947)	19,785<br>(17,972 - 21,694)	23<br>(13 - 36)	34<br>(20 - 54)	42<br>(24 - 66)
**Pakistan**									
**Under-5 years (rate per capita)**	0.395<br>(0.328 - 0.473)	0.313<br>(0.270 - 0.363)	0.313<br>(0.264 - 0.366)	0.544<br>(0.331 - 0.726)	0.305<br>(0.238 - 0.387)	0.305<br>(0.238 - 0.387)	0.093<br>(0.039 - 0.186)	0.074<br>(0.030 - 0.144)	0.074<br>(0.031 - 0.147)
**5-14 years (rate per capita)**	0.462<br>(0.411 - 0.519)	0.390<br>(0.343 - 0.441)	0.388<br>(0.338 - 0.442)	0.845<br>(0.633 - 1.133)	0.876<br>(0.626 - 1.208)	0.888<br>(0.633 - 1.230)	0.522<br>(0.293 - 0.833)	0.499<br>(0.280 - 0.802)	0.496<br>(0.283 - 0.797)
**15 - 49 years (rate per capita)**	0.452<br>(0.427 - 0.478)	0.449<br>(0.427 - 0.475)	0.448<br>(0.424 - 0.474)	0.685<br>(0.613 - 0.761)	0.685<br>(0.613 - 0.757)	0.690<br>(0.618 - 0.762)	1.515<br>(0.865 - 2.452)	1.514<br>(0.862 - 2.420)	1.546<br>(0.885 - 2.482)
**50 - 69 years (rate per capita)**	0.545<br>(0.518 - 0.574)	0.547<br>(0.519 - 0.576)	0.545<br>(0.516 - 0.573)	0.734<br>(0.650 - 0.838)	0.734<br>(0.652 - 0.833)	0.736<br>(0.649 - 0.837)	4.384<br>(2.664 - 6.782)	4.422<br>(2.682 - 6.828)	4.313<br>(2.635 - 6.620)
**70+ years (rate per capita)**	0.553<br>(0.530 - 0.581)	0.551<br>(0.527 - 0.578)	0.549<br>(0.525 - 0.577)	0.632<br>(0.556 - 0.715)	0.630<br>(0.556 - 0.715)	0.630<br>(0.556 - 0.715)	6.389<br>(4.121 - 9.261)	6.356<br>(4.106 - 9.321)	6.321<br>(4.082 - 9.252)
**All ages (rate per capita)**	0.456<br>(0.432 - 0.479)	0.428<br>(0.408 - 0.446)	0.430<br>(0.411 - 0.449)	0.705<br>(0.625 - 0.792)	0.685<br>(0.610 - 0.775)	0.686<br>(0.616 - 0.770)	1.396<br>(0.822 - 2.173)	1.466<br>(0.862 - 2.269)	1.546<br>(0.911 - 2.397)
**Age-standardised (rate per capita)**	0.473<br>(0.455 - 0.490)	0.452<br>(0.435 - 0.468)	0.452<br>(0.435 - 0.468)	0.706<br>(0.646 - 0.766)	0.693<br>(0.635 - 0.761)	0.693<br>(0.635 - 0.761)	2.121<br>(1.271 - 3.284)	2.113<br>(1.273 - 3.275)	2.108<br>(1.265 - 3.254)
**Total number of cases (thousands)**	49,201<br>(46,669 - 51,683)	65,873<br>(62,882 - 68,726)	81,338<br>(77,691 - 84,916)	76,105<br>(67,458 - 85,514)	105,492<br>(93,981 - 119,424)	129,645<br>(116,487 - 145,569)	151<br>(89 - 235)	226<br>(133 - 349)	292<br>(172 - 453)
**Sub-Saharan Africa**									
**Under-5 years (rate per capita)**	0.347<br>(0.317 - 0.382)	0.346<br>(0.313 - 0.381)	0.352<br>(0.320 - 0.388)	0.510<br>(0.304 - 0.699)	0.491<br>(0.301 - 0.661)	0.513<br>(0.307 - 0.700)	0.082<br>(0.034 - 0.165)	0.081<br>(0.035 - 0.164)	0.083<br>(0.035 - 0.166)
**5-14 years (rate per capita)**	0.459<br>(0.437 - 0.485)	0.465<br>(0.444 - 0.489)	0.466<br>(0.443 - 0.491)	0.721<br>(0.541 - 0.963)	0.723<br>(0.540 - 0.970)	0.725<br>(0.539 - 0.969)	0.505<br>(0.288 - 0.811)	0.510<br>(0.291 - 0.817)	0.510<br>(0.290 - 0.820)
**15 - 49 years (rate per capita)**	0.491<br>(0.477 - 0.506)	0.490<br>(0.474 - 0.505)	0.492<br>(0.476 - 0.506)	0.695<br>(0.607 - 0.788)	0.693<br>(0.604 - 0.788)	0.696<br>(0.607 - 0.789)	1.789<br>(1.044 - 2.860)	1.764<br>(1.029 - 2.833)	1.778<br>(1.037 - 2.859)
**50 - 69 years (rate per capita)**	0.542<br>(0.520 - 0.558)	0.542<br>(0.522 - 0.559)	0.542<br>(0.523 - 0.559)	0.712<br>(0.592 - 0.840)	0.711<br>(0.591 - 0.837)	0.712<br>(0.592 - 0.837)	4.086<br>(2.422 - 6.590)	4.093<br>(2.411 - 6.565)	4.101<br>(2.422 - 6.584)
**70+ years (rate per capita)**	0.549<br>(0.530 - 0.566)	0.549<br>(0.528 - 0.566)	0.549<br>(0.530 - 0.566)	0.621<br>(0.512 - 0.737)	0.621<br>(0.512 - 0.733)	0.624<br>(0.516 - 0.737)	5.143<br>(3.262 - 7.774)	5.108<br>(3.223 - 7.749)	5.076<br>(3.208 - 7.708)
**All ages (rate per capita)**	0.461<br>(0.449 - 0.473)	0.464<br>(0.452 - 0.476)	0.467<br>(0.455 - 0.479)	0.669<br>(0.591 - 0.757)	0.667<br>(0.593 - 0.753)	0.673<br>(0.598 - 0.759)	1.374<br>(0.809 - 2.152)	1.382<br>(0.815 - 2.163)	1.408<br>(0.828 - 2.212)
**Age-standardised (rate per capita)**	0.489<br>(0.477 - 0.499)	0.490<br>(0.478 - 0.499)	0.491<br>(0.479 - 0.501)	0.682<br>(0.626 - 0.748)	0.680<br>(0.625 - 0.746)	0.683<br>(0.627 - 0.750)	2.139<br>(1.278 - 3.352)	2.137<br>(1.280 - 3.365)	2.134<br>(1.273 - 3.370)
**Total number of cases (thousands)**	226,694<br>(220,682 - 232,161)	339,417<br>(330,665 - 348,282)	448,498<br>(436,870 - 460,146)	328,547<br>(290,508 - 371,750)	487,919<br>(434,269 - 551,024)	646,662<br>(574,689 - 729,030)	675<br>(397 - 1,057)	1,012<br>(597 - 1,583)	1,352<br>(795 - 2,124)

	Prevalence 1990	Prevalence 2005	Prevalence 2015	Incidence 1990	Incidence 2005	Incidence 2015	YLD rate 1990	YLD rate 2005	YLD rate 2015
**Southern Sub-Saharan Africa**									
**Under-5 years (rate per capita)**	0.279 (0.261 - 0.301)	0.298 (0.279 - 0.319)	0.298 (0.279 - 0.319)	0.497 (0.284 - 0.711)	0.541 (0.304 - 0.769)	0.543 (0.306 - 0.771)	0.066 (0.028 - 0.133)	0.070 (0.030 - 0.140)	0.070 (0.030 - 0.138)
**5-14 years (rate per capita)**	0.382 (0.357 - 0.404)	0.401 (0.374 - 0.425)	0.402 (0.379 - 0.425)	0.653 (0.500 - 0.862)	0.670 (0.514 - 0.878)	0.680 (0.517 - 0.895)	0.477 (0.273 - 0.751)	0.486 (0.277 - 0.769)	0.485 (0.279 - 0.769)
**15 - 49 years (rate per capita)**	0.472 (0.461 - 0.483)	0.472 (0.460 - 0.484)	0.473 (0.463 - 0.483)	0.699 (0.632 - 0.772)	0.699 (0.631 - 0.771)	0.701 (0.634 - 0.774)	2.554 (1.610 - 3.720)	2.548 (1.617 - 3.731)	2.602 (1.649 - 3.790)
**50 - 69 years (rate per capita)**	0.491 (0.479 - 0.503)	0.489 (0.476 - 0.502)	0.489 (0.476 - 0.502)	0.688 (0.607 - 0.789)	0.689 (0.607 - 0.790)	0.692 (0.608 - 0.789)	5.806 (3.772 - 8.401)	5.674 (3.705 - 8.189)	5.655 (3.696 - 8.124)
**70+ years (rate per capita)**	0.511 (0.500 - 0.521)	0.508 (0.498 - 0.518)	0.507 (0.497 - 0.517)	0.582 (0.512 - 0.656)	0.582 (0.511 - 0.657)	0.579 (0.507 - 0.650)	7.604 (5.043 - 10.585)	7.468 (4.968 - 10.377)	7.461 (4.961 - 10.345)
**All ages (rate per capita)**	0.423 (0.414 - 0.432)	0.439 (0.430 - 0.448)	0.442 (0.433 - 0.451)	0.654 (0.586 - 0.735)	0.670 (0.606 - 0.743)	0.674 (0.612 - 0.744)	2.060 (1.309 - 2.973)	2.254 (1.442 - 3.253)	2.401 (1.534 - 3.466)
**Age-standardised (rate per capita)**	0.449 (0.441 - 0.456)	0.454 (0.445 - 0.463)	0.453 (0.446 - 0.462)	0.665 (0.612 - 0.723)	0.674 (0.620 - 0.731)	0.674 (0.620 - 0.736)	2.990 (1.926 - 4.310)	2.967 (1.916 - 4.279)	2.965 (1.914 - 4.271)
**Total number of cases (thousands)**	22,587 (22,095 - 23,081)	30,018 (29,393 - 30,684)	34,231 (33,539 - 34,923)	34,889 (31,299 - 39,242)	45,818 (41,442 - 50,830)	52,182 (47,368 - 57,635)	110 (70 - 159)	154 (99 - 223)	186 (119 - 268)
**Botswana**									
**Under-5 years (rate per capita)**	0.361 (0.293 - 0.439)	0.358 (0.285 - 0.439)	0.358 (0.283 - 0.440)	0.565 (0.329 - 0.766)	0.563 (0.328 - 0.763)	0.563 (0.328 - 0.763)	0.085 (0.035 - 0.175)	0.084 (0.035 - 0.174)	0.084 (0.035 - 0.173)
**5-14 years (rate per capita)**	0.430 (0.366 - 0.501)	0.427 (0.364 - 0.493)	0.428 (0.363 - 0.494)	0.782 (0.565 - 1.044)	0.768 (0.556 - 1.025)	0.775 (0.560 - 1.032)	0.496 (0.286 - 0.802)	0.498 (0.284 - 0.807)	0.498 (0.287 - 0.813)
**15 - 49 years (rate per capita)**	0.482 (0.431 - 0.536)	0.482 (0.426 - 0.536)	0.487 (0.435 - 0.544)	0.749 (0.612 - 0.878)	0.749 (0.611 - 0.880)	0.753 (0.620 - 0.883)	2.334 (1.460 - 3.471)	2.318 (1.456 - 3.470)	2.475 (1.549 - 3.709)
**50 - 69 years (rate per capita)**	0.525 (0.467 - 0.587)	0.525 (0.471 - 0.585)	0.525 (0.473 - 0.587)	0.747 (0.540 - 0.962)	0.745 (0.539 - 0.959)	0.747 (0.536 - 0.967)	5.547 (3.565 - 8.050)	5.478 (3.508 - 7.948)	5.480 (3.520 - 7.890)
**70+ years (rate per capita)**	0.546 (0.494 - 0.599)	0.544 (0.491 - 0.600)	0.545 (0.491 - 0.605)	0.674 (0.472 - 0.884)	0.673 (0.471 - 0.880)	0.672 (0.472 - 0.879)	7.588 (4.930 - 10.683)	7.527 (4.886 - 10.466)	7.535 (4.904 - 10.513)
**All ages (rate per capita)**	0.451 (0.418 - 0.487)	0.459 (0.423 - 0.495)	0.465 (0.430 - 0.501)	0.727 (0.632 - 0.829)	0.729 (0.637 - 0.826)	0.733 (0.646 - 0.825)	1.749 (1.098 - 2.572)	1.983 (1.252 - 2.931)	2.203 (1.393 - 3.240)
**Age-standardised (rate per capita)**	0.477 (0.449 - 0.511)	0.477 (0.448 - 0.511)	0.477 (0.447 - 0.511)	0.732 (0.649 - 0.817)	0.732 (0.649 - 0.818)	0.732 (0.649 - 0.818)	2.876 (1.826 - 4.152)	2.849 (1.814 - 4.126)	2.847 (1.811 - 4.101)
**Total number of cases (thousands)**	624 (578 - 674)	856 (789 - 925)	1,051 (972 - 1,132)	1,006 (875 - 1,147)	1,362 (1,190 - 1,543)	1,656 (1,459 - 1,863)	2 (2 - 4)	4 (2 - 5)	5 (3 - 7)
**Lesotho**									
**Under-5 years (rate per capita)**	0.358 (0.283 - 0.440)	0.359 (0.285 - 0.436)	0.357 (0.282 - 0.433)	0.563 (0.328 - 0.763)	0.564 (0.328 - 0.764)	0.559 (0.326 - 0.758)	0.084 (0.036 - 0.176)	0.085 (0.035 - 0.175)	0.084 (0.036 - 0.171)
**5-14 years (rate per capita)**	0.428 (0.361 - 0.494)	0.429 (0.363 - 0.495)	0.427 (0.358 - 0.493)	0.781 (0.564 - 1.041)	0.773 (0.559 - 1.030)	0.770 (0.558 - 1.027)	0.496 (0.281 - 0.794)	0.497 (0.284 - 0.812)	0.497 (0.284 - 0.790)
**15 - 49 years (rate per capita)**	0.482 (0.430 - 0.536)	0.478 (0.425 - 0.533)	0.480 (0.428 - 0.536)	0.747 (0.609 - 0.875)	0.746 (0.606 - 0.880)	0.750 (0.611 - 0.881)	2.282 (1.416 - 3.407)	2.238 (1.401 - 3.331)	2.274 (1.421 - 3.389)
**50 - 69 years (rate per capita)**	0.527 (0.475 - 0.582)	0.524 (0.471 - 0.587)	0.526 (0.473 - 0.585)	0.747 (0.540 - 0.964)	0.746 (0.543 - 0.959)	0.748 (0.541 - 0.965)	5.658 (3.632 - 8.140)	5.549 (3.553 - 8.021)	5.638 (3.612 - 8.084)
**70+ years (rate per capita)**	0.545 (0.489 - 0.601)	0.540 (0.488 - 0.598)	0.537 (0.488 - 0.591)	0.673 (0.470 - 0.880)	0.669 (0.476 - 0.867)	0.664 (0.478 - 0.858)	7.621 (4.923 - 10.876)	7.464 (4.825 - 10.586)	7.379 (4.781 - 10.444)
**All ages (rate per capita)**	0.453 (0.419 - 0.487)	0.455 (0.422 - 0.490)	0.457 (0.423 - 0.494)	0.725 (0.632 - 0.827)	0.727 (0.634 - 0.827)	0.728 (0.636 - 0.824)	1.886 (1.183 - 2.757)	1.944 (1.220 - 2.846)	1.990 (1.253 - 2.910)
**Age-standardised (rate per capita)**	0.478 (0.449 - 0.511)	0.477 (0.448 - 0.510)	0.477 (0.449 - 0.510)	0.732 (0.650 - 0.816)	0.732 (0.649 - 0.817)	0.732 (0.650 - 0.818)	2.890 (1.832 - 4.189)	2.853 (1.810 - 4.138)	2.837 (1.810 - 4.101)
**Total number of cases (thousands)**	726 (671 - 781)	877 (813 - 945)	973 (900 - 1,052)	1,162 (1,014 - 1,326)	1,400 (1,221 - 1,594)	1,549 (1,354 - 1,755)	3 (2 - 4)	4 (2 - 5)	4 (3 - 6)
**Namibia**									
**Under-5 years (rate per capita)**	0.356 (0.280 - 0.433)	0.357 (0.282 - 0.431)	0.357 (0.281 - 0.440)	0.556 (0.324 - 0.755)	0.563 (0.328 - 0.763)	0.560 (0.326 - 0.759)	0.083 (0.035 - 0.172)	0.084 (0.035 - 0.175)	0.084 (0.035 - 0.171)
**5-14 years (rate per capita)**	0.442 (0.392 - 0.502)	0.435 (0.387 - 0.493)	0.438 (0.388 - 0.496)	0.707 (0.527 - 0.930)	0.697 (0.516 - 0.926)	0.701 (0.518 - 0.931)	0.506 (0.287 - 0.803)	0.503 (0.284 - 0.805)	0.502 (0.284 - 0.811)
**15 - 49 years (rate per capita)**	0.519 (0.496 - 0.547)	0.520 (0.497 - 0.546)	0.520 (0.497 - 0.544)	0.683 (0.616 - 0.758)	0.686 (0.621 - 0.762)	0.687 (0.622 - 0.761)	2.996 (1.915 - 4.318)	3.113 (2.007 - 4.471)	3.117 (1.994 - 4.491)
**50 - 69 years (rate per capita)**	0.538 (0.512 - 0.565)	0.535 (0.509 - 0.559)	0.534 (0.509 - 0.561)	0.709 (0.624 - 0.808)	0.710 (0.623 - 0.808)	0.711 (0.625 - 0.810)	7.033 (4.571 - 10.031)	6.910 (4.513 - 9.829)	6.845 (4.486 - 9.714)
**70+ years (rate per capita)**	0.579 (0.555 - 0.607)	0.576 (0.555 - 0.601)	0.575 (0.553 - 0.600)	0.613 (0.538 - 0.691)	0.610 (0.536 - 0.685)	0.610 (0.536 - 0.684)	9.681 (6.475 - 13.541)	9.564 (6.403 - 13.266)	9.558 (6.405 - 13.357)
**All ages (rate per capita)**	0.474 (0.450 - 0.497)	0.478 (0.457 - 0.499)	0.481 (0.461 - 0.503)	0.669 (0.595 - 0.754)	0.672 (0.604 - 0.755)	0.673 (0.607 - 0.751)	2.294 (1.473 - 3.302)	2.449 (1.576 - 3.536)	2.585 (1.671 - 3.714)
**Age-standardised (rate per capita)**	0.503 (0.486 - 0.521)	0.501 (0.485 - 0.517)	0.501 (0.485 - 0.518)	0.679 (0.626 - 0.736)	0.679 (0.625 - 0.738)	0.679 (0.625 - 0.738)	3.647 (2.371 - 5.211)	3.649 (2.372 - 5.211)	3.625 (2.362 - 5.191)
**Total number of cases (thousands)**	671 (638 - 704)	970 (927 - 1,014)	1,181 (1,132 - 1,233)	948 (843 - 1,068)	1,365 (1,225 - 1,533)	1,652 (1,489 - 1,842)	3 (2 - 5)	5 (3 - 7)	6 (4 - 9)
**South Africa**									
**Under-5 years (rate per capita)**	0.287 (0.266 - 0.308)	0.302 (0.283 - 0.324)	0.304 (0.283 - 0.326)	0.546 (0.303 - 0.777)	0.545 (0.310 - 0.770)	0.549 (0.314 - 0.769)	0.068 (0.029 - 0.134)	0.071 (0.031 - 0.142)	0.072 (0.031 - 0.142)
**5-14 years (rate per capita)**	0.391 (0.366 - 0.415)	0.401 (0.375 - 0.425)	0.403 (0.379 - 0.426)	0.659 (0.504 - 0.860)	0.671 (0.518 - 0.873)	0.683 (0.523 - 0.891)	0.481 (0.277 - 0.756)	0.488 (0.278 - 0.770)	0.488 (0.280 - 0.768)
**15 - 49 years (rate per capita)**	0.473 (0.463 - 0.484)	0.474 (0.463 - 0.486)	0.474 (0.462 - 0.484)	0.705 (0.638 - 0.778)	0.706 (0.640 - 0.777)	0.707 (0.641 - 0.777)	2.659 (1.684 - 3.885)	2.666 (1.690 - 3.879)	2.706 (1.720 - 3.943)
**50 - 69 years (rate per capita)**	0.494 (0.482 - 0.505)	0.490 (0.477 - 0.503)	0.489 (0.476 - 0.501)	0.689 (0.609 - 0.787)	0.690 (0.610 - 0.785)	0.692 (0.611 - 0.786)	5.985 (3.914 - 8.660)	5.775 (3.802 - 8.323)	5.731 (3.756 - 8.240)
**70+ years (rate per capita)**	0.512 (0.501 - 0.522)	0.509 (0.499 - 0.520)	0.508 (0.498 - 0.519)	0.579 (0.513 - 0.650)	0.579 (0.510 - 0.651)	0.577 (0.507 - 0.645)	7.738 (5.169 - 10.747)	7.586 (5.066 - 10.524)	7.567 (5.044 - 10.484)
**All ages (rate per capita)**	0.431 (0.422 - 0.439)	0.444 (0.434 - 0.453)	0.448 (0.440 - 0.457)	0.667 (0.598 - 0.747)	0.676 (0.614 - 0.742)	0.680 (0.624 - 0.744)	2.214 (1.406 - 3.185)	2.452 (1.570 - 3.544)	2.640 (1.691 - 3.812)
**Age-standardised (rate per capita)**	0.452 (0.444 - 0.460)	0.454 (0.446 - 0.463)	0.454 (0.447 - 0.462)	0.674 (0.620 - 0.736)	0.677 (0.622 - 0.733)	0.677 (0.624 - 0.736)	3.064 (1.971 - 4.418)	3.024 (1.954 - 4.350)	3.021 (1.952 - 4.354)
**Total number of cases (thousands)**	16,189 (15,854 - 16,503)	21,507 (21,026 - 21,954)	24,059 (23,639 - 24,526)	25,080 (22,490 - 28,075)	32,750 (29,788 - 35,989)	36,547 (33,543 - 39,945)	83 (53 - 120)	119 (76 - 172)	142 (91 - 205)
**Swaziland**									
**Under-5 years (rate per capita)**	0.372 (0.309 - 0.435)	0.372 (0.310 - 0.431)	0.375 (0.312 - 0.438)	0.566 (0.367 - 0.753)	0.565 (0.367 - 0.752)	0.568 (0.369 - 0.756)	0.088 (0.037 - 0.180)	0.088 (0.037 - 0.177)	0.089 (0.038 - 0.177)
**5-14 years (rate per capita)**	0.574 (0.518 - 0.631)	0.572 (0.516 - 0.631)	0.573 (0.516 - 0.631)	0.774 (0.600 - 0.990)	0.743 (0.578 - 0.947)	0.757 (0.589 - 0.969)	0.593 (0.332 - 0.988)	0.597 (0.330 - 0.985)	0.594 (0.330 - 0.989)
**15 - 49 years (rate per capita)**	0.603 (0.582 - 0.625)	0.601 (0.579 - 0.626)	0.600 (0.578 - 0.624)	0.684 (0.624 - 0.751)	0.683 (0.622 - 0.752)	0.688 (0.625 - 0.759)	2.328 (1.460 - 3.473)	2.238 (1.396 - 3.312)	2.292 (1.441 - 3.393)
**50 - 69 years (rate per capita)**	0.590 (0.560 - 0.620)	0.591 (0.560 - 0.620)	0.593 (0.562 - 0.621)	0.763 (0.673 - 0.856)	0.762 (0.673 - 0.853)	0.763 (0.674 - 0.853)	5.148 (3.303 - 7.504)	5.106 (3.287 - 7.410)	5.129 (3.332 - 7.403)
**70+ years (rate per capita)**	0.610 (0.584 - 0.639)	0.607 (0.581 - 0.633)	0.607 (0.582 - 0.634)	0.671 (0.592 - 0.755)	0.669 (0.593 - 0.750)	0.669 (0.592 - 0.750)	6.934 (4.582 - 9.798)	6.782 (4.483 - 9.510)	6.819 (4.517 - 9.582)
**All ages (rate per capita)**	0.549 (0.526 - 0.570)	0.560 (0.538 - 0.582)	0.563 (0.543 - 0.581)	0.693 (0.619 - 0.777)	0.689 (0.625 - 0.761)	0.694 (0.634 - 0.764)	1.676 (1.050 - 2.498)	1.791 (1.116 - 2.649)	1.920 (1.216 - 2.851)
**Age-standardised (rate per capita)**	0.577 (0.562 - 0.594)	0.577 (0.562 - 0.594)	0.577 (0.561 - 0.594)	0.704 (0.656 - 0.757)	0.704 (0.656 - 0.757)	0.704 (0.656 - 0.758)	2.785 (1.765 - 4.044)	2.745 (1.739 - 3.947)	2.732 (1.744 - 3.958)
**Total number of cases (thousands)**	474 (454 - 493)	619 (595 - 644)	725 (700 - 749)	599 (535 - 672)	762 (691 - 842)	895 (816 - 985)	1 (1 - 2)	2 (1 - 3)	2 (2 - 4)
**Zimbabwe**									
**Under-5 years (rate per capita)**	0.217 (0.176 - 0.266)	0.258 (0.214 - 0.296)	0.259 (0.217 - 0.295)	0.326 (0.190 - 0.622)	0.519 (0.266 - 0.773)	0.522 (0.268 - 0.778)	0.050 (0.021 - 0.104)	0.060 (0.025 - 0.119)	0.060 (0.026 - 0.120)
**5-14 years (rate per capita)**	0.319 (0.273 - 0.362)	0.374 (0.324 - 0.429)	0.378 (0.331 - 0.430)	0.584 (0.411 - 0.806)	0.630 (0.462 - 0.859)	0.645 (0.472 - 0.880)	0.446 (0.258 - 0.698)	0.465 (0.268 - 0.739)	0.465 (0.267 - 0.742)
**15 - 49 years (rate per capita)**	0.447 (0.421 - 0.476)	0.445 (0.419 - 0.474)	0.448 (0.423 - 0.476)	0.664 (0.593 - 0.742)	0.660 (0.589 - 0.742)	0.666 (0.595 - 0.746)	2.162 (1.365 - 3.188)	2.082 (1.316 - 3.066)	2.209 (1.405 - 3.270)
**50 - 69 years (rate per capita)**	0.452 (0.425 - 0.480)	0.453 (0.427 - 0.479)	0.454 (0.428 - 0.483)	0.654 (0.572 - 0.748)	0.653 (0.571 - 0.746)	0.655 (0.571 - 0.753)	4.929 (3.193 - 7.157)	4.913 (3.175 - 7.071)	4.909 (3.188 - 7.118)
**70+ years (rate per capita)**	0.478 (0.454 - 0.505)	0.473 (0.449 - 0.500)	0.472 (0.448 - 0.497)	0.560 (0.491 - 0.636)	0.556 (0.486 - 0.629)	0.554 (0.484 - 0.627)	6.600 (4.346 - 9.317)	6.478 (4.279 - 9.116)	6.494 (4.296 - 9.121)
**All ages (rate per capita)**	0.371 (0.350 - 0.393)	0.398 (0.376 - 0.420)	0.401 (0.379 - 0.422)	0.580 (0.510 - 0.677)	0.628 (0.547 - 0.723)	0.635 (0.555 - 0.729)	1.575 (0.995 - 2.314)	1.613 (1.017 - 2.339)	1.670 (1.058 - 2.445)
**Age-standardised (rate per capita)**	0.411 (0.394 - 0.429)	0.425 (0.408 - 0.444)	0.425 (0.409 - 0.442)	0.613 (0.558 - 0.674)	0.640 (0.583 - 0.703)	0.640 (0.582 - 0.703)	2.600 (1.662 - 3.771)	2.588 (1.662 - 3.746)	2.597 (1.668 - 3.769)
**Total number of cases (thousands)**	3,902 (3,678 - 4,126)	5,188 (4,892 - 5,474)	6,243 (5,909 - 6,572)	6,095 (5,361 - 7,112)	8,179 (7,124 - 9,415)	9,882 (8,643 - 11,358)	17 (10 - 24)	21 (13 - 30)	26 (16 - 38)
**Western Sub-Saharan Africa**									
**Under-5 years (rate per capita)**	0.350 (0.302 - 0.402)	0.355 (0.305 - 0.412)	0.360 (0.309 - 0.416)	0.496 (0.287 - 0.690)	0.498 (0.288 - 0.693)	0.503 (0.292 - 0.700)	0.082 (0.035 - 0.175)	0.083 (0.035 - 0.174)	0.084 (0.036 - 0.178)
**5-14 years (rate per capita)**	0.470 (0.433 - 0.507)	0.473 (0.438 - 0.509)	0.475 (0.439 - 0.511)	0.715 (0.519 - 0.991)	0.707 (0.517 - 0.968)	0.707 (0.519 - 0.966)	0.505 (0.286 - 0.811)	0.506 (0.287 - 0.810)	0.508 (0.288 - 0.809)
**15 - 49 years (rate per capita)**	0.493 (0.470 - 0.520)	0.493 (0.468 - 0.518)	0.494 (0.470 - 0.519)	0.659 (0.560 - 0.769)	0.659 (0.559 - 0.770)	0.660 (0.560 - 0.771)	1.713 (0.970 - 2.800)	1.715 (0.970 - 2.825)	1.741 (0.982 - 2.868)
**50 - 69 years (rate per capita)**	0.533 (0.509 - 0.563)	0.536 (0.511 - 0.564)	0.536 (0.511 - 0.563)	0.664 (0.529 - 0.811)	0.663 (0.530 - 0.811)	0.663 (0.530 - 0.811)	3.846 (2.211 - 6.207)	3.884 (2.222 - 6.275)	3.903 (2.232 - 6.324)
**70+ years (rate per capita)**	0.540 (0.513 - 0.565)	0.542 (0.516 - 0.568)	0.539 (0.514 - 0.565)	0.562 (0.440 - 0.697)	0.558 (0.440 - 0.690)	0.558 (0.440 - 0.690)	4.647 (2.872 - 7.192)	4.685 (2.896 - 7.260)	4.701 (2.923 - 7.271)
**All ages (rate per capita)**	0.465 (0.447 - 0.484)	0.468 (0.449 - 0.486)	0.470 (0.451 - 0.488)	0.644 (0.560 - 0.741)	0.642 (0.563 - 0.736)	0.645 (0.565 - 0.739)	1.318 (0.759 - 2.118)	1.329 (0.764 - 2.133)	1.346 (0.774 - 2.164)
**Age-standardised (rate per capita)**	0.488 (0.473 - 0.504)	0.490 (0.475 - 0.505)	0.490 (0.475 - 0.505)	0.649 (0.585 - 0.720)	0.648 (0.584 - 0.718)	0.648 (0.584 - 0.718)	2.012 (1.177 - 3.223)	2.033 (1.188 - 3.295)	2.045 (1.189 - 3.310)
**Total number of cases (thousands)**	92,477 (88,911 - 96,092)	138,901 (133,331 - 144,223)	183,817 (176,373 - 190,813)	127,889 (111,181 - 147,174)	190,765 (167,105 - 218,707)	252,083 (220,827 - 289,056)	262 (151 - 421)	395 (227 - 634)	527 (303 - 846)
**Benin**									

	Prevalence 1990	Prevalence 2005	Prevalence 2015	Incidence 1990	Incidence 2005	Incidence 2015	YLD rate 1990	YLD rate 2005	YLD rate 2015
Under-5 years (rate per capita	0.353 (0.267 - 0.442)	0.359 (0.275 - 0.451)	0.360 (0.277 - 0.455)	0.497 (0.288 - 0.691)	0.504 (0.292 - 0.701)	0.508 (0.294 - 0.707)	0.082 (0.033 - 0.174)	0.084 (0.034 - 0.177)	0.085 (0.035 - 0.181)
5-14 years (rate per capita	0.467 (0.404 - 0.538)	0.466 (0.404 - 0.532)	0.464 (0.401 - 0.530)	0.728 (0.518 - 1.011)	0.725 (0.517 - 1.005)	0.721 (0.517 - 0.996)	0.505 (0.288 - 0.805)	0.506 (0.286 - 0.812)	0.507 (0.286 - 0.809)
15 - 49 years (rate per capita	0.512 (0.469 - 0.560)	0.511 (0.467 - 0.555)	0.512 (0.467 - 0.557)	0.673 (0.561 - 0.793)	0.675 (0.563 - 0.795)	0.675 (0.564 - 0.794)	1.912 (1.073 - 3.162)	1.930 (1.079 - 3.215)	1.957 (1.084 - 3.248)
50 - 69 years (rate per capita	0.589 (0.543 - 0.648)	0.590 (0.540 - 0.647)	0.590 (0.543 - 0.646)	0.678 (0.532 - 0.841)	0.678 (0.532 - 0.841)	0.678 (0.531 - 0.846)	4.653 (2.646 - 7.733)	4.672 (2.665 - 7.806)	4.677 (2.634 - 7.786)
70+ years (rate per capita	0.588 (0.542 - 0.640)	0.589 (0.539 - 0.638)	0.589 (0.540 - 0.637)	0.571 (0.441 - 0.718)	0.570 (0.441 - 0.715)	0.572 (0.441 - 0.720)	5.472 (3.294 - 8.479)	5.491 (3.311 - 8.518)	5.532 (3.343 - 8.644)
All ages (rate per capita	0.478 (0.447 - 0.511)	0.479 (0.449 - 0.513)	0.483 (0.452 - 0.517)	0.654 (0.567 - 0.755)	0.657 (0.571 - 0.757)	0.659 (0.575 - 0.757)	1.478 (0.851 - 2.394)	1.491 (0.854 - 2.457)	1.557 (0.892 - 2.567)
Age-standardised (rate per capita	0.512 (0.487 - 0.541)	0.512 (0.485 - 0.544)	0.512 (0.485 - 0.541)	0.662 (0.594 - 0.739)	0.662 (0.593 - 0.739)	0.662 (0.593 - 0.739)	2.341 (1.355 - 3.873)	2.352 (1.360 - 3.912)	2.365 (1.359 - 3.893)
Total number of cases (thousands)	2,390 (2,237 - 2,560)	3,926 (3,673 - 4,205)	5,267 (4,933 - 5,641)	3,273 (2,838 - 3,779)	5,383 (4,679 - 6,199)	7,194 (6,274 - 8,261)	7 (4 - 12)	12 (7 - 20)	17 (10 - 28)
Burkina Faso									
Under-5 years (rate per capita	0.275 (0.219 - 0.334)	0.278 (0.221 - 0.337)	0.283 (0.229 - 0.334)	0.452 (0.221 - 0.686)	0.452 (0.214 - 0.687)	0.457 (0.216 - 0.695)	0.064 (0.027 - 0.133)	0.065 (0.027 - 0.127)	0.066 (0.027 - 0.135)
5-14 years (rate per capita	0.485 (0.434 - 0.541)	0.523 (0.482 - 0.559)	0.522 (0.483 - 0.561)	0.612 (0.455 - 0.828)	0.494 (0.374 - 0.627)	0.493 (0.373 - 0.627)	0.507 (0.286 - 0.808)	0.513 (0.291 - 0.821)	0.515 (0.292 - 0.834)
15 - 49 years (rate per capita	0.507 (0.482 - 0.535)	0.507 (0.483 - 0.534)	0.508 (0.484 - 0.536)	0.620 (0.551 - 0.690)	0.621 (0.557 - 0.691)	0.622 (0.559 - 0.692)	1.810 (0.989 - 3.034)	1.804 (0.990 - 3.031)	1.849 (1.011 - 3.116)
50 - 69 years (rate per capita	0.579 (0.535 - 0.624)	0.577 (0.532 - 0.627)	0.577 (0.532 - 0.625)	0.643 (0.566 - 0.728)	0.644 (0.564 - 0.732)	0.644 (0.562 - 0.733)	4.293 (2.411 - 7.338)	4.279 (2.405 - 7.331)	4.300 (2.421 - 7.379)
70+ years (rate per capita	0.584 (0.542 - 0.627)	0.578 (0.536 - 0.621)	0.583 (0.539 - 0.628)	0.546 (0.479 - 0.621)	0.544 (0.477 - 0.615)	0.545 (0.477 - 0.617)	4.913 (2.904 - 7.994)	4.902 (2.907 - 7.881)	4.963 (2.962 - 8.079)
All ages (rate per capita	0.464 (0.439 - 0.485)	0.475 (0.451 - 0.495)	0.479 (0.457 - 0.498)	0.587 (0.514 - 0.680)	0.555 (0.483 - 0.631)	0.558 (0.488 - 0.632)	1.365 (0.764 - 2.278)	1.332 (0.739 - 2.215)	1.375 (0.761 - 2.289)
Age-standardised (rate per capita	0.503 (0.483 - 0.524)	0.510 (0.488 - 0.532)	0.510 (0.490 - 0.531)	0.605 (0.556 - 0.662)	0.586 (0.537 - 0.636)	0.586 (0.536 - 0.636)	2.190 (1.234 - 3.709)	2.192 (1.236 - 3.707)	2.214 (1.256 - 3.757)
Total number of cases (thousands)	4,089 (3,872 - 4,276)	6,378 (6,065 - 6,658)	8,667 (8,263 - 9,019)	5,170 (4,526 - 5,991)	7,453 (6,488 - 8,477)	10,093 (8,827 - 11,431)	12 (7 - 20)	18 (10 - 30)	25 (14 - 41)
Cameroon									
Under-5 years (rate per capita	0.358 (0.276 - 0.446)	0.359 (0.276 - 0.450)	0.363 (0.274 - 0.452)	0.503 (0.291 - 0.699)	0.504 (0.291 - 0.700)	0.509 (0.294 - 0.707)	0.084 (0.035 - 0.182)	0.084 (0.036 - 0.186)	0.085 (0.035 - 0.182)
5-14 years (rate per capita	0.468 (0.405 - 0.525)	0.466 (0.405 - 0.539)	0.466 (0.402 - 0.529)	0.732 (0.520 - 1.017)	0.721 (0.517 - 0.997)	0.722 (0.517 - 0.999)	0.504 (0.285 - 0.808)	0.506 (0.285 - 0.818)	0.507 (0.285 - 0.816)
15 - 49 years (rate per capita	0.511 (0.468 - 0.553)	0.507 (0.465 - 0.557)	0.509 (0.463 - 0.557)	0.674 (0.561 - 0.792)	0.673 (0.558 - 0.798)	0.675 (0.563 - 0.799)	1.923 (1.078 - 3.187)	1.871 (1.047 - 3.128)	1.920 (1.071 - 3.189)
50 - 69 years (rate per capita	0.591 (0.544 - 0.647)	0.590 (0.545 - 0.646)	0.590 (0.543 - 0.644)	0.677 (0.529 - 0.840)	0.677 (0.529 - 0.839)	0.677 (0.530 - 0.841)	4.649 (2.642 - 7.783)	4.651 (2.641 - 7.771)	4.670 (2.653 - 7.796)
70+ years (rate per capita	0.585 (0.538 - 0.633)	0.583 (0.533 - 0.630)	0.581 (0.533 - 0.627)	0.566 (0.442 - 0.712)	0.565 (0.443 - 0.711)	0.564 (0.442 - 0.708)	5.415 (3.281 - 8.447)	5.405 (3.256 - 8.398)	5.424 (3.267 - 8.442)
All ages (rate per capita	0.479 (0.447 - 0.512)	0.479 (0.448 - 0.515)	0.482 (0.449 - 0.514)	0.655 (0.568 - 0.757)	0.655 (0.570 - 0.757)	0.659 (0.574 - 0.759)	1.494 (0.856 - 2.436)	1.477 (0.848 - 2.442)	1.523 (0.870 - 2.504)
Age-standardised (rate per capita	0.512 (0.487 - 0.541)	0.511 (0.487 - 0.540)	0.512 (0.486 - 0.540)	0.662 (0.593 - 0.739)	0.662 (0.593 - 0.739)	0.662 (0.593 - 0.739)	2.340 (1.347 - 3.877)	2.339 (1.343 - 3.917)	2.355 (1.365 - 3.944)
Total number of cases (thousands)	5,788 (5,412 - 6,195)	8,687 (8,137 - 9,355)	11,269 (10,518 - 12,026)	7,928 (6,871 - 9,156)	11,889 (10,339 - 13,736)	15,413 (13,423 - 17,753)	18 (10 - 29)	27 (15 - 44)	36 (20 - 59)
Cape Verde									
Under-5 years (rate per capita	0.364 (0.278 - 0.458)	0.370 (0.282 - 0.466)	0.365 (0.279 - 0.462)	0.512 (0.296 - 0.712)	0.519 (0.301 - 0.722)	0.515 (0.298 - 0.716)	0.085 (0.036 - 0.181)	0.087 (0.036 - 0.185)	0.086 (0.036 - 0.183)
5-14 years (rate per capita	0.467 (0.405 - 0.539)	0.459 (0.398 - 0.521)	0.461 (0.398 - 0.524)	0.734 (0.522 - 1.022)	0.701 (0.504 - 0.966)	0.712 (0.512 - 0.982)	0.506 (0.283 - 0.810)	0.512 (0.290 - 0.830)	0.510 (0.289 - 0.816)
15 - 49 years (rate per capita	0.504 (0.458 - 0.549)	0.511 (0.467 - 0.554)	0.511 (0.467 - 0.554)	0.670 (0.556 - 0.799)	0.672 (0.561 - 0.791)	0.679 (0.567 - 0.801)	1.790 (1.000 - 2.965)	1.905 (1.064 - 3.157)	2.010 (1.102 - 3.363)
50 - 69 years (rate per capita	0.590 (0.543 - 0.646)	0.590 (0.544 - 0.647)	0.587 (0.538 - 0.645)	0.680 (0.531 - 0.850)	0.669 (0.530 - 0.822)	0.682 (0.533 - 0.853)	4.727 (2.700 - 7.889)	4.769 (2.730 - 7.931)	4.617 (2.615 - 7.829)
70+ years (rate per capita	0.579 (0.536 - 0.625)	0.588 (0.537 - 0.638)	0.569 (0.523 - 0.612)	0.561 (0.444 - 0.701)	0.569 (0.442 - 0.718)	0.554 (0.443 - 0.680)	5.467 (3.295 - 8.506)	5.561 (3.351 - 8.646)	5.381 (3.236 - 8.347)
All ages (rate per capita	0.478 (0.448 - 0.515)	0.489 (0.460 - 0.523)	0.496 (0.468 - 0.525)	0.656 (0.566 - 0.761)	0.657 (0.577 - 0.750)	0.664 (0.588 - 0.750)	1.502 (0.862 - 2.430)	1.680 (0.957 - 2.744)	1.913 (1.090 - 3.139)
Age-standardised (rate per capita	0.512 (0.485 - 0.541)	0.511 (0.487 - 0.541)	0.512 (0.488 - 0.539)	0.662 (0.593 - 0.739)	0.663 (0.594 - 0.739)	0.663 (0.593 - 0.739)	2.373 (1.380 - 3.974)	2.371 (1.368 - 4.000)	2.376 (1.375 - 3.960)
Total number of cases (thousands)	164 (154 - 176)	233 (219 - 248)	258 (243 - 273)	225 (194 - 261)	312 (275 - 357)	346 (306 - 390)	1 (0 - 1)	1 (0 - 1)	1 (1 - 2)
Chad									
Under-5 years (rate per capita	0.349 (0.269 - 0.440)	0.353 (0.268 - 0.446)	0.357 (0.271 - 0.450)	0.494 (0.286 - 0.687)	0.498 (0.288 - 0.692)	0.503 (0.291 - 0.699)	0.082 (0.034 - 0.175)	0.083 (0.035 - 0.175)	0.084 (0.035 - 0.180)
5-14 years (rate per capita	0.466 (0.401 - 0.534)	0.467 (0.405 - 0.533)	0.467 (0.401 - 0.531)	0.731 (0.520 - 1.016)	0.731 (0.520 - 1.015)	0.726 (0.517 - 1.007)	0.505 (0.282 - 0.802)	0.504 (0.285 - 0.805)	0.506 (0.284 - 0.810)
15 - 49 years (rate per capita	0.512 (0.469 - 0.556)	0.512 (0.469 - 0.556)	0.510 (0.465 - 0.556)	0.674 (0.562 - 0.793)	0.672 (0.559 - 0.796)	0.672 (0.559 - 0.796)	2.053 (1.158 - 3.332)	2.004 (1.130 - 3.262)	1.987 (1.134 - 3.244)
50 - 69 years (rate per capita	0.593 (0.547 - 0.649)	0.592 (0.543 - 0.646)	0.593 (0.545 - 0.648)	0.678 (0.531 - 0.841)	0.677 (0.530 - 0.842)	0.679 (0.532 - 0.844)	4.893 (2.847 - 8.174)	4.867 (2.830 - 8.034)	4.873 (2.822 - 8.118)
70+ years (rate per capita	0.589 (0.540 - 0.637)	0.587 (0.540 - 0.634)	0.586 (0.540 - 0.633)	0.568 (0.442 - 0.714)	0.567 (0.442 - 0.714)	0.566 (0.443 - 0.714)	5.760 (3.580 - 8.939)	5.744 (3.566 - 8.895)	5.731 (3.578 - 8.902)
All ages (rate per capita	0.475 (0.443 - 0.509)	0.474 (0.444 - 0.510)	0.475 (0.442 - 0.508)	0.653 (0.565 - 0.755)	0.653 (0.563 - 0.760)	0.655 (0.566 - 0.760)	1.535 (0.884 - 2.454)	1.443 (0.823 - 2.314)	1.444 (0.821 - 2.310)
Age-standardised (rate per capita	0.513 (0.487 - 0.543)	0.514 (0.490 - 0.543)	0.513 (0.486 - 0.543)	0.662 (0.593 - 0.739)	0.662 (0.593 - 0.739)	0.662 (0.593 - 0.739)	2.476 (1.457 - 4.045)	2.479 (1.448 - 4.061)	2.479 (1.452 - 4.075)
Total number of cases (thousands)	2,830 (2,638 - 3,031)	4,766 (4,464 - 5,128)	6,679 (6,217 - 7,150)	3,889 (3,368 - 4,499)	6,567 (5,662 - 7,641)	9,209 (7,955 - 10,691)	9 (5 - 15)	15 (8 - 23)	20 (12 - 32)
Cote d'Ivoire									
Under-5 years (rate per capita	0.355 (0.271 - 0.449)	0.362 (0.269 - 0.454)	0.360 (0.269 - 0.450)	0.501 (0.290 - 0.697)	0.506 (0.293 - 0.704)	0.506 (0.293 - 0.704)	0.083 (0.034 - 0.179)	0.084 (0.035 - 0.178)	0.084 (0.034 - 0.178)
5-14 years (rate per capita	0.466 (0.402 - 0.530)	0.467 (0.407 - 0.539)	0.465 (0.402 - 0.531)	0.728 (0.518 - 1.010)	0.728 (0.518 - 1.010)	0.721 (0.517 - 0.995)	0.504 (0.287 - 0.805)	0.504 (0.286 - 0.817)	0.506 (0.284 - 0.811)
15 - 49 years (rate per capita	0.515 (0.474 - 0.562)	0.515 (0.474 - 0.558)	0.513 (0.470 - 0.558)	0.676 (0.567 - 0.793)	0.675 (0.565 - 0.795)	0.674 (0.562 - 0.794)	2.214 (1.280 - 3.576)	2.196 (1.282 - 3.511)	2.174 (1.270 - 3.487)
50 - 69 years (rate per capita	0.593 (0.546 - 0.647)	0.596 (0.548 - 0.648)	0.595 (0.551 - 0.647)	0.678 (0.529 - 0.848)	0.677 (0.529 - 0.844)	0.677 (0.530 - 0.844)	5.150 (3.015 - 8.394)	5.223 (3.061 - 8.486)	5.232 (3.084 - 8.534)
70+ years (rate per capita	0.594 (0.544 - 0.642)	0.598 (0.553 - 0.644)	0.596 (0.549 - 0.645)	0.567 (0.440 - 0.717)	0.569 (0.438 - 0.717)	0.568 (0.437 - 0.718)	6.203 (3.926 - 9.344)	6.332 (4.015 - 9.601)	6.358 (4.015 - 9.659)
All ages (rate per capita	0.482 (0.452 - 0.515)	0.484 (0.455 - 0.518)	0.484 (0.455 - 0.519)	0.658 (0.573 - 0.757)	0.660 (0.573 - 0.760)	0.658 (0.574 - 0.758)	1.690 (0.992 - 2.721)	1.704 (0.986 - 2.734)	1.727 (1.012 - 2.771)
Age-standardised (rate per capita	0.514 (0.489 - 0.543)	0.516 (0.491 - 0.542)	0.515 (0.489 - 0.543)	0.662 (0.592 - 0.739)	0.662 (0.592 - 0.738)	0.662 (0.593 - 0.739)	2.616 (1.563 - 4.216)	2.638 (1.569 - 4.280)	2.642 (1.581 - 4.254)
Total number of cases (thousands)	5,855 (5,497 - 6,265)	8,774 (8,248 - 9,385)	10,987 (10,323 - 11,766)	7,998 (6,963 - 9,208)	11,949 (10,384 - 13,764)	14,928 (13,023 - 17,197)	21 (12 - 33)	31 (18 - 50)	39 (23 - 63)
The Gambia									
Under-5 years (rate per capita	0.356 (0.269 - 0.449)	0.357 (0.276 - 0.452)	0.361 (0.265 - 0.454)	0.501 (0.290 - 0.697)	0.505 (0.293 - 0.703)	0.508 (0.294 - 0.706)	0.083 (0.034 - 0.178)	0.084 (0.035 - 0.179)	0.085 (0.036 - 0.184)
5-14 years (rate per capita	0.467 (0.403 - 0.533)	0.469 (0.404 - 0.536)	0.467 (0.408 - 0.532)	0.733 (0.521 - 1.019)	0.731 (0.520 - 1.015)	0.728 (0.518 - 1.010)	0.506 (0.286 - 0.811)	0.506 (0.289 - 0.814)	0.506 (0.285 - 0.813)
15 - 49 years (rate per capita	0.532 (0.486 - 0.575)	0.534 (0.491 - 0.576)	0.535 (0.490 - 0.580)	0.678 (0.564 - 0.807)	0.677 (0.564 - 0.802)	0.678 (0.565 - 0.801)	2.174 (1.200 - 3.696)	2.187 (1.215 - 3.724)	2.211 (1.238 - 3.783)
50 - 69 years (rate per capita	0.645 (0.611 - 0.682)	0.644 (0.611 - 0.685)	0.642 (0.605 - 0.683)	0.674 (0.527 - 0.841)	0.675 (0.528 - 0.839)	0.679 (0.532 - 0.847)	5.317 (3.010 - 8.869)	5.280 (2.975 - 8.858)	5.247 (2.958 - 8.790)
70+ years (rate per capita	0.633 (0.594 - 0.671)	0.627 (0.590 - 0.663)	0.629 (0.590 - 0.665)	0.568 (0.436 - 0.720)	0.564 (0.438 - 0.714)	0.565 (0.436 - 0.714)	6.062 (3.572 - 9.464)	6.012 (3.566 - 9.399)	6.016 (3.531 - 9.425)
All ages (rate per capita	0.489 (0.458 - 0.522)	0.490 (0.458 - 0.524)	0.492 (0.460 - 0.526)	0.656 (0.568 - 0.760)	0.657 (0.569 - 0.759)	0.658 (0.571 - 0.760)	1.580 (0.880 - 2.596)	1.576 (0.877 - 2.578)	1.595 (0.889 - 2.639)
Age-standardised (rate per capita	0.538 (0.513 - 0.564)	0.538 (0.512 - 0.564)	0.538 (0.512 - 0.564)	0.663 (0.593 - 0.739)	0.663 (0.593 - 0.739)	0.663 (0.594 - 0.740)	2.663 (1.511 - 4.373)	2.661 (1.518 - 4.367)	2.663 (1.510 - 4.365)
Total number of cases (thousands)	451 (422 - 482)	711 (664 - 760)	984 (919 - 1,052)	605 (524 - 700)	952 (824 - 1,101)	1,317 (1,142 - 1,521)	1 (1 - 2)	2 (1 - 4)	3 (2 - 5)
Ghana									
Under-5 years (rate per capita	0.360 (0.271 - 0.452)	0.358 (0.269 - 0.454)	0.360 (0.272 - 0.450)	0.505 (0.292 - 0.702)	0.506 (0.293 - 0.704)	0.510 (0.295 - 0.709)	0.084 (0.034 - 0.179)	0.084 (0.034 - 0.177)	0.085 (0.035 - 0.181)
5-14 years (rate per capita	0.399 (0.342 - 0.465)	0.400 (0.343 - 0.461)	0.402 (0.347 - 0.465)	0.660 (0.461 - 0.939)	0.655 (0.464 - 0.933)	0.660 (0.466 - 0.944)	0.458 (0.262 - 0.730)	0.462 (0.264 - 0.736)	0.462 (0.267 - 0.728)
15 - 49 years (rate per capita	0.423 (0.400 - 0.451)	0.425 (0.398 - 0.453)	0.426 (0.401 - 0.455)	0.573 (0.511 - 0.643)	0.574 (0.512 - 0.641)	0.575 (0.514 - 0.641)	1.785 (0.978 - 2.987)	1.812 (0.996 - 3.027)	1.871 (1.026 - 3.154)
50 - 69 years (rate per capita	0.527 (0.478 - 0.578)	0.529 (0.478 - 0.586)	0.527 (0.480 - 0.581)	0.569 (0.495 - 0.650)	0.568 (0.496 - 0.654)	0.568 (0.496 - 0.653)	4.225 (2.354 - 7.301)	4.244 (2.376 - 7.341)	4.255 (2.374 - 7.319)
70+ years (rate per capita	0.533 (0.486 - 0.579)	0.530 (0.479 - 0.576)	0.524 (0.478 - 0.571)	0.483 (0.423 - 0.553)	0.477 (0.414 - 0.548)	0.473 (0.411 - 0.542)	4.891 (2.917 - 7.894)	4.870 (2.893 - 7.846)	4.847 (2.907 - 7.838)
All ages (rate per capita	0.416 (0.389 - 0.449)	0.419 (0.391 - 0.449)	0.422 (0.394 - 0.448)	0.583 (0.510 - 0.678)	0.582 (0.512 - 0.666)	0.583 (0.516 - 0.666)	1.386 (0.766 - 2.305)	1.481 (0.826 - 2.463)	1.544 (0.862 - 2.594)
Age-standardised (rate per capita	0.443 (0.420 - 0.471)	0.444 (0.420 - 0.469)	0.443 (0.419 - 0.467)	0.575 (0.522 - 0.637)	0.575 (0.523 - 0.639)	0.575 (0.523 - 0.639)	2.147 (1.202 - 3.618)	2.151 (1.222 - 3.675)	2.163 (1.229 - 3.667)
Total number of cases (thousands)	6,092 (5,688 - 6,574)	8,971 (8,371 - 9,605)	11,558 (10,814 - 12,272)	8,540 (7,458 - 9,925)	12,445 (10,959 - 14,248)	15,997 (14,137 - 18,246)	20 (11 - 34)	32 (18 - 53)	42 (24 - 71)
Guinea									
Under-5 years (rate per capita	0.347 (0.264 - 0.439)	0.357 (0.277 - 0.447)	0.359 (0.274 - 0.451)	0.491 (0.284 - 0.683)	0.504 (0.292 - 0.701)	0.506 (0.293 - 0.704)	0.081 (0.034 - 0.171)	0.084 (0.035 - 0.176)	0.084 (0.035 - 0.176)

	Prevalence 1990	Prevalence 2005	Prevalence 2015	Incidence 1990	Incidence 2005	Incidence 2015	YLD rate 1990	YLD rate 2005	YLD rate 2015
5-14 years (rate per capita)	0.467 (0.403 - 0.534)	0.466 (0.405 - 0.530)	0.464 (0.406 - 0.529)	0.729 (0.519 - 1.012)	0.725 (0.517 - 1.004)	0.723 (0.517 - 0.999)	0.504 (0.286 - 0.811)	0.505 (0.286 - 0.810)	0.506 (0.284 - 0.815)
15 - 49 years (rate per capita)	0.512 (0.465 - 0.561)	0.511 (0.467 - 0.554)	0.510 (0.467 - 0.555)	0.676 (0.567 - 0.795)	0.674 (0.563 - 0.792)	0.675 (0.563 - 0.795)	1.974 (1.104 - 3.277)	1.943 (1.080 - 3.255)	1.936 (1.085 - 3.212)
50 - 69 years (rate per capita)	0.591 (0.541 - 0.652)	0.589 (0.540 - 0.646)	0.591 (0.542 - 0.646)	0.677 (0.530 - 0.840)	0.677 (0.530 - 0.841)	0.678 (0.531 - 0.844)	4.693 (2.670 - 7.875)	4.673 (2.665 - 7.831)	4.703 (2.656 - 7.896)
70+ years (rate per capita)	0.593 (0.544 - 0.642)	0.589 (0.543 - 0.638)	0.589 (0.538 - 0.642)	0.573 (0.439 - 0.726)	0.570 (0.439 - 0.721)	0.569 (0.439 - 0.720)	5.561 (3.348 - 8.718)	5.549 (3.367 - 8.642)	5.562 (3.348 - 8.665)
All ages (rate per capita)	0.480 (0.448 - 0.514)	0.480 (0.451 - 0.513)	0.482 (0.454 - 0.515)	0.655 (0.570 - 0.752)	0.656 (0.572 - 0.756)	0.659 (0.574 - 0.759)	1.580 (0.907 - 2.569)	1.538 (0.878 - 2.516)	1.572 (0.898 - 2.579)
Age-standardised (rate per capita)	0.512 (0.485 - 0.543)	0.511 (0.487 - 0.539)	0.511 (0.486 - 0.540)	0.662 (0.593 - 0.739)	0.662 (0.593 - 0.739)	0.662 (0.593 - 0.739)	2.354 (1.355 - 3.886)	2.359 (1.353 - 3.948)	2.367 (1.365 - 3.975)
Total number of cases (thousands)	2,894 (2,703 - 3,097)	4,660 (4,377 - 4,980)	6,064 (5,708 - 6,480)	3,950 (3,435 - 4,532)	6,368 (5,544 - 7,337)	8,285 (7,215 - 9,540)	10 (5 - 15)	15 (9 - 24)	20 (11 - 32)
**Guinea-Bissau**									
Under-5 years (rate per capita)	0.353 (0.267 - 0.446)	0.355 (0.272 - 0.453)	0.361 (0.275 - 0.452)	0.498 (0.288 - 0.693)	0.502 (0.291 - 0.698)	0.505 (0.293 - 0.703)	0.083 (0.035 - 0.178)	0.084 (0.034 - 0.178)	0.084 (0.035 - 0.180)
5-14 years (rate per capita)	0.512 (0.448 - 0.584)	0.494 (0.431 - 0.558)	0.495 (0.433 - 0.569)	0.737 (0.531 - 1.024)	0.711 (0.513 - 0.987)	0.714 (0.514 - 0.992)	0.540 (0.303 - 0.880)	0.530 (0.303 - 0.858)	0.530 (0.302 - 0.851)
15 - 49 years (rate per capita)	0.550 (0.524 - 0.579)	0.547 (0.522 - 0.577)	0.547 (0.523 - 0.574)	0.639 (0.578 - 0.708)	0.637 (0.577 - 0.708)	0.640 (0.579 - 0.711)	2.008 (1.123 - 3.318)	1.948 (1.087 - 3.230)	1.980 (1.107 - 3.303)
50 - 69 years (rate per capita)	0.612 (0.568 - 0.655)	0.609 (0.568 - 0.657)	0.610 (0.572 - 0.655)	0.672 (0.593 - 0.764)	0.673 (0.590 - 0.766)	0.673 (0.593 - 0.765)	4.739 (2.691 - 7.895)	4.684 (2.654 - 7.867)	4.714 (2.692 - 7.863)
70+ years (rate per capita)	0.617 (0.572 - 0.661)	0.611 (0.570 - 0.653)	0.612 (0.571 - 0.655)	0.572 (0.496 - 0.651)	0.568 (0.500 - 0.642)	0.570 (0.502 - 0.648)	5.571 (3.367 - 8.719)	5.532 (3.351 - 8.625)	5.543 (3.339 - 8.613)
All ages (rate per capita)	0.511 (0.484 - 0.539)	0.508 (0.483 - 0.539)	0.511 (0.486 - 0.540)	0.641 (0.564 - 0.732)	0.636 (0.561 - 0.725)	0.639 (0.567 - 0.725)	1.592 (0.913 - 2.602)	1.577 (0.902 - 2.624)	1.625 (0.931 - 2.680)
Age-standardised (rate per capita)	0.543 (0.521 - 0.563)	0.540 (0.519 - 0.562)	0.540 (0.518 - 0.561)	0.644 (0.593 - 0.704)	0.642 (0.588 - 0.705)	0.642 (0.588 - 0.705)	2.381 (1.375 - 3.939)	2.381 (1.369 - 3.976)	2.383 (1.390 - 4.018)
Total number of cases (thousands)	541 (513 - 570)	746 (708 - 790)	945 (898 - 999)	678 (597 - 774)	934 (823 - 1,064)	1,181 (1,047 - 1,340)	2 (1 - 3)	2 (1 - 4)	3 (2 - 5)
**Liberia**									
Under-5 years (rate per capita)	0.360 (0.271 - 0.458)	0.356 (0.270 - 0.447)	0.362 (0.275 - 0.455)	0.507 (0.294 - 0.705)	0.502 (0.291 - 0.698)	0.509 (0.295 - 0.708)	0.084 (0.035 - 0.180)	0.084 (0.035 - 0.176)	0.085 (0.035 - 0.181)
5-14 years (rate per capita)	0.466 (0.404 - 0.531)	0.468 (0.406 - 0.536)	0.464 (0.401 - 0.528)	0.727 (0.518 - 1.008)	0.730 (0.519 - 1.013)	0.721 (0.516 - 0.996)	0.503 (0.284 - 0.811)	0.503 (0.287 - 0.807)	0.505 (0.284 - 0.815)
15 - 49 years (rate per capita)	0.512 (0.467 - 0.561)	0.511 (0.468 - 0.554)	0.513 (0.468 - 0.558)	0.674 (0.562 - 0.793)	0.675 (0.562 - 0.796)	0.676 (0.566 - 0.794)	1.917 (1.078 - 3.182)	1.913 (1.078 - 3.176)	1.956 (1.089 - 3.238)
50 - 69 years (rate per capita)	0.589 (0.541 - 0.644)	0.591 (0.546 - 0.644)	0.590 (0.542 - 0.644)	0.677 (0.531 - 0.843)	0.677 (0.530 - 0.840)	0.678 (0.531 - 0.845)	4.607 (2.634 - 7.661)	4.625 (2.673 - 7.757)	4.617 (2.644 - 7.723)
70+ years (rate per capita)	0.593 (0.542 - 0.643)	0.590 (0.543 - 0.642)	0.589 (0.541 - 0.637)	0.572 (0.438 - 0.727)	0.570 (0.439 - 0.721)	0.570 (0.439 - 0.721)	5.447 (3.299 - 8.418)	5.407 (3.288 - 8.378)	5.458 (3.324 - 8.522)
All ages (rate per capita)	0.478 (0.449 - 0.515)	0.481 (0.451 - 0.512)	0.484 (0.452 - 0.516)	0.655 (0.569 - 0.756)	0.658 (0.572 - 0.756)	0.660 (0.576 - 0.758)	1.473 (0.847 - 2.391)	1.509 (0.867 - 2.494)	1.560 (0.891 - 2.557)
Age-standardised (rate per capita)	0.512 (0.488 - 0.542)	0.512 (0.487 - 0.539)	0.512 (0.487 - 0.542)	0.662 (0.593 - 0.738)	0.662 (0.593 - 0.739)	0.662 (0.593 - 0.739)	2.328 (1.345 - 3.823)	2.323 (1.344 - 3.861)	2.338 (1.356 - 3.915)
Total number of cases (thousands)	1,023 (960 - 1,102)	1,572 (1,476 - 1,675)	2,183 (2,040 - 2,328)	1,401 (1,217 - 1,617)	2,152 (1,872 - 2,474)	2,976 (2,597 - 3,417)	3 (2 - 5)	5 (3 - 8)	7 (4 - 12)
**Mali**									
Under-5 years (rate per capita)	0.350 (0.264 - 0.443)	0.354 (0.268 - 0.444)	0.357 (0.270 - 0.451)	0.495 (0.287 - 0.689)	0.497 (0.288 - 0.691)	0.504 (0.292 - 0.702)	0.082 (0.034 - 0.176)	0.082 (0.035 - 0.176)	0.084 (0.035 - 0.175)
5-14 years (rate per capita)	0.466 (0.403 - 0.530)	0.465 (0.401 - 0.529)	0.468 (0.407 - 0.532)	0.725 (0.517 - 1.003)	0.727 (0.518 - 1.008)	0.731 (0.520 - 1.014)	0.504 (0.285 - 0.809)	0.505 (0.287 - 0.817)	0.506 (0.285 - 0.800)
15 - 49 years (rate per capita)	0.510 (0.465 - 0.556)	0.508 (0.461 - 0.555)	0.510 (0.466 - 0.556)	0.671 (0.560 - 0.792)	0.673 (0.559 - 0.799)	0.674 (0.561 - 0.795)	1.883 (1.030 - 3.117)	1.857 (1.023 - 3.066)	1.912 (1.047 - 3.159)
50 - 69 years (rate per capita)	0.590 (0.542 - 0.649)	0.590 (0.541 - 0.650)	0.589 (0.542 - 0.645)	0.675 (0.529 - 0.835)	0.677 (0.531 - 0.840)	0.677 (0.531 - 0.841)	4.501 (2.573 - 7.631)	4.471 (2.540 - 7.615)	4.509 (2.577 - 7.633)
70+ years (rate per capita)	0.590 (0.534 - 0.644)	0.585 (0.537 - 0.635)	0.585 (0.531 - 0.633)	0.573 (0.436 - 0.726)	0.567 (0.439 - 0.715)	0.568 (0.440 - 0.718)	5.231 (3.164 - 8.287)	5.168 (3.110 - 8.139)	5.218 (3.131 - 8.275)
All ages (rate per capita)	0.476 (0.446 - 0.509)	0.474 (0.443 - 0.507)	0.476 (0.445 - 0.512)	0.651 (0.565 - 0.754)	0.653 (0.565 - 0.754)	0.658 (0.569 - 0.764)	1.458 (0.819 - 2.364)	1.390 (0.779 - 2.289)	1.387 (0.779 - 2.274)
Age-standardised (rate per capita)	0.511 (0.486 - 0.539)	0.511 (0.486 - 0.542)	0.511 (0.487 - 0.541)	0.662 (0.593 - 0.739)	0.662 (0.593 - 0.738)	0.662 (0.593 - 0.739)	2.280 (1.296 - 3.784)	2.280 (1.295 - 3.797)	2.301 (1.310 - 3.812)
Total number of cases (thousands)	4,052 (3,790 - 4,332)	6,110 (5,710 - 6,541)	8,365 (7,822 - 8,990)	5,540 (4,804 - 6,410)	8,421 (7,293 - 9,730)	11,557 (10,004 - 13,426)	12 (7 - 20)	18 (10 - 30)	24 (14 - 40)
**Mauritania**									
Under-5 years (rate per capita)	0.358 (0.271 - 0.450)	0.361 (0.271 - 0.454)	0.361 (0.276 - 0.454)	0.505 (0.292 - 0.702)	0.507 (0.293 - 0.705)	0.511 (0.296 - 0.710)	0.084 (0.035 - 0.179)	0.084 (0.035 - 0.178)	0.085 (0.035 - 0.180)
5-14 years (rate per capita)	0.465 (0.398 - 0.533)	0.467 (0.409 - 0.532)	0.465 (0.401 - 0.527)	0.724 (0.517 - 1.001)	0.723 (0.517 - 0.999)	0.721 (0.516 - 0.995)	0.505 (0.290 - 0.816)	0.506 (0.284 - 0.812)	0.507 (0.285 - 0.814)
15 - 49 years (rate per capita)	0.510 (0.463 - 0.558)	0.514 (0.473 - 0.557)	0.515 (0.472 - 0.557)	0.674 (0.561 - 0.797)	0.676 (0.564 - 0.797)	0.677 (0.568 - 0.795)	2.042 (1.173 - 3.327)	2.115 (1.185 - 3.458)	2.152 (1.229 - 3.539)
50 - 69 years (rate per capita)	0.595 (0.547 - 0.652)	0.592 (0.547 - 0.650)	0.591 (0.542 - 0.646)	0.678 (0.531 - 0.842)	0.678 (0.532 - 0.844)	0.679 (0.532 - 0.847)	4.977 (2.905 - 8.161)	4.986 (2.897 - 8.267)	4.964 (2.919 - 8.198)
70+ years (rate per capita)	0.594 (0.545 - 0.642)	0.592 (0.541 - 0.637)	0.589 (0.541 - 0.634)	0.572 (0.440 - 0.723)	0.570 (0.442 - 0.717)	0.569 (0.443 - 0.716)	5.921 (3.740 - 9.055)	5.939 (3.712 - 9.193)	5.912 (3.708 - 9.139)
All ages (rate per capita)	0.480 (0.447 - 0.516)	0.485 (0.455 - 0.518)	0.488 (0.458 - 0.520)	0.656 (0.570 - 0.758)	0.659 (0.575 - 0.757)	0.661 (0.578 - 0.756)	1.590 (0.916 - 2.530)	1.672 (0.953 - 2.678)	1.749 (1.007 - 2.844)
Age-standardised (rate per capita)	0.513 (0.486 - 0.545)	0.514 (0.490 - 0.543)	0.513 (0.488 - 0.542)	0.663 (0.593 - 0.739)	0.663 (0.593 - 0.739)	0.663 (0.593 - 0.739)	2.510 (1.472 - 4.109)	2.523 (1.479 - 4.121)	2.524 (1.481 - 4.106)
Total number of cases (thousands)	973 (907 - 1,046)	1,533 (1,438 - 1,638)	1,991 (1,870 - 2,126)	1,332 (1,157 - 1,537)	2,085 (1,819 - 2,395)	2,699 (2,363 - 3,088)	3 (2 - 5)	5 (3 - 8)	7 (4 - 12)
**Niger**									
Under-5 years (rate per capita)	0.435 (0.370 - 0.486)	0.444 (0.378 - 0.497)	0.449 (0.381 - 0.505)	0.496 (0.336 - 0.647)	0.507 (0.343 - 0.661)	0.512 (0.346 - 0.668)	0.102 (0.044 - 0.193)	0.104 (0.045 - 0.197)	0.105 (0.045 - 0.200)
5-14 years (rate per capita)	0.653 (0.601 - 0.706)	0.654 (0.600 - 0.706)	0.653 (0.599 - 0.703)	0.707 (0.546 - 0.914)	0.711 (0.547 - 0.923)	0.710 (0.546 - 0.922)	0.580 (0.325 - 0.955)	0.578 (0.321 - 0.943)	0.579 (0.325 - 0.946)
15 - 49 years (rate per capita)	0.541 (0.518 - 0.565)	0.541 (0.518 - 0.566)	0.541 (0.517 - 0.569)	0.637 (0.575 - 0.704)	0.635 (0.574 - 0.700)	0.632 (0.570 - 0.701)	1.794 (1.051 - 2.872)	1.806 (1.053 - 2.877)	1.746 (1.019 - 2.807)
50 - 69 years (rate per capita)	0.557 (0.527 - 0.587)	0.555 (0.526 - 0.584)	0.556 (0.527 - 0.589)	0.675 (0.590 - 0.771)	0.675 (0.590 - 0.770)	0.673 (0.588 - 0.769)	4.035 (2.392 - 6.260)	4.033 (2.386 - 6.297)	4.052 (2.394 - 6.358)
70+ years (rate per capita)	0.569 (0.541 - 0.601)	0.565 (0.536 - 0.595)	0.564 (0.537 - 0.593)	0.577 (0.506 - 0.653)	0.573 (0.503 - 0.649)	0.573 (0.503 - 0.649)	4.986 (3.072 - 7.631)	4.972 (3.086 - 7.528)	4.971 (3.053 - 7.521)
All ages (rate per capita)	0.552 (0.530 - 0.574)	0.555 (0.533 - 0.576)	0.556 (0.532 - 0.578)	0.631 (0.565 - 0.712)	0.632 (0.566 - 0.716)	0.633 (0.565 - 0.718)	1.328 (0.779 - 2.096)	1.324 (0.777 - 2.086)	1.290 (0.755 - 2.031)
Age-standardised (rate per capita)	0.557 (0.541 - 0.572)	0.556 (0.539 - 0.572)	0.556 (0.540 - 0.573)	0.642 (0.592 - 0.695)	0.641 (0.591 - 0.695)	0.641 (0.591 - 0.695)	2.116 (1.245 - 3.295)	2.117 (1.246 - 3.312)	2.126 (1.245 - 3.293)
Total number of cases (thousands)	4,374 (4,199 - 4,542)	7,463 (7,170 - 7,755)	11,049 (10,563 - 11,485)	4,994 (4,475 - 5,639)	8,511 (7,611 - 9,636)	12,571 (11,224 - 14,264)	11 (6 - 17)	18 (10 - 28)	26 (15 - 40)
**Nigeria**									
Under-5 years (rate per capita)	0.350 (0.274 - 0.443)	0.355 (0.272 - 0.448)	0.360 (0.275 - 0.452)	0.498 (0.288 - 0.693)	0.500 (0.290 - 0.696)	0.506 (0.293 - 0.705)	0.083 (0.034 - 0.179)	0.083 (0.034 - 0.175)	0.084 (0.035 - 0.183)
5-14 years (rate per capita)	0.466 (0.401 - 0.527)	0.465 (0.405 - 0.530)	0.466 (0.402 - 0.535)	0.726 (0.517 - 1.005)	0.727 (0.517 - 1.006)	0.726 (0.517 - 1.005)	0.505 (0.285 - 0.814)	0.505 (0.285 - 0.804)	0.506 (0.285 - 0.808)
15 - 49 years (rate per capita)	0.485 (0.445 - 0.530)	0.485 (0.441 - 0.532)	0.485 (0.441 - 0.532)	0.669 (0.560 - 0.787)	0.669 (0.557 - 0.790)	0.670 (0.560 - 0.791)	1.472 (0.854 - 2.301)	1.480 (0.859 - 2.338)	1.498 (0.865 - 2.361)
50 - 69 years (rate per capita)	0.485 (0.447 - 0.536)	0.491 (0.452 - 0.534)	0.490 (0.451 - 0.535)	0.670 (0.524 - 0.837)	0.670 (0.525 - 0.839)	0.670 (0.524 - 0.837)	3.042 (1.785 - 4.729)	3.103 (1.809 - 4.923)	3.111 (1.821 - 4.931)
70+ years (rate per capita)	0.488 (0.442 - 0.534)	0.494 (0.449 - 0.540)	0.491 (0.448 - 0.536)	0.568 (0.432 - 0.720)	0.567 (0.432 - 0.718)	0.567 (0.431 - 0.718)	3.665 (2.263 - 5.599)	3.691 (2.279 - 5.713)	3.721 (2.281 - 5.657)
All ages (rate per capita)	0.456 (0.426 - 0.488)	0.458 (0.426 - 0.490)	0.459 (0.428 - 0.491)	0.653 (0.567 - 0.753)	0.653 (0.567 - 0.752)	0.656 (0.569 - 0.755)	1.133 (0.659 - 1.761)	1.149 (0.667 - 1.800)	1.152 (0.668 - 1.805)
Age-standardised (rate per capita)	0.470 (0.445 - 0.497)	0.472 (0.447 - 0.498)	0.471 (0.446 - 0.497)	0.657 (0.588 - 0.734)	0.658 (0.588 - 0.735)	0.657 (0.588 - 0.734)	1.659 (0.966 - 2.579)	1.684 (0.977 - 2.634)	1.690 (0.984 - 2.661)
Total number of cases (thousands)	43,667 (40,837 - 46,773)	63,930 (59,521 - 68,498)	83,726 (78,172 - 89,690)	62,507 (54,328 - 72,102)	91,265 (79,236 - 105,107)	119,649 (103,916 - 137,700)	108 (63 - 169)	160 (93 - 251)	210 (122 - 329)
**Sao Tome and Principe**									
Under-5 years (rate per capita)	0.361 (0.267 - 0.456)	0.362 (0.275 - 0.455)	0.365 (0.274 - 0.458)	0.506 (0.293 - 0.704)	0.510 (0.295 - 0.709)	0.513 (0.297 - 0.713)	0.084 (0.035 - 0.180)	0.085 (0.035 - 0.182)	0.086 (0.035 - 0.185)
5-14 years (rate per capita)	0.461 (0.397 - 0.529)	0.467 (0.401 - 0.535)	0.465 (0.403 - 0.531)	0.707 (0.508 - 0.974)	0.724 (0.517 - 1.002)	0.719 (0.516 - 0.992)	0.509 (0.287 - 0.817)	0.507 (0.286 - 0.812)	0.507 (0.286 - 0.812)
15 - 49 years (rate per capita)	0.504 (0.456 - 0.556)	0.508 (0.463 - 0.556)	0.512 (0.469 - 0.556)	0.670 (0.554 - 0.795)	0.674 (0.560 - 0.796)	0.676 (0.564 - 0.796)	1.794 (1.001 - 2.983)	1.875 (1.038 - 3.131)	1.967 (1.100 - 3.272)
50 - 69 years (rate per capita)	0.592 (0.545 - 0.648)	0.592 (0.545 - 0.648)	0.588 (0.541 - 0.645)	0.676 (0.528 - 0.837)	0.671 (0.525 - 0.823)	0.680 (0.534 - 0.850)	4.765 (2.708 - 8.018)	4.783 (2.742 - 7.938)	4.642 (2.637 - 7.822)
70+ years (rate per capita)	0.576 (0.529 - 0.619)	0.577 (0.528 - 0.626)	0.570 (0.520 - 0.614)	0.560 (0.441 - 0.701)	0.560 (0.442 - 0.703)	0.553 (0.440 - 0.680)	5.456 (3.263 - 8.541)	5.475 (3.259 - 8.538)	5.391 (3.224 - 8.367)
All ages (rate per capita)	0.476 (0.445 - 0.513)	0.479 (0.449 - 0.515)	0.482 (0.454 - 0.516)	0.650 (0.562 - 0.756)	0.656 (0.572 - 0.756)	0.659 (0.575 - 0.757)	1.499 (0.862 - 2.410)	1.488 (0.844 - 2.451)	1.539 (0.880 - 2.531)
Age-standardised (rate per capita)	0.511 (0.485 - 0.543)	0.512 (0.487 - 0.542)	0.512 (0.488 - 0.541)	0.662 (0.593 - 0.738)	0.662 (0.593 - 0.739)	0.662 (0.593 - 0.739)	2.373 (1.370 - 3.950)	2.373 (1.357 - 3.937)	2.373 (1.382 - 3.971)
Total number of cases (thousands)	54 (51 - 58)	74 (69 - 79)	92 (87 - 99)	74 (64 - 86)	101 (88 - 116)	126 (110 - 145)	0 (0 - 0)	0 (0 - 0)	0 (0 - 0)
**Senegal**									
Under-5 years (rate per capita)	0.356 (0.272 - 0.452)	0.358 (0.269 - 0.453)	0.359 (0.275 - 0.455)	0.502 (0.291 - 0.698)	0.505 (0.292 - 0.702)	0.508 (0.294 - 0.707)	0.083 (0.034 - 0.178)	0.084 (0.035 - 0.179)	0.085 (0.035 - 0.179)
5-14 years (rate per capita)	0.465 (0.401 - 0.527)	0.465 (0.401 - 0.534)	0.466 (0.401 - 0.531)	0.725 (0.517 - 1.004)	0.721 (0.517 - 0.995)	0.726 (0.517 - 1.006)	0.506 (0.286 - 0.815)	0.507 (0.288 - 0.810)	0.507 (0.290 - 0.806)

	Prevalence 1990	Prevalence 2005	Prevalence 2015	Incidence 1990	Incidence 2005	Incidence 2015	YLD rate 1990	YLD rate 2005	YLD rate 2015
15 - 49 years (rate per capita)	0.512 (0.468 - 0.555)	0.511 (0.465 - 0.555)	0.512 (0.467 - 0.560)	0.672 (0.560 - 0.794)	0.673 (0.559 - 0.798)	0.676 (0.563 - 0.797)	2.041 (1.144 - 3.347)	2.037 (1.159 - 3.314)	2.096 (1.188 - 3.434)
50 - 69 years (rate per capita)	0.592 (0.545 - 0.647)	0.593 (0.543 - 0.645)	0.592 (0.548 - 0.643)	0.678 (0.530 - 0.842)	0.677 (0.531 - 0.838)	0.679 (0.533 - 0.845)	4.972 (2.863 - 8.321)	5.005 (2.927 - 8.325)	4.966 (2.865 - 8.250)
70+ years (rate per capita)	0.593 (0.545 - 0.641)	0.593 (0.547 - 0.638)	0.587 (0.540 - 0.631)	0.573 (0.439 - 0.724)	0.570 (0.441 - 0.720)	0.569 (0.442 - 0.717)	5.994 (3.700 - 9.308)	5.961 (3.688 - 9.275)	5.931 (3.713 - 9.200)
All ages (rate per capita)	0.478 (0.446 - 0.513)	0.480 (0.448 - 0.515)	0.481 (0.451 - 0.515)	0.655 (0.567 - 0.759)	0.656 (0.570 - 0.757)	0.659 (0.573 - 0.758)	1.553 (0.888 - 2.502)	1.582 (0.908 - 2.529)	1.609 (0.919 - 2.602)
Age-standardised (rate per capita)	0.513 (0.488 - 0.541)	0.514 (0.488 - 0.544)	0.513 (0.489 - 0.541)	0.662 (0.593 - 0.739)	0.662 (0.593 - 0.739)	0.663 (0.594 - 0.739)	2.519 (1.473 - 4.149)	2.528 (1.476 - 4.192)	2.525 (1.475 - 4.131)
Total number of cases (thousands)	3,588 (3,351 - 3,850)	5,416 (5,055 - 5,810)	7,268 (6,812 - 7,777)	4,918 (4,259 - 5,699)	7,397 (6,431 - 8,540)	9,954 (8,655 - 11,455)	12 (7 - 19)	18 (10 - 29)	24 (14 - 39)
**Sierra Leone**									
Under-5 years (rate per capita)	0.234 (0.180 - 0.284)	0.226 (0.174 - 0.280)	0.230 (0.176 - 0.285)	0.444 (0.183 - 0.709)	0.407 (0.175 - 0.677)	0.416 (0.179 - 0.692)	0.054 (0.022 - 0.111)	0.052 (0.021 - 0.108)	0.053 (0.021 - 0.112)
5-14 years (rate per capita)	0.496 (0.448 - 0.538)	0.445 (0.397 - 0.498)	0.443 (0.389 - 0.494)	0.681 (0.494 - 0.927)	0.588 (0.427 - 0.801)	0.587 (0.426 - 0.797)	0.509 (0.289 - 0.813)	0.495 (0.283 - 0.794)	0.498 (0.283 - 0.810)
15 - 49 years (rate per capita)	0.514 (0.491 - 0.542)	0.507 (0.482 - 0.535)	0.508 (0.483 - 0.534)	0.622 (0.561 - 0.690)	0.622 (0.556 - 0.691)	0.624 (0.558 - 0.693)	1.979 (1.119 - 3.280)	1.898 (1.071 - 3.142)	1.946 (1.100 - 3.246)
50 - 69 years (rate per capita)	0.581 (0.536 - 0.631)	0.582 (0.537 - 0.632)	0.581 (0.535 - 0.632)	0.642 (0.564 - 0.732)	0.643 (0.562 - 0.732)	0.642 (0.560 - 0.732)	4.649 (2.634 - 7.782)	4.639 (2.639 - 7.750)	4.683 (2.654 - 7.841)
70+ years (rate per capita)	0.594 (0.551 - 0.635)	0.595 (0.548 - 0.641)	0.591 (0.547 - 0.636)	0.549 (0.477 - 0.634)	0.551 (0.476 - 0.627)	0.548 (0.475 - 0.622)	5.574 (3.355 - 8.728)	5.548 (3.346 - 8.687)	5.581 (3.365 - 8.824)
All ages (rate per capita)	0.468 (0.445 - 0.487)	0.451 (0.425 - 0.472)	0.454 (0.431 - 0.475)	0.607 (0.531 - 0.703)	0.578 (0.504 - 0.671)	0.582 (0.509 - 0.673)	1.565 (0.905 - 2.572)	1.489 (0.861 - 2.427)	1.531 (0.881 - 2.478)
Age-standardised (rate per capita)	0.504 (0.484 - 0.524)	0.493 (0.472 - 0.514)	0.492 (0.471 - 0.515)	0.615 (0.560 - 0.679)	0.597 (0.543 - 0.660)	0.597 (0.543 - 0.660)	2.345 (1.358 - 3.899)	2.335 (1.351 - 3.855)	2.358 (1.354 - 3.891)
Total number of cases (thousands)	1,835 (1,747 - 1,911)	2,274 (2,144 - 2,381)	2,933 (2,784 - 3,067)	2,381 (2,081 - 2,755)	2,915 (2,545 - 3,384)	3,759 (3,290 - 4,350)	6 (4 - 10)	8 (4 - 12)	10 (6 - 16)
**Togo**									
Under-5 years (rate per capita)	0.356 (0.272 - 0.452)	0.356 (0.271 - 0.452)	0.362 (0.276 - 0.457)	0.503 (0.291 - 0.700)	0.504 (0.291 - 0.700)	0.509 (0.295 - 0.708)	0.084 (0.035 - 0.176)	0.084 (0.034 - 0.174)	0.085 (0.034 - 0.180)
5-14 years (rate per capita)	0.467 (0.405 - 0.533)	0.464 (0.402 - 0.531)	0.464 (0.399 - 0.528)	0.726 (0.517 - 1.007)	0.722 (0.517 - 0.997)	0.724 (0.517 - 1.003)	0.506 (0.287 - 0.814)	0.507 (0.286 - 0.817)	0.508 (0.283 - 0.814)
15 - 49 years (rate per capita)	0.512 (0.468 - 0.558)	0.510 (0.466 - 0.554)	0.512 (0.467 - 0.561)	0.673 (0.560 - 0.793)	0.673 (0.559 - 0.799)	0.677 (0.564 - 0.797)	1.917 (1.057 - 3.175)	1.893 (1.054 - 3.138)	1.971 (1.092 - 3.281)
50 - 69 years (rate per capita)	0.591 (0.543 - 0.647)	0.591 (0.541 - 0.647)	0.591 (0.543 - 0.648)	0.677 (0.531 - 0.842)	0.678 (0.532 - 0.843)	0.678 (0.532 - 0.844)	4.684 (2.662 - 7.884)	4.685 (2.668 - 7.944)	4.700 (2.657 - 7.870)
70+ years (rate per capita)	0.590 (0.541 - 0.641)	0.590 (0.540 - 0.644)	0.589 (0.540 - 0.639)	0.572 (0.440 - 0.723)	0.570 (0.440 - 0.721)	0.571 (0.440 - 0.721)	5.542 (3.338 - 8.708)	5.541 (3.343 - 8.703)	5.567 (3.359 - 8.663)
All ages (rate per capita)	0.479 (0.446 - 0.515)	0.479 (0.448 - 0.515)	0.483 (0.451 - 0.516)	0.656 (0.569 - 0.759)	0.656 (0.571 - 0.757)	0.661 (0.576 - 0.760)	1.471 (0.843 - 2.397)	1.494 (0.857 - 2.442)	1.555 (0.889 - 2.580)
Age-standardised (rate per capita)	0.513 (0.486 - 0.541)	0.512 (0.486 - 0.543)	0.512 (0.487 - 0.542)	0.662 (0.593 - 0.739)	0.662 (0.593 - 0.739)	0.662 (0.593 - 0.739)	2.360 (1.367 - 3.917)	2.361 (1.350 - 3.927)	2.372 (1.380 - 3.936)
Total number of cases (thousands)	1,813 (1,690 - 1,950)	2,675 (2,503 - 2,877)	3,528 (3,292 - 3,771)	2,484 (2,155 - 2,874)	3,664 (3,186 - 4,227)	4,828 (4,209 - 5,552)	6 (3 - 9)	8 (5 - 14)	11 (6 - 19)
**Eastern Sub-Saharan Africa**									
Under-5 years (rate per capita)	0.361 (0.329 - 0.394)	0.344 (0.313 - 0.376)	0.354 (0.324 - 0.388)	0.529 (0.320 - 0.721)	0.469 (0.307 - 0.612)	0.518 (0.317 - 0.702)	0.085 (0.036 - 0.166)	0.081 (0.035 - 0.159)	0.083 (0.036 - 0.163)
5-14 years (rate per capita)	0.470 (0.442 - 0.500)	0.475 (0.447 - 0.504)	0.473 (0.444 - 0.504)	0.739 (0.556 - 0.965)	0.744 (0.555 - 0.983)	0.744 (0.555 - 0.982)	0.513 (0.294 - 0.827)	0.520 (0.298 - 0.842)	0.518 (0.295 - 0.837)
15 - 49 years (rate per capita)	0.490 (0.472 - 0.510)	0.487 (0.468 - 0.510)	0.488 (0.469 - 0.511)	0.725 (0.630 - 0.813)	0.720 (0.627 - 0.807)	0.724 (0.632 - 0.811)	1.552 (0.869 - 2.630)	1.517 (0.852 - 2.539)	1.543 (0.860 - 2.578)
50 - 69 years (rate per capita)	0.563 (0.532 - 0.589)	0.561 (0.530 - 0.591)	0.561 (0.532 - 0.591)	0.769 (0.635 - 0.897)	0.766 (0.637 - 0.892)	0.764 (0.634 - 0.888)	3.573 (1.994 - 6.187)	3.555 (1.996 - 6.184)	3.571 (1.999 - 6.244)
70+ years (rate per capita)	0.572 (0.541 - 0.601)	0.568 (0.537 - 0.597)	0.567 (0.538 - 0.595)	0.695 (0.563 - 0.830)	0.693 (0.564 - 0.819)	0.695 (0.566 - 0.826)	4.225 (2.483 - 6.852)	4.201 (2.474 - 6.832)	4.201 (2.467 - 6.770)
All ages (rate per capita)	0.467 (0.453 - 0.481)	0.465 (0.450 - 0.480)	0.469 (0.455 - 0.485)	0.695 (0.616 - 0.783)	0.685 (0.611 - 0.771)	0.698 (0.623 - 0.784)	1.182 (0.664 - 1.969)	1.182 (0.664 - 1.958)	1.224 (0.690 - 2.030)
Age-standardised (rate per capita)	0.498 (0.482 - 0.512)	0.496 (0.482 - 0.511)	0.496 (0.483 - 0.511)	0.718 (0.658 - 0.785)	0.710 (0.653 - 0.776)	0.716 (0.658 - 0.784)	1.865 (1.052 - 3.152)	1.856 (1.050 - 3.145)	1.862 (1.049 - 3.160)
Total number of cases (thousands)	86,918 (84,233 - 89,495)	131,437 (127,264 - 135,700)	176,833 (171,629 - 182,742)	129,278 (114,595 - 145,639)	193,594 (172,752 - 217,898)	263,144 (234,697 - 295,449)	220 (124 - 366)	334 (188 - 554)	461 (260 - 765)
**Burundi**									
Under-5 years (rate per capita)	0.398 (0.321 - 0.480)	0.393 (0.319 - 0.475)	0.400 (0.322 - 0.483)	0.550 (0.340 - 0.732)	0.547 (0.338 - 0.728)	0.555 (0.343 - 0.739)	0.094 (0.039 - 0.186)	0.093 (0.039 - 0.189)	0.095 (0.040 - 0.189)
5-14 years (rate per capita)	0.465 (0.391 - 0.544)	0.459 (0.389 - 0.536)	0.464 (0.395 - 0.538)	0.842 (0.598 - 1.148)	0.825 (0.580 - 1.121)	0.840 (0.595 - 1.144)	0.510 (0.288 - 0.829)	0.512 (0.288 - 0.847)	0.511 (0.288 - 0.831)
15 - 49 years (rate per capita)	0.483 (0.426 - 0.540)	0.478 (0.424 - 0.537)	0.479 (0.426 - 0.542)	0.785 (0.635 - 0.919)	0.780 (0.628 - 0.915)	0.784 (0.632 - 0.920)	1.594 (0.883 - 2.724)	1.530 (0.857 - 2.627)	1.579 (0.876 - 2.695)
50 - 69 years (rate per capita)	0.583 (0.516 - 0.657)	0.580 (0.512 - 0.651)	0.578 (0.510 - 0.657)	0.801 (0.585 - 1.000)	0.799 (0.587 - 0.993)	0.801 (0.579 - 1.003)	3.852 (2.135 - 6.852)	3.796 (2.115 - 6.728)	3.846 (2.124 - 6.858)
70+ years (rate per capita)	0.592 (0.516 - 0.667)	0.588 (0.524 - 0.658)	0.588 (0.520 - 0.659)	0.745 (0.528 - 0.968)	0.743 (0.526 - 0.961)	0.740 (0.526 - 0.954)	4.603 (2.651 - 7.579)	4.594 (2.668 - 7.523)	4.618 (2.679 - 7.585)
All ages (rate per capita)	0.470 (0.433 - 0.510)	0.467 (0.431 - 0.507)	0.469 (0.433 - 0.512)	0.753 (0.651 - 0.863)	0.752 (0.648 - 0.868)	0.756 (0.655 - 0.866)	1.206 (0.680 - 2.076)	1.201 (0.676 - 2.058)	1.237 (0.687 - 2.108)
Age-standardised (rate per capita)	0.501 (0.467 - 0.542)	0.500 (0.467 - 0.537)	0.499 (0.463 - 0.539)	0.772 (0.684 - 0.867)	0.772 (0.684 - 0.867)	0.772 (0.684 - 0.867)	1.957 (1.085 - 3.396)	1.953 (1.089 - 3.342)	1.971 (1.100 - 3.353)
Total number of cases (thousands)	2,644 (2,438 - 2,868)	3,714 (3,431 - 4,040)	5,278 (4,870 - 5,760)	4,240 (3,663 - 4,856)	5,988 (5,157 - 6,908)	8,504 (7,367 - 9,744)	7 (4 - 12)	10 (5 - 16)	14 (8 - 24)
**Comoros**									
Under-5 years (rate per capita)	0.404 (0.319 - 0.488)	0.404 (0.323 - 0.482)	0.408 (0.322 - 0.491)	0.556 (0.344 - 0.741)	0.558 (0.345 - 0.743)	0.562 (0.347 - 0.748)	0.094 (0.040 - 0.189)	0.095 (0.040 - 0.191)	0.096 (0.040 - 0.195)
5-14 years (rate per capita)	0.463 (0.388 - 0.542)	0.460 (0.385 - 0.535)	0.462 (0.392 - 0.536)	0.837 (0.592 - 1.140)	0.828 (0.583 - 1.128)	0.830 (0.585 - 1.130)	0.509 (0.290 - 0.835)	0.510 (0.288 - 0.842)	0.510 (0.292 - 0.839)
15 - 49 years (rate per capita)	0.480 (0.427 - 0.539)	0.483 (0.434 - 0.546)	0.483 (0.435 - 0.542)	0.784 (0.631 - 0.919)	0.783 (0.632 - 0.920)	0.785 (0.636 - 0.919)	1.651 (0.922 - 2.822)	1.669 (0.943 - 2.853)	1.710 (0.963 - 2.904)
50 - 69 years (rate per capita)	0.581 (0.513 - 0.653)	0.583 (0.513 - 0.657)	0.579 (0.514 - 0.650)	0.801 (0.586 - 1.000)	0.800 (0.587 - 0.996)	0.801 (0.583 - 0.999)	4.014 (2.296 - 7.062)	4.004 (2.258 - 7.053)	3.995 (2.265 - 7.107)
70+ years (rate per capita)	0.596 (0.526 - 0.666)	0.592 (0.524 - 0.670)	0.591 (0.520 - 0.661)	0.748 (0.527 - 0.972)	0.745 (0.529 - 0.965)	0.744 (0.528 - 0.965)	4.856 (2.895 - 7.699)	4.853 (2.877 - 7.873)	4.856 (2.875 - 7.749)
All ages (rate per capita)	0.471 (0.434 - 0.511)	0.474 (0.438 - 0.515)	0.476 (0.443 - 0.517)	0.758 (0.656 - 0.873)	0.759 (0.658 - 0.868)	0.763 (0.663 - 0.867)	1.286 (0.727 - 2.186)	1.335 (0.753 - 2.285)	1.406 (0.792 - 2.426)
Age-standardised (rate per capita)	0.501 (0.466 - 0.538)	0.502 (0.469 - 0.541)	0.501 (0.468 - 0.540)	0.773 (0.684 - 0.867)	0.773 (0.684 - 0.867)	0.773 (0.684 - 0.867)	2.048 (1.172 - 3.462)	2.054 (1.178 - 3.521)	2.058 (1.175 - 3.546)
Total number of cases (thousands)	196 (180 - 212)	294 (273 - 320)	377 (351 - 409)	314 (272 - 362)	472 (409 - 539)	604 (525 - 686)	1 (0 - 1)	1 (0 - 1)	1 (1 - 2)
**Djibouti**									
Under-5 years (rate per capita)	0.395 (0.315 - 0.471)	0.408 (0.330 - 0.493)	0.409 (0.326 - 0.485)	0.547 (0.338 - 0.728)	0.562 (0.347 - 0.749)	0.564 (0.349 - 0.752)	0.093 (0.040 - 0.188)	0.095 (0.040 - 0.191)	0.096 (0.041 - 0.193)
5-14 years (rate per capita)	0.459 (0.386 - 0.534)	0.455 (0.383 - 0.535)	0.459 (0.384 - 0.535)	0.824 (0.579 - 1.121)	0.816 (0.569 - 1.110)	0.822 (0.577 - 1.117)	0.510 (0.289 - 0.835)	0.511 (0.291 - 0.843)	0.511 (0.292 - 0.833)
15 - 49 years (rate per capita)	0.481 (0.433 - 0.540)	0.482 (0.433 - 0.542)	0.486 (0.435 - 0.548)	0.784 (0.634 - 0.919)	0.784 (0.635 - 0.918)	0.787 (0.640 - 0.921)	1.581 (0.872 - 2.735)	1.602 (0.885 - 2.758)	1.666 (0.917 - 2.858)
50 - 69 years (rate per capita)	0.580 (0.516 - 0.653)	0.583 (0.519 - 0.659)	0.580 (0.516 - 0.654)	0.801 (0.586 - 0.997)	0.801 (0.584 - 1.002)	0.800 (0.585 - 0.995)	3.834 (2.120 - 6.822)	3.851 (2.122 - 6.894)	3.821 (2.122 - 6.777)
70+ years (rate per capita)	0.591 (0.523 - 0.663)	0.592 (0.522 - 0.669)	0.590 (0.527 - 0.659)	0.747 (0.527 - 0.973)	0.744 (0.527 - 0.964)	0.742 (0.527 - 0.961)	4.611 (2.642 - 7.669)	4.584 (2.633 - 7.541)	4.567 (2.632 - 7.488)
All ages (rate per capita)	0.470 (0.435 - 0.510)	0.476 (0.441 - 0.519)	0.484 (0.448 - 0.525)	0.757 (0.654 - 0.868)	0.763 (0.665 - 0.870)	0.769 (0.676 - 0.869)	1.267 (0.707 - 2.160)	1.386 (0.776 - 2.403)	1.538 (0.852 - 2.658)
Age-standardised (rate per capita)	0.500 (0.468 - 0.539)	0.501 (0.468 - 0.540)	0.501 (0.466 - 0.543)	0.772 (0.684 - 0.867)	0.772 (0.684 - 0.867)	0.772 (0.684 - 0.867)	1.959 (1.093 - 3.368)	1.957 (1.095 - 3.380)	1.958 (1.091 - 3.423)
Total number of cases (thousands)	270 (250 - 293)	373 (345 - 406)	431 (399 - 467)	435 (376 - 498)	597 (520 - 680)	684 (601 - 774)	1 (0 - 1)	1 (1 - 2)	1 (1 - 2)
**Eritrea**									
Under-5 years (rate per capita)	0.401 (0.321 - 0.486)	0.400 (0.320 - 0.485)	0.408 (0.331 - 0.489)	0.556 (0.344 - 0.740)	0.552 (0.342 - 0.735)	0.564 (0.349 - 0.751)	0.094 (0.040 - 0.184)	0.094 (0.040 - 0.188)	0.096 (0.041 - 0.192)
5-14 years (rate per capita)	0.462 (0.387 - 0.538)	0.458 (0.386 - 0.536)	0.462 (0.393 - 0.543)	0.835 (0.589 - 1.136)	0.822 (0.577 - 1.117)	0.833 (0.588 - 1.134)	0.507 (0.292 - 0.825)	0.508 (0.290 - 0.838)	0.509 (0.290 - 0.822)
15 - 49 years (rate per capita)	0.481 (0.429 - 0.540)	0.477 (0.419 - 0.537)	0.479 (0.428 - 0.542)	0.783 (0.634 - 0.919)	0.780 (0.624 - 0.918)	0.786 (0.636 - 0.921)	1.585 (0.877 - 2.697)	1.478 (0.831 - 2.536)	1.576 (0.884 - 2.680)
50 - 69 years (rate per capita)	0.578 (0.505 - 0.653)	0.580 (0.512 - 0.656)	0.579 (0.510 - 0.654)	0.800 (0.583 - 1.000)	0.801 (0.581 - 1.003)	0.801 (0.586 - 0.999)	3.780 (2.083 - 6.769)	3.802 (2.117 - 6.751)	3.832 (2.119 - 6.775)
70+ years (rate per capita)	0.595 (0.521 - 0.679)	0.592 (0.523 - 0.667)	0.593 (0.523 - 0.665)	0.753 (0.529 - 0.984)	0.752 (0.529 - 0.983)	0.749 (0.530 - 0.976)	4.626 (2.652 - 7.609)	4.599 (2.638 - 7.581)	4.606 (2.651 - 7.506)
All ages (rate per capita)	0.468 (0.430 - 0.509)	0.468 (0.432 - 0.511)	0.472 (0.436 - 0.509)	0.757 (0.653 - 0.873)	0.755 (0.651 - 0.869)	0.764 (0.664 - 0.872)	1.170 (0.656 - 2.021)	1.207 (0.676 - 2.054)	1.256 (0.707 - 2.148)
Age-standardised (rate per capita)	0.500 (0.465 - 0.539)	0.501 (0.468 - 0.542)	0.499 (0.464 - 0.536)	0.773 (0.684 - 0.867)	0.773 (0.684 - 0.867)	0.773 (0.684 - 0.867)	1.953 (1.096 - 3.394)	1.942 (1.080 - 3.355)	1.946 (1.091 - 3.331)
Total number of cases (thousands)	1,471 (1,352 - 1,599)	1,965 (1,813 - 2,145)	2,473 (2,288 - 2,668)	2,379 (2,051 - 2,744)	3,169 (2,733 - 3,644)	4,006 (3,483 - 4,570)	4 (2 - 6)	5 (3 - 9)	7 (4 - 11)
**Ethiopia**									
Under-5 years (rate per capita)	0.395 (0.312 - 0.476)	0.404 (0.322 - 0.487)	0.404 (0.328 - 0.490)	0.545 (0.337 - 0.726)	0.559 (0.345 - 0.744)	0.561 (0.347 - 0.747)	0.092 (0.038 - 0.186)	0.095 (0.041 - 0.192)	0.096 (0.040 - 0.192)
5-14 years (rate per capita)	0.465 (0.389 - 0.546)	0.464 (0.393 - 0.539)	0.458 (0.382 - 0.534)	0.837 (0.591 - 1.139)	0.836 (0.590 - 1.137)	0.823 (0.578 - 1.119)	0.508 (0.288 - 0.828)	0.509 (0.286 - 0.826)	0.512 (0.294 - 0.833)
15 - 49 years (rate per capita)	0.483 (0.433 - 0.541)	0.480 (0.430 - 0.546)	0.480 (0.428 - 0.539)	0.784 (0.633 - 0.916)	0.782 (0.630 - 0.921)	0.783 (0.629 - 0.921)	1.578 (0.865 - 2.715)	1.533 (0.835 - 2.652)	1.537 (0.843 - 2.654)

	Prevalence 1990	Prevalence 2005	Prevalence 2015	Incidence 1990	Incidence 2005	Incidence 2015	YLD rate 1990	YLD rate 2005	YLD rate 2015
50 - 69 years (rate per capita)	0.579 (0.513 - 0.656)	0.580 (0.506 - 0.654)	0.581 (0.512 - 0.662)	0.800 (0.586 - 0.998)	0.801 (0.584 - 1.001)	0.801 (0.585 - 0.999)	3.681 (1.988 - 6.775)	3.682 (1.992 - 6.626)	3.707 (2.022 - 6.770)
70+ years (rate per capita)	0.592 (0.517 - 0.665)	0.587 (0.512 - 0.659)	0.587 (0.519 - 0.661)	0.745 (0.524 - 0.971)	0.742 (0.527 - 0.963)	0.740 (0.526 - 0.956)	4.340 (2.493 - 7.188)	4.308 (2.487 - 7.057)	4.322 (2.513 - 7.243)
All ages (rate per capita)	0.472 (0.437 - 0.509)	0.472 (0.436 - 0.515)	0.473 (0.440 - 0.518)	0.754 (0.655 - 0.864)	0.759 (0.657 - 0.875)	0.761 (0.659 - 0.869)	1.231 (0.679 - 2.159)	1.210 (0.670 - 2.085)	1.282 (0.707 - 2.238)
Age-standardised (rate per capita)	0.500 (0.468 - 0.536)	0.500 (0.467 - 0.542)	0.500 (0.467 - 0.538)	0.772 (0.684 - 0.867)	0.772 (0.684 - 0.867)	0.772 (0.684 - 0.867)	1.892 (1.041 - 3.310)	1.890 (1.045 - 3.308)	1.898 (1.040 - 3.279)
Total number of cases (thousands)	22,587 (20,936 - 24,360)	36,077 (33,310 - 39,385)	47,019 (43,744 - 51,485)	36,123 (31,379 - 41,386)	58,045 (50,234 - 66,883)	75,690 (65,500 - 86,430)	59 (33 - 103)	92 (51 - 159)	127 (70 - 223)
**Kenya**									
Under-5 years (rate per capita)	0.315 (0.295 - 0.335)	0.328 (0.309 - 0.347)	0.324 (0.305 - 0.345)	0.511 (0.286 - 0.733)	0.499 (0.284 - 0.698)	0.514 (0.292 - 0.732)	0.074 (0.032 - 0.152)	0.077 (0.033 - 0.155)	0.077 (0.033 - 0.156)
5-14 years (rate per capita)	0.394 (0.373 - 0.412)	0.402 (0.376 - 0.423)	0.399 (0.376 - 0.417)	0.582 (0.430 - 0.770)	0.576 (0.422 - 0.770)	0.578 (0.431 - 0.764)	0.464 (0.268 - 0.725)	0.469 (0.270 - 0.736)	0.467 (0.270 - 0.733)
15 - 49 years (rate per capita)	0.372 (0.362 - 0.381)	0.361 (0.351 - 0.369)	0.368 (0.359 - 0.376)	0.427 (0.380 - 0.475)	0.421 (0.375 - 0.470)	0.427 (0.382 - 0.477)	1.668 (0.896 - 2.892)	1.606 (0.869 - 2.765)	1.688 (0.909 - 2.913)
50 - 69 years (rate per capita)	0.514 (0.500 - 0.528)	0.502 (0.488 - 0.517)	0.505 (0.492 - 0.518)	0.425 (0.375 - 0.481)	0.423 (0.374 - 0.484)	0.426 (0.375 - 0.485)	3.822 (1.999 - 6.726)	3.714 (1.949 - 6.495)	3.752 (1.967 - 6.530)
70+ years (rate per capita)	0.495 (0.482 - 0.509)	0.495 (0.480 - 0.508)	0.489 (0.475 - 0.503)	0.357 (0.315 - 0.404)	0.357 (0.313 - 0.403)	0.355 (0.313 - 0.402)	3.949 (2.219 - 6.516)	3.963 (2.224 - 6.594)	3.936 (2.224 - 6.525)
All ages (rate per capita)	0.379 (0.369 - 0.388)	0.378 (0.369 - 0.387)	0.381 (0.373 - 0.391)	0.488 (0.419 - 0.572)	0.474 (0.411 - 0.551)	0.479 (0.419 - 0.552)	1.182 (0.641 - 1.972)	1.234 (0.676 - 2.057)	1.306 (0.709 - 2.183)
Age-standardised (rate per capita)	0.413 (0.404 - 0.422)	0.409 (0.401 - 0.417)	0.408 (0.399 - 0.415)	0.456 (0.414 - 0.506)	0.453 (0.409 - 0.503)	0.455 (0.413 - 0.503)	1.989 (1.074 - 3.430)	1.932 (1.053 - 3.286)	1.938 (1.053 - 3.319)
Total number of cases (thousands)	8,885 (8,644 - 9,104)	13,379 (13,064 - 13,706)	17,620 (17,219 - 18,040)	11,444 (9,825 - 13,409)	16,781 (14,571 - 19,531)	22,133 (19,352 - 25,513)	28 (15 - 46)	44 (24 - 73)	60 (33 - 101)
**Madagascar**									
Under-5 years (rate per capita)	0.462 (0.399 - 0.515)	0.458 (0.401 - 0.511)	0.459 (0.403 - 0.508)	0.529 (0.379 - 0.683)	0.552 (0.399 - 0.699)	0.551 (0.399 - 0.698)	0.108 (0.047 - 0.209)	0.108 (0.048 - 0.210)	0.108 (0.047 - 0.210)
5-14 years (rate per capita)	0.789 (0.760 - 0.816)	0.666 (0.613 - 0.712)	0.666 (0.615 - 0.713)	0.942 (0.800 - 1.125)	0.871 (0.713 - 1.091)	0.866 (0.710 - 1.085)	0.728 (0.390 - 1.228)	0.638 (0.352 - 1.060)	0.642 (0.352 - 1.055)
15 - 49 years (rate per capita)	0.639 (0.618 - 0.658)	0.637 (0.616 - 0.658)	0.636 (0.616 - 0.657)	0.825 (0.774 - 0.883)	0.833 (0.784 - 0.889)	0.832 (0.784 - 0.888)	1.471 (0.860 - 2.348)	1.507 (0.876 - 2.399)	1.511 (0.881 - 2.418)
50 - 69 years (rate per capita)	0.626 (0.603 - 0.649)	0.624 (0.600 - 0.647)	0.623 (0.600 - 0.648)	0.976 (0.907 - 1.044)	0.976 (0.901 - 1.041)	0.977 (0.902 - 1.043)	3.009 (1.787 - 4.616)	3.004 (1.776 - 4.629)	3.011 (1.779 - 4.682)
70+ years (rate per capita)	0.634 (0.611 - 0.658)	0.638 (0.615 - 0.662)	0.633 (0.611 - 0.658)	0.943 (0.877 - 1.010)	0.943 (0.873 - 1.009)	0.941 (0.875 - 1.004)	3.688 (2.275 - 5.565)	3.729 (2.281 - 5.638)	3.728 (2.313 - 5.652)
All ages (rate per capita)	0.645 (0.627 - 0.661)	0.614 (0.592 - 0.632)	0.616 (0.598 - 0.634)	0.817 (0.758 - 0.884)	0.808 (0.745 - 0.882)	0.812 (0.751 - 0.877)	1.184 (0.688 - 1.888)	1.177 (0.689 - 1.873)	1.229 (0.716 - 1.944)
Age-standardised (rate per capita)	0.647 (0.634 - 0.659)	0.624 (0.608 - 0.639)	0.624 (0.610 - 0.637)	0.859 (0.817 - 0.907)	0.849 (0.805 - 0.897)	0.849 (0.805 - 0.897)	1.713 (1.012 - 2.661)	1.706 (1.007 - 2.645)	1.713 (1.009 - 2.669)
Total number of cases (thousands)	7,456 (7,248 - 7,640)	11,218 (10,820 - 11,553)	14,894 (14,458 - 15,327)	9,433 (8,758 - 10,215)	14,778 (13,626 - 16,116)	19,637 (18,178 - 21,223)	14 (8 - 22)	22 (13 - 34)	30 (17 - 47)
**Malawi**									
Under-5 years (rate per capita)	0.394 (0.312 - 0.470)	0.400 (0.320 - 0.480)	0.404 (0.322 - 0.486)	0.543 (0.336 - 0.724)	0.551 (0.340 - 0.734)	0.557 (0.344 - 0.742)	0.092 (0.039 - 0.181)	0.094 (0.040 - 0.189)	0.095 (0.041 - 0.195)
5-14 years (rate per capita)	0.461 (0.389 - 0.538)	0.463 (0.393 - 0.544)	0.463 (0.389 - 0.540)	0.830 (0.586 - 1.130)	0.832 (0.587 - 1.131)	0.834 (0.588 - 1.134)	0.509 (0.289 - 0.830)	0.509 (0.292 - 0.835)	0.510 (0.291 - 0.833)
15 - 49 years (rate per capita)	0.482 (0.432 - 0.541)	0.477 (0.423 - 0.538)	0.477 (0.426 - 0.537)	0.784 (0.633 - 0.919)	0.781 (0.626 - 0.919)	0.783 (0.630 - 0.923)	1.641 (0.919 - 2.770)	1.547 (0.866 - 2.622)	1.585 (0.884 - 2.699)
50 - 69 years (rate per capita)	0.581 (0.519 - 0.653)	0.582 (0.515 - 0.654)	0.580 (0.513 - 0.653)	0.801 (0.588 - 0.997)	0.802 (0.583 - 1.007)	0.801 (0.590 - 0.998)	3.913 (2.202 - 6.931)	3.939 (2.231 - 6.901)	3.986 (2.258 - 6.986)
70+ years (rate per capita)	0.598 (0.526 - 0.671)	0.591 (0.527 - 0.662)	0.590 (0.522 - 0.666)	0.753 (0.524 - 0.985)	0.744 (0.528 - 0.966)	0.746 (0.529 - 0.967)	4.751 (2.801 - 7.607)	4.686 (2.778 - 7.591)	4.722 (2.781 - 7.690)
All ages (rate per capita)	0.469 (0.433 - 0.508)	0.469 (0.434 - 0.512)	0.469 (0.432 - 0.509)	0.753 (0.653 - 0.864)	0.755 (0.650 - 0.872)	0.759 (0.656 - 0.870)	1.254 (0.706 - 2.149)	1.220 (0.686 - 2.059)	1.249 (0.704 - 2.100)
Age-standardised (rate per capita)	0.500 (0.468 - 0.539)	0.501 (0.468 - 0.540)	0.499 (0.466 - 0.536)	0.772 (0.684 - 0.867)	0.773 (0.684 - 0.867)	0.773 (0.684 - 0.867)	2.002 (1.135 - 3.412)	1.999 (1.135 - 3.421)	2.007 (1.141 - 3.454)
Total number of cases (thousands)	4,405 (4,073 - 4,777)	5,997 (5,544 - 6,553)	8,081 (7,438 - 8,770)	7,076 (6,140 - 8,122)	9,652 (8,317 - 11,151)	13,063 (11,298 - 14,982)	12 (7 - 20)	16 (9 - 26)	22 (12 - 36)
**Mozambique**									
Under-5 years (rate per capita)	0.397 (0.319 - 0.479)	0.399 (0.319 - 0.482)	0.402 (0.317 - 0.484)	0.548 (0.339 - 0.730)	0.552 (0.341 - 0.735)	0.557 (0.344 - 0.742)	0.093 (0.039 - 0.184)	0.094 (0.040 - 0.189)	0.095 (0.040 - 0.189)
5-14 years (rate per capita)	0.483 (0.424 - 0.550)	0.485 (0.425 - 0.556)	0.485 (0.424 - 0.554)	0.743 (0.531 - 1.021)	0.750 (0.535 - 1.030)	0.744 (0.530 - 1.020)	0.519 (0.292 - 0.840)	0.520 (0.291 - 0.843)	0.522 (0.293 - 0.840)
15 - 49 years (rate per capita)	0.541 (0.512 - 0.572)	0.536 (0.510 - 0.565)	0.535 (0.513 - 0.565)	0.744 (0.677 - 0.816)	0.746 (0.678 - 0.819)	0.745 (0.677 - 0.820)	1.600 (0.890 - 2.763)	1.594 (0.899 - 2.709)	1.584 (0.879 - 2.736)
50 - 69 years (rate per capita)	0.589 (0.530 - 0.655)	0.590 (0.538 - 0.651)	0.591 (0.537 - 0.649)	0.818 (0.723 - 0.919)	0.816 (0.719 - 0.915)	0.816 (0.720 - 0.915)	3.811 (2.129 - 6.780)	3.799 (2.111 - 6.736)	3.824 (2.141 - 6.861)
70+ years (rate per capita)	0.609 (0.555 - 0.669)	0.607 (0.554 - 0.662)	0.605 (0.553 - 0.657)	0.724 (0.635 - 0.823)	0.720 (0.632 - 0.812)	0.716 (0.631 - 0.806)	4.556 (2.634 - 7.562)	4.540 (2.656 - 7.449)	4.532 (2.618 - 7.544)
All ages (rate per capita)	0.502 (0.474 - 0.532)	0.503 (0.477 - 0.530)	0.504 (0.479 - 0.532)	0.713 (0.631 - 0.806)	0.717 (0.638 - 0.803)	0.717 (0.638 - 0.804)	1.247 (0.700 - 2.129)	1.262 (0.708 - 2.154)	1.263 (0.711 - 2.158)
Age-standardised (rate per capita)	0.535 (0.511 - 0.565)	0.534 (0.510 - 0.557)	0.533 (0.512 - 0.558)	0.743 (0.687 - 0.801)	0.742 (0.687 - 0.802)	0.742 (0.687 - 0.802)	1.962 (1.100 - 3.367)	1.954 (1.094 - 3.357)	1.965 (1.104 - 3.427)
Total number of cases (thousands)	6,762 (6,383 - 7,153)	10,628 (10,066 - 11,199)	14,098 (13,395 - 14,898)	9,598 (8,494 - 10,852)	15,142 (13,487 - 16,961)	20,077 (17,869 - 22,493)	17 (9 - 29)	27 (15 - 45)	35 (20 - 60)
**Rwanda**									
Under-5 years (rate per capita)	0.406 (0.330 - 0.489)	0.402 (0.318 - 0.482)	0.409 (0.324 - 0.493)	0.560 (0.346 - 0.746)	0.552 (0.341 - 0.735)	0.564 (0.349 - 0.752)	0.095 (0.040 - 0.187)	0.094 (0.041 - 0.189)	0.096 (0.041 - 0.195)
5-14 years (rate per capita)	0.465 (0.397 - 0.542)	0.463 (0.395 - 0.533)	0.462 (0.389 - 0.538)	0.838 (0.592 - 1.141)	0.834 (0.588 - 1.134)	0.831 (0.586 - 1.130)	0.509 (0.287 - 0.836)	0.508 (0.287 - 0.819)	0.512 (0.292 - 0.832)
15 - 49 years (rate per capita)	0.478 (0.425 - 0.541)	0.477 (0.425 - 0.543)	0.480 (0.429 - 0.543)	0.783 (0.629 - 0.920)	0.781 (0.626 - 0.916)	0.786 (0.637 - 0.922)	1.555 (0.858 - 2.685)	1.515 (0.846 - 2.597)	1.586 (0.874 - 2.726)
50 - 69 years (rate per capita)	0.581 (0.511 - 0.653)	0.577 (0.507 - 0.651)	0.578 (0.511 - 0.651)	0.802 (0.581 - 1.006)	0.800 (0.583 - 0.997)	0.801 (0.585 - 0.996)	3.869 (2.138 - 6.877)	3.787 (2.111 - 6.841)	3.809 (2.105 - 6.855)
70+ years (rate per capita)	0.588 (0.519 - 0.666)	0.580 (0.513 - 0.653)	0.582 (0.516 - 0.650)	0.744 (0.529 - 0.963)	0.734 (0.533 - 0.937)	0.737 (0.533 - 0.942)	4.612 (2.643 - 7.612)	4.511 (2.613 - 7.484)	4.558 (2.635 - 7.501)
All ages (rate per capita)	0.468 (0.430 - 0.511)	0.470 (0.432 - 0.513)	0.475 (0.439 - 0.515)	0.756 (0.647 - 0.872)	0.757 (0.653 - 0.868)	0.765 (0.664 - 0.873)	1.143 (0.640 - 1.946)	1.226 (0.685 - 2.080)	1.315 (0.728 - 2.243)
Age-standardised (rate per capita)	0.500 (0.465 - 0.540)	0.500 (0.468 - 0.538)	0.500 (0.466 - 0.540)	0.772 (0.684 - 0.867)	0.772 (0.684 - 0.867)	0.772 (0.684 - 0.867)	1.962 (1.099 - 3.351)	1.951 (1.091 - 3.378)	1.962 (1.091 - 3.430)
Total number of cases (thousands)	3,394 (3,119 - 3,708)	4,241 (3,906 - 4,629)	5,520 (5,103 - 5,987)	5,482 (4,696 - 6,326)	6,833 (5,896 - 7,836)	8,902 (7,720 - 10,151)	8 (5 - 14)	11 (6 - 19)	15 (8 - 26)
**Somalia**									
Under-5 years (rate per capita)	0.399 (0.314 - 0.483)	0.399 (0.316 - 0.478)	0.401 (0.319 - 0.481)	0.552 (0.341 - 0.735)	0.552 (0.341 - 0.735)	0.555 (0.343 - 0.739)	0.094 (0.040 - 0.186)	0.094 (0.040 - 0.188)	0.094 (0.040 - 0.189)
5-14 years (rate per capita)	0.463 (0.388 - 0.541)	0.464 (0.394 - 0.541)	0.464 (0.389 - 0.539)	0.836 (0.590 - 1.137)	0.840 (0.595 - 1.144)	0.834 (0.588 - 1.135)	0.508 (0.292 - 0.834)	0.507 (0.291 - 0.829)	0.508 (0.292 - 0.831)
15 - 49 years (rate per capita)	0.483 (0.428 - 0.545)	0.481 (0.427 - 0.542)	0.480 (0.427 - 0.540)	0.784 (0.634 - 0.920)	0.783 (0.632 - 0.918)	0.782 (0.630 - 0.920)	1.598 (0.887 - 2.746)	1.577 (0.876 - 2.686)	1.545 (0.860 - 2.627)
50 - 69 years (rate per capita)	0.580 (0.517 - 0.655)	0.582 (0.516 - 0.660)	0.580 (0.509 - 0.655)	0.801 (0.587 - 0.998)	0.801 (0.588 - 0.998)	0.801 (0.586 - 0.999)	3.825 (2.133 - 6.778)	3.812 (2.114 - 6.816)	3.811 (2.130 - 6.714)
70+ years (rate per capita)	0.591 (0.524 - 0.667)	0.593 (0.524 - 0.667)	0.591 (0.522 - 0.662)	0.746 (0.528 - 0.972)	0.744 (0.528 - 0.965)	0.744 (0.526 - 0.965)	4.602 (2.614 - 7.597)	4.570 (2.633 - 7.514)	4.574 (2.639 - 7.447)
All ages (rate per capita)	0.471 (0.434 - 0.511)	0.470 (0.434 - 0.510)	0.470 (0.433 - 0.509)	0.752 (0.654 - 0.860)	0.755 (0.652 - 0.869)	0.755 (0.652 - 0.870)	1.260 (0.705 - 2.145)	1.204 (0.676 - 2.045)	1.198 (0.671 - 2.026)
Age-standardised (rate per capita)	0.500 (0.468 - 0.540)	0.501 (0.467 - 0.541)	0.500 (0.464 - 0.538)	0.772 (0.684 - 0.867)	0.772 (0.684 - 0.867)	0.772 (0.684 - 0.867)	1.952 (1.085 - 3.352)	1.946 (1.089 - 3.318)	1.945 (1.086 - 3.282)
Total number of cases (thousands)	3,016 (2,777 - 3,269)	3,997 (3,695 - 4,341)	5,096 (4,699 - 5,525)	4,816 (4,188 - 5,505)	6,418 (5,545 - 7,390)	8,192 (7,075 - 9,444)	8 (5 - 14)	10 (6 - 17)	13 (7 - 22)
**South Sudan**									
Under-5 years (rate per capita)	0.400 (0.316 - 0.481)	0.397 (0.318 - 0.482)	0.399 (0.316 - 0.479)	0.552 (0.341 - 0.735)	0.550 (0.340 - 0.733)	0.553 (0.342 - 0.736)	0.094 (0.040 - 0.188)	0.093 (0.040 - 0.188)	0.094 (0.040 - 0.190)
5-14 years (rate per capita)	0.461 (0.390 - 0.538)	0.462 (0.388 - 0.533)	0.461 (0.389 - 0.538)	0.832 (0.587 - 1.131)	0.833 (0.587 - 1.133)	0.830 (0.585 - 1.129)	0.508 (0.289 - 0.840)	0.508 (0.289 - 0.831)	0.508 (0.285 - 0.825)
15 - 49 years (rate per capita)	0.482 (0.432 - 0.546)	0.481 (0.431 - 0.544)	0.480 (0.428 - 0.543)	0.784 (0.635 - 0.918)	0.784 (0.633 - 0.920)	0.784 (0.633 - 0.919)	1.594 (0.883 - 2.737)	1.578 (0.872 - 2.692)	1.574 (0.872 - 2.691)
50 - 69 years (rate per capita)	0.578 (0.511 - 0.658)	0.580 (0.514 - 0.650)	0.581 (0.512 - 0.658)	0.801 (0.586 - 0.997)	0.802 (0.584 - 1.004)	0.800 (0.588 - 0.996)	3.759 (2.114 - 6.698)	3.804 (2.136 - 6.784)	3.784 (2.109 - 6.642)
70+ years (rate per capita)	0.595 (0.523 - 0.670)	0.594 (0.527 - 0.660)	0.592 (0.522 - 0.668)	0.750 (0.526 - 0.979)	0.747 (0.526 - 0.974)	0.745 (0.528 - 0.969)	4.484 (2.598 - 7.414)	4.484 (2.597 - 7.295)	4.494 (2.622 - 7.389)
All ages (rate per capita)	0.471 (0.435 - 0.513)	0.472 (0.437 - 0.513)	0.473 (0.438 - 0.510)	0.753 (0.654 - 0.860)	0.758 (0.661 - 0.867)	0.761 (0.661 - 0.868)	1.258 (0.705 - 2.145)	1.270 (0.717 - 2.145)	1.306 (0.731 - 2.201)
Age-standardised (rate per capita)	0.500 (0.466 - 0.542)	0.500 (0.467 - 0.539)	0.500 (0.467 - 0.535)	0.772 (0.684 - 0.867)	0.772 (0.684 - 0.867)	0.772 (0.684 - 0.867)	1.928 (1.085 - 3.305)	1.928 (1.086 - 3.279)	1.931 (1.073 - 3.284)
Total number of cases (thousands)	2,743 (2,531 - 2,990)	3,825 (3,538 - 4,161)	5,813 (5,381 - 6,270)	4,384 (3,806 - 5,009)	6,144 (5,354 - 7,023)	9,345 (8,123 - 10,667)	7 (4 - 12)	10 (6 - 17)	16 (9 - 27)
**Tanzania**									
Under-5 years (rate per capita)	0.249 (0.202 - 0.294)	0.152 (0.130 - 0.173)	0.190 (0.150 - 0.232)	0.460 (0.230 - 0.723)	0.194 (0.130 - 0.269)	0.342 (0.167 - 0.622)	0.058 (0.024 - 0.117)	0.035 (0.015 - 0.068)	0.044 (0.019 - 0.088)
5-14 years (rate per capita)	0.428 (0.386 - 0.470)	0.527 (0.475 - 0.577)	0.505 (0.446 - 0.565)	0.525 (0.393 - 0.664)	0.612 (0.473 - 0.776)	0.630 (0.467 - 0.829)	0.505 (0.284 - 0.810)	0.589 (0.329 - 0.976)	0.557 (0.313 - 0.913)
15 - 49 years (rate per capita)	0.542 (0.519 - 0.567)	0.548 (0.523 - 0.573)	0.549 (0.527 - 0.572)	0.729 (0.664 - 0.802)	0.729 (0.665 - 0.800)	0.730 (0.671 - 0.797)	1.349 (0.803 - 2.123)	1.349 (0.800 - 2.131)	1.370 (0.818 - 2.165)
50 - 69 years (rate per capita)	0.518 (0.486 - 0.546)	0.519 (0.487 - 0.551)	0.518 (0.484 - 0.549)	0.805 (0.714 - 0.900)	0.806 (0.714 - 0.895)	0.805 (0.708 - 0.898)	2.670 (1.624 - 4.020)	2.696 (1.649 - 4.078)	2.699 (1.646 - 4.068)

	Prevalence 1990	Prevalence 2005	Prevalence 2015	Incidence 1990	Incidence 2005	Incidence 2015	YLD rate 1990	YLD rate 2005	YLD rate 2015
70+ years (rate per capita)	0.526 (0.498 - 0.559)	0.525 (0.495 - 0.556)	0.520 (0.494 - 0.551)	0.720 (0.635 - 0.810)	0.716 (0.627 - 0.811)	0.713 (0.625 - 0.803)	3.407 (2.141 - 5.024)	3.397 (2.141 - 5.003)	3.407 (2.132 - 5.047)
All ages (rate per capita)	0.455 (0.435 - 0.476)	0.468 (0.446 - 0.488)	0.472 (0.450 - 0.494)	0.629 (0.557 - 0.708)	0.606 (0.554 - 0.666)	0.640 (0.570 - 0.726)	1.011 (0.600 - 1.575)	1.042 (0.623 - 1.621)	1.051 (0.626 - 1.638)
Age-standardised (rate per capita)	0.492 (0.476 - 0.509)	0.504 (0.485 - 0.522)	0.503 (0.486 - 0.520)	0.687 (0.634 - 0.743)	0.677 (0.634 - 0.725)	0.692 (0.641 - 0.750)	1.518 (0.917 - 2.329)	1.536 (0.926 - 2.347)	1.537 (0.925 - 2.352)
Total number of cases (thousands)	11,577 (11,069 - 12,118)	18,306 (17,459 - 19,069)	25,170 (24,002 - 26,356)	16,008 (14,172 - 18,020)	23,705 (21,659 - 26,031)	34,145 (30,428 - 38,731)	26 (15 - 40)	41 (24 - 63)	56 (33 - 87)
**Uganda**									
Under-5 years (rate per capita)	0.302 (0.257 - 0.341)	0.253 (0.231 - 0.278)	0.310 (0.261 - 0.349)	0.527 (0.290 - 0.744)	0.305 (0.264 - 0.359)	0.539 (0.296 - 0.760)	0.071 (0.030 - 0.139)	0.060 (0.026 - 0.114)	0.073 (0.032 - 0.143)
5-14 years (rate per capita)	0.456 (0.402 - 0.511)	0.421 (0.372 - 0.474)	0.455 (0.401 - 0.503)	0.679 (0.504 - 0.882)	0.633 (0.465 - 0.879)	0.677 (0.503 - 0.879)	0.485 (0.280 - 0.776)	0.478 (0.269 - 0.753)	0.488 (0.275 - 0.787)
15 - 49 years (rate per capita)	0.461 (0.434 - 0.491)	0.458 (0.428 - 0.491)	0.460 (0.431 - 0.491)	0.726 (0.655 - 0.800)	0.724 (0.653 - 0.800)	0.725 (0.655 - 0.800)	1.532 (0.857 - 2.615)	1.483 (0.836 - 2.511)	1.520 (0.845 - 2.578)
50 - 69 years (rate per capita)	0.530 (0.473 - 0.597)	0.529 (0.471 - 0.594)	0.528 (0.469 - 0.594)	0.754 (0.662 - 0.860)	0.754 (0.662 - 0.860)	0.754 (0.663 - 0.857)	3.796 (2.111 - 6.795)	3.781 (2.092 - 6.695)	3.809 (2.111 - 6.779)
70+ years (rate per capita)	0.551 (0.489 - 0.605)	0.549 (0.486 - 0.601)	0.542 (0.484 - 0.598)	0.660 (0.574 - 0.751)	0.656 (0.573 - 0.745)	0.650 (0.570 - 0.736)	4.561 (2.620 - 7.583)	4.561 (2.637 - 7.545)	4.540 (2.619 - 7.564)
All ages (rate per capita)	0.434 (0.410 - 0.456)	0.412 (0.390 - 0.434)	0.435 (0.410 - 0.456)	0.674 (0.595 - 0.764)	0.614 (0.552 - 0.689)	0.676 (0.597 - 0.769)	1.154 (0.651 - 1.975)	1.094 (0.616 - 1.848)	1.118 (0.626 - 1.903)
Age-standardised (rate per capita)	0.468 (0.444 - 0.495)	0.458 (0.433 - 0.483)	0.468 (0.445 - 0.492)	0.703 (0.649 - 0.759)	0.675 (0.627 - 0.730)	0.704 (0.648 - 0.760)	1.924 (1.079 - 3.316)	1.923 (1.075 - 3.292)	1.934 (1.077 - 3.371)
Total number of cases (thousands)	7,566 (7,137 - 7,947)	11,580 (10,957 - 12,185)	17,042 (16,067 - 17,842)	11,743 (10,360 - 13,309)	17,246 (15,514 - 19,352)	26,479 (23,382 - 30,094)	20 (11 - 34)	31 (17 - 52)	44 (25 - 75)
**Zambia**									
Under-5 years (rate per capita)	0.395 (0.315 - 0.475)	0.399 (0.321 - 0.478)	0.403 (0.328 - 0.482)	0.547 (0.338 - 0.729)	0.551 (0.340 - 0.734)	0.557 (0.344 - 0.742)	0.093 (0.040 - 0.187)	0.094 (0.041 - 0.190)	0.095 (0.040 - 0.193)
5-14 years (rate per capita)	0.462 (0.404 - 0.530)	0.457 (0.401 - 0.524)	0.458 (0.405 - 0.530)	0.733 (0.521 - 1.010)	0.729 (0.518 - 1.003)	0.730 (0.518 - 1.004)	0.505 (0.286 - 0.813)	0.502 (0.287 - 0.808)	0.501 (0.282 - 0.805)
15 - 49 years (rate per capita)	0.510 (0.482 - 0.541)	0.506 (0.477 - 0.535)	0.509 (0.480 - 0.538)	0.740 (0.673 - 0.818)	0.740 (0.671 - 0.820)	0.743 (0.675 - 0.821)	1.586 (0.878 - 2.723)	1.532 (0.854 - 2.602)	1.590 (0.879 - 2.724)
50 - 69 years (rate per capita)	0.563 (0.508 - 0.626)	0.565 (0.514 - 0.624)	0.567 (0.513 - 0.625)	0.794 (0.699 - 0.896)	0.796 (0.699 - 0.896)	0.795 (0.699 - 0.893)	3.786 (2.102 - 6.775)	3.810 (2.097 - 6.775)	3.812 (2.104 - 6.815)
70+ years (rate per capita)	0.582 (0.526 - 0.632)	0.581 (0.525 - 0.633)	0.580 (0.526 - 0.629)	0.696 (0.610 - 0.789)	0.694 (0.608 - 0.789)	0.692 (0.606 - 0.784)	4.542 (2.657 - 7.386)	4.507 (2.672 - 7.363)	4.499 (2.653 - 7.340)
All ages (rate per capita)	0.480 (0.453 - 0.505)	0.478 (0.452 - 0.504)	0.480 (0.455 - 0.510)	0.705 (0.623 - 0.796)	0.705 (0.624 - 0.792)	0.709 (0.629 - 0.795)	1.201 (0.666 - 2.061)	1.179 (0.663 - 2.029)	1.202 (0.669 - 2.074)
Age-standardised (rate per capita)	0.510 (0.486 - 0.536)	0.509 (0.486 - 0.532)	0.510 (0.486 - 0.537)	0.733 (0.677 - 0.794)	0.732 (0.678 - 0.792)	0.732 (0.678 - 0.792)	1.959 (1.094 - 3.366)	1.952 (1.101 - 3.343)	1.955 (1.085 - 3.400)
Total number of cases (thousands)	3,902 (3,685 - 4,110)	5,760 (5,451 - 6,080)	7,807 (7,396 - 8,283)	5,737 (5,068 - 6,475)	8,501 (7,528 - 9,558)	11,513 (10,219 - 12,922)	10 (5 - 17)	14 (8 - 24)	20 (11 - 34)
**Central Sub-Saharan Africa**									
Under-5 years (rate per capita)	0.342 (0.298 - 0.397)	0.345 (0.295 - 0.399)	0.347 (0.297 - 0.399)	0.509 (0.304 - 0.707)	0.513 (0.307 - 0.712)	0.517 (0.309 - 0.718)	0.081 (0.033 - 0.167)	0.081 (0.034 - 0.166)	0.082 (0.034 - 0.170)
5-14 years (rate per capita)	0.447 (0.408 - 0.491)	0.447 (0.408 - 0.490)	0.447 (0.407 - 0.490)	0.746 (0.543 - 0.992)	0.744 (0.542 - 0.990)	0.743 (0.541 - 0.988)	0.502 (0.285 - 0.797)	0.503 (0.288 - 0.806)	0.504 (0.287 - 0.810)
15 - 49 years (rate per capita)	0.510 (0.492 - 0.532)	0.510 (0.490 - 0.531)	0.510 (0.489 - 0.531)	0.724 (0.648 - 0.810)	0.723 (0.646 - 0.810)	0.723 (0.646 - 0.810)	2.052 (1.237 - 3.133)	2.033 (1.230 - 3.104)	2.037 (1.232 - 3.110)
50 - 69 years (rate per capita)	0.556 (0.533 - 0.579)	0.557 (0.535 - 0.580)	0.557 (0.534 - 0.579)	0.736 (0.645 - 0.849)	0.736 (0.644 - 0.848)	0.736 (0.645 - 0.849)	4.919 (3.063 - 7.408)	4.948 (3.069 - 7.410)	4.971 (3.069 - 7.483)
70+ years (rate per capita)	0.567 (0.546 - 0.589)	0.566 (0.546 - 0.588)	0.565 (0.546 - 0.587)	0.637 (0.546 - 0.735)	0.636 (0.545 - 0.733)	0.634 (0.544 - 0.731)	6.276 (4.050 - 9.178)	6.310 (4.068 - 9.262)	6.351 (4.111 - 9.291)
All ages (rate per capita)	0.466 (0.448 - 0.483)	0.466 (0.447 - 0.485)	0.467 (0.450 - 0.486)	0.688 (0.608 - 0.778)	0.688 (0.607 - 0.779)	0.691 (0.610 - 0.781)	1.563 (0.945 - 2.363)	1.534 (0.930 - 2.322)	1.551 (0.942 - 2.351)
Age-standardised (rate per capita)	0.500 (0.486 - 0.513)	0.500 (0.486 - 0.514)	0.500 (0.487 - 0.513)	0.705 (0.650 - 0.764)	0.705 (0.650 - 0.764)	0.705 (0.650 - 0.764)	2.507 (1.540 - 3.759)	2.516 (1.551 - 3.794)	2.529 (1.555 - 3.793)
Total number of cases (thousands)	24,712 (23,762 - 25,658)	39,061 (37,483 - 40,687)	53,617 (51,617 - 55,764)	36,491 (32,261 - 41,275)	57,742 (50,928 - 65,378)	79,253 (70,050 - 89,666)	83 (50 - 125)	129 (78 - 195)	178 (108 - 270)
**Angola**									
Under-5 years (rate per capita)	0.341 (0.277 - 0.411)	0.343 (0.273 - 0.410)	0.348 (0.282 - 0.417)	0.507 (0.303 - 0.705)	0.511 (0.305 - 0.709)	0.517 (0.309 - 0.717)	0.081 (0.034 - 0.162)	0.082 (0.034 - 0.169)	0.083 (0.035 - 0.171)
5-14 years (rate per capita)	0.448 (0.397 - 0.507)	0.448 (0.397 - 0.510)	0.448 (0.398 - 0.509)	0.745 (0.543 - 0.992)	0.747 (0.543 - 0.993)	0.745 (0.542 - 0.991)	0.502 (0.285 - 0.802)	0.503 (0.286 - 0.810)	0.504 (0.286 - 0.805)
15 - 49 years (rate per capita)	0.510 (0.484 - 0.540)	0.509 (0.485 - 0.537)	0.509 (0.483 - 0.536)	0.723 (0.646 - 0.810)	0.722 (0.645 - 0.809)	0.722 (0.644 - 0.810)	2.021 (1.226 - 3.096)	2.021 (1.219 - 3.109)	2.021 (1.214 - 3.094)
50 - 69 years (rate per capita)	0.556 (0.528 - 0.584)	0.556 (0.526 - 0.587)	0.556 (0.526 - 0.587)	0.736 (0.645 - 0.849)	0.735 (0.644 - 0.851)	0.737 (0.645 - 0.851)	4.954 (3.061 - 7.453)	4.933 (3.069 - 7.423)	4.945 (3.060 - 7.450)
70+ years (rate per capita)	0.568 (0.541 - 0.604)	0.568 (0.541 - 0.599)	0.566 (0.538 - 0.599)	0.640 (0.548 - 0.739)	0.637 (0.546 - 0.735)	0.636 (0.544 - 0.732)	6.398 (4.145 - 9.337)	6.411 (4.164 - 9.388)	6.409 (4.159 - 9.355)
All ages (rate per capita)	0.462 (0.437 - 0.486)	0.462 (0.437 - 0.488)	0.464 (0.439 - 0.489)	0.684 (0.601 - 0.777)	0.686 (0.602 - 0.780)	0.688 (0.605 - 0.782)	1.472 (0.891 - 2.239)	1.455 (0.881 - 2.220)	1.466 (0.884 - 2.240)
Age-standardised (rate per capita)	0.500 (0.482 - 0.519)	0.500 (0.482 - 0.519)	0.500 (0.482 - 0.519)	0.705 (0.650 - 0.764)	0.705 (0.650 - 0.764)	0.705 (0.650 - 0.764)	2.520 (1.549 - 3.783)	2.527 (1.561 - 3.814)	2.534 (1.558 - 3.815)
Total number of cases (thousands)	5,199 (4,916 - 5,473)	8,340 (7,884 - 8,797)	11,718 (11,084 - 12,360)	7,701 (6,767 - 8,753)	12,371 (10,863 - 14,074)	17,379 (15,280 - 19,738)	17 (10 - 25)	26 (16 - 40)	37 (22 - 57)
**Central African Republic**									
Under-5 years (rate per capita)	0.344 (0.280 - 0.413)	0.348 (0.282 - 0.417)	0.350 (0.287 - 0.419)	0.513 (0.307 - 0.712)	0.518 (0.309 - 0.719)	0.518 (0.310 - 0.719)	0.081 (0.032 - 0.165)	0.082 (0.034 - 0.169)	0.082 (0.033 - 0.168)
5-14 years (rate per capita)	0.447 (0.394 - 0.506)	0.447 (0.398 - 0.507)	0.444 (0.390 - 0.507)	0.743 (0.541 - 0.989)	0.738 (0.538 - 0.981)	0.730 (0.533 - 0.967)	0.503 (0.283 - 0.807)	0.504 (0.286 - 0.810)	0.506 (0.286 - 0.805)
15 - 49 years (rate per capita)	0.512 (0.488 - 0.540)	0.510 (0.484 - 0.539)	0.509 (0.485 - 0.539)	0.724 (0.649 - 0.809)	0.723 (0.645 - 0.810)	0.725 (0.649 - 0.811)	2.073 (1.248 - 3.176)	2.031 (1.235 - 3.113)	2.060 (1.250 - 3.136)
50 - 69 years (rate per capita)	0.558 (0.531 - 0.589)	0.557 (0.529 - 0.588)	0.557 (0.530 - 0.586)	0.736 (0.645 - 0.846)	0.735 (0.644 - 0.845)	0.736 (0.645 - 0.846)	4.979 (3.087 - 7.449)	4.976 (3.076 - 7.432)	4.975 (3.072 - 7.462)
70+ years (rate per capita)	0.565 (0.537 - 0.597)	0.564 (0.538 - 0.594)	0.563 (0.538 - 0.593)	0.635 (0.545 - 0.731)	0.634 (0.544 - 0.731)	0.633 (0.543 - 0.728)	6.335 (4.078 - 9.258)	6.317 (4.086 - 9.184)	6.314 (4.066 - 9.216)
All ages (rate per capita)	0.472 (0.450 - 0.495)	0.473 (0.452 - 0.495)	0.476 (0.454 - 0.497)	0.692 (0.616 - 0.778)	0.693 (0.619 - 0.779)	0.695 (0.623 - 0.775)	1.711 (1.039 - 2.576)	1.689 (1.029 - 2.546)	1.750 (1.066 - 2.625)
Age-standardised (rate per capita)	0.500 (0.483 - 0.519)	0.500 (0.483 - 0.519)	0.500 (0.483 - 0.518)	0.705 (0.650 - 0.764)	0.705 (0.650 - 0.764)	0.705 (0.649 - 0.764)	2.519 (1.555 - 3.775)	2.515 (1.558 - 3.792)	2.523 (1.554 - 3.803)
Total number of cases (thousands)	1,386 (1,320 - 1,454)	1,922 (1,835 - 2,012)	2,333 (2,224 - 2,438)	2,032 (1,808 - 2,285)	2,817 (2,513 - 3,165)	3,409 (3,055 - 3,800)	5 (3 - 8)	7 (4 - 10)	9 (5 - 13)
**Congo**									
Under-5 years (rate per capita)	0.348 (0.279 - 0.416)	0.346 (0.287 - 0.413)	0.350 (0.284 - 0.426)	0.518 (0.309 - 0.719)	0.515 (0.308 - 0.716)	0.521 (0.312 - 0.724)	0.082 (0.034 - 0.166)	0.082 (0.033 - 0.166)	0.083 (0.034 - 0.168)
5-14 years (rate per capita)	0.445 (0.391 - 0.505)	0.446 (0.395 - 0.508)	0.448 (0.396 - 0.512)	0.737 (0.538 - 0.979)	0.742 (0.541 - 0.987)	0.743 (0.541 - 0.988)	0.504 (0.285 - 0.806)	0.504 (0.291 - 0.805)	0.504 (0.283 - 0.810)
15 - 49 years (rate per capita)	0.509 (0.483 - 0.539)	0.509 (0.485 - 0.540)	0.512 (0.488 - 0.539)	0.723 (0.645 - 0.810)	0.725 (0.648 - 0.811)	0.726 (0.652 - 0.809)	2.033 (1.224 - 3.103)	2.059 (1.254 - 3.138)	2.105 (1.275 - 3.195)
50 - 69 years (rate per capita)	0.557 (0.528 - 0.586)	0.556 (0.527 - 0.586)	0.556 (0.528 - 0.586)	0.736 (0.645 - 0.845)	0.736 (0.645 - 0.846)	0.736 (0.645 - 0.846)	4.981 (3.056 - 7.491)	4.983 (3.069 - 7.517)	4.996 (3.086 - 7.537)
70+ years (rate per capita)	0.563 (0.539 - 0.593)	0.563 (0.537 - 0.593)	0.563 (0.537 - 0.591)	0.633 (0.543 - 0.729)	0.632 (0.543 - 0.727)	0.631 (0.542 - 0.723)	6.346 (4.114 - 9.197)	6.340 (4.086 - 9.230)	6.353 (4.119 - 9.220)
All ages (rate per capita)	0.470 (0.448 - 0.494)	0.471 (0.449 - 0.493)	0.473 (0.451 - 0.495)	0.691 (0.614 - 0.780)	0.693 (0.618 - 0.779)	0.695 (0.620 - 0.782)	1.644 (1.000 - 2.475)	1.668 (1.015 - 2.520)	1.689 (1.031 - 2.545)
Age-standardised (rate per capita)	0.500 (0.482 - 0.517)	0.500 (0.483 - 0.519)	0.500 (0.483 - 0.518)	0.705 (0.650 - 0.764)	0.705 (0.650 - 0.764)	0.705 (0.650 - 0.764)	2.524 (1.559 - 3.801)	2.526 (1.558 - 3.808)	2.533 (1.565 - 3.824)
Total number of cases (thousands)	1,126 (1,073 - 1,183)	1,657 (1,581 - 1,735)	2,189 (2,088 - 2,293)	1,657 (1,472 - 1,868)	2,439 (2,175 - 2,741)	3,218 (2,868 - 3,618)	4 (2 - 6)	6 (4 - 9)	8 (5 - 12)
**Democratic Republic of the Congo**									
Under-5 years (rate per capita)	0.342 (0.281 - 0.411)	0.345 (0.278 - 0.416)	0.346 (0.280 - 0.415)	0.509 (0.304 - 0.706)	0.513 (0.307 - 0.712)	0.517 (0.309 - 0.717)	0.081 (0.033 - 0.166)	0.081 (0.034 - 0.163)	0.082 (0.035 - 0.168)
5-14 years (rate per capita)	0.448 (0.392 - 0.506)	0.446 (0.395 - 0.507)	0.447 (0.394 - 0.501)	0.746 (0.543 - 0.993)	0.744 (0.542 - 0.990)	0.743 (0.541 - 0.988)	0.502 (0.284 - 0.799)	0.503 (0.286 - 0.805)	0.504 (0.285 - 0.811)
15 - 49 years (rate per capita)	0.510 (0.486 - 0.539)	0.510 (0.485 - 0.538)	0.510 (0.484 - 0.539)	0.724 (0.648 - 0.809)	0.723 (0.646 - 0.810)	0.723 (0.645 - 0.810)	2.060 (1.236 - 3.152)	2.031 (1.231 - 3.098)	2.033 (1.231 - 3.120)
50 - 69 years (rate per capita)	0.556 (0.526 - 0.587)	0.557 (0.527 - 0.588)	0.557 (0.528 - 0.586)	0.736 (0.645 - 0.849)	0.736 (0.645 - 0.848)	0.736 (0.644 - 0.848)	4.892 (3.049 - 7.388)	4.944 (3.071 - 7.411)	4.975 (3.070 - 7.481)
70+ years (rate per capita)	0.567 (0.539 - 0.598)	0.567 (0.540 - 0.597)	0.566 (0.541 - 0.596)	0.638 (0.546 - 0.736)	0.636 (0.545 - 0.733)	0.635 (0.544 - 0.731)	6.226 (4.007 - 9.102)	6.281 (4.047 - 9.211)	6.343 (4.093 - 9.302)
All ages (rate per capita)	0.466 (0.441 - 0.489)	0.465 (0.440 - 0.489)	0.467 (0.444 - 0.490)	0.688 (0.609 - 0.778)	0.688 (0.606 - 0.779)	0.690 (0.610 - 0.781)	1.563 (0.945 - 2.360)	1.530 (0.928 - 2.311)	1.548 (0.939 - 2.345)
Age-standardised (rate per capita)	0.500 (0.481 - 0.516)	0.500 (0.483 - 0.518)	0.500 (0.483 - 0.518)	0.705 (0.650 - 0.764)	0.705 (0.650 - 0.764)	0.705 (0.650 - 0.764)	2.499 (1.534 - 3.754)	2.512 (1.553 - 3.787)	2.527 (1.556 - 3.784)
Total number of cases (thousands)	16,368 (15,489 - 17,202)	26,183 (24,751 - 27,533)	36,146 (34,395 - 37,949)	24,179 (21,391 - 27,339)	38,721 (34,134 - 43,855)	53,454 (47,209 - 60,498)	55 (33 - 83)	86 (52 - 130)	120 (73 - 182)
**Equatorial Guinea**									
Under-5 years (rate per capita)	0.339 (0.274 - 0.402)	0.342 (0.273 - 0.415)	0.348 (0.281 - 0.419)	0.506 (0.303 - 0.703)	0.513 (0.307 - 0.713)	0.517 (0.309 - 0.718)	0.080 (0.033 - 0.163)	0.081 (0.034 - 0.162)	0.082 (0.035 - 0.166)
5-14 years (rate per capita)	0.455 (0.403 - 0.513)	0.446 (0.393 - 0.507)	0.447 (0.394 - 0.508)	0.772 (0.557 - 1.028)	0.738 (0.538 - 0.980)	0.742 (0.540 - 0.986)	0.498 (0.283 - 0.804)	0.504 (0.285 - 0.802)	0.505 (0.287 - 0.813)
15 - 49 years (rate per capita)	0.507 (0.480 - 0.536)	0.518 (0.496 - 0.544)	0.511 (0.488 - 0.538)	0.730 (0.654 - 0.822)	0.727 (0.657 - 0.804)	0.725 (0.649 - 0.811)	2.086 (1.272 - 3.177)	2.245 (1.358 - 3.434)	2.141 (1.300 - 3.287)
50 - 69 years (rate per capita)	0.560 (0.534 - 0.586)	0.555 (0.523 - 0.588)	0.553 (0.522 - 0.586)	0.736 (0.644 - 0.844)	0.732 (0.641 - 0.848)	0.738 (0.642 - 0.859)	5.099 (3.164 - 7.587)	4.976 (3.069 - 7.508)	4.929 (3.042 - 7.413)
70+ years (rate per capita)	0.565 (0.537 - 0.597)	0.562 (0.535 - 0.590)	0.559 (0.534 - 0.587)	0.635 (0.545 - 0.731)	0.630 (0.541 - 0.724)	0.626 (0.538 - 0.718)	6.374 (4.133 - 9.302)	6.383 (4.150 - 9.316)	6.423 (4.162 - 9.352)

	Prevalence 1990	Prevalence 2005	Prevalence 2015	Incidence 1990	Incidence 2005	Incidence 2015	YLD rate 1990	YLD rate 2005	YLD rate 2015
**All ages (rate per capita)**	0.472 (0.450 - 0.493)	0.476 (0.454 - 0.498)	0.477 (0.455 - 0.498)	0.695 (0.623 - 0.777)	0.695 (0.623 - 0.777)	0.697 (0.626 - 0.777)	1.800 (1.101 - 2.714)	1.765 (1.071 - 2.676)	1.836 (1.116 - 2.789)
**Age-standardised (rate per capita)**	0.500 (0.481 - 0.519)	0.500 (0.482 - 0.518)	0.500 (0.482 - 0.518)	0.706 (0.650 - 0.764)	0.705 (0.650 - 0.764)	0.705 (0.649 - 0.764)	2.541 (1.562 - 3.800)	2.558 (1.573 - 3.833)	2.566 (1.582 - 3.855)
**Total number of cases (thousands)**	178 (170 - 186)	298 (284 - 312)	403 (384 - 421)	262 (235 - 293)	435 (390 - 486)	589 (529 - 657)	1 (0 - 1)	1 (1 - 2)	2 (1 - 2)
**Gabon**									
**Under-5 years (rate per capita)**	0.345 (0.281 - 0.416)	0.351 (0.290 - 0.423)	0.351 (0.287 - 0.417)	0.518 (0.310 - 0.719)	0.521 (0.311 - 0.723)	0.523 (0.313 - 0.726)	0.082 (0.034 - 0.170)	0.083 (0.034 - 0.169)	0.083 (0.034 - 0.169)
**5-14 years (rate per capita)**	0.448 (0.394 - 0.510)	0.446 (0.393 - 0.506)	0.446 (0.395 - 0.511)	0.744 (0.542 - 0.991)	0.734 (0.535 - 0.972)	0.737 (0.537 - 0.977)	0.502 (0.286 - 0.798)	0.504 (0.284 - 0.797)	0.504 (0.287 - 0.800)
**15 - 49 years (rate per capita)**	0.512 (0.487 - 0.539)	0.511 (0.487 - 0.539)	0.511 (0.486 - 0.540)	0.723 (0.648 - 0.808)	0.724 (0.648 - 0.810)	0.727 (0.652 - 0.812)	2.096 (1.269 - 3.197)	2.059 (1.250 - 3.148)	2.115 (1.277 - 3.213)
**50 - 69 years (rate per capita)**	0.559 (0.532 - 0.588)	0.559 (0.531 - 0.589)	0.558 (0.530 - 0.586)	0.736 (0.645 - 0.845)	0.735 (0.644 - 0.842)	0.735 (0.644 - 0.845)	5.014 (3.117 - 7.525)	5.055 (3.144 - 7.596)	5.035 (3.143 - 7.541)
**70+ years (rate per capita)**	0.563 (0.535 - 0.595)	0.560 (0.534 - 0.591)	0.556 (0.532 - 0.586)	0.632 (0.543 - 0.725)	0.630 (0.541 - 0.721)	0.626 (0.539 - 0.717)	6.311 (4.105 - 9.200)	6.262 (4.079 - 9.176)	6.246 (4.072 - 9.119)
**All ages (rate per capita)**	0.477 (0.455 - 0.499)	0.478 (0.458 - 0.500)	0.480 (0.459 - 0.502)	0.693 (0.618 - 0.777)	0.695 (0.624 - 0.776)	0.698 (0.627 - 0.776)	1.882 (1.155 - 2.834)	1.837 (1.125 - 2.771)	1.881 (1.152 - 2.821)
**Age-standardised (rate per capita)**	0.500 (0.483 - 0.519)	0.500 (0.484 - 0.519)	0.500 (0.483 - 0.520)	0.705 (0.649 - 0.765)	0.706 (0.650 - 0.764)	0.706 (0.650 - 0.764)	2.521 (1.556 - 3.777)	2.526 (1.554 - 3.780)	2.536 (1.561 - 3.792)
**Total number of cases (thousands)**	454 (434 - 475)	661 (633 - 691)	828 (793 - 866)	661 (589 - 740)	960 (862 - 1,072)	1,205 (1,083 - 1,340)	2 (1 - 3)	3 (2 - 4)	3 (2 - 5)

Appendix 2, Table 2. Prevalence, incidence, and YLDs per 1,000 population (with 95% uncertainty intervals) for untreated caries in deciduous teeth, 1990 and 2015, by country and by age

	Prevalence 1990	Prevalence 2005	Prevalence 2015	Incidence 1990	Incidence 2005	Incidence 2015	YLD rate 1990	YLD rate 2005	YLD rate 2015
**Global**									
**Under-5 years (rate per capita)**	0.398 (0.314 - 0.478)	0.385 (0.304 - 0.466)	0.391 (0.305 - 0.475)	0.554 (0.375 - 0.711)	0.526 (0.357 - 0.679)	0.542 (0.362 - 0.705)	0.101 (0.043 - 0.197)	0.097 (0.042 - 0.191)	0.099 (0.042 - 0.194)
**5-14 years (rate per capita)**	0.273 (0.213 - 0.341)	0.255 (0.197 - 0.320)	0.250 (0.189 - 0.322)	0.468 (0.320 - 0.659)	0.444 (0.300 - 0.638)	0.453 (0.296 - 0.658)	0.072 (0.031 - 0.143)	0.067 (0.028 - 0.133)	0.065 (0.028 - 0.132)
**All ages (rate per capita)**	0.105 (0.088 - 0.124)	0.084 (0.071 - 0.100)	0.078 (0.064 - 0.093)	0.164 (0.125 - 0.214)	0.133 (0.101 - 0.176)	0.126 (0.094 - 0.168)	0.027 (0.012 - 0.054)	0.022 (0.009 - 0.043)	0.020 (0.009 - 0.040)
**Age-standardised (rate per capita)**	0.082 (0.069 - 0.097)	0.080 (0.067 - 0.095)	0.078 (0.064 - 0.093)	0.129 (0.098 - 0.168)	0.125 (0.096 - 0.166)	0.126 (0.094 - 0.169)	0.021 (0.009 - 0.042)	0.021 (0.009 - 0.041)	0.020 (0.009 - 0.040)
**Total number of cases (thousands)**	554,512 (469,139 - 655,413)	548,229 (461,611 - 653,372)	572,694 (475,090 - 686,991)	869,802 (664,326 - 1,137,325)	864,268 (657,102 - 1,146,866)	925,919 (695,825 - 1,238,951)	144 (62 - 285)	141 (62 - 280)	147 (63 - 291)
**High-income**									
**Under-5 years (rate per capita)**	0.198 (0.145 - 0.265)	0.183 (0.132 - 0.247)	0.207 (0.141 - 0.285)	0.359 (0.184 - 0.586)	0.333 (0.166 - 0.565)	0.404 (0.188 - 0.700)	0.075 (0.030 - 0.154)	0.070 (0.028 - 0.140)	0.079 (0.031 - 0.159)
**5-14 years (rate per capita)**	0.244 (0.201 - 0.302)	0.221 (0.179 - 0.274)	0.231 (0.179 - 0.295)	0.301 (0.210 - 0.432)	0.276 (0.190 - 0.405)	0.289 (0.192 - 0.426)	0.093 (0.039 - 0.184)	0.084 (0.035 - 0.169)	0.088 (0.036 - 0.179)
**All ages (rate per capita)**	0.047 (0.039 - 0.056)	0.038 (0.031 - 0.045)	0.037 (0.029 - 0.046)	0.066 (0.048 - 0.093)	0.053 (0.038 - 0.077)	0.055 (0.037 - 0.080)	0.018 (0.008 - 0.036)	0.014 (0.006 - 0.029)	0.014 (0.006 - 0.029)
**Age-standardised (rate per capita)**	0.060 (0.051 - 0.072)	0.056 (0.046 - 0.066)	0.059 (0.046 - 0.072)	0.085 (0.062 - 0.119)	0.079 (0.056 - 0.113)	0.086 (0.059 - 0.127)	0.023 (0.010 - 0.046)	0.021 (0.009 - 0.043)	0.022 (0.009 - 0.046)
**Total number of cases (thousands)**	42,124 (35,283 - 49,868)	37,541 (31,297 - 44,608)	39,802 (31,323 - 48,744)	58,988 (43,423 - 83,038)	52,999 (38,073 - 76,348)	58,369 (39,762 - 85,318)	16 (7 - 32)	14 (6 - 29)	15 (6 - 31)
**High-income North America**									
**Under-5 years (rate per capita)**	0.183 (0.128 - 0.249)	0.162 (0.110 - 0.231)	0.192 (0.127 - 0.268)	0.367 (0.172 - 0.620)	0.311 (0.144 - 0.589)	0.398 (0.177 - 0.719)	0.070 (0.027 - 0.143)	0.062 (0.024 - 0.127)	0.073 (0.028 - 0.150)
**5-14 years (rate per capita)**	0.182 (0.134 - 0.246)	0.154 (0.113 - 0.206)	0.190 (0.139 - 0.247)	0.283 (0.180 - 0.428)	0.238 (0.152 - 0.369)	0.294 (0.191 - 0.450)	0.069 (0.029 - 0.146)	0.059 (0.024 - 0.126)	0.072 (0.030 - 0.152)
**All ages (rate per capita)**	0.040 (0.031 - 0.050)	0.032 (0.024 - 0.041)	0.036 (0.027 - 0.045)	0.068 (0.044 - 0.098)	0.053 (0.034 - 0.081)	0.061 (0.040 - 0.093)	0.015 (0.006 - 0.031)	0.012 (0.005 - 0.025)	0.014 (0.006 - 0.028)
**Age-standardised (rate per capita)**	0.048 (0.037 - 0.060)	0.042 (0.032 - 0.054)	0.050 (0.038 - 0.062)	0.081 (0.053 - 0.118)	0.071 (0.046 - 0.108)	0.086 (0.056 - 0.131)	0.018 (0.007 - 0.037)	0.016 (0.007 - 0.034)	0.019 (0.008 - 0.039)
**Total number of cases (thousands)**	11,006 (8,491 - 13,789)	10,454 (7,966 - 13,303)	12,813 (9,778 - 16,067)	18,828 (12,222 - 27,340)	17,464 (11,227 - 26,514)	22,071 (14,383 - 33,541)	4 (2 - 9)	4 (2 - 8)	5 (2 - 10)
**Canada**									
**Under-5 years (rate per capita)**	0.208 (0.138 - 0.283)	0.161 (0.099 - 0.266)	0.209 (0.138 - 0.284)	0.432 (0.181 - 0.738)	0.252 (0.111 - 0.620)	0.434 (0.181 - 0.740)	0.079 (0.030 - 0.164)	0.061 (0.022 - 0.142)	0.079 (0.029 - 0.165)
**5-14 years (rate per capita)**	0.212 (0.155 - 0.294)	0.118 (0.078 - 0.172)	0.210 (0.153 - 0.291)	0.306 (0.185 - 0.469)	0.196 (0.106 - 0.346)	0.302 (0.183 - 0.465)	0.081 (0.033 - 0.168)	0.045 (0.018 - 0.103)	0.080 (0.032 - 0.170)
**All ages (rate per capita)**	0.043 (0.033 - 0.056)	0.023 (0.016 - 0.032)	0.032 (0.024 - 0.041)	0.071 (0.045 - 0.106)	0.037 (0.021 - 0.066)	0.053 (0.033 - 0.078)	0.016 (0.007 - 0.034)	0.009 (0.003 - 0.019)	0.012 (0.005 - 0.025)
**Age-standardised (rate per capita)**	0.055 (0.042 - 0.071)	0.036 (0.026 - 0.051)	0.055 (0.042 - 0.071)	0.091 (0.057 - 0.136)	0.059 (0.033 - 0.105)	0.092 (0.058 - 0.136)	0.021 (0.009 - 0.043)	0.014 (0.006 - 0.030)	0.021 (0.009 - 0.044)
**Total number of cases (thousands)**	1,199 (907 - 1,542)	740 (519 - 1,038)	1,146 (872 - 1,471)	1,974 (1,244 - 2,926)	1,202 (683 - 2,114)	1,903 (1,196 - 2,825)	0 (0 - 1)	0 (0 - 1)	0 (0 - 1)
**Greenland**									
**Under-5 years (rate per capita)**	0.196 (0.125 - 0.277)	0.201 (0.128 - 0.284)	0.200 (0.128 - 0.283)	0.409 (0.167 - 0.695)	0.420 (0.172 - 0.713)	0.419 (0.171 - 0.711)	0.046 (0.018 - 0.094)	0.047 (0.019 - 0.099)	0.047 (0.018 - 0.098)
**5-14 years (rate per capita)**	0.211 (0.148 - 0.287)	0.185 (0.129 - 0.255)	0.192 (0.134 - 0.263)	0.327 (0.192 - 0.513)	0.288 (0.167 - 0.451)	0.299 (0.174 - 0.469)	0.049 (0.020 - 0.107)	0.043 (0.017 - 0.095)	0.045 (0.018 - 0.098)
**All ages (rate per capita)**	0.068 (0.048 - 0.086)	0.062 (0.044 - 0.078)	0.054 (0.039 - 0.069)	0.126 (0.071 - 0.192)	0.113 (0.065 - 0.172)	0.100 (0.057 - 0.154)	0.016 (0.007 - 0.033)	0.014 (0.006 - 0.030)	0.013 (0.005 - 0.027)
**Age-standardised (rate per capita)**	0.051 (0.037 - 0.066)	0.051 (0.037 - 0.066)	0.051 (0.037 - 0.066)	0.090 (0.053 - 0.135)	0.090 (0.053 - 0.135)	0.090 (0.053 - 0.135)	0.012 (0.005 - 0.026)	0.012 (0.005 - 0.026)	0.012 (0.005 - 0.025)
**Total number of cases (thousands)**	4 (3 - 5)	3 (2 - 4)	3 (2 - 4)	7 (4 - 11)	6 (4 - 9)	5 (3 - 8)	0 (0 - 0)	0 (0 - 0)	0 (0 - 0)
**United States**									
**Under-5 years (rate per capita)**	0.181 (0.127 - 0.246)	0.162 (0.111 - 0.229)	0.190 (0.126 - 0.267)	0.360 (0.172 - 0.609)	0.316 (0.145 - 0.587)	0.395 (0.177 - 0.718)	0.069 (0.026 - 0.140)	0.062 (0.023 - 0.127)	0.072 (0.027 - 0.149)
**5-14 years (rate per capita)**	0.179 (0.132 - 0.241)	0.158 (0.116 - 0.210)	0.188 (0.138 - 0.245)	0.281 (0.180 - 0.425)	0.242 (0.157 - 0.374)	0.293 (0.191 - 0.449)	0.068 (0.028 - 0.142)	0.060 (0.025 - 0.128)	0.072 (0.029 - 0.150)
**All ages (rate per capita)**	0.039 (0.030 - 0.049)	0.033 (0.025 - 0.041)	0.036 (0.027 - 0.045)	0.067 (0.044 - 0.097)	0.055 (0.036 - 0.083)	0.062 (0.041 - 0.095)	0.015 (0.006 - 0.030)	0.012 (0.005 - 0.026)	0.014 (0.006 - 0.028)
**Age-standardised (rate per capita)**	0.047 (0.036 - 0.058)	0.043 (0.033 - 0.054)	0.049 (0.037 - 0.062)	0.080 (0.052 - 0.116)	0.072 (0.047 - 0.109)	0.086 (0.056 - 0.130)	0.018 (0.007 - 0.036)	0.016 (0.007 - 0.034)	0.019 (0.008 - 0.039)
**Total number of cases (thousands)**	9,798 (7,554 - 12,240)	9,706 (7,420 - 12,269)	11,660 (8,854 - 14,662)	16,838 (11,003 - 24,359)	16,249 (10,513 - 24,670)	20,155 (13,142 - 30,576)	4 (2 - 8)	4 (2 - 8)	4 (2 - 9)
**Australasia**									
**Under-5 years (rate per capita)**	0.134 (0.074 - 0.225)	0.108 (0.084 - 0.142)	0.113 (0.073 - 0.172)	0.283 (0.109 - 0.686)	0.176 (0.113 - 0.275)	0.217 (0.101 - 0.449)	0.051 (0.018 - 0.113)	0.041 (0.017 - 0.082)	0.043 (0.016 - 0.094)
**5-14 years (rate per capita)**	0.161 (0.109 - 0.218)	0.156 (0.134 - 0.184)	0.149 (0.100 - 0.203)	0.192 (0.110 - 0.322)	0.189 (0.125 - 0.284)	0.176 (0.103 - 0.291)	0.061 (0.024 - 0.134)	0.059 (0.025 - 0.117)	0.057 (0.022 - 0.124)
**All ages (rate per capita)**	0.034 (0.023 - 0.046)	0.028 (0.024 - 0.032)	0.025 (0.017 - 0.033)	0.049 (0.027 - 0.088)	0.037 (0.027 - 0.054)	0.035 (0.020 - 0.060)	0.013 (0.005 - 0.028)	0.011 (0.005 - 0.022)	0.009 (0.004 - 0.020)
**Age-standardised (rate per capita)**	0.040 (0.027 - 0.054)	0.037 (0.032 - 0.043)	0.036 (0.025 - 0.048)	0.059 (0.032 - 0.107)	0.049 (0.036 - 0.072)	0.050 (0.030 - 0.087)	0.015 (0.006 - 0.033)	0.014 (0.006 - 0.029)	0.014 (0.005 - 0.029)
**Total number of cases (thousands)**	686 (469 - 932)	690 (599 - 795)	711 (498 - 947)	1,002 (545 - 1,802)	907 (651 - 1,316)	997 (585 - 1,726)	0 (0 - 1)	0 (0 - 1)	0 (0 - 1)
**Australia**									
**Under-5 years (rate per capita)**	0.133 (0.073 - 0.225)	0.105 (0.082 - 0.130)	0.108 (0.070 - 0.160)	0.280 (0.107 - 0.685)	0.165 (0.115 - 0.224)	0.201 (0.097 - 0.412)	0.051 (0.018 - 0.112)	0.040 (0.017 - 0.081)	0.041 (0.015 - 0.088)
**5-14 years (rate per capita)**	0.160 (0.109 - 0.217)	0.152 (0.130 - 0.180)	0.145 (0.097 - 0.199)	0.192 (0.109 - 0.323)	0.177 (0.117 - 0.262)	0.172 (0.100 - 0.286)	0.061 (0.024 - 0.132)	0.058 (0.025 - 0.114)	0.055 (0.022 - 0.120)
**All ages (rate per capita)**	0.033 (0.023 - 0.045)	0.027 (0.024 - 0.031)	0.024 (0.017 - 0.032)	0.048 (0.026 - 0.087)	0.034 (0.025 - 0.046)	0.033 (0.019 - 0.056)	0.013 (0.005 - 0.027)	0.010 (0.004 - 0.020)	0.009 (0.004 - 0.019)
**Age-standardised (rate per capita)**	0.040 (0.027 - 0.054)	0.036 (0.032 - 0.041)	0.035 (0.025 - 0.047)	0.058 (0.031 - 0.106)	0.046 (0.034 - 0.063)	0.048 (0.029 - 0.083)	0.015 (0.006 - 0.032)	0.014 (0.006 - 0.027)	0.013 (0.005 - 0.028)
**Total number of cases (thousands)**	564 (385 - 768)	550 (482 - 629)	574 (403 - 767)	823 (445 - 1,481)	693 (508 - 944)	795 (472 - 1,371)	0 (0 - 0)	0 (0 - 0)	0 (0 - 0)
**New Zealand**									
**Under-5 years (rate per capita)**	0.140 (0.078 - 0.229)	0.121 (0.071 - 0.224)	0.142 (0.079 - 0.232)	0.295 (0.114 - 0.693)	0.225 (0.087 - 0.581)	0.299 (0.116 - 0.703)	0.053 (0.019 - 0.119)	0.046 (0.016 - 0.106)	0.054 (0.019 - 0.117)
**5-14 years (rate per capita)**	0.165 (0.112 - 0.226)	0.174 (0.139 - 0.223)	0.168 (0.114 - 0.230)	0.195 (0.113 - 0.319)	0.246 (0.147 - 0.420)	0.198 (0.115 - 0.325)	0.063 (0.025 - 0.138)	0.066 (0.028 - 0.138)	0.064 (0.025 - 0.140)
**All ages (rate per capita)**	0.036 (0.025 - 0.049)	0.034 (0.027 - 0.045)	0.030 (0.021 - 0.041)	0.053 (0.029 - 0.094)	0.052 (0.032 - 0.101)	0.044 (0.024 - 0.079)	0.014 (0.005 - 0.030)	0.013 (0.005 - 0.027)	0.011 (0.004 - 0.025)
**Age-standardised (rate per capita)**	0.042 (0.029 - 0.056)	0.042 (0.033 - 0.057)	0.042 (0.029 - 0.056)	0.061 (0.034 - 0.108)	0.064 (0.040 - 0.127)	0.061 (0.034 - 0.108)	0.016 (0.006 - 0.035)	0.016 (0.007 - 0.034)	0.016 (0.006 - 0.035)
**Total number of cases (thousands)**	122 (84 - 165)	140 (110 - 188)	137 (95 - 186)	179 (99 - 319)	213 (134 - 417)	202 (111 - 360)	0 (0 - 0)	0 (0 - 0)	0 (0 - 0)
**High-income Asia Pacific**									
**Under-5 years (rate per capita)**	0.346 (0.271 - 0.434)	0.328 (0.254 - 0.407)	0.357 (0.280 - 0.448)	0.570 (0.336 - 0.784)	0.545 (0.314 - 0.761)	0.570 (0.344 - 0.779)	0.131 (0.056 - 0.265)	0.125 (0.053 - 0.250)	0.135 (0.058 - 0.274)
**5-14 years (rate per capita)**	0.310 (0.256 - 0.377)	0.317 (0.264 - 0.389)	0.310 (0.257 - 0.379)	0.462 (0.342 - 0.642)	0.472 (0.351 - 0.647)	0.460 (0.338 - 0.633)	0.118 (0.049 - 0.239)	0.121 (0.050 - 0.247)	0.118 (0.049 - 0.241)
**All ages (rate per capita)**	0.066 (0.056 - 0.077)	0.049 (0.041 - 0.057)	0.043 (0.036 - 0.050)	0.101 (0.077 - 0.134)	0.075 (0.058 - 0.098)	0.065 (0.050 - 0.085)	0.025 (0.011 - 0.051)	0.018 (0.008 - 0.037)	0.016 (0.007 - 0.033)
**Age-standardised (rate per capita)**	0.087 (0.074 - 0.102)	0.085 (0.072 - 0.099)	0.088 (0.075 - 0.103)	0.134 (0.102 - 0.175)	0.132 (0.100 - 0.172)	0.134 (0.103 - 0.174)	0.033 (0.014 - 0.066)	0.032 (0.014 - 0.065)	0.034 (0.014 - 0.068)
**Total number of cases (thousands)**	11,093 (9,387 - 13,008)	8,628 (7,304 - 10,108)	7,835 (6,648 - 9,169)	17,103 (13,049 - 22,562)	13,305 (10,199 - 17,420)	11,951 (9,194 - 15,580)	4 (2 - 9)	3 (1 - 7)	3 (1 - 6)
**Brunei**									
**Under-5 years (rate per capita)**	0.329 (0.257 - 0.407)	0.335 (0.262 - 0.415)	0.335 (0.262 - 0.414)	0.544 (0.315 - 0.753)	0.554 (0.321 - 0.766)	0.554 (0.321 - 0.766)	0.077 (0.033 - 0.157)	0.079 (0.033 - 0.155)	0.079 (0.034 - 0.156)
**5-14 years (rate per capita)**	0.351 (0.291 - 0.428)	0.334 (0.275 - 0.408)	0.317 (0.261 - 0.389)	0.483 (0.357 - 0.669)	0.460 (0.339 - 0.641)	0.439 (0.321 - 0.613)	0.083 (0.035 - 0.168)	0.078 (0.033 - 0.157)	0.075 (0.031 - 0.152)
**All ages (rate per capita)**	0.122 (0.103 - 0.142)	0.094 (0.080 - 0.110)	0.075 (0.063 - 0.087)	0.181 (0.136 - 0.239)	0.138 (0.105 - 0.184)	0.110 (0.084 - 0.147)	0.029 (0.012 - 0.058)	0.022 (0.009 - 0.044)	0.018 (0.008 - 0.036)
**Age-standardised (rate per capita)**	0.088 (0.074 - 0.102)	0.088 (0.074 - 0.102)	0.088 (0.074 - 0.102)	0.129 (0.098 - 0.171)	0.129 (0.098 - 0.172)	0.129 (0.098 - 0.172)	0.021 (0.009 - 0.041)	0.021 (0.009 - 0.041)	0.021 (0.009 - 0.042)
**Total number of cases (thousands)**	32 (27 - 37)	34 (29 - 40)	32 (27 - 37)	47 (35 - 62)	50 (38 - 67)	47 (35 - 62)	0 (0 - 0)	0 (0 - 0)	0 (0 - 0)
**Japan**									
**Under-5 years (rate per capita)**	0.352 (0.275 - 0.446)	0.327 (0.251 - 0.408)	0.367 (0.287 - 0.468)	0.573 (0.340 - 0.787)	0.543 (0.309 - 0.764)	0.573 (0.352 - 0.780)	0.134 (0.056 - 0.269)	0.124 (0.052 - 0.251)	0.140 (0.059 - 0.281)
**5-14 years (rate per capita)**	0.301 (0.249 - 0.368)	0.316 (0.261 - 0.388)	0.306 (0.253 - 0.373)	0.458 (0.336 - 0.636)	0.475 (0.355 - 0.649)	0.462 (0.340 - 0.636)	0.114 (0.047 - 0.230)	0.120 (0.050 - 0.248)	0.116 (0.048 - 0.236)
**All ages (rate per capita)**	0.059 (0.049 - 0.069)	0.044 (0.038 - 0.052)	0.042 (0.035 - 0.049)	0.091 (0.069 - 0.120)	0.069 (0.053 - 0.090)	0.063 (0.049 - 0.083)	0.022 (0.010 - 0.045)	0.017 (0.007 - 0.034)	0.016 (0.007 - 0.032)

	Prevalence 1990	Prevalence 2005	Prevalence 2015	Incidence 1990	Incidence 2005	Incidence 2015	YLD rate 1990	YLD rate 2005	YLD rate 2015
Age-standardised (rate per capita)	0.087 (0.073 - 0.102)	0.085 (0.072 - 0.099)	0.089 (0.075 - 0.104)	0.135 (0.103 - 0.176)	0.132 (0.100 - 0.172)	0.135 (0.104 - 0.175)	0.033 (0.014 - 0.066)	0.032 (0.014 - 0.065)	0.034 (0.015 - 0.069)
Total number of cases (thousands)	7,197 (6,075 - 8,459)	5,597 (4,734 - 6,577)	5,329 (4,511 - 6,249)	11,208 (8,508 - 14,695)	8,711 (6,645 - 11,377)	8,140 (6,283 - 10,596)	3 (1 - 6)	2 (1 - 4)	2 (1 - 4)
**Singapore**									
Under-5 years (rate per capita)	0.322 (0.251 - 0.398)	0.240 (0.196 - 0.303)	0.330 (0.257 - 0.407)	0.550 (0.313 - 0.769)	0.278 (0.210 - 0.394)	0.563 (0.321 - 0.788)	0.123 (0.052 - 0.244)	0.091 (0.039 - 0.185)	0.125 (0.055 - 0.250)
5-14 years (rate per capita)	0.336 (0.280 - 0.408)	0.277 (0.221 - 0.341)	0.308 (0.255 - 0.376)	0.483 (0.360 - 0.660)	0.483 (0.332 - 0.691)	0.444 (0.328 - 0.614)	0.128 (0.053 - 0.260)	0.105 (0.044 - 0.213)	0.117 (0.049 - 0.240)
All ages (rate per capita)	0.076 (0.065 - 0.089)	0.053 (0.044 - 0.062)	0.050 (0.042 - 0.059)	0.117 (0.087 - 0.153)	0.084 (0.062 - 0.114)	0.077 (0.058 - 0.101)	0.029 (0.012 - 0.058)	0.020 (0.008 - 0.040)	0.019 (0.008 - 0.039)
Age-standardised (rate per capita)	0.087 (0.074 - 0.102)	0.071 (0.060 - 0.084)	0.087 (0.074 - 0.102)	0.133 (0.100 - 0.174)	0.111 (0.084 - 0.150)	0.133 (0.100 - 0.174)	0.033 (0.014 - 0.066)	0.027 (0.011 - 0.054)	0.033 (0.014 - 0.066)
Total number of cases (thousands)	209 (178 - 245)	187 (157 - 221)	196 (166 - 230)	320 (239 - 419)	299 (221 - 406)	300 (226 - 397)	0 (0 - 0)	0 (0 - 0)	0 (0 - 0)
**South Korea**									
Under-5 years (rate per capita)	0.335 (0.262 - 0.415)	0.339 (0.265 - 0.419)	0.334 (0.261 - 0.413)	0.565 (0.327 - 0.783)	0.571 (0.330 - 0.791)	0.563 (0.325 - 0.781)	0.127 (0.053 - 0.261)	0.129 (0.054 - 0.259)	0.127 (0.054 - 0.260)
5-14 years (rate per capita)	0.326 (0.269 - 0.399)	0.323 (0.266 - 0.396)	0.319 (0.263 - 0.392)	0.468 (0.343 - 0.651)	0.464 (0.339 - 0.647)	0.459 (0.335 - 0.641)	0.124 (0.052 - 0.254)	0.123 (0.051 - 0.248)	0.122 (0.051 - 0.245)
All ages (rate per capita)	0.085 (0.072 - 0.100)	0.059 (0.050 - 0.070)	0.045 (0.038 - 0.053)	0.128 (0.098 - 0.170)	0.090 (0.068 - 0.119)	0.069 (0.052 - 0.091)	0.032 (0.014 - 0.066)	0.023 (0.009 - 0.046)	0.017 (0.007 - 0.035)
Age-standardised (rate per capita)	0.088 (0.074 - 0.102)	0.088 (0.074 - 0.102)	0.088 (0.074 - 0.102)	0.133 (0.101 - 0.176)	0.133 (0.101 - 0.176)	0.133 (0.101 - 0.176)	0.033 (0.014 - 0.067)	0.033 (0.014 - 0.067)	0.033 (0.014 - 0.067)
Total number of cases (thousands)	3,656 (3,100 - 4,297)	2,809 (2,383 - 3,311)	2,278 (1,928 - 2,672)	5,527 (4,214 - 7,315)	4,244 (3,241 - 5,622)	3,463 (2,623 - 4,593)	1 (1 - 3)	1 (0 - 2)	1 (0 - 2)
**Western Europe**									
Under-5 years (rate per capita)	0.135 (0.094 - 0.198)	0.140 (0.097 - 0.197)	0.169 (0.102 - 0.253)	0.237 (0.120 - 0.443)	0.261 (0.126 - 0.461)	0.358 (0.146 - 0.690)	0.052 (0.020 - 0.104)	0.053 (0.021 - 0.108)	0.064 (0.023 - 0.135)
5-14 years (rate per capita)	0.260 (0.222 - 0.307)	0.240 (0.198 - 0.291)	0.239 (0.178 - 0.314)	0.245 (0.159 - 0.351)	0.238 (0.158 - 0.351)	0.231 (0.138 - 0.359)	0.099 (0.043 - 0.193)	0.091 (0.038 - 0.182)	0.091 (0.037 - 0.189)
All ages (rate per capita)	0.040 (0.035 - 0.047)	0.034 (0.029 - 0.040)	0.033 (0.025 - 0.042)	0.044 (0.032 - 0.065)	0.040 (0.028 - 0.059)	0.042 (0.026 - 0.066)	0.015 (0.007 - 0.030)	0.013 (0.005 - 0.025)	0.013 (0.005 - 0.026)
Age-standardised (rate per capita)	0.058 (0.049 - 0.067)	0.055 (0.046 - 0.064)	0.056 (0.042 - 0.071)	0.064 (0.046 - 0.094)	0.065 (0.046 - 0.096)	0.072 (0.044 - 0.113)	0.022 (0.009 - 0.043)	0.021 (0.009 - 0.041)	0.021 (0.009 - 0.044)
Total number of cases (thousands)	15,375 (13,181 - 17,856)	13,784 (11,643 - 16,214)	14,414 (10,827 - 18,070)	16,943 (12,161 - 24,707)	16,306 (11,505 - 23,969)	18,260 (11,227 - 28,455)	6 (3 - 11)	5 (2 - 10)	5 (2 - 11)
**Andorra**									
Under-5 years (rate per capita)	0.226 (0.126 - 0.363)	0.224 (0.126 - 0.360)	0.232 (0.130 - 0.374)	0.426 (0.185 - 0.694)	0.423 (0.183 - 0.690)	0.439 (0.190 - 0.715)	0.053 (0.020 - 0.114)	0.053 (0.020 - 0.114)	0.055 (0.021 - 0.118)
5-14 years (rate per capita)	0.216 (0.142 - 0.320)	0.216 (0.142 - 0.320)	0.221 (0.146 - 0.326)	0.329 (0.172 - 0.523)	0.329 (0.172 - 0.523)	0.336 (0.176 - 0.534)	0.051 (0.020 - 0.113)	0.051 (0.020 - 0.110)	0.052 (0.021 - 0.114)
All ages (rate per capita)	0.040 (0.028 - 0.055)	0.036 (0.025 - 0.049)	0.034 (0.024 - 0.047)	0.066 (0.041 - 0.097)	0.059 (0.037 - 0.086)	0.056 (0.035 - 0.082)	0.009 (0.004 - 0.020)	0.008 (0.003 - 0.017)	0.008 (0.003 - 0.017)
Age-standardised (rate per capita)	0.059 (0.041 - 0.080)	0.059 (0.041 - 0.080)	0.059 (0.041 - 0.080)	0.097 (0.061 - 0.142)	0.097 (0.060 - 0.142)	0.097 (0.061 - 0.142)	0.014 (0.006 - 0.029)	0.014 (0.006 - 0.029)	0.014 (0.006 - 0.029)
Total number of cases (thousands)	2 (1 - 3)	3 (2 - 4)	3 (2 - 4)	3 (2 - 5)	4 (3 - 6)	4 (3 - 7)	0 (0 - 0)	0 (0 - 0)	0 (0 - 0)
**Austria**									
Under-5 years (rate per capita)	0.227 (0.127 - 0.365)	0.228 (0.128 - 0.367)	0.227 (0.127 - 0.365)	0.434 (0.187 - 0.711)	0.437 (0.188 - 0.715)	0.433 (0.187 - 0.710)	0.086 (0.031 - 0.185)	0.087 (0.031 - 0.190)	0.086 (0.031 - 0.184)
5-14 years (rate per capita)	0.226 (0.149 - 0.333)	0.209 (0.137 - 0.309)	0.221 (0.146 - 0.326)	0.351 (0.183 - 0.559)	0.324 (0.168 - 0.517)	0.344 (0.179 - 0.547)	0.086 (0.034 - 0.182)	0.079 (0.032 - 0.168)	0.084 (0.034 - 0.177)
All ages (rate per capita)	0.039 (0.028 - 0.054)	0.034 (0.024 - 0.047)	0.031 (0.022 - 0.042)	0.066 (0.041 - 0.097)	0.057 (0.035 - 0.084)	0.052 (0.032 - 0.076)	0.015 (0.006 - 0.031)	0.013 (0.005 - 0.027)	0.012 (0.005 - 0.024)
Age-standardised (rate per capita)	0.059 (0.041 - 0.080)	0.059 (0.041 - 0.080)	0.059 (0.041 - 0.080)	0.098 (0.061 - 0.144)	0.098 (0.061 - 0.144)	0.098 (0.061 - 0.144)	0.022 (0.009 - 0.046)	0.022 (0.009 - 0.046)	0.022 (0.009 - 0.046)
Total number of cases (thousands)	302 (212 - 414)	281 (197 - 389)	267 (188 - 367)	504 (312 - 743)	468 (289 - 694)	447 (277 - 657)	0 (0 - 0)	0 (0 - 0)	0 (0 - 0)
**Belgium**									
Under-5 years (rate per capita)	0.090 (0.061 - 0.136)	0.072 (0.053 - 0.095)	0.135 (0.073 - 0.224)	0.136 (0.075 - 0.271)	0.109 (0.064 - 0.184)	0.283 (0.100 - 0.671)	0.034 (0.013 - 0.071)	0.028 (0.011 - 0.056)	0.052 (0.018 - 0.118)
5-14 years (rate per capita)	0.153 (0.114 - 0.202)	0.156 (0.114 - 0.208)	0.198 (0.139 - 0.269)	0.126 (0.077 - 0.211)	0.135 (0.080 - 0.231)	0.176 (0.096 - 0.304)	0.058 (0.024 - 0.125)	0.059 (0.025 - 0.124)	0.075 (0.030 - 0.162)
All ages (rate per capita)	0.024 (0.019 - 0.031)	0.022 (0.017 - 0.028)	0.029 (0.020 - 0.039)	0.023 (0.015 - 0.038)	0.022 (0.014 - 0.034)	0.035 (0.018 - 0.065)	0.009 (0.004 - 0.019)	0.008 (0.004 - 0.017)	0.011 (0.004 - 0.024)
Age-standardised (rate per capita)	0.035 (0.027 - 0.045)	0.034 (0.027 - 0.044)	0.047 (0.032 - 0.062)	0.034 (0.023 - 0.056)	0.034 (0.023 - 0.054)	0.056 (0.029 - 0.104)	0.013 (0.006 - 0.027)	0.013 (0.005 - 0.027)	0.018 (0.007 - 0.039)
Total number of cases (thousands)	238 (185 - 307)	232 (182 - 297)	326 (227 - 437)	232 (152 - 377)	228 (152 - 361)	394 (202 - 734)	0 (0 - 0)	0 (0 - 0)	0 (0 - 0)
**Cyprus**									
Under-5 years (rate per capita)	0.229 (0.128 - 0.368)	0.230 (0.129 - 0.370)	0.231 (0.130 - 0.372)	0.433 (0.187 - 0.705)	0.435 (0.188 - 0.708)	0.437 (0.189 - 0.712)	0.054 (0.020 - 0.118)	0.054 (0.021 - 0.119)	0.054 (0.021 - 0.116)
5-14 years (rate per capita)	0.216 (0.142 - 0.319)	0.202 (0.133 - 0.300)	0.219 (0.144 - 0.323)	0.329 (0.172 - 0.522)	0.308 (0.160 - 0.493)	0.333 (0.174 - 0.529)	0.051 (0.020 - 0.107)	0.047 (0.018 - 0.104)	0.051 (0.020 - 0.112)
All ages (rate per capita)	0.058 (0.041 - 0.079)	0.042 (0.029 - 0.058)	0.032 (0.022 - 0.044)	0.095 (0.059 - 0.139)	0.069 (0.043 - 0.101)	0.052 (0.032 - 0.077)	0.014 (0.006 - 0.029)	0.010 (0.004 - 0.020)	0.007 (0.003 - 0.016)
Age-standardised (rate per capita)	0.059 (0.041 - 0.080)	0.059 (0.041 - 0.080)	0.059 (0.041 - 0.080)	0.097 (0.061 - 0.142)	0.097 (0.060 - 0.142)	0.097 (0.061 - 0.142)	0.014 (0.006 - 0.029)	0.014 (0.006 - 0.029)	0.014 (0.006 - 0.029)
Total number of cases (thousands)	38 (27 - 53)	31 (22 - 43)	28 (20 - 39)	63 (40 - 93)	52 (32 - 76)	46 (29 - 69)	0 (0 - 0)	0 (0 - 0)	0 (0 - 0)
**Denmark**									
Under-5 years (rate per capita)	0.224 (0.126 - 0.360)	0.228 (0.128 - 0.367)	0.228 (0.128 - 0.367)	0.428 (0.184 - 0.702)	0.436 (0.188 - 0.714)	0.436 (0.188 - 0.715)	0.085 (0.032 - 0.182)	0.086 (0.031 - 0.190)	0.087 (0.032 - 0.190)
5-14 years (rate per capita)	0.204 (0.134 - 0.303)	0.219 (0.144 - 0.323)	0.219 (0.144 - 0.324)	0.317 (0.164 - 0.507)	0.339 (0.176 - 0.539)	0.340 (0.177 - 0.541)	0.078 (0.031 - 0.164)	0.083 (0.032 - 0.178)	0.083 (0.034 - 0.178)
All ages (rate per capita)	0.036 (0.025 - 0.049)	0.042 (0.029 - 0.057)	0.037 (0.026 - 0.051)	0.060 (0.037 - 0.088)	0.069 (0.043 - 0.102)	0.062 (0.038 - 0.091)	0.014 (0.005 - 0.028)	0.016 (0.006 - 0.033)	0.014 (0.006 - 0.029)
Age-standardised (rate per capita)	0.059 (0.041 - 0.080)	0.059 (0.041 - 0.080)	0.059 (0.041 - 0.080)	0.098 (0.061 - 0.144)	0.098 (0.061 - 0.144)	0.098 (0.061 - 0.144)	0.022 (0.009 - 0.046)	0.022 (0.009 - 0.046)	0.022 (0.009 - 0.046)
Total number of cases (thousands)	184 (129 - 254)	225 (158 - 309)	212 (149 - 291)	308 (192 - 453)	375 (233 - 553)	353 (219 - 522)	0 (0 - 0)	0 (0 - 0)	0 (0 - 0)
**Finland**									
Under-5 years (rate per capita)	0.114 (0.089 - 0.146)	0.167 (0.101 - 0.251)	0.168 (0.101 - 0.251)	0.175 (0.110 - 0.282)	0.365 (0.145 - 0.710)	0.365 (0.146 - 0.712)	0.043 (0.018 - 0.087)	0.064 (0.023 - 0.138)	0.064 (0.023 - 0.137)
5-14 years (rate per capita)	0.186 (0.137 - 0.255)	0.227 (0.157 - 0.305)	0.244 (0.168 - 0.323)	0.200 (0.123 - 0.309)	0.231 (0.141 - 0.369)	0.248 (0.152 - 0.394)	0.071 (0.029 - 0.146)	0.086 (0.035 - 0.183)	0.093 (0.037 - 0.196)
All ages (rate per capita)	0.031 (0.025 - 0.040)	0.036 (0.027 - 0.046)	0.033 (0.025 - 0.042)	0.037 (0.025 - 0.055)	0.048 (0.028 - 0.076)	0.044 (0.026 - 0.071)	0.012 (0.005 - 0.024)	0.014 (0.006 - 0.028)	0.013 (0.005 - 0.026)
Age-standardised (rate per capita)	0.042 (0.034 - 0.054)	0.056 (0.042 - 0.072)	0.057 (0.042 - 0.072)	0.050 (0.034 - 0.075)	0.075 (0.045 - 0.120)	0.075 (0.045 - 0.120)	0.016 (0.007 - 0.032)	0.022 (0.009 - 0.044)	0.022 (0.009 - 0.045)
Total number of cases (thousands)	157 (124 - 200)	190 (140 - 243)	186 (137 - 235)	185 (126 - 276)	249 (149 - 398)	246 (146 - 394)	0 (0 - 0)	0 (0 - 0)	0 (0 - 0)
**France**									
Under-5 years (rate per capita)	0.123 (0.066 - 0.242)	0.096 (0.069 - 0.135)	0.153 (0.083 - 0.248)	0.216 (0.073 - 0.635)	0.138 (0.085 - 0.223)	0.334 (0.111 - 0.701)	0.047 (0.015 - 0.112)	0.037 (0.014 - 0.074)	0.058 (0.020 - 0.131)
5-14 years (rate per capita)	0.303 (0.242 - 0.358)	0.127 (0.098 - 0.165)	0.226 (0.157 - 0.320)	0.259 (0.129 - 0.387)	0.091 (0.053 - 0.163)	0.202 (0.092 - 0.367)	0.115 (0.049 - 0.234)	0.048 (0.020 - 0.100)	0.086 (0.034 - 0.184)
All ages (rate per capita)	0.049 (0.039 - 0.062)	0.022 (0.018 - 0.027)	0.037 (0.026 - 0.050)	0.049 (0.027 - 0.090)	0.020 (0.013 - 0.031)	0.045 (0.023 - 0.074)	0.019 (0.008 - 0.038)	0.008 (0.003 - 0.016)	0.014 (0.005 - 0.029)
Age-standardised (rate per capita)	0.063 (0.050 - 0.080)	0.031 (0.025 - 0.038)	0.053 (0.037 - 0.072)	0.064 (0.035 - 0.118)	0.028 (0.019 - 0.044)	0.065 (0.034 - 0.109)	0.024 (0.010 - 0.049)	0.012 (0.005 - 0.023)	0.020 (0.008 - 0.042)
Total number of cases (thousands)	2,776 (2,209 - 3,514)	1,319 (1,074 - 1,645)	2,381 (1,665 - 3,247)	2,786 (1,538 - 5,089)	1,207 (795 - 1,883)	2,903 (1,509 - 4,850)	1 (0 - 2)	1 (0 - 1)	1 (0 - 2)
**Germany**									
Under-5 years (rate per capita)	0.108 (0.085 - 0.138)	0.105 (0.067 - 0.167)	0.146 (0.082 - 0.232)	0.159 (0.107 - 0.213)	0.174 (0.076 - 0.393)	0.305 (0.121 - 0.665)	0.041 (0.017 - 0.084)	0.040 (0.015 - 0.085)	0.056 (0.020 - 0.117)
5-14 years (rate per capita)	0.279 (0.245 - 0.315)	0.265 (0.210 - 0.319)	0.199 (0.141 - 0.265)	0.242 (0.114 - 0.393)	0.315 (0.158 - 0.523)	0.177 (0.097 - 0.295)	0.106 (0.046 - 0.208)	0.101 (0.042 - 0.205)	0.076 (0.030 - 0.161)
All ages (rate per capita)	0.035 (0.031 - 0.039)	0.031 (0.025 - 0.037)	0.023 (0.016 - 0.030)	0.034 (0.020 - 0.050)	0.039 (0.022 - 0.064)	0.028 (0.015 - 0.047)	0.013 (0.006 - 0.026)	0.012 (0.005 - 0.024)	0.009 (0.004 - 0.018)
Age-standardised (rate per capita)	0.057 (0.051 - 0.064)	0.056 (0.046 - 0.066)	0.051 (0.036 - 0.065)	0.055 (0.033 - 0.082)	0.071 (0.041 - 0.118)	0.061 (0.034 - 0.105)	0.022 (0.009 - 0.042)	0.021 (0.009 - 0.043)	0.019 (0.008 - 0.040)
Total number of cases (thousands)	2,780 (2,489 - 3,102)	2,541 (2,057 - 3,009)	1,931 (1,376 - 2,497)	2,683 (1,582 - 3,981)	3,189 (1,807 - 5,297)	2,304 (1,277 - 3,967)	1 (0 - 2)	1 (0 - 2)	1 (0 - 2)
**Greece**									
Under-5 years (rate per capita)	0.120 (0.089 - 0.170)	0.185 (0.128 - 0.251)	0.174 (0.105 - 0.256)	0.174 (0.104 - 0.287)	0.357 (0.184 - 0.631)	0.372 (0.153 - 0.735)	0.046 (0.019 - 0.094)	0.070 (0.028 - 0.148)	0.066 (0.024 - 0.144)
5-14 years (rate per capita)	0.275 (0.240 - 0.313)	0.335 (0.298 - 0.377)	0.246 (0.182 - 0.321)	0.263 (0.128 - 0.411)	0.167 (0.100 - 0.253)	0.219 (0.134 - 0.338)	0.105 (0.045 - 0.202)	0.128 (0.055 - 0.250)	0.094 (0.039 - 0.198)
All ages (rate per capita)	0.044 (0.039 - 0.050)	0.042 (0.037 - 0.047)	0.032 (0.024 - 0.040)	0.046 (0.025 - 0.069)	0.034 (0.022 - 0.053)	0.038 (0.023 - 0.062)	0.017 (0.007 - 0.033)	0.016 (0.007 - 0.032)	0.012 (0.005 - 0.025)

	Prevalence 1990	Prevalence 2005	Prevalence 2015	Incidence 1990	Incidence 2005	Incidence 2015	YLD rate 1990	YLD rate 2005	YLD rate 2015
**Age-standardised (rate per capita)**	0.060 (0.052 - 0.067)	0.075 (0.066 - 0.084)	0.058 (0.043 - 0.072)	0.062 (0.036 - 0.091)	0.062 (0.040 - 0.097)	0.071 (0.042 - 0.113)	0.023 (0.010 - 0.044)	0.029 (0.012 - 0.056)	0.022 (0.009 - 0.045)
**Total number of cases (thousands)**	449 (393 - 504)	468 (413 - 524)	350 (261 - 437)	462 (256 - 695)	373 (244 - 586)	420 (251 - 673)	0 (0 - 0)	0 (0 - 0)	0 (0 - 0)
**Iceland**									
**Under-5 years (rate per capita)**	0.227 (0.127 - 0.364)	0.226 (0.127 - 0.364)	0.229 (0.128 - 0.368)	0.433 (0.187 - 0.710)	0.432 (0.186 - 0.708)	0.437 (0.188 - 0.716)	0.086 (0.032 - 0.188)	0.086 (0.032 - 0.187)	0.087 (0.032 - 0.187)
**5-14 years (rate per capita)**	0.222 (0.146 - 0.327)	0.214 (0.141 - 0.316)	0.222 (0.146 - 0.328)	0.344 (0.179 - 0.547)	0.332 (0.172 - 0.529)	0.345 (0.180 - 0.549)	0.084 (0.033 - 0.181)	0.081 (0.032 - 0.175)	0.084 (0.034 - 0.177)
**All ages (rate per capita)**	0.056 (0.039 - 0.076)	0.048 (0.034 - 0.066)	0.044 (0.031 - 0.061)	0.093 (0.058 - 0.137)	0.080 (0.050 - 0.118)	0.074 (0.046 - 0.109)	0.021 (0.009 - 0.044)	0.018 (0.007 - 0.038)	0.017 (0.007 - 0.035)
**Age-standardised (rate per capita)**	0.059 (0.041 - 0.080)	0.059 (0.041 - 0.080)	0.059 (0.041 - 0.080)	0.098 (0.061 - 0.144)	0.098 (0.061 - 0.144)	0.098 (0.061 - 0.144)	0.022 (0.009 - 0.046)	0.022 (0.009 - 0.046)	0.022 (0.009 - 0.046)
**Total number of cases (thousands)**	14 (10 - 19)	14 (10 - 20)	14 (10 - 20)	24 (15 - 35)	24 (15 - 35)	24 (15 - 35)	0 (0 - 0)	0 (0 - 0)	0 (0 - 0)
**Ireland**									
**Under-5 years (rate per capita)**	0.138 (0.091 - 0.211)	0.165 (0.101 - 0.252)	0.169 (0.104 - 0.258)	0.241 (0.116 - 0.500)	0.350 (0.150 - 0.697)	0.359 (0.154 - 0.713)	0.052 (0.020 - 0.107)	0.063 (0.023 - 0.137)	0.064 (0.024 - 0.138)
**5-14 years (rate per capita)**	0.208 (0.152 - 0.270)	0.243 (0.179 - 0.312)	0.248 (0.182 - 0.318)	0.186 (0.112 - 0.299)	0.215 (0.128 - 0.342)	0.219 (0.131 - 0.349)	0.079 (0.033 - 0.168)	0.093 (0.038 - 0.191)	0.094 (0.039 - 0.194)
**All ages (rate per capita)**	0.051 (0.039 - 0.064)	0.045 (0.033 - 0.056)	0.047 (0.035 - 0.059)	0.054 (0.035 - 0.087)	0.055 (0.032 - 0.089)	0.057 (0.034 - 0.092)	0.019 (0.008 - 0.039)	0.017 (0.007 - 0.035)	0.018 (0.007 - 0.036)
**Age-standardised (rate per capita)**	0.049 (0.038 - 0.062)	0.057 (0.042 - 0.072)	0.057 (0.042 - 0.072)	0.054 (0.036 - 0.089)	0.069 (0.041 - 0.112)	0.069 (0.041 - 0.112)	0.019 (0.008 - 0.038)	0.022 (0.009 - 0.045)	0.022 (0.009 - 0.044)
**Total number of cases (thousands)**	178 (138 - 225)	187 (138 - 236)	225 (166 - 283)	192 (124 - 308)	229 (135 - 371)	273 (162 - 443)	0 (0 - 0)	0 (0 - 0)	0 (0 - 0)
**Israel**									
**Under-5 years (rate per capita)**	0.157 (0.108 - 0.234)	0.182 (0.111 - 0.259)	0.183 (0.112 - 0.260)	0.287 (0.144 - 0.565)	0.390 (0.158 - 0.711)	0.392 (0.159 - 0.715)	0.060 (0.023 - 0.121)	0.069 (0.026 - 0.143)	0.070 (0.027 - 0.143)
**5-14 years (rate per capita)**	0.241 (0.192 - 0.301)	0.277 (0.214 - 0.347)	0.275 (0.212 - 0.345)	0.210 (0.124 - 0.320)	0.245 (0.151 - 0.374)	0.244 (0.150 - 0.372)	0.092 (0.039 - 0.187)	0.106 (0.043 - 0.214)	0.105 (0.043 - 0.207)
**All ages (rate per capita)**	0.066 (0.055 - 0.079)	0.068 (0.053 - 0.082)	0.067 (0.053 - 0.082)	0.074 (0.048 - 0.116)	0.083 (0.052 - 0.125)	0.083 (0.052 - 0.126)	0.025 (0.011 - 0.050)	0.026 (0.011 - 0.053)	0.025 (0.011 - 0.052)
**Age-standardised (rate per capita)**	0.056 (0.047 - 0.067)	0.062 (0.049 - 0.076)	0.062 (0.049 - 0.076)	0.062 (0.041 - 0.099)	0.076 (0.048 - 0.115)	0.076 (0.048 - 0.115)	0.021 (0.009 - 0.043)	0.024 (0.010 - 0.048)	0.024 (0.010 - 0.048)
**Total number of cases (thousands)**	298 (248 - 356)	446 (351 - 542)	538 (423 - 656)	331 (217 - 523)	547 (344 - 827)	665 (417 - 1,010)	0 (0 - 0)	0 (0 - 0)	0 (0 - 0)
**Italy**									
**Under-5 years (rate per capita)**	0.121 (0.079 - 0.192)	0.097 (0.084 - 0.113)	0.146 (0.082 - 0.235)	0.217 (0.103 - 0.497)	0.140 (0.112 - 0.170)	0.306 (0.118 - 0.688)	0.046 (0.017 - 0.096)	0.037 (0.016 - 0.072)	0.056 (0.019 - 0.124)
**5-14 years (rate per capita)**	0.174 (0.131 - 0.228)	0.217 (0.184 - 0.255)	0.216 (0.156 - 0.285)	0.146 (0.082 - 0.252)	0.105 (0.078 - 0.140)	0.185 (0.107 - 0.316)	0.066 (0.027 - 0.142)	0.082 (0.035 - 0.161)	0.082 (0.033 - 0.176)
**All ages (rate per capita)**	0.026 (0.020 - 0.033)	0.025 (0.022 - 0.029)	0.026 (0.019 - 0.034)	0.028 (0.017 - 0.051)	0.016 (0.014 - 0.020)	0.030 (0.016 - 0.052)	0.010 (0.004 - 0.020)	0.010 (0.004 - 0.019)	0.010 (0.004 - 0.021)
**Age-standardised (rate per capita)**	0.044 (0.034 - 0.056)	0.047 (0.041 - 0.054)	0.049 (0.036 - 0.065)	0.047 (0.029 - 0.088)	0.031 (0.026 - 0.038)	0.059 (0.032 - 0.103)	0.017 (0.007 - 0.034)	0.018 (0.008 - 0.035)	0.019 (0.008 - 0.040)
**Total number of cases (thousands)**	1,490 (1,154 - 1,897)	1,462 (1,277 - 1,689)	1,630 (1,185 - 2,116)	1,578 (978 - 2,883)	963 (791 - 1,181)	1,868 (1,036 - 3,272)	1 (0 - 1)	1 (0 - 1)	1 (0 - 1)
**Luxembourg**									
**Under-5 years (rate per capita)**	0.225 (0.126 - 0.361)	0.228 (0.128 - 0.366)	0.225 (0.126 - 0.362)	0.430 (0.185 - 0.704)	0.436 (0.188 - 0.713)	0.430 (0.185 - 0.705)	0.085 (0.030 - 0.186)	0.087 (0.032 - 0.187)	0.086 (0.031 - 0.183)
**5-14 years (rate per capita)**	0.224 (0.148 - 0.330)	0.222 (0.146 - 0.327)	0.216 (0.143 - 0.320)	0.348 (0.181 - 0.554)	0.344 (0.179 - 0.547)	0.336 (0.175 - 0.535)	0.085 (0.033 - 0.180)	0.084 (0.033 - 0.179)	0.082 (0.033 - 0.174)
**All ages (rate per capita)**	0.038 (0.027 - 0.053)	0.041 (0.029 - 0.057)	0.037 (0.026 - 0.051)	0.064 (0.040 - 0.095)	0.069 (0.043 - 0.102)	0.062 (0.038 - 0.091)	0.015 (0.006 - 0.031)	0.016 (0.006 - 0.033)	0.014 (0.006 - 0.029)
**Age-standardised (rate per capita)**	0.059 (0.041 - 0.080)	0.059 (0.041 - 0.080)	0.059 (0.041 - 0.080)	0.098 (0.061 - 0.144)	0.098 (0.061 - 0.144)	0.099 (0.061 - 0.144)	0.022 (0.009 - 0.047)	0.022 (0.009 - 0.047)	0.022 (0.009 - 0.046)
**Total number of cases (thousands)**	15 (10 - 20)	19 (13 - 26)	21 (14 - 28)	24 (15 - 36)	31 (19 - 46)	34 (21 - 51)	0 (0 - 0)	0 (0 - 0)	0 (0 - 0)
**Malta**									
**Under-5 years (rate per capita)**	0.228 (0.128 - 0.367)	0.231 (0.130 - 0.372)	0.227 (0.127 - 0.365)	0.436 (0.188 - 0.714)	0.442 (0.190 - 0.724)	0.434 (0.187 - 0.711)	0.087 (0.032 - 0.185)	0.088 (0.031 - 0.191)	0.086 (0.032 - 0.185)
**5-14 years (rate per capita)**	0.215 (0.141 - 0.318)	0.212 (0.140 - 0.314)	0.209 (0.138 - 0.310)	0.333 (0.173 - 0.531)	0.330 (0.171 - 0.526)	0.325 (0.169 - 0.518)	0.082 (0.032 - 0.177)	0.081 (0.031 - 0.172)	0.079 (0.031 - 0.168)
**All ages (rate per capita)**	0.053 (0.037 - 0.072)	0.039 (0.027 - 0.054)	0.031 (0.022 - 0.043)	0.088 (0.055 - 0.129)	0.065 (0.040 - 0.097)	0.051 (0.032 - 0.076)	0.020 (0.008 - 0.042)	0.015 (0.006 - 0.031)	0.012 (0.005 - 0.024)
**Age-standardised (rate per capita)**	0.059 (0.041 - 0.080)	0.059 (0.041 - 0.080)	0.059 (0.041 - 0.080)	0.098 (0.061 - 0.144)	0.099 (0.061 - 0.144)	0.098 (0.061 - 0.144)	0.022 (0.009 - 0.047)	0.022 (0.009 - 0.046)	0.022 (0.009 - 0.046)
**Total number of cases (thousands)**	19 (13 - 26)	16 (11 - 22)	13 (9 - 18)	31 (20 - 46)	26 (16 - 38)	22 (13 - 32)	0 (0 - 0)	0 (0 - 0)	0 (0 - 0)
**Netherlands**									
**Under-5 years (rate per capita)**	0.151 (0.088 - 0.239)	0.158 (0.101 - 0.241)	0.153 (0.088 - 0.241)	0.319 (0.128 - 0.690)	0.331 (0.145 - 0.678)	0.321 (0.128 - 0.695)	0.058 (0.021 - 0.126)	0.060 (0.021 - 0.127)	0.058 (0.021 - 0.126)
**5-14 years (rate per capita)**	0.214 (0.151 - 0.287)	0.223 (0.160 - 0.286)	0.219 (0.155 - 0.294)	0.193 (0.115 - 0.314)	0.204 (0.122 - 0.334)	0.198 (0.118 - 0.321)	0.082 (0.033 - 0.178)	0.085 (0.035 - 0.176)	0.084 (0.033 - 0.172)
**All ages (rate per capita)**	0.035 (0.025 - 0.046)	0.037 (0.029 - 0.045)	0.033 (0.024 - 0.044)	0.043 (0.024 - 0.074)	0.045 (0.028 - 0.074)	0.039 (0.022 - 0.068)	0.013 (0.005 - 0.029)	0.014 (0.006 - 0.028)	0.013 (0.005 - 0.027)
**Age-standardised (rate per capita)**	0.051 (0.036 - 0.068)	0.053 (0.042 - 0.065)	0.051 (0.036 - 0.068)	0.063 (0.035 - 0.109)	0.065 (0.041 - 0.108)	0.063 (0.035 - 0.109)	0.020 (0.008 - 0.042)	0.020 (0.008 - 0.041)	0.020 (0.008 - 0.041)
**Total number of cases (thousands)**	523 (369 - 693)	599 (469 - 733)	573 (406 - 757)	640 (357 - 1,113)	731 (463 - 1,205)	679 (383 - 1,163)	0 (0 - 0)	0 (0 - 0)	0 (0 - 0)
**Norway**									
**Under-5 years (rate per capita)**	0.061 (0.037 - 0.090)	0.094 (0.040 - 0.180)	0.094 (0.040 - 0.180)	0.103 (0.054 - 0.161)	0.187 (0.052 - 0.487)	0.187 (0.052 - 0.486)	0.023 (0.009 - 0.049)	0.036 (0.010 - 0.089)	0.036 (0.010 - 0.090)
**5-14 years (rate per capita)**	0.126 (0.063 - 0.208)	0.141 (0.076 - 0.214)	0.137 (0.074 - 0.208)	0.178 (0.095 - 0.325)	0.189 (0.082 - 0.351)	0.184 (0.080 - 0.343)	0.048 (0.017 - 0.109)	0.053 (0.020 - 0.124)	0.052 (0.019 - 0.123)
**All ages (rate per capita)**	0.020 (0.012 - 0.029)	0.025 (0.014 - 0.036)	0.022 (0.013 - 0.032)	0.029 (0.017 - 0.048)	0.037 (0.017 - 0.067)	0.033 (0.015 - 0.059)	0.007 (0.003 - 0.016)	0.009 (0.003 - 0.021)	0.008 (0.003 - 0.018)
**Age-standardised (rate per capita)**	0.028 (0.017 - 0.041)	0.033 (0.019 - 0.048)	0.033 (0.019 - 0.048)	0.041 (0.025 - 0.067)	0.050 (0.023 - 0.091)	0.050 (0.023 - 0.091)	0.011 (0.004 - 0.023)	0.013 (0.005 - 0.028)	0.013 (0.005 - 0.027)
**Total number of cases (thousands)**	83 (51 - 124)	114 (66 - 168)	112 (65 - 165)	121 (73 - 202)	170 (80 - 308)	169 (78 - 306)	0 (0 - 0)	0 (0 - 0)	0 (0 - 0)
**Portugal**									
**Under-5 years (rate per capita)**	0.169 (0.098 - 0.260)	0.167 (0.098 - 0.258)	0.169 (0.098 - 0.260)	0.359 (0.144 - 0.715)	0.356 (0.143 - 0.710)	0.360 (0.145 - 0.717)	0.064 (0.023 - 0.139)	0.064 (0.023 - 0.136)	0.064 (0.024 - 0.140)
**5-14 years (rate per capita)**	0.218 (0.152 - 0.284)	0.235 (0.164 - 0.304)	0.246 (0.172 - 0.316)	0.195 (0.119 - 0.306)	0.210 (0.129 - 0.329)	0.220 (0.135 - 0.343)	0.083 (0.034 - 0.174)	0.090 (0.036 - 0.182)	0.094 (0.038 - 0.196)
**All ages (rate per capita)**	0.042 (0.030 - 0.053)	0.033 (0.024 - 0.042)	0.032 (0.023 - 0.041)	0.050 (0.030 - 0.082)	0.040 (0.024 - 0.066)	0.038 (0.023 - 0.060)	0.016 (0.007 - 0.033)	0.013 (0.005 - 0.026)	0.012 (0.005 - 0.026)
**Age-standardised (rate per capita)**	0.056 (0.040 - 0.071)	0.056 (0.040 - 0.071)	0.056 (0.040 - 0.071)	0.069 (0.040 - 0.113)	0.069 (0.040 - 0.113)	0.069 (0.041 - 0.113)	0.021 (0.009 - 0.043)	0.021 (0.009 - 0.044)	0.021 (0.009 - 0.045)
**Total number of cases (thousands)**	422 (300 - 532)	350 (249 - 441)	350 (249 - 439)	501 (301 - 815)	427 (252 - 700)	405 (245 - 652)	0 (0 - 0)	0 (0 - 0)	0 (0 - 0)
**Spain**									
**Under-5 years (rate per capita)**	0.109 (0.072 - 0.175)	0.174 (0.089 - 0.262)	0.182 (0.107 - 0.264)	0.141 (0.073 - 0.303)	0.346 (0.105 - 0.702)	0.393 (0.153 - 0.736)	0.041 (0.016 - 0.088)	0.066 (0.022 - 0.144)	0.069 (0.025 - 0.145)
**5-14 years (rate per capita)**	0.336 (0.274 - 0.389)	0.377 (0.300 - 0.444)	0.287 (0.214 - 0.388)	0.312 (0.203 - 0.410)	0.409 (0.241 - 0.615)	0.256 (0.143 - 0.406)	0.128 (0.056 - 0.251)	0.143 (0.062 - 0.294)	0.109 (0.044 - 0.227)
**All ages (rate per capita)**	0.055 (0.047 - 0.063)	0.044 (0.036 - 0.052)	0.037 (0.028 - 0.047)	0.053 (0.038 - 0.074)	0.056 (0.032 - 0.090)	0.043 (0.025 - 0.065)	0.021 (0.009 - 0.040)	0.017 (0.007 - 0.034)	0.014 (0.006 - 0.029)
**Age-standardised (rate per capita)**	0.070 (0.060 - 0.079)	0.081 (0.066 - 0.096)	0.062 (0.047 - 0.080)	0.068 (0.048 - 0.097)	0.103 (0.058 - 0.166)	0.076 (0.044 - 0.115)	0.026 (0.011 - 0.051)	0.031 (0.013 - 0.063)	0.023 (0.009 - 0.048)
**Total number of cases (thousands)**	2,139 (1,844 - 2,436)	1,916 (1,556 - 2,275)	1,780 (1,357 - 2,303)	2,072 (1,462 - 2,871)	2,420 (1,367 - 3,912)	2,094 (1,241 - 3,181)	1 (0 - 2)	1 (0 - 1)	1 (0 - 1)
**Sweden**									
**Under-5 years (rate per capita)**	0.111 (0.091 - 0.135)	0.132 (0.102 - 0.173)	0.189 (0.115 - 0.261)	0.139 (0.098 - 0.195)	0.208 (0.123 - 0.368)	0.396 (0.162 - 0.705)	0.042 (0.017 - 0.085)	0.050 (0.021 - 0.100)	0.072 (0.027 - 0.153)
**5-14 years (rate per capita)**	0.195 (0.152 - 0.249)	0.256 (0.202 - 0.321)	0.291 (0.240 - 0.365)	0.199 (0.112 - 0.340)	0.268 (0.160 - 0.451)	0.279 (0.185 - 0.396)	0.074 (0.030 - 0.157)	0.098 (0.041 - 0.195)	0.111 (0.046 - 0.224)
**All ages (rate per capita)**	0.030 (0.024 - 0.036)	0.038 (0.031 - 0.046)	0.042 (0.033 - 0.051)	0.032 (0.021 - 0.050)	0.043 (0.028 - 0.072)	0.053 (0.034 - 0.078)	0.011 (0.005 - 0.023)	0.014 (0.006 - 0.029)	0.016 (0.007 - 0.032)
**Age-standardised (rate per capita)**	0.044 (0.036 - 0.054)	0.060 (0.050 - 0.073)	0.063 (0.050 - 0.076)	0.047 (0.031 - 0.075)	0.069 (0.045 - 0.113)	0.080 (0.051 - 0.118)	0.017 (0.007 - 0.035)	0.023 (0.010 - 0.045)	0.024 (0.010 - 0.048)
**Total number of cases (thousands)**	252 (208 - 305)	340 (278 - 415)	411 (327 - 497)	271 (180 - 428)	390 (255 - 648)	518 (332 - 762)	0 (0 - 0)	0 (0 - 0)	0 (0 - 0)
**Switzerland**									
**Under-5 years (rate per capita)**	0.226 (0.127 - 0.364)	0.228 (0.128 - 0.367)	0.226 (0.127 - 0.363)	0.432 (0.186 - 0.708)	0.436 (0.188 - 0.714)	0.432 (0.186 - 0.707)	0.086 (0.032 - 0.186)	0.087 (0.031 - 0.191)	0.086 (0.031 - 0.190)
**5-14 years (rate per capita)**	0.222 (0.146 - 0.328)	0.212 (0.139 - 0.314)	0.217 (0.143 - 0.321)	0.345 (0.179 - 0.549)	0.329 (0.171 - 0.525)	0.337 (0.175 - 0.535)	0.084 (0.034 - 0.180)	0.081 (0.032 - 0.169)	0.082 (0.033 - 0.174)
**All ages (rate per capita)**	0.038 (0.027 - 0.052)	0.035 (0.025 - 0.049)	0.032 (0.023 - 0.044)	0.064 (0.040 - 0.094)	0.059 (0.036 - 0.087)	0.054 (0.034 - 0.079)	0.015 (0.006 - 0.030)	0.013 (0.005 - 0.028)	0.012 (0.005 - 0.025)

	Prevalence 1990	Prevalence 2005	Prevalence 2015	Incidence 1990	Incidence 2005	Incidence 2015	YLD rate 1990	YLD rate 2005	YLD rate 2015
Age-standardised (rate per capita)	0.059 (0.041 - 0.080)	0.059 (0.041 - 0.080)	0.059 (0.041 - 0.080)	0.098 (0.061 - 0.144)	0.098 (0.061 - 0.144)	0.098 (0.061 - 0.144)	0.022 (0.009 - 0.046)	0.022 (0.009 - 0.046)	0.022 (0.009 - 0.046)
Total number of cases (thousands)	256 (180 - 351)	261 (183 - 361)	266 (187 - 365)	429 (266 - 629)	436 (270 - 644)	446 (278 - 652)	0 (0 - 0)	0 (0 - 0)	0 (0 - 0)
**United Kingdom**									
Under-5 years (rate per capita)	0.181 (0.132 - 0.234)	0.196 (0.132 - 0.260)	0.203 (0.135 - 0.268)	0.354 (0.178 - 0.570)	0.412 (0.179 - 0.684)	0.436 (0.183 - 0.723)	0.069 (0.028 - 0.141)	0.075 (0.030 - 0.154)	0.078 (0.030 - 0.160)
5-14 years (rate per capita)	0.290 (0.226 - 0.361)	0.282 (0.217 - 0.356)	0.293 (0.227 - 0.368)	0.302 (0.216 - 0.428)	0.315 (0.217 - 0.460)	0.324 (0.224 - 0.471)	0.110 (0.047 - 0.219)	0.107 (0.044 - 0.216)	0.111 (0.047 - 0.224)
All ages (rate per capita)	0.048 (0.041 - 0.057)	0.046 (0.038 - 0.055)	0.043 (0.035 - 0.052)	0.061 (0.043 - 0.085)	0.062 (0.043 - 0.089)	0.061 (0.041 - 0.088)	0.018 (0.008 - 0.036)	0.017 (0.007 - 0.035)	0.016 (0.007 - 0.033)
Age-standardised (rate per capita)	0.065 (0.055 - 0.078)	0.068 (0.056 - 0.082)	0.069 (0.057 - 0.084)	0.083 (0.059 - 0.116)	0.094 (0.064 - 0.134)	0.096 (0.065 - 0.137)	0.025 (0.011 - 0.050)	0.026 (0.011 - 0.052)	0.026 (0.011 - 0.053)
Total number of cases (thousands)	2,745 (2,318 - 3,263)	2,755 (2,276 - 3,311)	2,784 (2,255 - 3,357)	3,485 (2,478 - 4,871)	3,749 (2,583 - 5,384)	3,926 (2,609 - 5,632)	1 (0 - 2)	1 (0 - 2)	1 (0 - 2)
**England**									
Under-5 years (rate per capita)	0.174 (0.122 - 0.229)	0.199 (0.129 - 0.269)	0.202 (0.132 - 0.271)	0.352 (0.165 - 0.574)	0.437 (0.181 - 0.733)	0.444 (0.182 - 0.747)	0.066 (0.027 - 0.137)	0.076 (0.029 - 0.156)	0.077 (0.029 - 0.159)
5-14 years (rate per capita)	0.285 (0.218 - 0.360)	0.281 (0.212 - 0.356)	0.289 (0.222 - 0.366)	0.305 (0.213 - 0.436)	0.309 (0.212 - 0.455)	0.318 (0.218 - 0.464)	0.108 (0.045 - 0.215)	0.107 (0.044 - 0.216)	0.110 (0.046 - 0.223)
All ages (rate per capita)	0.046 (0.038 - 0.056)	0.046 (0.037 - 0.056)	0.043 (0.035 - 0.053)	0.061 (0.042 - 0.086)	0.063 (0.043 - 0.092)	0.062 (0.040 - 0.089)	0.018 (0.007 - 0.035)	0.017 (0.007 - 0.035)	0.016 (0.007 - 0.034)
Age-standardised (rate per capita)	0.064 (0.053 - 0.077)	0.068 (0.056 - 0.082)	0.068 (0.055 - 0.083)	0.083 (0.058 - 0.118)	0.095 (0.063 - 0.136)	0.095 (0.063 - 0.138)	0.024 (0.010 - 0.049)	0.026 (0.011 - 0.052)	0.026 (0.011 - 0.053)
Total number of cases (thousands)	2,214 (1,835 - 2,670)	2,319 (1,888 - 2,803)	2,341 (1,881 - 2,842)	2,895 (2,009 - 4,107)	3,190 (2,149 - 4,620)	3,334 (2,180 - 4,824)	1 (0 - 2)	1 (0 - 2)	1 (0 - 2)
**Northern Ireland**									
Under-5 years (rate per capita)	0.242 (0.217 - 0.269)	0.293 (0.227 - 0.344)	0.294 (0.230 - 0.345)	0.391 (0.330 - 0.441)	0.547 (0.293 - 0.793)	0.546 (0.289 - 0.784)	0.092 (0.040 - 0.180)	0.112 (0.047 - 0.228)	0.112 (0.046 - 0.229)
5-14 years (rate per capita)	0.346 (0.278 - 0.411)	0.341 (0.286 - 0.408)	0.345 (0.288 - 0.410)	0.454 (0.332 - 0.636)	0.447 (0.330 - 0.646)	0.452 (0.331 - 0.653)	0.131 (0.056 - 0.260)	0.130 (0.055 - 0.260)	0.131 (0.055 - 0.264)
All ages (rate per capita)	0.076 (0.066 - 0.087)	0.066 (0.056 - 0.076)	0.060 (0.051 - 0.068)	0.106 (0.086 - 0.136)	0.097 (0.071 - 0.135)	0.089 (0.064 - 0.124)	0.029 (0.012 - 0.057)	0.025 (0.011 - 0.051)	0.023 (0.010 - 0.045)
Age-standardised (rate per capita)	0.081 (0.070 - 0.092)	0.087 (0.074 - 0.100)	0.087 (0.075 - 0.100)	0.112 (0.091 - 0.144)	0.129 (0.094 - 0.178)	0.129 (0.094 - 0.179)	0.031 (0.013 - 0.060)	0.033 (0.014 - 0.067)	0.033 (0.014 - 0.066)
Total number of cases (thousands)	122 (105 - 138)	114 (97 - 132)	109 (93 - 125)	170 (137 - 218)	168 (123 - 233)	163 (118 - 227)	0 (0 - 0)	0 (0 - 0)	0 (0 - 0)
**Scotland**									
Under-5 years (rate per capita)	0.196 (0.156 - 0.257)	0.126 (0.104 - 0.155)	0.162 (0.123 - 0.205)	0.286 (0.183 - 0.469)	0.170 (0.112 - 0.282)	0.273 (0.144 - 0.444)	0.075 (0.030 - 0.150)	0.048 (0.020 - 0.098)	0.062 (0.025 - 0.124)
5-14 years (rate per capita)	0.293 (0.254 - 0.339)	0.273 (0.216 - 0.353)	0.298 (0.241 - 0.377)	0.193 (0.157 - 0.253)	0.331 (0.223 - 0.507)	0.335 (0.236 - 0.483)	0.111 (0.049 - 0.213)	0.103 (0.043 - 0.217)	0.113 (0.048 - 0.227)
All ages (rate per capita)	0.049 (0.044 - 0.055)	0.038 (0.032 - 0.047)	0.037 (0.031 - 0.045)	0.042 (0.034 - 0.059)	0.047 (0.034 - 0.068)	0.047 (0.034 - 0.064)	0.019 (0.008 - 0.036)	0.015 (0.006 - 0.030)	0.014 (0.006 - 0.028)
Age-standardised (rate per capita)	0.067 (0.060 - 0.076)	0.061 (0.051 - 0.075)	0.068 (0.056 - 0.082)	0.059 (0.047 - 0.082)	0.076 (0.055 - 0.106)	0.084 (0.062 - 0.115)	0.026 (0.011 - 0.050)	0.023 (0.010 - 0.047)	0.026 (0.011 - 0.051)
Total number of cases (thousands)	249 (221 - 281)	195 (162 - 241)	198 (163 - 242)	215 (172 - 300)	241 (174 - 344)	248 (182 - 342)	0 (0 - 0)	0 (0 - 0)	0 (0 - 0)
**Wales**									
Under-5 years (rate per capita)	0.231 (0.161 - 0.294)	0.181 (0.133 - 0.245)	0.231 (0.157 - 0.297)	0.474 (0.222 - 0.753)	0.275 (0.158 - 0.512)	0.470 (0.211 - 0.756)	0.088 (0.035 - 0.183)	0.069 (0.027 - 0.138)	0.088 (0.034 - 0.180)
5-14 years (rate per capita)	0.328 (0.264 - 0.406)	0.270 (0.215 - 0.342)	0.308 (0.252 - 0.377)	0.326 (0.225 - 0.455)	0.293 (0.198 - 0.426)	0.321 (0.224 - 0.456)	0.124 (0.051 - 0.249)	0.102 (0.043 - 0.210)	0.117 (0.050 - 0.234)
All ages (rate per capita)	0.056 (0.047 - 0.067)	0.043 (0.036 - 0.052)	0.044 (0.036 - 0.052)	0.072 (0.049 - 0.099)	0.051 (0.037 - 0.073)	0.059 (0.039 - 0.081)	0.021 (0.009 - 0.043)	0.016 (0.007 - 0.033)	0.017 (0.007 - 0.033)
Age-standardised (rate per capita)	0.076 (0.064 - 0.091)	0.065 (0.055 - 0.079)	0.075 (0.062 - 0.089)	0.098 (0.067 - 0.135)	0.078 (0.056 - 0.112)	0.099 (0.067 - 0.135)	0.029 (0.012 - 0.058)	0.025 (0.011 - 0.050)	0.029 (0.012 - 0.057)
Total number of cases (thousands)	161 (134 - 192)	127 (106 - 155)	135 (111 - 160)	206 (141 - 283)	150 (108 - 216)	180 (121 - 248)	0 (0 - 0)	0 (0 - 0)	0 (0 - 0)
**Southern Latin America**									
Under-5 years (rate per capita)	0.256 (0.189 - 0.332)	0.241 (0.161 - 0.330)	0.241 (0.164 - 0.328)	0.470 (0.224 - 0.721)	0.435 (0.189 - 0.721)	0.437 (0.193 - 0.720)	0.098 (0.039 - 0.199)	0.092 (0.035 - 0.191)	0.092 (0.035 - 0.193)
5-14 years (rate per capita)	0.277 (0.209 - 0.360)	0.273 (0.212 - 0.355)	0.275 (0.207 - 0.360)	0.284 (0.157 - 0.469)	0.277 (0.154 - 0.456)	0.280 (0.155 - 0.462)	0.105 (0.043 - 0.213)	0.103 (0.043 - 0.210)	0.104 (0.043 - 0.210)
All ages (rate per capita)	0.081 (0.065 - 0.099)	0.068 (0.054 - 0.084)	0.062 (0.049 - 0.077)	0.104 (0.069 - 0.152)	0.086 (0.056 - 0.129)	0.079 (0.051 - 0.118)	0.031 (0.013 - 0.062)	0.026 (0.011 - 0.052)	0.024 (0.010 - 0.048)
Age-standardised (rate per capita)	0.071 (0.057 - 0.087)	0.069 (0.055 - 0.086)	0.069 (0.055 - 0.086)	0.092 (0.061 - 0.133)	0.088 (0.057 - 0.132)	0.088 (0.057 - 0.132)	0.027 (0.011 - 0.055)	0.026 (0.011 - 0.053)	0.026 (0.011 - 0.053)
Total number of cases (thousands)	3,964 (3,194 - 4,858)	3,985 (3,167 - 4,946)	4,029 (3,193 - 5,019)	5,110 (3,391 - 7,438)	5,018 (3,275 - 7,559)	5,089 (3,306 - 7,649)	2 (1 - 3)	2 (1 - 3)	2 (1 - 3)
**Argentina**									
Under-5 years (rate per capita)	0.227 (0.150 - 0.315)	0.227 (0.147 - 0.315)	0.229 (0.151 - 0.316)	0.427 (0.179 - 0.720)	0.426 (0.173 - 0.720)	0.429 (0.180 - 0.723)	0.087 (0.033 - 0.180)	0.086 (0.033 - 0.179)	0.087 (0.033 - 0.184)
5-14 years (rate per capita)	0.265 (0.195 - 0.351)	0.266 (0.208 - 0.350)	0.269 (0.198 - 0.355)	0.268 (0.145 - 0.447)	0.267 (0.146 - 0.443)	0.272 (0.147 - 0.452)	0.101 (0.041 - 0.206)	0.101 (0.042 - 0.206)	0.102 (0.041 - 0.208)
All ages (rate per capita)	0.077 (0.060 - 0.098)	0.068 (0.054 - 0.085)	0.064 (0.050 - 0.081)	0.099 (0.063 - 0.149)	0.086 (0.055 - 0.132)	0.082 (0.052 - 0.124)	0.029 (0.012 - 0.059)	0.026 (0.011 - 0.052)	0.024 (0.010 - 0.049)
Age-standardised (rate per capita)	0.067 (0.052 - 0.084)	0.067 (0.053 - 0.083)	0.067 (0.052 - 0.084)	0.085 (0.055 - 0.129)	0.085 (0.054 - 0.129)	0.085 (0.055 - 0.129)	0.025 (0.010 - 0.051)	0.025 (0.011 - 0.051)	0.025 (0.010 - 0.051)
Total number of cases (thousands)	2,527 (1,975 - 3,193)	2,663 (2,101 - 3,311)	2,792 (2,183 - 3,521)	3,226 (2,059 - 4,877)	3,378 (2,147 - 5,151)	3,554 (2,273 - 5,366)	1 (0 - 2)	1 (0 - 2)	1 (0 - 2)
**Chile**									
Under-5 years (rate per capita)	0.327 (0.267 - 0.385)	0.278 (0.196 - 0.368)	0.278 (0.195 - 0.368)	0.582 (0.334 - 0.763)	0.463 (0.226 - 0.723)	0.461 (0.225 - 0.722)	0.125 (0.053 - 0.252)	0.106 (0.042 - 0.215)	0.106 (0.042 - 0.215)
5-14 years (rate per capita)	0.313 (0.241 - 0.394)	0.290 (0.221 - 0.372)	0.293 (0.224 - 0.375)	0.323 (0.183 - 0.537)	0.299 (0.167 - 0.497)	0.301 (0.169 - 0.500)	0.119 (0.050 - 0.238)	0.110 (0.046 - 0.224)	0.111 (0.047 - 0.223)
All ages (rate per capita)	0.093 (0.078 - 0.110)	0.069 (0.055 - 0.084)	0.058 (0.046 - 0.071)	0.122 (0.083 - 0.167)	0.085 (0.055 - 0.125)	0.071 (0.047 - 0.105)	0.035 (0.015 - 0.071)	0.026 (0.011 - 0.053)	0.022 (0.009 - 0.044)
Age-standardised (rate per capita)	0.083 (0.069 - 0.097)	0.077 (0.061 - 0.093)	0.077 (0.061 - 0.093)	0.107 (0.073 - 0.148)	0.094 (0.062 - 0.138)	0.094 (0.062 - 0.138)	0.031 (0.013 - 0.063)	0.029 (0.012 - 0.059)	0.029 (0.012 - 0.057)
Total number of cases (thousands)	1,225 (1,023 - 1,444)	1,111 (889 - 1,356)	1,041 (833 - 1,269)	1,605 (1,090 - 2,192)	1,362 (893 - 2,018)	1,277 (840 - 1,886)	0 (0 - 1)	0 (0 - 1)	0 (0 - 1)
**Uruguay**									
Under-5 years (rate per capita)	0.255 (0.170 - 0.368)	0.258 (0.172 - 0.372)	0.257 (0.172 - 0.371)	0.438 (0.208 - 0.709)	0.442 (0.210 - 0.715)	0.441 (0.210 - 0.714)	0.097 (0.039 - 0.205)	0.098 (0.039 - 0.210)	0.098 (0.037 - 0.211)
5-14 years (rate per capita)	0.264 (0.195 - 0.353)	0.269 (0.198 - 0.359)	0.270 (0.199 - 0.360)	0.298 (0.158 - 0.508)	0.303 (0.161 - 0.514)	0.303 (0.161 - 0.515)	0.100 (0.041 - 0.203)	0.102 (0.041 - 0.211)	0.102 (0.042 - 0.211)
All ages (rate per capita)	0.068 (0.052 - 0.086)	0.063 (0.049 - 0.080)	0.057 (0.044 - 0.072)	0.090 (0.058 - 0.136)	0.083 (0.054 - 0.126)	0.075 (0.048 - 0.114)	0.026 (0.011 - 0.054)	0.024 (0.010 - 0.049)	0.022 (0.009 - 0.045)
Age-standardised (rate per capita)	0.070 (0.054 - 0.088)	0.070 (0.054 - 0.088)	0.070 (0.054 - 0.088)	0.092 (0.060 - 0.140)	0.092 (0.060 - 0.140)	0.092 (0.060 - 0.140)	0.027 (0.011 - 0.056)	0.027 (0.011 - 0.054)	0.027 (0.011 - 0.055)
Total number of cases (thousands)	212 (163 - 267)	211 (163 - 266)	196 (151 - 247)	279 (181 - 425)	277 (179 - 421)	257 (166 - 391)	0 (0 - 0)	0 (0 - 0)	0 (0 - 0)
**Central Europe, Eastern Europe, and Central Asia**									
Under-5 years (rate per capita)	0.425 (0.342 - 0.509)	0.426 (0.346 - 0.510)	0.426 (0.343 - 0.511)	0.608 (0.422 - 0.778)	0.619 (0.429 - 0.786)	0.618 (0.427 - 0.791)	0.141 (0.061 - 0.278)	0.139 (0.060 - 0.272)	0.140 (0.060 - 0.273)
5-14 years (rate per capita)	0.339 (0.284 - 0.408)	0.304 (0.254 - 0.368)	0.353 (0.291 - 0.426)	0.488 (0.366 - 0.662)	0.436 (0.321 - 0.599)	0.508 (0.382 - 0.684)	0.116 (0.049 - 0.231)	0.101 (0.043 - 0.201)	0.118 (0.050 - 0.238)
All ages (rate per capita)	0.092 (0.080 - 0.106)	0.062 (0.054 - 0.072)	0.066 (0.057 - 0.076)	0.132 (0.105 - 0.169)	0.090 (0.070 - 0.115)	0.095 (0.075 - 0.119)	0.031 (0.013 - 0.062)	0.021 (0.009 - 0.040)	0.022 (0.009 - 0.042)
Age-standardised (rate per capita)	0.095 (0.083 - 0.109)	0.096 (0.083 - 0.110)	0.095 (0.082 - 0.110)	0.137 (0.108 - 0.175)	0.138 (0.109 - 0.174)	0.138 (0.109 - 0.175)	0.032 (0.014 - 0.064)	0.032 (0.014 - 0.061)	0.032 (0.014 - 0.062)
Total number of cases (thousands)	38,082 (33,033 - 43,767)	25,326 (21,882 - 29,144)	27,473 (23,705 - 31,731)	54,639 (43,239 - 69,901)	36,465 (28,551 - 46,534)	39,656 (31,248 - 49,864)	13 (6 - 25)	8 (4 - 16)	9 (4 - 18)
**Eastern Europe**									
Under-5 years (rate per capita)	0.367 (0.286 - 0.471)	0.366 (0.284 - 0.473)	0.370 (0.289 - 0.482)	0.568 (0.358 - 0.774)	0.590 (0.363 - 0.780)	0.592 (0.366 - 0.791)	0.137 (0.058 - 0.275)	0.137 (0.058 - 0.275)	0.138 (0.057 - 0.280)
5-14 years (rate per capita)	0.335 (0.279 - 0.403)	0.301 (0.254 - 0.363)	0.370 (0.308 - 0.445)	0.481 (0.362 - 0.658)	0.416 (0.314 - 0.573)	0.517 (0.396 - 0.698)	0.125 (0.052 - 0.252)	0.112 (0.048 - 0.225)	0.139 (0.058 - 0.281)
All ages (rate per capita)	0.077 (0.067 - 0.092)	0.049 (0.042 - 0.057)	0.054 (0.046 - 0.064)	0.114 (0.090 - 0.149)	0.071 (0.055 - 0.090)	0.080 (0.062 - 0.103)	0.029 (0.012 - 0.059)	0.018 (0.008 - 0.036)	0.020 (0.009 - 0.041)
Age-standardised (rate per capita)	0.089 (0.077 - 0.105)	0.092 (0.079 - 0.107)	0.091 (0.078 - 0.107)	0.131 (0.103 - 0.171)	0.134 (0.104 - 0.169)	0.134 (0.104 - 0.172)	0.033 (0.014 - 0.067)	0.034 (0.015 - 0.068)	0.034 (0.014 - 0.068)
Total number of cases (thousands)	17,135 (14,835 - 20,270)	10,247 (8,802 - 12,026)	11,675 (9,994 - 13,718)	25,249 (19,808 - 32,977)	15,005 (11,609 - 19,084)	17,248 (13,310 - 22,059)	6 (3 - 13)	4 (2 - 8)	4 (2 - 9)
**Belarus**									
Under-5 years (rate per capita)	0.406 (0.305 - 0.524)	0.396 (0.297 - 0.512)	0.399 (0.299 - 0.515)	0.594 (0.381 - 0.769)	0.580 (0.372 - 0.750)	0.584 (0.375 - 0.756)	0.095 (0.040 - 0.190)	0.093 (0.039 - 0.188)	0.094 (0.040 - 0.187)
5-14 years (rate per capita)	0.347 (0.289 - 0.418)	0.294 (0.243 - 0.358)	0.341 (0.284 - 0.411)	0.464 (0.349 - 0.634)	0.397 (0.293 - 0.548)	0.455 (0.342 - 0.621)	0.082 (0.034 - 0.164)	0.069 (0.028 - 0.139)	0.080 (0.033 - 0.164)
All ages (rate per capita)	0.083 (0.071 - 0.098)	0.050 (0.043 - 0.060)	0.052 (0.044 - 0.062)	0.115 (0.090 - 0.146)	0.070 (0.055 - 0.089)	0.073 (0.057 - 0.092)	0.020 (0.008 - 0.039)	0.012 (0.005 - 0.025)	0.012 (0.005 - 0.025)

	Prevalence 1990	Prevalence 2005	Prevalence 2015	Incidence 1990	Incidence 2005	Incidence 2015	YLD rate 1990	YLD rate 2005	YLD rate 2015
Age-standardised (rate per capita)	0.094 (0.079 - 0.111)	0.094 (0.079 - 0.111)	0.094 (0.079 - 0.111)	0.130 (0.102 - 0.165)	0.130 (0.102 - 0.165)	0.130 (0.102 - 0.165)	0.022 (0.009 - 0.044)	0.022 (0.009 - 0.045)	0.022 (0.009 - 0.045)
Total number of cases (thousands)	843 (716 - 992)	488 (414 - 578)	504 (423 - 598)	1,165 (916 - 1,481)	679 (533 - 865)	702 (551 - 885)	0 (0 - 0)	0 (0 - 0)	0 (0 - 0)
**Estonia**									
Under-5 years (rate per capita)	0.407 (0.305 - 0.525)	0.394 (0.295 - 0.508)	0.402 (0.302 - 0.520)	0.612 (0.388 - 0.801)	0.592 (0.375 - 0.775)	0.605 (0.384 - 0.792)	0.155 (0.064 - 0.320)	0.150 (0.062 - 0.309)	0.153 (0.062 - 0.313)
5-14 years (rate per capita)	0.342 (0.285 - 0.412)	0.298 (0.246 - 0.362)	0.370 (0.309 - 0.444)	0.476 (0.358 - 0.645)	0.418 (0.309 - 0.576)	0.513 (0.389 - 0.692)	0.130 (0.054 - 0.264)	0.113 (0.047 - 0.235)	0.141 (0.060 - 0.288)
All ages (rate per capita)	0.080 (0.068 - 0.094)	0.050 (0.042 - 0.060)	0.057 (0.048 - 0.067)	0.114 (0.089 - 0.145)	0.073 (0.057 - 0.093)	0.081 (0.063 - 0.102)	0.030 (0.013 - 0.060)	0.019 (0.008 - 0.038)	0.022 (0.009 - 0.043)
Age-standardised (rate per capita)	0.094 (0.079 - 0.111)	0.094 (0.079 - 0.111)	0.094 (0.079 - 0.111)	0.135 (0.105 - 0.170)	0.135 (0.105 - 0.170)	0.135 (0.105 - 0.170)	0.036 (0.015 - 0.071)	0.036 (0.015 - 0.072)	0.036 (0.015 - 0.071)
Total number of cases (thousands)	125 (106 - 147)	68 (57 - 80)	77 (65 - 90)	179 (139 - 226)	98 (76 - 125)	110 (86 - 139)	0 (0 - 0)	0 (0 - 0)	0 (0 - 0)
**Latvia**									
Under-5 years (rate per capita)	0.372 (0.307 - 0.471)	0.383 (0.298 - 0.489)	0.391 (0.305 - 0.499)	0.565 (0.493 - 0.675)	0.592 (0.374 - 0.786)	0.604 (0.382 - 0.803)	0.141 (0.060 - 0.275)	0.146 (0.060 - 0.291)	0.149 (0.062 - 0.301)
5-14 years (rate per capita)	0.347 (0.285 - 0.412)	0.279 (0.231 - 0.338)	0.379 (0.318 - 0.450)	0.486 (0.365 - 0.658)	0.395 (0.294 - 0.550)	0.528 (0.403 - 0.710)	0.132 (0.055 - 0.265)	0.106 (0.045 - 0.219)	0.144 (0.060 - 0.295)
All ages (rate per capita)	0.075 (0.064 - 0.086)	0.045 (0.038 - 0.053)	0.050 (0.043 - 0.059)	0.108 (0.090 - 0.132)	0.066 (0.051 - 0.085)	0.073 (0.057 - 0.093)	0.028 (0.012 - 0.056)	0.017 (0.007 - 0.034)	0.019 (0.008 - 0.038)
Age-standardised (rate per capita)	0.091 (0.078 - 0.105)	0.093 (0.080 - 0.109)	0.093 (0.080 - 0.109)	0.132 (0.110 - 0.161)	0.135 (0.106 - 0.173)	0.135 (0.106 - 0.173)	0.035 (0.015 - 0.068)	0.035 (0.015 - 0.071)	0.035 (0.015 - 0.071)
Total number of cases (thousands)	198 (170 - 228)	103 (88 - 123)	111 (95 - 130)	286 (239 - 350)	151 (118 - 195)	161 (126 - 205)	0 (0 - 0)	0 (0 - 0)	0 (0 - 0)
**Lithuania**									
Under-5 years (rate per capita)	0.398 (0.311 - 0.509)	0.365 (0.307 - 0.445)	0.406 (0.332 - 0.503)	0.604 (0.386 - 0.800)	0.578 (0.438 - 0.733)	0.641 (0.480 - 0.801)	0.152 (0.064 - 0.302)	0.139 (0.060 - 0.270)	0.155 (0.066 - 0.301)
5-14 years (rate per capita)	0.346 (0.288 - 0.410)	0.329 (0.288 - 0.382)	0.334 (0.287 - 0.390)	0.482 (0.368 - 0.659)	0.405 (0.301 - 0.572)	0.442 (0.337 - 0.603)	0.132 (0.056 - 0.269)	0.125 (0.053 - 0.250)	0.127 (0.055 - 0.250)
All ages (rate per capita)	0.081 (0.070 - 0.095)	0.057 (0.051 - 0.064)	0.049 (0.043 - 0.056)	0.117 (0.091 - 0.149)	0.076 (0.059 - 0.101)	0.070 (0.055 - 0.087)	0.031 (0.013 - 0.062)	0.022 (0.009 - 0.042)	0.019 (0.008 - 0.036)
Age-standardised (rate per capita)	0.094 (0.080 - 0.110)	0.097 (0.088 - 0.109)	0.098 (0.087 - 0.112)	0.135 (0.105 - 0.172)	0.131 (0.103 - 0.169)	0.138 (0.111 - 0.172)	0.036 (0.015 - 0.071)	0.037 (0.016 - 0.073)	0.037 (0.016 - 0.073)
Total number of cases (thousands)	299 (256 - 350)	194 (175 - 220)	154 (136 - 177)	431 (336 - 548)	260 (202 - 343)	219 (175 - 273)	0 (0 - 0)	0 (0 - 0)	0 (0 - 0)
**Moldova**									
Under-5 years (rate per capita)	0.405 (0.304 - 0.524)	0.402 (0.302 - 0.520)	0.402 (0.302 - 0.519)	0.610 (0.387 - 0.798)	0.605 (0.384 - 0.792)	0.605 (0.384 - 0.792)	0.154 (0.064 - 0.316)	0.153 (0.063 - 0.314)	0.153 (0.063 - 0.315)
5-14 years (rate per capita)	0.348 (0.290 - 0.420)	0.289 (0.239 - 0.353)	0.344 (0.287 - 0.415)	0.485 (0.365 - 0.657)	0.406 (0.301 - 0.565)	0.479 (0.361 - 0.649)	0.133 (0.055 - 0.272)	0.110 (0.046 - 0.221)	0.131 (0.055 - 0.269)
All ages (rate per capita)	0.103 (0.087 - 0.121)	0.060 (0.051 - 0.071)	0.057 (0.048 - 0.067)	0.148 (0.115 - 0.186)	0.086 (0.067 - 0.111)	0.082 (0.064 - 0.103)	0.039 (0.017 - 0.079)	0.023 (0.010 - 0.046)	0.022 (0.009 - 0.044)
Age-standardised (rate per capita)	0.094 (0.079 - 0.111)	0.094 (0.079 - 0.111)	0.094 (0.079 - 0.111)	0.135 (0.105 - 0.170)	0.135 (0.105 - 0.170)	0.135 (0.105 - 0.170)	0.036 (0.015 - 0.073)	0.036 (0.015 - 0.071)	0.036 (0.015 - 0.072)
Total number of cases (thousands)	449 (379 - 529)	250 (212 - 297)	232 (196 - 274)	644 (501 - 812)	360 (280 - 464)	333 (259 - 420)	0 (0 - 0)	0 (0 - 0)	0 (0 - 0)
**Russia**									
Under-5 years (rate per capita)	0.350 (0.273 - 0.454)	0.354 (0.278 - 0.461)	0.357 (0.281 - 0.472)	0.550 (0.344 - 0.770)	0.589 (0.351 - 0.788)	0.589 (0.355 - 0.797)	0.133 (0.056 - 0.266)	0.135 (0.057 - 0.273)	0.136 (0.056 - 0.274)
5-14 years (rate per capita)	0.332 (0.276 - 0.402)	0.304 (0.256 - 0.363)	0.377 (0.310 - 0.453)	0.484 (0.362 - 0.668)	0.420 (0.313 - 0.575)	0.529 (0.404 - 0.713)	0.126 (0.053 - 0.256)	0.116 (0.050 - 0.233)	0.143 (0.061 - 0.293)
All ages (rate per capita)	0.077 (0.066 - 0.091)	0.048 (0.042 - 0.057)	0.056 (0.048 - 0.066)	0.115 (0.089 - 0.152)	0.072 (0.055 - 0.091)	0.084 (0.065 - 0.108)	0.029 (0.012 - 0.059)	0.018 (0.008 - 0.036)	0.021 (0.009 - 0.043)
Age-standardised (rate per capita)	0.086 (0.075 - 0.103)	0.091 (0.079 - 0.107)	0.090 (0.077 - 0.106)	0.129 (0.100 - 0.171)	0.134 (0.104 - 0.170)	0.134 (0.103 - 0.173)	0.033 (0.014 - 0.066)	0.035 (0.015 - 0.068)	0.034 (0.015 - 0.069)
Total number of cases (thousands)	11,339 (9,805 - 13,496)	6,910 (5,942 - 8,105)	8,346 (7,151 - 9,829)	16,986 (13,183 - 22,416)	10,240 (7,868 - 13,084)	12,486 (9,583 - 15,971)	4 (2 - 9)	3 (1 - 5)	3 (1 - 6)
**Ukraine**									
Under-5 years (rate per capita)	0.406 (0.304 - 0.524)	0.395 (0.297 - 0.510)	0.401 (0.301 - 0.517)	0.610 (0.387 - 0.799)	0.594 (0.377 - 0.778)	0.602 (0.382 - 0.789)	0.154 (0.063 - 0.312)	0.150 (0.062 - 0.308)	0.152 (0.063 - 0.308)
5-14 years (rate per capita)	0.340 (0.283 - 0.410)	0.293 (0.243 - 0.357)	0.358 (0.298 - 0.430)	0.474 (0.356 - 0.642)	0.412 (0.305 - 0.571)	0.497 (0.376 - 0.672)	0.129 (0.055 - 0.262)	0.112 (0.047 - 0.229)	0.136 (0.057 - 0.276)
All ages (rate per capita)	0.076 (0.064 - 0.089)	0.048 (0.040 - 0.056)	0.048 (0.041 - 0.057)	0.108 (0.085 - 0.138)	0.069 (0.053 - 0.088)	0.070 (0.054 - 0.088)	0.029 (0.012 - 0.058)	0.018 (0.008 - 0.036)	0.018 (0.008 - 0.037)
Age-standardised (rate per capita)	0.094 (0.079 - 0.111)	0.094 (0.079 - 0.111)	0.094 (0.079 - 0.111)	0.135 (0.105 - 0.170)	0.135 (0.105 - 0.170)	0.135 (0.105 - 0.170)	0.036 (0.015 - 0.072)	0.036 (0.015 - 0.071)	0.036 (0.015 - 0.071)
Total number of cases (thousands)	3,881 (3,300 - 4,562)	2,233 (1,894 - 2,642)	2,251 (1,891 - 2,663)	5,559 (4,337 - 7,058)	3,216 (2,510 - 4,134)	3,238 (2,524 - 4,084)	1 (1 - 3)	1 (0 - 2)	1 (0 - 2)
**Central Europe**									
Under-5 years (rate per capita)	0.484 (0.403 - 0.555)	0.483 (0.407 - 0.549)	0.480 (0.400 - 0.550)	0.663 (0.485 - 0.820)	0.666 (0.492 - 0.812)	0.662 (0.483 - 0.817)	0.168 (0.073 - 0.333)	0.167 (0.073 - 0.329)	0.168 (0.073 - 0.332)
5-14 years (rate per capita)	0.354 (0.302 - 0.417)	0.325 (0.274 - 0.387)	0.348 (0.293 - 0.417)	0.481 (0.365 - 0.649)	0.447 (0.333 - 0.621)	0.480 (0.358 - 0.666)	0.124 (0.052 - 0.249)	0.112 (0.047 - 0.223)	0.121 (0.051 - 0.245)
All ages (rate per capita)	0.093 (0.082 - 0.105)	0.061 (0.054 - 0.069)	0.057 (0.050 - 0.064)	0.127 (0.102 - 0.161)	0.084 (0.067 - 0.108)	0.078 (0.062 - 0.099)	0.032 (0.014 - 0.064)	0.021 (0.009 - 0.041)	0.020 (0.009 - 0.039)
Age-standardised (rate per capita)	0.105 (0.093 - 0.118)	0.104 (0.092 - 0.117)	0.103 (0.090 - 0.116)	0.144 (0.115 - 0.181)	0.142 (0.113 - 0.180)	0.142 (0.113 - 0.180)	0.037 (0.016 - 0.072)	0.036 (0.016 - 0.070)	0.036 (0.016 - 0.070)
Total number of cases (thousands)	11,471 (10,134 - 12,878)	7,246 (6,367 - 8,173)	6,611 (5,796 - 7,467)	15,650 (12,591 - 19,854)	9,985 (7,903 - 12,742)	9,122 (7,263 - 11,605)	4 (2 - 8)	3 (1 - 5)	2 (1 - 5)
**Albania**									
Under-5 years (rate per capita)	0.474 (0.380 - 0.550)	0.488 (0.391 - 0.567)	0.469 (0.376 - 0.545)	0.633 (0.459 - 0.784)	0.652 (0.474 - 0.808)	0.627 (0.455 - 0.776)	0.111 (0.046 - 0.216)	0.115 (0.048 - 0.224)	0.110 (0.047 - 0.215)
5-14 years (rate per capita)	0.348 (0.286 - 0.420)	0.313 (0.257 - 0.379)	0.304 (0.250 - 0.370)	0.474 (0.338 - 0.668)	0.431 (0.303 - 0.617)	0.420 (0.294 - 0.607)	0.082 (0.034 - 0.170)	0.074 (0.031 - 0.152)	0.072 (0.030 - 0.148)
All ages (rate per capita)	0.128 (0.111 - 0.146)	0.095 (0.081 - 0.109)	0.067 (0.057 - 0.076)	0.173 (0.136 - 0.224)	0.129 (0.100 - 0.170)	0.091 (0.071 - 0.119)	0.030 (0.013 - 0.060)	0.022 (0.010 - 0.044)	0.016 (0.007 - 0.032)
Age-standardised (rate per capita)	0.101 (0.087 - 0.116)	0.101 (0.087 - 0.116)	0.101 (0.087 - 0.116)	0.137 (0.107 - 0.177)	0.137 (0.107 - 0.177)	0.137 (0.107 - 0.177)	0.024 (0.010 - 0.048)	0.024 (0.010 - 0.047)	0.024 (0.010 - 0.048)
Total number of cases (thousands)	421 (362 - 480)	293 (251 - 337)	194 (166 - 221)	569 (447 - 734)	399 (310 - 526)	264 (204 - 345)	0 (0 - 0)	0 (0 - 0)	0 (0 - 0)
**Bosnia and Herzegovina**									
Under-5 years (rate per capita)	0.479 (0.390 - 0.555)	0.475 (0.386 - 0.550)	0.473 (0.385 - 0.548)	0.651 (0.474 - 0.804)	0.645 (0.470 - 0.796)	0.643 (0.468 - 0.793)	0.113 (0.048 - 0.222)	0.112 (0.048 - 0.220)	0.111 (0.047 - 0.221)
5-14 years (rate per capita)	0.355 (0.299 - 0.424)	0.339 (0.285 - 0.406)	0.353 (0.297 - 0.422)	0.471 (0.351 - 0.646)	0.452 (0.334 - 0.623)	0.469 (0.349 - 0.643)	0.083 (0.036 - 0.168)	0.080 (0.033 - 0.162)	0.083 (0.035 - 0.169)
All ages (rate per capita)	0.095 (0.083 - 0.109)	0.061 (0.053 - 0.070)	0.053 (0.046 - 0.061)	0.128 (0.101 - 0.162)	0.082 (0.065 - 0.105)	0.071 (0.056 - 0.090)	0.022 (0.010 - 0.044)	0.014 (0.006 - 0.029)	0.013 (0.005 - 0.025)
Age-standardised (rate per capita)	0.102 (0.089 - 0.116)	0.102 (0.089 - 0.116)	0.102 (0.089 - 0.116)	0.137 (0.108 - 0.173)	0.137 (0.108 - 0.173)	0.137 (0.108 - 0.173)	0.024 (0.010 - 0.047)	0.024 (0.010 - 0.049)	0.024 (0.010 - 0.048)
Total number of cases (thousands)	432 (376 - 492)	234 (203 - 268)	202 (176 - 231)	578 (457 - 735)	314 (248 - 404)	271 (215 - 344)	0 (0 - 0)	0 (0 - 0)	0 (0 - 0)
**Bulgaria**									
Under-5 years (rate per capita)	0.476 (0.391 - 0.550)	0.464 (0.379 - 0.537)	0.473 (0.386 - 0.547)	0.666 (0.496 - 0.814)	0.657 (0.474 - 0.812)	0.669 (0.483 - 0.827)	0.181 (0.078 - 0.363)	0.177 (0.075 - 0.350)	0.180 (0.077 - 0.365)
5-14 years (rate per capita)	0.327 (0.275 - 0.395)	0.307 (0.255 - 0.372)	0.354 (0.297 - 0.425)	0.453 (0.336 - 0.632)	0.437 (0.321 - 0.617)	0.497 (0.371 - 0.695)	0.124 (0.051 - 0.250)	0.117 (0.048 - 0.235)	0.134 (0.056 - 0.270)
All ages (rate per capita)	0.076 (0.067 - 0.087)	0.049 (0.042 - 0.056)	0.052 (0.045 - 0.059)	0.106 (0.084 - 0.137)	0.069 (0.054 - 0.090)	0.073 (0.059 - 0.094)	0.029 (0.012 - 0.057)	0.019 (0.008 - 0.037)	0.020 (0.008 - 0.040)
Age-standardised (rate per capita)	0.102 (0.090 - 0.115)	0.101 (0.087 - 0.115)	0.101 (0.087 - 0.115)	0.141 (0.112 - 0.181)	0.143 (0.114 - 0.183)	0.143 (0.114 - 0.183)	0.039 (0.016 - 0.076)	0.039 (0.017 - 0.076)	0.039 (0.016 - 0.077)
Total number of cases (thousands)	672 (591 - 766)	375 (323 - 429)	379 (328 - 430)	934 (738 - 1,208)	532 (418 - 690)	534 (425 - 681)	0 (0 - 1)	0 (0 - 0)	0 (0 - 0)
**Croatia**									
Under-5 years (rate per capita)	0.485 (0.398 - 0.555)	0.479 (0.395 - 0.548)	0.481 (0.396 - 0.549)	0.679 (0.488 - 0.837)	0.665 (0.481 - 0.822)	0.667 (0.482 - 0.824)	0.185 (0.079 - 0.365)	0.183 (0.078 - 0.355)	0.183 (0.078 - 0.375)
5-14 years (rate per capita)	0.343 (0.287 - 0.408)	0.393 (0.342 - 0.452)	0.400 (0.348 - 0.459)	0.475 (0.349 - 0.663)	0.513 (0.370 - 0.701)	0.520 (0.378 - 0.709)	0.130 (0.054 - 0.266)	0.150 (0.064 - 0.296)	0.152 (0.065 - 0.300)
All ages (rate per capita)	0.076 (0.067 - 0.086)	0.067 (0.059 - 0.074)	0.064 (0.056 - 0.070)	0.106 (0.083 - 0.137)	0.089 (0.071 - 0.113)	0.085 (0.068 - 0.108)	0.029 (0.012 - 0.057)	0.025 (0.011 - 0.049)	0.024 (0.011 - 0.048)
Age-standardised (rate per capita)	0.103 (0.090 - 0.116)	0.111 (0.097 - 0.123)	0.111 (0.097 - 0.123)	0.143 (0.112 - 0.182)	0.148 (0.119 - 0.188)	0.148 (0.119 - 0.188)	0.039 (0.017 - 0.077)	0.042 (0.018 - 0.082)	0.042 (0.018 - 0.084)
Total number of cases (thousands)	364 (318 - 413)	292 (257 - 325)	270 (237 - 299)	507 (398 - 653)	389 (309 - 497)	359 (287 - 457)	0 (0 - 0)	0 (0 - 0)	0 (0 - 0)
**Czech Republic**									
Under-5 years (rate per capita)	0.477 (0.382 - 0.554)	0.466 (0.374 - 0.541)	0.477 (0.382 - 0.554)	0.662 (0.478 - 0.825)	0.647 (0.467 - 0.806)	0.662 (0.478 - 0.824)	0.182 (0.079 - 0.365)	0.178 (0.077 - 0.365)	0.182 (0.077 - 0.366)
5-14 years (rate per capita)	0.305 (0.251 - 0.370)	0.299 (0.246 - 0.364)	0.367 (0.303 - 0.443)	0.438 (0.307 - 0.625)	0.430 (0.301 - 0.614)	0.517 (0.372 - 0.718)	0.116 (0.049 - 0.235)	0.114 (0.048 - 0.232)	0.140 (0.058 - 0.287)
All ages (rate per capita)	0.076 (0.065 - 0.087)	0.052 (0.044 - 0.059)	0.058 (0.050 - 0.066)	0.108 (0.083 - 0.142)	0.073 (0.056 - 0.096)	0.081 (0.063 - 0.104)	0.029 (0.012 - 0.058)	0.020 (0.008 - 0.039)	0.022 (0.009 - 0.044)

	Prevalence 1990	Prevalence 2005	Prevalence 2015	Incidence 1990	Incidence 2005	Incidence 2015	YLD rate 1990	YLD rate 2005	YLD rate 2015
**Age-standardised (rate per capita)**	0.101 (0.087 - 0.116)	0.101 (0.087 - 0.116)	0.101 (0.087 - 0.116)	0.143 (0.111 - 0.185)	0.143 (0.111 - 0.185)	0.143 (0.111 - 0.185)	0.039 (0.017 - 0.078)	0.039 (0.017 - 0.078)	0.039 (0.017 - 0.078)
**Total number of cases (thousands)**	783 (669 - 899)	531 (454 - 608)	618 (533 - 703)	1,110 (856 - 1,461)	753 (577 - 990)	865 (675 - 1,114)	0 (0 - 1)	0 (0 - 0)	0 (0 - 0)
**Hungary**									
**Under-5 years (rate per capita)**	0.457 (0.372 - 0.535)	0.457 (0.366 - 0.538)	0.459 (0.371 - 0.538)	0.652 (0.460 - 0.817)	0.656 (0.460 - 0.824)	0.657 (0.464 - 0.822)	0.174 (0.075 - 0.341)	0.174 (0.074 - 0.347)	0.175 (0.074 - 0.354)
**5-14 years (rate per capita)**	0.307 (0.257 - 0.368)	0.343 (0.292 - 0.398)	0.361 (0.302 - 0.431)	0.427 (0.314 - 0.596)	0.462 (0.325 - 0.650)	0.500 (0.374 - 0.693)	0.117 (0.049 - 0.234)	0.131 (0.055 - 0.257)	0.137 (0.057 - 0.275)
**All ages (rate per capita)**	0.071 (0.062 - 0.082)	0.058 (0.051 - 0.066)	0.053 (0.046 - 0.061)	0.100 (0.078 - 0.130)	0.081 (0.063 - 0.103)	0.075 (0.059 - 0.095)	0.027 (0.012 - 0.053)	0.022 (0.010 - 0.043)	0.020 (0.009 - 0.040)
**Age-standardised (rate per capita)**	0.101 (0.088 - 0.115)	0.105 (0.092 - 0.118)	0.100 (0.087 - 0.115)	0.141 (0.110 - 0.178)	0.144 (0.113 - 0.182)	0.141 (0.111 - 0.179)	0.038 (0.017 - 0.075)	0.040 (0.017 - 0.077)	0.038 (0.016 - 0.075)
**Total number of cases (thousands)**	742 (645 - 853)	590 (514 - 666)	542 (469 - 621)	1,042 (811 - 1,351)	813 (635 - 1,037)	761 (601 - 964)	0 (0 - 1)	0 (0 - 0)	0 (0 - 0)
**Macedonia**									
**Under-5 years (rate per capita)**	0.478 (0.383 - 0.556)	0.480 (0.384 - 0.557)	0.474 (0.380 - 0.550)	0.639 (0.464 - 0.792)	0.641 (0.465 - 0.794)	0.633 (0.459 - 0.784)	0.113 (0.047 - 0.221)	0.113 (0.048 - 0.221)	0.111 (0.047 - 0.217)
**5-14 years (rate per capita)**	0.346 (0.284 - 0.417)	0.323 (0.265 - 0.391)	0.323 (0.265 - 0.390)	0.471 (0.336 - 0.663)	0.443 (0.313 - 0.630)	0.443 (0.313 - 0.630)	0.081 (0.034 - 0.169)	0.076 (0.032 - 0.156)	0.076 (0.032 - 0.158)
**All ages (rate per capita)**	0.103 (0.089 - 0.118)	0.075 (0.064 - 0.086)	0.063 (0.054 - 0.072)	0.139 (0.109 - 0.180)	0.102 (0.079 - 0.133)	0.086 (0.067 - 0.111)	0.024 (0.010 - 0.049)	0.018 (0.008 - 0.036)	0.015 (0.006 - 0.030)
**Age-standardised (rate per capita)**	0.101 (0.087 - 0.116)	0.101 (0.087 - 0.116)	0.101 (0.087 - 0.116)	0.137 (0.107 - 0.177)	0.137 (0.107 - 0.177)	0.137 (0.107 - 0.177)	0.024 (0.010 - 0.049)	0.024 (0.010 - 0.048)	0.024 (0.010 - 0.048)
**Total number of cases (thousands)**	207 (178 - 236)	153 (131 - 176)	131 (113 - 150)	279 (218 - 361)	208 (162 - 272)	178 (138 - 231)	0 (0 - 0)	0 (0 - 0)	0 (0 - 0)
**Montenegro**									
**Under-5 years (rate per capita)**	0.478 (0.383 - 0.555)	0.476 (0.381 - 0.552)	0.479 (0.384 - 0.556)	0.639 (0.464 - 0.791)	0.636 (0.461 - 0.787)	0.640 (0.464 - 0.792)	0.112 (0.048 - 0.216)	0.112 (0.048 - 0.218)	0.113 (0.048 - 0.219)
**5-14 years (rate per capita)**	0.346 (0.284 - 0.418)	0.330 (0.271 - 0.399)	0.337 (0.276 - 0.407)	0.471 (0.336 - 0.664)	0.452 (0.320 - 0.638)	0.460 (0.327 - 0.648)	0.081 (0.034 - 0.170)	0.078 (0.032 - 0.159)	0.079 (0.033 - 0.161)
**All ages (rate per capita)**	0.099 (0.085 - 0.113)	0.077 (0.066 - 0.088)	0.071 (0.061 - 0.082)	0.134 (0.105 - 0.173)	0.104 (0.081 - 0.135)	0.097 (0.076 - 0.126)	0.023 (0.010 - 0.047)	0.018 (0.008 - 0.036)	0.017 (0.007 - 0.033)
**Age-standardised (rate per capita)**	0.101 (0.087 - 0.116)	0.101 (0.087 - 0.116)	0.101 (0.087 - 0.116)	0.137 (0.107 - 0.177)	0.137 (0.107 - 0.177)	0.137 (0.107 - 0.177)	0.024 (0.010 - 0.048)	0.024 (0.010 - 0.048)	0.024 (0.010 - 0.047)
**Total number of cases (thousands)**	61 (53 - 70)	47 (41 - 54)	45 (38 - 51)	82 (65 - 107)	64 (50 - 83)	60 (47 - 79)	0 (0 - 0)	0 (0 - 0)	0 (0 - 0)
**Poland**									
**Under-5 years (rate per capita)**	0.504 (0.422 - 0.565)	0.509 (0.439 - 0.566)	0.499 (0.418 - 0.560)	0.685 (0.510 - 0.842)	0.693 (0.533 - 0.823)	0.679 (0.505 - 0.835)	0.192 (0.084 - 0.384)	0.194 (0.084 - 0.379)	0.191 (0.083 - 0.375)
**5-14 years (rate per capita)**	0.357 (0.300 - 0.428)	0.319 (0.266 - 0.381)	0.348 (0.293 - 0.418)	0.490 (0.363 - 0.678)	0.440 (0.327 - 0.610)	0.480 (0.355 - 0.666)	0.136 (0.058 - 0.278)	0.121 (0.051 - 0.246)	0.132 (0.055 - 0.272)
**All ages (rate per capita)**	0.102 (0.090 - 0.115)	0.062 (0.054 - 0.070)	0.055 (0.048 - 0.061)	0.139 (0.111 - 0.177)	0.085 (0.067 - 0.108)	0.075 (0.059 - 0.094)	0.039 (0.017 - 0.077)	0.024 (0.010 - 0.046)	0.021 (0.009 - 0.041)
**Age-standardised (rate per capita)**	0.105 (0.093 - 0.118)	0.107 (0.095 - 0.119)	0.105 (0.093 - 0.118)	0.144 (0.114 - 0.182)	0.146 (0.117 - 0.181)	0.144 (0.114 - 0.182)	0.040 (0.017 - 0.079)	0.041 (0.018 - 0.081)	0.040 (0.017 - 0.079)
**Total number of cases (thousands)**	3,898 (3,441 - 4,389)	2,361 (2,078 - 2,655)	2,124 (1,876 - 2,382)	5,331 (4,243 - 6,775)	3,239 (2,564 - 4,124)	2,906 (2,313 - 3,656)	1 (1 - 3)	1 (0 - 2)	1 (0 - 2)
**Romania**									
**Under-5 years (rate per capita)**	0.466 (0.375 - 0.545)	0.455 (0.365 - 0.539)	0.459 (0.369 - 0.545)	0.653 (0.459 - 0.828)	0.655 (0.456 - 0.818)	0.662 (0.461 - 0.827)	0.178 (0.075 - 0.357)	0.173 (0.074 - 0.346)	0.175 (0.074 - 0.344)
**5-14 years (rate per capita)**	0.411 (0.357 - 0.465)	0.324 (0.269 - 0.392)	0.339 (0.282 - 0.409)	0.538 (0.416 - 0.700)	0.460 (0.336 - 0.634)	0.479 (0.352 - 0.657)	0.156 (0.068 - 0.304)	0.123 (0.051 - 0.249)	0.129 (0.053 - 0.263)
**All ages (rate per capita)**	0.101 (0.089 - 0.111)	0.058 (0.050 - 0.067)	0.059 (0.051 - 0.067)	0.135 (0.111 - 0.168)	0.083 (0.065 - 0.106)	0.084 (0.066 - 0.106)	0.038 (0.017 - 0.075)	0.022 (0.009 - 0.044)	0.022 (0.010 - 0.044)
**Age-standardised (rate per capita)**	0.115 (0.101 - 0.126)	0.100 (0.086 - 0.114)	0.100 (0.086 - 0.114)	0.154 (0.125 - 0.191)	0.142 (0.112 - 0.180)	0.142 (0.112 - 0.180)	0.044 (0.019 - 0.086)	0.038 (0.016 - 0.075)	0.038 (0.016 - 0.075)
**Total number of cases (thousands)**	2,359 (2,079 - 2,596)	1,247 (1,080 - 1,434)	1,146 (995 - 1,313)	3,161 (2,591 - 3,927)	1,780 (1,398 - 2,273)	1,631 (1,283 - 2,073)	1 (0 - 2)	0 (0 - 1)	0 (0 - 1)
**Serbia**									
**Under-5 years (rate per capita)**	0.499 (0.415 - 0.564)	0.520 (0.447 - 0.578)	0.497 (0.413 - 0.561)	0.652 (0.487 - 0.794)	0.677 (0.521 - 0.802)	0.649 (0.484 - 0.790)	0.118 (0.051 - 0.227)	0.123 (0.053 - 0.235)	0.117 (0.050 - 0.227)
**5-14 years (rate per capita)**	0.346 (0.293 - 0.420)	0.350 (0.297 - 0.412)	0.333 (0.280 - 0.404)	0.452 (0.337 - 0.641)	0.448 (0.335 - 0.625)	0.436 (0.324 - 0.620)	0.081 (0.034 - 0.167)	0.082 (0.035 - 0.162)	0.078 (0.033 - 0.162)
**All ages (rate per capita)**	0.094 (0.083 - 0.106)	0.077 (0.069 - 0.086)	0.063 (0.055 - 0.071)	0.123 (0.098 - 0.157)	0.099 (0.080 - 0.126)	0.082 (0.066 - 0.105)	0.022 (0.009 - 0.044)	0.018 (0.008 - 0.035)	0.015 (0.006 - 0.030)
**Age-standardised (rate per capita)**	0.105 (0.093 - 0.119)	0.108 (0.096 - 0.120)	0.105 (0.093 - 0.119)	0.137 (0.110 - 0.174)	0.139 (0.113 - 0.175)	0.137 (0.110 - 0.174)	0.025 (0.011 - 0.049)	0.025 (0.011 - 0.049)	0.025 (0.011 - 0.050)
**Total number of cases (thousands)**	893 (788 - 1,011)	707 (632 - 791)	557 (490 - 632)	1,165 (935 - 1,491)	912 (736 - 1,159)	728 (584 - 932)	0 (0 - 0)	0 (0 - 0)	0 (0 - 0)
**Slovakia**									
**Under-5 years (rate per capita)**	0.480 (0.384 - 0.557)	0.473 (0.379 - 0.550)	0.475 (0.381 - 0.552)	0.641 (0.465 - 0.794)	0.633 (0.459 - 0.784)	0.635 (0.460 - 0.786)	0.113 (0.048 - 0.225)	0.111 (0.047 - 0.219)	0.112 (0.047 - 0.218)
**5-14 years (rate per capita)**	0.329 (0.270 - 0.398)	0.306 (0.251 - 0.371)	0.341 (0.279 - 0.411)	0.451 (0.319 - 0.637)	0.422 (0.296 - 0.609)	0.464 (0.330 - 0.654)	0.077 (0.032 - 0.163)	0.072 (0.030 - 0.147)	0.080 (0.033 - 0.167)
**All ages (rate per capita)**	0.096 (0.082 - 0.109)	0.060 (0.051 - 0.069)	0.053 (0.046 - 0.061)	0.129 (0.101 - 0.169)	0.081 (0.063 - 0.108)	0.072 (0.056 - 0.093)	0.022 (0.010 - 0.045)	0.014 (0.006 - 0.028)	0.013 (0.005 - 0.025)
**Age-standardised (rate per capita)**	0.101 (0.087 - 0.116)	0.101 (0.087 - 0.116)	0.101 (0.087 - 0.116)	0.137 (0.107 - 0.177)	0.137 (0.107 - 0.177)	0.137 (0.107 - 0.177)	0.024 (0.010 - 0.048)	0.024 (0.010 - 0.046)	0.024 (0.010 - 0.047)
**Total number of cases (thousands)**	503 (431 - 575)	322 (275 - 369)	296 (255 - 337)	683 (533 - 891)	439 (340 - 580)	400 (313 - 518)	0 (0 - 0)	0 (0 - 0)	0 (0 - 0)
**Slovenia**									
**Under-5 years (rate per capita)**	0.388 (0.304 - 0.492)	0.380 (0.304 - 0.487)	0.382 (0.306 - 0.489)	0.604 (0.378 - 0.811)	0.611 (0.375 - 0.816)	0.615 (0.377 - 0.821)	0.148 (0.063 - 0.296)	0.145 (0.061 - 0.296)	0.146 (0.062 - 0.295)
**5-14 years (rate per capita)**	0.300 (0.245 - 0.367)	0.310 (0.250 - 0.383)	0.344 (0.278 - 0.421)	0.458 (0.319 - 0.650)	0.455 (0.335 - 0.639)	0.501 (0.372 - 0.691)	0.114 (0.047 - 0.231)	0.118 (0.049 - 0.238)	0.131 (0.054 - 0.266)
**All ages (rate per capita)**	0.068 (0.057 - 0.080)	0.047 (0.040 - 0.056)	0.052 (0.045 - 0.062)	0.104 (0.076 - 0.139)	0.071 (0.054 - 0.093)	0.079 (0.061 - 0.103)	0.026 (0.011 - 0.050)	0.018 (0.008 - 0.035)	0.020 (0.008 - 0.039)
**Age-standardised (rate per capita)**	0.088 (0.075 - 0.104)	0.091 (0.078 - 0.108)	0.091 (0.078 - 0.108)	0.135 (0.100 - 0.180)	0.138 (0.105 - 0.179)	0.138 (0.105 - 0.179)	0.034 (0.014 - 0.066)	0.035 (0.015 - 0.069)	0.035 (0.015 - 0.068)
**Total number of cases (thousands)**	136 (115 - 160)	93 (79 - 111)	108 (92 - 128)	208 (153 - 280)	141 (108 - 186)	163 (125 - 213)	0 (0 - 0)	0 (0 - 0)	0 (0 - 0)
**Central Asia**									
**Under-5 years (rate per capita)**	0.467 (0.372 - 0.551)	0.464 (0.370 - 0.547)	0.469 (0.374 - 0.552)	0.624 (0.452 - 0.777)	0.619 (0.448 - 0.770)	0.626 (0.453 - 0.779)	0.124 (0.053 - 0.242)	0.121 (0.052 - 0.237)	0.124 (0.053 - 0.241)
**5-14 years (rate per capita)**	0.330 (0.265 - 0.408)	0.290 (0.231 - 0.361)	0.334 (0.268 - 0.413)	0.511 (0.361 - 0.690)	0.453 (0.315 - 0.629)	0.516 (0.366 - 0.697)	0.088 (0.037 - 0.175)	0.075 (0.031 - 0.150)	0.088 (0.037 - 0.176)
**All ages (rate per capita)**	0.138 (0.117 - 0.160)	0.102 (0.086 - 0.118)	0.106 (0.089 - 0.122)	0.200 (0.153 - 0.251)	0.149 (0.114 - 0.192)	0.153 (0.117 - 0.192)	0.037 (0.016 - 0.072)	0.026 (0.011 - 0.052)	0.028 (0.012 - 0.055)
**Age-standardised (rate per capita)**	0.096 (0.081 - 0.112)	0.096 (0.081 - 0.112)	0.096 (0.081 - 0.112)	0.140 (0.108 - 0.178)	0.140 (0.108 - 0.178)	0.140 (0.108 - 0.178)	0.026 (0.011 - 0.050)	0.025 (0.011 - 0.049)	0.025 (0.011 - 0.050)
**Total number of cases (thousands)**	9,476 (8,021 - 10,982)	7,833 (6,613 - 9,117)	9,187 (7,771 - 10,648)	13,740 (10,544 - 17,276)	11,475 (8,742 - 14,748)	13,286 (10,222 - 16,689)	3 (1 - 5)	2 (1 - 4)	2 (1 - 5)
**Armenia**									
**Under-5 years (rate per capita)**	0.473 (0.377 - 0.557)	0.468 (0.373 - 0.551)	0.473 (0.377 - 0.557)	0.626 (0.454 - 0.778)	0.619 (0.450 - 0.770)	0.626 (0.455 - 0.779)	0.111 (0.046 - 0.219)	0.110 (0.046 - 0.213)	0.111 (0.047 - 0.217)
**5-14 years (rate per capita)**	0.331 (0.266 - 0.410)	0.272 (0.216 - 0.340)	0.335 (0.269 - 0.414)	0.509 (0.360 - 0.688)	0.426 (0.294 - 0.601)	0.514 (0.364 - 0.694)	0.078 (0.033 - 0.160)	0.064 (0.026 - 0.130)	0.079 (0.033 - 0.159)
**All ages (rate per capita)**	0.115 (0.097 - 0.133)	0.073 (0.061 - 0.085)	0.070 (0.059 - 0.081)	0.166 (0.128 - 0.209)	0.106 (0.081 - 0.137)	0.101 (0.077 - 0.126)	0.027 (0.012 - 0.053)	0.017 (0.007 - 0.035)	0.016 (0.007 - 0.032)
**Age-standardised (rate per capita)**	0.096 (0.081 - 0.112)	0.096 (0.081 - 0.112)	0.096 (0.081 - 0.112)	0.139 (0.107 - 0.177)	0.139 (0.107 - 0.177)	0.139 (0.107 - 0.177)	0.023 (0.010 - 0.045)	0.023 (0.010 - 0.045)	0.023 (0.010 - 0.045)
**Total number of cases (thousands)**	405 (343 - 470)	220 (185 - 257)	210 (178 - 243)	585 (451 - 738)	321 (244 - 415)	302 (233 - 380)	0 (0 - 0)	0 (0 - 0)	0 (0 - 0)
**Azerbaijan**									
**Under-5 years (rate per capita)**	0.462 (0.369 - 0.545)	0.460 (0.367 - 0.542)	0.471 (0.375 - 0.555)	0.613 (0.445 - 0.762)	0.609 (0.443 - 0.758)	0.624 (0.453 - 0.776)	0.109 (0.046 - 0.214)	0.108 (0.046 - 0.217)	0.111 (0.046 - 0.216)
**5-14 years (rate per capita)**	0.331 (0.266 - 0.409)	0.279 (0.222 - 0.348)	0.318 (0.255 - 0.394)	0.508 (0.360 - 0.687)	0.435 (0.301 - 0.610)	0.490 (0.345 - 0.666)	0.078 (0.033 - 0.158)	0.065 (0.027 - 0.131)	0.074 (0.031 - 0.152)
**All ages (rate per capita)**	0.127 (0.108 - 0.148)	0.090 (0.076 - 0.105)	0.085 (0.072 - 0.099)	0.184 (0.141 - 0.232)	0.131 (0.099 - 0.169)	0.122 (0.094 - 0.152)	0.030 (0.013 - 0.059)	0.021 (0.009 - 0.042)	0.020 (0.009 - 0.040)
**Age-standardised (rate per capita)**	0.096 (0.081 - 0.112)	0.096 (0.081 - 0.112)	0.096 (0.081 - 0.112)	0.139 (0.107 - 0.177)	0.139 (0.107 - 0.177)	0.139 (0.107 - 0.176)	0.023 (0.010 - 0.044)	0.023 (0.010 - 0.045)	0.023 (0.010 - 0.045)
**Total number of cases (thousands)**	911 (772 - 1,056)	775 (654 - 907)	835 (702 - 968)	1,314 (1,012 - 1,658)	1,133 (860 - 1,461)	1,189 (919 - 1,487)	0 (0 - 0)	0 (0 - 0)	0 (0 - 0)
**Georgia**									
**Under-5 years (rate per capita)**	0.473 (0.377 - 0.558)	0.468 (0.373 - 0.551)	0.475 (0.378 - 0.559)	0.627 (0.455 - 0.780)	0.620 (0.450 - 0.771)	0.629 (0.457 - 0.782)	0.112 (0.047 - 0.218)	0.110 (0.046 - 0.218)	0.112 (0.047 - 0.219)
**5-14 years (rate per capita)**	0.317 (0.255 - 0.395)	0.261 (0.207 - 0.328)	0.336 (0.271 - 0.416)	0.490 (0.345 - 0.666)	0.411 (0.282 - 0.585)	0.516 (0.366 - 0.697)	0.074 (0.031 - 0.154)	0.062 (0.025 - 0.127)	0.079 (0.032 - 0.161)
**All ages (rate per capita)**	0.091 (0.077 - 0.106)	0.062 (0.052 - 0.073)	0.068 (0.058 - 0.079)	0.132 (0.102 - 0.167)	0.091 (0.069 - 0.117)	0.098 (0.075 - 0.123)	0.021 (0.009 - 0.043)	0.015 (0.006 - 0.030)	0.016 (0.007 - 0.032)

	Prevalence 1990	Prevalence 2005	Prevalence 2015	Incidence 1990	Incidence 2005	Incidence 2015	YLD rate 1990	YLD rate 2005	YLD rate 2015
Age-standardised (rate per capita)	0.096 (0.081 - 0.112)	0.096 (0.081 - 0.112)	0.096 (0.081 - 0.112)	0.139 (0.107 - 0.177)	0.139 (0.107 - 0.177)	0.139 (0.107 - 0.176)	0.023 (0.010 - 0.046)	0.023 (0.010 - 0.046)	0.023 (0.010 - 0.045)
Total number of cases (thousands)	498 (422 - 579)	281 (236 - 328)	273 (231 - 317)	722 (555 - 915)	410 (313 - 528)	391 (302 - 492)	0 (0 - 0)	0 (0 - 0)	0 (0 - 0)
**Kazakhstan**									
Under-5 years (rate per capita)	0.473 (0.377 - 0.558)	0.457 (0.364 - 0.539)	0.470 (0.374 - 0.554)	0.651 (0.466 - 0.814)	0.629 (0.450 - 0.787)	0.646 (0.463 - 0.809)	0.180 (0.076 - 0.365)	0.174 (0.074 - 0.342)	0.179 (0.075 - 0.365)
5-14 years (rate per capita)	0.322 (0.259 - 0.399)	0.284 (0.226 - 0.354)	0.358 (0.289 - 0.440)	0.513 (0.362 - 0.696)	0.457 (0.316 - 0.641)	0.566 (0.404 - 0.762)	0.122 (0.051 - 0.249)	0.108 (0.045 - 0.217)	0.137 (0.056 - 0.278)
All ages (rate per capita)	0.120 (0.101 - 0.139)	0.084 (0.071 - 0.098)	0.106 (0.090 - 0.123)	0.179 (0.137 - 0.226)	0.127 (0.097 - 0.163)	0.158 (0.121 - 0.198)	0.046 (0.020 - 0.090)	0.032 (0.014 - 0.063)	0.041 (0.017 - 0.081)
Age-standardised (rate per capita)	0.096 (0.081 - 0.112)	0.096 (0.081 - 0.112)	0.096 (0.081 - 0.112)	0.144 (0.110 - 0.183)	0.144 (0.111 - 0.183)	0.144 (0.111 - 0.183)	0.037 (0.016 - 0.072)	0.037 (0.016 - 0.071)	0.037 (0.016 - 0.073)
Total number of cases (thousands)	1,991 (1,683 - 2,308)	1,307 (1,101 - 1,522)	1,863 (1,580 - 2,156)	2,971 (2,275 - 3,759)	1,968 (1,497 - 2,528)	2,765 (2,122 - 3,474)	1 (0 - 1)	0 (0 - 1)	1 (0 - 1)
**Kyrgyzstan**									
Under-5 years (rate per capita)	0.467 (0.372 - 0.550)	0.465 (0.371 - 0.548)	0.467 (0.372 - 0.550)	0.618 (0.449 - 0.769)	0.617 (0.447 - 0.767)	0.618 (0.449 - 0.769)	0.110 (0.046 - 0.212)	0.110 (0.046 - 0.224)	0.110 (0.046 - 0.215)
5-14 years (rate per capita)	0.331 (0.266 - 0.410)	0.293 (0.234 - 0.365)	0.341 (0.275 - 0.421)	0.509 (0.361 - 0.689)	0.455 (0.317 - 0.630)	0.523 (0.371 - 0.707)	0.078 (0.032 - 0.156)	0.069 (0.029 - 0.141)	0.080 (0.033 - 0.164)
All ages (rate per capita)	0.144 (0.122 - 0.167)	0.109 (0.092 - 0.127)	0.121 (0.102 - 0.140)	0.208 (0.160 - 0.261)	0.159 (0.122 - 0.205)	0.173 (0.134 - 0.217)	0.034 (0.014 - 0.068)	0.026 (0.011 - 0.052)	0.028 (0.012 - 0.057)
Age-standardised (rate per capita)	0.096 (0.081 - 0.112)	0.096 (0.081 - 0.112)	0.096 (0.081 - 0.112)	0.139 (0.107 - 0.177)	0.139 (0.107 - 0.177)	0.139 (0.107 - 0.177)	0.023 (0.009 - 0.045)	0.023 (0.010 - 0.046)	0.023 (0.010 - 0.045)
Total number of cases (thousands)	635 (537 - 736)	563 (476 - 655)	713 (604 - 826)	913 (702 - 1,148)	820 (626 - 1,054)	1,021 (788 - 1,278)	0 (0 - 0)	0 (0 - 0)	0 (0 - 0)
**Mongolia**									
Under-5 years (rate per capita)	0.470 (0.374 - 0.554)	0.462 (0.368 - 0.544)	0.468 (0.372 - 0.551)	0.622 (0.451 - 0.774)	0.612 (0.444 - 0.761)	0.619 (0.449 - 0.770)	0.110 (0.046 - 0.217)	0.109 (0.046 - 0.212)	0.110 (0.046 - 0.215)
5-14 years (rate per capita)	0.326 (0.262 - 0.404)	0.286 (0.229 - 0.357)	0.343 (0.277 - 0.424)	0.502 (0.355 - 0.679)	0.446 (0.310 - 0.622)	0.527 (0.374 - 0.710)	0.077 (0.031 - 0.157)	0.067 (0.028 - 0.138)	0.081 (0.033 - 0.170)
All ages (rate per capita)	0.154 (0.130 - 0.179)	0.099 (0.084 - 0.116)	0.110 (0.093 - 0.128)	0.222 (0.171 - 0.280)	0.145 (0.110 - 0.187)	0.158 (0.122 - 0.198)	0.036 (0.015 - 0.073)	0.023 (0.010 - 0.047)	0.026 (0.011 - 0.052)
Age-standardised (rate per capita)	0.096 (0.081 - 0.112)	0.096 (0.081 - 0.112)	0.096 (0.081 - 0.112)	0.139 (0.107 - 0.176)	0.139 (0.107 - 0.176)	0.139 (0.107 - 0.177)	0.023 (0.010 - 0.045)	0.023 (0.010 - 0.045)	0.023 (0.010 - 0.045)
Total number of cases (thousands)	337 (285 - 391)	251 (212 - 293)	326 (276 - 378)	486 (373 - 612)	366 (279 - 473)	467 (361 - 584)	0 (0 - 0)	0 (0 - 0)	0 (0 - 0)
**Tajikistan**									
Under-5 years (rate per capita)	0.462 (0.368 - 0.545)	0.465 (0.371 - 0.548)	0.465 (0.371 - 0.548)	0.612 (0.444 - 0.761)	0.616 (0.447 - 0.766)	0.616 (0.447 - 0.766)	0.108 (0.046 - 0.209)	0.109 (0.046 - 0.213)	0.109 (0.046 - 0.213)
5-14 years (rate per capita)	0.341 (0.275 - 0.421)	0.310 (0.249 - 0.387)	0.327 (0.263 - 0.404)	0.524 (0.372 - 0.708)	0.480 (0.337 - 0.655)	0.503 (0.355 - 0.680)	0.080 (0.033 - 0.168)	0.073 (0.030 - 0.148)	0.077 (0.031 - 0.154)
All ages (rate per capita)	0.170 (0.144 - 0.197)	0.141 (0.119 - 0.164)	0.134 (0.113 - 0.155)	0.243 (0.188 - 0.304)	0.204 (0.157 - 0.260)	0.192 (0.148 - 0.241)	0.040 (0.017 - 0.079)	0.033 (0.014 - 0.067)	0.031 (0.013 - 0.062)
Age-standardised (rate per capita)	0.096 (0.081 - 0.112)	0.096 (0.081 - 0.112)	0.096 (0.081 - 0.112)	0.139 (0.107 - 0.177)	0.139 (0.107 - 0.177)	0.139 (0.107 - 0.177)	0.023 (0.010 - 0.045)	0.023 (0.010 - 0.045)	0.023 (0.010 - 0.045)
Total number of cases (thousands)	898 (760 - 1,040)	966 (817 - 1,123)	1,137 (961 - 1,319)	1,286 (992 - 1,608)	1,400 (1,075 - 1,779)	1,631 (1,257 - 2,052)	0 (0 - 0)	0 (0 - 0)	0 (0 - 1)
**Turkmenistan**									
Under-5 years (rate per capita)	0.462 (0.368 - 0.544)	0.467 (0.372 - 0.551)	0.468 (0.373 - 0.552)	0.611 (0.444 - 0.760)	0.619 (0.449 - 0.769)	0.620 (0.450 - 0.771)	0.108 (0.046 - 0.216)	0.110 (0.047 - 0.215)	0.110 (0.046 - 0.224)
5-14 years (rate per capita)	0.330 (0.265 - 0.408)	0.288 (0.230 - 0.359)	0.312 (0.251 - 0.389)	0.507 (0.359 - 0.685)	0.448 (0.311 - 0.623)	0.483 (0.339 - 0.658)	0.077 (0.032 - 0.158)	0.068 (0.028 - 0.138)	0.074 (0.031 - 0.149)
All ages (rate per capita)	0.154 (0.130 - 0.178)	0.113 (0.095 - 0.132)	0.104 (0.088 - 0.121)	0.221 (0.170 - 0.278)	0.164 (0.126 - 0.211)	0.150 (0.116 - 0.191)	0.036 (0.015 - 0.072)	0.027 (0.011 - 0.054)	0.024 (0.011 - 0.048)
Age-standardised (rate per capita)	0.096 (0.081 - 0.112)	0.096 (0.081 - 0.112)	0.096 (0.081 - 0.112)	0.139 (0.107 - 0.176)	0.139 (0.107 - 0.177)	0.139 (0.107 - 0.177)	0.023 (0.010 - 0.045)	0.023 (0.010 - 0.046)	0.023 (0.010 - 0.045)
Total number of cases (thousands)	564 (476 - 653)	537 (453 - 625)	560 (474 - 650)	810 (624 - 1,019)	781 (597 - 1,001)	810 (622 - 1,028)	0 (0 - 0)	0 (0 - 0)	0 (0 - 0)
**Uzbekistan**									
Under-5 years (rate per capita)	0.466 (0.372 - 0.550)	0.467 (0.372 - 0.550)	0.469 (0.374 - 0.552)	0.618 (0.448 - 0.768)	0.619 (0.449 - 0.769)	0.621 (0.451 - 0.772)	0.110 (0.046 - 0.212)	0.110 (0.046 - 0.212)	0.110 (0.047 - 0.214)
5-14 years (rate per capita)	0.334 (0.269 - 0.413)	0.293 (0.235 - 0.366)	0.329 (0.264 - 0.407)	0.513 (0.364 - 0.694)	0.456 (0.318 - 0.630)	0.506 (0.357 - 0.683)	0.078 (0.033 - 0.160)	0.069 (0.028 - 0.141)	0.077 (0.032 - 0.156)
All ages (rate per capita)	0.158 (0.133 - 0.183)	0.113 (0.095 - 0.131)	0.109 (0.092 - 0.127)	0.227 (0.175 - 0.284)	0.164 (0.125 - 0.211)	0.157 (0.121 - 0.198)	0.037 (0.016 - 0.074)	0.026 (0.011 - 0.053)	0.026 (0.011 - 0.051)
Age-standardised (rate per capita)	0.096 (0.081 - 0.112)	0.096 (0.081 - 0.112)	0.096 (0.081 - 0.112)	0.139 (0.107 - 0.177)	0.139 (0.107 - 0.177)	0.139 (0.107 - 0.177)	0.023 (0.010 - 0.045)	0.023 (0.010 - 0.045)	0.023 (0.010 - 0.045)
Total number of cases (thousands)	3,238 (2,739 - 3,753)	2,934 (2,478 - 3,412)	3,271 (2,768 - 3,791)	4,652 (3,583 - 5,838)	4,275 (3,259 - 5,499)	4,708 (3,616 - 5,926)	1 (0 - 2)	1 (0 - 1)	1 (0 - 2)
**Latin America and Caribbean**									
Under-5 years (rate per capita)	0.343 (0.273 - 0.421)	0.321 (0.258 - 0.394)	0.348 (0.277 - 0.432)	0.552 (0.328 - 0.763)	0.506 (0.308 - 0.702)	0.531 (0.330 - 0.728)	0.101 (0.045 - 0.201)	0.097 (0.042 - 0.190)	0.104 (0.045 - 0.208)
5-14 years (rate per capita)	0.266 (0.202 - 0.335)	0.251 (0.190 - 0.313)	0.253 (0.192 - 0.316)	0.354 (0.237 - 0.528)	0.325 (0.215 - 0.487)	0.337 (0.224 - 0.504)	0.078 (0.033 - 0.158)	0.075 (0.032 - 0.152)	0.075 (0.032 - 0.150)
All ages (rate per capita)	0.109 (0.091 - 0.129)	0.083 (0.069 - 0.098)	0.074 (0.061 - 0.088)	0.157 (0.115 - 0.214)	0.117 (0.085 - 0.161)	0.104 (0.076 - 0.143)	0.032 (0.014 - 0.063)	0.025 (0.011 - 0.049)	0.022 (0.009 - 0.043)
Age-standardised (rate per capita)	0.076 (0.063 - 0.090)	0.072 (0.060 - 0.085)	0.076 (0.063 - 0.090)	0.109 (0.080 - 0.149)	0.101 (0.074 - 0.138)	0.107 (0.079 - 0.147)	0.022 (0.009 - 0.044)	0.021 (0.009 - 0.042)	0.023 (0.010 - 0.044)
Total number of cases (thousands)	43,324 (36,193 - 51,448)	42,111 (35,125 - 49,756)	42,072 (35,030 - 50,011)	62,614 (45,888 - 85,082)	59,241 (43,196 - 81,202)	59,320 (43,591 - 81,419)	13 (5 - 25)	13 (5 - 25)	13 (5 - 25)
**Central Latin America**									
Under-5 years (rate per capita)	0.328 (0.259 - 0.409)	0.326 (0.256 - 0.406)	0.330 (0.259 - 0.413)	0.551 (0.320 - 0.774)	0.550 (0.318 - 0.777)	0.555 (0.320 - 0.785)	0.120 (0.051 - 0.239)	0.119 (0.052 - 0.236)	0.121 (0.052 - 0.243)
5-14 years (rate per capita)	0.256 (0.192 - 0.327)	0.250 (0.187 - 0.318)	0.247 (0.186 - 0.314)	0.361 (0.242 - 0.538)	0.356 (0.239 - 0.529)	0.350 (0.235 - 0.524)	0.093 (0.039 - 0.198)	0.091 (0.038 - 0.189)	0.090 (0.037 - 0.191)
All ages (rate per capita)	0.110 (0.091 - 0.132)	0.089 (0.074 - 0.107)	0.076 (0.063 - 0.091)	0.167 (0.121 - 0.230)	0.137 (0.099 - 0.188)	0.116 (0.084 - 0.161)	0.040 (0.017 - 0.081)	0.033 (0.014 - 0.066)	0.028 (0.012 - 0.057)
Age-standardised (rate per capita)	0.073 (0.060 - 0.088)	0.072 (0.060 - 0.086)	0.073 (0.060 - 0.087)	0.111 (0.080 - 0.152)	0.110 (0.080 - 0.151)	0.111 (0.080 - 0.153)	0.027 (0.011 - 0.054)	0.026 (0.011 - 0.053)	0.027 (0.011 - 0.054)
Total number of cases (thousands)	18,580 (15,385 - 22,342)	19,569 (16,218 - 23,471)	19,164 (15,869 - 23,033)	28,270 (20,402 - 38,785)	29,920 (21,686 - 41,090)	29,198 (21,062 - 40,477)	7 (3 - 14)	7 (3 - 15)	7 (3 - 14)
**Colombia**									
Under-5 years (rate per capita)	0.314 (0.245 - 0.387)	0.315 (0.246 - 0.389)	0.316 (0.247 - 0.390)	0.548 (0.307 - 0.784)	0.550 (0.308 - 0.787)	0.551 (0.309 - 0.789)	0.120 (0.051 - 0.249)	0.120 (0.050 - 0.242)	0.120 (0.051 - 0.243)
5-14 years (rate per capita)	0.259 (0.188 - 0.334)	0.245 (0.177 - 0.316)	0.245 (0.177 - 0.316)	0.351 (0.229 - 0.528)	0.333 (0.216 - 0.507)	0.333 (0.216 - 0.507)	0.098 (0.041 - 0.208)	0.093 (0.039 - 0.195)	0.093 (0.038 - 0.195)
All ages (rate per capita)	0.102 (0.083 - 0.123)	0.078 (0.063 - 0.094)	0.065 (0.053 - 0.079)	0.153 (0.108 - 0.214)	0.117 (0.083 - 0.164)	0.098 (0.070 - 0.137)	0.039 (0.016 - 0.080)	0.030 (0.013 - 0.061)	0.025 (0.010 - 0.050)
Age-standardised (rate per capita)	0.072 (0.058 - 0.086)	0.072 (0.058 - 0.086)	0.072 (0.058 - 0.086)	0.108 (0.076 - 0.151)	0.108 (0.076 - 0.151)	0.108 (0.076 - 0.151)	0.027 (0.012 - 0.057)	0.027 (0.012 - 0.056)	0.027 (0.012 - 0.055)
Total number of cases (thousands)	3,487 (2,840 - 4,212)	3,359 (2,729 - 4,068)	3,145 (2,552 - 3,817)	5,266 (3,721 - 7,331)	5,065 (3,582 - 7,085)	4,734 (3,356 - 6,620)	1 (1 - 3)	1 (1 - 3)	1 (1 - 2)
**Costa Rica**									
Under-5 years (rate per capita)	0.313 (0.244 - 0.386)	0.316 (0.246 - 0.389)	0.316 (0.246 - 0.389)	0.546 (0.306 - 0.782)	0.551 (0.309 - 0.788)	0.551 (0.309 - 0.788)	0.119 (0.050 - 0.240)	0.120 (0.049 - 0.240)	0.120 (0.051 - 0.242)
5-14 years (rate per capita)	0.266 (0.192 - 0.342)	0.245 (0.178 - 0.317)	0.249 (0.180 - 0.322)	0.360 (0.235 - 0.538)	0.333 (0.217 - 0.508)	0.338 (0.220 - 0.512)	0.101 (0.042 - 0.210)	0.093 (0.039 - 0.196)	0.095 (0.039 - 0.200)
All ages (rate per capita)	0.099 (0.081 - 0.120)	0.073 (0.060 - 0.089)	0.060 (0.049 - 0.073)	0.150 (0.106 - 0.209)	0.110 (0.078 - 0.154)	0.091 (0.064 - 0.127)	0.038 (0.016 - 0.078)	0.028 (0.012 - 0.057)	0.023 (0.010 - 0.047)
Age-standardised (rate per capita)	0.072 (0.058 - 0.086)	0.072 (0.058 - 0.086)	0.072 (0.058 - 0.086)	0.108 (0.076 - 0.151)	0.108 (0.076 - 0.151)	0.108 (0.076 - 0.151)	0.027 (0.012 - 0.056)	0.027 (0.012 - 0.055)	0.027 (0.011 - 0.056)
Total number of cases (thousands)	308 (251 - 370)	312 (253 - 379)	291 (236 - 352)	466 (329 - 647)	469 (332 - 655)	438 (310 - 612)	0 (0 - 0)	0 (0 - 0)	0 (0 - 0)
**El Salvador**									
Under-5 years (rate per capita)	0.314 (0.245 - 0.387)	0.319 (0.249 - 0.393)	0.316 (0.246 - 0.389)	0.538 (0.303 - 0.767)	0.546 (0.307 - 0.779)	0.541 (0.305 - 0.772)	0.074 (0.031 - 0.148)	0.075 (0.031 - 0.150)	0.074 (0.032 - 0.149)
5-14 years (rate per capita)	0.258 (0.186 - 0.332)	0.250 (0.181 - 0.322)	0.240 (0.174 - 0.311)	0.341 (0.223 - 0.515)	0.331 (0.216 - 0.503)	0.319 (0.208 - 0.490)	0.060 (0.025 - 0.128)	0.059 (0.024 - 0.121)	0.056 (0.023 - 0.118)
All ages (rate per capita)	0.112 (0.091 - 0.135)	0.092 (0.075 - 0.112)	0.072 (0.058 - 0.087)	0.167 (0.118 - 0.231)	0.136 (0.097 - 0.189)	0.106 (0.075 - 0.148)	0.026 (0.011 - 0.054)	0.022 (0.009 - 0.044)	0.017 (0.007 - 0.034)
Age-standardised (rate per capita)	0.072 (0.058 - 0.086)	0.072 (0.058 - 0.086)	0.072 (0.058 - 0.086)	0.106 (0.075 - 0.147)	0.106 (0.075 - 0.147)	0.106 (0.075 - 0.147)	0.017 (0.007 - 0.034)	0.017 (0.007 - 0.034)	0.017 (0.007 - 0.034)
Total number of cases (thousands)	593 (484 - 715)	551 (447 - 668)	441 (358 - 536)	881 (624 - 1,222)	809 (577 - 1,128)	650 (462 - 907)	0 (0 - 0)	0 (0 - 0)	0 (0 - 0)
**Guatemala**									
Under-5 years (rate per capita)	0.310 (0.242 - 0.382)	0.313 (0.244 - 0.386)	0.313 (0.244 - 0.386)	0.540 (0.303 - 0.773)	0.546 (0.306 - 0.781)	0.546 (0.306 - 0.781)	0.118 (0.050 - 0.239)	0.119 (0.050 - 0.240)	0.119 (0.050 - 0.242)
5-14 years (rate per capita)	0.268 (0.194 - 0.344)	0.262 (0.190 - 0.337)	0.252 (0.183 - 0.326)	0.363 (0.237 - 0.541)	0.355 (0.232 - 0.532)	0.343 (0.223 - 0.517)	0.101 (0.042 - 0.210)	0.099 (0.041 - 0.212)	0.096 (0.040 - 0.198)
All ages (rate per capita)	0.129 (0.106 - 0.156)	0.116 (0.095 - 0.140)	0.100 (0.082 - 0.121)	0.196 (0.139 - 0.273)	0.176 (0.124 - 0.245)	0.152 (0.107 - 0.212)	0.049 (0.021 - 0.100)	0.044 (0.019 - 0.090)	0.038 (0.016 - 0.077)

	Prevalence 1990	Prevalence 2005	Prevalence 2015	Incidence 1990	Incidence 2005	Incidence 2015	YLD rate 1990	YLD rate 2005	YLD rate 2015
Age-standardised (rate per capita)	0.072 (0.058 - 0.086)	0.072 (0.058 - 0.086)	0.072 (0.058 - 0.086)	0.108 (0.076 - 0.151)	0.108 (0.076 - 0.151)	0.108 (0.076 - 0.151)	0.027 (0.012 - 0.055)	0.027 (0.012 - 0.055)	0.027 (0.011 - 0.055)
Total number of cases (thousands)	1,191 (973 - 1,434)	1,537 (1,252 - 1,853)	1,641 (1,337 - 1,984)	1,805 (1,275 - 2,513)	2,324 (1,641 - 3,228)	2,482 (1,753 - 3,467)	0 (0 - 1)	1 (0 - 1)	1 (0 - 1)
**Honduras**									
Under-5 years (rate per capita)	0.311 (0.242 - 0.383)	0.316 (0.246 - 0.389)	0.315 (0.246 - 0.389)	0.532 (0.299 - 0.759)	0.541 (0.304 - 0.772)	0.540 (0.304 - 0.771)	0.073 (0.031 - 0.147)	0.074 (0.032 - 0.147)	0.074 (0.032 - 0.147)
5-14 years (rate per capita)	0.267 (0.194 - 0.344)	0.256 (0.185 - 0.330)	0.245 (0.177 - 0.317)	0.354 (0.232 - 0.529)	0.339 (0.222 - 0.513)	0.325 (0.212 - 0.497)	0.063 (0.026 - 0.130)	0.060 (0.025 - 0.126)	0.057 (0.024 - 0.119)
All ages (rate per capita)	0.130 (0.106 - 0.156)	0.110 (0.089 - 0.133)	0.086 (0.069 - 0.104)	0.193 (0.136 - 0.266)	0.162 (0.116 - 0.226)	0.126 (0.090 - 0.176)	0.030 (0.013 - 0.061)	0.026 (0.011 - 0.053)	0.020 (0.009 - 0.041)
Age-standardised (rate per capita)	0.072 (0.058 - 0.086)	0.072 (0.058 - 0.086)	0.072 (0.058 - 0.086)	0.106 (0.075 - 0.147)	0.106 (0.075 - 0.147)	0.106 (0.075 - 0.147)	0.017 (0.007 - 0.034)	0.017 (0.007 - 0.035)	0.017 (0.007 - 0.034)
Total number of cases (thousands)	637 (521 - 768)	758 (617 - 915)	692 (562 - 840)	947 (671 - 1,310)	1,119 (797 - 1,557)	1,021 (727 - 1,424)	0 (0 - 0)	0 (0 - 0)	0 (0 - 0)
**Mexico**									
Under-5 years (rate per capita)	0.344 (0.272 - 0.428)	0.337 (0.267 - 0.424)	0.345 (0.272 - 0.433)	0.559 (0.335 - 0.776)	0.553 (0.328 - 0.772)	0.563 (0.330 - 0.784)	0.131 (0.056 - 0.260)	0.128 (0.055 - 0.257)	0.131 (0.056 - 0.266)
5-14 years (rate per capita)	0.251 (0.192 - 0.319)	0.250 (0.188 - 0.315)	0.246 (0.187 - 0.309)	0.369 (0.248 - 0.553)	0.374 (0.254 - 0.548)	0.364 (0.246 - 0.544)	0.095 (0.040 - 0.202)	0.095 (0.040 - 0.198)	0.093 (0.038 - 0.191)
All ages (rate per capita)	0.111 (0.092 - 0.132)	0.091 (0.075 - 0.108)	0.077 (0.064 - 0.092)	0.170 (0.123 - 0.232)	0.141 (0.103 - 0.193)	0.119 (0.087 - 0.163)	0.042 (0.018 - 0.085)	0.034 (0.015 - 0.070)	0.029 (0.012 - 0.060)
Age-standardised (rate per capita)	0.074 (0.062 - 0.089)	0.073 (0.061 - 0.087)	0.074 (0.061 - 0.089)	0.114 (0.083 - 0.156)	0.113 (0.083 - 0.154)	0.114 (0.084 - 0.156)	0.028 (0.012 - 0.057)	0.028 (0.012 - 0.056)	0.028 (0.012 - 0.058)
Total number of cases (thousands)	9,470 (7,902 - 11,331)	9,941 (8,288 - 11,818)	9,772 (8,119 - 11,724)	14,549 (10,533 - 19,884)	15,463 (11,318 - 21,142)	15,098 (11,054 - 20,654)	4 (2 - 7)	4 (2 - 8)	4 (2 - 8)
**Nicaragua**									
Under-5 years (rate per capita)	0.313 (0.245 - 0.386)	0.315 (0.246 - 0.389)	0.316 (0.247 - 0.390)	0.537 (0.302 - 0.766)	0.540 (0.304 - 0.770)	0.542 (0.305 - 0.773)	0.074 (0.032 - 0.150)	0.074 (0.032 - 0.147)	0.074 (0.032 - 0.146)
5-14 years (rate per capita)	0.267 (0.193 - 0.343)	0.250 (0.181 - 0.323)	0.252 (0.183 - 0.325)	0.353 (0.232 - 0.529)	0.332 (0.217 - 0.504)	0.334 (0.219 - 0.507)	0.063 (0.025 - 0.130)	0.059 (0.024 - 0.122)	0.059 (0.024 - 0.125)
All ages (rate per capita)	0.130 (0.106 - 0.157)	0.098 (0.080 - 0.119)	0.082 (0.067 - 0.100)	0.193 (0.137 - 0.268)	0.144 (0.103 - 0.201)	0.122 (0.087 - 0.169)	0.031 (0.013 - 0.063)	0.023 (0.010 - 0.047)	0.019 (0.008 - 0.040)
Age-standardised (rate per capita)	0.072 (0.058 - 0.086)	0.072 (0.058 - 0.086)	0.072 (0.058 - 0.086)	0.106 (0.075 - 0.147)	0.106 (0.075 - 0.147)	0.106 (0.075 - 0.147)	0.017 (0.007 - 0.034)	0.017 (0.007 - 0.034)	0.017 (0.007 - 0.035)
Total number of cases (thousands)	542 (442 - 653)	527 (429 - 639)	501 (407 - 606)	804 (570 - 1,113)	778 (554 - 1,084)	740 (527 - 1,030)	0 (0 - 0)	0 (0 - 0)	0 (0 - 0)
**Panama**									
Under-5 years (rate per capita)	0.313 (0.245 - 0.386)	0.314 (0.245 - 0.387)	0.314 (0.245 - 0.387)	0.537 (0.302 - 0.766)	0.538 (0.303 - 0.767)	0.538 (0.303 - 0.768)	0.074 (0.031 - 0.144)	0.074 (0.032 - 0.147)	0.074 (0.032 - 0.149)
5-14 years (rate per capita)	0.254 (0.184 - 0.328)	0.257 (0.186 - 0.332)	0.254 (0.184 - 0.328)	0.337 (0.220 - 0.510)	0.341 (0.223 - 0.514)	0.337 (0.220 - 0.510)	0.060 (0.025 - 0.125)	0.060 (0.025 - 0.127)	0.060 (0.024 - 0.124)
All ages (rate per capita)	0.099 (0.080 - 0.119)	0.084 (0.068 - 0.101)	0.075 (0.061 - 0.090)	0.146 (0.104 - 0.203)	0.124 (0.088 - 0.172)	0.110 (0.078 - 0.153)	0.023 (0.010 - 0.047)	0.020 (0.008 - 0.040)	0.018 (0.007 - 0.036)
Age-standardised (rate per capita)	0.072 (0.058 - 0.086)	0.072 (0.058 - 0.086)	0.072 (0.058 - 0.086)	0.106 (0.075 - 0.147)	0.106 (0.075 - 0.147)	0.106 (0.075 - 0.147)	0.017 (0.007 - 0.034)	0.017 (0.007 - 0.034)	0.017 (0.007 - 0.034)
Total number of cases (thousands)	244 (199 - 295)	278 (226 - 336)	293 (238 - 354)	362 (257 - 502)	411 (292 - 571)	433 (308 - 602)	0 (0 - 0)	0 (0 - 0)	0 (0 - 0)
**Venezuela**									
Under-5 years (rate per capita)	0.313 (0.244 - 0.386)	0.314 (0.245 - 0.387)	0.315 (0.246 - 0.388)	0.546 (0.306 - 0.781)	0.547 (0.307 - 0.783)	0.549 (0.308 - 0.785)	0.119 (0.050 - 0.248)	0.119 (0.051 - 0.243)	0.120 (0.050 - 0.244)
5-14 years (rate per capita)	0.261 (0.189 - 0.336)	0.250 (0.181 - 0.323)	0.252 (0.182 - 0.325)	0.353 (0.231 - 0.530)	0.340 (0.221 - 0.514)	0.342 (0.223 - 0.517)	0.099 (0.041 - 0.208)	0.095 (0.039 - 0.202)	0.096 (0.040 - 0.200)
All ages (rate per capita)	0.106 (0.086 - 0.128)	0.086 (0.070 - 0.104)	0.077 (0.062 - 0.093)	0.161 (0.113 - 0.223)	0.130 (0.092 - 0.182)	0.116 (0.082 - 0.162)	0.040 (0.017 - 0.082)	0.033 (0.014 - 0.068)	0.029 (0.012 - 0.058)
Age-standardised (rate per capita)	0.072 (0.058 - 0.086)	0.072 (0.058 - 0.086)	0.072 (0.058 - 0.086)	0.108 (0.076 - 0.151)	0.108 (0.076 - 0.151)	0.108 (0.076 - 0.151)	0.027 (0.012 - 0.055)	0.027 (0.011 - 0.056)	0.027 (0.012 - 0.055)
Total number of cases (thousands)	2,106 (1,717 - 2,536)	2,306 (1,876 - 2,785)	2,388 (1,943 - 2,885)	3,191 (2,252 - 4,435)	3,481 (2,461 - 4,865)	3,602 (2,548 - 5,035)	1 (0 - 2)	1 (0 - 2)	1 (0 - 2)
**Andean Latin America**									
Under-5 years (rate per capita)	0.414 (0.326 - 0.510)	0.418 (0.329 - 0.515)	0.418 (0.329 - 0.515)	0.569 (0.382 - 0.741)	0.574 (0.386 - 0.748)	0.574 (0.386 - 0.748)	0.097 (0.041 - 0.192)	0.098 (0.041 - 0.194)	0.098 (0.041 - 0.191)
5-14 years (rate per capita)	0.296 (0.224 - 0.364)	0.290 (0.219 - 0.357)	0.291 (0.220 - 0.358)	0.362 (0.247 - 0.537)	0.355 (0.242 - 0.530)	0.356 (0.243 - 0.531)	0.069 (0.029 - 0.141)	0.068 (0.028 - 0.138)	0.068 (0.029 - 0.138)
All ages (rate per capita)	0.132 (0.111 - 0.156)	0.110 (0.092 - 0.130)	0.098 (0.082 - 0.115)	0.171 (0.128 - 0.226)	0.142 (0.107 - 0.190)	0.126 (0.094 - 0.168)	0.031 (0.013 - 0.062)	0.026 (0.011 - 0.052)	0.023 (0.010 - 0.045)
Age-standardised (rate per capita)	0.087 (0.073 - 0.103)	0.087 (0.073 - 0.103)	0.087 (0.073 - 0.103)	0.113 (0.085 - 0.150)	0.113 (0.085 - 0.150)	0.113 (0.085 - 0.150)	0.020 (0.009 - 0.041)	0.020 (0.009 - 0.041)	0.020 (0.009 - 0.041)
Total number of cases (thousands)	5,155 (4,312 - 6,084)	5,574 (4,661 - 6,593)	5,687 (4,755 - 6,726)	6,654 (5,007 - 8,799)	7,189 (5,400 - 9,592)	7,333 (5,506 - 9,781)	1 (1 - 2)	1 (1 - 3)	1 (1 - 3)
**Bolivia**									
Under-5 years (rate per capita)	0.413 (0.325 - 0.509)	0.418 (0.329 - 0.515)	0.419 (0.329 - 0.516)	0.567 (0.382 - 0.740)	0.574 (0.387 - 0.749)	0.575 (0.388 - 0.750)	0.096 (0.041 - 0.187)	0.098 (0.041 - 0.192)	0.098 (0.041 - 0.193)
5-14 years (rate per capita)	0.300 (0.227 - 0.372)	0.295 (0.224 - 0.367)	0.288 (0.218 - 0.358)	0.369 (0.250 - 0.548)	0.364 (0.246 - 0.544)	0.357 (0.240 - 0.537)	0.070 (0.029 - 0.145)	0.069 (0.029 - 0.142)	0.068 (0.028 - 0.137)
All ages (rate per capita)	0.142 (0.118 - 0.168)	0.126 (0.104 - 0.148)	0.109 (0.091 - 0.129)	0.184 (0.139 - 0.241)	0.163 (0.122 - 0.214)	0.141 (0.106 - 0.187)	0.033 (0.014 - 0.066)	0.029 (0.013 - 0.059)	0.026 (0.011 - 0.051)
Age-standardised (rate per capita)	0.087 (0.073 - 0.103)	0.087 (0.073 - 0.103)	0.087 (0.073 - 0.103)	0.113 (0.085 - 0.150)	0.113 (0.085 - 0.150)	0.113 (0.085 - 0.150)	0.020 (0.009 - 0.041)	0.020 (0.009 - 0.041)	0.020 (0.009 - 0.040)
Total number of cases (thousands)	981 (815 - 1,158)	1,151 (957 - 1,360)	1,176 (980 - 1,390)	1,271 (957 - 1,663)	1,491 (1,120 - 1,963)	1,523 (1,142 - 2,016)	0 (0 - 0)	0 (0 - 1)	0 (0 - 1)
**Ecuador**									
Under-5 years (rate per capita)	0.412 (0.326 - 0.510)	0.414 (0.328 - 0.513)	0.415 (0.328 - 0.514)	0.568 (0.379 - 0.737)	0.571 (0.381 - 0.742)	0.572 (0.381 - 0.743)	0.096 (0.041 - 0.192)	0.097 (0.041 - 0.195)	0.097 (0.041 - 0.193)
5-14 years (rate per capita)	0.301 (0.228 - 0.370)	0.294 (0.222 - 0.362)	0.296 (0.223 - 0.364)	0.360 (0.248 - 0.527)	0.353 (0.243 - 0.519)	0.354 (0.244 - 0.520)	0.070 (0.029 - 0.141)	0.069 (0.029 - 0.137)	0.069 (0.029 - 0.138)
All ages (rate per capita)	0.131 (0.110 - 0.154)	0.110 (0.092 - 0.129)	0.098 (0.082 - 0.115)	0.167 (0.126 - 0.221)	0.140 (0.105 - 0.186)	0.125 (0.094 - 0.166)	0.031 (0.013 - 0.061)	0.026 (0.011 - 0.051)	0.023 (0.010 - 0.045)
Age-standardised (rate per capita)	0.088 (0.074 - 0.103)	0.088 (0.074 - 0.103)	0.088 (0.074 - 0.103)	0.112 (0.084 - 0.149)	0.112 (0.084 - 0.149)	0.112 (0.084 - 0.149)	0.020 (0.009 - 0.041)	0.021 (0.009 - 0.041)	0.021 (0.009 - 0.040)
Total number of cases (thousands)	1,343 (1,129 - 1,577)	1,511 (1,269 - 1,773)	1,581 (1,329 - 1,855)	1,715 (1,286 - 2,264)	1,925 (1,445 - 2,562)	2,015 (1,513 - 2,678)	0 (0 - 1)	0 (0 - 1)	0 (0 - 1)
**Peru**									
Under-5 years (rate per capita)	0.415 (0.326 - 0.511)	0.420 (0.330 - 0.517)	0.420 (0.330 - 0.517)	0.570 (0.384 - 0.744)	0.576 (0.388 - 0.752)	0.576 (0.388 - 0.751)	0.097 (0.041 - 0.195)	0.099 (0.041 - 0.196)	0.098 (0.042 - 0.194)
5-14 years (rate per capita)	0.292 (0.221 - 0.362)	0.285 (0.215 - 0.354)	0.289 (0.219 - 0.359)	0.360 (0.243 - 0.540)	0.353 (0.237 - 0.533)	0.357 (0.241 - 0.537)	0.068 (0.029 - 0.143)	0.067 (0.028 - 0.139)	0.068 (0.029 - 0.139)
All ages (rate per capita)	0.130 (0.108 - 0.153)	0.105 (0.088 - 0.125)	0.093 (0.078 - 0.110)	0.168 (0.126 - 0.221)	0.136 (0.102 - 0.181)	0.121 (0.091 - 0.160)	0.030 (0.013 - 0.060)	0.025 (0.010 - 0.049)	0.022 (0.009 - 0.044)
Age-standardised (rate per capita)	0.087 (0.073 - 0.103)	0.087 (0.073 - 0.103)	0.087 (0.073 - 0.103)	0.113 (0.085 - 0.150)	0.113 (0.085 - 0.150)	0.113 (0.085 - 0.150)	0.020 (0.009 - 0.041)	0.020 (0.009 - 0.041)	0.020 (0.009 - 0.041)
Total number of cases (thousands)	2,831 (2,354 - 3,348)	2,912 (2,425 - 3,444)	2,930 (2,442 - 3,463)	3,669 (2,753 - 4,838)	3,773 (2,827 - 5,009)	3,795 (2,847 - 5,024)	1 (0 - 1)	1 (0 - 1)	1 (0 - 1)
**Caribbean**									
Under-5 years (rate per capita)	0.442 (0.345 - 0.533)	0.444 (0.347 - 0.535)	0.445 (0.347 - 0.536)	0.586 (0.403 - 0.753)	0.588 (0.405 - 0.756)	0.588 (0.405 - 0.755)	0.125 (0.054 - 0.243)	0.122 (0.052 - 0.241)	0.121 (0.052 - 0.237)
5-14 years (rate per capita)	0.333 (0.256 - 0.407)	0.321 (0.247 - 0.394)	0.319 (0.245 - 0.392)	0.376 (0.247 - 0.542)	0.363 (0.238 - 0.530)	0.361 (0.236 - 0.529)	0.095 (0.040 - 0.188)	0.089 (0.038 - 0.175)	0.087 (0.037 - 0.172)
All ages (rate per capita)	0.122 (0.102 - 0.142)	0.104 (0.087 - 0.121)	0.092 (0.077 - 0.107)	0.148 (0.113 - 0.191)	0.125 (0.095 - 0.162)	0.111 (0.084 - 0.143)	0.035 (0.015 - 0.067)	0.029 (0.012 - 0.055)	0.025 (0.011 - 0.048)
Age-standardised (rate per capita)	0.096 (0.080 - 0.111)	0.096 (0.080 - 0.111)	0.096 (0.080 - 0.111)	0.116 (0.088 - 0.149)	0.116 (0.088 - 0.149)	0.115 (0.088 - 0.149)	0.027 (0.012 - 0.052)	0.026 (0.011 - 0.051)	0.026 (0.011 - 0.050)
Total number of cases (thousands)	4,369 (3,663 - 5,084)	4,338 (3,629 - 5,057)	4,145 (3,463 - 4,836)	5,300 (4,047 - 6,833)	5,244 (3,970 - 6,761)	5,014 (3,798 - 6,465)	1 (1 - 2)	1 (1 - 2)	1 (0 - 2)
**Antigua and Barbuda**									
Under-5 years (rate per capita)	0.445 (0.347 - 0.537)	0.445 (0.347 - 0.536)	0.444 (0.346 - 0.535)	0.603 (0.413 - 0.777)	0.603 (0.412 - 0.776)	0.601 (0.411 - 0.774)	0.169 (0.071 - 0.344)	0.169 (0.070 - 0.340)	0.168 (0.069 - 0.341)
5-14 years (rate per capita)	0.362 (0.279 - 0.440)	0.323 (0.247 - 0.396)	0.314 (0.240 - 0.386)	0.414 (0.274 - 0.584)	0.374 (0.244 - 0.545)	0.365 (0.237 - 0.537)	0.137 (0.058 - 0.279)	0.122 (0.051 - 0.245)	0.119 (0.051 - 0.241)
All ages (rate per capita)	0.113 (0.095 - 0.131)	0.103 (0.086 - 0.120)	0.086 (0.072 - 0.101)	0.138 (0.105 - 0.180)	0.127 (0.096 - 0.165)	0.107 (0.081 - 0.139)	0.043 (0.018 - 0.086)	0.039 (0.017 - 0.077)	0.033 (0.014 - 0.065)
Age-standardised (rate per capita)	0.095 (0.080 - 0.111)	0.095 (0.080 - 0.111)	0.095 (0.080 - 0.111)	0.118 (0.089 - 0.153)	0.118 (0.090 - 0.153)	0.118 (0.090 - 0.153)	0.036 (0.016 - 0.073)	0.036 (0.015 - 0.072)	0.036 (0.015 - 0.072)
Total number of cases (thousands)	7 (6 - 8)	8 (7 - 10)	8 (7 - 9)	8 (6 - 11)	11 (8 - 14)	10 (7 - 13)	0 (0 - 0)	0 (0 - 0)	0 (0 - 0)
**The Bahamas**									
Under-5 years (rate per capita)	0.442 (0.345 - 0.533)	0.445 (0.347 - 0.536)	0.443 (0.345 - 0.534)	0.580 (0.400 - 0.745)	0.583 (0.403 - 0.749)	0.581 (0.401 - 0.746)	0.104 (0.043 - 0.206)	0.104 (0.045 - 0.201)	0.104 (0.044 - 0.206)
5-14 years (rate per capita)	0.340 (0.261 - 0.415)	0.312 (0.239 - 0.384)	0.332 (0.255 - 0.407)	0.378 (0.249 - 0.543)	0.351 (0.228 - 0.516)	0.371 (0.244 - 0.537)	0.079 (0.033 - 0.160)	0.073 (0.031 - 0.148)	0.078 (0.033 - 0.157)
All ages (rate per capita)	0.123 (0.103 - 0.143)	0.088 (0.074 - 0.103)	0.077 (0.065 - 0.090)	0.146 (0.111 - 0.188)	0.105 (0.080 - 0.137)	0.092 (0.070 - 0.119)	0.029 (0.012 - 0.057)	0.021 (0.009 - 0.041)	0.018 (0.008 - 0.036)

	Prevalence 1990	Prevalence 2005	Prevalence 2015	Incidence 1990	Incidence 2005	Incidence 2015	YLD rate 1990	YLD rate 2005	YLD rate 2015
Age-standardised (rate per capita)	0.095 (0.080 - 0.111)	0.095 (0.080 - 0.111)	0.095 (0.080 - 0.111)	0.114 (0.087 - 0.147)	0.114 (0.087 - 0.147)	0.114 (0.087 - 0.147)	0.022 (0.010 - 0.045)	0.022 (0.010 - 0.044)	0.022 (0.009 - 0.044)
Total number of cases (thousands)	32 (26 - 37)	29 (24 - 34)	30 (25 - 35)	38 (29 - 48)	34 (26 - 45)	36 (27 - 46)	0 (0 - 0)	0 (0 - 0)	0 (0 - 0)
**Barbados**									
Under-5 years (rate per capita)	0.446 (0.348 - 0.538)	0.446 (0.348 - 0.538)	0.445 (0.347 - 0.537)	0.604 (0.413 - 0.777)	0.604 (0.413 - 0.777)	0.603 (0.412 - 0.776)	0.169 (0.071 - 0.341)	0.169 (0.070 - 0.341)	0.169 (0.070 - 0.343)
5-14 years (rate per capita)	0.318 (0.244 - 0.391)	0.314 (0.241 - 0.387)	0.316 (0.242 - 0.389)	0.370 (0.241 - 0.542)	0.366 (0.238 - 0.538)	0.368 (0.239 - 0.541)	0.121 (0.050 - 0.245)	0.119 (0.050 - 0.243)	0.120 (0.051 - 0.243)
All ages (rate per capita)	0.087 (0.073 - 0.102)	0.073 (0.061 - 0.086)	0.069 (0.057 - 0.080)	0.108 (0.082 - 0.140)	0.091 (0.068 - 0.118)	0.085 (0.064 - 0.110)	0.033 (0.014 - 0.066)	0.028 (0.012 - 0.056)	0.026 (0.011 - 0.052)
Age-standardised (rate per capita)	0.095 (0.080 - 0.111)	0.095 (0.080 - 0.111)	0.095 (0.080 - 0.111)	0.118 (0.090 - 0.153)	0.118 (0.090 - 0.153)	0.119 (0.090 - 0.153)	0.036 (0.015 - 0.073)	0.036 (0.016 - 0.073)	0.036 (0.015 - 0.073)
Total number of cases (thousands)	23 (19 - 26)	20 (17 - 23)	19 (16 - 23)	28 (21 - 36)	25 (19 - 32)	24 (18 - 31)	0 (0 - 0)	0 (0 - 0)	0 (0 - 0)
**Belize**									
Under-5 years (rate per capita)	0.441 (0.344 - 0.532)	0.443 (0.346 - 0.535)	0.440 (0.343 - 0.531)	0.578 (0.399 - 0.743)	0.581 (0.401 - 0.746)	0.577 (0.399 - 0.742)	0.103 (0.044 - 0.205)	0.104 (0.045 - 0.206)	0.103 (0.043 - 0.200)
5-14 years (rate per capita)	0.343 (0.263 - 0.418)	0.336 (0.258 - 0.411)	0.315 (0.241 - 0.388)	0.381 (0.252 - 0.546)	0.375 (0.247 - 0.540)	0.354 (0.230 - 0.520)	0.080 (0.035 - 0.163)	0.079 (0.033 - 0.159)	0.074 (0.031 - 0.148)
All ages (rate per capita)	0.166 (0.140 - 0.194)	0.144 (0.120 - 0.167)	0.115 (0.096 - 0.135)	0.200 (0.153 - 0.256)	0.171 (0.130 - 0.221)	0.139 (0.105 - 0.179)	0.039 (0.017 - 0.077)	0.034 (0.015 - 0.068)	0.027 (0.012 - 0.053)
Age-standardised (rate per capita)	0.095 (0.080 - 0.111)	0.095 (0.080 - 0.111)	0.095 (0.080 - 0.111)	0.114 (0.087 - 0.147)	0.114 (0.087 - 0.147)	0.114 (0.087 - 0.147)	0.022 (0.010 - 0.045)	0.022 (0.010 - 0.045)	0.022 (0.010 - 0.044)
Total number of cases (thousands)	31 (26 - 37)	40 (34 - 47)	41 (35 - 48)	38 (29 - 48)	48 (37 - 62)	50 (38 - 64)	0 (0 - 0)	0 (0 - 0)	0 (0 - 0)
**Bermuda**									
Under-5 years (rate per capita)	0.445 (0.347 - 0.537)	0.444 (0.346 - 0.536)	0.443 (0.346 - 0.535)	0.603 (0.413 - 0.776)	0.602 (0.412 - 0.775)	0.601 (0.411 - 0.773)	0.169 (0.071 - 0.344)	0.169 (0.070 - 0.335)	0.168 (0.070 - 0.342)
5-14 years (rate per capita)	0.333 (0.256 - 0.407)	0.320 (0.245 - 0.393)	0.318 (0.243 - 0.391)	0.385 (0.252 - 0.555)	0.372 (0.242 - 0.544)	0.370 (0.241 - 0.542)	0.126 (0.053 - 0.259)	0.121 (0.051 - 0.246)	0.121 (0.051 - 0.244)
All ages (rate per capita)	0.108 (0.090 - 0.126)	0.093 (0.077 - 0.109)	0.081 (0.068 - 0.096)	0.132 (0.098 - 0.173)	0.113 (0.084 - 0.149)	0.099 (0.074 - 0.131)	0.041 (0.017 - 0.082)	0.035 (0.015 - 0.070)	0.031 (0.013 - 0.062)
Age-standardised (rate per capita)	0.095 (0.080 - 0.111)	0.095 (0.080 - 0.111)	0.095 (0.080 - 0.111)	0.118 (0.090 - 0.153)	0.118 (0.090 - 0.153)	0.118 (0.090 - 0.153)	0.036 (0.015 - 0.073)	0.036 (0.016 - 0.072)	0.036 (0.015 - 0.073)
Total number of cases (thousands)	6 (5 - 7)	6 (5 - 7)	5 (5 - 6)	7 (5 - 9)	7 (5 - 9)	7 (5 - 9)	0 (0 - 0)	0 (0 - 0)	0 (0 - 0)
**Cuba**									
Under-5 years (rate per capita)	0.444 (0.346 - 0.536)	0.448 (0.350 - 0.541)	0.447 (0.349 - 0.539)	0.602 (0.412 - 0.775)	0.608 (0.416 - 0.782)	0.606 (0.415 - 0.780)	0.168 (0.071 - 0.343)	0.170 (0.069 - 0.345)	0.170 (0.070 - 0.342)
5-14 years (rate per capita)	0.320 (0.245 - 0.393)	0.314 (0.240 - 0.386)	0.311 (0.238 - 0.383)	0.372 (0.242 - 0.544)	0.365 (0.237 - 0.536)	0.362 (0.235 - 0.532)	0.121 (0.051 - 0.247)	0.119 (0.050 - 0.240)	0.118 (0.050 - 0.241)
All ages (rate per capita)	0.084 (0.070 - 0.098)	0.070 (0.058 - 0.081)	0.058 (0.048 - 0.068)	0.105 (0.080 - 0.135)	0.086 (0.065 - 0.112)	0.072 (0.054 - 0.093)	0.032 (0.014 - 0.064)	0.026 (0.011 - 0.053)	0.022 (0.009 - 0.044)
Age-standardised (rate per capita)	0.095 (0.080 - 0.111)	0.095 (0.080 - 0.111)	0.095 (0.080 - 0.111)	0.119 (0.090 - 0.153)	0.119 (0.090 - 0.153)	0.119 (0.090 - 0.153)	0.036 (0.015 - 0.072)	0.036 (0.016 - 0.073)	0.036 (0.015 - 0.073)
Total number of cases (thousands)	889 (744 - 1,037)	785 (655 - 916)	659 (550 - 770)	1,109 (841 - 1,431)	973 (732 - 1,259)	818 (616 - 1,059)	0 (0 - 1)	0 (0 - 1)	0 (0 - 1)
**Dominica**									
Under-5 years (rate per capita)	0.445 (0.347 - 0.536)	0.446 (0.348 - 0.538)	0.444 (0.346 - 0.535)	0.583 (0.403 - 0.749)	0.584 (0.404 - 0.751)	0.582 (0.402 - 0.748)	0.104 (0.044 - 0.205)	0.104 (0.044 - 0.202)	0.104 (0.044 - 0.205)
5-14 years (rate per capita)	0.333 (0.256 - 0.407)	0.320 (0.245 - 0.393)	0.318 (0.243 - 0.391)	0.371 (0.244 - 0.537)	0.359 (0.234 - 0.525)	0.357 (0.233 - 0.522)	0.078 (0.033 - 0.160)	0.075 (0.032 - 0.152)	0.074 (0.032 - 0.152)
All ages (rate per capita)	0.122 (0.103 - 0.143)	0.099 (0.082 - 0.115)	0.089 (0.074 - 0.104)	0.147 (0.112 - 0.189)	0.117 (0.089 - 0.153)	0.106 (0.080 - 0.138)	0.029 (0.012 - 0.058)	0.023 (0.010 - 0.046)	0.021 (0.009 - 0.041)
Age-standardised (rate per capita)	0.095 (0.080 - 0.111)	0.095 (0.080 - 0.111)	0.095 (0.080 - 0.111)	0.114 (0.087 - 0.147)	0.114 (0.087 - 0.147)	0.114 (0.087 - 0.147)	0.022 (0.010 - 0.045)	0.022 (0.010 - 0.044)	0.022 (0.009 - 0.044)
Total number of cases (thousands)	8 (7 - 10)	7 (6 - 8)	6 (5 - 7)	10 (8 - 13)	8 (6 - 11)	8 (6 - 10)	0 (0 - 0)	0 (0 - 0)	0 (0 - 0)
**Dominican Republic**									
Under-5 years (rate per capita)	0.441 (0.344 - 0.532)	0.443 (0.345 - 0.534)	0.445 (0.347 - 0.536)	0.578 (0.399 - 0.743)	0.580 (0.401 - 0.746)	0.583 (0.402 - 0.749)	0.103 (0.044 - 0.202)	0.104 (0.044 - 0.201)	0.104 (0.045 - 0.205)
5-14 years (rate per capita)	0.333 (0.256 - 0.407)	0.321 (0.246 - 0.395)	0.324 (0.248 - 0.397)	0.371 (0.244 - 0.537)	0.360 (0.235 - 0.526)	0.362 (0.237 - 0.528)	0.078 (0.033 - 0.160)	0.075 (0.032 - 0.152)	0.076 (0.032 - 0.150)
All ages (rate per capita)	0.144 (0.121 - 0.168)	0.121 (0.101 - 0.141)	0.109 (0.091 - 0.127)	0.173 (0.132 - 0.222)	0.145 (0.110 - 0.187)	0.131 (0.099 - 0.168)	0.034 (0.015 - 0.067)	0.028 (0.012 - 0.056)	0.026 (0.011 - 0.051)
Age-standardised (rate per capita)	0.095 (0.080 - 0.111)	0.095 (0.080 - 0.111)	0.095 (0.080 - 0.111)	0.114 (0.087 - 0.147)	0.114 (0.087 - 0.147)	0.114 (0.087 - 0.147)	0.022 (0.010 - 0.044)	0.022 (0.010 - 0.045)	0.022 (0.010 - 0.044)
Total number of cases (thousands)	1,038 (869 - 1,209)	1,116 (933 - 1,302)	1,150 (961 - 1,341)	1,244 (948 - 1,600)	1,339 (1,017 - 1,725)	1,378 (1,046 - 1,774)	0 (0 - 0)	0 (0 - 1)	0 (0 - 1)
**Grenada**									
Under-5 years (rate per capita)	0.450 (0.351 - 0.542)	0.444 (0.346 - 0.535)	0.444 (0.346 - 0.536)	0.590 (0.407 - 0.758)	0.582 (0.402 - 0.748)	0.582 (0.402 - 0.749)	0.105 (0.045 - 0.207)	0.104 (0.044 - 0.205)	0.104 (0.043 - 0.205)
5-14 years (rate per capita)	0.359 (0.277 - 0.437)	0.304 (0.232 - 0.375)	0.329 (0.252 - 0.403)	0.397 (0.264 - 0.565)	0.342 (0.223 - 0.505)	0.368 (0.241 - 0.533)	0.084 (0.035 - 0.168)	0.071 (0.030 - 0.145)	0.077 (0.032 - 0.156)
All ages (rate per capita)	0.154 (0.129 - 0.179)	0.105 (0.087 - 0.122)	0.098 (0.082 - 0.115)	0.184 (0.140 - 0.234)	0.126 (0.095 - 0.164)	0.118 (0.090 - 0.152)	0.036 (0.015 - 0.070)	0.025 (0.010 - 0.049)	0.023 (0.010 - 0.045)
Age-standardised (rate per capita)	0.095 (0.080 - 0.111)	0.095 (0.080 - 0.111)	0.095 (0.080 - 0.111)	0.114 (0.087 - 0.147)	0.114 (0.087 - 0.147)	0.114 (0.087 - 0.147)	0.022 (0.010 - 0.044)	0.022 (0.010 - 0.045)	0.022 (0.010 - 0.044)
Total number of cases (thousands)	15 (12 - 17)	11 (9 - 13)	11 (9 - 12)	18 (13 - 23)	13 (10 - 17)	13 (10 - 16)	0 (0 - 0)	0 (0 - 0)	0 (0 - 0)
**Guyana**									
Under-5 years (rate per capita)	0.445 (0.347 - 0.537)	0.450 (0.351 - 0.543)	0.441 (0.344 - 0.532)	0.584 (0.403 - 0.750)	0.590 (0.408 - 0.758)	0.578 (0.399 - 0.743)	0.104 (0.045 - 0.204)	0.105 (0.045 - 0.204)	0.103 (0.044 - 0.200)
5-14 years (rate per capita)	0.305 (0.233 - 0.377)	0.332 (0.255 - 0.406)	0.273 (0.207 - 0.341)	0.343 (0.223 - 0.505)	0.371 (0.244 - 0.537)	0.312 (0.199 - 0.472)	0.071 (0.030 - 0.142)	0.078 (0.033 - 0.158)	0.064 (0.027 - 0.127)
All ages (rate per capita)	0.136 (0.113 - 0.159)	0.136 (0.114 - 0.159)	0.095 (0.079 - 0.111)	0.166 (0.126 - 0.214)	0.162 (0.122 - 0.210)	0.115 (0.086 - 0.151)	0.032 (0.014 - 0.064)	0.032 (0.014 - 0.063)	0.022 (0.010 - 0.044)
Age-standardised (rate per capita)	0.095 (0.080 - 0.111)	0.095 (0.080 - 0.111)	0.095 (0.080 - 0.111)	0.114 (0.087 - 0.147)	0.114 (0.087 - 0.147)	0.114 (0.087 - 0.147)	0.022 (0.010 - 0.045)	0.022 (0.009 - 0.044)	0.022 (0.010 - 0.044)
Total number of cases (thousands)	102 (85 - 119)	101 (84 - 118)	73 (61 - 86)	124 (95 - 160)	120 (91 - 156)	88 (67 - 116)	0 (0 - 0)	0 (0 - 0)	0 (0 - 0)
**Haiti**									
Under-5 years (rate per capita)	0.437 (0.341 - 0.527)	0.441 (0.344 - 0.531)	0.442 (0.345 - 0.533)	0.573 (0.396 - 0.736)	0.578 (0.399 - 0.742)	0.580 (0.400 - 0.745)	0.102 (0.042 - 0.199)	0.103 (0.044 - 0.204)	0.103 (0.043 - 0.203)
5-14 years (rate per capita)	0.352 (0.271 - 0.428)	0.328 (0.251 - 0.401)	0.326 (0.250 - 0.400)	0.390 (0.259 - 0.555)	0.366 (0.240 - 0.531)	0.364 (0.239 - 0.529)	0.082 (0.034 - 0.169)	0.077 (0.032 - 0.157)	0.076 (0.032 - 0.153)
All ages (rate per capita)	0.166 (0.139 - 0.193)	0.140 (0.117 - 0.163)	0.124 (0.103 - 0.144)	0.198 (0.151 - 0.254)	0.167 (0.127 - 0.215)	0.148 (0.113 - 0.191)	0.039 (0.016 - 0.077)	0.033 (0.014 - 0.065)	0.029 (0.012 - 0.057)
Age-standardised (rate per capita)	0.095 (0.080 - 0.111)	0.095 (0.080 - 0.111)	0.095 (0.080 - 0.111)	0.114 (0.087 - 0.147)	0.114 (0.087 - 0.147)	0.114 (0.087 - 0.147)	0.022 (0.009 - 0.044)	0.022 (0.010 - 0.044)	0.022 (0.009 - 0.044)
Total number of cases (thousands)	1,176 (988 - 1,368)	1,294 (1,082 - 1,508)	1,327 (1,109 - 1,546)	1,406 (1,074 - 1,801)	1,551 (1,179 - 1,993)	1,590 (1,208 - 2,045)	0 (0 - 1)	0 (0 - 1)	0 (0 - 1)
**Jamaica**									
Under-5 years (rate per capita)	0.446 (0.347 - 0.537)	0.447 (0.349 - 0.539)	0.445 (0.347 - 0.537)	0.584 (0.403 - 0.751)	0.586 (0.405 - 0.753)	0.584 (0.403 - 0.750)	0.104 (0.045 - 0.203)	0.104 (0.044 - 0.205)	0.104 (0.045 - 0.201)
5-14 years (rate per capita)	0.333 (0.256 - 0.407)	0.322 (0.246 - 0.395)	0.308 (0.235 - 0.380)	0.371 (0.244 - 0.537)	0.360 (0.236 - 0.526)	0.347 (0.225 - 0.511)	0.078 (0.033 - 0.157)	0.075 (0.032 - 0.155)	0.072 (0.030 - 0.147)
All ages (rate per capita)	0.132 (0.111 - 0.154)	0.111 (0.093 - 0.129)	0.087 (0.073 - 0.102)	0.159 (0.121 - 0.204)	0.132 (0.100 - 0.171)	0.105 (0.080 - 0.136)	0.031 (0.013 - 0.061)	0.026 (0.011 - 0.052)	0.020 (0.009 - 0.041)
Age-standardised (rate per capita)	0.095 (0.080 - 0.111)	0.095 (0.080 - 0.111)	0.095 (0.080 - 0.111)	0.114 (0.087 - 0.147)	0.114 (0.087 - 0.147)	0.114 (0.087 - 0.147)	0.022 (0.010 - 0.044)	0.022 (0.010 - 0.044)	0.022 (0.010 - 0.044)
Total number of cases (thousands)	318 (266 - 370)	298 (249 - 348)	247 (206 - 289)	381 (290 - 490)	356 (269 - 461)	298 (226 - 384)	0 (0 - 0)	0 (0 - 0)	0 (0 - 0)
**Puerto Rico**									
Under-5 years (rate per capita)	0.445 (0.347 - 0.537)	0.449 (0.350 - 0.541)	0.447 (0.348 - 0.539)	0.603 (0.413 - 0.776)	0.608 (0.416 - 0.782)	0.606 (0.414 - 0.780)	0.169 (0.071 - 0.341)	0.170 (0.071 - 0.343)	0.170 (0.070 - 0.348)
5-14 years (rate per capita)	0.310 (0.237 - 0.382)	0.312 (0.238 - 0.384)	0.309 (0.236 - 0.381)	0.361 (0.234 - 0.531)	0.363 (0.236 - 0.533)	0.361 (0.234 - 0.529)	0.118 (0.050 - 0.240)	0.118 (0.051 - 0.244)	0.117 (0.049 - 0.238)
All ages (rate per capita)	0.097 (0.081 - 0.114)	0.079 (0.066 - 0.092)	0.067 (0.056 - 0.078)	0.121 (0.091 - 0.156)	0.097 (0.073 - 0.126)	0.083 (0.062 - 0.107)	0.037 (0.016 - 0.074)	0.030 (0.013 - 0.059)	0.025 (0.011 - 0.051)
Age-standardised (rate per capita)	0.095 (0.080 - 0.111)	0.095 (0.080 - 0.111)	0.095 (0.080 - 0.111)	0.118 (0.090 - 0.153)	0.119 (0.090 - 0.153)	0.119 (0.090 - 0.153)	0.036 (0.015 - 0.072)	0.036 (0.016 - 0.072)	0.036 (0.015 - 0.073)
Total number of cases (thousands)	344 (287 - 402)	296 (247 - 345)	246 (205 - 287)	428 (322 - 552)	367 (276 - 476)	305 (230 - 395)	0 (0 - 0)	0 (0 - 0)	0 (0 - 0)
**Saint Lucia**									
Under-5 years (rate per capita)	0.445 (0.347 - 0.537)	0.446 (0.347 - 0.538)	0.446 (0.348 - 0.538)	0.603 (0.413 - 0.776)	0.604 (0.413 - 0.777)	0.604 (0.413 - 0.777)	0.169 (0.069 - 0.338)	0.169 (0.070 - 0.345)	0.169 (0.072 - 0.334)
5-14 years (rate per capita)	0.332 (0.255 - 0.406)	0.307 (0.234 - 0.378)	0.311 (0.238 - 0.383)	0.384 (0.251 - 0.554)	0.358 (0.232 - 0.526)	0.362 (0.235 - 0.533)	0.125 (0.053 - 0.255)	0.116 (0.049 - 0.239)	0.118 (0.050 - 0.241)
All ages (rate per capita)	0.139 (0.117 - 0.162)	0.100 (0.083 - 0.117)	0.082 (0.069 - 0.096)	0.173 (0.132 - 0.223)	0.124 (0.093 - 0.161)	0.102 (0.077 - 0.132)	0.053 (0.022 - 0.106)	0.038 (0.016 - 0.077)	0.031 (0.013 - 0.062)

	Prevalence 1990	Prevalence 2005	Prevalence 2015	Incidence 1990	Incidence 2005	Incidence 2015	YLD rate 1990	YLD rate 2005	YLD rate 2015
Age-standardised (rate per capita)	0.095 (0.080 - 0.111)	0.095 (0.080 - 0.111)	0.095 (0.080 - 0.111)	0.118 (0.090 - 0.153)	0.118 (0.090 - 0.153)	0.118 (0.090 - 0.153)	0.036 (0.015 - 0.073)	0.036 (0.015 - 0.074)	0.036 (0.016 - 0.073)
Total number of cases (thousands)	19 (16 - 23)	17 (14 - 19)	15 (13 - 18)	24 (18 - 31)	20 (15 - 27)	19 (14 - 24)	0 (0 - 0)	0 (0 - 0)	0 (0 - 0)
**Saint Vincent and the Grenadines**									
Under-5 years (rate per capita)	0.405 (0.316 - 0.513)	0.400 (0.314 - 0.512)	0.400 (0.314 - 0.512)	0.590 (0.373 - 0.780)	0.587 (0.379 - 0.784)	0.586 (0.379 - 0.784)	0.154 (0.064 - 0.311)	0.152 (0.064 - 0.310)	0.152 (0.064 - 0.303)
5-14 years (rate per capita)	0.383 (0.327 - 0.434)	0.307 (0.231 - 0.378)	0.310 (0.233 - 0.381)	0.421 (0.280 - 0.613)	0.349 (0.230 - 0.535)	0.352 (0.232 - 0.540)	0.145 (0.061 - 0.285)	0.116 (0.048 - 0.237)	0.118 (0.049 - 0.242)
All ages (rate per capita)	0.148 (0.128 - 0.168)	0.097 (0.081 - 0.114)	0.084 (0.070 - 0.099)	0.180 (0.134 - 0.241)	0.122 (0.089 - 0.168)	0.106 (0.077 - 0.146)	0.056 (0.024 - 0.108)	0.037 (0.015 - 0.074)	0.032 (0.014 - 0.064)
Age-standardised (rate per capita)	0.102 (0.088 - 0.116)	0.090 (0.075 - 0.106)	0.090 (0.075 - 0.106)	0.125 (0.093 - 0.167)	0.114 (0.083 - 0.156)	0.114 (0.083 - 0.156)	0.039 (0.016 - 0.075)	0.034 (0.014 - 0.069)	0.034 (0.015 - 0.069)
Total number of cases (thousands)	16 (14 - 18)	11 (9 - 12)	9 (8 - 11)	19 (14 - 26)	13 (10 - 18)	12 (8 - 16)	0 (0 - 0)	0 (0 - 0)	0 (0 - 0)
**Suriname**									
Under-5 years (rate per capita)	0.471 (0.385 - 0.539)	0.484 (0.399 - 0.550)	0.474 (0.388 - 0.542)	0.582 (0.422 - 0.729)	0.601 (0.444 - 0.743)	0.586 (0.424 - 0.733)	0.110 (0.048 - 0.211)	0.113 (0.049 - 0.214)	0.111 (0.048 - 0.215)
5-14 years (rate per capita)	0.359 (0.286 - 0.432)	0.412 (0.355 - 0.471)	0.332 (0.262 - 0.401)	0.376 (0.262 - 0.541)	0.403 (0.264 - 0.608)	0.351 (0.242 - 0.513)	0.084 (0.037 - 0.168)	0.097 (0.041 - 0.191)	0.078 (0.032 - 0.155)
All ages (rate per capita)	0.137 (0.118 - 0.155)	0.129 (0.115 - 0.143)	0.101 (0.087 - 0.116)	0.155 (0.119 - 0.198)	0.139 (0.105 - 0.185)	0.114 (0.088 - 0.149)	0.032 (0.014 - 0.063)	0.030 (0.013 - 0.058)	0.024 (0.010 - 0.047)
Age-standardised (rate per capita)	0.100 (0.086 - 0.114)	0.112 (0.099 - 0.124)	0.100 (0.086 - 0.114)	0.113 (0.087 - 0.147)	0.121 (0.092 - 0.161)	0.113 (0.087 - 0.147)	0.023 (0.010 - 0.046)	0.026 (0.011 - 0.050)	0.023 (0.010 - 0.046)
Total number of cases (thousands)	56 (48 - 63)	63 (56 - 70)	55 (47 - 63)	63 (49 - 81)	68 (52 - 91)	62 (48 - 81)	0 (0 - 0)	0 (0 - 0)	0 (0 - 0)
**Trinidad and Tobago**									
Under-5 years (rate per capita)	0.453 (0.354 - 0.547)	0.441 (0.344 - 0.532)	0.447 (0.349 - 0.539)	0.614 (0.420 - 0.791)	0.598 (0.409 - 0.770)	0.606 (0.414 - 0.780)	0.172 (0.071 - 0.352)	0.167 (0.070 - 0.339)	0.170 (0.072 - 0.342)
5-14 years (rate per capita)	0.343 (0.264 - 0.419)	0.298 (0.227 - 0.368)	0.330 (0.253 - 0.404)	0.396 (0.260 - 0.566)	0.348 (0.226 - 0.515)	0.382 (0.250 - 0.553)	0.130 (0.055 - 0.266)	0.113 (0.048 - 0.231)	0.125 (0.053 - 0.253)
All ages (rate per capita)	0.129 (0.108 - 0.150)	0.076 (0.063 - 0.089)	0.077 (0.064 - 0.090)	0.159 (0.120 - 0.205)	0.094 (0.071 - 0.123)	0.095 (0.072 - 0.123)	0.049 (0.021 - 0.098)	0.029 (0.012 - 0.057)	0.029 (0.013 - 0.058)
Age-standardised (rate per capita)	0.095 (0.080 - 0.111)	0.095 (0.080 - 0.111)	0.095 (0.080 - 0.111)	0.118 (0.090 - 0.153)	0.118 (0.090 - 0.153)	0.118 (0.090 - 0.153)	0.036 (0.015 - 0.073)	0.036 (0.016 - 0.072)	0.036 (0.016 - 0.072)
Total number of cases (thousands)	158 (132 - 184)	98 (82 - 115)	105 (88 - 122)	195 (147 - 252)	123 (92 - 159)	130 (98 - 167)	0 (0 - 0)	0 (0 - 0)	0 (0 - 0)
**Virgin Islands, U.S.**									
Under-5 years (rate per capita)	0.446 (0.348 - 0.538)	0.441 (0.344 - 0.532)	0.450 (0.351 - 0.542)	0.585 (0.404 - 0.752)	0.578 (0.399 - 0.743)	0.590 (0.407 - 0.758)	0.105 (0.045 - 0.209)	0.103 (0.043 - 0.204)	0.105 (0.045 - 0.207)
5-14 years (rate per capita)	0.320 (0.245 - 0.393)	0.305 (0.233 - 0.376)	0.325 (0.249 - 0.399)	0.358 (0.234 - 0.524)	0.344 (0.223 - 0.506)	0.363 (0.238 - 0.529)	0.075 (0.032 - 0.153)	0.071 (0.030 - 0.144)	0.076 (0.033 - 0.155)
All ages (rate per capita)	0.113 (0.094 - 0.132)	0.079 (0.066 - 0.092)	0.076 (0.063 - 0.088)	0.136 (0.104 - 0.175)	0.095 (0.072 - 0.124)	0.091 (0.069 - 0.117)	0.026 (0.011 - 0.053)	0.019 (0.008 - 0.037)	0.018 (0.008 - 0.035)
Age-standardised (rate per capita)	0.095 (0.080 - 0.111)	0.095 (0.080 - 0.111)	0.095 (0.080 - 0.111)	0.114 (0.087 - 0.147)	0.114 (0.087 - 0.147)	0.114 (0.087 - 0.147)	0.022 (0.010 - 0.045)	0.022 (0.010 - 0.045)	0.022 (0.010 - 0.044)
Total number of cases (thousands)	12 (10 - 14)	9 (7 - 10)	8 (7 - 9)	14 (11 - 19)	10 (8 - 13)	10 (7 - 12)	0 (0 - 0)	0 (0 - 0)	0 (0 - 0)
**Tropical Latin America**									
Under-5 years (rate per capita)	0.317 (0.252 - 0.389)	0.257 (0.207 - 0.312)	0.323 (0.259 - 0.407)	0.539 (0.302 - 0.757)	0.409 (0.243 - 0.595)	0.464 (0.294 - 0.636)	0.074 (0.032 - 0.148)	0.060 (0.026 - 0.121)	0.076 (0.032 - 0.152)
5-14 years (rate per capita)	0.255 (0.198 - 0.323)	0.223 (0.172 - 0.290)	0.234 (0.180 - 0.295)	0.338 (0.217 - 0.510)	0.266 (0.161 - 0.413)	0.307 (0.195 - 0.472)	0.060 (0.025 - 0.124)	0.052 (0.022 - 0.109)	0.055 (0.023 - 0.111)
All ages (rate per capita)	0.098 (0.082 - 0.118)	0.065 (0.054 - 0.078)	0.061 (0.051 - 0.073)	0.145 (0.103 - 0.198)	0.087 (0.061 - 0.125)	0.083 (0.060 - 0.117)	0.023 (0.010 - 0.046)	0.015 (0.006 - 0.031)	0.014 (0.006 - 0.029)
Age-standardised (rate per capita)	0.071 (0.059 - 0.085)	0.061 (0.051 - 0.073)	0.072 (0.060 - 0.086)	0.105 (0.074 - 0.144)	0.082 (0.057 - 0.117)	0.097 (0.070 - 0.137)	0.017 (0.007 - 0.033)	0.014 (0.006 - 0.029)	0.017 (0.007 - 0.033)
Total number of cases (thousands)	15,219 (12,617 - 18,236)	12,630 (10,516 - 15,161)	13,076 (10,951 - 15,563)	22,391 (15,877 - 30,647)	16,888 (11,750 - 24,323)	17,776 (12,852 - 25,185)	4 (2 - 7)	3 (1 - 6)	3 (1 - 6)
**Brazil**									
Under-5 years (rate per capita)	0.318 (0.253 - 0.390)	0.255 (0.206 - 0.310)	0.325 (0.260 - 0.409)	0.540 (0.302 - 0.757)	0.405 (0.241 - 0.589)	0.462 (0.293 - 0.631)	0.075 (0.032 - 0.148)	0.060 (0.026 - 0.120)	0.076 (0.033 - 0.153)
5-14 years (rate per capita)	0.255 (0.197 - 0.323)	0.222 (0.171 - 0.288)	0.233 (0.179 - 0.294)	0.339 (0.218 - 0.510)	0.264 (0.159 - 0.410)	0.306 (0.194 - 0.472)	0.060 (0.025 - 0.124)	0.052 (0.022 - 0.109)	0.055 (0.023 - 0.110)
All ages (rate per capita)	0.098 (0.081 - 0.117)	0.064 (0.053 - 0.077)	0.060 (0.051 - 0.072)	0.144 (0.102 - 0.197)	0.085 (0.059 - 0.123)	0.082 (0.059 - 0.116)	0.023 (0.010 - 0.046)	0.015 (0.006 - 0.030)	0.014 (0.006 - 0.028)
Age-standardised (rate per capita)	0.071 (0.059 - 0.085)	0.061 (0.050 - 0.073)	0.072 (0.060 - 0.085)	0.105 (0.074 - 0.144)	0.081 (0.056 - 0.117)	0.097 (0.070 - 0.137)	0.017 (0.007 - 0.033)	0.014 (0.006 - 0.029)	0.017 (0.007 - 0.033)
Total number of cases (thousands)	14,732 (12,229 - 17,648)	12,078 (10,051 - 14,493)	12,539 (10,512 - 14,898)	21,680 (15,382 - 29,650)	16,090 (11,178 - 23,228)	16,997 (12,301 - 24,054)	3 (1 - 7)	3 (1 - 6)	3 (1 - 6)
**Paraguay**									
Under-5 years (rate per capita)	0.294 (0.225 - 0.363)	0.297 (0.228 - 0.367)	0.296 (0.227 - 0.366)	0.524 (0.287 - 0.759)	0.529 (0.290 - 0.766)	0.527 (0.289 - 0.764)	0.069 (0.029 - 0.138)	0.070 (0.030 - 0.137)	0.069 (0.030 - 0.137)
5-14 years (rate per capita)	0.269 (0.198 - 0.345)	0.253 (0.187 - 0.326)	0.252 (0.185 - 0.324)	0.334 (0.207 - 0.521)	0.316 (0.196 - 0.495)	0.314 (0.194 - 0.492)	0.063 (0.026 - 0.130)	0.060 (0.024 - 0.121)	0.059 (0.024 - 0.121)
All ages (rate per capita)	0.115 (0.094 - 0.139)	0.095 (0.078 - 0.115)	0.081 (0.066 - 0.098)	0.168 (0.115 - 0.241)	0.137 (0.095 - 0.198)	0.117 (0.081 - 0.168)	0.027 (0.011 - 0.055)	0.022 (0.009 - 0.045)	0.019 (0.008 - 0.038)
Age-standardised (rate per capita)	0.070 (0.057 - 0.085)	0.070 (0.057 - 0.085)	0.070 (0.057 - 0.085)	0.101 (0.070 - 0.146)	0.101 (0.070 - 0.146)	0.101 (0.070 - 0.146)	0.016 (0.007 - 0.033)	0.016 (0.007 - 0.033)	0.016 (0.007 - 0.033)
Total number of cases (thousands)	487 (398 - 587)	553 (452 - 669)	537 (439 - 650)	711 (488 - 1,017)	798 (550 - 1,148)	779 (536 - 1,121)	0 (0 - 0)	0 (0 - 0)	0 (0 - 0)
**Southeast Asia, East Asia, and Oceania**									
Under-5 years (rate per capita)	0.444 (0.367 - 0.518)	0.437 (0.359 - 0.515)	0.440 (0.361 - 0.518)	0.596 (0.436 - 0.739)	0.575 (0.406 - 0.728)	0.594 (0.423 - 0.745)	0.108 (0.047 - 0.209)	0.109 (0.048 - 0.211)	0.109 (0.048 - 0.211)
5-14 years (rate per capita)	0.293 (0.236 - 0.364)	0.278 (0.226 - 0.341)	0.253 (0.197 - 0.324)	0.486 (0.343 - 0.679)	0.453 (0.325 - 0.636)	0.481 (0.336 - 0.683)	0.071 (0.031 - 0.145)	0.070 (0.031 - 0.142)	0.063 (0.027 - 0.131)
All ages (rate per capita)	0.107 (0.093 - 0.124)	0.076 (0.066 - 0.087)	0.064 (0.054 - 0.075)	0.162 (0.127 - 0.206)	0.113 (0.088 - 0.149)	0.105 (0.081 - 0.138)	0.026 (0.011 - 0.051)	0.019 (0.008 - 0.038)	0.016 (0.007 - 0.032)
Age-standardised (rate per capita)	0.090 (0.078 - 0.104)	0.091 (0.079 - 0.104)	0.083 (0.070 - 0.098)	0.136 (0.107 - 0.175)	0.136 (0.105 - 0.177)	0.136 (0.105 - 0.179)	0.022 (0.009 - 0.043)	0.023 (0.010 - 0.045)	0.021 (0.009 - 0.041)
Total number of cases (thousands)	178,304 (155,082 - 206,752)	147,263 (128,945 - 170,504)	134,638 (113,783 - 158,060)	269,000 (210,855 - 343,032)	221,118 (172,009 - 290,412)	220,342 (170,181 - 290,116)	43 (19 - 85)	37 (16 - 74)	33 (15 - 67)
**East Asia**									
Under-5 years (rate per capita)	0.458 (0.380 - 0.529)	0.458 (0.386 - 0.529)	0.461 (0.385 - 0.531)	0.581 (0.432 - 0.724)	0.564 (0.407 - 0.711)	0.587 (0.432 - 0.732)	0.108 (0.046 - 0.209)	0.108 (0.047 - 0.210)	0.109 (0.048 - 0.211)
5-14 years (rate per capita)	0.306 (0.249 - 0.380)	0.271 (0.221 - 0.333)	0.250 (0.197 - 0.317)	0.480 (0.340 - 0.669)	0.438 (0.309 - 0.623)	0.479 (0.331 - 0.689)	0.072 (0.032 - 0.148)	0.064 (0.028 - 0.129)	0.059 (0.025 - 0.123)
All ages (rate per capita)	0.103 (0.091 - 0.119)	0.065 (0.058 - 0.076)	0.056 (0.048 - 0.065)	0.147 (0.115 - 0.188)	0.095 (0.073 - 0.127)	0.089 (0.068 - 0.117)	0.024 (0.010 - 0.048)	0.015 (0.007 - 0.030)	0.013 (0.006 - 0.026)
Age-standardised (rate per capita)	0.094 (0.082 - 0.108)	0.092 (0.081 - 0.106)	0.084 (0.072 - 0.098)	0.134 (0.104 - 0.173)	0.135 (0.103 - 0.177)	0.135 (0.104 - 0.177)	0.022 (0.009 - 0.043)	0.022 (0.009 - 0.043)	0.020 (0.009 - 0.040)
Total number of cases (thousands)	123,568 (108,501 - 142,051)	88,880 (78,219 - 103,355)	79,752 (68,379 - 92,947)	176,015 (137,570 - 225,032)	129,777 (99,681 - 172,238)	127,332 (97,965 - 167,422)	29 (13 - 57)	21 (9 - 41)	19 (8 - 38)
**China**									
Under-5 years (rate per capita)	0.459 (0.382 - 0.529)	0.460 (0.388 - 0.529)	0.463 (0.387 - 0.532)	0.582 (0.433 - 0.723)	0.563 (0.407 - 0.709)	0.588 (0.433 - 0.732)	0.108 (0.046 - 0.209)	0.108 (0.047 - 0.211)	0.109 (0.048 - 0.211)
5-14 years (rate per capita)	0.309 (0.251 - 0.382)	0.272 (0.223 - 0.333)	0.252 (0.199 - 0.318)	0.481 (0.341 - 0.667)	0.437 (0.309 - 0.619)	0.481 (0.333 - 0.691)	0.072 (0.032 - 0.149)	0.064 (0.028 - 0.130)	0.059 (0.025 - 0.124)
All ages (rate per capita)	0.104 (0.092 - 0.120)	0.065 (0.058 - 0.076)	0.056 (0.048 - 0.065)	0.148 (0.115 - 0.188)	0.095 (0.073 - 0.126)	0.089 (0.069 - 0.117)	0.024 (0.011 - 0.048)	0.015 (0.007 - 0.030)	0.013 (0.006 - 0.026)
Age-standardised (rate per capita)	0.094 (0.083 - 0.109)	0.093 (0.082 - 0.107)	0.084 (0.072 - 0.099)	0.134 (0.105 - 0.173)	0.135 (0.104 - 0.177)	0.135 (0.104 - 0.177)	0.022 (0.009 - 0.044)	0.022 (0.010 - 0.043)	0.020 (0.009 - 0.040)
Total number of cases (thousands)	120,321 (105,787 - 138,031)	85,961 (75,792 - 99,799)	77,332 (66,379 - 90,105)	170,466 (133,286 - 217,418)	124,757 (96,107 - 165,226)	123,211 (94,904 - 161,807)	28 (12 - 56)	20 (9 - 40)	18 (8 - 36)
**North Korea**									
Under-5 years (rate per capita)	0.416 (0.319 - 0.512)	0.421 (0.323 - 0.518)	0.419 (0.321 - 0.516)	0.575 (0.395 - 0.741)	0.582 (0.400 - 0.750)	0.579 (0.398 - 0.747)	0.098 (0.041 - 0.197)	0.099 (0.042 - 0.197)	0.099 (0.042 - 0.197)
5-14 years (rate per capita)	0.252 (0.188 - 0.331)	0.230 (0.171 - 0.306)	0.224 (0.166 - 0.300)	0.498 (0.328 - 0.757)	0.457 (0.299 - 0.705)	0.446 (0.290 - 0.693)	0.059 (0.024 - 0.125)	0.054 (0.022 - 0.113)	0.053 (0.022 - 0.114)
All ages (rate per capita)	0.083 (0.067 - 0.102)	0.071 (0.057 - 0.088)	0.061 (0.049 - 0.075)	0.141 (0.104 - 0.191)	0.122 (0.089 - 0.169)	0.104 (0.077 - 0.144)	0.019 (0.008 - 0.039)	0.017 (0.007 - 0.034)	0.014 (0.006 - 0.030)
Age-standardised (rate per capita)	0.078 (0.063 - 0.096)	0.078 (0.063 - 0.096)	0.078 (0.063 - 0.096)	0.132 (0.098 - 0.182)	0.132 (0.098 - 0.182)	0.132 (0.098 - 0.182)	0.018 (0.008 - 0.037)	0.018 (0.008 - 0.037)	0.018 (0.008 - 0.038)
Total number of cases (thousands)	1,677 (1,353 - 2,050)	1,691 (1,353 - 2,081)	1,536 (1,232 - 1,889)	2,836 (2,102 - 3,852)	2,893 (2,126 - 4,022)	2,610 (1,925 - 3,622)	0 (0 - 1)	0 (0 - 1)	0 (0 - 1)
**Taiwan**									
Under-5 years (rate per capita)	0.420 (0.322 - 0.517)	0.434 (0.332 - 0.533)	0.418 (0.320 - 0.513)	0.580 (0.399 - 0.748)	0.599 (0.412 - 0.773)	0.576 (0.396 - 0.744)	0.099 (0.041 - 0.197)	0.102 (0.043 - 0.203)	0.098 (0.041 - 0.192)
5-14 years (rate per capita)	0.228 (0.170 - 0.304)	0.226 (0.168 - 0.302)	0.187 (0.136 - 0.261)	0.454 (0.297 - 0.702)	0.450 (0.294 - 0.698)	0.377 (0.240 - 0.618)	0.054 (0.022 - 0.118)	0.053 (0.022 - 0.112)	0.044 (0.018 - 0.096)
All ages (rate per capita)	0.078 (0.062 - 0.096)	0.054 (0.043 - 0.067)	0.036 (0.029 - 0.045)	0.134 (0.098 - 0.188)	0.094 (0.068 - 0.132)	0.062 (0.046 - 0.089)	0.018 (0.008 - 0.037)	0.013 (0.005 - 0.026)	0.009 (0.004 - 0.018)

	Prevalence 1990	Prevalence 2005	Prevalence 2015	Incidence 1990	Incidence 2005	Incidence 2015	YLD rate 1990	YLD rate 2005	YLD rate 2015
Age-standardised (rate per capita)	0.078 (0.063 - 0.096)	0.078 (0.063 - 0.096)	0.078 (0.063 - 0.096)	0.132 (0.098 - 0.182)	0.132 (0.098 - 0.182)	0.132 (0.098 - 0.182)	0.018 (0.008 - 0.037)	0.018 (0.008 - 0.037)	0.018 (0.008 - 0.037)
Total number of cases (thousands)	1,570 (1,254 - 1,941)	1,228 (981 - 1,521)	884 (703 - 1,102)	2,713 (1,985 - 3,808)	2,128 (1,556 - 2,990)	1,511 (1,108 - 2,152)	0 (0 - 1)	0 (0 - 1)	0 (0 - 0)
**Southeast Asia**									
Under-5 years (rate per capita)	0.415 (0.334 - 0.502)	0.410 (0.323 - 0.499)	0.412 (0.323 - 0.503)	0.628 (0.448 - 0.784)	0.590 (0.404 - 0.755)	0.606 (0.413 - 0.777)	0.109 (0.048 - 0.214)	0.111 (0.048 - 0.216)	0.111 (0.048 - 0.216)
5-14 years (rate per capita)	0.269 (0.208 - 0.342)	0.291 (0.236 - 0.353)	0.258 (0.194 - 0.332)	0.497 (0.348 - 0.715)	0.478 (0.344 - 0.665)	0.485 (0.336 - 0.694)	0.070 (0.029 - 0.144)	0.082 (0.036 - 0.162)	0.069 (0.029 - 0.143)
All ages (rate per capita)	0.117 (0.099 - 0.137)	0.099 (0.085 - 0.115)	0.083 (0.068 - 0.098)	0.198 (0.155 - 0.258)	0.154 (0.120 - 0.198)	0.140 (0.107 - 0.183)	0.031 (0.013 - 0.060)	0.027 (0.012 - 0.054)	0.022 (0.010 - 0.044)
Age-standardised (rate per capita)	0.083 (0.070 - 0.097)	0.088 (0.075 - 0.102)	0.082 (0.068 - 0.097)	0.140 (0.110 - 0.182)	0.137 (0.107 - 0.176)	0.139 (0.106 - 0.181)	0.022 (0.009 - 0.043)	0.024 (0.011 - 0.048)	0.022 (0.010 - 0.043)
Total number of cases (thousands)	53,956 (45,508 - 63,401)	57,372 (49,090 - 66,657)	53,781 (44,521 - 63,928)	91,586 (71,430 - 118,874)	89,541 (69,766 - 115,179)	91,027 (69,824 - 118,911)	14 (6 - 28)	16 (7 - 31)	14 (6 - 28)
**Cambodia**									
Under-5 years (rate per capita)	0.453 (0.364 - 0.526)	0.457 (0.372 - 0.526)	0.457 (0.367 - 0.530)	0.614 (0.450 - 0.758)	0.624 (0.460 - 0.768)	0.619 (0.454 - 0.764)	0.106 (0.046 - 0.206)	0.107 (0.045 - 0.204)	0.107 (0.046 - 0.206)
5-14 years (rate per capita)	0.346 (0.272 - 0.434)	0.275 (0.221 - 0.355)	0.290 (0.226 - 0.371)	0.595 (0.432 - 0.834)	0.465 (0.333 - 0.663)	0.505 (0.363 - 0.727)	0.081 (0.034 - 0.161)	0.064 (0.027 - 0.135)	0.068 (0.029 - 0.144)
All ages (rate per capita)	0.174 (0.147 - 0.202)	0.124 (0.107 - 0.147)	0.111 (0.093 - 0.130)	0.267 (0.213 - 0.339)	0.193 (0.151 - 0.245)	0.173 (0.136 - 0.224)	0.040 (0.018 - 0.081)	0.029 (0.012 - 0.058)	0.026 (0.011 - 0.052)
Age-standardised (rate per capita)	0.089 (0.075 - 0.105)	0.092 (0.079 - 0.108)	0.089 (0.075 - 0.105)	0.140 (0.110 - 0.182)	0.141 (0.111 - 0.177)	0.140 (0.110 - 0.182)	0.021 (0.009 - 0.041)	0.021 (0.009 - 0.043)	0.021 (0.009 - 0.042)
Total number of cases (thousands)	1,565 (1,325 - 1,817)	1,662 (1,430 - 1,965)	1,730 (1,457 - 2,026)	2,407 (1,923 - 3,053)	2,578 (2,023 - 3,281)	2,696 (2,126 - 3,499)	0 (0 - 1)	0 (0 - 1)	0 (0 - 1)
**Indonesia**									
Under-5 years (rate per capita)	0.425 (0.343 - 0.511)	0.406 (0.313 - 0.508)	0.409 (0.316 - 0.513)	0.667 (0.496 - 0.796)	0.598 (0.398 - 0.770)	0.603 (0.401 - 0.777)	0.099 (0.042 - 0.196)	0.095 (0.039 - 0.190)	0.096 (0.041 - 0.190)
5-14 years (rate per capita)	0.279 (0.215 - 0.348)	0.261 (0.195 - 0.337)	0.258 (0.192 - 0.334)	0.485 (0.345 - 0.698)	0.480 (0.342 - 0.685)	0.474 (0.337 - 0.679)	0.065 (0.028 - 0.135)	0.061 (0.026 - 0.128)	0.060 (0.026 - 0.126)
All ages (rate per capita)	0.119 (0.102 - 0.140)	0.093 (0.076 - 0.112)	0.086 (0.070 - 0.103)	0.199 (0.157 - 0.253)	0.156 (0.121 - 0.202)	0.143 (0.111 - 0.186)	0.028 (0.012 - 0.055)	0.022 (0.009 - 0.044)	0.020 (0.009 - 0.040)
Age-standardised (rate per capita)	0.086 (0.073 - 0.101)	0.082 (0.067 - 0.099)	0.082 (0.067 - 0.099)	0.143 (0.113 - 0.181)	0.137 (0.106 - 0.178)	0.137 (0.106 - 0.178)	0.020 (0.009 - 0.040)	0.019 (0.008 - 0.039)	0.019 (0.008 - 0.038)
Total number of cases (thousands)	21,608 (18,527 - 25,396)	21,066 (17,277 - 25,336)	22,111 (18,134 - 26,596)	36,004 (28,386 - 45,934)	35,359 (27,409 - 45,810)	36,944 (28,579 - 47,878)	5 (2 - 10)	5 (2 - 10)	5 (2 - 10)
**Laos**									
Under-5 years (rate per capita)	0.415 (0.327 - 0.505)	0.449 (0.363 - 0.520)	0.426 (0.336 - 0.518)	0.592 (0.410 - 0.746)	0.647 (0.483 - 0.774)	0.608 (0.421 - 0.766)	0.097 (0.041 - 0.191)	0.105 (0.044 - 0.202)	0.100 (0.042 - 0.194)
5-14 years (rate per capita)	0.282 (0.216 - 0.362)	0.266 (0.201 - 0.345)	0.275 (0.210 - 0.355)	0.511 (0.362 - 0.720)	0.479 (0.331 - 0.694)	0.500 (0.353 - 0.710)	0.066 (0.027 - 0.140)	0.062 (0.026 - 0.133)	0.065 (0.027 - 0.139)
All ages (rate per capita)	0.146 (0.121 - 0.174)	0.131 (0.109 - 0.156)	0.114 (0.094 - 0.136)	0.238 (0.184 - 0.303)	0.215 (0.168 - 0.280)	0.187 (0.144 - 0.239)	0.034 (0.015 - 0.070)	0.031 (0.013 - 0.061)	0.027 (0.012 - 0.055)
Age-standardised (rate per capita)	0.085 (0.070 - 0.101)	0.087 (0.073 - 0.104)	0.085 (0.070 - 0.101)	0.139 (0.106 - 0.178)	0.143 (0.112 - 0.184)	0.139 (0.106 - 0.178)	0.020 (0.009 - 0.040)	0.020 (0.009 - 0.041)	0.020 (0.009 - 0.041)
Total number of cases (thousands)	618 (511 - 734)	753 (628 - 897)	776 (639 - 926)	1,006 (779 - 1,278)	1,235 (965 - 1,603)	1,271 (979 - 1,626)	0 (0 - 0)	0 (0 - 0)	0 (0 - 0)
**Malaysia**									
Under-5 years (rate per capita)	0.385 (0.300 - 0.491)	0.394 (0.307 - 0.503)	0.386 (0.301 - 0.493)	0.587 (0.369 - 0.769)	0.601 (0.378 - 0.787)	0.590 (0.371 - 0.772)	0.090 (0.038 - 0.180)	0.092 (0.040 - 0.187)	0.091 (0.039 - 0.182)
5-14 years (rate per capita)	0.268 (0.202 - 0.341)	0.256 (0.192 - 0.327)	0.241 (0.180 - 0.311)	0.490 (0.338 - 0.707)	0.468 (0.322 - 0.684)	0.441 (0.301 - 0.647)	0.063 (0.026 - 0.132)	0.060 (0.025 - 0.127)	0.057 (0.024 - 0.120)
All ages (rate per capita)	0.114 (0.094 - 0.137)	0.090 (0.074 - 0.108)	0.071 (0.058 - 0.085)	0.194 (0.146 - 0.256)	0.153 (0.115 - 0.203)	0.120 (0.090 - 0.159)	0.027 (0.011 - 0.055)	0.021 (0.009 - 0.043)	0.017 (0.007 - 0.034)
Age-standardised (rate per capita)	0.079 (0.065 - 0.095)	0.079 (0.065 - 0.095)	0.079 (0.065 - 0.095)	0.134 (0.101 - 0.178)	0.134 (0.101 - 0.178)	0.134 (0.101 - 0.178)	0.019 (0.008 - 0.038)	0.019 (0.008 - 0.038)	0.019 (0.008 - 0.038)
Total number of cases (thousands)	2,079 (1,714 - 2,482)	2,303 (1,894 - 2,765)	2,138 (1,760 - 2,575)	3,520 (2,655 - 4,648)	3,921 (2,954 - 5,211)	3,627 (2,728 - 4,830)	0 (0 - 1)	1 (0 - 1)	1 (0 - 1)
**Maldives**									
Under-5 years (rate per capita)	0.420 (0.315 - 0.520)	0.421 (0.316 - 0.522)	0.421 (0.315 - 0.521)	0.602 (0.391 - 0.777)	0.604 (0.392 - 0.780)	0.603 (0.392 - 0.778)	0.098 (0.041 - 0.195)	0.099 (0.043 - 0.195)	0.099 (0.041 - 0.197)
5-14 years (rate per capita)	0.280 (0.204 - 0.364)	0.227 (0.164 - 0.300)	0.257 (0.187 - 0.336)	0.554 (0.371 - 0.801)	0.453 (0.298 - 0.676)	0.511 (0.340 - 0.745)	0.065 (0.027 - 0.137)	0.053 (0.021 - 0.114)	0.060 (0.024 - 0.128)
All ages (rate per capita)	0.160 (0.128 - 0.195)	0.096 (0.077 - 0.117)	0.087 (0.069 - 0.106)	0.273 (0.204 - 0.359)	0.167 (0.123 - 0.226)	0.149 (0.111 - 0.198)	0.037 (0.016 - 0.075)	0.022 (0.010 - 0.046)	0.020 (0.009 - 0.041)
Age-standardised (rate per capita)	0.081 (0.065 - 0.098)	0.081 (0.065 - 0.098)	0.081 (0.065 - 0.099)	0.140 (0.104 - 0.186)	0.140 (0.104 - 0.186)	0.140 (0.104 - 0.186)	0.019 (0.008 - 0.038)	0.019 (0.008 - 0.039)	0.019 (0.008 - 0.038)
Total number of cases (thousands)	36 (29 - 44)	29 (23 - 36)	32 (25 - 38)	61 (46 - 80)	51 (38 - 69)	54 (40 - 72)	0 (0 - 0)	0 (0 - 0)	0 (0 - 0)
**Mauritius**									
Under-5 years (rate per capita)	0.419 (0.314 - 0.519)	0.427 (0.320 - 0.530)	0.425 (0.319 - 0.527)	0.619 (0.396 - 0.800)	0.632 (0.404 - 0.817)	0.629 (0.402 - 0.813)	0.159 (0.067 - 0.323)	0.162 (0.069 - 0.329)	0.162 (0.067 - 0.334)
5-14 years (rate per capita)	0.243 (0.176 - 0.319)	0.236 (0.170 - 0.311)	0.230 (0.166 - 0.303)	0.495 (0.325 - 0.728)	0.480 (0.315 - 0.712)	0.467 (0.305 - 0.696)	0.093 (0.038 - 0.187)	0.090 (0.037 - 0.181)	0.087 (0.037 - 0.179)
All ages (rate per capita)	0.089 (0.071 - 0.109)	0.073 (0.058 - 0.089)	0.055 (0.044 - 0.068)	0.158 (0.117 - 0.211)	0.130 (0.096 - 0.175)	0.099 (0.073 - 0.135)	0.034 (0.015 - 0.068)	0.028 (0.012 - 0.055)	0.021 (0.009 - 0.042)
Age-standardised (rate per capita)	0.081 (0.065 - 0.098)	0.081 (0.065 - 0.098)	0.081 (0.065 - 0.098)	0.143 (0.106 - 0.192)	0.143 (0.106 - 0.192)	0.143 (0.106 - 0.192)	0.031 (0.013 - 0.061)	0.031 (0.013 - 0.061)	0.031 (0.013 - 0.062)
Total number of cases (thousands)	95 (76 - 116)	89 (71 - 109)	71 (57 - 87)	168 (124 - 224)	159 (117 - 214)	127 (93 - 172)	0 (0 - 0)	0 (0 - 0)	0 (0 - 0)
**Myanmar**									
Under-5 years (rate per capita)	0.308 (0.227 - 0.419)	0.282 (0.199 - 0.412)	0.310 (0.228 - 0.421)	0.558 (0.308 - 0.789)	0.439 (0.242 - 0.715)	0.562 (0.310 - 0.794)	0.072 (0.030 - 0.149)	0.066 (0.027 - 0.138)	0.073 (0.031 - 0.149)
5-14 years (rate per capita)	0.230 (0.174 - 0.298)	0.200 (0.149 - 0.263)	0.217 (0.164 - 0.282)	0.480 (0.337 - 0.690)	0.457 (0.299 - 0.670)	0.453 (0.316 - 0.656)	0.054 (0.022 - 0.111)	0.047 (0.019 - 0.098)	0.051 (0.021 - 0.106)
All ages (rate per capita)	0.097 (0.076 - 0.118)	0.070 (0.055 - 0.089)	0.068 (0.054 - 0.084)	0.191 (0.138 - 0.257)	0.138 (0.099 - 0.196)	0.135 (0.098 - 0.182)	0.023 (0.010 - 0.047)	0.016 (0.007 - 0.034)	0.016 (0.007 - 0.033)
Age-standardised (rate per capita)	0.066 (0.052 - 0.081)	0.059 (0.046 - 0.075)	0.066 (0.052 - 0.081)	0.131 (0.095 - 0.176)	0.117 (0.084 - 0.166)	0.131 (0.095 - 0.176)	0.016 (0.007 - 0.032)	0.014 (0.006 - 0.029)	0.016 (0.007 - 0.032)
Total number of cases (thousands)	4,059 (3,212 - 4,962)	3,503 (2,725 - 4,458)	3,687 (2,915 - 4,531)	8,015 (5,808 - 10,794)	6,910 (4,958 - 9,804)	7,297 (5,316 - 9,851)	1 (0 - 2)	1 (0 - 2)	1 (0 - 2)
**Philippines**									
Under-5 years (rate per capita)	0.465 (0.384 - 0.530)	0.483 (0.399 - 0.549)	0.469 (0.387 - 0.535)	0.643 (0.472 - 0.798)	0.638 (0.470 - 0.791)	0.648 (0.476 - 0.805)	0.176 (0.077 - 0.348)	0.183 (0.078 - 0.359)	0.178 (0.077 - 0.348)
5-14 years (rate per capita)	0.296 (0.231 - 0.377)	0.501 (0.462 - 0.539)	0.287 (0.223 - 0.367)	0.531 (0.372 - 0.749)	0.514 (0.369 - 0.678)	0.515 (0.361 - 0.734)	0.112 (0.047 - 0.227)	0.189 (0.083 - 0.369)	0.108 (0.045 - 0.218)
All ages (rate per capita)	0.147 (0.125 - 0.170)	0.183 (0.167 - 0.197)	0.112 (0.095 - 0.131)	0.234 (0.185 - 0.298)	0.207 (0.165 - 0.256)	0.180 (0.142 - 0.230)	0.056 (0.024 - 0.109)	0.069 (0.031 - 0.135)	0.043 (0.018 - 0.084)
Age-standardised (rate per capita)	0.091 (0.077 - 0.106)	0.128 (0.117 - 0.138)	0.091 (0.077 - 0.106)	0.146 (0.115 - 0.187)	0.144 (0.115 - 0.178)	0.146 (0.115 - 0.187)	0.035 (0.015 - 0.068)	0.049 (0.021 - 0.095)	0.035 (0.015 - 0.068)
Total number of cases (thousands)	9,077 (7,734 - 10,504)	15,786 (14,345 - 16,933)	11,326 (9,604 - 13,164)	14,483 (11,467 - 18,429)	17,814 (14,188 - 22,006)	18,142 (14,317 - 23,205)	3 (1 - 7)	6 (3 - 12)	4 (2 - 8)
**Sri Lanka**									
Under-5 years (rate per capita)	0.314 (0.243 - 0.391)	0.259 (0.219 - 0.312)	0.263 (0.222 - 0.317)	0.583 (0.327 - 0.814)	0.472 (0.310 - 0.663)	0.478 (0.314 - 0.672)	0.119 (0.050 - 0.244)	0.099 (0.042 - 0.197)	0.100 (0.042 - 0.197)
5-14 years (rate per capita)	0.241 (0.183 - 0.310)	0.234 (0.175 - 0.299)	0.239 (0.178 - 0.304)	0.483 (0.340 - 0.703)	0.476 (0.332 - 0.662)	0.485 (0.337 - 0.674)	0.091 (0.037 - 0.191)	0.089 (0.037 - 0.183)	0.091 (0.038 - 0.186)
All ages (rate per capita)	0.085 (0.069 - 0.102)	0.062 (0.052 - 0.073)	0.061 (0.050 - 0.072)	0.166 (0.122 - 0.225)	0.122 (0.093 - 0.161)	0.119 (0.091 - 0.159)	0.032 (0.014 - 0.066)	0.024 (0.010 - 0.048)	0.023 (0.010 - 0.046)
Age-standardised (rate per capita)	0.069 (0.057 - 0.083)	0.064 (0.053 - 0.076)	0.064 (0.053 - 0.076)	0.135 (0.099 - 0.183)	0.126 (0.096 - 0.166)	0.126 (0.095 - 0.166)	0.026 (0.011 - 0.054)	0.024 (0.010 - 0.049)	0.024 (0.010 - 0.049)
Total number of cases (thousands)	1,471 (1,201 - 1,766)	1,219 (1,010 - 1,429)	1,264 (1,039 - 1,484)	2,870 (2,120 - 3,901)	2,380 (1,808 - 3,155)	2,474 (1,878 - 3,297)	1 (0 - 1)	0 (0 - 1)	0 (0 - 1)
**Seychelles**									
Under-5 years (rate per capita)	0.424 (0.318 - 0.526)	0.418 (0.313 - 0.518)	0.426 (0.319 - 0.527)	0.608 (0.395 - 0.785)	0.599 (0.389 - 0.773)	0.611 (0.396 - 0.788)	0.100 (0.042 - 0.198)	0.098 (0.042 - 0.195)	0.100 (0.043 - 0.206)
5-14 years (rate per capita)	0.250 (0.181 - 0.327)	0.241 (0.174 - 0.316)	0.265 (0.193 - 0.345)	0.496 (0.330 - 0.729)	0.479 (0.318 - 0.710)	0.525 (0.350 - 0.765)	0.059 (0.024 - 0.125)	0.056 (0.023 - 0.121)	0.062 (0.026 - 0.132)
All ages (rate per capita)	0.111 (0.089 - 0.135)	0.077 (0.061 - 0.093)	0.076 (0.061 - 0.093)	0.192 (0.143 - 0.257)	0.131 (0.097 - 0.174)	0.131 (0.098 - 0.174)	0.026 (0.011 - 0.053)	0.018 (0.008 - 0.036)	0.018 (0.008 - 0.037)
Age-standardised (rate per capita)	0.081 (0.065 - 0.098)	0.081 (0.065 - 0.099)	0.081 (0.065 - 0.098)	0.140 (0.104 - 0.186)	0.140 (0.104 - 0.186)	0.140 (0.104 - 0.186)	0.019 (0.008 - 0.039)	0.019 (0.008 - 0.039)	0.019 (0.008 - 0.039)
Total number of cases (thousands)	8 (6 - 10)	7 (5 - 8)	7 (6 - 9)	14 (10 - 18)	12 (9 - 15)	13 (9 - 17)	0 (0 - 0)	0 (0 - 0)	0 (0 - 0)
**Thailand**									
Under-5 years (rate per capita)	0.424 (0.318 - 0.526)	0.425 (0.319 - 0.527)	0.426 (0.320 - 0.528)	0.609 (0.395 - 0.785)	0.610 (0.396 - 0.787)	0.612 (0.397 - 0.789)	0.100 (0.043 - 0.200)	0.100 (0.043 - 0.198)	0.100 (0.042 - 0.196)
5-14 years (rate per capita)	0.240 (0.174 - 0.315)	0.241 (0.174 - 0.316)	0.243 (0.175 - 0.318)	0.478 (0.317 - 0.709)	0.479 (0.318 - 0.710)	0.483 (0.320 - 0.714)	0.056 (0.023 - 0.120)	0.056 (0.023 - 0.121)	0.057 (0.024 - 0.120)
All ages (rate per capita)	0.090 (0.072 - 0.111)	0.065 (0.052 - 0.079)	0.053 (0.042 - 0.065)	0.157 (0.117 - 0.211)	0.114 (0.084 - 0.154)	0.092 (0.068 - 0.124)	0.021 (0.009 - 0.043)	0.015 (0.007 - 0.031)	0.012 (0.005 - 0.025)

	Prevalence 1990	Prevalence 2005	Prevalence 2015	Incidence 1990	Incidence 2005	Incidence 2015	YLD rate 1990	YLD rate 2005	YLD rate 2015
**Age-standardised (rate per capita)**	0.081 (0.065 - 0.098)	0.081 (0.065 - 0.099)	0.081 (0.065 - 0.099)	0.140 (0.104 - 0.186)	0.140 (0.104 - 0.186)	0.140 (0.104 - 0.186)	0.019 (0.008 - 0.039)	0.019 (0.008 - 0.039)	0.019 (0.008 - 0.038)
**Total number of cases (thousands)**	5,121 (4,103 - 6,262)	4,267 (3,422 - 5,222)	3,591 (2,878 - 4,388)	8,910 (6,602 - 11,958)	7,473 (5,518 - 10,086)	6,259 (4,641 - 8,392)	1 (1 - 2)	1 (0 - 2)	1 (0 - 2)
**Timor-Leste**									
**Under-5 years (rate per capita)**	0.408 (0.306 - 0.506)	0.417 (0.313 - 0.517)	0.420 (0.314 - 0.520)	0.586 (0.380 - 0.756)	0.599 (0.388 - 0.773)	0.602 (0.390 - 0.777)	0.095 (0.040 - 0.194)	0.098 (0.041 - 0.194)	0.098 (0.042 - 0.194)
**5-14 years (rate per capita)**	0.285 (0.208 - 0.370)	0.269 (0.196 - 0.350)	0.247 (0.179 - 0.323)	0.563 (0.378 - 0.813)	0.532 (0.356 - 0.774)	0.490 (0.326 - 0.722)	0.066 (0.028 - 0.141)	0.063 (0.026 - 0.136)	0.058 (0.024 - 0.124)
**All ages (rate per capita)**	0.136 (0.109 - 0.165)	0.156 (0.125 - 0.191)	0.136 (0.108 - 0.164)	0.230 (0.172 - 0.300)	0.270 (0.202 - 0.360)	0.229 (0.170 - 0.301)	0.032 (0.014 - 0.065)	0.037 (0.016 - 0.074)	0.032 (0.014 - 0.064)
**Age-standardised (rate per capita)**	0.081 (0.065 - 0.098)	0.081 (0.065 - 0.098)	0.081 (0.065 - 0.098)	0.140 (0.104 - 0.186)	0.140 (0.104 - 0.186)	0.140 (0.104 - 0.186)	0.019 (0.008 - 0.039)	0.019 (0.008 - 0.038)	0.019 (0.008 - 0.038)
**Total number of cases (thousands)**	102 (82 - 123)	156 (125 - 190)	161 (129 - 196)	172 (129 - 224)	269 (201 - 359)	272 (202 - 358)	0 (0 - 0)	0 (0 - 0)	0 (0 - 0)
**Vietnam**									
**Under-5 years (rate per capita)**	0.419 (0.314 - 0.519)	0.421 (0.315 - 0.521)	0.422 (0.316 - 0.522)	0.601 (0.390 - 0.776)	0.603 (0.391 - 0.778)	0.605 (0.393 - 0.780)	0.098 (0.042 - 0.194)	0.099 (0.042 - 0.198)	0.099 (0.042 - 0.197)
**5-14 years (rate per capita)**	0.257 (0.187 - 0.336)	0.222 (0.160 - 0.294)	0.257 (0.186 - 0.335)	0.510 (0.339 - 0.744)	0.443 (0.291 - 0.663)	0.510 (0.339 - 0.744)	0.060 (0.025 - 0.128)	0.052 (0.021 - 0.110)	0.060 (0.025 - 0.127)
**All ages (rate per capita)**	0.118 (0.094 - 0.144)	0.077 (0.061 - 0.094)	0.073 (0.058 - 0.089)	0.203 (0.151 - 0.270)	0.134 (0.099 - 0.180)	0.126 (0.094 - 0.167)	0.028 (0.012 - 0.056)	0.018 (0.008 - 0.037)	0.017 (0.007 - 0.035)
**Age-standardised (rate per capita)**	0.081 (0.065 - 0.098)	0.081 (0.065 - 0.099)	0.081 (0.065 - 0.099)	0.140 (0.104 - 0.186)	0.140 (0.104 - 0.186)	0.140 (0.104 - 0.186)	0.019 (0.008 - 0.038)	0.019 (0.008 - 0.039)	0.019 (0.008 - 0.039)
**Total number of cases (thousands)**	8,056 (6,434 - 9,802)	6,461 (5,182 - 7,935)	6,824 (5,453 - 8,301)	13,856 (10,316 - 18,444)	11,271 (8,318 - 15,204)	11,745 (8,750 - 15,630)	2 (1 - 4)	2 (1 - 3)	2 (1 - 3)
**Oceania**									
**Under-5 years (rate per capita)**	0.331 (0.248 - 0.442)	0.334 (0.249 - 0.447)	0.334 (0.250 - 0.448)	0.548 (0.318 - 0.754)	0.552 (0.320 - 0.757)	0.552 (0.320 - 0.759)	0.077 (0.033 - 0.155)	0.078 (0.034 - 0.154)	0.078 (0.033 - 0.157)
**5-14 years (rate per capita)**	0.260 (0.197 - 0.333)	0.268 (0.207 - 0.341)	0.257 (0.195 - 0.330)	0.493 (0.348 - 0.690)	0.501 (0.355 - 0.694)	0.487 (0.344 - 0.684)	0.061 (0.026 - 0.128)	0.063 (0.026 - 0.132)	0.060 (0.025 - 0.126)
**All ages (rate per capita)**	0.118 (0.096 - 0.144)	0.111 (0.091 - 0.135)	0.101 (0.082 - 0.123)	0.212 (0.160 - 0.276)	0.198 (0.149 - 0.256)	0.181 (0.136 - 0.235)	0.028 (0.012 - 0.056)	0.026 (0.011 - 0.053)	0.024 (0.010 - 0.048)
**Age-standardised (rate per capita)**	0.073 (0.060 - 0.090)	0.074 (0.061 - 0.090)	0.074 (0.060 - 0.090)	0.132 (0.100 - 0.173)	0.133 (0.100 - 0.173)	0.132 (0.100 - 0.173)	0.017 (0.007 - 0.035)	0.017 (0.007 - 0.036)	0.017 (0.007 - 0.035)
**Total number of cases (thousands)**	781 (636 - 949)	1,011 (828 - 1,226)	1,105 (901 - 1,347)	1,399 (1,054 - 1,824)	1,799 (1,355 - 2,330)	1,983 (1,497 - 2,584)	0 (0 - 0)	0 (0 - 0)	0 (0 - 1)
**American Samoa**									
**Under-5 years (rate per capita)**	0.329 (0.246 - 0.443)	0.332 (0.248 - 0.447)	0.333 (0.249 - 0.449)	0.543 (0.315 - 0.745)	0.548 (0.318 - 0.752)	0.549 (0.319 - 0.754)	0.077 (0.033 - 0.155)	0.078 (0.033 - 0.155)	0.078 (0.033 - 0.156)
**5-14 years (rate per capita)**	0.258 (0.196 - 0.333)	0.273 (0.208 - 0.350)	0.282 (0.215 - 0.361)	0.490 (0.345 - 0.687)	0.517 (0.365 - 0.715)	0.534 (0.378 - 0.732)	0.061 (0.025 - 0.129)	0.064 (0.027 - 0.136)	0.066 (0.027 - 0.142)
**All ages (rate per capita)**	0.114 (0.092 - 0.140)	0.116 (0.095 - 0.142)	0.122 (0.099 - 0.148)	0.203 (0.151 - 0.263)	0.209 (0.158 - 0.271)	0.219 (0.166 - 0.283)	0.027 (0.012 - 0.053)	0.027 (0.012 - 0.055)	0.029 (0.012 - 0.058)
**Age-standardised (rate per capita)**	0.074 (0.060 - 0.090)	0.074 (0.060 - 0.090)	0.074 (0.060 - 0.090)	0.132 (0.100 - 0.172)	0.132 (0.100 - 0.172)	0.132 (0.100 - 0.172)	0.017 (0.007 - 0.035)	0.017 (0.007 - 0.035)	0.017 (0.007 - 0.035)
**Total number of cases (thousands)**	5 (4 - 7)	8 (6 - 9)	10 (8 - 12)	10 (7 - 13)	13 (10 - 17)	18 (14 - 24)	0 (0 - 0)	0 (0 - 0)	0 (0 - 0)
**Federated States of Micronesia**									
**Under-5 years (rate per capita)**	0.335 (0.250 - 0.451)	0.342 (0.255 - 0.461)	0.337 (0.251 - 0.454)	0.553 (0.321 - 0.759)	0.564 (0.327 - 0.775)	0.556 (0.322 - 0.763)	0.079 (0.033 - 0.162)	0.080 (0.034 - 0.165)	0.079 (0.033 - 0.161)
**5-14 years (rate per capita)**	0.268 (0.204 - 0.344)	0.249 (0.189 - 0.322)	0.242 (0.183 - 0.313)	0.507 (0.359 - 0.705)	0.473 (0.332 - 0.666)	0.459 (0.321 - 0.649)	0.063 (0.026 - 0.131)	0.058 (0.024 - 0.122)	0.057 (0.023 - 0.119)
**All ages (rate per capita)**	0.130 (0.105 - 0.158)	0.111 (0.091 - 0.136)	0.094 (0.077 - 0.115)	0.232 (0.175 - 0.301)	0.200 (0.150 - 0.260)	0.169 (0.128 - 0.222)	0.030 (0.013 - 0.062)	0.026 (0.011 - 0.052)	0.022 (0.009 - 0.045)
**Age-standardised (rate per capita)**	0.074 (0.060 - 0.090)	0.074 (0.060 - 0.090)	0.074 (0.060 - 0.090)	0.132 (0.100 - 0.172)	0.132 (0.100 - 0.172)	0.132 (0.100 - 0.172)	0.017 (0.007 - 0.035)	0.017 (0.007 - 0.035)	0.017 (0.007 - 0.035)
**Total number of cases (thousands)**	13 (10 - 15)	12 (10 - 15)	10 (8 - 12)	22 (17 - 29)	22 (16 - 28)	18 (13 - 23)	0 (0 - 0)	0 (0 - 0)	0 (0 - 0)
**Fiji**									
**Under-5 years (rate per capita)**	0.323 (0.248 - 0.408)	0.329 (0.256 - 0.416)	0.324 (0.249 - 0.409)	0.550 (0.318 - 0.769)	0.568 (0.330 - 0.771)	0.552 (0.319 - 0.771)	0.076 (0.032 - 0.155)	0.077 (0.032 - 0.152)	0.076 (0.032 - 0.151)
**5-14 years (rate per capita)**	0.260 (0.195 - 0.329)	0.311 (0.264 - 0.366)	0.257 (0.193 - 0.325)	0.496 (0.353 - 0.694)	0.508 (0.363 - 0.705)	0.489 (0.348 - 0.685)	0.061 (0.025 - 0.130)	0.073 (0.031 - 0.146)	0.060 (0.025 - 0.130)
**All ages (rate per capita)**	0.110 (0.091 - 0.132)	0.099 (0.083 - 0.114)	0.080 (0.066 - 0.097)	0.201 (0.149 - 0.265)	0.165 (0.122 - 0.218)	0.147 (0.110 - 0.194)	0.026 (0.011 - 0.052)	0.023 (0.010 - 0.045)	0.019 (0.008 - 0.038)
**Age-standardised (rate per capita)**	0.072 (0.059 - 0.086)	0.083 (0.070 - 0.096)	0.072 (0.059 - 0.086)	0.131 (0.098 - 0.174)	0.138 (0.103 - 0.183)	0.131 (0.098 - 0.174)	0.017 (0.007 - 0.034)	0.019 (0.008 - 0.038)	0.017 (0.007 - 0.034)
**Total number of cases (thousands)**	81 (67 - 97)	82 (69 - 94)	72 (59 - 86)	147 (110 - 195)	136 (101 - 180)	131 (98 - 173)	0 (0 - 0)	0 (0 - 0)	0 (0 - 0)
**Guam**									
**Under-5 years (rate per capita)**	0.336 (0.250 - 0.452)	0.341 (0.254 - 0.459)	0.336 (0.251 - 0.453)	0.554 (0.321 - 0.760)	0.562 (0.326 - 0.772)	0.555 (0.322 - 0.762)	0.079 (0.034 - 0.154)	0.080 (0.034 - 0.165)	0.079 (0.034 - 0.154)
**5-14 years (rate per capita)**	0.262 (0.199 - 0.336)	0.255 (0.194 - 0.329)	0.245 (0.186 - 0.317)	0.496 (0.350 - 0.694)	0.484 (0.340 - 0.679)	0.466 (0.326 - 0.657)	0.062 (0.026 - 0.129)	0.060 (0.025 - 0.126)	0.058 (0.024 - 0.125)
**All ages (rate per capita)**	0.089 (0.072 - 0.109)	0.084 (0.069 - 0.103)	0.070 (0.057 - 0.086)	0.159 (0.119 - 0.205)	0.151 (0.114 - 0.196)	0.126 (0.095 - 0.165)	0.021 (0.009 - 0.043)	0.020 (0.008 - 0.041)	0.016 (0.007 - 0.033)
**Age-standardised (rate per capita)**	0.074 (0.060 - 0.090)	0.074 (0.060 - 0.090)	0.074 (0.060 - 0.090)	0.132 (0.100 - 0.172)	0.132 (0.100 - 0.172)	0.132 (0.100 - 0.172)	0.017 (0.007 - 0.036)	0.017 (0.007 - 0.036)	0.017 (0.007 - 0.035)
**Total number of cases (thousands)**	12 (10 - 14)	13 (11 - 16)	12 (10 - 15)	21 (16 - 27)	24 (18 - 31)	21 (16 - 28)	0 (0 - 0)	0 (0 - 0)	0 (0 - 0)
**Kiribati**									
**Under-5 years (rate per capita)**	0.332 (0.247 - 0.446)	0.333 (0.249 - 0.449)	0.334 (0.249 - 0.449)	0.547 (0.317 - 0.751)	0.550 (0.319 - 0.755)	0.551 (0.319 - 0.756)	0.078 (0.034 - 0.154)	0.078 (0.033 - 0.158)	0.078 (0.033 - 0.156)
**5-14 years (rate per capita)**	0.271 (0.207 - 0.348)	0.267 (0.203 - 0.342)	0.277 (0.211 - 0.354)	0.513 (0.363 - 0.710)	0.504 (0.357 - 0.702)	0.523 (0.370 - 0.719)	0.063 (0.026 - 0.132)	0.062 (0.026 - 0.128)	0.064 (0.026 - 0.135)
**All ages (rate per capita)**	0.119 (0.097 - 0.146)	0.110 (0.090 - 0.134)	0.105 (0.085 - 0.127)	0.213 (0.159 - 0.275)	0.198 (0.150 - 0.257)	0.187 (0.141 - 0.242)	0.028 (0.012 - 0.057)	0.026 (0.011 - 0.052)	0.024 (0.010 - 0.049)
**Age-standardised (rate per capita)**	0.074 (0.060 - 0.090)	0.074 (0.060 - 0.090)	0.074 (0.060 - 0.090)	0.132 (0.100 - 0.172)	0.132 (0.100 - 0.172)	0.132 (0.100 - 0.172)	0.017 (0.007 - 0.035)	0.017 (0.007 - 0.034)	0.017 (0.007 - 0.035)
**Total number of cases (thousands)**	9 (7 - 11)	10 (8 - 13)	12 (10 - 14)	15 (12 - 20)	19 (14 - 24)	21 (16 - 27)	0 (0 - 0)	0 (0 - 0)	0 (0 - 0)
**Marshall Islands**									
**Under-5 years (rate per capita)**	0.333 (0.249 - 0.448)	0.334 (0.249 - 0.450)	0.339 (0.253 - 0.457)	0.549 (0.319 - 0.754)	0.551 (0.320 - 0.757)	0.560 (0.325 - 0.768)	0.078 (0.033 - 0.156)	0.078 (0.033 - 0.155)	0.079 (0.034 - 0.162)
**5-14 years (rate per capita)**	0.260 (0.198 - 0.335)	0.264 (0.201 - 0.339)	0.257 (0.195 - 0.331)	0.493 (0.348 - 0.691)	0.500 (0.354 - 0.699)	0.487 (0.343 - 0.684)	0.061 (0.025 - 0.128)	0.062 (0.026 - 0.130)	0.060 (0.025 - 0.128)
**All ages (rate per capita)**	0.126 (0.102 - 0.155)	0.115 (0.093 - 0.140)	0.103 (0.084 - 0.126)	0.224 (0.167 - 0.291)	0.204 (0.153 - 0.264)	0.184 (0.139 - 0.240)	0.030 (0.013 - 0.060)	0.027 (0.012 - 0.054)	0.024 (0.010 - 0.049)
**Age-standardised (rate per capita)**	0.074 (0.060 - 0.090)	0.074 (0.060 - 0.090)	0.074 (0.060 - 0.090)	0.132 (0.100 - 0.172)	0.132 (0.100 - 0.172)	0.132 (0.100 - 0.172)	0.017 (0.007 - 0.035)	0.017 (0.007 - 0.035)	0.017 (0.007 - 0.035)
**Total number of cases (thousands)**	6 (5 - 7)	7 (6 - 8)	7 (6 - 9)	11 (8 - 14)	12 (9 - 16)	13 (10 - 17)	0 (0 - 0)	0 (0 - 0)	0 (0 - 0)
**Northern Mariana Islands**									
**Under-5 years (rate per capita)**	0.328 (0.245 - 0.442)	0.334 (0.249 - 0.450)	0.334 (0.249 - 0.450)	0.541 (0.314 - 0.743)	0.551 (0.320 - 0.756)	0.551 (0.320 - 0.757)	0.077 (0.032 - 0.158)	0.079 (0.034 - 0.160)	0.078 (0.033 - 0.158)
**5-14 years (rate per capita)**	0.264 (0.200 - 0.338)	0.270 (0.206 - 0.346)	0.262 (0.199 - 0.336)	0.499 (0.353 - 0.697)	0.510 (0.361 - 0.708)	0.496 (0.350 - 0.694)	0.062 (0.026 - 0.132)	0.063 (0.026 - 0.135)	0.062 (0.026 - 0.129)
**All ages (rate per capita)**	0.068 (0.055 - 0.083)	0.065 (0.053 - 0.079)	0.059 (0.049 - 0.073)	0.121 (0.091 - 0.157)	0.116 (0.088 - 0.151)	0.107 (0.081 - 0.139)	0.016 (0.007 - 0.033)	0.015 (0.007 - 0.031)	0.014 (0.006 - 0.029)
**Age-standardised (rate per capita)**	0.074 (0.060 - 0.090)	0.074 (0.060 - 0.090)	0.074 (0.060 - 0.090)	0.132 (0.100 - 0.172)	0.132 (0.100 - 0.172)	0.132 (0.100 - 0.172)	0.017 (0.007 - 0.036)	0.017 (0.007 - 0.035)	0.017 (0.008 - 0.036)
**Total number of cases (thousands)**	3 (2 - 4)	5 (4 - 7)	7 (6 - 8)	5 (4 - 7)	10 (7 - 12)	12 (9 - 16)	0 (0 - 0)	0 (0 - 0)	0 (0 - 0)
**Papua New Guinea**									
**Under-5 years (rate per capita)**	0.331 (0.247 - 0.446)	0.333 (0.249 - 0.449)	0.335 (0.250 - 0.451)	0.546 (0.317 - 0.750)	0.550 (0.319 - 0.755)	0.552 (0.320 - 0.758)	0.077 (0.033 - 0.157)	0.078 (0.034 - 0.154)	0.078 (0.033 - 0.159)
**5-14 years (rate per capita)**	0.260 (0.197 - 0.334)	0.267 (0.203 - 0.343)	0.258 (0.196 - 0.332)	0.492 (0.347 - 0.689)	0.506 (0.358 - 0.704)	0.488 (0.344 - 0.685)	0.060 (0.025 - 0.126)	0.062 (0.026 - 0.133)	0.060 (0.025 - 0.126)
**All ages (rate per capita)**	0.121 (0.099 - 0.148)	0.117 (0.095 - 0.142)	0.106 (0.087 - 0.130)	0.217 (0.163 - 0.282)	0.209 (0.157 - 0.271)	0.190 (0.144 - 0.247)	0.028 (0.012 - 0.057)	0.027 (0.012 - 0.056)	0.025 (0.011 - 0.051)
**Age-standardised (rate per capita)**	0.074 (0.060 - 0.090)	0.074 (0.060 - 0.090)	0.074 (0.060 - 0.090)	0.132 (0.100 - 0.172)	0.132 (0.100 - 0.172)	0.132 (0.100 - 0.172)	0.017 (0.007 - 0.035)	0.017 (0.007 - 0.035)	0.017 (0.007 - 0.035)
**Total number of cases (thousands)**	505 (411 - 616)	711 (579 - 867)	811 (660 - 989)	903 (680 - 1,174)	1,272 (956 - 1,653)	1,453 (1,096 - 1,889)	0 (0 - 0)	0 (0 - 0)	0 (0 - 0)
**Samoa**									
**Under-5 years (rate per capita)**	0.338 (0.252 - 0.455)	0.337 (0.252 - 0.454)	0.340 (0.254 - 0.458)	0.558 (0.324 - 0.766)	0.556 (0.323 - 0.764)	0.560 (0.325 - 0.769)	0.079 (0.034 - 0.164)	0.079 (0.034 - 0.157)	0.080 (0.033 - 0.162)
**5-14 years (rate per capita)**	0.252 (0.191 - 0.325)	0.257 (0.195 - 0.331)	0.262 (0.199 - 0.336)	0.478 (0.335 - 0.672)	0.487 (0.343 - 0.683)	0.496 (0.350 - 0.694)	0.059 (0.024 - 0.126)	0.060 (0.025 - 0.127)	0.061 (0.025 - 0.129)
**All ages (rate per capita)**	0.119 (0.097 - 0.146)	0.114 (0.093 - 0.140)	0.108 (0.088 - 0.132)	0.213 (0.159 - 0.277)	0.204 (0.154 - 0.266)	0.194 (0.147 - 0.253)	0.028 (0.012 - 0.057)	0.027 (0.011 - 0.055)	0.025 (0.011 - 0.052)

	Prevalence 1990	Prevalence 2005	Prevalence 2015	Incidence 1990	Incidence 2005	Incidence 2015	YLD rate 1990	YLD rate 2005	YLD rate 2015
**Age-standardised (rate per capita)**	0.074 (0.060 - 0.090)	0.074 (0.060 - 0.090)	0.074 (0.060 - 0.090)	0.132 (0.100 - 0.172)	0.132 (0.100 - 0.172)	0.132 (0.100 - 0.172)	0.017 (0.007 - 0.035)	0.017 (0.007 - 0.036)	0.017 (0.007 - 0.035)
**Total number of cases (thousands)**	20 (16 - 24)	21 (17 - 25)	21 (17 - 26)	35 (26 - 46)	37 (28 - 48)	38 (28 - 49)	0 (0 - 0)	0 (0 - 0)	0 (0 - 0)
**Solomon Islands**									
**Under-5 years (rate per capita)**	0.333 (0.248 - 0.448)	0.333 (0.249 - 0.449)	0.337 (0.252 - 0.454)	0.549 (0.318 - 0.753)	0.550 (0.319 - 0.755)	0.556 (0.323 - 0.763)	0.078 (0.033 - 0.159)	0.078 (0.033 - 0.156)	0.079 (0.034 - 0.159)
**5-14 years (rate per capita)**	0.259 (0.197 - 0.333)	0.261 (0.198 - 0.335)	0.261 (0.198 - 0.335)	0.491 (0.346 - 0.688)	0.494 (0.349 - 0.693)	0.494 (0.349 - 0.692)	0.061 (0.025 - 0.129)	0.061 (0.025 - 0.129)	0.061 (0.026 - 0.132)
**All ages (rate per capita)**	0.133 (0.108 - 0.162)	0.120 (0.098 - 0.147)	0.115 (0.093 - 0.140)	0.237 (0.177 - 0.308)	0.215 (0.161 - 0.280)	0.205 (0.155 - 0.267)	0.031 (0.013 - 0.063)	0.028 (0.012 - 0.058)	0.027 (0.012 - 0.054)
**Age-standardised (rate per capita)**	0.074 (0.060 - 0.090)	0.074 (0.060 - 0.090)	0.074 (0.060 - 0.090)	0.132 (0.100 - 0.172)	0.132 (0.100 - 0.172)	0.132 (0.100 - 0.172)	0.017 (0.007 - 0.035)	0.017 (0.007 - 0.035)	0.017 (0.007 - 0.035)
**Total number of cases (thousands)**	42 (34 - 51)	57 (46 - 69)	67 (55 - 82)	75 (56 - 97)	102 (76 - 132)	120 (91 - 156)	0 (0 - 0)	0 (0 - 0)	0 (0 - 0)
**Tonga**									
**Under-5 years (rate per capita)**	0.339 (0.253 - 0.456)	0.336 (0.250 - 0.452)	0.340 (0.254 - 0.457)	0.558 (0.324 - 0.767)	0.553 (0.321 - 0.760)	0.560 (0.325 - 0.770)	0.079 (0.034 - 0.160)	0.079 (0.033 - 0.159)	0.080 (0.034 - 0.162)
**5-14 years (rate per capita)**	0.269 (0.205 - 0.345)	0.258 (0.196 - 0.332)	0.254 (0.193 - 0.328)	0.509 (0.360 - 0.707)	0.489 (0.344 - 0.685)	0.482 (0.339 - 0.677)	0.063 (0.026 - 0.132)	0.061 (0.025 - 0.129)	0.060 (0.025 - 0.127)
**All ages (rate per capita)**	0.119 (0.097 - 0.145)	0.109 (0.089 - 0.133)	0.105 (0.085 - 0.128)	0.212 (0.159 - 0.275)	0.195 (0.147 - 0.254)	0.188 (0.142 - 0.245)	0.028 (0.012 - 0.057)	0.026 (0.011 - 0.052)	0.025 (0.011 - 0.050)
**Age-standardised (rate per capita)**	0.074 (0.060 - 0.090)	0.074 (0.060 - 0.090)	0.074 (0.060 - 0.090)	0.132 (0.100 - 0.172)	0.132 (0.100 - 0.172)	0.132 (0.100 - 0.172)	0.017 (0.007 - 0.035)	0.017 (0.007 - 0.035)	0.017 (0.007 - 0.035)
**Total number of cases (thousands)**	11 (9 - 14)	11 (9 - 13)	11 (9 - 14)	21 (15 - 27)	20 (15 - 26)	20 (15 - 26)	0 (0 - 0)	0 (0 - 0)	0 (0 - 0)
**Vanuatu**									
**Under-5 years (rate per capita)**	0.333 (0.249 - 0.449)	0.336 (0.251 - 0.452)	0.335 (0.250 - 0.451)	0.550 (0.319 - 0.755)	0.554 (0.321 - 0.761)	0.552 (0.320 - 0.758)	0.078 (0.034 - 0.156)	0.079 (0.033 - 0.155)	0.078 (0.034 - 0.158)
**5-14 years (rate per capita)**	0.267 (0.203 - 0.343)	0.261 (0.199 - 0.336)	0.268 (0.204 - 0.344)	0.506 (0.358 - 0.705)	0.495 (0.349 - 0.693)	0.508 (0.359 - 0.706)	0.063 (0.026 - 0.131)	0.061 (0.025 - 0.130)	0.063 (0.027 - 0.133)
**All ages (rate per capita)**	0.129 (0.105 - 0.157)	0.114 (0.093 - 0.139)	0.105 (0.086 - 0.128)	0.231 (0.173 - 0.299)	0.204 (0.154 - 0.265)	0.189 (0.142 - 0.245)	0.030 (0.013 - 0.062)	0.027 (0.011 - 0.054)	0.025 (0.011 - 0.050)
**Age-standardised (rate per capita)**	0.074 (0.060 - 0.090)	0.074 (0.060 - 0.090)	0.074 (0.060 - 0.090)	0.132 (0.100 - 0.172)	0.132 (0.100 - 0.172)	0.132 (0.100 - 0.172)	0.017 (0.007 - 0.035)	0.017 (0.007 - 0.035)	0.017 (0.007 - 0.035)
**Total number of cases (thousands)**	19 (15 - 23)	24 (19 - 29)	28 (22 - 34)	34 (26 - 44)	43 (32 - 56)	50 (37 - 64)	0 (0 - 0)	0 (0 - 0)	0 (0 - 0)
**North Africa and Middle East**									
**Under-5 years (rate per capita)**	0.375 (0.276 - 0.473)	0.375 (0.283 - 0.466)	0.382 (0.280 - 0.479)	0.562 (0.348 - 0.751)	0.574 (0.395 - 0.745)	0.568 (0.356 - 0.757)	0.088 (0.038 - 0.173)	0.088 (0.038 - 0.171)	0.090 (0.038 - 0.176)
**5-14 years (rate per capita)**	0.284 (0.214 - 0.366)	0.269 (0.200 - 0.347)	0.272 (0.197 - 0.357)	0.526 (0.351 - 0.760)	0.496 (0.326 - 0.732)	0.516 (0.335 - 0.757)	0.067 (0.028 - 0.139)	0.063 (0.027 - 0.131)	0.064 (0.027 - 0.133)
**All ages (rate per capita)**	0.134 (0.109 - 0.163)	0.103 (0.084 - 0.124)	0.097 (0.079 - 0.119)	0.228 (0.170 - 0.301)	0.175 (0.132 - 0.231)	0.166 (0.122 - 0.221)	0.032 (0.014 - 0.064)	0.024 (0.010 - 0.048)	0.023 (0.010 - 0.046)
**Age-standardised (rate per capita)**	0.080 (0.065 - 0.097)	0.081 (0.067 - 0.098)	0.080 (0.064 - 0.098)	0.136 (0.101 - 0.181)	0.138 (0.104 - 0.183)	0.137 (0.100 - 0.183)	0.019 (0.008 - 0.038)	0.019 (0.008 - 0.038)	0.019 (0.008 - 0.037)
**Total number of cases (thousands)**	45,167 (36,688 - 54,696)	47,653 (39,243 - 57,625)	54,912 (44,541 - 67,352)	76,626 (57,029 - 101,244)	81,444 (61,362 - 107,557)	93,998 (69,248 - 125,135)	11 (5 - 22)	11 (5 - 22)	13 (6 - 26)
**North Africa and Middle East**									
**Under-5 years (rate per capita)**	0.375 (0.276 - 0.473)	0.375 (0.283 - 0.466)	0.382 (0.280 - 0.479)	0.562 (0.348 - 0.751)	0.574 (0.395 - 0.745)	0.568 (0.356 - 0.757)	0.088 (0.038 - 0.173)	0.088 (0.038 - 0.171)	0.090 (0.038 - 0.176)
**5-14 years (rate per capita)**	0.284 (0.214 - 0.366)	0.269 (0.200 - 0.347)	0.272 (0.197 - 0.357)	0.526 (0.351 - 0.760)	0.496 (0.326 - 0.732)	0.516 (0.335 - 0.757)	0.067 (0.028 - 0.139)	0.063 (0.027 - 0.131)	0.064 (0.027 - 0.133)
**All ages (rate per capita)**	0.134 (0.109 - 0.163)	0.103 (0.084 - 0.124)	0.097 (0.079 - 0.119)	0.228 (0.170 - 0.301)	0.175 (0.132 - 0.231)	0.166 (0.122 - 0.221)	0.032 (0.014 - 0.064)	0.024 (0.010 - 0.048)	0.023 (0.010 - 0.046)
**Age-standardised (rate per capita)**	0.080 (0.065 - 0.097)	0.081 (0.067 - 0.098)	0.080 (0.064 - 0.098)	0.136 (0.101 - 0.181)	0.138 (0.104 - 0.183)	0.137 (0.100 - 0.183)	0.019 (0.008 - 0.038)	0.019 (0.008 - 0.038)	0.019 (0.008 - 0.037)
**Total number of cases (thousands)**	45,167 (36,688 - 54,696)	47,653 (39,243 - 57,625)	54,912 (44,541 - 67,352)	76,626 (57,029 - 101,244)	81,444 (61,362 - 107,557)	93,998 (69,248 - 125,135)	11 (5 - 22)	11 (5 - 22)	13 (6 - 26)
**Afghanistan**									
**Under-5 years (rate per capita)**	0.421 (0.291 - 0.529)	0.423 (0.293 - 0.532)	0.430 (0.297 - 0.541)	0.579 (0.350 - 0.773)	0.582 (0.352 - 0.778)	0.591 (0.357 - 0.790)	0.098 (0.041 - 0.196)	0.099 (0.042 - 0.200)	0.100 (0.043 - 0.204)
**5-14 years (rate per capita)**	0.259 (0.168 - 0.372)	0.263 (0.170 - 0.377)	0.249 (0.161 - 0.358)	0.564 (0.325 - 0.872)	0.571 (0.330 - 0.882)	0.543 (0.311 - 0.842)	0.060 (0.024 - 0.131)	0.061 (0.025 - 0.132)	0.058 (0.023 - 0.125)
**All ages (rate per capita)**	0.161 (0.123 - 0.207)	0.156 (0.119 - 0.201)	0.138 (0.106 - 0.180)	0.280 (0.200 - 0.375)	0.274 (0.194 - 0.371)	0.247 (0.172 - 0.339)	0.037 (0.016 - 0.077)	0.036 (0.016 - 0.074)	0.032 (0.014 - 0.066)
**Age-standardised (rate per capita)**	0.080 (0.062 - 0.105)	0.080 (0.062 - 0.105)	0.080 (0.062 - 0.105)	0.144 (0.100 - 0.198)	0.144 (0.100 - 0.198)	0.144 (0.100 - 0.198)	0.019 (0.008 - 0.038)	0.019 (0.008 - 0.039)	0.019 (0.008 - 0.038)
**Total number of cases (thousands)**	1,981 (1,519 - 2,544)	3,807 (2,900 - 4,919)	4,500 (3,448 - 5,865)	3,442 (2,465 - 4,615)	6,693 (4,741 - 9,053)	8,062 (5,608 - 11,068)	0 (0 - 1)	1 (0 - 2)	1 (0 - 2)
**Algeria**									
**Under-5 years (rate per capita)**	0.432 (0.299 - 0.544)	0.424 (0.293 - 0.534)	0.431 (0.298 - 0.542)	0.595 (0.359 - 0.795)	0.583 (0.352 - 0.780)	0.593 (0.358 - 0.792)	0.101 (0.043 - 0.206)	0.100 (0.041 - 0.202)	0.101 (0.042 - 0.200)
**5-14 years (rate per capita)**	0.252 (0.163 - 0.361)	0.220 (0.141 - 0.319)	0.268 (0.174 - 0.383)	0.548 (0.315 - 0.848)	0.482 (0.272 - 0.752)	0.582 (0.337 - 0.898)	0.059 (0.024 - 0.126)	0.052 (0.021 - 0.111)	0.063 (0.026 - 0.136)
**All ages (rate per capita)**	0.138 (0.105 - 0.179)	0.084 (0.064 - 0.110)	0.095 (0.072 - 0.123)	0.245 (0.171 - 0.336)	0.151 (0.104 - 0.209)	0.167 (0.118 - 0.225)	0.032 (0.014 - 0.067)	0.020 (0.008 - 0.040)	0.022 (0.010 - 0.045)
**Age-standardised (rate per capita)**	0.080 (0.062 - 0.105)	0.080 (0.062 - 0.105)	0.080 (0.062 - 0.105)	0.144 (0.100 - 0.198)	0.144 (0.100 - 0.198)	0.144 (0.100 - 0.198)	0.019 (0.008 - 0.039)	0.019 (0.008 - 0.039)	0.019 (0.008 - 0.039)
**Total number of cases (thousands)**	3,571 (2,732 - 4,637)	2,789 (2,128 - 3,665)	3,766 (2,870 - 4,859)	6,370 (4,433 - 8,715)	5,036 (3,485 - 6,995)	6,616 (4,694 - 8,937)	1 (0 - 2)	1 (0 - 1)	1 (0 - 2)
**Bahrain**									
**Under-5 years (rate per capita)**	0.431 (0.298 - 0.542)	0.425 (0.294 - 0.535)	0.433 (0.299 - 0.544)	0.593 (0.358 - 0.792)	0.585 (0.354 - 0.782)	0.595 (0.360 - 0.795)	0.101 (0.043 - 0.204)	0.100 (0.042 - 0.201)	0.102 (0.043 - 0.207)
**5-14 years (rate per capita)**	0.261 (0.169 - 0.374)	0.258 (0.167 - 0.370)	0.254 (0.164 - 0.364)	0.568 (0.328 - 0.878)	0.560 (0.323 - 0.867)	0.552 (0.318 - 0.855)	0.061 (0.025 - 0.133)	0.061 (0.024 - 0.130)	0.060 (0.024 - 0.126)
**All ages (rate per capita)**	0.111 (0.085 - 0.142)	0.083 (0.063 - 0.109)	0.066 (0.051 - 0.086)	0.192 (0.138 - 0.258)	0.150 (0.104 - 0.208)	0.119 (0.083 - 0.163)	0.026 (0.011 - 0.053)	0.019 (0.008 - 0.040)	0.016 (0.007 - 0.031)
**Age-standardised (rate per capita)**	0.080 (0.062 - 0.105)	0.080 (0.062 - 0.105)	0.080 (0.062 - 0.105)	0.144 (0.100 - 0.198)	0.144 (0.100 - 0.198)	0.144 (0.100 - 0.198)	0.019 (0.008 - 0.039)	0.019 (0.008 - 0.038)	0.019 (0.008 - 0.038)
**Total number of cases (thousands)**	55 (42 - 71)	72 (55 - 94)	90 (69 - 118)	96 (69 - 129)	130 (90 - 179)	162 (113 - 223)	0 (0 - 0)	0 (0 - 0)	0 (0 - 0)
**Egypt**									
**Under-5 years (rate per capita)**	0.430 (0.297 - 0.541)	0.427 (0.296 - 0.538)	0.428 (0.296 - 0.538)	0.592 (0.357 - 0.791)	0.588 (0.355 - 0.786)	0.589 (0.355 - 0.787)	0.101 (0.042 - 0.202)	0.100 (0.042 - 0.204)	0.100 (0.043 - 0.198)
**5-14 years (rate per capita)**	0.258 (0.167 - 0.371)	0.237 (0.152 - 0.341)	0.250 (0.162 - 0.360)	0.562 (0.324 - 0.869)	0.516 (0.294 - 0.808)	0.545 (0.313 - 0.844)	0.061 (0.025 - 0.133)	0.056 (0.023 - 0.118)	0.059 (0.024 - 0.126)
**All ages (rate per capita)**	0.132 (0.101 - 0.171)	0.100 (0.077 - 0.130)	0.105 (0.080 - 0.136)	0.234 (0.165 - 0.319)	0.179 (0.124 - 0.245)	0.185 (0.131 - 0.250)	0.031 (0.013 - 0.063)	0.023 (0.010 - 0.048)	0.025 (0.011 - 0.050)
**Age-standardised (rate per capita)**	0.080 (0.062 - 0.105)	0.080 (0.062 - 0.105)	0.080 (0.062 - 0.105)	0.144 (0.100 - 0.198)	0.144 (0.100 - 0.198)	0.144 (0.100 - 0.198)	0.019 (0.008 - 0.038)	0.019 (0.008 - 0.039)	0.019 (0.008 - 0.038)
**Total number of cases (thousands)**	7,457 (5,684 - 9,653)	7,493 (5,740 - 9,757)	9,592 (7,311 - 12,387)	13,199 (9,271 - 17,946)	13,368 (9,287 - 18,320)	16,848 (11,943 - 22,798)	2 (1 - 4)	2 (1 - 4)	2 (1 - 5)
**Iran**									
**Under-5 years (rate per capita)**	0.369 (0.284 - 0.478)	0.365 (0.317 - 0.451)	0.368 (0.283 - 0.476)	0.623 (0.377 - 0.822)	0.731 (0.566 - 0.852)	0.621 (0.376 - 0.820)	0.087 (0.037 - 0.173)	0.086 (0.036 - 0.171)	0.086 (0.037 - 0.174)
**5-14 years (rate per capita)**	0.347 (0.293 - 0.415)	0.339 (0.307 - 0.382)	0.342 (0.288 - 0.409)	0.570 (0.441 - 0.755)	0.516 (0.388 - 0.709)	0.562 (0.434 - 0.745)	0.082 (0.034 - 0.165)	0.080 (0.034 - 0.156)	0.081 (0.033 - 0.164)
**All ages (rate per capita)**	0.160 (0.136 - 0.187)	0.092 (0.084 - 0.102)	0.083 (0.070 - 0.097)	0.266 (0.210 - 0.337)	0.155 (0.128 - 0.190)	0.137 (0.108 - 0.174)	0.038 (0.016 - 0.076)	0.022 (0.009 - 0.042)	0.019 (0.008 - 0.039)
**Age-standardised (rate per capita)**	0.088 (0.075 - 0.103)	0.099 (0.090 - 0.109)	0.088 (0.075 - 0.103)	0.147 (0.116 - 0.186)	0.165 (0.137 - 0.200)	0.147 (0.116 - 0.186)	0.021 (0.009 - 0.042)	0.023 (0.010 - 0.045)	0.021 (0.009 - 0.042)
**Total number of cases (thousands)**	8,971 (7,633 - 10,483)	6,498 (5,911 - 7,228)	6,535 (5,563 - 7,634)	14,893 (11,769 - 18,855)	10,928 (9,019 - 13,443)	10,851 (8,572 - 13,747)	2 (1 - 4)	2 (1 - 3)	2 (1 - 3)
**Iraq**									
**Under-5 years (rate per capita)**	0.426 (0.295 - 0.536)	0.426 (0.294 - 0.535)	0.427 (0.295 - 0.537)	0.586 (0.354 - 0.783)	0.586 (0.354 - 0.783)	0.587 (0.354 - 0.784)	0.099 (0.042 - 0.197)	0.100 (0.042 - 0.201)	0.100 (0.043 - 0.203)
**5-14 years (rate per capita)**	0.251 (0.162 - 0.361)	0.254 (0.164 - 0.365)	0.257 (0.166 - 0.368)	0.546 (0.314 - 0.847)	0.553 (0.318 - 0.857)	0.558 (0.322 - 0.864)	0.059 (0.023 - 0.126)	0.060 (0.024 - 0.129)	0.060 (0.024 - 0.130)
**All ages (rate per capita)**	0.145 (0.111 - 0.188)	0.134 (0.103 - 0.174)	0.132 (0.101 - 0.171)	0.257 (0.180 - 0.351)	0.238 (0.167 - 0.324)	0.233 (0.164 - 0.317)	0.034 (0.015 - 0.068)	0.032 (0.013 - 0.065)	0.031 (0.013 - 0.063)
**Age-standardised (rate per capita)**	0.080 (0.062 - 0.105)	0.080 (0.062 - 0.105)	0.080 (0.062 - 0.105)	0.144 (0.100 - 0.198)	0.144 (0.100 - 0.198)	0.144 (0.100 - 0.198)	0.019 (0.008 - 0.038)	0.019 (0.008 - 0.039)	0.019 (0.008 - 0.038)
**Total number of cases (thousands)**	2,534 (1,934 - 3,279)	3,635 (2,774 - 4,706)	4,804 (3,661 - 6,216)	4,493 (3,148 - 6,122)	6,446 (4,518 - 8,780)	8,495 (5,972 - 11,545)	1 (0 - 1)	1 (0 - 2)	1 (0 - 2)
**Jordan**									
**Under-5 years (rate per capita)**	0.319 (0.236 - 0.416)	0.307 (0.251 - 0.359)	0.322 (0.239 - 0.421)	0.536 (0.295 - 0.751)	0.342 (0.278 - 0.434)	0.542 (0.298 - 0.759)	0.075 (0.032 - 0.150)	0.072 (0.030 - 0.139)	0.076 (0.032 - 0.157)
**5-14 years (rate per capita)**	0.307 (0.243 - 0.379)	0.288 (0.223 - 0.355)	0.298 (0.235 - 0.369)	0.463 (0.333 - 0.662)	0.432 (0.296 - 0.649)	0.449 (0.323 - 0.645)	0.072 (0.030 - 0.149)	0.068 (0.029 - 0.136)	0.070 (0.029 - 0.145)
**All ages (rate per capita)**	0.143 (0.120 - 0.169)	0.112 (0.095 - 0.130)	0.108 (0.091 - 0.129)	0.224 (0.164 - 0.296)	0.151 (0.118 - 0.201)	0.170 (0.125 - 0.226)	0.034 (0.014 - 0.067)	0.026 (0.011 - 0.052)	0.025 (0.011 - 0.051)

	Prevalence 1990	Prevalence 2005	Prevalence 2015	Incidence 1990	Incidence 2005	Incidence 2015	YLD rate 1990	YLD rate 2005	YLD rate 2015
**Age-standardised (rate per capita)**	0.079 (0.066 - 0.094)	0.076 (0.064 - 0.088)	0.079 (0.066 - 0.094)	0.124 (0.091 - 0.165)	0.103 (0.080 - 0.137)	0.124 (0.091 - 0.165)	0.019 (0.008 - 0.037)	0.018 (0.008 - 0.035)	0.019 (0.008 - 0.037)
**Total number of cases (thousands)**	475 (398 - 564)	600 (507 - 698)	820 (687 - 974)	746 (548 - 987)	810 (635 - 1,075)	1,290 (944 - 1,709)	0 (0 - 0)	0 (0 - 0)	0 (0 - 0)
**Kuwait**									
**Under-5 years (rate per capita)**	0.264 (0.217 - 0.324)	0.285 (0.236 - 0.345)	0.292 (0.218 - 0.367)	0.435 (0.365 - 0.523)	0.451 (0.339 - 0.610)	0.540 (0.287 - 0.784)	0.101 (0.043 - 0.200)	0.109 (0.047 - 0.211)	0.111 (0.046 - 0.226)
**5-14 years (rate per capita)**	0.285 (0.227 - 0.350)	0.294 (0.230 - 0.366)	0.321 (0.251 - 0.393)	0.458 (0.330 - 0.672)	0.435 (0.296 - 0.636)	0.514 (0.360 - 0.730)	0.109 (0.044 - 0.216)	0.112 (0.047 - 0.228)	0.122 (0.051 - 0.254)
**All ages (rate per capita)**	0.097 (0.082 - 0.114)	0.077 (0.066 - 0.090)	0.069 (0.058 - 0.082)	0.156 (0.126 - 0.204)	0.117 (0.093 - 0.155)	0.118 (0.085 - 0.156)	0.037 (0.015 - 0.073)	0.029 (0.013 - 0.058)	0.026 (0.011 - 0.053)
**Age-standardised (rate per capita)**	0.069 (0.059 - 0.081)	0.073 (0.062 - 0.086)	0.076 (0.063 - 0.089)	0.112 (0.090 - 0.146)	0.112 (0.088 - 0.149)	0.128 (0.093 - 0.172)	0.026 (0.011 - 0.052)	0.028 (0.012 - 0.055)	0.029 (0.012 - 0.057)
**Total number of cases (thousands)**	198 (168 - 233)	176 (150 - 204)	271 (226 - 321)	321 (258 - 419)	267 (211 - 353)	461 (331 - 608)	0 (0 - 0)	0 (0 - 0)	0 (0 - 0)
**Lebanon**									
**Under-5 years (rate per capita)**	0.434 (0.300 - 0.546)	0.438 (0.303 - 0.551)	0.419 (0.290 - 0.527)	0.597 (0.361 - 0.798)	0.603 (0.364 - 0.806)	0.577 (0.348 - 0.771)	0.101 (0.043 - 0.206)	0.103 (0.044 - 0.207)	0.098 (0.041 - 0.195)
**5-14 years (rate per capita)**	0.243 (0.157 - 0.350)	0.229 (0.147 - 0.331)	0.225 (0.144 - 0.325)	0.529 (0.303 - 0.826)	0.501 (0.285 - 0.784)	0.492 (0.279 - 0.768)	0.056 (0.023 - 0.122)	0.054 (0.022 - 0.114)	0.053 (0.022 - 0.112)
**All ages (rate per capita)**	0.108 (0.083 - 0.141)	0.077 (0.059 - 0.103)	0.064 (0.048 - 0.084)	0.193 (0.135 - 0.264)	0.144 (0.098 - 0.202)	0.118 (0.080 - 0.165)	0.025 (0.011 - 0.051)	0.018 (0.008 - 0.037)	0.015 (0.006 - 0.030)
**Age-standardised (rate per capita)**	0.080 (0.062 - 0.105)	0.080 (0.062 - 0.105)	0.080 (0.062 - 0.105)	0.144 (0.100 - 0.198)	0.144 (0.100 - 0.198)	0.144 (0.100 - 0.198)	0.019 (0.008 - 0.038)	0.019 (0.008 - 0.038)	0.019 (0.008 - 0.038)
**Total number of cases (thousands)**	296 (227 - 384)	305 (231 - 405)	368 (278 - 487)	527 (368 - 720)	567 (387 - 798)	678 (464 - 951)	0 (0 - 0)	0 (0 - 0)	0 (0 - 0)
**Libya**									
**Under-5 years (rate per capita)**	0.434 (0.300 - 0.546)	0.426 (0.295 - 0.536)	0.435 (0.301 - 0.548)	0.597 (0.361 - 0.798)	0.587 (0.354 - 0.784)	0.599 (0.362 - 0.801)	0.102 (0.042 - 0.207)	0.100 (0.042 - 0.204)	0.102 (0.044 - 0.206)
**5-14 years (rate per capita)**	0.245 (0.158 - 0.352)	0.237 (0.152 - 0.341)	0.252 (0.163 - 0.362)	0.533 (0.305 - 0.830)	0.517 (0.294 - 0.809)	0.549 (0.316 - 0.851)	0.057 (0.023 - 0.122)	0.056 (0.023 - 0.117)	0.059 (0.024 - 0.126)
**All ages (rate per capita)**	0.129 (0.099 - 0.168)	0.090 (0.069 - 0.117)	0.095 (0.073 - 0.124)	0.231 (0.161 - 0.317)	0.160 (0.111 - 0.219)	0.170 (0.118 - 0.233)	0.030 (0.013 - 0.062)	0.021 (0.009 - 0.043)	0.022 (0.009 - 0.045)
**Age-standardised (rate per capita)**	0.080 (0.062 - 0.105)	0.080 (0.062 - 0.105)	0.080 (0.062 - 0.105)	0.144 (0.100 - 0.198)	0.144 (0.100 - 0.198)	0.144 (0.100 - 0.198)	0.019 (0.008 - 0.039)	0.019 (0.008 - 0.039)	0.019 (0.008 - 0.038)
**Total number of cases (thousands)**	566 (433 - 738)	521 (399 - 679)	599 (458 - 779)	1,013 (704 - 1,391)	930 (646 - 1,274)	1,071 (745 - 1,468)	0 (0 - 0)	0 (0 - 0)	0 (0 - 0)
**Morocco**									
**Under-5 years (rate per capita)**	0.432 (0.299 - 0.543)	0.431 (0.298 - 0.542)	0.431 (0.298 - 0.543)	0.594 (0.359 - 0.794)	0.593 (0.358 - 0.792)	0.593 (0.358 - 0.793)	0.101 (0.043 - 0.203)	0.101 (0.043 - 0.205)	0.101 (0.043 - 0.199)
**5-14 years (rate per capita)**	0.252 (0.162 - 0.361)	0.232 (0.149 - 0.334)	0.248 (0.160 - 0.356)	0.547 (0.315 - 0.848)	0.506 (0.288 - 0.794)	0.540 (0.309 - 0.838)	0.059 (0.024 - 0.128)	0.054 (0.022 - 0.115)	0.058 (0.023 - 0.124)
**All ages (rate per capita)**	0.126 (0.096 - 0.163)	0.090 (0.069 - 0.118)	0.086 (0.065 - 0.111)	0.225 (0.156 - 0.308)	0.163 (0.113 - 0.226)	0.152 (0.106 - 0.208)	0.029 (0.013 - 0.060)	0.021 (0.009 - 0.043)	0.020 (0.009 - 0.041)
**Age-standardised (rate per capita)**	0.080 (0.062 - 0.105)	0.080 (0.062 - 0.105)	0.080 (0.062 - 0.105)	0.144 (0.100 - 0.198)	0.144 (0.100 - 0.198)	0.144 (0.100 - 0.198)	0.019 (0.008 - 0.038)	0.019 (0.008 - 0.039)	0.019 (0.008 - 0.038)
**Total number of cases (thousands)**	3,144 (2,407 - 4,093)	2,750 (2,103 - 3,608)	2,945 (2,251 - 3,815)	5,628 (3,915 - 7,718)	4,967 (3,442 - 6,870)	5,231 (3,658 - 7,138)	1 (0 - 2)	1 (0 - 1)	1 (0 - 1)
**Palestine**									
**Under-5 years (rate per capita)**	0.422 (0.292 - 0.531)	0.430 (0.298 - 0.541)	0.428 (0.296 - 0.538)	0.580 (0.351 - 0.776)	0.592 (0.358 - 0.791)	0.588 (0.355 - 0.786)	0.099 (0.042 - 0.199)	0.101 (0.043 - 0.204)	0.101 (0.042 - 0.203)
**5-14 years (rate per capita)**	0.257 (0.166 - 0.369)	0.249 (0.161 - 0.358)	0.251 (0.162 - 0.361)	0.559 (0.322 - 0.865)	0.542 (0.311 - 0.841)	0.546 (0.314 - 0.846)	0.060 (0.024 - 0.128)	0.059 (0.024 - 0.127)	0.059 (0.024 - 0.130)
**All ages (rate per capita)**	0.159 (0.121 - 0.204)	0.145 (0.111 - 0.188)	0.128 (0.098 - 0.165)	0.277 (0.198 - 0.374)	0.257 (0.180 - 0.351)	0.226 (0.159 - 0.308)	0.037 (0.016 - 0.077)	0.034 (0.015 - 0.069)	0.030 (0.013 - 0.061)
**Age-standardised (rate per capita)**	0.080 (0.062 - 0.105)	0.080 (0.062 - 0.105)	0.080 (0.062 - 0.105)	0.144 (0.100 - 0.198)	0.144 (0.100 - 0.198)	0.144 (0.100 - 0.198)	0.019 (0.008 - 0.039)	0.019 (0.008 - 0.038)	0.019 (0.008 - 0.039)
**Total number of cases (thousands)**	335 (256 - 431)	521 (398 - 675)	597 (456 - 773)	586 (418 - 789)	927 (647 - 1,266)	1,058 (743 - 1,440)	0 (0 - 0)	0 (0 - 0)	0 (0 - 0)
**Oman**									
**Under-5 years (rate per capita)**	0.383 (0.285 - 0.497)	0.383 (0.285 - 0.498)	0.376 (0.280 - 0.490)	0.581 (0.366 - 0.760)	0.569 (0.363 - 0.754)	0.560 (0.357 - 0.741)	0.090 (0.038 - 0.181)	0.090 (0.038 - 0.179)	0.088 (0.037 - 0.176)
**5-14 years (rate per capita)**	0.347 (0.290 - 0.422)	0.298 (0.238 - 0.367)	0.341 (0.276 - 0.417)	0.503 (0.383 - 0.679)	0.449 (0.330 - 0.632)	0.511 (0.379 - 0.705)	0.082 (0.034 - 0.167)	0.070 (0.029 - 0.144)	0.080 (0.034 - 0.164)
**All ages (rate per capita)**	0.165 (0.139 - 0.195)	0.110 (0.093 - 0.131)	0.072 (0.061 - 0.086)	0.244 (0.188 - 0.307)	0.166 (0.129 - 0.214)	0.108 (0.084 - 0.138)	0.039 (0.017 - 0.076)	0.026 (0.011 - 0.053)	0.017 (0.007 - 0.035)
**Age-standardised (rate per capita)**	0.089 (0.075 - 0.105)	0.087 (0.073 - 0.103)	0.087 (0.073 - 0.103)	0.131 (0.102 - 0.166)	0.130 (0.101 - 0.168)	0.130 (0.101 - 0.168)	0.021 (0.009 - 0.041)	0.020 (0.009 - 0.041)	0.020 (0.009 - 0.042)
**Total number of cases (thousands)**	301 (254 - 357)	276 (232 - 329)	324 (274 - 383)	445 (343 - 559)	415 (322 - 537)	484 (378 - 619)	0 (0 - 0)	0 (0 - 0)	0 (0 - 0)
**Qatar**									
**Under-5 years (rate per capita)**	0.432 (0.299 - 0.544)	0.422 (0.292 - 0.531)	0.423 (0.293 - 0.532)	0.594 (0.359 - 0.794)	0.581 (0.351 - 0.776)	0.582 (0.351 - 0.777)	0.101 (0.042 - 0.207)	0.099 (0.042 - 0.197)	0.099 (0.042 - 0.199)
**5-14 years (rate per capita)**	0.275 (0.179 - 0.393)	0.264 (0.171 - 0.378)	0.249 (0.161 - 0.358)	0.598 (0.347 - 0.922)	0.573 (0.332 - 0.886)	0.542 (0.311 - 0.841)	0.065 (0.026 - 0.140)	0.062 (0.024 - 0.131)	0.059 (0.023 - 0.126)
**All ages (rate per capita)**	0.095 (0.073 - 0.123)	0.072 (0.055 - 0.095)	0.046 (0.036 - 0.061)	0.169 (0.118 - 0.230)	0.131 (0.091 - 0.181)	0.083 (0.058 - 0.114)	0.022 (0.010 - 0.045)	0.017 (0.007 - 0.035)	0.011 (0.005 - 0.023)
**Age-standardised (rate per capita)**	0.080 (0.062 - 0.105)	0.080 (0.062 - 0.105)	0.080 (0.062 - 0.105)	0.144 (0.100 - 0.198)	0.144 (0.100 - 0.198)	0.144 (0.100 - 0.198)	0.019 (0.008 - 0.038)	0.019 (0.008 - 0.039)	0.019 (0.008 - 0.039)
**Total number of cases (thousands)**	45 (35 - 59)	60 (46 - 79)	103 (79 - 135)	81 (56 - 110)	109 (75 - 150)	185 (128 - 254)	0 (0 - 0)	0 (0 - 0)	0 (0 - 0)
**Saudi Arabia**									
**Under-5 years (rate per capita)**	0.307 (0.235 - 0.397)	0.326 (0.249 - 0.418)	0.343 (0.259 - 0.441)	0.461 (0.262 - 0.678)	0.463 (0.275 - 0.667)	0.493 (0.292 - 0.698)	0.072 (0.031 - 0.145)	0.077 (0.032 - 0.156)	0.081 (0.035 - 0.164)
**5-14 years (rate per capita)**	0.294 (0.237 - 0.362)	0.287 (0.227 - 0.355)	0.284 (0.222 - 0.355)	0.375 (0.251 - 0.542)	0.370 (0.241 - 0.542)	0.404 (0.269 - 0.584)	0.069 (0.029 - 0.142)	0.067 (0.029 - 0.137)	0.067 (0.028 - 0.136)
**All ages (rate per capita)**	0.125 (0.106 - 0.149)	0.101 (0.085 - 0.120)	0.086 (0.072 - 0.104)	0.171 (0.124 - 0.237)	0.134 (0.097 - 0.185)	0.123 (0.090 - 0.166)	0.029 (0.013 - 0.059)	0.024 (0.010 - 0.047)	0.020 (0.009 - 0.040)
**Age-standardised (rate per capita)**	0.073 (0.062 - 0.088)	0.078 (0.066 - 0.093)	0.078 (0.065 - 0.093)	0.100 (0.073 - 0.139)	0.105 (0.076 - 0.144)	0.111 (0.081 - 0.150)	0.017 (0.007 - 0.035)	0.018 (0.008 - 0.037)	0.018 (0.008 - 0.036)
**Total number of cases (thousands)**	2,045 (1,730 - 2,439)	2,492 (2,104 - 2,960)	2,713 (2,275 - 3,257)	2,794 (2,030 - 3,863)	3,330 (2,406 - 4,571)	3,871 (2,824 - 5,228)	0 (0 - 1)	1 (0 - 1)	1 (0 - 1)
**Sudan**									
**Under-5 years (rate per capita)**	0.221 (0.166 - 0.306)	0.273 (0.202 - 0.346)	0.275 (0.203 - 0.347)	0.325 (0.201 - 0.574)	0.529 (0.271 - 0.770)	0.531 (0.272 - 0.773)	0.051 (0.021 - 0.108)	0.064 (0.027 - 0.128)	0.064 (0.027 - 0.130)
**5-14 years (rate per capita)**	0.271 (0.217 - 0.336)	0.294 (0.228 - 0.367)	0.287 (0.222 - 0.359)	0.481 (0.340 - 0.675)	0.478 (0.353 - 0.655)	0.467 (0.345 - 0.640)	0.063 (0.027 - 0.131)	0.069 (0.028 - 0.140)	0.067 (0.028 - 0.137)
**All ages (rate per capita)**	0.114 (0.095 - 0.139)	0.124 (0.103 - 0.148)	0.115 (0.095 - 0.137)	0.191 (0.138 - 0.268)	0.216 (0.156 - 0.285)	0.200 (0.145 - 0.265)	0.027 (0.011 - 0.055)	0.029 (0.012 - 0.058)	0.027 (0.012 - 0.054)
**Age-standardised (rate per capita)**	0.065 (0.054 - 0.079)	0.073 (0.060 - 0.087)	0.073 (0.060 - 0.087)	0.109 (0.078 - 0.153)	0.126 (0.092 - 0.168)	0.126 (0.092 - 0.168)	0.015 (0.006 - 0.031)	0.017 (0.007 - 0.033)	0.017 (0.007 - 0.034)
**Total number of cases (thousands)**	2,288 (1,905 - 2,785)	3,976 (3,299 - 4,737)	4,643 (3,856 - 5,539)	3,822 (2,768 - 5,365)	6,927 (5,008 - 9,153)	8,075 (5,861 - 10,694)	1 (0 - 1)	1 (0 - 2)	1 (0 - 2)
**Syria**									
**Under-5 years (rate per capita)**	0.281 (0.195 - 0.370)	0.278 (0.232 - 0.338)	0.282 (0.236 - 0.344)	0.531 (0.267 - 0.777)	0.516 (0.430 - 0.618)	0.525 (0.437 - 0.628)	0.066 (0.027 - 0.131)	0.065 (0.028 - 0.126)	0.066 (0.028 - 0.131)
**5-14 years (rate per capita)**	0.290 (0.220 - 0.364)	0.279 (0.200 - 0.352)	0.268 (0.191 - 0.339)	0.470 (0.326 - 0.653)	0.446 (0.302 - 0.640)	0.428 (0.290 - 0.620)	0.068 (0.029 - 0.140)	0.066 (0.027 - 0.134)	0.063 (0.025 - 0.130)
**All ages (rate per capita)**	0.134 (0.107 - 0.162)	0.108 (0.086 - 0.127)	0.102 (0.081 - 0.121)	0.231 (0.167 - 0.308)	0.183 (0.146 - 0.232)	0.173 (0.138 - 0.220)	0.032 (0.013 - 0.063)	0.026 (0.011 - 0.051)	0.024 (0.010 - 0.048)
**Age-standardised (rate per capita)**	0.073 (0.058 - 0.088)	0.072 (0.057 - 0.084)	0.072 (0.057 - 0.084)	0.125 (0.091 - 0.167)	0.121 (0.097 - 0.153)	0.121 (0.097 - 0.153)	0.017 (0.007 - 0.034)	0.017 (0.007 - 0.034)	0.017 (0.007 - 0.034)
**Total number of cases (thousands)**	1,671 (1,335 - 2,016)	1,968 (1,569 - 2,309)	1,907 (1,515 - 2,245)	2,868 (2,076 - 3,837)	3,320 (2,658 - 4,214)	3,216 (2,566 - 4,101)	0 (0 - 1)	0 (0 - 1)	0 (0 - 1)
**Tunisia**									
**Under-5 years (rate per capita)**	0.434 (0.300 - 0.546)	0.431 (0.298 - 0.542)	0.430 (0.298 - 0.541)	0.597 (0.361 - 0.798)	0.593 (0.358 - 0.793)	0.592 (0.357 - 0.791)	0.102 (0.042 - 0.207)	0.101 (0.042 - 0.207)	0.101 (0.043 - 0.206)
**5-14 years (rate per capita)**	0.248 (0.160 - 0.357)	0.226 (0.145 - 0.327)	0.248 (0.160 - 0.356)	0.540 (0.310 - 0.839)	0.494 (0.280 - 0.773)	0.540 (0.310 - 0.838)	0.058 (0.023 - 0.128)	0.053 (0.021 - 0.118)	0.058 (0.023 - 0.127)
**All ages (rate per capita)**	0.116 (0.089 - 0.152)	0.074 (0.057 - 0.098)	0.074 (0.056 - 0.095)	0.208 (0.145 - 0.286)	0.134 (0.093 - 0.186)	0.130 (0.092 - 0.178)	0.027 (0.012 - 0.056)	0.017 (0.007 - 0.036)	0.017 (0.007 - 0.036)
**Age-standardised (rate per capita)**	0.080 (0.062 - 0.105)	0.080 (0.062 - 0.105)	0.080 (0.062 - 0.105)	0.144 (0.100 - 0.198)	0.144 (0.100 - 0.198)	0.144 (0.100 - 0.198)	0.019 (0.008 - 0.039)	0.019 (0.008 - 0.039)	0.019 (0.008 - 0.039)
**Total number of cases (thousands)**	958 (734 - 1,248)	752 (574 - 988)	829 (632 - 1,072)	1,716 (1,193 - 2,354)	1,360 (941 - 1,887)	1,467 (1,030 - 1,997)	0 (0 - 0)	0 (0 - 0)	0 (0 - 0)
**Turkey**									
**Under-5 years (rate per capita)**	0.320 (0.243 - 0.411)	0.309 (0.239 - 0.399)	0.304 (0.231 - 0.397)	0.550 (0.309 - 0.770)	0.519 (0.318 - 0.738)	0.491 (0.284 - 0.724)	0.075 (0.032 - 0.149)	0.073 (0.030 - 0.146)	0.071 (0.031 - 0.146)
**5-14 years (rate per capita)**	0.300 (0.242 - 0.370)	0.292 (0.231 - 0.354)	0.281 (0.219 - 0.347)	0.461 (0.345 - 0.635)	0.457 (0.332 - 0.645)	0.448 (0.327 - 0.632)	0.070 (0.029 - 0.147)	0.069 (0.029 - 0.140)	0.066 (0.027 - 0.135)
**All ages (rate per capita)**	0.110 (0.093 - 0.131)	0.085 (0.072 - 0.099)	0.073 (0.062 - 0.086)	0.177 (0.135 - 0.230)	0.136 (0.104 - 0.182)	0.116 (0.089 - 0.155)	0.026 (0.011 - 0.052)	0.020 (0.009 - 0.040)	0.017 (0.007 - 0.035)

	Prevalence 1990	Prevalence 2005	Prevalence 2015	Incidence 1990	Incidence 2005	Incidence 2015	YLD rate 1990	YLD rate 2005	YLD rate 2015
Age-standardised (rate per capita)	0.079 (0.067 - 0.094)	0.078 (0.066 - 0.091)	0.076 (0.065 - 0.090)	0.127 (0.097 - 0.166)	0.125 (0.095 - 0.166)	0.122 (0.093 - 0.162)	0.019 (0.008 - 0.038)	0.018 (0.008 - 0.036)	0.018 (0.008 - 0.036)
Total number of cases (thousands)	6,037 (5,059 - 7,177)	5,782 (4,932 - 6,764)	5,692 (4,839 - 6,772)	9,686 (7,368 - 12,591)	9,287 (7,115 - 12,410)	9,128 (6,968 - 12,166)	1 (1 - 3)	1 (1 - 3)	1 (1 - 3)
**United Arab Emirates**									
Under-5 years (rate per capita)	0.304 (0.241 - 0.376)	0.282 (0.212 - 0.370)	0.265 (0.193 - 0.368)	0.549 (0.336 - 0.764)	0.459 (0.262 - 0.705)	0.376 (0.222 - 0.685)	0.071 (0.030 - 0.140)	0.066 (0.028 - 0.132)	0.062 (0.026 - 0.130)
5-14 years (rate per capita)	0.325 (0.260 - 0.395)	0.265 (0.205 - 0.326)	0.259 (0.198 - 0.332)	0.510 (0.378 - 0.706)	0.442 (0.309 - 0.628)	0.485 (0.328 - 0.699)	0.076 (0.033 - 0.154)	0.062 (0.026 - 0.128)	0.061 (0.026 - 0.130)
All ages (rate per capita)	0.096 (0.082 - 0.112)	0.049 (0.041 - 0.058)	0.036 (0.029 - 0.044)	0.159 (0.121 - 0.208)	0.082 (0.060 - 0.112)	0.061 (0.044 - 0.089)	0.023 (0.010 - 0.045)	0.012 (0.005 - 0.023)	0.009 (0.004 - 0.018)
Age-standardised (rate per capita)	0.078 (0.066 - 0.091)	0.072 (0.061 - 0.086)	0.065 (0.053 - 0.080)	0.129 (0.098 - 0.170)	0.120 (0.087 - 0.164)	0.111 (0.079 - 0.163)	0.018 (0.008 - 0.036)	0.017 (0.007 - 0.034)	0.015 (0.007 - 0.032)
Total number of cases (thousands)	173 (148 - 202)	222 (186 - 262)	331 (269 - 405)	288 (219 - 375)	367 (267 - 503)	561 (401 - 813)	0 (0 - 0)	0 (0 - 0)	0 (0 - 0)
**Yemen**									
Under-5 years (rate per capita)	0.418 (0.290 - 0.526)	0.426 (0.295 - 0.536)	0.429 (0.297 - 0.539)	0.576 (0.348 - 0.769)	0.586 (0.354 - 0.784)	0.590 (0.356 - 0.788)	0.098 (0.042 - 0.193)	0.099 (0.043 - 0.201)	0.100 (0.042 - 0.200)
5-14 years (rate per capita)	0.265 (0.172 - 0.380)	0.243 (0.156 - 0.349)	0.250 (0.162 - 0.360)	0.576 (0.333 - 0.890)	0.529 (0.303 - 0.826)	0.545 (0.313 - 0.844)	0.062 (0.025 - 0.132)	0.057 (0.023 - 0.120)	0.059 (0.024 - 0.125)
All ages (rate per capita)	0.170 (0.130 - 0.219)	0.142 (0.109 - 0.184)	0.128 (0.098 - 0.165)	0.298 (0.213 - 0.402)	0.253 (0.176 - 0.345)	0.227 (0.159 - 0.310)	0.040 (0.017 - 0.080)	0.033 (0.014 - 0.069)	0.030 (0.013 - 0.060)
Age-standardised (rate per capita)	0.080 (0.062 - 0.105)	0.080 (0.062 - 0.105)	0.080 (0.062 - 0.105)	0.144 (0.100 - 0.198)	0.144 (0.100 - 0.198)	0.144 (0.100 - 0.198)	0.019 (0.008 - 0.038)	0.019 (0.008 - 0.039)	0.019 (0.008 - 0.038)
Total number of cases (thousands)	2,039 (1,558 - 2,626)	2,920 (2,234 - 3,790)	3,439 (2,628 - 4,454)	3,568 (2,545 - 4,808)	5,195 (3,625 - 7,099)	6,110 (4,273 - 8,336)	0 (0 - 1)	1 (0 - 1)	1 (0 - 2)
**South Asia**									
Under-5 years (rate per capita)	0.463 (0.364 - 0.551)	0.458 (0.363 - 0.541)	0.455 (0.361 - 0.534)	0.587 (0.403 - 0.748)	0.557 (0.392 - 0.701)	0.554 (0.394 - 0.695)	0.108 (0.045 - 0.210)	0.107 (0.046 - 0.208)	0.106 (0.045 - 0.207)
5-14 years (rate per capita)	0.245 (0.179 - 0.318)	0.231 (0.165 - 0.301)	0.219 (0.159 - 0.291)	0.543 (0.353 - 0.782)	0.516 (0.327 - 0.757)	0.489 (0.293 - 0.732)	0.057 (0.023 - 0.121)	0.054 (0.022 - 0.112)	0.051 (0.021 - 0.108)
All ages (rate per capita)	0.127 (0.104 - 0.153)	0.104 (0.085 - 0.126)	0.088 (0.073 - 0.106)	0.218 (0.162 - 0.284)	0.178 (0.135 - 0.233)	0.151 (0.111 - 0.203)	0.030 (0.013 - 0.059)	0.024 (0.010 - 0.049)	0.021 (0.009 - 0.042)
Age-standardised (rate per capita)	0.082 (0.067 - 0.100)	0.081 (0.066 - 0.097)	0.079 (0.065 - 0.094)	0.143 (0.106 - 0.186)	0.138 (0.105 - 0.181)	0.134 (0.099 - 0.180)	0.019 (0.008 - 0.039)	0.019 (0.008 - 0.038)	0.018 (0.008 - 0.037)
Total number of cases (thousands)	140,138 (114,562 - 168,989)	152,556 (125,061 - 184,214)	149,221 (123,253 - 179,367)	240,448 (179,086 - 313,300)	261,870 (198,230 - 342,079)	256,118 (187,948 - 343,546)	33 (14 - 65)	36 (15 - 72)	35 (15 - 71)
**South Asia**									
Under-5 years (rate per capita)	0.463 (0.364 - 0.551)	0.458 (0.363 - 0.541)	0.455 (0.361 - 0.534)	0.587 (0.403 - 0.748)	0.557 (0.392 - 0.701)	0.554 (0.394 - 0.695)	0.108 (0.045 - 0.210)	0.107 (0.046 - 0.208)	0.106 (0.045 - 0.207)
5-14 years (rate per capita)	0.245 (0.179 - 0.318)	0.231 (0.165 - 0.301)	0.219 (0.159 - 0.291)	0.543 (0.353 - 0.782)	0.516 (0.327 - 0.757)	0.489 (0.293 - 0.732)	0.057 (0.023 - 0.121)	0.054 (0.022 - 0.112)	0.051 (0.021 - 0.108)
All ages (rate per capita)	0.127 (0.104 - 0.153)	0.104 (0.085 - 0.126)	0.088 (0.073 - 0.106)	0.218 (0.162 - 0.284)	0.178 (0.135 - 0.233)	0.151 (0.111 - 0.203)	0.030 (0.013 - 0.059)	0.024 (0.010 - 0.049)	0.021 (0.009 - 0.042)
Age-standardised (rate per capita)	0.082 (0.067 - 0.100)	0.081 (0.066 - 0.097)	0.079 (0.065 - 0.094)	0.143 (0.106 - 0.186)	0.138 (0.105 - 0.181)	0.134 (0.099 - 0.180)	0.019 (0.008 - 0.039)	0.019 (0.008 - 0.038)	0.018 (0.008 - 0.037)
Total number of cases (thousands)	140,138 (114,562 - 168,989)	152,556 (125,061 - 184,214)	149,221 (123,253 - 179,367)	240,448 (179,086 - 313,300)	261,870 (198,230 - 342,079)	256,118 (187,948 - 343,546)	33 (14 - 65)	36 (15 - 72)	35 (15 - 71)
**Bangladesh**									
Under-5 years (rate per capita)	0.475 (0.367 - 0.574)	0.482 (0.372 - 0.582)	0.482 (0.372 - 0.581)	0.570 (0.399 - 0.730)	0.578 (0.405 - 0.740)	0.577 (0.405 - 0.740)	0.111 (0.047 - 0.222)	0.113 (0.048 - 0.220)	0.113 (0.047 - 0.228)
5-14 years (rate per capita)	0.217 (0.130 - 0.321)	0.209 (0.125 - 0.309)	0.203 (0.120 - 0.300)	0.575 (0.296 - 0.878)	0.555 (0.285 - 0.851)	0.540 (0.276 - 0.829)	0.051 (0.020 - 0.109)	0.049 (0.020 - 0.106)	0.048 (0.018 - 0.103)
All ages (rate per capita)	0.132 (0.103 - 0.170)	0.104 (0.081 - 0.134)	0.086 (0.067 - 0.112)	0.243 (0.169 - 0.329)	0.194 (0.133 - 0.264)	0.162 (0.111 - 0.223)	0.031 (0.013 - 0.064)	0.024 (0.010 - 0.050)	0.020 (0.009 - 0.042)
Age-standardised (rate per capita)	0.079 (0.062 - 0.102)	0.079 (0.062 - 0.102)	0.079 (0.062 - 0.102)	0.147 (0.101 - 0.200)	0.147 (0.101 - 0.200)	0.147 (0.101 - 0.200)	0.018 (0.008 - 0.038)	0.018 (0.008 - 0.038)	0.019 (0.008 - 0.038)
Total number of cases (thousands)	13,989 (10,918 - 18,042)	14,818 (11,542 - 19,196)	13,885 (10,775 - 18,049)	25,709 (17,914 - 34,913)	27,678 (19,015 - 37,749)	26,139 (17,841 - 35,875)	3 (1 - 7)	3 (1 - 7)	3 (1 - 7)
**Bhutan**									
Under-5 years (rate per capita)	0.478 (0.368 - 0.577)	0.480 (0.371 - 0.580)	0.483 (0.373 - 0.583)	0.573 (0.402 - 0.734)	0.576 (0.404 - 0.738)	0.579 (0.406 - 0.741)	0.112 (0.048 - 0.226)	0.113 (0.047 - 0.222)	0.113 (0.048 - 0.226)
5-14 years (rate per capita)	0.219 (0.131 - 0.323)	0.204 (0.121 - 0.302)	0.208 (0.124 - 0.307)	0.579 (0.299 - 0.884)	0.543 (0.278 - 0.834)	0.552 (0.283 - 0.847)	0.051 (0.020 - 0.113)	0.048 (0.018 - 0.106)	0.049 (0.019 - 0.106)
All ages (rate per capita)	0.138 (0.108 - 0.178)	0.100 (0.078 - 0.130)	0.079 (0.061 - 0.103)	0.250 (0.176 - 0.339)	0.189 (0.129 - 0.259)	0.151 (0.102 - 0.207)	0.032 (0.014 - 0.066)	0.023 (0.010 - 0.048)	0.019 (0.008 - 0.039)
Age-standardised (rate per capita)	0.079 (0.062 - 0.102)	0.079 (0.062 - 0.102)	0.079 (0.062 - 0.102)	0.147 (0.101 - 0.200)	0.147 (0.101 - 0.200)	0.147 (0.101 - 0.200)	0.019 (0.008 - 0.038)	0.019 (0.008 - 0.038)	0.019 (0.008 - 0.038)
Total number of cases (thousands)	74 (58 - 95)	65 (51 - 85)	62 (48 - 80)	134 (94 - 181)	123 (84 - 169)	117 (79 - 160)	0 (0 - 0)	0 (0 - 0)	0 (0 - 0)
**India**									
Under-5 years (rate per capita)	0.470 (0.366 - 0.557)	0.476 (0.372 - 0.561)	0.478 (0.375 - 0.561)	0.597 (0.409 - 0.756)	0.595 (0.411 - 0.757)	0.601 (0.417 - 0.764)	0.110 (0.046 - 0.214)	0.111 (0.047 - 0.218)	0.112 (0.047 - 0.216)
5-14 years (rate per capita)	0.252 (0.186 - 0.323)	0.246 (0.179 - 0.318)	0.232 (0.169 - 0.303)	0.538 (0.359 - 0.771)	0.500 (0.324 - 0.732)	0.465 (0.286 - 0.696)	0.059 (0.024 - 0.124)	0.057 (0.023 - 0.119)	0.054 (0.022 - 0.114)
All ages (rate per capita)	0.126 (0.103 - 0.152)	0.107 (0.087 - 0.128)	0.090 (0.075 - 0.108)	0.212 (0.159 - 0.274)	0.175 (0.132 - 0.227)	0.147 (0.109 - 0.196)	0.029 (0.013 - 0.059)	0.025 (0.011 - 0.050)	0.021 (0.009 - 0.043)
Age-standardised (rate per capita)	0.084 (0.069 - 0.102)	0.085 (0.069 - 0.102)	0.083 (0.069 - 0.099)	0.143 (0.107 - 0.185)	0.139 (0.105 - 0.180)	0.134 (0.100 - 0.179)	0.020 (0.008 - 0.040)	0.020 (0.008 - 0.040)	0.019 (0.008 - 0.039)
Total number of cases (thousands)	109,696 (89,788 - 132,154)	121,906 (99,849 - 146,410)	118,100 (98,083 - 141,235)	184,796 (138,569 - 238,482)	199,728 (151,505 - 259,420)	192,552 (142,767 - 257,588)	26 (11 - 52)	28 (12 - 58)	28 (12 - 56)
**Nepal**									
Under-5 years (rate per capita)	0.505 (0.430 - 0.568)	0.520 (0.445 - 0.581)	0.518 (0.441 - 0.583)	0.541 (0.395 - 0.670)	0.567 (0.431 - 0.704)	0.556 (0.406 - 0.687)	0.118 (0.051 - 0.227)	0.122 (0.053 - 0.235)	0.122 (0.053 - 0.232)
5-14 years (rate per capita)	0.252 (0.192 - 0.319)	0.255 (0.205 - 0.311)	0.230 (0.174 - 0.293)	0.544 (0.366 - 0.772)	0.545 (0.372 - 0.763)	0.501 (0.332 - 0.720)	0.059 (0.025 - 0.122)	0.060 (0.026 - 0.123)	0.054 (0.022 - 0.112)
All ages (rate per capita)	0.148 (0.128 - 0.169)	0.137 (0.121 - 0.155)	0.104 (0.089 - 0.120)	0.230 (0.175 - 0.297)	0.220 (0.165 - 0.283)	0.169 (0.126 - 0.223)	0.035 (0.015 - 0.068)	0.032 (0.014 - 0.063)	0.024 (0.011 - 0.049)
Age-standardised (rate per capita)	0.087 (0.075 - 0.100)	0.089 (0.078 - 0.101)	0.087 (0.075 - 0.100)	0.139 (0.105 - 0.181)	0.142 (0.106 - 0.182)	0.139 (0.105 - 0.181)	0.020 (0.009 - 0.040)	0.021 (0.009 - 0.041)	0.021 (0.009 - 0.041)
Total number of cases (thousands)	2,774 (2,402 - 3,172)	3,499 (3,078 - 3,951)	2,969 (2,548 - 3,435)	4,317 (3,283 - 5,572)	5,613 (4,208 - 7,225)	4,838 (3,599 - 6,370)	1 (0 - 1)	1 (0 - 2)	1 (0 - 1)
**Pakistan**									
Under-5 years (rate per capita)	0.398 (0.311 - 0.522)	0.316 (0.256 - 0.395)	0.316 (0.256 - 0.395)	0.544 (0.331 - 0.726)	0.305 (0.238 - 0.387)	0.305 (0.238 - 0.387)	0.093 (0.039 - 0.186)	0.074 (0.030 - 0.144)	0.074 (0.031 - 0.147)
5-14 years (rate per capita)	0.221 (0.160 - 0.285)	0.149 (0.095 - 0.209)	0.153 (0.098 - 0.216)	0.549 (0.341 - 0.815)	0.582 (0.346 - 0.895)	0.601 (0.358 - 0.924)	0.052 (0.022 - 0.108)	0.035 (0.014 - 0.077)	0.036 (0.015 - 0.078)
All ages (rate per capita)	0.126 (0.104 - 0.156)	0.080 (0.063 - 0.099)	0.075 (0.060 - 0.093)	0.236 (0.170 - 0.314)	0.187 (0.125 - 0.267)	0.172 (0.116 - 0.243)	0.029 (0.012 - 0.059)	0.019 (0.008 - 0.039)	0.018 (0.007 - 0.036)
Age-standardised (rate per capita)	0.072 (0.059 - 0.089)	0.054 (0.043 - 0.067)	0.054 (0.043 - 0.067)	0.139 (0.099 - 0.187)	0.127 (0.085 - 0.181)	0.127 (0.085 - 0.181)	0.017 (0.007 - 0.034)	0.013 (0.005 - 0.026)	0.013 (0.005 - 0.026)
Total number of cases (thousands)	13,606 (11,179 - 16,844)	12,266 (9,682 - 15,217)	14,206 (11,345 - 17,582)	25,492 (18,354 - 33,902)	28,727 (19,219 - 41,048)	32,473 (21,919 - 45,876)	3 (1 - 6)	3 (1 - 6)	3 (1 - 7)
**Sub-Saharan Africa**									
Under-5 years (rate per capita)	0.351 (0.252 - 0.461)	0.349 (0.251 - 0.456)	0.356 (0.256 - 0.466)	0.510 (0.304 - 0.699)	0.491 (0.301 - 0.661)	0.513 (0.307 - 0.700)	0.082 (0.034 - 0.165)	0.081 (0.035 - 0.164)	0.083 (0.035 - 0.166)
5-14 years (rate per capita)	0.269 (0.198 - 0.352)	0.263 (0.191 - 0.349)	0.268 (0.196 - 0.354)	0.462 (0.298 - 0.687)	0.454 (0.290 - 0.683)	0.459 (0.293 - 0.684)	0.063 (0.027 - 0.129)	0.062 (0.026 - 0.130)	0.063 (0.026 - 0.132)
All ages (rate per capita)	0.137 (0.110 - 0.171)	0.131 (0.105 - 0.164)	0.130 (0.104 - 0.162)	0.219 (0.157 - 0.299)	0.206 (0.148 - 0.284)	0.206 (0.147 - 0.285)	0.032 (0.014 - 0.065)	0.031 (0.013 - 0.062)	0.030 (0.013 - 0.062)
Age-standardised (rate per capita)	0.076 (0.061 - 0.095)	0.075 (0.060 - 0.094)	0.076 (0.061 - 0.096)	0.122 (0.087 - 0.169)	0.119 (0.086 - 0.166)	0.122 (0.087 - 0.170)	0.018 (0.008 - 0.036)	0.017 (0.008 - 0.036)	0.018 (0.008 - 0.037)
Total number of cases (thousands)	67,372 (54,270 - 83,879)	95,778 (77,157 - 120,055)	124,575 (100,178 - 155,940)	107,486 (76,917 - 146,912)	151,131 (108,617 - 207,868)	198,116 (141,063 - 273,976)	16 (7 - 32)	22 (10 - 45)	29 (13 - 60)
**Southern Sub-Saharan Africa**									
Under-5 years (rate per capita)	0.281 (0.216 - 0.359)	0.300 (0.230 - 0.376)	0.300 (0.231 - 0.378)	0.497 (0.284 - 0.711)	0.541 (0.304 - 0.769)	0.543 (0.306 - 0.771)	0.066 (0.028 - 0.133)	0.070 (0.030 - 0.140)	0.070 (0.030 - 0.138)
5-14 years (rate per capita)	0.207 (0.155 - 0.272)	0.215 (0.160 - 0.281)	0.222 (0.166 - 0.287)	0.413 (0.275 - 0.597)	0.419 (0.286 - 0.606)	0.437 (0.294 - 0.634)	0.048 (0.020 - 0.106)	0.050 (0.020 - 0.106)	0.052 (0.021 - 0.111)
All ages (rate per capita)	0.094 (0.077 - 0.114)	0.083 (0.067 - 0.100)	0.077 (0.063 - 0.093)	0.179 (0.129 - 0.248)	0.157 (0.113 - 0.217)	0.147 (0.106 - 0.203)	0.022 (0.009 - 0.045)	0.019 (0.008 - 0.039)	0.018 (0.008 - 0.037)
Age-standardised (rate per capita)	0.061 (0.050 - 0.073)	0.064 (0.052 - 0.078)	0.064 (0.052 - 0.077)	0.115 (0.083 - 0.159)	0.122 (0.088 - 0.168)	0.122 (0.088 - 0.168)	0.014 (0.006 - 0.029)	0.015 (0.006 - 0.030)	0.015 (0.006 - 0.030)
Total number of cases (thousands)	5,032 (4,127 - 6,097)	5,678 (4,604 - 6,867)	5,988 (4,866 - 7,190)	9,538 (6,873 - 13,221)	10,728 (7,765 - 14,822)	11,368 (8,177 - 15,688)	1 (1 - 2)	1 (1 - 3)	1 (1 - 3)
**Botswana**									
Under-5 years (rate per capita)	0.363 (0.258 - 0.491)	0.361 (0.257 - 0.489)	0.361 (0.257 - 0.489)	0.565 (0.329 - 0.766)	0.563 (0.328 - 0.763)	0.563 (0.328 - 0.763)	0.085 (0.035 - 0.175)	0.084 (0.035 - 0.174)	0.084 (0.035 - 0.173)
5-14 years (rate per capita)	0.237 (0.171 - 0.320)	0.223 (0.160 - 0.302)	0.230 (0.165 - 0.310)	0.465 (0.291 - 0.684)	0.438 (0.273 - 0.648)	0.451 (0.281 - 0.661)	0.056 (0.023 - 0.121)	0.052 (0.021 - 0.111)	0.054 (0.022 - 0.113)

	Prevalence 1990	Prevalence 2005	Prevalence 2015	Incidence 1990	Incidence 2005	Incidence 2015	YLD rate 1990	YLD rate 2005	YLD rate 2015
All ages (rate per capita)	0.127 (0.099 - 0.157)	0.096 (0.075 - 0.120)	0.089 (0.069 - 0.109)	0.225 (0.159 - 0.304)	0.171 (0.121 - 0.232)	0.157 (0.111 - 0.212)	0.030 (0.013 - 0.061)	0.022 (0.010 - 0.047)	0.021 (0.009 - 0.043)
Age-standardised (rate per capita)	0.071 (0.056 - 0.089)	0.071 (0.056 - 0.089)	0.071 (0.056 - 0.089)	0.127 (0.090 - 0.172)	0.127 (0.090 - 0.172)	0.127 (0.090 - 0.172)	0.017 (0.007 - 0.034)	0.017 (0.007 - 0.035)	0.017 (0.007 - 0.035)
Total number of cases (thousands)	176 (138 - 218)	180 (140 - 223)	200 (156 - 247)	312 (221 - 421)	319 (227 - 433)	354 (251 - 479)	0 (0 - 0)	0 (0 - 0)	0 (0 - 0)
**Lesotho**									
Under-5 years (rate per capita)	0.361 (0.257 - 0.489)	0.362 (0.257 - 0.490)	0.359 (0.255 - 0.486)	0.563 (0.328 - 0.763)	0.564 (0.328 - 0.764)	0.559 (0.326 - 0.758)	0.084 (0.036 - 0.176)	0.085 (0.035 - 0.175)	0.084 (0.036 - 0.171)
5-14 years (rate per capita)	0.235 (0.169 - 0.317)	0.228 (0.164 - 0.308)	0.225 (0.162 - 0.305)	0.462 (0.289 - 0.678)	0.448 (0.279 - 0.658)	0.443 (0.276 - 0.653)	0.055 (0.022 - 0.119)	0.053 (0.022 - 0.113)	0.053 (0.021 - 0.113)
All ages (rate per capita)	0.124 (0.097 - 0.153)	0.108 (0.085 - 0.135)	0.098 (0.077 - 0.121)	0.220 (0.156 - 0.297)	0.193 (0.137 - 0.262)	0.174 (0.123 - 0.236)	0.029 (0.012 - 0.059)	0.025 (0.011 - 0.051)	0.023 (0.010 - 0.048)
Age-standardised (rate per capita)	0.071 (0.056 - 0.089)	0.071 (0.056 - 0.089)	0.071 (0.056 - 0.089)	0.127 (0.090 - 0.172)	0.127 (0.090 - 0.172)	0.127 (0.090 - 0.172)	0.017 (0.007 - 0.034)	0.017 (0.007 - 0.034)	0.017 (0.007 - 0.035)
Total number of cases (thousands)	199 (156 - 246)	208 (163 - 260)	209 (163 - 259)	353 (249 - 476)	372 (264 - 505)	370 (261 - 502)	0 (0 - 0)	0 (0 - 0)	0 (0 - 0)
**Namibia**									
Under-5 years (rate per capita)	0.357 (0.254 - 0.484)	0.361 (0.257 - 0.489)	0.360 (0.255 - 0.487)	0.556 (0.324 - 0.755)	0.563 (0.328 - 0.763)	0.560 (0.326 - 0.759)	0.083 (0.035 - 0.172)	0.084 (0.035 - 0.175)	0.084 (0.035 - 0.171)
5-14 years (rate per capita)	0.233 (0.167 - 0.314)	0.227 (0.163 - 0.307)	0.230 (0.165 - 0.311)	0.456 (0.285 - 0.670)	0.446 (0.278 - 0.656)	0.451 (0.282 - 0.662)	0.054 (0.022 - 0.115)	0.053 (0.021 - 0.113)	0.054 (0.022 - 0.115)
All ages (rate per capita)	0.122 (0.096 - 0.151)	0.109 (0.085 - 0.136)	0.102 (0.079 - 0.125)	0.216 (0.153 - 0.291)	0.194 (0.137 - 0.263)	0.180 (0.127 - 0.242)	0.029 (0.012 - 0.058)	0.025 (0.011 - 0.052)	0.024 (0.010 - 0.048)
Age-standardised (rate per capita)	0.071 (0.056 - 0.089)	0.071 (0.056 - 0.089)	0.071 (0.056 - 0.089)	0.127 (0.090 - 0.172)	0.127 (0.090 - 0.172)	0.127 (0.090 - 0.172)	0.017 (0.007 - 0.034)	0.017 (0.007 - 0.034)	0.017 (0.007 - 0.034)
Total number of cases (thousands)	174 (135 - 214)	221 (173 - 275)	249 (195 - 308)	306 (217 - 412)	394 (279 - 533)	441 (312 - 595)	0 (0 - 0)	0 (0 - 0)	0 (0 - 0)
**South Africa**									
Under-5 years (rate per capita)	0.290 (0.223 - 0.359)	0.305 (0.235 - 0.384)	0.307 (0.238 - 0.385)	0.546 (0.303 - 0.777)	0.545 (0.310 - 0.770)	0.549 (0.314 - 0.769)	0.068 (0.029 - 0.134)	0.071 (0.031 - 0.142)	0.072 (0.031 - 0.142)
5-14 years (rate per capita)	0.216 (0.163 - 0.281)	0.215 (0.162 - 0.281)	0.221 (0.168 - 0.283)	0.418 (0.285 - 0.603)	0.420 (0.292 - 0.602)	0.438 (0.301 - 0.631)	0.051 (0.021 - 0.106)	0.050 (0.020 - 0.105)	0.052 (0.022 - 0.107)
All ages (rate per capita)	0.092 (0.076 - 0.111)	0.076 (0.062 - 0.093)	0.068 (0.056 - 0.082)	0.177 (0.126 - 0.243)	0.144 (0.105 - 0.197)	0.130 (0.094 - 0.176)	0.022 (0.009 - 0.043)	0.018 (0.008 - 0.036)	0.016 (0.007 - 0.032)
Age-standardised (rate per capita)	0.064 (0.052 - 0.077)	0.065 (0.053 - 0.079)	0.065 (0.053 - 0.078)	0.122 (0.087 - 0.168)	0.123 (0.089 - 0.168)	0.123 (0.089 - 0.168)	0.015 (0.006 - 0.030)	0.015 (0.007 - 0.031)	0.015 (0.007 - 0.030)
Total number of cases (thousands)	3,465 (2,845 - 4,179)	3,704 (3,009 - 4,488)	3,674 (3,001 - 4,399)	6,637 (4,720 - 9,152)	6,978 (5,087 - 9,567)	6,961 (5,027 - 9,472)	1 (0 - 2)	1 (0 - 2)	1 (0 - 2)
**Swaziland**									
Under-5 years (rate per capita)	0.376 (0.290 - 0.477)	0.376 (0.289 - 0.476)	0.378 (0.291 - 0.479)	0.566 (0.367 - 0.753)	0.565 (0.367 - 0.752)	0.568 (0.369 - 0.756)	0.088 (0.037 - 0.180)	0.088 (0.037 - 0.177)	0.089 (0.038 - 0.177)
5-14 years (rate per capita)	0.273 (0.211 - 0.341)	0.247 (0.190 - 0.310)	0.259 (0.199 - 0.324)	0.461 (0.320 - 0.653)	0.419 (0.290 - 0.603)	0.438 (0.304 - 0.626)	0.064 (0.027 - 0.133)	0.058 (0.024 - 0.121)	0.060 (0.025 - 0.124)
All ages (rate per capita)	0.151 (0.126 - 0.179)	0.122 (0.101 - 0.144)	0.113 (0.093 - 0.133)	0.242 (0.183 - 0.314)	0.196 (0.146 - 0.258)	0.182 (0.136 - 0.237)	0.035 (0.015 - 0.071)	0.028 (0.012 - 0.057)	0.026 (0.011 - 0.054)
Age-standardised (rate per capita)	0.078 (0.064 - 0.092)	0.078 (0.064 - 0.092)	0.078 (0.064 - 0.092)	0.125 (0.093 - 0.164)	0.125 (0.093 - 0.164)	0.125 (0.093 - 0.164)	0.018 (0.008 - 0.037)	0.018 (0.008 - 0.036)	0.018 (0.008 - 0.037)
Total number of cases (thousands)	131 (109 - 154)	135 (111 - 159)	146 (120 - 172)	209 (158 - 271)	217 (162 - 285)	234 (175 - 306)	0 (0 - 0)	0 (0 - 0)	0 (0 - 0)
**Zimbabwe**									
Under-5 years (rate per capita)	0.215 (0.161 - 0.294)	0.256 (0.192 - 0.314)	0.258 (0.193 - 0.316)	0.326 (0.190 - 0.622)	0.519 (0.266 - 0.773)	0.522 (0.268 - 0.778)	0.050 (0.021 - 0.104)	0.060 (0.025 - 0.119)	0.060 (0.026 - 0.120)
5-14 years (rate per capita)	0.164 (0.110 - 0.228)	0.206 (0.146 - 0.275)	0.219 (0.156 - 0.290)	0.372 (0.215 - 0.582)	0.405 (0.263 - 0.615)	0.430 (0.281 - 0.642)	0.038 (0.016 - 0.086)	0.048 (0.019 - 0.104)	0.051 (0.021 - 0.115)
All ages (rate per capita)	0.084 (0.067 - 0.108)	0.094 (0.075 - 0.115)	0.097 (0.078 - 0.118)	0.164 (0.106 - 0.251)	0.188 (0.127 - 0.270)	0.193 (0.131 - 0.276)	0.020 (0.008 - 0.042)	0.022 (0.009 - 0.045)	0.023 (0.010 - 0.047)
Age-standardised (rate per capita)	0.046 (0.036 - 0.059)	0.059 (0.047 - 0.072)	0.059 (0.047 - 0.072)	0.090 (0.058 - 0.138)	0.117 (0.080 - 0.168)	0.117 (0.080 - 0.168)	0.011 (0.005 - 0.023)	0.014 (0.006 - 0.028)	0.014 (0.006 - 0.028)
Total number of cases (thousands)	887 (702 - 1,132)	1,229 (982 - 1,497)	1,510 (1,207 - 1,833)	1,720 (1,115 - 2,639)	2,448 (1,661 - 3,515)	3,007 (2,036 - 4,300)	0 (0 - 0)	0 (0 - 1)	0 (0 - 1)
**Western Sub-Saharan Africa**									
Under-5 years (rate per capita)	0.355 (0.238 - 0.486)	0.358 (0.241 - 0.489)	0.363 (0.244 - 0.495)	0.496 (0.287 - 0.690)	0.498 (0.288 - 0.693)	0.503 (0.292 - 0.700)	0.082 (0.035 - 0.175)	0.083 (0.035 - 0.174)	0.084 (0.036 - 0.178)
5-14 years (rate per capita)	0.279 (0.197 - 0.379)	0.283 (0.202 - 0.378)	0.284 (0.205 - 0.379)	0.479 (0.305 - 0.742)	0.471 (0.300 - 0.730)	0.470 (0.300 - 0.728)	0.065 (0.027 - 0.134)	0.066 (0.028 - 0.138)	0.066 (0.028 - 0.137)
All ages (rate per capita)	0.140 (0.109 - 0.183)	0.138 (0.108 - 0.179)	0.138 (0.108 - 0.179)	0.221 (0.158 - 0.307)	0.213 (0.152 - 0.294)	0.212 (0.152 - 0.293)	0.033 (0.014 - 0.068)	0.032 (0.014 - 0.067)	0.032 (0.014 - 0.067)
Age-standardised (rate per capita)	0.078 (0.061 - 0.102)	0.079 (0.062 - 0.103)	0.079 (0.062 - 0.103)	0.124 (0.088 - 0.173)	0.123 (0.088 - 0.172)	0.123 (0.088 - 0.172)	0.018 (0.008 - 0.038)	0.018 (0.008 - 0.038)	0.019 (0.008 - 0.038)
Total number of cases (thousands)	27,912 (21,660 - 36,406)	41,012 (32,092 - 53,066)	54,062 (42,403 - 69,903)	43,837 (31,328 - 61,005)	63,188 (45,270 - 87,183)	83,017 (59,499 - 114,695)	7 (3 - 13)	10 (4 - 20)	13 (5 - 26)
**Benin**									
Under-5 years (rate per capita)	0.356 (0.232 - 0.493)	0.361 (0.236 - 0.499)	0.364 (0.238 - 0.504)	0.497 (0.288 - 0.691)	0.504 (0.292 - 0.701)	0.508 (0.294 - 0.707)	0.082 (0.033 - 0.174)	0.084 (0.034 - 0.177)	0.085 (0.035 - 0.181)
5-14 years (rate per capita)	0.272 (0.182 - 0.376)	0.269 (0.180 - 0.373)	0.264 (0.177 - 0.367)	0.487 (0.303 - 0.767)	0.482 (0.300 - 0.760)	0.475 (0.295 - 0.748)	0.064 (0.025 - 0.133)	0.063 (0.026 - 0.133)	0.062 (0.025 - 0.132)
All ages (rate per capita)	0.141 (0.106 - 0.187)	0.136 (0.103 - 0.181)	0.128 (0.097 - 0.170)	0.226 (0.161 - 0.315)	0.219 (0.156 - 0.305)	0.206 (0.147 - 0.288)	0.033 (0.014 - 0.068)	0.032 (0.013 - 0.066)	0.030 (0.013 - 0.063)
Age-standardised (rate per capita)	0.077 (0.058 - 0.103)	0.077 (0.058 - 0.103)	0.077 (0.058 - 0.103)	0.125 (0.088 - 0.175)	0.125 (0.088 - 0.175)	0.125 (0.088 - 0.175)	0.018 (0.008 - 0.037)	0.018 (0.008 - 0.037)	0.018 (0.008 - 0.038)
Total number of cases (thousands)	704 (532 - 936)	1,111 (841 - 1,478)	1,395 (1,058 - 1,859)	1,131 (807 - 1,575)	1,790 (1,275 - 2,497)	2,253 (1,607 - 3,148)	0 (0 - 0)	0 (0 - 1)	0 (0 - 1)
**Burkina Faso**									
Under-5 years (rate per capita)	0.277 (0.196 - 0.366)	0.281 (0.196 - 0.369)	0.284 (0.198 - 0.373)	0.452 (0.221 - 0.686)	0.452 (0.214 - 0.687)	0.457 (0.216 - 0.695)	0.064 (0.027 - 0.133)	0.065 (0.027 - 0.127)	0.066 (0.027 - 0.135)
5-14 years (rate per capita)	0.290 (0.212 - 0.374)	0.375 (0.324 - 0.429)	0.374 (0.323 - 0.428)	0.400 (0.258 - 0.607)	0.299 (0.193 - 0.428)	0.298 (0.193 - 0.427)	0.068 (0.029 - 0.140)	0.087 (0.038 - 0.170)	0.088 (0.038 - 0.167)
All ages (rate per capita)	0.135 (0.110 - 0.163)	0.157 (0.135 - 0.179)	0.155 (0.133 - 0.177)	0.199 (0.139 - 0.279)	0.168 (0.110 - 0.235)	0.163 (0.107 - 0.228)	0.031 (0.013 - 0.064)	0.036 (0.016 - 0.071)	0.036 (0.016 - 0.069)
Age-standardised (rate per capita)	0.073 (0.059 - 0.088)	0.088 (0.076 - 0.100)	0.088 (0.076 - 0.100)	0.107 (0.075 - 0.150)	0.090 (0.059 - 0.126)	0.090 (0.059 - 0.126)	0.017 (0.007 - 0.034)	0.020 (0.009 - 0.040)	0.020 (0.009 - 0.039)
Total number of cases (thousands)	1,186 (966 - 1,440)	2,110 (1,809 - 2,411)	2,799 (2,409 - 3,196)	1,751 (1,224 - 2,456)	2,255 (1,473 - 3,152)	2,954 (1,942 - 4,124)	0 (0 - 1)	0 (0 - 1)	1 (0 - 1)
**Cameroon**									
Under-5 years (rate per capita)	0.360 (0.235 - 0.498)	0.361 (0.235 - 0.499)	0.364 (0.238 - 0.504)	0.503 (0.291 - 0.699)	0.504 (0.291 - 0.700)	0.509 (0.294 - 0.707)	0.084 (0.035 - 0.182)	0.084 (0.036 - 0.186)	0.085 (0.035 - 0.182)
5-14 years (rate per capita)	0.275 (0.185 - 0.381)	0.265 (0.178 - 0.368)	0.266 (0.178 - 0.369)	0.493 (0.307 - 0.775)	0.475 (0.295 - 0.749)	0.477 (0.296 - 0.752)	0.064 (0.027 - 0.132)	0.062 (0.025 - 0.130)	0.062 (0.025 - 0.134)
All ages (rate per capita)	0.144 (0.109 - 0.191)	0.134 (0.101 - 0.178)	0.129 (0.098 - 0.172)	0.231 (0.165 - 0.321)	0.216 (0.154 - 0.301)	0.209 (0.149 - 0.291)	0.033 (0.014 - 0.071)	0.031 (0.013 - 0.065)	0.030 (0.013 - 0.063)
Age-standardised (rate per capita)	0.077 (0.058 - 0.103)	0.077 (0.058 - 0.103)	0.077 (0.058 - 0.103)	0.125 (0.088 - 0.175)	0.125 (0.088 - 0.175)	0.125 (0.088 - 0.175)	0.018 (0.008 - 0.038)	0.018 (0.008 - 0.037)	0.018 (0.008 - 0.037)
Total number of cases (thousands)	1,737 (1,313 - 2,308)	2,433 (1,839 - 3,239)	3,026 (2,292 - 4,030)	2,789 (1,993 - 3,881)	3,919 (2,791 - 5,468)	4,883 (3,481 - 6,821)	0 (0 - 1)	1 (0 - 1)	1 (0 - 1)
**Cape Verde**									
Under-5 years (rate per capita)	0.367 (0.239 - 0.507)	0.372 (0.243 - 0.515)	0.369 (0.241 - 0.510)	0.512 (0.296 - 0.712)	0.519 (0.301 - 0.722)	0.515 (0.298 - 0.716)	0.085 (0.036 - 0.181)	0.087 (0.036 - 0.185)	0.086 (0.036 - 0.183)
5-14 years (rate per capita)	0.277 (0.187 - 0.384)	0.245 (0.163 - 0.342)	0.255 (0.171 - 0.356)	0.497 (0.310 - 0.781)	0.441 (0.272 - 0.694)	0.460 (0.284 - 0.722)	0.065 (0.026 - 0.135)	0.057 (0.023 - 0.120)	0.060 (0.025 - 0.129)
All ages (rate per capita)	0.145 (0.110 - 0.193)	0.108 (0.082 - 0.145)	0.087 (0.066 - 0.117)	0.233 (0.166 - 0.323)	0.177 (0.124 - 0.250)	0.142 (0.100 - 0.199)	0.034 (0.015 - 0.072)	0.025 (0.011 - 0.053)	0.020 (0.009 - 0.043)
Age-standardised (rate per capita)	0.077 (0.058 - 0.103)	0.077 (0.058 - 0.103)	0.077 (0.058 - 0.103)	0.125 (0.088 - 0.175)	0.125 (0.088 - 0.175)	0.125 (0.088 - 0.175)	0.018 (0.008 - 0.038)	0.018 (0.008 - 0.038)	0.018 (0.008 - 0.038)
Total number of cases (thousands)	50 (38 - 66)	51 (39 - 69)	45 (34 - 61)	80 (57 - 111)	84 (59 - 119)	74 (52 - 104)	0 (0 - 0)	0 (0 - 0)	0 (0 - 0)
**Chad**									
Under-5 years (rate per capita)	0.354 (0.231 - 0.490)	0.356 (0.233 - 0.493)	0.360 (0.235 - 0.498)	0.494 (0.286 - 0.687)	0.498 (0.288 - 0.692)	0.503 (0.291 - 0.699)	0.082 (0.034 - 0.175)	0.083 (0.035 - 0.175)	0.084 (0.035 - 0.180)
5-14 years (rate per capita)	0.274 (0.185 - 0.380)	0.274 (0.184 - 0.379)	0.270 (0.181 - 0.374)	0.492 (0.307 - 0.774)	0.491 (0.306 - 0.772)	0.484 (0.301 - 0.762)	0.064 (0.026 - 0.132)	0.064 (0.026 - 0.132)	0.063 (0.025 - 0.131)
All ages (rate per capita)	0.146 (0.110 - 0.194)	0.151 (0.114 - 0.200)	0.146 (0.110 - 0.194)	0.234 (0.167 - 0.325)	0.242 (0.173 - 0.337)	0.235 (0.168 - 0.328)	0.034 (0.015 - 0.070)	0.035 (0.015 - 0.073)	0.034 (0.015 - 0.072)
Age-standardised (rate per capita)	0.077 (0.058 - 0.103)	0.077 (0.058 - 0.103)	0.077 (0.058 - 0.103)	0.125 (0.088 - 0.175)	0.125 (0.088 - 0.175)	0.125 (0.088 - 0.175)	0.018 (0.008 - 0.037)	0.018 (0.008 - 0.038)	0.018 (0.008 - 0.038)
Total number of cases (thousands)	869 (657 - 1,155)	1,514 (1,144 - 2,011)	2,054 (1,553 - 2,730)	1,394 (995 - 1,938)	2,432 (1,737 - 3,386)	3,305 (2,356 - 4,606)	0 (0 - 0)	0 (0 - 1)	0 (0 - 1)
**Cote d'Ivoire**									
Under-5 years (rate per capita)	0.359 (0.234 - 0.497)	0.362 (0.236 - 0.501)	0.362 (0.237 - 0.501)	0.501 (0.290 - 0.697)	0.506 (0.293 - 0.704)	0.506 (0.293 - 0.704)	0.083 (0.034 - 0.179)	0.084 (0.035 - 0.178)	0.084 (0.034 - 0.178)
5-14 years (rate per capita)	0.271 (0.182 - 0.376)	0.271 (0.182 - 0.376)	0.264 (0.177 - 0.367)	0.487 (0.303 - 0.766)	0.487 (0.303 - 0.766)	0.474 (0.294 - 0.746)	0.063 (0.027 - 0.133)	0.063 (0.026 - 0.135)	0.062 (0.025 - 0.130)

	Prevalence 1990	Prevalence 2005	Prevalence 2015	Incidence 1990	Incidence 2005	Incidence 2015	YLD rate 1990	YLD rate 2005	YLD rate 2015
**All ages (rate per capita)**	0.137 (0.103 - 0.182)	0.135 (0.102 - 0.179)	0.128 (0.097 - 0.170)	0.220 (0.157 - 0.307)	0.218 (0.155 - 0.304)	0.206 (0.147 - 0.288)	0.032 (0.014 - 0.066)	0.031 (0.013 - 0.065)	0.030 (0.013 - 0.063)
**Age-standardised (rate per capita)**	0.077 (0.058 - 0.103)	0.077 (0.058 - 0.103)	0.077 (0.058 - 0.103)	0.125 (0.088 - 0.175)	0.125 (0.088 - 0.175)	0.125 (0.088 - 0.175)	0.018 (0.008 - 0.037)	0.018 (0.008 - 0.037)	0.018 (0.008 - 0.038)
**Total number of cases (thousands)**	1,663 (1,258 - 2,210)	2,441 (1,850 - 3,251)	2,901 (2,198 - 3,864)	2,677 (1,907 - 3,731)	3,940 (2,804 - 5,504)	4,682 (3,340 - 6,542)	0 (0 - 1)	1 (0 - 1)	1 (0 - 1)
**The Gambia**									
**Under-5 years (rate per capita)**	0.359 (0.234 - 0.496)	0.362 (0.236 - 0.501)	0.364 (0.237 - 0.503)	0.501 (0.290 - 0.697)	0.505 (0.293 - 0.703)	0.508 (0.294 - 0.706)	0.083 (0.034 - 0.178)	0.084 (0.035 - 0.179)	0.085 (0.036 - 0.184)
**5-14 years (rate per capita)**	0.276 (0.186 - 0.382)	0.274 (0.184 - 0.380)	0.271 (0.182 - 0.376)	0.494 (0.308 - 0.776)	0.491 (0.306 - 0.773)	0.487 (0.303 - 0.766)	0.064 (0.026 - 0.136)	0.064 (0.027 - 0.135)	0.063 (0.027 - 0.134)
**All ages (rate per capita)**	0.144 (0.109 - 0.191)	0.144 (0.109 - 0.192)	0.143 (0.108 - 0.190)	0.231 (0.165 - 0.320)	0.231 (0.165 - 0.321)	0.230 (0.164 - 0.320)	0.034 (0.015 - 0.070)	0.034 (0.014 - 0.071)	0.033 (0.014 - 0.070)
**Age-standardised (rate per capita)**	0.077 (0.058 - 0.103)	0.077 (0.058 - 0.103)	0.077 (0.058 - 0.103)	0.125 (0.088 - 0.175)	0.125 (0.088 - 0.175)	0.125 (0.088 - 0.175)	0.018 (0.008 - 0.038)	0.018 (0.008 - 0.037)	0.018 (0.008 - 0.038)
**Total number of cases (thousands)**	133 (101 - 176)	209 (158 - 278)	287 (216 - 381)	213 (152 - 295)	335 (239 - 465)	460 (329 - 640)	0 (0 - 0)	0 (0 - 0)	0 (0 - 0)
**Ghana**									
**Under-5 years (rate per capita)**	0.361 (0.236 - 0.500)	0.363 (0.237 - 0.502)	0.365 (0.238 - 0.505)	0.505 (0.292 - 0.702)	0.506 (0.293 - 0.704)	0.510 (0.295 - 0.709)	0.084 (0.034 - 0.179)	0.084 (0.034 - 0.177)	0.085 (0.035 - 0.181)
**5-14 years (rate per capita)**	0.267 (0.179 - 0.370)	0.260 (0.174 - 0.361)	0.265 (0.178 - 0.368)	0.479 (0.298 - 0.755)	0.467 (0.289 - 0.733)	0.476 (0.296 - 0.749)	0.062 (0.025 - 0.132)	0.061 (0.025 - 0.132)	0.062 (0.026 - 0.131)
**All ages (rate per capita)**	0.133 (0.101 - 0.177)	0.120 (0.091 - 0.160)	0.118 (0.089 - 0.157)	0.214 (0.153 - 0.299)	0.194 (0.138 - 0.271)	0.190 (0.135 - 0.265)	0.031 (0.013 - 0.064)	0.028 (0.012 - 0.058)	0.028 (0.012 - 0.059)
**Age-standardised (rate per capita)**	0.077 (0.058 - 0.103)	0.077 (0.058 - 0.103)	0.077 (0.058 - 0.103)	0.125 (0.088 - 0.175)	0.125 (0.088 - 0.175)	0.125 (0.088 - 0.175)	0.018 (0.008 - 0.037)	0.018 (0.008 - 0.038)	0.018 (0.008 - 0.039)
**Total number of cases (thousands)**	1,946 (1,473 - 2,590)	2,563 (1,947 - 3,415)	3,229 (2,443 - 4,299)	3,138 (2,236 - 4,383)	4,142 (2,950 - 5,794)	5,206 (3,710 - 7,269)	0 (0 - 1)	1 (0 - 1)	1 (0 - 2)
**Guinea**									
**Under-5 years (rate per capita)**	0.351 (0.229 - 0.486)	0.361 (0.236 - 0.500)	0.363 (0.237 - 0.502)	0.491 (0.284 - 0.683)	0.504 (0.292 - 0.701)	0.506 (0.293 - 0.704)	0.081 (0.034 - 0.171)	0.084 (0.035 - 0.176)	0.084 (0.035 - 0.176)
**5-14 years (rate per capita)**	0.272 (0.183 - 0.377)	0.268 (0.180 - 0.372)	0.266 (0.179 - 0.369)	0.488 (0.304 - 0.768)	0.482 (0.299 - 0.759)	0.478 (0.297 - 0.753)	0.063 (0.026 - 0.132)	0.063 (0.026 - 0.130)	0.062 (0.024 - 0.128)
**All ages (rate per capita)**	0.133 (0.101 - 0.177)	0.135 (0.102 - 0.179)	0.128 (0.097 - 0.171)	0.214 (0.153 - 0.298)	0.217 (0.155 - 0.302)	0.207 (0.147 - 0.289)	0.031 (0.013 - 0.066)	0.031 (0.014 - 0.065)	0.030 (0.013 - 0.063)
**Age-standardised (rate per capita)**	0.077 (0.058 - 0.103)	0.077 (0.058 - 0.103)	0.077 (0.058 - 0.103)	0.125 (0.088 - 0.175)	0.125 (0.088 - 0.175)	0.125 (0.088 - 0.175)	0.018 (0.008 - 0.038)	0.018 (0.008 - 0.037)	0.018 (0.008 - 0.038)
**Total number of cases (thousands)**	803 (607 - 1,067)	1,307 (988 - 1,737)	1,611 (1,222 - 2,147)	1,291 (921 - 1,798)	2,103 (1,499 - 2,931)	2,602 (1,853 - 3,638)	0 (0 - 0)	0 (0 - 1)	0 (0 - 1)
**Guinea-Bissau**									
**Under-5 years (rate per capita)**	0.357 (0.233 - 0.494)	0.360 (0.235 - 0.498)	0.362 (0.236 - 0.501)	0.498 (0.288 - 0.693)	0.502 (0.291 - 0.698)	0.505 (0.293 - 0.703)	0.083 (0.035 - 0.178)	0.084 (0.034 - 0.178)	0.084 (0.035 - 0.180)
**5-14 years (rate per capita)**	0.276 (0.186 - 0.383)	0.263 (0.176 - 0.366)	0.266 (0.178 - 0.369)	0.495 (0.309 - 0.778)	0.473 (0.293 - 0.745)	0.477 (0.296 - 0.752)	0.065 (0.027 - 0.134)	0.062 (0.025 - 0.131)	0.062 (0.026 - 0.130)
**All ages (rate per capita)**	0.138 (0.105 - 0.184)	0.128 (0.097 - 0.171)	0.124 (0.094 - 0.165)	0.222 (0.159 - 0.309)	0.207 (0.148 - 0.289)	0.200 (0.142 - 0.279)	0.032 (0.014 - 0.067)	0.030 (0.013 - 0.063)	0.029 (0.012 - 0.061)
**Age-standardised (rate per capita)**	0.077 (0.058 - 0.103)	0.077 (0.058 - 0.103)	0.077 (0.058 - 0.103)	0.125 (0.088 - 0.175)	0.125 (0.088 - 0.175)	0.125 (0.088 - 0.175)	0.018 (0.008 - 0.037)	0.018 (0.008 - 0.038)	0.018 (0.008 - 0.038)
**Total number of cases (thousands)**	146 (111 - 194)	188 (142 - 251)	229 (173 - 305)	235 (168 - 327)	304 (216 - 424)	369 (263 - 515)	0 (0 - 0)	0 (0 - 0)	0 (0 - 0)
**Liberia**									
**Under-5 years (rate per capita)**	0.363 (0.237 - 0.502)	0.359 (0.235 - 0.497)	0.364 (0.238 - 0.504)	0.507 (0.294 - 0.705)	0.502 (0.291 - 0.698)	0.509 (0.295 - 0.708)	0.084 (0.035 - 0.180)	0.084 (0.035 - 0.176)	0.085 (0.035 - 0.181)
**5-14 years (rate per capita)**	0.270 (0.182 - 0.375)	0.273 (0.184 - 0.378)	0.264 (0.177 - 0.367)	0.485 (0.302 - 0.764)	0.490 (0.305 - 0.770)	0.475 (0.295 - 0.747)	0.063 (0.025 - 0.131)	0.064 (0.026 - 0.134)	0.062 (0.026 - 0.130)
**All ages (rate per capita)**	0.142 (0.108 - 0.189)	0.133 (0.101 - 0.177)	0.128 (0.097 - 0.170)	0.227 (0.162 - 0.316)	0.214 (0.153 - 0.299)	0.207 (0.147 - 0.289)	0.033 (0.014 - 0.069)	0.031 (0.014 - 0.064)	0.030 (0.013 - 0.062)
**Age-standardised (rate per capita)**	0.077 (0.058 - 0.103)	0.077 (0.058 - 0.103)	0.077 (0.058 - 0.103)	0.125 (0.088 - 0.175)	0.125 (0.088 - 0.175)	0.125 (0.088 - 0.175)	0.018 (0.008 - 0.038)	0.018 (0.008 - 0.037)	0.018 (0.008 - 0.037)
**Total number of cases (thousands)**	304 (230 - 403)	436 (330 - 580)	576 (437 - 767)	486 (347 - 676)	702 (501 - 978)	931 (662 - 1,303)	0 (0 - 0)	0 (0 - 0)	0 (0 - 0)
**Mali**									
**Under-5 years (rate per capita)**	0.355 (0.231 - 0.491)	0.356 (0.232 - 0.492)	0.361 (0.236 - 0.500)	0.495 (0.287 - 0.689)	0.497 (0.288 - 0.691)	0.504 (0.292 - 0.702)	0.082 (0.034 - 0.176)	0.082 (0.035 - 0.176)	0.084 (0.035 - 0.175)
**5-14 years (rate per capita)**	0.268 (0.180 - 0.372)	0.271 (0.182 - 0.375)	0.274 (0.184 - 0.379)	0.481 (0.299 - 0.758)	0.486 (0.302 - 0.764)	0.491 (0.306 - 0.772)	0.063 (0.026 - 0.133)	0.063 (0.026 - 0.132)	0.064 (0.026 - 0.136)
**All ages (rate per capita)**	0.142 (0.107 - 0.188)	0.143 (0.108 - 0.190)	0.146 (0.111 - 0.194)	0.228 (0.163 - 0.317)	0.229 (0.163 - 0.319)	0.235 (0.168 - 0.329)	0.033 (0.014 - 0.069)	0.033 (0.014 - 0.070)	0.034 (0.015 - 0.072)
**Age-standardised (rate per capita)**	0.077 (0.058 - 0.103)	0.077 (0.058 - 0.103)	0.077 (0.058 - 0.103)	0.125 (0.088 - 0.175)	0.125 (0.088 - 0.175)	0.125 (0.088 - 0.175)	0.018 (0.008 - 0.038)	0.018 (0.008 - 0.038)	0.018 (0.008 - 0.038)
**Total number of cases (thousands)**	1,206 (911 - 1,603)	1,840 (1,389 - 2,445)	2,565 (1,943 - 3,413)	1,939 (1,384 - 2,700)	2,953 (2,109 - 4,110)	4,136 (2,946 - 5,773)	0 (0 - 1)	0 (0 - 1)	1 (0 - 1)
**Mauritania**									
**Under-5 years (rate per capita)**	0.361 (0.236 - 0.500)	0.363 (0.237 - 0.502)	0.366 (0.239 - 0.506)	0.505 (0.292 - 0.702)	0.507 (0.293 - 0.705)	0.511 (0.296 - 0.710)	0.084 (0.035 - 0.179)	0.084 (0.035 - 0.178)	0.085 (0.035 - 0.180)
**5-14 years (rate per capita)**	0.267 (0.179 - 0.370)	0.266 (0.179 - 0.369)	0.264 (0.177 - 0.367)	0.479 (0.298 - 0.755)	0.478 (0.297 - 0.752)	0.474 (0.294 - 0.746)	0.062 (0.026 - 0.131)	0.062 (0.025 - 0.133)	0.062 (0.025 - 0.131)
**All ages (rate per capita)**	0.136 (0.103 - 0.181)	0.127 (0.096 - 0.169)	0.122 (0.092 - 0.162)	0.219 (0.156 - 0.305)	0.205 (0.146 - 0.286)	0.196 (0.140 - 0.275)	0.032 (0.014 - 0.065)	0.030 (0.013 - 0.062)	0.028 (0.012 - 0.060)
**Age-standardised (rate per capita)**	0.077 (0.058 - 0.103)	0.077 (0.058 - 0.103)	0.077 (0.058 - 0.103)	0.125 (0.088 - 0.175)	0.125 (0.088 - 0.175)	0.125 (0.088 - 0.175)	0.018 (0.008 - 0.037)	0.018 (0.008 - 0.038)	0.018 (0.008 - 0.038)
**Total number of cases (thousands)**	276 (208 - 367)	402 (304 - 535)	497 (377 - 662)	444 (316 - 619)	648 (461 - 904)	803 (573 - 1,122)	0 (0 - 0)	0 (0 - 0)	0 (0 - 0)
**Niger**									
**Under-5 years (rate per capita)**	0.437 (0.339 - 0.512)	0.447 (0.347 - 0.523)	0.451 (0.350 - 0.528)	0.496 (0.336 - 0.647)	0.507 (0.343 - 0.661)	0.512 (0.346 - 0.668)	0.102 (0.044 - 0.193)	0.104 (0.045 - 0.197)	0.105 (0.045 - 0.200)
**5-14 years (rate per capita)**	0.463 (0.378 - 0.533)	0.474 (0.389 - 0.545)	0.472 (0.387 - 0.543)	0.466 (0.325 - 0.656)	0.476 (0.333 - 0.670)	0.474 (0.331 - 0.667)	0.108 (0.046 - 0.207)	0.111 (0.048 - 0.215)	0.111 (0.047 - 0.216)
**All ages (rate per capita)**	0.216 (0.188 - 0.242)	0.227 (0.198 - 0.254)	0.233 (0.204 - 0.262)	0.228 (0.177 - 0.300)	0.239 (0.186 - 0.315)	0.247 (0.191 - 0.324)	0.050 (0.022 - 0.099)	0.053 (0.023 - 0.103)	0.055 (0.024 - 0.106)
**Age-standardised (rate per capita)**	0.119 (0.103 - 0.133)	0.119 (0.103 - 0.133)	0.119 (0.103 - 0.133)	0.125 (0.096 - 0.165)	0.125 (0.096 - 0.165)	0.125 (0.096 - 0.165)	0.028 (0.012 - 0.054)	0.028 (0.012 - 0.054)	0.028 (0.012 - 0.054)
**Total number of cases (thousands)**	1,709 (1,491 - 1,920)	3,048 (2,659 - 3,419)	4,635 (4,046 - 5,202)	1,809 (1,405 - 2,377)	3,221 (2,496 - 4,233)	4,895 (3,795 - 6,431)	0 (0 - 1)	1 (0 - 1)	1 (0 - 2)
**Nigeria**									
**Under-5 years (rate per capita)**	0.357 (0.233 - 0.494)	0.358 (0.234 - 0.495)	0.363 (0.237 - 0.502)	0.498 (0.288 - 0.693)	0.500 (0.290 - 0.696)	0.506 (0.293 - 0.705)	0.083 (0.034 - 0.179)	0.083 (0.034 - 0.175)	0.084 (0.035 - 0.183)
**5-14 years (rate per capita)**	0.269 (0.181 - 0.373)	0.270 (0.181 - 0.374)	0.269 (0.181 - 0.373)	0.483 (0.300 - 0.760)	0.484 (0.301 - 0.762)	0.483 (0.301 - 0.760)	0.063 (0.026 - 0.131)	0.063 (0.026 - 0.135)	0.063 (0.026 - 0.130)
**All ages (rate per capita)**	0.136 (0.103 - 0.182)	0.133 (0.101 - 0.177)	0.135 (0.102 - 0.179)	0.220 (0.157 - 0.307)	0.214 (0.153 - 0.298)	0.217 (0.155 - 0.303)	0.032 (0.014 - 0.067)	0.031 (0.014 - 0.064)	0.031 (0.013 - 0.066)
**Age-standardised (rate per capita)**	0.077 (0.058 - 0.103)	0.077 (0.058 - 0.103)	0.077 (0.058 - 0.103)	0.125 (0.088 - 0.175)	0.125 (0.088 - 0.175)	0.125 (0.088 - 0.175)	0.018 (0.008 - 0.038)	0.018 (0.008 - 0.037)	0.018 (0.008 - 0.038)
**Total number of cases (thousands)**	13,068 (9,882 - 17,379)	18,583 (14,040 - 24,694)	24,580 (18,588 - 32,687)	21,049 (15,000 - 29,357)	29,877 (21,331 - 41,614)	39,591 (28,223 - 55,222)	3 (1 - 6)	4 (2 - 9)	6 (2 - 12)
**Sao Tome and Principe**									
**Under-5 years (rate per capita)**	0.363 (0.237 - 0.502)	0.365 (0.238 - 0.505)	0.367 (0.240 - 0.508)	0.506 (0.293 - 0.704)	0.510 (0.295 - 0.709)	0.513 (0.297 - 0.713)	0.084 (0.035 - 0.180)	0.085 (0.035 - 0.182)	0.086 (0.035 - 0.185)
**5-14 years (rate per capita)**	0.251 (0.167 - 0.349)	0.268 (0.180 - 0.371)	0.262 (0.176 - 0.364)	0.451 (0.278 - 0.710)	0.480 (0.298 - 0.757)	0.471 (0.292 - 0.741)	0.059 (0.024 - 0.124)	0.063 (0.025 - 0.133)	0.061 (0.025 - 0.129)
**All ages (rate per capita)**	0.139 (0.106 - 0.186)	0.133 (0.101 - 0.177)	0.129 (0.098 - 0.172)	0.225 (0.160 - 0.315)	0.214 (0.153 - 0.299)	0.208 (0.148 - 0.292)	0.032 (0.014 - 0.069)	0.031 (0.013 - 0.065)	0.030 (0.013 - 0.063)
**Age-standardised (rate per capita)**	0.077 (0.058 - 0.103)	0.077 (0.058 - 0.103)	0.077 (0.058 - 0.103)	0.125 (0.088 - 0.175)	0.125 (0.088 - 0.175)	0.125 (0.088 - 0.175)	0.018 (0.008 - 0.038)	0.018 (0.008 - 0.037)	0.018 (0.008 - 0.038)
**Total number of cases (thousands)**	16 (12 - 21)	20 (15 - 27)	25 (19 - 33)	26 (18 - 36)	33 (23 - 46)	40 (28 - 56)	0 (0 - 0)	0 (0 - 0)	0 (0 - 0)
**Senegal**									
**Under-5 years (rate per capita)**	0.360 (0.235 - 0.498)	0.361 (0.236 - 0.500)	0.364 (0.238 - 0.504)	0.502 (0.291 - 0.698)	0.505 (0.292 - 0.702)	0.508 (0.294 - 0.707)	0.083 (0.034 - 0.178)	0.084 (0.035 - 0.179)	0.085 (0.035 - 0.179)
**5-14 years (rate per capita)**	0.268 (0.180 - 0.372)	0.264 (0.177 - 0.367)	0.269 (0.181 - 0.373)	0.482 (0.299 - 0.759)	0.474 (0.294 - 0.747)	0.483 (0.301 - 0.761)	0.063 (0.025 - 0.132)	0.062 (0.025 - 0.129)	0.063 (0.027 - 0.133)
**All ages (rate per capita)**	0.143 (0.108 - 0.190)	0.133 (0.101 - 0.177)	0.134 (0.101 - 0.178)	0.231 (0.164 - 0.322)	0.215 (0.153 - 0.299)	0.215 (0.154 - 0.300)	0.033 (0.014 - 0.069)	0.031 (0.013 - 0.064)	0.031 (0.013 - 0.065)
**Age-standardised (rate per capita)**	0.077 (0.058 - 0.103)	0.077 (0.058 - 0.103)	0.077 (0.058 - 0.103)	0.125 (0.088 - 0.175)	0.125 (0.088 - 0.175)	0.125 (0.088 - 0.175)	0.018 (0.008 - 0.037)	0.018 (0.008 - 0.037)	0.018 (0.008 - 0.038)
**Total number of cases (thousands)**	1,075 (813 - 1,430)	1,503 (1,136 - 2,001)	2,022 (1,528 - 2,688)	1,732 (1,233 - 2,415)	2,422 (1,726 - 3,380)	3,254 (2,320 - 4,537)	0 (0 - 1)	0 (0 - 1)	0 (0 - 1)
**Sierra Leone**									
**Under-5 years (rate per capita)**	0.233 (0.154 - 0.312)	0.224 (0.145 - 0.308)	0.229 (0.148 - 0.315)	0.444 (0.183 - 0.709)	0.407 (0.175 - 0.677)	0.416 (0.179 - 0.692)	0.054 (0.022 - 0.111)	0.052 (0.021 - 0.108)	0.053 (0.021 - 0.112)
**5-14 years (rate per capita)**	0.314 (0.257 - 0.378)	0.245 (0.172 - 0.319)	0.242 (0.170 - 0.316)	0.470 (0.286 - 0.708)	0.375 (0.229 - 0.580)	0.371 (0.227 - 0.576)	0.073 (0.031 - 0.142)	0.057 (0.024 - 0.121)	0.057 (0.024 - 0.119)

	Prevalence 1990	Prevalence 2005	Prevalence 2015	Incidence 1990	Incidence 2005	Incidence 2015	YLD rate 1990	YLD rate 2005	YLD rate 2015
All ages (rate per capita)	0.124 (0.101 - 0.147)	0.103 (0.080 - 0.129)	0.101 (0.078 - 0.125)	0.203 (0.136 - 0.293)	0.169 (0.110 - 0.252)	0.165 (0.108 - 0.245)	0.029 (0.012 - 0.057)	0.024 (0.010 - 0.049)	0.024 (0.010 - 0.048)
Age-standardised (rate per capita)	0.073 (0.059 - 0.086)	0.061 (0.047 - 0.076)	0.061 (0.047 - 0.076)	0.118 (0.079 - 0.171)	0.099 (0.065 - 0.147)	0.099 (0.065 - 0.147)	0.017 (0.007 - 0.033)	0.014 (0.006 - 0.029)	0.014 (0.006 - 0.029)
Total number of cases (thousands)	487 (396 - 578)	521 (403 - 649)	651 (505 - 810)	795 (533 - 1,150)	852 (556 - 1,270)	1,064 (695 - 1,583)	0 (0 - 0)	0 (0 - 0)	0 (0 - 0)
Togo									
Under-5 years (rate per capita)	0.360 (0.235 - 0.499)	0.361 (0.235 - 0.499)	0.364 (0.238 - 0.504)	0.503 (0.291 - 0.700)	0.504 (0.291 - 0.700)	0.509 (0.295 - 0.708)	0.084 (0.035 - 0.176)	0.084 (0.034 - 0.174)	0.085 (0.034 - 0.180)
5-14 years (rate per capita)	0.269 (0.181 - 0.374)	0.265 (0.178 - 0.368)	0.268 (0.180 - 0.371)	0.484 (0.301 - 0.762)	0.476 (0.295 - 0.749)	0.480 (0.298 - 0.757)	0.063 (0.025 - 0.134)	0.062 (0.025 - 0.131)	0.063 (0.025 - 0.135)
All ages (rate per capita)	0.141 (0.107 - 0.187)	0.131 (0.099 - 0.174)	0.128 (0.097 - 0.171)	0.227 (0.162 - 0.317)	0.211 (0.150 - 0.294)	0.207 (0.148 - 0.290)	0.033 (0.014 - 0.068)	0.030 (0.013 - 0.064)	0.030 (0.013 - 0.064)
Age-standardised (rate per capita)	0.077 (0.058 - 0.103)	0.077 (0.058 - 0.103)	0.077 (0.058 - 0.103)	0.125 (0.088 - 0.175)	0.125 (0.088 - 0.175)	0.125 (0.088 - 0.175)	0.018 (0.008 - 0.037)	0.018 (0.008 - 0.038)	0.018 (0.008 - 0.038)
Total number of cases (thousands)	534 (404 - 710)	730 (552 - 972)	937 (711 - 1,249)	860 (612 - 1,199)	1,176 (837 - 1,640)	1,514 (1,077 - 2,118)	0 (0 - 0)	0 (0 - 0)	0 (0 - 0)
Eastern Sub-Saharan Africa									
Under-5 years (rate per capita)	0.364 (0.273 - 0.459)	0.348 (0.264 - 0.433)	0.357 (0.270 - 0.451)	0.529 (0.320 - 0.721)	0.469 (0.307 - 0.612)	0.518 (0.317 - 0.702)	0.085 (0.036 - 0.166)	0.081 (0.035 - 0.159)	0.083 (0.036 - 0.163)
5-14 years (rate per capita)	0.280 (0.211 - 0.358)	0.260 (0.188 - 0.343)	0.266 (0.194 - 0.350)	0.451 (0.290 - 0.661)	0.438 (0.271 - 0.663)	0.446 (0.280 - 0.662)	0.065 (0.027 - 0.133)	0.061 (0.025 - 0.128)	0.062 (0.026 - 0.132)
All ages (rate per capita)	0.147 (0.122 - 0.178)	0.134 (0.109 - 0.165)	0.131 (0.107 - 0.162)	0.226 (0.163 - 0.304)	0.205 (0.148 - 0.280)	0.207 (0.147 - 0.284)	0.034 (0.015 - 0.070)	0.031 (0.014 - 0.063)	0.031 (0.013 - 0.063)
Age-standardised (rate per capita)	0.079 (0.065 - 0.096)	0.074 (0.060 - 0.091)	0.076 (0.062 - 0.095)	0.122 (0.087 - 0.165)	0.114 (0.082 - 0.157)	0.121 (0.086 - 0.167)	0.018 (0.008 - 0.037)	0.017 (0.008 - 0.035)	0.018 (0.008 - 0.037)
Total number of cases (thousands)	27,409 (22,644 - 33,093)	37,906 (30,918 - 46,617)	49,513 (40,263 - 61,028)	42,088 (30,258 - 56,537)	58,023 (41,865 - 79,046)	77,869 (55,399 - 106,936)	6 (3 - 13)	9 (4 - 18)	12 (5 - 24)
Burundi									
Under-5 years (rate per capita)	0.401 (0.284 - 0.517)	0.399 (0.283 - 0.514)	0.405 (0.287 - 0.521)	0.550 (0.340 - 0.732)	0.547 (0.338 - 0.728)	0.555 (0.343 - 0.739)	0.094 (0.039 - 0.186)	0.093 (0.039 - 0.189)	0.095 (0.040 - 0.189)
5-14 years (rate per capita)	0.279 (0.192 - 0.378)	0.258 (0.177 - 0.351)	0.276 (0.190 - 0.375)	0.506 (0.298 - 0.782)	0.469 (0.274 - 0.731)	0.501 (0.295 - 0.776)	0.065 (0.026 - 0.138)	0.061 (0.024 - 0.129)	0.065 (0.026 - 0.135)
All ages (rate per capita)	0.159 (0.126 - 0.200)	0.142 (0.112 - 0.180)	0.149 (0.118 - 0.187)	0.252 (0.177 - 0.343)	0.227 (0.159 - 0.312)	0.236 (0.165 - 0.323)	0.037 (0.016 - 0.076)	0.033 (0.014 - 0.069)	0.035 (0.015 - 0.072)
Age-standardised (rate per capita)	0.080 (0.063 - 0.102)	0.080 (0.063 - 0.102)	0.080 (0.063 - 0.102)	0.130 (0.091 - 0.180)	0.130 (0.091 - 0.180)	0.130 (0.091 - 0.180)	0.019 (0.008 - 0.039)	0.019 (0.008 - 0.039)	0.019 (0.008 - 0.039)
Total number of cases (thousands)	894 (706 - 1,124)	1,134 (891 - 1,433)	1,676 (1,323 - 2,108)	1,416 (994 - 1,928)	1,809 (1,269 - 2,483)	2,656 (1,861 - 3,628)	0 (0 - 0)	0 (0 - 1)	0 (0 - 1)
Comoros									
Under-5 years (rate per capita)	0.406 (0.288 - 0.523)	0.407 (0.289 - 0.525)	0.410 (0.291 - 0.528)	0.556 (0.344 - 0.741)	0.558 (0.345 - 0.743)	0.562 (0.347 - 0.748)	0.094 (0.040 - 0.189)	0.095 (0.040 - 0.191)	0.096 (0.040 - 0.195)
5-14 years (rate per capita)	0.272 (0.187 - 0.370)	0.262 (0.179 - 0.356)	0.264 (0.181 - 0.358)	0.494 (0.291 - 0.767)	0.476 (0.278 - 0.742)	0.479 (0.281 - 0.747)	0.064 (0.026 - 0.135)	0.061 (0.025 - 0.130)	0.062 (0.025 - 0.129)
All ages (rate per capita)	0.151 (0.119 - 0.191)	0.135 (0.106 - 0.171)	0.130 (0.102 - 0.164)	0.241 (0.169 - 0.329)	0.216 (0.152 - 0.296)	0.207 (0.145 - 0.283)	0.035 (0.015 - 0.072)	0.032 (0.014 - 0.064)	0.030 (0.013 - 0.062)
Age-standardised (rate per capita)	0.080 (0.063 - 0.102)	0.080 (0.063 - 0.102)	0.080 (0.063 - 0.102)	0.130 (0.091 - 0.180)	0.130 (0.091 - 0.180)	0.130 (0.091 - 0.180)	0.019 (0.008 - 0.039)	0.019 (0.008 - 0.038)	0.019 (0.008 - 0.039)
Total number of cases (thousands)	63 (49 - 79)	84 (66 - 106)	103 (81 - 130)	100 (70 - 137)	134 (94 - 184)	164 (115 - 224)	0 (0 - 0)	0 (0 - 0)	0 (0 - 0)
Djibouti									
Under-5 years (rate per capita)	0.399 (0.283 - 0.514)	0.410 (0.291 - 0.529)	0.412 (0.292 - 0.531)	0.547 (0.338 - 0.728)	0.562 (0.347 - 0.749)	0.564 (0.349 - 0.752)	0.093 (0.040 - 0.188)	0.095 (0.040 - 0.191)	0.096 (0.041 - 0.193)
5-14 years (rate per capita)	0.257 (0.176 - 0.350)	0.247 (0.168 - 0.337)	0.254 (0.173 - 0.346)	0.467 (0.273 - 0.729)	0.450 (0.262 - 0.702)	0.462 (0.269 - 0.721)	0.060 (0.024 - 0.128)	0.058 (0.023 - 0.123)	0.059 (0.024 - 0.126)
All ages (rate per capita)	0.135 (0.106 - 0.171)	0.115 (0.090 - 0.146)	0.102 (0.080 - 0.130)	0.216 (0.151 - 0.298)	0.185 (0.130 - 0.257)	0.164 (0.115 - 0.227)	0.031 (0.013 - 0.064)	0.027 (0.012 - 0.055)	0.024 (0.010 - 0.049)
Age-standardised (rate per capita)	0.080 (0.063 - 0.102)	0.080 (0.063 - 0.102)	0.080 (0.063 - 0.102)	0.130 (0.091 - 0.180)	0.130 (0.091 - 0.180)	0.130 (0.091 - 0.180)	0.019 (0.008 - 0.039)	0.019 (0.008 - 0.038)	0.019 (0.008 - 0.038)
Total number of cases (thousands)	77 (61 - 98)	90 (71 - 115)	91 (71 - 115)	124 (87 - 171)	145 (102 - 201)	146 (102 - 202)	0 (0 - 0)	0 (0 - 0)	0 (0 - 0)
Eritrea									
Under-5 years (rate per capita)	0.406 (0.287 - 0.522)	0.403 (0.286 - 0.519)	0.412 (0.292 - 0.530)	0.556 (0.344 - 0.740)	0.552 (0.342 - 0.735)	0.564 (0.349 - 0.751)	0.094 (0.040 - 0.184)	0.094 (0.040 - 0.188)	0.096 (0.041 - 0.192)
5-14 years (rate per capita)	0.270 (0.185 - 0.366)	0.253 (0.173 - 0.345)	0.267 (0.184 - 0.363)	0.489 (0.288 - 0.762)	0.462 (0.269 - 0.720)	0.486 (0.285 - 0.757)	0.063 (0.026 - 0.133)	0.059 (0.024 - 0.127)	0.062 (0.025 - 0.135)
All ages (rate per capita)	0.152 (0.119 - 0.192)	0.131 (0.103 - 0.165)	0.138 (0.108 - 0.174)	0.242 (0.170 - 0.331)	0.209 (0.146 - 0.287)	0.221 (0.155 - 0.304)	0.035 (0.015 - 0.072)	0.030 (0.013 - 0.062)	0.032 (0.014 - 0.065)
Age-standardised (rate per capita)	0.080 (0.063 - 0.102)	0.080 (0.063 - 0.102)	0.080 (0.063 - 0.102)	0.130 (0.091 - 0.180)	0.130 (0.091 - 0.180)	0.130 (0.091 - 0.180)	0.019 (0.008 - 0.038)	0.019 (0.008 - 0.039)	0.019 (0.008 - 0.038)
Total number of cases (thousands)	477 (375 - 602)	549 (431 - 694)	722 (567 - 915)	761 (534 - 1,040)	876 (615 - 1,205)	1,158 (812 - 1,592)	0 (0 - 0)	0 (0 - 0)	0 (0 - 0)
Ethiopia									
Under-5 years (rate per capita)	0.398 (0.282 - 0.513)	0.408 (0.289 - 0.525)	0.410 (0.290 - 0.527)	0.545 (0.337 - 0.726)	0.559 (0.345 - 0.744)	0.561 (0.347 - 0.747)	0.092 (0.038 - 0.186)	0.095 (0.041 - 0.192)	0.096 (0.040 - 0.192)
5-14 years (rate per capita)	0.272 (0.187 - 0.369)	0.270 (0.186 - 0.367)	0.255 (0.174 - 0.347)	0.493 (0.290 - 0.766)	0.491 (0.289 - 0.764)	0.464 (0.271 - 0.724)	0.064 (0.026 - 0.135)	0.063 (0.026 - 0.132)	0.060 (0.024 - 0.126)
All ages (rate per capita)	0.149 (0.117 - 0.188)	0.148 (0.117 - 0.188)	0.128 (0.101 - 0.163)	0.237 (0.166 - 0.324)	0.238 (0.167 - 0.326)	0.207 (0.145 - 0.286)	0.035 (0.015 - 0.071)	0.035 (0.015 - 0.071)	0.030 (0.013 - 0.062)
Age-standardised (rate per capita)	0.080 (0.063 - 0.102)	0.080 (0.063 - 0.102)	0.080 (0.063 - 0.102)	0.130 (0.091 - 0.180)	0.130 (0.091 - 0.180)	0.130 (0.091 - 0.180)	0.019 (0.008 - 0.039)	0.019 (0.008 - 0.038)	0.019 (0.008 - 0.039)
Total number of cases (thousands)	7,136 (5,609 - 9,003)	11,340 (8,912 - 14,350)	12,773 (10,018 - 16,223)	11,361 (7,955 - 15,515)	18,172 (12,750 - 24,895)	20,548 (14,414 - 28,438)	2 (1 - 3)	3 (1 - 5)	3 (1 - 6)
Kenya									
Under-5 years (rate per capita)	0.318 (0.243 - 0.411)	0.331 (0.256 - 0.425)	0.328 (0.253 - 0.418)	0.511 (0.286 - 0.733)	0.499 (0.284 - 0.698)	0.514 (0.292 - 0.732)	0.074 (0.032 - 0.152)	0.077 (0.033 - 0.155)	0.077 (0.033 - 0.156)
5-14 years (rate per capita)	0.273 (0.213 - 0.347)	0.269 (0.207 - 0.345)	0.271 (0.213 - 0.344)	0.443 (0.300 - 0.629)	0.429 (0.290 - 0.620)	0.435 (0.295 - 0.616)	0.064 (0.027 - 0.130)	0.063 (0.026 - 0.132)	0.064 (0.026 - 0.131)
All ages (rate per capita)	0.142 (0.117 - 0.170)	0.126 (0.105 - 0.151)	0.123 (0.102 - 0.147)	0.230 (0.167 - 0.311)	0.196 (0.141 - 0.265)	0.196 (0.142 - 0.264)	0.033 (0.014 - 0.066)	0.030 (0.013 - 0.059)	0.029 (0.012 - 0.058)
Age-standardised (rate per capita)	0.073 (0.060 - 0.087)	0.075 (0.062 - 0.090)	0.074 (0.061 - 0.088)	0.118 (0.085 - 0.160)	0.117 (0.084 - 0.159)	0.118 (0.086 - 0.160)	0.017 (0.007 - 0.034)	0.018 (0.007 - 0.035)	0.017 (0.007 - 0.035)
Total number of cases (thousands)	3,335 (2,743 - 3,974)	4,471 (3,710 - 5,350)	5,692 (4,719 - 6,770)	5,389 (3,904 - 7,300)	6,954 (5,007 - 9,391)	9,032 (6,563 - 12,207)	1 (0 - 2)	1 (0 - 2)	1 (1 - 3)
Madagascar									
Under-5 years (rate per capita)	0.466 (0.378 - 0.539)	0.465 (0.378 - 0.536)	0.464 (0.377 - 0.535)	0.529 (0.379 - 0.683)	0.552 (0.399 - 0.699)	0.551 (0.399 - 0.698)	0.108 (0.047 - 0.209)	0.108 (0.048 - 0.210)	0.108 (0.047 - 0.210)
5-14 years (rate per capita)	0.504 (0.453 - 0.548)	0.334 (0.274 - 0.423)	0.325 (0.266 - 0.413)	0.441 (0.313 - 0.601)	0.440 (0.312 - 0.650)	0.429 (0.303 - 0.637)	0.117 (0.051 - 0.224)	0.078 (0.033 - 0.161)	0.076 (0.032 - 0.157)
All ages (rate per capita)	0.221 (0.199 - 0.241)	0.172 (0.147 - 0.200)	0.157 (0.134 - 0.182)	0.215 (0.169 - 0.279)	0.216 (0.166 - 0.281)	0.197 (0.152 - 0.257)	0.051 (0.023 - 0.099)	0.040 (0.017 - 0.080)	0.037 (0.016 - 0.072)
Age-standardised (rate per capita)	0.129 (0.117 - 0.140)	0.096 (0.083 - 0.113)	0.096 (0.083 - 0.113)	0.124 (0.097 - 0.161)	0.122 (0.094 - 0.160)	0.122 (0.094 - 0.160)	0.030 (0.013 - 0.057)	0.022 (0.010 - 0.045)	0.022 (0.010 - 0.045)
Total number of cases (thousands)	2,551 (2,301 - 2,788)	3,140 (2,694 - 3,651)	3,791 (3,249 - 4,415)	2,487 (1,952 - 3,228)	3,947 (3,042 - 5,130)	4,775 (3,684 - 6,221)	1 (0 - 1)	1 (0 - 1)	1 (0 - 2)
Malawi									
Under-5 years (rate per capita)	0.397 (0.281 - 0.511)	0.402 (0.285 - 0.518)	0.407 (0.288 - 0.524)	0.543 (0.336 - 0.724)	0.551 (0.340 - 0.734)	0.557 (0.344 - 0.742)	0.092 (0.039 - 0.181)	0.094 (0.040 - 0.189)	0.095 (0.041 - 0.195)
5-14 years (rate per capita)	0.265 (0.182 - 0.360)	0.266 (0.183 - 0.361)	0.268 (0.184 - 0.364)	0.480 (0.282 - 0.749)	0.483 (0.283 - 0.753)	0.487 (0.286 - 0.759)	0.062 (0.025 - 0.130)	0.062 (0.025 - 0.131)	0.063 (0.026 - 0.137)
All ages (rate per capita)	0.146 (0.115 - 0.184)	0.149 (0.117 - 0.189)	0.145 (0.114 - 0.183)	0.231 (0.162 - 0.316)	0.239 (0.167 - 0.326)	0.232 (0.163 - 0.317)	0.034 (0.015 - 0.069)	0.035 (0.015 - 0.071)	0.034 (0.014 - 0.070)
Age-standardised (rate per capita)	0.080 (0.063 - 0.102)	0.080 (0.063 - 0.102)	0.080 (0.063 - 0.102)	0.130 (0.091 - 0.180)	0.130 (0.091 - 0.180)	0.130 (0.091 - 0.180)	0.019 (0.008 - 0.038)	0.019 (0.008 - 0.038)	0.019 (0.008 - 0.039)
Total number of cases (thousands)	1,368 (1,077 - 1,726)	1,911 (1,502 - 2,413)	2,493 (1,959 - 3,154)	2,176 (1,525 - 2,969)	3,051 (2,140 - 4,170)	3,991 (2,800 - 5,465)	0 (0 - 1)	0 (0 - 1)	1 (0 - 1)
Mozambique									
Under-5 years (rate per capita)	0.400 (0.283 - 0.515)	0.403 (0.285 - 0.519)	0.407 (0.288 - 0.523)	0.548 (0.339 - 0.730)	0.552 (0.341 - 0.735)	0.557 (0.344 - 0.742)	0.093 (0.039 - 0.184)	0.094 (0.040 - 0.189)	0.095 (0.040 - 0.189)
5-14 years (rate per capita)	0.264 (0.181 - 0.359)	0.271 (0.186 - 0.367)	0.265 (0.182 - 0.360)	0.479 (0.281 - 0.748)	0.491 (0.289 - 0.764)	0.481 (0.282 - 0.750)	0.061 (0.025 - 0.129)	0.063 (0.025 - 0.135)	0.062 (0.025 - 0.132)
All ages (rate per capita)	0.151 (0.119 - 0.191)	0.147 (0.116 - 0.186)	0.145 (0.114 - 0.183)	0.241 (0.169 - 0.330)	0.235 (0.165 - 0.320)	0.231 (0.162 - 0.317)	0.035 (0.015 - 0.072)	0.034 (0.015 - 0.070)	0.034 (0.014 - 0.069)
Age-standardised (rate per capita)	0.080 (0.063 - 0.102)	0.080 (0.063 - 0.102)	0.080 (0.063 - 0.102)	0.130 (0.091 - 0.180)	0.130 (0.091 - 0.180)	0.130 (0.091 - 0.180)	0.019 (0.008 - 0.038)	0.019 (0.008 - 0.038)	0.019 (0.008 - 0.038)
Total number of cases (thousands)	2,034 (1,599 - 2,571)	3,114 (2,448 - 3,928)	4,045 (3,179 - 5,117)	3,249 (2,279 - 4,447)	4,959 (3,476 - 6,770)	6,471 (4,539 - 8,868)	0 (0 - 1)	1 (0 - 1)	1 (0 - 2)
Rwanda									
Under-5 years (rate per capita)	0.409 (0.289 - 0.526)	0.403 (0.285 - 0.519)	0.412 (0.292 - 0.530)	0.560 (0.346 - 0.746)	0.552 (0.341 - 0.735)	0.564 (0.349 - 0.752)	0.095 (0.040 - 0.187)	0.094 (0.041 - 0.189)	0.096 (0.041 - 0.195)
5-14 years (rate per capita)	0.274 (0.188 - 0.371)	0.269 (0.185 - 0.365)	0.265 (0.182 - 0.360)	0.496 (0.292 - 0.769)	0.487 (0.287 - 0.760)	0.482 (0.283 - 0.751)	0.064 (0.026 - 0.136)	0.063 (0.025 - 0.135)	0.062 (0.025 - 0.129)

	Prevalence 1990	Prevalence 2005	Prevalence 2015	Incidence 1990	Incidence 2005	Incidence 2015	YLD rate 1990	YLD rate 2005	YLD rate 2015
All ages (rate per capita)	0.163 (0.128 - 0.206)	0.136 (0.107 - 0.172)	0.131 (0.103 - 0.166)	0.260 (0.182 - 0.355)	0.217 (0.152 - 0.297)	0.210 (0.147 - 0.291)	0.038 (0.017 - 0.077)	0.032 (0.014 - 0.066)	0.031 (0.013 - 0.062)
Age-standardised (rate per capita)	0.080 (0.063 - 0.102)	0.080 (0.063 - 0.102)	0.080 (0.063 - 0.102)	0.130 (0.091 - 0.180)	0.130 (0.091 - 0.180)	0.130 (0.091 - 0.180)	0.019 (0.008 - 0.038)	0.019 (0.008 - 0.039)	0.019 (0.008 - 0.038)
Total number of cases (thousands)	1,184 (931 - 1,493)	1,231 (969 - 1,554)	1,522 (1,194 - 1,932)	1,883 (1,317 - 2,574)	1,964 (1,377 - 2,682)	2,448 (1,710 - 3,380)	0 (0 - 1)	0 (0 - 1)	0 (0 - 1)
**Somalia**									
Under-5 years (rate per capita)	0.403 (0.285 - 0.519)	0.403 (0.285 - 0.519)	0.405 (0.287 - 0.522)	0.552 (0.341 - 0.735)	0.552 (0.341 - 0.735)	0.555 (0.343 - 0.739)	0.094 (0.040 - 0.186)	0.094 (0.040 - 0.188)	0.094 (0.040 - 0.189)
5-14 years (rate per capita)	0.271 (0.186 - 0.367)	0.276 (0.190 - 0.374)	0.269 (0.185 - 0.366)	0.491 (0.289 - 0.764)	0.500 (0.295 - 0.775)	0.489 (0.287 - 0.761)	0.063 (0.025 - 0.133)	0.064 (0.026 - 0.134)	0.063 (0.025 - 0.131)
All ages (rate per capita)	0.149 (0.118 - 0.187)	0.157 (0.124 - 0.198)	0.152 (0.119 - 0.192)	0.236 (0.166 - 0.321)	0.250 (0.175 - 0.341)	0.242 (0.170 - 0.330)	0.035 (0.015 - 0.070)	0.037 (0.016 - 0.074)	0.035 (0.015 - 0.071)
Age-standardised (rate per capita)	0.080 (0.063 - 0.102)	0.080 (0.063 - 0.102)	0.080 (0.063 - 0.102)	0.130 (0.091 - 0.180)	0.130 (0.091 - 0.180)	0.130 (0.091 - 0.180)	0.019 (0.008 - 0.038)	0.019 (0.008 - 0.038)	0.019 (0.008 - 0.038)
Total number of cases (thousands)	956 (755 - 1,200)	1,336 (1,051 - 1,684)	1,649 (1,296 - 2,080)	1,509 (1,062 - 2,054)	2,124 (1,485 - 2,904)	2,626 (1,841 - 3,584)	0 (0 - 0)	0 (0 - 1)	0 (0 - 1)
**South Sudan**									
Under-5 years (rate per capita)	0.403 (0.285 - 0.519)	0.402 (0.285 - 0.517)	0.404 (0.286 - 0.520)	0.552 (0.341 - 0.735)	0.550 (0.340 - 0.733)	0.553 (0.342 - 0.736)	0.094 (0.040 - 0.188)	0.093 (0.040 - 0.188)	0.094 (0.040 - 0.190)
5-14 years (rate per capita)	0.266 (0.183 - 0.362)	0.267 (0.183 - 0.363)	0.263 (0.181 - 0.358)	0.483 (0.284 - 0.754)	0.485 (0.285 - 0.756)	0.478 (0.280 - 0.746)	0.062 (0.025 - 0.128)	0.062 (0.025 - 0.134)	0.062 (0.025 - 0.130)
All ages (rate per capita)	0.145 (0.115 - 0.183)	0.141 (0.111 - 0.179)	0.132 (0.104 - 0.167)	0.230 (0.162 - 0.314)	0.226 (0.159 - 0.309)	0.212 (0.148 - 0.292)	0.034 (0.015 - 0.069)	0.033 (0.014 - 0.067)	0.031 (0.013 - 0.063)
Age-standardised (rate per capita)	0.080 (0.063 - 0.102)	0.080 (0.063 - 0.102)	0.080 (0.063 - 0.102)	0.130 (0.091 - 0.180)	0.130 (0.091 - 0.180)	0.130 (0.091 - 0.180)	0.019 (0.008 - 0.039)	0.019 (0.008 - 0.038)	0.019 (0.008 - 0.038)
Total number of cases (thousands)	846 (667 - 1,063)	1,144 (899 - 1,447)	1,620 (1,272 - 2,054)	1,337 (941 - 1,828)	1,831 (1,284 - 2,506)	2,601 (1,822 - 3,584)	0 (0 - 0)	0 (0 - 1)	0 (0 - 1)
**Tanzania**									
Under-5 years (rate per capita)	0.250 (0.179 - 0.320)	0.151 (0.120 - 0.187)	0.190 (0.132 - 0.258)	0.460 (0.230 - 0.723)	0.194 (0.130 - 0.269)	0.342 (0.167 - 0.622)	0.058 (0.024 - 0.117)	0.035 (0.015 - 0.068)	0.044 (0.019 - 0.088)
5-14 years (rate per capita)	0.220 (0.172 - 0.269)	0.176 (0.119 - 0.252)	0.231 (0.162 - 0.313)	0.297 (0.197 - 0.428)	0.251 (0.151 - 0.409)	0.338 (0.204 - 0.510)	0.051 (0.022 - 0.105)	0.041 (0.016 - 0.091)	0.054 (0.022 - 0.116)
All ages (rate per capita)	0.107 (0.086 - 0.124)	0.075 (0.059 - 0.097)	0.097 (0.071 - 0.121)	0.166 (0.109 - 0.233)	0.103 (0.071 - 0.155)	0.153 (0.097 - 0.224)	0.025 (0.011 - 0.049)	0.018 (0.008 - 0.036)	0.023 (0.009 - 0.047)
Age-standardised (rate per capita)	0.058 (0.048 - 0.068)	0.043 (0.033 - 0.056)	0.055 (0.040 - 0.069)	0.090 (0.059 - 0.126)	0.059 (0.040 - 0.089)	0.086 (0.054 - 0.125)	0.014 (0.006 - 0.027)	0.010 (0.004 - 0.021)	0.013 (0.005 - 0.027)
Total number of cases (thousands)	2,712 (2,195 - 3,160)	2,933 (2,307 - 3,800)	5,184 (3,811 - 6,472)	4,237 (2,764 - 5,944)	4,024 (2,781 - 6,072)	8,164 (5,180 - 11,973)	1 (0 - 1)	1 (0 - 1)	1 (1 - 3)
**Uganda**									
Under-5 years (rate per capita)	0.304 (0.237 - 0.368)	0.256 (0.224 - 0.293)	0.310 (0.242 - 0.376)	0.527 (0.290 - 0.744)	0.305 (0.264 - 0.359)	0.539 (0.296 - 0.760)	0.071 (0.030 - 0.139)	0.060 (0.026 - 0.114)	0.073 (0.032 - 0.143)
5-14 years (rate per capita)	0.301 (0.226 - 0.385)	0.261 (0.197 - 0.332)	0.298 (0.224 - 0.382)	0.458 (0.303 - 0.650)	0.411 (0.257 - 0.643)	0.453 (0.299 - 0.643)	0.070 (0.030 - 0.148)	0.061 (0.025 - 0.128)	0.070 (0.030 - 0.148)
All ages (rate per capita)	0.145 (0.121 - 0.171)	0.128 (0.107 - 0.150)	0.146 (0.121 - 0.172)	0.234 (0.169 - 0.315)	0.182 (0.136 - 0.250)	0.235 (0.168 - 0.316)	0.034 (0.015 - 0.069)	0.030 (0.013 - 0.059)	0.034 (0.015 - 0.069)
Age-standardised (rate per capita)	0.076 (0.063 - 0.091)	0.065 (0.054 - 0.077)	0.076 (0.063 - 0.091)	0.123 (0.088 - 0.166)	0.094 (0.069 - 0.132)	0.123 (0.088 - 0.166)	0.018 (0.008 - 0.036)	0.015 (0.007 - 0.030)	0.018 (0.008 - 0.036)
Total number of cases (thousands)	2,533 (2,111 - 2,984)	3,600 (3,002 - 4,220)	5,718 (4,753 - 6,734)	4,079 (2,937 - 5,488)	5,120 (3,815 - 7,033)	9,198 (6,594 - 12,377)	1 (0 - 1)	1 (0 - 2)	1 (1 - 3)
**Zambia**									
Under-5 years (rate per capita)	0.400 (0.283 - 0.515)	0.402 (0.285 - 0.518)	0.407 (0.288 - 0.524)	0.547 (0.338 - 0.729)	0.551 (0.340 - 0.734)	0.557 (0.344 - 0.742)	0.093 (0.040 - 0.187)	0.094 (0.041 - 0.190)	0.095 (0.040 - 0.193)
5-14 years (rate per capita)	0.265 (0.182 - 0.361)	0.267 (0.183 - 0.363)	0.268 (0.184 - 0.364)	0.482 (0.283 - 0.752)	0.485 (0.285 - 0.756)	0.486 (0.286 - 0.758)	0.062 (0.025 - 0.131)	0.062 (0.026 - 0.131)	0.063 (0.026 - 0.134)
All ages (rate per capita)	0.151 (0.119 - 0.191)	0.150 (0.118 - 0.189)	0.148 (0.116 - 0.187)	0.241 (0.169 - 0.328)	0.239 (0.167 - 0.326)	0.236 (0.166 - 0.323)	0.035 (0.015 - 0.071)	0.035 (0.015 - 0.072)	0.035 (0.015 - 0.071)
Age-standardised (rate per capita)	0.080 (0.063 - 0.102)	0.080 (0.063 - 0.102)	0.080 (0.063 - 0.102)	0.130 (0.091 - 0.180)	0.130 (0.091 - 0.180)	0.130 (0.091 - 0.180)	0.019 (0.008 - 0.038)	0.019 (0.008 - 0.039)	0.019 (0.008 - 0.038)
Total number of cases (thousands)	1,229 (966 - 1,550)	1,807 (1,420 - 2,280)	2,405 (1,891 - 3,038)	1,958 (1,373 - 2,671)	2,879 (2,018 - 3,927)	3,843 (2,695 - 5,253)	0 (0 - 1)	0 (0 - 1)	1 (0 - 1)
**Central Sub-Saharan Africa**									
Under-5 years (rate per capita)	0.347 (0.250 - 0.468)	0.350 (0.252 - 0.472)	0.353 (0.254 - 0.475)	0.509 (0.304 - 0.707)	0.513 (0.307 - 0.712)	0.517 (0.309 - 0.718)	0.081 (0.033 - 0.167)	0.081 (0.034 - 0.166)	0.082 (0.034 - 0.170)
5-14 years (rate per capita)	0.243 (0.172 - 0.328)	0.242 (0.171 - 0.326)	0.241 (0.170 - 0.325)	0.479 (0.299 - 0.710)	0.476 (0.297 - 0.707)	0.474 (0.295 - 0.703)	0.057 (0.024 - 0.119)	0.056 (0.023 - 0.118)	0.056 (0.024 - 0.117)
All ages (rate per capita)	0.132 (0.104 - 0.163)	0.133 (0.104 - 0.164)	0.131 (0.102 - 0.161)	0.227 (0.162 - 0.311)	0.229 (0.163 - 0.314)	0.225 (0.160 - 0.310)	0.031 (0.013 - 0.064)	0.031 (0.013 - 0.064)	0.030 (0.013 - 0.063)
Age-standardised (rate per capita)	0.071 (0.056 - 0.088)	0.071 (0.056 - 0.088)	0.071 (0.056 - 0.088)	0.124 (0.088 - 0.171)	0.124 (0.088 - 0.171)	0.124 (0.088 - 0.171)	0.017 (0.007 - 0.034)	0.017 (0.007 - 0.034)	0.017 (0.007 - 0.034)
Total number of cases (thousands)	7,020 (5,514 - 8,646)	11,182 (8,765 - 13,775)	15,013 (11,749 - 18,526)	12,024 (8,571 - 16,495)	19,192 (13,661 - 26,333)	25,862 (18,392 - 35,545)	2 (1 - 3)	3 (1 - 5)	3 (2 - 7)
**Angola**									
Under-5 years (rate per capita)	0.346 (0.249 - 0.467)	0.349 (0.251 - 0.470)	0.353 (0.254 - 0.475)	0.507 (0.303 - 0.705)	0.511 (0.305 - 0.709)	0.517 (0.309 - 0.717)	0.081 (0.034 - 0.162)	0.082 (0.034 - 0.169)	0.083 (0.035 - 0.171)
5-14 years (rate per capita)	0.243 (0.172 - 0.328)	0.244 (0.173 - 0.330)	0.242 (0.172 - 0.327)	0.478 (0.299 - 0.710)	0.481 (0.301 - 0.713)	0.477 (0.298 - 0.708)	0.057 (0.024 - 0.120)	0.057 (0.023 - 0.119)	0.056 (0.023 - 0.120)
All ages (rate per capita)	0.139 (0.110 - 0.171)	0.140 (0.110 - 0.172)	0.138 (0.108 - 0.170)	0.238 (0.170 - 0.326)	0.239 (0.170 - 0.328)	0.237 (0.169 - 0.326)	0.032 (0.014 - 0.066)	0.033 (0.014 - 0.068)	0.032 (0.014 - 0.067)
Age-standardised (rate per capita)	0.071 (0.056 - 0.088)	0.071 (0.056 - 0.088)	0.071 (0.056 - 0.088)	0.124 (0.088 - 0.171)	0.124 (0.088 - 0.171)	0.124 (0.088 - 0.171)	0.017 (0.007 - 0.035)	0.017 (0.007 - 0.035)	0.017 (0.007 - 0.035)
Total number of cases (thousands)	1,567 (1,235 - 1,929)	2,520 (1,981 - 3,103)	3,490 (2,735 - 4,301)	2,675 (1,911 - 3,670)	4,312 (3,076 - 5,914)	5,992 (4,265 - 8,222)	0 (0 - 1)	1 (0 - 1)	1 (0 - 2)
**Central African Republic**									
Under-5 years (rate per capita)	0.350 (0.252 - 0.472)	0.353 (0.254 - 0.476)	0.353 (0.254 - 0.476)	0.513 (0.307 - 0.712)	0.518 (0.309 - 0.719)	0.518 (0.310 - 0.719)	0.081 (0.032 - 0.165)	0.082 (0.034 - 0.169)	0.082 (0.033 - 0.168)
5-14 years (rate per capita)	0.241 (0.171 - 0.326)	0.237 (0.168 - 0.320)	0.230 (0.162 - 0.310)	0.474 (0.296 - 0.704)	0.466 (0.290 - 0.692)	0.453 (0.281 - 0.676)	0.056 (0.023 - 0.115)	0.055 (0.023 - 0.117)	0.054 (0.022 - 0.113)
All ages (rate per capita)	0.122 (0.096 - 0.151)	0.118 (0.092 - 0.145)	0.108 (0.084 - 0.133)	0.211 (0.150 - 0.290)	0.203 (0.145 - 0.280)	0.186 (0.133 - 0.256)	0.029 (0.012 - 0.059)	0.027 (0.012 - 0.057)	0.025 (0.011 - 0.052)
Age-standardised (rate per capita)	0.071 (0.056 - 0.088)	0.071 (0.056 - 0.088)	0.071 (0.056 - 0.088)	0.124 (0.088 - 0.171)	0.124 (0.088 - 0.171)	0.124 (0.088 - 0.171)	0.017 (0.007 - 0.034)	0.017 (0.007 - 0.034)	0.017 (0.007 - 0.034)
Total number of cases (thousands)	359 (282 - 444)	478 (375 - 591)	528 (414 - 654)	620 (441 - 852)	826 (589 - 1,136)	914 (652 - 1,257)	0 (0 - 0)	0 (0 - 0)	0 (0 - 0)
**Congo**									
Under-5 years (rate per capita)	0.353 (0.254 - 0.476)	0.352 (0.253 - 0.474)	0.356 (0.256 - 0.479)	0.518 (0.309 - 0.719)	0.515 (0.308 - 0.716)	0.521 (0.312 - 0.724)	0.082 (0.034 - 0.166)	0.082 (0.033 - 0.166)	0.083 (0.034 - 0.168)
5-14 years (rate per capita)	0.236 (0.167 - 0.318)	0.240 (0.170 - 0.324)	0.241 (0.170 - 0.325)	0.465 (0.289 - 0.690)	0.473 (0.295 - 0.701)	0.474 (0.296 - 0.703)	0.055 (0.022 - 0.117)	0.056 (0.023 - 0.118)	0.056 (0.024 - 0.119)
All ages (rate per capita)	0.123 (0.097 - 0.152)	0.120 (0.094 - 0.148)	0.122 (0.096 - 0.151)	0.213 (0.152 - 0.293)	0.207 (0.147 - 0.284)	0.210 (0.150 - 0.289)	0.029 (0.012 - 0.059)	0.028 (0.012 - 0.058)	0.028 (0.012 - 0.059)
Age-standardised (rate per capita)	0.071 (0.056 - 0.088)	0.071 (0.056 - 0.088)	0.071 (0.056 - 0.088)	0.124 (0.088 - 0.171)	0.124 (0.088 - 0.171)	0.124 (0.088 - 0.171)	0.017 (0.007 - 0.035)	0.017 (0.007 - 0.034)	0.017 (0.007 - 0.035)
Total number of cases (thousands)	296 (231 - 365)	424 (332 - 522)	564 (442 - 697)	510 (363 - 702)	729 (518 - 1,001)	974 (693 - 1,338)	0 (0 - 0)	0 (0 - 0)	0 (0 - 0)
**Democratic Republic of the Congo**									
Under-5 years (rate per capita)	0.347 (0.250 - 0.468)	0.350 (0.252 - 0.472)	0.353 (0.254 - 0.475)	0.509 (0.304 - 0.706)	0.513 (0.307 - 0.712)	0.517 (0.309 - 0.717)	0.081 (0.033 - 0.166)	0.081 (0.034 - 0.163)	0.082 (0.035 - 0.168)
5-14 years (rate per capita)	0.244 (0.173 - 0.329)	0.242 (0.171 - 0.326)	0.241 (0.170 - 0.325)	0.480 (0.300 - 0.712)	0.476 (0.297 - 0.706)	0.474 (0.296 - 0.703)	0.057 (0.024 - 0.120)	0.056 (0.023 - 0.117)	0.056 (0.023 - 0.118)
All ages (rate per capita)	0.132 (0.104 - 0.163)	0.134 (0.105 - 0.165)	0.131 (0.103 - 0.162)	0.226 (0.161 - 0.310)	0.230 (0.164 - 0.315)	0.226 (0.161 - 0.311)	0.031 (0.013 - 0.064)	0.031 (0.013 - 0.064)	0.031 (0.013 - 0.063)
Age-standardised (rate per capita)	0.071 (0.056 - 0.088)	0.071 (0.056 - 0.088)	0.071 (0.056 - 0.088)	0.124 (0.088 - 0.171)	0.124 (0.088 - 0.171)	0.124 (0.088 - 0.171)	0.017 (0.007 - 0.034)	0.017 (0.007 - 0.034)	0.017 (0.007 - 0.034)
Total number of cases (thousands)	4,641 (3,646 - 5,716)	7,538 (5,908 - 9,285)	10,156 (7,952 - 12,540)	7,949 (5,667 - 10,905)	12,939 (9,209 - 17,754)	17,508 (12,456 - 24,060)	1 (0 - 2)	2 (1 - 4)	2 (1 - 5)
**Equatorial Guinea**									
Under-5 years (rate per capita)	0.345 (0.249 - 0.466)	0.350 (0.252 - 0.472)	0.353 (0.254 - 0.476)	0.506 (0.303 - 0.703)	0.513 (0.307 - 0.713)	0.517 (0.309 - 0.718)	0.080 (0.033 - 0.163)	0.081 (0.034 - 0.162)	0.082 (0.035 - 0.166)
5-14 years (rate per capita)	0.267 (0.191 - 0.358)	0.236 (0.167 - 0.319)	0.240 (0.170 - 0.324)	0.524 (0.331 - 0.763)	0.466 (0.290 - 0.691)	0.472 (0.294 - 0.700)	0.062 (0.025 - 0.130)	0.055 (0.023 - 0.117)	0.056 (0.023 - 0.119)
All ages (rate per capita)	0.116 (0.092 - 0.142)	0.115 (0.090 - 0.142)	0.111 (0.087 - 0.137)	0.195 (0.141 - 0.265)	0.199 (0.142 - 0.274)	0.192 (0.137 - 0.264)	0.027 (0.012 - 0.055)	0.027 (0.012 - 0.055)	0.026 (0.011 - 0.053)
Age-standardised (rate per capita)	0.071 (0.056 - 0.088)	0.071 (0.056 - 0.088)	0.071 (0.056 - 0.088)	0.124 (0.088 - 0.171)	0.124 (0.088 - 0.171)	0.124 (0.088 - 0.171)	0.017 (0.007 - 0.034)	0.017 (0.007 - 0.034)	0.017 (0.007 - 0.034)
Total number of cases (thousands)	44 (35 - 54)	72 (56 - 89)	94 (74 - 116)	74 (53 - 100)	125 (89 - 171)	162 (116 - 223)	0 (0 - 0)	0 (0 - 0)	0 (0 - 0)
**Gabon**									
Under-5 years (rate per capita)	0.353 (0.255 - 0.476)	0.355 (0.256 - 0.479)	0.357 (0.257 - 0.481)	0.518 (0.310 - 0.719)	0.521 (0.311 - 0.723)	0.523 (0.313 - 0.726)	0.082 (0.034 - 0.170)	0.083 (0.034 - 0.169)	0.083 (0.034 - 0.169)
5-14 years (rate per capita)	0.242 (0.172 - 0.327)	0.233 (0.165 - 0.314)	0.235 (0.166 - 0.317)	0.477 (0.298 - 0.708)	0.458 (0.285 - 0.683)	0.463 (0.288 - 0.689)	0.056 (0.023 - 0.118)	0.054 (0.022 - 0.113)	0.055 (0.022 - 0.115)

	Prevalence 1990	Prevalence 2005	Prevalence 2015	Incidence 1990	Incidence 2005	Incidence 2015	YLD rate 1990	YLD rate 2005	YLD rate 2015
**All ages (rate per capita)**	0.119<br>(0.093 - 0.147)	0.109<br>(0.086 - 0.135)	0.104<br>(0.082 - 0.129)	0.205<br>(0.146 - 0.282)	0.190<br>(0.135 - 0.261)	0.181<br>(0.129 - 0.248)	0.028<br>(0.012 - 0.057)	0.025<br>(0.011 - 0.053)	0.024<br>(0.010 - 0.050)
**Age-standardised (rate per capita)**	0.071<br>(0.056 - 0.088)	0.071<br>(0.056 - 0.088)	0.071<br>(0.056 - 0.088)	0.124<br>(0.088 - 0.171)	0.124<br>(0.088 - 0.171)	0.124<br>(0.088 - 0.171)	0.017<br>(0.007 - 0.034)	0.017<br>(0.007 - 0.034)	0.017<br>(0.007 - 0.034)
**Total number of cases (thousands)**	113<br>(89 - 140)	151<br>(119 - 187)	180<br>(141 - 223)	195<br>(139 - 269)	262<br>(187 - 360)	312<br>(222 - 428)	0<br>(0 - 0)	0<br>(0 - 0)	0<br>(0 - 0)

Appendix 2, Table 3. Prevalence, incidence, and YLDs per 1,000 population (with 95% uncertainty intervals) for untreated caries in permanent teeth, 1990 and 2015, by country and by age

	Prevalence 1990	Prevalence 2005	Prevalence 2015	Incidence 1990	Incidence 2005	Incidence 2015	YLD rate 1990	YLD rate 2005	YLD rate 2015
**Global**									
5 -14 years (rate per capita)	0.266 (0.216 - 0.322)	0.260 (0.212 - 0.316)	0.263 (0.211 - 0.322)	0.359 (0.281 - 0.460)	0.350 (0.273 - 0.449)	0.347 (0.268 - 0.451)	0.183 (0.080 - 0.349)	0.176 (0.077 - 0.338)	0.177 (0.078 - 0.343)
15 - 49 years (rate per capita)	0.415 (0.382 - 0.449)	0.412 (0.380 - 0.445)	0.415 (0.382 - 0.449)	0.744 (0.676 - 0.814)	0.737 (0.670 - 0.804)	0.741 (0.671 - 0.807)	0.295 (0.129 - 0.567)	0.286 (0.124 - 0.554)	0.285 (0.125 - 0.550)
50 - 69 years (rate per capita)	0.385 (0.345 - 0.427)	0.380 (0.341 - 0.424)	0.378 (0.339 - 0.423)	0.806 (0.718 - 0.891)	0.791 (0.707 - 0.883)	0.790 (0.704 - 0.881)	0.284 (0.125 - 0.543)	0.273 (0.122 - 0.524)	0.268 (0.119 - 0.515)
70+ years (rate per capita)	0.359 (0.327 - 0.394)	0.357 (0.326 - 0.391)	0.351 (0.321 - 0.384)	0.771 (0.692 - 0.849)	0.759 (0.683 - 0.835)	0.751 (0.677 - 0.825)	0.270 (0.122 - 0.508)	0.262 (0.119 - 0.494)	0.256 (0.116 - 0.481)
All ages (rate per capita)	0.328 (0.306 - 0.348)	0.338 (0.316 - 0.358)	0.342 (0.320 - 0.364)	0.583 (0.545 - 0.623)	0.604 (0.565 - 0.643)	0.616 (0.577 - 0.656)	0.234 (0.104 - 0.445)	0.236 (0.105 - 0.450)	0.236 (0.105 - 0.450)
Age-standardized (rate per capita)	0.343 (0.322 - 0.362)	0.339 (0.318 - 0.358)	0.341 (0.320 - 0.362)	0.627 (0.589 - 0.665)	0.616 (0.578 - 0.654)	0.616 (0.577 - 0.656)	0.246 (0.109 - 0.468)	0.237 (0.106 - 0.453)	0.236 (0.105 - 0.451)
Total number of cases (thousands)	1,739,354 (1,623,031 - 1,844,514)	2,201,369 (2,062,260 - 2,332,640)	2,521,198 (2,361,418 - 2,679,669)	3,093,652 (2,887,820 - 3,306,063)	3,938,102 (3,683,008 - 4,194,842)	4,540,874 (4,250,707 - 4,836,656)	1,239 (551 - 2,361)	1,536 (682 - 2,933)	1,743 (777 - 3,315)
**High-income**									
5 -14 years (rate per capita)	0.212 (0.175 - 0.257)	0.196 (0.158 - 0.243)	0.205 (0.161 - 0.259)	0.323 (0.256 - 0.429)	0.311 (0.240 - 0.426)	0.324 (0.247 - 0.450)	0.211 (0.094 - 0.406)	0.195 (0.086 - 0.380)	0.204 (0.089 - 0.401)
15 - 49 years (rate per capita)	0.410 (0.380 - 0.440)	0.406 (0.378 - 0.434)	0.410 (0.381 - 0.439)	0.795 (0.733 - 0.858)	0.793 (0.732 - 0.854)	0.798 (0.736 - 0.861)	0.405 (0.176 - 0.761)	0.401 (0.173 - 0.755)	0.405 (0.175 - 0.766)
50 - 69 years (rate per capita)	0.384 (0.350 - 0.422)	0.377 (0.343 - 0.414)	0.376 (0.340 - 0.414)	0.878 (0.795 - 0.959)	0.867 (0.783 - 0.950)	0.866 (0.784 - 0.951)	0.371 (0.159 - 0.708)	0.364 (0.155 - 0.690)	0.363 (0.155 - 0.690)
70+ years (rate per capita)	0.346 (0.320 - 0.376)	0.343 (0.317 - 0.371)	0.337 (0.312 - 0.365)	0.812 (0.738 - 0.885)	0.803 (0.732 - 0.874)	0.795 (0.725 - 0.866)	0.321 (0.143 - 0.597)	0.317 (0.142 - 0.594)	0.311 (0.137 - 0.579)
All ages (rate per capita)	0.344 (0.327 - 0.362)	0.343 (0.326 - 0.360)	0.347 (0.329 - 0.364)	0.694 (0.656 - 0.731)	0.705 (0.669 - 0.742)	0.718 (0.680 - 0.756)	0.337 (0.148 - 0.635)	0.335 (0.148 - 0.630)	0.338 (0.149 - 0.639)
Age-standardized (rate per capita)	0.328 (0.311 - 0.345)	0.321 (0.304 - 0.337)	0.325 (0.307 - 0.342)	0.654 (0.618 - 0.691)	0.645 (0.610 - 0.680)	0.649 (0.611 - 0.686)	0.322 (0.141 - 0.609)	0.315 (0.139 - 0.597)	0.318 (0.140 - 0.605)
Total number of cases (thousands)	309,095 (293,778 - 324,534)	341,777 (324,319 - 358,230)	371,336 (352,542 - 389,805)	622,763 (588,829 - 655,956)	702,206 (666,538 - 739,280)	768,058 (728,049 - 809,346)	302 (133 - 570)	334 (147 - 628)	362 (160 - 683)
**High-income North America**									
5 -14 years (rate per capita)	0.158 (0.121 - 0.212)	0.143 (0.107 - 0.195)	0.147 (0.112 - 0.200)	0.277 (0.205 - 0.411)	0.271 (0.197 - 0.421)	0.281 (0.207 - 0.429)	0.157 (0.067 - 0.313)	0.142 (0.061 - 0.279)	0.147 (0.062 - 0.289)
15 - 49 years (rate per capita)	0.338 (0.310 - 0.368)	0.319 (0.293 - 0.346)	0.330 (0.303 - 0.358)	0.786 (0.717 - 0.858)	0.756 (0.689 - 0.825)	0.771 (0.704 - 0.840)	0.334 (0.144 - 0.631)	0.314 (0.138 - 0.599)	0.326 (0.141 - 0.621)
50 - 69 years (rate per capita)	0.312 (0.280 - 0.350)	0.308 (0.273 - 0.346)	0.314 (0.278 - 0.353)	0.790 (0.703 - 0.878)	0.780 (0.690 - 0.878)	0.790 (0.701 - 0.887)	0.300 (0.130 - 0.572)	0.296 (0.127 - 0.561)	0.301 (0.130 - 0.581)
70+ years (rate per capita)	0.272 (0.247 - 0.301)	0.270 (0.246 - 0.296)	0.267 (0.244 - 0.294)	0.699 (0.620 - 0.782)	0.688 (0.615 - 0.765)	0.683 (0.615 - 0.760)	0.250 (0.112 - 0.471)	0.247 (0.111 - 0.468)	0.243 (0.109 - 0.456)
All ages (rate per capita)	0.277 (0.260 - 0.294)	0.267 (0.250 - 0.283)	0.277 (0.260 - 0.293)	0.648 (0.606 - 0.689)	0.638 (0.597 - 0.680)	0.659 (0.618 - 0.702)	0.271 (0.118 - 0.513)	0.260 (0.115 - 0.494)	0.269 (0.118 - 0.514)
Age-standardized (rate per capita)	0.264 (0.248 - 0.280)	0.252 (0.237 - 0.268)	0.261 (0.244 - 0.277)	0.614 (0.573 - 0.655)	0.599 (0.560 - 0.641)	0.612 (0.571 - 0.656)	0.258 (0.113 - 0.491)	0.247 (0.109 - 0.472)	0.255 (0.111 - 0.488)
Total number of cases (thousands)	76,952 (72,214 - 81,778)	87,526 (81,989 - 92,798)	99,596 (93,424 - 105,494)	179,952 (168,166 - 191,463)	209,353 (196,003 - 223,054)	237,108 (222,333 - 252,652)	75 (33 - 142)	85 (38 - 162)	97 (42 - 185)
**Canada**									
5 -14 years (rate per capita)	0.147 (0.109 - 0.201)	0.151 (0.112 - 0.206)	0.148 (0.110 - 0.202)	0.266 (0.195 - 0.422)	0.272 (0.199 - 0.426)	0.267 (0.196 - 0.423)	0.147 (0.061 - 0.289)	0.151 (0.064 - 0.294)	0.148 (0.063 - 0.294)
15 - 49 years (rate per capita)	0.332 (0.303 - 0.362)	0.326 (0.299 - 0.356)	0.326 (0.299 - 0.356)	0.763 (0.693 - 0.838)	0.755 (0.686 - 0.827)	0.753 (0.687 - 0.825)	0.329 (0.142 - 0.622)	0.322 (0.140 - 0.610)	0.323 (0.140 - 0.610)
50 - 69 years (rate per capita)	0.304 (0.269 - 0.342)	0.305 (0.270 - 0.344)	0.305 (0.269 - 0.343)	0.770 (0.680 - 0.870)	0.771 (0.679 - 0.872)	0.771 (0.679 - 0.870)	0.293 (0.127 - 0.555)	0.295 (0.125 - 0.560)	0.295 (0.127 - 0.556)
70+ years (rate per capita)	0.269 (0.242 - 0.299)	0.265 (0.239 - 0.293)	0.261 (0.237 - 0.287)	0.681 (0.600 - 0.761)	0.670 (0.593 - 0.749)	0.662 (0.587 - 0.740)	0.248 (0.111 - 0.477)	0.244 (0.109 - 0.464)	0.240 (0.107 - 0.463)
All ages (rate per capita)	0.275 (0.257 - 0.292)	0.277 (0.259 - 0.295)	0.279 (0.261 - 0.296)	0.638 (0.594 - 0.684)	0.652 (0.610 - 0.694)	0.661 (0.617 - 0.706)	0.269 (0.117 - 0.513)	0.271 (0.119 - 0.513)	0.272 (0.119 - 0.517)
Age-standardized (rate per capita)	0.256 (0.239 - 0.273)	0.256 (0.238 - 0.272)	0.256 (0.238 - 0.272)	0.593 (0.552 - 0.636)	0.593 (0.551 - 0.635)	0.592 (0.551 - 0.635)	0.251 (0.109 - 0.478)	0.251 (0.110 - 0.474)	0.251 (0.110 - 0.475)
Total number of cases (thousands)	7,584 (7,091 - 8,074)	8,930 (8,354 - 9,500)	10,089 (9,434 - 10,716)	17,604 (16,399 - 18,888)	20,986 (19,634 - 22,361)	23,893 (22,308 - 25,503)	7 (3 - 14)	9 (4 - 17)	10 (4 - 19)
**Greenland**									
5 -14 years (rate per capita)	0.142 (0.106 - 0.194)	0.150 (0.112 - 0.205)	0.148 (0.110 - 0.203)	0.255 (0.187 - 0.409)	0.267 (0.197 - 0.419)	0.264 (0.194 - 0.414)	0.086 (0.035 - 0.176)	0.090 (0.037 - 0.184)	0.089 (0.037 - 0.185)
15 - 49 years (rate per capita)	0.330 (0.300 - 0.361)	0.323 (0.297 - 0.352)	0.322 (0.295 - 0.351)	0.734 (0.668 - 0.809)	0.725 (0.662 - 0.793)	0.720 (0.657 - 0.786)	0.196 (0.086 - 0.387)	0.191 (0.084 - 0.385)	0.191 (0.084 - 0.378)
50 - 69 years (rate per capita)	0.305 (0.268 - 0.345)	0.303 (0.266 - 0.344)	0.304 (0.267 - 0.344)	0.748 (0.658 - 0.846)	0.744 (0.654 - 0.842)	0.744 (0.654 - 0.842)	0.176 (0.075 - 0.340)	0.175 (0.075 - 0.344)	0.176 (0.075 - 0.342)
70+ years (rate per capita)	0.280 (0.246 - 0.319)	0.281 (0.246 - 0.322)	0.280 (0.244 - 0.321)	0.689 (0.595 - 0.777)	0.691 (0.593 - 0.784)	0.688 (0.589 - 0.786)	0.154 (0.069 - 0.295)	0.155 (0.070 - 0.295)	0.154 (0.069 - 0.300)
All ages (rate per capita)	0.236 (0.219 - 0.254)	0.240 (0.223 - 0.256)	0.246 (0.230 - 0.263)	0.525 (0.486 - 0.568)	0.538 (0.501 - 0.578)	0.559 (0.521 - 0.599)	0.139 (0.060 - 0.270)	0.141 (0.060 - 0.270)	0.145 (0.063 - 0.278)
Age-standardized (rate per capita)	0.255 (0.237 - 0.271)	0.255 (0.237 - 0.271)	0.255 (0.237 - 0.271)	0.574 (0.534 - 0.616)	0.573 (0.534 - 0.615)	0.573 (0.533 - 0.615)	0.149 (0.064 - 0.288)	0.149 (0.064 - 0.287)	0.150 (0.065 - 0.286)
Total number of cases (thousands)	13 (12 - 14)	13 (12 - 14)	13 (12 - 14)	29 (27 - 32)	29 (27 - 32)	30 (28 - 32)	0 (0 - 0)	0 (0 - 0)	0 (0 - 0)
**United States**									
5 -14 years (rate per capita)	0.159 (0.122 - 0.212)	0.142 (0.106 - 0.194)	0.147 (0.112 - 0.200)	0.278 (0.206 - 0.409)	0.271 (0.196 - 0.421)	0.283 (0.208 - 0.430)	0.159 (0.068 - 0.316)	0.142 (0.060 - 0.278)	0.147 (0.063 - 0.289)
15 - 49 years (rate per capita)	0.339 (0.311 - 0.369)	0.318 (0.292 - 0.345)	0.330 (0.303 - 0.359)	0.789 (0.719 - 0.862)	0.756 (0.688 - 0.825)	0.773 (0.705 - 0.843)	0.334 (0.145 - 0.632)	0.313 (0.138 - 0.596)	0.326 (0.141 - 0.623)
50 - 69 years (rate per capita)	0.313 (0.281 - 0.350)	0.308 (0.274 - 0.347)	0.315 (0.280 - 0.354)	0.792 (0.703 - 0.880)	0.781 (0.690 - 0.877)	0.792 (0.703 - 0.892)	0.301 (0.130 - 0.574)	0.296 (0.127 - 0.561)	0.302 (0.130 - 0.581)
70+ years (rate per capita)	0.272 (0.247 - 0.301)	0.270 (0.246 - 0.297)	0.267 (0.244 - 0.295)	0.700 (0.622 - 0.785)	0.690 (0.618 - 0.768)	0.685 (0.616 - 0.763)	0.250 (0.112 - 0.473)	0.247 (0.111 - 0.469)	0.243 (0.109 - 0.459)
All ages (rate per capita)	0.277 (0.260 - 0.295)	0.266 (0.249 - 0.281)	0.277 (0.259 - 0.293)	0.649 (0.606 - 0.691)	0.636 (0.595 - 0.678)	0.659 (0.617 - 0.702)	0.271 (0.118 - 0.513)	0.259 (0.114 - 0.492)	0.269 (0.118 - 0.514)
Age-standardized (rate per capita)	0.265 (0.248 - 0.281)	0.252 (0.236 - 0.268)	0.261 (0.244 - 0.278)	0.616 (0.575 - 0.657)	0.600 (0.560 - 0.642)	0.614 (0.573 - 0.658)	0.259 (0.113 - 0.492)	0.246 (0.108 - 0.471)	0.255 (0.112 - 0.489)
Total number of cases (thousands)	69,321 (65,050 - 73,690)	78,549 (73,595 - 83,246)	89,459 (83,943 - 94,669)	162,241 (151,435 - 172,636)	188,256 (176,032 - 200,559)	213,102 (199,568 - 227,068)	68 (30 - 128)	77 (34 - 145)	87 (38 - 166)
**Australasia**									
5 -14 years (rate per capita)	0.081 (0.060 - 0.113)	0.075 (0.062 - 0.090)	0.082 (0.060 - 0.114)	0.121 (0.087 - 0.185)	0.110 (0.086 - 0.138)	0.122 (0.087 - 0.187)	0.080 (0.034 - 0.160)	0.075 (0.032 - 0.145)	0.081 (0.034 - 0.160)
15 - 49 years (rate per capita)	0.240 (0.218 - 0.264)	0.245 (0.224 - 0.267)	0.239 (0.216 - 0.262)	0.400 (0.357 - 0.441)	0.408 (0.368 - 0.446)	0.402 (0.361 - 0.443)	0.237 (0.102 - 0.445)	0.242 (0.105 - 0.457)	0.236 (0.103 - 0.447)
50 - 69 years (rate per capita)	0.201 (0.177 - 0.229)	0.210 (0.185 - 0.235)	0.201 (0.176 - 0.229)	0.418 (0.368 - 0.478)	0.425 (0.371 - 0.490)	0.418 (0.369 - 0.478)	0.194 (0.083 - 0.371)	0.203 (0.087 - 0.385)	0.194 (0.084 - 0.377)
70+ years (rate per capita)	0.176 (0.157 - 0.198)	0.177 (0.159 - 0.196)	0.169 (0.152 - 0.189)	0.361 (0.317 - 0.408)	0.358 (0.319 - 0.401)	0.349 (0.310 - 0.392)	0.164 (0.072 - 0.318)	0.164 (0.073 - 0.312)	0.156 (0.070 - 0.303)
All ages (rate per capita)	0.188 (0.175 - 0.202)	0.193 (0.180 - 0.204)	0.190 (0.177 - 0.203)	0.330 (0.306 - 0.356)	0.340 (0.317 - 0.364)	0.342 (0.318 - 0.367)	0.184 (0.080 - 0.345)	0.189 (0.083 - 0.355)	0.186 (0.082 - 0.350)
Age-standardized (rate per capita)	0.179 (0.166 - 0.192)	0.182 (0.170 - 0.193)	0.179 (0.166 - 0.192)	0.315 (0.292 - 0.339)	0.317 (0.293 - 0.339)	0.315 (0.292 - 0.339)	0.175 (0.077 - 0.330)	0.179 (0.078 - 0.334)	0.175 (0.077 - 0.331)
Total number of cases (thousands)	3,829 (3,562 - 4,119)	4,729 (4,413 - 5,013)	5,487 (5,114 - 5,877)	6,726 (6,239 - 7,259)	8,346 (7,778 - 8,931)	9,885 (9,189 - 10,613)	4 (2 - 7)	5 (2 - 9)	5 (2 - 10)
**Australia**									
5 -14 years (rate per capita)	0.081 (0.060 - 0.114)	0.077 (0.063 - 0.093)	0.082 (0.060 - 0.114)	0.121 (0.086 - 0.185)	0.113 (0.088 - 0.142)	0.122 (0.087 - 0.186)	0.081 (0.034 - 0.161)	0.077 (0.033 - 0.149)	0.082 (0.034 - 0.162)
15 - 49 years (rate per capita)	0.241 (0.217 - 0.264)	0.247 (0.226 - 0.268)	0.240 (0.217 - 0.264)	0.401 (0.359 - 0.443)	0.409 (0.370 - 0.447)	0.403 (0.361 - 0.445)	0.238 (0.102 - 0.445)	0.244 (0.105 - 0.457)	0.237 (0.103 - 0.451)
50 - 69 years (rate per capita)	0.201 (0.177 - 0.230)	0.212 (0.185 - 0.236)	0.202 (0.176 - 0.231)	0.418 (0.368 - 0.478)	0.426 (0.371 - 0.494)	0.418 (0.368 - 0.479)	0.194 (0.083 - 0.373)	0.205 (0.088 - 0.388)	0.195 (0.084 - 0.377)
70+ years (rate per capita)	0.177 (0.156 - 0.199)	0.178 (0.160 - 0.197)	0.170 (0.152 - 0.189)	0.362 (0.315 - 0.409)	0.359 (0.319 - 0.403)	0.349 (0.309 - 0.393)	0.164 (0.073 - 0.317)	0.165 (0.074 - 0.315)	0.157 (0.070 - 0.303)
All ages (rate per capita)	0.188 (0.175 - 0.203)	0.195 (0.182 - 0.207)	0.191 (0.178 - 0.205)	0.331 (0.306 - 0.357)	0.343 (0.319 - 0.367)	0.343 (0.319 - 0.368)	0.185 (0.080 - 0.347)	0.191 (0.083 - 0.358)	0.187 (0.083 - 0.353)
Age-standardized (rate per capita)	0.179 (0.166 - 0.193)	0.184 (0.171 - 0.195)	0.179 (0.166 - 0.193)	0.315 (0.292 - 0.340)	0.318 (0.294 - 0.341)	0.315 (0.292 - 0.340)	0.176 (0.077 - 0.331)	0.180 (0.078 - 0.337)	0.176 (0.077 - 0.332)
Total number of cases (thousands)	3,207 (2,982 - 3,450)	3,973 (3,706 - 4,212)	4,644 (4,330 - 4,980)	5,633 (5,218 - 6,084)	6,990 (6,512 - 7,483)	8,350 (7,761 - 8,962)	3 (1 - 6)	4 (2 - 7)	5 (2 - 9)
**New Zealand**									
5 -14 years (rate per capita)	0.079 (0.059 - 0.107)	0.063 (0.050 - 0.079)	0.081 (0.059 - 0.114)	0.120 (0.087 - 0.184)	0.098 (0.074 - 0.127)	0.121 (0.086 - 0.196)	0.078 (0.033 - 0.154)	0.063 (0.027 - 0.123)	0.080 (0.033 - 0.166)
15 - 49 years (rate per capita)	0.239 (0.216 - 0.264)	0.239 (0.215 - 0.262)	0.233 (0.211 - 0.256)	0.397 (0.357 - 0.439)	0.401 (0.362 - 0.442)	0.397 (0.358 - 0.437)	0.236 (0.102 - 0.447)	0.236 (0.103 - 0.453)	0.230 (0.099 - 0.432)
50 - 69 years (rate per capita)	0.200 (0.175 - 0.227)	0.199 (0.174 - 0.229)	0.200 (0.174 - 0.228)	0.416 (0.369 - 0.476)	0.416 (0.363 - 0.480)	0.416 (0.366 - 0.476)	0.193 (0.083 - 0.360)	0.193 (0.082 - 0.373)	0.193 (0.082 - 0.371)

	Prevalence 1990	Prevalence 2005	Prevalence 2015	Incidence 1990	Incidence 2005	Incidence 2015	YLD rate 1990	YLD rate 2005	YLD rate 2015
70+ years (rate per capita)	0.175 (0.157 - 0.196)	0.170 (0.153 - 0.191)	0.168 (0.151 - 0.187)	0.359 (0.313 - 0.408)	0.352 (0.312 - 0.395)	0.348 (0.308 - 0.394)	0.163 (0.073 - 0.314)	0.158 (0.071 - 0.302)	0.155 (0.069 - 0.298)
All ages (rate per capita)	0.184 (0.171 - 0.198)	0.183 (0.169 - 0.196)	0.185 (0.171 - 0.198)	0.324 (0.300 - 0.350)	0.328 (0.304 - 0.351)	0.336 (0.313 - 0.362)	0.181 (0.079 - 0.343)	0.179 (0.078 - 0.341)	0.181 (0.079 - 0.338)
Age-standardized (rate per capita)	0.178 (0.165 - 0.191)	0.175 (0.163 - 0.188)	0.178 (0.165 - 0.192)	0.314 (0.291 - 0.340)	0.311 (0.287 - 0.333)	0.314 (0.291 - 0.339)	0.175 (0.077 - 0.331)	0.172 (0.076 - 0.328)	0.175 (0.075 - 0.330)
Total number of cases (thousands)	622 (577 - 669)	756 (701 - 811)	844 (781 - 903)	1,093 (1,013 - 1,181)	1,356 (1,257 - 1,452)	1,535 (1,427 - 1,651)	1 (0 - 1)	1 (0 - 1)	1 (0 - 2)
**High-income Asia Pacific**									
5 -14 years (rate per capita)	0.130 (0.097 - 0.177)	0.120 (0.098 - 0.153)	0.130 (0.097 - 0.177)	0.288 (0.209 - 0.438)	0.267 (0.209 - 0.376)	0.288 (0.209 - 0.438)	0.130 (0.055 - 0.255)	0.120 (0.052 - 0.231)	0.130 (0.055 - 0.255)
15 - 49 years (rate per capita)	0.345 (0.316 - 0.374)	0.355 (0.324 - 0.386)	0.356 (0.326 - 0.386)	0.799 (0.727 - 0.873)	0.820 (0.749 - 0.895)	0.827 (0.752 - 0.903)	0.342 (0.149 - 0.651)	0.352 (0.152 - 0.669)	0.353 (0.153 - 0.676)
50 - 69 years (rate per capita)	0.331 (0.293 - 0.373)	0.330 (0.293 - 0.371)	0.328 (0.292 - 0.369)	0.836 (0.738 - 0.943)	0.834 (0.732 - 0.941)	0.833 (0.737 - 0.937)	0.322 (0.136 - 0.617)	0.321 (0.136 - 0.616)	0.319 (0.136 - 0.615)
70+ years (rate per capita)	0.285 (0.258 - 0.316)	0.282 (0.256 - 0.310)	0.277 (0.253 - 0.305)	0.730 (0.650 - 0.812)	0.723 (0.644 - 0.802)	0.712 (0.639 - 0.790)	0.266 (0.119 - 0.504)	0.263 (0.117 - 0.503)	0.258 (0.115 - 0.496)
All ages (rate per capita)	0.286 (0.268 - 0.304)	0.299 (0.282 - 0.315)	0.301 (0.283 - 0.318)	0.680 (0.634 - 0.724)	0.716 (0.671 - 0.760)	0.727 (0.682 - 0.772)	0.282 (0.123 - 0.532)	0.293 (0.129 - 0.554)	0.294 (0.129 - 0.557)
Age-standardized (rate per capita)	0.270 (0.252 - 0.287)	0.268 (0.251 - 0.284)	0.270 (0.252 - 0.286)	0.639 (0.596 - 0.683)	0.636 (0.593 - 0.677)	0.638 (0.595 - 0.682)	0.266 (0.116 - 0.505)	0.264 (0.115 - 0.500)	0.265 (0.116 - 0.504)
Total number of cases (thousands)	48,370 (45,241 - 51,280)	52,948 (49,941 - 55,876)	55,063 (51,857 - 58,093)	114,768 (107,127 - 122,351)	126,940 (118,984 - 134,734)	133,033 (124,707 - 141,140)	48 (21 - 90)	52 (23 - 98)	54 (24 - 102)
**Brunei**									
5 -14 years (rate per capita)	0.124 (0.092 - 0.171)	0.127 (0.094 - 0.175)	0.130 (0.096 - 0.178)	0.265 (0.193 - 0.412)	0.270 (0.196 - 0.416)	0.275 (0.201 - 0.419)	0.074 (0.030 - 0.150)	0.076 (0.031 - 0.154)	0.078 (0.032 - 0.153)
15 - 49 years (rate per capita)	0.348 (0.315 - 0.379)	0.349 (0.317 - 0.381)	0.349 (0.319 - 0.382)	0.758 (0.689 - 0.835)	0.764 (0.696 - 0.839)	0.768 (0.699 - 0.842)	0.207 (0.091 - 0.408)	0.207 (0.091 - 0.417)	0.208 (0.090 - 0.414)
50 - 69 years (rate per capita)	0.326 (0.287 - 0.370)	0.327 (0.287 - 0.372)	0.327 (0.287 - 0.371)	0.796 (0.707 - 0.903)	0.796 (0.702 - 0.906)	0.798 (0.707 - 0.907)	0.190 (0.081 - 0.369)	0.191 (0.081 - 0.370)	0.191 (0.081 - 0.377)
70+ years (rate per capita)	0.279 (0.251 - 0.311)	0.282 (0.253 - 0.314)	0.282 (0.254 - 0.316)	0.693 (0.615 - 0.778)	0.699 (0.619 - 0.786)	0.701 (0.621 - 0.788)	0.155 (0.070 - 0.294)	0.157 (0.070 - 0.298)	0.157 (0.071 - 0.301)
All ages (rate per capita)	0.248 (0.228 - 0.268)	0.271 (0.251 - 0.291)	0.283 (0.264 - 0.303)	0.546 (0.502 - 0.595)	0.602 (0.557 - 0.649)	0.635 (0.589 - 0.679)	0.147 (0.064 - 0.283)	0.161 (0.069 - 0.311)	0.168 (0.073 - 0.323)
Age-standardized (rate per capita)	0.268 (0.250 - 0.285)	0.268 (0.250 - 0.285)	0.268 (0.250 - 0.285)	0.607 (0.565 - 0.649)	0.608 (0.566 - 0.649)	0.608 (0.566 - 0.649)	0.158 (0.068 - 0.305)	0.158 (0.068 - 0.301)	0.158 (0.069 - 0.301)
Total number of cases (thousands)	65 (60 - 70)	99 (91 - 106)	120 (112 - 128)	143 (131 - 155)	219 (203 - 236)	269 (249 - 287)	0 (0 - 0)	0 (0 - 0)	0 (0 - 0)
**Japan**									
5 -14 years (rate per capita)	0.131 (0.098 - 0.180)	0.128 (0.096 - 0.176)	0.130 (0.097 - 0.179)	0.294 (0.213 - 0.446)	0.288 (0.208 - 0.444)	0.292 (0.212 - 0.445)	0.131 (0.055 - 0.254)	0.128 (0.054 - 0.250)	0.130 (0.055 - 0.253)
15 - 49 years (rate per capita)	0.347 (0.317 - 0.376)	0.357 (0.326 - 0.389)	0.360 (0.329 - 0.390)	0.809 (0.734 - 0.883)	0.830 (0.755 - 0.906)	0.840 (0.762 - 0.918)	0.344 (0.150 - 0.655)	0.355 (0.154 - 0.678)	0.357 (0.155 - 0.684)
50 - 69 years (rate per capita)	0.332 (0.293 - 0.374)	0.330 (0.292 - 0.371)	0.329 (0.291 - 0.370)	0.839 (0.739 - 0.948)	0.838 (0.738 - 0.945)	0.837 (0.738 - 0.946)	0.323 (0.137 - 0.619)	0.322 (0.136 - 0.619)	0.320 (0.137 - 0.618)
70+ years (rate per capita)	0.285 (0.257 - 0.316)	0.281 (0.255 - 0.310)	0.276 (0.252 - 0.303)	0.730 (0.647 - 0.812)	0.723 (0.643 - 0.802)	0.712 (0.636 - 0.790)	0.266 (0.119 - 0.506)	0.263 (0.117 - 0.503)	0.258 (0.115 - 0.493)
All ages (rate per capita)	0.291 (0.273 - 0.308)	0.301 (0.284 - 0.319)	0.301 (0.284 - 0.318)	0.698 (0.651 - 0.743)	0.728 (0.681 - 0.773)	0.733 (0.686 - 0.780)	0.286 (0.125 - 0.543)	0.295 (0.130 - 0.558)	0.294 (0.129 - 0.557)
Age-standardized (rate per capita)	0.271 (0.253 - 0.287)	0.270 (0.253 - 0.287)	0.270 (0.253 - 0.287)	0.644 (0.600 - 0.688)	0.643 (0.599 - 0.688)	0.643 (0.599 - 0.688)	0.266 (0.116 - 0.504)	0.266 (0.116 - 0.504)	0.267 (0.116 - 0.505)
Total number of cases (thousands)	35,757 (33,491 - 37,842)	37,991 (35,760 - 40,150)	38,630 (36,386 - 40,801)	85,699 (79,944 - 91,265)	91,821 (85,831 - 97,481)	94,071 (87,958 - 100,033)	35 (15 - 67)	37 (16 - 70)	38 (17 - 71)
**Singapore**									
5 -14 years (rate per capita)	0.126 (0.094 - 0.174)	0.129 (0.095 - 0.178)	0.131 (0.097 - 0.180)	0.272 (0.198 - 0.419)	0.277 (0.202 - 0.423)	0.281 (0.204 - 0.425)	0.126 (0.054 - 0.246)	0.129 (0.055 - 0.250)	0.131 (0.056 - 0.256)
15 - 49 years (rate per capita)	0.354 (0.321 - 0.386)	0.355 (0.323 - 0.387)	0.351 (0.320 - 0.383)	0.802 (0.727 - 0.880)	0.812 (0.739 - 0.888)	0.804 (0.732 - 0.879)	0.351 (0.151 - 0.663)	0.352 (0.153 - 0.674)	0.349 (0.151 - 0.668)
50 - 69 years (rate per capita)	0.326 (0.288 - 0.368)	0.327 (0.288 - 0.371)	0.327 (0.288 - 0.371)	0.823 (0.730 - 0.933)	0.822 (0.727 - 0.935)	0.825 (0.729 - 0.940)	0.318 (0.137 - 0.611)	0.320 (0.136 - 0.615)	0.320 (0.138 - 0.621)
70+ years (rate per capita)	0.284 (0.255 - 0.317)	0.279 (0.252 - 0.309)	0.274 (0.249 - 0.303)	0.722 (0.640 - 0.816)	0.711 (0.632 - 0.797)	0.701 (0.626 - 0.783)	0.266 (0.119 - 0.505)	0.262 (0.116 - 0.499)	0.258 (0.114 - 0.486)
All ages (rate per capita)	0.285 (0.264 - 0.305)	0.293 (0.274 - 0.313)	0.298 (0.279 - 0.317)	0.657 (0.610 - 0.706)	0.687 (0.638 - 0.732)	0.704 (0.656 - 0.750)	0.281 (0.122 - 0.533)	0.289 (0.126 - 0.554)	0.294 (0.128 - 0.556)
Age-standardized (rate per capita)	0.268 (0.251 - 0.286)	0.268 (0.251 - 0.286)	0.269 (0.251 - 0.286)	0.628 (0.585 - 0.671)	0.628 (0.585 - 0.671)	0.628 (0.585 - 0.671)	0.264 (0.115 - 0.503)	0.265 (0.116 - 0.508)	0.265 (0.115 - 0.506)
Total number of cases (thousands)	780 (724 - 837)	1,043 (974 - 1,113)	1,169 (1,094 - 1,244)	1,800 (1,673 - 1,935)	2,443 (2,270 - 2,605)	2,761 (2,574 - 2,944)	1 (0 - 1)	1 (0 - 2)	1 (1 - 2)
**South Korea**									
5 -14 years (rate per capita)	0.128 (0.094 - 0.174)	0.105 (0.097 - 0.112)	0.129 (0.095 - 0.175)	0.276 (0.200 - 0.423)	0.226 (0.198 - 0.258)	0.278 (0.202 - 0.424)	0.128 (0.054 - 0.255)	0.105 (0.046 - 0.203)	0.129 (0.055 - 0.256)
15 - 49 years (rate per capita)	0.342 (0.311 - 0.373)	0.350 (0.321 - 0.381)	0.350 (0.320 - 0.381)	0.775 (0.700 - 0.850)	0.802 (0.730 - 0.879)	0.803 (0.730 - 0.880)	0.338 (0.148 - 0.636)	0.347 (0.152 - 0.663)	0.347 (0.151 - 0.667)
50 - 69 years (rate per capita)	0.328 (0.288 - 0.370)	0.326 (0.289 - 0.367)	0.326 (0.287 - 0.368)	0.824 (0.728 - 0.941)	0.822 (0.722 - 0.931)	0.823 (0.727 - 0.940)	0.318 (0.136 - 0.608)	0.317 (0.136 - 0.607)	0.317 (0.135 - 0.615)
70+ years (rate per capita)	0.288 (0.257 - 0.322)	0.285 (0.256 - 0.317)	0.281 (0.253 - 0.312)	0.731 (0.645 - 0.830)	0.725 (0.638 - 0.819)	0.716 (0.635 - 0.807)	0.268 (0.119 - 0.512)	0.266 (0.121 - 0.503)	0.262 (0.116 - 0.504)
All ages (rate per capita)	0.273 (0.254 - 0.293)	0.292 (0.274 - 0.310)	0.301 (0.282 - 0.320)	0.630 (0.583 - 0.681)	0.685 (0.640 - 0.730)	0.715 (0.670 - 0.763)	0.269 (0.118 - 0.517)	0.287 (0.125 - 0.543)	0.296 (0.128 - 0.564)
Age-standardized (rate per capita)	0.268 (0.251 - 0.287)	0.263 (0.248 - 0.280)	0.268 (0.250 - 0.286)	0.629 (0.587 - 0.673)	0.619 (0.577 - 0.658)	0.628 (0.586 - 0.672)	0.263 (0.115 - 0.504)	0.259 (0.113 - 0.490)	0.264 (0.115 - 0.507)
Total number of cases (thousands)	11,769 (10,931 - 12,621)	13,815 (12,967 - 14,700)	15,144 (14,178 - 16,101)	27,127 (25,106 - 29,344)	32,457 (30,307 - 34,576)	35,932 (33,709 - 38,342)	12 (5 - 22)	14 (6 - 26)	15 (6 - 28)
**Western Europe**									
5 -14 years (rate per capita)	0.291 (0.254 - 0.327)	0.267 (0.222 - 0.317)	0.281 (0.227 - 0.339)	0.388 (0.325 - 0.458)	0.376 (0.296 - 0.476)	0.392 (0.302 - 0.507)	0.290 (0.129 - 0.559)	0.266 (0.117 - 0.515)	0.280 (0.124 - 0.549)
15 - 49 years (rate per capita)	0.491 (0.458 - 0.524)	0.494 (0.463 - 0.528)	0.494 (0.463 - 0.527)	0.829 (0.769 - 0.886)	0.843 (0.784 - 0.899)	0.844 (0.786 - 0.903)	0.485 (0.210 - 0.920)	0.488 (0.212 - 0.926)	0.488 (0.214 - 0.925)
50 - 69 years (rate per capita)	0.448 (0.410 - 0.488)	0.449 (0.411 - 0.488)	0.451 (0.414 - 0.491)	0.962 (0.876 - 1.040)	0.965 (0.880 - 1.043)	0.967 (0.880 - 1.046)	0.432 (0.188 - 0.823)	0.434 (0.188 - 0.829)	0.436 (0.190 - 0.832)
70+ years (rate per capita)	0.412 (0.383 - 0.443)	0.412 (0.384 - 0.443)	0.405 (0.378 - 0.435)	0.921 (0.844 - 0.993)	0.921 (0.851 - 0.989)	0.913 (0.846 - 0.983)	0.381 (0.170 - 0.712)	0.382 (0.169 - 0.714)	0.375 (0.165 - 0.698)
All ages (rate per capita)	0.420 (0.401 - 0.438)	0.423 (0.404 - 0.441)	0.424 (0.405 - 0.442)	0.762 (0.726 - 0.797)	0.784 (0.747 - 0.820)	0.795 (0.755 - 0.830)	0.410 (0.181 - 0.775)	0.412 (0.181 - 0.779)	0.413 (0.182 - 0.782)
Age-standardized (rate per capita)	0.398 (0.379 - 0.415)	0.395 (0.376 - 0.413)	0.398 (0.378 - 0.418)	0.705 (0.671 - 0.740)	0.706 (0.670 - 0.742)	0.708 (0.672 - 0.746)	0.390 (0.171 - 0.732)	0.388 (0.170 - 0.731)	0.391 (0.171 - 0.736)
Total number of cases (thousands)	160,120 (152,841 - 167,010)	172,116 (164,573 - 179,581)	183,708 (175,730 - 191,584)	290,737 (276,924 - 303,986)	319,368 (304,046 - 333,763)	344,585 (327,428 - 360,119)	156 (69 - 295)	168 (74 - 317)	179 (79 - 339)
**Andorra**									
5 -14 years (rate per capita)	0.253 (0.180 - 0.348)	0.253 (0.180 - 0.348)	0.250 (0.178 - 0.344)	0.415 (0.258 - 0.582)	0.415 (0.258 - 0.582)	0.410 (0.257 - 0.576)	0.152 (0.061 - 0.313)	0.152 (0.061 - 0.306)	0.150 (0.061 - 0.301)
15 - 49 years (rate per capita)	0.468 (0.402 - 0.534)	0.470 (0.403 - 0.534)	0.470 (0.403 - 0.536)	0.805 (0.697 - 0.920)	0.812 (0.705 - 0.924)	0.814 (0.704 - 0.927)	0.278 (0.120 - 0.551)	0.279 (0.122 - 0.547)	0.278 (0.120 - 0.548)
50 - 69 years (rate per capita)	0.448 (0.369 - 0.527)	0.448 (0.369 - 0.525)	0.448 (0.368 - 0.528)	0.909 (0.737 - 1.077)	0.909 (0.737 - 1.080)	0.909 (0.739 - 1.080)	0.260 (0.112 - 0.526)	0.260 (0.112 - 0.527)	0.261 (0.111 - 0.529)
70+ years (rate per capita)	0.414 (0.348 - 0.484)	0.412 (0.346 - 0.483)	0.407 (0.343 - 0.473)	0.885 (0.730 - 1.033)	0.885 (0.731 - 1.031)	0.879 (0.730 - 1.020)	0.230 (0.106 - 0.437)	0.229 (0.104 - 0.446)	0.225 (0.103 - 0.428)
All ages (rate per capita)	0.404 (0.364 - 0.445)	0.409 (0.371 - 0.449)	0.410 (0.371 - 0.450)	0.739 (0.668 - 0.812)	0.756 (0.684 - 0.828)	0.766 (0.694 - 0.840)	0.237 (0.104 - 0.463)	0.240 (0.105 - 0.467)	0.239 (0.105 - 0.464)
Age-standardized (rate per capita)	0.381 (0.342 - 0.421)	0.381 (0.342 - 0.421)	0.380 (0.342 - 0.421)	0.687 (0.619 - 0.756)	0.687 (0.619 - 0.756)	0.687 (0.619 - 0.756)	0.224 (0.098 - 0.436)	0.224 (0.098 - 0.435)	0.224 (0.097 - 0.434)
Total number of cases (thousands)	20 (18 - 22)	30 (27 - 32)	33 (30 - 36)	37 (34 - 41)	55 (49 - 60)	61 (55 - 67)	0 (0 - 0)	0 (0 - 0)	0 (0 - 0)
**Austria**									
5 -14 years (rate per capita)	0.247 (0.176 - 0.341)	0.257 (0.182 - 0.354)	0.250 (0.178 - 0.344)	0.418 (0.261 - 0.590)	0.434 (0.268 - 0.611)	0.423 (0.263 - 0.597)	0.247 (0.103 - 0.496)	0.257 (0.107 - 0.523)	0.250 (0.105 - 0.503)
15 - 49 years (rate per capita)	0.469 (0.403 - 0.536)	0.470 (0.403 - 0.534)	0.468 (0.403 - 0.535)	0.851 (0.729 - 0.970)	0.859 (0.734 - 0.974)	0.860 (0.738 - 0.973)	0.463 (0.196 - 0.874)	0.464 (0.197 - 0.879)	0.462 (0.197 - 0.876)
50 - 69 years (rate per capita)	0.449 (0.370 - 0.528)	0.448 (0.369 - 0.527)	0.448 (0.368 - 0.526)	0.956 (0.785 - 1.125)	0.956 (0.783 - 1.127)	0.953 (0.778 - 1.129)	0.432 (0.184 - 0.830)	0.433 (0.184 - 0.827)	0.434 (0.184 - 0.838)
70+ years (rate per capita)	0.414 (0.347 - 0.484)	0.412 (0.347 - 0.482)	0.409 (0.343 - 0.475)	0.924 (0.765 - 1.072)	0.923 (0.768 - 1.069)	0.923 (0.773 - 1.068)	0.382 (0.173 - 0.743)	0.380 (0.171 - 0.729)	0.378 (0.168 - 0.726)
All ages (rate per capita)	0.407 (0.367 - 0.448)	0.413 (0.374 - 0.451)	0.413 (0.374 - 0.454)	0.781 (0.704 - 0.859)	0.800 (0.723 - 0.877)	0.812 (0.734 - 0.888)	0.397 (0.173 - 0.754)	0.402 (0.176 - 0.764)	0.403 (0.174 - 0.760)
Age-standardized (rate per capita)	0.381 (0.342 - 0.421)	0.381 (0.342 - 0.421)	0.381 (0.342 - 0.421)	0.722 (0.650 - 0.793)	0.722 (0.649 - 0.793)	0.722 (0.649 - 0.792)	0.373 (0.164 - 0.711)	0.373 (0.163 - 0.709)	0.373 (0.164 - 0.711)
Total number of cases (thousands)	3,117 (2,812 - 3,434)	3,393 (3,075 - 3,712)	3,584 (3,241 - 3,938)	5,989 (5,399 - 6,586)	6,579 (5,945 - 7,212)	7,040 (6,367 - 7,702)	3 (1 - 6)	3 (1 - 6)	3 (2 - 7)
**Belgium**									
5 -14 years (rate per capita)	0.132 (0.101 - 0.175)	0.131 (0.096 - 0.182)	0.134 (0.099 - 0.187)	0.170 (0.125 - 0.244)	0.160 (0.116 - 0.238)	0.172 (0.125 - 0.251)	0.132 (0.056 - 0.263)	0.131 (0.055 - 0.264)	0.134 (0.058 - 0.274)
15 - 49 years (rate per capita)	0.293 (0.262 - 0.325)	0.290 (0.259 - 0.322)	0.290 (0.259 - 0.322)	0.478 (0.428 - 0.529)	0.482 (0.433 - 0.533)	0.481 (0.432 - 0.532)	0.289 (0.124 - 0.554)	0.286 (0.123 - 0.550)	0.287 (0.123 - 0.546)

	Prevalence 1990	Prevalence 2005	Prevalence 2015	Incidence 1990	Incidence 2005	Incidence 2015	YLD rate 1990	YLD rate 2005	YLD rate 2015
**50 - 69 years (rate per capita)**	0.258 (0.223 - 0.297)	0.258 (0.223 - 0.299)	0.259 (0.222 - 0.301)	0.550 (0.482 - 0.627)	0.550 (0.481 - 0.631)	0.550 (0.479 - 0.635)	0.249 (0.106 - 0.486)	0.249 (0.107 - 0.487)	0.250 (0.107 - 0.485)
**70+ years (rate per capita)**	0.232 (0.205 - 0.261)	0.232 (0.205 - 0.263)	0.228 (0.204 - 0.255)	0.500 (0.437 - 0.562)	0.501 (0.437 - 0.567)	0.492 (0.437 - 0.554)	0.213 (0.095 - 0.400)	0.213 (0.095 - 0.404)	0.209 (0.094 - 0.393)
**All ages (rate per capita)**	0.242 (0.224 - 0.261)	0.241 (0.223 - 0.262)	0.241 (0.222 - 0.260)	0.430 (0.399 - 0.462)	0.436 (0.405 - 0.468)	0.439 (0.408 - 0.472)	0.237 (0.104 - 0.449)	0.235 (0.102 - 0.449)	0.235 (0.102 - 0.444)
**Age-standardized (rate per capita)**	0.226 (0.209 - 0.244)	0.225 (0.207 - 0.244)	0.227 (0.208 - 0.246)	0.392 (0.364 - 0.423)	0.390 (0.362 - 0.420)	0.393 (0.364 - 0.425)	0.222 (0.097 - 0.420)	0.221 (0.096 - 0.420)	0.222 (0.097 - 0.424)
**Total number of cases (thousands)**	2,411 (2,232 - 2,594)	2,527 (2,342 - 2,744)	2,729 (2,516 - 2,943)	4,281 (3,973 - 4,601)	4,567 (4,247 - 4,910)	4,980 (4,624 - 5,353)	2 (1 - 4)	2 (1 - 5)	3 (1 - 5)
**Cyprus**									
**5 -14 years (rate per capita)**	0.253 (0.180 - 0.348)	0.261 (0.185 - 0.357)	0.251 (0.179 - 0.346)	0.415 (0.259 - 0.582)	0.426 (0.264 - 0.596)	0.412 (0.257 - 0.579)	0.152 (0.060 - 0.301)	0.157 (0.062 - 0.316)	0.151 (0.061 - 0.302)
**15 - 49 years (rate per capita)**	0.465 (0.402 - 0.530)	0.467 (0.402 - 0.533)	0.470 (0.404 - 0.537)	0.800 (0.688 - 0.917)	0.805 (0.695 - 0.918)	0.805 (0.695 - 0.920)	0.275 (0.119 - 0.545)	0.277 (0.120 - 0.550)	0.279 (0.120 - 0.552)
**50 - 69 years (rate per capita)**	0.448 (0.367 - 0.527)	0.448 (0.368 - 0.525)	0.448 (0.367 - 0.526)	0.907 (0.736 - 1.074)	0.908 (0.737 - 1.075)	0.908 (0.737 - 1.077)	0.260 (0.112 - 0.524)	0.259 (0.112 - 0.524)	0.260 (0.113 - 0.528)
**70+ years (rate per capita)**	0.411 (0.344 - 0.480)	0.411 (0.345 - 0.480)	0.410 (0.343 - 0.479)	0.885 (0.735 - 1.031)	0.884 (0.731 - 1.028)	0.883 (0.732 - 1.026)	0.228 (0.102 - 0.436)	0.227 (0.104 - 0.432)	0.226 (0.103 - 0.440)
**All ages (rate per capita)**	0.381 (0.342 - 0.422)	0.402 (0.362 - 0.443)	0.418 (0.376 - 0.460)	0.687 (0.618 - 0.756)	0.732 (0.661 - 0.803)	0.762 (0.688 - 0.837)	0.224 (0.099 - 0.432)	0.235 (0.103 - 0.458)	0.245 (0.106 - 0.479)
**Age-standardized (rate per capita)**	0.381 (0.342 - 0.421)	0.381 (0.342 - 0.421)	0.381 (0.342 - 0.421)	0.686 (0.619 - 0.756)	0.686 (0.619 - 0.756)	0.686 (0.619 - 0.755)	0.224 (0.098 - 0.432)	0.224 (0.098 - 0.434)	0.224 (0.098 - 0.433)
**Total number of cases (thousands)**	255 (228 - 282)	302 (273 - 333)	373 (336 - 410)	459 (413 - 505)	551 (498 - 604)	680 (614 - 746)	0 (0 - 0)	0 (0 - 0)	0 (0 - 0)
**Denmark**									
**5 -14 years (rate per capita)**	0.260 (0.184 - 0.356)	0.251 (0.179 - 0.346)	0.251 (0.178 - 0.346)	0.438 (0.269 - 0.617)	0.425 (0.264 - 0.600)	0.425 (0.264 - 0.599)	0.260 (0.107 - 0.525)	0.251 (0.105 - 0.511)	0.251 (0.105 - 0.506)
**15 - 49 years (rate per capita)**	0.468 (0.401 - 0.534)	0.471 (0.404 - 0.535)	0.466 (0.402 - 0.530)	0.853 (0.732 - 0.968)	0.858 (0.735 - 0.974)	0.854 (0.731 - 0.967)	0.462 (0.196 - 0.871)	0.465 (0.196 - 0.875)	0.460 (0.196 - 0.872)
**50 - 69 years (rate per capita)**	0.448 (0.369 - 0.527)	0.448 (0.368 - 0.529)	0.447 (0.371 - 0.528)	0.956 (0.782 - 1.125)	0.955 (0.777 - 1.130)	0.957 (0.788 - 1.124)	0.432 (0.184 - 0.836)	0.433 (0.184 - 0.839)	0.432 (0.184 - 0.830)
**70+ years (rate per capita)**	0.414 (0.347 - 0.486)	0.410 (0.345 - 0.478)	0.412 (0.345 - 0.482)	0.927 (0.769 - 1.077)	0.922 (0.769 - 1.066)	0.926 (0.770 - 1.075)	0.383 (0.173 - 0.742)	0.378 (0.170 - 0.728)	0.381 (0.170 - 0.735)
**All ages (rate per capita)**	0.408 (0.369 - 0.449)	0.403 (0.365 - 0.442)	0.406 (0.368 - 0.446)	0.787 (0.710 - 0.863)	0.782 (0.707 - 0.856)	0.795 (0.717 - 0.875)	0.398 (0.173 - 0.752)	0.393 (0.172 - 0.740)	0.396 (0.173 - 0.743)
**Age-standardized (rate per capita)**	0.380 (0.342 - 0.421)	0.380 (0.342 - 0.421)	0.380 (0.342 - 0.421)	0.722 (0.649 - 0.793)	0.722 (0.649 - 0.793)	0.722 (0.649 - 0.792)	0.373 (0.162 - 0.707)	0.373 (0.162 - 0.707)	0.373 (0.164 - 0.710)
**Total number of cases (thousands)**	2,097 (1,894 - 2,306)	2,181 (1,977 - 2,394)	2,318 (2,101 - 2,549)	4,041 (3,646 - 4,435)	4,236 (3,828 - 4,638)	4,542 (4,094 - 4,999)	2 (1 - 4)	2 (1 - 4)	2 (1 - 4)
**Finland**									
**5 -14 years (rate per capita)**	0.290 (0.233 - 0.363)	0.298 (0.240 - 0.373)	0.288 (0.230 - 0.359)	0.454 (0.346 - 0.586)	0.466 (0.353 - 0.600)	0.449 (0.343 - 0.582)	0.290 (0.126 - 0.571)	0.298 (0.131 - 0.594)	0.287 (0.126 - 0.572)
**15 - 49 years (rate per capita)**	0.519 (0.486 - 0.554)	0.514 (0.482 - 0.547)	0.516 (0.483 - 0.551)	1.003 (0.940 - 1.063)	1.006 (0.943 - 1.066)	0.999 (0.938 - 1.060)	0.513 (0.225 - 0.978)	0.508 (0.220 - 0.969)	0.510 (0.224 - 0.960)
**50 - 69 years (rate per capita)**	0.467 (0.432 - 0.506)	0.466 (0.430 - 0.503)	0.466 (0.431 - 0.505)	1.162 (1.080 - 1.231)	1.160 (1.073 - 1.231)	1.163 (1.080 - 1.235)	0.451 (0.199 - 0.856)	0.450 (0.198 - 0.845)	0.450 (0.197 - 0.844)
**70+ years (rate per capita)**	0.438 (0.405 - 0.474)	0.433 (0.400 - 0.467)	0.427 (0.399 - 0.460)	1.133 (1.064 - 1.195)	1.130 (1.066 - 1.191)	1.123 (1.057 - 1.180)	0.405 (0.181 - 0.773)	0.399 (0.177 - 0.745)	0.394 (0.176 - 0.740)
**All ages (rate per capita)**	0.439 (0.418 - 0.460)	0.439 (0.418 - 0.458)	0.440 (0.421 - 0.462)	0.912 (0.870 - 0.954)	0.940 (0.900 - 0.979)	0.953 (0.913 - 0.995)	0.429 (0.189 - 0.806)	0.428 (0.188 - 0.802)	0.429 (0.188 - 0.809)
**Age-standardized (rate per capita)**	0.417 (0.395 - 0.439)	0.416 (0.394 - 0.436)	0.416 (0.395 - 0.438)	0.848 (0.806 - 0.890)	0.849 (0.809 - 0.891)	0.848 (0.806 - 0.891)	0.409 (0.180 - 0.770)	0.408 (0.178 - 0.769)	0.408 (0.179 - 0.762)
**Total number of cases (thousands)**	2,189 (2,087 - 2,295)	2,304 (2,196 - 2,405)	2,445 (2,335 - 2,562)	4,548 (4,342 - 4,761)	4,931 (4,723 - 5,137)	5,289 (5,065 - 5,520)	2 (1 - 4)	2 (1 - 4)	2 (1 - 4)
**France**									
**5 -14 years (rate per capita)**	0.382 (0.370 - 0.393)	0.353 (0.298 - 0.414)	0.386 (0.325 - 0.448)	0.459 (0.409 - 0.504)	0.511 (0.412 - 0.634)	0.536 (0.429 - 0.654)	0.381 (0.166 - 0.726)	0.353 (0.155 - 0.696)	0.386 (0.171 - 0.751)
**15 - 49 years (rate per capita)**	0.594 (0.564 - 0.623)	0.590 (0.563 - 0.616)	0.590 (0.562 - 0.619)	0.946 (0.891 - 1.003)	0.952 (0.895 - 1.008)	0.954 (0.898 - 1.009)	0.587 (0.257 - 1.108)	0.584 (0.253 - 1.119)	0.584 (0.254 - 1.117)
**50 - 69 years (rate per capita)**	0.538 (0.501 - 0.573)	0.538 (0.502 - 0.575)	0.538 (0.500 - 0.573)	1.078 (1.006 - 1.142)	1.077 (1.005 - 1.141)	1.077 (1.003 - 1.142)	0.520 (0.223 - 0.971)	0.522 (0.226 - 0.986)	0.521 (0.224 - 0.979)
**70+ years (rate per capita)**	0.494 (0.466 - 0.524)	0.496 (0.468 - 0.528)	0.486 (0.459 - 0.513)	1.053 (0.999 - 1.107)	1.058 (1.004 - 1.111)	1.049 (0.999 - 1.102)	0.457 (0.203 - 0.854)	0.460 (0.204 - 0.868)	0.449 (0.200 - 0.843)
**All ages (rate per capita)**	0.505 (0.487 - 0.521)	0.501 (0.483 - 0.520)	0.503 (0.485 - 0.520)	0.854 (0.818 - 0.890)	0.880 (0.840 - 0.915)	0.889 (0.850 - 0.926)	0.495 (0.216 - 0.935)	0.490 (0.214 - 0.944)	0.491 (0.213 - 0.937)
**Age-standardized (rate per capita)**	0.487 (0.471 - 0.503)	0.481 (0.462 - 0.501)	0.487 (0.468 - 0.505)	0.808 (0.773 - 0.842)	0.816 (0.775 - 0.854)	0.820 (0.778 - 0.860)	0.479 (0.209 - 0.901)	0.473 (0.207 - 0.906)	0.479 (0.207 - 0.909)
**Total number of cases (thousands)**	28,635 (27,635 - 29,555)	30,693 (29,570 - 31,831)	32,835 (31,664 - 33,945)	48,443 (46,369 - 50,441)	53,842 (51,434 - 56,030)	57,988 (55,444 - 60,408)	28 (12 - 53)	30 (13 - 58)	32 (14 - 61)
**Germany**									
**5 -14 years (rate per capita)**	0.194 (0.164 - 0.229)	0.222 (0.176 - 0.271)	0.223 (0.175 - 0.290)	0.301 (0.239 - 0.377)	0.323 (0.240 - 0.427)	0.326 (0.240 - 0.451)	0.193 (0.082 - 0.376)	0.221 (0.097 - 0.440)	0.223 (0.098 - 0.436)
**15 - 49 years (rate per capita)**	0.450 (0.413 - 0.486)	0.447 (0.411 - 0.484)	0.445 (0.410 - 0.483)	0.843 (0.771 - 0.916)	0.855 (0.780 - 0.926)	0.858 (0.784 - 0.937)	0.444 (0.192 - 0.843)	0.441 (0.192 - 0.835)	0.440 (0.193 - 0.840)
**50 - 69 years (rate per capita)**	0.407 (0.364 - 0.452)	0.406 (0.367 - 0.451)	0.407 (0.365 - 0.455)	0.991 (0.883 - 1.094)	0.995 (0.889 - 1.100)	0.991 (0.882 - 1.098)	0.393 (0.171 - 0.751)	0.392 (0.168 - 0.761)	0.393 (0.171 - 0.758)
**70+ years (rate per capita)**	0.368 (0.334 - 0.403)	0.369 (0.334 - 0.405)	0.368 (0.333 - 0.406)	0.927 (0.828 - 1.032)	0.930 (0.834 - 1.033)	0.930 (0.834 - 1.031)	0.341 (0.152 - 0.648)	0.342 (0.153 - 0.655)	0.342 (0.151 - 0.652)
**All ages (rate per capita)**	0.381 (0.359 - 0.402)	0.385 (0.364 - 0.407)	0.385 (0.363 - 0.406)	0.785 (0.736 - 0.831)	0.809 (0.761 - 0.858)	0.825 (0.773 - 0.874)	0.371 (0.162 - 0.702)	0.376 (0.167 - 0.704)	0.374 (0.165 - 0.718)
**Age-standardized (rate per capita)**	0.349 (0.329 - 0.370)	0.353 (0.332 - 0.374)	0.352 (0.331 - 0.373)	0.700 (0.657 - 0.745)	0.702 (0.659 - 0.749)	0.701 (0.657 - 0.746)	0.342 (0.150 - 0.645)	0.346 (0.152 - 0.655)	0.346 (0.152 - 0.652)
**Total number of cases (thousands)**	30,131 (28,430 - 31,820)	31,734 (29,932 - 33,476)	32,188 (30,383 - 33,923)	62,144 (58,306 - 65,765)	66,632 (62,693 - 70,673)	69,006 (64,659 - 73,084)	29 (13 - 56)	31 (14 - 58)	31 (14 - 60)
**Greece**									
**5 -14 years (rate per capita)**	0.408 (0.371 - 0.440)	0.266 (0.236 - 0.296)	0.356 (0.290 - 0.418)	0.438 (0.370 - 0.500)	0.344 (0.270 - 0.410)	0.452 (0.349 - 0.566)	0.407 (0.179 - 0.784)	0.266 (0.114 - 0.519)	0.355 (0.156 - 0.693)
**15 - 49 years (rate per capita)**	0.590 (0.562 - 0.617)	0.585 (0.556 - 0.614)	0.585 (0.556 - 0.613)	0.894 (0.831 - 0.954)	0.905 (0.843 - 0.964)	0.912 (0.850 - 0.969)	0.583 (0.257 - 1.114)	0.579 (0.251 - 1.106)	0.578 (0.254 - 1.114)
**50 - 69 years (rate per capita)**	0.540 (0.503 - 0.577)	0.539 (0.504 - 0.576)	0.540 (0.503 - 0.575)	1.049 (0.970 - 1.121)	1.052 (0.975 - 1.125)	1.050 (0.970 - 1.121)	0.522 (0.230 - 0.974)	0.522 (0.228 - 0.978)	0.523 (0.229 - 0.987)
**70+ years (rate per capita)**	0.503 (0.473 - 0.536)	0.507 (0.475 - 0.539)	0.493 (0.466 - 0.522)	1.033 (0.968 - 1.095)	1.038 (0.973 - 1.099)	1.024 (0.962 - 1.080)	0.466 (0.209 - 0.876)	0.472 (0.211 - 0.902)	0.457 (0.205 - 0.861)
**All ages (rate per capita)**	0.513 (0.495 - 0.532)	0.505 (0.486 - 0.524)	0.510 (0.492 - 0.529)	0.832 (0.793 - 0.868)	0.856 (0.815 - 0.895)	0.877 (0.837 - 0.915)	0.502 (0.222 - 0.952)	0.493 (0.217 - 0.941)	0.497 (0.219 - 0.949)
**Age-standardized (rate per capita)**	0.489 (0.471 - 0.506)	0.461 (0.444 - 0.480)	0.478 (0.456 - 0.497)	0.769 (0.731 - 0.804)	0.755 (0.715 - 0.794)	0.774 (0.734 - 0.817)	0.480 (0.211 - 0.908)	0.453 (0.198 - 0.858)	0.469 (0.206 - 0.892)
**Total number of cases (thousands)**	5,197 (5,015 - 5,385)	5,590 (5,385 - 5,799)	5,572 (5,374 - 5,775)	8,431 (8,032 - 8,796)	9,476 (9,019 - 9,910)	9,580 (9,143 - 9,997)	5 (2 - 10)	5 (2 - 10)	5 (2 - 10)
**Iceland**									
**5 -14 years (rate per capita)**	0.389 (0.327 - 0.449)	0.416 (0.346 - 0.472)	0.388 (0.326 - 0.447)	0.441 (0.337 - 0.540)	0.460 (0.359 - 0.556)	0.439 (0.338 - 0.539)	0.388 (0.173 - 0.754)	0.415 (0.183 - 0.811)	0.388 (0.172 - 0.725)
**15 - 49 years (rate per capita)**	0.605 (0.576 - 0.637)	0.606 (0.578 - 0.635)	0.604 (0.574 - 0.634)	0.831 (0.772 - 0.893)	0.840 (0.782 - 0.898)	0.837 (0.776 - 0.898)	0.599 (0.262 - 1.131)	0.600 (0.260 - 1.140)	0.598 (0.258 - 1.136)
**50 - 69 years (rate per capita)**	0.569 (0.533 - 0.605)	0.569 (0.531 - 0.606)	0.569 (0.531 - 0.605)	0.989 (0.910 - 1.063)	0.983 (0.901 - 1.057)	0.986 (0.903 - 1.060)	0.551 (0.244 - 1.033)	0.552 (0.241 - 1.036)	0.552 (0.243 - 1.029)
**70+ years (rate per capita)**	0.528 (0.498 - 0.556)	0.527 (0.499 - 0.557)	0.518 (0.491 - 0.548)	0.974 (0.899 - 1.037)	0.972 (0.899 - 1.032)	0.965 (0.897 - 1.024)	0.489 (0.219 - 0.926)	0.488 (0.217 - 0.924)	0.479 (0.214 - 0.904)
**All ages (rate per capita)**	0.507 (0.486 - 0.527)	0.521 (0.501 - 0.540)	0.519 (0.498 - 0.539)	0.731 (0.690 - 0.775)	0.762 (0.721 - 0.801)	0.775 (0.733 - 0.815)	0.498 (0.219 - 0.931)	0.511 (0.225 - 0.964)	0.509 (0.223 - 0.964)
**Age-standardized (rate per capita)**	0.501 (0.480 - 0.521)	0.506 (0.486 - 0.525)	0.501 (0.479 - 0.522)	0.727 (0.687 - 0.770)	0.729 (0.688 - 0.770)	0.727 (0.685 - 0.766)	0.492 (0.216 - 0.919)	0.497 (0.218 - 0.935)	0.493 (0.214 - 0.930)
**Total number of cases (thousands)**	129 (124 - 134)	155 (149 - 160)	169 (163 - 176)	186 (176 - 197)	226 (214 - 238)	253 (239 - 266)	0 (0 - 0)	0 (0 - 0)	0 (0 - 0)
**Ireland**									
**5 -14 years (rate per capita)**	0.249 (0.194 - 0.311)	0.245 (0.189 - 0.317)	0.243 (0.187 - 0.314)	0.343 (0.262 - 0.468)	0.339 (0.256 - 0.462)	0.336 (0.252 - 0.460)	0.248 (0.107 - 0.481)	0.245 (0.106 - 0.484)	0.242 (0.104 - 0.476)
**15 - 49 years (rate per capita)**	0.478 (0.439 - 0.515)	0.486 (0.448 - 0.526)	0.497 (0.459 - 0.537)	0.837 (0.763 - 0.910)	0.853 (0.780 - 0.932)	0.867 (0.791 - 0.944)	0.472 (0.206 - 0.892)	0.480 (0.209 - 0.907)	0.491 (0.212 - 0.917)
**50 - 69 years (rate per capita)**	0.446 (0.399 - 0.492)	0.446 (0.398 - 0.494)	0.446 (0.398 - 0.493)	1.019 (0.914 - 1.120)	1.017 (0.908 - 1.116)	1.018 (0.908 - 1.120)	0.431 (0.186 - 0.819)	0.432 (0.189 - 0.818)	0.432 (0.190 - 0.811)
**70+ years (rate per capita)**	0.416 (0.376 - 0.457)	0.409 (0.370 - 0.450)	0.406 (0.369 - 0.447)	0.978 (0.874 - 1.078)	0.969 (0.868 - 1.062)	0.966 (0.866 - 1.057)	0.386 (0.172 - 0.725)	0.379 (0.168 - 0.717)	0.376 (0.167 - 0.706)
**All ages (rate per capita)**	0.387 (0.362 - 0.410)	0.404 (0.380 - 0.427)	0.407 (0.384 - 0.430)	0.717 (0.672 - 0.761)	0.760 (0.712 - 0.806)	0.768 (0.721 - 0.815)	0.379 (0.168 - 0.717)	0.396 (0.174 - 0.753)	0.399 (0.175 - 0.742)
**Age-standardized (rate per capita)**	0.387 (0.362 - 0.409)	0.387 (0.363 - 0.409)	0.387 (0.363 - 0.410)	0.720 (0.676 - 0.764)	0.721 (0.675 - 0.766)	0.721 (0.675 - 0.766)	0.379 (0.167 - 0.713)	0.379 (0.166 - 0.720)	0.380 (0.167 - 0.713)
**Total number of cases (thousands)**	1,365 (1,278 - 1,447)	1,687 (1,585 - 1,784)	1,951 (1,838 - 2,061)	2,530 (2,372 - 2,687)	3,173 (2,973 - 3,364)	3,679 (3,454 - 3,902)	1 (1 - 3)	2 (1 - 3)	2 (1 - 4)
**Israel**									
**5 -14 years (rate per capita)**	0.331 (0.269 - 0.393)	0.308 (0.245 - 0.374)	0.309 (0.246 - 0.375)	0.402 (0.308 - 0.520)	0.384 (0.290 - 0.507)	0.385 (0.291 - 0.508)	0.331 (0.147 - 0.636)	0.308 (0.134 - 0.596)	0.309 (0.135 - 0.602)

	Prevalence 1990	Prevalence 2005	Prevalence 2015	Incidence 1990	Incidence 2005	Incidence 2015	YLD rate 1990	YLD rate 2005	YLD rate 2015
15 - 49 years (rate per capita)	0.543 (0.510 - 0.578)	0.545 (0.512 - 0.578)	0.546 (0.513 - 0.579)	0.840 (0.774 - 0.907)	0.845 (0.780 - 0.910)	0.849 (0.786 - 0.913)	0.537 (0.232 - 1.021)	0.539 (0.235 - 1.029)	0.540 (0.236 - 1.023)
50 - 69 years (rate per capita)	0.512 (0.471 - 0.550)	0.512 (0.471 - 0.553)	0.512 (0.470 - 0.551)	1.025 (0.938 - 1.107)	1.019 (0.926 - 1.106)	1.024 (0.930 - 1.108)	0.495 (0.216 - 0.928)	0.494 (0.213 - 0.924)	0.493 (0.214 - 0.930)
70+ years (rate per capita)	0.477 (0.443 - 0.514)	0.472 (0.439 - 0.506)	0.465 (0.434 - 0.497)	0.999 (0.910 - 1.078)	0.991 (0.910 - 1.065)	0.984 (0.905 - 1.057)	0.443 (0.198 - 0.844)	0.436 (0.195 - 0.822)	0.428 (0.193 - 0.811)
All ages (rate per capita)	0.433 (0.409 - 0.455)	0.437 (0.415 - 0.459)	0.437 (0.415 - 0.458)	0.695 (0.650 - 0.739)	0.717 (0.677 - 0.761)	0.723 (0.681 - 0.766)	0.426 (0.188 - 0.806)	0.429 (0.189 - 0.811)	0.428 (0.189 - 0.815)
Age-standardized (rate per capita)	0.448 (0.425 - 0.469)	0.446 (0.424 - 0.468)	0.446 (0.424 - 0.468)	0.736 (0.693 - 0.778)	0.734 (0.692 - 0.778)	0.734 (0.692 - 0.778)	0.440 (0.194 - 0.826)	0.438 (0.193 - 0.828)	0.437 (0.193 - 0.832)
Total number of cases (thousands)	1,947 (1,840 - 2,048)	2,886 (2,739 - 3,030)	3,516 (3,343 - 3,683)	3,124 (2,923 - 3,322)	4,737 (4,472 - 5,025)	5,817 (5,484 - 6,167)	2 (1 - 4)	3 (1 - 5)	3 (2 - 7)
**Italy**									
5 -14 years (rate per capita)	0.288 (0.216 - 0.352)	0.196 (0.170 - 0.218)	0.235 (0.178 - 0.304)	0.316 (0.228 - 0.430)	0.210 (0.165 - 0.258)	0.275 (0.199 - 0.405)	0.287 (0.124 - 0.565)	0.195 (0.085 - 0.375)	0.235 (0.101 - 0.466)
15 - 49 years (rate per capita)	0.428 (0.395 - 0.463)	0.437 (0.403 - 0.473)	0.433 (0.400 - 0.469)	0.630 (0.571 - 0.690)	0.650 (0.588 - 0.716)	0.654 (0.590 - 0.720)	0.423 (0.184 - 0.797)	0.432 (0.189 - 0.816)	0.428 (0.187 - 0.806)
50 - 69 years (rate per capita)	0.396 (0.354 - 0.439)	0.396 (0.355 - 0.440)	0.397 (0.354 - 0.444)	0.748 (0.656 - 0.848)	0.748 (0.661 - 0.845)	0.749 (0.660 - 0.858)	0.382 (0.160 - 0.728)	0.382 (0.165 - 0.715)	0.384 (0.168 - 0.729)
70+ years (rate per capita)	0.359 (0.327 - 0.393)	0.356 (0.324 - 0.390)	0.352 (0.322 - 0.384)	0.689 (0.606 - 0.772)	0.688 (0.613 - 0.769)	0.680 (0.605 - 0.758)	0.331 (0.148 - 0.633)	0.329 (0.148 - 0.636)	0.324 (0.142 - 0.622)
All ages (rate per capita)	0.376 (0.354 - 0.398)	0.373 (0.353 - 0.394)	0.375 (0.353 - 0.397)	0.596 (0.556 - 0.638)	0.607 (0.567 - 0.648)	0.620 (0.578 - 0.663)	0.368 (0.163 - 0.697)	0.363 (0.161 - 0.685)	0.365 (0.160 - 0.684)
Age-standardized (rate per capita)	0.353 (0.331 - 0.375)	0.338 (0.319 - 0.357)	0.346 (0.325 - 0.367)	0.542 (0.504 - 0.584)	0.524 (0.489 - 0.560)	0.536 (0.499 - 0.579)	0.346 (0.153 - 0.655)	0.331 (0.146 - 0.621)	0.340 (0.149 - 0.648)
Total number of cases (thousands)	21,407 (20,122 - 22,642)	21,852 (20,661 - 23,055)	23,521 (22,191 - 24,916)	33,888 (31,605 - 36,298)	35,583 (33,202 - 37,967)	38,912 (36,310 - 41,664)	21 (9 - 40)	21 (9 - 40)	23 (10 - 43)
**Luxembourg**									
5 -14 years (rate per capita)	0.248 (0.176 - 0.342)	0.250 (0.177 - 0.344)	0.253 (0.180 - 0.348)	0.420 (0.262 - 0.593)	0.422 (0.263 - 0.597)	0.427 (0.265 - 0.603)	0.248 (0.102 - 0.498)	0.249 (0.104 - 0.504)	0.253 (0.106 - 0.511)
15 - 49 years (rate per capita)	0.472 (0.403 - 0.537)	0.471 (0.404 - 0.535)	0.470 (0.403 - 0.537)	0.855 (0.732 - 0.972)	0.860 (0.737 - 0.976)	0.859 (0.737 - 0.974)	0.465 (0.195 - 0.878)	0.465 (0.198 - 0.885)	0.464 (0.197 - 0.873)
50 - 69 years (rate per capita)	0.448 (0.368 - 0.527)	0.448 (0.368 - 0.526)	0.448 (0.366 - 0.527)	0.955 (0.781 - 1.128)	0.953 (0.779 - 1.128)	0.953 (0.776 - 1.128)	0.431 (0.184 - 0.833)	0.432 (0.183 - 0.827)	0.432 (0.186 - 0.834)
70+ years (rate per capita)	0.416 (0.349 - 0.490)	0.416 (0.348 - 0.490)	0.408 (0.344 - 0.475)	0.929 (0.767 - 1.082)	0.930 (0.771 - 1.084)	0.919 (0.767 - 1.063)	0.381 (0.172 - 0.741)	0.383 (0.172 - 0.742)	0.375 (0.168 - 0.719)
All ages (rate per capita)	0.409 (0.368 - 0.448)	0.405 (0.366 - 0.445)	0.408 (0.368 - 0.447)	0.784 (0.708 - 0.862)	0.781 (0.707 - 0.856)	0.790 (0.713 - 0.865)	0.398 (0.174 - 0.757)	0.395 (0.174 - 0.749)	0.399 (0.173 - 0.753)
Age-standardized (rate per capita)	0.381 (0.342 - 0.421)	0.380 (0.342 - 0.421)	0.380 (0.342 - 0.421)	0.722 (0.650 - 0.793)	0.722 (0.649 - 0.793)	0.722 (0.649 - 0.792)	0.372 (0.163 - 0.707)	0.372 (0.164 - 0.713)	0.373 (0.163 - 0.707)
Total number of cases (thousands)	155 (140 - 170)	185 (167 - 203)	227 (205 - 249)	298 (269 - 328)	356 (322 - 390)	440 (397 - 482)	0 (0 - 0)	0 (0 - 0)	0 (0 - 0)
**Malta**									
5 -14 years (rate per capita)	0.254 (0.180 - 0.349)	0.255 (0.181 - 0.351)	0.258 (0.183 - 0.355)	0.429 (0.265 - 0.605)	0.431 (0.266 - 0.608)	0.434 (0.268 - 0.612)	0.253 (0.106 - 0.517)	0.255 (0.106 - 0.519)	0.258 (0.107 - 0.524)
15 - 49 years (rate per capita)	0.472 (0.404 - 0.537)	0.467 (0.402 - 0.533)	0.469 (0.401 - 0.532)	0.852 (0.731 - 0.970)	0.854 (0.729 - 0.968)	0.854 (0.731 - 0.970)	0.465 (0.197 - 0.885)	0.461 (0.198 - 0.864)	0.463 (0.195 - 0.879)
50 - 69 years (rate per capita)	0.448 (0.368 - 0.526)	0.448 (0.368 - 0.527)	0.447 (0.370 - 0.527)	0.954 (0.779 - 1.128)	0.954 (0.777 - 1.128)	0.957 (0.785 - 1.125)	0.432 (0.184 - 0.827)	0.433 (0.184 - 0.834)	0.432 (0.185 - 0.836)
70+ years (rate per capita)	0.418 (0.348 - 0.492)	0.417 (0.348 - 0.491)	0.419 (0.348 - 0.493)	0.933 (0.769 - 1.086)	0.931 (0.769 - 1.085)	0.934 (0.768 - 1.088)	0.386 (0.175 - 0.749)	0.386 (0.174 - 0.750)	0.387 (0.175 - 0.752)
All ages (rate per capita)	0.391 (0.352 - 0.432)	0.407 (0.369 - 0.449)	0.415 (0.376 - 0.457)	0.739 (0.665 - 0.813)	0.787 (0.709 - 0.865)	0.812 (0.734 - 0.894)	0.383 (0.168 - 0.728)	0.398 (0.173 - 0.754)	0.405 (0.177 - 0.772)
Age-standardized (rate per capita)	0.381 (0.342 - 0.421)	0.380 (0.342 - 0.420)	0.380 (0.342 - 0.421)	0.722 (0.649 - 0.792)	0.722 (0.649 - 0.793)	0.722 (0.649 - 0.793)	0.373 (0.163 - 0.710)	0.373 (0.163 - 0.708)	0.373 (0.164 - 0.713)
Total number of cases (thousands)	139 (125 - 153)	162 (146 - 178)	174 (157 - 191)	263 (236 - 289)	313 (282 - 343)	340 (307 - 374)	0 (0 - 0)	0 (0 - 0)	0 (0 - 0)
**Netherlands**									
5 -14 years (rate per capita)	0.176 (0.137 - 0.228)	0.175 (0.136 - 0.227)	0.174 (0.135 - 0.225)	0.282 (0.207 - 0.384)	0.282 (0.206 - 0.383)	0.279 (0.205 - 0.381)	0.175 (0.073 - 0.346)	0.175 (0.075 - 0.345)	0.173 (0.074 - 0.345)
15 - 49 years (rate per capita)	0.401 (0.366 - 0.438)	0.402 (0.366 - 0.438)	0.395 (0.361 - 0.432)	0.817 (0.743 - 0.897)	0.829 (0.755 - 0.909)	0.824 (0.750 - 0.904)	0.396 (0.172 - 0.753)	0.396 (0.172 - 0.754)	0.390 (0.171 - 0.738)
50 - 69 years (rate per capita)	0.360 (0.320 - 0.404)	0.361 (0.318 - 0.406)	0.361 (0.319 - 0.404)	0.954 (0.842 - 1.064)	0.953 (0.842 - 1.064)	0.954 (0.845 - 1.068)	0.347 (0.152 - 0.662)	0.348 (0.153 - 0.670)	0.347 (0.154 - 0.661)
70+ years (rate per capita)	0.326 (0.295 - 0.362)	0.324 (0.294 - 0.360)	0.323 (0.292 - 0.358)	0.887 (0.792 - 0.986)	0.883 (0.790 - 0.979)	0.879 (0.786 - 0.975)	0.301 (0.133 - 0.568)	0.299 (0.132 - 0.569)	0.298 (0.132 - 0.568)
All ages (rate per capita)	0.335 (0.313 - 0.356)	0.333 (0.310 - 0.353)	0.332 (0.310 - 0.352)	0.734 (0.687 - 0.783)	0.746 (0.700 - 0.793)	0.759 (0.710 - 0.806)	0.328 (0.143 - 0.623)	0.325 (0.143 - 0.613)	0.323 (0.142 - 0.610)
Age-standardized (rate per capita)	0.312 (0.290 - 0.332)	0.312 (0.290 - 0.332)	0.312 (0.290 - 0.332)	0.675 (0.631 - 0.718)	0.675 (0.631 - 0.719)	0.675 (0.631 - 0.718)	0.305 (0.134 - 0.579)	0.305 (0.134 - 0.579)	0.306 (0.134 - 0.581)
Total number of cases (thousands)	5,008 (4,676 - 5,318)	5,424 (5,060 - 5,753)	5,704 (5,325 - 6,052)	10,966 (10,266 - 11,693)	12,164 (11,412 - 12,924)	13,053 (12,209 - 13,852)	5 (2 - 9)	5 (2 - 10)	6 (2 - 10)
**Norway**									
5 -14 years (rate per capita)	0.206 (0.175 - 0.249)	0.219 (0.173 - 0.277)	0.244 (0.189 - 0.310)	0.303 (0.238 - 0.392)	0.320 (0.242 - 0.427)	0.340 (0.253 - 0.461)	0.206 (0.089 - 0.396)	0.219 (0.093 - 0.435)	0.244 (0.107 - 0.472)
15 - 49 years (rate per capita)	0.477 (0.444 - 0.511)	0.477 (0.444 - 0.512)	0.472 (0.439 - 0.508)	0.853 (0.783 - 0.925)	0.860 (0.788 - 0.931)	0.857 (0.788 - 0.927)	0.471 (0.203 - 0.902)	0.471 (0.206 - 0.898)	0.466 (0.203 - 0.888)
50 - 69 years (rate per capita)	0.438 (0.396 - 0.482)	0.438 (0.395 - 0.484)	0.438 (0.395 - 0.481)	1.018 (0.914 - 1.124)	1.015 (0.910 - 1.122)	1.017 (0.909 - 1.122)	0.422 (0.183 - 0.814)	0.423 (0.184 - 0.807)	0.422 (0.185 - 0.816)
70+ years (rate per capita)	0.403 (0.369 - 0.439)	0.394 (0.362 - 0.428)	0.392 (0.360 - 0.426)	0.969 (0.872 - 1.071)	0.953 (0.862 - 1.046)	0.950 (0.858 - 1.043)	0.373 (0.167 - 0.710)	0.364 (0.161 - 0.687)	0.361 (0.159 - 0.672)
All ages (rate per capita)	0.397 (0.377 - 0.417)	0.395 (0.376 - 0.415)	0.401 (0.380 - 0.423)	0.774 (0.727 - 0.819)	0.778 (0.735 - 0.822)	0.794 (0.749 - 0.841)	0.387 (0.169 - 0.745)	0.386 (0.169 - 0.728)	0.391 (0.171 - 0.743)
Age-standardized (rate per capita)	0.375 (0.354 - 0.396)	0.375 (0.354 - 0.396)	0.379 (0.357 - 0.400)	0.715 (0.671 - 0.757)	0.716 (0.674 - 0.757)	0.718 (0.675 - 0.763)	0.367 (0.159 - 0.704)	0.367 (0.161 - 0.699)	0.371 (0.163 - 0.700)
Total number of cases (thousands)	1,681 (1,595 - 1,768)	1,827 (1,736 - 1,919)	2,071 (1,964 - 2,185)	3,277 (3,081 - 3,471)	3,597 (3,395 - 3,799)	4,102 (3,868 - 4,345)	2 (1 - 3)	2 (1 - 3)	2 (1 - 4)
**Portugal**									
5 -14 years (rate per capita)	0.268 (0.207 - 0.338)	0.259 (0.200 - 0.329)	0.254 (0.195 - 0.321)	0.363 (0.272 - 0.479)	0.352 (0.265 - 0.473)	0.345 (0.260 - 0.467)	0.268 (0.115 - 0.531)	0.259 (0.112 - 0.513)	0.253 (0.111 - 0.502)
15 - 49 years (rate per capita)	0.490 (0.452 - 0.532)	0.498 (0.461 - 0.541)	0.501 (0.464 - 0.544)	0.845 (0.774 - 0.915)	0.864 (0.792 - 0.935)	0.873 (0.803 - 0.947)	0.483 (0.212 - 0.914)	0.492 (0.214 - 0.937)	0.494 (0.214 - 0.944)
50 - 69 years (rate per capita)	0.456 (0.408 - 0.503)	0.456 (0.408 - 0.502)	0.456 (0.408 - 0.503)	1.024 (0.918 - 1.123)	1.024 (0.919 - 1.120)	1.023 (0.917 - 1.120)	0.438 (0.192 - 0.834)	0.439 (0.192 - 0.831)	0.440 (0.193 - 0.836)
70+ years (rate per capita)	0.423 (0.383 - 0.466)	0.422 (0.381 - 0.465)	0.418 (0.378 - 0.460)	0.982 (0.884 - 1.078)	0.981 (0.883 - 1.076)	0.973 (0.879 - 1.066)	0.390 (0.174 - 0.735)	0.390 (0.172 - 0.730)	0.386 (0.171 - 0.714)
All ages (rate per capita)	0.415 (0.390 - 0.438)	0.429 (0.404 - 0.451)	0.432 (0.408 - 0.455)	0.773 (0.727 - 0.821)	0.816 (0.766 - 0.863)	0.833 (0.783 - 0.882)	0.405 (0.178 - 0.752)	0.418 (0.183 - 0.789)	0.420 (0.184 - 0.792)
Age-standardized (rate per capita)	0.396 (0.371 - 0.419)	0.396 (0.371 - 0.419)	0.396 (0.371 - 0.419)	0.725 (0.681 - 0.771)	0.725 (0.681 - 0.771)	0.725 (0.681 - 0.771)	0.387 (0.170 - 0.722)	0.388 (0.170 - 0.727)	0.388 (0.169 - 0.728)
Total number of cases (thousands)	4,128 (3,882 - 4,356)	4,522 (4,266 - 4,760)	4,665 (4,404 - 4,913)	7,695 (7,238 - 8,168)	8,607 (8,080 - 9,105)	8,999 (8,455 - 9,524)	4 (2 - 7)	4 (2 - 8)	5 (2 - 9)
**Spain**									
5 -14 years (rate per capita)	0.412 (0.364 - 0.463)	0.369 (0.311 - 0.432)	0.357 (0.299 - 0.417)	0.601 (0.506 - 0.693)	0.554 (0.445 - 0.678)	0.534 (0.428 - 0.662)	0.411 (0.180 - 0.804)	0.369 (0.163 - 0.720)	0.357 (0.157 - 0.708)
15 - 49 years (rate per capita)	0.578 (0.548 - 0.606)	0.579 (0.550 - 0.608)	0.580 (0.553 - 0.609)	0.973 (0.915 - 1.031)	0.982 (0.924 - 1.037)	0.984 (0.927 - 1.038)	0.572 (0.251 - 1.083)	0.574 (0.250 - 1.093)	0.575 (0.250 - 1.092)
50 - 69 years (rate per capita)	0.520 (0.484 - 0.557)	0.520 (0.483 - 0.555)	0.521 (0.483 - 0.559)	1.109 (1.039 - 1.174)	1.109 (1.038 - 1.171)	1.107 (1.034 - 1.172)	0.503 (0.221 - 0.935)	0.504 (0.219 - 0.953)	0.506 (0.222 - 0.955)
70+ years (rate per capita)	0.484 (0.453 - 0.515)	0.480 (0.452 - 0.511)	0.474 (0.447 - 0.503)	1.089 (1.032 - 1.150)	1.091 (1.036 - 1.146)	1.078 (1.027 - 1.129)	0.448 (0.200 - 0.845)	0.445 (0.198 - 0.842)	0.439 (0.196 - 0.821)
All ages (rate per capita)	0.502 (0.482 - 0.520)	0.506 (0.487 - 0.523)	0.505 (0.486 - 0.522)	0.905 (0.864 - 0.941)	0.933 (0.891 - 0.971)	0.936 (0.895 - 0.974)	0.492 (0.215 - 0.920)	0.495 (0.216 - 0.952)	0.494 (0.219 - 0.950)
Age-standardized (rate per capita)	0.478 (0.459 - 0.496)	0.471 (0.451 - 0.490)	0.473 (0.453 - 0.491)	0.849 (0.811 - 0.884)	0.847 (0.805 - 0.886)	0.846 (0.803 - 0.886)	0.470 (0.205 - 0.878)	0.463 (0.202 - 0.880)	0.466 (0.206 - 0.891)
Total number of cases (thousands)	19,547 (18,784 - 20,263)	21,916 (21,107 - 22,680)	24,609 (23,702 - 25,455)	35,248 (33,649 - 36,641)	40,422 (38,641 - 42,099)	45,626 (43,637 - 47,471)	19 (8 - 36)	21 (9 - 41)	24 (11 - 46)
**Sweden**									
5 -14 years (rate per capita)	0.210 (0.172 - 0.260)	0.236 (0.180 - 0.302)	0.208 (0.160 - 0.274)	0.218 (0.167 - 0.284)	0.252 (0.186 - 0.362)	0.234 (0.170 - 0.364)	0.210 (0.091 - 0.410)	0.236 (0.101 - 0.472)	0.208 (0.089 - 0.415)
15 - 49 years (rate per capita)	0.417 (0.381 - 0.454)	0.423 (0.387 - 0.459)	0.423 (0.387 - 0.459)	0.567 (0.511 - 0.624)	0.579 (0.523 - 0.635)	0.581 (0.525 - 0.639)	0.412 (0.179 - 0.781)	0.419 (0.181 - 0.795)	0.418 (0.181 - 0.793)
50 - 69 years (rate per capita)	0.378 (0.332 - 0.425)	0.395 (0.353 - 0.443)	0.394 (0.353 - 0.441)	0.655 (0.578 - 0.742)	0.664 (0.587 - 0.758)	0.663 (0.585 - 0.750)	0.365 (0.155 - 0.699)	0.382 (0.166 - 0.725)	0.381 (0.162 - 0.721)
70+ years (rate per capita)	0.341 (0.308 - 0.377)	0.351 (0.321 - 0.382)	0.349 (0.318 - 0.382)	0.598 (0.527 - 0.671)	0.601 (0.539 - 0.664)	0.601 (0.539 - 0.667)	0.317 (0.141 - 0.603)	0.324 (0.144 - 0.608)	0.323 (0.143 - 0.622)
All ages (rate per capita)	0.349 (0.328 - 0.370)	0.362 (0.341 - 0.384)	0.359 (0.337 - 0.380)	0.513 (0.477 - 0.548)	0.532 (0.497 - 0.569)	0.533 (0.497 - 0.571)	0.341 (0.150 - 0.650)	0.354 (0.156 - 0.664)	0.350 (0.154 - 0.667)
Age-standardized (rate per capita)	0.331 (0.310 - 0.352)	0.339 (0.318 - 0.362)	0.340 (0.318 - 0.363)	0.468 (0.435 - 0.503)	0.481 (0.447 - 0.519)	0.482 (0.448 - 0.521)	0.325 (0.144 - 0.617)	0.333 (0.146 - 0.632)	0.334 (0.146 - 0.631)
Total number of cases (thousands)	2,983 (2,801 - 3,166)	3,267 (3,075 - 3,461)	3,517 (3,306 - 3,730)	4,385 (4,079 - 4,686)	4,800 (4,486 - 5,133)	5,227 (4,871 - 5,605)	3 (1 - 6)	3 (1 - 6)	3 (2 - 7)
**Switzerland**									

	Prevalence 1990	Prevalence 2005	Prevalence 2015	Incidence 1990	Incidence 2005	Incidence 2015	YLD rate 1990	YLD rate 2005	YLD rate 2015
5 -14 years (rate per capita)	0.249 (0.177 - 0.344)	0.255 (0.181 - 0.351)	0.252 (0.180 - 0.348)	0.422 (0.263 - 0.596)	0.431 (0.266 - 0.609)	0.427 (0.265 - 0.603)	0.249 (0.103 - 0.507)	0.255 (0.105 - 0.517)	0.252 (0.104 - 0.513)
15 - 49 years (rate per capita)	0.470 (0.403 - 0.535)	0.471 (0.403 - 0.535)	0.470 (0.402 - 0.538)	0.854 (0.734 - 0.971)	0.859 (0.736 - 0.975)	0.859 (0.738 - 0.973)	0.463 (0.198 - 0.868)	0.464 (0.196 - 0.878)	0.464 (0.197 - 0.879)
50 - 69 years (rate per capita)	0.448 (0.369 - 0.525)	0.448 (0.368 - 0.527)	0.448 (0.368 - 0.525)	0.955 (0.779 - 1.128)	0.954 (0.777 - 1.130)	0.954 (0.782 - 1.126)	0.432 (0.183 - 0.828)	0.433 (0.185 - 0.843)	0.433 (0.184 - 0.822)
70+ years (rate per capita)	0.413 (0.347 - 0.483)	0.410 (0.345 - 0.479)	0.407 (0.343 - 0.474)	0.925 (0.767 - 1.072)	0.922 (0.769 - 1.067)	0.919 (0.768 - 1.062)	0.379 (0.170 - 0.730)	0.378 (0.169 - 0.729)	0.376 (0.170 - 0.713)
All ages (rate per capita)	0.408 (0.367 - 0.447)	0.411 (0.373 - 0.450)	0.412 (0.373 - 0.452)	0.784 (0.708 - 0.860)	0.798 (0.720 - 0.875)	0.806 (0.727 - 0.882)	0.397 (0.174 - 0.751)	0.401 (0.175 - 0.754)	0.402 (0.175 - 0.757)
Age-standardized (rate per capita)	0.381 (0.342 - 0.421)	0.381 (0.342 - 0.421)	0.380 (0.342 - 0.421)	0.722 (0.649 - 0.793)	0.722 (0.649 - 0.793)	0.722 (0.649 - 0.793)	0.372 (0.162 - 0.707)	0.373 (0.163 - 0.709)	0.373 (0.163 - 0.711)
Total number of cases (thousands)	2,735 (2,465 - 3,001)	3,055 (2,771 - 3,345)	3,414 (3,086 - 3,746)	5,260 (4,753 - 5,771)	5,930 (5,355 - 6,503)	6,673 (6,019 - 7,300)	3 (1 - 5)	3 (1 - 6)	3 (1 - 6)
**United Kingdom**									
5 -14 years (rate per capita)	0.280 (0.225 - 0.343)	0.279 (0.222 - 0.342)	0.275 (0.219 - 0.338)	0.381 (0.288 - 0.502)	0.389 (0.295 - 0.508)	0.384 (0.290 - 0.505)	0.279 (0.123 - 0.543)	0.278 (0.122 - 0.539)	0.274 (0.120 - 0.524)
15 - 49 years (rate per capita)	0.507 (0.473 - 0.542)	0.509 (0.476 - 0.546)	0.507 (0.474 - 0.543)	0.854 (0.784 - 0.919)	0.861 (0.797 - 0.930)	0.863 (0.797 - 0.931)	0.501 (0.218 - 0.955)	0.503 (0.219 - 0.956)	0.501 (0.220 - 0.955)
50 - 69 years (rate per capita)	0.475 (0.435 - 0.517)	0.475 (0.434 - 0.517)	0.476 (0.435 - 0.518)	1.013 (0.912 - 1.105)	1.011 (0.910 - 1.105)	1.012 (0.913 - 1.105)	0.460 (0.202 - 0.875)	0.460 (0.199 - 0.878)	0.462 (0.200 - 0.885)
70+ years (rate per capita)	0.441 (0.409 - 0.477)	0.436 (0.405 - 0.471)	0.434 (0.403 - 0.467)	0.987 (0.898 - 1.069)	0.982 (0.894 - 1.063)	0.979 (0.892 - 1.059)	0.412 (0.184 - 0.779)	0.407 (0.181 - 0.778)	0.404 (0.180 - 0.772)
All ages (rate per capita)	0.431 (0.410 - 0.451)	0.436 (0.415 - 0.455)	0.434 (0.414 - 0.455)	0.785 (0.741 - 0.828)	0.801 (0.757 - 0.845)	0.809 (0.763 - 0.852)	0.422 (0.187 - 0.795)	0.426 (0.187 - 0.804)	0.425 (0.188 - 0.806)
Age-standardized (rate per capita)	0.411 (0.389 - 0.432)	0.411 (0.390 - 0.432)	0.411 (0.390 - 0.432)	0.730 (0.686 - 0.772)	0.730 (0.686 - 0.775)	0.730 (0.686 - 0.775)	0.404 (0.179 - 0.769)	0.403 (0.177 - 0.761)	0.403 (0.177 - 0.761)
Total number of cases (thousands)	24,684 (23,456 - 25,813)	26,248 (25,007 - 27,440)	27,911 (26,602 - 29,202)	44,957 (42,426 - 47,420)	48,263 (45,601 - 50,894)	51,958 (49,048 - 54,751)	24 (11 - 45)	26 (11 - 48)	27 (12 - 52)
**England**									
5 -14 years (rate per capita)	0.271 (0.216 - 0.334)	0.270 (0.213 - 0.336)	0.266 (0.210 - 0.331)	0.373 (0.282 - 0.500)	0.382 (0.288 - 0.506)	0.377 (0.283 - 0.501)	0.270 (0.120 - 0.528)	0.269 (0.118 - 0.523)	0.266 (0.116 - 0.510)
15 - 49 years (rate per capita)	0.498 (0.464 - 0.535)	0.500 (0.467 - 0.538)	0.498 (0.465 - 0.536)	0.855 (0.784 - 0.922)	0.862 (0.797 - 0.933)	0.865 (0.798 - 0.935)	0.493 (0.215 - 0.941)	0.494 (0.216 - 0.941)	0.493 (0.216 - 0.939)
50 - 69 years (rate per capita)	0.466 (0.424 - 0.509)	0.466 (0.424 - 0.509)	0.466 (0.423 - 0.510)	1.014 (0.911 - 1.112)	1.013 (0.909 - 1.113)	1.015 (0.911 - 1.112)	0.452 (0.199 - 0.857)	0.452 (0.196 - 0.861)	0.453 (0.196 - 0.864)
70+ years (rate per capita)	0.434 (0.400 - 0.471)	0.428 (0.395 - 0.463)	0.425 (0.393 - 0.459)	0.987 (0.895 - 1.073)	0.982 (0.889 - 1.067)	0.979 (0.888 - 1.063)	0.406 (0.181 - 0.771)	0.400 (0.178 - 0.767)	0.397 (0.176 - 0.761)
All ages (rate per capita)	0.424 (0.402 - 0.444)	0.427 (0.406 - 0.448)	0.426 (0.405 - 0.447)	0.786 (0.741 - 0.832)	0.801 (0.756 - 0.846)	0.808 (0.762 - 0.852)	0.415 (0.184 - 0.783)	0.418 (0.184 - 0.788)	0.416 (0.184 - 0.790)
Age-standardized (rate per capita)	0.404 (0.381 - 0.425)	0.403 (0.381 - 0.424)	0.403 (0.381 - 0.424)	0.729 (0.685 - 0.775)	0.730 (0.686 - 0.775)	0.730 (0.686 - 0.776)	0.396 (0.176 - 0.757)	0.396 (0.174 - 0.747)	0.396 (0.174 - 0.747)
Total number of cases (thousands)	20,221 (19,164 - 21,177)	21,567 (20,493 - 22,609)	22,994 (21,858 - 24,124)	37,506 (35,357 - 39,670)	40,428 (38,149 - 42,698)	43,617 (41,149 - 46,018)	20 (9 - 37)	21 (9 - 40)	22 (10 - 43)
**Northern Ireland**									
5 -14 years (rate per capita)	0.279 (0.224 - 0.343)	0.274 (0.217 - 0.339)	0.273 (0.216 - 0.338)	0.382 (0.289 - 0.508)	0.385 (0.289 - 0.510)	0.383 (0.289 - 0.508)	0.278 (0.123 - 0.540)	0.273 (0.118 - 0.523)	0.272 (0.117 - 0.527)
15 - 49 years (rate per capita)	0.499 (0.465 - 0.535)	0.501 (0.468 - 0.539)	0.501 (0.467 - 0.539)	0.848 (0.775 - 0.918)	0.858 (0.793 - 0.928)	0.864 (0.799 - 0.932)	0.493 (0.215 - 0.934)	0.495 (0.218 - 0.943)	0.495 (0.217 - 0.946)
50 - 69 years (rate per capita)	0.470 (0.429 - 0.513)	0.470 (0.427 - 0.513)	0.470 (0.428 - 0.514)	1.015 (0.911 - 1.114)	1.015 (0.909 - 1.113)	1.015 (0.909 - 1.112)	0.455 (0.198 - 0.863)	0.455 (0.198 - 0.869)	0.455 (0.196 - 0.872)
70+ years (rate per capita)	0.439 (0.404 - 0.476)	0.434 (0.401 - 0.470)	0.432 (0.399 - 0.467)	0.992 (0.897 - 1.078)	0.988 (0.894 - 1.077)	0.985 (0.893 - 1.072)	0.410 (0.182 - 0.787)	0.405 (0.183 - 0.779)	0.401 (0.178 - 0.769)
All ages (rate per capita)	0.411 (0.389 - 0.432)	0.425 (0.403 - 0.445)	0.426 (0.405 - 0.447)	0.743 (0.699 - 0.788)	0.782 (0.737 - 0.827)	0.798 (0.752 - 0.843)	0.403 (0.177 - 0.767)	0.415 (0.182 - 0.781)	0.416 (0.184 - 0.790)
Age-standardized (rate per capita)	0.408 (0.386 - 0.429)	0.406 (0.385 - 0.427)	0.406 (0.385 - 0.427)	0.731 (0.686 - 0.777)	0.731 (0.688 - 0.777)	0.731 (0.688 - 0.777)	0.400 (0.177 - 0.764)	0.398 (0.175 - 0.754)	0.398 (0.175 - 0.755)
Total number of cases (thousands)	657 (622 - 690)	732 (695 - 768)	781 (742 - 819)	1,188 (1,117 - 1,259)	1,347 (1,270 - 1,425)	1,462 (1,377 - 1,544)	1 (0 - 1)	1 (0 - 1)	1 (0 - 1)
**Scotland**									
5 -14 years (rate per capita)	0.367 (0.309 - 0.420)	0.378 (0.316 - 0.434)	0.374 (0.313 - 0.429)	0.452 (0.341 - 0.563)	0.458 (0.358 - 0.565)	0.455 (0.354 - 0.562)	0.366 (0.161 - 0.708)	0.376 (0.167 - 0.721)	0.372 (0.164 - 0.723)
15 - 49 years (rate per capita)	0.588 (0.561 - 0.617)	0.601 (0.575 - 0.627)	0.600 (0.574 - 0.627)	0.846 (0.785 - 0.906)	0.848 (0.788 - 0.906)	0.850 (0.790 - 0.908)	0.580 (0.255 - 1.099)	0.592 (0.259 - 1.119)	0.592 (0.259 - 1.134)
50 - 69 years (rate per capita)	0.556 (0.522 - 0.588)	0.568 (0.536 - 0.601)	0.568 (0.536 - 0.602)	0.992 (0.910 - 1.069)	0.982 (0.904 - 1.051)	0.981 (0.905 - 1.051)	0.537 (0.235 - 1.012)	0.548 (0.242 - 1.034)	0.550 (0.241 - 1.036)
70+ years (rate per capita)	0.518 (0.490 - 0.547)	0.522 (0.495 - 0.551)	0.520 (0.494 - 0.548)	0.984 (0.907 - 1.050)	0.980 (0.913 - 1.039)	0.978 (0.912 - 1.036)	0.483 (0.216 - 0.911)	0.484 (0.218 - 0.899)	0.482 (0.215 - 0.897)
All ages (rate per capita)	0.509 (0.491 - 0.526)	0.527 (0.509 - 0.544)	0.528 (0.510 - 0.545)	0.787 (0.746 - 0.828)	0.805 (0.762 - 0.846)	0.817 (0.774 - 0.860)	0.497 (0.220 - 0.938)	0.513 (0.227 - 0.970)	0.515 (0.226 - 0.978)
Age-standardized (rate per capita)	0.486 (0.467 - 0.504)	0.496 (0.477 - 0.514)	0.496 (0.477 - 0.514)	0.735 (0.693 - 0.775)	0.730 (0.689 - 0.770)	0.730 (0.689 - 0.770)	0.477 (0.210 - 0.894)	0.485 (0.213 - 0.916)	0.486 (0.213 - 0.918)
Total number of cases (thousands)	2,587 (2,494 - 2,674)	2,676 (2,587 - 2,764)	2,810 (2,716 - 2,902)	4,001 (3,788 - 4,208)	4,090 (3,872 - 4,300)	4,348 (4,121 - 4,576)	3 (1 - 5)	3 (1 - 5)	3 (1 - 5)
**Wales**									
5 -14 years (rate per capita)	0.277 (0.223 - 0.341)	0.276 (0.219 - 0.342)	0.273 (0.217 - 0.339)	0.380 (0.287 - 0.506)	0.388 (0.292 - 0.511)	0.384 (0.289 - 0.509)	0.276 (0.123 - 0.534)	0.275 (0.118 - 0.533)	0.273 (0.118 - 0.522)
15 - 49 years (rate per capita)	0.502 (0.468 - 0.538)	0.501 (0.467 - 0.539)	0.498 (0.463 - 0.535)	0.857 (0.786 - 0.924)	0.862 (0.796 - 0.932)	0.863 (0.796 - 0.930)	0.495 (0.216 - 0.940)	0.494 (0.217 - 0.941)	0.491 (0.217 - 0.940)
50 - 69 years (rate per capita)	0.470 (0.429 - 0.513)	0.469 (0.427 - 0.512)	0.470 (0.428 - 0.514)	1.017 (0.913 - 1.114)	1.016 (0.910 - 1.116)	1.017 (0.912 - 1.115)	0.454 (0.200 - 0.864)	0.454 (0.199 - 0.868)	0.454 (0.198 - 0.871)
70+ years (rate per capita)	0.439 (0.404 - 0.476)	0.432 (0.399 - 0.466)	0.430 (0.398 - 0.465)	0.991 (0.897 - 1.078)	0.985 (0.892 - 1.071)	0.983 (0.891 - 1.069)	0.408 (0.180 - 0.758)	0.400 (0.178 - 0.769)	0.399 (0.179 - 0.759)
All ages (rate per capita)	0.426 (0.405 - 0.447)	0.430 (0.409 - 0.451)	0.431 (0.409 - 0.452)	0.791 (0.745 - 0.836)	0.810 (0.764 - 0.855)	0.823 (0.776 - 0.868)	0.416 (0.184 - 0.785)	0.419 (0.184 - 0.788)	0.420 (0.186 - 0.800)
Age-standardized (rate per capita)	0.408 (0.386 - 0.429)	0.406 (0.385 - 0.427)	0.406 (0.385 - 0.427)	0.731 (0.686 - 0.777)	0.731 (0.688 - 0.777)	0.731 (0.688 - 0.777)	0.400 (0.177 - 0.762)	0.398 (0.175 - 0.751)	0.398 (0.175 - 0.752)
Total number of cases (thousands)	1,219 (1,156 - 1,277)	1,273 (1,212 - 1,334)	1,326 (1,260 - 1,392)	2,262 (2,130 - 2,390)	2,398 (2,262 - 2,530)	2,532 (2,387 - 2,670)	1 (1 - 2)	1 (1 - 2)	1 (1 - 2)
**Southern Latin America**									
5 -14 years (rate per capita)	0.288 (0.229 - 0.355)	0.292 (0.233 - 0.359)	0.288 (0.229 - 0.354)	0.340 (0.254 - 0.456)	0.343 (0.256 - 0.462)	0.340 (0.254 - 0.456)	0.288 (0.124 - 0.567)	0.291 (0.127 - 0.577)	0.288 (0.125 - 0.572)
15 - 49 years (rate per capita)	0.513 (0.479 - 0.548)	0.514 (0.479 - 0.548)	0.517 (0.482 - 0.552)	0.749 (0.685 - 0.815)	0.753 (0.690 - 0.820)	0.759 (0.695 - 0.827)	0.508 (0.223 - 0.965)	0.509 (0.222 - 0.955)	0.512 (0.224 - 0.971)
50 - 69 years (rate per capita)	0.471 (0.426 - 0.516)	0.471 (0.425 - 0.517)	0.471 (0.425 - 0.516)	0.926 (0.825 - 1.028)	0.925 (0.825 - 1.027)	0.925 (0.824 - 1.028)	0.456 (0.198 - 0.872)	0.456 (0.199 - 0.863)	0.456 (0.197 - 0.876)
70+ years (rate per capita)	0.431 (0.393 - 0.469)	0.426 (0.390 - 0.461)	0.421 (0.386 - 0.456)	0.860 (0.759 - 0.960)	0.854 (0.759 - 0.949)	0.848 (0.756 - 0.940)	0.403 (0.181 - 0.761)	0.398 (0.178 - 0.743)	0.393 (0.177 - 0.741)
All ages (rate per capita)	0.405 (0.382 - 0.427)	0.418 (0.394 - 0.440)	0.424 (0.403 - 0.446)	0.624 (0.586 - 0.667)	0.652 (0.612 - 0.696)	0.670 (0.630 - 0.712)	0.398 (0.176 - 0.763)	0.411 (0.181 - 0.784)	0.417 (0.184 - 0.792)
Age-standardized (rate per capita)	0.416 (0.394 - 0.438)	0.416 (0.394 - 0.438)	0.416 (0.394 - 0.437)	0.652 (0.613 - 0.694)	0.653 (0.613 - 0.697)	0.653 (0.613 - 0.695)	0.409 (0.181 - 0.777)	0.409 (0.180 - 0.780)	0.409 (0.181 - 0.779)
Total number of cases (thousands)	19,823 (18,733 - 20,932)	24,457 (23,101 - 25,753)	27,483 (26,100 - 28,880)	30,579 (28,693 - 32,648)	38,199 (35,849 - 40,776)	43,447 (40,834 - 46,152)	20 (9 - 37)	24 (11 - 46)	27 (12 - 51)
**Argentina**									
5 -14 years (rate per capita)	0.290 (0.229 - 0.355)	0.292 (0.233 - 0.358)	0.288 (0.228 - 0.353)	0.342 (0.256 - 0.463)	0.344 (0.255 - 0.466)	0.340 (0.255 - 0.461)	0.289 (0.125 - 0.569)	0.292 (0.127 - 0.579)	0.287 (0.123 - 0.570)
15 - 49 years (rate per capita)	0.512 (0.479 - 0.548)	0.513 (0.477 - 0.548)	0.516 (0.483 - 0.553)	0.750 (0.687 - 0.817)	0.751 (0.684 - 0.816)	0.756 (0.693 - 0.824)	0.507 (0.222 - 0.962)	0.508 (0.222 - 0.958)	0.512 (0.224 - 0.968)
50 - 69 years (rate per capita)	0.471 (0.426 - 0.516)	0.471 (0.426 - 0.518)	0.471 (0.426 - 0.516)	0.925 (0.824 - 1.030)	0.924 (0.818 - 1.032)	0.925 (0.824 - 1.030)	0.457 (0.199 - 0.867)	0.457 (0.198 - 0.869)	0.457 (0.197 - 0.877)
70+ years (rate per capita)	0.432 (0.392 - 0.473)	0.426 (0.390 - 0.463)	0.421 (0.385 - 0.457)	0.862 (0.758 - 0.965)	0.854 (0.761 - 0.951)	0.849 (0.752 - 0.942)	0.404 (0.182 - 0.772)	0.398 (0.177 - 0.746)	0.394 (0.175 - 0.740)
All ages (rate per capita)	0.403 (0.381 - 0.426)	0.414 (0.390 - 0.437)	0.419 (0.398 - 0.442)	0.624 (0.585 - 0.667)	0.645 (0.605 - 0.689)	0.659 (0.619 - 0.700)	0.396 (0.175 - 0.759)	0.407 (0.178 - 0.773)	0.412 (0.182 - 0.786)
Age-standardized (rate per capita)	0.416 (0.395 - 0.438)	0.416 (0.393 - 0.439)	0.416 (0.394 - 0.438)	0.653 (0.613 - 0.694)	0.653 (0.611 - 0.697)	0.653 (0.613 - 0.694)	0.409 (0.181 - 0.776)	0.409 (0.179 - 0.776)	0.409 (0.181 - 0.780)
Total number of cases (thousands)	13,178 (12,455 - 13,929)	16,195 (15,263 - 17,094)	18,203 (17,267 - 19,177)	20,420 (19,133 - 21,818)	25,258 (23,663 - 26,956)	28,592 (26,871 - 30,404)	13 (6 - 25)	16 (7 - 30)	18 (8 - 34)
**Chile**									
5 -14 years (rate per capita)	0.283 (0.223 - 0.351)	0.290 (0.228 - 0.359)	0.289 (0.227 - 0.357)	0.336 (0.252 - 0.455)	0.342 (0.256 - 0.459)	0.340 (0.255 - 0.459)	0.283 (0.125 - 0.556)	0.289 (0.126 - 0.572)	0.289 (0.126 - 0.563)
15 - 49 years (rate per capita)	0.514 (0.479 - 0.550)	0.515 (0.481 - 0.549)	0.517 (0.483 - 0.552)	0.746 (0.680 - 0.817)	0.759 (0.697 - 0.827)	0.764 (0.701 - 0.833)	0.509 (0.223 - 0.957)	0.510 (0.223 - 0.951)	0.512 (0.224 - 0.967)
50 - 69 years (rate per capita)	0.471 (0.424 - 0.517)	0.470 (0.423 - 0.517)	0.470 (0.423 - 0.516)	0.926 (0.829 - 1.029)	0.925 (0.826 - 1.028)	0.926 (0.828 - 1.030)	0.456 (0.196 - 0.865)	0.456 (0.196 - 0.863)	0.456 (0.195 - 0.868)
70+ years (rate per capita)	0.428 (0.389 - 0.465)	0.425 (0.387 - 0.461)	0.421 (0.385 - 0.456)	0.856 (0.758 - 0.956)	0.853 (0.757 - 0.950)	0.848 (0.755 - 0.943)	0.399 (0.179 - 0.755)	0.396 (0.179 - 0.746)	0.392 (0.177 - 0.742)
All ages (rate per capita)	0.407 (0.384 - 0.431)	0.426 (0.403 - 0.449)	0.435 (0.412 - 0.458)	0.617 (0.575 - 0.661)	0.665 (0.621 - 0.710)	0.696 (0.652 - 0.742)	0.401 (0.177 - 0.768)	0.419 (0.184 - 0.796)	0.428 (0.188 - 0.807)
Age-standardized (rate per capita)	0.415 (0.393 - 0.438)	0.415 (0.393 - 0.438)	0.415 (0.393 - 0.438)	0.652 (0.609 - 0.694)	0.652 (0.609 - 0.694)	0.652 (0.610 - 0.694)	0.408 (0.181 - 0.778)	0.408 (0.180 - 0.777)	0.408 (0.179 - 0.779)
Total number of cases (thousands)	5,354 (5,044 - 5,672)	6,857 (6,482 - 7,234)	7,815 (7,400 - 8,222)	8,116 (7,556 - 8,686)	10,704 (10,003 - 11,437)	12,493 (11,711 - 13,324)	5 (2 - 10)	7 (3 - 13)	8 (3 - 14)

	Prevalence 1990	Prevalence 2005	Prevalence 2015	Incidence 1990	Incidence 2005	Incidence 2015	YLD rate 1990	YLD rate 2005	YLD rate 2015
**Uruguay**									
5 -14 years (rate per capita)	0.291 (0.229 - 0.359)	0.288 (0.227 - 0.357)	0.288 (0.226 - 0.356)	0.342 (0.256 - 0.459)	0.340 (0.254 - 0.458)	0.340 (0.254 - 0.458)	0.290 (0.127 - 0.569)	0.287 (0.127 - 0.558)	0.287 (0.126 - 0.556)
15 - 49 years (rate per capita)	0.513 (0.478 - 0.548)	0.515 (0.481 - 0.550)	0.514 (0.480 - 0.549)	0.750 (0.688 - 0.819)	0.757 (0.694 - 0.825)	0.759 (0.697 - 0.828)	0.507 (0.221 - 0.948)	0.510 (0.223 - 0.969)	0.510 (0.222 - 0.953)
50 - 69 years (rate per capita)	0.470 (0.425 - 0.517)	0.471 (0.424 - 0.517)	0.470 (0.424 - 0.517)	0.928 (0.831 - 1.027)	0.927 (0.832 - 1.026)	0.927 (0.831 - 1.027)	0.455 (0.196 - 0.864)	0.456 (0.195 - 0.874)	0.456 (0.193 - 0.865)
70+ years (rate per capita)	0.428 (0.390 - 0.466)	0.425 (0.388 - 0.461)	0.416 (0.383 - 0.450)	0.857 (0.758 - 0.957)	0.852 (0.757 - 0.950)	0.842 (0.753 - 0.936)	0.400 (0.180 - 0.744)	0.396 (0.179 - 0.742)	0.388 (0.174 - 0.722)
All ages (rate per capita)	0.415 (0.392 - 0.437)	0.421 (0.399 - 0.443)	0.426 (0.404 - 0.448)	0.656 (0.614 - 0.698)	0.671 (0.629 - 0.714)	0.687 (0.644 - 0.730)	0.407 (0.179 - 0.774)	0.413 (0.181 - 0.783)	0.418 (0.183 - 0.788)
Age-standardized (rate per capita)	0.415 (0.393 - 0.438)	0.415 (0.393 - 0.438)	0.415 (0.393 - 0.438)	0.652 (0.609 - 0.694)	0.652 (0.609 - 0.694)	0.652 (0.609 - 0.694)	0.408 (0.180 - 0.775)	0.408 (0.180 - 0.776)	0.408 (0.179 - 0.775)
Total number of cases (thousands)	1,290 (1,219 - 1,360)	1,404 (1,329 - 1,475)	1,464 (1,387 - 1,538)	2,042 (1,910 - 2,172)	2,236 (2,096 - 2,378)	2,360 (2,212 - 2,508)	1 (1 - 2)	1 (1 - 3)	1 (1 - 3)
**Central Europe, Eastern Europe, and Central Asia**									
5 -14 years (rate per capita)	0.377 (0.318 - 0.435)	0.388 (0.327 - 0.450)	0.363 (0.304 - 0.423)	0.606 (0.481 - 0.727)	0.617 (0.490 - 0.745)	0.592 (0.468 - 0.714)	0.338 (0.150 - 0.655)	0.336 (0.149 - 0.655)	0.314 (0.136 - 0.605)
15 - 49 years (rate per capita)	0.512 (0.473 - 0.551)	0.511 (0.472 - 0.548)	0.507 (0.467 - 0.546)	0.846 (0.769 - 0.923)	0.845 (0.770 - 0.918)	0.848 (0.772 - 0.924)	0.464 (0.200 - 0.882)	0.457 (0.199 - 0.874)	0.449 (0.196 - 0.858)
50 - 69 years (rate per capita)	0.475 (0.426 - 0.524)	0.476 (0.426 - 0.525)	0.476 (0.423 - 0.527)	0.939 (0.828 - 1.048)	0.937 (0.828 - 1.042)	0.935 (0.818 - 1.046)	0.427 (0.185 - 0.812)	0.426 (0.186 - 0.813)	0.423 (0.185 - 0.819)
70+ years (rate per capita)	0.453 (0.412 - 0.494)	0.460 (0.416 - 0.501)	0.450 (0.410 - 0.491)	0.926 (0.823 - 1.022)	0.927 (0.822 - 1.030)	0.925 (0.824 - 1.021)	0.393 (0.176 - 0.745)	0.396 (0.178 - 0.745)	0.391 (0.176 - 0.735)
All ages (rate per capita)	0.435 (0.411 - 0.459)	0.455 (0.429 - 0.479)	0.445 (0.421 - 0.469)	0.758 (0.709 - 0.803)	0.794 (0.745 - 0.841)	0.793 (0.743 - 0.840)	0.392 (0.173 - 0.743)	0.405 (0.180 - 0.763)	0.393 (0.175 - 0.742)
Age-standardized (rate per capita)	0.432 (0.408 - 0.456)	0.431 (0.406 - 0.455)	0.431 (0.406 - 0.455)	0.750 (0.701 - 0.794)	0.748 (0.699 - 0.794)	0.747 (0.699 - 0.792)	0.389 (0.172 - 0.737)	0.383 (0.169 - 0.725)	0.379 (0.167 - 0.724)
Total number of cases (thousands)	179,812 (169,814 - 189,705)	184,828 (174,195 - 194,658)	186,352 (176,286 - 196,280)	313,353 (292,754 - 331,825)	322,493 (302,551 - 341,654)	332,147 (311,217 - 351,664)	162 (71 - 307)	165 (73 - 310)	165 (73 - 311)
**Eastern Europe**									
5 -14 years (rate per capita)	0.370 (0.311 - 0.428)	0.386 (0.326 - 0.446)	0.351 (0.294 - 0.410)	0.618 (0.493 - 0.738)	0.637 (0.513 - 0.764)	0.601 (0.474 - 0.728)	0.363 (0.161 - 0.707)	0.378 (0.167 - 0.744)	0.345 (0.151 - 0.674)
15 - 49 years (rate per capita)	0.503 (0.466 - 0.538)	0.503 (0.469 - 0.537)	0.499 (0.461 - 0.535)	0.879 (0.803 - 0.954)	0.879 (0.804 - 0.950)	0.884 (0.808 - 0.960)	0.488 (0.211 - 0.916)	0.489 (0.215 - 0.935)	0.485 (0.210 - 0.923)
50 - 69 years (rate per capita)	0.466 (0.422 - 0.513)	0.467 (0.421 - 0.512)	0.468 (0.421 - 0.515)	0.968 (0.860 - 1.074)	0.969 (0.863 - 1.069)	0.968 (0.852 - 1.075)	0.439 (0.193 - 0.825)	0.440 (0.193 - 0.839)	0.442 (0.193 - 0.842)
70+ years (rate per capita)	0.442 (0.406 - 0.480)	0.448 (0.411 - 0.488)	0.442 (0.405 - 0.482)	0.954 (0.851 - 1.050)	0.960 (0.854 - 1.059)	0.955 (0.853 - 1.052)	0.399 (0.179 - 0.753)	0.404 (0.181 - 0.764)	0.400 (0.178 - 0.753)
All ages (rate per capita)	0.433 (0.411 - 0.456)	0.454 (0.432 - 0.476)	0.442 (0.420 - 0.463)	0.800 (0.751 - 0.844)	0.839 (0.788 - 0.888)	0.837 (0.786 - 0.885)	0.417 (0.184 - 0.785)	0.436 (0.192 - 0.834)	0.424 (0.186 - 0.802)
Age-standardized (rate per capita)	0.425 (0.403 - 0.447)	0.425 (0.402 - 0.447)	0.424 (0.401 - 0.446)	0.774 (0.725 - 0.819)	0.774 (0.726 - 0.822)	0.774 (0.725 - 0.820)	0.409 (0.180 - 0.772)	0.409 (0.181 - 0.780)	0.408 (0.180 - 0.778)
Total number of cases (thousands)	95,881 (90,953 - 100,781)	95,856 (91,071 - 100,327)	95,134 (90,344 - 99,592)	176,960 (166,209 - 186,665)	177,047 (166,342 - 187,260)	180,060 (168,962 - 190,305)	92 (41 - 174)	92 (41 - 176)	91 (40 - 172)
**Belarus**									
5 -14 years (rate per capita)	0.316 (0.256 - 0.384)	0.336 (0.271 - 0.406)	0.318 (0.258 - 0.386)	0.569 (0.442 - 0.707)	0.595 (0.463 - 0.738)	0.573 (0.445 - 0.710)	0.189 (0.080 - 0.377)	0.201 (0.087 - 0.408)	0.191 (0.081 - 0.376)
15 - 49 years (rate per capita)	0.453 (0.406 - 0.499)	0.453 (0.408 - 0.495)	0.451 (0.406 - 0.494)	0.831 (0.741 - 0.927)	0.832 (0.745 - 0.921)	0.835 (0.746 - 0.933)	0.269 (0.118 - 0.526)	0.269 (0.117 - 0.529)	0.267 (0.117 - 0.525)
50 - 69 years (rate per capita)	0.422 (0.364 - 0.477)	0.422 (0.363 - 0.477)	0.421 (0.361 - 0.481)	0.909 (0.778 - 1.036)	0.908 (0.779 - 1.030)	0.905 (0.766 - 1.039)	0.243 (0.107 - 0.475)	0.243 (0.106 - 0.472)	0.243 (0.105 - 0.477)
70+ years (rate per capita)	0.395 (0.352 - 0.445)	0.405 (0.357 - 0.459)	0.395 (0.352 - 0.444)	0.888 (0.767 - 1.006)	0.898 (0.770 - 1.024)	0.888 (0.770 - 1.003)	0.218 (0.099 - 0.417)	0.223 (0.101 - 0.435)	0.218 (0.100 - 0.413)
All ages (rate per capita)	0.388 (0.360 - 0.417)	0.408 (0.379 - 0.436)	0.399 (0.371 - 0.426)	0.750 (0.697 - 0.806)	0.790 (0.732 - 0.845)	0.788 (0.731 - 0.844)	0.228 (0.100 - 0.431)	0.239 (0.105 - 0.454)	0.234 (0.101 - 0.443)
Age-standardized (rate per capita)	0.381 (0.352 - 0.409)	0.381 (0.352 - 0.409)	0.381 (0.352 - 0.409)	0.728 (0.676 - 0.783)	0.729 (0.676 - 0.784)	0.729 (0.676 - 0.784)	0.224 (0.098 - 0.425)	0.224 (0.098 - 0.425)	0.224 (0.098 - 0.425)
Total number of cases (thousands)	3,933 (3,650 - 4,225)	3,944 (3,667 - 4,218)	3,838 (3,564 - 4,099)	7,611 (7,070 - 8,171)	7,637 (7,082 - 8,169)	7,577 (7,022 - 8,112)	2 (1 - 4)	2 (1 - 4)	2 (1 - 4)
**Estonia**									
5 -14 years (rate per capita)	0.223 (0.179 - 0.277)	0.219 (0.178 - 0.267)	0.215 (0.172 - 0.267)	0.483 (0.368 - 0.622)	0.480 (0.369 - 0.611)	0.469 (0.358 - 0.606)	0.223 (0.096 - 0.431)	0.218 (0.092 - 0.422)	0.215 (0.093 - 0.415)
15 - 49 years (rate per capita)	0.335 (0.306 - 0.365)	0.335 (0.305 - 0.368)	0.331 (0.302 - 0.361)	0.781 (0.703 - 0.860)	0.779 (0.706 - 0.854)	0.782 (0.703 - 0.859)	0.331 (0.141 - 0.630)	0.331 (0.143 - 0.632)	0.328 (0.141 - 0.630)
50 - 69 years (rate per capita)	0.304 (0.269 - 0.343)	0.304 (0.268 - 0.340)	0.304 (0.268 - 0.343)	0.828 (0.731 - 0.937)	0.830 (0.739 - 0.935)	0.827 (0.727 - 0.943)	0.291 (0.127 - 0.559)	0.292 (0.127 - 0.557)	0.292 (0.126 - 0.567)
70+ years (rate per capita)	0.283 (0.255 - 0.312)	0.286 (0.258 - 0.319)	0.281 (0.253 - 0.309)	0.793 (0.708 - 0.886)	0.801 (0.709 - 0.896)	0.790 (0.707 - 0.879)	0.260 (0.114 - 0.496)	0.264 (0.119 - 0.502)	0.260 (0.113 - 0.493)
All ages (rate per capita)	0.283 (0.264 - 0.302)	0.294 (0.274 - 0.312)	0.289 (0.270 - 0.307)	0.692 (0.646 - 0.740)	0.724 (0.676 - 0.773)	0.722 (0.676 - 0.770)	0.277 (0.121 - 0.525)	0.287 (0.125 - 0.549)	0.282 (0.122 - 0.539)
Age-standardized (rate per capita)	0.277 (0.258 - 0.296)	0.275 (0.256 - 0.293)	0.277 (0.258 - 0.296)	0.668 (0.621 - 0.717)	0.663 (0.618 - 0.713)	0.668 (0.621 - 0.717)	0.272 (0.118 - 0.515)	0.269 (0.118 - 0.516)	0.273 (0.119 - 0.518)
Total number of cases (thousands)	443 (412 - 471)	396 (370 - 421)	391 (365 - 416)	1,081 (1,010 - 1,156)	975 (911 - 1,041)	978 (916 - 1,042)	0 (0 - 1)	0 (0 - 1)	0 (0 - 1)
**Latvia**									
5 -14 years (rate per capita)	0.317 (0.256 - 0.384)	0.342 (0.275 - 0.413)	0.304 (0.246 - 0.371)	0.591 (0.457 - 0.740)	0.626 (0.484 - 0.781)	0.574 (0.441 - 0.721)	0.316 (0.139 - 0.635)	0.342 (0.150 - 0.680)	0.304 (0.133 - 0.603)
15 - 49 years (rate per capita)	0.452 (0.408 - 0.496)	0.453 (0.408 - 0.497)	0.450 (0.405 - 0.494)	0.875 (0.779 - 0.974)	0.874 (0.777 - 0.971)	0.878 (0.780 - 0.978)	0.447 (0.194 - 0.865)	0.448 (0.195 - 0.863)	0.446 (0.193 - 0.859)
50 - 69 years (rate per capita)	0.422 (0.363 - 0.479)	0.422 (0.365 - 0.478)	0.421 (0.363 - 0.477)	0.950 (0.807 - 1.086)	0.952 (0.815 - 1.085)	0.949 (0.807 - 1.087)	0.404 (0.173 - 0.772)	0.405 (0.176 - 0.780)	0.405 (0.173 - 0.783)
70+ years (rate per capita)	0.395 (0.352 - 0.444)	0.401 (0.356 - 0.453)	0.395 (0.351 - 0.444)	0.924 (0.799 - 1.049)	0.933 (0.803 - 1.063)	0.925 (0.801 - 1.051)	0.362 (0.161 - 0.687)	0.369 (0.165 - 0.703)	0.364 (0.162 - 0.689)
All ages (rate per capita)	0.389 (0.362 - 0.417)	0.409 (0.381 - 0.437)	0.402 (0.373 - 0.429)	0.793 (0.732 - 0.851)	0.835 (0.772 - 0.898)	0.836 (0.772 - 0.896)	0.380 (0.168 - 0.734)	0.399 (0.176 - 0.763)	0.392 (0.171 - 0.755)
Age-standardized (rate per capita)	0.381 (0.352 - 0.409)	0.381 (0.352 - 0.409)	0.381 (0.352 - 0.409)	0.763 (0.703 - 0.820)	0.763 (0.703 - 0.820)	0.763 (0.703 - 0.821)	0.373 (0.165 - 0.722)	0.373 (0.165 - 0.719)	0.373 (0.165 - 0.722)
Total number of cases (thousands)	1,031 (959 - 1,105)	941 (876 - 1,004)	890 (826 - 948)	2,100 (1,939 - 2,255)	1,921 (1,776 - 2,064)	1,848 (1,708 - 1,982)	1 (0 - 2)	1 (0 - 2)	1 (0 - 2)
**Lithuania**									
5 -14 years (rate per capita)	0.394 (0.334 - 0.443)	0.415 (0.361 - 0.456)	0.374 (0.312 - 0.433)	0.633 (0.519 - 0.748)	0.656 (0.547 - 0.771)	0.624 (0.498 - 0.754)	0.392 (0.173 - 0.759)	0.413 (0.184 - 0.784)	0.374 (0.164 - 0.722)
15 - 49 years (rate per capita)	0.507 (0.471 - 0.542)	0.508 (0.474 - 0.543)	0.501 (0.468 - 0.535)	0.881 (0.807 - 0.950)	0.883 (0.806 - 0.949)	0.885 (0.814 - 0.958)	0.502 (0.219 - 0.945)	0.503 (0.220 - 0.953)	0.496 (0.217 - 0.936)
50 - 69 years (rate per capita)	0.468 (0.425 - 0.509)	0.468 (0.426 - 0.511)	0.467 (0.424 - 0.514)	0.980 (0.875 - 1.087)	0.979 (0.873 - 1.079)	0.978 (0.870 - 1.088)	0.448 (0.196 - 0.856)	0.449 (0.195 - 0.856)	0.449 (0.192 - 0.856)
70+ years (rate per capita)	0.436 (0.404 - 0.471)	0.448 (0.411 - 0.485)	0.438 (0.403 - 0.472)	0.952 (0.856 - 1.039)	0.965 (0.865 - 1.057)	0.955 (0.859 - 1.044)	0.400 (0.180 - 0.759)	0.411 (0.187 - 0.773)	0.402 (0.179 - 0.758)
All ages (rate per capita)	0.439 (0.417 - 0.458)	0.459 (0.439 - 0.478)	0.449 (0.428 - 0.470)	0.803 (0.758 - 0.850)	0.844 (0.795 - 0.889)	0.852 (0.806 - 0.899)	0.429 (0.190 - 0.812)	0.448 (0.198 - 0.852)	0.438 (0.194 - 0.824)
Age-standardized (rate per capita)	0.432 (0.410 - 0.451)	0.432 (0.412 - 0.451)	0.424 (0.402 - 0.446)	0.781 (0.735 - 0.827)	0.781 (0.733 - 0.821)	0.778 (0.731 - 0.823)	0.423 (0.187 - 0.797)	0.423 (0.187 - 0.804)	0.416 (0.183 - 0.793)
Total number of cases (thousands)	1,618 (1,535 - 1,689)	1,568 (1,499 - 1,634)	1,416 (1,349 - 1,482)	2,960 (2,791 - 3,131)	2,883 (2,717 - 3,036)	2,686 (2,540 - 2,834)	2 (1 - 3)	2 (1 - 3)	1 (1 - 3)
**Moldova**									
5 -14 years (rate per capita)	0.315 (0.256 - 0.383)	0.338 (0.273 - 0.408)	0.317 (0.256 - 0.385)	0.589 (0.456 - 0.738)	0.620 (0.481 - 0.775)	0.592 (0.457 - 0.741)	0.315 (0.139 - 0.629)	0.338 (0.147 - 0.674)	0.316 (0.139 - 0.633)
15 - 49 years (rate per capita)	0.454 (0.407 - 0.499)	0.455 (0.410 - 0.500)	0.452 (0.404 - 0.496)	0.873 (0.772 - 0.974)	0.870 (0.773 - 0.965)	0.876 (0.775 - 0.976)	0.449 (0.194 - 0.854)	0.450 (0.195 - 0.866)	0.447 (0.193 - 0.857)
50 - 69 years (rate per capita)	0.422 (0.364 - 0.477)	0.421 (0.363 - 0.476)	0.421 (0.362 - 0.478)	0.950 (0.810 - 1.084)	0.946 (0.810 - 1.080)	0.949 (0.804 - 1.088)	0.404 (0.175 - 0.773)	0.405 (0.176 - 0.771)	0.405 (0.175 - 0.776)
70+ years (rate per capita)	0.404 (0.357 - 0.458)	0.405 (0.357 - 0.460)	0.398 (0.353 - 0.449)	0.936 (0.803 - 1.069)	0.938 (0.803 - 1.070)	0.929 (0.802 - 1.056)	0.371 (0.167 - 0.708)	0.374 (0.166 - 0.716)	0.367 (0.163 - 0.695)
All ages (rate per capita)	0.376 (0.348 - 0.405)	0.406 (0.375 - 0.435)	0.402 (0.372 - 0.431)	0.753 (0.695 - 0.811)	0.810 (0.744 - 0.871)	0.821 (0.756 - 0.881)	0.369 (0.161 - 0.715)	0.398 (0.177 - 0.762)	0.394 (0.173 - 0.753)
Age-standardized (rate per capita)	0.381 (0.352 - 0.409)	0.381 (0.352 - 0.409)	0.381 (0.352 - 0.409)	0.763 (0.703 - 0.821)	0.763 (0.703 - 0.821)	0.763 (0.703 - 0.821)	0.373 (0.162 - 0.718)	0.373 (0.165 - 0.721)	0.373 (0.165 - 0.717)
Total number of cases (thousands)	1,642 (1,520 - 1,767)	1,692 (1,561 - 1,814)	1,635 (1,514 - 1,753)	3,287 (3,032 - 3,539)	3,375 (3,103 - 3,629)	3,336 (3,075 - 3,582)	2 (1 - 3)	2 (1 - 3)	2 (1 - 3)
**Russia**									
5 -14 years (rate per capita)	0.394 (0.336 - 0.450)	0.409 (0.351 - 0.466)	0.366 (0.307 - 0.423)	0.632 (0.510 - 0.747)	0.648 (0.527 - 0.770)	0.609 (0.477 - 0.732)	0.393 (0.175 - 0.760)	0.408 (0.180 - 0.799)	0.365 (0.160 - 0.711)
15 - 49 years (rate per capita)	0.526 (0.493 - 0.561)	0.526 (0.493 - 0.559)	0.521 (0.486 - 0.555)	0.885 (0.813 - 0.956)	0.884 (0.813 - 0.955)	0.891 (0.819 - 0.962)	0.520 (0.226 - 0.970)	0.519 (0.228 - 0.983)	0.515 (0.223 - 0.976)
50 - 69 years (rate per capita)	0.490 (0.447 - 0.532)	0.490 (0.448 - 0.532)	0.489 (0.445 - 0.535)	0.981 (0.877 - 1.086)	0.982 (0.881 - 1.079)	0.980 (0.867 - 1.085)	0.469 (0.205 - 0.879)	0.469 (0.205 - 0.892)	0.470 (0.207 - 0.895)
70+ years (rate per capita)	0.467 (0.431 - 0.503)	0.473 (0.436 - 0.510)	0.464 (0.428 - 0.502)	0.972 (0.870 - 1.063)	0.977 (0.873 - 1.065)	0.972 (0.877 - 1.057)	0.428 (0.192 - 0.815)	0.434 (0.195 - 0.819)	0.427 (0.191 - 0.800)
All ages (rate per capita)	0.455 (0.433 - 0.475)	0.476 (0.454 - 0.497)	0.461 (0.440 - 0.482)	0.806 (0.760 - 0.850)	0.846 (0.797 - 0.894)	0.843 (0.794 - 0.890)	0.444 (0.195 - 0.835)	0.464 (0.205 - 0.886)	0.449 (0.196 - 0.853)
Age-standardized (rate per capita)	0.447 (0.426 - 0.468)	0.446 (0.424 - 0.466)	0.444 (0.422 - 0.465)	0.783 (0.737 - 0.827)	0.783 (0.735 - 0.829)	0.782 (0.735 - 0.826)	0.437 (0.192 - 0.824)	0.436 (0.193 - 0.832)	0.434 (0.192 - 0.827)

	Prevalence 1990	Prevalence 2005	Prevalence 2015	Incidence 1990	Incidence 2005	Incidence 2015	YLD rate 1990	YLD rate 2005	YLD rate 2015
Total number of cases (thousands)	67,085 (63,950 - 70,124)	68,103 (64,970 - 71,194)	68,275 (65,147 - 71,437)	118,952 (112,088 - 125,385)	121,108 (114,027 - 127,961)	124,830 (117,538 - 131,825)	66 (29 - 123)	66 (29 - 127)	66 (29 - 126)
**Ukraine**									
5 -14 years (rate per capita)	0.319 (0.258 - 0.386)	0.336 (0.271 - 0.406)	0.311 (0.252 - 0.378)	0.594 (0.459 - 0.743)	0.618 (0.480 - 0.772)	0.584 (0.450 - 0.733)	0.318 (0.139 - 0.636)	0.336 (0.148 - 0.673)	0.311 (0.136 - 0.624)
15 - 49 years (rate per capita)	0.453 (0.407 - 0.498)	0.453 (0.408 - 0.496)	0.450 (0.406 - 0.495)	0.874 (0.775 - 0.975)	0.874 (0.777 - 0.970)	0.878 (0.779 - 0.978)	0.448 (0.192 - 0.857)	0.448 (0.195 - 0.866)	0.445 (0.192 - 0.859)
50 - 69 years (rate per capita)	0.422 (0.364 - 0.478)	0.422 (0.365 - 0.476)	0.421 (0.361 - 0.479)	0.949 (0.812 - 1.084)	0.951 (0.814 - 1.080)	0.949 (0.803 - 1.088)	0.404 (0.175 - 0.782)	0.404 (0.175 - 0.771)	0.405 (0.176 - 0.774)
70+ years (rate per capita)	0.399 (0.354 - 0.451)	0.403 (0.356 - 0.456)	0.399 (0.350 - 0.453)	0.930 (0.800 - 1.062)	0.935 (0.803 - 1.068)	0.930 (0.803 - 1.058)	0.367 (0.163 - 0.699)	0.371 (0.166 - 0.706)	0.368 (0.164 - 0.696)
All ages (rate per capita)	0.392 (0.364 - 0.420)	0.409 (0.381 - 0.437)	0.402 (0.374 - 0.429)	0.799 (0.737 - 0.858)	0.834 (0.771 - 0.896)	0.834 (0.770 - 0.894)	0.383 (0.169 - 0.734)	0.399 (0.176 - 0.769)	0.391 (0.171 - 0.753)
Age-standardized (rate per capita)	0.381 (0.352 - 0.409)	0.381 (0.352 - 0.409)	0.381 (0.352 - 0.409)	0.763 (0.703 - 0.820)	0.763 (0.703 - 0.820)	0.763 (0.703 - 0.820)	0.373 (0.164 - 0.718)	0.373 (0.164 - 0.719)	0.373 (0.165 - 0.721)
Total number of cases (thousands)	20,129 (18,663 - 21,543)	19,212 (17,875 - 20,512)	18,688 (17,397 - 19,937)	40,971 (37,817 - 44,036)	39,147 (36,206 - 42,082)	38,804 (35,796 - 41,600)	20 (9 - 38)	19 (8 - 36)	18 (8 - 35)
**Central Europe**									
5 -14 years (rate per capita)	0.402 (0.332 - 0.475)	0.403 (0.327 - 0.482)	0.391 (0.319 - 0.467)	0.609 (0.470 - 0.745)	0.611 (0.468 - 0.751)	0.598 (0.459 - 0.738)	0.368 (0.162 - 0.714)	0.365 (0.158 - 0.717)	0.356 (0.154 - 0.695)
15 - 49 years (rate per capita)	0.530 (0.482 - 0.578)	0.527 (0.478 - 0.575)	0.523 (0.473 - 0.574)	0.810 (0.721 - 0.892)	0.812 (0.724 - 0.897)	0.816 (0.727 - 0.904)	0.480 (0.207 - 0.938)	0.479 (0.208 - 0.923)	0.475 (0.206 - 0.918)
50 - 69 years (rate per capita)	0.497 (0.433 - 0.559)	0.494 (0.429 - 0.558)	0.494 (0.428 - 0.560)	0.897 (0.774 - 1.016)	0.894 (0.765 - 1.021)	0.894 (0.762 - 1.025)	0.441 (0.193 - 0.845)	0.438 (0.189 - 0.832)	0.437 (0.190 - 0.837)
70+ years (rate per capita)	0.475 (0.421 - 0.530)	0.480 (0.422 - 0.536)	0.468 (0.417 - 0.522)	0.883 (0.769 - 0.992)	0.884 (0.768 - 0.995)	0.877 (0.766 - 0.983)	0.409 (0.184 - 0.782)	0.411 (0.186 - 0.784)	0.400 (0.179 - 0.757)
All ages (rate per capita)	0.460 (0.428 - 0.491)	0.474 (0.442 - 0.507)	0.471 (0.439 - 0.504)	0.740 (0.686 - 0.794)	0.774 (0.718 - 0.832)	0.782 (0.724 - 0.842)	0.414 (0.182 - 0.801)	0.426 (0.188 - 0.818)	0.422 (0.186 - 0.807)
Age-standardized (rate per capita)	0.449 (0.418 - 0.481)	0.447 (0.415 - 0.479)	0.447 (0.415 - 0.478)	0.721 (0.667 - 0.775)	0.720 (0.665 - 0.774)	0.719 (0.663 - 0.775)	0.405 (0.179 - 0.783)	0.403 (0.179 - 0.779)	0.402 (0.178 - 0.774)
Total number of cases (thousands)	56,584 (52,655 - 60,424)	56,080 (52,252 - 59,947)	54,936 (51,199 - 58,776)	91,122 (84,447 - 97,726)	91,550 (84,885 - 98,379)	91,277 (84,463 - 98,310)	51 (22 - 99)	50 (22 - 97)	49 (22 - 94)
**Albania**									
5 -14 years (rate per capita)	0.368 (0.276 - 0.466)	0.386 (0.289 - 0.485)	0.390 (0.292 - 0.489)	0.585 (0.427 - 0.758)	0.600 (0.440 - 0.769)	0.604 (0.445 - 0.773)	0.221 (0.093 - 0.440)	0.231 (0.096 - 0.463)	0.234 (0.099 - 0.462)
15 - 49 years (rate per capita)	0.509 (0.441 - 0.575)	0.511 (0.445 - 0.580)	0.510 (0.444 - 0.574)	0.767 (0.654 - 0.877)	0.769 (0.659 - 0.874)	0.769 (0.657 - 0.876)	0.303 (0.130 - 0.601)	0.304 (0.131 - 0.600)	0.303 (0.131 - 0.593)
50 - 69 years (rate per capita)	0.484 (0.391 - 0.574)	0.484 (0.393 - 0.572)	0.485 (0.394 - 0.575)	0.840 (0.670 - 1.010)	0.840 (0.673 - 1.009)	0.840 (0.671 - 1.010)	0.280 (0.120 - 0.575)	0.281 (0.121 - 0.573)	0.281 (0.119 - 0.574)
70+ years (rate per capita)	0.472 (0.388 - 0.557)	0.472 (0.388 - 0.558)	0.467 (0.389 - 0.549)	0.836 (0.676 - 0.990)	0.837 (0.677 - 0.990)	0.835 (0.677 - 0.985)	0.261 (0.117 - 0.492)	0.262 (0.117 - 0.496)	0.259 (0.117 - 0.492)
All ages (rate per capita)	0.416 (0.373 - 0.460)	0.444 (0.400 - 0.489)	0.453 (0.411 - 0.497)	0.650 (0.583 - 0.722)	0.698 (0.630 - 0.771)	0.722 (0.648 - 0.799)	0.246 (0.107 - 0.475)	0.262 (0.115 - 0.509)	0.267 (0.116 - 0.519)
Age-standardized (rate per capita)	0.432 (0.390 - 0.473)	0.432 (0.390 - 0.474)	0.432 (0.390 - 0.474)	0.687 (0.621 - 0.758)	0.686 (0.621 - 0.758)	0.687 (0.622 - 0.758)	0.254 (0.111 - 0.491)	0.255 (0.112 - 0.494)	0.255 (0.111 - 0.493)
Total number of cases (thousands)	1,363 (1,223 - 1,507)	1,371 (1,234 - 1,509)	1,312 (1,191 - 1,438)	2,131 (1,912 - 2,368)	2,153 (1,945 - 2,380)	2,090 (1,876 - 2,314)	1 (0 - 2)	1 (0 - 2)	1 (0 - 2)
**Bosnia and Herzegovina**									
5 -14 years (rate per capita)	0.454 (0.393 - 0.512)	0.462 (0.402 - 0.522)	0.454 (0.394 - 0.513)	0.571 (0.464 - 0.677)	0.576 (0.472 - 0.683)	0.572 (0.465 - 0.678)	0.272 (0.117 - 0.526)	0.276 (0.118 - 0.534)	0.272 (0.116 - 0.535)
15 - 49 years (rate per capita)	0.599 (0.566 - 0.632)	0.596 (0.564 - 0.628)	0.593 (0.561 - 0.626)	0.751 (0.694 - 0.811)	0.757 (0.701 - 0.816)	0.761 (0.705 - 0.820)	0.356 (0.155 - 0.699)	0.353 (0.155 - 0.686)	0.351 (0.153 - 0.689)
50 - 69 years (rate per capita)	0.552 (0.507 - 0.597)	0.553 (0.508 - 0.597)	0.553 (0.510 - 0.597)	0.865 (0.776 - 0.951)	0.865 (0.780 - 0.949)	0.866 (0.779 - 0.949)	0.320 (0.140 - 0.621)	0.318 (0.138 - 0.618)	0.318 (0.137 - 0.619)
70+ years (rate per capita)	0.529 (0.494 - 0.565)	0.545 (0.505 - 0.584)	0.525 (0.491 - 0.561)	0.855 (0.773 - 0.926)	0.858 (0.766 - 0.935)	0.856 (0.775 - 0.925)	0.294 (0.132 - 0.565)	0.302 (0.135 - 0.580)	0.289 (0.130 - 0.557)
All ages (rate per capita)	0.517 (0.494 - 0.539)	0.540 (0.518 - 0.561)	0.536 (0.514 - 0.557)	0.688 (0.647 - 0.727)	0.735 (0.692 - 0.775)	0.749 (0.705 - 0.788)	0.305 (0.133 - 0.593)	0.316 (0.139 - 0.613)	0.313 (0.136 - 0.609)
Age-standardized (rate per capita)	0.508 (0.487 - 0.528)	0.508 (0.487 - 0.528)	0.508 (0.486 - 0.528)	0.680 (0.641 - 0.717)	0.680 (0.641 - 0.717)	0.680 (0.641 - 0.717)	0.299 (0.130 - 0.581)	0.298 (0.131 - 0.577)	0.298 (0.129 - 0.579)
Total number of cases (thousands)	2,339 (2,234 - 2,436)	2,072 (1,989 - 2,154)	2,042 (1,960 - 2,122)	3,110 (2,925 - 3,287)	2,821 (2,659 - 2,975)	2,852 (2,688 - 3,004)	1 (1 - 3)	1 (1 - 2)	1 (1 - 2)
**Bulgaria**									
5 -14 years (rate per capita)	0.481 (0.426 - 0.534)	0.481 (0.421 - 0.540)	0.455 (0.398 - 0.513)	0.725 (0.639 - 0.809)	0.712 (0.606 - 0.830)	0.694 (0.585 - 0.812)	0.480 (0.213 - 0.935)	0.480 (0.210 - 0.945)	0.455 (0.200 - 0.896)
15 - 49 years (rate per capita)	0.595 (0.564 - 0.624)	0.593 (0.564 - 0.621)	0.590 (0.559 - 0.618)	0.932 (0.874 - 0.990)	0.934 (0.877 - 0.994)	0.941 (0.882 - 0.999)	0.589 (0.256 - 1.116)	0.588 (0.259 - 1.119)	0.584 (0.253 - 1.116)
50 - 69 years (rate per capita)	0.545 (0.504 - 0.581)	0.544 (0.506 - 0.581)	0.545 (0.506 - 0.582)	1.046 (0.967 - 1.119)	1.045 (0.965 - 1.121)	1.045 (0.967 - 1.120)	0.524 (0.229 - 0.992)	0.524 (0.230 - 0.986)	0.524 (0.230 - 0.982)
70+ years (rate per capita)	0.523 (0.492 - 0.556)	0.526 (0.492 - 0.561)	0.515 (0.484 - 0.548)	1.023 (0.959 - 1.083)	1.025 (0.961 - 1.089)	1.020 (0.961 - 1.078)	0.482 (0.217 - 0.924)	0.485 (0.217 - 0.907)	0.475 (0.213 - 0.892)
All ages (rate per capita)	0.523 (0.503 - 0.543)	0.536 (0.517 - 0.555)	0.528 (0.510 - 0.545)	0.878 (0.836 - 0.917)	0.912 (0.871 - 0.953)	0.916 (0.873 - 0.955)	0.512 (0.227 - 0.968)	0.523 (0.230 - 0.988)	0.514 (0.227 - 0.973)
Age-standardized (rate per capita)	0.506 (0.486 - 0.525)	0.505 (0.486 - 0.525)	0.505 (0.486 - 0.524)	0.835 (0.795 - 0.873)	0.831 (0.790 - 0.871)	0.831 (0.790 - 0.871)	0.497 (0.218 - 0.940)	0.496 (0.217 - 0.934)	0.496 (0.218 - 0.934)
Total number of cases (thousands)	4,619 (4,442 - 4,789)	4,122 (3,976 - 4,262)	3,836 (3,705 - 3,964)	7,748 (7,378 - 8,096)	7,012 (6,694 - 7,322)	6,655 (6,343 - 6,944)	5 (2 - 9)	4 (2 - 8)	4 (2 - 7)
**Croatia**									
5 -14 years (rate per capita)	0.407 (0.349 - 0.469)	0.356 (0.311 - 0.405)	0.353 (0.308 - 0.401)	0.495 (0.387 - 0.603)	0.413 (0.337 - 0.488)	0.410 (0.335 - 0.484)	0.407 (0.181 - 0.799)	0.356 (0.154 - 0.700)	0.352 (0.154 - 0.701)
15 - 49 years (rate per capita)	0.515 (0.481 - 0.551)	0.514 (0.480 - 0.550)	0.513 (0.479 - 0.549)	0.636 (0.577 - 0.698)	0.637 (0.577 - 0.696)	0.638 (0.578 - 0.698)	0.510 (0.221 - 0.961)	0.509 (0.221 - 0.959)	0.508 (0.222 - 0.957)
50 - 69 years (rate per capita)	0.470 (0.424 - 0.515)	0.470 (0.427 - 0.516)	0.470 (0.428 - 0.516)	0.705 (0.623 - 0.795)	0.705 (0.619 - 0.793)	0.706 (0.621 - 0.799)	0.451 (0.194 - 0.850)	0.452 (0.195 - 0.861)	0.452 (0.198 - 0.850)
70+ years (rate per capita)	0.445 (0.406 - 0.482)	0.453 (0.413 - 0.494)	0.439 (0.403 - 0.473)	0.675 (0.597 - 0.752)	0.678 (0.598 - 0.768)	0.672 (0.597 - 0.753)	0.409 (0.181 - 0.793)	0.418 (0.186 - 0.791)	0.404 (0.179 - 0.754)
All ages (rate per capita)	0.453 (0.430 - 0.478)	0.454 (0.432 - 0.477)	0.450 (0.429 - 0.472)	0.597 (0.557 - 0.638)	0.603 (0.565 - 0.641)	0.607 (0.568 - 0.645)	0.444 (0.194 - 0.847)	0.443 (0.194 - 0.831)	0.439 (0.193 - 0.823)
Age-standardized (rate per capita)	0.438 (0.415 - 0.462)	0.430 (0.409 - 0.451)	0.430 (0.409 - 0.451)	0.568 (0.529 - 0.607)	0.553 (0.517 - 0.588)	0.554 (0.517 - 0.588)	0.430 (0.190 - 0.822)	0.422 (0.184 - 0.796)	0.422 (0.184 - 0.792)
Total number of cases (thousands)	2,164 (2,054 - 2,280)	1,990 (1,894 - 2,091)	1,910 (1,820 - 2,003)	2,851 (2,659 - 3,048)	2,643 (2,476 - 2,810)	2,574 (2,411 - 2,737)	2 (1 - 4)	2 (1 - 4)	2 (1 - 3)
**Czech Republic**									
5 -14 years (rate per capita)	0.390 (0.292 - 0.489)	0.392 (0.294 - 0.492)	0.359 (0.268 - 0.458)	0.634 (0.463 - 0.813)	0.637 (0.466 - 0.816)	0.603 (0.434 - 0.787)	0.389 (0.163 - 0.771)	0.392 (0.167 - 0.778)	0.359 (0.154 - 0.697)
15 - 49 years (rate per capita)	0.508 (0.443 - 0.574)	0.506 (0.441 - 0.572)	0.504 (0.437 - 0.570)	0.824 (0.709 - 0.934)	0.825 (0.710 - 0.938)	0.830 (0.717 - 0.948)	0.503 (0.217 - 0.979)	0.501 (0.216 - 0.974)	0.499 (0.214 - 0.975)
50 - 69 years (rate per capita)	0.487 (0.400 - 0.578)	0.484 (0.394 - 0.575)	0.488 (0.397 - 0.580)	0.891 (0.722 - 1.064)	0.889 (0.714 - 1.061)	0.892 (0.723 - 1.073)	0.468 (0.201 - 0.905)	0.467 (0.200 - 0.905)	0.468 (0.202 - 0.896)
70+ years (rate per capita)	0.465 (0.389 - 0.542)	0.466 (0.389 - 0.545)	0.458 (0.386 - 0.531)	0.878 (0.715 - 1.026)	0.879 (0.714 - 1.030)	0.875 (0.719 - 1.017)	0.427 (0.189 - 0.816)	0.429 (0.190 - 0.819)	0.421 (0.185 - 0.805)
All ages (rate per capita)	0.451 (0.408 - 0.494)	0.462 (0.418 - 0.506)	0.456 (0.414 - 0.501)	0.761 (0.689 - 0.836)	0.790 (0.712 - 0.869)	0.788 (0.712 - 0.870)	0.441 (0.193 - 0.849)	0.451 (0.197 - 0.865)	0.444 (0.193 - 0.850)
Age-standardized (rate per capita)	0.432 (0.390 - 0.474)	0.432 (0.390 - 0.474)	0.432 (0.390 - 0.473)	0.727 (0.656 - 0.800)	0.727 (0.656 - 0.800)	0.728 (0.656 - 0.800)	0.424 (0.184 - 0.819)	0.424 (0.186 - 0.820)	0.424 (0.185 - 0.819)
Total number of cases (thousands)	4,643 (4,201 - 5,089)	4,747 (4,295 - 5,204)	4,875 (4,425 - 5,358)	7,841 (7,094 - 8,610)	8,118 (7,319 - 8,935)	8,432 (7,614 - 9,302)	5 (2 - 9)	5 (2 - 9)	5 (2 - 9)
**Hungary**									
5 -14 years (rate per capita)	0.411 (0.358 - 0.465)	0.402 (0.343 - 0.463)	0.388 (0.331 - 0.445)	0.597 (0.480 - 0.719)	0.587 (0.467 - 0.708)	0.575 (0.459 - 0.694)	0.410 (0.183 - 0.787)	0.401 (0.180 - 0.791)	0.387 (0.172 - 0.752)
15 - 49 years (rate per capita)	0.528 (0.491 - 0.564)	0.523 (0.488 - 0.559)	0.524 (0.487 - 0.561)	0.818 (0.745 - 0.890)	0.820 (0.749 - 0.889)	0.821 (0.750 - 0.893)	0.522 (0.227 - 0.996)	0.518 (0.227 - 0.976)	0.519 (0.226 - 0.988)
50 - 69 years (rate per capita)	0.479 (0.433 - 0.524)	0.477 (0.430 - 0.526)	0.478 (0.434 - 0.528)	0.919 (0.817 - 1.023)	0.918 (0.819 - 1.017)	0.920 (0.821 - 1.024)	0.459 (0.201 - 0.877)	0.458 (0.198 - 0.856)	0.459 (0.199 - 0.862)
70+ years (rate per capita)	0.462 (0.425 - 0.501)	0.462 (0.423 - 0.500)	0.454 (0.417 - 0.490)	0.894 (0.802 - 0.986)	0.896 (0.799 - 0.989)	0.890 (0.801 - 0.978)	0.423 (0.188 - 0.797)	0.425 (0.190 - 0.800)	0.416 (0.185 - 0.785)
All ages (rate per capita)	0.463 (0.440 - 0.486)	0.468 (0.445 - 0.490)	0.467 (0.445 - 0.489)	0.766 (0.720 - 0.814)	0.789 (0.742 - 0.835)	0.796 (0.748 - 0.842)	0.452 (0.199 - 0.859)	0.456 (0.202 - 0.868)	0.456 (0.201 - 0.861)
Age-standardized (rate per capita)	0.444 (0.420 - 0.466)	0.443 (0.420 - 0.465)	0.445 (0.421 - 0.467)	0.724 (0.680 - 0.769)	0.723 (0.679 - 0.766)	0.725 (0.679 - 0.767)	0.435 (0.192 - 0.828)	0.434 (0.191 - 0.826)	0.437 (0.193 - 0.830)
Total number of cases (thousands)	4,808 (4,571 - 5,053)	4,714 (4,483 - 4,935)	4,753 (4,523 - 4,972)	7,958 (7,479 - 8,455)	7,953 (7,480 - 8,420)	8,091 (7,608 - 8,558)	5 (2 - 9)	5 (2 - 9)	5 (2 - 9)
**Macedonia**									
5 -14 years (rate per capita)	0.370 (0.277 - 0.468)	0.381 (0.285 - 0.480)	0.381 (0.285 - 0.480)	0.586 (0.428 - 0.759)	0.595 (0.437 - 0.764)	0.596 (0.437 - 0.764)	0.222 (0.093 - 0.440)	0.229 (0.096 - 0.454)	0.229 (0.097 - 0.460)
15 - 49 years (rate per capita)	0.508 (0.442 - 0.574)	0.507 (0.443 - 0.573)	0.506 (0.442 - 0.571)	0.771 (0.660 - 0.879)	0.774 (0.664 - 0.880)	0.777 (0.669 - 0.884)	0.302 (0.129 - 0.600)	0.302 (0.128 - 0.595)	0.301 (0.129 - 0.593)
50 - 69 years (rate per capita)	0.485 (0.395 - 0.575)	0.485 (0.394 - 0.571)	0.485 (0.395 - 0.576)	0.840 (0.672 - 1.010)	0.839 (0.672 - 1.005)	0.841 (0.672 - 1.009)	0.280 (0.121 - 0.578)	0.280 (0.120 - 0.576)	0.280 (0.120 - 0.579)
70+ years (rate per capita)	0.469 (0.389 - 0.552)	0.476 (0.389 - 0.564)	0.469 (0.388 - 0.551)	0.836 (0.676 - 0.987)	0.837 (0.675 - 0.996)	0.834 (0.677 - 0.986)	0.259 (0.117 - 0.488)	0.263 (0.116 - 0.505)	0.259 (0.116 - 0.492)
All ages (rate per capita)	0.433 (0.390 - 0.476)	0.452 (0.409 - 0.495)	0.456 (0.413 - 0.499)	0.684 (0.617 - 0.757)	0.719 (0.649 - 0.792)	0.733 (0.662 - 0.810)	0.255 (0.111 - 0.497)	0.266 (0.116 - 0.519)	0.268 (0.116 - 0.527)

	Prevalence 1990	Prevalence 2005	Prevalence 2015	Incidence 1990	Incidence 2005	Incidence 2015	YLD rate 1990	YLD rate 2005	YLD rate 2015
Age-standardized (rate per capita)	0.432 (0.390 - 0.473)	0.432 (0.390 - 0.474)	0.432 (0.390 - 0.473)	0.687 (0.621 - 0.758)	0.687 (0.621 - 0.758)	0.687 (0.621 - 0.758)	0.254 (0.111 - 0.493)	0.254 (0.112 - 0.494)	0.254 (0.111 - 0.496)
Total number of cases (thousands)	867 (781 - 953)	923 (835 - 1,012)	947 (858 - 1,038)	1,370 (1,235 - 1,515)	1,468 (1,325 - 1,618)	1,523 (1,376 - 1,683)	1 (0 - 1)	1 (0 - 1)	1 (0 - 1)
**Montenegro**									
5 -14 years (rate per capita)	0.369 (0.277 - 0.468)	0.377 (0.283 - 0.476)	0.374 (0.280 - 0.473)	0.586 (0.428 - 0.759)	0.593 (0.435 - 0.763)	0.590 (0.433 - 0.762)	0.222 (0.094 - 0.438)	0.226 (0.093 - 0.446)	0.224 (0.095 - 0.450)
15 - 49 years (rate per capita)	0.508 (0.442 - 0.573)	0.508 (0.443 - 0.574)	0.507 (0.443 - 0.573)	0.771 (0.660 - 0.878)	0.773 (0.661 - 0.880)	0.775 (0.667 - 0.884)	0.302 (0.129 - 0.596)	0.302 (0.129 - 0.591)	0.301 (0.129 - 0.594)
50 - 69 years (rate per capita)	0.484 (0.393 - 0.576)	0.485 (0.394 - 0.572)	0.486 (0.395 - 0.576)	0.840 (0.671 - 1.010)	0.839 (0.673 - 1.003)	0.841 (0.673 - 1.010)	0.280 (0.120 - 0.578)	0.281 (0.120 - 0.578)	0.281 (0.121 - 0.573)
70+ years (rate per capita)	0.459 (0.387 - 0.533)	0.475 (0.389 - 0.562)	0.466 (0.389 - 0.544)	0.830 (0.681 - 0.969)	0.837 (0.675 - 0.995)	0.833 (0.678 - 0.980)	0.254 (0.114 - 0.484)	0.263 (0.117 - 0.505)	0.257 (0.117 - 0.486)
All ages (rate per capita)	0.435 (0.392 - 0.478)	0.449 (0.407 - 0.492)	0.451 (0.409 - 0.493)	0.690 (0.623 - 0.763)	0.717 (0.646 - 0.788)	0.727 (0.658 - 0.801)	0.256 (0.112 - 0.502)	0.264 (0.115 - 0.511)	0.265 (0.114 - 0.518)
Age-standardized (rate per capita)	0.432 (0.390 - 0.473)	0.432 (0.390 - 0.474)	0.432 (0.390 - 0.473)	0.687 (0.621 - 0.758)	0.687 (0.622 - 0.758)	0.687 (0.621 - 0.758)	0.254 (0.111 - 0.495)	0.254 (0.111 - 0.493)	0.254 (0.111 - 0.493)
Total number of cases (thousands)	267 (241 - 294)	277 (251 - 304)	282 (256 - 309)	425 (383 - 469)	442 (399 - 486)	455 (412 - 502)	0 (0 - 0)	0 (0 - 0)	0 (0 - 0)
**Poland**									
5 -14 years (rate per capita)	0.370 (0.277 - 0.468)	0.388 (0.291 - 0.488)	0.373 (0.280 - 0.472)	0.613 (0.446 - 0.794)	0.632 (0.463 - 0.812)	0.617 (0.451 - 0.798)	0.369 (0.156 - 0.724)	0.388 (0.165 - 0.761)	0.373 (0.159 - 0.728)
15 - 49 years (rate per capita)	0.507 (0.441 - 0.574)	0.507 (0.443 - 0.573)	0.505 (0.439 - 0.571)	0.824 (0.707 - 0.934)	0.822 (0.705 - 0.933)	0.826 (0.710 - 0.940)	0.502 (0.215 - 0.981)	0.502 (0.216 - 0.971)	0.500 (0.213 - 0.970)
50 - 69 years (rate per capita)	0.486 (0.397 - 0.575)	0.484 (0.392 - 0.572)	0.484 (0.393 - 0.578)	0.890 (0.718 - 1.063)	0.888 (0.711 - 1.060)	0.890 (0.711 - 1.070)	0.466 (0.199 - 0.896)	0.465 (0.196 - 0.898)	0.466 (0.199 - 0.900)
70+ years (rate per capita)	0.465 (0.389 - 0.541)	0.469 (0.390 - 0.550)	0.458 (0.386 - 0.531)	0.878 (0.716 - 1.027)	0.880 (0.715 - 1.034)	0.874 (0.720 - 1.015)	0.425 (0.188 - 0.815)	0.430 (0.191 - 0.830)	0.419 (0.189 - 0.812)
All ages (rate per capita)	0.436 (0.394 - 0.479)	0.461 (0.417 - 0.505)	0.457 (0.414 - 0.501)	0.737 (0.663 - 0.809)	0.780 (0.704 - 0.861)	0.789 (0.710 - 0.869)	0.427 (0.188 - 0.821)	0.450 (0.195 - 0.863)	0.446 (0.193 - 0.850)
Age-standardized (rate per capita)	0.432 (0.390 - 0.474)	0.432 (0.390 - 0.474)	0.432 (0.390 - 0.474)	0.727 (0.656 - 0.800)	0.727 (0.656 - 0.800)	0.727 (0.656 - 0.800)	0.423 (0.184 - 0.820)	0.423 (0.183 - 0.816)	0.424 (0.184 - 0.817)
Total number of cases (thousands)	16,679 (15,072 - 18,310)	17,591 (15,930 - 19,291)	17,801 (16,116 - 19,510)	28,188 (25,351 - 30,925)	29,811 (26,896 - 32,873)	30,683 (27,630 - 33,832)	16 (7 - 31)	17 (7 - 33)	17 (8 - 33)
**Romania**									
5 -14 years (rate per capita)	0.459 (0.405 - 0.508)	0.437 (0.375 - 0.496)	0.428 (0.368 - 0.487)	0.614 (0.500 - 0.721)	0.606 (0.489 - 0.721)	0.600 (0.484 - 0.716)	0.457 (0.204 - 0.893)	0.436 (0.191 - 0.862)	0.428 (0.190 - 0.829)
15 - 49 years (rate per capita)	0.565 (0.531 - 0.602)	0.562 (0.528 - 0.598)	0.556 (0.523 - 0.593)	0.816 (0.750 - 0.885)	0.822 (0.757 - 0.886)	0.833 (0.765 - 0.897)	0.560 (0.246 - 1.062)	0.556 (0.242 - 1.050)	0.551 (0.241 - 1.046)
50 - 69 years (rate per capita)	0.514 (0.470 - 0.559)	0.514 (0.468 - 0.560)	0.515 (0.469 - 0.559)	0.930 (0.826 - 1.026)	0.928 (0.829 - 1.023)	0.930 (0.830 - 1.027)	0.494 (0.215 - 0.935)	0.495 (0.214 - 0.940)	0.496 (0.217 - 0.941)
70+ years (rate per capita)	0.497 (0.459 - 0.538)	0.502 (0.462 - 0.544)	0.489 (0.454 - 0.527)	0.912 (0.820 - 0.998)	0.915 (0.823 - 1.007)	0.909 (0.822 - 0.993)	0.459 (0.208 - 0.851)	0.465 (0.208 - 0.873)	0.452 (0.203 - 0.868)
All ages (rate per capita)	0.491 (0.469 - 0.513)	0.503 (0.480 - 0.526)	0.497 (0.475 - 0.520)	0.754 (0.708 - 0.799)	0.791 (0.744 - 0.835)	0.801 (0.754 - 0.845)	0.482 (0.214 - 0.911)	0.492 (0.219 - 0.933)	0.486 (0.216 - 0.926)
Age-standardized (rate per capita)	0.479 (0.457 - 0.500)	0.475 (0.453 - 0.497)	0.475 (0.453 - 0.497)	0.734 (0.688 - 0.778)	0.733 (0.689 - 0.775)	0.733 (0.689 - 0.775)	0.470 (0.208 - 0.886)	0.467 (0.208 - 0.893)	0.467 (0.207 - 0.892)
Total number of cases (thousands)	11,509 (10,982 - 12,008)	10,772 (10,285 - 11,263)	9,714 (9,283 - 10,161)	17,662 (16,585 - 18,708)	16,937 (15,940 - 17,887)	15,645 (14,725 - 16,494)	11 (5 - 21)	11 (5 - 20)	9 (4 - 18)
**Serbia**									
5 -14 years (rate per capita)	0.375 (0.281 - 0.474)	0.376 (0.282 - 0.475)	0.381 (0.286 - 0.480)	0.590 (0.433 - 0.762)	0.591 (0.434 - 0.763)	0.596 (0.437 - 0.764)	0.225 (0.094 - 0.439)	0.226 (0.094 - 0.444)	0.228 (0.097 - 0.453)
15 - 49 years (rate per capita)	0.508 (0.442 - 0.574)	0.507 (0.443 - 0.573)	0.506 (0.443 - 0.574)	0.774 (0.664 - 0.882)	0.774 (0.664 - 0.880)	0.777 (0.666 - 0.885)	0.302 (0.128 - 0.600)	0.301 (0.130 - 0.591)	0.301 (0.128 - 0.595)
50 - 69 years (rate per capita)	0.486 (0.396 - 0.576)	0.484 (0.395 - 0.572)	0.487 (0.400 - 0.580)	0.841 (0.674 - 1.010)	0.840 (0.671 - 1.006)	0.842 (0.677 - 1.009)	0.280 (0.119 - 0.576)	0.280 (0.121 - 0.576)	0.281 (0.119 - 0.572)
70+ years (rate per capita)	0.463 (0.388 - 0.540)	0.475 (0.390 - 0.562)	0.462 (0.387 - 0.538)	0.832 (0.679 - 0.976)	0.837 (0.675 - 0.996)	0.832 (0.680 - 0.974)	0.256 (0.115 - 0.484)	0.262 (0.118 - 0.494)	0.254 (0.115 - 0.486)
All ages (rate per capita)	0.441 (0.398 - 0.483)	0.451 (0.409 - 0.494)	0.456 (0.415 - 0.499)	0.703 (0.636 - 0.775)	0.725 (0.655 - 0.798)	0.739 (0.667 - 0.815)	0.259 (0.113 - 0.506)	0.264 (0.116 - 0.515)	0.267 (0.116 - 0.523)
Age-standardized (rate per capita)	0.432 (0.390 - 0.474)	0.432 (0.390 - 0.474)	0.432 (0.390 - 0.474)	0.687 (0.621 - 0.758)	0.687 (0.621 - 0.758)	0.687 (0.621 - 0.758)	0.254 (0.111 - 0.495)	0.254 (0.112 - 0.493)	0.254 (0.111 - 0.493)
Total number of cases (thousands)	4,191 (3,788 - 4,595)	4,155 (3,770 - 4,550)	4,042 (3,676 - 4,423)	6,684 (6,046 - 7,373)	6,677 (6,034 - 7,347)	6,549 (5,910 - 7,215)	2 (1 - 5)	2 (1 - 5)	2 (1 - 5)
**Slovakia**									
5 -14 years (rate per capita)	0.378 (0.283 - 0.477)	0.389 (0.291 - 0.489)	0.372 (0.279 - 0.471)	0.593 (0.435 - 0.763)	0.603 (0.444 - 0.772)	0.588 (0.431 - 0.761)	0.227 (0.095 - 0.449)	0.234 (0.098 - 0.468)	0.223 (0.093 - 0.442)
15 - 49 years (rate per capita)	0.508 (0.441 - 0.574)	0.507 (0.443 - 0.572)	0.505 (0.439 - 0.570)	0.773 (0.663 - 0.881)	0.774 (0.663 - 0.882)	0.778 (0.666 - 0.893)	0.302 (0.128 - 0.600)	0.301 (0.129 - 0.595)	0.300 (0.128 - 0.596)
50 - 69 years (rate per capita)	0.487 (0.399 - 0.575)	0.484 (0.393 - 0.573)	0.485 (0.395 - 0.577)	0.841 (0.676 - 1.010)	0.839 (0.669 - 1.011)	0.841 (0.671 - 1.012)	0.281 (0.121 - 0.578)	0.280 (0.122 - 0.577)	0.280 (0.121 - 0.575)
70+ years (rate per capita)	0.465 (0.390 - 0.542)	0.468 (0.390 - 0.548)	0.460 (0.387 - 0.534)	0.833 (0.678 - 0.978)	0.833 (0.679 - 0.982)	0.830 (0.681 - 0.970)	0.257 (0.116 - 0.489)	0.259 (0.117 - 0.493)	0.254 (0.115 - 0.481)
All ages (rate per capita)	0.439 (0.396 - 0.482)	0.460 (0.417 - 0.505)	0.459 (0.416 - 0.504)	0.696 (0.628 - 0.767)	0.735 (0.662 - 0.808)	0.743 (0.669 - 0.819)	0.258 (0.113 - 0.500)	0.271 (0.118 - 0.531)	0.269 (0.116 - 0.530)
Age-standardized (rate per capita)	0.432 (0.390 - 0.474)	0.432 (0.390 - 0.474)	0.432 (0.390 - 0.474)	0.687 (0.621 - 0.758)	0.687 (0.621 - 0.758)	0.687 (0.621 - 0.758)	0.254 (0.111 - 0.493)	0.254 (0.111 - 0.495)	0.254 (0.112 - 0.497)
Total number of cases (thousands)	2,312 (2,088 - 2,539)	2,480 (2,247 - 2,720)	2,548 (2,310 - 2,799)	3,670 (3,309 - 4,041)	3,959 (3,566 - 4,354)	4,125 (3,716 - 4,552)	1 (1 - 3)	1 (1 - 3)	1 (1 - 3)
**Slovenia**									
5 -14 years (rate per capita)	0.302 (0.261 - 0.354)	0.375 (0.313 - 0.438)	0.356 (0.298 - 0.416)	0.513 (0.407 - 0.650)	0.568 (0.452 - 0.698)	0.549 (0.438 - 0.680)	0.301 (0.129 - 0.581)	0.375 (0.163 - 0.730)	0.356 (0.152 - 0.686)
15 - 49 years (rate per capita)	0.478 (0.442 - 0.517)	0.484 (0.446 - 0.521)	0.480 (0.442 - 0.517)	0.810 (0.739 - 0.886)	0.810 (0.736 - 0.888)	0.815 (0.742 - 0.892)	0.473 (0.205 - 0.901)	0.479 (0.209 - 0.917)	0.476 (0.206 - 0.905)
50 - 69 years (rate per capita)	0.436 (0.391 - 0.484)	0.435 (0.390 - 0.483)	0.435 (0.389 - 0.483)	0.900 (0.793 - 1.008)	0.898 (0.796 - 1.005)	0.900 (0.797 - 1.009)	0.418 (0.181 - 0.788)	0.419 (0.181 - 0.794)	0.420 (0.182 - 0.788)
70+ years (rate per capita)	0.418 (0.379 - 0.459)	0.423 (0.386 - 0.464)	0.412 (0.377 - 0.450)	0.867 (0.769 - 0.968)	0.871 (0.771 - 0.976)	0.863 (0.769 - 0.962)	0.384 (0.171 - 0.719)	0.390 (0.175 - 0.738)	0.380 (0.169 - 0.714)
All ages (rate per capita)	0.410 (0.387 - 0.434)	0.434 (0.410 - 0.457)	0.423 (0.398 - 0.446)	0.739 (0.692 - 0.789)	0.779 (0.731 - 0.827)	0.777 (0.727 - 0.824)	0.402 (0.176 - 0.763)	0.424 (0.187 - 0.810)	0.412 (0.182 - 0.783)
Age-standardized (rate per capita)	0.393 (0.370 - 0.415)	0.410 (0.386 - 0.432)	0.409 (0.385 - 0.432)	0.703 (0.656 - 0.752)	0.710 (0.664 - 0.758)	0.710 (0.664 - 0.758)	0.385 (0.168 - 0.730)	0.403 (0.177 - 0.766)	0.403 (0.175 - 0.764)
Total number of cases (thousands)	823 (776 - 870)	866 (818 - 913)	872 (822 - 920)	1,484 (1,389 - 1,584)	1,555 (1,460 - 1,651)	1,603 (1,501 - 1,700)	1 (0 - 2)	1 (0 - 2)	1 (0 - 2)
**Central Asia**									
5 -14 years (rate per capita)	0.359 (0.299 - 0.416)	0.379 (0.317 - 0.439)	0.356 (0.297 - 0.412)	0.576 (0.459 - 0.693)	0.595 (0.473 - 0.713)	0.573 (0.456 - 0.690)	0.246 (0.107 - 0.472)	0.252 (0.108 - 0.488)	0.240 (0.103 - 0.456)
15 - 49 years (rate per capita)	0.508 (0.471 - 0.545)	0.508 (0.473 - 0.546)	0.504 (0.468 - 0.541)	0.805 (0.737 - 0.875)	0.804 (0.737 - 0.867)	0.808 (0.743 - 0.876)	0.352 (0.156 - 0.687)	0.343 (0.152 - 0.670)	0.339 (0.150 - 0.659)
50 - 69 years (rate per capita)	0.465 (0.419 - 0.512)	0.465 (0.420 - 0.514)	0.463 (0.416 - 0.513)	0.899 (0.803 - 1.004)	0.897 (0.807 - 1.000)	0.896 (0.797 - 1.003)	0.319 (0.142 - 0.620)	0.315 (0.139 - 0.611)	0.309 (0.135 - 0.600)
70+ years (rate per capita)	0.445 (0.407 - 0.481)	0.453 (0.412 - 0.493)	0.444 (0.407 - 0.480)	0.887 (0.796 - 0.979)	0.891 (0.793 - 0.985)	0.887 (0.794 - 0.981)	0.294 (0.132 - 0.561)	0.290 (0.131 - 0.549)	0.287 (0.130 - 0.542)
All ages (rate per capita)	0.397 (0.374 - 0.421)	0.427 (0.402 - 0.452)	0.417 (0.393 - 0.440)	0.658 (0.614 - 0.704)	0.700 (0.656 - 0.745)	0.699 (0.655 - 0.742)	0.274 (0.122 - 0.526)	0.287 (0.126 - 0.553)	0.279 (0.124 - 0.538)
Age-standardized (rate per capita)	0.425 (0.401 - 0.447)	0.425 (0.401 - 0.447)	0.424 (0.401 - 0.447)	0.720 (0.677 - 0.762)	0.718 (0.675 - 0.760)	0.718 (0.675 - 0.760)	0.293 (0.130 - 0.562)	0.285 (0.126 - 0.548)	0.284 (0.125 - 0.545)
Total number of cases (thousands)	27,347 (25,755 - 28,970)	32,892 (30,943 - 34,802)	36,282 (34,218 - 38,286)	45,271 (42,240 - 48,405)	53,896 (50,499 - 57,384)	60,810 (56,946 - 64,597)	19 (8 - 36)	22 (10 - 43)	24 (11 - 47)
**Armenia**									
5 -14 years (rate per capita)	0.358 (0.298 - 0.415)	0.386 (0.321 - 0.448)	0.355 (0.296 - 0.411)	0.570 (0.453 - 0.686)	0.600 (0.481 - 0.718)	0.569 (0.453 - 0.685)	0.214 (0.091 - 0.408)	0.231 (0.098 - 0.453)	0.213 (0.090 - 0.410)
15 - 49 years (rate per capita)	0.505 (0.468 - 0.543)	0.506 (0.472 - 0.544)	0.502 (0.466 - 0.540)	0.798 (0.731 - 0.868)	0.797 (0.733 - 0.862)	0.802 (0.734 - 0.871)	0.299 (0.132 - 0.592)	0.300 (0.130 - 0.596)	0.299 (0.130 - 0.600)
50 - 69 years (rate per capita)	0.465 (0.419 - 0.513)	0.466 (0.422 - 0.513)	0.464 (0.419 - 0.512)	0.886 (0.792 - 0.991)	0.885 (0.798 - 0.982)	0.886 (0.790 - 0.992)	0.268 (0.116 - 0.530)	0.268 (0.115 - 0.524)	0.267 (0.117 - 0.527)
70+ years (rate per capita)	0.441 (0.405 - 0.475)	0.452 (0.412 - 0.491)	0.436 (0.400 - 0.470)	0.873 (0.783 - 0.964)	0.880 (0.784 - 0.974)	0.871 (0.781 - 0.958)	0.242 (0.109 - 0.459)	0.250 (0.112 - 0.475)	0.240 (0.107 - 0.452)
All ages (rate per capita)	0.414 (0.390 - 0.437)	0.444 (0.419 - 0.467)	0.438 (0.416 - 0.460)	0.686 (0.641 - 0.732)	0.734 (0.690 - 0.778)	0.748 (0.701 - 0.792)	0.244 (0.106 - 0.470)	0.261 (0.112 - 0.507)	0.257 (0.112 - 0.497)
Age-standardized (rate per capita)	0.425 (0.402 - 0.447)	0.424 (0.401 - 0.447)	0.425 (0.402 - 0.447)	0.710 (0.667 - 0.752)	0.710 (0.667 - 0.752)	0.709 (0.667 - 0.752)	0.249 (0.108 - 0.480)	0.249 (0.108 - 0.484)	0.250 (0.109 - 0.484)
Total number of cases (thousands)	1,461 (1,376 - 1,542)	1,339 (1,265 - 1,410)	1,318 (1,251 - 1,385)	2,422 (2,263 - 2,583)	2,215 (2,083 - 2,347)	2,249 (2,108 - 2,382)	1 (0 - 2)	1 (0 - 2)	1 (0 - 1)
**Azerbaijan**									
5 -14 years (rate per capita)	0.358 (0.298 - 0.415)	0.384 (0.320 - 0.445)	0.364 (0.304 - 0.421)	0.570 (0.453 - 0.687)	0.596 (0.477 - 0.713)	0.578 (0.462 - 0.695)	0.214 (0.092 - 0.417)	0.230 (0.097 - 0.442)	0.218 (0.092 - 0.423)
15 - 49 years (rate per capita)	0.508 (0.470 - 0.546)	0.507 (0.473 - 0.545)	0.502 (0.467 - 0.540)	0.792 (0.724 - 0.864)	0.797 (0.733 - 0.860)	0.801 (0.735 - 0.869)	0.302 (0.133 - 0.593)	0.301 (0.131 - 0.602)	0.299 (0.130 - 0.593)
50 - 69 years (rate per capita)	0.464 (0.418 - 0.512)	0.465 (0.420 - 0.513)	0.463 (0.414 - 0.511)	0.886 (0.791 - 0.992)	0.884 (0.794 - 0.983)	0.884 (0.784 - 0.995)	0.269 (0.116 - 0.528)	0.269 (0.118 - 0.531)	0.268 (0.117 - 0.526)
70+ years (rate per capita)	0.457 (0.416 - 0.498)	0.460 (0.414 - 0.507)	0.445 (0.407 - 0.481)	0.882 (0.783 - 0.980)	0.884 (0.780 - 0.985)	0.876 (0.783 - 0.969)	0.254 (0.115 - 0.483)	0.257 (0.115 - 0.486)	0.247 (0.111 - 0.467)

	Prevalence 1990	Prevalence 2005	Prevalence 2015	Incidence 1990	Incidence 2005	Incidence 2015	YLD rate 1990	YLD rate 2005	YLD rate 2015
All ages (rate per capita)	0.406 (0.382 - 0.430)	0.436 (0.410 - 0.460)	0.426 (0.403 - 0.449)	0.662 (0.617 - 0.709)	0.707 (0.663 - 0.753)	0.714 (0.669 - 0.758)	0.241 (0.106 - 0.466)	0.258 (0.113 - 0.499)	0.251 (0.109 - 0.493)
Age-standardized (rate per capita)	0.425 (0.401 - 0.447)	0.424 (0.401 - 0.447)	0.424 (0.401 - 0.447)	0.710 (0.667 - 0.752)	0.710 (0.667 - 0.752)	0.710 (0.667 - 0.752)	0.250 (0.108 - 0.483)	0.250 (0.109 - 0.484)	0.250 (0.108 - 0.487)
Total number of cases (thousands)	2,907 (2,734 - 3,080)	3,776 (3,549 - 3,986)	4,168 (3,941 - 4,395)	4,735 (4,413 - 5,069)	6,124 (5,743 - 6,517)	6,985 (6,540 - 7,416)	2 (1 - 3)	2 (1 - 4)	2 (1 - 5)
**Georgia**									
5 -14 years (rate per capita)	0.365 (0.305 - 0.423)	0.394 (0.327 - 0.456)	0.354 (0.295 - 0.410)	0.577 (0.461 - 0.695)	0.603 (0.483 - 0.721)	0.569 (0.453 - 0.685)	0.219 (0.092 - 0.417)	0.236 (0.101 - 0.459)	0.212 (0.090 - 0.406)
15 - 49 years (rate per capita)	0.504 (0.469 - 0.542)	0.505 (0.471 - 0.542)	0.502 (0.467 - 0.539)	0.800 (0.735 - 0.865)	0.800 (0.737 - 0.864)	0.804 (0.740 - 0.870)	0.300 (0.130 - 0.598)	0.300 (0.130 - 0.592)	0.298 (0.130 - 0.593)
50 - 69 years (rate per capita)	0.466 (0.421 - 0.514)	0.466 (0.423 - 0.513)	0.465 (0.420 - 0.513)	0.887 (0.794 - 0.989)	0.887 (0.800 - 0.983)	0.887 (0.792 - 0.989)	0.270 (0.116 - 0.530)	0.270 (0.117 - 0.527)	0.269 (0.118 - 0.529)
70+ years (rate per capita)	0.446 (0.408 - 0.481)	0.452 (0.412 - 0.491)	0.439 (0.403 - 0.472)	0.875 (0.783 - 0.968)	0.880 (0.784 - 0.974)	0.872 (0.784 - 0.961)	0.249 (0.112 - 0.471)	0.252 (0.113 - 0.476)	0.244 (0.109 - 0.462)
All ages (rate per capita)	0.428 (0.405 - 0.450)	0.447 (0.425 - 0.470)	0.436 (0.415 - 0.458)	0.719 (0.675 - 0.762)	0.751 (0.707 - 0.795)	0.749 (0.704 - 0.793)	0.252 (0.109 - 0.492)	0.263 (0.114 - 0.509)	0.256 (0.112 - 0.501)
Age-standardized (rate per capita)	0.425 (0.401 - 0.447)	0.425 (0.402 - 0.447)	0.424 (0.401 - 0.447)	0.710 (0.667 - 0.752)	0.710 (0.667 - 0.752)	0.710 (0.667 - 0.752)	0.251 (0.108 - 0.488)	0.251 (0.109 - 0.485)	0.250 (0.109 - 0.487)
Total number of cases (thousands)	2,336 (2,212 - 2,459)	2,019 (1,919 - 2,123)	1,748 (1,662 - 1,834)	3,928 (3,689 - 4,160)	3,389 (3,189 - 3,589)	3,001 (2,820 - 3,176)	1 (1 - 3)	1 (1 - 2)	1 (0 - 2)
**Kazakhstan**									
5 -14 years (rate per capita)	0.363 (0.303 - 0.420)	0.382 (0.319 - 0.442)	0.344 (0.286 - 0.399)	0.601 (0.474 - 0.726)	0.622 (0.493 - 0.747)	0.581 (0.457 - 0.704)	0.361 (0.160 - 0.710)	0.381 (0.167 - 0.741)	0.343 (0.152 - 0.664)
15 - 49 years (rate per capita)	0.506 (0.470 - 0.544)	0.506 (0.472 - 0.544)	0.502 (0.467 - 0.540)	0.846 (0.774 - 0.917)	0.846 (0.775 - 0.915)	0.851 (0.778 - 0.922)	0.501 (0.218 - 0.960)	0.502 (0.218 - 0.953)	0.498 (0.216 - 0.950)
50 - 69 years (rate per capita)	0.465 (0.419 - 0.512)	0.465 (0.421 - 0.514)	0.464 (0.417 - 0.513)	0.932 (0.831 - 1.040)	0.932 (0.835 - 1.038)	0.932 (0.827 - 1.045)	0.449 (0.195 - 0.856)	0.448 (0.195 - 0.855)	0.447 (0.195 - 0.844)
70+ years (rate per capita)	0.446 (0.408 - 0.482)	0.453 (0.412 - 0.492)	0.452 (0.412 - 0.491)	0.919 (0.823 - 1.019)	0.925 (0.823 - 1.030)	0.925 (0.821 - 1.029)	0.413 (0.183 - 0.781)	0.419 (0.188 - 0.801)	0.418 (0.187 - 0.789)
All ages (rate per capita)	0.410 (0.387 - 0.432)	0.436 (0.411 - 0.459)	0.415 (0.392 - 0.438)	0.713 (0.664 - 0.759)	0.756 (0.708 - 0.803)	0.736 (0.688 - 0.780)	0.403 (0.178 - 0.779)	0.429 (0.189 - 0.819)	0.408 (0.180 - 0.780)
Age-standardized (rate per capita)	0.425 (0.401 - 0.447)	0.425 (0.401 - 0.447)	0.425 (0.401 - 0.447)	0.750 (0.704 - 0.794)	0.750 (0.703 - 0.794)	0.750 (0.704 - 0.794)	0.417 (0.184 - 0.804)	0.417 (0.184 - 0.794)	0.417 (0.184 - 0.794)
Total number of cases (thousands)	6,800 (6,417 - 7,177)	6,746 (6,356 - 7,108)	7,279 (6,883 - 7,674)	11,837 (11,021 - 12,602)	11,698 (10,950 - 12,431)	12,908 (12,062 - 13,680)	7 (3 - 13)	7 (3 - 13)	7 (3 - 14)
**Kyrgyzstan**									
5 -14 years (rate per capita)	0.358 (0.298 - 0.415)	0.378 (0.316 - 0.437)	0.353 (0.294 - 0.408)	0.570 (0.453 - 0.686)	0.588 (0.469 - 0.706)	0.565 (0.449 - 0.681)	0.214 (0.090 - 0.407)	0.226 (0.096 - 0.438)	0.211 (0.089 - 0.403)
15 - 49 years (rate per capita)	0.509 (0.471 - 0.547)	0.508 (0.473 - 0.546)	0.505 (0.469 - 0.543)	0.791 (0.724 - 0.862)	0.793 (0.729 - 0.858)	0.796 (0.731 - 0.863)	0.302 (0.133 - 0.597)	0.302 (0.130 - 0.596)	0.300 (0.130 - 0.597)
50 - 69 years (rate per capita)	0.466 (0.421 - 0.513)	0.464 (0.419 - 0.513)	0.463 (0.416 - 0.512)	0.886 (0.794 - 0.989)	0.883 (0.792 - 0.986)	0.884 (0.787 - 0.993)	0.269 (0.117 - 0.528)	0.269 (0.116 - 0.532)	0.269 (0.116 - 0.540)
70+ years (rate per capita)	0.444 (0.406 - 0.479)	0.451 (0.411 - 0.490)	0.442 (0.405 - 0.476)	0.874 (0.784 - 0.966)	0.879 (0.784 - 0.974)	0.874 (0.783 - 0.965)	0.246 (0.111 - 0.467)	0.251 (0.113 - 0.475)	0.245 (0.110 - 0.464)
All ages (rate per capita)	0.393 (0.369 - 0.417)	0.423 (0.397 - 0.448)	0.407 (0.383 - 0.430)	0.640 (0.596 - 0.686)	0.681 (0.636 - 0.727)	0.669 (0.623 - 0.713)	0.232 (0.101 - 0.447)	0.250 (0.108 - 0.484)	0.241 (0.105 - 0.467)
Age-standardized (rate per capita)	0.425 (0.402 - 0.447)	0.425 (0.401 - 0.447)	0.424 (0.401 - 0.447)	0.710 (0.667 - 0.752)	0.710 (0.667 - 0.752)	0.710 (0.667 - 0.752)	0.250 (0.108 - 0.483)	0.250 (0.107 - 0.486)	0.250 (0.108 - 0.484)
Total number of cases (thousands)	1,729 (1,625 - 1,833)	2,175 (2,042 - 2,303)	2,398 (2,257 - 2,533)	2,816 (2,622 - 3,017)	3,504 (3,273 - 3,740)	3,940 (3,675 - 4,202)	1 (0 - 2)	1 (1 - 2)	1 (1 - 3)
**Mongolia**									
5 -14 years (rate per capita)	0.361 (0.301 - 0.419)	0.381 (0.318 - 0.441)	0.352 (0.293 - 0.407)	0.572 (0.456 - 0.689)	0.592 (0.473 - 0.709)	0.564 (0.448 - 0.679)	0.217 (0.092 - 0.420)	0.229 (0.098 - 0.435)	0.211 (0.089 - 0.411)
15 - 49 years (rate per capita)	0.511 (0.472 - 0.550)	0.509 (0.473 - 0.547)	0.503 (0.468 - 0.540)	0.786 (0.719 - 0.857)	0.792 (0.728 - 0.859)	0.801 (0.736 - 0.869)	0.305 (0.132 - 0.603)	0.303 (0.133 - 0.603)	0.299 (0.130 - 0.603)
50 - 69 years (rate per capita)	0.465 (0.420 - 0.512)	0.465 (0.419 - 0.513)	0.463 (0.415 - 0.512)	0.887 (0.794 - 0.990)	0.885 (0.794 - 0.989)	0.883 (0.784 - 0.990)	0.270 (0.116 - 0.532)	0.269 (0.117 - 0.529)	0.269 (0.117 - 0.531)
70+ years (rate per capita)	0.454 (0.411 - 0.497)	0.457 (0.413 - 0.500)	0.450 (0.410 - 0.490)	0.881 (0.781 - 0.978)	0.882 (0.782 - 0.980)	0.879 (0.784 - 0.970)	0.254 (0.113 - 0.483)	0.255 (0.114 - 0.485)	0.250 (0.113 - 0.473)
All ages (rate per capita)	0.389 (0.364 - 0.414)	0.432 (0.405 - 0.458)	0.414 (0.391 - 0.438)	0.623 (0.578 - 0.670)	0.691 (0.644 - 0.739)	0.684 (0.639 - 0.728)	0.231 (0.101 - 0.443)	0.257 (0.112 - 0.496)	0.246 (0.107 - 0.477)
Age-standardized (rate per capita)	0.425 (0.402 - 0.447)	0.424 (0.401 - 0.447)	0.424 (0.401 - 0.447)	0.710 (0.667 - 0.752)	0.710 (0.667 - 0.752)	0.710 (0.667 - 0.752)	0.251 (0.109 - 0.488)	0.250 (0.108 - 0.485)	0.250 (0.108 - 0.486)
Total number of cases (thousands)	851 (796 - 905)	1,095 (1,026 - 1,159)	1,224 (1,154 - 1,294)	1,364 (1,264 - 1,465)	1,750 (1,631 - 1,871)	2,019 (1,887 - 2,151)	1 (0 - 1)	1 (0 - 1)	1 (0 - 1)
**Tajikistan**									
5 -14 years (rate per capita)	0.353 (0.294 - 0.408)	0.368 (0.308 - 0.427)	0.360 (0.300 - 0.417)	0.565 (0.449 - 0.680)	0.580 (0.463 - 0.698)	0.572 (0.456 - 0.689)	0.211 (0.091 - 0.407)	0.221 (0.093 - 0.424)	0.215 (0.092 - 0.415)
15 - 49 years (rate per capita)	0.510 (0.472 - 0.550)	0.511 (0.475 - 0.550)	0.507 (0.470 - 0.545)	0.787 (0.718 - 0.858)	0.788 (0.723 - 0.856)	0.793 (0.726 - 0.861)	0.304 (0.132 - 0.598)	0.304 (0.133 - 0.605)	0.302 (0.132 - 0.600)
50 - 69 years (rate per capita)	0.465 (0.419 - 0.512)	0.464 (0.419 - 0.514)	0.462 (0.414 - 0.512)	0.886 (0.793 - 0.991)	0.884 (0.795 - 0.986)	0.883 (0.783 - 0.992)	0.269 (0.116 - 0.528)	0.269 (0.117 - 0.524)	0.268 (0.117 - 0.533)
70+ years (rate per capita)	0.442 (0.405 - 0.476)	0.453 (0.411 - 0.494)	0.444 (0.406 - 0.480)	0.873 (0.784 - 0.961)	0.881 (0.783 - 0.977)	0.876 (0.785 - 0.967)	0.245 (0.109 - 0.470)	0.252 (0.113 - 0.477)	0.247 (0.111 - 0.472)
All ages (rate per capita)	0.373 (0.348 - 0.397)	0.402 (0.376 - 0.427)	0.399 (0.374 - 0.423)	0.601 (0.557 - 0.647)	0.639 (0.594 - 0.686)	0.646 (0.602 - 0.692)	0.221 (0.096 - 0.424)	0.239 (0.104 - 0.462)	0.237 (0.103 - 0.459)
Age-standardized (rate per capita)	0.425 (0.401 - 0.447)	0.424 (0.401 - 0.447)	0.424 (0.401 - 0.447)	0.710 (0.667 - 0.752)	0.710 (0.667 - 0.752)	0.710 (0.667 - 0.752)	0.250 (0.108 - 0.485)	0.250 (0.108 - 0.484)	0.250 (0.108 - 0.484)
Total number of cases (thousands)	1,971 (1,841 - 2,096)	2,757 (2,577 - 2,930)	3,393 (3,181 - 3,598)	3,178 (2,942 - 3,420)	4,383 (4,068 - 4,700)	5,494 (5,120 - 5,882)	1 (1 - 2)	2 (1 - 3)	2 (1 - 4)
**Turkmenistan**									
5 -14 years (rate per capita)	0.359 (0.299 - 0.416)	0.380 (0.318 - 0.440)	0.368 (0.307 - 0.426)	0.570 (0.454 - 0.687)	0.591 (0.472 - 0.709)	0.579 (0.463 - 0.697)	0.215 (0.091 - 0.413)	0.228 (0.097 - 0.439)	0.220 (0.095 - 0.422)
15 - 49 years (rate per capita)	0.509 (0.471 - 0.548)	0.509 (0.473 - 0.547)	0.505 (0.469 - 0.542)	0.789 (0.722 - 0.860)	0.792 (0.728 - 0.858)	0.797 (0.732 - 0.864)	0.303 (0.133 - 0.594)	0.303 (0.132 - 0.604)	0.300 (0.130 - 0.597)
50 - 69 years (rate per capita)	0.465 (0.419 - 0.512)	0.464 (0.419 - 0.513)	0.463 (0.417 - 0.513)	0.886 (0.793 - 0.990)	0.884 (0.792 - 0.986)	0.885 (0.788 - 0.993)	0.269 (0.118 - 0.530)	0.269 (0.117 - 0.526)	0.269 (0.117 - 0.528)
70+ years (rate per capita)	0.448 (0.409 - 0.485)	0.454 (0.412 - 0.494)	0.445 (0.407 - 0.481)	0.876 (0.785 - 0.969)	0.881 (0.784 - 0.975)	0.876 (0.785 - 0.968)	0.250 (0.111 - 0.481)	0.253 (0.113 - 0.481)	0.247 (0.111 - 0.470)
All ages (rate per capita)	0.387 (0.362 - 0.411)	0.421 (0.395 - 0.446)	0.422 (0.398 - 0.446)	0.623 (0.577 - 0.669)	0.675 (0.629 - 0.720)	0.691 (0.646 - 0.737)	0.229 (0.101 - 0.440)	0.249 (0.109 - 0.478)	0.250 (0.109 - 0.487)
Age-standardized (rate per capita)	0.425 (0.402 - 0.447)	0.425 (0.401 - 0.447)	0.425 (0.401 - 0.447)	0.710 (0.667 - 0.752)	0.710 (0.667 - 0.752)	0.710 (0.667 - 0.752)	0.250 (0.108 - 0.481)	0.250 (0.109 - 0.485)	0.250 (0.108 - 0.484)
Total number of cases (thousands)	1,416 (1,325 - 1,505)	1,997 (1,875 - 2,116)	2,273 (2,142 - 2,401)	2,281 (2,115 - 2,452)	3,203 (2,988 - 3,420)	3,721 (3,476 - 3,966)	1 (0 - 2)	1 (1 - 2)	1 (1 - 3)
**Uzbekistan**									
5 -14 years (rate per capita)	0.357 (0.297 - 0.413)	0.377 (0.316 - 0.436)	0.359 (0.299 - 0.416)	0.568 (0.452 - 0.684)	0.588 (0.469 - 0.706)	0.571 (0.455 - 0.688)	0.214 (0.090 - 0.415)	0.226 (0.096 - 0.434)	0.215 (0.091 - 0.412)
15 - 49 years (rate per capita)	0.510 (0.472 - 0.548)	0.509 (0.474 - 0.548)	0.505 (0.469 - 0.543)	0.788 (0.721 - 0.859)	0.791 (0.726 - 0.857)	0.797 (0.731 - 0.863)	0.303 (0.134 - 0.598)	0.303 (0.131 - 0.600)	0.300 (0.130 - 0.597)
50 - 69 years (rate per capita)	0.465 (0.419 - 0.512)	0.465 (0.419 - 0.514)	0.463 (0.417 - 0.513)	0.886 (0.792 - 0.990)	0.884 (0.794 - 0.986)	0.885 (0.789 - 0.993)	0.269 (0.115 - 0.525)	0.269 (0.118 - 0.526)	0.269 (0.117 - 0.529)
70+ years (rate per capita)	0.439 (0.404 - 0.473)	0.451 (0.411 - 0.490)	0.441 (0.404 - 0.475)	0.871 (0.784 - 0.961)	0.879 (0.785 - 0.972)	0.874 (0.783 - 0.965)	0.244 (0.109 - 0.461)	0.251 (0.112 - 0.474)	0.245 (0.110 - 0.465)
All ages (rate per capita)	0.384 (0.359 - 0.408)	0.422 (0.396 - 0.447)	0.417 (0.393 - 0.440)	0.619 (0.574 - 0.666)	0.677 (0.631 - 0.723)	0.685 (0.639 - 0.730)	0.227 (0.100 - 0.438)	0.250 (0.110 - 0.483)	0.247 (0.108 - 0.480)
Age-standardized (rate per capita)	0.425 (0.401 - 0.447)	0.425 (0.401 - 0.447)	0.424 (0.401 - 0.447)	0.710 (0.667 - 0.752)	0.710 (0.667 - 0.752)	0.710 (0.667 - 0.752)	0.250 (0.108 - 0.485)	0.250 (0.109 - 0.483)	0.250 (0.108 - 0.485)
Total number of cases (thousands)	7,877 (7,373 - 8,371)	10,989 (10,310 - 11,638)	12,481 (11,759 - 13,181)	12,711 (11,787 - 13,656)	17,630 (16,436 - 18,823)	20,494 (19,145 - 21,848)	5 (2 - 9)	7 (3 - 13)	7 (3 - 14)
**Latin America and Caribbean**									
5 -14 years (rate per capita)	0.356 (0.301 - 0.416)	0.359 (0.304 - 0.419)	0.367 (0.309 - 0.428)	0.471 (0.376 - 0.576)	0.468 (0.374 - 0.573)	0.474 (0.380 - 0.582)	0.268 (0.119 - 0.507)	0.274 (0.121 - 0.513)	0.280 (0.124 - 0.529)
15 - 49 years (rate per capita)	0.467 (0.432 - 0.504)	0.466 (0.433 - 0.501)	0.465 (0.431 - 0.499)	0.748 (0.684 - 0.813)	0.750 (0.688 - 0.812)	0.746 (0.684 - 0.808)	0.352 (0.154 - 0.681)	0.350 (0.153 - 0.681)	0.350 (0.153 - 0.678)
50 - 69 years (rate per capita)	0.427 (0.385 - 0.472)	0.427 (0.384 - 0.471)	0.426 (0.385 - 0.471)	0.830 (0.747 - 0.911)	0.831 (0.747 - 0.913)	0.829 (0.748 - 0.910)	0.308 (0.136 - 0.595)	0.308 (0.136 - 0.593)	0.308 (0.136 - 0.591)
70+ years (rate per capita)	0.406 (0.370 - 0.443)	0.405 (0.371 - 0.440)	0.402 (0.369 - 0.436)	0.801 (0.722 - 0.874)	0.807 (0.733 - 0.878)	0.809 (0.735 - 0.876)	0.288 (0.133 - 0.544)	0.283 (0.130 - 0.532)	0.279 (0.127 - 0.522)
All ages (rate per capita)	0.374 (0.351 - 0.398)	0.390 (0.367 - 0.412)	0.399 (0.377 - 0.420)	0.593 (0.554 - 0.634)	0.629 (0.590 - 0.667)	0.651 (0.614 - 0.688)	0.280 (0.124 - 0.532)	0.291 (0.130 - 0.554)	0.298 (0.133 - 0.567)
Age-standardized (rate per capita)	0.393 (0.371 - 0.413)	0.394 (0.372 - 0.414)	0.394 (0.373 - 0.414)	0.655 (0.618 - 0.692)	0.654 (0.618 - 0.690)	0.650 (0.615 - 0.687)	0.292 (0.130 - 0.555)	0.292 (0.131 - 0.558)	0.293 (0.131 - 0.559)
Total number of cases (thousands)	149,118 (139,667 - 158,486)	197,161 (185,668 - 208,124)	227,477 (214,601 - 239,374)	236,448 (220,732 - 252,627)	317,709 (298,418 - 336,996)	370,953 (349,941 - 392,132)	112 (49 - 212)	147 (66 - 280)	170 (76 - 323)
**Central Latin America**									
5 -14 years (rate per capita)	0.334 (0.273 - 0.405)	0.341 (0.278 - 0.413)	0.348 (0.285 - 0.420)	0.360 (0.272 - 0.471)	0.360 (0.274 - 0.470)	0.368 (0.283 - 0.478)	0.319 (0.140 - 0.623)	0.325 (0.144 - 0.630)	0.333 (0.145 - 0.648)
15 - 49 years (rate per capita)	0.444 (0.404 - 0.484)	0.439 (0.402 - 0.477)	0.437 (0.400 - 0.473)	0.564 (0.501 - 0.632)	0.566 (0.506 - 0.631)	0.568 (0.508 - 0.634)	0.424 (0.185 - 0.806)	0.419 (0.182 - 0.793)	0.416 (0.182 - 0.783)
50 - 69 years (rate per capita)	0.387 (0.341 - 0.439)	0.384 (0.338 - 0.435)	0.384 (0.338 - 0.438)	0.600 (0.521 - 0.692)	0.600 (0.518 - 0.696)	0.598 (0.518 - 0.691)	0.358 (0.155 - 0.676)	0.357 (0.154 - 0.678)	0.357 (0.153 - 0.676)

	Prevalence 1990	Prevalence 2005	Prevalence 2015	Incidence 1990	Incidence 2005	Incidence 2015	YLD rate 1990	YLD rate 2005	YLD rate 2015
70+ years (rate per capita)	0.365 (0.326 - 0.405)	0.364 (0.326 - 0.405)	0.360 (0.324 - 0.398)	0.558 (0.490 - 0.627)	0.558 (0.489 - 0.631)	0.554 (0.488 - 0.623)	0.325 (0.147 - 0.617)	0.323 (0.145 - 0.616)	0.319 (0.143 - 0.603)
All ages (rate per capita)	0.348 (0.321 - 0.375)	0.361 (0.334 - 0.387)	0.369 (0.343 - 0.395)	0.438 (0.398 - 0.485)	0.464 (0.424 - 0.507)	0.483 (0.444 - 0.524)	0.330 (0.146 - 0.631)	0.343 (0.150 - 0.651)	0.350 (0.153 - 0.665)
Age-standardized (rate per capita)	0.366 (0.342 - 0.391)	0.366 (0.342 - 0.391)	0.366 (0.341 - 0.390)	0.487 (0.449 - 0.528)	0.486 (0.449 - 0.528)	0.487 (0.450 - 0.528)	0.345 (0.152 - 0.660)	0.346 (0.151 - 0.658)	0.346 (0.152 - 0.657)
Total number of cases (thousands)	58,800 (54,242 - 63,352)	79,062 (73,190 - 84,719)	93,012 (86,386 - 99,483)	73,951 (67,178 - 82,017)	101,450 (92,765 - 110,956)	121,513 (111,773 - 132,002)	56 (25 - 107)	75 (33 - 142)	88 (39 - 167)
**Colombia**									
5 -14 years (rate per capita)	0.291 (0.234 - 0.362)	0.311 (0.248 - 0.381)	0.311 (0.248 - 0.381)	0.316 (0.234 - 0.424)	0.330 (0.247 - 0.446)	0.330 (0.247 - 0.446)	0.289 (0.125 - 0.580)	0.310 (0.134 - 0.597)	0.310 (0.136 - 0.607)
15 - 49 years (rate per capita)	0.390 (0.354 - 0.428)	0.388 (0.354 - 0.423)	0.385 (0.350 - 0.420)	0.522 (0.466 - 0.583)	0.523 (0.468 - 0.583)	0.525 (0.469 - 0.585)	0.387 (0.168 - 0.740)	0.384 (0.168 - 0.725)	0.381 (0.167 - 0.722)
50 - 69 years (rate per capita)	0.336 (0.293 - 0.382)	0.336 (0.293 - 0.384)	0.336 (0.294 - 0.384)	0.560 (0.488 - 0.642)	0.560 (0.487 - 0.643)	0.560 (0.488 - 0.642)	0.324 (0.141 - 0.618)	0.325 (0.139 - 0.617)	0.325 (0.138 - 0.621)
70+ years (rate per capita)	0.318 (0.285 - 0.358)	0.316 (0.282 - 0.353)	0.315 (0.282 - 0.351)	0.523 (0.458 - 0.589)	0.519 (0.458 - 0.584)	0.517 (0.457 - 0.583)	0.295 (0.132 - 0.567)	0.293 (0.131 - 0.569)	0.291 (0.131 - 0.564)
All ages (rate per capita)	0.310 (0.284 - 0.336)	0.327 (0.302 - 0.352)	0.331 (0.308 - 0.355)	0.410 (0.374 - 0.454)	0.440 (0.405 - 0.483)	0.457 (0.422 - 0.498)	0.306 (0.134 - 0.584)	0.323 (0.142 - 0.613)	0.326 (0.145 - 0.621)
Age-standardized (rate per capita)	0.322 (0.299 - 0.345)	0.323 (0.301 - 0.347)	0.323 (0.301 - 0.347)	0.448 (0.415 - 0.487)	0.449 (0.415 - 0.488)	0.449 (0.415 - 0.488)	0.316 (0.138 - 0.599)	0.318 (0.141 - 0.604)	0.318 (0.141 - 0.604)
Total number of cases (thousands)	10,649 (9,767 - 11,541)	14,156 (13,091 - 15,218)	15,977 (14,846 - 17,131)	14,075 (12,848 - 15,585)	19,066 (17,519 - 20,918)	22,080 (20,361 - 24,032)	11 (5 - 20)	14 (6 - 27)	16 (7 - 30)
**Costa Rica**									
5 -14 years (rate per capita)	0.328 (0.260 - 0.410)	0.346 (0.275 - 0.429)	0.343 (0.273 - 0.425)	0.362 (0.264 - 0.484)	0.377 (0.276 - 0.499)	0.374 (0.274 - 0.497)	0.326 (0.142 - 0.644)	0.345 (0.151 - 0.682)	0.342 (0.150 - 0.666)
15 - 49 years (rate per capita)	0.442 (0.385 - 0.493)	0.441 (0.386 - 0.491)	0.438 (0.383 - 0.489)	0.587 (0.500 - 0.682)	0.586 (0.500 - 0.677)	0.588 (0.504 - 0.680)	0.439 (0.185 - 0.834)	0.438 (0.186 - 0.840)	0.435 (0.184 - 0.822)
50 - 69 years (rate per capita)	0.390 (0.328 - 0.463)	0.390 (0.327 - 0.463)	0.390 (0.328 - 0.462)	0.614 (0.503 - 0.755)	0.614 (0.503 - 0.756)	0.614 (0.504 - 0.753)	0.378 (0.164 - 0.731)	0.377 (0.163 - 0.738)	0.378 (0.163 - 0.732)
70+ years (rate per capita)	0.368 (0.315 - 0.424)	0.365 (0.313 - 0.420)	0.363 (0.313 - 0.417)	0.567 (0.473 - 0.672)	0.566 (0.474 - 0.668)	0.564 (0.474 - 0.666)	0.341 (0.154 - 0.649)	0.338 (0.150 - 0.643)	0.336 (0.147 - 0.641)
All ages (rate per capita)	0.352 (0.319 - 0.386)	0.375 (0.342 - 0.409)	0.379 (0.346 - 0.413)	0.463 (0.411 - 0.522)	0.499 (0.444 - 0.556)	0.516 (0.463 - 0.574)	0.348 (0.151 - 0.668)	0.370 (0.160 - 0.707)	0.373 (0.163 - 0.710)
Age-standardized (rate per capita)	0.369 (0.337 - 0.402)	0.369 (0.337 - 0.402)	0.369 (0.337 - 0.402)	0.500 (0.450 - 0.554)	0.500 (0.450 - 0.554)	0.500 (0.450 - 0.554)	0.363 (0.159 - 0.695)	0.363 (0.158 - 0.693)	0.363 (0.158 - 0.690)
Total number of cases (thousands)	1,089 (988 - 1,195)	1,595 (1,453 - 1,741)	1,821 (1,663 - 1,986)	1,434 (1,271 - 1,615)	2,123 (1,890 - 2,363)	2,483 (2,224 - 2,761)	1 (0 - 2)	2 (1 - 3)	2 (1 - 3)
**El Salvador**									
5 -14 years (rate per capita)	0.335 (0.267 - 0.417)	0.342 (0.272 - 0.424)	0.351 (0.279 - 0.435)	0.349 (0.257 - 0.468)	0.354 (0.261 - 0.470)	0.360 (0.266 - 0.477)	0.200 (0.084 - 0.389)	0.205 (0.085 - 0.401)	0.210 (0.089 - 0.409)
15 - 49 years (rate per capita)	0.448 (0.392 - 0.498)	0.445 (0.389 - 0.495)	0.444 (0.388 - 0.494)	0.554 (0.472 - 0.643)	0.557 (0.475 - 0.646)	0.557 (0.475 - 0.648)	0.266 (0.114 - 0.521)	0.264 (0.111 - 0.519)	0.264 (0.113 - 0.513)
50 - 69 years (rate per capita)	0.390 (0.328 - 0.462)	0.391 (0.330 - 0.463)	0.391 (0.329 - 0.465)	0.590 (0.482 - 0.725)	0.589 (0.482 - 0.725)	0.589 (0.481 - 0.727)	0.225 (0.096 - 0.445)	0.226 (0.096 - 0.445)	0.226 (0.096 - 0.447)
70+ years (rate per capita)	0.372 (0.315 - 0.431)	0.369 (0.314 - 0.426)	0.364 (0.313 - 0.417)	0.549 (0.456 - 0.651)	0.547 (0.457 - 0.648)	0.544 (0.458 - 0.643)	0.206 (0.094 - 0.388)	0.204 (0.093 - 0.385)	0.201 (0.092 - 0.375)
All ages (rate per capita)	0.345 (0.313 - 0.380)	0.364 (0.331 - 0.399)	0.376 (0.343 - 0.411)	0.423 (0.374 - 0.475)	0.453 (0.403 - 0.505)	0.476 (0.426 - 0.529)	0.204 (0.089 - 0.389)	0.215 (0.092 - 0.409)	0.222 (0.096 - 0.422)
Age-standardized (rate per capita)	0.369 (0.337 - 0.402)	0.369 (0.338 - 0.403)	0.369 (0.338 - 0.403)	0.478 (0.430 - 0.528)	0.478 (0.430 - 0.528)	0.478 (0.430 - 0.528)	0.217 (0.094 - 0.413)	0.217 (0.094 - 0.414)	0.217 (0.094 - 0.413)
Total number of cases (thousands)	1,825 (1,653 - 2,008)	2,168 (1,972 - 2,377)	2,310 (2,108 - 2,520)	2,239 (1,980 - 2,515)	2,702 (2,403 - 3,010)	2,922 (2,616 - 3,245)	1 (0 - 2)	1 (1 - 2)	1 (1 - 3)
**Guatemala**									
5 -14 years (rate per capita)	0.326 (0.259 - 0.408)	0.331 (0.263 - 0.413)	0.340 (0.270 - 0.421)	0.361 (0.263 - 0.483)	0.365 (0.267 - 0.487)	0.372 (0.272 - 0.495)	0.323 (0.141 - 0.639)	0.330 (0.144 - 0.641)	0.339 (0.148 - 0.671)
15 - 49 years (rate per capita)	0.450 (0.394 - 0.500)	0.449 (0.393 - 0.499)	0.447 (0.390 - 0.497)	0.581 (0.492 - 0.675)	0.582 (0.493 - 0.678)	0.584 (0.494 - 0.677)	0.444 (0.190 - 0.843)	0.443 (0.188 - 0.837)	0.442 (0.186 - 0.836)
50 - 69 years (rate per capita)	0.390 (0.328 - 0.464)	0.390 (0.328 - 0.463)	0.391 (0.329 - 0.464)	0.614 (0.503 - 0.758)	0.614 (0.503 - 0.754)	0.613 (0.503 - 0.752)	0.375 (0.162 - 0.722)	0.374 (0.163 - 0.729)	0.374 (0.163 - 0.718)
70+ years (rate per capita)	0.371 (0.314 - 0.429)	0.370 (0.315 - 0.427)	0.364 (0.313 - 0.417)	0.569 (0.472 - 0.678)	0.568 (0.472 - 0.676)	0.565 (0.474 - 0.667)	0.341 (0.154 - 0.648)	0.339 (0.150 - 0.648)	0.332 (0.146 - 0.631)
All ages (rate per capita)	0.330 (0.298 - 0.365)	0.343 (0.310 - 0.377)	0.356 (0.323 - 0.390)	0.421 (0.369 - 0.476)	0.440 (0.386 - 0.497)	0.460 (0.406 - 0.517)	0.325 (0.141 - 0.626)	0.338 (0.146 - 0.644)	0.351 (0.152 - 0.673)
Age-standardized (rate per capita)	0.369 (0.337 - 0.402)	0.369 (0.337 - 0.402)	0.369 (0.337 - 0.402)	0.501 (0.450 - 0.555)	0.501 (0.449 - 0.555)	0.501 (0.450 - 0.555)	0.361 (0.158 - 0.690)	0.361 (0.157 - 0.685)	0.361 (0.157 - 0.689)
Total number of cases (thousands)	3,041 (2,741 - 3,357)	4,526 (4,094 - 4,982)	5,823 (5,284 - 6,386)	3,874 (3,399 - 4,383)	5,804 (5,095 - 6,558)	7,529 (6,636 - 8,450)	3 (1 - 6)	4 (2 - 9)	6 (2 - 11)
**Honduras**									
5 -14 years (rate per capita)	0.326 (0.259 - 0.408)	0.337 (0.268 - 0.418)	0.346 (0.276 - 0.430)	0.343 (0.251 - 0.462)	0.350 (0.257 - 0.468)	0.357 (0.264 - 0.474)	0.194 (0.081 - 0.378)	0.201 (0.084 - 0.393)	0.207 (0.088 - 0.412)
15 - 49 years (rate per capita)	0.450 (0.393 - 0.500)	0.448 (0.392 - 0.499)	0.445 (0.389 - 0.496)	0.553 (0.469 - 0.645)	0.554 (0.471 - 0.645)	0.556 (0.474 - 0.647)	0.267 (0.113 - 0.524)	0.266 (0.112 - 0.522)	0.265 (0.112 - 0.521)
50 - 69 years (rate per capita)	0.390 (0.329 - 0.463)	0.389 (0.326 - 0.464)	0.390 (0.327 - 0.463)	0.590 (0.482 - 0.726)	0.590 (0.481 - 0.724)	0.590 (0.482 - 0.725)	0.226 (0.096 - 0.450)	0.226 (0.096 - 0.447)	0.226 (0.096 - 0.446)
70+ years (rate per capita)	0.369 (0.315 - 0.426)	0.366 (0.314 - 0.420)	0.362 (0.312 - 0.414)	0.547 (0.458 - 0.651)	0.545 (0.458 - 0.644)	0.543 (0.459 - 0.639)	0.205 (0.094 - 0.385)	0.203 (0.092 - 0.383)	0.200 (0.092 - 0.381)
All ages (rate per capita)	0.330 (0.297 - 0.365)	0.351 (0.317 - 0.387)	0.369 (0.335 - 0.405)	0.400 (0.352 - 0.453)	0.428 (0.378 - 0.482)	0.459 (0.408 - 0.515)	0.195 (0.085 - 0.370)	0.208 (0.090 - 0.396)	0.219 (0.094 - 0.421)
Age-standardized (rate per capita)	0.369 (0.337 - 0.402)	0.369 (0.337 - 0.402)	0.369 (0.337 - 0.402)	0.478 (0.430 - 0.528)	0.478 (0.430 - 0.528)	0.478 (0.430 - 0.528)	0.217 (0.094 - 0.413)	0.217 (0.093 - 0.415)	0.217 (0.094 - 0.416)
Total number of cases (thousands)	1,624 (1,462 - 1,796)	2,420 (2,187 - 2,668)	2,990 (2,716 - 3,276)	1,966 (1,734 - 2,226)	2,951 (2,607 - 3,324)	3,713 (3,303 - 4,166)	1 (0 - 2)	1 (1 - 3)	2 (1 - 3)
**Mexico**									
5 -14 years (rate per capita)	0.351 (0.290 - 0.421)	0.351 (0.289 - 0.421)	0.363 (0.299 - 0.432)	0.378 (0.289 - 0.485)	0.367 (0.284 - 0.477)	0.382 (0.299 - 0.490)	0.350 (0.156 - 0.674)	0.350 (0.155 - 0.681)	0.362 (0.158 - 0.708)
15 - 49 years (rate per capita)	0.464 (0.428 - 0.500)	0.455 (0.422 - 0.492)	0.452 (0.419 - 0.488)	0.577 (0.518 - 0.639)	0.580 (0.523 - 0.637)	0.580 (0.526 - 0.640)	0.461 (0.202 - 0.861)	0.452 (0.196 - 0.851)	0.449 (0.195 - 0.849)
50 - 69 years (rate per capita)	0.404 (0.362 - 0.450)	0.401 (0.357 - 0.448)	0.401 (0.359 - 0.449)	0.612 (0.539 - 0.699)	0.614 (0.534 - 0.699)	0.611 (0.535 - 0.695)	0.389 (0.166 - 0.740)	0.387 (0.167 - 0.735)	0.387 (0.165 - 0.719)
70+ years (rate per capita)	0.380 (0.344 - 0.416)	0.378 (0.343 - 0.416)	0.373 (0.341 - 0.408)	0.570 (0.506 - 0.637)	0.571 (0.506 - 0.637)	0.566 (0.501 - 0.634)	0.350 (0.159 - 0.665)	0.349 (0.156 - 0.669)	0.344 (0.155 - 0.649)
All ages (rate per capita)	0.365 (0.339 - 0.391)	0.375 (0.350 - 0.399)	0.384 (0.360 - 0.408)	0.451 (0.412 - 0.497)	0.474 (0.437 - 0.516)	0.494 (0.458 - 0.534)	0.361 (0.159 - 0.689)	0.370 (0.163 - 0.706)	0.379 (0.167 - 0.716)
Age-standardized (rate per capita)	0.381 (0.359 - 0.403)	0.380 (0.358 - 0.402)	0.380 (0.358 - 0.403)	0.499 (0.462 - 0.538)	0.497 (0.462 - 0.536)	0.498 (0.463 - 0.536)	0.375 (0.166 - 0.718)	0.374 (0.165 - 0.711)	0.374 (0.165 - 0.707)
Total number of cases (thousands)	31,263 (29,007 - 33,482)	41,113 (38,429 - 43,765)	48,749 (45,737 - 51,781)	38,629 (35,324 - 42,530)	52,049 (47,961 - 56,594)	62,757 (58,192 - 67,822)	31 (14 - 59)	41 (18 - 78)	48 (21 - 91)
**Nicaragua**									
5 -14 years (rate per capita)	0.327 (0.259 - 0.408)	0.342 (0.272 - 0.424)	0.340 (0.270 - 0.422)	0.343 (0.251 - 0.463)	0.354 (0.261 - 0.470)	0.352 (0.259 - 0.470)	0.195 (0.082 - 0.381)	0.205 (0.085 - 0.402)	0.204 (0.084 - 0.399)
15 - 49 years (rate per capita)	0.450 (0.394 - 0.501)	0.447 (0.392 - 0.498)	0.442 (0.386 - 0.493)	0.553 (0.470 - 0.642)	0.555 (0.472 - 0.645)	0.558 (0.478 - 0.650)	0.268 (0.114 - 0.521)	0.266 (0.112 - 0.522)	0.263 (0.111 - 0.515)
50 - 69 years (rate per capita)	0.390 (0.328 - 0.464)	0.390 (0.326 - 0.463)	0.390 (0.327 - 0.466)	0.589 (0.482 - 0.727)	0.589 (0.482 - 0.722)	0.591 (0.481 - 0.733)	0.226 (0.096 - 0.448)	0.227 (0.096 - 0.453)	0.226 (0.096 - 0.450)
70+ years (rate per capita)	0.373 (0.315 - 0.432)	0.367 (0.314 - 0.421)	0.362 (0.312 - 0.416)	0.549 (0.457 - 0.653)	0.546 (0.458 - 0.646)	0.544 (0.459 - 0.640)	0.207 (0.093 - 0.392)	0.203 (0.093 - 0.385)	0.200 (0.092 - 0.382)
All ages (rate per capita)	0.332 (0.299 - 0.367)	0.361 (0.327 - 0.396)	0.368 (0.335 - 0.403)	0.401 (0.353 - 0.454)	0.443 (0.392 - 0.498)	0.464 (0.414 - 0.518)	0.197 (0.085 - 0.374)	0.214 (0.092 - 0.409)	0.218 (0.094 - 0.419)
Age-standardized (rate per capita)	0.369 (0.337 - 0.402)	0.369 (0.337 - 0.402)	0.369 (0.337 - 0.402)	0.478 (0.430 - 0.528)	0.478 (0.430 - 0.528)	0.478 (0.430 - 0.528)	0.217 (0.094 - 0.416)	0.218 (0.094 - 0.415)	0.218 (0.094 - 0.417)
Total number of cases (thousands)	1,380 (1,244 - 1,527)	1,945 (1,760 - 2,136)	2,243 (2,040 - 2,453)	1,666 (1,467 - 1,887)	2,385 (2,111 - 2,684)	2,825 (2,519 - 3,152)	1 (0 - 2)	1 (0 - 2)	1 (1 - 3)
**Panama**									
5 -14 years (rate per capita)	0.409 (0.338 - 0.488)	0.395 (0.323 - 0.473)	0.398 (0.326 - 0.476)	0.365 (0.290 - 0.457)	0.356 (0.279 - 0.456)	0.358 (0.281 - 0.457)	0.244 (0.104 - 0.480)	0.236 (0.101 - 0.457)	0.238 (0.101 - 0.461)
56 - 49 years	0.492 (0.455 - 0.528)	0.484 (0.448 - 0.521)	0.483 (0.446 - 0.519)	0.531 (0.478 - 0.588)	0.535 (0.484 - 0.590)	0.536 (0.486 - 0.589)	0.292 (0.128 - 0.573)	0.288 (0.126 - 0.571)	0.287 (0.125 - 0.566)
91 - 69 years	0.433 (0.388 - 0.480)	0.433 (0.386 - 0.481)	0.433 (0.386 - 0.481)	0.587 (0.519 - 0.672)	0.588 (0.519 - 0.669)	0.588 (0.519 - 0.669)	0.252 (0.108 - 0.494)	0.251 (0.107 - 0.487)	0.251 (0.107 - 0.489)
70+ years (rate per capita)	0.413 (0.374 - 0.451)	0.408 (0.372 - 0.446)	0.407 (0.371 - 0.443)	0.554 (0.491 - 0.620)	0.552 (0.491 - 0.619)	0.551 (0.491 - 0.617)	0.230 (0.102 - 0.439)	0.227 (0.101 - 0.436)	0.225 (0.100 - 0.427)
All ages (rate per capita)	0.402 (0.376 - 0.430)	0.407 (0.382 - 0.431)	0.411 (0.386 - 0.434)	0.431 (0.394 - 0.469)	0.451 (0.416 - 0.487)	0.463 (0.429 - 0.497)	0.238 (0.104 - 0.458)	0.241 (0.104 - 0.463)	0.243 (0.104 - 0.463)
Age-standardized (rate per capita)	0.414 (0.390 - 0.438)	0.411 (0.387 - 0.434)	0.411 (0.387 - 0.434)	0.468 (0.434 - 0.503)	0.468 (0.434 - 0.501)	0.468 (0.434 - 0.501)	0.244 (0.106 - 0.472)	0.242 (0.105 - 0.466)	0.242 (0.104 - 0.463)
Total number of cases (thousands)	995 (931 - 1,064)	1,351 (1,268 - 1,431)	1,614 (1,516 - 1,706)	1,067 (975 - 1,160)	1,499 (1,384 - 1,618)	1,819 (1,685 - 1,952)	1 (0 - 1)	1 (0 - 2)	1 (0 - 2)
**Venezuela**									
5 -14 years (rate per capita)	0.332 (0.264 - 0.414)	0.342 (0.272 - 0.423)	0.340 (0.271 - 0.422)	0.366 (0.267 - 0.488)	0.373 (0.273 - 0.496)	0.372 (0.272 - 0.495)	0.331 (0.145 - 0.655)	0.341 (0.149 - 0.673)	0.340 (0.149 - 0.680)
15 - 49 years (rate per capita)	0.445 (0.389 - 0.495)	0.443 (0.387 - 0.493)	0.440 (0.385 - 0.491)	0.585 (0.496 - 0.680)	0.585 (0.499 - 0.678)	0.587 (0.501 - 0.679)	0.441 (0.186 - 0.836)	0.439 (0.186 - 0.829)	0.437 (0.186 - 0.832)

	Prevalence 1990	Prevalence 2005	Prevalence 2015	Incidence 1990	Incidence 2005	Incidence 2015	YLD rate 1990	YLD rate 2005	YLD rate 2015
**50 - 69 years (rate per capita)**	0.390 (0.327 - 0.463)	0.390 (0.327 - 0.464)	0.390 (0.326 - 0.464)	0.614 (0.503 - 0.755)	0.614 (0.503 - 0.754)	0.614 (0.502 - 0.757)	0.378 (0.163 - 0.726)	0.377 (0.167 - 0.734)	0.377 (0.163 - 0.720)
**70+ years (rate per capita)**	0.370 (0.314 - 0.427)	0.367 (0.314 - 0.422)	0.366 (0.313 - 0.421)	0.568 (0.472 - 0.676)	0.567 (0.473 - 0.670)	0.566 (0.473 - 0.669)	0.343 (0.152 - 0.653)	0.340 (0.152 - 0.650)	0.338 (0.151 - 0.640)
**All ages (rate per capita)**	0.349 (0.317 - 0.383)	0.366 (0.332 - 0.400)	0.369 (0.337 - 0.403)	0.453 (0.400 - 0.511)	0.481 (0.426 - 0.536)	0.495 (0.441 - 0.550)	0.346 (0.148 - 0.660)	0.361 (0.156 - 0.691)	0.364 (0.158 - 0.696)
**Age-standardized (rate per capita)**	0.369 (0.337 - 0.402)	0.369 (0.337 - 0.402)	0.369 (0.337 - 0.402)	0.500 (0.450 - 0.555)	0.500 (0.450 - 0.555)	0.500 (0.450 - 0.555)	0.363 (0.157 - 0.691)	0.363 (0.158 - 0.689)	0.363 (0.157 - 0.693)
**Total number of cases (thousands)**	6,935 (6,289 - 7,609)	9,788 (8,900 - 10,721)	11,485 (10,485 - 12,549)	9,000 (7,937 - 10,150)	12,871 (11,398 - 14,350)	15,385 (13,732 - 17,110)	7 (3 - 13)	10 (4 - 19)	11 (5 - 22)
**Andean Latin America**									
**5 -14 years (rate per capita)**	0.498 (0.439 - 0.552)	0.502 (0.443 - 0.557)	0.502 (0.443 - 0.556)	0.451 (0.368 - 0.537)	0.453 (0.370 - 0.539)	0.452 (0.370 - 0.538)	0.297 (0.129 - 0.574)	0.300 (0.131 - 0.583)	0.300 (0.130 - 0.584)
**15 - 49 years (rate per capita)**	0.650 (0.614 - 0.683)	0.648 (0.613 - 0.682)	0.646 (0.612 - 0.680)	0.668 (0.615 - 0.728)	0.672 (0.619 - 0.731)	0.676 (0.623 - 0.734)	0.386 (0.171 - 0.755)	0.385 (0.171 - 0.750)	0.384 (0.170 - 0.751)
**50 - 69 years (rate per capita)**	0.598 (0.554 - 0.639)	0.598 (0.554 - 0.639)	0.598 (0.554 - 0.639)	0.791 (0.710 - 0.873)	0.791 (0.709 - 0.873)	0.791 (0.710 - 0.873)	0.346 (0.149 - 0.673)	0.346 (0.150 - 0.677)	0.346 (0.150 - 0.670)
**70+ years (rate per capita)**	0.584 (0.544 - 0.621)	0.577 (0.540 - 0.613)	0.571 (0.535 - 0.605)	0.792 (0.715 - 0.860)	0.792 (0.719 - 0.858)	0.793 (0.720 - 0.857)	0.324 (0.145 - 0.626)	0.320 (0.143 - 0.618)	0.316 (0.142 - 0.611)
**All ages (rate per capita)**	0.513 (0.490 - 0.536)	0.534 (0.511 - 0.558)	0.543 (0.521 - 0.566)	0.534 (0.497 - 0.571)	0.566 (0.530 - 0.603)	0.586 (0.549 - 0.623)	0.303 (0.134 - 0.590)	0.316 (0.139 - 0.613)	0.321 (0.141 - 0.625)
**Age-standardized (rate per capita)**	0.549 (0.528 - 0.571)	0.549 (0.528 - 0.571)	0.549 (0.528 - 0.571)	0.606 (0.571 - 0.641)	0.606 (0.571 - 0.641)	0.606 (0.571 - 0.641)	0.323 (0.142 - 0.628)	0.324 (0.142 - 0.628)	0.324 (0.142 - 0.630)
**Total number of cases (thousands)**	19,992 (19,105 - 20,911)	27,026 (25,866 - 28,218)	31,694 (30,364 - 33,023)	20,797 (19,391 - 22,265)	28,615 (26,820 - 30,490)	34,170 (32,028 - 36,320)	12 (5 - 23)	16 (7 - 31)	19 (8 - 36)
**Bolivia**									
**5 -14 years (rate per capita)**	0.494 (0.435 - 0.549)	0.497 (0.438 - 0.552)	0.502 (0.443 - 0.558)	0.449 (0.365 - 0.537)	0.450 (0.367 - 0.539)	0.453 (0.370 - 0.542)	0.294 (0.129 - 0.568)	0.297 (0.129 - 0.574)	0.300 (0.130 - 0.583)
**15 - 49 years (rate per capita)**	0.650 (0.614 - 0.684)	0.649 (0.613 - 0.683)	0.648 (0.613 - 0.682)	0.667 (0.615 - 0.728)	0.669 (0.616 - 0.730)	0.672 (0.620 - 0.732)	0.385 (0.170 - 0.752)	0.385 (0.170 - 0.755)	0.385 (0.171 - 0.753)
**50 - 69 years (rate per capita)**	0.599 (0.556 - 0.642)	0.598 (0.554 - 0.641)	0.598 (0.555 - 0.641)	0.792 (0.710 - 0.873)	0.791 (0.709 - 0.874)	0.792 (0.709 - 0.873)	0.346 (0.150 - 0.666)	0.346 (0.150 - 0.671)	0.346 (0.150 - 0.670)
**70+ years (rate per capita)**	0.590 (0.549 - 0.630)	0.579 (0.540 - 0.616)	0.569 (0.533 - 0.604)	0.791 (0.712 - 0.863)	0.792 (0.717 - 0.859)	0.793 (0.722 - 0.856)	0.327 (0.145 - 0.632)	0.320 (0.142 - 0.621)	0.314 (0.141 - 0.599)
**All ages (rate per capita)**	0.501 (0.478 - 0.525)	0.518 (0.495 - 0.542)	0.534 (0.511 - 0.557)	0.521 (0.484 - 0.559)	0.545 (0.507 - 0.582)	0.568 (0.531 - 0.604)	0.296 (0.130 - 0.574)	0.307 (0.133 - 0.593)	0.315 (0.139 - 0.614)
**Age-standardized (rate per capita)**	0.549 (0.527 - 0.572)	0.549 (0.527 - 0.572)	0.549 (0.527 - 0.572)	0.606 (0.570 - 0.641)	0.606 (0.570 - 0.641)	0.606 (0.570 - 0.641)	0.323 (0.142 - 0.626)	0.323 (0.140 - 0.626)	0.323 (0.142 - 0.629)
**Total number of cases (thousands)**	3,454 (3,296 - 3,616)	4,754 (4,539 - 4,974)	5,746 (5,499 - 6,001)	3,590 (3,334 - 3,848)	4,998 (4,649 - 5,334)	6,113 (5,720 - 6,505)	2 (1 - 4)	3 (1 - 5)	3 (1 - 7)
**Ecuador**									
**5 -14 years (rate per capita)**	0.496 (0.434 - 0.550)	0.501 (0.438 - 0.556)	0.500 (0.437 - 0.555)	0.450 (0.366 - 0.536)	0.453 (0.370 - 0.539)	0.452 (0.369 - 0.538)	0.296 (0.129 - 0.577)	0.299 (0.129 - 0.573)	0.299 (0.130 - 0.576)
**15 - 49 years (rate per capita)**	0.650 (0.615 - 0.684)	0.648 (0.614 - 0.683)	0.647 (0.613 - 0.681)	0.668 (0.615 - 0.728)	0.673 (0.620 - 0.729)	0.676 (0.623 - 0.732)	0.386 (0.171 - 0.753)	0.385 (0.171 - 0.753)	0.384 (0.169 - 0.748)
**50 - 69 years (rate per capita)**	0.598 (0.554 - 0.639)	0.598 (0.553 - 0.639)	0.598 (0.553 - 0.639)	0.791 (0.706 - 0.871)	0.790 (0.706 - 0.871)	0.791 (0.706 - 0.872)	0.346 (0.150 - 0.666)	0.346 (0.149 - 0.670)	0.345 (0.149 - 0.665)
**70+ years (rate per capita)**	0.578 (0.539 - 0.616)	0.570 (0.533 - 0.605)	0.571 (0.533 - 0.606)	0.794 (0.714 - 0.864)	0.794 (0.717 - 0.861)	0.794 (0.717 - 0.861)	0.320 (0.145 - 0.620)	0.315 (0.141 - 0.609)	0.315 (0.141 - 0.607)
**All ages (rate per capita)**	0.514 (0.491 - 0.537)	0.535 (0.512 - 0.558)	0.544 (0.521 - 0.566)	0.536 (0.499 - 0.574)	0.568 (0.531 - 0.605)	0.587 (0.550 - 0.623)	0.304 (0.135 - 0.589)	0.316 (0.139 - 0.615)	0.321 (0.140 - 0.622)
**Age-standardized (rate per capita)**	0.549 (0.528 - 0.570)	0.549 (0.528 - 0.570)	0.549 (0.528 - 0.570)	0.606 (0.570 - 0.642)	0.606 (0.570 - 0.642)	0.606 (0.570 - 0.642)	0.323 (0.143 - 0.627)	0.323 (0.142 - 0.627)	0.323 (0.141 - 0.628)
**Total number of cases (thousands)**	5,266 (5,027 - 5,502)	7,360 (7,043 - 7,672)	8,781 (8,415 - 9,141)	5,487 (5,115 - 5,880)	7,809 (7,303 - 8,326)	9,476 (8,879 - 10,069)	3 (1 - 6)	4 (2 - 8)	5 (2 - 10)
**Peru**									
**5 -14 years (rate per capita)**	0.500 (0.441 - 0.556)	0.505 (0.445 - 0.561)	0.502 (0.443 - 0.558)	0.451 (0.369 - 0.541)	0.454 (0.371 - 0.543)	0.452 (0.370 - 0.542)	0.298 (0.130 - 0.574)	0.302 (0.131 - 0.585)	0.300 (0.130 - 0.586)
**15 - 49 years (rate per capita)**	0.649 (0.613 - 0.683)	0.648 (0.613 - 0.682)	0.645 (0.612 - 0.680)	0.668 (0.615 - 0.728)	0.673 (0.621 - 0.732)	0.677 (0.625 - 0.736)	0.386 (0.171 - 0.761)	0.385 (0.170 - 0.752)	0.384 (0.170 - 0.751)
**50 - 69 years (rate per capita)**	0.598 (0.553 - 0.641)	0.598 (0.554 - 0.641)	0.598 (0.554 - 0.641)	0.791 (0.709 - 0.876)	0.791 (0.710 - 0.875)	0.791 (0.709 - 0.875)	0.347 (0.149 - 0.675)	0.347 (0.151 - 0.680)	0.347 (0.151 - 0.673)
**70+ years (rate per capita)**	0.585 (0.545 - 0.623)	0.581 (0.541 - 0.618)	0.572 (0.535 - 0.607)	0.791 (0.715 - 0.861)	0.791 (0.717 - 0.859)	0.792 (0.721 - 0.857)	0.325 (0.146 - 0.630)	0.323 (0.144 - 0.622)	0.318 (0.143 - 0.615)
**All ages (rate per capita)**	0.516 (0.493 - 0.540)	0.539 (0.516 - 0.563)	0.547 (0.523 - 0.570)	0.537 (0.498 - 0.575)	0.572 (0.535 - 0.609)	0.592 (0.554 - 0.629)	0.305 (0.134 - 0.595)	0.319 (0.141 - 0.619)	0.323 (0.143 - 0.630)
**Age-standardized (rate per capita)**	0.549 (0.527 - 0.572)	0.549 (0.527 - 0.572)	0.549 (0.527 - 0.572)	0.606 (0.570 - 0.641)	0.606 (0.570 - 0.641)	0.606 (0.570 - 0.641)	0.323 (0.142 - 0.628)	0.324 (0.143 - 0.629)	0.324 (0.143 - 0.632)
**Total number of cases (thousands)**	11,272 (10,761 - 11,804)	14,911 (14,274 - 15,574)	17,166 (16,429 - 17,906)	11,721 (10,874 - 12,559)	15,809 (14,782 - 16,829)	18,581 (17,406 - 19,761)	7 (3 - 13)	9 (4 - 17)	10 (4 - 20)
**Caribbean**									
**5 -14 years (rate per capita)**	0.291 (0.228 - 0.355)	0.296 (0.233 - 0.361)	0.297 (0.233 - 0.362)	0.461 (0.350 - 0.593)	0.467 (0.354 - 0.598)	0.468 (0.355 - 0.598)	0.216 (0.092 - 0.417)	0.214 (0.093 - 0.414)	0.210 (0.091 - 0.408)
**15 - 49 years (rate per capita)**	0.422 (0.372 - 0.471)	0.421 (0.373 - 0.466)	0.420 (0.373 - 0.465)	0.833 (0.728 - 0.938)	0.834 (0.733 - 0.936)	0.833 (0.733 - 0.938)	0.333 (0.144 - 0.644)	0.321 (0.140 - 0.627)	0.312 (0.135 - 0.602)
**50 - 69 years (rate per capita)**	0.399 (0.343 - 0.459)	0.399 (0.343 - 0.459)	0.399 (0.343 - 0.458)	0.898 (0.760 - 1.037)	0.898 (0.761 - 1.036)	0.896 (0.761 - 1.034)	0.317 (0.140 - 0.595)	0.317 (0.141 - 0.599)	0.313 (0.139 - 0.593)
**70+ years (rate per capita)**	0.378 (0.327 - 0.432)	0.376 (0.326 - 0.428)	0.374 (0.325 - 0.426)	0.884 (0.749 - 1.014)	0.879 (0.746 - 1.005)	0.877 (0.746 - 1.002)	0.301 (0.137 - 0.579)	0.293 (0.134 - 0.560)	0.290 (0.131 - 0.558)
**All ages (rate per capita)**	0.340 (0.311 - 0.369)	0.352 (0.322 - 0.380)	0.357 (0.328 - 0.386)	0.666 (0.609 - 0.725)	0.696 (0.639 - 0.753)	0.714 (0.657 - 0.772)	0.265 (0.118 - 0.499)	0.268 (0.119 - 0.506)	0.267 (0.118 - 0.502)
**Age-standardized (rate per capita)**	0.354 (0.325 - 0.382)	0.354 (0.325 - 0.382)	0.354 (0.325 - 0.382)	0.712 (0.655 - 0.769)	0.711 (0.654 - 0.768)	0.709 (0.653 - 0.767)	0.278 (0.124 - 0.524)	0.271 (0.121 - 0.511)	0.265 (0.118 - 0.498)
**Total number of cases (thousands)**	12,152 (11,121 - 13,184)	14,713 (13,488 - 15,892)	16,162 (14,857 - 17,463)	23,814 (21,775 - 25,915)	29,112 (26,715 - 31,515)	32,326 (29,718 - 34,925)	9 (4 - 18)	11 (5 - 21)	12 (5 - 23)
**Antigua and Barbuda**									
**5 -14 years (rate per capita)**	0.278 (0.217 - 0.344)	0.296 (0.232 - 0.362)	0.300 (0.235 - 0.366)	0.454 (0.338 - 0.592)	0.478 (0.362 - 0.619)	0.484 (0.366 - 0.623)	0.277 (0.121 - 0.543)	0.295 (0.128 - 0.581)	0.299 (0.130 - 0.584)
**15 - 49 years (rate per capita)**	0.421 (0.370 - 0.470)	0.421 (0.374 - 0.465)	0.420 (0.372 - 0.465)	0.854 (0.746 - 0.963)	0.858 (0.751 - 0.963)	0.857 (0.751 - 0.958)	0.416 (0.178 - 0.798)	0.415 (0.180 - 0.787)	0.415 (0.182 - 0.791)
**50 - 69 years (rate per capita)**	0.401 (0.344 - 0.460)	0.400 (0.344 - 0.459)	0.399 (0.343 - 0.460)	0.919 (0.774 - 1.062)	0.914 (0.777 - 1.055)	0.911 (0.764 - 1.055)	0.386 (0.170 - 0.728)	0.385 (0.166 - 0.721)	0.384 (0.169 - 0.718)
**70+ years (rate per capita)**	0.382 (0.329 - 0.436)	0.371 (0.324 - 0.420)	0.377 (0.327 - 0.430)	0.901 (0.760 - 1.036)	0.884 (0.754 - 1.007)	0.895 (0.757 - 1.022)	0.354 (0.158 - 0.671)	0.342 (0.151 - 0.640)	0.347 (0.155 - 0.656)
**All ages (rate per capita)**	0.348 (0.318 - 0.377)	0.353 (0.322 - 0.382)	0.361 (0.331 - 0.391)	0.704 (0.641 - 0.770)	0.714 (0.652 - 0.777)	0.739 (0.676 - 0.800)	0.342 (0.150 - 0.652)	0.346 (0.153 - 0.663)	0.354 (0.155 - 0.675)
**Age-standardized (rate per capita)**	0.355 (0.326 - 0.383)	0.355 (0.326 - 0.383)	0.355 (0.326 - 0.383)	0.728 (0.667 - 0.789)	0.727 (0.667 - 0.788)	0.727 (0.667 - 0.788)	0.348 (0.152 - 0.660)	0.347 (0.153 - 0.661)	0.347 (0.152 - 0.665)
**Total number of cases (thousands)**	21 (19 - 23)	29 (27 - 32)	33 (30 - 36)	43 (39 - 47)	59 (54 - 64)	68 (62 - 73)	0 (0 - 0)	0 (0 - 0)	0 (0 - 0)
**The Bahamas**									
**5 -14 years (rate per capita)**	0.288 (0.225 - 0.353)	0.301 (0.236 - 0.366)	0.291 (0.228 - 0.356)	0.451 (0.342 - 0.581)	0.468 (0.357 - 0.596)	0.456 (0.346 - 0.586)	0.172 (0.072 - 0.333)	0.180 (0.075 - 0.355)	0.174 (0.074 - 0.344)
**15 - 49 years (rate per capita)**	0.422 (0.372 - 0.470)	0.421 (0.373 - 0.465)	0.420 (0.373 - 0.466)	0.816 (0.714 - 0.921)	0.819 (0.723 - 0.922)	0.820 (0.723 - 0.926)	0.250 (0.106 - 0.491)	0.249 (0.107 - 0.485)	0.249 (0.106 - 0.488)
**50 - 69 years (rate per capita)**	0.399 (0.344 - 0.459)	0.399 (0.344 - 0.459)	0.400 (0.344 - 0.459)	0.876 (0.743 - 1.010)	0.876 (0.742 - 1.009)	0.876 (0.742 - 1.009)	0.231 (0.100 - 0.449)	0.230 (0.102 - 0.454)	0.231 (0.100 - 0.453)
**70+ years (rate per capita)**	0.383 (0.329 - 0.439)	0.381 (0.328 - 0.435)	0.380 (0.329 - 0.433)	0.869 (0.733 - 1.005)	0.866 (0.734 - 0.998)	0.864 (0.733 - 0.993)	0.212 (0.096 - 0.411)	0.210 (0.094 - 0.402)	0.209 (0.095 - 0.403)
**All ages (rate per capita)**	0.342 (0.311 - 0.372)	0.363 (0.332 - 0.392)	0.366 (0.335 - 0.396)	0.653 (0.595 - 0.714)	0.706 (0.648 - 0.765)	0.725 (0.667 - 0.786)	0.202 (0.088 - 0.384)	0.214 (0.092 - 0.403)	0.215 (0.093 - 0.407)
**Age-standardized (rate per capita)**	0.355 (0.326 - 0.383)	0.355 (0.326 - 0.383)	0.355 (0.326 - 0.383)	0.697 (0.644 - 0.753)	0.697 (0.643 - 0.753)	0.697 (0.644 - 0.753)	0.208 (0.091 - 0.395)	0.208 (0.090 - 0.392)	0.208 (0.091 - 0.395)
**Total number of cases (thousands)**	88 (80 - 96)	119 (109 - 128)	142 (130 - 153)	168 (153 - 184)	231 (212 - 250)	281 (258 - 304)	0 (0 - 0)	0 (0 - 0)	0 (0 - 0)
**Barbados**									
**5 -14 years (rate per capita)**	0.298 (0.234 - 0.363)	0.300 (0.235 - 0.365)	0.299 (0.234 - 0.364)	0.481 (0.364 - 0.621)	0.484 (0.366 - 0.623)	0.483 (0.365 - 0.622)	0.297 (0.130 - 0.587)	0.298 (0.129 - 0.589)	0.298 (0.129 - 0.584)
**15 - 49 years (rate per capita)**	0.421 (0.371 - 0.468)	0.419 (0.371 - 0.463)	0.419 (0.371 - 0.463)	0.855 (0.749 - 0.961)	0.860 (0.757 - 0.964)	0.860 (0.757 - 0.963)	0.416 (0.180 - 0.802)	0.414 (0.181 - 0.787)	0.414 (0.180 - 0.787)
**50 - 69 years (rate per capita)**	0.401 (0.344 - 0.459)	0.400 (0.344 - 0.459)	0.400 (0.344 - 0.459)	0.919 (0.779 - 1.061)	0.914 (0.774 - 1.057)	0.916 (0.773 - 1.062)	0.384 (0.168 - 0.727)	0.383 (0.170 - 0.719)	0.383 (0.168 - 0.718)
**70+ years (rate per capita)**	0.378 (0.328 - 0.430)	0.374 (0.326 - 0.424)	0.374 (0.325 - 0.424)	0.896 (0.759 - 1.024)	0.889 (0.755 - 1.014)	0.889 (0.756 - 1.014)	0.349 (0.156 - 0.655)	0.344 (0.153 - 0.655)	0.343 (0.152 - 0.648)
**All ages (rate per capita)**	0.362 (0.332 - 0.390)	0.368 (0.338 - 0.397)	0.369 (0.340 - 0.397)	0.738 (0.673 - 0.801)	0.763 (0.700 - 0.823)	0.775 (0.714 - 0.835)	0.355 (0.155 - 0.671)	0.359 (0.158 - 0.683)	0.359 (0.156 - 0.680)
**Age-standardized (rate per capita)**	0.355 (0.326 - 0.382)	0.355 (0.326 - 0.383)	0.355 (0.326 - 0.382)	0.728 (0.667 - 0.789)	0.727 (0.667 - 0.789)	0.727 (0.667 - 0.788)	0.347 (0.153 - 0.661)	0.347 (0.153 - 0.660)	0.347 (0.152 - 0.660)
**Total number of cases (thousands)**	94 (86 - 102)	101 (92 - 109)	105 (96 - 113)	192 (175 - 209)	209 (192 - 225)	220 (203 - 237)	0 (0 - 0)	0 (0 - 0)	0 (0 - 0)
**Belize**									
**5 -14 years (rate per capita)**	0.287 (0.224 - 0.352)	0.290 (0.226 - 0.354)	0.299 (0.235 - 0.365)	0.450 (0.340 - 0.580)	0.453 (0.344 - 0.584)	0.466 (0.355 - 0.594)	0.171 (0.071 - 0.332)	0.173 (0.072 - 0.339)	0.179 (0.074 - 0.350)

	Prevalence 1990	Prevalence 2005	Prevalence 2015	Incidence 1990	Incidence 2005	Incidence 2015	YLD rate 1990	YLD rate 2005	YLD rate 2015
15 - 49 years (rate per capita)	0.425 (0.373 - 0.476)	0.423 (0.373 - 0.471)	0.422 (0.373 - 0.470)	0.810 (0.708 - 0.918)	0.814 (0.716 - 0.920)	0.815 (0.717 - 0.919)	0.252 (0.106 - 0.490)	0.251 (0.107 - 0.491)	0.250 (0.108 - 0.489)
50 - 69 years (rate per capita)	0.400 (0.343 - 0.460)	0.400 (0.343 - 0.458)	0.399 (0.343 - 0.460)	0.878 (0.741 - 1.013)	0.876 (0.744 - 1.008)	0.874 (0.736 - 1.009)	0.231 (0.101 - 0.458)	0.231 (0.099 - 0.451)	0.231 (0.101 - 0.452)
70+ years (rate per capita)	0.378 (0.326 - 0.431)	0.384 (0.330 - 0.441)	0.381 (0.328 - 0.436)	0.861 (0.732 - 0.987)	0.869 (0.732 - 1.006)	0.865 (0.731 - 0.997)	0.210 (0.096 - 0.403)	0.213 (0.097 - 0.411)	0.211 (0.095 - 0.412)
All ages (rate per capita)	0.313 (0.282 - 0.343)	0.331 (0.300 - 0.362)	0.347 (0.315 - 0.376)	0.584 (0.528 - 0.645)	0.622 (0.564 - 0.683)	0.659 (0.601 - 0.720)	0.185 (0.080 - 0.351)	0.196 (0.085 - 0.371)	0.205 (0.089 - 0.389)
Age-standardized (rate per capita)	0.355 (0.326 - 0.383)	0.355 (0.326 - 0.383)	0.355 (0.326 - 0.383)	0.697 (0.644 - 0.753)	0.697 (0.644 - 0.753)	0.697 (0.644 - 0.753)	0.208 (0.090 - 0.393)	0.208 (0.090 - 0.394)	0.208 (0.090 - 0.396)
Total number of cases (thousands)	59 (53 - 65)	93 (84 - 102)	124 (113 - 135)	110 (100 - 122)	175 (159 - 192)	236 (216 - 258)	0 (0 - 0)	0 (0 - 0)	0 (0 - 0)
**Bermuda**									
5 -14 years (rate per capita)	0.291 (0.228 - 0.356)	0.297 (0.233 - 0.362)	0.298 (0.234 - 0.364)	0.472 (0.357 - 0.612)	0.480 (0.363 - 0.620)	0.481 (0.364 - 0.621)	0.290 (0.125 - 0.579)	0.296 (0.129 - 0.588)	0.297 (0.130 - 0.591)
15 - 49 years (rate per capita)	0.422 (0.372 - 0.472)	0.422 (0.373 - 0.467)	0.421 (0.373 - 0.466)	0.852 (0.743 - 0.958)	0.856 (0.750 - 0.957)	0.856 (0.753 - 0.958)	0.418 (0.180 - 0.804)	0.417 (0.181 - 0.792)	0.416 (0.180 - 0.792)
50 - 69 years (rate per capita)	0.400 (0.344 - 0.460)	0.400 (0.343 - 0.460)	0.400 (0.344 - 0.459)	0.915 (0.773 - 1.059)	0.915 (0.774 - 1.057)	0.914 (0.775 - 1.058)	0.386 (0.169 - 0.726)	0.386 (0.168 - 0.725)	0.386 (0.169 - 0.722)
70+ years (rate per capita)	0.379 (0.328 - 0.433)	0.377 (0.326 - 0.429)	0.376 (0.326 - 0.427)	0.897 (0.758 - 1.028)	0.893 (0.757 - 1.022)	0.892 (0.756 - 1.020)	0.352 (0.158 - 0.666)	0.348 (0.155 - 0.651)	0.347 (0.154 - 0.656)
All ages (rate per capita)	0.356 (0.325 - 0.386)	0.364 (0.333 - 0.393)	0.368 (0.338 - 0.398)	0.712 (0.649 - 0.776)	0.735 (0.673 - 0.797)	0.754 (0.690 - 0.817)	0.350 (0.154 - 0.664)	0.357 (0.156 - 0.674)	0.361 (0.159 - 0.684)
Age-standardized (rate per capita)	0.355 (0.326 - 0.383)	0.355 (0.326 - 0.383)	0.355 (0.326 - 0.383)	0.727 (0.667 - 0.788)	0.727 (0.667 - 0.788)	0.727 (0.667 - 0.788)	0.348 (0.153 - 0.656)	0.348 (0.153 - 0.656)	0.348 (0.153 - 0.659)
Total number of cases (thousands)	19 (18 - 21)	23 (21 - 25)	25 (23 - 27)	39 (35 - 42)	46 (42 - 50)	50 (46 - 55)	0 (0 - 0)	0 (0 - 0)	0 (0 - 0)
**Cuba**									
5 -14 years (rate per capita)	0.297 (0.233 - 0.363)	0.300 (0.235 - 0.365)	0.301 (0.236 - 0.367)	0.480 (0.363 - 0.620)	0.484 (0.366 - 0.624)	0.486 (0.368 - 0.625)	0.295 (0.129 - 0.579)	0.299 (0.132 - 0.596)	0.300 (0.131 - 0.596)
15 - 49 years (rate per capita)	0.422 (0.371 - 0.470)	0.420 (0.371 - 0.464)	0.418 (0.370 - 0.464)	0.853 (0.745 - 0.962)	0.861 (0.755 - 0.964)	0.862 (0.755 - 0.964)	0.417 (0.180 - 0.796)	0.415 (0.182 - 0.782)	0.413 (0.181 - 0.790)
50 - 69 years (rate per capita)	0.400 (0.344 - 0.460)	0.400 (0.344 - 0.460)	0.400 (0.343 - 0.459)	0.915 (0.774 - 1.058)	0.915 (0.774 - 1.058)	0.913 (0.776 - 1.057)	0.386 (0.168 - 0.725)	0.386 (0.170 - 0.732)	0.386 (0.169 - 0.725)
70+ years (rate per capita)	0.377 (0.326 - 0.429)	0.375 (0.325 - 0.426)	0.373 (0.325 - 0.425)	0.893 (0.755 - 1.023)	0.890 (0.755 - 1.018)	0.888 (0.754 - 1.014)	0.350 (0.156 - 0.661)	0.347 (0.156 - 0.663)	0.345 (0.154 - 0.651)
All ages (rate per capita)	0.362 (0.331 - 0.391)	0.371 (0.340 - 0.399)	0.374 (0.345 - 0.404)	0.738 (0.672 - 0.804)	0.770 (0.704 - 0.833)	0.790 (0.726 - 0.855)	0.356 (0.156 - 0.673)	0.363 (0.160 - 0.690)	0.366 (0.160 - 0.693)
Age-standardized (rate per capita)	0.355 (0.326 - 0.383)	0.355 (0.326 - 0.383)	0.355 (0.325 - 0.383)	0.727 (0.667 - 0.788)	0.727 (0.667 - 0.788)	0.727 (0.667 - 0.788)	0.348 (0.153 - 0.660)	0.348 (0.153 - 0.661)	0.348 (0.153 - 0.660)
Total number of cases (thousands)	3,828 (3,503 - 4,134)	4,180 (3,835 - 4,500)	4,266 (3,933 - 4,602)	7,804 (7,101 - 8,500)	8,678 (7,937 - 9,385)	9,002 (8,268 - 9,740)	4 (2 - 7)	4 (2 - 8)	4 (2 - 8)
**Dominica**									
5 -14 years (rate per capita)	0.291 (0.228 - 0.356)	0.297 (0.233 - 0.362)	0.298 (0.234 - 0.364)	0.455 (0.346 - 0.585)	0.463 (0.352 - 0.592)	0.464 (0.353 - 0.593)	0.174 (0.073 - 0.339)	0.177 (0.074 - 0.349)	0.178 (0.075 - 0.352)
15 - 49 years (rate per capita)	0.422 (0.372 - 0.472)	0.422 (0.373 - 0.467)	0.421 (0.373 - 0.466)	0.814 (0.714 - 0.920)	0.818 (0.719 - 0.922)	0.819 (0.720 - 0.921)	0.250 (0.105 - 0.488)	0.250 (0.106 - 0.491)	0.249 (0.107 - 0.490)
50 - 69 years (rate per capita)	0.400 (0.344 - 0.460)	0.400 (0.343 - 0.460)	0.400 (0.344 - 0.459)	0.878 (0.742 - 1.012)	0.877 (0.742 - 1.010)	0.877 (0.741 - 1.009)	0.231 (0.099 - 0.454)	0.230 (0.099 - 0.449)	0.230 (0.100 - 0.448)
70+ years (rate per capita)	0.379 (0.328 - 0.433)	0.377 (0.326 - 0.429)	0.376 (0.326 - 0.427)	0.863 (0.732 - 0.992)	0.860 (0.733 - 0.984)	0.858 (0.733 - 0.981)	0.210 (0.094 - 0.406)	0.208 (0.095 - 0.400)	0.206 (0.093 - 0.397)
All ages (rate per capita)	0.340 (0.311 - 0.369)	0.357 (0.327 - 0.386)	0.360 (0.331 - 0.390)	0.652 (0.595 - 0.711)	0.693 (0.636 - 0.750)	0.707 (0.651 - 0.765)	0.201 (0.086 - 0.381)	0.210 (0.092 - 0.398)	0.212 (0.091 - 0.402)
Age-standardized (rate per capita)	0.355 (0.326 - 0.383)	0.355 (0.326 - 0.383)	0.355 (0.326 - 0.383)	0.697 (0.644 - 0.753)	0.697 (0.644 - 0.753)	0.697 (0.644 - 0.753)	0.208 (0.090 - 0.394)	0.208 (0.091 - 0.393)	0.208 (0.090 - 0.396)
Total number of cases (thousands)	24 (22 - 26)	25 (23 - 27)	26 (24 - 28)	45 (41 - 49)	48 (44 - 52)	51 (47 - 55)	0 (0 - 0)	0 (0 - 0)	0 (0 - 0)
**Dominican Republic**									
5 -14 years (rate per capita)	0.291 (0.228 - 0.356)	0.296 (0.232 - 0.362)	0.295 (0.231 - 0.360)	0.455 (0.346 - 0.585)	0.462 (0.351 - 0.591)	0.461 (0.350 - 0.590)	0.174 (0.073 - 0.341)	0.177 (0.075 - 0.344)	0.177 (0.073 - 0.346)
15 - 49 years (rate per capita)	0.423 (0.372 - 0.473)	0.422 (0.373 - 0.469)	0.421 (0.373 - 0.467)	0.812 (0.713 - 0.918)	0.815 (0.718 - 0.919)	0.817 (0.720 - 0.921)	0.251 (0.107 - 0.492)	0.251 (0.107 - 0.491)	0.250 (0.106 - 0.492)
50 - 69 years (rate per capita)	0.400 (0.343 - 0.460)	0.400 (0.344 - 0.459)	0.400 (0.344 - 0.459)	0.877 (0.739 - 1.009)	0.876 (0.742 - 1.008)	0.877 (0.740 - 1.008)	0.232 (0.101 - 0.447)	0.232 (0.101 - 0.452)	0.232 (0.099 - 0.456)
70+ years (rate per capita)	0.384 (0.328 - 0.442)	0.378 (0.327 - 0.431)	0.374 (0.325 - 0.426)	0.869 (0.731 - 1.006)	0.862 (0.731 - 0.988)	0.856 (0.731 - 0.977)	0.214 (0.096 - 0.418)	0.210 (0.095 - 0.409)	0.208 (0.093 - 0.398)
All ages (rate per capita)	0.328 (0.298 - 0.359)	0.342 (0.312 - 0.371)	0.349 (0.319 - 0.377)	0.618 (0.561 - 0.678)	0.654 (0.598 - 0.711)	0.674 (0.618 - 0.731)	0.194 (0.084 - 0.367)	0.202 (0.088 - 0.382)	0.206 (0.089 - 0.393)
Age-standardized (rate per capita)	0.355 (0.326 - 0.383)	0.355 (0.326 - 0.383)	0.355 (0.326 - 0.383)	0.697 (0.643 - 0.753)	0.697 (0.644 - 0.753)	0.697 (0.644 - 0.753)	0.209 (0.091 - 0.396)	0.209 (0.090 - 0.395)	0.209 (0.091 - 0.399)
Total number of cases (thousands)	2,363 (2,148 - 2,585)	3,163 (2,887 - 3,432)	3,671 (3,360 - 3,972)	4,448 (4,039 - 4,875)	6,040 (5,523 - 6,573)	7,094 (6,507 - 7,700)	1 (1 - 3)	2 (1 - 4)	2 (1 - 4)
**Grenada**									
5 -14 years (rate per capita)	0.279 (0.218 - 0.345)	0.305 (0.239 - 0.370)	0.293 (0.229 - 0.357)	0.440 (0.331 - 0.571)	0.473 (0.362 - 0.600)	0.458 (0.348 - 0.588)	0.166 (0.069 - 0.325)	0.182 (0.077 - 0.358)	0.175 (0.072 - 0.350)
15 - 49 years (rate per capita)	0.424 (0.374 - 0.474)	0.424 (0.375 - 0.474)	0.421 (0.371 - 0.469)	0.812 (0.712 - 0.917)	0.811 (0.712 - 0.916)	0.817 (0.717 - 0.922)	0.251 (0.107 - 0.486)	0.251 (0.107 - 0.489)	0.249 (0.106 - 0.488)
50 - 69 years (rate per capita)	0.401 (0.344 - 0.460)	0.400 (0.344 - 0.459)	0.399 (0.343 - 0.458)	0.881 (0.746 - 1.014)	0.878 (0.744 - 1.009)	0.875 (0.739 - 1.009)	0.231 (0.099 - 0.457)	0.230 (0.099 - 0.452)	0.230 (0.100 - 0.447)
70+ years (rate per capita)	0.384 (0.327 - 0.442)	0.377 (0.328 - 0.430)	0.374 (0.327 - 0.425)	0.871 (0.735 - 1.009)	0.860 (0.728 - 0.986)	0.856 (0.731 - 0.978)	0.213 (0.095 - 0.412)	0.208 (0.094 - 0.398)	0.206 (0.094 - 0.395)
All ages (rate per capita)	0.320 (0.293 - 0.347)	0.355 (0.324 - 0.386)	0.354 (0.324 - 0.382)	0.611 (0.558 - 0.667)	0.675 (0.615 - 0.735)	0.689 (0.633 - 0.749)	0.188 (0.082 - 0.353)	0.209 (0.090 - 0.396)	0.208 (0.090 - 0.394)
Age-standardized (rate per capita)	0.355 (0.326 - 0.383)	0.355 (0.326 - 0.383)	0.355 (0.325 - 0.383)	0.697 (0.643 - 0.753)	0.697 (0.644 - 0.753)	0.697 (0.644 - 0.753)	0.207 (0.090 - 0.393)	0.208 (0.090 - 0.394)	0.208 (0.090 - 0.394)
Total number of cases (thousands)	31 (28 - 33)	37 (33 - 40)	38 (35 - 41)	59 (54 - 64)	70 (64 - 76)	74 (68 - 80)	0 (0 - 0)	0 (0 - 0)	0 (0 - 0)
**Guyana**									
5 -14 years (rate per capita)	0.305 (0.239 - 0.370)	0.292 (0.228 - 0.357)	0.319 (0.250 - 0.389)	0.472 (0.361 - 0.599)	0.456 (0.346 - 0.586)	0.491 (0.376 - 0.622)	0.181 (0.076 - 0.353)	0.174 (0.074 - 0.342)	0.190 (0.080 - 0.368)
15 - 49 years (rate per capita)	0.423 (0.372 - 0.473)	0.421 (0.374 - 0.465)	0.424 (0.376 - 0.471)	0.813 (0.714 - 0.919)	0.820 (0.724 - 0.923)	0.812 (0.713 - 0.912)	0.250 (0.105 - 0.491)	0.248 (0.107 - 0.488)	0.251 (0.106 - 0.489)
50 - 69 years (rate per capita)	0.400 (0.344 - 0.460)	0.399 (0.343 - 0.459)	0.399 (0.343 - 0.458)	0.878 (0.744 - 1.010)	0.875 (0.738 - 1.008)	0.875 (0.735 - 1.011)	0.230 (0.101 - 0.453)	0.230 (0.100 - 0.443)	0.230 (0.100 - 0.445)
70+ years (rate per capita)	0.372 (0.323 - 0.423)	0.379 (0.328 - 0.432)	0.388 (0.330 - 0.448)	0.854 (0.729 - 0.971)	0.862 (0.731 - 0.992)	0.875 (0.734 - 1.019)	0.205 (0.093 - 0.392)	0.209 (0.095 - 0.405)	0.214 (0.096 - 0.410)
All ages (rate per capita)	0.328 (0.298 - 0.357)	0.337 (0.306 - 0.367)	0.359 (0.327 - 0.391)	0.618 (0.563 - 0.678)	0.641 (0.586 - 0.696)	0.686 (0.628 - 0.747)	0.193 (0.084 - 0.369)	0.198 (0.086 - 0.374)	0.211 (0.092 - 0.400)
Age-standardized (rate per capita)	0.355 (0.326 - 0.383)	0.355 (0.326 - 0.383)	0.355 (0.326 - 0.383)	0.697 (0.643 - 0.753)	0.697 (0.643 - 0.753)	0.697 (0.644 - 0.753)	0.208 (0.090 - 0.393)	0.208 (0.091 - 0.393)	0.208 (0.091 - 0.393)
Total number of cases (thousands)	246 (223 - 268)	250 (227 - 272)	277 (252 - 301)	463 (421 - 508)	476 (435 - 517)	528 (484 - 575)	0 (0 - 0)	0 (0 - 0)	0 (0 - 0)
**Haiti**									
5 -14 years (rate per capita)	0.282 (0.220 - 0.348)	0.293 (0.230 - 0.358)	0.294 (0.230 - 0.359)	0.444 (0.334 - 0.573)	0.459 (0.349 - 0.588)	0.460 (0.349 - 0.589)	0.167 (0.070 - 0.333)	0.175 (0.074 - 0.337)	0.175 (0.073 - 0.345)
15 - 49 years (rate per capita)	0.423 (0.372 - 0.473)	0.424 (0.373 - 0.474)	0.422 (0.372 - 0.471)	0.813 (0.714 - 0.919)	0.811 (0.711 - 0.917)	0.815 (0.714 - 0.921)	0.249 (0.106 - 0.483)	0.250 (0.106 - 0.487)	0.249 (0.106 - 0.487)
50 - 69 years (rate per capita)	0.400 (0.344 - 0.459)	0.400 (0.344 - 0.459)	0.400 (0.344 - 0.459)	0.877 (0.742 - 1.009)	0.877 (0.743 - 1.010)	0.876 (0.741 - 1.009)	0.229 (0.099 - 0.445)	0.229 (0.099 - 0.449)	0.228 (0.099 - 0.446)
70+ years (rate per capita)	0.386 (0.329 - 0.446)	0.385 (0.328 - 0.444)	0.384 (0.329 - 0.441)	0.874 (0.734 - 1.016)	0.871 (0.733 - 1.011)	0.870 (0.732 - 1.007)	0.213 (0.095 - 0.406)	0.212 (0.095 - 0.407)	0.209 (0.095 - 0.405)
All ages (rate per capita)	0.313 (0.284 - 0.342)	0.332 (0.301 - 0.363)	0.341 (0.311 - 0.371)	0.589 (0.535 - 0.647)	0.624 (0.568 - 0.684)	0.649 (0.593 - 0.710)	0.184 (0.080 - 0.348)	0.195 (0.085 - 0.371)	0.200 (0.087 - 0.379)
Age-standardized (rate per capita)	0.355 (0.326 - 0.383)	0.355 (0.326 - 0.383)	0.355 (0.326 - 0.383)	0.697 (0.643 - 0.753)	0.697 (0.643 - 0.753)	0.697 (0.643 - 0.753)	0.207 (0.090 - 0.391)	0.207 (0.090 - 0.390)	0.206 (0.090 - 0.390)
Total number of cases (thousands)	2,224 (2,019 - 2,431)	3,077 (2,793 - 3,360)	3,658 (3,330 - 3,979)	4,182 (3,797 - 4,592)	5,785 (5,264 - 6,336)	6,963 (6,358 - 7,615)	1 (1 - 2)	2 (1 - 3)	2 (1 - 4)
**Jamaica**									
5 -14 years (rate per capita)	0.291 (0.228 - 0.356)	0.296 (0.232 - 0.361)	0.302 (0.237 - 0.368)	0.455 (0.346 - 0.586)	0.462 (0.351 - 0.591)	0.470 (0.359 - 0.598)	0.174 (0.073 - 0.341)	0.177 (0.075 - 0.342)	0.180 (0.076 - 0.355)
15 - 49 years (rate per capita)	0.423 (0.372 - 0.474)	0.422 (0.373 - 0.467)	0.421 (0.372 - 0.468)	0.812 (0.712 - 0.918)	0.817 (0.718 - 0.921)	0.817 (0.717 - 0.921)	0.251 (0.107 - 0.493)	0.250 (0.107 - 0.488)	0.249 (0.106 - 0.492)
50 - 69 years (rate per capita)	0.401 (0.344 - 0.460)	0.400 (0.344 - 0.459)	0.400 (0.344 - 0.460)	0.880 (0.744 - 1.013)	0.876 (0.742 - 1.009)	0.877 (0.740 - 1.010)	0.231 (0.100 - 0.459)	0.231 (0.100 - 0.450)	0.230 (0.101 - 0.453)
70+ years (rate per capita)	0.375 (0.326 - 0.427)	0.373 (0.325 - 0.425)	0.371 (0.324 - 0.421)	0.857 (0.732 - 0.978)	0.855 (0.731 - 0.975)	0.852 (0.731 - 0.968)	0.209 (0.095 - 0.400)	0.207 (0.093 - 0.398)	0.205 (0.093 - 0.390)
All ages (rate per capita)	0.335 (0.306 - 0.363)	0.349 (0.320 - 0.377)	0.360 (0.330 - 0.389)	0.637 (0.581 - 0.695)	0.673 (0.617 - 0.728)	0.704 (0.648 - 0.763)	0.197 (0.086 - 0.379)	0.205 (0.090 - 0.387)	0.211 (0.092 - 0.400)
Age-standardized (rate per capita)	0.355 (0.326 - 0.383)	0.355 (0.326 - 0.383)	0.355 (0.326 - 0.383)	0.697 (0.644 - 0.753)	0.697 (0.643 - 0.753)	0.697 (0.644 - 0.753)	0.208 (0.091 - 0.396)	0.208 (0.091 - 0.394)	0.208 (0.091 - 0.394)
Total number of cases (thousands)	803 (734 - 872)	938 (859 - 1,014)	1,018 (935 - 1,102)	1,528 (1,393 - 1,668)	1,808 (1,659 - 1,958)	1,991 (1,833 - 2,160)	0 (0 - 1)	1 (0 - 1)	1 (0 - 1)
**Puerto Rico**									

	Prevalence 1990	Prevalence 2005	Prevalence 2015	Incidence 1990	Incidence 2005	Incidence 2015	YLD rate 1990	YLD rate 2005	YLD rate 2015
5 -14 years (rate per capita)	0.302 (0.236 - 0.367)	0.301 (0.236 - 0.366)	0.302 (0.236 - 0.367)	0.486 (0.368 - 0.625)	0.485 (0.367 - 0.624)	0.487 (0.368 - 0.626)	0.300 (0.131 - 0.593)	0.299 (0.130 - 0.588)	0.301 (0.133 - 0.588)
15 - 49 years (rate per capita)	0.421 (0.374 - 0.467)	0.420 (0.374 - 0.466)	0.420 (0.374 - 0.465)	0.855 (0.750 - 0.958)	0.857 (0.752 - 0.960)	0.858 (0.754 - 0.962)	0.416 (0.180 - 0.796)	0.415 (0.181 - 0.791)	0.415 (0.181 - 0.787)
50 - 69 years (rate per capita)	0.400 (0.344 - 0.459)	0.400 (0.343 - 0.460)	0.400 (0.345 - 0.460)	0.917 (0.774 - 1.060)	0.916 (0.774 - 1.060)	0.917 (0.774 - 1.060)	0.385 (0.169 - 0.725)	0.384 (0.170 - 0.727)	0.384 (0.168 - 0.726)
70+ years (rate per capita)	0.377 (0.326 - 0.429)	0.372 (0.324 - 0.423)	0.372 (0.324 - 0.422)	0.893 (0.756 - 1.022)	0.887 (0.755 - 1.010)	0.886 (0.755 - 1.008)	0.348 (0.156 - 0.652)	0.343 (0.153 - 0.647)	0.341 (0.153 - 0.647)
All ages (rate per capita)	0.355 (0.326 - 0.383)	0.365 (0.336 - 0.394)	0.370 (0.341 - 0.398)	0.722 (0.663 - 0.782)	0.755 (0.695 - 0.817)	0.774 (0.713 - 0.836)	0.348 (0.153 - 0.664)	0.357 (0.157 - 0.679)	0.361 (0.159 - 0.682)
Age-standardized (rate per capita)	0.355 (0.326 - 0.382)	0.355 (0.326 - 0.382)	0.354 (0.326 - 0.382)	0.727 (0.667 - 0.788)	0.727 (0.667 - 0.789)	0.727 (0.667 - 0.789)	0.347 (0.153 - 0.661)	0.347 (0.153 - 0.660)	0.347 (0.154 - 0.659)
Total number of cases (thousands)	1,255 (1,153 - 1,353)	1,374 (1,264 - 1,481)	1,365 (1,257 - 1,468)	2,552 (2,342 - 2,765)	2,842 (2,614 - 3,075)	2,852 (2,626 - 3,081)	1 (1 - 2)	1 (1 - 3)	1 (1 - 3)
**Saint Lucia**									
5 -14 years (rate per capita)	0.292 (0.228 - 0.356)	0.303 (0.238 - 0.369)	0.301 (0.236 - 0.367)	0.473 (0.357 - 0.613)	0.489 (0.370 - 0.627)	0.486 (0.367 - 0.625)	0.288 (0.126 - 0.573)	0.302 (0.133 - 0.606)	0.300 (0.131 - 0.594)
15 - 49 years (rate per capita)	0.424 (0.373 - 0.473)	0.422 (0.373 - 0.468)	0.420 (0.374 - 0.466)	0.850 (0.740 - 0.959)	0.854 (0.747 - 0.958)	0.856 (0.751 - 0.959)	0.417 (0.179 - 0.797)	0.417 (0.179 - 0.788)	0.415 (0.180 - 0.793)
50 - 69 years (rate per capita)	0.400 (0.345 - 0.460)	0.400 (0.344 - 0.460)	0.399 (0.344 - 0.459)	0.917 (0.773 - 1.062)	0.914 (0.774 - 1.057)	0.913 (0.773 - 1.057)	0.382 (0.169 - 0.724)	0.384 (0.168 - 0.722)	0.384 (0.170 - 0.717)
70+ years (rate per capita)	0.385 (0.329 - 0.443)	0.368 (0.321 - 0.416)	0.373 (0.325 - 0.423)	0.907 (0.759 - 1.049)	0.880 (0.753 - 1.000)	0.887 (0.754 - 1.012)	0.355 (0.160 - 0.675)	0.339 (0.151 - 0.639)	0.343 (0.152 - 0.646)
All ages (rate per capita)	0.330 (0.302 - 0.359)	0.356 (0.326 - 0.386)	0.364 (0.334 - 0.393)	0.654 (0.596 - 0.715)	0.715 (0.652 - 0.777)	0.746 (0.682 - 0.807)	0.322 (0.143 - 0.611)	0.350 (0.154 - 0.668)	0.356 (0.158 - 0.679)
Age-standardized (rate per capita)	0.355 (0.326 - 0.383)	0.355 (0.326 - 0.383)	0.355 (0.326 - 0.383)	0.727 (0.667 - 0.788)	0.727 (0.667 - 0.788)	0.727 (0.667 - 0.788)	0.345 (0.152 - 0.655)	0.347 (0.152 - 0.663)	0.347 (0.154 - 0.660)
Total number of cases (thousands)	46 (42 - 50)	59 (54 - 64)	67 (62 - 73)	91 (83 - 100)	118 (108 - 129)	138 (126 - 149)	0 (0 - 0)	0 (0 - 0)	0 (0 - 0)
**Saint Vincent and the Grenadines**									
5 -14 years (rate per capita)	0.401 (0.359 - 0.429)	0.352 (0.291 - 0.412)	0.350 (0.289 - 0.411)	0.537 (0.438 - 0.647)	0.500 (0.385 - 0.623)	0.499 (0.384 - 0.621)	0.397 (0.174 - 0.769)	0.350 (0.157 - 0.683)	0.349 (0.155 - 0.692)
15 - 49 years (rate per capita)	0.494 (0.458 - 0.529)	0.478 (0.445 - 0.513)	0.478 (0.444 - 0.512)	0.861 (0.783 - 0.941)	0.870 (0.797 - 0.943)	0.871 (0.800 - 0.944)	0.489 (0.214 - 0.926)	0.473 (0.209 - 0.891)	0.472 (0.209 - 0.883)
50 - 69 years (rate per capita)	0.455 (0.414 - 0.501)	0.455 (0.412 - 0.502)	0.455 (0.410 - 0.502)	0.956 (0.856 - 1.061)	0.954 (0.855 - 1.060)	0.952 (0.851 - 1.057)	0.438 (0.189 - 0.839)	0.437 (0.187 - 0.830)	0.436 (0.187 - 0.827)
70+ years (rate per capita)	0.433 (0.396 - 0.470)	0.431 (0.396 - 0.468)	0.430 (0.394 - 0.465)	0.935 (0.833 - 1.033)	0.930 (0.835 - 1.031)	0.928 (0.833 - 1.027)	0.401 (0.181 - 0.764)	0.397 (0.177 - 0.748)	0.395 (0.176 - 0.746)
All ages (rate per capita)	0.403 (0.382 - 0.423)	0.405 (0.382 - 0.428)	0.412 (0.389 - 0.433)	0.684 (0.635 - 0.733)	0.731 (0.681 - 0.781)	0.756 (0.706 - 0.806)	0.396 (0.174 - 0.751)	0.398 (0.176 - 0.755)	0.403 (0.178 - 0.764)
Age-standardized (rate per capita)	0.419 (0.398 - 0.437)	0.405 (0.383 - 0.427)	0.405 (0.383 - 0.427)	0.755 (0.709 - 0.798)	0.747 (0.700 - 0.796)	0.747 (0.700 - 0.796)	0.410 (0.181 - 0.778)	0.396 (0.175 - 0.750)	0.396 (0.175 - 0.750)
Total number of cases (thousands)	43 (41 - 45)	44 (42 - 47)	45 (43 - 48)	74 (68 - 79)	80 (74 - 85)	83 (77 - 89)	0 (0 - 0)	0 (0 - 0)	0 (0 - 0)
**Suriname**									
5 -14 years (rate per capita)	0.241 (0.191 - 0.299)	0.231 (0.186 - 0.290)	0.251 (0.199 - 0.313)	0.397 (0.304 - 0.528)	0.390 (0.296 - 0.511)	0.413 (0.316 - 0.540)	0.143 (0.060 - 0.279)	0.138 (0.057 - 0.276)	0.150 (0.063 - 0.294)
15 - 49 years (rate per capita)	0.365 (0.331 - 0.398)	0.363 (0.332 - 0.396)	0.363 (0.331 - 0.394)	0.772 (0.696 - 0.852)	0.777 (0.703 - 0.850)	0.777 (0.704 - 0.849)	0.216 (0.095 - 0.424)	0.215 (0.092 - 0.429)	0.215 (0.095 - 0.432)
50 - 69 years (rate per capita)	0.341 (0.300 - 0.387)	0.341 (0.304 - 0.385)	0.341 (0.300 - 0.388)	0.836 (0.738 - 0.945)	0.836 (0.741 - 0.935)	0.834 (0.736 - 0.944)	0.197 (0.084 - 0.386)	0.197 (0.084 - 0.384)	0.197 (0.084 - 0.385)
70+ years (rate per capita)	0.321 (0.290 - 0.356)	0.326 (0.291 - 0.363)	0.323 (0.291 - 0.358)	0.806 (0.715 - 0.898)	0.817 (0.722 - 0.915)	0.809 (0.715 - 0.902)	0.179 (0.080 - 0.348)	0.181 (0.081 - 0.347)	0.179 (0.080 - 0.351)
All ages (rate per capita)	0.288 (0.264 - 0.309)	0.297 (0.277 - 0.317)	0.305 (0.283 - 0.326)	0.602 (0.555 - 0.651)	0.632 (0.589 - 0.678)	0.654 (0.608 - 0.700)	0.170 (0.074 - 0.325)	0.175 (0.075 - 0.340)	0.180 (0.079 - 0.346)
Age-standardized (rate per capita)	0.304 (0.283 - 0.324)	0.301 (0.282 - 0.321)	0.304 (0.283 - 0.324)	0.657 (0.612 - 0.702)	0.655 (0.612 - 0.698)	0.657 (0.612 - 0.702)	0.179 (0.078 - 0.342)	0.177 (0.076 - 0.342)	0.179 (0.078 - 0.343)
Total number of cases (thousands)	117 (108 - 126)	146 (136 - 156)	166 (154 - 177)	246 (227 - 266)	310 (289 - 333)	355 (330 - 380)	0 (0 - 0)	0 (0 - 0)	0 (0 - 0)
**Trinidad and Tobago**									
5 -14 years (rate per capita)	0.286 (0.224 - 0.351)	0.308 (0.241 - 0.375)	0.293 (0.229 - 0.357)	0.465 (0.352 - 0.604)	0.494 (0.375 - 0.633)	0.474 (0.358 - 0.615)	0.285 (0.124 - 0.565)	0.306 (0.133 - 0.603)	0.291 (0.127 - 0.580)
15 - 49 years (rate per capita)	0.421 (0.372 - 0.469)	0.421 (0.373 - 0.466)	0.418 (0.372 - 0.464)	0.855 (0.747 - 0.960)	0.855 (0.748 - 0.960)	0.861 (0.757 - 0.967)	0.416 (0.180 - 0.795)	0.416 (0.181 - 0.794)	0.413 (0.179 - 0.781)
50 - 69 years (rate per capita)	0.400 (0.344 - 0.461)	0.400 (0.344 - 0.459)	0.400 (0.343 - 0.460)	0.915 (0.774 - 1.058)	0.913 (0.773 - 1.059)	0.914 (0.773 - 1.059)	0.383 (0.169 - 0.722)	0.383 (0.168 - 0.719)	0.383 (0.169 - 0.720)
70+ years (rate per capita)	0.382 (0.329 - 0.437)	0.379 (0.328 - 0.432)	0.381 (0.328 - 0.435)	0.902 (0.760 - 1.038)	0.897 (0.759 - 1.029)	0.901 (0.760 - 1.034)	0.353 (0.158 - 0.671)	0.349 (0.156 - 0.660)	0.351 (0.159 - 0.664)
All ages (rate per capita)	0.340 (0.310 - 0.369)	0.369 (0.338 - 0.399)	0.365 (0.334 - 0.394)	0.679 (0.619 - 0.738)	0.752 (0.684 - 0.816)	0.760 (0.698 - 0.822)	0.334 (0.146 - 0.636)	0.361 (0.158 - 0.692)	0.357 (0.156 - 0.676)
Age-standardized (rate per capita)	0.355 (0.326 - 0.383)	0.355 (0.326 - 0.383)	0.355 (0.326 - 0.383)	0.727 (0.667 - 0.788)	0.727 (0.667 - 0.788)	0.727 (0.667 - 0.788)	0.347 (0.152 - 0.662)	0.347 (0.152 - 0.663)	0.347 (0.153 - 0.658)
Total number of cases (thousands)	417 (380 - 452)	479 (439 - 518)	497 (455 - 536)	833 (760 - 906)	976 (889 - 1,060)	1,035 (950 - 1,119)	0 (0 - 1)	0 (0 - 1)	0 (0 - 1)
**Virgin Islands, U.S.**									
5 -14 years (rate per capita)	0.297 (0.233 - 0.363)	0.304 (0.238 - 0.370)	0.295 (0.231 - 0.359)	0.463 (0.352 - 0.592)	0.472 (0.361 - 0.600)	0.460 (0.350 - 0.589)	0.178 (0.074 - 0.349)	0.182 (0.077 - 0.364)	0.176 (0.074 - 0.346)
15 - 49 years (rate per capita)	0.421 (0.375 - 0.466)	0.420 (0.372 - 0.465)	0.420 (0.373 - 0.466)	0.819 (0.722 - 0.918)	0.821 (0.725 - 0.920)	0.820 (0.722 - 0.919)	0.249 (0.107 - 0.490)	0.249 (0.107 - 0.491)	0.249 (0.107 - 0.494)
50 - 69 years (rate per capita)	0.400 (0.343 - 0.459)	0.400 (0.343 - 0.459)	0.401 (0.345 - 0.459)	0.875 (0.745 - 1.008)	0.879 (0.739 - 1.013)	0.881 (0.744 - 1.014)	0.231 (0.100 - 0.451)	0.231 (0.100 - 0.454)	0.231 (0.101 - 0.452)
70+ years (rate per capita)	0.388 (0.330 - 0.447)	0.378 (0.326 - 0.430)	0.381 (0.327 - 0.436)	0.875 (0.734 - 1.019)	0.862 (0.732 - 0.988)	0.866 (0.732 - 0.999)	0.216 (0.097 - 0.418)	0.209 (0.095 - 0.404)	0.210 (0.096 - 0.404)
All ages (rate per capita)	0.345 (0.314 - 0.373)	0.364 (0.335 - 0.393)	0.364 (0.336 - 0.391)	0.667 (0.612 - 0.721)	0.724 (0.668 - 0.782)	0.735 (0.677 - 0.793)	0.203 (0.089 - 0.384)	0.214 (0.093 - 0.405)	0.212 (0.094 - 0.403)
Age-standardized (rate per capita)	0.355 (0.326 - 0.383)	0.355 (0.326 - 0.383)	0.355 (0.326 - 0.382)	0.697 (0.644 - 0.753)	0.697 (0.643 - 0.753)	0.697 (0.643 - 0.753)	0.208 (0.091 - 0.394)	0.208 (0.091 - 0.395)	0.208 (0.091 - 0.393)
Total number of cases (thousands)	37 (33 - 40)	39 (36 - 42)	39 (36 - 42)	71 (65 - 76)	78 (72 - 85)	78 (72 - 85)	0 (0 - 0)	0 (0 - 0)	0 (0 - 0)
**Tropical Latin America**									
5 -14 years (rate per capita)	0.357 (0.306 - 0.414)	0.355 (0.304 - 0.412)	0.365 (0.311 - 0.422)	0.609 (0.502 - 0.722)	0.616 (0.505 - 0.736)	0.627 (0.515 - 0.746)	0.213 (0.093 - 0.412)	0.212 (0.090 - 0.409)	0.218 (0.095 - 0.424)
15 - 49 years (rate per capita)	0.459 (0.427 - 0.492)	0.461 (0.431 - 0.491)	0.459 (0.429 - 0.491)	0.941 (0.877 - 1.005)	0.951 (0.891 - 1.015)	0.955 (0.892 - 1.014)	0.272 (0.120 - 0.543)	0.273 (0.119 - 0.546)	0.272 (0.120 - 0.542)
50 - 69 years (rate per capita)	0.434 (0.397 - 0.472)	0.436 (0.400 - 0.473)	0.436 (0.401 - 0.474)	1.037 (0.947 - 1.122)	1.042 (0.954 - 1.121)	1.044 (0.961 - 1.128)	0.251 (0.108 - 0.488)	0.252 (0.109 - 0.485)	0.252 (0.109 - 0.491)
70+ years (rate per capita)	0.416 (0.385 - 0.452)	0.412 (0.382 - 0.446)	0.409 (0.381 - 0.442)	1.054 (0.967 - 1.126)	1.053 (0.973 - 1.122)	1.050 (0.972 - 1.113)	0.231 (0.104 - 0.445)	0.228 (0.103 - 0.431)	0.226 (0.102 - 0.432)
All ages (rate per capita)	0.376 (0.355 - 0.399)	0.393 (0.372 - 0.414)	0.404 (0.384 - 0.424)	0.762 (0.720 - 0.803)	0.816 (0.776 - 0.857)	0.853 (0.814 - 0.893)	0.222 (0.097 - 0.425)	0.232 (0.101 - 0.446)	0.238 (0.104 - 0.461)
Age-standardized (rate per capita)	0.392 (0.373 - 0.412)	0.393 (0.373 - 0.412)	0.392 (0.373 - 0.412)	0.827 (0.788 - 0.866)	0.832 (0.793 - 0.870)	0.832 (0.795 - 0.870)	0.230 (0.100 - 0.443)	0.231 (0.100 - 0.445)	0.231 (0.101 - 0.447)
Total number of cases (thousands)	58,173 (54,892 - 61,723)	76,360 (72,302 - 80,387)	86,609 (82,302 - 91,036)	117,887 (111,398 - 124,335)	158,533 (150,597 - 166,488)	182,943 (174,521 - 191,582)	34 (15 - 66)	45 (20 - 87)	51 (22 - 99)
**Brazil**									
5 -14 years (rate per capita)	0.357 (0.306 - 0.414)	0.354 (0.303 - 0.411)	0.364 (0.310 - 0.421)	0.610 (0.503 - 0.723)	0.617 (0.507 - 0.737)	0.629 (0.517 - 0.748)	0.213 (0.093 - 0.411)	0.212 (0.090 - 0.408)	0.218 (0.094 - 0.424)
15 - 49 years (rate per capita)	0.458 (0.427 - 0.492)	0.460 (0.430 - 0.490)	0.458 (0.429 - 0.490)	0.941 (0.877 - 1.006)	0.951 (0.891 - 1.015)	0.956 (0.893 - 1.015)	0.272 (0.120 - 0.543)	0.273 (0.119 - 0.545)	0.272 (0.120 - 0.541)
50 - 69 years (rate per capita)	0.434 (0.396 - 0.472)	0.436 (0.400 - 0.473)	0.436 (0.401 - 0.474)	1.037 (0.947 - 1.121)	1.042 (0.954 - 1.121)	1.044 (0.961 - 1.129)	0.251 (0.107 - 0.488)	0.252 (0.109 - 0.485)	0.252 (0.109 - 0.491)
70+ years (rate per capita)	0.416 (0.385 - 0.452)	0.412 (0.382 - 0.445)	0.409 (0.381 - 0.441)	1.054 (0.967 - 1.125)	1.054 (0.974 - 1.122)	1.050 (0.972 - 1.113)	0.230 (0.104 - 0.445)	0.228 (0.103 - 0.430)	0.226 (0.102 - 0.432)
All ages (rate per capita)	0.376 (0.355 - 0.399)	0.393 (0.373 - 0.414)	0.404 (0.384 - 0.424)	0.763 (0.721 - 0.805)	0.818 (0.778 - 0.859)	0.855 (0.816 - 0.895)	0.222 (0.097 - 0.425)	0.232 (0.101 - 0.447)	0.238 (0.104 - 0.461)
Age-standardized (rate per capita)	0.391 (0.372 - 0.411)	0.393 (0.373 - 0.411)	0.392 (0.373 - 0.412)	0.828 (0.788 - 0.866)	0.832 (0.793 - 0.870)	0.832 (0.795 - 0.870)	0.230 (0.100 - 0.442)	0.231 (0.100 - 0.444)	0.230 (0.101 - 0.446)
Total number of cases (thousands)	56,629 (53,416 - 60,081)	74,105 (70,164 - 77,972)	83,970 (79,790 - 88,210)	114,910 (108,596 - 121,196)	154,156 (146,487 - 161,818)	177,722 (169,552 - 186,016)	33 (15 - 64)	44 (19 - 84)	49 (22 - 96)
**Paraguay**									
5 -14 years (rate per capita)	0.362 (0.306 - 0.422)	0.372 (0.314 - 0.432)	0.373 (0.315 - 0.433)	0.576 (0.460 - 0.693)	0.586 (0.472 - 0.704)	0.588 (0.473 - 0.705)	0.216 (0.094 - 0.424)	0.223 (0.094 - 0.430)	0.223 (0.095 - 0.440)
15 - 49 years (rate per capita)	0.472 (0.440 - 0.509)	0.473 (0.441 - 0.509)	0.471 (0.439 - 0.507)	0.928 (0.858 - 0.999)	0.928 (0.859 - 0.999)	0.932 (0.862 - 1.002)	0.280 (0.124 - 0.551)	0.281 (0.122 - 0.549)	0.279 (0.123 - 0.549)
50 - 69 years (rate per capita)	0.442 (0.402 - 0.482)	0.441 (0.402 - 0.482)	0.441 (0.402 - 0.481)	1.040 (0.942 - 1.131)	1.038 (0.940 - 1.132)	1.040 (0.942 - 1.132)	0.255 (0.110 - 0.499)	0.255 (0.110 - 0.492)	0.254 (0.109 - 0.494)
70+ years (rate per capita)	0.422 (0.387 - 0.458)	0.417 (0.384 - 0.450)	0.416 (0.383 - 0.450)	1.047 (0.956 - 1.125)	1.041 (0.952 - 1.117)	1.040 (0.953 - 1.116)	0.235 (0.104 - 0.455)	0.230 (0.104 - 0.442)	0.230 (0.102 - 0.438)
All ages (rate per capita)	0.366 (0.343 - 0.390)	0.388 (0.365 - 0.412)	0.397 (0.375 - 0.420)	0.704 (0.659 - 0.749)	0.753 (0.706 - 0.798)	0.785 (0.737 - 0.829)	0.216 (0.095 - 0.414)	0.229 (0.099 - 0.441)	0.234 (0.102 - 0.456)
Age-standardized (rate per capita)	0.401 (0.381 - 0.422)	0.401 (0.381 - 0.422)	0.401 (0.381 - 0.422)	0.819 (0.774 - 0.861)	0.819 (0.774 - 0.861)	0.819 (0.774 - 0.861)	0.236 (0.103 - 0.457)	0.236 (0.102 - 0.453)	0.236 (0.103 - 0.458)
Total number of cases (thousands)	1,545 (1,450 - 1,648)	2,255 (2,122 - 2,396)	2,639 (2,493 - 2,791)	2,977 (2,786 - 3,165)	4,376 (4,103 - 4,639)	5,221 (4,905 - 5,517)	1 (0 - 2)	1 (1 - 3)	2 (1 - 3)

	Prevalence 1990	Prevalence 2005	Prevalence 2015	Incidence 1990	Incidence 2005	Incidence 2015	YLD rate 1990	YLD rate 2005	YLD rate 2015
**Southeast Asia, East Asia, and Oceania**									
5 -14 years (rate per capita)	0.244 (0.196 - 0.303)	0.229 (0.185 - 0.283)	0.247 (0.198 - 0.305)	0.333 (0.252 - 0.445)	0.319 (0.243 - 0.427)	0.331 (0.251 - 0.446)	0.156 (0.067 - 0.303)	0.150 (0.065 - 0.287)	0.163 (0.071 - 0.310)
15 - 49 years (rate per capita)	0.370 (0.340 - 0.401)	0.364 (0.335 - 0.393)	0.366 (0.336 - 0.395)	0.721 (0.654 - 0.792)	0.712 (0.648 - 0.774)	0.724 (0.657 - 0.788)	0.230 (0.100 - 0.446)	0.227 (0.099 - 0.447)	0.230 (0.100 - 0.450)
50 - 69 years (rate per capita)	0.329 (0.294 - 0.367)	0.334 (0.299 - 0.374)	0.332 (0.295 - 0.372)	0.733 (0.649 - 0.822)	0.725 (0.642 - 0.816)	0.734 (0.653 - 0.821)	0.198 (0.085 - 0.390)	0.202 (0.086 - 0.390)	0.201 (0.086 - 0.392)
70+ years (rate per capita)	0.317 (0.285 - 0.351)	0.316 (0.286 - 0.349)	0.308 (0.278 - 0.339)	0.703 (0.625 - 0.786)	0.689 (0.612 - 0.772)	0.684 (0.609 - 0.759)	0.184 (0.083 - 0.354)	0.183 (0.082 - 0.348)	0.179 (0.081 - 0.343)
All ages (rate per capita)	0.297 (0.276 - 0.317)	0.310 (0.291 - 0.329)	0.315 (0.295 - 0.334)	0.565 (0.523 - 0.608)	0.600 (0.559 - 0.638)	0.621 (0.581 - 0.663)	0.184 (0.080 - 0.353)	0.193 (0.084 - 0.372)	0.197 (0.086 - 0.378)
Age-standardized (rate per capita)	0.302 (0.283 - 0.320)	0.298 (0.279 - 0.315)	0.305 (0.286 - 0.323)	0.590 (0.551 - 0.630)	0.580 (0.543 - 0.620)	0.589 (0.552 - 0.629)	0.186 (0.081 - 0.355)	0.185 (0.081 - 0.355)	0.191 (0.083 - 0.366)
Total number of cases (thousands)	494,127 (459,153 - 527,670)	603,847 (566,396 - 641,093)	659,563 (617,750 - 700,505)	939,051 (869,938 - 1,011,230)	1,169,251 (1,089,764 - 1,243,352)	1,301,918 (1,217,855 - 1,388,935)	306 (133 - 586)	376 (164 - 726)	412 (179 - 791)
**East Asia**									
5 -14 years (rate per capita)	0.205 (0.161 - 0.262)	0.169 (0.130 - 0.219)	0.189 (0.145 - 0.243)	0.331 (0.245 - 0.451)	0.306 (0.222 - 0.430)	0.321 (0.233 - 0.449)	0.122 (0.051 - 0.243)	0.101 (0.042 - 0.200)	0.113 (0.047 - 0.221)
15 - 49 years (rate per capita)	0.340 (0.313 - 0.370)	0.329 (0.302 - 0.356)	0.329 (0.302 - 0.357)	0.712 (0.646 - 0.781)	0.698 (0.633 - 0.758)	0.711 (0.643 - 0.778)	0.202 (0.088 - 0.395)	0.196 (0.087 - 0.387)	0.195 (0.085 - 0.384)
50 - 69 years (rate per capita)	0.308 (0.275 - 0.343)	0.314 (0.280 - 0.351)	0.310 (0.274 - 0.348)	0.710 (0.630 - 0.797)	0.702 (0.623 - 0.793)	0.711 (0.630 - 0.794)	0.179 (0.076 - 0.350)	0.183 (0.078 - 0.353)	0.180 (0.077 - 0.353)
70+ years (rate per capita)	0.294 (0.265 - 0.328)	0.294 (0.266 - 0.325)	0.284 (0.257 - 0.314)	0.686 (0.608 - 0.771)	0.671 (0.598 - 0.752)	0.664 (0.592 - 0.738)	0.166 (0.073 - 0.322)	0.166 (0.073 - 0.316)	0.160 (0.072 - 0.307)
All ages (rate per capita)	0.274 (0.255 - 0.293)	0.283 (0.265 - 0.301)	0.286 (0.268 - 0.304)	0.567 (0.525 - 0.613)	0.601 (0.559 - 0.642)	0.622 (0.580 - 0.665)	0.162 (0.070 - 0.311)	0.167 (0.073 - 0.322)	0.169 (0.074 - 0.326)
Age-standardized (rate per capita)	0.275 (0.258 - 0.292)	0.265 (0.248 - 0.281)	0.270 (0.252 - 0.288)	0.579 (0.539 - 0.621)	0.564 (0.526 - 0.607)	0.576 (0.537 - 0.618)	0.162 (0.070 - 0.308)	0.156 (0.069 - 0.301)	0.160 (0.070 - 0.304)
Total number of cases (thousands)	327,437 (304,224 - 350,133)	384,562 (359,922 - 408,748)	410,270 (383,376 - 436,097)	677,479 (626,823 - 732,291)	816,451 (759,473 - 872,328)	891,298 (831,649 - 953,269)	194 (84 - 371)	227 (100 - 438)	242 (106 - 467)
**China**									
5 -14 years (rate per capita)	0.206 (0.162 - 0.263)	0.168 (0.129 - 0.218)	0.188 (0.145 - 0.243)	0.332 (0.246 - 0.452)	0.306 (0.222 - 0.430)	0.321 (0.233 - 0.451)	0.123 (0.051 - 0.244)	0.101 (0.041 - 0.200)	0.113 (0.047 - 0.221)
15 - 49 years (rate per capita)	0.341 (0.314 - 0.371)	0.330 (0.303 - 0.357)	0.329 (0.302 - 0.357)	0.713 (0.647 - 0.783)	0.698 (0.634 - 0.759)	0.712 (0.644 - 0.779)	0.203 (0.089 - 0.395)	0.196 (0.087 - 0.387)	0.196 (0.085 - 0.385)
50 - 69 years (rate per capita)	0.308 (0.276 - 0.343)	0.315 (0.280 - 0.352)	0.310 (0.275 - 0.349)	0.711 (0.630 - 0.798)	0.702 (0.623 - 0.793)	0.712 (0.631 - 0.795)	0.179 (0.076 - 0.351)	0.184 (0.078 - 0.354)	0.181 (0.077 - 0.354)
70+ years (rate per capita)	0.295 (0.265 - 0.328)	0.295 (0.266 - 0.326)	0.285 (0.257 - 0.314)	0.686 (0.608 - 0.772)	0.671 (0.597 - 0.751)	0.665 (0.592 - 0.739)	0.166 (0.073 - 0.323)	0.166 (0.074 - 0.316)	0.161 (0.072 - 0.307)
All ages (rate per capita)	0.275 (0.255 - 0.294)	0.283 (0.265 - 0.301)	0.287 (0.268 - 0.305)	0.567 (0.525 - 0.614)	0.601 (0.559 - 0.642)	0.623 (0.581 - 0.666)	0.162 (0.070 - 0.311)	0.168 (0.074 - 0.323)	0.169 (0.074 - 0.327)
Age-standardized (rate per capita)	0.276 (0.258 - 0.293)	0.265 (0.249 - 0.282)	0.271 (0.252 - 0.288)	0.580 (0.540 - 0.622)	0.564 (0.526 - 0.608)	0.577 (0.538 - 0.619)	0.163 (0.070 - 0.309)	0.157 (0.069 - 0.301)	0.160 (0.070 - 0.305)
Total number of cases (thousands)	316,951 (294,599 - 338,989)	372,184 (348,277 - 395,779)	396,839 (370,914 - 421,686)	654,876 (605,780 - 708,528)	789,210 (734,158 - 843,289)	861,424 (803,666 - 921,288)	188 (81 - 359)	220 (97 - 424)	234 (102 - 452)
**North Korea**									
5 -14 years (rate per capita)	0.174 (0.131 - 0.228)	0.184 (0.139 - 0.240)	0.187 (0.141 - 0.244)	0.289 (0.210 - 0.408)	0.303 (0.220 - 0.422)	0.307 (0.223 - 0.425)	0.104 (0.043 - 0.204)	0.110 (0.046 - 0.220)	0.112 (0.047 - 0.221)
15 - 49 years (rate per capita)	0.319 (0.290 - 0.350)	0.312 (0.285 - 0.341)	0.312 (0.285 - 0.341)	0.690 (0.623 - 0.762)	0.688 (0.626 - 0.754)	0.690 (0.627 - 0.756)	0.190 (0.082 - 0.373)	0.185 (0.081 - 0.370)	0.186 (0.081 - 0.365)
50 - 69 years (rate per capita)	0.289 (0.255 - 0.329)	0.293 (0.258 - 0.329)	0.289 (0.255 - 0.327)	0.696 (0.609 - 0.791)	0.710 (0.621 - 0.801)	0.698 (0.613 - 0.789)	0.168 (0.071 - 0.327)	0.170 (0.072 - 0.331)	0.168 (0.071 - 0.329)
70+ years (rate per capita)	0.281 (0.248 - 0.317)	0.283 (0.250 - 0.320)	0.281 (0.248 - 0.317)	0.678 (0.594 - 0.771)	0.682 (0.598 - 0.776)	0.678 (0.593 - 0.769)	0.158 (0.071 - 0.307)	0.159 (0.071 - 0.308)	0.158 (0.070 - 0.306)
All ages (rate per capita)	0.258 (0.238 - 0.277)	0.262 (0.243 - 0.280)	0.267 (0.247 - 0.285)	0.556 (0.512 - 0.604)	0.573 (0.531 - 0.620)	0.588 (0.547 - 0.633)	0.153 (0.066 - 0.296)	0.155 (0.066 - 0.294)	0.157 (0.068 - 0.300)
Age-standardized (rate per capita)	0.257 (0.239 - 0.274)	0.256 (0.238 - 0.274)	0.256 (0.238 - 0.274)	0.562 (0.522 - 0.604)	0.561 (0.522 - 0.604)	0.561 (0.521 - 0.603)	0.151 (0.065 - 0.290)	0.151 (0.065 - 0.289)	0.151 (0.065 - 0.289)
Total number of cases (thousands)	5,217 (4,811 - 5,599)	6,229 (5,779 - 6,656)	6,707 (6,215 - 7,169)	11,216 (10,332 - 12,188)	13,619 (12,618 - 14,729)	14,804 (13,750 - 15,929)	3 (1 - 6)	4 (2 - 7)	4 (2 - 8)
**Taiwan**									
5 -14 years (rate per capita)	0.185 (0.139 - 0.241)	0.186 (0.140 - 0.242)	0.203 (0.153 - 0.264)	0.304 (0.221 - 0.422)	0.305 (0.222 - 0.423)	0.330 (0.238 - 0.443)	0.110 (0.046 - 0.218)	0.111 (0.046 - 0.222)	0.122 (0.050 - 0.246)
15 - 49 years (rate per capita)	0.315 (0.286 - 0.345)	0.311 (0.283 - 0.340)	0.311 (0.283 - 0.339)	0.692 (0.624 - 0.760)	0.691 (0.629 - 0.757)	0.691 (0.627 - 0.757)	0.187 (0.082 - 0.369)	0.185 (0.079 - 0.365)	0.185 (0.080 - 0.367)
50 - 69 years (rate per capita)	0.289 (0.255 - 0.326)	0.289 (0.254 - 0.327)	0.289 (0.254 - 0.328)	0.700 (0.615 - 0.789)	0.695 (0.612 - 0.787)	0.696 (0.609 - 0.790)	0.167 (0.071 - 0.325)	0.167 (0.071 - 0.329)	0.167 (0.072 - 0.325)
70+ years (rate per capita)	0.276 (0.244 - 0.311)	0.268 (0.239 - 0.300)	0.260 (0.234 - 0.289)	0.668 (0.585 - 0.758)	0.649 (0.567 - 0.735)	0.632 (0.560 - 0.710)	0.155 (0.068 - 0.300)	0.150 (0.067 - 0.292)	0.145 (0.065 - 0.277)
All ages (rate per capita)	0.260 (0.241 - 0.279)	0.271 (0.252 - 0.289)	0.277 (0.258 - 0.296)	0.562 (0.520 - 0.611)	0.599 (0.556 - 0.644)	0.622 (0.579 - 0.664)	0.153 (0.066 - 0.292)	0.160 (0.069 - 0.304)	0.163 (0.070 - 0.311)
Age-standardized (rate per capita)	0.255 (0.237 - 0.273)	0.256 (0.238 - 0.273)	0.256 (0.238 - 0.273)	0.559 (0.520 - 0.601)	0.560 (0.520 - 0.602)	0.560 (0.520 - 0.603)	0.150 (0.065 - 0.285)	0.151 (0.065 - 0.287)	0.151 (0.064 - 0.288)
Total number of cases (thousands)	5,269 (4,872 - 5,658)	6,150 (5,728 - 6,571)	6,723 (6,263 - 7,181)	11,387 (10,525 - 12,381)	13,621 (12,641 - 14,632)	15,071 (14,029 - 16,088)	3 (1 - 6)	4 (2 - 7)	4 (2 - 8)
**Southeast Asia**									
5 -14 years (rate per capita)	0.316 (0.260 - 0.383)	0.326 (0.273 - 0.387)	0.326 (0.270 - 0.392)	0.335 (0.256 - 0.439)	0.340 (0.266 - 0.429)	0.346 (0.268 - 0.446)	0.218 (0.096 - 0.414)	0.231 (0.102 - 0.435)	0.233 (0.103 - 0.434)
15 - 49 years (rate per capita)	0.452 (0.413 - 0.492)	0.447 (0.411 - 0.484)	0.447 (0.411 - 0.482)	0.746 (0.672 - 0.818)	0.749 (0.681 - 0.816)	0.753 (0.688 - 0.820)	0.306 (0.133 - 0.594)	0.304 (0.133 - 0.585)	0.306 (0.134 - 0.588)
50 - 69 years (rate per capita)	0.397 (0.349 - 0.447)	0.398 (0.350 - 0.450)	0.399 (0.352 - 0.452)	0.803 (0.705 - 0.905)	0.803 (0.704 - 0.904)	0.805 (0.705 - 0.907)	0.258 (0.113 - 0.495)	0.262 (0.115 - 0.503)	0.263 (0.116 - 0.507)
70+ years (rate per capita)	0.394 (0.351 - 0.438)	0.390 (0.348 - 0.434)	0.390 (0.348 - 0.432)	0.759 (0.663 - 0.852)	0.753 (0.657 - 0.850)	0.753 (0.658 - 0.849)	0.246 (0.111 - 0.471)	0.241 (0.109 - 0.461)	0.245 (0.110 - 0.467)
All ages (rate per capita)	0.355 (0.330 - 0.380)	0.371 (0.347 - 0.394)	0.375 (0.351 - 0.398)	0.559 (0.517 - 0.604)	0.599 (0.557 - 0.640)	0.621 (0.581 - 0.662)	0.240 (0.107 - 0.460)	0.252 (0.112 - 0.481)	0.256 (0.115 - 0.488)
Age-standardized (rate per capita)	0.370 (0.346 - 0.392)	0.371 (0.347 - 0.393)	0.373 (0.350 - 0.395)	0.622 (0.582 - 0.664)	0.621 (0.581 - 0.659)	0.624 (0.585 - 0.662)	0.248 (0.110 - 0.473)	0.250 (0.112 - 0.477)	0.254 (0.114 - 0.485)
Total number of cases (thousands)	163,829 (152,297 - 175,174)	215,288 (201,159 - 228,829)	244,354 (228,605 - 259,278)	258,137 (238,561 - 278,781)	347,880 (323,057 - 371,374)	404,481 (378,431 - 430,788)	111 (49 - 212)	146 (65 - 279)	167 (75 - 318)
**Cambodia**									
5 -14 years (rate per capita)	0.278 (0.221 - 0.352)	0.343 (0.270 - 0.415)	0.314 (0.251 - 0.393)	0.284 (0.210 - 0.408)	0.326 (0.249 - 0.423)	0.309 (0.234 - 0.421)	0.165 (0.070 - 0.326)	0.204 (0.087 - 0.396)	0.187 (0.080 - 0.360)
15 - 49 years (rate per capita)	0.463 (0.425 - 0.502)	0.465 (0.426 - 0.504)	0.458 (0.417 - 0.498)	0.724 (0.657 - 0.799)	0.719 (0.654 - 0.791)	0.730 (0.662 - 0.807)	0.273 (0.120 - 0.541)	0.276 (0.121 - 0.545)	0.272 (0.116 - 0.541)
50 - 69 years (rate per capita)	0.405 (0.362 - 0.455)	0.407 (0.360 - 0.459)	0.407 (0.362 - 0.457)	0.798 (0.710 - 0.893)	0.797 (0.704 - 0.896)	0.799 (0.708 - 0.897)	0.233 (0.101 - 0.458)	0.235 (0.101 - 0.459)	0.235 (0.100 - 0.465)
70+ years (rate per capita)	0.420 (0.370 - 0.469)	0.417 (0.372 - 0.466)	0.412 (0.366 - 0.459)	0.762 (0.669 - 0.865)	0.760 (0.664 - 0.855)	0.752 (0.662 - 0.850)	0.232 (0.103 - 0.447)	0.231 (0.104 - 0.448)	0.229 (0.102 - 0.436)
All ages (rate per capita)	0.322 (0.297 - 0.346)	0.372 (0.343 - 0.400)	0.369 (0.341 - 0.394)	0.481 (0.443 - 0.525)	0.542 (0.498 - 0.586)	0.570 (0.528 - 0.615)	0.190 (0.083 - 0.367)	0.220 (0.095 - 0.422)	0.218 (0.095 - 0.424)
Age-standardized (rate per capita)	0.378 (0.352 - 0.400)	0.379 (0.354 - 0.402)	0.377 (0.352 - 0.400)	0.607 (0.567 - 0.648)	0.608 (0.566 - 0.648)	0.607 (0.567 - 0.648)	0.221 (0.096 - 0.426)	0.223 (0.097 - 0.428)	0.222 (0.096 - 0.430)
Total number of cases (thousands)	2,902 (2,673 - 3,117)	4,978 (4,588 - 5,354)	5,759 (5,311 - 6,139)	4,337 (3,993 - 4,731)	7,245 (6,661 - 7,841)	8,884 (8,227 - 9,585)	2 (1 - 3)	3 (1 - 6)	3 (1 - 7)
**Indonesia**									
5 -14 years (rate per capita)	0.321 (0.256 - 0.395)	0.318 (0.256 - 0.390)	0.320 (0.258 - 0.393)	0.307 (0.228 - 0.413)	0.313 (0.241 - 0.419)	0.315 (0.242 - 0.421)	0.191 (0.080 - 0.373)	0.190 (0.080 - 0.369)	0.191 (0.080 - 0.365)
15 - 49 years (rate per capita)	0.452 (0.417 - 0.491)	0.449 (0.410 - 0.486)	0.445 (0.408 - 0.481)	0.724 (0.657 - 0.800)	0.730 (0.666 - 0.798)	0.732 (0.667 - 0.800)	0.269 (0.117 - 0.538)	0.267 (0.115 - 0.529)	0.264 (0.114 - 0.524)
50 - 69 years (rate per capita)	0.397 (0.351 - 0.445)	0.398 (0.352 - 0.447)	0.396 (0.350 - 0.447)	0.794 (0.701 - 0.902)	0.793 (0.698 - 0.896)	0.791 (0.695 - 0.899)	0.229 (0.098 - 0.457)	0.230 (0.099 - 0.448)	0.229 (0.098 - 0.447)
70+ years (rate per capita)	0.404 (0.360 - 0.447)	0.402 (0.362 - 0.446)	0.401 (0.361 - 0.444)	0.751 (0.656 - 0.852)	0.748 (0.654 - 0.843)	0.747 (0.653 - 0.842)	0.224 (0.100 - 0.429)	0.223 (0.099 - 0.430)	0.222 (0.099 - 0.424)
All ages (rate per capita)	0.358 (0.331 - 0.384)	0.370 (0.344 - 0.395)	0.371 (0.345 - 0.395)	0.543 (0.501 - 0.590)	0.581 (0.538 - 0.625)	0.596 (0.552 - 0.638)	0.212 (0.092 - 0.410)	0.219 (0.094 - 0.422)	0.219 (0.094 - 0.424)
Age-standardized (rate per capita)	0.371 (0.347 - 0.393)	0.371 (0.346 - 0.394)	0.371 (0.346 - 0.394)	0.604 (0.565 - 0.647)	0.605 (0.564 - 0.645)	0.605 (0.564 - 0.645)	0.218 (0.094 - 0.424)	0.218 (0.093 - 0.419)	0.218 (0.093 - 0.421)
Total number of cases (thousands)	64,970 (60,007 - 69,662)	83,901 (78,030 - 89,482)	95,575 (88,978 - 101,769)	98,386 (90,852 - 106,947)	131,759 (122,041 - 141,619)	153,472 (142,228 - 164,238)	38 (17 - 74)	50 (21 - 96)	56 (24 - 109)
**Laos**									
5 -14 years (rate per capita)	0.271 (0.187 - 0.377)	0.283 (0.193 - 0.391)	0.275 (0.190 - 0.381)	0.379 (0.227 - 0.553)	0.393 (0.231 - 0.571)	0.384 (0.228 - 0.560)	0.162 (0.063 - 0.333)	0.169 (0.067 - 0.355)	0.165 (0.067 - 0.343)
15 - 49 years (rate per capita)	0.431 (0.358 - 0.504)	0.431 (0.359 - 0.503)	0.429 (0.357 - 0.500)	0.764 (0.614 - 0.891)	0.764 (0.616 - 0.891)	0.766 (0.620 - 0.891)	0.255 (0.107 - 0.506)	0.255 (0.107 - 0.510)	0.255 (0.106 - 0.501)
50 - 69 years (rate per capita)	0.397 (0.308 - 0.485)	0.396 (0.308 - 0.483)	0.396 (0.307 - 0.484)	0.796 (0.608 - 0.987)	0.795 (0.608 - 0.982)	0.795 (0.604 - 0.984)	0.228 (0.098 - 0.465)	0.228 (0.098 - 0.463)	0.229 (0.098 - 0.464)
70+ years (rate per capita)	0.393 (0.302 - 0.488)	0.389 (0.302 - 0.482)	0.387 (0.302 - 0.477)	0.770 (0.576 - 0.969)	0.766 (0.576 - 0.964)	0.763 (0.575 - 0.957)	0.217 (0.096 - 0.428)	0.215 (0.095 - 0.418)	0.214 (0.096 - 0.415)
All ages (rate per capita)	0.311 (0.266 - 0.356)	0.330 (0.283 - 0.378)	0.337 (0.292 - 0.384)	0.533 (0.457 - 0.609)	0.566 (0.483 - 0.645)	0.589 (0.509 - 0.667)	0.183 (0.080 - 0.356)	0.195 (0.085 - 0.378)	0.200 (0.087 - 0.389)
Age-standardized (rate per capita)	0.353 (0.310 - 0.397)	0.353 (0.310 - 0.397)	0.353 (0.310 - 0.397)	0.639 (0.562 - 0.712)	0.639 (0.562 - 0.712)	0.639 (0.562 - 0.712)	0.207 (0.091 - 0.405)	0.207 (0.091 - 0.406)	0.207 (0.092 - 0.405)

	Prevalence 1990	Prevalence 2005	Prevalence 2015	Incidence 1990	Incidence 2005	Incidence 2015	YLD rate 1990	YLD rate 2005	YLD rate 2015
Total number of cases (thousands)	1,311 (1,122 - 1,504)	1,894 (1,620 - 2,170)	2,294 (1,984 - 2,613)	2,253 (1,929 - 2,570)	3,245 (2,772 - 3,701)	4,006 (3,459 - 4,537)	1 (0 - 2)	1 (0 - 2)	1 (1 - 3)
**Malaysia**									
5 -14 years (rate per capita)	0.212 (0.161 - 0.277)	0.196 (0.158 - 0.234)	0.227 (0.172 - 0.295)	0.197 (0.144 - 0.297)	0.173 (0.133 - 0.232)	0.205 (0.152 - 0.299)	0.126 (0.051 - 0.251)	0.117 (0.050 - 0.221)	0.136 (0.057 - 0.269)
15 - 49 years (rate per capita)	0.314 (0.280 - 0.347)	0.311 (0.279 - 0.345)	0.309 (0.277 - 0.342)	0.477 (0.419 - 0.540)	0.477 (0.420 - 0.540)	0.479 (0.423 - 0.541)	0.186 (0.080 - 0.372)	0.184 (0.080 - 0.366)	0.183 (0.079 - 0.363)
50 - 69 years (rate per capita)	0.264 (0.226 - 0.308)	0.264 (0.226 - 0.310)	0.265 (0.227 - 0.308)	0.499 (0.430 - 0.584)	0.498 (0.426 - 0.585)	0.500 (0.431 - 0.585)	0.153 (0.066 - 0.301)	0.153 (0.065 - 0.296)	0.153 (0.065 - 0.304)
70+ years (rate per capita)	0.266 (0.231 - 0.304)	0.266 (0.232 - 0.303)	0.264 (0.231 - 0.302)	0.462 (0.401 - 0.528)	0.463 (0.400 - 0.534)	0.460 (0.399 - 0.525)	0.148 (0.066 - 0.283)	0.148 (0.066 - 0.285)	0.147 (0.065 - 0.288)
All ages (rate per capita)	0.243 (0.219 - 0.266)	0.251 (0.229 - 0.272)	0.262 (0.239 - 0.284)	0.349 (0.313 - 0.388)	0.372 (0.336 - 0.409)	0.398 (0.361 - 0.438)	0.143 (0.062 - 0.275)	0.148 (0.064 - 0.285)	0.155 (0.067 - 0.297)
Age-standardized (rate per capita)	0.254 (0.232 - 0.275)	0.250 (0.230 - 0.269)	0.254 (0.232 - 0.275)	0.390 (0.355 - 0.425)	0.384 (0.351 - 0.418)	0.390 (0.355 - 0.425)	0.149 (0.064 - 0.286)	0.147 (0.063 - 0.283)	0.150 (0.065 - 0.285)
Total number of cases (thousands)	4,406 (3,974 - 4,834)	6,451 (5,891 - 6,986)	7,951 (7,248 - 8,608)	6,343 (5,691 - 7,048)	9,557 (8,646 - 10,504)	12,064 (10,947 - 13,282)	3 (1 - 5)	4 (2 - 7)	5 (2 - 9)
**Maldives**									
5 -14 years (rate per capita)	0.260 (0.180 - 0.361)	0.294 (0.197 - 0.405)	0.275 (0.189 - 0.380)	0.366 (0.222 - 0.534)	0.407 (0.235 - 0.583)	0.383 (0.227 - 0.559)	0.156 (0.060 - 0.319)	0.176 (0.069 - 0.365)	0.165 (0.066 - 0.343)
15 - 49 years (rate per capita)	0.431 (0.358 - 0.506)	0.431 (0.359 - 0.504)	0.424 (0.354 - 0.493)	0.763 (0.613 - 0.892)	0.764 (0.616 - 0.889)	0.772 (0.634 - 0.902)	0.256 (0.107 - 0.508)	0.256 (0.108 - 0.506)	0.253 (0.106 - 0.502)
50 - 69 years (rate per capita)	0.394 (0.303 - 0.483)	0.398 (0.310 - 0.485)	0.394 (0.301 - 0.486)	0.793 (0.600 - 0.980)	0.797 (0.608 - 0.979)	0.793 (0.598 - 0.986)	0.227 (0.096 - 0.462)	0.230 (0.099 - 0.474)	0.229 (0.096 - 0.469)
70+ years (rate per capita)	0.395 (0.298 - 0.496)	0.390 (0.299 - 0.484)	0.382 (0.299 - 0.468)	0.775 (0.568 - 0.988)	0.768 (0.570 - 0.969)	0.756 (0.571 - 0.945)	0.219 (0.098 - 0.429)	0.217 (0.097 - 0.431)	0.212 (0.095 - 0.416)
All ages (rate per capita)	0.296 (0.253 - 0.341)	0.352 (0.300 - 0.402)	0.351 (0.304 - 0.398)	0.506 (0.433 - 0.581)	0.606 (0.519 - 0.689)	0.629 (0.545 - 0.708)	0.175 (0.076 - 0.339)	0.208 (0.091 - 0.404)	0.208 (0.090 - 0.406)
Age-standardized (rate per capita)	0.352 (0.309 - 0.396)	0.353 (0.309 - 0.396)	0.353 (0.309 - 0.397)	0.639 (0.562 - 0.712)	0.639 (0.562 - 0.712)	0.639 (0.562 - 0.712)	0.207 (0.091 - 0.407)	0.208 (0.092 - 0.406)	0.208 (0.092 - 0.410)
Total number of cases (thousands)	66 (57 - 76)	107 (92 - 123)	127 (110 - 145)	113 (97 - 130)	185 (158 - 211)	228 (198 - 257)	0 (0 - 0)	0 (0 - 0)	0 (0 - 0)
**Mauritius**									
5 -14 years (rate per capita)	0.284 (0.193 - 0.392)	0.288 (0.194 - 0.398)	0.293 (0.196 - 0.403)	0.409 (0.237 - 0.597)	0.415 (0.239 - 0.605)	0.420 (0.240 - 0.612)	0.283 (0.116 - 0.570)	0.288 (0.122 - 0.582)	0.292 (0.121 - 0.588)
15 - 49 years (rate per capita)	0.426 (0.355 - 0.495)	0.421 (0.349 - 0.489)	0.422 (0.352 - 0.490)	0.806 (0.661 - 0.937)	0.810 (0.670 - 0.945)	0.808 (0.668 - 0.941)	0.422 (0.179 - 0.834)	0.417 (0.178 - 0.821)	0.417 (0.176 - 0.815)
50 - 69 years (rate per capita)	0.396 (0.308 - 0.483)	0.395 (0.303 - 0.485)	0.396 (0.308 - 0.484)	0.830 (0.635 - 1.027)	0.828 (0.627 - 1.027)	0.830 (0.633 - 1.027)	0.383 (0.163 - 0.748)	0.381 (0.163 - 0.742)	0.381 (0.164 - 0.743)
70+ years (rate per capita)	0.390 (0.302 - 0.483)	0.380 (0.301 - 0.463)	0.378 (0.302 - 0.462)	0.800 (0.600 - 1.009)	0.785 (0.598 - 0.986)	0.783 (0.597 - 0.979)	0.362 (0.155 - 0.704)	0.351 (0.154 - 0.678)	0.349 (0.152 - 0.675)
All ages (rate per capita)	0.352 (0.305 - 0.399)	0.360 (0.315 - 0.407)	0.372 (0.325 - 0.418)	0.651 (0.562 - 0.734)	0.682 (0.595 - 0.766)	0.713 (0.623 - 0.797)	0.347 (0.151 - 0.685)	0.355 (0.156 - 0.690)	0.365 (0.160 - 0.708)
Age-standardized (rate per capita)	0.353 (0.310 - 0.397)	0.353 (0.310 - 0.397)	0.353 (0.309 - 0.397)	0.668 (0.586 - 0.748)	0.668 (0.586 - 0.748)	0.668 (0.586 - 0.748)	0.346 (0.153 - 0.676)	0.346 (0.153 - 0.676)	0.346 (0.152 - 0.672)
Total number of cases (thousands)	374 (324 - 425)	440 (384 - 497)	474 (414 - 533)	692 (597 - 780)	833 (727 - 935)	909 (794 - 1,015)	0 (0 - 1)	0 (0 - 1)	0 (0 - 1)
**Myanmar**									
5 -14 years (rate per capita)	0.226 (0.180 - 0.284)	0.220 (0.177 - 0.276)	0.234 (0.186 - 0.294)	0.262 (0.195 - 0.347)	0.261 (0.193 - 0.343)	0.272 (0.201 - 0.356)	0.134 (0.056 - 0.267)	0.132 (0.055 - 0.265)	0.140 (0.058 - 0.276)
15 - 49 years (rate per capita)	0.372 (0.335 - 0.409)	0.365 (0.327 - 0.400)	0.362 (0.328 - 0.397)	0.684 (0.614 - 0.757)	0.688 (0.617 - 0.760)	0.688 (0.620 - 0.757)	0.220 (0.095 - 0.440)	0.216 (0.093 - 0.433)	0.215 (0.093 - 0.429)
50 - 69 years (rate per capita)	0.311 (0.269 - 0.358)	0.310 (0.268 - 0.357)	0.310 (0.267 - 0.358)	0.725 (0.638 - 0.825)	0.722 (0.634 - 0.819)	0.723 (0.635 - 0.829)	0.179 (0.077 - 0.351)	0.178 (0.076 - 0.345)	0.178 (0.076 - 0.351)
70+ years (rate per capita)	0.311 (0.270 - 0.356)	0.309 (0.272 - 0.351)	0.306 (0.267 - 0.348)	0.689 (0.605 - 0.779)	0.684 (0.602 - 0.778)	0.680 (0.599 - 0.768)	0.172 (0.078 - 0.331)	0.170 (0.076 - 0.322)	0.169 (0.075 - 0.322)
All ages (rate per capita)	0.281 (0.256 - 0.305)	0.289 (0.265 - 0.313)	0.297 (0.272 - 0.320)	0.496 (0.454 - 0.542)	0.533 (0.489 - 0.578)	0.554 (0.510 - 0.597)	0.165 (0.071 - 0.316)	0.170 (0.074 - 0.334)	0.175 (0.075 - 0.339)
Age-standardized (rate per capita)	0.293 (0.271 - 0.315)	0.292 (0.269 - 0.313)	0.293 (0.271 - 0.315)	0.559 (0.518 - 0.600)	0.559 (0.518 - 0.601)	0.559 (0.518 - 0.600)	0.171 (0.074 - 0.333)	0.171 (0.074 - 0.334)	0.172 (0.074 - 0.331)
Total number of cases (thousands)	11,805 (10,773 - 12,839)	14,432 (13,255 - 15,638)	16,020 (14,717 - 17,298)	20,870 (19,090 - 22,764)	26,605 (24,437 - 28,859)	29,932 (27,570 - 32,265)	7 (3 - 13)	9 (4 - 17)	9 (4 - 18)
**Philippines**									
5 -14 years (rate per capita)	0.458 (0.400 - 0.514)	0.469 (0.427 - 0.512)	0.465 (0.406 - 0.522)	0.483 (0.393 - 0.582)	0.458 (0.394 - 0.527)	0.488 (0.398 - 0.588)	0.455 (0.204 - 0.888)	0.465 (0.209 - 0.904)	0.463 (0.208 - 0.899)
15 - 49 years (rate per capita)	0.595 (0.565 - 0.625)	0.593 (0.563 - 0.623)	0.591 (0.562 - 0.620)	0.926 (0.865 - 0.988)	0.929 (0.869 - 0.994)	0.934 (0.874 - 0.995)	0.587 (0.257 - 1.125)	0.585 (0.255 - 1.119)	0.584 (0.252 - 1.117)
50 - 69 years (rate per capita)	0.543 (0.505 - 0.578)	0.543 (0.503 - 0.580)	0.543 (0.505 - 0.578)	1.041 (0.955 - 1.114)	1.040 (0.952 - 1.114)	1.041 (0.956 - 1.115)	0.521 (0.227 - 0.973)	0.519 (0.224 - 0.984)	0.521 (0.226 - 0.984)
70+ years (rate per capita)	0.543 (0.509 - 0.579)	0.542 (0.507 - 0.578)	0.541 (0.508 - 0.577)	1.006 (0.941 - 1.069)	1.005 (0.938 - 1.072)	1.005 (0.941 - 1.067)	0.500 (0.224 - 0.938)	0.499 (0.225 - 0.952)	0.498 (0.223 - 0.935)
All ages (rate per capita)	0.465 (0.443 - 0.486)	0.479 (0.461 - 0.498)	0.491 (0.469 - 0.510)	0.682 (0.641 - 0.722)	0.706 (0.668 - 0.744)	0.752 (0.711 - 0.791)	0.457 (0.199 - 0.873)	0.471 (0.207 - 0.892)	0.483 (0.212 - 0.911)
Age-standardized (rate per capita)	0.502 (0.482 - 0.520)	0.503 (0.485 - 0.521)	0.502 (0.482 - 0.520)	0.801 (0.762 - 0.839)	0.795 (0.756 - 0.832)	0.801 (0.762 - 0.839)	0.490 (0.215 - 0.932)	0.491 (0.216 - 0.930)	0.491 (0.216 - 0.929)
Total number of cases (thousands)	28,753 (27,411 - 30,058)	41,265 (39,663 - 42,885)	49,478 (47,320 - 51,420)	42,215 (39,654 - 44,673)	60,827 (57,524 - 64,037)	75,782 (71,714 - 79,756)	28 (12 - 54)	41 (18 - 77)	49 (21 - 92)
**Sri Lanka**									
5 -14 years (rate per capita)	0.279 (0.192 - 0.386)	0.282 (0.193 - 0.390)	0.280 (0.192 - 0.387)	0.403 (0.236 - 0.588)	0.407 (0.237 - 0.594)	0.403 (0.236 - 0.588)	0.277 (0.114 - 0.558)	0.282 (0.116 - 0.563)	0.279 (0.115 - 0.554)
15 - 49 years (rate per capita)	0.426 (0.355 - 0.495)	0.424 (0.352 - 0.490)	0.422 (0.352 - 0.490)	0.806 (0.660 - 0.937)	0.808 (0.666 - 0.942)	0.808 (0.667 - 0.941)	0.421 (0.178 - 0.828)	0.419 (0.177 - 0.824)	0.418 (0.177 - 0.823)
50 - 69 years (rate per capita)	0.397 (0.309 - 0.484)	0.396 (0.307 - 0.485)	0.397 (0.309 - 0.483)	0.830 (0.632 - 1.027)	0.830 (0.630 - 1.028)	0.832 (0.633 - 1.030)	0.383 (0.163 - 0.754)	0.381 (0.163 - 0.748)	0.383 (0.165 - 0.749)
70+ years (rate per capita)	0.383 (0.301 - 0.472)	0.379 (0.300 - 0.464)	0.380 (0.300 - 0.466)	0.790 (0.595 - 0.994)	0.784 (0.596 - 0.983)	0.787 (0.598 - 0.984)	0.355 (0.159 - 0.695)	0.349 (0.153 - 0.674)	0.351 (0.154 - 0.685)
All ages (rate per capita)	0.345 (0.299 - 0.392)	0.356 (0.310 - 0.401)	0.357 (0.312 - 0.402)	0.637 (0.550 - 0.717)	0.671 (0.585 - 0.751)	0.679 (0.596 - 0.760)	0.340 (0.148 - 0.668)	0.349 (0.154 - 0.679)	0.350 (0.155 - 0.680)
Age-standardized (rate per capita)	0.353 (0.309 - 0.396)	0.353 (0.309 - 0.397)	0.353 (0.310 - 0.397)	0.668 (0.586 - 0.748)	0.668 (0.586 - 0.748)	0.668 (0.586 - 0.748)	0.345 (0.152 - 0.674)	0.346 (0.153 - 0.678)	0.346 (0.152 - 0.673)
Total number of cases (thousands)	5,985 (5,191 - 6,790)	6,949 (6,059 - 7,843)	7,414 (6,475 - 8,348)	11,050 (9,532 - 12,440)	13,109 (11,434 - 14,684)	14,095 (12,376 - 15,771)	6 (3 - 12)	7 (3 - 13)	7 (3 - 14)
**Seychelles**									
5 -14 years (rate per capita)	0.279 (0.192 - 0.387)	0.285 (0.194 - 0.394)	0.270 (0.187 - 0.375)	0.389 (0.230 - 0.567)	0.396 (0.231 - 0.573)	0.378 (0.226 - 0.550)	0.168 (0.068 - 0.350)	0.171 (0.069 - 0.352)	0.162 (0.062 - 0.333)
15 - 49 years (rate per capita)	0.429 (0.357 - 0.503)	0.422 (0.352 - 0.490)	0.420 (0.349 - 0.488)	0.766 (0.615 - 0.896)	0.773 (0.641 - 0.901)	0.775 (0.642 - 0.906)	0.255 (0.107 - 0.508)	0.251 (0.107 - 0.497)	0.250 (0.106 - 0.493)
50 - 69 years (rate per capita)	0.398 (0.311 - 0.485)	0.395 (0.303 - 0.485)	0.394 (0.301 - 0.486)	0.798 (0.604 - 0.987)	0.793 (0.610 - 0.973)	0.794 (0.596 - 0.985)	0.231 (0.098 - 0.470)	0.229 (0.098 - 0.473)	0.229 (0.095 - 0.469)
70+ years (rate per capita)	0.374 (0.300 - 0.455)	0.378 (0.301 - 0.459)	0.371 (0.298 - 0.452)	0.748 (0.576 - 0.927)	0.751 (0.573 - 0.935)	0.744 (0.576 - 0.921)	0.209 (0.092 - 0.401)	0.209 (0.095 - 0.405)	0.205 (0.092 - 0.399)
All ages (rate per capita)	0.336 (0.290 - 0.383)	0.357 (0.310 - 0.404)	0.354 (0.310 - 0.398)	0.587 (0.509 - 0.666)	0.644 (0.563 - 0.721)	0.650 (0.571 - 0.726)	0.199 (0.087 - 0.386)	0.211 (0.092 - 0.410)	0.209 (0.092 - 0.411)
Age-standardized (rate per capita)	0.353 (0.309 - 0.397)	0.353 (0.309 - 0.397)	0.353 (0.309 - 0.396)	0.639 (0.562 - 0.712)	0.639 (0.562 - 0.712)	0.639 (0.563 - 0.713)	0.208 (0.092 - 0.408)	0.208 (0.091 - 0.408)	0.208 (0.092 - 0.408)
Total number of cases (thousands)	24 (21 - 27)	32 (28 - 36)	34 (30 - 38)	42 (36 - 47)	57 (50 - 64)	63 (55 - 70)	0 (0 - 0)	0 (0 - 0)	0 (0 - 0)
**Thailand**									
5 -14 years (rate per capita)	0.286 (0.194 - 0.395)	0.285 (0.194 - 0.394)	0.284 (0.193 - 0.393)	0.396 (0.232 - 0.574)	0.396 (0.231 - 0.573)	0.395 (0.231 - 0.572)	0.171 (0.068 - 0.349)	0.171 (0.066 - 0.356)	0.170 (0.067 - 0.354)
15 - 49 years (rate per capita)	0.427 (0.356 - 0.497)	0.421 (0.350 - 0.488)	0.419 (0.347 - 0.487)	0.768 (0.626 - 0.893)	0.773 (0.641 - 0.903)	0.775 (0.645 - 0.908)	0.254 (0.107 - 0.503)	0.250 (0.106 - 0.497)	0.249 (0.106 - 0.493)
50 - 69 years (rate per capita)	0.396 (0.307 - 0.485)	0.396 (0.306 - 0.483)	0.396 (0.307 - 0.485)	0.795 (0.602 - 0.984)	0.795 (0.607 - 0.980)	0.796 (0.605 - 0.988)	0.230 (0.097 - 0.467)	0.230 (0.097 - 0.466)	0.230 (0.098 - 0.471)
70+ years (rate per capita)	0.381 (0.301 - 0.468)	0.383 (0.301 - 0.472)	0.377 (0.300 - 0.457)	0.757 (0.575 - 0.945)	0.759 (0.572 - 0.948)	0.750 (0.571 - 0.933)	0.212 (0.095 - 0.408)	0.214 (0.096 - 0.417)	0.209 (0.094 - 0.405)
All ages (rate per capita)	0.352 (0.305 - 0.400)	0.367 (0.320 - 0.413)	0.371 (0.326 - 0.417)	0.620 (0.540 - 0.699)	0.668 (0.583 - 0.744)	0.689 (0.604 - 0.767)	0.208 (0.090 - 0.405)	0.216 (0.096 - 0.425)	0.218 (0.096 - 0.432)
Age-standardized (rate per capita)	0.353 (0.310 - 0.397)	0.353 (0.309 - 0.397)	0.353 (0.309 - 0.397)	0.639 (0.562 - 0.712)	0.639 (0.562 - 0.712)	0.639 (0.562 - 0.712)	0.208 (0.091 - 0.408)	0.208 (0.092 - 0.411)	0.208 (0.091 - 0.407)
Total number of cases (thousands)	19,939 (17,259 - 22,634)	24,090 (20,993 - 27,151)	25,221 (22,129 - 28,315)	35,147 (30,602 - 39,577)	43,856 (38,287 - 48,887)	46,807 (41,025 - 52,090)	12 (5 - 23)	14 (6 - 28)	15 (7 - 29)
**Timor-Leste**									
5 -14 years (rate per capita)	0.257 (0.178 - 0.357)	0.267 (0.185 - 0.372)	0.281 (0.193 - 0.389)	0.362 (0.220 - 0.531)	0.375 (0.226 - 0.545)	0.392 (0.231 - 0.569)	0.154 (0.061 - 0.311)	0.160 (0.064 - 0.328)	0.168 (0.067 - 0.348)
15 - 49 years (rate per capita)	0.429 (0.357 - 0.500)	0.431 (0.359 - 0.504)	0.430 (0.358 - 0.502)	0.767 (0.619 - 0.893)	0.761 (0.616 - 0.889)	0.765 (0.617 - 0.890)	0.254 (0.107 - 0.506)	0.256 (0.108 - 0.507)	0.255 (0.107 - 0.504)
50 - 69 years (rate per capita)	0.394 (0.301 - 0.487)	0.395 (0.306 - 0.485)	0.398 (0.310 - 0.482)	0.793 (0.601 - 0.985)	0.795 (0.601 - 0.984)	0.798 (0.613 - 0.972)	0.228 (0.096 - 0.463)	0.229 (0.097 - 0.471)	0.230 (0.099 - 0.464)
70+ years (rate per capita)	0.395 (0.302 - 0.492)	0.395 (0.302 - 0.492)	0.393 (0.301 - 0.487)	0.774 (0.573 - 0.978)	0.773 (0.575 - 0.977)	0.772 (0.573 - 0.972)	0.219 (0.099 - 0.435)	0.219 (0.097 - 0.427)	0.218 (0.097 - 0.436)
All ages (rate per capita)	0.312 (0.269 - 0.358)	0.302 (0.258 - 0.350)	0.313 (0.268 - 0.357)	0.543 (0.464 - 0.617)	0.511 (0.437 - 0.588)	0.540 (0.466 - 0.614)	0.185 (0.080 - 0.361)	0.179 (0.077 - 0.347)	0.185 (0.080 - 0.358)

	Prevalence 1990	Prevalence 2005	Prevalence 2015	Incidence 1990	Incidence 2005	Incidence 2015	YLD rate 1990	YLD rate 2005	YLD rate 2015
Age-standardized (rate per capita)	0.353 (0.309 - 0.397)	0.353 (0.309 - 0.397)	0.353 (0.309 - 0.396)	0.639 (0.562 - 0.712)	0.639 (0.562 - 0.712)	0.639 (0.562 - 0.712)	0.207 (0.092 - 0.407)	0.207 (0.091 - 0.407)	0.207 (0.091 - 0.408)
Total number of cases (thousands)	234 (201 - 268)	300 (257 - 348)	373 (319 - 425)	406 (348 - 462)	509 (435 - 586)	643 (554 - 731)	0 (0 - 0)	0 (0 - 0)	0 (0 - 0)
**Vietnam**									
5 -14 years (rate per capita)	0.290 (0.236 - 0.359)	0.312 (0.252 - 0.387)	0.290 (0.236 - 0.359)	0.295 (0.223 - 0.403)	0.313 (0.238 - 0.410)	0.295 (0.224 - 0.402)	0.173 (0.073 - 0.337)	0.186 (0.080 - 0.369)	0.174 (0.074 - 0.340)
15 - 49 years (rate per capita)	0.437 (0.401 - 0.475)	0.421 (0.387 - 0.457)	0.422 (0.387 - 0.457)	0.717 (0.649 - 0.794)	0.718 (0.650 - 0.791)	0.727 (0.659 - 0.799)	0.259 (0.113 - 0.511)	0.250 (0.109 - 0.493)	0.251 (0.108 - 0.497)
50 - 69 years (rate per capita)	0.379 (0.337 - 0.425)	0.370 (0.328 - 0.415)	0.375 (0.331 - 0.422)	0.783 (0.697 - 0.884)	0.774 (0.685 - 0.871)	0.779 (0.689 - 0.886)	0.219 (0.093 - 0.426)	0.214 (0.093 - 0.421)	0.218 (0.093 - 0.423)
70+ years (rate per capita)	0.371 (0.335 - 0.410)	0.363 (0.329 - 0.399)	0.355 (0.324 - 0.389)	0.725 (0.642 - 0.816)	0.715 (0.633 - 0.798)	0.703 (0.626 - 0.785)	0.206 (0.092 - 0.392)	0.201 (0.090 - 0.389)	0.197 (0.087 - 0.381)
All ages (rate per capita)	0.335 (0.310 - 0.359)	0.358 (0.332 - 0.383)	0.356 (0.334 - 0.379)	0.526 (0.485 - 0.573)	0.588 (0.542 - 0.634)	0.610 (0.568 - 0.656)	0.197 (0.085 - 0.381)	0.211 (0.093 - 0.410)	0.211 (0.092 - 0.412)
Age-standardized (rate per capita)	0.352 (0.330 - 0.374)	0.347 (0.323 - 0.369)	0.352 (0.330 - 0.374)	0.596 (0.556 - 0.639)	0.593 (0.553 - 0.634)	0.596 (0.556 - 0.639)	0.207 (0.089 - 0.400)	0.204 (0.089 - 0.394)	0.208 (0.090 - 0.402)
Total number of cases (thousands)	22,836 (21,184 - 24,466)	30,155 (28,005 - 32,309)	33,308 (31,183 - 35,433)	35,922 (33,075 - 39,095)	49,606 (45,723 - 53,469)	57,044 (53,114 - 61,275)	13 (6 - 26)	18 (8 - 35)	20 (9 - 39)
**Oceania**									
5 -14 years (rate per capita)	0.403 (0.341 - 0.469)	0.401 (0.340 - 0.467)	0.405 (0.344 - 0.472)	0.362 (0.282 - 0.460)	0.361 (0.280 - 0.461)	0.363 (0.283 - 0.462)	0.239 (0.104 - 0.457)	0.239 (0.105 - 0.461)	0.241 (0.105 - 0.465)
15 - 49 years (rate per capita)	0.572 (0.535 - 0.608)	0.567 (0.531 - 0.603)	0.566 (0.530 - 0.602)	0.707 (0.647 - 0.772)	0.714 (0.654 - 0.779)	0.715 (0.656 - 0.781)	0.338 (0.150 - 0.664)	0.335 (0.149 - 0.659)	0.335 (0.149 - 0.654)
50 - 69 years (rate per capita)	0.498 (0.452 - 0.547)	0.498 (0.451 - 0.547)	0.499 (0.453 - 0.547)	0.830 (0.739 - 0.921)	0.829 (0.741 - 0.919)	0.830 (0.739 - 0.922)	0.285 (0.123 - 0.559)	0.285 (0.123 - 0.549)	0.285 (0.122 - 0.558)
70+ years (rate per capita)	0.509 (0.465 - 0.553)	0.507 (0.464 - 0.549)	0.505 (0.462 - 0.547)	0.785 (0.697 - 0.880)	0.784 (0.697 - 0.877)	0.784 (0.697 - 0.877)	0.280 (0.126 - 0.537)	0.278 (0.125 - 0.529)	0.277 (0.125 - 0.525)
All ages (rate per capita)	0.434 (0.405 - 0.458)	0.440 (0.412 - 0.463)	0.450 (0.423 - 0.474)	0.521 (0.484 - 0.559)	0.541 (0.504 - 0.580)	0.559 (0.523 - 0.598)	0.255 (0.113 - 0.492)	0.259 (0.114 - 0.502)	0.265 (0.117 - 0.511)
Age-standardized (rate per capita)	0.469 (0.445 - 0.491)	0.469 (0.445 - 0.491)	0.469 (0.444 - 0.491)	0.620 (0.582 - 0.660)	0.620 (0.581 - 0.660)	0.620 (0.582 - 0.660)	0.274 (0.120 - 0.528)	0.274 (0.120 - 0.531)	0.274 (0.120 - 0.531)
Total number of cases (thousands)	2,861 (2,673 - 3,025)	3,997 (3,747 - 4,212)	4,939 (4,637 - 5,203)	3,436 (3,195 - 3,692)	4,920 (4,583 - 5,275)	6,139 (5,735 - 6,561)	2 (1 - 3)	2 (1 - 5)	3 (1 - 6)
**American Samoa**									
5 -14 years (rate per capita)	0.404 (0.342 - 0.472)	0.392 (0.332 - 0.457)	0.384 (0.325 - 0.449)	0.363 (0.282 - 0.463)	0.356 (0.276 - 0.460)	0.352 (0.273 - 0.456)	0.242 (0.105 - 0.469)	0.234 (0.101 - 0.447)	0.230 (0.100 - 0.449)
15 - 49 years (rate per capita)	0.568 (0.531 - 0.605)	0.561 (0.526 - 0.596)	0.556 (0.520 - 0.591)	0.712 (0.651 - 0.779)	0.724 (0.664 - 0.787)	0.732 (0.670 - 0.797)	0.337 (0.149 - 0.669)	0.332 (0.146 - 0.651)	0.329 (0.145 - 0.648)
50 - 69 years (rate per capita)	0.499 (0.452 - 0.548)	0.497 (0.450 - 0.548)	0.496 (0.448 - 0.548)	0.829 (0.739 - 0.919)	0.829 (0.741 - 0.922)	0.829 (0.740 - 0.923)	0.287 (0.124 - 0.558)	0.286 (0.123 - 0.561)	0.285 (0.124 - 0.556)
70+ years (rate per capita)	0.496 (0.455 - 0.535)	0.504 (0.461 - 0.546)	0.510 (0.464 - 0.556)	0.780 (0.697 - 0.870)	0.783 (0.697 - 0.874)	0.786 (0.696 - 0.881)	0.273 (0.122 - 0.521)	0.278 (0.124 - 0.531)	0.281 (0.126 - 0.539)
All ages (rate per capita)	0.428 (0.401 - 0.451)	0.432 (0.406 - 0.456)	0.426 (0.400 - 0.449)	0.525 (0.489 - 0.563)	0.541 (0.503 - 0.579)	0.540 (0.501 - 0.577)	0.253 (0.112 - 0.491)	0.256 (0.111 - 0.495)	0.251 (0.110 - 0.488)
Age-standardized (rate per capita)	0.469 (0.445 - 0.491)	0.469 (0.444 - 0.491)	0.469 (0.445 - 0.491)	0.620 (0.581 - 0.660)	0.620 (0.581 - 0.661)	0.620 (0.581 - 0.661)	0.275 (0.120 - 0.533)	0.275 (0.120 - 0.531)	0.275 (0.120 - 0.535)
Total number of cases (thousands)	20 (19 - 22)	28 (26 - 29)	35 (33 - 37)	25 (23 - 27)	35 (32 - 37)	45 (42 - 48)	0 (0 - 0)	0 (0 - 0)	0 (0 - 0)
**Federated States of Micronesia**									
5 -14 years (rate per capita)	0.396 (0.335 - 0.462)	0.412 (0.349 - 0.481)	0.419 (0.354 - 0.489)	0.359 (0.278 - 0.462)	0.367 (0.284 - 0.464)	0.370 (0.287 - 0.466)	0.236 (0.103 - 0.452)	0.247 (0.107 - 0.470)	0.250 (0.109 - 0.480)
15 - 49 years (rate per capita)	0.575 (0.538 - 0.611)	0.575 (0.538 - 0.611)	0.575 (0.536 - 0.612)	0.703 (0.642 - 0.768)	0.701 (0.638 - 0.768)	0.701 (0.639 - 0.768)	0.341 (0.151 - 0.673)	0.341 (0.150 - 0.670)	0.342 (0.152 - 0.673)
50 - 69 years (rate per capita)	0.503 (0.456 - 0.550)	0.495 (0.445 - 0.548)	0.501 (0.455 - 0.549)	0.831 (0.741 - 0.920)	0.828 (0.736 - 0.925)	0.831 (0.739 - 0.923)	0.289 (0.125 - 0.558)	0.285 (0.122 - 0.558)	0.287 (0.124 - 0.560)
70+ years (rate per capita)	0.509 (0.464 - 0.552)	0.491 (0.452 - 0.530)	0.493 (0.452 - 0.532)	0.785 (0.696 - 0.879)	0.778 (0.697 - 0.866)	0.779 (0.697 - 0.868)	0.281 (0.126 - 0.536)	0.270 (0.121 - 0.522)	0.271 (0.122 - 0.517)
All ages (rate per capita)	0.425 (0.396 - 0.450)	0.445 (0.416 - 0.470)	0.462 (0.433 - 0.488)	0.503 (0.466 - 0.543)	0.532 (0.494 - 0.572)	0.562 (0.523 - 0.604)	0.251 (0.111 - 0.485)	0.263 (0.116 - 0.503)	0.273 (0.121 - 0.524)
Age-standardized (rate per capita)	0.469 (0.445 - 0.491)	0.469 (0.445 - 0.491)	0.469 (0.445 - 0.491)	0.620 (0.581 - 0.660)	0.620 (0.581 - 0.660)	0.620 (0.581 - 0.661)	0.275 (0.120 - 0.535)	0.275 (0.121 - 0.533)	0.275 (0.120 - 0.532)
Total number of cases (thousands)	41 (38 - 43)	48 (45 - 51)	49 (46 - 51)	49 (45 - 53)	57 (53 - 62)	59 (55 - 63)	0 (0 - 0)	0 (0 - 0)	0 (0 - 0)
**Fiji**									
5 -14 years (rate per capita)	0.401 (0.338 - 0.469)	0.424 (0.356 - 0.491)	0.404 (0.341 - 0.473)	0.359 (0.281 - 0.455)	0.372 (0.290 - 0.465)	0.361 (0.282 - 0.456)	0.239 (0.104 - 0.467)	0.252 (0.111 - 0.491)	0.241 (0.105 - 0.466)
15 - 49 years (rate per capita)	0.567 (0.530 - 0.604)	0.562 (0.527 - 0.598)	0.562 (0.526 - 0.599)	0.714 (0.652 - 0.783)	0.720 (0.661 - 0.784)	0.722 (0.660 - 0.790)	0.335 (0.147 - 0.655)	0.332 (0.145 - 0.654)	0.332 (0.146 - 0.654)
50 - 69 years (rate per capita)	0.498 (0.449 - 0.547)	0.498 (0.450 - 0.549)	0.499 (0.452 - 0.547)	0.831 (0.738 - 0.924)	0.830 (0.742 - 0.920)	0.831 (0.739 - 0.922)	0.284 (0.123 - 0.553)	0.284 (0.122 - 0.553)	0.284 (0.125 - 0.555)
70+ years (rate per capita)	0.508 (0.461 - 0.552)	0.510 (0.466 - 0.553)	0.509 (0.461 - 0.553)	0.785 (0.696 - 0.883)	0.786 (0.696 - 0.880)	0.785 (0.696 - 0.884)	0.279 (0.127 - 0.529)	0.280 (0.127 - 0.532)	0.280 (0.125 - 0.531)
All ages (rate per capita)	0.438 (0.411 - 0.463)	0.461 (0.437 - 0.485)	0.465 (0.441 - 0.488)	0.536 (0.498 - 0.575)	0.585 (0.547 - 0.624)	0.603 (0.564 - 0.643)	0.258 (0.114 - 0.495)	0.271 (0.118 - 0.526)	0.273 (0.119 - 0.524)
Age-standardized (rate per capita)	0.469 (0.445 - 0.491)	0.471 (0.448 - 0.494)	0.469 (0.445 - 0.491)	0.619 (0.582 - 0.659)	0.620 (0.583 - 0.658)	0.619 (0.582 - 0.659)	0.274 (0.120 - 0.527)	0.275 (0.120 - 0.538)	0.274 (0.119 - 0.528)
Total number of cases (thousands)	321 (302 - 340)	382 (362 - 401)	414 (393 - 435)	393 (366 - 422)	484 (453 - 517)	537 (503 - 574)	0 (0 - 0)	0 (0 - 0)	0 (0 - 0)
**Guam**									
5 -14 years (rate per capita)	0.402 (0.340 - 0.468)	0.407 (0.345 - 0.475)	0.416 (0.352 - 0.485)	0.361 (0.281 - 0.463)	0.364 (0.283 - 0.464)	0.369 (0.286 - 0.465)	0.240 (0.104 - 0.463)	0.244 (0.106 - 0.465)	0.249 (0.109 - 0.476)
15 - 49 years (rate per capita)	0.563 (0.526 - 0.599)	0.559 (0.524 - 0.594)	0.561 (0.525 - 0.596)	0.721 (0.659 - 0.787)	0.726 (0.666 - 0.791)	0.722 (0.663 - 0.785)	0.334 (0.149 - 0.659)	0.332 (0.147 - 0.650)	0.333 (0.146 - 0.656)
50 - 69 years (rate per capita)	0.500 (0.454 - 0.548)	0.499 (0.452 - 0.548)	0.501 (0.455 - 0.548)	0.830 (0.738 - 0.920)	0.830 (0.739 - 0.921)	0.830 (0.739 - 0.919)	0.289 (0.125 - 0.563)	0.288 (0.124 - 0.569)	0.289 (0.123 - 0.566)
70+ years (rate per capita)	0.504 (0.461 - 0.546)	0.499 (0.457 - 0.539)	0.492 (0.452 - 0.532)	0.784 (0.696 - 0.876)	0.781 (0.697 - 0.872)	0.779 (0.697 - 0.868)	0.282 (0.127 - 0.542)	0.277 (0.124 - 0.535)	0.273 (0.123 - 0.530)
All ages (rate per capita)	0.456 (0.430 - 0.479)	0.462 (0.437 - 0.484)	0.474 (0.449 - 0.497)	0.580 (0.542 - 0.622)	0.600 (0.562 - 0.640)	0.625 (0.586 - 0.667)	0.270 (0.120 - 0.524)	0.272 (0.119 - 0.528)	0.279 (0.122 - 0.542)
Age-standardized (rate per capita)	0.468 (0.444 - 0.491)	0.469 (0.445 - 0.491)	0.469 (0.445 - 0.491)	0.620 (0.581 - 0.661)	0.620 (0.581 - 0.660)	0.620 (0.581 - 0.660)	0.276 (0.122 - 0.534)	0.276 (0.120 - 0.535)	0.276 (0.121 - 0.534)
Total number of cases (thousands)	60 (57 - 63)	73 (69 - 77)	81 (76 - 84)	76 (71 - 82)	95 (89 - 101)	106 (100 - 113)	0 (0 - 0)	0 (0 - 0)	0 (0 - 0)
**Kiribati**									
5 -14 years (rate per capita)	0.393 (0.334 - 0.459)	0.397 (0.336 - 0.463)	0.389 (0.329 - 0.454)	0.357 (0.276 - 0.461)	0.359 (0.279 - 0.462)	0.355 (0.275 - 0.459)	0.232 (0.101 - 0.445)	0.235 (0.102 - 0.454)	0.231 (0.100 - 0.442)
15 - 49 years (rate per capita)	0.567 (0.529 - 0.605)	0.570 (0.534 - 0.606)	0.568 (0.531 - 0.603)	0.713 (0.652 - 0.781)	0.710 (0.650 - 0.775)	0.713 (0.652 - 0.780)	0.335 (0.150 - 0.662)	0.336 (0.151 - 0.664)	0.335 (0.147 - 0.658)
50 - 69 years (rate per capita)	0.500 (0.454 - 0.548)	0.498 (0.451 - 0.548)	0.497 (0.449 - 0.548)	0.829 (0.739 - 0.920)	0.829 (0.740 - 0.923)	0.829 (0.740 - 0.923)	0.286 (0.124 - 0.561)	0.284 (0.123 - 0.557)	0.284 (0.123 - 0.553)
70+ years (rate per capita)	0.510 (0.466 - 0.554)	0.514 (0.468 - 0.559)	0.509 (0.465 - 0.552)	0.786 (0.696 - 0.880)	0.787 (0.696 - 0.882)	0.785 (0.694 - 0.878)	0.281 (0.125 - 0.536)	0.282 (0.126 - 0.530)	0.279 (0.126 - 0.531)
All ages (rate per capita)	0.426 (0.399 - 0.450)	0.444 (0.416 - 0.468)	0.444 (0.418 - 0.467)	0.524 (0.487 - 0.563)	0.541 (0.504 - 0.581)	0.555 (0.518 - 0.595)	0.250 (0.111 - 0.482)	0.261 (0.115 - 0.502)	0.261 (0.115 - 0.506)
Age-standardized (rate per capita)	0.469 (0.445 - 0.492)	0.469 (0.445 - 0.492)	0.469 (0.445 - 0.491)	0.620 (0.581 - 0.660)	0.620 (0.581 - 0.660)	0.620 (0.581 - 0.660)	0.274 (0.120 - 0.529)	0.273 (0.121 - 0.528)	0.274 (0.121 - 0.531)
Total number of cases (thousands)	31 (29 - 33)	42 (39 - 44)	50 (47 - 53)	38 (35 - 41)	51 (47 - 55)	63 (58 - 67)	0 (0 - 0)	0 (0 - 0)	0 (0 - 0)
**Marshall Islands**									
5 -14 years (rate per capita)	0.403 (0.341 - 0.470)	0.399 (0.338 - 0.466)	0.405 (0.344 - 0.473)	0.362 (0.281 - 0.463)	0.360 (0.280 - 0.462)	0.363 (0.282 - 0.463)	0.239 (0.104 - 0.460)	0.238 (0.104 - 0.460)	0.241 (0.103 - 0.465)
15 - 49 years (rate per capita)	0.572 (0.535 - 0.608)	0.567 (0.530 - 0.603)	0.567 (0.531 - 0.603)	0.707 (0.647 - 0.773)	0.714 (0.653 - 0.779)	0.715 (0.655 - 0.780)	0.338 (0.150 - 0.662)	0.335 (0.149 - 0.661)	0.335 (0.148 - 0.658)
50 - 69 years (rate per capita)	0.498 (0.451 - 0.548)	0.498 (0.451 - 0.548)	0.499 (0.452 - 0.548)	0.830 (0.739 - 0.922)	0.829 (0.740 - 0.922)	0.830 (0.739 - 0.922)	0.284 (0.121 - 0.554)	0.283 (0.121 - 0.551)	0.283 (0.122 - 0.548)
70+ years (rate per capita)	0.510 (0.465 - 0.554)	0.507 (0.463 - 0.550)	0.505 (0.462 - 0.548)	0.786 (0.696 - 0.879)	0.785 (0.697 - 0.877)	0.784 (0.696 - 0.876)	0.279 (0.126 - 0.535)	0.277 (0.124 - 0.524)	0.275 (0.124 - 0.527)
All ages (rate per capita)	0.418 (0.391 - 0.442)	0.432 (0.404 - 0.456)	0.447 (0.420 - 0.471)	0.502 (0.466 - 0.540)	0.531 (0.495 - 0.570)	0.555 (0.518 - 0.593)	0.247 (0.110 - 0.476)	0.254 (0.112 - 0.494)	0.263 (0.115 - 0.512)
Age-standardized (rate per capita)	0.469 (0.445 - 0.491)	0.469 (0.445 - 0.491)	0.469 (0.445 - 0.491)	0.620 (0.581 - 0.660)	0.620 (0.581 - 0.660)	0.620 (0.581 - 0.660)	0.274 (0.121 - 0.524)	0.273 (0.119 - 0.529)	0.273 (0.120 - 0.534)
Total number of cases (thousands)	20 (19 - 21)	26 (24 - 27)	32 (30 - 34)	24 (22 - 26)	32 (30 - 34)	40 (37 - 43)	0 (0 - 0)	0 (0 - 0)	0 (0 - 0)
**Northern Mariana Islands**									
5 -14 years (rate per capita)	0.400 (0.338 - 0.466)	0.395 (0.335 - 0.461)	0.402 (0.340 - 0.468)	0.361 (0.280 - 0.462)	0.358 (0.277 - 0.461)	0.361 (0.280 - 0.463)	0.239 (0.105 - 0.452)	0.236 (0.102 - 0.454)	0.240 (0.104 - 0.454)
15 - 49 years (rate per capita)	0.557 (0.517 - 0.596)	0.553 (0.513 - 0.592)	0.551 (0.511 - 0.590)	0.730 (0.665 - 0.804)	0.736 (0.672 - 0.807)	0.738 (0.672 - 0.806)	0.331 (0.148 - 0.651)	0.328 (0.144 - 0.644)	0.327 (0.146 - 0.642)
50 - 69 years (rate per capita)	0.494 (0.443 - 0.547)	0.492 (0.439 - 0.547)	0.491 (0.437 - 0.548)	0.827 (0.733 - 0.924)	0.826 (0.731 - 0.927)	0.825 (0.727 - 0.928)	0.286 (0.122 - 0.555)	0.285 (0.122 - 0.562)	0.285 (0.124 - 0.550)
70+ years (rate per capita)	0.502 (0.460 - 0.544)	0.499 (0.458 - 0.541)	0.498 (0.457 - 0.539)	0.783 (0.694 - 0.875)	0.782 (0.697 - 0.870)	0.781 (0.696 - 0.870)	0.279 (0.126 - 0.531)	0.277 (0.124 - 0.536)	0.276 (0.124 - 0.529)

	Prevalence 1990	Prevalence 2005	Prevalence 2015	Incidence 1990	Incidence 2005	Incidence 2015	YLD rate 1990	YLD rate 2005	YLD rate 2015
All ages (rate per capita)	0.479 (0.450 - 0.506)	0.482 (0.453 - 0.508)	0.487 (0.457 - 0.515)	0.617 (0.571 - 0.667)	0.630 (0.584 - 0.680)	0.641 (0.593 - 0.691)	0.284 (0.127 - 0.554)	0.286 (0.127 - 0.557)	0.289 (0.128 - 0.563)
Age-standardized (rate per capita)	0.469 (0.445 - 0.491)	0.469 (0.445 - 0.492)	0.469 (0.445 - 0.492)	0.619 (0.581 - 0.659)	0.619 (0.581 - 0.659)	0.619 (0.581 - 0.659)	0.276 (0.120 - 0.533)	0.276 (0.120 - 0.534)	0.276 (0.120 - 0.531)
Total number of cases (thousands)	21 (19 - 22)	40 (37 - 42)	56 (53 - 60)	27 (25 - 29)	52 (48 - 56)	74 (69 - 80)	0 (0 - 0)	0 (0 - 0)	0 (0 - 0)
**Papua New Guinea**									
5 -14 years (rate per capita)	0.403 (0.342 - 0.470)	0.397 (0.336 - 0.463)	0.405 (0.343 - 0.472)	0.362 (0.282 - 0.463)	0.359 (0.278 - 0.462)	0.363 (0.282 - 0.463)	0.239 (0.104 - 0.456)	0.236 (0.103 - 0.455)	0.241 (0.104 - 0.464)
15 - 49 years (rate per capita)	0.573 (0.537 - 0.609)	0.568 (0.531 - 0.605)	0.568 (0.531 - 0.604)	0.705 (0.644 - 0.770)	0.713 (0.653 - 0.778)	0.714 (0.654 - 0.779)	0.338 (0.149 - 0.663)	0.335 (0.151 - 0.657)	0.335 (0.150 - 0.655)
50 - 69 years (rate per capita)	0.498 (0.450 - 0.548)	0.498 (0.450 - 0.548)	0.498 (0.451 - 0.548)	0.829 (0.739 - 0.923)	0.829 (0.740 - 0.923)	0.830 (0.739 - 0.923)	0.285 (0.123 - 0.558)	0.284 (0.123 - 0.548)	0.284 (0.122 - 0.558)
70+ years (rate per capita)	0.514 (0.469 - 0.559)	0.511 (0.466 - 0.555)	0.510 (0.466 - 0.553)	0.787 (0.695 - 0.883)	0.786 (0.696 - 0.880)	0.786 (0.696 - 0.880)	0.282 (0.127 - 0.543)	0.279 (0.126 - 0.534)	0.279 (0.125 - 0.521)
All ages (rate per capita)	0.432 (0.404 - 0.458)	0.434 (0.406 - 0.458)	0.446 (0.418 - 0.470)	0.514 (0.477 - 0.554)	0.529 (0.491 - 0.568)	0.547 (0.510 - 0.586)	0.255 (0.112 - 0.489)	0.255 (0.113 - 0.493)	0.262 (0.116 - 0.506)
Age-standardized (rate per capita)	0.469 (0.445 - 0.491)	0.469 (0.445 - 0.491)	0.469 (0.445 - 0.491)	0.620 (0.581 - 0.660)	0.620 (0.581 - 0.660)	0.620 (0.581 - 0.660)	0.273 (0.120 - 0.527)	0.273 (0.120 - 0.529)	0.274 (0.120 - 0.532)
Total number of cases (thousands)	1,802 (1,682 - 1,909)	2,642 (2,471 - 2,788)	3,403 (3,188 - 3,588)	2,144 (1,989 - 2,308)	3,223 (2,993 - 3,463)	4,179 (3,891 - 4,473)	1 (0 - 2)	2 (1 - 3)	2 (1 - 4)
**Samoa**									
5 -14 years (rate per capita)	0.409 (0.347 - 0.478)	0.406 (0.344 - 0.473)	0.401 (0.340 - 0.468)	0.366 (0.283 - 0.464)	0.364 (0.282 - 0.463)	0.361 (0.281 - 0.463)	0.245 (0.107 - 0.470)	0.243 (0.105 - 0.466)	0.240 (0.104 - 0.464)
15 - 49 years (rate per capita)	0.577 (0.539 - 0.614)	0.567 (0.531 - 0.602)	0.569 (0.533 - 0.605)	0.699 (0.637 - 0.766)	0.715 (0.655 - 0.780)	0.710 (0.650 - 0.774)	0.343 (0.151 - 0.676)	0.336 (0.149 - 0.662)	0.338 (0.150 - 0.666)
50 - 69 years (rate per capita)	0.502 (0.455 - 0.549)	0.500 (0.454 - 0.548)	0.499 (0.451 - 0.547)	0.830 (0.739 - 0.921)	0.829 (0.740 - 0.918)	0.830 (0.738 - 0.922)	0.288 (0.125 - 0.563)	0.287 (0.125 - 0.563)	0.286 (0.123 - 0.556)
70+ years (rate per capita)	0.507 (0.463 - 0.549)	0.498 (0.457 - 0.539)	0.492 (0.452 - 0.531)	0.784 (0.696 - 0.878)	0.781 (0.695 - 0.873)	0.779 (0.697 - 0.868)	0.280 (0.127 - 0.539)	0.275 (0.125 - 0.532)	0.271 (0.122 - 0.522)
All ages (rate per capita)	0.432 (0.404 - 0.457)	0.435 (0.407 - 0.459)	0.443 (0.416 - 0.467)	0.514 (0.477 - 0.553)	0.535 (0.498 - 0.573)	0.550 (0.513 - 0.588)	0.256 (0.112 - 0.490)	0.257 (0.114 - 0.497)	0.262 (0.115 - 0.505)
Age-standardized (rate per capita)	0.468 (0.444 - 0.491)	0.469 (0.444 - 0.491)	0.469 (0.445 - 0.491)	0.620 (0.581 - 0.661)	0.620 (0.581 - 0.660)	0.620 (0.581 - 0.660)	0.275 (0.121 - 0.536)	0.275 (0.121 - 0.538)	0.275 (0.121 - 0.533)
Total number of cases (thousands)	72 (67 - 76)	79 (74 - 83)	86 (81 - 91)	86 (80 - 92)	97 (90 - 104)	107 (99 - 114)	0 (0 - 0)	0 (0 - 0)	0 (0 - 0)
**Solomon Islands**									
5 -14 years (rate per capita)	0.404 (0.342 - 0.471)	0.402 (0.340 - 0.469)	0.402 (0.340 - 0.469)	0.363 (0.282 - 0.463)	0.362 (0.281 - 0.463)	0.362 (0.281 - 0.463)	0.241 (0.105 - 0.457)	0.240 (0.105 - 0.464)	0.240 (0.105 - 0.460)
15 - 49 years (rate per capita)	0.575 (0.537 - 0.611)	0.570 (0.533 - 0.606)	0.569 (0.533 - 0.605)	0.703 (0.642 - 0.770)	0.710 (0.649 - 0.776)	0.711 (0.651 - 0.777)	0.340 (0.151 - 0.667)	0.338 (0.149 - 0.669)	0.337 (0.149 - 0.661)
50 - 69 years (rate per capita)	0.499 (0.452 - 0.548)	0.499 (0.452 - 0.547)	0.499 (0.452 - 0.548)	0.830 (0.738 - 0.920)	0.830 (0.738 - 0.922)	0.829 (0.740 - 0.921)	0.287 (0.122 - 0.555)	0.287 (0.123 - 0.556)	0.286 (0.122 - 0.559)
70+ years (rate per capita)	0.505 (0.461 - 0.546)	0.506 (0.462 - 0.548)	0.504 (0.461 - 0.546)	0.783 (0.696 - 0.876)	0.784 (0.697 - 0.877)	0.783 (0.697 - 0.877)	0.279 (0.125 - 0.534)	0.280 (0.126 - 0.534)	0.278 (0.125 - 0.534)
All ages (rate per capita)	0.416 (0.387 - 0.441)	0.429 (0.401 - 0.453)	0.438 (0.409 - 0.462)	0.491 (0.454 - 0.530)	0.518 (0.481 - 0.557)	0.532 (0.495 - 0.570)	0.246 (0.108 - 0.471)	0.254 (0.111 - 0.490)	0.259 (0.113 - 0.500)
Age-standardized (rate per capita)	0.469 (0.444 - 0.491)	0.469 (0.445 - 0.491)	0.469 (0.445 - 0.491)	0.620 (0.581 - 0.660)	0.620 (0.581 - 0.660)	0.620 (0.581 - 0.660)	0.275 (0.120 - 0.532)	0.275 (0.120 - 0.532)	0.275 (0.120 - 0.534)
Total number of cases (thousands)	131 (122 - 139)	203 (189 - 214)	256 (240 - 271)	155 (143 - 167)	245 (227 - 263)	312 (290 - 334)	0 (0 - 0)	0 (0 - 0)	0 (0 - 0)
**Tonga**									
5 -14 years (rate per capita)	0.395 (0.335 - 0.461)	0.405 (0.343 - 0.472)	0.408 (0.346 - 0.476)	0.358 (0.277 - 0.461)	0.363 (0.282 - 0.463)	0.365 (0.283 - 0.464)	0.236 (0.102 - 0.455)	0.242 (0.106 - 0.463)	0.244 (0.105 - 0.471)
15 - 49 years (rate per capita)	0.576 (0.537 - 0.613)	0.568 (0.532 - 0.604)	0.570 (0.534 - 0.606)	0.700 (0.638 - 0.769)	0.712 (0.650 - 0.778)	0.710 (0.649 - 0.775)	0.342 (0.153 - 0.668)	0.337 (0.149 - 0.665)	0.338 (0.149 - 0.669)
50 - 69 years (rate per capita)	0.503 (0.456 - 0.550)	0.503 (0.458 - 0.549)	0.499 (0.452 - 0.548)	0.831 (0.740 - 0.921)	0.829 (0.741 - 0.919)	0.831 (0.737 - 0.925)	0.287 (0.125 - 0.560)	0.288 (0.124 - 0.562)	0.286 (0.123 - 0.559)
70+ years (rate per capita)	0.500 (0.458 - 0.541)	0.498 (0.456 - 0.539)	0.485 (0.448 - 0.524)	0.782 (0.696 - 0.873)	0.781 (0.695 - 0.873)	0.776 (0.696 - 0.863)	0.275 (0.123 - 0.532)	0.274 (0.121 - 0.520)	0.266 (0.120 - 0.510)
All ages (rate per capita)	0.433 (0.406 - 0.458)	0.441 (0.413 - 0.465)	0.448 (0.420 - 0.472)	0.523 (0.486 - 0.563)	0.543 (0.506 - 0.581)	0.553 (0.517 - 0.591)	0.256 (0.112 - 0.489)	0.260 (0.114 - 0.501)	0.264 (0.116 - 0.509)
Age-standardized (rate per capita)	0.469 (0.445 - 0.491)	0.469 (0.445 - 0.491)	0.469 (0.445 - 0.491)	0.620 (0.581 - 0.660)	0.620 (0.581 - 0.660)	0.620 (0.581 - 0.660)	0.275 (0.120 - 0.532)	0.275 (0.121 - 0.531)	0.275 (0.120 - 0.533)
Total number of cases (thousands)	42 (39 - 44)	45 (42 - 47)	48 (45 - 50)	50 (47 - 54)	55 (51 - 59)	59 (55 - 63)	0 (0 - 0)	0 (0 - 0)	0 (0 - 0)
**Vanuatu**									
5 -14 years (rate per capita)	0.396 (0.336 - 0.463)	0.402 (0.340 - 0.469)	0.396 (0.335 - 0.462)	0.359 (0.278 - 0.462)	0.362 (0.281 - 0.463)	0.359 (0.278 - 0.461)	0.237 (0.104 - 0.457)	0.240 (0.104 - 0.467)	0.236 (0.102 - 0.459)
15 - 49 years (rate per capita)	0.570 (0.533 - 0.606)	0.570 (0.533 - 0.606)	0.566 (0.530 - 0.602)	0.710 (0.650 - 0.776)	0.710 (0.649 - 0.775)	0.716 (0.655 - 0.780)	0.338 (0.149 - 0.672)	0.338 (0.148 - 0.669)	0.335 (0.148 - 0.657)
50 - 69 years (rate per capita)	0.500 (0.454 - 0.548)	0.499 (0.452 - 0.548)	0.499 (0.452 - 0.548)	0.829 (0.740 - 0.919)	0.830 (0.739 - 0.920)	0.829 (0.741 - 0.921)	0.288 (0.123 - 0.565)	0.287 (0.125 - 0.568)	0.287 (0.124 - 0.561)
70+ years (rate per capita)	0.496 (0.454 - 0.535)	0.499 (0.457 - 0.539)	0.512 (0.467 - 0.556)	0.780 (0.696 - 0.871)	0.781 (0.696 - 0.873)	0.786 (0.695 - 0.881)	0.275 (0.124 - 0.529)	0.276 (0.125 - 0.529)	0.283 (0.126 - 0.539)
All ages (rate per capita)	0.420 (0.392 - 0.445)	0.439 (0.411 - 0.463)	0.447 (0.419 - 0.470)	0.506 (0.469 - 0.544)	0.533 (0.496 - 0.572)	0.556 (0.518 - 0.595)	0.248 (0.109 - 0.480)	0.260 (0.114 - 0.504)	0.264 (0.116 - 0.513)
Age-standardized (rate per capita)	0.469 (0.444 - 0.491)	0.469 (0.444 - 0.491)	0.469 (0.445 - 0.491)	0.620 (0.581 - 0.660)	0.620 (0.581 - 0.660)	0.620 (0.581 - 0.660)	0.275 (0.121 - 0.531)	0.275 (0.120 - 0.531)	0.275 (0.120 - 0.533)
Total number of cases (thousands)	62 (58 - 66)	92 (86 - 97)	117 (110 - 124)	75 (69 - 80)	111 (104 - 120)	146 (136 - 156)	0 (0 - 0)	0 (0 - 0)	0 (0 - 0)
**North Africa and Middle East**									
5 -14 years (rate per capita)	0.352 (0.289 - 0.419)	0.354 (0.291 - 0.422)	0.350 (0.285 - 0.419)	0.515 (0.401 - 0.637)	0.521 (0.406 - 0.643)	0.523 (0.408 - 0.647)	0.211 (0.090 - 0.411)	0.212 (0.091 - 0.408)	0.210 (0.089 - 0.411)
15 - 49 years (rate per capita)	0.469 (0.422 - 0.512)	0.468 (0.422 - 0.511)	0.468 (0.422 - 0.512)	0.797 (0.718 - 0.882)	0.798 (0.720 - 0.880)	0.803 (0.724 - 0.885)	0.278 (0.120 - 0.551)	0.278 (0.120 - 0.552)	0.278 (0.121 - 0.548)
50 - 69 years (rate per capita)	0.452 (0.397 - 0.503)	0.451 (0.395 - 0.505)	0.450 (0.394 - 0.504)	0.882 (0.774 - 0.982)	0.879 (0.767 - 0.983)	0.876 (0.763 - 0.980)	0.260 (0.113 - 0.503)	0.259 (0.113 - 0.504)	0.259 (0.113 - 0.505)
70+ years (rate per capita)	0.445 (0.398 - 0.491)	0.447 (0.398 - 0.494)	0.441 (0.394 - 0.487)	0.882 (0.775 - 0.986)	0.880 (0.770 - 0.982)	0.875 (0.768 - 0.976)	0.244 (0.109 - 0.466)	0.246 (0.111 - 0.469)	0.242 (0.108 - 0.464)
All ages (rate per capita)	0.362 (0.334 - 0.390)	0.386 (0.357 - 0.414)	0.389 (0.359 - 0.417)	0.607 (0.558 - 0.657)	0.655 (0.604 - 0.707)	0.668 (0.619 - 0.717)	0.214 (0.094 - 0.411)	0.228 (0.101 - 0.440)	0.230 (0.102 - 0.441)
Age-standardized (rate per capita)	0.403 (0.375 - 0.430)	0.402 (0.374 - 0.428)	0.401 (0.373 - 0.430)	0.702 (0.655 - 0.751)	0.700 (0.654 - 0.749)	0.702 (0.655 - 0.751)	0.237 (0.104 - 0.453)	0.236 (0.103 - 0.450)	0.236 (0.104 - 0.451)
Total number of cases (thousands)	121,658 (112,271 - 131,038)	179,580 (165,970 - 192,566)	220,038 (203,440 - 235,874)	204,056 (187,364 - 220,789)	304,553 (280,904 - 328,768)	378,081 (350,730 - 406,106)	72 (32 - 138)	106 (47 - 204)	130 (57 - 250)
**North Africa and Middle East**									
5 -14 years (rate per capita)	0.352 (0.289 - 0.419)	0.354 (0.291 - 0.422)	0.350 (0.285 - 0.419)	0.515 (0.401 - 0.637)	0.521 (0.406 - 0.643)	0.523 (0.408 - 0.647)	0.211 (0.090 - 0.411)	0.212 (0.091 - 0.408)	0.210 (0.089 - 0.411)
15 - 49 years (rate per capita)	0.469 (0.422 - 0.512)	0.468 (0.422 - 0.511)	0.468 (0.422 - 0.512)	0.797 (0.718 - 0.882)	0.798 (0.720 - 0.880)	0.803 (0.724 - 0.885)	0.278 (0.120 - 0.551)	0.278 (0.120 - 0.552)	0.278 (0.121 - 0.548)
50 - 69 years (rate per capita)	0.452 (0.397 - 0.503)	0.451 (0.395 - 0.505)	0.450 (0.394 - 0.504)	0.882 (0.774 - 0.982)	0.879 (0.767 - 0.983)	0.876 (0.763 - 0.980)	0.260 (0.113 - 0.503)	0.259 (0.113 - 0.504)	0.259 (0.113 - 0.505)
70+ years (rate per capita)	0.445 (0.398 - 0.491)	0.447 (0.398 - 0.494)	0.441 (0.394 - 0.487)	0.882 (0.775 - 0.986)	0.880 (0.770 - 0.982)	0.875 (0.768 - 0.976)	0.244 (0.109 - 0.466)	0.246 (0.111 - 0.469)	0.242 (0.108 - 0.464)
All ages (rate per capita)	0.362 (0.334 - 0.390)	0.386 (0.357 - 0.414)	0.389 (0.359 - 0.417)	0.607 (0.558 - 0.657)	0.655 (0.604 - 0.707)	0.668 (0.619 - 0.717)	0.214 (0.094 - 0.411)	0.228 (0.101 - 0.440)	0.230 (0.102 - 0.441)
Age-standardized (rate per capita)	0.403 (0.375 - 0.430)	0.402 (0.374 - 0.428)	0.401 (0.373 - 0.430)	0.702 (0.655 - 0.751)	0.700 (0.654 - 0.749)	0.702 (0.655 - 0.751)	0.237 (0.104 - 0.453)	0.236 (0.103 - 0.450)	0.236 (0.104 - 0.451)
Total number of cases (thousands)	121,658 (112,271 - 131,038)	179,580 (165,970 - 192,566)	220,038 (203,440 - 235,874)	204,056 (187,364 - 220,789)	304,553 (280,904 - 328,768)	378,081 (350,730 - 406,106)	72 (32 - 138)	106 (47 - 204)	130 (57 - 250)
**Afghanistan**									
5 -14 years (rate per capita)	0.331 (0.251 - 0.422)	0.329 (0.250 - 0.421)	0.337 (0.256 - 0.429)	0.537 (0.393 - 0.692)	0.535 (0.393 - 0.690)	0.545 (0.401 - 0.701)	0.196 (0.081 - 0.381)	0.196 (0.083 - 0.383)	0.201 (0.085 - 0.391)
15 - 49 years (rate per capita)	0.455 (0.392 - 0.514)	0.454 (0.390 - 0.515)	0.454 (0.391 - 0.515)	0.808 (0.692 - 0.920)	0.807 (0.691 - 0.923)	0.807 (0.691 - 0.923)	0.261 (0.111 - 0.521)	0.264 (0.112 - 0.522)	0.266 (0.112 - 0.524)
50 - 69 years (rate per capita)	0.446 (0.367 - 0.517)	0.445 (0.366 - 0.517)	0.445 (0.367 - 0.517)	0.874 (0.706 - 1.026)	0.873 (0.702 - 1.028)	0.874 (0.705 - 1.027)	0.251 (0.108 - 0.501)	0.247 (0.107 - 0.485)	0.243 (0.106 - 0.478)
70+ years (rate per capita)	0.446 (0.366 - 0.525)	0.441 (0.366 - 0.517)	0.442 (0.366 - 0.518)	0.883 (0.717 - 1.038)	0.878 (0.721 - 1.026)	0.879 (0.720 - 1.026)	0.240 (0.109 - 0.474)	0.237 (0.107 - 0.463)	0.236 (0.107 - 0.466)
All ages (rate per capita)	0.324 (0.289 - 0.360)	0.330 (0.293 - 0.367)	0.350 (0.311 - 0.389)	0.568 (0.499 - 0.630)	0.580 (0.510 - 0.646)	0.613 (0.539 - 0.680)	0.187 (0.081 - 0.364)	0.192 (0.084 - 0.371)	0.204 (0.089 - 0.395)
Age-standardized (rate per capita)	0.392 (0.355 - 0.431)	0.392 (0.355 - 0.431)	0.392 (0.355 - 0.431)	0.709 (0.644 - 0.774)	0.709 (0.644 - 0.775)	0.709 (0.644 - 0.774)	0.223 (0.099 - 0.439)	0.224 (0.099 - 0.437)	0.225 (0.100 - 0.437)
Total number of cases (thousands)	3,985 (3,551 - 4,435)	8,071 (7,165 - 8,970)	11,401 (10,152 - 12,670)	6,984 (6,135 - 7,757)	14,173 (12,462 - 15,771)	19,983 (17,591 - 22,185)	2 (1 - 4)	5 (2 - 9)	7 (3 - 13)
**Algeria**									
5 -14 years (rate per capita)	0.336 (0.255 - 0.428)	0.355 (0.271 - 0.448)	0.325 (0.248 - 0.418)	0.543 (0.400 - 0.699)	0.567 (0.412 - 0.726)	0.531 (0.388 - 0.686)	0.201 (0.084 - 0.395)	0.213 (0.089 - 0.413)	0.195 (0.082 - 0.385)
15 - 49 years (rate per capita)	0.454 (0.390 - 0.515)	0.455 (0.391 - 0.512)	0.456 (0.391 - 0.514)	0.807 (0.690 - 0.924)	0.810 (0.696 - 0.923)	0.814 (0.702 - 0.926)	0.269 (0.114 - 0.537)	0.270 (0.114 - 0.532)	0.270 (0.115 - 0.532)
50 - 69 years (rate per capita)	0.446 (0.368 - 0.517)	0.446 (0.366 - 0.517)	0.446 (0.367 - 0.518)	0.875 (0.711 - 1.027)	0.873 (0.705 - 1.026)	0.874 (0.707 - 1.026)	0.257 (0.111 - 0.507)	0.257 (0.110 - 0.509)	0.256 (0.111 - 0.513)

	Prevalence 1990	Prevalence 2005	Prevalence 2015	Incidence 1990	Incidence 2005	Incidence 2015	YLD rate 1990	YLD rate 2005	YLD rate 2015
**70+ years (rate per capita)**	0.438 (0.365 - 0.510)	0.436 (0.364 - 0.506)	0.428 (0.360 - 0.496)	0.876 (0.721 - 1.021)	0.874 (0.723 - 1.016)	0.867 (0.721 - 0.999)	0.241 (0.108 - 0.474)	0.240 (0.107 - 0.464)	0.235 (0.105 - 0.460)
**All ages (rate per capita)**	0.349 (0.310 - 0.387)	0.391 (0.350 - 0.433)	0.379 (0.340 - 0.417)	0.615 (0.542 - 0.682)	0.694 (0.621 - 0.765)	0.682 (0.615 - 0.748)	0.207 (0.089 - 0.398)	0.231 (0.100 - 0.446)	0.223 (0.098 - 0.434)
**Age-standardized (rate per capita)**	0.392 (0.355 - 0.431)	0.392 (0.355 - 0.431)	0.392 (0.355 - 0.431)	0.709 (0.644 - 0.774)	0.709 (0.644 - 0.774)	0.709 (0.644 - 0.774)	0.230 (0.101 - 0.444)	0.230 (0.102 - 0.445)	0.230 (0.102 - 0.444)
**Total number of cases (thousands)**	9,073 (8,052 - 10,056)	13,066 (11,699 - 14,450)	15,026 (13,491 - 16,527)	15,957 (14,086 - 17,698)	23,193 (20,742 - 25,533)	27,040 (24,374 - 29,665)	5 (2 - 10)	8 (3 - 15)	9 (4 - 17)
**Bahrain**									
**5 -14 years (rate per capita)**	0.330 (0.251 - 0.422)	0.332 (0.252 - 0.424)	0.334 (0.254 - 0.426)	0.536 (0.393 - 0.691)	0.539 (0.395 - 0.694)	0.542 (0.398 - 0.697)	0.198 (0.081 - 0.389)	0.199 (0.083 - 0.390)	0.201 (0.083 - 0.401)
**15 - 49 years (rate per capita)**	0.456 (0.387 - 0.519)	0.456 (0.392 - 0.516)	0.456 (0.390 - 0.518)	0.815 (0.698 - 0.933)	0.817 (0.707 - 0.930)	0.818 (0.708 - 0.935)	0.270 (0.114 - 0.527)	0.269 (0.114 - 0.529)	0.270 (0.115 - 0.533)
**50 - 69 years (rate per capita)**	0.444 (0.366 - 0.516)	0.443 (0.358 - 0.521)	0.441 (0.357 - 0.521)	0.873 (0.699 - 1.028)	0.869 (0.696 - 1.024)	0.868 (0.692 - 1.031)	0.254 (0.109 - 0.508)	0.254 (0.109 - 0.504)	0.253 (0.109 - 0.510)
**70+ years (rate per capita)**	0.431 (0.360 - 0.500)	0.437 (0.364 - 0.509)	0.438 (0.364 - 0.510)	0.869 (0.725 - 1.004)	0.875 (0.722 - 1.018)	0.875 (0.723 - 1.019)	0.234 (0.105 - 0.449)	0.238 (0.107 - 0.459)	0.238 (0.108 - 0.463)
**All ages (rate per capita)**	0.367 (0.325 - 0.409)	0.392 (0.347 - 0.436)	0.404 (0.360 - 0.448)	0.652 (0.578 - 0.728)	0.700 (0.627 - 0.779)	0.728 (0.653 - 0.807)	0.217 (0.094 - 0.421)	0.232 (0.102 - 0.444)	0.238 (0.104 - 0.467)
**Age-standardized (rate per capita)**	0.391 (0.354 - 0.431)	0.391 (0.354 - 0.430)	0.391 (0.354 - 0.430)	0.710 (0.644 - 0.776)	0.710 (0.644 - 0.776)	0.710 (0.644 - 0.776)	0.229 (0.102 - 0.441)	0.229 (0.101 - 0.438)	0.229 (0.100 - 0.443)
**Total number of cases (thousands)**	183 (162 - 204)	339 (299 - 376)	553 (492 - 612)	326 (289 - 364)	604 (541 - 672)	995 (892 - 1,104)	0 (0 - 0)	0 (0 - 0)	0 (0 - 1)
**Egypt**									
**5 -14 years (rate per capita)**	0.333 (0.272 - 0.399)	0.338 (0.274 - 0.406)	0.330 (0.266 - 0.397)	0.483 (0.383 - 0.605)	0.493 (0.390 - 0.616)	0.484 (0.383 - 0.606)	0.199 (0.084 - 0.399)	0.203 (0.087 - 0.398)	0.198 (0.084 - 0.390)
**15 - 49 years (rate per capita)**	0.451 (0.410 - 0.490)	0.447 (0.408 - 0.487)	0.449 (0.410 - 0.489)	0.796 (0.727 - 0.872)	0.795 (0.723 - 0.868)	0.800 (0.729 - 0.875)	0.267 (0.117 - 0.532)	0.265 (0.115 - 0.523)	0.266 (0.118 - 0.527)
**50 - 69 years (rate per capita)**	0.427 (0.380 - 0.478)	0.425 (0.376 - 0.479)	0.426 (0.377 - 0.480)	0.887 (0.784 - 0.991)	0.885 (0.783 - 0.995)	0.886 (0.786 - 0.994)	0.245 (0.106 - 0.473)	0.244 (0.107 - 0.474)	0.244 (0.105 - 0.473)
**70+ years (rate per capita)**	0.424 (0.379 - 0.469)	0.422 (0.376 - 0.468)	0.419 (0.375 - 0.464)	0.882 (0.780 - 0.986)	0.881 (0.780 - 0.984)	0.877 (0.780 - 0.980)	0.233 (0.105 - 0.449)	0.232 (0.104 - 0.442)	0.229 (0.104 - 0.436)
**All ages (rate per capita)**	0.347 (0.322 - 0.373)	0.369 (0.343 - 0.394)	0.363 (0.339 - 0.388)	0.605 (0.564 - 0.651)	0.652 (0.606 - 0.699)	0.647 (0.603 - 0.693)	0.205 (0.090 - 0.390)	0.218 (0.095 - 0.416)	0.214 (0.094 - 0.411)
**Age-standardized (rate per capita)**	0.385 (0.360 - 0.410)	0.383 (0.359 - 0.407)	0.383 (0.359 - 0.407)	0.696 (0.653 - 0.741)	0.695 (0.651 - 0.742)	0.695 (0.651 - 0.742)	0.226 (0.099 - 0.436)	0.224 (0.097 - 0.430)	0.224 (0.098 - 0.430)
**Total number of cases (thousands)**	19,554 (18,123 - 20,992)	27,613 (25,675 - 29,490)	33,131 (30,900 - 35,388)	34,071 (31,753 - 36,637)	48,763 (45,316 - 52,319)	58,945 (54,943 - 63,198)	12 (5 - 22)	16 (7 - 31)	20 (9 - 37)
**Iran**									
**5 -14 years (rate per capita)**	0.387 (0.323 - 0.447)	0.392 (0.334 - 0.455)	0.390 (0.326 - 0.450)	0.501 (0.396 - 0.610)	0.471 (0.378 - 0.572)	0.503 (0.398 - 0.612)	0.231 (0.099 - 0.447)	0.235 (0.099 - 0.459)	0.234 (0.100 - 0.454)
**15 - 49 years (rate per capita)**	0.504 (0.466 - 0.542)	0.504 (0.464 - 0.543)	0.505 (0.468 - 0.544)	0.771 (0.706 - 0.843)	0.772 (0.709 - 0.844)	0.780 (0.717 - 0.852)	0.298 (0.132 - 0.591)	0.299 (0.132 - 0.588)	0.299 (0.133 - 0.592)
**50 - 69 years (rate per capita)**	0.486 (0.439 - 0.535)	0.484 (0.436 - 0.532)	0.485 (0.436 - 0.533)	0.865 (0.774 - 0.956)	0.862 (0.770 - 0.958)	0.862 (0.768 - 0.953)	0.280 (0.120 - 0.540)	0.279 (0.118 - 0.541)	0.279 (0.119 - 0.545)
**70+ years (rate per capita)**	0.480 (0.438 - 0.525)	0.478 (0.436 - 0.518)	0.470 (0.432 - 0.509)	0.866 (0.772 - 0.956)	0.864 (0.768 - 0.949)	0.859 (0.771 - 0.940)	0.265 (0.117 - 0.509)	0.263 (0.118 - 0.510)	0.258 (0.114 - 0.493)
**All ages (rate per capita)**	0.386 (0.360 - 0.411)	0.438 (0.411 - 0.465)	0.441 (0.414 - 0.464)	0.577 (0.534 - 0.622)	0.664 (0.620 - 0.712)	0.686 (0.643 - 0.734)	0.228 (0.099 - 0.443)	0.259 (0.115 - 0.504)	0.260 (0.115 - 0.504)
**Age-standardized (rate per capita)**	0.435 (0.411 - 0.458)	0.431 (0.409 - 0.452)	0.435 (0.411 - 0.458)	0.683 (0.643 - 0.726)	0.672 (0.632 - 0.712)	0.683 (0.643 - 0.726)	0.255 (0.112 - 0.494)	0.253 (0.111 - 0.489)	0.256 (0.111 - 0.493)
**Total number of cases (thousands)**	21,588 (20,141 - 23,005)	30,933 (29,001 - 32,834)	34,824 (32,743 - 36,668)	32,292 (29,906 - 34,792)	46,882 (43,758 - 50,248)	54,188 (50,797 - 57,981)	13 (6 - 25)	18 (8 - 36)	21 (9 - 40)
**Iraq**									
**5 -14 years (rate per capita)**	0.408 (0.350 - 0.467)	0.406 (0.348 - 0.465)	0.404 (0.347 - 0.463)	0.598 (0.494 - 0.710)	0.596 (0.491 - 0.707)	0.594 (0.489 - 0.705)	0.243 (0.104 - 0.462)	0.243 (0.103 - 0.469)	0.242 (0.104 - 0.466)
**15 - 49 years (rate per capita)**	0.522 (0.488 - 0.557)	0.522 (0.490 - 0.557)	0.522 (0.492 - 0.556)	0.925 (0.858 - 0.996)	0.927 (0.861 - 0.996)	0.929 (0.866 - 0.996)	0.306 (0.133 - 0.595)	0.307 (0.135 - 0.602)	0.308 (0.135 - 0.602)
**50 - 69 years (rate per capita)**	0.494 (0.456 - 0.533)	0.494 (0.455 - 0.533)	0.494 (0.455 - 0.533)	1.030 (0.951 - 1.111)	1.029 (0.949 - 1.108)	1.030 (0.950 - 1.111)	0.280 (0.122 - 0.539)	0.278 (0.122 - 0.538)	0.278 (0.122 - 0.536)
**70+ years (rate per capita)**	0.485 (0.450 - 0.522)	0.483 (0.449 - 0.520)	0.484 (0.450 - 0.522)	1.021 (0.948 - 1.091)	1.019 (0.947 - 1.089)	1.020 (0.948 - 1.091)	0.263 (0.118 - 0.497)	0.261 (0.115 - 0.490)	0.261 (0.117 - 0.496)
**All ages (rate per capita)**	0.397 (0.371 - 0.419)	0.406 (0.381 - 0.428)	0.407 (0.383 - 0.429)	0.683 (0.639 - 0.729)	0.703 (0.659 - 0.749)	0.708 (0.665 - 0.753)	0.232 (0.102 - 0.443)	0.238 (0.105 - 0.459)	0.240 (0.105 - 0.460)
**Age-standardized (rate per capita)**	0.449 (0.428 - 0.469)	0.449 (0.428 - 0.469)	0.449 (0.428 - 0.469)	0.816 (0.776 - 0.856)	0.816 (0.776 - 0.856)	0.816 (0.776 - 0.856)	0.260 (0.114 - 0.495)	0.260 (0.114 - 0.499)	0.261 (0.115 - 0.501)
**Total number of cases (thousands)**	6,925 (6,476 - 7,318)	10,986 (10,311 - 11,585)	14,839 (13,943 - 15,638)	11,930 (11,162 - 12,736)	19,019 (17,830 - 20,284)	25,781 (24,222 - 27,424)	4 (2 - 8)	6 (3 - 12)	9 (4 - 17)
**Jordan**									
**5 -14 years (rate per capita)**	0.332 (0.253 - 0.424)	0.337 (0.256 - 0.429)	0.337 (0.257 - 0.429)	0.540 (0.396 - 0.695)	0.544 (0.401 - 0.701)	0.545 (0.401 - 0.702)	0.199 (0.082 - 0.400)	0.202 (0.084 - 0.400)	0.202 (0.083 - 0.396)
**15 - 49 years (rate per capita)**	0.453 (0.388 - 0.518)	0.455 (0.391 - 0.513)	0.456 (0.393 - 0.513)	0.805 (0.686 - 0.924)	0.809 (0.695 - 0.927)	0.811 (0.700 - 0.923)	0.268 (0.114 - 0.524)	0.269 (0.115 - 0.532)	0.270 (0.116 - 0.533)
**50 - 69 years (rate per capita)**	0.445 (0.368 - 0.517)	0.447 (0.370 - 0.518)	0.444 (0.364 - 0.517)	0.874 (0.706 - 1.027)	0.877 (0.714 - 1.027)	0.872 (0.700 - 1.027)	0.255 (0.109 - 0.506)	0.255 (0.109 - 0.509)	0.255 (0.109 - 0.505)
**70+ years (rate per capita)**	0.430 (0.360 - 0.499)	0.439 (0.364 - 0.512)	0.437 (0.364 - 0.509)	0.868 (0.723 - 1.002)	0.877 (0.721 - 1.022)	0.875 (0.722 - 1.018)	0.234 (0.105 - 0.452)	0.240 (0.110 - 0.466)	0.239 (0.107 - 0.469)
**All ages (rate per capita)**	0.343 (0.305 - 0.382)	0.362 (0.322 - 0.400)	0.370 (0.330 - 0.409)	0.604 (0.530 - 0.673)	0.639 (0.568 - 0.707)	0.655 (0.587 - 0.722)	0.203 (0.088 - 0.392)	0.214 (0.094 - 0.413)	0.218 (0.094 - 0.422)
**Age-standardized (rate per capita)**	0.392 (0.355 - 0.431)	0.392 (0.355 - 0.431)	0.392 (0.355 - 0.431)	0.709 (0.644 - 0.774)	0.709 (0.644 - 0.775)	0.709 (0.644 - 0.774)	0.229 (0.101 - 0.441)	0.229 (0.102 - 0.440)	0.229 (0.102 - 0.443)
**Total number of cases (thousands)**	1,144 (1,017 - 1,274)	1,937 (1,725 - 2,144)	2,800 (2,498 - 3,099)	2,012 (1,766 - 2,244)	3,421 (3,040 - 3,787)	4,962 (4,443 - 5,464)	1 (0 - 1)	1 (1 - 2)	2 (1 - 3)
**Kuwait**									
**5 -14 years (rate per capita)**	0.332 (0.252 - 0.424)	0.332 (0.252 - 0.423)	0.324 (0.245 - 0.415)	0.561 (0.410 - 0.730)	0.561 (0.410 - 0.730)	0.550 (0.398 - 0.720)	0.332 (0.142 - 0.665)	0.332 (0.143 - 0.647)	0.324 (0.139 - 0.643)
**15 - 49 years (rate per capita)**	0.457 (0.394 - 0.516)	0.456 (0.390 - 0.517)	0.458 (0.391 - 0.519)	0.854 (0.735 - 0.965)	0.860 (0.740 - 0.975)	0.862 (0.743 - 0.984)	0.451 (0.197 - 0.874)	0.451 (0.195 - 0.868)	0.453 (0.195 - 0.868)
**50 - 69 years (rate per capita)**	0.442 (0.357 - 0.518)	0.443 (0.360 - 0.517)	0.442 (0.358 - 0.520)	0.913 (0.730 - 1.083)	0.915 (0.733 - 1.081)	0.913 (0.728 - 1.080)	0.426 (0.182 - 0.797)	0.425 (0.181 - 0.798)	0.425 (0.182 - 0.796)
**70+ years (rate per capita)**	0.429 (0.360 - 0.496)	0.444 (0.364 - 0.521)	0.437 (0.364 - 0.509)	0.910 (0.758 - 1.054)	0.928 (0.750 - 1.095)	0.919 (0.755 - 1.070)	0.394 (0.176 - 0.765)	0.408 (0.182 - 0.799)	0.400 (0.179 - 0.774)
**All ages (rate per capita)**	0.374 (0.333 - 0.415)	0.388 (0.343 - 0.431)	0.396 (0.351 - 0.441)	0.694 (0.615 - 0.766)	0.728 (0.646 - 0.809)	0.747 (0.665 - 0.830)	0.370 (0.163 - 0.707)	0.383 (0.169 - 0.732)	0.391 (0.171 - 0.754)
**Age-standardized (rate per capita)**	0.392 (0.355 - 0.431)	0.391 (0.354 - 0.431)	0.391 (0.355 - 0.431)	0.745 (0.674 - 0.814)	0.746 (0.673 - 0.815)	0.745 (0.673 - 0.813)	0.383 (0.170 - 0.730)	0.382 (0.172 - 0.728)	0.383 (0.171 - 0.729)
**Total number of cases (thousands)**	769 (683 - 853)	883 (780 - 980)	1,546 (1,370 - 1,721)	1,425 (1,263 - 1,573)	1,656 (1,470 - 1,840)	2,913 (2,593 - 3,239)	1 (0 - 1)	1 (0 - 2)	2 (1 - 3)
**Lebanon**									
**5 -14 years (rate per capita)**	0.341 (0.260 - 0.432)	0.350 (0.267 - 0.441)	0.353 (0.270 - 0.444)	0.550 (0.403 - 0.707)	0.560 (0.409 - 0.719)	0.563 (0.410 - 0.722)	0.201 (0.083 - 0.393)	0.210 (0.088 - 0.412)	0.211 (0.089 - 0.414)
**15 - 49 years (rate per capita)**	0.455 (0.391 - 0.511)	0.456 (0.394 - 0.512)	0.456 (0.392 - 0.512)	0.809 (0.696 - 0.923)	0.811 (0.702 - 0.920)	0.812 (0.699 - 0.924)	0.263 (0.113 - 0.514)	0.265 (0.113 - 0.523)	0.268 (0.116 - 0.529)
**50 - 69 years (rate per capita)**	0.447 (0.370 - 0.518)	0.447 (0.370 - 0.518)	0.446 (0.368 - 0.517)	0.876 (0.713 - 1.027)	0.877 (0.715 - 1.027)	0.875 (0.708 - 1.029)	0.251 (0.108 - 0.499)	0.251 (0.107 - 0.497)	0.250 (0.108 - 0.496)
**70+ years (rate per capita)**	0.433 (0.362 - 0.504)	0.437 (0.363 - 0.508)	0.431 (0.361 - 0.501)	0.871 (0.723 - 1.009)	0.875 (0.722 - 1.018)	0.870 (0.723 - 1.006)	0.232 (0.105 - 0.439)	0.236 (0.106 - 0.464)	0.232 (0.104 - 0.448)
**All ages (rate per capita)**	0.371 (0.333 - 0.410)	0.400 (0.360 - 0.442)	0.406 (0.366 - 0.446)	0.662 (0.595 - 0.727)	0.715 (0.646 - 0.782)	0.732 (0.660 - 0.801)	0.214 (0.095 - 0.411)	0.232 (0.102 - 0.447)	0.237 (0.104 - 0.457)
**Age-standardized (rate per capita)**	0.392 (0.356 - 0.431)	0.392 (0.355 - 0.431)	0.392 (0.356 - 0.431)	0.709 (0.644 - 0.774)	0.709 (0.644 - 0.775)	0.709 (0.644 - 0.775)	0.225 (0.099 - 0.430)	0.226 (0.100 - 0.435)	0.227 (0.100 - 0.438)
**Total number of cases (thousands)**	1,014 (908 - 1,119)	1,578 (1,420 - 1,743)	2,342 (2,107 - 2,572)	1,808 (1,627 - 1,986)	2,822 (2,548 - 3,089)	4,216 (3,803 - 4,617)	1 (0 - 1)	1 (0 - 2)	1 (1 - 3)
**Libya**									
**5 -14 years (rate per capita)**	0.340 (0.259 - 0.431)	0.345 (0.262 - 0.437)	0.335 (0.255 - 0.427)	0.548 (0.403 - 0.706)	0.554 (0.407 - 0.713)	0.542 (0.399 - 0.699)	0.204 (0.084 - 0.399)	0.207 (0.087 - 0.410)	0.201 (0.084 - 0.397)
**15 - 49 years (rate per capita)**	0.454 (0.391 - 0.514)	0.455 (0.391 - 0.513)	0.457 (0.396 - 0.515)	0.807 (0.690 - 0.923)	0.811 (0.697 - 0.926)	0.814 (0.707 - 0.924)	0.269 (0.114 - 0.529)	0.270 (0.115 - 0.535)	0.270 (0.116 - 0.533)
**50 - 69 years (rate per capita)**	0.445 (0.368 - 0.517)	0.445 (0.367 - 0.517)	0.445 (0.366 - 0.516)	0.875 (0.707 - 1.028)	0.874 (0.707 - 1.027)	0.872 (0.701 - 1.026)	0.256 (0.110 - 0.508)	0.256 (0.109 - 0.503)	0.256 (0.111 - 0.503)
**70+ years (rate per capita)**	0.441 (0.365 - 0.516)	0.438 (0.365 - 0.510)	0.435 (0.363 - 0.505)	0.878 (0.720 - 1.025)	0.875 (0.722 - 1.019)	0.873 (0.723 - 1.013)	0.243 (0.110 - 0.471)	0.241 (0.108 - 0.472)	0.238 (0.107 - 0.456)
**All ages (rate per capita)**	0.357 (0.318 - 0.395)	0.386 (0.344 - 0.426)	0.383 (0.344 - 0.423)	0.628 (0.555 - 0.695)	0.685 (0.613 - 0.757)	0.684 (0.615 - 0.752)	0.211 (0.093 - 0.407)	0.228 (0.099 - 0.442)	0.226 (0.099 - 0.437)
**Age-standardized (rate per capita)**	0.392 (0.355 - 0.431)	0.392 (0.355 - 0.431)	0.392 (0.355 - 0.431)	0.709 (0.644 - 0.775)	0.709 (0.644 - 0.775)	0.709 (0.644 - 0.774)	0.230 (0.101 - 0.443)	0.230 (0.101 - 0.443)	0.230 (0.102 - 0.444)
**Total number of cases (thousands)**	1,567 (1,397 - 1,736)	2,241 (1,998 - 2,475)	2,411 (2,163 - 2,662)	2,756 (2,436 - 3,050)	3,981 (3,562 - 4,400)	4,305 (3,872 - 4,732)	1 (0 - 2)	1 (1 - 3)	1 (1 - 3)
**Morocco**									
**5 -14 years (rate per capita)**	0.336 (0.255 - 0.428)	0.348 (0.265 - 0.440)	0.338 (0.257 - 0.429)	0.543 (0.400 - 0.699)	0.558 (0.408 - 0.717)	0.546 (0.402 - 0.703)	0.201 (0.084 - 0.395)	0.208 (0.086 - 0.404)	0.203 (0.085 - 0.396)
**15 - 49 years (rate per capita)**	0.454 (0.389 - 0.514)	0.456 (0.393 - 0.512)	0.456 (0.393 - 0.512)	0.808 (0.691 - 0.927)	0.811 (0.701 - 0.921)	0.812 (0.701 - 0.924)	0.269 (0.113 - 0.527)	0.269 (0.115 - 0.531)	0.270 (0.115 - 0.535)

	Prevalence 1990	Prevalence 2005	Prevalence 2015	Incidence 1990	Incidence 2005	Incidence 2015	YLD rate 1990	YLD rate 2005	YLD rate 2015
50 - 69 years (rate per capita)	0.447 (0.370 - 0.518)	0.446 (0.367 - 0.517)	0.445 (0.367 - 0.518)	0.876 (0.713 - 1.029)	0.874 (0.708 - 1.025)	0.874 (0.703 - 1.030)	0.255 (0.110 - 0.503)	0.255 (0.109 - 0.501)	0.255 (0.110 - 0.508)
70+ years (rate per capita)	0.439 (0.365 - 0.513)	0.440 (0.366 - 0.512)	0.432 (0.362 - 0.501)	0.876 (0.719 - 1.023)	0.876 (0.721 - 1.022)	0.870 (0.722 - 1.007)	0.240 (0.107 - 0.465)	0.240 (0.108 - 0.467)	0.235 (0.106 - 0.457)
All ages (rate per capita)	0.359 (0.320 - 0.397)	0.387 (0.347 - 0.428)	0.388 (0.349 - 0.426)	0.635 (0.565 - 0.702)	0.688 (0.618 - 0.755)	0.698 (0.629 - 0.764)	0.211 (0.092 - 0.407)	0.228 (0.100 - 0.440)	0.227 (0.101 - 0.440)
Age-standardized (rate per capita)	0.392 (0.355 - 0.431)	0.392 (0.356 - 0.431)	0.392 (0.356 - 0.431)	0.709 (0.644 - 0.774)	0.709 (0.644 - 0.774)	0.709 (0.644 - 0.774)	0.229 (0.101 - 0.441)	0.229 (0.101 - 0.440)	0.229 (0.102 - 0.442)
Total number of cases (thousands)	8,983 (8,002 - 9,940)	11,784 (10,557 - 13,025)	13,324 (11,984 - 14,660)	15,886 (14,152 - 17,573)	20,966 (18,834 - 22,998)	23,978 (21,633 - 26,268)	5 (2 - 10)	7 (3 - 13)	8 (3 - 15)
**Palestine**									
5 -14 years (rate per capita)	0.332 (0.252 - 0.424)	0.337 (0.257 - 0.429)	0.336 (0.255 - 0.428)	0.539 (0.395 - 0.694)	0.545 (0.401 - 0.701)	0.544 (0.401 - 0.700)	0.198 (0.083 - 0.390)	0.202 (0.084 - 0.400)	0.202 (0.084 - 0.396)
15 - 49 years (rate per capita)	0.454 (0.388 - 0.517)	0.455 (0.391 - 0.515)	0.454 (0.392 - 0.514)	0.807 (0.691 - 0.921)	0.807 (0.692 - 0.923)	0.808 (0.692 - 0.923)	0.267 (0.114 - 0.527)	0.268 (0.113 - 0.528)	0.269 (0.114 - 0.536)
50 - 69 years (rate per capita)	0.445 (0.366 - 0.516)	0.446 (0.368 - 0.517)	0.445 (0.366 - 0.516)	0.872 (0.700 - 1.027)	0.874 (0.707 - 1.027)	0.872 (0.703 - 1.026)	0.255 (0.109 - 0.503)	0.254 (0.108 - 0.502)	0.254 (0.109 - 0.499)
70+ years (rate per capita)	0.438 (0.365 - 0.511)	0.440 (0.365 - 0.515)	0.440 (0.366 - 0.512)	0.876 (0.720 - 1.021)	0.877 (0.720 - 1.024)	0.877 (0.722 - 1.023)	0.241 (0.108 - 0.474)	0.242 (0.109 - 0.476)	0.241 (0.109 - 0.462)
All ages (rate per capita)	0.327 (0.291 - 0.364)	0.344 (0.306 - 0.382)	0.355 (0.316 - 0.393)	0.573 (0.502 - 0.638)	0.602 (0.529 - 0.668)	0.625 (0.554 - 0.692)	0.193 (0.084 - 0.370)	0.203 (0.088 - 0.391)	0.210 (0.091 - 0.405)
Age-standardized (rate per capita)	0.392 (0.355 - 0.431)	0.392 (0.355 - 0.431)	0.392 (0.355 - 0.431)	0.709 (0.644 - 0.774)	0.709 (0.644 - 0.774)	0.709 (0.644 - 0.774)	0.228 (0.101 - 0.438)	0.229 (0.101 - 0.441)	0.229 (0.101 - 0.441)
Total number of cases (thousands)	691 (614 - 769)	1,238 (1,103 - 1,376)	1,658 (1,476 - 1,835)	1,210 (1,061 - 1,347)	2,167 (1,906 - 2,406)	2,922 (2,588 - 3,235)	0 (0 - 1)	1 (0 - 1)	1 (0 - 2)
**Oman**									
5 -14 years (rate per capita)	0.327 (0.249 - 0.419)	0.345 (0.263 - 0.437)	0.324 (0.247 - 0.416)	0.533 (0.390 - 0.688)	0.555 (0.408 - 0.714)	0.529 (0.386 - 0.684)	0.196 (0.081 - 0.385)	0.207 (0.085 - 0.403)	0.195 (0.081 - 0.380)
15 - 49 years (rate per capita)	0.455 (0.391 - 0.514)	0.455 (0.391 - 0.512)	0.455 (0.386 - 0.519)	0.812 (0.702 - 0.924)	0.811 (0.699 - 0.924)	0.819 (0.699 - 0.939)	0.270 (0.114 - 0.537)	0.270 (0.114 - 0.537)	0.270 (0.117 - 0.535)
50 - 69 years (rate per capita)	0.444 (0.364 - 0.517)	0.444 (0.362 - 0.516)	0.443 (0.365 - 0.516)	0.871 (0.697 - 1.026)	0.872 (0.699 - 1.027)	0.873 (0.697 - 1.027)	0.255 (0.109 - 0.505)	0.255 (0.110 - 0.508)	0.254 (0.108 - 0.506)
70+ years (rate per capita)	0.440 (0.366 - 0.512)	0.432 (0.362 - 0.501)	0.436 (0.362 - 0.506)	0.876 (0.722 - 1.023)	0.871 (0.722 - 1.008)	0.874 (0.723 - 1.018)	0.240 (0.108 - 0.474)	0.235 (0.106 - 0.455)	0.236 (0.106 - 0.462)
All ages (rate per capita)	0.336 (0.298 - 0.372)	0.380 (0.338 - 0.421)	0.400 (0.351 - 0.445)	0.591 (0.524 - 0.656)	0.671 (0.596 - 0.742)	0.721 (0.638 - 0.808)	0.199 (0.086 - 0.382)	0.225 (0.098 - 0.436)	0.236 (0.103 - 0.461)
Age-standardized (rate per capita)	0.391 (0.354 - 0.430)	0.391 (0.355 - 0.431)	0.391 (0.354 - 0.430)	0.710 (0.644 - 0.775)	0.710 (0.644 - 0.775)	0.710 (0.644 - 0.777)	0.229 (0.102 - 0.442)	0.229 (0.101 - 0.441)	0.229 (0.101 - 0.443)
Total number of cases (thousands)	612 (545 - 679)	952 (848 - 1,054)	1,792 (1,571 - 1,995)	1,079 (957 - 1,196)	1,680 (1,493 - 1,860)	3,230 (2,860 - 3,622)	0 (0 - 1)	1 (0 - 1)	1 (0 - 2)
**Qatar**									
5 -14 years (rate per capita)	0.321 (0.242 - 0.412)	0.328 (0.250 - 0.420)	0.336 (0.256 - 0.428)	0.525 (0.381 - 0.681)	0.534 (0.391 - 0.689)	0.545 (0.401 - 0.702)	0.193 (0.080 - 0.380)	0.197 (0.082 - 0.391)	0.202 (0.084 - 0.393)
15 - 49 years (rate per capita)	0.457 (0.389 - 0.519)	0.456 (0.390 - 0.519)	0.455 (0.386 - 0.520)	0.818 (0.706 - 0.939)	0.820 (0.711 - 0.938)	0.821 (0.704 - 0.946)	0.271 (0.116 - 0.536)	0.270 (0.115 - 0.533)	0.270 (0.117 - 0.536)
50 - 69 years (rate per capita)	0.440 (0.353 - 0.521)	0.439 (0.349 - 0.523)	0.440 (0.352 - 0.519)	0.868 (0.696 - 1.033)	0.867 (0.687 - 1.034)	0.868 (0.692 - 1.035)	0.253 (0.109 - 0.502)	0.252 (0.107 - 0.516)	0.253 (0.109 - 0.510)
70+ years (rate per capita)	0.426 (0.358 - 0.494)	0.434 (0.361 - 0.503)	0.443 (0.364 - 0.520)	0.866 (0.724 - 0.997)	0.873 (0.722 - 1.016)	0.882 (0.720 - 1.039)	0.232 (0.104 - 0.447)	0.236 (0.106 - 0.459)	0.241 (0.109 - 0.472)
All ages (rate per capita)	0.382 (0.335 - 0.428)	0.400 (0.354 - 0.444)	0.418 (0.365 - 0.468)	0.682 (0.604 - 0.763)	0.718 (0.644 - 0.801)	0.755 (0.665 - 0.850)	0.226 (0.098 - 0.439)	0.236 (0.103 - 0.457)	0.248 (0.109 - 0.485)
Age-standardized (rate per capita)	0.390 (0.353 - 0.430)	0.391 (0.354 - 0.430)	0.390 (0.353 - 0.429)	0.710 (0.645 - 0.777)	0.710 (0.645 - 0.777)	0.710 (0.645 - 0.777)	0.229 (0.101 - 0.443)	0.229 (0.101 - 0.444)	0.229 (0.100 - 0.439)
Total number of cases (thousands)	182 (160 - 204)	332 (294 - 368)	929 (810 - 1,040)	325 (288 - 364)	596 (534 - 664)	1,676 (1,476 - 1,887)	0 (0 - 0)	0 (0 - 0)	1 (0 - 1)
**Saudi Arabia**									
5 -14 years (rate per capita)	0.299 (0.251 - 0.349)	0.301 (0.245 - 0.358)	0.310 (0.254 - 0.367)	0.396 (0.318 - 0.485)	0.423 (0.331 - 0.536)	0.426 (0.334 - 0.535)	0.179 (0.076 - 0.349)	0.180 (0.076 - 0.349)	0.185 (0.079 - 0.361)
15 - 49 years (rate per capita)	0.412 (0.379 - 0.446)	0.410 (0.377 - 0.444)	0.407 (0.374 - 0.440)	0.665 (0.601 - 0.734)	0.664 (0.603 - 0.730)	0.664 (0.602 - 0.726)	0.245 (0.107 - 0.484)	0.244 (0.107 - 0.485)	0.241 (0.104 - 0.484)
50 - 69 years (rate per capita)	0.393 (0.356 - 0.439)	0.388 (0.346 - 0.435)	0.380 (0.336 - 0.428)	0.712 (0.627 - 0.804)	0.699 (0.617 - 0.791)	0.693 (0.603 - 0.793)	0.226 (0.097 - 0.439)	0.224 (0.097 - 0.438)	0.220 (0.094 - 0.426)
70+ years (rate per capita)	0.391 (0.355 - 0.431)	0.383 (0.350 - 0.418)	0.377 (0.344 - 0.414)	0.706 (0.628 - 0.792)	0.691 (0.615 - 0.775)	0.688 (0.611 - 0.769)	0.216 (0.097 - 0.418)	0.212 (0.096 - 0.402)	0.208 (0.093 - 0.397)
All ages (rate per capita)	0.314 (0.292 - 0.335)	0.337 (0.314 - 0.360)	0.346 (0.323 - 0.367)	0.491 (0.452 - 0.534)	0.538 (0.496 - 0.584)	0.559 (0.518 - 0.602)	0.186 (0.081 - 0.359)	0.200 (0.088 - 0.387)	0.205 (0.089 - 0.391)
Age-standardized (rate per capita)	0.353 (0.333 - 0.373)	0.348 (0.328 - 0.368)	0.347 (0.326 - 0.367)	0.573 (0.535 - 0.611)	0.571 (0.531 - 0.611)	0.571 (0.532 - 0.611)	0.207 (0.090 - 0.401)	0.205 (0.089 - 0.396)	0.204 (0.088 - 0.392)
Total number of cases (thousands)	5,124 (4,762 - 5,462)	8,354 (7,779 - 8,907)	10,864 (10,146 - 11,526)	8,007 (7,381 - 8,711)	13,333 (12,274 - 14,457)	17,565 (16,281 - 18,919)	3 (1 - 6)	5 (2 - 10)	6 (3 - 12)
**Sudan**									
5 -14 years (rate per capita)	0.334 (0.254 - 0.426)	0.334 (0.253 - 0.425)	0.337 (0.257 - 0.429)	0.541 (0.397 - 0.696)	0.541 (0.397 - 0.696)	0.545 (0.401 - 0.701)	0.199 (0.083 - 0.397)	0.199 (0.083 - 0.391)	0.201 (0.085 - 0.393)
15 - 49 years (rate per capita)	0.454 (0.392 - 0.514)	0.455 (0.391 - 0.514)	0.455 (0.392 - 0.513)	0.807 (0.691 - 0.923)	0.808 (0.694 - 0.923)	0.809 (0.695 - 0.922)	0.267 (0.114 - 0.524)	0.268 (0.114 - 0.524)	0.268 (0.115 - 0.530)
50 - 69 years (rate per capita)	0.446 (0.367 - 0.517)	0.445 (0.367 - 0.517)	0.445 (0.367 - 0.517)	0.874 (0.706 - 1.026)	0.874 (0.705 - 1.026)	0.874 (0.705 - 1.026)	0.255 (0.110 - 0.508)	0.254 (0.110 - 0.502)	0.255 (0.110 - 0.507)
70+ years (rate per capita)	0.439 (0.365 - 0.512)	0.438 (0.365 - 0.509)	0.437 (0.364 - 0.508)	0.876 (0.720 - 1.022)	0.875 (0.721 - 1.020)	0.875 (0.722 - 1.017)	0.240 (0.108 - 0.463)	0.238 (0.108 - 0.464)	0.238 (0.107 - 0.463)
All ages (rate per capita)	0.340 (0.304 - 0.378)	0.345 (0.306 - 0.381)	0.355 (0.316 - 0.392)	0.598 (0.527 - 0.663)	0.607 (0.538 - 0.672)	0.626 (0.557 - 0.691)	0.200 (0.088 - 0.384)	0.203 (0.089 - 0.392)	0.209 (0.092 - 0.401)
Age-standardized (rate per capita)	0.392 (0.355 - 0.431)	0.392 (0.355 - 0.431)	0.392 (0.355 - 0.431)	0.709 (0.644 - 0.774)	0.709 (0.644 - 0.774)	0.709 (0.644 - 0.774)	0.228 (0.100 - 0.443)	0.228 (0.101 - 0.440)	0.228 (0.102 - 0.441)
Total number of cases (thousands)	6,808 (6,071 - 7,551)	11,063 (9,834 - 12,239)	14,333 (12,779 - 15,833)	11,960 (10,547 - 13,258)	19,483 (17,271 - 21,558)	25,275 (22,497 - 27,915)	4 (2 - 8)	7 (3 - 13)	8 (4 - 16)
**Syria**									
5 -14 years (rate per capita)	0.334 (0.254 - 0.426)	0.337 (0.256 - 0.429)	0.343 (0.261 - 0.435)	0.541 (0.398 - 0.697)	0.545 (0.401 - 0.701)	0.552 (0.404 - 0.710)	0.200 (0.083 - 0.390)	0.202 (0.085 - 0.390)	0.205 (0.086 - 0.406)
15 - 49 years (rate per capita)	0.454 (0.389 - 0.518)	0.454 (0.391 - 0.514)	0.455 (0.392 - 0.513)	0.806 (0.689 - 0.925)	0.808 (0.692 - 0.924)	0.809 (0.695 - 0.918)	0.269 (0.114 - 0.527)	0.270 (0.115 - 0.535)	0.269 (0.115 - 0.525)
50 - 69 years (rate per capita)	0.446 (0.370 - 0.518)	0.445 (0.366 - 0.516)	0.445 (0.366 - 0.517)	0.877 (0.712 - 1.029)	0.873 (0.704 - 1.026)	0.873 (0.704 - 1.027)	0.258 (0.109 - 0.512)	0.257 (0.110 - 0.516)	0.257 (0.111 - 0.507)
70+ years (rate per capita)	0.438 (0.365 - 0.510)	0.437 (0.364 - 0.509)	0.433 (0.362 - 0.501)	0.876 (0.721 - 1.021)	0.875 (0.723 - 1.020)	0.871 (0.723 - 1.008)	0.243 (0.109 - 0.469)	0.242 (0.108 - 0.471)	0.238 (0.107 - 0.459)
All ages (rate per capita)	0.340 (0.302 - 0.378)	0.362 (0.322 - 0.401)	0.369 (0.330 - 0.408)	0.596 (0.523 - 0.663)	0.638 (0.566 - 0.706)	0.652 (0.583 - 0.719)	0.201 (0.087 - 0.387)	0.214 (0.094 - 0.413)	0.218 (0.095 - 0.420)
Age-standardized (rate per capita)	0.392 (0.355 - 0.431)	0.392 (0.355 - 0.431)	0.392 (0.355 - 0.431)	0.709 (0.644 - 0.775)	0.709 (0.644 - 0.775)	0.709 (0.644 - 0.774)	0.230 (0.101 - 0.444)	0.230 (0.101 - 0.446)	0.229 (0.102 - 0.441)
Total number of cases (thousands)	4,229 (3,755 - 4,703)	6,575 (5,857 - 7,279)	6,869 (6,140 - 7,592)	7,410 (6,504 - 8,253)	11,598 (10,282 - 12,832)	12,146 (10,850 - 13,393)	3 (1 - 5)	4 (2 - 8)	4 (2 - 8)
**Tunisia**									
5 -14 years (rate per capita)	0.354 (0.290 - 0.424)	0.382 (0.309 - 0.449)	0.365 (0.296 - 0.431)	0.498 (0.393 - 0.618)	0.517 (0.410 - 0.630)	0.502 (0.393 - 0.615)	0.212 (0.090 - 0.423)	0.229 (0.097 - 0.454)	0.219 (0.093 - 0.433)
15 - 49 years (rate per capita)	0.471 (0.428 - 0.514)	0.471 (0.429 - 0.512)	0.472 (0.431 - 0.514)	0.799 (0.728 - 0.876)	0.803 (0.735 - 0.874)	0.808 (0.741 - 0.881)	0.279 (0.124 - 0.555)	0.280 (0.122 - 0.561)	0.280 (0.122 - 0.561)
50 - 69 years (rate per capita)	0.448 (0.397 - 0.503)	0.447 (0.396 - 0.501)	0.446 (0.393 - 0.502)	0.894 (0.794 - 0.996)	0.892 (0.794 - 0.994)	0.892 (0.791 - 0.996)	0.258 (0.112 - 0.503)	0.258 (0.111 - 0.505)	0.257 (0.112 - 0.503)
70+ years (rate per capita)	0.447 (0.397 - 0.498)	0.443 (0.394 - 0.490)	0.434 (0.391 - 0.479)	0.894 (0.795 - 0.995)	0.891 (0.796 - 0.989)	0.881 (0.791 - 0.977)	0.247 (0.112 - 0.467)	0.245 (0.110 - 0.463)	0.239 (0.109 - 0.455)
All ages (rate per capita)	0.378 (0.351 - 0.405)	0.413 (0.384 - 0.440)	0.409 (0.382 - 0.435)	0.636 (0.593 - 0.684)	0.704 (0.661 - 0.752)	0.711 (0.669 - 0.757)	0.223 (0.098 - 0.431)	0.244 (0.105 - 0.470)	0.241 (0.105 - 0.465)
Age-standardized (rate per capita)	0.403 (0.377 - 0.429)	0.405 (0.379 - 0.430)	0.405 (0.379 - 0.430)	0.701 (0.659 - 0.746)	0.702 (0.662 - 0.746)	0.702 (0.662 - 0.746)	0.237 (0.103 - 0.455)	0.238 (0.103 - 0.459)	0.238 (0.103 - 0.457)
Total number of cases (thousands)	3,113 (2,886 - 3,334)	4,180 (3,884 - 4,452)	4,603 (4,292 - 4,892)	5,240 (4,885 - 5,633)	7,125 (6,690 - 7,609)	8,003 (7,526 - 8,519)	2 (1 - 4)	2 (1 - 5)	3 (1 - 5)
**Turkey**									
5 -14 years (rate per capita)	0.372 (0.310 - 0.437)	0.372 (0.309 - 0.440)	0.376 (0.314 - 0.443)	0.509 (0.398 - 0.623)	0.509 (0.405 - 0.624)	0.514 (0.402 - 0.627)	0.222 (0.094 - 0.441)	0.223 (0.095 - 0.438)	0.226 (0.097 - 0.441)
15 - 49 years (rate per capita)	0.486 (0.446 - 0.527)	0.488 (0.449 - 0.527)	0.487 (0.448 - 0.526)	0.803 (0.735 - 0.874)	0.807 (0.741 - 0.877)	0.809 (0.742 - 0.877)	0.288 (0.125 - 0.573)	0.290 (0.124 - 0.571)	0.289 (0.125 - 0.576)
50 - 69 years (rate per capita)	0.462 (0.414 - 0.512)	0.465 (0.417 - 0.516)	0.463 (0.416 - 0.513)	0.898 (0.802 - 0.998)	0.898 (0.803 - 0.992)	0.898 (0.804 - 0.997)	0.265 (0.114 - 0.519)	0.268 (0.116 - 0.522)	0.267 (0.116 - 0.522)
70+ years (rate per capita)	0.449 (0.408 - 0.490)	0.461 (0.419 - 0.504)	0.450 (0.409 - 0.490)	0.887 (0.795 - 0.978)	0.896 (0.796 - 0.990)	0.889 (0.800 - 0.978)	0.246 (0.111 - 0.464)	0.254 (0.116 - 0.487)	0.248 (0.110 - 0.477)
All ages (rate per capita)	0.395 (0.369 - 0.419)	0.415 (0.390 - 0.439)	0.423 (0.398 - 0.446)	0.648 (0.605 - 0.693)	0.690 (0.648 - 0.736)	0.711 (0.669 - 0.755)	0.232 (0.102 - 0.449)	0.245 (0.106 - 0.475)	0.249 (0.109 - 0.484)
Age-standardized (rate per capita)	0.417 (0.393 - 0.440)	0.419 (0.395 - 0.442)	0.417 (0.393 - 0.440)	0.706 (0.663 - 0.748)	0.706 (0.664 - 0.752)	0.706 (0.663 - 0.748)	0.244 (0.106 - 0.473)	0.246 (0.106 - 0.476)	0.245 (0.107 - 0.477)
Total number of cases (thousands)	21,561 (20,155 - 22,896)	28,361 (26,633 - 29,964)	33,149 (31,191 - 34,997)	35,396 (33,055 - 37,848)	47,097 (44,227 - 50,234)	55,761 (52,432 - 59,173)	13 (6 - 25)	17 (7 - 32)	20 (9 - 38)
**United Arab Emirates**									
5 -14 years (rate per capita)	0.325 (0.247 - 0.417)	0.343 (0.261 - 0.434)	0.330 (0.251 - 0.422)	0.530 (0.388 - 0.685)	0.553 (0.405 - 0.711)	0.536 (0.393 - 0.691)	0.195 (0.081 - 0.387)	0.205 (0.086 - 0.407)	0.198 (0.083 - 0.387)

	Prevalence 1990	Prevalence 2005	Prevalence 2015	Incidence 1990	Incidence 2005	Incidence 2015	YLD rate 1990	YLD rate 2005	YLD rate 2015
15 - 49 years (rate per capita)	0.456 (0.389 - 0.518)	0.455 (0.387 - 0.518)	0.457 (0.389 - 0.521)	0.817 (0.704 - 0.934)	0.819 (0.702 - 0.938)	0.821 (0.712 - 0.943)	0.270 (0.115 - 0.533)	0.270 (0.117 - 0.531)	0.270 (0.116 - 0.533)
50 - 69 years (rate per capita)	0.441 (0.354 - 0.521)	0.439 (0.350 - 0.521)	0.439 (0.349 - 0.522)	0.868 (0.694 - 1.032)	0.867 (0.689 - 1.034)	0.867 (0.689 - 1.033)	0.253 (0.109 - 0.510)	0.253 (0.109 - 0.510)	0.253 (0.109 - 0.506)
70+ years (rate per capita)	0.440 (0.366 - 0.517)	0.436 (0.362 - 0.508)	0.436 (0.361 - 0.509)	0.877 (0.718 - 1.028)	0.875 (0.723 - 1.020)	0.876 (0.720 - 1.025)	0.240 (0.107 - 0.468)	0.239 (0.108 - 0.465)	0.238 (0.106 - 0.460)
All ages (rate per capita)	0.376 (0.330 - 0.422)	0.412 (0.359 - 0.461)	0.420 (0.369 - 0.470)	0.669 (0.592 - 0.750)	0.737 (0.651 - 0.831)	0.758 (0.674 - 0.854)	0.222 (0.096 - 0.433)	0.244 (0.106 - 0.474)	0.248 (0.108 - 0.485)
Age-standardized (rate per capita)	0.391 (0.353 - 0.430)	0.390 (0.353 - 0.430)	0.390 (0.353 - 0.430)	0.710 (0.644 - 0.777)	0.710 (0.644 - 0.777)	0.710 (0.644 - 0.777)	0.228 (0.101 - 0.439)	0.229 (0.101 - 0.440)	0.229 (0.101 - 0.440)
Total number of cases (thousands)	679 (596 - 761)	1,846 (1,609 - 2,069)	3,843 (3,372 - 4,302)	1,207 (1,068 - 1,353)	3,308 (2,921 - 3,727)	6,937 (6,163 - 7,807)	0 (0 - 1)	1 (0 - 2)	2 (1 - 4)
**Yemen**									
5 -14 years (rate per capita)	0.327 (0.249 - 0.420)	0.341 (0.260 - 0.432)	0.336 (0.256 - 0.428)	0.533 (0.390 - 0.688)	0.550 (0.403 - 0.707)	0.544 (0.401 - 0.700)	0.195 (0.081 - 0.386)	0.203 (0.085 - 0.390)	0.201 (0.084 - 0.394)
15 - 49 years (rate per capita)	0.454 (0.390 - 0.517)	0.454 (0.389 - 0.518)	0.454 (0.389 - 0.515)	0.806 (0.690 - 0.921)	0.806 (0.689 - 0.921)	0.808 (0.691 - 0.928)	0.268 (0.114 - 0.524)	0.267 (0.113 - 0.525)	0.268 (0.113 - 0.530)
50 - 69 years (rate per capita)	0.446 (0.368 - 0.517)	0.444 (0.365 - 0.517)	0.446 (0.368 - 0.518)	0.874 (0.707 - 1.025)	0.872 (0.697 - 1.026)	0.875 (0.713 - 1.028)	0.255 (0.110 - 0.507)	0.254 (0.109 - 0.504)	0.255 (0.110 - 0.506)
70+ years (rate per capita)	0.441 (0.366 - 0.515)	0.435 (0.364 - 0.506)	0.439 (0.364 - 0.514)	0.878 (0.721 - 1.025)	0.873 (0.723 - 1.015)	0.877 (0.719 - 1.023)	0.241 (0.108 - 0.470)	0.237 (0.107 - 0.453)	0.240 (0.108 - 0.464)
All ages (rate per capita)	0.317 (0.282 - 0.354)	0.344 (0.306 - 0.383)	0.356 (0.316 - 0.394)	0.555 (0.485 - 0.620)	0.603 (0.529 - 0.671)	0.626 (0.555 - 0.696)	0.187 (0.081 - 0.359)	0.203 (0.088 - 0.390)	0.210 (0.091 - 0.405)
Age-standardized (rate per capita)	0.392 (0.356 - 0.431)	0.392 (0.355 - 0.431)	0.392 (0.355 - 0.431)	0.709 (0.644 - 0.774)	0.709 (0.644 - 0.775)	0.709 (0.644 - 0.774)	0.229 (0.101 - 0.442)	0.228 (0.101 - 0.440)	0.229 (0.101 - 0.442)
Total number of cases (thousands)	3,793 (3,377 - 4,231)	7,082 (6,299 - 7,884)	9,570 (8,508 - 10,614)	6,640 (5,800 - 7,419)	12,400 (10,883 - 13,802)	16,860 (14,940 - 18,722)	2 (1 - 4)	4 (2 - 8)	6 (2 - 11)
**South Asia**									
5 -14 years (rate per capita)	0.252 (0.201 - 0.316)	0.256 (0.203 - 0.323)	0.255 (0.203 - 0.321)	0.301 (0.230 - 0.396)	0.309 (0.232 - 0.406)	0.302 (0.229 - 0.397)	0.150 (0.063 - 0.294)	0.153 (0.064 - 0.301)	0.152 (0.064 - 0.300)
15 - 49 years (rate per capita)	0.411 (0.376 - 0.447)	0.406 (0.373 - 0.441)	0.408 (0.374 - 0.441)	0.707 (0.636 - 0.779)	0.706 (0.639 - 0.775)	0.710 (0.642 - 0.779)	0.242 (0.106 - 0.480)	0.240 (0.104 - 0.475)	0.241 (0.105 - 0.479)
50 - 69 years (rate per capita)	0.389 (0.345 - 0.436)	0.390 (0.347 - 0.437)	0.391 (0.348 - 0.439)	0.734 (0.646 - 0.832)	0.734 (0.648 - 0.830)	0.735 (0.647 - 0.833)	0.222 (0.096 - 0.429)	0.223 (0.095 - 0.431)	0.224 (0.096 - 0.435)
70+ years (rate per capita)	0.351 (0.313 - 0.393)	0.351 (0.316 - 0.390)	0.349 (0.313 - 0.386)	0.634 (0.556 - 0.719)	0.632 (0.557 - 0.710)	0.627 (0.552 - 0.701)	0.192 (0.086 - 0.366)	0.192 (0.087 - 0.367)	0.192 (0.086 - 0.368)
All ages (rate per capita)	0.309 (0.285 - 0.333)	0.323 (0.299 - 0.346)	0.333 (0.309 - 0.356)	0.507 (0.466 - 0.551)	0.538 (0.497 - 0.582)	0.560 (0.518 - 0.602)	0.181 (0.079 - 0.345)	0.190 (0.082 - 0.364)	0.196 (0.085 - 0.379)
Age-standardized (rate per capita)	0.337 (0.316 - 0.358)	0.335 (0.315 - 0.356)	0.336 (0.314 - 0.358)	0.577 (0.536 - 0.617)	0.576 (0.536 - 0.615)	0.575 (0.535 - 0.615)	0.197 (0.085 - 0.379)	0.196 (0.084 - 0.377)	0.197 (0.085 - 0.381)
Total number of cases (thousands)	341,092 (314,777 - 367,221)	473,297 (439,236 - 507,038)	563,101 (523,178 - 601,639)	559,429 (514,801 - 608,665)	788,901 (729,865 - 854,154)	946,304 (875,652 - 1,017,836)	200 (87 - 381)	278 (121 - 534)	331 (143 - 642)
**South Asia**									
5 -14 years (rate per capita)	0.252 (0.201 - 0.316)	0.256 (0.203 - 0.323)	0.255 (0.203 - 0.321)	0.301 (0.230 - 0.396)	0.309 (0.232 - 0.406)	0.302 (0.229 - 0.397)	0.150 (0.063 - 0.294)	0.153 (0.064 - 0.301)	0.152 (0.064 - 0.300)
15 - 49 years (rate per capita)	0.411 (0.376 - 0.447)	0.406 (0.373 - 0.441)	0.408 (0.374 - 0.441)	0.707 (0.636 - 0.779)	0.706 (0.639 - 0.775)	0.710 (0.642 - 0.779)	0.242 (0.106 - 0.480)	0.240 (0.104 - 0.475)	0.241 (0.105 - 0.479)
50 - 69 years (rate per capita)	0.389 (0.345 - 0.436)	0.390 (0.347 - 0.437)	0.391 (0.348 - 0.439)	0.734 (0.646 - 0.832)	0.734 (0.648 - 0.830)	0.735 (0.647 - 0.833)	0.222 (0.096 - 0.429)	0.223 (0.095 - 0.431)	0.224 (0.096 - 0.435)
70+ years (rate per capita)	0.351 (0.313 - 0.393)	0.351 (0.316 - 0.390)	0.349 (0.313 - 0.386)	0.634 (0.556 - 0.719)	0.632 (0.557 - 0.710)	0.627 (0.552 - 0.701)	0.192 (0.086 - 0.366)	0.192 (0.087 - 0.367)	0.192 (0.086 - 0.368)
All ages (rate per capita)	0.309 (0.285 - 0.333)	0.323 (0.299 - 0.346)	0.333 (0.309 - 0.356)	0.507 (0.466 - 0.551)	0.538 (0.497 - 0.582)	0.560 (0.518 - 0.602)	0.181 (0.079 - 0.345)	0.190 (0.082 - 0.364)	0.196 (0.085 - 0.379)
Age-standardized (rate per capita)	0.337 (0.316 - 0.358)	0.335 (0.315 - 0.356)	0.336 (0.314 - 0.358)	0.577 (0.536 - 0.617)	0.576 (0.536 - 0.615)	0.575 (0.535 - 0.615)	0.197 (0.085 - 0.379)	0.196 (0.084 - 0.377)	0.197 (0.085 - 0.381)
Total number of cases (thousands)	341,092 (314,777 - 367,221)	473,297 (439,236 - 507,038)	563,101 (523,178 - 601,639)	559,429 (514,801 - 608,665)	788,901 (729,865 - 854,154)	946,304 (875,652 - 1,017,836)	200 (87 - 381)	278 (121 - 534)	331 (143 - 642)
**Bangladesh**									
5 -14 years (rate per capita)	0.245 (0.192 - 0.313)	0.253 (0.198 - 0.323)	0.259 (0.203 - 0.331)	0.290 (0.211 - 0.391)	0.298 (0.217 - 0.397)	0.304 (0.220 - 0.402)	0.145 (0.060 - 0.287)	0.151 (0.062 - 0.297)	0.154 (0.064 - 0.310)
15 - 49 years (rate per capita)	0.397 (0.356 - 0.439)	0.396 (0.356 - 0.435)	0.394 (0.354 - 0.433)	0.681 (0.602 - 0.769)	0.687 (0.609 - 0.770)	0.691 (0.616 - 0.773)	0.233 (0.101 - 0.457)	0.233 (0.100 - 0.461)	0.233 (0.100 - 0.454)
50 - 69 years (rate per capita)	0.374 (0.327 - 0.428)	0.375 (0.327 - 0.430)	0.374 (0.325 - 0.428)	0.717 (0.617 - 0.823)	0.716 (0.618 - 0.822)	0.719 (0.617 - 0.825)	0.213 (0.093 - 0.419)	0.214 (0.092 - 0.414)	0.215 (0.092 - 0.420)
70+ years (rate per capita)	0.343 (0.298 - 0.392)	0.336 (0.296 - 0.378)	0.330 (0.292 - 0.370)	0.626 (0.539 - 0.725)	0.617 (0.532 - 0.704)	0.610 (0.527 - 0.690)	0.187 (0.086 - 0.364)	0.184 (0.084 - 0.358)	0.181 (0.082 - 0.348)
All ages (rate per capita)	0.292 (0.266 - 0.319)	0.314 (0.286 - 0.340)	0.325 (0.298 - 0.353)	0.473 (0.429 - 0.526)	0.519 (0.473 - 0.574)	0.549 (0.502 - 0.602)	0.171 (0.074 - 0.322)	0.184 (0.080 - 0.351)	0.191 (0.083 - 0.367)
Age-standardized (rate per capita)	0.325 (0.301 - 0.350)	0.325 (0.301 - 0.350)	0.325 (0.301 - 0.351)	0.561 (0.517 - 0.608)	0.561 (0.517 - 0.609)	0.561 (0.517 - 0.609)	0.189 (0.081 - 0.362)	0.190 (0.082 - 0.365)	0.190 (0.082 - 0.365)
Total number of cases (thousands)	30,918 (28,167 - 33,859)	44,800 (40,872 - 48,572)	52,344 (47,999 - 56,742)	50,123 (45,504 - 55,757)	74,179 (67,540 - 81,921)	88,343 (80,768 - 96,901)	18 (8 - 34)	26 (11 - 50)	31 (13 - 59)
**Bhutan**									
5 -14 years (rate per capita)	0.244 (0.190 - 0.312)	0.258 (0.202 - 0.330)	0.254 (0.199 - 0.325)	0.288 (0.210 - 0.390)	0.303 (0.220 - 0.401)	0.299 (0.218 - 0.398)	0.145 (0.060 - 0.290)	0.154 (0.063 - 0.304)	0.152 (0.063 - 0.299)
15 - 49 years (rate per capita)	0.397 (0.358 - 0.437)	0.396 (0.355 - 0.435)	0.392 (0.353 - 0.430)	0.684 (0.606 - 0.764)	0.682 (0.602 - 0.771)	0.694 (0.617 - 0.779)	0.235 (0.100 - 0.462)	0.235 (0.101 - 0.461)	0.233 (0.100 - 0.458)
50 - 69 years (rate per capita)	0.374 (0.326 - 0.429)	0.373 (0.326 - 0.427)	0.373 (0.326 - 0.427)	0.717 (0.615 - 0.823)	0.715 (0.617 - 0.821)	0.716 (0.615 - 0.821)	0.215 (0.092 - 0.420)	0.215 (0.092 - 0.421)	0.215 (0.092 - 0.421)
70+ years (rate per capita)	0.339 (0.297 - 0.386)	0.335 (0.295 - 0.378)	0.327 (0.290 - 0.368)	0.622 (0.537 - 0.714)	0.616 (0.532 - 0.704)	0.607 (0.525 - 0.687)	0.187 (0.085 - 0.366)	0.185 (0.084 - 0.359)	0.180 (0.082 - 0.350)
All ages (rate per capita)	0.287 (0.262 - 0.313)	0.317 (0.289 - 0.344)	0.329 (0.302 - 0.357)	0.466 (0.425 - 0.516)	0.521 (0.473 - 0.576)	0.562 (0.514 - 0.618)	0.169 (0.073 - 0.322)	0.187 (0.082 - 0.359)	0.194 (0.084 - 0.372)
Age-standardized (rate per capita)	0.325 (0.301 - 0.350)	0.325 (0.301 - 0.350)	0.325 (0.301 - 0.350)	0.561 (0.516 - 0.608)	0.560 (0.516 - 0.608)	0.560 (0.516 - 0.608)	0.191 (0.082 - 0.366)	0.191 (0.083 - 0.367)	0.190 (0.082 - 0.365)
Total number of cases (thousands)	153 (140 - 167)	206 (188 - 224)	255 (234 - 276)	249 (227 - 275)	339 (308 - 375)	435 (398 - 479)	0 (0 - 0)	0 (0 - 0)	0 (0 - 0)
**India**									
5 -14 years (rate per capita)	0.251 (0.201 - 0.316)	0.257 (0.203 - 0.324)	0.257 (0.204 - 0.319)	0.304 (0.232 - 0.399)	0.314 (0.236 - 0.412)	0.304 (0.232 - 0.402)	0.150 (0.063 - 0.298)	0.153 (0.065 - 0.305)	0.153 (0.065 - 0.298)
15 - 49 years (rate per capita)	0.414 (0.379 - 0.449)	0.409 (0.377 - 0.442)	0.411 (0.378 - 0.445)	0.715 (0.644 - 0.786)	0.713 (0.646 - 0.781)	0.717 (0.651 - 0.785)	0.244 (0.108 - 0.484)	0.241 (0.105 - 0.479)	0.243 (0.106 - 0.484)
50 - 69 years (rate per capita)	0.392 (0.348 - 0.438)	0.393 (0.350 - 0.439)	0.395 (0.351 - 0.442)	0.739 (0.650 - 0.836)	0.739 (0.652 - 0.837)	0.739 (0.653 - 0.835)	0.223 (0.096 - 0.431)	0.224 (0.096 - 0.433)	0.226 (0.097 - 0.441)
70+ years (rate per capita)	0.354 (0.315 - 0.395)	0.355 (0.320 - 0.393)	0.353 (0.317 - 0.390)	0.638 (0.559 - 0.723)	0.636 (0.563 - 0.715)	0.631 (0.556 - 0.703)	0.193 (0.087 - 0.366)	0.194 (0.087 - 0.370)	0.194 (0.086 - 0.370)
All ages (rate per capita)	0.314 (0.291 - 0.337)	0.326 (0.304 - 0.349)	0.338 (0.314 - 0.360)	0.517 (0.476 - 0.561)	0.547 (0.506 - 0.591)	0.569 (0.528 - 0.612)	0.184 (0.080 - 0.351)	0.192 (0.083 - 0.369)	0.199 (0.086 - 0.386)
Age-standardized (rate per capita)	0.340 (0.318 - 0.359)	0.338 (0.317 - 0.358)	0.339 (0.317 - 0.360)	0.582 (0.540 - 0.622)	0.581 (0.541 - 0.620)	0.579 (0.539 - 0.620)	0.198 (0.086 - 0.382)	0.197 (0.085 - 0.380)	0.198 (0.085 - 0.385)
Total number of cases (thousands)	273,106 (253,019 - 293,483)	373,590 (347,766 - 398,832)	443,138 (412,409 - 472,704)	450,455 (414,166 - 488,441)	626,311 (579,550 - 675,702)	746,765 (692,418 - 802,191)	160 (70 - 306)	219 (95 - 422)	260 (113 - 507)
**Nepal**									
5 -14 years (rate per capita)	0.227 (0.177 - 0.292)	0.239 (0.190 - 0.302)	0.245 (0.191 - 0.314)	0.274 (0.199 - 0.376)	0.284 (0.213 - 0.390)	0.293 (0.214 - 0.391)	0.135 (0.056 - 0.267)	0.142 (0.059 - 0.281)	0.146 (0.061 - 0.288)
15 - 49 years (rate per capita)	0.379 (0.342 - 0.415)	0.380 (0.347 - 0.416)	0.380 (0.343 - 0.415)	0.667 (0.595 - 0.744)	0.668 (0.599 - 0.739)	0.667 (0.596 - 0.743)	0.224 (0.097 - 0.446)	0.225 (0.096 - 0.442)	0.225 (0.097 - 0.448)
50 - 69 years (rate per capita)	0.358 (0.313 - 0.405)	0.356 (0.312 - 0.404)	0.358 (0.313 - 0.405)	0.705 (0.616 - 0.806)	0.701 (0.621 - 0.799)	0.703 (0.618 - 0.802)	0.206 (0.089 - 0.404)	0.205 (0.088 - 0.402)	0.206 (0.088 - 0.410)
70+ years (rate per capita)	0.328 (0.289 - 0.370)	0.323 (0.286 - 0.365)	0.322 (0.286 - 0.363)	0.614 (0.536 - 0.700)	0.608 (0.531 - 0.689)	0.607 (0.533 - 0.689)	0.181 (0.082 - 0.347)	0.179 (0.080 - 0.345)	0.179 (0.081 - 0.346)
All ages (rate per capita)	0.275 (0.250 - 0.299)	0.288 (0.265 - 0.313)	0.306 (0.281 - 0.332)	0.458 (0.418 - 0.504)	0.479 (0.438 - 0.525)	0.518 (0.474 - 0.564)	0.162 (0.070 - 0.309)	0.170 (0.073 - 0.322)	0.181 (0.079 - 0.344)
Age-standardized (rate per capita)	0.310 (0.287 - 0.332)	0.311 (0.290 - 0.333)	0.310 (0.287 - 0.332)	0.547 (0.505 - 0.589)	0.547 (0.505 - 0.589)	0.547 (0.506 - 0.589)	0.181 (0.078 - 0.348)	0.182 (0.078 - 0.351)	0.182 (0.078 - 0.349)
Total number of cases (thousands)	5,149 (4,691 - 5,604)	7,339 (6,754 - 7,980)	8,749 (8,030 - 9,472)	8,580 (7,822 - 9,443)	12,207 (11,173 - 13,392)	14,781 (13,533 - 16,103)	3 (1 - 6)	4 (2 - 8)	5 (2 - 10)
**Pakistan**									
5 -14 years (rate per capita)	0.264 (0.201 - 0.343)	0.255 (0.197 - 0.325)	0.247 (0.192 - 0.318)	0.296 (0.219 - 0.412)	0.294 (0.221 - 0.392)	0.287 (0.216 - 0.388)	0.158 (0.065 - 0.304)	0.152 (0.064 - 0.299)	0.147 (0.062 - 0.292)
15 - 49 years (rate per capita)	0.405 (0.367 - 0.442)	0.402 (0.364 - 0.439)	0.400 (0.362 - 0.437)	0.677 (0.606 - 0.753)	0.677 (0.606 - 0.749)	0.682 (0.610 - 0.754)	0.239 (0.103 - 0.472)	0.238 (0.104 - 0.469)	0.237 (0.103 - 0.466)
50 - 69 years (rate per capita)	0.379 (0.333 - 0.429)	0.380 (0.334 - 0.429)	0.379 (0.333 - 0.428)	0.716 (0.631 - 0.818)	0.716 (0.634 - 0.816)	0.717 (0.630 - 0.819)	0.218 (0.095 - 0.425)	0.218 (0.094 - 0.425)	0.218 (0.094 - 0.426)
70+ years (rate per capita)	0.341 (0.302 - 0.385)	0.340 (0.301 - 0.382)	0.340 (0.301 - 0.382)	0.618 (0.542 - 0.700)	0.615 (0.542 - 0.699)	0.616 (0.543 - 0.699)	0.188 (0.085 - 0.362)	0.187 (0.083 - 0.359)	0.187 (0.083 - 0.360)
All ages (rate per capita)	0.294 (0.269 - 0.323)	0.308 (0.282 - 0.333)	0.310 (0.286 - 0.335)	0.464 (0.423 - 0.507)	0.493 (0.451 - 0.539)	0.508 (0.466 - 0.555)	0.173 (0.076 - 0.327)	0.181 (0.079 - 0.347)	0.183 (0.079 - 0.350)
Age-standardized (rate per capita)	0.334 (0.310 - 0.359)	0.329 (0.307 - 0.353)	0.329 (0.307 - 0.353)	0.559 (0.518 - 0.601)	0.557 (0.518 - 0.601)	0.558 (0.518 - 0.601)	0.196 (0.085 - 0.372)	0.193 (0.083 - 0.373)	0.193 (0.084 - 0.370)
Total number of cases (thousands)	31,765 (28,974 - 34,906)	47,362 (43,398 - 51,291)	58,614 (54,023 - 63,246)	50,022 (45,606 - 54,689)	75,865 (69,438 - 83,061)	95,979 (88,063 - 104,886)	19 (8 - 35)	28 (12 - 53)	35 (15 - 66)
**Sub-Saharan Africa**									

	Prevalence 1990	Prevalence 2005	Prevalence 2015	Incidence 1990	Incidence 2005	Incidence 2015	YLD rate 1990	YLD rate 2005	YLD rate 2015
5 -14 years (rate per capita)	0.216 (0.165 - 0.280)	0.226 (0.173 - 0.291)	0.224 (0.168 - 0.290)	0.260 (0.182 - 0.354)	0.269 (0.191 - 0.368)	0.266 (0.187 - 0.367)	0.129 (0.054 - 0.251)	0.135 (0.056 - 0.265)	0.134 (0.056 - 0.260)
15 - 49 years (rate per capita)	0.447 (0.396 - 0.495)	0.446 (0.395 - 0.495)	0.447 (0.396 - 0.496)	0.687 (0.599 - 0.781)	0.685 (0.598 - 0.780)	0.688 (0.600 - 0.782)	0.263 (0.112 - 0.514)	0.263 (0.112 - 0.512)	0.264 (0.112 - 0.517)
50 - 69 years (rate per capita)	0.364 (0.305 - 0.428)	0.363 (0.305 - 0.427)	0.364 (0.306 - 0.427)	0.696 (0.573 - 0.826)	0.695 (0.573 - 0.823)	0.695 (0.575 - 0.822)	0.208 (0.089 - 0.411)	0.208 (0.089 - 0.409)	0.209 (0.089 - 0.411)
70+ years (rate per capita)	0.335 (0.283 - 0.393)	0.335 (0.283 - 0.391)	0.336 (0.285 - 0.392)	0.607 (0.495 - 0.721)	0.607 (0.499 - 0.719)	0.610 (0.501 - 0.723)	0.183 (0.082 - 0.351)	0.183 (0.083 - 0.350)	0.184 (0.083 - 0.353)
All ages (rate per capita)	0.294 (0.264 - 0.323)	0.302 (0.271 - 0.331)	0.305 (0.274 - 0.335)	0.445 (0.399 - 0.493)	0.455 (0.406 - 0.506)	0.462 (0.413 - 0.514)	0.173 (0.076 - 0.330)	0.177 (0.077 - 0.338)	0.180 (0.078 - 0.343)
Age-standardized (rate per capita)	0.345 (0.315 - 0.374)	0.346 (0.317 - 0.375)	0.346 (0.317 - 0.375)	0.552 (0.504 - 0.602)	0.553 (0.505 - 0.604)	0.553 (0.506 - 0.604)	0.201 (0.088 - 0.383)	0.202 (0.088 - 0.384)	0.202 (0.088 - 0.387)
Total number of cases (thousands)	144,451 (129,500 - 158,655)	220,878 (198,283 - 242,420)	293,331 (263,578 - 321,857)	218,551 (196,019 - 242,243)	332,989 (297,501 - 370,183)	443,414 (396,479 - 493,604)	85 (37 - 162)	130 (57 - 247)	173 (75 - 330)
**Southern Sub-Saharan Africa**									
5 -14 years (rate per capita)	0.185 (0.143 - 0.239)	0.198 (0.153 - 0.257)	0.193 (0.148 - 0.252)	0.241 (0.176 - 0.329)	0.251 (0.181 - 0.345)	0.243 (0.176 - 0.330)	0.110 (0.046 - 0.219)	0.118 (0.050 - 0.232)	0.115 (0.049 - 0.226)
15 - 49 years (rate per capita)	0.424 (0.386 - 0.462)	0.424 (0.387 - 0.462)	0.423 (0.385 - 0.460)	0.693 (0.626 - 0.765)	0.693 (0.624 - 0.765)	0.695 (0.627 - 0.768)	0.251 (0.108 - 0.495)	0.249 (0.107 - 0.492)	0.249 (0.107 - 0.493)
50 - 69 years (rate per capita)	0.328 (0.288 - 0.373)	0.328 (0.288 - 0.374)	0.329 (0.288 - 0.374)	0.672 (0.590 - 0.774)	0.673 (0.591 - 0.773)	0.675 (0.592 - 0.774)	0.188 (0.081 - 0.367)	0.187 (0.081 - 0.369)	0.187 (0.081 - 0.366)
70+ years (rate per capita)	0.294 (0.262 - 0.329)	0.294 (0.262 - 0.329)	0.292 (0.262 - 0.327)	0.569 (0.499 - 0.643)	0.569 (0.499 - 0.643)	0.566 (0.494 - 0.637)	0.161 (0.072 - 0.312)	0.160 (0.072 - 0.305)	0.159 (0.072 - 0.304)
All ages (rate per capita)	0.289 (0.265 - 0.312)	0.310 (0.286 - 0.334)	0.315 (0.290 - 0.339)	0.470 (0.432 - 0.516)	0.508 (0.466 - 0.553)	0.522 (0.480 - 0.568)	0.170 (0.074 - 0.328)	0.182 (0.079 - 0.351)	0.185 (0.080 - 0.358)
Age-standardized (rate per capita)	0.320 (0.298 - 0.341)	0.322 (0.299 - 0.343)	0.322 (0.299 - 0.343)	0.544 (0.504 - 0.584)	0.545 (0.506 - 0.586)	0.545 (0.505 - 0.587)	0.187 (0.081 - 0.361)	0.187 (0.081 - 0.360)	0.188 (0.080 - 0.364)
Total number of cases (thousands)	15,435 (14,136 - 16,671)	21,240 (19,595 - 22,877)	24,417 (22,424 - 26,229)	25,104 (23,045 - 27,521)	34,737 (31,918 - 37,873)	40,382 (37,147 - 43,969)	9 (4 - 18)	12 (5 - 24)	14 (6 - 28)
**Botswana**									
5 -14 years (rate per capita)	0.213 (0.133 - 0.316)	0.222 (0.139 - 0.326)	0.218 (0.137 - 0.321)	0.317 (0.173 - 0.481)	0.330 (0.175 - 0.494)	0.324 (0.174 - 0.489)	0.127 (0.048 - 0.262)	0.133 (0.050 - 0.274)	0.130 (0.049 - 0.264)
15 - 49 years (rate per capita)	0.443 (0.361 - 0.517)	0.442 (0.362 - 0.516)	0.443 (0.363 - 0.519)	0.743 (0.606 - 0.872)	0.743 (0.605 - 0.874)	0.747 (0.614 - 0.877)	0.262 (0.105 - 0.521)	0.260 (0.106 - 0.509)	0.260 (0.107 - 0.509)
50 - 69 years (rate per capita)	0.385 (0.282 - 0.493)	0.385 (0.282 - 0.492)	0.384 (0.284 - 0.495)	0.731 (0.525 - 0.946)	0.730 (0.525 - 0.943)	0.731 (0.520 - 0.951)	0.221 (0.095 - 0.458)	0.219 (0.094 - 0.453)	0.218 (0.094 - 0.442)
70+ years (rate per capita)	0.351 (0.259 - 0.458)	0.351 (0.258 - 0.457)	0.351 (0.260 - 0.456)	0.660 (0.458 - 0.871)	0.659 (0.458 - 0.867)	0.658 (0.459 - 0.866)	0.193 (0.086 - 0.381)	0.191 (0.085 - 0.377)	0.191 (0.085 - 0.378)
All ages (rate per capita)	0.299 (0.251 - 0.347)	0.330 (0.279 - 0.380)	0.338 (0.288 - 0.386)	0.498 (0.423 - 0.574)	0.553 (0.472 - 0.636)	0.571 (0.491 - 0.652)	0.176 (0.076 - 0.342)	0.194 (0.083 - 0.381)	0.198 (0.084 - 0.385)
Age-standardized (rate per capita)	0.348 (0.300 - 0.395)	0.348 (0.300 - 0.395)	0.348 (0.300 - 0.395)	0.599 (0.522 - 0.677)	0.598 (0.522 - 0.677)	0.598 (0.522 - 0.677)	0.204 (0.089 - 0.397)	0.203 (0.088 - 0.397)	0.202 (0.089 - 0.396)
Total number of cases (thousands)	414 (348 - 480)	617 (520 - 710)	763 (652 - 872)	689 (586 - 794)	1,033 (881 - 1,188)	1,290 (1,109 - 1,474)	0 (0 - 0)	0 (0 - 1)	0 (0 - 1)
**Lesotho**									
5 -14 years (rate per capita)	0.214 (0.134 - 0.317)	0.219 (0.137 - 0.322)	0.220 (0.138 - 0.324)	0.319 (0.173 - 0.483)	0.325 (0.174 - 0.490)	0.328 (0.174 - 0.491)	0.128 (0.049 - 0.263)	0.131 (0.050 - 0.271)	0.132 (0.050 - 0.267)
15 - 49 years (rate per capita)	0.443 (0.361 - 0.519)	0.442 (0.360 - 0.521)	0.441 (0.358 - 0.520)	0.741 (0.604 - 0.869)	0.741 (0.600 - 0.874)	0.744 (0.606 - 0.876)	0.262 (0.105 - 0.517)	0.260 (0.105 - 0.509)	0.260 (0.105 - 0.512)
50 - 69 years (rate per capita)	0.384 (0.288 - 0.493)	0.385 (0.286 - 0.493)	0.385 (0.289 - 0.492)	0.731 (0.525 - 0.950)	0.730 (0.527 - 0.942)	0.732 (0.525 - 0.949)	0.221 (0.094 - 0.461)	0.219 (0.094 - 0.451)	0.218 (0.093 - 0.454)
70+ years (rate per capita)	0.351 (0.259 - 0.456)	0.348 (0.260 - 0.452)	0.344 (0.260 - 0.445)	0.659 (0.458 - 0.868)	0.656 (0.461 - 0.855)	0.651 (0.466 - 0.846)	0.193 (0.085 - 0.382)	0.190 (0.084 - 0.378)	0.187 (0.083 - 0.368)
All ages (rate per capita)	0.300 (0.252 - 0.346)	0.317 (0.267 - 0.365)	0.327 (0.276 - 0.376)	0.500 (0.427 - 0.578)	0.529 (0.452 - 0.610)	0.549 (0.469 - 0.634)	0.177 (0.076 - 0.341)	0.186 (0.080 - 0.361)	0.192 (0.081 - 0.377)
Age-standardized (rate per capita)	0.349 (0.301 - 0.395)	0.349 (0.301 - 0.396)	0.348 (0.300 - 0.395)	0.598 (0.522 - 0.676)	0.598 (0.522 - 0.677)	0.599 (0.522 - 0.678)	0.204 (0.089 - 0.399)	0.203 (0.088 - 0.394)	0.202 (0.088 - 0.395)
Total number of cases (thousands)	480 (404 - 554)	610 (514 - 703)	695 (587 - 801)	802 (685 - 926)	1,020 (870 - 1,175)	1,169 (998 - 1,349)	0 (0 - 1)	0 (0 - 1)	0 (0 - 1)
**Namibia**									
5 -14 years (rate per capita)	0.232 (0.182 - 0.295)	0.227 (0.174 - 0.301)	0.225 (0.172 - 0.299)	0.250 (0.188 - 0.339)	0.251 (0.181 - 0.353)	0.249 (0.179 - 0.352)	0.138 (0.058 - 0.267)	0.136 (0.057 - 0.267)	0.134 (0.057 - 0.265)
15 - 49 years (rate per capita)	0.471 (0.431 - 0.511)	0.469 (0.430 - 0.509)	0.469 (0.430 - 0.508)	0.676 (0.610 - 0.752)	0.680 (0.614 - 0.756)	0.680 (0.615 - 0.754)	0.279 (0.121 - 0.547)	0.277 (0.120 - 0.548)	0.277 (0.121 - 0.545)
50 - 69 years (rate per capita)	0.367 (0.321 - 0.419)	0.366 (0.322 - 0.417)	0.367 (0.322 - 0.418)	0.690 (0.606 - 0.788)	0.691 (0.605 - 0.789)	0.692 (0.607 - 0.790)	0.211 (0.091 - 0.416)	0.210 (0.092 - 0.415)	0.210 (0.091 - 0.411)
70+ years (rate per capita)	0.348 (0.308 - 0.393)	0.345 (0.308 - 0.386)	0.345 (0.308 - 0.385)	0.598 (0.523 - 0.677)	0.595 (0.521 - 0.671)	0.595 (0.521 - 0.670)	0.192 (0.086 - 0.374)	0.190 (0.086 - 0.368)	0.190 (0.086 - 0.367)
All ages (rate per capita)	0.317 (0.293 - 0.343)	0.331 (0.305 - 0.357)	0.337 (0.313 - 0.362)	0.448 (0.413 - 0.491)	0.473 (0.434 - 0.521)	0.488 (0.449 - 0.537)	0.187 (0.081 - 0.356)	0.195 (0.084 - 0.375)	0.199 (0.087 - 0.385)
Age-standardized (rate per capita)	0.362 (0.339 - 0.383)	0.359 (0.336 - 0.381)	0.359 (0.336 - 0.381)	0.544 (0.509 - 0.584)	0.544 (0.506 - 0.585)	0.544 (0.506 - 0.585)	0.212 (0.092 - 0.409)	0.210 (0.091 - 0.410)	0.210 (0.092 - 0.409)
Total number of cases (thousands)	450 (415 - 486)	671 (619 - 724)	828 (767 - 888)	635 (585 - 696)	961 (880 - 1,058)	1,198 (1,100 - 1,317)	0 (0 - 1)	0 (0 - 1)	0 (0 - 1)
**South Africa**									
5 -14 years (rate per capita)	0.185 (0.142 - 0.241)	0.197 (0.151 - 0.258)	0.194 (0.149 - 0.256)	0.241 (0.173 - 0.333)	0.251 (0.180 - 0.347)	0.244 (0.177 - 0.332)	0.110 (0.047 - 0.222)	0.118 (0.049 - 0.234)	0.116 (0.049 - 0.227)
15 - 49 years (rate per capita)	0.423 (0.388 - 0.458)	0.422 (0.387 - 0.457)	0.421 (0.385 - 0.457)	0.698 (0.631 - 0.772)	0.699 (0.634 - 0.770)	0.701 (0.634 - 0.771)	0.250 (0.108 - 0.496)	0.249 (0.107 - 0.492)	0.248 (0.108 - 0.488)
50 - 69 years (rate per capita)	0.325 (0.288 - 0.369)	0.325 (0.286 - 0.370)	0.326 (0.286 - 0.371)	0.672 (0.593 - 0.772)	0.673 (0.593 - 0.769)	0.675 (0.595 - 0.770)	0.186 (0.080 - 0.361)	0.185 (0.080 - 0.364)	0.186 (0.080 - 0.361)
70+ years (rate per capita)	0.291 (0.261 - 0.322)	0.290 (0.260 - 0.324)	0.289 (0.261 - 0.322)	0.565 (0.499 - 0.637)	0.566 (0.497 - 0.639)	0.564 (0.495 - 0.632)	0.159 (0.071 - 0.307)	0.159 (0.071 - 0.299)	0.157 (0.071 - 0.300)
All ages (rate per capita)	0.295 (0.271 - 0.317)	0.316 (0.292 - 0.339)	0.323 (0.299 - 0.346)	0.486 (0.446 - 0.531)	0.526 (0.485 - 0.570)	0.545 (0.503 - 0.591)	0.174 (0.075 - 0.334)	0.185 (0.080 - 0.355)	0.189 (0.081 - 0.366)
Age-standardized (rate per capita)	0.318 (0.296 - 0.339)	0.320 (0.299 - 0.341)	0.320 (0.298 - 0.342)	0.545 (0.506 - 0.587)	0.547 (0.508 - 0.588)	0.547 (0.508 - 0.588)	0.186 (0.080 - 0.359)	0.186 (0.080 - 0.358)	0.187 (0.080 - 0.362)
Total number of cases (thousands)	11,081 (10,206 - 11,927)	15,327 (14,177 - 16,436)	17,352 (16,065 - 18,586)	18,255 (16,761 - 19,977)	25,499 (23,534 - 27,625)	29,253 (26,999 - 31,731)	7 (3 - 13)	9 (4 - 17)	10 (4 - 20)
**Swaziland**									
5 -14 years (rate per capita)	0.363 (0.280 - 0.440)	0.382 (0.295 - 0.461)	0.373 (0.288 - 0.451)	0.313 (0.227 - 0.409)	0.324 (0.240 - 0.416)	0.319 (0.233 - 0.414)	0.216 (0.089 - 0.434)	0.227 (0.094 - 0.454)	0.222 (0.093 - 0.453)
15 - 49 years (rate per capita)	0.584 (0.546 - 0.620)	0.583 (0.544 - 0.620)	0.580 (0.539 - 0.616)	0.679 (0.619 - 0.746)	0.677 (0.617 - 0.747)	0.683 (0.620 - 0.754)	0.345 (0.152 - 0.668)	0.342 (0.151 - 0.660)	0.340 (0.153 - 0.662)
50 - 69 years (rate per capita)	0.484 (0.438 - 0.533)	0.485 (0.438 - 0.532)	0.486 (0.440 - 0.532)	0.748 (0.658 - 0.842)	0.748 (0.659 - 0.839)	0.748 (0.659 - 0.838)	0.277 (0.120 - 0.539)	0.274 (0.122 - 0.537)	0.273 (0.119 - 0.535)
70+ years (rate per capita)	0.464 (0.420 - 0.510)	0.460 (0.419 - 0.504)	0.460 (0.418 - 0.504)	0.658 (0.579 - 0.742)	0.656 (0.579 - 0.738)	0.656 (0.579 - 0.738)	0.255 (0.114 - 0.483)	0.250 (0.112 - 0.468)	0.250 (0.113 - 0.469)
All ages (rate per capita)	0.398 (0.368 - 0.426)	0.435 (0.403 - 0.464)	0.441 (0.412 - 0.469)	0.447 (0.410 - 0.489)	0.489 (0.450 - 0.533)	0.508 (0.469 - 0.555)	0.234 (0.102 - 0.449)	0.255 (0.110 - 0.487)	0.258 (0.113 - 0.493)
Age-standardized (rate per capita)	0.469 (0.443 - 0.493)	0.469 (0.443 - 0.492)	0.468 (0.443 - 0.492)	0.572 (0.536 - 0.612)	0.573 (0.536 - 0.612)	0.573 (0.537 - 0.613)	0.274 (0.119 - 0.528)	0.271 (0.118 - 0.520)	0.271 (0.118 - 0.522)
Total number of cases (thousands)	344 (318 - 368)	481 (446 - 514)	569 (531 - 605)	386 (354 - 422)	541 (497 - 589)	655 (604 - 715)	0 (0 - 0)	0 (0 - 1)	0 (0 - 1)
**Zimbabwe**									
5 -14 years (rate per capita)	0.157 (0.122 - 0.201)	0.174 (0.134 - 0.227)	0.167 (0.128 - 0.217)	0.212 (0.150 - 0.288)	0.224 (0.156 - 0.302)	0.214 (0.149 - 0.289)	0.094 (0.039 - 0.187)	0.104 (0.043 - 0.206)	0.100 (0.042 - 0.203)
15 - 49 years (rate per capita)	0.405 (0.361 - 0.446)	0.404 (0.358 - 0.448)	0.404 (0.360 - 0.445)	0.659 (0.588 - 0.737)	0.655 (0.584 - 0.736)	0.660 (0.590 - 0.741)	0.239 (0.102 - 0.476)	0.237 (0.102 - 0.465)	0.238 (0.102 - 0.474)
50 - 69 years (rate per capita)	0.301 (0.259 - 0.348)	0.302 (0.260 - 0.350)	0.302 (0.261 - 0.350)	0.640 (0.558 - 0.734)	0.639 (0.558 - 0.733)	0.641 (0.557 - 0.739)	0.173 (0.073 - 0.339)	0.173 (0.075 - 0.341)	0.173 (0.075 - 0.340)
70+ years (rate per capita)	0.277 (0.240 - 0.318)	0.273 (0.240 - 0.311)	0.272 (0.240 - 0.309)	0.548 (0.480 - 0.624)	0.543 (0.473 - 0.617)	0.542 (0.472 - 0.614)	0.153 (0.069 - 0.298)	0.150 (0.068 - 0.289)	0.150 (0.068 - 0.290)
All ages (rate per capita)	0.254 (0.229 - 0.278)	0.271 (0.244 - 0.298)	0.270 (0.244 - 0.296)	0.413 (0.374 - 0.458)	0.436 (0.394 - 0.487)	0.438 (0.397 - 0.486)	0.150 (0.065 - 0.292)	0.159 (0.069 - 0.305)	0.159 (0.069 - 0.309)
Age-standardized (rate per capita)	0.300 (0.277 - 0.322)	0.302 (0.277 - 0.325)	0.302 (0.277 - 0.326)	0.517 (0.478 - 0.556)	0.517 (0.479 - 0.559)	0.517 (0.479 - 0.559)	0.176 (0.075 - 0.342)	0.176 (0.076 - 0.338)	0.176 (0.077 - 0.342)
Total number of cases (thousands)	2,667 (2,410 - 2,920)	3,534 (3,175 - 3,881)	4,209 (3,796 - 4,608)	4,337 (3,931 - 4,809)	5,683 (5,136 - 6,341)	6,817 (6,191 - 7,566)	2 (1 - 3)	2 (1 - 4)	2 (1 - 5)
**Western Sub-Saharan Africa**									
5 -14 years (rate per capita)	0.213 (0.160 - 0.286)	0.214 (0.161 - 0.285)	0.215 (0.162 - 0.287)	0.236 (0.160 - 0.329)	0.236 (0.160 - 0.326)	0.236 (0.160 - 0.327)	0.127 (0.052 - 0.250)	0.127 (0.053 - 0.252)	0.128 (0.053 - 0.256)
15 - 49 years (rate per capita)	0.444 (0.383 - 0.500)	0.444 (0.383 - 0.501)	0.444 (0.383 - 0.500)	0.650 (0.551 - 0.762)	0.650 (0.550 - 0.762)	0.651 (0.551 - 0.763)	0.261 (0.109 - 0.505)	0.261 (0.109 - 0.507)	0.262 (0.109 - 0.510)
50 - 69 years (rate per capita)	0.342 (0.278 - 0.414)	0.342 (0.278 - 0.413)	0.342 (0.278 - 0.413)	0.648 (0.513 - 0.796)	0.647 (0.513 - 0.797)	0.647 (0.514 - 0.795)	0.195 (0.085 - 0.389)	0.196 (0.085 - 0.388)	0.196 (0.083 - 0.391)
70+ years (rate per capita)	0.316 (0.254 - 0.383)	0.313 (0.252 - 0.379)	0.313 (0.252 - 0.379)	0.548 (0.425 - 0.680)	0.544 (0.425 - 0.673)	0.544 (0.426 - 0.673)	0.172 (0.077 - 0.330)	0.171 (0.077 - 0.325)	0.172 (0.077 - 0.327)
All ages (rate per capita)	0.290 (0.256 - 0.324)	0.295 (0.260 - 0.329)	0.297 (0.262 - 0.331)	0.418 (0.367 - 0.473)	0.424 (0.373 - 0.482)	0.427 (0.375 - 0.484)	0.170 (0.073 - 0.324)	0.173 (0.075 - 0.330)	0.175 (0.076 - 0.335)
Age-standardized (rate per capita)	0.337 (0.302 - 0.370)	0.336 (0.302 - 0.370)	0.336 (0.303 - 0.370)	0.517 (0.461 - 0.575)	0.516 (0.462 - 0.575)	0.516 (0.462 - 0.575)	0.196 (0.085 - 0.374)	0.196 (0.085 - 0.374)	0.197 (0.085 - 0.377)
Total number of cases (thousands)	57,662 (50,929 - 64,351)	87,511 (77,091 - 97,567)	116,018 (102,330 - 129,266)	82,966 (72,988 - 93,975)	125,915 (110,640 - 143,053)	166,841 (146,787 - 189,299)	34 (15 - 64)	51 (22 - 98)	68 (30 - 131)

	Prevalence 1990	Prevalence 2005	Prevalence 2015	Incidence 1990	Incidence 2005	Incidence 2015	YLD rate 1990	YLD rate 2005	YLD rate 2015
**Benin**									
**5 -14 years (rate per capita)**	0.216 (0.158 - 0.295)	0.218 (0.159 - 0.297)	0.221 (0.162 - 0.300)	0.241 (0.159 - 0.344)	0.243 (0.160 - 0.348)	0.246 (0.162 - 0.350)	0.129 (0.052 - 0.257)	0.130 (0.053 - 0.263)	0.132 (0.054 - 0.269)
**15 - 49 years (rate per capita)**	0.451 (0.383 - 0.514)	0.450 (0.382 - 0.512)	0.450 (0.382 - 0.512)	0.662 (0.551 - 0.782)	0.663 (0.552 - 0.783)	0.663 (0.553 - 0.783)	0.265 (0.110 - 0.518)	0.265 (0.109 - 0.509)	0.265 (0.110 - 0.516)
**50 - 69 years (rate per capita)**	0.349 (0.279 - 0.428)	0.349 (0.278 - 0.430)	0.349 (0.276 - 0.430)	0.658 (0.513 - 0.821)	0.658 (0.513 - 0.824)	0.658 (0.511 - 0.824)	0.199 (0.085 - 0.395)	0.199 (0.085 - 0.398)	0.200 (0.084 - 0.398)
**70+ years (rate per capita)**	0.320 (0.252 - 0.392)	0.319 (0.252 - 0.391)	0.320 (0.252 - 0.394)	0.555 (0.424 - 0.703)	0.554 (0.424 - 0.700)	0.555 (0.425 - 0.705)	0.173 (0.078 - 0.332)	0.174 (0.078 - 0.339)	0.175 (0.077 - 0.336)
**All ages (rate per capita)**	0.292 (0.255 - 0.329)	0.299 (0.261 - 0.337)	0.307 (0.269 - 0.346)	0.421 (0.366 - 0.482)	0.432 (0.375 - 0.495)	0.445 (0.387 - 0.510)	0.171 (0.073 - 0.329)	0.176 (0.075 - 0.338)	0.181 (0.077 - 0.347)
**Age-standardized (rate per capita)**	0.342 (0.305 - 0.379)	0.342 (0.305 - 0.379)	0.342 (0.305 - 0.379)	0.527 (0.464 - 0.593)	0.527 (0.464 - 0.593)	0.527 (0.464 - 0.592)	0.199 (0.086 - 0.379)	0.199 (0.087 - 0.379)	0.200 (0.086 - 0.379)
**Total number of cases (thousands)**	1,460 (1,276 - 1,648)	2,449 (2,140 - 2,763)	3,353 (2,931 - 3,776)	2,107 (1,831 - 2,411)	3,535 (3,068 - 4,053)	4,859 (4,226 - 5,561)	1 (0 - 2)	1 (1 - 3)	2 (1 - 4)
**Burkina Faso**									
**5 -14 years (rate per capita)**	0.213 (0.166 - 0.277)	0.189 (0.162 - 0.216)	0.189 (0.162 - 0.217)	0.213 (0.158 - 0.284)	0.195 (0.144 - 0.255)	0.195 (0.144 - 0.256)	0.126 (0.053 - 0.245)	0.112 (0.047 - 0.213)	0.113 (0.047 - 0.218)
**15 - 49 years (rate per capita)**	0.450 (0.411 - 0.490)	0.451 (0.412 - 0.491)	0.450 (0.411 - 0.490)	0.609 (0.541 - 0.679)	0.610 (0.546 - 0.680)	0.611 (0.548 - 0.680)	0.264 (0.115 - 0.517)	0.265 (0.117 - 0.522)	0.267 (0.116 - 0.526)
**50 - 69 years (rate per capita)**	0.336 (0.294 - 0.380)	0.336 (0.296 - 0.381)	0.335 (0.295 - 0.381)	0.624 (0.546 - 0.712)	0.625 (0.546 - 0.715)	0.625 (0.545 - 0.716)	0.191 (0.082 - 0.371)	0.192 (0.082 - 0.377)	0.193 (0.082 - 0.383)
**70+ years (rate per capita)**	0.316 (0.278 - 0.358)	0.315 (0.279 - 0.354)	0.316 (0.279 - 0.356)	0.531 (0.462 - 0.606)	0.529 (0.462 - 0.598)	0.530 (0.463 - 0.599)	0.172 (0.076 - 0.331)	0.173 (0.077 - 0.328)	0.175 (0.077 - 0.332)
**All ages (rate per capita)**	0.286 (0.263 - 0.312)	0.284 (0.263 - 0.306)	0.289 (0.268 - 0.311)	0.381 (0.347 - 0.422)	0.380 (0.346 - 0.416)	0.388 (0.353 - 0.424)	0.168 (0.072 - 0.322)	0.167 (0.074 - 0.325)	0.171 (0.075 - 0.329)
**Age-standardized (rate per capita)**	0.337 (0.314 - 0.360)	0.333 (0.312 - 0.356)	0.333 (0.312 - 0.355)	0.488 (0.452 - 0.527)	0.485 (0.450 - 0.519)	0.485 (0.450 - 0.519)	0.196 (0.085 - 0.380)	0.195 (0.085 - 0.375)	0.196 (0.085 - 0.378)
**Total number of cases (thousands)**	2,523 (2,313 - 2,750)	3,813 (3,540 - 4,108)	5,223 (4,849 - 5,621)	3,361 (3,054 - 3,722)	5,112 (4,647 - 5,592)	7,019 (6,389 - 7,673)	1 (1 - 3)	2 (1 - 4)	3 (1 - 6)
**Cameroon**									
**5 -14 years (rate per capita)**	0.214 (0.156 - 0.292)	0.221 (0.161 - 0.300)	0.220 (0.161 - 0.299)	0.239 (0.157 - 0.342)	0.246 (0.162 - 0.350)	0.245 (0.161 - 0.349)	0.127 (0.052 - 0.254)	0.131 (0.053 - 0.264)	0.131 (0.053 - 0.266)
**15 - 49 years (rate per capita)**	0.451 (0.383 - 0.513)	0.451 (0.382 - 0.516)	0.449 (0.381 - 0.513)	0.662 (0.551 - 0.781)	0.661 (0.549 - 0.785)	0.664 (0.552 - 0.787)	0.265 (0.110 - 0.514)	0.265 (0.110 - 0.515)	0.265 (0.110 - 0.515)
**50 - 69 years (rate per capita)**	0.349 (0.277 - 0.429)	0.349 (0.278 - 0.429)	0.348 (0.278 - 0.429)	0.657 (0.510 - 0.823)	0.657 (0.510 - 0.823)	0.656 (0.510 - 0.821)	0.198 (0.083 - 0.392)	0.198 (0.085 - 0.400)	0.199 (0.085 - 0.398)
**70+ years (rate per capita)**	0.316 (0.252 - 0.386)	0.315 (0.252 - 0.383)	0.314 (0.252 - 0.381)	0.550 (0.424 - 0.699)	0.548 (0.426 - 0.696)	0.547 (0.427 - 0.694)	0.171 (0.077 - 0.326)	0.170 (0.077 - 0.323)	0.170 (0.077 - 0.322)
**All ages (rate per capita)**	0.289 (0.253 - 0.326)	0.300 (0.262 - 0.339)	0.306 (0.268 - 0.344)	0.418 (0.365 - 0.477)	0.432 (0.375 - 0.497)	0.443 (0.385 - 0.508)	0.169 (0.072 - 0.326)	0.176 (0.075 - 0.336)	0.180 (0.077 - 0.345)
**Age-standardized (rate per capita)**	0.342 (0.305 - 0.379)	0.342 (0.305 - 0.379)	0.341 (0.304 - 0.379)	0.526 (0.464 - 0.592)	0.526 (0.464 - 0.592)	0.526 (0.464 - 0.592)	0.198 (0.086 - 0.381)	0.199 (0.086 - 0.378)	0.199 (0.086 - 0.381)
**Total number of cases (thousands)**	3,493 (3,058 - 3,940)	5,450 (4,755 - 6,145)	7,154 (6,261 - 8,060)	5,055 (4,411 - 5,770)	7,843 (6,802 - 9,015)	10,358 (8,999 - 11,883)	2 (1 - 4)	3 (1 - 6)	4 (2 - 8)
**Cape Verde**									
**5 -14 years (rate per capita)**	0.212 (0.155 - 0.290)	0.235 (0.172 - 0.315)	0.227 (0.166 - 0.307)	0.237 (0.156 - 0.340)	0.260 (0.169 - 0.361)	0.252 (0.165 - 0.355)	0.127 (0.052 - 0.257)	0.140 (0.056 - 0.283)	0.136 (0.055 - 0.273)
**15 - 49 years (rate per capita)**	0.453 (0.381 - 0.522)	0.453 (0.384 - 0.515)	0.446 (0.379 - 0.509)	0.660 (0.546 - 0.789)	0.660 (0.549 - 0.780)	0.667 (0.556 - 0.790)	0.268 (0.110 - 0.520)	0.269 (0.112 - 0.528)	0.264 (0.109 - 0.515)
**50 - 69 years (rate per capita)**	0.350 (0.278 - 0.431)	0.349 (0.282 - 0.425)	0.350 (0.276 - 0.433)	0.660 (0.512 - 0.827)	0.650 (0.511 - 0.803)	0.662 (0.515 - 0.832)	0.201 (0.085 - 0.410)	0.200 (0.086 - 0.401)	0.202 (0.085 - 0.409)
**70+ years (rate per capita)**	0.311 (0.253 - 0.376)	0.318 (0.253 - 0.389)	0.305 (0.252 - 0.364)	0.545 (0.427 - 0.683)	0.552 (0.425 - 0.704)	0.538 (0.427 - 0.665)	0.171 (0.078 - 0.327)	0.175 (0.078 - 0.337)	0.167 (0.076 - 0.317)
**All ages (rate per capita)**	0.287 (0.251 - 0.324)	0.330 (0.288 - 0.371)	0.343 (0.301 - 0.383)	0.416 (0.363 - 0.479)	0.473 (0.412 - 0.540)	0.513 (0.449 - 0.585)	0.170 (0.073 - 0.326)	0.195 (0.083 - 0.372)	0.202 (0.086 - 0.386)
**Age-standardized (rate per capita)**	0.343 (0.305 - 0.380)	0.342 (0.305 - 0.379)	0.342 (0.305 - 0.379)	0.527 (0.464 - 0.592)	0.527 (0.464 - 0.593)	0.527 (0.464 - 0.593)	0.201 (0.086 - 0.384)	0.201 (0.087 - 0.384)	0.201 (0.086 - 0.383)
**Total number of cases (thousands)**	99 (86 - 111)	157 (137 - 176)	178 (157 - 200)	143 (125 - 164)	225 (196 - 257)	267 (234 - 304)	0 (0 - 0)	0 (0 - 0)	0 (0 - 0)
**Chad**									
**5 -14 years (rate per capita)**	0.214 (0.156 - 0.293)	0.215 (0.157 - 0.293)	0.217 (0.159 - 0.296)	0.239 (0.158 - 0.342)	0.240 (0.158 - 0.342)	0.242 (0.160 - 0.346)	0.128 (0.052 - 0.259)	0.128 (0.052 - 0.258)	0.130 (0.052 - 0.265)
**15 - 49 years (rate per capita)**	0.451 (0.383 - 0.513)	0.451 (0.383 - 0.516)	0.452 (0.382 - 0.518)	0.663 (0.552 - 0.782)	0.661 (0.548 - 0.784)	0.661 (0.548 - 0.785)	0.265 (0.111 - 0.514)	0.266 (0.110 - 0.515)	0.266 (0.110 - 0.511)
**50 - 69 years (rate per capita)**	0.349 (0.278 - 0.429)	0.349 (0.278 - 0.428)	0.349 (0.277 - 0.429)	0.657 (0.511 - 0.823)	0.657 (0.510 - 0.820)	0.658 (0.512 - 0.827)	0.199 (0.085 - 0.398)	0.198 (0.085 - 0.402)	0.199 (0.085 - 0.399)
**70+ years (rate per capita)**	0.317 (0.252 - 0.388)	0.316 (0.252 - 0.386)	0.315 (0.252 - 0.384)	0.551 (0.423 - 0.698)	0.550 (0.425 - 0.699)	0.549 (0.425 - 0.699)	0.173 (0.078 - 0.329)	0.171 (0.077 - 0.330)	0.171 (0.077 - 0.325)
**All ages (rate per capita)**	0.285 (0.250 - 0.322)	0.283 (0.246 - 0.321)	0.290 (0.251 - 0.328)	0.412 (0.358 - 0.472)	0.405 (0.350 - 0.465)	0.413 (0.357 - 0.476)	0.168 (0.072 - 0.319)	0.167 (0.071 - 0.319)	0.171 (0.073 - 0.327)
**Age-standardized (rate per capita)**	0.342 (0.305 - 0.379)	0.342 (0.305 - 0.379)	0.341 (0.304 - 0.379)	0.526 (0.464 - 0.592)	0.526 (0.464 - 0.592)	0.526 (0.464 - 0.592)	0.199 (0.086 - 0.380)	0.199 (0.086 - 0.379)	0.199 (0.086 - 0.379)
**Total number of cases (thousands)**	1,700 (1,488 - 1,916)	2,850 (2,478 - 3,224)	4,075 (3,536 - 4,608)	2,454 (2,134 - 2,810)	4,070 (3,520 - 4,676)	5,812 (5,025 - 6,698)	1 (0 - 2)	2 (1 - 3)	2 (1 - 5)
**Cote d'Ivoire**									
**5 -14 years (rate per capita)**	0.216 (0.158 - 0.295)	0.216 (0.158 - 0.295)	0.221 (0.162 - 0.301)	0.241 (0.159 - 0.344)	0.241 (0.159 - 0.344)	0.247 (0.162 - 0.350)	0.129 (0.053 - 0.264)	0.129 (0.053 - 0.262)	0.132 (0.054 - 0.266)
**15 - 49 years (rate per capita)**	0.448 (0.381 - 0.509)	0.449 (0.381 - 0.511)	0.450 (0.382 - 0.512)	0.664 (0.556 - 0.781)	0.663 (0.553 - 0.783)	0.663 (0.551 - 0.782)	0.263 (0.111 - 0.511)	0.264 (0.110 - 0.513)	0.265 (0.110 - 0.515)
**50 - 69 years (rate per capita)**	0.347 (0.275 - 0.428)	0.347 (0.277 - 0.429)	0.347 (0.276 - 0.428)	0.656 (0.508 - 0.826)	0.656 (0.508 - 0.826)	0.655 (0.508 - 0.823)	0.197 (0.084 - 0.391)	0.198 (0.085 - 0.398)	0.199 (0.084 - 0.401)
**70+ years (rate per capita)**	0.316 (0.250 - 0.387)	0.318 (0.249 - 0.391)	0.317 (0.250 - 0.391)	0.549 (0.421 - 0.696)	0.550 (0.418 - 0.696)	0.550 (0.418 - 0.695)	0.170 (0.075 - 0.324)	0.173 (0.078 - 0.333)	0.173 (0.077 - 0.334)
**All ages (rate per capita)**	0.296 (0.259 - 0.334)	0.299 (0.262 - 0.338)	0.307 (0.268 - 0.345)	0.430 (0.375 - 0.491)	0.435 (0.378 - 0.496)	0.444 (0.386 - 0.508)	0.173 (0.074 - 0.333)	0.176 (0.075 - 0.336)	0.180 (0.077 - 0.345)
**Age-standardized (rate per capita)**	0.341 (0.304 - 0.377)	0.341 (0.304 - 0.378)	0.341 (0.304 - 0.378)	0.526 (0.464 - 0.592)	0.526 (0.464 - 0.592)	0.526 (0.464 - 0.592)	0.198 (0.086 - 0.379)	0.198 (0.086 - 0.377)	0.199 (0.086 - 0.380)
**Total number of cases (thousands)**	3,595 (3,149 - 4,058)	5,423 (4,745 - 6,119)	6,954 (6,084 - 7,832)	5,228 (4,562 - 5,965)	7,871 (6,854 - 8,993)	10,070 (8,763 - 11,524)	2 (1 - 4)	3 (1 - 6)	4 (2 - 8)
**The Gambia**									
**5 -14 years (rate per capita)**	0.213 (0.156 - 0.292)	0.214 (0.157 - 0.293)	0.216 (0.158 - 0.295)	0.238 (0.157 - 0.341)	0.239 (0.158 - 0.342)	0.241 (0.159 - 0.344)	0.127 (0.053 - 0.258)	0.128 (0.053 - 0.255)	0.129 (0.052 - 0.267)
**15 - 49 years (rate per capita)**	0.449 (0.380 - 0.515)	0.451 (0.381 - 0.516)	0.451 (0.382 - 0.514)	0.663 (0.550 - 0.791)	0.663 (0.550 - 0.787)	0.663 (0.551 - 0.785)	0.265 (0.110 - 0.516)	0.266 (0.110 - 0.520)	0.267 (0.110 - 0.521)
**50 - 69 years (rate per capita)**	0.347 (0.277 - 0.427)	0.348 (0.277 - 0.428)	0.348 (0.276 - 0.430)	0.654 (0.508 - 0.820)	0.655 (0.508 - 0.818)	0.658 (0.511 - 0.826)	0.199 (0.084 - 0.397)	0.200 (0.085 - 0.406)	0.200 (0.084 - 0.405)
**70+ years (rate per capita)**	0.320 (0.251 - 0.396)	0.316 (0.250 - 0.388)	0.317 (0.251 - 0.389)	0.553 (0.419 - 0.701)	0.549 (0.422 - 0.701)	0.550 (0.421 - 0.698)	0.176 (0.079 - 0.338)	0.174 (0.078 - 0.336)	0.174 (0.078 - 0.335)
**All ages (rate per capita)**	0.289 (0.251 - 0.327)	0.291 (0.253 - 0.329)	0.292 (0.255 - 0.330)	0.417 (0.362 - 0.481)	0.417 (0.361 - 0.480)	0.420 (0.364 - 0.482)	0.171 (0.073 - 0.328)	0.172 (0.073 - 0.330)	0.173 (0.074 - 0.334)
**Age-standardized (rate per capita)**	0.341 (0.304 - 0.378)	0.342 (0.305 - 0.379)	0.342 (0.305 - 0.379)	0.526 (0.464 - 0.592)	0.526 (0.464 - 0.592)	0.526 (0.463 - 0.592)	0.200 (0.086 - 0.384)	0.200 (0.086 - 0.382)	0.200 (0.087 - 0.386)
**Total number of cases (thousands)**	267 (232 - 301)	422 (367 - 477)	585 (510 - 661)	385 (334 - 444)	605 (524 - 696)	840 (728 - 964)	0 (0 - 0)	0 (0 - 0)	0 (0 - 1)
**Ghana**									
**5 -14 years (rate per capita)**	0.141 (0.112 - 0.173)	0.148 (0.117 - 0.185)	0.146 (0.115 - 0.182)	0.181 (0.127 - 0.245)	0.188 (0.128 - 0.255)	0.184 (0.125 - 0.249)	0.084 (0.035 - 0.166)	0.088 (0.037 - 0.171)	0.087 (0.036 - 0.168)
**15 - 49 years (rate per capita)**	0.347 (0.310 - 0.385)	0.347 (0.311 - 0.383)	0.345 (0.309 - 0.380)	0.562 (0.500 - 0.631)	0.563 (0.501 - 0.629)	0.564 (0.502 - 0.630)	0.204 (0.088 - 0.403)	0.204 (0.089 - 0.403)	0.204 (0.088 - 0.405)
**50 - 69 years (rate per capita)**	0.249 (0.213 - 0.290)	0.249 (0.213 - 0.288)	0.249 (0.213 - 0.290)	0.550 (0.477 - 0.630)	0.549 (0.476 - 0.637)	0.549 (0.477 - 0.636)	0.142 (0.060 - 0.279)	0.142 (0.061 - 0.278)	0.143 (0.061 - 0.279)
**70+ years (rate per capita)**	0.228 (0.197 - 0.264)	0.225 (0.196 - 0.259)	0.223 (0.195 - 0.255)	0.468 (0.406 - 0.534)	0.461 (0.399 - 0.533)	0.458 (0.397 - 0.527)	0.125 (0.056 - 0.241)	0.124 (0.055 - 0.235)	0.123 (0.056 - 0.236)
**All ages (rate per capita)**	0.223 (0.202 - 0.245)	0.234 (0.213 - 0.256)	0.235 (0.214 - 0.257)	0.362 (0.327 - 0.402)	0.381 (0.344 - 0.422)	0.386 (0.349 - 0.425)	0.131 (0.056 - 0.256)	0.138 (0.060 - 0.268)	0.139 (0.060 - 0.270)
**Age-standardized (rate per capita)**	0.253 (0.233 - 0.274)	0.254 (0.234 - 0.274)	0.254 (0.234 - 0.274)	0.440 (0.404 - 0.476)	0.440 (0.404 - 0.476)	0.440 (0.404 - 0.476)	0.147 (0.063 - 0.286)	0.148 (0.064 - 0.291)	0.149 (0.064 - 0.290)
**Total number of cases (thousands)**	3,267 (2,953 - 3,588)	5,007 (4,550 - 5,484)	6,445 (5,869 - 7,033)	5,299 (4,782 - 5,877)	8,143 (7,351 - 9,016)	10,575 (9,581 - 11,653)	2 (1 - 4)	3 (1 - 6)	4 (2 - 7)
**Guinea**									
**5 -14 years (rate per capita)**	0.216 (0.158 - 0.295)	0.218 (0.159 - 0.297)	0.220 (0.161 - 0.299)	0.241 (0.159 - 0.344)	0.243 (0.160 - 0.348)	0.245 (0.161 - 0.349)	0.128 (0.052 - 0.256)	0.130 (0.053 - 0.260)	0.131 (0.053 - 0.263)
**15 - 49 years (rate per capita)**	0.449 (0.382 - 0.511)	0.450 (0.382 - 0.512)	0.450 (0.381 - 0.512)	0.664 (0.555 - 0.784)	0.662 (0.552 - 0.781)	0.663 (0.551 - 0.784)	0.264 (0.110 - 0.513)	0.265 (0.110 - 0.514)	0.265 (0.110 - 0.518)
**50 - 69 years (rate per capita)**	0.349 (0.278 - 0.429)	0.349 (0.278 - 0.428)	0.349 (0.277 - 0.429)	0.657 (0.511 - 0.824)	0.656 (0.510 - 0.820)	0.658 (0.512 - 0.828)	0.199 (0.086 - 0.402)	0.200 (0.085 - 0.403)	0.200 (0.086 - 0.404)
**70+ years (rate per capita)**	0.322 (0.252 - 0.398)	0.319 (0.251 - 0.392)	0.319 (0.251 - 0.391)	0.556 (0.421 - 0.707)	0.553 (0.422 - 0.704)	0.553 (0.422 - 0.704)	0.176 (0.079 - 0.337)	0.175 (0.079 - 0.333)	0.175 (0.079 - 0.338)
**All ages (rate per capita)**	0.297 (0.260 - 0.333)	0.298 (0.261 - 0.337)	0.306 (0.268 - 0.345)	0.433 (0.378 - 0.495)	0.432 (0.377 - 0.494)	0.444 (0.387 - 0.508)	0.174 (0.075 - 0.332)	0.175 (0.074 - 0.336)	0.180 (0.077 - 0.345)
**Age-standardized (rate per capita)**	0.342 (0.305 - 0.379)	0.341 (0.305 - 0.379)	0.341 (0.304 - 0.378)	0.526 (0.464 - 0.592)	0.526 (0.464 - 0.592)	0.526 (0.464 - 0.592)	0.199 (0.087 - 0.380)	0.199 (0.087 - 0.380)	0.200 (0.086 - 0.382)

	Prevalence 1990	Prevalence 2005	Prevalence 2015	Incidence 1990	Incidence 2005	Incidence 2015	YLD rate 1990	YLD rate 2005	YLD rate 2015
Total number of cases (thousands)	1,789 (1,570 - 2,011)	2,895 (2,530 - 3,265)	3,846 (3,371 - 4,332)	2,614 (2,282 - 2,984)	4,195 (3,653 - 4,788)	5,588 (4,866 - 6,391)	1 (0 - 2)	2 (1 - 3)	2 (1 - 4)
**Guinea-Bissau**									
5 -14 years (rate per capita)	0.272 (0.204 - 0.354)	0.261 (0.199 - 0.338)	0.259 (0.197 - 0.336)	0.242 (0.178 - 0.344)	0.238 (0.176 - 0.328)	0.237 (0.175 - 0.327)	0.162 (0.066 - 0.320)	0.155 (0.064 - 0.306)	0.154 (0.063 - 0.310)
15 - 49 years (rate per capita)	0.495 (0.453 - 0.535)	0.495 (0.454 - 0.534)	0.494 (0.452 - 0.532)	0.627 (0.567 - 0.696)	0.626 (0.564 - 0.695)	0.629 (0.566 - 0.699)	0.291 (0.128 - 0.575)	0.292 (0.128 - 0.579)	0.291 (0.127 - 0.567)
50 - 69 years (rate per capita)	0.384 (0.337 - 0.436)	0.384 (0.335 - 0.441)	0.384 (0.336 - 0.439)	0.652 (0.574 - 0.744)	0.652 (0.572 - 0.747)	0.653 (0.573 - 0.746)	0.219 (0.095 - 0.433)	0.219 (0.095 - 0.428)	0.219 (0.096 - 0.431)
70+ years (rate per capita)	0.364 (0.316 - 0.412)	0.358 (0.316 - 0.405)	0.360 (0.316 - 0.409)	0.555 (0.480 - 0.632)	0.552 (0.485 - 0.627)	0.554 (0.486 - 0.631)	0.199 (0.088 - 0.383)	0.196 (0.088 - 0.376)	0.197 (0.088 - 0.382)
All ages (rate per capita)	0.333 (0.305 - 0.362)	0.340 (0.311 - 0.369)	0.345 (0.317 - 0.373)	0.412 (0.378 - 0.450)	0.422 (0.386 - 0.463)	0.431 (0.395 - 0.474)	0.196 (0.085 - 0.372)	0.200 (0.087 - 0.381)	0.203 (0.088 - 0.387)
Age-standardized (rate per capita)	0.383 (0.358 - 0.410)	0.380 (0.354 - 0.405)	0.380 (0.354 - 0.405)	0.508 (0.471 - 0.545)	0.506 (0.470 - 0.544)	0.506 (0.470 - 0.544)	0.224 (0.097 - 0.433)	0.222 (0.096 - 0.427)	0.222 (0.097 - 0.425)
Total number of cases (thousands)	352 (322 - 383)	499 (457 - 542)	637 (586 - 690)	436 (400 - 476)	619 (567 - 680)	798 (729 - 876)	0 (0 - 0)	0 (0 - 1)	0 (0 - 1)
**Liberia**									
5 -14 years (rate per capita)	0.217 (0.158 - 0.296)	0.215 (0.157 - 0.293)	0.221 (0.161 - 0.300)	0.242 (0.160 - 0.345)	0.240 (0.158 - 0.343)	0.246 (0.162 - 0.350)	0.129 (0.053 - 0.258)	0.128 (0.051 - 0.255)	0.131 (0.053 - 0.265)
15 - 49 years (rate per capita)	0.450 (0.383 - 0.512)	0.450 (0.381 - 0.512)	0.449 (0.382 - 0.511)	0.662 (0.552 - 0.781)	0.663 (0.551 - 0.784)	0.664 (0.554 - 0.783)	0.263 (0.109 - 0.508)	0.262 (0.108 - 0.506)	0.264 (0.108 - 0.511)
50 - 69 years (rate per capita)	0.349 (0.277 - 0.429)	0.349 (0.278 - 0.428)	0.349 (0.277 - 0.430)	0.657 (0.511 - 0.824)	0.656 (0.510 - 0.821)	0.658 (0.512 - 0.826)	0.197 (0.084 - 0.388)	0.197 (0.085 - 0.396)	0.197 (0.084 - 0.390)
70+ years (rate per capita)	0.321 (0.251 - 0.397)	0.320 (0.252 - 0.394)	0.319 (0.251 - 0.393)	0.555 (0.419 - 0.705)	0.554 (0.422 - 0.705)	0.553 (0.422 - 0.704)	0.172 (0.076 - 0.329)	0.171 (0.077 - 0.328)	0.172 (0.078 - 0.335)
All ages (rate per capita)	0.291 (0.254 - 0.329)	0.300 (0.263 - 0.338)	0.307 (0.269 - 0.346)	0.421 (0.367 - 0.481)	0.436 (0.379 - 0.500)	0.446 (0.388 - 0.510)	0.170 (0.073 - 0.325)	0.175 (0.075 - 0.336)	0.180 (0.077 - 0.344)
Age-standardized (rate per capita)	0.342 (0.305 - 0.379)	0.341 (0.305 - 0.379)	0.341 (0.304 - 0.378)	0.526 (0.464 - 0.592)	0.526 (0.464 - 0.592)	0.526 (0.464 - 0.592)	0.197 (0.086 - 0.376)	0.197 (0.086 - 0.376)	0.198 (0.086 - 0.377)
Total number of cases (thousands)	622 (543 - 702)	982 (859 - 1,105)	1,385 (1,213 - 1,559)	900 (784 - 1,028)	1,426 (1,240 - 1,635)	2,011 (1,750 - 2,299)	0 (0 - 1)	1 (0 - 1)	1 (0 - 2)
**Mali**									
5 -14 years (rate per capita)	0.218 (0.159 - 0.297)	0.217 (0.158 - 0.295)	0.214 (0.156 - 0.293)	0.244 (0.160 - 0.348)	0.242 (0.159 - 0.345)	0.240 (0.158 - 0.342)	0.130 (0.053 - 0.260)	0.129 (0.052 - 0.259)	0.128 (0.052 - 0.261)
15 - 49 years (rate per capita)	0.452 (0.384 - 0.515)	0.450 (0.381 - 0.516)	0.451 (0.382 - 0.513)	0.660 (0.549 - 0.780)	0.662 (0.549 - 0.787)	0.663 (0.551 - 0.784)	0.265 (0.110 - 0.514)	0.265 (0.109 - 0.515)	0.266 (0.111 - 0.518)
50 - 69 years (rate per capita)	0.349 (0.279 - 0.428)	0.349 (0.278 - 0.429)	0.349 (0.277 - 0.430)	0.656 (0.511 - 0.819)	0.657 (0.512 - 0.822)	0.658 (0.513 - 0.825)	0.198 (0.085 - 0.399)	0.199 (0.085 - 0.404)	0.200 (0.086 - 0.395)
70+ years (rate per capita)	0.322 (0.251 - 0.399)	0.317 (0.251 - 0.388)	0.318 (0.251 - 0.390)	0.556 (0.419 - 0.706)	0.551 (0.424 - 0.701)	0.552 (0.423 - 0.702)	0.175 (0.078 - 0.334)	0.172 (0.077 - 0.331)	0.174 (0.078 - 0.331)
All ages (rate per capita)	0.290 (0.253 - 0.327)	0.290 (0.253 - 0.328)	0.290 (0.253 - 0.328)	0.417 (0.364 - 0.476)	0.417 (0.362 - 0.481)	0.416 (0.360 - 0.478)	0.170 (0.072 - 0.325)	0.170 (0.072 - 0.325)	0.171 (0.073 - 0.331)
Age-standardized (rate per capita)	0.342 (0.305 - 0.379)	0.342 (0.305 - 0.378)	0.341 (0.304 - 0.378)	0.526 (0.464 - 0.592)	0.527 (0.464 - 0.592)	0.527 (0.464 - 0.592)	0.198 (0.086 - 0.377)	0.199 (0.086 - 0.378)	0.200 (0.086 - 0.383)
Total number of cases (thousands)	2,465 (2,155 - 2,780)	3,744 (3,260 - 4,225)	5,102 (4,446 - 5,763)	3,544 (3,094 - 4,049)	5,383 (4,666 - 6,199)	7,306 (6,321 - 8,405)	1 (1 - 3)	2 (1 - 4)	3 (1 - 6)
**Mauritania**									
5 -14 years (rate per capita)	0.219 (0.160 - 0.298)	0.220 (0.161 - 0.299)	0.221 (0.162 - 0.300)	0.244 (0.161 - 0.349)	0.245 (0.161 - 0.349)	0.246 (0.162 - 0.350)	0.131 (0.054 - 0.266)	0.131 (0.054 - 0.267)	0.132 (0.053 - 0.268)
15 - 49 years (rate per capita)	0.451 (0.382 - 0.514)	0.449 (0.381 - 0.511)	0.449 (0.382 - 0.509)	0.662 (0.551 - 0.785)	0.664 (0.553 - 0.785)	0.665 (0.557 - 0.783)	0.265 (0.111 - 0.509)	0.265 (0.110 - 0.513)	0.265 (0.109 - 0.518)
50 - 69 years (rate per capita)	0.349 (0.278 - 0.429)	0.349 (0.278 - 0.429)	0.349 (0.276 - 0.430)	0.658 (0.512 - 0.824)	0.657 (0.511 - 0.822)	0.658 (0.511 - 0.825)	0.199 (0.085 - 0.392)	0.199 (0.086 - 0.398)	0.200 (0.084 - 0.397)
70+ years (rate per capita)	0.320 (0.252 - 0.394)	0.318 (0.252 - 0.390)	0.318 (0.253 - 0.388)	0.554 (0.423 - 0.707)	0.553 (0.423 - 0.701)	0.552 (0.425 - 0.701)	0.174 (0.078 - 0.334)	0.173 (0.078 - 0.330)	0.173 (0.078 - 0.332)
All ages (rate per capita)	0.298 (0.261 - 0.336)	0.307 (0.269 - 0.346)	0.312 (0.274 - 0.350)	0.430 (0.373 - 0.494)	0.446 (0.389 - 0.511)	0.456 (0.398 - 0.519)	0.175 (0.075 - 0.335)	0.181 (0.078 - 0.347)	0.184 (0.079 - 0.352)
Age-standardized (rate per capita)	0.342 (0.305 - 0.379)	0.341 (0.304 - 0.379)	0.341 (0.304 - 0.379)	0.527 (0.464 - 0.592)	0.527 (0.464 - 0.592)	0.527 (0.464 - 0.592)	0.199 (0.087 - 0.378)	0.199 (0.087 - 0.380)	0.200 (0.086 - 0.382)
Total number of cases (thousands)	605 (529 - 682)	971 (850 - 1,093)	1,275 (1,120 - 1,432)	873 (758 - 1,002)	1,413 (1,229 - 1,616)	1,864 (1,627 - 2,119)	0 (0 - 1)	1 (0 - 1)	1 (0 - 1)
**Niger**									
5 -14 years (rate per capita)	0.267 (0.205 - 0.344)	0.259 (0.199 - 0.336)	0.260 (0.200 - 0.337)	0.241 (0.178 - 0.347)	0.235 (0.174 - 0.343)	0.236 (0.174 - 0.344)	0.159 (0.065 - 0.315)	0.154 (0.064 - 0.303)	0.155 (0.064 - 0.309)
15 - 49 years (rate per capita)	0.502 (0.463 - 0.540)	0.502 (0.464 - 0.541)	0.505 (0.465 - 0.544)	0.629 (0.566 - 0.696)	0.627 (0.565 - 0.692)	0.624 (0.562 - 0.693)	0.296 (0.132 - 0.580)	0.297 (0.130 - 0.581)	0.299 (0.131 - 0.588)
50 - 69 years (rate per capita)	0.391 (0.345 - 0.442)	0.390 (0.344 - 0.441)	0.389 (0.343 - 0.440)	0.660 (0.575 - 0.756)	0.659 (0.575 - 0.755)	0.658 (0.572 - 0.753)	0.224 (0.097 - 0.435)	0.224 (0.097 - 0.437)	0.224 (0.097 - 0.436)
70+ years (rate per capita)	0.375 (0.329 - 0.421)	0.370 (0.327 - 0.415)	0.370 (0.327 - 0.415)	0.563 (0.491 - 0.640)	0.559 (0.487 - 0.634)	0.559 (0.488 - 0.635)	0.206 (0.092 - 0.396)	0.203 (0.091 - 0.389)	0.203 (0.091 - 0.389)
All ages (rate per capita)	0.326 (0.301 - 0.354)	0.319 (0.294 - 0.346)	0.317 (0.292 - 0.346)	0.397 (0.364 - 0.437)	0.388 (0.355 - 0.427)	0.382 (0.349 - 0.420)	0.192 (0.083 - 0.362)	0.188 (0.082 - 0.356)	0.187 (0.081 - 0.353)
Age-standardized (rate per capita)	0.387 (0.362 - 0.411)	0.386 (0.362 - 0.410)	0.386 (0.362 - 0.410)	0.509 (0.472 - 0.547)	0.508 (0.472 - 0.547)	0.508 (0.472 - 0.547)	0.226 (0.098 - 0.435)	0.226 (0.099 - 0.432)	0.226 (0.099 - 0.435)
Total number of cases (thousands)	2,585 (2,386 - 2,804)	4,296 (3,960 - 4,661)	6,292 (5,793 - 6,863)	3,146 (2,884 - 3,457)	5,224 (4,779 - 5,741)	7,583 (6,927 - 8,345)	2 (1 - 3)	3 (1 - 5)	4 (2 - 7)
**Nigeria**									
5 -14 years (rate per capita)	0.218 (0.159 - 0.296)	0.217 (0.158 - 0.296)	0.218 (0.159 - 0.296)	0.243 (0.160 - 0.347)	0.242 (0.160 - 0.346)	0.243 (0.160 - 0.347)	0.130 (0.053 - 0.257)	0.129 (0.052 - 0.258)	0.130 (0.053 - 0.259)
15 - 49 years (rate per capita)	0.450 (0.382 - 0.511)	0.449 (0.381 - 0.512)	0.449 (0.382 - 0.511)	0.663 (0.554 - 0.780)	0.663 (0.551 - 0.784)	0.664 (0.554 - 0.784)	0.264 (0.109 - 0.511)	0.264 (0.110 - 0.512)	0.265 (0.109 - 0.517)
50 - 69 years (rate per capita)	0.349 (0.277 - 0.429)	0.348 (0.277 - 0.429)	0.348 (0.277 - 0.429)	0.657 (0.511 - 0.825)	0.658 (0.511 - 0.826)	0.657 (0.510 - 0.824)	0.199 (0.085 - 0.398)	0.199 (0.084 - 0.396)	0.200 (0.086 - 0.403)
70+ years (rate per capita)	0.322 (0.251 - 0.398)	0.321 (0.251 - 0.397)	0.321 (0.251 - 0.397)	0.557 (0.420 - 0.707)	0.555 (0.420 - 0.705)	0.555 (0.419 - 0.705)	0.176 (0.078 - 0.339)	0.175 (0.078 - 0.338)	0.176 (0.078 - 0.337)
All ages (rate per capita)	0.296 (0.259 - 0.334)	0.300 (0.262 - 0.338)	0.300 (0.262 - 0.338)	0.429 (0.374 - 0.490)	0.435 (0.379 - 0.499)	0.435 (0.378 - 0.497)	0.174 (0.074 - 0.332)	0.176 (0.075 - 0.339)	0.176 (0.076 - 0.340)
Age-standardized (rate per capita)	0.341 (0.304 - 0.378)	0.341 (0.304 - 0.378)	0.341 (0.304 - 0.378)	0.526 (0.464 - 0.592)	0.526 (0.464 - 0.592)	0.526 (0.464 - 0.592)	0.199 (0.086 - 0.378)	0.199 (0.086 - 0.378)	0.199 (0.087 - 0.381)
Total number of cases (thousands)	28,361 (24,812 - 32,019)	41,897 (36,664 - 47,224)	54,690 (47,899 - 61,618)	41,083 (35,839 - 46,937)	60,814 (52,875 - 69,723)	79,310 (68,907 - 90,787)	17 (7 - 32)	25 (10 - 47)	32 (14 - 62)
**Sao Tome and Principe**									
5 -14 years (rate per capita)	0.231 (0.169 - 0.311)	0.219 (0.160 - 0.298)	0.223 (0.163 - 0.302)	0.256 (0.167 - 0.358)	0.244 (0.161 - 0.349)	0.248 (0.163 - 0.351)	0.138 (0.056 - 0.283)	0.131 (0.053 - 0.260)	0.133 (0.054 - 0.273)
15 - 49 years (rate per capita)	0.453 (0.383 - 0.520)	0.451 (0.381 - 0.517)	0.449 (0.382 - 0.512)	0.659 (0.545 - 0.785)	0.663 (0.551 - 0.786)	0.665 (0.552 - 0.784)	0.268 (0.111 - 0.520)	0.267 (0.111 - 0.519)	0.266 (0.110 - 0.516)
50 - 69 years (rate per capita)	0.348 (0.278 - 0.427)	0.348 (0.281 - 0.425)	0.349 (0.276 - 0.431)	0.656 (0.510 - 0.818)	0.651 (0.507 - 0.803)	0.660 (0.514 - 0.828)	0.200 (0.086 - 0.406)	0.200 (0.085 - 0.405)	0.201 (0.085 - 0.403)
70+ years (rate per capita)	0.310 (0.252 - 0.375)	0.311 (0.252 - 0.376)	0.305 (0.252 - 0.365)	0.544 (0.426 - 0.683)	0.544 (0.426 - 0.685)	0.538 (0.426 - 0.663)	0.171 (0.077 - 0.327)	0.171 (0.077 - 0.324)	0.167 (0.076 - 0.318)
All ages (rate per capita)	0.292 (0.255 - 0.331)	0.302 (0.263 - 0.341)	0.307 (0.268 - 0.345)	0.418 (0.363 - 0.480)	0.434 (0.377 - 0.498)	0.444 (0.387 - 0.507)	0.172 (0.075 - 0.334)	0.178 (0.076 - 0.340)	0.181 (0.077 - 0.346)
Age-standardized (rate per capita)	0.342 (0.305 - 0.379)	0.342 (0.305 - 0.379)	0.342 (0.305 - 0.379)	0.526 (0.464 - 0.592)	0.527 (0.464 - 0.592)	0.527 (0.464 - 0.592)	0.200 (0.087 - 0.382)	0.200 (0.087 - 0.382)	0.200 (0.087 - 0.383)
Total number of cases (thousands)	33 (29 - 38)	46 (40 - 52)	59 (51 - 66)	48 (41 - 55)	67 (58 - 77)	85 (74 - 97)	0 (0 - 0)	0 (0 - 0)	0 (0 - 0)
**Senegal**									
5 -14 years (rate per capita)	0.218 (0.159 - 0.297)	0.221 (0.162 - 0.301)	0.218 (0.159 - 0.296)	0.243 (0.160 - 0.348)	0.246 (0.162 - 0.350)	0.243 (0.160 - 0.347)	0.130 (0.053 - 0.266)	0.132 (0.053 - 0.279)	0.130 (0.053 - 0.257)
15 - 49 years (rate per capita)	0.452 (0.384 - 0.515)	0.451 (0.382 - 0.517)	0.450 (0.382 - 0.513)	0.661 (0.549 - 0.781)	0.662 (0.549 - 0.786)	0.664 (0.552 - 0.786)	0.267 (0.111 - 0.525)	0.267 (0.110 - 0.517)	0.266 (0.111 - 0.518)
50 - 69 years (rate per capita)	0.348 (0.277 - 0.429)	0.349 (0.279 - 0.429)	0.349 (0.278 - 0.430)	0.657 (0.511 - 0.825)	0.656 (0.510 - 0.819)	0.659 (0.513 - 0.824)	0.201 (0.087 - 0.403)	0.201 (0.086 - 0.409)	0.202 (0.086 - 0.406)
70+ years (rate per capita)	0.321 (0.252 - 0.396)	0.319 (0.252 - 0.391)	0.317 (0.253 - 0.388)	0.556 (0.422 - 0.707)	0.553 (0.422 - 0.704)	0.552 (0.425 - 0.702)	0.178 (0.079 - 0.343)	0.176 (0.078 - 0.342)	0.175 (0.079 - 0.335)
All ages (rate per capita)	0.291 (0.254 - 0.329)	0.302 (0.263 - 0.341)	0.302 (0.263 - 0.340)	0.417 (0.363 - 0.478)	0.434 (0.375 - 0.499)	0.436 (0.379 - 0.500)	0.171 (0.074 - 0.330)	0.178 (0.076 - 0.341)	0.178 (0.076 - 0.342)
Age-standardized (rate per capita)	0.342 (0.305 - 0.379)	0.342 (0.305 - 0.379)	0.342 (0.305 - 0.379)	0.526 (0.464 - 0.592)	0.527 (0.464 - 0.592)	0.527 (0.464 - 0.593)	0.200 (0.087 - 0.384)	0.200 (0.086 - 0.383)	0.201 (0.087 - 0.383)
Total number of cases (thousands)	2,183 (1,906 - 2,467)	3,408 (2,966 - 3,846)	4,556 (3,980 - 5,132)	3,134 (2,726 - 3,591)	4,895 (4,237 - 5,636)	6,589 (5,722 - 7,562)	1 (1 - 2)	2 (1 - 4)	3 (1 - 5)
**Sierra Leone**									
5 -14 years (rate per capita)	0.206 (0.168 - 0.248)	0.211 (0.166 - 0.274)	0.213 (0.168 - 0.276)	0.211 (0.159 - 0.272)	0.214 (0.155 - 0.288)	0.215 (0.156 - 0.290)	0.123 (0.053 - 0.236)	0.125 (0.053 - 0.246)	0.127 (0.053 - 0.252)
15 - 49 years (rate per capita)	0.451 (0.413 - 0.488)	0.448 (0.409 - 0.489)	0.448 (0.409 - 0.488)	0.610 (0.549 - 0.678)	0.611 (0.545 - 0.680)	0.612 (0.547 - 0.681)	0.265 (0.114 - 0.521)	0.262 (0.115 - 0.516)	0.264 (0.115 - 0.523)
50 - 69 years (rate per capita)	0.334 (0.289 - 0.382)	0.334 (0.291 - 0.384)	0.334 (0.291 - 0.384)	0.622 (0.545 - 0.711)	0.622 (0.540 - 0.713)	0.622 (0.540 - 0.711)	0.190 (0.081 - 0.374)	0.190 (0.083 - 0.367)	0.191 (0.083 - 0.372)
70+ years (rate per capita)	0.318 (0.275 - 0.365)	0.320 (0.274 - 0.368)	0.317 (0.273 - 0.363)	0.532 (0.457 - 0.617)	0.533 (0.459 - 0.608)	0.531 (0.458 - 0.605)	0.174 (0.079 - 0.338)	0.173 (0.078 - 0.332)	0.174 (0.079 - 0.335)
All ages (rate per capita)	0.293 (0.273 - 0.314)	0.298 (0.274 - 0.324)	0.304 (0.280 - 0.330)	0.397 (0.364 - 0.432)	0.402 (0.365 - 0.443)	0.410 (0.373 - 0.451)	0.172 (0.075 - 0.332)	0.174 (0.077 - 0.331)	0.179 (0.078 - 0.341)

	Prevalence 1990	Prevalence 2005	Prevalence 2015	Incidence 1990	Incidence 2005	Incidence 2015	YLD rate 1990	YLD rate 2005	YLD rate 2015
Age-standardized (rate per capita)	0.337 (0.315 - 0.357)	0.336 (0.314 - 0.359)	0.336 (0.313 - 0.359)	0.486 (0.451 - 0.523)	0.487 (0.451 - 0.524)	0.487 (0.451 - 0.524)	0.196 (0.085 - 0.378)	0.195 (0.085 - 0.374)	0.196 (0.085 - 0.378)
Total number of cases (thousands)	1,150 (1,070 - 1,231)	1,504 (1,385 - 1,636)	1,963 (1,807 - 2,135)	1,556 (1,429 - 1,694)	2,026 (1,844 - 2,234)	2,647 (2,411 - 2,914)	1 (0 - 1)	1 (0 - 2)	1 (1 - 2)
**Togo**									
5 -14 years (rate per capita)	0.218 (0.159 - 0.296)	0.221 (0.161 - 0.300)	0.219 (0.160 - 0.298)	0.243 (0.160 - 0.346)	0.246 (0.162 - 0.350)	0.244 (0.161 - 0.349)	0.130 (0.053 - 0.260)	0.132 (0.054 - 0.264)	0.131 (0.054 - 0.266)
15 - 49 years (rate per capita)	0.451 (0.383 - 0.514)	0.451 (0.382 - 0.515)	0.449 (0.382 - 0.512)	0.662 (0.549 - 0.781)	0.662 (0.550 - 0.786)	0.665 (0.553 - 0.786)	0.266 (0.110 - 0.518)	0.266 (0.110 - 0.516)	0.266 (0.109 - 0.521)
50 - 69 years (rate per capita)	0.349 (0.277 - 0.430)	0.349 (0.277 - 0.430)	0.349 (0.277 - 0.430)	0.657 (0.511 - 0.824)	0.658 (0.512 - 0.825)	0.658 (0.512 - 0.824)	0.200 (0.085 - 0.401)	0.200 (0.085 - 0.404)	0.201 (0.084 - 0.410)
70+ years (rate per capita)	0.321 (0.252 - 0.394)	0.320 (0.252 - 0.393)	0.320 (0.252 - 0.393)	0.555 (0.423 - 0.708)	0.554 (0.423 - 0.706)	0.554 (0.423 - 0.706)	0.175 (0.077 - 0.341)	0.175 (0.078 - 0.335)	0.176 (0.079 - 0.337)
All ages (rate per capita)	0.294 (0.257 - 0.332)	0.304 (0.265 - 0.343)	0.307 (0.269 - 0.346)	0.422 (0.367 - 0.483)	0.438 (0.380 - 0.504)	0.446 (0.388 - 0.510)	0.173 (0.074 - 0.332)	0.179 (0.077 - 0.340)	0.182 (0.078 - 0.347)
Age-standardized (rate per capita)	0.342 (0.305 - 0.379)	0.342 (0.305 - 0.379)	0.342 (0.305 - 0.379)	0.527 (0.464 - 0.592)	0.526 (0.464 - 0.592)	0.527 (0.464 - 0.592)	0.200 (0.087 - 0.383)	0.200 (0.086 - 0.381)	0.200 (0.086 - 0.383)
Total number of cases (thousands)	1,112 (971 - 1,255)	1,698 (1,481 - 1,915)	2,244 (1,964 - 2,530)	1,598 (1,388 - 1,830)	2,448 (2,120 - 2,814)	3,259 (2,833 - 3,726)	1 (0 - 1)	1 (0 - 2)	1 (1 - 3)
**Eastern Sub-Saharan Africa**									
5 -14 years (rate per capita)	0.225 (0.172 - 0.288)	0.244 (0.186 - 0.312)	0.236 (0.175 - 0.306)	0.288 (0.197 - 0.394)	0.306 (0.217 - 0.418)	0.299 (0.204 - 0.415)	0.134 (0.057 - 0.263)	0.146 (0.062 - 0.289)	0.141 (0.059 - 0.280)
15 - 49 years (rate per capita)	0.451 (0.400 - 0.503)	0.449 (0.397 - 0.501)	0.450 (0.398 - 0.502)	0.717 (0.623 - 0.805)	0.712 (0.620 - 0.798)	0.716 (0.625 - 0.804)	0.266 (0.114 - 0.525)	0.265 (0.113 - 0.520)	0.266 (0.114 - 0.523)
50 - 69 years (rate per capita)	0.396 (0.330 - 0.466)	0.395 (0.328 - 0.466)	0.394 (0.330 - 0.464)	0.754 (0.620 - 0.883)	0.751 (0.621 - 0.878)	0.749 (0.619 - 0.875)	0.227 (0.098 - 0.459)	0.227 (0.099 - 0.458)	0.227 (0.098 - 0.456)
70+ years (rate per capita)	0.371 (0.309 - 0.442)	0.369 (0.309 - 0.437)	0.370 (0.311 - 0.438)	0.682 (0.550 - 0.814)	0.679 (0.550 - 0.807)	0.682 (0.553 - 0.814)	0.204 (0.092 - 0.396)	0.203 (0.091 - 0.390)	0.204 (0.092 - 0.395)
All ages (rate per capita)	0.297 (0.269 - 0.330)	0.307 (0.277 - 0.340)	0.312 (0.282 - 0.345)	0.464 (0.414 - 0.513)	0.475 (0.423 - 0.527)	0.487 (0.434 - 0.540)	0.175 (0.076 - 0.335)	0.181 (0.079 - 0.347)	0.184 (0.081 - 0.354)
Age-standardized (rate per capita)	0.358 (0.326 - 0.388)	0.360 (0.329 - 0.389)	0.359 (0.327 - 0.389)	0.588 (0.534 - 0.639)	0.588 (0.536 - 0.639)	0.587 (0.536 - 0.638)	0.209 (0.091 - 0.401)	0.210 (0.092 - 0.404)	0.210 (0.092 - 0.403)
Total number of cases (thousands)	55,280 (50,040 - 61,323)	86,691 (78,284 - 96,135)	117,660 (106,180 - 130,076)	86,301 (77,076 - 95,524)	134,230 (119,678 - 148,894)	183,410 (163,521 - 203,470)	33 (14 - 62)	51 (22 - 98)	69 (30 - 133)
**Burundi**									
5 -14 years (rate per capita)	0.216 (0.132 - 0.326)	0.229 (0.140 - 0.343)	0.218 (0.133 - 0.327)	0.337 (0.186 - 0.513)	0.356 (0.194 - 0.540)	0.340 (0.187 - 0.516)	0.129 (0.049 - 0.273)	0.137 (0.052 - 0.286)	0.131 (0.049 - 0.274)
15 - 49 years (rate per capita)	0.441 (0.363 - 0.527)	0.441 (0.360 - 0.530)	0.441 (0.362 - 0.528)	0.777 (0.628 - 0.914)	0.773 (0.621 - 0.907)	0.776 (0.627 - 0.914)	0.260 (0.106 - 0.520)	0.260 (0.107 - 0.513)	0.262 (0.107 - 0.527)
50 - 69 years (rate per capita)	0.407 (0.301 - 0.521)	0.407 (0.297 - 0.520)	0.407 (0.298 - 0.525)	0.784 (0.567 - 0.983)	0.782 (0.570 - 0.977)	0.784 (0.563 - 0.987)	0.234 (0.102 - 0.485)	0.235 (0.101 - 0.492)	0.236 (0.101 - 0.490)
70+ years (rate per capita)	0.379 (0.276 - 0.496)	0.377 (0.275 - 0.491)	0.374 (0.275 - 0.486)	0.730 (0.511 - 0.954)	0.727 (0.509 - 0.947)	0.725 (0.509 - 0.938)	0.209 (0.092 - 0.412)	0.208 (0.091 - 0.414)	0.208 (0.091 - 0.409)
All ages (rate per capita)	0.286 (0.241 - 0.340)	0.301 (0.252 - 0.356)	0.295 (0.248 - 0.350)	0.497 (0.418 - 0.573)	0.520 (0.435 - 0.606)	0.515 (0.434 - 0.595)	0.168 (0.073 - 0.328)	0.177 (0.077 - 0.344)	0.175 (0.075 - 0.341)
Age-standardized (rate per capita)	0.356 (0.307 - 0.406)	0.356 (0.307 - 0.405)	0.356 (0.307 - 0.405)	0.634 (0.550 - 0.715)	0.634 (0.550 - 0.716)	0.634 (0.550 - 0.715)	0.208 (0.091 - 0.407)	0.208 (0.091 - 0.407)	0.209 (0.090 - 0.409)
Total number of cases (thousands)	1,608 (1,356 - 1,911)	2,393 (2,003 - 2,832)	3,323 (2,793 - 3,935)	2,795 (2,355 - 3,226)	4,139 (3,460 - 4,821)	5,789 (4,880 - 6,687)	1 (0 - 2)	1 (1 - 3)	2 (1 - 4)
**Comoros**									
5 -14 years (rate per capita)	0.220 (0.135 - 0.330)	0.227 (0.139 - 0.339)	0.226 (0.138 - 0.337)	0.343 (0.187 - 0.520)	0.353 (0.192 - 0.535)	0.351 (0.191 - 0.532)	0.132 (0.049 - 0.278)	0.136 (0.050 - 0.286)	0.135 (0.051 - 0.283)
15 - 49 years (rate per capita)	0.441 (0.362 - 0.528)	0.441 (0.363 - 0.527)	0.441 (0.362 - 0.526)	0.775 (0.622 - 0.911)	0.775 (0.625 - 0.911)	0.777 (0.628 - 0.912)	0.261 (0.107 - 0.521)	0.261 (0.107 - 0.521)	0.261 (0.106 - 0.523)
50 - 69 years (rate per capita)	0.407 (0.299 - 0.522)	0.407 (0.298 - 0.521)	0.407 (0.295 - 0.523)	0.784 (0.567 - 0.982)	0.782 (0.570 - 0.976)	0.783 (0.565 - 0.983)	0.234 (0.101 - 0.482)	0.235 (0.101 - 0.482)	0.235 (0.101 - 0.482)
70+ years (rate per capita)	0.381 (0.277 - 0.501)	0.378 (0.275 - 0.495)	0.377 (0.275 - 0.493)	0.732 (0.511 - 0.957)	0.729 (0.510 - 0.953)	0.728 (0.509 - 0.951)	0.209 (0.093 - 0.410)	0.208 (0.091 - 0.412)	0.208 (0.091 - 0.411)
All ages (rate per capita)	0.294 (0.248 - 0.349)	0.309 (0.260 - 0.365)	0.315 (0.266 - 0.372)	0.511 (0.430 - 0.593)	0.538 (0.454 - 0.621)	0.550 (0.468 - 0.632)	0.174 (0.075 - 0.339)	0.183 (0.079 - 0.354)	0.186 (0.079 - 0.359)
Age-standardized (rate per capita)	0.356 (0.307 - 0.405)	0.356 (0.307 - 0.405)	0.356 (0.307 - 0.405)	0.634 (0.550 - 0.716)	0.634 (0.550 - 0.715)	0.634 (0.550 - 0.715)	0.208 (0.090 - 0.410)	0.208 (0.091 - 0.407)	0.209 (0.091 - 0.405)
Total number of cases (thousands)	122 (103 - 145)	192 (162 - 227)	249 (211 - 295)	212 (178 - 246)	334 (282 - 386)	435 (370 - 501)	0 (0 - 0)	0 (0 - 0)	0 (0 - 0)
**Djibouti**									
5 -14 years (rate per capita)	0.230 (0.140 - 0.344)	0.236 (0.144 - 0.352)	0.232 (0.141 - 0.346)	0.357 (0.194 - 0.542)	0.366 (0.199 - 0.554)	0.360 (0.196 - 0.547)	0.137 (0.051 - 0.291)	0.141 (0.053 - 0.299)	0.139 (0.052 - 0.291)
15 - 49 years (rate per capita)	0.441 (0.362 - 0.527)	0.442 (0.363 - 0.526)	0.442 (0.364 - 0.527)	0.776 (0.625 - 0.911)	0.776 (0.626 - 0.911)	0.778 (0.633 - 0.913)	0.261 (0.106 - 0.519)	0.261 (0.107 - 0.521)	0.261 (0.107 - 0.518)
50 - 69 years (rate per capita)	0.407 (0.298 - 0.524)	0.407 (0.300 - 0.522)	0.407 (0.298 - 0.522)	0.783 (0.566 - 0.981)	0.784 (0.565 - 0.986)	0.783 (0.568 - 0.978)	0.235 (0.102 - 0.485)	0.234 (0.101 - 0.482)	0.234 (0.100 - 0.479)
70+ years (rate per capita)	0.380 (0.276 - 0.501)	0.377 (0.275 - 0.494)	0.376 (0.275 - 0.492)	0.731 (0.510 - 0.956)	0.728 (0.509 - 0.951)	0.727 (0.509 - 0.949)	0.210 (0.091 - 0.415)	0.208 (0.091 - 0.412)	0.206 (0.089 - 0.410)
All ages (rate per capita)	0.308 (0.259 - 0.365)	0.328 (0.277 - 0.386)	0.340 (0.290 - 0.396)	0.536 (0.450 - 0.620)	0.572 (0.485 - 0.659)	0.598 (0.507 - 0.682)	0.182 (0.078 - 0.353)	0.193 (0.083 - 0.380)	0.200 (0.085 - 0.390)
Age-standardized (rate per capita)	0.356 (0.307 - 0.405)	0.356 (0.307 - 0.405)	0.356 (0.307 - 0.405)	0.634 (0.550 - 0.715)	0.634 (0.550 - 0.716)	0.634 (0.550 - 0.715)	0.208 (0.091 - 0.404)	0.208 (0.091 - 0.409)	0.208 (0.091 - 0.407)
Total number of cases (thousands)	177 (149 - 209)	257 (217 - 302)	303 (258 - 352)	308 (258 - 356)	447 (379 - 515)	532 (452 - 607)	0 (0 - 0)	0 (0 - 0)	0 (0 - 0)
**Eritrea**									
5 -14 years (rate per capita)	0.222 (0.136 - 0.333)	0.232 (0.141 - 0.346)	0.223 (0.137 - 0.334)	0.346 (0.188 - 0.523)	0.360 (0.196 - 0.547)	0.348 (0.189 - 0.526)	0.132 (0.050 - 0.278)	0.138 (0.052 - 0.289)	0.133 (0.051 - 0.276)
15 - 49 years (rate per capita)	0.442 (0.364 - 0.527)	0.440 (0.358 - 0.531)	0.441 (0.360 - 0.528)	0.775 (0.624 - 0.912)	0.773 (0.617 - 0.913)	0.778 (0.628 - 0.914)	0.260 (0.107 - 0.515)	0.258 (0.105 - 0.513)	0.259 (0.107 - 0.518)
50 - 69 years (rate per capita)	0.407 (0.292 - 0.522)	0.407 (0.299 - 0.525)	0.407 (0.300 - 0.522)	0.783 (0.565 - 0.983)	0.784 (0.564 - 0.985)	0.784 (0.567 - 0.982)	0.234 (0.102 - 0.491)	0.233 (0.100 - 0.476)	0.233 (0.100 - 0.478)
70+ years (rate per capita)	0.385 (0.276 - 0.508)	0.384 (0.277 - 0.505)	0.382 (0.277 - 0.502)	0.738 (0.514 - 0.970)	0.736 (0.514 - 0.967)	0.734 (0.514 - 0.961)	0.212 (0.092 - 0.416)	0.211 (0.092 - 0.420)	0.210 (0.091 - 0.414)
All ages (rate per capita)	0.295 (0.247 - 0.349)	0.312 (0.260 - 0.371)	0.309 (0.259 - 0.366)	0.510 (0.426 - 0.590)	0.541 (0.452 - 0.630)	0.538 (0.453 - 0.619)	0.173 (0.074 - 0.335)	0.183 (0.079 - 0.355)	0.182 (0.078 - 0.351)
Age-standardized (rate per capita)	0.356 (0.307 - 0.406)	0.356 (0.307 - 0.406)	0.356 (0.307 - 0.405)	0.634 (0.550 - 0.715)	0.634 (0.550 - 0.716)	0.634 (0.550 - 0.716)	0.207 (0.091 - 0.406)	0.207 (0.090 - 0.407)	0.207 (0.090 - 0.402)
Total number of cases (thousands)	926 (777 - 1,097)	1,309 (1,091 - 1,556)	1,619 (1,357 - 1,920)	1,602 (1,338 - 1,854)	2,271 (1,895 - 2,643)	2,819 (2,373 - 3,245)	1 (0 - 1)	1 (0 - 1)	1 (0 - 2)
**Ethiopia**									
5 -14 years (rate per capita)	0.221 (0.135 - 0.331)	0.222 (0.135 - 0.332)	0.231 (0.141 - 0.345)	0.343 (0.187 - 0.520)	0.345 (0.188 - 0.522)	0.359 (0.195 - 0.545)	0.132 (0.049 - 0.276)	0.132 (0.049 - 0.275)	0.138 (0.053 - 0.290)
15 - 49 years (rate per capita)	0.442 (0.363 - 0.526)	0.441 (0.362 - 0.528)	0.441 (0.363 - 0.528)	0.775 (0.626 - 0.909)	0.775 (0.623 - 0.911)	0.775 (0.622 - 0.911)	0.260 (0.106 - 0.522)	0.261 (0.106 - 0.517)	0.261 (0.106 - 0.517)
50 - 69 years (rate per capita)	0.407 (0.300 - 0.523)	0.407 (0.300 - 0.522)	0.407 (0.301 - 0.521)	0.783 (0.566 - 0.982)	0.784 (0.566 - 0.985)	0.784 (0.567 - 0.983)	0.234 (0.102 - 0.484)	0.235 (0.100 - 0.481)	0.235 (0.101 - 0.486)
70+ years (rate per capita)	0.379 (0.275 - 0.499)	0.377 (0.274 - 0.494)	0.375 (0.274 - 0.487)	0.730 (0.506 - 0.954)	0.727 (0.510 - 0.951)	0.725 (0.509 - 0.941)	0.209 (0.091 - 0.416)	0.208 (0.090 - 0.414)	0.207 (0.091 - 0.406)
All ages (rate per capita)	0.294 (0.249 - 0.347)	0.298 (0.250 - 0.353)	0.316 (0.265 - 0.373)	0.512 (0.432 - 0.593)	0.517 (0.434 - 0.600)	0.549 (0.464 - 0.635)	0.173 (0.074 - 0.337)	0.176 (0.075 - 0.344)	0.187 (0.080 - 0.361)
Age-standardized (rate per capita)	0.356 (0.307 - 0.405)	0.356 (0.307 - 0.405)	0.356 (0.307 - 0.405)	0.634 (0.550 - 0.715)	0.634 (0.550 - 0.715)	0.634 (0.550 - 0.715)	0.208 (0.091 - 0.406)	0.208 (0.091 - 0.407)	0.209 (0.091 - 0.406)
Total number of cases (thousands)	14,102 (11,932 - 16,632)	22,746 (19,129 - 26,977)	31,406 (26,377 - 37,117)	24,508 (20,691 - 28,417)	39,483 (33,188 - 45,862)	54,596 (46,112 - 63,140)	8 (4 - 16)	13 (6 - 26)	19 (8 - 36)
**Kenya**									
5 -14 years (rate per capita)	0.130 (0.100 - 0.171)	0.141 (0.107 - 0.191)	0.137 (0.103 - 0.184)	0.139 (0.100 - 0.198)	0.147 (0.107 - 0.220)	0.143 (0.104 - 0.207)	0.078 (0.032 - 0.155)	0.084 (0.035 - 0.169)	0.082 (0.034 - 0.165)
15 - 49 years (rate per capita)	0.281 (0.255 - 0.310)	0.275 (0.247 - 0.303)	0.276 (0.250 - 0.303)	0.415 (0.369 - 0.463)	0.410 (0.365 - 0.458)	0.415 (0.371 - 0.465)	0.166 (0.072 - 0.327)	0.162 (0.071 - 0.318)	0.163 (0.071 - 0.322)
50 - 69 years (rate per capita)	0.216 (0.189 - 0.247)	0.214 (0.186 - 0.245)	0.216 (0.190 - 0.247)	0.408 (0.358 - 0.465)	0.406 (0.356 - 0.467)	0.409 (0.358 - 0.469)	0.125 (0.053 - 0.245)	0.124 (0.053 - 0.239)	0.125 (0.053 - 0.241)
70+ years (rate per capita)	0.200 (0.177 - 0.225)	0.199 (0.174 - 0.225)	0.197 (0.175 - 0.222)	0.347 (0.304 - 0.394)	0.346 (0.302 - 0.393)	0.344 (0.303 - 0.392)	0.111 (0.050 - 0.216)	0.111 (0.049 - 0.214)	0.110 (0.048 - 0.211)
All ages (rate per capita)	0.177 (0.161 - 0.196)	0.188 (0.170 - 0.208)	0.190 (0.173 - 0.209)	0.252 (0.228 - 0.279)	0.271 (0.245 - 0.301)	0.276 (0.251 - 0.307)	0.104 (0.045 - 0.200)	0.111 (0.048 - 0.211)	0.112 (0.049 - 0.215)
Age-standardized (rate per capita)	0.213 (0.197 - 0.229)	0.212 (0.196 - 0.228)	0.212 (0.196 - 0.228)	0.328 (0.303 - 0.353)	0.326 (0.302 - 0.355)	0.327 (0.302 - 0.352)	0.125 (0.054 - 0.243)	0.124 (0.053 - 0.242)	0.124 (0.054 - 0.242)
Total number of cases (thousands)	4,143 (3,768 - 4,585)	6,668 (6,034 - 7,372)	8,766 (7,992 - 9,631)	5,905 (5,349 - 6,538)	9,587 (8,683 - 10,652)	12,771 (11,614 - 14,160)	2 (1 - 5)	4 (2 - 7)	5 (2 - 10)
**Madagascar**									
5 -14 years (rate per capita)	0.504 (0.450 - 0.553)	0.418 (0.355 - 0.479)	0.425 (0.361 - 0.486)	0.502 (0.441 - 0.561)	0.430 (0.358 - 0.523)	0.437 (0.364 - 0.529)	0.299 (0.132 - 0.584)	0.248 (0.109 - 0.489)	0.253 (0.111 - 0.494)
15 - 49 years (rate per capita)	0.637 (0.608 - 0.663)	0.632 (0.605 - 0.659)	0.633 (0.605 - 0.659)	0.821 (0.770 - 0.878)	0.829 (0.780 - 0.885)	0.828 (0.779 - 0.884)	0.374 (0.168 - 0.735)	0.372 (0.165 - 0.724)	0.374 (0.166 - 0.726)
50 - 69 years (rate per capita)	0.562 (0.526 - 0.598)	0.562 (0.524 - 0.599)	0.561 (0.522 - 0.598)	0.966 (0.896 - 1.034)	0.965 (0.891 - 1.032)	0.966 (0.893 - 1.033)	0.322 (0.141 - 0.621)	0.322 (0.141 - 0.630)	0.324 (0.141 - 0.636)
70+ years (rate per capita)	0.550 (0.518 - 0.585)	0.554 (0.518 - 0.593)	0.550 (0.515 - 0.588)	0.933 (0.866 - 1.000)	0.933 (0.863 - 0.999)	0.931 (0.865 - 0.995)	0.302 (0.136 - 0.573)	0.305 (0.137 - 0.581)	0.305 (0.135 - 0.579)

	Prevalence 1990	Prevalence 2005	Prevalence 2015	Incidence 1990	Incidence 2005	Incidence 2015	YLD rate 1990	YLD rate 2005	YLD rate 2015
All ages (rate per capita)	0.477 (0.457 - 0.497)	0.459 (0.435 - 0.479)	0.474 (0.450 - 0.494)	0.598 (0.568 - 0.632)	0.589 (0.556 - 0.625)	0.611 (0.577 - 0.648)	0.280 (0.124 - 0.546)	0.270 (0.120 - 0.516)	0.279 (0.124 - 0.538)
Age-standardized (rate per capita)	0.536 (0.518 - 0.553)	0.521 (0.502 - 0.539)	0.521 (0.502 - 0.539)	0.730 (0.699 - 0.764)	0.722 (0.690 - 0.755)	0.722 (0.690 - 0.755)	0.312 (0.138 - 0.612)	0.304 (0.135 - 0.588)	0.305 (0.135 - 0.592)
Total number of cases (thousands)	5,508 (5,280 - 5,736)	8,390 (7,956 - 8,762)	11,455 (10,891 - 11,940)	6,911 (6,556 - 7,305)	10,774 (10,164 - 11,427)	14,782 (13,970 - 15,677)	3 (1 - 6)	5 (2 - 9)	7 (3 - 13)
**Malawi**									
5 -14 years (rate per capita)	0.225 (0.138 - 0.337)	0.224 (0.138 - 0.336)	0.223 (0.136 - 0.334)	0.350 (0.191 - 0.530)	0.349 (0.190 - 0.528)	0.347 (0.189 - 0.525)	0.134 (0.050 - 0.283)	0.134 (0.050 - 0.281)	0.134 (0.049 - 0.281)
15 - 49 years (rate per capita)	0.442 (0.364 - 0.527)	0.440 (0.359 - 0.531)	0.441 (0.361 - 0.530)	0.775 (0.625 - 0.913)	0.774 (0.619 - 0.911)	0.775 (0.620 - 0.915)	0.260 (0.106 - 0.516)	0.259 (0.106 - 0.515)	0.261 (0.107 - 0.518)
50 - 69 years (rate per capita)	0.407 (0.299 - 0.523)	0.407 (0.302 - 0.521)	0.407 (0.303 - 0.517)	0.783 (0.568 - 0.979)	0.785 (0.566 - 0.990)	0.784 (0.573 - 0.986)	0.234 (0.101 - 0.480)	0.233 (0.101 - 0.479)	0.234 (0.102 - 0.486)
70+ years (rate per capita)	0.384 (0.275 - 0.508)	0.378 (0.275 - 0.495)	0.379 (0.276 - 0.496)	0.737 (0.512 - 0.968)	0.729 (0.509 - 0.952)	0.730 (0.510 - 0.954)	0.211 (0.092 - 0.420)	0.208 (0.091 - 0.407)	0.210 (0.092 - 0.418)
All ages (rate per capita)	0.298 (0.250 - 0.351)	0.295 (0.246 - 0.350)	0.301 (0.251 - 0.357)	0.516 (0.433 - 0.596)	0.511 (0.428 - 0.598)	0.522 (0.436 - 0.606)	0.175 (0.075 - 0.340)	0.173 (0.075 - 0.335)	0.178 (0.076 - 0.346)
Age-standardized (rate per capita)	0.356 (0.307 - 0.406)	0.356 (0.307 - 0.405)	0.356 (0.307 - 0.405)	0.634 (0.550 - 0.715)	0.634 (0.550 - 0.716)	0.634 (0.550 - 0.716)	0.207 (0.090 - 0.405)	0.207 (0.091 - 0.405)	0.208 (0.090 - 0.406)
Total number of cases (thousands)	2,798 (2,354 - 3,304)	3,768 (3,147 - 4,477)	5,176 (4,320 - 6,149)	4,851 (4,075 - 5,609)	6,537 (5,468 - 7,642)	8,985 (7,497 - 10,426)	2 (1 - 3)	2 (1 - 4)	3 (1 - 6)
**Mozambique**									
5 -14 years (rate per capita)	0.245 (0.189 - 0.319)	0.242 (0.181 - 0.318)	0.246 (0.184 - 0.322)	0.263 (0.192 - 0.361)	0.259 (0.188 - 0.362)	0.263 (0.191 - 0.364)	0.146 (0.061 - 0.284)	0.144 (0.059 - 0.284)	0.147 (0.061 - 0.291)
15 - 49 years (rate per capita)	0.510 (0.467 - 0.552)	0.504 (0.461 - 0.544)	0.504 (0.462 - 0.545)	0.736 (0.669 - 0.806)	0.738 (0.669 - 0.811)	0.737 (0.669 - 0.811)	0.299 (0.132 - 0.593)	0.296 (0.130 - 0.585)	0.297 (0.131 - 0.584)
50 - 69 years (rate per capita)	0.420 (0.371 - 0.475)	0.419 (0.370 - 0.473)	0.420 (0.371 - 0.473)	0.801 (0.706 - 0.901)	0.799 (0.701 - 0.897)	0.798 (0.702 - 0.897)	0.239 (0.105 - 0.468)	0.238 (0.104 - 0.463)	0.238 (0.103 - 0.468)
70+ years (rate per capita)	0.406 (0.355 - 0.460)	0.403 (0.354 - 0.454)	0.400 (0.352 - 0.450)	0.709 (0.622 - 0.808)	0.704 (0.619 - 0.797)	0.701 (0.617 - 0.794)	0.221 (0.100 - 0.426)	0.219 (0.100 - 0.424)	0.218 (0.100 - 0.422)
All ages (rate per capita)	0.329 (0.301 - 0.358)	0.332 (0.305 - 0.361)	0.336 (0.308 - 0.366)	0.467 (0.431 - 0.509)	0.477 (0.439 - 0.521)	0.481 (0.442 - 0.526)	0.192 (0.085 - 0.367)	0.194 (0.085 - 0.366)	0.197 (0.086 - 0.376)
Age-standardized (rate per capita)	0.396 (0.369 - 0.421)	0.394 (0.367 - 0.419)	0.394 (0.367 - 0.419)	0.604 (0.564 - 0.645)	0.604 (0.564 - 0.645)	0.604 (0.564 - 0.645)	0.230 (0.100 - 0.447)	0.228 (0.099 - 0.438)	0.229 (0.100 - 0.438)
Total number of cases (thousands)	4,425 (4,053 - 4,812)	7,010 (6,440 - 7,618)	9,405 (8,627 - 10,231)	6,280 (5,794 - 6,848)	10,072 (9,273 - 10,999)	13,460 (12,378 - 14,716)	3 (1 - 5)	4 (2 - 8)	6 (2 - 11)
**Rwanda**									
5 -14 years (rate per capita)	0.220 (0.134 - 0.329)	0.223 (0.136 - 0.334)	0.225 (0.138 - 0.336)	0.342 (0.187 - 0.518)	0.346 (0.189 - 0.524)	0.349 (0.190 - 0.529)	0.131 (0.049 - 0.275)	0.133 (0.049 - 0.272)	0.135 (0.051 - 0.280)
15 - 49 years (rate per capita)	0.441 (0.363 - 0.530)	0.441 (0.360 - 0.531)	0.441 (0.361 - 0.528)	0.775 (0.622 - 0.911)	0.773 (0.621 - 0.908)	0.777 (0.629 - 0.915)	0.261 (0.107 - 0.517)	0.259 (0.107 - 0.514)	0.260 (0.107 - 0.521)
50 - 69 years (rate per capita)	0.407 (0.302 - 0.521)	0.408 (0.293 - 0.523)	0.408 (0.296 - 0.524)	0.785 (0.564 - 0.991)	0.783 (0.566 - 0.979)	0.783 (0.569 - 0.977)	0.235 (0.100 - 0.483)	0.234 (0.101 - 0.483)	0.235 (0.101 - 0.476)
70+ years (rate per capita)	0.378 (0.276 - 0.493)	0.369 (0.271 - 0.480)	0.371 (0.275 - 0.483)	0.729 (0.511 - 0.950)	0.719 (0.518 - 0.922)	0.722 (0.516 - 0.928)	0.209 (0.091 - 0.413)	0.204 (0.089 - 0.405)	0.206 (0.090 - 0.403)
All ages (rate per capita)	0.284 (0.238 - 0.338)	0.308 (0.258 - 0.365)	0.315 (0.265 - 0.374)	0.491 (0.410 - 0.572)	0.534 (0.448 - 0.619)	0.549 (0.466 - 0.632)	0.168 (0.072 - 0.325)	0.181 (0.078 - 0.346)	0.186 (0.079 - 0.361)
Age-standardized (rate per capita)	0.356 (0.307 - 0.406)	0.356 (0.307 - 0.406)	0.356 (0.307 - 0.406)	0.634 (0.550 - 0.716)	0.634 (0.550 - 0.715)	0.634 (0.550 - 0.714)	0.208 (0.091 - 0.407)	0.207 (0.090 - 0.400)	0.208 (0.091 - 0.408)
Total number of cases (thousands)	2,061 (1,724 - 2,453)	2,781 (2,327 - 3,294)	3,665 (3,079 - 4,344)	3,564 (2,974 - 4,151)	4,822 (4,050 - 5,586)	6,388 (5,416 - 7,346)	1 (1 - 2)	2 (1 - 3)	2 (1 - 4)
**Somalia**									
5 -14 years (rate per capita)	0.221 (0.135 - 0.332)	0.218 (0.133 - 0.327)	0.222 (0.136 - 0.333)	0.345 (0.188 - 0.521)	0.340 (0.187 - 0.517)	0.346 (0.188 - 0.523)	0.132 (0.050 - 0.276)	0.130 (0.048 - 0.271)	0.133 (0.050 - 0.275)
15 - 49 years (rate per capita)	0.442 (0.364 - 0.527)	0.441 (0.363 - 0.527)	0.441 (0.362 - 0.528)	0.776 (0.625 - 0.913)	0.775 (0.624 - 0.912)	0.774 (0.622 - 0.910)	0.260 (0.105 - 0.517)	0.259 (0.108 - 0.514)	0.260 (0.105 - 0.513)
50 - 69 years (rate per capita)	0.407 (0.299 - 0.523)	0.407 (0.299 - 0.523)	0.407 (0.299 - 0.523)	0.783 (0.568 - 0.981)	0.783 (0.567 - 0.980)	0.783 (0.567 - 0.981)	0.234 (0.102 - 0.482)	0.233 (0.101 - 0.479)	0.233 (0.101 - 0.479)
70+ years (rate per capita)	0.380 (0.276 - 0.500)	0.377 (0.275 - 0.495)	0.377 (0.275 - 0.494)	0.731 (0.510 - 0.955)	0.728 (0.509 - 0.951)	0.728 (0.509 - 0.950)	0.209 (0.091 - 0.412)	0.207 (0.091 - 0.407)	0.207 (0.091 - 0.407)
All ages (rate per capita)	0.294 (0.249 - 0.346)	0.288 (0.242 - 0.340)	0.293 (0.246 - 0.347)	0.511 (0.432 - 0.590)	0.500 (0.420 - 0.580)	0.508 (0.426 - 0.590)	0.172 (0.074 - 0.334)	0.169 (0.073 - 0.329)	0.172 (0.075 - 0.331)
Age-standardized (rate per capita)	0.356 (0.307 - 0.405)	0.356 (0.307 - 0.405)	0.356 (0.307 - 0.405)	0.634 (0.550 - 0.715)	0.634 (0.550 - 0.715)	0.634 (0.550 - 0.715)	0.207 (0.090 - 0.406)	0.207 (0.091 - 0.404)	0.207 (0.091 - 0.402)
Total number of cases (thousands)	1,880 (1,591 - 2,215)	2,450 (2,063 - 2,896)	3,179 (2,669 - 3,770)	3,273 (2,764 - 3,777)	4,251 (3,574 - 4,931)	5,511 (4,627 - 6,404)	1 (0 - 2)	1 (1 - 3)	2 (1 - 4)
**South Sudan**									
5 -14 years (rate per capita)	0.224 (0.137 - 0.335)	0.224 (0.137 - 0.335)	0.226 (0.138 - 0.338)	0.349 (0.190 - 0.528)	0.348 (0.189 - 0.527)	0.351 (0.191 - 0.532)	0.133 (0.051 - 0.282)	0.133 (0.050 - 0.280)	0.135 (0.051 - 0.279)
15 - 49 years (rate per capita)	0.441 (0.363 - 0.525)	0.442 (0.364 - 0.527)	0.441 (0.363 - 0.527)	0.775 (0.627 - 0.909)	0.776 (0.624 - 0.913)	0.775 (0.625 - 0.912)	0.259 (0.107 - 0.512)	0.259 (0.107 - 0.519)	0.259 (0.107 - 0.516)
50 - 69 years (rate per capita)	0.407 (0.298 - 0.524)	0.407 (0.301 - 0.520)	0.407 (0.299 - 0.522)	0.783 (0.566 - 0.981)	0.784 (0.567 - 0.987)	0.783 (0.569 - 0.978)	0.231 (0.099 - 0.474)	0.231 (0.101 - 0.471)	0.232 (0.102 - 0.480)
70+ years (rate per capita)	0.383 (0.275 - 0.505)	0.380 (0.276 - 0.502)	0.378 (0.275 - 0.496)	0.735 (0.510 - 0.963)	0.732 (0.509 - 0.957)	0.729 (0.509 - 0.952)	0.204 (0.090 - 0.403)	0.204 (0.091 - 0.404)	0.204 (0.090 - 0.403)
All ages (rate per capita)	0.298 (0.252 - 0.351)	0.303 (0.255 - 0.358)	0.312 (0.263 - 0.368)	0.518 (0.437 - 0.598)	0.527 (0.444 - 0.609)	0.543 (0.459 - 0.627)	0.174 (0.075 - 0.337)	0.177 (0.076 - 0.340)	0.183 (0.079 - 0.353)
Age-standardized (rate per capita)	0.356 (0.307 - 0.405)	0.356 (0.307 - 0.405)	0.356 (0.307 - 0.405)	0.634 (0.550 - 0.715)	0.634 (0.550 - 0.715)	0.634 (0.550 - 0.715)	0.206 (0.090 - 0.403)	0.206 (0.090 - 0.401)	0.207 (0.090 - 0.403)
Total number of cases (thousands)	1,733 (1,466 - 2,042)	2,455 (2,070 - 2,899)	3,834 (3,236 - 4,524)	3,016 (2,547 - 3,483)	4,269 (3,597 - 4,936)	6,675 (5,645 - 7,704)	1 (0 - 2)	1 (1 - 3)	2 (1 - 4)
**Tanzania**									
5 -14 years (rate per capita)	0.236 (0.193 - 0.277)	0.394 (0.330 - 0.445)	0.315 (0.244 - 0.396)	0.228 (0.159 - 0.298)	0.361 (0.275 - 0.435)	0.292 (0.216 - 0.401)	0.140 (0.059 - 0.286)	0.234 (0.099 - 0.455)	0.188 (0.078 - 0.383)
15 - 49 years (rate per capita)	0.533 (0.493 - 0.571)	0.538 (0.500 - 0.574)	0.539 (0.503 - 0.576)	0.726 (0.661 - 0.799)	0.725 (0.662 - 0.797)	0.726 (0.667 - 0.793)	0.315 (0.140 - 0.617)	0.318 (0.142 - 0.630)	0.319 (0.141 - 0.631)
50 - 69 years (rate per capita)	0.453 (0.407 - 0.501)	0.453 (0.407 - 0.503)	0.453 (0.407 - 0.497)	0.798 (0.706 - 0.891)	0.798 (0.706 - 0.886)	0.797 (0.700 - 0.890)	0.261 (0.114 - 0.510)	0.261 (0.113 - 0.508)	0.262 (0.112 - 0.507)
70+ years (rate per capita)	0.436 (0.392 - 0.485)	0.432 (0.389 - 0.475)	0.428 (0.385 - 0.469)	0.711 (0.626 - 0.801)	0.707 (0.619 - 0.803)	0.704 (0.616 - 0.794)	0.242 (0.108 - 0.467)	0.239 (0.107 - 0.455)	0.238 (0.106 - 0.460)
All ages (rate per capita)	0.346 (0.321 - 0.369)	0.394 (0.368 - 0.417)	0.375 (0.346 - 0.403)	0.460 (0.423 - 0.502)	0.501 (0.464 - 0.540)	0.485 (0.449 - 0.526)	0.204 (0.090 - 0.401)	0.232 (0.101 - 0.452)	0.221 (0.097 - 0.422)
Age-standardized (rate per capita)	0.415 (0.393 - 0.438)	0.444 (0.420 - 0.465)	0.430 (0.406 - 0.453)	0.592 (0.552 - 0.634)	0.614 (0.576 - 0.655)	0.603 (0.567 - 0.643)	0.242 (0.107 - 0.473)	0.260 (0.113 - 0.504)	0.253 (0.109 - 0.485)
Total number of cases (thousands)	8,804 (8,172 - 9,406)	15,399 (14,377 - 16,297)	19,989 (18,491 - 21,485)	11,716 (10,759 - 12,777)	19,593 (18,156 - 21,134)	25,861 (23,986 - 28,075)	5 (2 - 10)	9 (4 - 18)	12 (5 - 23)
**Uganda**									
5 -14 years (rate per capita)	0.172 (0.133 - 0.226)	0.173 (0.134 - 0.226)	0.174 (0.135 - 0.227)	0.221 (0.157 - 0.290)	0.222 (0.158 - 0.291)	0.223 (0.158 - 0.293)	0.102 (0.043 - 0.207)	0.103 (0.043 - 0.206)	0.104 (0.043 - 0.205)
15 - 49 years (rate per capita)	0.420 (0.376 - 0.462)	0.419 (0.376 - 0.463)	0.420 (0.376 - 0.462)	0.718 (0.648 - 0.791)	0.716 (0.645 - 0.792)	0.717 (0.647 - 0.791)	0.247 (0.107 - 0.489)	0.247 (0.107 - 0.489)	0.249 (0.108 - 0.491)
50 - 69 years (rate per capita)	0.326 (0.282 - 0.377)	0.327 (0.282 - 0.377)	0.327 (0.283 - 0.377)	0.737 (0.647 - 0.841)	0.737 (0.645 - 0.842)	0.736 (0.646 - 0.839)	0.187 (0.080 - 0.367)	0.188 (0.080 - 0.368)	0.188 (0.080 - 0.368)
70+ years (rate per capita)	0.310 (0.267 - 0.357)	0.308 (0.266 - 0.353)	0.305 (0.264 - 0.348)	0.644 (0.558 - 0.734)	0.641 (0.557 - 0.728)	0.635 (0.555 - 0.718)	0.172 (0.077 - 0.333)	0.171 (0.077 - 0.327)	0.169 (0.075 - 0.328)
All ages (rate per capita)	0.258 (0.233 - 0.282)	0.255 (0.230 - 0.279)	0.261 (0.236 - 0.286)	0.435 (0.398 - 0.478)	0.427 (0.389 - 0.472)	0.437 (0.397 - 0.482)	0.152 (0.066 - 0.292)	0.150 (0.065 - 0.288)	0.154 (0.066 - 0.299)
Age-standardized (rate per capita)	0.316 (0.291 - 0.339)	0.316 (0.291 - 0.339)	0.316 (0.291 - 0.339)	0.572 (0.530 - 0.613)	0.572 (0.530 - 0.613)	0.572 (0.530 - 0.613)	0.184 (0.079 - 0.355)	0.185 (0.079 - 0.356)	0.185 (0.079 - 0.360)
Total number of cases (thousands)	4,493 (4,066 - 4,911)	7,162 (6,467 - 7,850)	10,224 (9,236 - 11,199)	7,578 (6,923 - 8,327)	11,998 (10,923 - 13,254)	17,098 (15,541 - 18,874)	3 (1 - 5)	4 (2 - 8)	6 (3 - 12)
**Zambia**									
5 -14 years (rate per capita)	0.217 (0.165 - 0.287)	0.210 (0.160 - 0.280)	0.209 (0.159 - 0.280)	0.251 (0.180 - 0.349)	0.244 (0.175 - 0.333)	0.244 (0.174 - 0.333)	0.129 (0.054 - 0.254)	0.125 (0.051 - 0.250)	0.125 (0.052 - 0.252)
15 - 49 years (rate per capita)	0.476 (0.431 - 0.518)	0.473 (0.428 - 0.516)	0.473 (0.430 - 0.516)	0.732 (0.667 - 0.811)	0.732 (0.664 - 0.811)	0.735 (0.669 - 0.812)	0.281 (0.121 - 0.556)	0.279 (0.121 - 0.545)	0.279 (0.122 - 0.552)
50 - 69 years (rate per capita)	0.383 (0.334 - 0.434)	0.383 (0.334 - 0.440)	0.383 (0.334 - 0.440)	0.777 (0.685 - 0.879)	0.778 (0.682 - 0.878)	0.777 (0.682 - 0.876)	0.221 (0.095 - 0.439)	0.220 (0.095 - 0.432)	0.219 (0.095 - 0.431)
70+ years (rate per capita)	0.363 (0.316 - 0.414)	0.361 (0.315 - 0.415)	0.359 (0.314 - 0.411)	0.680 (0.596 - 0.773)	0.679 (0.593 - 0.774)	0.676 (0.591 - 0.769)	0.202 (0.090 - 0.394)	0.200 (0.090 - 0.389)	0.199 (0.090 - 0.387)
All ages (rate per capita)	0.304 (0.276 - 0.332)	0.303 (0.276 - 0.329)	0.307 (0.280 - 0.334)	0.460 (0.422 - 0.505)	0.461 (0.424 - 0.507)	0.467 (0.429 - 0.513)	0.179 (0.078 - 0.345)	0.178 (0.078 - 0.342)	0.181 (0.078 - 0.350)
Age-standardized (rate per capita)	0.365 (0.339 - 0.388)	0.363 (0.337 - 0.387)	0.363 (0.337 - 0.387)	0.595 (0.554 - 0.635)	0.594 (0.554 - 0.635)	0.594 (0.554 - 0.634)	0.213 (0.093 - 0.411)	0.212 (0.092 - 0.410)	0.212 (0.091 - 0.408)
Total number of cases (thousands)	2,472 (2,243 - 2,701)	3,655 (3,328 - 3,973)	4,991 (4,552 - 5,419)	3,739 (3,436 - 4,109)	5,564 (5,112 - 6,118)	7,589 (6,970 - 8,335)	1 (1 - 3)	2 (1 - 4)	3 (1 - 6)
**Central Sub-Saharan Africa**									
5 -14 years (rate per capita)	0.226 (0.172 - 0.296)	0.227 (0.173 - 0.298)	0.228 (0.174 - 0.299)	0.267 (0.187 - 0.375)	0.268 (0.187 - 0.375)	0.269 (0.188 - 0.375)	0.134 (0.055 - 0.266)	0.135 (0.056 - 0.268)	0.136 (0.056 - 0.266)
15 - 49 years (rate per capita)	0.467 (0.425 - 0.510)	0.467 (0.424 - 0.510)	0.467 (0.424 - 0.509)	0.715 (0.640 - 0.800)	0.715 (0.638 - 0.801)	0.715 (0.638 - 0.801)	0.273 (0.119 - 0.535)	0.274 (0.119 - 0.533)	0.274 (0.119 - 0.539)
50 - 69 years (rate per capita)	0.378 (0.333 - 0.430)	0.378 (0.333 - 0.430)	0.378 (0.333 - 0.430)	0.717 (0.625 - 0.832)	0.717 (0.625 - 0.831)	0.717 (0.625 - 0.831)	0.213 (0.092 - 0.419)	0.214 (0.093 - 0.418)	0.214 (0.093 - 0.421)

	Prevalence 1990	Prevalence 2005	Prevalence 2015	Incidence 1990	Incidence 2005	Incidence 2015	YLD rate 1990	YLD rate 2005	YLD rate 2015
**70+ years (rate per capita)**	0.346 (0.302 - 0.397)	0.345 (0.301 - 0.395)	0.344 (0.301 - 0.393)	0.621 (0.530 - 0.719)	0.620 (0.529 - 0.717)	0.618 (0.528 - 0.714)	0.186 (0.084 - 0.361)	0.186 (0.084 - 0.361)	0.186 (0.083 - 0.363)
**All ages (rate per capita)**	0.303 (0.276 - 0.330)	0.303 (0.276 - 0.331)	0.307 (0.280 - 0.335)	0.456 (0.415 - 0.504)	0.454 (0.413 - 0.503)	0.460 (0.419 - 0.510)	0.177 (0.077 - 0.340)	0.177 (0.077 - 0.339)	0.180 (0.078 - 0.346)
**Age-standardized (rate per capita)**	0.361 (0.335 - 0.385)	0.361 (0.335 - 0.385)	0.361 (0.335 - 0.385)	0.573 (0.532 - 0.620)	0.573 (0.532 - 0.620)	0.572 (0.532 - 0.620)	0.208 (0.090 - 0.401)	0.209 (0.091 - 0.401)	0.209 (0.090 - 0.403)
**Total number of cases (thousands)**	16,074 (14,652 - 17,517)	25,437 (23,163 - 27,783)	35,236 (32,084 - 38,492)	24,180 (22,033 - 26,729)	38,107 (34,697 - 42,205)	52,780 (48,032 - 58,531)	9 (4 - 18)	15 (6 - 28)	21 (9 - 40)
**Angola**									
**5 -14 years (rate per capita)**	0.226 (0.172 - 0.297)	0.225 (0.172 - 0.296)	0.227 (0.173 - 0.297)	0.267 (0.187 - 0.375)	0.266 (0.186 - 0.374)	0.268 (0.187 - 0.375)	0.134 (0.055 - 0.267)	0.134 (0.056 - 0.269)	0.135 (0.056 - 0.267)
**15 - 49 years (rate per capita)**	0.467 (0.424 - 0.510)	0.467 (0.424 - 0.510)	0.467 (0.423 - 0.510)	0.715 (0.638 - 0.800)	0.714 (0.637 - 0.801)	0.714 (0.637 - 0.801)	0.273 (0.118 - 0.536)	0.274 (0.119 - 0.535)	0.275 (0.119 - 0.540)
**50 - 69 years (rate per capita)**	0.378 (0.333 - 0.430)	0.378 (0.333 - 0.431)	0.378 (0.333 - 0.431)	0.717 (0.625 - 0.831)	0.716 (0.624 - 0.833)	0.718 (0.626 - 0.834)	0.214 (0.092 - 0.420)	0.215 (0.093 - 0.418)	0.215 (0.093 - 0.420)
**70+ years (rate per capita)**	0.348 (0.303 - 0.400)	0.346 (0.302 - 0.397)	0.345 (0.301 - 0.395)	0.623 (0.530 - 0.723)	0.621 (0.529 - 0.719)	0.619 (0.529 - 0.716)	0.189 (0.085 - 0.367)	0.188 (0.084 - 0.368)	0.188 (0.085 - 0.361)
**All ages (rate per capita)**	0.295 (0.269 - 0.323)	0.296 (0.269 - 0.324)	0.299 (0.272 - 0.328)	0.441 (0.401 - 0.490)	0.442 (0.401 - 0.491)	0.446 (0.405 - 0.495)	0.172 (0.075 - 0.330)	0.174 (0.076 - 0.332)	0.176 (0.076 - 0.337)
**Age-standardized (rate per capita)**	0.361 (0.335 - 0.385)	0.361 (0.335 - 0.385)	0.361 (0.335 - 0.385)	0.573 (0.532 - 0.620)	0.573 (0.532 - 0.620)	0.572 (0.532 - 0.620)	0.209 (0.090 - 0.402)	0.209 (0.091 - 0.403)	0.210 (0.091 - 0.402)
**Total number of cases (thousands)**	3,324 (3,023 - 3,636)	5,346 (4,859 - 5,853)	7,560 (6,866 - 8,276)	4,969 (4,519 - 5,515)	7,970 (7,242 - 8,853)	11,261 (10,223 - 12,511)	2 (1 - 4)	3 (1 - 6)	4 (2 - 9)
**Central African Republic**									
**5 -14 years (rate per capita)**	0.228 (0.174 - 0.298)	0.231 (0.176 - 0.302)	0.236 (0.180 - 0.307)	0.269 (0.188 - 0.375)	0.272 (0.190 - 0.377)	0.278 (0.193 - 0.383)	0.135 (0.056 - 0.271)	0.137 (0.056 - 0.273)	0.140 (0.058 - 0.278)
**15 - 49 years (rate per capita)**	0.467 (0.424 - 0.510)	0.467 (0.424 - 0.509)	0.466 (0.423 - 0.510)	0.716 (0.641 - 0.800)	0.715 (0.637 - 0.801)	0.717 (0.641 - 0.803)	0.273 (0.118 - 0.537)	0.273 (0.118 - 0.540)	0.274 (0.119 - 0.537)
**50 - 69 years (rate per capita)**	0.378 (0.333 - 0.430)	0.378 (0.333 - 0.430)	0.378 (0.333 - 0.430)	0.717 (0.626 - 0.828)	0.717 (0.626 - 0.827)	0.717 (0.626 - 0.828)	0.214 (0.091 - 0.422)	0.214 (0.092 - 0.416)	0.214 (0.093 - 0.418)
**70+ years (rate per capita)**	0.344 (0.301 - 0.393)	0.344 (0.301 - 0.393)	0.343 (0.300 - 0.391)	0.619 (0.528 - 0.715)	0.618 (0.528 - 0.714)	0.617 (0.527 - 0.712)	0.185 (0.084 - 0.361)	0.185 (0.083 - 0.359)	0.185 (0.083 - 0.361)
**All ages (rate per capita)**	0.313 (0.286 - 0.341)	0.320 (0.292 - 0.348)	0.331 (0.303 - 0.359)	0.475 (0.434 - 0.524)	0.484 (0.441 - 0.535)	0.503 (0.458 - 0.554)	0.183 (0.079 - 0.350)	0.187 (0.081 - 0.358)	0.194 (0.085 - 0.373)
**Age-standardized (rate per capita)**	0.361 (0.335 - 0.385)	0.361 (0.335 - 0.385)	0.361 (0.335 - 0.385)	0.572 (0.532 - 0.620)	0.573 (0.532 - 0.620)	0.573 (0.532 - 0.620)	0.209 (0.091 - 0.404)	0.209 (0.091 - 0.402)	0.209 (0.091 - 0.403)
**Total number of cases (thousands)**	920 (839 - 999)	1,302 (1,188 - 1,415)	1,624 (1,485 - 1,761)	1,395 (1,275 - 1,537)	1,966 (1,792 - 2,174)	2,464 (2,247 - 2,716)	1 (0 - 1)	1 (0 - 1)	1 (0 - 2)
**Congo**									
**5 -14 years (rate per capita)**	0.232 (0.176 - 0.302)	0.229 (0.174 - 0.299)	0.228 (0.174 - 0.298)	0.273 (0.190 - 0.377)	0.269 (0.188 - 0.376)	0.269 (0.188 - 0.375)	0.137 (0.057 - 0.274)	0.136 (0.056 - 0.263)	0.136 (0.056 - 0.270)
**15 - 49 years (rate per capita)**	0.467 (0.424 - 0.510)	0.466 (0.423 - 0.509)	0.467 (0.425 - 0.509)	0.715 (0.637 - 0.801)	0.717 (0.640 - 0.803)	0.718 (0.642 - 0.801)	0.274 (0.119 - 0.538)	0.273 (0.119 - 0.536)	0.274 (0.118 - 0.539)
**50 - 69 years (rate per capita)**	0.378 (0.333 - 0.429)	0.378 (0.333 - 0.429)	0.378 (0.333 - 0.429)	0.717 (0.625 - 0.828)	0.717 (0.625 - 0.828)	0.717 (0.625 - 0.829)	0.214 (0.093 - 0.419)	0.214 (0.093 - 0.418)	0.215 (0.093 - 0.420)
**70+ years (rate per capita)**	0.342 (0.300 - 0.390)	0.342 (0.299 - 0.389)	0.341 (0.299 - 0.388)	0.617 (0.526 - 0.712)	0.616 (0.526 - 0.710)	0.615 (0.525 - 0.709)	0.185 (0.083 - 0.360)	0.185 (0.083 - 0.362)	0.185 (0.083 - 0.362)
**All ages (rate per capita)**	0.314 (0.286 - 0.342)	0.317 (0.289 - 0.344)	0.317 (0.290 - 0.344)	0.473 (0.431 - 0.523)	0.480 (0.437 - 0.529)	0.479 (0.437 - 0.528)	0.184 (0.080 - 0.351)	0.185 (0.080 - 0.357)	0.186 (0.080 - 0.355)
**Age-standardized (rate per capita)**	0.361 (0.335 - 0.385)	0.360 (0.335 - 0.385)	0.360 (0.335 - 0.385)	0.572 (0.532 - 0.620)	0.573 (0.532 - 0.620)	0.572 (0.532 - 0.620)	0.209 (0.091 - 0.398)	0.209 (0.091 - 0.405)	0.210 (0.090 - 0.404)
**Total number of cases (thousands)**	753 (686 - 821)	1,115 (1,018 - 1,212)	1,465 (1,340 - 1,592)	1,133 (1,032 - 1,253)	1,689 (1,539 - 1,864)	2,217 (2,023 - 2,442)	0 (0 - 1)	1 (0 - 1)	1 (0 - 2)
**Democratic Republic of the Congo**									
**5 -14 years (rate per capita)**	0.226 (0.172 - 0.296)	0.227 (0.173 - 0.298)	0.228 (0.174 - 0.299)	0.266 (0.186 - 0.374)	0.268 (0.187 - 0.375)	0.269 (0.188 - 0.375)	0.134 (0.055 - 0.265)	0.135 (0.055 - 0.269)	0.135 (0.055 - 0.265)
**15 - 49 years (rate per capita)**	0.467 (0.425 - 0.510)	0.467 (0.424 - 0.510)	0.467 (0.424 - 0.509)	0.715 (0.640 - 0.800)	0.715 (0.638 - 0.801)	0.715 (0.637 - 0.801)	0.273 (0.118 - 0.532)	0.273 (0.119 - 0.532)	0.274 (0.119 - 0.539)
**50 - 69 years (rate per capita)**	0.378 (0.333 - 0.431)	0.378 (0.333 - 0.430)	0.378 (0.333 - 0.430)	0.718 (0.625 - 0.832)	0.717 (0.625 - 0.830)	0.717 (0.624 - 0.830)	0.213 (0.092 - 0.418)	0.213 (0.093 - 0.416)	0.214 (0.093 - 0.420)
**70+ years (rate per capita)**	0.347 (0.303 - 0.398)	0.346 (0.302 - 0.396)	0.344 (0.301 - 0.394)	0.622 (0.531 - 0.720)	0.620 (0.530 - 0.717)	0.619 (0.529 - 0.715)	0.185 (0.083 - 0.358)	0.185 (0.084 - 0.359)	0.186 (0.083 - 0.363)
**All ages (rate per capita)**	0.303 (0.276 - 0.330)	0.302 (0.275 - 0.330)	0.307 (0.279 - 0.335)	0.456 (0.416 - 0.504)	0.453 (0.412 - 0.502)	0.459 (0.418 - 0.509)	0.177 (0.077 - 0.340)	0.177 (0.077 - 0.338)	0.180 (0.078 - 0.346)
**Age-standardized (rate per capita)**	0.361 (0.335 - 0.385)	0.361 (0.335 - 0.385)	0.361 (0.335 - 0.385)	0.573 (0.532 - 0.620)	0.573 (0.532 - 0.620)	0.572 (0.532 - 0.620)	0.208 (0.090 - 0.400)	0.209 (0.090 - 0.400)	0.209 (0.090 - 0.403)
**Total number of cases (thousands)**	10,658 (9,718 - 11,609)	17,017 (15,489 - 18,587)	23,737 (21,611 - 25,931)	16,038 (14,616 - 17,719)	25,486 (23,214 - 28,226)	35,535 (32,345 - 39,415)	6 (3 - 12)	10 (4 - 19)	14 (6 - 27)
**Equatorial Guinea**									
**5 -14 years (rate per capita)**	0.209 (0.159 - 0.277)	0.231 (0.176 - 0.302)	0.229 (0.174 - 0.299)	0.248 (0.174 - 0.358)	0.272 (0.190 - 0.377)	0.270 (0.188 - 0.376)	0.124 (0.051 - 0.254)	0.137 (0.057 - 0.276)	0.136 (0.057 - 0.271)
**15 - 49 years (rate per capita)**	0.461 (0.418 - 0.504)	0.468 (0.428 - 0.510)	0.464 (0.423 - 0.507)	0.722 (0.646 - 0.814)	0.718 (0.648 - 0.796)	0.716 (0.641 - 0.802)	0.272 (0.118 - 0.532)	0.276 (0.118 - 0.546)	0.274 (0.118 - 0.540)
**50 - 69 years (rate per capita)**	0.378 (0.333 - 0.430)	0.377 (0.330 - 0.431)	0.377 (0.328 - 0.433)	0.717 (0.625 - 0.826)	0.713 (0.622 - 0.832)	0.719 (0.623 - 0.842)	0.214 (0.093 - 0.420)	0.214 (0.093 - 0.417)	0.215 (0.093 - 0.423)
**70+ years (rate per capita)**	0.344 (0.301 - 0.394)	0.340 (0.298 - 0.387)	0.336 (0.296 - 0.382)	0.619 (0.529 - 0.715)	0.615 (0.525 - 0.709)	0.610 (0.521 - 0.702)	0.186 (0.084 - 0.364)	0.184 (0.083 - 0.360)	0.182 (0.083 - 0.355)
**All ages (rate per capita)**	0.316 (0.290 - 0.341)	0.325 (0.298 - 0.352)	0.325 (0.297 - 0.351)	0.493 (0.451 - 0.547)	0.489 (0.448 - 0.537)	0.499 (0.456 - 0.549)	0.185 (0.082 - 0.357)	0.190 (0.083 - 0.367)	0.191 (0.082 - 0.365)
**Age-standardized (rate per capita)**	0.360 (0.334 - 0.384)	0.360 (0.334 - 0.384)	0.360 (0.334 - 0.384)	0.573 (0.532 - 0.620)	0.572 (0.532 - 0.620)	0.572 (0.531 - 0.620)	0.209 (0.091 - 0.403)	0.210 (0.091 - 0.405)	0.210 (0.091 - 0.404)
**Total number of cases (thousands)**	119 (109 - 129)	203 (186 - 221)	274 (251 - 297)	186 (170 - 206)	306 (280 - 336)	421 (386 - 464)	0 (0 - 0)	0 (0 - 0)	0 (0 - 0)
**Gabon**									
**5 -14 years (rate per capita)**	0.227 (0.173 - 0.297)	0.234 (0.178 - 0.305)	0.232 (0.177 - 0.303)	0.268 (0.187 - 0.375)	0.275 (0.192 - 0.381)	0.273 (0.190 - 0.377)	0.135 (0.055 - 0.265)	0.139 (0.057 - 0.278)	0.138 (0.056 - 0.273)
**15 - 49 years (rate per capita)**	0.467 (0.425 - 0.510)	0.466 (0.424 - 0.510)	0.465 (0.423 - 0.508)	0.715 (0.640 - 0.800)	0.716 (0.640 - 0.801)	0.718 (0.642 - 0.803)	0.274 (0.118 - 0.537)	0.273 (0.119 - 0.542)	0.273 (0.118 - 0.537)
**50 - 69 years (rate per capita)**	0.379 (0.333 - 0.431)	0.378 (0.333 - 0.430)	0.378 (0.333 - 0.428)	0.718 (0.626 - 0.828)	0.716 (0.624 - 0.825)	0.716 (0.625 - 0.826)	0.214 (0.093 - 0.418)	0.214 (0.093 - 0.420)	0.214 (0.092 - 0.416)
**70+ years (rate per capita)**	0.341 (0.300 - 0.389)	0.340 (0.299 - 0.386)	0.336 (0.297 - 0.380)	0.616 (0.526 - 0.709)	0.614 (0.524 - 0.706)	0.610 (0.522 - 0.701)	0.184 (0.083 - 0.356)	0.184 (0.084 - 0.355)	0.181 (0.081 - 0.351)
**All ages (rate per capita)**	0.314 (0.288 - 0.340)	0.328 (0.299 - 0.355)	0.333 (0.306 - 0.360)	0.482 (0.443 - 0.530)	0.499 (0.457 - 0.549)	0.511 (0.468 - 0.561)	0.183 (0.080 - 0.350)	0.191 (0.083 - 0.367)	0.195 (0.085 - 0.373)
**Age-standardized (rate per capita)**	0.361 (0.335 - 0.385)	0.360 (0.334 - 0.385)	0.360 (0.334 - 0.385)	0.573 (0.531 - 0.620)	0.573 (0.532 - 0.620)	0.573 (0.532 - 0.620)	0.209 (0.090 - 0.402)	0.209 (0.091 - 0.404)	0.209 (0.090 - 0.401)
**Total number of cases (thousands)**	300 (274 - 324)	453 (414 - 491)	576 (528 - 621)	459 (422 - 505)	689 (631 - 759)	882 (808 - 968)	0 (0 - 0)	0 (0 - 1)	0 (0 - 1)

Appendix 2, Table 4. Prevalence, incidence rate, and YLD rate per 1,000 population (with 95% uncertainty intervals) for severe periodontal disease, 1990 and 2015, by country and by age

	Prevalence 1990	Prevalence 2005	Prevalence 2015	Incidence 1990	Incidence 2005	Incidence 2015	YLD rate 1990	YLD rate 2005	YLD rate 2015
**Global**									
15 - 49 years (rate per capita)	0.058 (0.048 - 0.070)	0.062 (0.052 - 0.075)	0.066 (0.055 - 0.080)	0.006 (0.005 - 0.008)	0.007 (0.006 - 0.008)	0.007 (0.006 - 0.009)	0.386 (0.145 - 0.810)	0.414 (0.156 - 0.871)	0.440 (0.165 - 0.922)
50 - 69 years (rate per capita)	0.179 (0.150 - 0.212)	0.179 (0.150 - 0.211)	0.180 (0.151 - 0.213)	0.010 (0.008 - 0.013)	0.010 (0.008 - 0.013)	0.011 (0.008 - 0.013)	1.162 (0.453 - 2.374)	1.160 (0.452 - 2.367)	1.172 (0.456 - 2.397)
70+ years (rate per capita)	0.149 (0.128 - 0.173)	0.151 (0.129 - 0.174)	0.151 (0.129 - 0.175)	0.007 (0.005 - 0.009)	0.007 (0.005 - 0.009)	0.007 (0.005 - 0.009)	0.933 (0.371 - 1.904)	0.941 (0.376 - 1.923)	0.943 (0.376 - 1.926)
All ages (rate per capita)	0.058 (0.050 - 0.067)	0.066 (0.057 - 0.076)	0.073 (0.063 - 0.085)	0.005 (0.004 - 0.006)	0.005 (0.005 - 0.006)	0.006 (0.005 - 0.007)	0.379 (0.147 - 0.787)	0.430 (0.166 - 0.891)	0.477 (0.184 - 0.983)
Age-standardized (rate per capita)	0.074 (0.064 - 0.085)	0.074 (0.064 - 0.085)	0.074 (0.064 - 0.086)	0.006 (0.005 - 0.007)	0.006 (0.005 - 0.007)	0.006 (0.005 - 0.007)	0.480 (0.186 - 0.991)	0.480 (0.187 - 0.992)	0.486 (0.188 - 1.003)
Total number of cases (thousands)	307,132 (267,144 - 357,059)	428,784 (372,953 - 498,682)	537,506 (465,114 - 625,889)	25,544 (22,256 - 29,596)	34,803 (30,208 - 40,385)	42,373 (36,657 - 49,116)	2,010 (780 - 4,174)	2,807 (1,086 - 5,808)	3,518 (1,357 - 7,247)
**High-income**									
15 - 49 years (rate per capita)	0.066 (0.055 - 0.078)	0.069 (0.058 - 0.082)	0.069 (0.057 - 0.082)	0.007 (0.006 - 0.008)	0.007 (0.006 - 0.009)	0.007 (0.006 - 0.009)	0.437 (0.165 - 0.924)	0.462 (0.175 - 0.969)	0.458 (0.172 - 0.955)
50 - 69 years (rate per capita)	0.173 (0.147 - 0.202)	0.171 (0.145 - 0.199)	0.166 (0.139 - 0.196)	0.010 (0.007 - 0.012)	0.010 (0.007 - 0.012)	0.010 (0.007 - 0.012)	1.127 (0.441 - 2.308)	1.116 (0.436 - 2.283)	1.081 (0.420 - 2.204)
70+ years (rate per capita)	0.130 (0.112 - 0.151)	0.132 (0.114 - 0.151)	0.129 (0.112 - 0.149)	0.006 (0.005 - 0.008)	0.006 (0.005 - 0.008)	0.006 (0.005 - 0.008)	0.815 (0.324 - 1.688)	0.822 (0.325 - 1.698)	0.807 (0.321 - 1.668)
All ages (rate per capita)	0.078 (0.069 - 0.090)	0.086 (0.076 - 0.099)	0.089 (0.077 - 0.103)	0.006 (0.005 - 0.007)	0.006 (0.006 - 0.007)	0.006 (0.006 - 0.007)	0.513 (0.200 - 1.065)	0.562 (0.219 - 1.169)	0.579 (0.224 - 1.195)
Age-standardized (rate per capita)	0.071 (0.062 - 0.082)	0.070 (0.061 - 0.080)	0.068 (0.059 - 0.079)	0.006 (0.005 - 0.006)	0.005 (0.005 - 0.006)	0.005 (0.005 - 0.006)	0.464 (0.181 - 0.962)	0.457 (0.177 - 0.954)	0.445 (0.172 - 0.920)
Total number of cases (thousands)	70,343 (61,466 - 80,829)	85,555 (75,311 - 98,098)	94,907 (82,592 - 110,496)	5,387 (4,682 - 6,192)	6,294 (5,485 - 7,230)	6,809 (5,895 - 7,858)	460 (180 - 956)	559 (218 - 1,164)	620 (240 - 1,278)
**High-income North America**									
15 - 49 years (rate per capita)	0.056 (0.047 - 0.067)	0.056 (0.047 - 0.067)	0.054 (0.045 - 0.064)	0.006 (0.005 - 0.007)	0.006 (0.005 - 0.007)	0.006 (0.005 - 0.007)	0.374 (0.143 - 0.799)	0.372 (0.141 - 0.788)	0.361 (0.136 - 0.765)
50 - 69 years (rate per capita)	0.146 (0.124 - 0.172)	0.143 (0.120 - 0.170)	0.142 (0.120 - 0.169)	0.009 (0.007 - 0.011)	0.009 (0.007 - 0.011)	0.009 (0.007 - 0.011)	0.949 (0.372 - 1.914)	0.930 (0.361 - 1.876)	0.923 (0.360 - 1.868)
70+ years (rate per capita)	0.122 (0.105 - 0.141)	0.122 (0.105 - 0.141)	0.121 (0.104 - 0.140)	0.006 (0.005 - 0.008)	0.006 (0.004 - 0.007)	0.006 (0.004 - 0.007)	0.755 (0.298 - 1.548)	0.754 (0.299 - 1.545)	0.743 (0.294 - 1.514)
All ages (rate per capita)	0.065 (0.057 - 0.074)	0.069 (0.060 - 0.079)	0.073 (0.064 - 0.084)	0.005 (0.005 - 0.006)	0.005 (0.005 - 0.006)	0.005 (0.005 - 0.006)	0.423 (0.165 - 0.871)	0.446 (0.174 - 0.931)	0.472 (0.185 - 0.988)
Age-standardized (rate per capita)	0.061 (0.053 - 0.070)	0.058 (0.051 - 0.067)	0.058 (0.051 - 0.067)	0.005 (0.004 - 0.006)	0.005 (0.004 - 0.005)	0.005 (0.004 - 0.005)	0.397 (0.154 - 0.825)	0.381 (0.148 - 0.792)	0.379 (0.147 - 0.791)
Total number of cases (thousands)	18,009 (15,832 - 20,658)	22,502 (19,673 - 25,997)	26,184 (22,898 - 30,250)	1,441 (1,264 - 1,656)	1,738 (1,511 - 2,002)	1,956 (1,708 - 2,241)	117 (46 - 242)	146 (57 - 306)	170 (67 - 356)
**Canada**									
15 - 49 years (rate per capita)	0.080 (0.066 - 0.095)	0.084 (0.071 - 0.101)	0.082 (0.068 - 0.098)	0.008 (0.007 - 0.010)	0.008 (0.007 - 0.011)	0.008 (0.007 - 0.010)	0.532 (0.205 - 1.118)	0.561 (0.211 - 1.165)	0.544 (0.206 - 1.142)
50 - 69 years (rate per capita)	0.200 (0.170 - 0.235)	0.198 (0.167 - 0.236)	0.198 (0.167 - 0.235)	0.011 (0.008 - 0.014)	0.011 (0.008 - 0.014)	0.011 (0.008 - 0.014)	1.304 (0.512 - 2.669)	1.290 (0.498 - 2.624)	1.292 (0.503 - 2.627)
70+ years (rate per capita)	0.157 (0.135 - 0.182)	0.154 (0.132 - 0.179)	0.153 (0.132 - 0.177)	0.006 (0.005 - 0.009)	0.006 (0.005 - 0.008)	0.006 (0.005 - 0.008)	0.977 (0.393 - 1.986)	0.958 (0.387 - 1.949)	0.948 (0.377 - 1.938)
All ages (rate per capita)	0.090 (0.079 - 0.103)	0.101 (0.088 - 0.117)	0.108 (0.094 - 0.125)	0.007 (0.006 - 0.008)	0.007 (0.006 - 0.009)	0.008 (0.007 - 0.009)	0.590 (0.230 - 1.204)	0.659 (0.254 - 1.378)	0.702 (0.275 - 1.475)
Age-standardized (rate per capita)	0.084 (0.074 - 0.096)	0.082 (0.071 - 0.094)	0.082 (0.072 - 0.095)	0.006 (0.006 - 0.007)	0.006 (0.005 - 0.007)	0.006 (0.005 - 0.007)	0.549 (0.213 - 1.128)	0.535 (0.206 - 1.113)	0.536 (0.210 - 1.127)
Total number of cases (thousands)	2,485 (2,178 - 2,851)	3,247 (2,827 - 3,776)	3,887 (3,401 - 4,510)	192 (168 - 220)	237 (206 - 274)	273 (237 - 313)	16 (6 - 33)	21 (8 - 44)	25 (10 - 53)
**Greenland**									
15 - 49 years (rate per capita)	0.060 (0.050 - 0.071)	0.070 (0.058 - 0.084)	0.066 (0.055 - 0.078)	0.007 (0.005 - 0.008)	0.007 (0.006 - 0.009)	0.007 (0.006 - 0.008)	0.397 (0.150 - 0.832)	0.466 (0.174 - 0.978)	0.437 (0.163 - 0.918)
50 - 69 years (rate per capita)	0.166 (0.139 - 0.197)	0.166 (0.139 - 0.197)	0.166 (0.139 - 0.198)	0.010 (0.007 - 0.012)	0.010 (0.007 - 0.012)	0.010 (0.007 - 0.013)	1.074 (0.417 - 2.180)	1.080 (0.417 - 2.218)	1.082 (0.416 - 2.203)
70+ years (rate per capita)	0.138 (0.118 - 0.161)	0.140 (0.119 - 0.164)	0.143 (0.121 - 0.167)	0.006 (0.004 - 0.009)	0.007 (0.004 - 0.009)	0.007 (0.004 - 0.009)	0.858 (0.343 - 1.746)	0.872 (0.336 - 1.764)	0.890 (0.346 - 1.797)
All ages (rate per capita)	0.052 (0.045 - 0.061)	0.064 (0.055 - 0.075)	0.072 (0.062 - 0.085)	0.005 (0.004 - 0.005)	0.005 (0.004 - 0.006)	0.006 (0.005 - 0.006)	0.345 (0.132 - 0.717)	0.423 (0.163 - 0.869)	0.473 (0.180 - 0.987)
Age-standardized (rate per capita)	0.068 (0.060 - 0.079)	0.068 (0.059 - 0.079)	0.068 (0.059 - 0.079)	0.005 (0.005 - 0.006)	0.005 (0.005 - 0.006)	0.005 (0.005 - 0.006)	0.445 (0.174 - 0.923)	0.445 (0.172 - 0.922)	0.446 (0.173 - 0.924)
Total number of cases (thousands)	3 (3 - 3)	4 (3 - 4)	4 (3 - 5)	0 (0 - 0)	0 (0 - 0)	0 (0 - 0)	0 (0 - 0)	0 (0 - 0)	0 (0 - 0)
**United States**									
15 - 49 years (rate per capita)	0.053 (0.045 - 0.064)	0.053 (0.044 - 0.063)	0.051 (0.043 - 0.061)	0.006 (0.005 - 0.007)	0.006 (0.005 - 0.007)	0.005 (0.005 - 0.007)	0.356 (0.135 - 0.759)	0.351 (0.133 - 0.744)	0.339 (0.128 - 0.718)
50 - 69 years (rate per capita)	0.140 (0.118 - 0.165)	0.137 (0.115 - 0.163)	0.136 (0.114 - 0.161)	0.009 (0.007 - 0.011)	0.009 (0.007 - 0.011)	0.009 (0.006 - 0.011)	0.909 (0.355 - 1.828)	0.888 (0.344 - 1.795)	0.879 (0.343 - 1.776)
70+ years (rate per capita)	0.118 (0.102 - 0.137)	0.118 (0.102 - 0.137)	0.117 (0.101 - 0.135)	0.006 (0.004 - 0.008)	0.006 (0.004 - 0.007)	0.006 (0.004 - 0.007)	0.733 (0.289 - 1.499)	0.730 (0.288 - 1.491)	0.718 (0.284 - 1.459)
All ages (rate per capita)	0.062 (0.055 - 0.071)	0.065 (0.057 - 0.075)	0.069 (0.060 - 0.080)	0.005 (0.004 - 0.006)	0.005 (0.004 - 0.006)	0.005 (0.005 - 0.006)	0.404 (0.158 - 0.835)	0.423 (0.165 - 0.883)	0.447 (0.174 - 0.932)
Age-standardized (rate per capita)	0.058 (0.051 - 0.067)	0.056 (0.049 - 0.064)	0.055 (0.048 - 0.064)	0.005 (0.004 - 0.005)	0.005 (0.004 - 0.005)	0.004 (0.004 - 0.005)	0.380 (0.147 - 0.790)	0.363 (0.141 - 0.755)	0.361 (0.139 - 0.751)
Total number of cases (thousands)	15,513 (13,627 - 17,790)	19,242 (16,793 - 22,181)	22,285 (19,474 - 25,739)	1,249 (1,095 - 1,435)	1,500 (1,304 - 1,725)	1,682 (1,467 - 1,922)	101 (39 - 209)	125 (49 - 261)	145 (56 - 301)
**Australasia**									
15 - 49 years (rate per capita)	0.064 (0.053 - 0.076)	0.064 (0.053 - 0.076)	0.062 (0.052 - 0.074)	0.007 (0.006 - 0.008)	0.007 (0.006 - 0.008)	0.007 (0.005 - 0.008)	0.424 (0.162 - 0.889)	0.425 (0.161 - 0.894)	0.413 (0.155 - 0.862)
50 - 69 years (rate per capita)	0.156 (0.132 - 0.183)	0.154 (0.129 - 0.182)	0.153 (0.128 - 0.180)	0.009 (0.007 - 0.011)	0.009 (0.007 - 0.012)	0.009 (0.007 - 0.012)	1.014 (0.396 - 2.050)	1.003 (0.391 - 2.057)	0.995 (0.383 - 2.015)
70+ years (rate per capita)	0.111 (0.096 - 0.129)	0.111 (0.095 - 0.129)	0.110 (0.095 - 0.128)	0.006 (0.004 - 0.008)	0.006 (0.004 - 0.007)	0.006 (0.004 - 0.007)	0.694 (0.277 - 1.407)	0.692 (0.277 - 1.403)	0.685 (0.273 - 1.378)
All ages (rate per capita)	0.069 (0.060 - 0.079)	0.074 (0.064 - 0.086)	0.076 (0.066 - 0.088)	0.006 (0.005 - 0.006)	0.006 (0.005 - 0.007)	0.006 (0.005 - 0.007)	0.452 (0.177 - 0.923)	0.484 (0.189 - 1.005)	0.498 (0.193 - 1.026)
Age-standardized (rate per capita)	0.066 (0.058 - 0.075)	0.063 (0.055 - 0.073)	0.063 (0.055 - 0.073)	0.005 (0.005 - 0.006)	0.005 (0.005 - 0.006)	0.005 (0.005 - 0.006)	0.433 (0.169 - 0.888)	0.414 (0.162 - 0.859)	0.414 (0.160 - 0.852)
Total number of cases (thousands)	1,405 (1,225 - 1,604)	1,814 (1,577 - 2,097)	2,198 (1,916 - 2,537)	114 (101 - 131)	143 (124 - 165)	171 (149 - 197)	9 (4 - 19)	12 (5 - 25)	14 (6 - 30)
**Australia**									
15 - 49 years (rate per capita)	0.067 (0.055 - 0.080)	0.066 (0.055 - 0.078)	0.064 (0.053 - 0.076)	0.007 (0.006 - 0.008)	0.007 (0.006 - 0.009)	0.007 (0.006 - 0.008)	0.448 (0.170 - 0.931)	0.440 (0.166 - 0.926)	0.425 (0.159 - 0.884)
50 - 69 years (rate per capita)	0.162 (0.137 - 0.190)	0.159 (0.134 - 0.189)	0.158 (0.133 - 0.187)	0.009 (0.007 - 0.012)	0.010 (0.007 - 0.012)	0.010 (0.007 - 0.012)	1.056 (0.413 - 2.129)	1.041 (0.405 - 2.141)	1.032 (0.398 - 2.094)
70+ years (rate per capita)	0.116 (0.100 - 0.135)	0.115 (0.099 - 0.135)	0.114 (0.098 - 0.133)	0.006 (0.004 - 0.008)	0.006 (0.004 - 0.008)	0.006 (0.004 - 0.007)	0.723 (0.288 - 1.470)	0.720 (0.287 - 1.467)	0.710 (0.283 - 1.434)
All ages (rate per capita)	0.072 (0.063 - 0.082)	0.077 (0.067 - 0.089)	0.079 (0.068 - 0.091)	0.006 (0.005 - 0.007)	0.006 (0.005 - 0.007)	0.006 (0.005 - 0.007)	0.475 (0.186 - 0.972)	0.506 (0.198 - 1.046)	0.514 (0.199 - 1.060)
Age-standardized (rate per capita)	0.069 (0.060 - 0.079)	0.066 (0.057 - 0.076)	0.065 (0.057 - 0.076)	0.006 (0.005 - 0.006)	0.005 (0.005 - 0.006)	0.005 (0.005 - 0.006)	0.453 (0.177 - 0.928)	0.430 (0.169 - 0.892)	0.429 (0.166 - 0.882)
Total number of cases (thousands)	1,233 (1,075 - 1,402)	1,575 (1,366 - 1,818)	1,913 (1,662 - 2,210)	99 (87 - 114)	123 (107 - 142)	148 (129 - 171)	8 (3 - 17)	10 (4 - 21)	13 (5 - 26)
**New Zealand**									
15 - 49 years (rate per capita)	0.045 (0.038 - 0.054)	0.052 (0.044 - 0.062)	0.052 (0.044 - 0.062)	0.005 (0.004 - 0.006)	0.005 (0.005 - 0.007)	0.005 (0.005 - 0.007)	0.303 (0.114 - 0.628)	0.346 (0.131 - 0.737)	0.349 (0.131 - 0.741)
50 - 69 years (rate per capita)	0.122 (0.102 - 0.145)	0.123 (0.103 - 0.147)	0.123 (0.103 - 0.146)	0.008 (0.006 - 0.010)	0.008 (0.006 - 0.010)	0.008 (0.006 - 0.010)	0.797 (0.307 - 1.614)	0.803 (0.310 - 1.629)	0.805 (0.310 - 1.625)
70+ years (rate per capita)	0.087 (0.075 - 0.103)	0.087 (0.075 - 0.102)	0.088 (0.076 - 0.103)	0.005 (0.004 - 0.007)	0.005 (0.004 - 0.007)	0.005 (0.004 - 0.007)	0.547 (0.216 - 1.096)	0.545 (0.215 - 1.093)	0.548 (0.218 - 1.104)
All ages (rate per capita)	0.051 (0.044 - 0.059)	0.058 (0.051 - 0.067)	0.062 (0.054 - 0.072)	0.004 (0.004 - 0.005)	0.005 (0.004 - 0.006)	0.005 (0.004 - 0.006)	0.334 (0.129 - 0.676)	0.379 (0.147 - 0.787)	0.409 (0.158 - 0.843)
Age-standardized (rate per capita)	0.050 (0.044 - 0.058)	0.051 (0.044 - 0.059)	0.051 (0.045 - 0.059)	0.004 (0.004 - 0.005)	0.004 (0.004 - 0.005)	0.004 (0.004 - 0.005)	0.329 (0.126 - 0.669)	0.333 (0.130 - 0.691)	0.337 (0.131 - 0.693)
Total number of cases (thousands)	172 (150 - 199)	239 (209 - 278)	285 (246 - 330)	15 (13 - 17)	20 (17 - 23)	23 (20 - 27)	1 (0 - 2)	2 (1 - 3)	2 (1 - 4)
**High-income Asia Pacific**									
15 - 49 years (rate per capita)	0.059 (0.049 - 0.071)	0.065 (0.054 - 0.077)	0.069 (0.057 - 0.083)	0.006 (0.005 - 0.008)	0.007 (0.006 - 0.008)	0.007 (0.006 - 0.009)	0.396 (0.149 - 0.839)	0.433 (0.163 - 0.919)	0.465 (0.176 - 0.984)
50 - 69 years (rate per capita)	0.163 (0.137 - 0.194)	0.162 (0.136 - 0.193)	0.161 (0.136 - 0.190)	0.010 (0.007 - 0.012)	0.010 (0.007 - 0.012)	0.010 (0.007 - 0.012)	1.072 (0.416 - 2.181)	1.068 (0.412 - 2.182)	1.061 (0.413 - 2.152)
70+ years (rate per capita)	0.127 (0.109 - 0.148)	0.125 (0.107 - 0.144)	0.123 (0.106 - 0.142)	0.006 (0.005 - 0.008)	0.006 (0.005 - 0.008)	0.006 (0.005 - 0.007)	0.804 (0.319 - 1.656)	0.789 (0.310 - 1.627)	0.775 (0.305 - 1.581)
All ages (rate per capita)	0.072 (0.063 - 0.084)	0.087 (0.075 - 0.100)	0.093 (0.082 - 0.107)	0.006 (0.005 - 0.007)	0.006 (0.006 - 0.007)	0.007 (0.006 - 0.008)	0.476 (0.184 - 0.995)	0.569 (0.221 - 1.181)	0.612 (0.239 - 1.276)
Age-standardized (rate per capita)	0.065 (0.057 - 0.075)	0.066 (0.057 - 0.076)	0.066 (0.057 - 0.076)	0.005 (0.005 - 0.006)	0.005 (0.005 - 0.006)	0.005 (0.005 - 0.006)	0.429 (0.167 - 0.899)	0.434 (0.167 - 0.905)	0.433 (0.166 - 0.907)
Total number of cases (thousands)	12,187 (10,584 - 14,121)	15,341 (13,350 - 17,673)	17,063 (14,911 - 19,527)	983 (854 - 1,138)	1,140 (993 - 1,307)	1,216 (1,063 - 1,395)	80 (31 - 168)	101 (39 - 209)	112 (44 - 233)
**Brunei**									
15 - 49 years (rate per capita)	0.051 (0.042 - 0.061)	0.056 (0.046 - 0.066)	0.059 (0.050 - 0.071)	0.006 (0.005 - 0.007)	0.006 (0.005 - 0.007)	0.006 (0.005 - 0.008)	0.340 (0.129 - 0.708)	0.372 (0.140 - 0.788)	0.397 (0.150 - 0.831)
50 - 69 years (rate per capita)	0.157 (0.132 - 0.186)	0.156 (0.130 - 0.188)	0.156 (0.131 - 0.187)	0.010 (0.007 - 0.012)	0.010 (0.007 - 0.012)	0.010 (0.007 - 0.012)	1.027 (0.393 - 2.073)	1.025 (0.393 - 2.094)	1.027 (0.389 - 2.075)

	Prevalence 1990	Prevalence 2005	Prevalence 2015	Incidence 1990	Incidence 2005	Incidence 2015	YLD rate 1990	YLD rate 2005	YLD rate 2015
70+ years (rate per capita)	0.122 (0.105 - 0.141)	0.122 (0.104 - 0.142)	0.122 (0.104 - 0.143)	0.006 (0.004 - 0.007)	0.006 (0.004 - 0.008)	0.006 (0.004 - 0.008)	0.764 (0.304 - 1.570)	0.765 (0.303 - 1.566)	0.765 (0.301 - 1.537)
All ages (rate per capita)	0.041 (0.035 - 0.048)	0.051 (0.044 - 0.060)	0.062 (0.054 - 0.073)	0.004 (0.003 - 0.005)	0.005 (0.004 - 0.006)	0.005 (0.005 - 0.006)	0.273 (0.105 - 0.568)	0.340 (0.130 - 0.712)	0.412 (0.157 - 0.850)
Age-standardized (rate per capita)	0.064 (0.056 - 0.075)	0.064 (0.055 - 0.074)	0.064 (0.055 - 0.074)	0.005 (0.004 - 0.006)	0.005 (0.004 - 0.006)	0.005 (0.004 - 0.006)	0.422 (0.163 - 0.879)	0.419 (0.162 - 0.880)	0.419 (0.160 - 0.870)
Total number of cases (thousands)	11 (9 - 12)	19 (16 - 22)	26 (23 - 31)	1 (1 - 1)	2 (1 - 2)	2 (2 - 3)	0 (0 - 0)	0 (0 - 0)	0 (0 - 0)
**Japan**									
15 - 49 years (rate per capita)	0.062 (0.052 - 0.075)	0.066 (0.054 - 0.078)	0.072 (0.059 - 0.085)	0.007 (0.006 - 0.008)	0.007 (0.006 - 0.009)	0.007 (0.006 - 0.009)	0.418 (0.157 - 0.892)	0.441 (0.165 - 0.936)	0.479 (0.180 - 1.013)
50 - 69 years (rate per capita)	0.165 (0.139 - 0.196)	0.164 (0.138 - 0.196)	0.163 (0.138 - 0.192)	0.010 (0.008 - 0.013)	0.010 (0.007 - 0.012)	0.010 (0.007 - 0.012)	1.085 (0.419 - 2.212)	1.081 (0.416 - 2.209)	1.075 (0.420 - 2.186)
70+ years (rate per capita)	0.128 (0.110 - 0.149)	0.126 (0.108 - 0.145)	0.124 (0.107 - 0.143)	0.006 (0.005 - 0.008)	0.006 (0.005 - 0.008)	0.006 (0.005 - 0.008)	0.810 (0.322 - 1.671)	0.794 (0.312 - 1.636)	0.780 (0.306 - 1.588)
All ages (rate per capita)	0.079 (0.069 - 0.092)	0.092 (0.081 - 0.107)	0.097 (0.085 - 0.111)	0.006 (0.005 - 0.007)	0.007 (0.006 - 0.008)	0.007 (0.006 - 0.008)	0.521 (0.202 - 1.086)	0.606 (0.236 - 1.252)	0.638 (0.250 - 1.321)
Age-standardized (rate per capita)	0.065 (0.057 - 0.076)	0.067 (0.058 - 0.077)	0.066 (0.058 - 0.076)	0.005 (0.005 - 0.006)	0.005 (0.005 - 0.006)	0.005 (0.005 - 0.006)	0.431 (0.167 - 0.904)	0.440 (0.169 - 0.915)	0.439 (0.168 - 0.917)
Total number of cases (thousands)	9,706 (8,442 - 11,273)	11,637 (10,149 - 13,428)	12,499 (10,922 - 14,258)	762 (657 - 887)	839 (729 - 960)	869 (763 - 992)	64 (25 - 133)	76 (30 - 158)	82 (32 - 169)
**Singapore**									
15 - 49 years (rate per capita)	0.056 (0.046 - 0.066)	0.067 (0.055 - 0.080)	0.065 (0.054 - 0.077)	0.006 (0.005 - 0.008)	0.007 (0.006 - 0.008)	0.007 (0.006 - 0.008)	0.373 (0.141 - 0.784)	0.447 (0.169 - 0.949)	0.433 (0.163 - 0.917)
50 - 69 years (rate per capita)	0.156 (0.132 - 0.185)	0.156 (0.131 - 0.187)	0.157 (0.131 - 0.186)	0.009 (0.007 - 0.012)	0.010 (0.007 - 0.012)	0.009 (0.007 - 0.012)	1.029 (0.403 - 2.097)	1.032 (0.395 - 2.098)	1.036 (0.399 - 2.124)
70+ years (rate per capita)	0.121 (0.104 - 0.141)	0.119 (0.103 - 0.138)	0.117 (0.101 - 0.137)	0.006 (0.004 - 0.008)	0.006 (0.004 - 0.007)	0.006 (0.004 - 0.007)	0.768 (0.301 - 1.572)	0.757 (0.301 - 1.560)	0.746 (0.297 - 1.535)
All ages (rate per capita)	0.059 (0.051 - 0.067)	0.074 (0.063 - 0.086)	0.081 (0.070 - 0.094)	0.005 (0.004 - 0.006)	0.006 (0.005 - 0.007)	0.006 (0.005 - 0.007)	0.389 (0.149 - 0.796)	0.489 (0.186 - 1.026)	0.536 (0.205 - 1.107)
Age-standardized (rate per capita)	0.064 (0.055 - 0.073)	0.063 (0.055 - 0.074)	0.063 (0.055 - 0.073)	0.005 (0.004 - 0.006)	0.005 (0.004 - 0.006)	0.005 (0.004 - 0.006)	0.420 (0.164 - 0.869)	0.420 (0.162 - 0.874)	0.420 (0.161 - 0.875)
Total number of cases (thousands)	161 (139 - 185)	262 (225 - 307)	317 (275 - 370)	14 (12 - 16)	21 (18 - 25)	24 (21 - 28)	1 (0 - 2)	2 (1 - 4)	2 (1 - 4)
**South Korea**									
15 - 49 years (rate per capita)	0.051 (0.043 - 0.061)	0.062 (0.052 - 0.075)	0.066 (0.055 - 0.079)	0.006 (0.005 - 0.007)	0.007 (0.005 - 0.008)	0.007 (0.006 - 0.008)	0.342 (0.129 - 0.713)	0.417 (0.158 - 0.873)	0.439 (0.165 - 0.933)
50 - 69 years (rate per capita)	0.155 (0.130 - 0.183)	0.155 (0.130 - 0.185)	0.156 (0.129 - 0.185)	0.009 (0.007 - 0.012)	0.009 (0.007 - 0.012)	0.009 (0.007 - 0.012)	1.013 (0.392 - 2.050)	1.020 (0.392 - 2.078)	1.023 (0.398 - 2.095)
70+ years (rate per capita)	0.121 (0.102 - 0.141)	0.121 (0.103 - 0.140)	0.119 (0.102 - 0.139)	0.006 (0.004 - 0.008)	0.006 (0.004 - 0.008)	0.006 (0.004 - 0.007)	0.758 (0.301 - 1.513)	0.758 (0.298 - 1.516)	0.750 (0.299 - 1.529)
All ages (rate per capita)	0.054 (0.046 - 0.062)	0.072 (0.062 - 0.084)	0.084 (0.073 - 0.098)	0.005 (0.004 - 0.006)	0.006 (0.005 - 0.007)	0.006 (0.005 - 0.007)	0.354 (0.135 - 0.741)	0.477 (0.184 - 0.995)	0.553 (0.215 - 1.139)
Age-standardized (rate per capita)	0.063 (0.055 - 0.073)	0.063 (0.055 - 0.073)	0.063 (0.055 - 0.074)	0.005 (0.004 - 0.006)	0.005 (0.004 - 0.006)	0.005 (0.004 - 0.006)	0.415 (0.160 - 0.869)	0.417 (0.162 - 0.874)	0.418 (0.163 - 0.872)
Total number of cases (thousands)	2,310 (1,993 - 2,685)	3,422 (2,956 - 3,996)	4,221 (3,654 - 4,903)	206 (178 - 237)	278 (241 - 322)	320 (275 - 372)	15 (6 - 32)	23 (9 - 47)	28 (11 - 57)
**Western Europe**									
15 - 49 years (rate per capita)	0.075 (0.063 - 0.090)	0.082 (0.069 - 0.097)	0.080 (0.065 - 0.097)	0.008 (0.006 - 0.009)	0.008 (0.007 - 0.010)	0.008 (0.006 - 0.010)	0.499 (0.188 - 1.048)	0.543 (0.206 - 1.146)	0.530 (0.198 - 1.108)
50 - 69 years (rate per capita)	0.190 (0.161 - 0.221)	0.192 (0.166 - 0.221)	0.184 (0.153 - 0.217)	0.010 (0.008 - 0.013)	0.011 (0.008 - 0.013)	0.010 (0.007 - 0.013)	1.241 (0.487 - 2.528)	1.256 (0.494 - 2.590)	1.201 (0.470 - 2.462)
70+ years (rate per capita)	0.135 (0.116 - 0.158)	0.139 (0.121 - 0.158)	0.136 (0.116 - 0.158)	0.006 (0.005 - 0.008)	0.006 (0.005 - 0.008)	0.006 (0.005 - 0.008)	0.846 (0.334 - 1.757)	0.868 (0.342 - 1.783)	0.848 (0.337 - 1.757)
All ages (rate per capita)	0.092 (0.080 - 0.106)	0.101 (0.089 - 0.114)	0.101 (0.087 - 0.118)	0.007 (0.006 - 0.008)	0.007 (0.006 - 0.008)	0.007 (0.006 - 0.008)	0.601 (0.235 - 1.248)	0.658 (0.258 - 1.370)	0.659 (0.256 - 1.375)
Age-standardized (rate per capita)	0.079 (0.069 - 0.091)	0.079 (0.070 - 0.089)	0.075 (0.064 - 0.088)	0.006 (0.005 - 0.007)	0.006 (0.005 - 0.007)	0.006 (0.005 - 0.007)	0.517 (0.202 - 1.076)	0.515 (0.201 - 1.069)	0.492 (0.190 - 1.025)
Total number of cases (thousands)	35,059 (30,641 - 40,239)	40,991 (36,271 - 46,430)	43,743 (37,790 - 51,065)	2,562 (2,221 - 2,927)	2,901 (2,525 - 3,322)	3,041 (2,612 - 3,542)	229 (90 - 476)	268 (105 - 558)	286 (111 - 596)
**Andorra**									
15 - 49 years (rate per capita)	0.082 (0.055 - 0.130)	0.091 (0.061 - 0.145)	0.095 (0.062 - 0.154)	0.009 (0.006 - 0.014)	0.010 (0.006 - 0.014)	0.010 (0.006 - 0.015)	0.549 (0.184 - 1.237)	0.610 (0.203 - 1.398)	0.637 (0.210 - 1.443)
50 - 69 years (rate per capita)	0.220 (0.146 - 0.302)	0.219 (0.146 - 0.302)	0.221 (0.148 - 0.301)	0.014 (0.007 - 0.020)	0.013 (0.007 - 0.020)	0.014 (0.007 - 0.020)	1.437 (0.510 - 3.238)	1.432 (0.519 - 3.272)	1.444 (0.511 - 3.302)
70+ years (rate per capita)	0.160 (0.105 - 0.218)	0.159 (0.104 - 0.217)	0.161 (0.104 - 0.216)	0.009 (0.005 - 0.016)	0.009 (0.004 - 0.016)	0.009 (0.004 - 0.016)	1.002 (0.361 - 2.129)	0.996 (0.356 - 2.123)	1.001 (0.360 - 2.144)
All ages (rate per capita)	0.104 (0.075 - 0.144)	0.114 (0.081 - 0.160)	0.122 (0.086 - 0.166)	0.008 (0.006 - 0.011)	0.009 (0.006 - 0.012)	0.009 (0.006 - 0.012)	0.684 (0.243 - 1.497)	0.746 (0.268 - 1.625)	0.796 (0.286 - 1.759)
Age-standardized (rate per capita)	0.089 (0.063 - 0.125)	0.089 (0.064 - 0.125)	0.089 (0.064 - 0.125)	0.007 (0.005 - 0.010)	0.007 (0.005 - 0.010)	0.007 (0.005 - 0.010)	0.585 (0.206 - 1.289)	0.583 (0.208 - 1.288)	0.587 (0.208 - 1.299)
Total number of cases (thousands)	5 (4 - 7)	8 (6 - 12)	10 (7 - 13)	0 (0 - 1)	1 (0 - 1)	1 (0 - 1)	0 (0 - 0)	0 (0 - 0)	0 (0 - 0)
**Austria**									
15 - 49 years (rate per capita)	0.082 (0.055 - 0.131)	0.093 (0.061 - 0.145)	0.095 (0.062 - 0.150)	0.009 (0.006 - 0.014)	0.010 (0.006 - 0.015)	0.010 (0.006 - 0.015)	0.548 (0.185 - 1.275)	0.619 (0.204 - 1.414)	0.637 (0.211 - 1.470)
50 - 69 years (rate per capita)	0.215 (0.142 - 0.294)	0.216 (0.143 - 0.290)	0.217 (0.143 - 0.295)	0.013 (0.007 - 0.020)	0.014 (0.007 - 0.020)	0.014 (0.007 - 0.020)	1.401 (0.501 - 3.118)	1.410 (0.510 - 3.160)	1.419 (0.510 - 3.214)
70+ years (rate per capita)	0.148 (0.097 - 0.199)	0.150 (0.098 - 0.198)	0.154 (0.100 - 0.203)	0.009 (0.004 - 0.016)	0.009 (0.004 - 0.017)	0.009 (0.005 - 0.017)	0.924 (0.335 - 1.974)	0.935 (0.346 - 2.021)	0.958 (0.347 - 2.026)
All ages (rate per capita)	0.103 (0.073 - 0.142)	0.112 (0.080 - 0.153)	0.121 (0.087 - 0.166)	0.008 (0.006 - 0.011)	0.009 (0.006 - 0.012)	0.009 (0.006 - 0.013)	0.670 (0.239 - 1.486)	0.735 (0.262 - 1.591)	0.790 (0.283 - 1.749)
Age-standardized (rate per capita)	0.088 (0.062 - 0.123)	0.087 (0.062 - 0.121)	0.088 (0.063 - 0.123)	0.007 (0.005 - 0.010)	0.007 (0.005 - 0.010)	0.007 (0.005 - 0.010)	0.574 (0.203 - 1.266)	0.573 (0.204 - 1.258)	0.577 (0.205 - 1.282)
Total number of cases (thousands)	786 (560 - 1,085)	924 (659 - 1,261)	1,048 (751 - 1,438)	64 (45 - 88)	74 (50 - 101)	81 (54 - 110)	5 (2 - 11)	6 (2 - 13)	7 (2 - 15)
**Belgium**									
15 - 49 years (rate per capita)	0.103 (0.086 - 0.124)	0.114 (0.094 - 0.138)	0.113 (0.093 - 0.136)	0.011 (0.009 - 0.013)	0.011 (0.009 - 0.014)	0.011 (0.009 - 0.013)	0.687 (0.260 - 1.414)	0.756 (0.286 - 1.564)	0.750 (0.282 - 1.551)
50 - 69 years (rate per capita)	0.241 (0.204 - 0.282)	0.243 (0.204 - 0.286)	0.244 (0.204 - 0.288)	0.013 (0.009 - 0.016)	0.013 (0.009 - 0.016)	0.013 (0.009 - 0.016)	1.569 (0.614 - 3.168)	1.581 (0.610 - 3.205)	1.588 (0.617 - 3.210)
70+ years (rate per capita)	0.151 (0.128 - 0.176)	0.153 (0.130 - 0.179)	0.150 (0.129 - 0.174)	0.007 (0.005 - 0.009)	0.007 (0.005 - 0.009)	0.007 (0.005 - 0.009)	0.935 (0.375 - 1.900)	0.949 (0.384 - 1.958)	0.931 (0.375 - 1.903)
All ages (rate per capita)	0.120 (0.103 - 0.138)	0.128 (0.112 - 0.150)	0.132 (0.114 - 0.153)	0.009 (0.008 - 0.010)	0.009 (0.008 - 0.010)	0.009 (0.008 - 0.010)	0.784 (0.303 - 1.618)	0.837 (0.327 - 1.745)	0.859 (0.333 - 1.767)
Age-standardized (rate per capita)	0.102 (0.088 - 0.118)	0.102 (0.089 - 0.119)	0.102 (0.089 - 0.119)	0.008 (0.007 - 0.009)	0.008 (0.007 - 0.009)	0.008 (0.007 - 0.009)	0.670 (0.259 - 1.375)	0.669 (0.259 - 1.408)	0.671 (0.259 - 1.391)
Total number of cases (thousands)	1,194 (1,029 - 1,375)	1,344 (1,171 - 1,567)	1,492 (1,291 - 1,735)	87 (76 - 99)	95 (82 - 110)	102 (89 - 117)	8 (3 - 16)	9 (3 - 18)	10 (4 - 20)
**Cyprus**									
15 - 49 years (rate per capita)	0.079 (0.052 - 0.126)	0.084 (0.055 - 0.134)	0.082 (0.055 - 0.128)	0.009 (0.006 - 0.013)	0.009 (0.006 - 0.014)	0.009 (0.006 - 0.013)	0.528 (0.179 - 1.198)	0.559 (0.184 - 1.294)	0.545 (0.185 - 1.231)
50 - 69 years (rate per capita)	0.222 (0.147 - 0.305)	0.220 (0.148 - 0.303)	0.222 (0.147 - 0.306)	0.014 (0.007 - 0.020)	0.013 (0.007 - 0.020)	0.014 (0.007 - 0.020)	1.449 (0.519 - 3.310)	1.435 (0.511 - 3.208)	1.450 (0.517 - 3.310)
70+ years (rate per capita)	0.164 (0.109 - 0.221)	0.162 (0.108 - 0.218)	0.164 (0.107 - 0.221)	0.009 (0.004 - 0.016)	0.009 (0.004 - 0.016)	0.009 (0.004 - 0.016)	1.022 (0.372 - 2.174)	1.008 (0.360 - 2.158)	1.018 (0.374 - 2.169)
All ages (rate per capita)	0.088 (0.063 - 0.123)	0.102 (0.072 - 0.142)	0.109 (0.078 - 0.152)	0.007 (0.005 - 0.010)	0.008 (0.006 - 0.011)	0.009 (0.006 - 0.012)	0.580 (0.207 - 1.276)	0.665 (0.238 - 1.445)	0.714 (0.256 - 1.554)
Age-standardized (rate per capita)	0.089 (0.063 - 0.125)	0.089 (0.063 - 0.124)	0.089 (0.064 - 0.124)	0.007 (0.005 - 0.010)	0.007 (0.005 - 0.010)	0.007 (0.005 - 0.010)	0.586 (0.210 - 1.289)	0.582 (0.207 - 1.267)	0.586 (0.208 - 1.297)
Total number of cases (thousands)	59 (42 - 82)	77 (54 - 107)	97 (70 - 135)	5 (3 - 7)	6 (4 - 8)	8 (5 - 11)	0 (0 - 1)	1 (0 - 1)	1 (0 - 1)
**Denmark**									
15 - 49 years (rate per capita)	0.132 (0.111 - 0.159)	0.138 (0.115 - 0.167)	0.139 (0.115 - 0.166)	0.013 (0.011 - 0.015)	0.013 (0.011 - 0.016)	0.013 (0.011 - 0.016)	0.880 (0.333 - 1.841)	0.920 (0.348 - 1.894)	0.922 (0.349 - 1.910)
50 - 69 years (rate per capita)	0.307 (0.261 - 0.358)	0.304 (0.259 - 0.357)	0.306 (0.262 - 0.355)	0.014 (0.010 - 0.018)	0.014 (0.010 - 0.018)	0.014 (0.010 - 0.017)	1.998 (0.776 - 4.035)	1.985 (0.774 - 4.006)	1.997 (0.785 - 4.042)
70+ years (rate per capita)	0.212 (0.184 - 0.243)	0.208 (0.180 - 0.238)	0.213 (0.186 - 0.244)	0.007 (0.005 - 0.009)	0.007 (0.005 - 0.009)	0.007 (0.005 - 0.009)	1.328 (0.537 - 2.683)	1.298 (0.528 - 2.682)	1.335 (0.543 - 2.676)
All ages (rate per capita)	0.153 (0.135 - 0.177)	0.160 (0.139 - 0.185)	0.167 (0.148 - 0.193)	0.010 (0.009 - 0.011)	0.010 (0.009 - 0.011)	0.010 (0.009 - 0.012)	1.003 (0.392 - 2.112)	1.047 (0.410 - 2.164)	1.094 (0.430 - 2.265)
Age-standardized (rate per capita)	0.129 (0.114 - 0.149)	0.127 (0.111 - 0.147)	0.130 (0.114 - 0.150)	0.009 (0.008 - 0.010)	0.009 (0.008 - 0.010)	0.009 (0.008 - 0.010)	0.848 (0.331 - 1.780)	0.835 (0.326 - 1.740)	0.851 (0.335 - 1.755)
Total number of cases (thousands)	788 (692 - 907)	867 (754 - 1,001)	956 (843 - 1,104)	52 (46 - 59)	55 (48 - 62)	58 (51 - 66)	5 (2 - 11)	6 (2 - 12)	6 (2 - 13)
**Finland**									
15 - 49 years (rate per capita)	0.062 (0.052 - 0.074)	0.063 (0.052 - 0.076)	0.061 (0.051 - 0.073)	0.006 (0.005 - 0.008)	0.006 (0.005 - 0.008)	0.006 (0.005 - 0.008)	0.413 (0.155 - 0.876)	0.419 (0.159 - 0.878)	0.406 (0.153 - 0.852)
50 - 69 years (rate per capita)	0.141 (0.118 - 0.166)	0.144 (0.120 - 0.171)	0.144 (0.120 - 0.170)	0.009 (0.007 - 0.011)	0.009 (0.007 - 0.011)	0.009 (0.007 - 0.011)	0.918 (0.357 - 1.861)	0.939 (0.361 - 1.901)	0.938 (0.366 - 1.888)
70+ years (rate per capita)	0.094 (0.081 - 0.110)	0.098 (0.084 - 0.114)	0.098 (0.084 - 0.114)	0.006 (0.004 - 0.007)	0.006 (0.004 - 0.007)	0.006 (0.004 - 0.007)	0.586 (0.230 - 1.206)	0.609 (0.239 - 1.247)	0.608 (0.244 - 1.252)
All ages (rate per capita)	0.069 (0.060 - 0.080)	0.077 (0.067 - 0.089)	0.079 (0.069 - 0.092)	0.006 (0.005 - 0.006)	0.006 (0.005 - 0.007)	0.006 (0.005 - 0.007)	0.450 (0.174 - 0.927)	0.500 (0.194 - 1.037)	0.518 (0.200 - 1.062)
Age-standardized (rate per capita)	0.059 (0.051 - 0.068)	0.060 (0.052 - 0.069)	0.060 (0.051 - 0.069)	0.005 (0.004 - 0.006)	0.005 (0.004 - 0.006)	0.005 (0.004 - 0.006)	0.386 (0.149 - 0.796)	0.390 (0.152 - 0.805)	0.391 (0.148 - 0.804)
Total number of cases (thousands)	343 (299 - 397)	402 (349 - 466)	441 (383 - 509)	28 (24 - 32)	31 (27 - 35)	33 (29 - 38)	2 (1 - 5)	3 (1 - 5)	3 (1 - 6)
**France**									

	Prevalence 1990	Prevalence 2005	Prevalence 2015	Incidence 1990	Incidence 2005	Incidence 2015	YLD rate 1990	YLD rate 2005	YLD rate 2015
15 - 49 years (rate per capita)	0.059 (0.049 - 0.070)	0.064 (0.054 - 0.076)	0.065 (0.054 - 0.078)	0.006 (0.005 - 0.008)	0.007 (0.005 - 0.008)	0.007 (0.005 - 0.008)	0.394 (0.149 - 0.814)	0.427 (0.162 - 0.895)	0.432 (0.164 - 0.914)
50 - 69 years (rate per capita)	0.151 (0.127 - 0.177)	0.154 (0.129 - 0.182)	0.153 (0.129 - 0.181)	0.009 (0.007 - 0.011)	0.009 (0.007 - 0.011)	0.009 (0.007 - 0.011)	0.983 (0.383 - 1.994)	1.007 (0.389 - 2.057)	1.003 (0.393 - 2.022)
70+ years (rate per capita)	0.108 (0.093 - 0.126)	0.111 (0.096 - 0.130)	0.109 (0.094 - 0.127)	0.006 (0.004 - 0.007)	0.006 (0.004 - 0.007)	0.006 (0.004 - 0.007)	0.675 (0.266 - 1.359)	0.696 (0.273 - 1.414)	0.680 (0.267 - 1.389)
All ages (rate per capita)	0.070 (0.061 - 0.081)	0.078 (0.068 - 0.090)	0.081 (0.070 - 0.093)	0.005 (0.005 - 0.006)	0.006 (0.005 - 0.007)	0.006 (0.005 - 0.007)	0.460 (0.178 - 0.953)	0.510 (0.197 - 1.065)	0.528 (0.208 - 1.087)
Age-standardized (rate per capita)	0.063 (0.054 - 0.072)	0.063 (0.055 - 0.073)	0.063 (0.055 - 0.073)	0.005 (0.004 - 0.006)	0.005 (0.004 - 0.006)	0.005 (0.004 - 0.006)	0.410 (0.158 - 0.849)	0.415 (0.160 - 0.871)	0.414 (0.161 - 0.856)
Total number of cases (thousands)	3,988 (3,480 - 4,572)	4,770 (4,158 - 5,505)	5,280 (4,592 - 6,053)	308 (269 - 352)	356 (309 - 408)	383 (332 - 439)	26 (10 - 54)	31 (12 - 65)	34 (14 - 71)
**Germany**									
15 - 49 years (rate per capita)	0.121 (0.103 - 0.139)	0.137 (0.123 - 0.149)	0.124 (0.104 - 0.149)	0.012 (0.010 - 0.014)	0.013 (0.011 - 0.014)	0.012 (0.010 - 0.014)	0.803 (0.306 - 1.682)	0.913 (0.363 - 1.930)	0.825 (0.310 - 1.725)
50 - 69 years (rate per capita)	0.272 (0.236 - 0.315)	0.290 (0.264 - 0.311)	0.261 (0.222 - 0.307)	0.013 (0.010 - 0.016)	0.014 (0.010 - 0.017)	0.013 (0.009 - 0.016)	1.778 (0.700 - 3.592)	1.890 (0.765 - 3.843)	1.707 (0.667 - 3.453)
70+ years (rate per capita)	0.177 (0.153 - 0.203)	0.203 (0.181 - 0.222)	0.181 (0.157 - 0.209)	0.006 (0.005 - 0.008)	0.007 (0.005 - 0.009)	0.007 (0.005 - 0.009)	1.103 (0.445 - 2.278)	1.272 (0.513 - 2.639)	1.135 (0.459 - 2.343)
All ages (rate per capita)	0.143 (0.127 - 0.161)	0.164 (0.152 - 0.173)	0.154 (0.135 - 0.177)	0.010 (0.008 - 0.011)	0.010 (0.009 - 0.011)	0.010 (0.008 - 0.011)	0.938 (0.370 - 1.941)	1.071 (0.431 - 2.191)	1.006 (0.393 - 2.116)
Age-standardized (rate per capita)	0.117 (0.104 - 0.131)	0.122 (0.113 - 0.129)	0.109 (0.095 - 0.126)	0.008 (0.007 - 0.009)	0.009 (0.008 - 0.009)	0.008 (0.007 - 0.009)	0.767 (0.302 - 1.592)	0.800 (0.323 - 1.648)	0.713 (0.278 - 1.499)
Total number of cases (thousands)	11,340 (10,094 - 12,715)	13,506 (12,488 - 14,240)	12,903 (11,330 - 14,827)	758 (670 - 848)	861 (778 - 941)	806 (701 - 918)	74 (29 - 154)	88 (36 - 180)	84 (33 - 177)
**Greece**									
15 - 49 years (rate per capita)	0.065 (0.055 - 0.076)	0.066 (0.055 - 0.078)	0.073 (0.061 - 0.087)	0.007 (0.006 - 0.008)	0.007 (0.006 - 0.008)	0.007 (0.006 - 0.009)	0.434 (0.167 - 0.909)	0.438 (0.165 - 0.931)	0.486 (0.184 - 1.007)
50 - 69 years (rate per capita)	0.168 (0.140 - 0.197)	0.154 (0.130 - 0.183)	0.155 (0.130 - 0.183)	0.010 (0.007 - 0.012)	0.009 (0.007 - 0.012)	0.009 (0.007 - 0.012)	1.096 (0.428 - 2.220)	1.010 (0.391 - 2.056)	1.016 (0.394 - 2.047)
70+ years (rate per capita)	0.111 (0.096 - 0.130)	0.109 (0.093 - 0.127)	0.107 (0.092 - 0.124)	0.006 (0.004 - 0.008)	0.006 (0.004 - 0.008)	0.006 (0.004 - 0.007)	0.697 (0.274 - 1.423)	0.682 (0.266 - 1.406)	0.667 (0.265 - 1.343)
All ages (rate per capita)	0.081 (0.071 - 0.093)	0.082 (0.071 - 0.094)	0.088 (0.077 - 0.102)	0.006 (0.005 - 0.007)	0.006 (0.006 - 0.007)	0.007 (0.006 - 0.008)	0.531 (0.209 - 1.088)	0.534 (0.207 - 1.109)	0.577 (0.226 - 1.197)
Age-standardized (rate per capita)	0.069 (0.060 - 0.079)	0.064 (0.056 - 0.074)	0.065 (0.056 - 0.075)	0.006 (0.005 - 0.006)	0.005 (0.005 - 0.006)	0.005 (0.005 - 0.006)	0.452 (0.178 - 0.928)	0.421 (0.162 - 0.877)	0.427 (0.164 - 0.890)
Total number of cases (thousands)	822 (718 - 939)	903 (789 - 1,041)	964 (840 - 1,113)	63 (55 - 72)	70 (61 - 80)	71 (62 - 82)	5 (2 - 11)	6 (2 - 12)	6 (2 - 13)
**Iceland**									
15 - 49 years (rate per capita)	0.078 (0.052 - 0.124)	0.086 (0.057 - 0.135)	0.083 (0.055 - 0.132)	0.009 (0.006 - 0.013)	0.009 (0.006 - 0.013)	0.009 (0.006 - 0.014)	0.521 (0.177 - 1.197)	0.575 (0.187 - 1.319)	0.553 (0.190 - 1.251)
50 - 69 years (rate per capita)	0.223 (0.147 - 0.306)	0.222 (0.148 - 0.300)	0.222 (0.148 - 0.303)	0.014 (0.007 - 0.020)	0.014 (0.007 - 0.020)	0.014 (0.007 - 0.019)	1.454 (0.521 - 3.319)	1.452 (0.519 - 3.236)	1.452 (0.513 - 3.283)
70+ years (rate per capita)	0.165 (0.108 - 0.222)	0.163 (0.107 - 0.219)	0.161 (0.107 - 0.216)	0.009 (0.004 - 0.017)	0.009 (0.004 - 0.016)	0.009 (0.004 - 0.015)	1.029 (0.371 - 2.213)	1.015 (0.359 - 2.162)	1.005 (0.356 - 2.129)
All ages (rate per capita)	0.087 (0.063 - 0.123)	0.099 (0.071 - 0.137)	0.106 (0.075 - 0.147)	0.007 (0.005 - 0.010)	0.008 (0.006 - 0.011)	0.008 (0.006 - 0.011)	0.574 (0.204 - 1.248)	0.651 (0.233 - 1.405)	0.695 (0.248 - 1.550)
Age-standardized (rate per capita)	0.090 (0.064 - 0.127)	0.089 (0.064 - 0.123)	0.089 (0.063 - 0.126)	0.007 (0.005 - 0.010)	0.007 (0.005 - 0.010)	0.007 (0.005 - 0.010)	0.592 (0.212 - 1.290)	0.586 (0.210 - 1.265)	0.588 (0.207 - 1.301)
Total number of cases (thousands)	22 (16 - 31)	29 (21 - 41)	35 (25 - 48)	2 (1 - 3)	2 (2 - 3)	3 (2 - 4)	0 (0 - 0)	0 (0 - 0)	0 (0 - 1)
**Ireland**									
15 - 49 years (rate per capita)	0.047 (0.038 - 0.056)	0.049 (0.040 - 0.059)	0.054 (0.044 - 0.065)	0.005 (0.004 - 0.006)	0.005 (0.004 - 0.007)	0.006 (0.005 - 0.007)	0.310 (0.116 - 0.653)	0.326 (0.123 - 0.686)	0.359 (0.136 - 0.759)
50 - 69 years (rate per capita)	0.122 (0.100 - 0.148)	0.120 (0.099 - 0.145)	0.122 (0.099 - 0.147)	0.008 (0.006 - 0.010)	0.008 (0.006 - 0.010)	0.008 (0.006 - 0.010)	0.797 (0.310 - 1.636)	0.784 (0.300 - 1.607)	0.795 (0.309 - 1.630)
70+ years (rate per capita)	0.083 (0.070 - 0.098)	0.081 (0.069 - 0.096)	0.082 (0.070 - 0.097)	0.006 (0.004 - 0.007)	0.005 (0.004 - 0.007)	0.005 (0.004 - 0.007)	0.522 (0.205 - 1.071)	0.506 (0.201 - 1.042)	0.515 (0.202 - 1.039)
All ages (rate per capita)	0.049 (0.042 - 0.058)	0.055 (0.047 - 0.064)	0.059 (0.050 - 0.069)	0.004 (0.004 - 0.005)	0.005 (0.004 - 0.006)	0.005 (0.004 - 0.006)	0.321 (0.123 - 0.670)	0.358 (0.136 - 0.742)	0.385 (0.147 - 0.804)
Age-standardized (rate per capita)	0.051 (0.044 - 0.060)	0.050 (0.043 - 0.059)	0.051 (0.043 - 0.059)	0.004 (0.004 - 0.005)	0.004 (0.004 - 0.005)	0.004 (0.004 - 0.005)	0.335 (0.128 - 0.704)	0.327 (0.124 - 0.677)	0.332 (0.127 - 0.695)
Total number of cases (thousands)	173 (148 - 203)	228 (196 - 269)	281 (241 - 330)	15 (13 - 18)	20 (17 - 23)	24 (21 - 28)	1 (0 - 2)	1 (1 - 3)	2 (1 - 4)
**Israel**									
15 - 49 years (rate per capita)	0.074 (0.049 - 0.115)	0.076 (0.051 - 0.121)	0.080 (0.053 - 0.129)	0.008 (0.005 - 0.013)	0.008 (0.006 - 0.013)	0.009 (0.006 - 0.013)	0.492 (0.166 - 1.091)	0.508 (0.170 - 1.160)	0.536 (0.183 - 1.200)
50 - 69 years (rate per capita)	0.221 (0.151 - 0.299)	0.221 (0.147 - 0.304)	0.221 (0.148 - 0.300)	0.013 (0.007 - 0.020)	0.014 (0.007 - 0.020)	0.013 (0.007 - 0.020)	1.443 (0.517 - 3.252)	1.441 (0.512 - 3.256)	1.439 (0.516 - 3.215)
70+ years (rate per capita)	0.164 (0.108 - 0.219)	0.161 (0.107 - 0.214)	0.161 (0.105 - 0.210)	0.009 (0.004 - 0.017)	0.009 (0.004 - 0.017)	0.009 (0.004 - 0.015)	1.022 (0.378 - 2.200)	1.006 (0.367 - 2.181)	0.996 (0.361 - 2.177)
All ages (rate per capita)	0.076 (0.055 - 0.105)	0.085 (0.061 - 0.119)	0.089 (0.063 - 0.123)	0.006 (0.005 - 0.009)	0.007 (0.005 - 0.009)	0.007 (0.005 - 0.010)	0.500 (0.177 - 1.104)	0.557 (0.196 - 1.239)	0.582 (0.208 - 1.283)
Age-standardized (rate per capita)	0.089 (0.064 - 0.123)	0.089 (0.064 - 0.124)	0.089 (0.063 - 0.124)	0.007 (0.005 - 0.010)	0.007 (0.005 - 0.010)	0.007 (0.005 - 0.010)	0.585 (0.208 - 1.297)	0.581 (0.205 - 1.296)	0.585 (0.208 - 1.293)
Total number of cases (thousands)	343 (246 - 472)	561 (403 - 784)	717 (510 - 987)	29 (20 - 40)	46 (32 - 62)	58 (40 - 78)	2 (1 - 5)	4 (1 - 8)	5 (2 - 10)
**Italy**									
15 - 49 years (rate per capita)	0.066 (0.058 - 0.073)	0.073 (0.060 - 0.087)	0.078 (0.065 - 0.094)	0.007 (0.006 - 0.008)	0.007 (0.006 - 0.009)	0.008 (0.006 - 0.009)	0.440 (0.174 - 0.916)	0.485 (0.183 - 1.013)	0.521 (0.197 - 1.085)
50 - 69 years (rate per capita)	0.184 (0.157 - 0.209)	0.174 (0.147 - 0.207)	0.174 (0.146 - 0.206)	0.010 (0.007 - 0.012)	0.009 (0.007 - 0.012)	0.009 (0.007 - 0.012)	1.200 (0.483 - 2.454)	1.139 (0.448 - 2.316)	1.140 (0.444 - 2.330)
70+ years (rate per capita)	0.144 (0.126 - 0.165)	0.140 (0.121 - 0.162)	0.139 (0.120 - 0.161)	0.006 (0.004 - 0.007)	0.006 (0.004 - 0.007)	0.006 (0.004 - 0.007)	0.899 (0.353 - 1.846)	0.876 (0.344 - 1.771)	0.867 (0.342 - 1.773)
All ages (rate per capita)	0.090 (0.081 - 0.099)	0.096 (0.084 - 0.111)	0.101 (0.089 - 0.117)	0.006 (0.006 - 0.007)	0.007 (0.006 - 0.008)	0.007 (0.006 - 0.008)	0.589 (0.235 - 1.198)	0.628 (0.246 - 1.306)	0.660 (0.258 - 1.383)
Age-standardized (rate per capita)	0.074 (0.066 - 0.081)	0.071 (0.062 - 0.082)	0.071 (0.062 - 0.082)	0.006 (0.005 - 0.006)	0.005 (0.005 - 0.006)	0.005 (0.005 - 0.006)	0.487 (0.195 - 0.985)	0.465 (0.180 - 0.970)	0.466 (0.180 - 0.974)
Total number of cases (thousands)	5,140 (4,583 - 5,635)	5,644 (4,948 - 6,481)	6,366 (5,562 - 7,317)	359 (318 - 397)	383 (334 - 441)	419 (364 - 481)	34 (13 - 68)	37 (14 - 76)	41 (16 - 87)
**Luxembourg**									
15 - 49 years (rate per capita)	0.085 (0.057 - 0.137)	0.094 (0.060 - 0.150)	0.094 (0.061 - 0.148)	0.009 (0.006 - 0.014)	0.010 (0.006 - 0.015)	0.010 (0.006 - 0.015)	0.569 (0.192 - 1.316)	0.625 (0.211 - 1.431)	0.627 (0.204 - 1.436)
50 - 69 years (rate per capita)	0.217 (0.145 - 0.297)	0.217 (0.146 - 0.301)	0.220 (0.144 - 0.302)	0.014 (0.007 - 0.020)	0.013 (0.007 - 0.020)	0.014 (0.007 - 0.020)	1.410 (0.502 - 3.100)	1.412 (0.502 - 3.232)	1.435 (0.512 - 3.207)
70+ years (rate per capita)	0.151 (0.099 - 0.202)	0.152 (0.098 - 0.206)	0.153 (0.102 - 0.201)	0.009 (0.004 - 0.016)	0.009 (0.004 - 0.017)	0.009 (0.004 - 0.017)	0.935 (0.337 - 2.048)	0.947 (0.339 - 2.042)	0.946 (0.342 - 1.999)
All ages (rate per capita)	0.105 (0.076 - 0.144)	0.108 (0.077 - 0.153)	0.112 (0.079 - 0.155)	0.009 (0.006 - 0.012)	0.009 (0.006 - 0.012)	0.009 (0.006 - 0.012)	0.688 (0.245 - 1.505)	0.709 (0.250 - 1.573)	0.734 (0.260 - 1.601)
Age-standardized (rate per capita)	0.088 (0.063 - 0.121)	0.087 (0.062 - 0.123)	0.088 (0.063 - 0.122)	0.007 (0.005 - 0.010)	0.007 (0.005 - 0.010)	0.007 (0.005 - 0.010)	0.574 (0.204 - 1.262)	0.572 (0.203 - 1.265)	0.579 (0.208 - 1.263)
Total number of cases (thousands)	40 (29 - 55)	49 (35 - 70)	62 (44 - 86)	3 (2 - 4)	4 (3 - 5)	5 (3 - 7)	0 (0 - 1)	0 (0 - 1)	0 (0 - 1)
**Malta**									
15 - 49 years (rate per capita)	0.082 (0.054 - 0.130)	0.087 (0.057 - 0.139)	0.088 (0.058 - 0.137)	0.009 (0.006 - 0.014)	0.009 (0.006 - 0.014)	0.009 (0.006 - 0.014)	0.547 (0.185 - 1.215)	0.583 (0.195 - 1.329)	0.586 (0.195 - 1.333)
50 - 69 years (rate per capita)	0.221 (0.146 - 0.303)	0.218 (0.146 - 0.298)	0.221 (0.147 - 0.302)	0.014 (0.007 - 0.020)	0.014 (0.007 - 0.020)	0.013 (0.007 - 0.020)	1.439 (0.512 - 3.249)	1.426 (0.503 - 3.220)	1.444 (0.516 - 3.248)
70+ years (rate per capita)	0.165 (0.107 - 0.225)	0.163 (0.109 - 0.222)	0.165 (0.109 - 0.226)	0.009 (0.004 - 0.017)	0.009 (0.004 - 0.017)	0.009 (0.004 - 0.017)	1.030 (0.370 - 2.170)	1.022 (0.368 - 2.155)	1.034 (0.365 - 2.206)
All ages (rate per capita)	0.092 (0.065 - 0.130)	0.110 (0.078 - 0.152)	0.120 (0.086 - 0.165)	0.008 (0.005 - 0.011)	0.009 (0.006 - 0.012)	0.009 (0.006 - 0.013)	0.605 (0.214 - 1.314)	0.717 (0.256 - 1.552)	0.786 (0.286 - 1.725)
Age-standardized (rate per capita)	0.089 (0.063 - 0.125)	0.088 (0.063 - 0.123)	0.090 (0.064 - 0.125)	0.007 (0.005 - 0.010)	0.007 (0.005 - 0.010)	0.007 (0.005 - 0.010)	0.584 (0.208 - 1.259)	0.580 (0.207 - 1.255)	0.588 (0.211 - 1.296)
Total number of cases (thousands)	33 (23 - 46)	43 (31 - 60)	50 (36 - 69)	3 (2 - 4)	3 (2 - 5)	4 (3 - 5)	0 (0 - 0)	0 (0 - 1)	0 (0 - 1)
**Netherlands**									
15 - 49 years (rate per capita)	0.082 (0.055 - 0.130)	0.092 (0.061 - 0.146)	0.092 (0.060 - 0.143)	0.009 (0.006 - 0.014)	0.010 (0.006 - 0.015)	0.010 (0.006 - 0.014)	0.548 (0.185 - 1.235)	0.614 (0.204 - 1.411)	0.615 (0.203 - 1.417)
50 - 69 years (rate per capita)	0.206 (0.139 - 0.282)	0.208 (0.138 - 0.285)	0.208 (0.136 - 0.283)	0.014 (0.007 - 0.020)	0.014 (0.007 - 0.020)	0.014 (0.007 - 0.020)	1.345 (0.485 - 3.021)	1.356 (0.482 - 3.030)	1.356 (0.487 - 3.051)
70+ years (rate per capita)	0.134 (0.089 - 0.178)	0.135 (0.090 - 0.182)	0.136 (0.089 - 0.180)	0.009 (0.005 - 0.017)	0.009 (0.005 - 0.018)	0.009 (0.004 - 0.017)	0.836 (0.302 - 1.818)	0.839 (0.302 - 1.788)	0.844 (0.305 - 1.811)
All ages (rate per capita)	0.095 (0.068 - 0.132)	0.106 (0.076 - 0.145)	0.112 (0.080 - 0.154)	0.008 (0.006 - 0.011)	0.009 (0.006 - 0.012)	0.009 (0.006 - 0.012)	0.624 (0.221 - 1.369)	0.696 (0.248 - 1.531)	0.735 (0.262 - 1.626)
Age-standardized (rate per capita)	0.085 (0.060 - 0.117)	0.084 (0.061 - 0.116)	0.085 (0.060 - 0.118)	0.007 (0.005 - 0.010)	0.007 (0.005 - 0.010)	0.007 (0.005 - 0.010)	0.555 (0.196 - 1.225)	0.554 (0.195 - 1.225)	0.557 (0.196 - 1.258)
Total number of cases (thousands)	1,422 (1,020 - 1,972)	1,733 (1,237 - 2,364)	1,933 (1,368 - 2,640)	124 (87 - 170)	144 (99 - 196)	155 (103 - 213)	9 (3 - 20)	11 (4 - 25)	13 (5 - 28)
**Norway**									
15 - 49 years (rate per capita)	0.085 (0.070 - 0.104)	0.090 (0.074 - 0.110)	0.090 (0.074 - 0.110)	0.009 (0.007 - 0.011)	0.009 (0.007 - 0.011)	0.009 (0.007 - 0.011)	0.567 (0.213 - 1.182)	0.600 (0.225 - 1.233)	0.601 (0.223 - 1.248)
50 - 69 years (rate per capita)	0.203 (0.168 - 0.243)	0.207 (0.170 - 0.249)	0.206 (0.171 - 0.245)	0.011 (0.008 - 0.014)	0.012 (0.008 - 0.015)	0.012 (0.009 - 0.014)	1.322 (0.508 - 2.706)	1.348 (0.527 - 2.748)	1.345 (0.514 - 2.746)
70+ years (rate per capita)	0.135 (0.116 - 0.158)	0.134 (0.114 - 0.157)	0.134 (0.114 - 0.158)	0.007 (0.005 - 0.009)	0.006 (0.005 - 0.008)	0.006 (0.005 - 0.008)	0.844 (0.339 - 1.730)	0.835 (0.334 - 1.707)	0.836 (0.331 - 1.714)
All ages (rate per capita)	0.097 (0.083 - 0.113)	0.103 (0.088 - 0.121)	0.107 (0.091 - 0.127)	0.007 (0.006 - 0.008)	0.008 (0.007 - 0.009)	0.008 (0.007 - 0.009)	0.633 (0.246 - 1.322)	0.671 (0.259 - 1.382)	0.700 (0.269 - 1.434)

	Prevalence 1990	Prevalence 2005	Prevalence 2015	Incidence 1990	Incidence 2005	Incidence 2015	YLD rate 1990	YLD rate 2005	YLD rate 2015
**Age-standardized (rate per capita)**	0.086 (0.073 - 0.101)	0.085 (0.073 - 0.100)	0.086 (0.073 - 0.101)	0.007 (0.006 - 0.008)	0.007 (0.006 - 0.008)	0.007 (0.006 - 0.008)	0.564 (0.220 - 1.173)	0.559 (0.216 - 1.151)	0.564 (0.216 - 1.162)
**Total number of cases (thousands)**	410 (351 - 479)	475 (406 - 557)	553 (472 - 654)	31 (27 - 36)	35 (30 - 41)	40 (34 - 47)	3 (1 - 6)	3 (1 - 6)	4 (1 - 7)
**Portugal**									
**15 - 49 years (rate per capita)**	0.079 (0.052 - 0.125)	0.089 (0.058 - 0.138)	0.096 (0.063 - 0.153)	0.009 (0.005 - 0.013)	0.009 (0.006 - 0.014)	0.010 (0.006 - 0.015)	0.523 (0.174 - 1.177)	0.591 (0.199 - 1.356)	0.642 (0.212 - 1.473)
**50 - 69 years (rate per capita)**	0.225 (0.149 - 0.309)	0.226 (0.155 - 0.303)	0.226 (0.147 - 0.309)	0.013 (0.007 - 0.020)	0.013 (0.007 - 0.020)	0.013 (0.007 - 0.020)	1.461 (0.538 - 3.284)	1.468 (0.529 - 3.286)	1.474 (0.531 - 3.307)
**70+ years (rate per capita)**	0.169 (0.110 - 0.228)	0.170 (0.116 - 0.225)	0.169 (0.110 - 0.224)	0.009 (0.004 - 0.016)	0.009 (0.004 - 0.017)	0.009 (0.004 - 0.016)	1.052 (0.382 - 2.261)	1.061 (0.376 - 2.269)	1.054 (0.384 - 2.261)
**All ages (rate per capita)**	0.102 (0.073 - 0.139)	0.116 (0.083 - 0.156)	0.124 (0.088 - 0.170)	0.008 (0.005 - 0.011)	0.009 (0.006 - 0.012)	0.009 (0.006 - 0.013)	0.662 (0.238 - 1.427)	0.754 (0.267 - 1.624)	0.811 (0.290 - 1.753)
**Age-standardized (rate per capita)**	0.090 (0.064 - 0.126)	0.090 (0.065 - 0.124)	0.091 (0.064 - 0.126)	0.007 (0.005 - 0.010)	0.007 (0.005 - 0.010)	0.007 (0.005 - 0.010)	0.588 (0.210 - 1.296)	0.592 (0.212 - 1.306)	0.595 (0.209 - 1.307)
**Total number of cases (thousands)**	1,011 (723 - 1,387)	1,220 (875 - 1,649)	1,344 (946 - 1,836)	78 (53 - 107)	93 (64 - 126)	99 (67 - 135)	7 (2 - 14)	8 (3 - 17)	9 (3 - 19)
**Spain**									
**15 - 49 years (rate per capita)**	0.026 (0.022 - 0.031)	0.030 (0.025 - 0.035)	0.032 (0.027 - 0.038)	0.003 (0.002 - 0.003)	0.003 (0.003 - 0.004)	0.003 (0.003 - 0.004)	0.173 (0.066 - 0.366)	0.198 (0.074 - 0.416)	0.217 (0.081 - 0.452)
**50 - 69 years (rate per capita)**	0.072 (0.060 - 0.085)	0.070 (0.059 - 0.083)	0.071 (0.059 - 0.084)	0.005 (0.004 - 0.006)	0.005 (0.004 - 0.006)	0.005 (0.004 - 0.006)	0.470 (0.185 - 0.963)	0.461 (0.178 - 0.951)	0.467 (0.180 - 0.960)
**70+ years (rate per capita)**	0.059 (0.051 - 0.069)	0.057 (0.049 - 0.066)	0.059 (0.051 - 0.069)	0.004 (0.003 - 0.005)	0.004 (0.003 - 0.005)	0.004 (0.003 - 0.005)	0.370 (0.149 - 0.754)	0.356 (0.142 - 0.720)	0.368 (0.145 - 0.751)
**All ages (rate per capita)**	0.033 (0.029 - 0.038)	0.037 (0.032 - 0.043)	0.040 (0.035 - 0.046)	0.003 (0.002 - 0.003)	0.003 (0.003 - 0.004)	0.003 (0.003 - 0.004)	0.218 (0.086 - 0.447)	0.244 (0.095 - 0.510)	0.263 (0.102 - 0.548)
**Age-standardized (rate per capita)**	0.030 (0.026 - 0.034)	0.029 (0.026 - 0.034)	0.030 (0.026 - 0.034)	0.003 (0.002 - 0.003)	0.003 (0.002 - 0.003)	0.003 (0.002 - 0.003)	0.195 (0.077 - 0.398)	0.193 (0.074 - 0.403)	0.195 (0.076 - 0.406)
**Total number of cases (thousands)**	1,299 (1,130 - 1,493)	1,614 (1,405 - 1,869)	1,956 (1,698 - 2,266)	109 (95 - 125)	133 (117 - 153)	160 (138 - 184)	8 (3 - 17)	11 (4 - 22)	13 (5 - 27)
**Sweden**									
**15 - 49 years (rate per capita)**	0.090 (0.074 - 0.108)	0.090 (0.074 - 0.109)	0.090 (0.075 - 0.108)	0.010 (0.008 - 0.012)	0.010 (0.008 - 0.012)	0.010 (0.008 - 0.012)	0.597 (0.225 - 1.252)	0.601 (0.227 - 1.275)	0.602 (0.228 - 1.271)
**50 - 69 years (rate per capita)**	0.245 (0.206 - 0.289)	0.245 (0.206 - 0.289)	0.244 (0.206 - 0.287)	0.014 (0.011 - 0.017)	0.014 (0.011 - 0.018)	0.014 (0.010 - 0.017)	1.598 (0.631 - 3.229)	1.604 (0.626 - 3.268)	1.591 (0.626 - 3.248)
**70+ years (rate per capita)**	0.206 (0.177 - 0.239)	0.204 (0.175 - 0.237)	0.204 (0.175 - 0.238)	0.009 (0.007 - 0.012)	0.009 (0.007 - 0.012)	0.009 (0.007 - 0.012)	1.294 (0.517 - 2.616)	1.272 (0.504 - 2.571)	1.272 (0.505 - 2.594)
**All ages (rate per capita)**	0.120 (0.105 - 0.138)	0.127 (0.110 - 0.146)	0.128 (0.112 - 0.147)	0.009 (0.008 - 0.010)	0.009 (0.008 - 0.010)	0.009 (0.008 - 0.010)	0.784 (0.308 - 1.608)	0.825 (0.321 - 1.722)	0.833 (0.326 - 1.707)
**Age-standardized (rate per capita)**	0.097 (0.084 - 0.112)	0.097 (0.084 - 0.112)	0.096 (0.084 - 0.112)	0.008 (0.007 - 0.009)	0.008 (0.007 - 0.009)	0.008 (0.007 - 0.009)	0.635 (0.249 - 1.316)	0.634 (0.245 - 1.336)	0.633 (0.247 - 1.307)
**Total number of cases (thousands)**	1,027 (898 - 1,183)	1,142 (995 - 1,319)	1,254 (1,098 - 1,444)	75 (65 - 86)	81 (71 - 94)	88 (77 - 102)	7 (3 - 14)	7 (3 - 16)	8 (3 - 17)
**Switzerland**									
**15 - 49 years (rate per capita)**	0.087 (0.057 - 0.139)	0.094 (0.060 - 0.148)	0.094 (0.061 - 0.151)	0.009 (0.006 - 0.014)	0.010 (0.006 - 0.015)	0.010 (0.006 - 0.015)	0.578 (0.196 - 1.320)	0.626 (0.208 - 1.414)	0.629 (0.208 - 1.445)
**50 - 69 years (rate per capita)**	0.217 (0.145 - 0.294)	0.217 (0.144 - 0.295)	0.217 (0.145 - 0.297)	0.014 (0.007 - 0.020)	0.014 (0.007 - 0.020)	0.014 (0.007 - 0.019)	1.413 (0.505 - 3.146)	1.417 (0.507 - 3.163)	1.418 (0.504 - 3.217)
**70+ years (rate per capita)**	0.153 (0.103 - 0.203)	0.151 (0.100 - 0.200)	0.151 (0.102 - 0.200)	0.009 (0.004 - 0.017)	0.009 (0.004 - 0.016)	0.009 (0.004 - 0.016)	0.948 (0.345 - 2.031)	0.941 (0.341 - 1.993)	0.945 (0.343 - 2.018)
**All ages (rate per capita)**	0.105 (0.076 - 0.145)	0.114 (0.081 - 0.154)	0.118 (0.084 - 0.163)	0.009 (0.006 - 0.012)	0.009 (0.006 - 0.012)	0.009 (0.006 - 0.012)	0.689 (0.245 - 1.518)	0.743 (0.264 - 1.622)	0.775 (0.276 - 1.694)
**Age-standardized (rate per capita)**	0.088 (0.063 - 0.122)	0.088 (0.063 - 0.120)	0.088 (0.062 - 0.123)	0.007 (0.005 - 0.010)	0.007 (0.005 - 0.010)	0.007 (0.005 - 0.010)	0.576 (0.204 - 1.285)	0.575 (0.205 - 1.270)	0.578 (0.203 - 1.281)
**Total number of cases (thousands)**	708 (508 - 975)	844 (604 - 1,148)	979 (697 - 1,347)	58 (41 - 79)	67 (46 - 90)	76 (51 - 102)	5 (2 - 10)	6 (2 - 12)	6 (2 - 14)
**United Kingdom**									
**15 - 49 years (rate per capita)**	0.056 (0.046 - 0.066)	0.060 (0.050 - 0.071)	0.060 (0.050 - 0.071)	0.006 (0.005 - 0.007)	0.006 (0.005 - 0.008)	0.006 (0.005 - 0.008)	0.370 (0.140 - 0.792)	0.397 (0.150 - 0.838)	0.397 (0.151 - 0.846)
**50 - 69 years (rate per capita)**	0.150 (0.126 - 0.177)	0.150 (0.126 - 0.178)	0.147 (0.124 - 0.174)	0.009 (0.007 - 0.011)	0.009 (0.007 - 0.011)	0.009 (0.007 - 0.011)	0.981 (0.381 - 1.993)	0.983 (0.381 - 1.997)	0.966 (0.377 - 1.958)
**70+ years (rate per capita)**	0.118 (0.102 - 0.137)	0.118 (0.102 - 0.137)	0.116 (0.100 - 0.135)	0.006 (0.004 - 0.007)	0.006 (0.004 - 0.007)	0.006 (0.004 - 0.007)	0.745 (0.293 - 1.519)	0.745 (0.294 - 1.519)	0.733 (0.288 - 1.492)
**All ages (rate per capita)**	0.071 (0.062 - 0.082)	0.076 (0.066 - 0.087)	0.078 (0.068 - 0.089)	0.005 (0.005 - 0.006)	0.006 (0.005 - 0.006)	0.006 (0.005 - 0.007)	0.466 (0.180 - 0.967)	0.496 (0.192 - 1.033)	0.507 (0.198 - 1.061)
**Age-standardized (rate per capita)**	0.061 (0.053 - 0.070)	0.061 (0.053 - 0.070)	0.060 (0.052 - 0.069)	0.005 (0.004 - 0.006)	0.005 (0.004 - 0.006)	0.005 (0.004 - 0.005)	0.400 (0.154 - 0.834)	0.399 (0.154 - 0.834)	0.394 (0.152 - 0.824)
**Total number of cases (thousands)**	4,073 (3,558 - 4,686)	4,565 (3,984 - 5,261)	4,980 (4,345 - 5,743)	309 (271 - 355)	339 (295 - 389)	364 (318 - 418)	27 (10 - 55)	30 (12 - 62)	33 (13 - 68)
**England**									
**15 - 49 years (rate per capita)**	0.056 (0.046 - 0.066)	0.059 (0.049 - 0.071)	0.060 (0.050 - 0.071)	0.006 (0.005 - 0.007)	0.006 (0.005 - 0.008)	0.006 (0.005 - 0.008)	0.371 (0.140 - 0.792)	0.396 (0.150 - 0.839)	0.398 (0.151 - 0.844)
**50 - 69 years (rate per capita)**	0.150 (0.126 - 0.177)	0.150 (0.126 - 0.178)	0.147 (0.124 - 0.174)	0.009 (0.007 - 0.011)	0.009 (0.007 - 0.011)	0.009 (0.007 - 0.011)	0.982 (0.381 - 1.992)	0.984 (0.381 - 2.001)	0.967 (0.376 - 1.966)
**70+ years (rate per capita)**	0.118 (0.102 - 0.137)	0.118 (0.102 - 0.137)	0.116 (0.100 - 0.135)	0.006 (0.004 - 0.007)	0.006 (0.004 - 0.007)	0.006 (0.004 - 0.007)	0.745 (0.293 - 1.519)	0.746 (0.295 - 1.521)	0.733 (0.288 - 1.493)
**All ages (rate per capita)**	0.071 (0.062 - 0.082)	0.076 (0.066 - 0.087)	0.077 (0.067 - 0.089)	0.005 (0.005 - 0.006)	0.006 (0.005 - 0.006)	0.006 (0.005 - 0.007)	0.467 (0.181 - 0.970)	0.495 (0.191 - 1.030)	0.505 (0.197 - 1.055)
**Age-standardized (rate per capita)**	0.061 (0.053 - 0.070)	0.061 (0.053 - 0.070)	0.060 (0.052 - 0.069)	0.005 (0.004 - 0.006)	0.005 (0.004 - 0.006)	0.005 (0.004 - 0.005)	0.400 (0.154 - 0.835)	0.399 (0.154 - 0.834)	0.394 (0.152 - 0.824)
**Total number of cases (thousands)**	3,402 (2,980 - 3,910)	3,814 (3,327 - 4,394)	4,164 (3,632 - 4,797)	258 (226 - 296)	283 (247 - 326)	305 (267 - 351)	22 (9 - 46)	25 (10 - 52)	27 (11 - 57)
**Northern Ireland**									
**15 - 49 years (rate per capita)**	0.053 (0.044 - 0.063)	0.058 (0.048 - 0.069)	0.059 (0.049 - 0.071)	0.006 (0.005 - 0.007)	0.006 (0.005 - 0.007)	0.006 (0.005 - 0.008)	0.351 (0.132 - 0.736)	0.383 (0.144 - 0.801)	0.396 (0.149 - 0.835)
**50 - 69 years (rate per capita)**	0.150 (0.126 - 0.177)	0.150 (0.126 - 0.178)	0.148 (0.124 - 0.175)	0.009 (0.007 - 0.011)	0.009 (0.007 - 0.011)	0.009 (0.007 - 0.011)	0.981 (0.378 - 2.011)	0.983 (0.383 - 1.997)	0.967 (0.376 - 1.975)
**70+ years (rate per capita)**	0.119 (0.102 - 0.139)	0.119 (0.102 - 0.139)	0.117 (0.101 - 0.136)	0.006 (0.004 - 0.007)	0.006 (0.004 - 0.007)	0.006 (0.004 - 0.007)	0.750 (0.296 - 1.524)	0.749 (0.293 - 1.525)	0.736 (0.291 - 1.490)
**All ages (rate per capita)**	0.063 (0.055 - 0.073)	0.071 (0.062 - 0.081)	0.075 (0.065 - 0.087)	0.005 (0.004 - 0.006)	0.005 (0.005 - 0.006)	0.006 (0.005 - 0.006)	0.412 (0.159 - 0.855)	0.462 (0.180 - 0.960)	0.491 (0.191 - 1.029)
**Age-standardized (rate per capita)**	0.061 (0.053 - 0.071)	0.061 (0.053 - 0.071)	0.060 (0.052 - 0.070)	0.005 (0.004 - 0.006)	0.005 (0.004 - 0.006)	0.005 (0.004 - 0.005)	0.400 (0.154 - 0.829)	0.399 (0.155 - 0.827)	0.395 (0.153 - 0.826)
**Total number of cases (thousands)**	101 (88 - 116)	122 (106 - 140)	138 (120 - 159)	8 (7 - 9)	9 (8 - 11)	10 (9 - 12)	1 (0 - 1)	1 (0 - 2)	1 (0 - 2)
**Scotland**									
**15 - 49 years (rate per capita)**	0.055 (0.046 - 0.065)	0.061 (0.051 - 0.072)	0.060 (0.050 - 0.072)	0.006 (0.005 - 0.007)	0.006 (0.005 - 0.008)	0.006 (0.005 - 0.008)	0.365 (0.137 - 0.771)	0.405 (0.153 - 0.859)	0.401 (0.151 - 0.843)
**50 - 69 years (rate per capita)**	0.150 (0.125 - 0.177)	0.150 (0.127 - 0.178)	0.148 (0.124 - 0.175)	0.009 (0.007 - 0.011)	0.009 (0.007 - 0.011)	0.009 (0.007 - 0.011)	0.977 (0.378 - 1.975)	0.980 (0.378 - 1.979)	0.964 (0.372 - 1.946)
**70+ years (rate per capita)**	0.118 (0.101 - 0.138)	0.119 (0.103 - 0.138)	0.117 (0.101 - 0.136)	0.006 (0.004 - 0.007)	0.006 (0.004 - 0.007)	0.006 (0.004 - 0.007)	0.743 (0.290 - 1.512)	0.745 (0.295 - 1.516)	0.732 (0.288 - 1.489)
**All ages (rate per capita)**	0.071 (0.062 - 0.082)	0.078 (0.069 - 0.090)	0.081 (0.071 - 0.094)	0.005 (0.005 - 0.006)	0.006 (0.005 - 0.007)	0.006 (0.005 - 0.007)	0.464 (0.180 - 0.955)	0.511 (0.200 - 1.071)	0.528 (0.204 - 1.079)
**Age-standardized (rate per capita)**	0.061 (0.053 - 0.070)	0.061 (0.053 - 0.070)	0.060 (0.052 - 0.069)	0.005 (0.004 - 0.006)	0.005 (0.004 - 0.006)	0.005 (0.004 - 0.005)	0.398 (0.154 - 0.826)	0.398 (0.154 - 0.836)	0.393 (0.151 - 0.814)
**Total number of cases (thousands)**	361 (314 - 416)	398 (348 - 458)	431 (376 - 499)	27 (24 - 31)	29 (25 - 34)	31 (27 - 36)	2 (1 - 5)	3 (1 - 5)	3 (1 - 6)
**Wales**									
**15 - 49 years (rate per capita)**	0.057 (0.047 - 0.067)	0.059 (0.049 - 0.071)	0.059 (0.049 - 0.070)	0.006 (0.005 - 0.007)	0.006 (0.005 - 0.008)	0.006 (0.005 - 0.007)	0.376 (0.143 - 0.790)	0.395 (0.148 - 0.841)	0.390 (0.148 - 0.820)
**50 - 69 years (rate per capita)**	0.150 (0.125 - 0.177)	0.150 (0.126 - 0.178)	0.148 (0.124 - 0.175)	0.009 (0.007 - 0.011)	0.009 (0.007 - 0.011)	0.009 (0.007 - 0.011)	0.979 (0.379 - 1.989)	0.981 (0.381 - 1.987)	0.964 (0.375 - 1.954)
**70+ years (rate per capita)**	0.118 (0.102 - 0.138)	0.118 (0.101 - 0.137)	0.117 (0.101 - 0.136)	0.006 (0.004 - 0.007)	0.006 (0.004 - 0.007)	0.006 (0.004 - 0.007)	0.743 (0.292 - 1.525)	0.741 (0.290 - 1.508)	0.732 (0.290 - 1.495)
**All ages (rate per capita)**	0.073 (0.064 - 0.084)	0.078 (0.068 - 0.090)	0.080 (0.070 - 0.093)	0.005 (0.005 - 0.006)	0.006 (0.005 - 0.006)	0.006 (0.005 - 0.007)	0.478 (0.187 - 0.995)	0.510 (0.198 - 1.064)	0.523 (0.205 - 1.096)
**Age-standardized (rate per capita)**	0.061 (0.053 - 0.070)	0.061 (0.053 - 0.070)	0.060 (0.052 - 0.069)	0.005 (0.004 - 0.006)	0.005 (0.004 - 0.006)	0.005 (0.004 - 0.005)	0.399 (0.156 - 0.832)	0.398 (0.154 - 0.835)	0.393 (0.153 - 0.821)
**Total number of cases (thousands)**	209 (182 - 242)	232 (201 - 266)	247 (214 - 285)	16 (14 - 18)	17 (15 - 19)	18 (15 - 20)	1 (1 - 3)	2 (1 - 3)	2 (1 - 3)
**Southern Latin America**									
**15 - 49 years (rate per capita)**	0.072 (0.061 - 0.086)	0.077 (0.065 - 0.091)	0.077 (0.064 - 0.091)	0.008 (0.007 - 0.009)	0.008 (0.007 - 0.010)	0.008 (0.007 - 0.010)	0.483 (0.184 - 1.019)	0.514 (0.196 - 1.088)	0.513 (0.194 - 1.071)
**50 - 69 years (rate per capita)**	0.201 (0.170 - 0.236)	0.206 (0.174 - 0.240)	0.205 (0.173 - 0.240)	0.011 (0.008 - 0.014)	0.011 (0.008 - 0.014)	0.011 (0.008 - 0.014)	1.314 (0.511 - 2.696)	1.347 (0.526 - 2.752)	1.341 (0.517 - 2.713)
**70+ years (rate per capita)**	0.160 (0.137 - 0.185)	0.159 (0.137 - 0.184)	0.157 (0.136 - 0.181)	0.007 (0.005 - 0.008)	0.006 (0.005 - 0.008)	0.006 (0.005 - 0.008)	1.008 (0.399 - 2.061)	1.002 (0.404 - 2.036)	0.989 (0.395 - 2.052)
**All ages (rate per capita)**	0.075 (0.066 - 0.086)	0.084 (0.073 - 0.096)	0.088 (0.077 - 0.102)	0.006 (0.005 - 0.007)	0.006 (0.006 - 0.007)	0.007 (0.006 - 0.007)	0.495 (0.192 - 1.027)	0.551 (0.214 - 1.145)	0.580 (0.225 - 1.204)
**Age-standardized (rate per capita)**	0.083 (0.073 - 0.096)	0.086 (0.075 - 0.098)	0.084 (0.073 - 0.097)	0.006 (0.006 - 0.007)	0.006 (0.006 - 0.007)	0.006 (0.006 - 0.007)	0.548 (0.213 - 1.136)	0.563 (0.218 - 1.169)	0.553 (0.214 - 1.149)
**Total number of cases (thousands)**	3,684 (3,213 - 4,224)	4,908 (4,300 - 5,612)	5,719 (4,997 - 6,586)	286 (251 - 329)	372 (325 - 424)	424 (372 - 485)	24 (9 - 50)	32 (13 - 67)	38 (15 - 78)
**Argentina**									
**15 - 49 years (rate per capita)**	0.064 (0.054 - 0.076)	0.067 (0.056 - 0.079)	0.066 (0.055 - 0.079)	0.007 (0.006 - 0.008)	0.007 (0.006 - 0.008)	0.007 (0.006 - 0.008)	0.430 (0.162 - 0.900)	0.444 (0.170 - 0.933)	0.443 (0.168 - 0.927)
**50 - 69 years (rate per capita)**	0.182 (0.154 - 0.215)	0.185 (0.155 - 0.217)	0.181 (0.153 - 0.213)	0.010 (0.008 - 0.013)	0.010 (0.008 - 0.013)	0.010 (0.008 - 0.013)	1.192 (0.461 - 2.450)	1.210 (0.469 - 2.468)	1.186 (0.459 - 2.394)

	Prevalence 1990	Prevalence 2005	Prevalence 2015	Incidence 1990	Incidence 2005	Incidence 2015	YLD rate 1990	YLD rate 2005	YLD rate 2015
**70+ years (rate per capita)**	0.147 (0.127 - 0.171)	0.146 (0.126 - 0.169)	0.143 (0.123 - 0.165)	0.006 (0.005 - 0.008)	0.006 (0.005 - 0.008)	0.006 (0.005 - 0.008)	0.932 (0.369 - 1.883)	0.922 (0.367 - 1.873)	0.899 (0.359 - 1.846)
**All ages (rate per capita)**	0.068 (0.060 - 0.079)	0.074 (0.064 - 0.084)	0.075 (0.065 - 0.087)	0.005 (0.005 - 0.006)	0.006 (0.005 - 0.006)	0.006 (0.005 - 0.007)	0.450 (0.175 - 0.934)	0.483 (0.188 - 1.002)	0.495 (0.189 - 1.023)
**Age-standardized (rate per capita)**	0.075 (0.065 - 0.086)	0.077 (0.067 - 0.088)	0.074 (0.065 - 0.086)	0.006 (0.005 - 0.007)	0.006 (0.005 - 0.007)	0.006 (0.005 - 0.007)	0.491 (0.191 - 1.018)	0.504 (0.195 - 1.043)	0.490 (0.188 - 1.015)
**Total number of cases (thousands)**	2,240 (1,949 - 2,577)	2,878 (2,511 - 3,290)	3,268 (2,843 - 3,765)	173 (152 - 198)	221 (194 - 252)	250 (219 - 285)	15 (6 - 31)	19 (7 - 39)	21 (8 - 44)
**Chile**									
**15 - 49 years (rate per capita)**	0.089 (0.074 - 0.105)	0.100 (0.084 - 0.117)	0.100 (0.084 - 0.119)	0.009 (0.008 - 0.011)	0.010 (0.008 - 0.012)	0.010 (0.008 - 0.012)	0.592 (0.227 - 1.249)	0.666 (0.253 - 1.419)	0.669 (0.250 - 1.392)
**50 - 69 years (rate per capita)**	0.255 (0.216 - 0.301)	0.254 (0.217 - 0.300)	0.251 (0.213 - 0.295)	0.013 (0.010 - 0.016)	0.013 (0.010 - 0.016)	0.013 (0.009 - 0.016)	1.670 (0.655 - 3.421)	1.665 (0.638 - 3.368)	1.646 (0.636 - 3.340)
**70+ years (rate per capita)**	0.197 (0.169 - 0.227)	0.194 (0.168 - 0.223)	0.190 (0.165 - 0.220)	0.007 (0.005 - 0.009)	0.007 (0.005 - 0.009)	0.007 (0.005 - 0.009)	1.242 (0.503 - 2.549)	1.221 (0.486 - 2.521)	1.197 (0.475 - 2.488)
**All ages (rate per capita)**	0.088 (0.077 - 0.101)	0.106 (0.093 - 0.123)	0.118 (0.103 - 0.136)	0.007 (0.006 - 0.008)	0.008 (0.007 - 0.009)	0.008 (0.007 - 0.010)	0.582 (0.225 - 1.198)	0.702 (0.273 - 1.484)	0.774 (0.296 - 1.583)
**Age-standardized (rate per capita)**	0.106 (0.092 - 0.122)	0.105 (0.093 - 0.121)	0.104 (0.091 - 0.120)	0.008 (0.007 - 0.009)	0.008 (0.007 - 0.009)	0.008 (0.007 - 0.009)	0.696 (0.270 - 1.432)	0.693 (0.271 - 1.468)	0.684 (0.262 - 1.401)
**Total number of cases (thousands)**	1,160 (1,010 - 1,334)	1,715 (1,503 - 1,976)	2,111 (1,842 - 2,438)	93 (81 - 106)	128 (111 - 147)	150 (130 - 171)	8 (3 - 16)	11 (4 - 24)	14 (5 - 28)
**Uruguay**									
**15 - 49 years (rate per capita)**	0.078 (0.065 - 0.093)	0.081 (0.068 - 0.097)	0.082 (0.068 - 0.098)	0.008 (0.007 - 0.010)	0.008 (0.007 - 0.010)	0.008 (0.007 - 0.010)	0.519 (0.197 - 1.105)	0.543 (0.206 - 1.140)	0.545 (0.205 - 1.148)
**50 - 69 years (rate per capita)**	0.215 (0.182 - 0.253)	0.215 (0.182 - 0.253)	0.214 (0.179 - 0.251)	0.012 (0.008 - 0.014)	0.012 (0.009 - 0.014)	0.012 (0.008 - 0.014)	1.407 (0.549 - 2.855)	1.405 (0.549 - 2.869)	1.399 (0.545 - 2.841)
**70+ years (rate per capita)**	0.169 (0.146 - 0.196)	0.167 (0.144 - 0.192)	0.163 (0.140 - 0.188)	0.007 (0.005 - 0.009)	0.007 (0.005 - 0.009)	0.006 (0.005 - 0.008)	1.066 (0.421 - 2.187)	1.051 (0.411 - 2.164)	1.022 (0.408 - 2.114)
**All ages (rate per capita)**	0.091 (0.080 - 0.105)	0.095 (0.083 - 0.109)	0.099 (0.086 - 0.114)	0.007 (0.006 - 0.007)	0.007 (0.006 - 0.008)	0.007 (0.006 - 0.008)	0.596 (0.231 - 1.233)	0.620 (0.240 - 1.291)	0.649 (0.252 - 1.341)
**Age-standardized (rate per capita)**	0.088 (0.077 - 0.102)	0.088 (0.077 - 0.102)	0.088 (0.076 - 0.102)	0.007 (0.006 - 0.008)	0.007 (0.006 - 0.008)	0.007 (0.006 - 0.008)	0.581 (0.225 - 1.203)	0.582 (0.225 - 1.208)	0.579 (0.224 - 1.195)
**Total number of cases (thousands)**	283 (249 - 326)	315 (276 - 362)	340 (296 - 390)	20 (18 - 23)	23 (20 - 26)	24 (21 - 28)	2 (1 - 4)	2 (1 - 4)	2 (1 - 5)
**Central Europe, Eastern Europe, and Central Asia**									
**15 - 49 years (rate per capita)**	0.048 (0.040 - 0.058)	0.050 (0.042 - 0.060)	0.051 (0.043 - 0.061)	0.005 (0.004 - 0.006)	0.005 (0.004 - 0.006)	0.005 (0.005 - 0.007)	0.321 (0.123 - 0.680)	0.335 (0.127 - 0.711)	0.343 (0.130 - 0.716)
**50 - 69 years (rate per capita)**	0.129 (0.109 - 0.154)	0.130 (0.110 - 0.154)	0.131 (0.110 - 0.156)	0.008 (0.006 - 0.010)	0.008 (0.006 - 0.010)	0.009 (0.006 - 0.010)	0.839 (0.329 - 1.699)	0.844 (0.327 - 1.717)	0.852 (0.331 - 1.738)
**70+ years (rate per capita)**	0.108 (0.093 - 0.127)	0.111 (0.095 - 0.130)	0.109 (0.093 - 0.128)	0.006 (0.005 - 0.008)	0.006 (0.005 - 0.008)	0.006 (0.005 - 0.008)	0.672 (0.267 - 1.365)	0.690 (0.271 - 1.411)	0.677 (0.269 - 1.392)
**All ages (rate per capita)**	0.056 (0.049 - 0.065)	0.062 (0.054 - 0.072)	0.067 (0.058 - 0.077)	0.005 (0.004 - 0.005)	0.005 (0.004 - 0.006)	0.005 (0.005 - 0.006)	0.366 (0.143 - 0.752)	0.406 (0.158 - 0.836)	0.437 (0.171 - 0.902)
**Age-standardized (rate per capita)**	0.054 (0.047 - 0.063)	0.054 (0.047 - 0.063)	0.054 (0.048 - 0.063)	0.004 (0.004 - 0.005)	0.004 (0.004 - 0.005)	0.004 (0.004 - 0.005)	0.353 (0.137 - 0.727)	0.355 (0.138 - 0.729)	0.356 (0.138 - 0.734)
**Total number of cases (thousands)**	23,178 (20,157 - 26,778)	25,299 (21,965 - 29,198)	28,095 (24,470 - 32,366)	1,909 (1,661 - 2,182)	2,045 (1,783 - 2,345)	2,224 (1,938 - 2,538)	151 (59 - 311)	165 (64 - 339)	183 (72 - 378)
**Eastern Europe**									
**15 - 49 years (rate per capita)**	0.042 (0.035 - 0.051)	0.045 (0.037 - 0.054)	0.045 (0.038 - 0.054)	0.005 (0.004 - 0.006)	0.005 (0.004 - 0.006)	0.005 (0.004 - 0.006)	0.282 (0.107 - 0.599)	0.298 (0.113 - 0.633)	0.302 (0.114 - 0.633)
**50 - 69 years (rate per capita)**	0.114 (0.096 - 0.135)	0.114 (0.096 - 0.135)	0.114 (0.095 - 0.137)	0.007 (0.006 - 0.009)	0.007 (0.006 - 0.009)	0.008 (0.006 - 0.009)	0.735 (0.286 - 1.488)	0.734 (0.284 - 1.495)	0.743 (0.289 - 1.511)
**70+ years (rate per capita)**	0.095 (0.082 - 0.112)	0.097 (0.083 - 0.114)	0.097 (0.083 - 0.114)	0.005 (0.004 - 0.007)	0.006 (0.004 - 0.007)	0.006 (0.004 - 0.007)	0.591 (0.233 - 1.191)	0.601 (0.238 - 1.216)	0.603 (0.238 - 1.233)
**All ages (rate per capita)**	0.052 (0.046 - 0.060)	0.058 (0.050 - 0.067)	0.062 (0.054 - 0.072)	0.004 (0.004 - 0.005)	0.005 (0.004 - 0.005)	0.005 (0.004 - 0.006)	0.341 (0.132 - 0.700)	0.376 (0.145 - 0.781)	0.405 (0.157 - 0.838)
**Age-standardized (rate per capita)**	0.047 (0.041 - 0.054)	0.047 (0.041 - 0.055)	0.047 (0.041 - 0.055)	0.004 (0.003 - 0.005)	0.004 (0.003 - 0.005)	0.004 (0.003 - 0.005)	0.307 (0.118 - 0.632)	0.308 (0.119 - 0.637)	0.308 (0.119 - 0.639)
**Total number of cases (thousands)**	11,574 (10,087 - 13,374)	12,206 (10,595 - 14,065)	13,396 (11,699 - 15,485)	950 (823 - 1,087)	986 (856 - 1,136)	1,057 (916 - 1,207)	75 (29 - 155)	79 (31 - 165)	87 (34 - 180)
**Belarus**									
**15 - 49 years (rate per capita)**	0.042 (0.034 - 0.050)	0.047 (0.039 - 0.055)	0.046 (0.038 - 0.054)	0.005 (0.004 - 0.006)	0.005 (0.004 - 0.006)	0.005 (0.004 - 0.006)	0.278 (0.105 - 0.593)	0.311 (0.117 - 0.659)	0.303 (0.115 - 0.638)
**50 - 69 years (rate per capita)**	0.114 (0.096 - 0.136)	0.115 (0.097 - 0.137)	0.115 (0.095 - 0.137)	0.007 (0.006 - 0.009)	0.007 (0.006 - 0.009)	0.008 (0.006 - 0.009)	0.743 (0.286 - 1.512)	0.746 (0.292 - 1.507)	0.745 (0.288 - 1.506)
**70+ years (rate per capita)**	0.097 (0.082 - 0.113)	0.099 (0.084 - 0.116)	0.098 (0.083 - 0.114)	0.005 (0.004 - 0.007)	0.006 (0.004 - 0.007)	0.006 (0.004 - 0.007)	0.602 (0.238 - 1.223)	0.617 (0.246 - 1.251)	0.607 (0.244 - 1.232)
**All ages (rate per capita)**	0.052 (0.045 - 0.060)	0.059 (0.051 - 0.068)	0.063 (0.054 - 0.072)	0.004 (0.004 - 0.005)	0.005 (0.004 - 0.005)	0.005 (0.004 - 0.006)	0.340 (0.131 - 0.704)	0.384 (0.149 - 0.794)	0.408 (0.159 - 0.842)
**Age-standardized (rate per capita)**	0.047 (0.041 - 0.055)	0.048 (0.042 - 0.055)	0.047 (0.041 - 0.055)	0.004 (0.003 - 0.005)	0.004 (0.003 - 0.005)	0.004 (0.003 - 0.005)	0.309 (0.120 - 0.642)	0.313 (0.123 - 0.651)	0.310 (0.120 - 0.638)
**Total number of cases (thousands)**	529 (460 - 612)	570 (496 - 659)	602 (518 - 697)	43 (37 - 49)	45 (39 - 52)	47 (41 - 54)	3 (1 - 7)	4 (1 - 8)	4 (2 - 8)
**Estonia**									
**15 - 49 years (rate per capita)**	0.044 (0.036 - 0.052)	0.044 (0.037 - 0.053)	0.046 (0.038 - 0.055)	0.005 (0.004 - 0.006)	0.005 (0.004 - 0.006)	0.005 (0.004 - 0.006)	0.292 (0.110 - 0.618)	0.296 (0.113 - 0.612)	0.307 (0.116 - 0.649)
**50 - 69 years (rate per capita)**	0.114 (0.096 - 0.135)	0.114 (0.096 - 0.136)	0.115 (0.096 - 0.137)	0.008 (0.006 - 0.009)	0.008 (0.006 - 0.009)	0.008 (0.006 - 0.009)	0.741 (0.289 - 1.526)	0.742 (0.291 - 1.513)	0.747 (0.288 - 1.530)
**70+ years (rate per capita)**	0.096 (0.082 - 0.112)	0.097 (0.083 - 0.115)	0.097 (0.083 - 0.112)	0.005 (0.004 - 0.007)	0.006 (0.004 - 0.007)	0.006 (0.004 - 0.007)	0.598 (0.234 - 1.204)	0.607 (0.239 - 1.230)	0.602 (0.236 - 1.224)
**All ages (rate per capita)**	0.054 (0.046 - 0.062)	0.060 (0.052 - 0.069)	0.063 (0.055 - 0.073)	0.004 (0.004 - 0.005)	0.005 (0.004 - 0.005)	0.005 (0.004 - 0.006)	0.350 (0.137 - 0.728)	0.389 (0.153 - 0.800)	0.409 (0.159 - 0.837)
**Age-standardized (rate per capita)**	0.047 (0.041 - 0.055)	0.047 (0.041 - 0.055)	0.047 (0.041 - 0.055)	0.004 (0.003 - 0.005)	0.004 (0.003 - 0.005)	0.004 (0.003 - 0.005)	0.309 (0.121 - 0.644)	0.310 (0.121 - 0.643)	0.311 (0.119 - 0.637)
**Total number of cases (thousands)**	84 (73 - 97)	81 (70 - 93)	85 (75 - 98)	7 (6 - 8)	6 (6 - 7)	7 (6 - 8)	1 (0 - 1)	1 (0 - 1)	1 (0 - 1)
**Latvia**									
**15 - 49 years (rate per capita)**	0.044 (0.037 - 0.053)	0.045 (0.037 - 0.055)	0.046 (0.038 - 0.056)	0.005 (0.004 - 0.006)	0.005 (0.004 - 0.006)	0.005 (0.004 - 0.006)	0.294 (0.112 - 0.616)	0.301 (0.114 - 0.636)	0.309 (0.116 - 0.656)
**50 - 69 years (rate per capita)**	0.116 (0.097 - 0.140)	0.117 (0.098 - 0.139)	0.117 (0.097 - 0.140)	0.007 (0.006 - 0.009)	0.007 (0.006 - 0.009)	0.008 (0.006 - 0.009)	0.753 (0.290 - 1.545)	0.757 (0.296 - 1.545)	0.760 (0.293 - 1.539)
**70+ years (rate per capita)**	0.100 (0.086 - 0.118)	0.102 (0.087 - 0.120)	0.101 (0.087 - 0.118)	0.005 (0.004 - 0.007)	0.006 (0.004 - 0.007)	0.006 (0.004 - 0.007)	0.622 (0.249 - 1.264)	0.635 (0.251 - 1.283)	0.631 (0.245 - 1.271)
**All ages (rate per capita)**	0.055 (0.048 - 0.064)	0.062 (0.054 - 0.071)	0.065 (0.057 - 0.075)	0.004 (0.004 - 0.005)	0.005 (0.004 - 0.005)	0.005 (0.004 - 0.006)	0.361 (0.140 - 0.742)	0.401 (0.156 - 0.834)	0.424 (0.166 - 0.869)
**Age-standardized (rate per capita)**	0.048 (0.042 - 0.056)	0.048 (0.042 - 0.056)	0.048 (0.042 - 0.056)	0.004 (0.003 - 0.005)	0.004 (0.003 - 0.005)	0.004 (0.003 - 0.005)	0.313 (0.122 - 0.645)	0.315 (0.123 - 0.659)	0.316 (0.123 - 0.650)
**Total number of cases (thousands)**	147 (128 - 171)	142 (123 - 163)	144 (125 - 166)	12 (10 - 13)	11 (10 - 13)	11 (10 - 13)	1 (0 - 2)	1 (0 - 2)	1 (0 - 2)
**Lithuania**									
**15 - 49 years (rate per capita)**	0.042 (0.035 - 0.051)	0.046 (0.038 - 0.055)	0.046 (0.038 - 0.055)	0.005 (0.004 - 0.006)	0.005 (0.004 - 0.006)	0.005 (0.004 - 0.006)	0.281 (0.106 - 0.581)	0.304 (0.114 - 0.641)	0.307 (0.115 - 0.638)
**50 - 69 years (rate per capita)**	0.115 (0.096 - 0.136)	0.115 (0.097 - 0.136)	0.114 (0.096 - 0.136)	0.007 (0.006 - 0.009)	0.007 (0.006 - 0.009)	0.008 (0.006 - 0.009)	0.741 (0.290 - 1.492)	0.742 (0.292 - 1.513)	0.742 (0.290 - 1.510)
**70+ years (rate per capita)**	0.097 (0.083 - 0.113)	0.099 (0.084 - 0.116)	0.097 (0.083 - 0.115)	0.005 (0.004 - 0.007)	0.006 (0.004 - 0.007)	0.006 (0.004 - 0.007)	0.598 (0.236 - 1.223)	0.610 (0.241 - 1.251)	0.603 (0.241 - 1.229)
**All ages (rate per capita)**	0.052 (0.045 - 0.060)	0.058 (0.051 - 0.067)	0.064 (0.055 - 0.074)	0.004 (0.004 - 0.005)	0.005 (0.004 - 0.005)	0.005 (0.004 - 0.006)	0.336 (0.131 - 0.691)	0.378 (0.147 - 0.772)	0.413 (0.162 - 0.853)
**Age-standardized (rate per capita)**	0.047 (0.041 - 0.055)	0.048 (0.041 - 0.055)	0.048 (0.041 - 0.055)	0.004 (0.003 - 0.005)	0.004 (0.003 - 0.005)	0.004 (0.003 - 0.005)	0.309 (0.120 - 0.635)	0.310 (0.120 - 0.637)	0.310 (0.121 - 0.641)
**Total number of cases (thousands)**	190 (165 - 220)	199 (173 - 230)	201 (174 - 233)	16 (13 - 18)	16 (14 - 18)	16 (14 - 18)	1 (0 - 3)	1 (1 - 3)	1 (1 - 3)
**Moldova**									
**15 - 49 years (rate per capita)**	0.041 (0.034 - 0.049)	0.042 (0.034 - 0.050)	0.043 (0.035 - 0.051)	0.005 (0.004 - 0.006)	0.004 (0.004 - 0.005)	0.005 (0.004 - 0.006)	0.274 (0.105 - 0.579)	0.278 (0.105 - 0.593)	0.285 (0.108 - 0.593)
**50 - 69 years (rate per capita)**	0.114 (0.095 - 0.136)	0.113 (0.096 - 0.136)	0.115 (0.096 - 0.137)	0.007 (0.006 - 0.009)	0.007 (0.006 - 0.009)	0.008 (0.006 - 0.009)	0.739 (0.290 - 1.493)	0.737 (0.285 - 1.491)	0.747 (0.291 - 1.533)
**70+ years (rate per capita)**	0.099 (0.085 - 0.117)	0.099 (0.085 - 0.117)	0.098 (0.084 - 0.115)	0.006 (0.004 - 0.007)	0.006 (0.004 - 0.007)	0.006 (0.004 - 0.007)	0.615 (0.242 - 1.265)	0.619 (0.242 - 1.256)	0.613 (0.240 - 1.244)
**All ages (rate per capita)**	0.046 (0.040 - 0.053)	0.051 (0.044 - 0.059)	0.057 (0.050 - 0.066)	0.004 (0.003 - 0.004)	0.004 (0.004 - 0.005)	0.005 (0.004 - 0.005)	0.298 (0.116 - 0.618)	0.334 (0.129 - 0.693)	0.374 (0.143 - 0.777)
**Age-standardized (rate per capita)**	0.047 (0.041 - 0.055)	0.047 (0.041 - 0.055)	0.047 (0.041 - 0.055)	0.004 (0.003 - 0.005)	0.004 (0.003 - 0.005)	0.004 (0.003 - 0.005)	0.308 (0.119 - 0.637)	0.309 (0.119 - 0.637)	0.310 (0.119 - 0.645)
**Total number of cases (thousands)**	199 (173 - 231)	213 (184 - 247)	232 (201 - 270)	17 (14 - 19)	18 (15 - 20)	19 (16 - 22)	1 (1 - 3)	1 (1 - 3)	2 (1 - 3)
**Russia**									
**15 - 49 years (rate per capita)**	0.042 (0.035 - 0.050)	0.045 (0.037 - 0.053)	0.045 (0.038 - 0.054)	0.005 (0.004 - 0.006)	0.005 (0.004 - 0.006)	0.005 (0.004 - 0.006)	0.281 (0.107 - 0.597)	0.297 (0.112 - 0.632)	0.301 (0.114 - 0.632)
**50 - 69 years (rate per capita)**	0.113 (0.094 - 0.135)	0.113 (0.095 - 0.134)	0.114 (0.095 - 0.136)	0.007 (0.006 - 0.009)	0.007 (0.006 - 0.009)	0.008 (0.006 - 0.009)	0.731 (0.284 - 1.478)	0.729 (0.283 - 1.487)	0.740 (0.286 - 1.500)
**70+ years (rate per capita)**	0.094 (0.081 - 0.110)	0.096 (0.081 - 0.113)	0.096 (0.082 - 0.112)	0.006 (0.004 - 0.007)	0.006 (0.004 - 0.007)	0.006 (0.004 - 0.007)	0.582 (0.229 - 1.164)	0.592 (0.233 - 1.199)	0.594 (0.233 - 1.213)
**All ages (rate per capita)**	0.051 (0.045 - 0.059)	0.057 (0.049 - 0.066)	0.062 (0.054 - 0.071)	0.004 (0.004 - 0.005)	0.005 (0.004 - 0.005)	0.005 (0.004 - 0.006)	0.335 (0.130 - 0.685)	0.371 (0.143 - 0.772)	0.400 (0.156 - 0.830)
**Age-standardized (rate per capita)**	0.047 (0.041 - 0.054)	0.047 (0.041 - 0.054)	0.047 (0.041 - 0.054)	0.004 (0.003 - 0.005)	0.004 (0.003 - 0.005)	0.004 (0.003 - 0.005)	0.305 (0.118 - 0.626)	0.306 (0.118 - 0.634)	0.307 (0.118 - 0.638)
**Total number of cases (thousands)**	7,580 (6,578 - 8,769)	8,180 (7,078 - 9,453)	9,124 (7,979 - 10,544)	630 (544 - 723)	667 (580 - 771)	725 (628 - 828)	49 (19 - 101)	53 (20 - 110)	59 (23 - 123)
**Ukraine**									

	Prevalence 1990	Prevalence 2005	Prevalence 2015	Incidence 1990	Incidence 2005	Incidence 2015	YLD rate 1990	YLD rate 2005	YLD rate 2015
15 - 49 years (rate per capita)	0.043 (0.036 - 0.052)	0.045 (0.038 - 0.054)	0.046 (0.038 - 0.055)	0.005 (0.004 - 0.006)	0.005 (0.004 - 0.006)	0.005 (0.004 - 0.006)	0.287 (0.108 - 0.604)	0.300 (0.113 - 0.633)	0.304 (0.115 - 0.633)
50 - 69 years (rate per capita)	0.115 (0.097 - 0.136)	0.115 (0.096 - 0.136)	0.115 (0.097 - 0.139)	0.007 (0.006 - 0.009)	0.007 (0.006 - 0.009)	0.008 (0.006 - 0.009)	0.743 (0.290 - 1.522)	0.743 (0.290 - 1.519)	0.750 (0.292 - 1.544)
70+ years (rate per capita)	0.098 (0.083 - 0.114)	0.099 (0.085 - 0.117)	0.100 (0.086 - 0.117)	0.005 (0.004 - 0.007)	0.006 (0.004 - 0.007)	0.006 (0.004 - 0.007)	0.607 (0.239 - 1.235)	0.618 (0.245 - 1.241)	0.624 (0.248 - 1.276)
All ages (rate per capita)	0.055 (0.048 - 0.064)	0.060 (0.052 - 0.069)	0.065 (0.056 - 0.075)	0.004 (0.004 - 0.005)	0.005 (0.004 - 0.005)	0.005 (0.004 - 0.006)	0.361 (0.141 - 0.747)	0.391 (0.152 - 0.812)	0.420 (0.164 - 0.864)
Age-standardized (rate per capita)	0.048 (0.041 - 0.055)	0.048 (0.041 - 0.055)	0.048 (0.042 - 0.055)	0.004 (0.003 - 0.005)	0.004 (0.003 - 0.005)	0.004 (0.003 - 0.005)	0.310 (0.120 - 0.642)	0.311 (0.120 - 0.645)	0.311 (0.121 - 0.641)
Total number of cases (thousands)	2,843 (2,477 - 3,282)	2,823 (2,458 - 3,245)	3,007 (2,623 - 3,480)	226 (197 - 257)	223 (194 - 255)	233 (203 - 266)	18 (7 - 38)	18 (7 - 38)	20 (8 - 40)
**Central Europe**									
15 - 49 years (rate per capita)	0.062 (0.051 - 0.074)	0.064 (0.053 - 0.076)	0.067 (0.055 - 0.079)	0.007 (0.005 - 0.008)	0.007 (0.005 - 0.008)	0.007 (0.006 - 0.008)	0.414 (0.157 - 0.871)	0.424 (0.161 - 0.892)	0.444 (0.169 - 0.926)
50 - 69 years (rate per capita)	0.161 (0.135 - 0.190)	0.160 (0.135 - 0.189)	0.161 (0.136 - 0.190)	0.010 (0.008 - 0.012)	0.010 (0.008 - 0.012)	0.010 (0.008 - 0.013)	1.044 (0.409 - 2.121)	1.038 (0.403 - 2.109)	1.048 (0.409 - 2.140)
70+ years (rate per capita)	0.135 (0.115 - 0.157)	0.136 (0.116 - 0.159)	0.134 (0.115 - 0.156)	0.007 (0.005 - 0.009)	0.007 (0.005 - 0.009)	0.007 (0.005 - 0.009)	0.837 (0.337 - 1.715)	0.847 (0.339 - 1.735)	0.833 (0.337 - 1.717)
All ages (rate per capita)	0.072 (0.063 - 0.083)	0.082 (0.072 - 0.095)	0.090 (0.078 - 0.103)	0.006 (0.005 - 0.007)	0.006 (0.006 - 0.007)	0.007 (0.006 - 0.008)	0.471 (0.185 - 0.966)	0.535 (0.208 - 1.091)	0.583 (0.229 - 1.194)
Age-standardized (rate per capita)	0.067 (0.058 - 0.077)	0.067 (0.058 - 0.077)	0.067 (0.059 - 0.077)	0.005 (0.005 - 0.006)	0.005 (0.005 - 0.006)	0.005 (0.005 - 0.006)	0.439 (0.172 - 0.901)	0.438 (0.170 - 0.895)	0.439 (0.171 - 0.900)
Total number of cases (thousands)	8,888 (7,729 - 10,249)	9,708 (8,468 - 11,203)	10,452 (9,073 - 12,034)	708 (618 - 811)	749 (654 - 853)	787 (687 - 896)	58 (23 - 119)	63 (25 - 129)	68 (27 - 139)
**Albania**									
15 - 49 years (rate per capita)	0.054 (0.045 - 0.064)	0.061 (0.051 - 0.073)	0.058 (0.048 - 0.069)	0.006 (0.005 - 0.007)	0.006 (0.005 - 0.008)	0.006 (0.005 - 0.007)	0.357 (0.136 - 0.751)	0.407 (0.153 - 0.855)	0.388 (0.147 - 0.822)
50 - 69 years (rate per capita)	0.163 (0.136 - 0.193)	0.163 (0.138 - 0.194)	0.163 (0.136 - 0.193)	0.010 (0.008 - 0.013)	0.010 (0.008 - 0.013)	0.010 (0.008 - 0.013)	1.060 (0.416 - 2.160)	1.063 (0.419 - 2.162)	1.061 (0.414 - 2.155)
70+ years (rate per capita)	0.140 (0.118 - 0.165)	0.140 (0.118 - 0.166)	0.139 (0.118 - 0.164)	0.007 (0.005 - 0.010)	0.007 (0.005 - 0.010)	0.007 (0.005 - 0.009)	0.873 (0.341 - 1.786)	0.879 (0.349 - 1.795)	0.871 (0.343 - 1.770)
All ages (rate per capita)	0.052 (0.045 - 0.060)	0.066 (0.057 - 0.078)	0.078 (0.068 - 0.091)	0.005 (0.004 - 0.005)	0.005 (0.005 - 0.006)	0.006 (0.005 - 0.007)	0.344 (0.134 - 0.713)	0.436 (0.169 - 0.892)	0.512 (0.200 - 1.045)
Age-standardized (rate per capita)	0.068 (0.059 - 0.078)	0.068 (0.059 - 0.079)	0.068 (0.059 - 0.079)	0.005 (0.005 - 0.006)	0.005 (0.005 - 0.006)	0.005 (0.005 - 0.006)	0.445 (0.172 - 0.918)	0.445 (0.172 - 0.909)	0.445 (0.172 - 0.908)
Total number of cases (thousands)	171 (149 - 198)	205 (177 - 239)	227 (197 - 262)	15 (13 - 17)	17 (14 - 19)	18 (15 - 20)	1 (0 - 2)	1 (1 - 3)	1 (1 - 3)
**Bosnia and Herzegovina**									
15 - 49 years (rate per capita)	0.058 (0.049 - 0.069)	0.068 (0.056 - 0.081)	0.069 (0.057 - 0.082)	0.006 (0.005 - 0.008)	0.007 (0.006 - 0.008)	0.007 (0.006 - 0.009)	0.390 (0.147 - 0.801)	0.448 (0.168 - 0.937)	0.458 (0.172 - 0.954)
50 - 69 years (rate per capita)	0.160 (0.134 - 0.190)	0.160 (0.135 - 0.189)	0.161 (0.136 - 0.190)	0.010 (0.008 - 0.013)	0.010 (0.008 - 0.013)	0.010 (0.008 - 0.013)	1.044 (0.404 - 2.115)	1.037 (0.403 - 2.114)	1.043 (0.407 - 2.106)
70+ years (rate per capita)	0.128 (0.110 - 0.148)	0.133 (0.113 - 0.154)	0.130 (0.111 - 0.151)	0.007 (0.005 - 0.009)	0.008 (0.005 - 0.010)	0.007 (0.005 - 0.009)	0.800 (0.315 - 1.627)	0.826 (0.328 - 1.697)	0.803 (0.320 - 1.634)
All ages (rate per capita)	0.066 (0.057 - 0.076)	0.083 (0.072 - 0.096)	0.092 (0.080 - 0.106)	0.006 (0.005 - 0.006)	0.007 (0.006 - 0.008)	0.007 (0.006 - 0.008)	0.431 (0.166 - 0.888)	0.539 (0.208 - 1.118)	0.594 (0.232 - 1.241)
Age-standardized (rate per capita)	0.067 (0.058 - 0.078)	0.067 (0.059 - 0.078)	0.068 (0.059 - 0.078)	0.006 (0.005 - 0.006)	0.006 (0.005 - 0.006)	0.006 (0.005 - 0.006)	0.439 (0.170 - 0.904)	0.439 (0.169 - 0.906)	0.440 (0.171 - 0.916)
Total number of cases (thousands)	297 (256 - 344)	318 (277 - 369)	349 (305 - 403)	25 (22 - 29)	25 (22 - 29)	27 (23 - 30)	2 (1 - 4)	2 (1 - 4)	2 (1 - 5)
**Bulgaria**									
15 - 49 years (rate per capita)	0.051 (0.042 - 0.061)	0.052 (0.043 - 0.062)	0.056 (0.046 - 0.066)	0.005 (0.004 - 0.006)	0.005 (0.004 - 0.007)	0.006 (0.005 - 0.007)	0.339 (0.130 - 0.703)	0.345 (0.131 - 0.725)	0.370 (0.140 - 0.788)
50 - 69 years (rate per capita)	0.130 (0.109 - 0.155)	0.130 (0.108 - 0.155)	0.131 (0.109 - 0.155)	0.009 (0.006 - 0.011)	0.009 (0.007 - 0.011)	0.009 (0.007 - 0.011)	0.844 (0.331 - 1.716)	0.844 (0.328 - 1.709)	0.849 (0.332 - 1.741)
70+ years (rate per capita)	0.115 (0.097 - 0.135)	0.115 (0.097 - 0.136)	0.114 (0.096 - 0.134)	0.007 (0.005 - 0.008)	0.007 (0.005 - 0.009)	0.007 (0.005 - 0.008)	0.712 (0.289 - 1.469)	0.719 (0.287 - 1.474)	0.709 (0.283 - 1.446)
All ages (rate per capita)	0.064 (0.056 - 0.074)	0.072 (0.063 - 0.084)	0.077 (0.067 - 0.089)	0.005 (0.004 - 0.006)	0.006 (0.005 - 0.006)	0.006 (0.005 - 0.007)	0.420 (0.166 - 0.866)	0.471 (0.185 - 0.955)	0.502 (0.199 - 1.039)
Age-standardized (rate per capita)	0.054 (0.047 - 0.062)	0.054 (0.047 - 0.063)	0.054 (0.047 - 0.063)	0.004 (0.004 - 0.005)	0.005 (0.004 - 0.005)	0.005 (0.004 - 0.005)	0.352 (0.139 - 0.726)	0.353 (0.138 - 0.721)	0.354 (0.140 - 0.734)
Total number of cases (thousands)	568 (495 - 657)	556 (482 - 642)	562 (487 - 648)	45 (39 - 52)	43 (38 - 50)	43 (37 - 49)	4 (1 - 8)	4 (1 - 7)	4 (1 - 8)
**Croatia**									
15 - 49 years (rate per capita)	0.094 (0.080 - 0.110)	0.099 (0.083 - 0.117)	0.096 (0.079 - 0.114)	0.009 (0.008 - 0.011)	0.010 (0.008 - 0.012)	0.009 (0.008 - 0.011)	0.626 (0.241 - 1.338)	0.659 (0.250 - 1.369)	0.639 (0.242 - 1.344)
50 - 69 years (rate per capita)	0.229 (0.195 - 0.266)	0.228 (0.194 - 0.266)	0.220 (0.186 - 0.257)	0.013 (0.010 - 0.017)	0.013 (0.010 - 0.016)	0.013 (0.009 - 0.016)	1.485 (0.592 - 3.038)	1.479 (0.590 - 3.057)	1.427 (0.559 - 2.907)
70+ years (rate per capita)	0.187 (0.160 - 0.216)	0.183 (0.156 - 0.213)	0.174 (0.150 - 0.201)	0.008 (0.006 - 0.011)	0.008 (0.006 - 0.011)	0.008 (0.006 - 0.010)	1.162 (0.463 - 2.364)	1.141 (0.464 - 2.304)	1.080 (0.434 - 2.216)
All ages (rate per capita)	0.114 (0.101 - 0.129)	0.124 (0.109 - 0.142)	0.126 (0.110 - 0.144)	0.008 (0.007 - 0.009)	0.009 (0.008 - 0.010)	0.009 (0.008 - 0.010)	0.744 (0.294 - 1.500)	0.808 (0.319 - 1.662)	0.817 (0.321 - 1.697)
Age-standardized (rate per capita)	0.097 (0.086 - 0.110)	0.096 (0.084 - 0.110)	0.092 (0.081 - 0.107)	0.007 (0.007 - 0.008)	0.007 (0.006 - 0.008)	0.007 (0.006 - 0.008)	0.634 (0.250 - 1.278)	0.628 (0.246 - 1.283)	0.604 (0.235 - 1.262)
Total number of cases (thousands)	544 (481 - 614)	544 (478 - 623)	533 (467 - 611)	40 (35 - 45)	38 (33 - 43)	37 (32 - 42)	4 (1 - 7)	4 (1 - 7)	3 (1 - 7)
**Czech Republic**									
15 - 49 years (rate per capita)	0.067 (0.055 - 0.080)	0.066 (0.054 - 0.078)	0.073 (0.060 - 0.087)	0.007 (0.006 - 0.008)	0.007 (0.006 - 0.008)	0.007 (0.006 - 0.009)	0.446 (0.167 - 0.938)	0.438 (0.165 - 0.923)	0.486 (0.183 - 1.033)
50 - 69 years (rate per capita)	0.164 (0.139 - 0.194)	0.164 (0.137 - 0.194)	0.165 (0.139 - 0.195)	0.010 (0.008 - 0.013)	0.010 (0.008 - 0.013)	0.010 (0.008 - 0.013)	1.066 (0.418 - 2.180)	1.069 (0.415 - 2.164)	1.072 (0.421 - 2.189)
70+ years (rate per capita)	0.136 (0.117 - 0.159)	0.137 (0.117 - 0.159)	0.136 (0.117 - 0.159)	0.007 (0.005 - 0.009)	0.007 (0.005 - 0.009)	0.007 (0.005 - 0.009)	0.847 (0.337 - 1.736)	0.851 (0.338 - 1.741)	0.848 (0.336 - 1.741)
All ages (rate per capita)	0.078 (0.068 - 0.090)	0.088 (0.076 - 0.101)	0.094 (0.082 - 0.108)	0.006 (0.005 - 0.007)	0.007 (0.006 - 0.008)	0.007 (0.006 - 0.008)	0.507 (0.196 - 1.049)	0.572 (0.221 - 1.181)	0.611 (0.238 - 1.256)
Age-standardized (rate per capita)	0.069 (0.060 - 0.079)	0.068 (0.060 - 0.079)	0.069 (0.060 - 0.079)	0.006 (0.005 - 0.006)	0.006 (0.005 - 0.006)	0.006 (0.005 - 0.006)	0.449 (0.174 - 0.930)	0.448 (0.172 - 0.924)	0.449 (0.175 - 0.930)
Total number of cases (thousands)	799 (697 - 922)	901 (782 - 1,040)	1,003 (875 - 1,150)	63 (55 - 72)	69 (60 - 78)	75 (66 - 86)	5 (2 - 11)	6 (2 - 12)	7 (3 - 13)
**Hungary**									
15 - 49 years (rate per capita)	0.045 (0.037 - 0.053)	0.042 (0.035 - 0.050)	0.045 (0.038 - 0.054)	0.005 (0.004 - 0.006)	0.005 (0.004 - 0.005)	0.005 (0.004 - 0.006)	0.298 (0.113 - 0.630)	0.282 (0.106 - 0.598)	0.303 (0.114 - 0.631)
50 - 69 years (rate per capita)	0.114 (0.095 - 0.136)	0.109 (0.093 - 0.128)	0.113 (0.094 - 0.135)	0.008 (0.006 - 0.009)	0.007 (0.006 - 0.009)	0.008 (0.006 - 0.009)	0.736 (0.291 - 1.507)	0.708 (0.278 - 1.441)	0.734 (0.289 - 1.510)
70+ years (rate per capita)	0.101 (0.087 - 0.119)	0.098 (0.084 - 0.113)	0.099 (0.086 - 0.116)	0.006 (0.005 - 0.008)	0.006 (0.005 - 0.008)	0.006 (0.005 - 0.007)	0.626 (0.247 - 1.257)	0.605 (0.242 - 1.243)	0.615 (0.247 - 1.248)
All ages (rate per capita)	0.056 (0.048 - 0.064)	0.058 (0.052 - 0.067)	0.064 (0.055 - 0.073)	0.005 (0.004 - 0.005)	0.005 (0.004 - 0.005)	0.005 (0.004 - 0.006)	0.362 (0.141 - 0.753)	0.379 (0.150 - 0.778)	0.413 (0.161 - 0.865)
Age-standardized (rate per capita)	0.047 (0.041 - 0.055)	0.045 (0.040 - 0.052)	0.047 (0.041 - 0.054)	0.004 (0.003 - 0.005)	0.004 (0.003 - 0.004)	0.004 (0.003 - 0.005)	0.307 (0.120 - 0.640)	0.295 (0.116 - 0.610)	0.306 (0.119 - 0.639)
Total number of cases (thousands)	578 (503 - 669)	588 (521 - 674)	646 (561 - 746)	47 (41 - 54)	47 (41 - 54)	51 (44 - 58)	4 (1 - 8)	4 (2 - 8)	4 (2 - 9)
**Macedonia**									
15 - 49 years (rate per capita)	0.060 (0.050 - 0.071)	0.064 (0.053 - 0.077)	0.067 (0.055 - 0.080)	0.006 (0.005 - 0.008)	0.007 (0.006 - 0.008)	0.007 (0.006 - 0.008)	0.399 (0.151 - 0.858)	0.431 (0.164 - 0.910)	0.447 (0.170 - 0.932)
50 - 69 years (rate per capita)	0.164 (0.138 - 0.194)	0.163 (0.137 - 0.193)	0.164 (0.138 - 0.194)	0.010 (0.008 - 0.013)	0.010 (0.008 - 0.013)	0.010 (0.008 - 0.013)	1.068 (0.418 - 2.193)	1.061 (0.420 - 2.161)	1.068 (0.421 - 2.175)
70+ years (rate per capita)	0.141 (0.119 - 0.166)	0.142 (0.119 - 0.167)	0.140 (0.118 - 0.165)	0.007 (0.005 - 0.010)	0.007 (0.005 - 0.010)	0.007 (0.005 - 0.010)	0.877 (0.346 - 1.807)	0.885 (0.353 - 1.822)	0.870 (0.347 - 1.788)
All ages (rate per capita)	0.065 (0.056 - 0.076)	0.076 (0.066 - 0.089)	0.085 (0.073 - 0.098)	0.005 (0.005 - 0.006)	0.006 (0.005 - 0.007)	0.007 (0.006 - 0.008)	0.429 (0.167 - 0.892)	0.501 (0.194 - 1.031)	0.553 (0.219 - 1.130)
Age-standardized (rate per capita)	0.069 (0.059 - 0.079)	0.068 (0.059 - 0.079)	0.068 (0.059 - 0.079)	0.006 (0.005 - 0.006)	0.006 (0.005 - 0.006)	0.006 (0.005 - 0.006)	0.449 (0.177 - 0.934)	0.448 (0.174 - 0.922)	0.448 (0.176 - 0.917)
Total number of cases (thousands)	131 (113 - 152)	156 (135 - 181)	176 (152 - 204)	11 (9 - 12)	12 (11 - 14)	14 (12 - 16)	1 (0 - 2)	1 (0 - 2)	1 (0 - 2)
**Montenegro**									
15 - 49 years (rate per capita)	0.059 (0.049 - 0.071)	0.064 (0.053 - 0.076)	0.066 (0.054 - 0.078)	0.006 (0.005 - 0.008)	0.007 (0.005 - 0.008)	0.007 (0.006 - 0.008)	0.392 (0.149 - 0.823)	0.428 (0.161 - 0.900)	0.439 (0.163 - 0.935)
50 - 69 years (rate per capita)	0.164 (0.137 - 0.194)	0.163 (0.137 - 0.193)	0.164 (0.138 - 0.194)	0.010 (0.008 - 0.013)	0.010 (0.008 - 0.013)	0.010 (0.008 - 0.013)	1.067 (0.419 - 2.174)	1.061 (0.412 - 2.162)	1.070 (0.418 - 2.184)
70+ years (rate per capita)	0.135 (0.116 - 0.159)	0.141 (0.119 - 0.165)	0.138 (0.118 - 0.162)	0.007 (0.005 - 0.009)	0.007 (0.005 - 0.010)	0.007 (0.005 - 0.009)	0.847 (0.337 - 1.712)	0.883 (0.347 - 1.811)	0.863 (0.341 - 1.793)
All ages (rate per capita)	0.067 (0.058 - 0.077)	0.079 (0.068 - 0.091)	0.084 (0.073 - 0.097)	0.006 (0.005 - 0.006)	0.006 (0.005 - 0.007)	0.006 (0.006 - 0.007)	0.439 (0.170 - 0.905)	0.515 (0.203 - 1.058)	0.550 (0.215 - 1.134)
Age-standardized (rate per capita)	0.068 (0.059 - 0.079)	0.068 (0.059 - 0.079)	0.069 (0.059 - 0.079)	0.006 (0.005 - 0.006)	0.006 (0.005 - 0.006)	0.006 (0.005 - 0.006)	0.447 (0.174 - 0.920)	0.448 (0.176 - 0.919)	0.449 (0.174 - 0.926)
Total number of cases (thousands)	41 (36 - 48)	49 (42 - 56)	53 (46 - 61)	3 (3 - 4)	4 (3 - 4)	4 (4 - 5)	0 (0 - 1)	0 (0 - 1)	0 (0 - 1)
**Poland**									
15 - 49 years (rate per capita)	0.064 (0.053 - 0.076)	0.064 (0.054 - 0.077)	0.064 (0.052 - 0.076)	0.007 (0.006 - 0.008)	0.007 (0.006 - 0.008)	0.007 (0.006 - 0.008)	0.425 (0.160 - 0.880)	0.429 (0.162 - 0.903)	0.424 (0.161 - 0.878)
50 - 69 years (rate per capita)	0.165 (0.138 - 0.194)	0.163 (0.137 - 0.194)	0.165 (0.137 - 0.196)	0.010 (0.008 - 0.013)	0.010 (0.008 - 0.013)	0.010 (0.008 - 0.013)	1.067 (0.420 - 2.173)	1.059 (0.411 - 2.135)	1.070 (0.416 - 2.191)
70+ years (rate per capita)	0.138 (0.116 - 0.161)	0.139 (0.118 - 0.164)	0.136 (0.116 - 0.160)	0.007 (0.005 - 0.009)	0.007 (0.005 - 0.009)	0.007 (0.005 - 0.009)	0.852 (0.339 - 1.745)	0.861 (0.346 - 1.763)	0.844 (0.337 - 1.725)
All ages (rate per capita)	0.071 (0.062 - 0.083)	0.082 (0.071 - 0.096)	0.090 (0.078 - 0.105)	0.006 (0.005 - 0.007)	0.006 (0.006 - 0.007)	0.007 (0.006 - 0.008)	0.466 (0.182 - 0.948)	0.536 (0.207 - 1.094)	0.587 (0.231 - 1.207)

	Prevalence 1990	Prevalence 2005	Prevalence 2015	Incidence 1990	Incidence 2005	Incidence 2015	YLD rate 1990	YLD rate 2005	YLD rate 2015
Age-standardized (rate per capita)	0.069 (0.060 - 0.079)	0.068 (0.059 - 0.079)	0.069 (0.060 - 0.080)	0.006 (0.005 - 0.006)	0.006 (0.005 - 0.006)	0.006 (0.005 - 0.006)	0.448 (0.176 - 0.915)	0.447 (0.173 - 0.917)	0.448 (0.176 - 0.921)
Total number of cases (thousands)	2,729 (2,358 - 3,155)	3,141 (2,729 - 3,657)	3,511 (3,046 - 4,067)	220 (191 - 251)	244 (213 - 280)	268 (233 - 305)	18 (7 - 36)	20 (8 - 42)	23 (9 - 47)
**Romania**									
15 - 49 years (rate per capita)	0.061 (0.050 - 0.073)	0.064 (0.052 - 0.076)	0.074 (0.061 - 0.089)	0.006 (0.005 - 0.008)	0.007 (0.006 - 0.008)	0.007 (0.006 - 0.009)	0.405 (0.153 - 0.847)	0.424 (0.160 - 0.887)	0.496 (0.186 - 1.033)
50 - 69 years (rate per capita)	0.164 (0.137 - 0.194)	0.163 (0.137 - 0.193)	0.165 (0.138 - 0.194)	0.010 (0.008 - 0.013)	0.010 (0.008 - 0.013)	0.010 (0.008 - 0.013)	1.065 (0.420 - 2.159)	1.061 (0.416 - 2.153)	1.072 (0.427 - 2.190)
70+ years (rate per capita)	0.138 (0.117 - 0.163)	0.140 (0.117 - 0.166)	0.137 (0.116 - 0.161)	0.007 (0.005 - 0.009)	0.007 (0.005 - 0.010)	0.007 (0.005 - 0.009)	0.862 (0.342 - 1.763)	0.875 (0.344 - 1.788)	0.855 (0.343 - 1.769)
All ages (rate per capita)	0.074 (0.064 - 0.085)	0.084 (0.073 - 0.097)	0.093 (0.081 - 0.107)	0.006 (0.005 - 0.007)	0.006 (0.006 - 0.007)	0.007 (0.006 - 0.008)	0.480 (0.190 - 0.993)	0.546 (0.212 - 1.126)	0.607 (0.239 - 1.244)
Age-standardized (rate per capita)	0.069 (0.059 - 0.079)	0.068 (0.060 - 0.079)	0.069 (0.060 - 0.079)	0.006 (0.005 - 0.006)	0.006 (0.005 - 0.006)	0.006 (0.005 - 0.006)	0.448 (0.177 - 0.925)	0.448 (0.173 - 0.929)	0.449 (0.175 - 0.921)
Total number of cases (thousands)	1,722 (1,490 - 1,998)	1,792 (1,562 - 2,076)	1,819 (1,577 - 2,093)	136 (118 - 156)	139 (121 - 157)	135 (118 - 154)	11 (4 - 23)	12 (5 - 24)	12 (5 - 24)
**Serbia**									
15 - 49 years (rate per capita)	0.064 (0.052 - 0.076)	0.064 (0.053 - 0.077)	0.068 (0.057 - 0.081)	0.007 (0.006 - 0.008)	0.007 (0.006 - 0.008)	0.007 (0.006 - 0.008)	0.424 (0.161 - 0.895)	0.429 (0.162 - 0.903)	0.454 (0.170 - 0.958)
50 - 69 years (rate per capita)	0.165 (0.138 - 0.195)	0.164 (0.138 - 0.194)	0.165 (0.138 - 0.194)	0.010 (0.008 - 0.013)	0.010 (0.008 - 0.013)	0.010 (0.008 - 0.013)	1.069 (0.418 - 2.199)	1.066 (0.413 - 2.174)	1.067 (0.421 - 2.184)
70+ years (rate per capita)	0.138 (0.117 - 0.162)	0.141 (0.119 - 0.168)	0.138 (0.116 - 0.162)	0.007 (0.005 - 0.009)	0.007 (0.005 - 0.010)	0.007 (0.005 - 0.009)	0.860 (0.343 - 1.771)	0.879 (0.348 - 1.816)	0.856 (0.337 - 1.762)
All ages (rate per capita)	0.074 (0.064 - 0.086)	0.083 (0.072 - 0.095)	0.090 (0.078 - 0.103)	0.006 (0.005 - 0.007)	0.006 (0.006 - 0.007)	0.007 (0.006 - 0.008)	0.487 (0.190 - 1.001)	0.541 (0.214 - 1.097)	0.584 (0.229 - 1.194)
Age-standardized (rate per capita)	0.069 (0.059 - 0.079)	0.069 (0.059 - 0.079)	0.069 (0.060 - 0.080)	0.006 (0.005 - 0.006)	0.006 (0.005 - 0.006)	0.006 (0.005 - 0.006)	0.449 (0.175 - 0.923)	0.449 (0.175 - 0.917)	0.448 (0.174 - 0.924)
Total number of cases (thousands)	708 (610 - 819)	765 (664 - 878)	795 (692 - 914)	56 (49 - 65)	58 (51 - 66)	59 (52 - 68)	5 (2 - 10)	5 (2 - 10)	5 (2 - 11)
**Slovakia**									
15 - 49 years (rate per capita)	0.063 (0.052 - 0.074)	0.065 (0.054 - 0.077)	0.067 (0.056 - 0.081)	0.007 (0.006 - 0.008)	0.007 (0.006 - 0.008)	0.007 (0.006 - 0.008)	0.418 (0.158 - 0.878)	0.432 (0.162 - 0.910)	0.449 (0.171 - 0.937)
50 - 69 years (rate per capita)	0.171 (0.144 - 0.202)	0.170 (0.142 - 0.202)	0.171 (0.144 - 0.201)	0.010 (0.008 - 0.013)	0.010 (0.008 - 0.013)	0.010 (0.008 - 0.013)	1.112 (0.436 - 2.259)	1.106 (0.426 - 2.269)	1.114 (0.435 - 2.271)
70+ years (rate per capita)	0.152 (0.131 - 0.176)	0.152 (0.130 - 0.176)	0.150 (0.129 - 0.173)	0.007 (0.005 - 0.009)	0.007 (0.005 - 0.009)	0.007 (0.005 - 0.009)	0.945 (0.372 - 1.920)	0.948 (0.377 - 1.938)	0.934 (0.371 - 1.881)
All ages (rate per capita)	0.072 (0.062 - 0.083)	0.083 (0.072 - 0.097)	0.092 (0.080 - 0.106)	0.006 (0.005 - 0.006)	0.006 (0.006 - 0.007)	0.007 (0.006 - 0.008)	0.469 (0.182 - 0.978)	0.544 (0.210 - 1.133)	0.601 (0.235 - 1.252)
Age-standardized (rate per capita)	0.071 (0.062 - 0.082)	0.071 (0.062 - 0.082)	0.071 (0.062 - 0.081)	0.006 (0.005 - 0.006)	0.006 (0.005 - 0.006)	0.006 (0.005 - 0.006)	0.462 (0.179 - 0.968)	0.463 (0.178 - 0.964)	0.463 (0.181 - 0.964)
Total number of cases (thousands)	378 (329 - 435)	448 (389 - 521)	511 (445 - 588)	30 (26 - 34)	34 (30 - 39)	38 (33 - 43)	2 (1 - 5)	3 (1 - 6)	3 (1 - 7)
**Slovenia**									
15 - 49 years (rate per capita)	0.098 (0.081 - 0.116)	0.099 (0.082 - 0.119)	0.105 (0.087 - 0.125)	0.010 (0.008 - 0.012)	0.010 (0.008 - 0.012)	0.010 (0.008 - 0.012)	0.653 (0.246 - 1.368)	0.662 (0.253 - 1.385)	0.704 (0.267 - 1.480)
50 - 69 years (rate per capita)	0.230 (0.194 - 0.267)	0.222 (0.189 - 0.259)	0.223 (0.189 - 0.262)	0.013 (0.009 - 0.016)	0.013 (0.010 - 0.016)	0.013 (0.010 - 0.016)	1.491 (0.590 - 2.991)	1.445 (0.569 - 2.958)	1.455 (0.571 - 2.956)
70+ years (rate per capita)	0.170 (0.147 - 0.196)	0.170 (0.146 - 0.196)	0.169 (0.146 - 0.196)	0.008 (0.006 - 0.010)	0.008 (0.006 - 0.011)	0.008 (0.006 - 0.010)	1.051 (0.426 - 2.136)	1.057 (0.424 - 2.156)	1.050 (0.426 - 2.122)
All ages (rate per capita)	0.110 (0.095 - 0.125)	0.122 (0.107 - 0.141)	0.130 (0.114 - 0.149)	0.008 (0.007 - 0.010)	0.009 (0.008 - 0.010)	0.009 (0.008 - 0.010)	0.718 (0.282 - 1.488)	0.798 (0.311 - 1.672)	0.848 (0.330 - 1.734)
Age-standardized (rate per capita)	0.098 (0.085 - 0.112)	0.094 (0.083 - 0.109)	0.094 (0.082 - 0.108)	0.008 (0.007 - 0.009)	0.007 (0.006 - 0.008)	0.007 (0.006 - 0.008)	0.642 (0.251 - 1.329)	0.618 (0.240 - 1.288)	0.619 (0.240 - 1.280)
Total number of cases (thousands)	220 (192 - 252)	244 (214 - 281)	268 (234 - 308)	17 (15 - 19)	18 (16 - 20)	19 (16 - 22)	1 (1 - 3)	2 (1 - 3)	2 (1 - 4)
**Central Asia**									
15 - 49 years (rate per capita)	0.041 (0.035 - 0.049)	0.046 (0.038 - 0.055)	0.047 (0.039 - 0.056)	0.005 (0.004 - 0.006)	0.005 (0.004 - 0.006)	0.005 (0.004 - 0.006)	0.276 (0.106 - 0.584)	0.306 (0.116 - 0.656)	0.313 (0.118 - 0.660)
50 - 69 years (rate per capita)	0.129 (0.108 - 0.154)	0.128 (0.108 - 0.152)	0.129 (0.108 - 0.155)	0.009 (0.006 - 0.011)	0.009 (0.007 - 0.011)	0.009 (0.007 - 0.011)	0.841 (0.327 - 1.706)	0.834 (0.324 - 1.696)	0.844 (0.324 - 1.720)
70+ years (rate per capita)	0.100 (0.085 - 0.117)	0.103 (0.087 - 0.120)	0.101 (0.086 - 0.119)	0.006 (0.005 - 0.008)	0.006 (0.005 - 0.008)	0.006 (0.005 - 0.008)	0.625 (0.246 - 1.259)	0.642 (0.250 - 1.300)	0.631 (0.247 - 1.265)
All ages (rate per capita)	0.039 (0.034 - 0.046)	0.044 (0.038 - 0.051)	0.049 (0.042 - 0.057)	0.004 (0.003 - 0.004)	0.004 (0.003 - 0.005)	0.004 (0.004 - 0.005)	0.260 (0.101 - 0.537)	0.289 (0.112 - 0.601)	0.321 (0.123 - 0.670)
Age-standardized (rate per capita)	0.054 (0.047 - 0.062)	0.054 (0.047 - 0.062)	0.054 (0.047 - 0.062)	0.005 (0.004 - 0.005)	0.005 (0.004 - 0.005)	0.005 (0.004 - 0.005)	0.353 (0.137 - 0.729)	0.353 (0.136 - 0.724)	0.353 (0.137 - 0.731)
Total number of cases (thousands)	2,716 (2,349 - 3,134)	3,385 (2,927 - 3,927)	4,247 (3,673 - 4,932)	251 (218 - 288)	310 (269 - 358)	380 (330 - 437)	18 (7 - 37)	22 (9 - 46)	28 (11 - 58)
**Armenia**									
15 - 49 years (rate per capita)	0.044 (0.037 - 0.053)	0.050 (0.041 - 0.059)	0.049 (0.041 - 0.058)	0.005 (0.004 - 0.006)	0.005 (0.004 - 0.006)	0.005 (0.005 - 0.006)	0.294 (0.112 - 0.610)	0.331 (0.125 - 0.694)	0.327 (0.125 - 0.684)
50 - 69 years (rate per capita)	0.130 (0.108 - 0.155)	0.128 (0.106 - 0.151)	0.129 (0.108 - 0.154)	0.009 (0.006 - 0.011)	0.009 (0.007 - 0.011)	0.009 (0.006 - 0.011)	0.841 (0.324 - 1.721)	0.827 (0.322 - 1.682)	0.838 (0.324 - 1.707)
70+ years (rate per capita)	0.100 (0.085 - 0.119)	0.103 (0.088 - 0.121)	0.100 (0.085 - 0.118)	0.006 (0.005 - 0.008)	0.006 (0.005 - 0.008)	0.006 (0.005 - 0.008)	0.621 (0.246 - 1.242)	0.641 (0.251 - 1.311)	0.616 (0.244 - 1.245)
All ages (rate per capita)	0.047 (0.041 - 0.054)	0.055 (0.048 - 0.064)	0.063 (0.055 - 0.073)	0.004 (0.004 - 0.005)	0.005 (0.004 - 0.005)	0.005 (0.005 - 0.006)	0.308 (0.119 - 0.640)	0.361 (0.139 - 0.745)	0.411 (0.161 - 0.854)
Age-standardized (rate per capita)	0.054 (0.047 - 0.062)	0.054 (0.047 - 0.062)	0.054 (0.047 - 0.062)	0.005 (0.004 - 0.005)	0.005 (0.004 - 0.005)	0.005 (0.004 - 0.005)	0.352 (0.137 - 0.732)	0.351 (0.136 - 0.723)	0.351 (0.137 - 0.727)
Total number of cases (thousands)	166 (144 - 192)	167 (145 - 193)	190 (165 - 219)	15 (13 - 17)	14 (12 - 17)	16 (14 - 18)	1 (0 - 2)	1 (0 - 2)	1 (0 - 3)
**Azerbaijan**									
15 - 49 years (rate per capita)	0.041 (0.034 - 0.049)	0.049 (0.041 - 0.058)	0.049 (0.041 - 0.058)	0.005 (0.004 - 0.006)	0.005 (0.004 - 0.006)	0.005 (0.004 - 0.006)	0.272 (0.104 - 0.562)	0.327 (0.124 - 0.695)	0.327 (0.123 - 0.695)
50 - 69 years (rate per capita)	0.129 (0.107 - 0.155)	0.128 (0.108 - 0.152)	0.130 (0.108 - 0.157)	0.009 (0.006 - 0.011)	0.009 (0.007 - 0.011)	0.009 (0.007 - 0.011)	0.842 (0.328 - 1.738)	0.833 (0.323 - 1.709)	0.849 (0.325 - 1.733)
70+ years (rate per capita)	0.102 (0.086 - 0.120)	0.105 (0.089 - 0.125)	0.102 (0.087 - 0.120)	0.006 (0.005 - 0.008)	0.007 (0.005 - 0.009)	0.006 (0.005 - 0.008)	0.639 (0.249 - 1.295)	0.660 (0.257 - 1.333)	0.637 (0.249 - 1.317)
All ages (rate per capita)	0.040 (0.035 - 0.047)	0.047 (0.041 - 0.055)	0.056 (0.048 - 0.065)	0.004 (0.003 - 0.004)	0.004 (0.004 - 0.005)	0.005 (0.004 - 0.006)	0.265 (0.102 - 0.547)	0.310 (0.118 - 0.650)	0.366 (0.141 - 0.761)
Age-standardized (rate per capita)	0.054 (0.046 - 0.062)	0.054 (0.047 - 0.062)	0.054 (0.047 - 0.063)	0.005 (0.004 - 0.005)	0.005 (0.004 - 0.005)	0.005 (0.004 - 0.005)	0.352 (0.137 - 0.725)	0.353 (0.137 - 0.739)	0.354 (0.136 - 0.738)
Total number of cases (thousands)	288 (247 - 334)	407 (352 - 472)	545 (471 - 637)	27 (23 - 31)	37 (32 - 43)	48 (41 - 55)	2 (1 - 4)	3 (1 - 6)	4 (1 - 7)
**Georgia**									
15 - 49 years (rate per capita)	0.048 (0.040 - 0.057)	0.052 (0.043 - 0.062)	0.053 (0.043 - 0.063)	0.005 (0.004 - 0.006)	0.005 (0.005 - 0.007)	0.006 (0.005 - 0.007)	0.319 (0.121 - 0.660)	0.348 (0.131 - 0.736)	0.353 (0.133 - 0.753)
50 - 69 years (rate per capita)	0.131 (0.110 - 0.157)	0.130 (0.110 - 0.154)	0.131 (0.110 - 0.157)	0.009 (0.006 - 0.011)	0.009 (0.006 - 0.010)	0.009 (0.006 - 0.011)	0.858 (0.332 - 1.742)	0.849 (0.330 - 1.720)	0.856 (0.333 - 1.758)
70+ years (rate per capita)	0.105 (0.090 - 0.124)	0.107 (0.091 - 0.126)	0.105 (0.090 - 0.123)	0.006 (0.005 - 0.008)	0.006 (0.005 - 0.008)	0.006 (0.005 - 0.008)	0.661 (0.262 - 1.317)	0.672 (0.270 - 1.340)	0.655 (0.262 - 1.325)
All ages (rate per capita)	0.057 (0.050 - 0.066)	0.063 (0.055 - 0.073)	0.067 (0.058 - 0.077)	0.005 (0.004 - 0.005)	0.005 (0.004 - 0.006)	0.005 (0.005 - 0.006)	0.375 (0.144 - 0.773)	0.412 (0.160 - 0.860)	0.437 (0.170 - 0.914)
Age-standardized (rate per capita)	0.055 (0.047 - 0.063)	0.055 (0.047 - 0.063)	0.055 (0.047 - 0.063)	0.005 (0.004 - 0.005)	0.005 (0.004 - 0.005)	0.005 (0.004 - 0.005)	0.359 (0.138 - 0.740)	0.358 (0.139 - 0.749)	0.358 (0.139 - 0.747)
Total number of cases (thousands)	312 (271 - 363)	284 (247 - 329)	268 (233 - 309)	26 (22 - 30)	23 (20 - 27)	22 (19 - 25)	2 (1 - 4)	2 (1 - 4)	2 (1 - 4)
**Kazakhstan**									
15 - 49 years (rate per capita)	0.045 (0.037 - 0.053)	0.048 (0.040 - 0.058)	0.050 (0.042 - 0.059)	0.005 (0.004 - 0.006)	0.005 (0.004 - 0.006)	0.005 (0.005 - 0.007)	0.298 (0.112 - 0.633)	0.321 (0.121 - 0.681)	0.331 (0.125 - 0.701)
50 - 69 years (rate per capita)	0.127 (0.107 - 0.152)	0.126 (0.106 - 0.150)	0.128 (0.107 - 0.152)	0.009 (0.006 - 0.011)	0.009 (0.006 - 0.011)	0.009 (0.007 - 0.011)	0.828 (0.322 - 1.681)	0.821 (0.319 - 1.683)	0.834 (0.319 - 1.707)
70+ years (rate per capita)	0.095 (0.081 - 0.111)	0.097 (0.082 - 0.114)	0.097 (0.083 - 0.114)	0.006 (0.005 - 0.008)	0.006 (0.005 - 0.008)	0.006 (0.005 - 0.008)	0.595 (0.237 - 1.202)	0.607 (0.240 - 1.219)	0.607 (0.236 - 1.227)
All ages (rate per capita)	0.044 (0.038 - 0.052)	0.050 (0.043 - 0.058)	0.052 (0.045 - 0.061)	0.004 (0.004 - 0.005)	0.004 (0.004 - 0.005)	0.005 (0.004 - 0.005)	0.292 (0.114 - 0.606)	0.328 (0.127 - 0.688)	0.344 (0.131 - 0.714)
Age-standardized (rate per capita)	0.053 (0.046 - 0.062)	0.053 (0.046 - 0.062)	0.053 (0.046 - 0.062)	0.005 (0.004 - 0.005)	0.005 (0.004 - 0.005)	0.005 (0.004 - 0.005)	0.348 (0.134 - 0.717)	0.348 (0.135 - 0.732)	0.349 (0.134 - 0.721)
Total number of cases (thousands)	738 (638 - 856)	773 (667 - 902)	919 (792 - 1,067)	67 (58 - 77)	69 (60 - 80)	81 (71 - 93)	5 (2 - 10)	5 (2 - 11)	6 (2 - 13)
**Kyrgyzstan**									
15 - 49 years (rate per capita)	0.040 (0.034 - 0.048)	0.045 (0.038 - 0.054)	0.045 (0.038 - 0.054)	0.005 (0.004 - 0.006)	0.005 (0.004 - 0.006)	0.005 (0.004 - 0.006)	0.269 (0.103 - 0.563)	0.300 (0.113 - 0.629)	0.302 (0.113 - 0.632)
50 - 69 years (rate per capita)	0.129 (0.108 - 0.154)	0.128 (0.107 - 0.153)	0.130 (0.108 - 0.155)	0.009 (0.006 - 0.011)	0.009 (0.007 - 0.011)	0.009 (0.007 - 0.011)	0.841 (0.325 - 1.716)	0.837 (0.322 - 1.703)	0.846 (0.327 - 1.718)
70+ years (rate per capita)	0.100 (0.085 - 0.118)	0.103 (0.087 - 0.121)	0.101 (0.086 - 0.119)	0.006 (0.005 - 0.008)	0.006 (0.005 - 0.008)	0.006 (0.005 - 0.008)	0.626 (0.248 - 1.254)	0.642 (0.253 - 1.288)	0.633 (0.251 - 1.295)
All ages (rate per capita)	0.038 (0.033 - 0.044)	0.041 (0.035 - 0.048)	0.044 (0.038 - 0.051)	0.004 (0.003 - 0.004)	0.004 (0.003 - 0.004)	0.004 (0.003 - 0.005)	0.250 (0.098 - 0.516)	0.270 (0.105 - 0.566)	0.291 (0.112 - 0.608)
Age-standardized (rate per capita)	0.054 (0.047 - 0.062)	0.054 (0.047 - 0.062)	0.054 (0.047 - 0.062)	0.005 (0.004 - 0.005)	0.005 (0.004 - 0.005)	0.005 (0.004 - 0.005)	0.353 (0.137 - 0.726)	0.354 (0.136 - 0.733)	0.354 (0.137 - 0.732)
Total number of cases (thousands)	167 (145 - 193)	211 (182 - 245)	260 (225 - 303)	15 (13 - 18)	20 (17 - 23)	24 (21 - 27)	1 (0 - 2)	1 (1 - 3)	2 (1 - 4)
**Mongolia**									
15 - 49 years (rate per capita)	0.038 (0.032 - 0.045)	0.044 (0.037 - 0.052)	0.049 (0.041 - 0.058)	0.005 (0.004 - 0.005)	0.005 (0.004 - 0.006)	0.005 (0.004 - 0.006)	0.255 (0.097 - 0.532)	0.291 (0.110 - 0.621)	0.326 (0.122 - 0.695)
50 - 69 years (rate per capita)	0.130 (0.109 - 0.155)	0.130 (0.109 - 0.154)	0.129 (0.108 - 0.155)	0.009 (0.006 - 0.011)	0.009 (0.007 - 0.011)	0.009 (0.007 - 0.011)	0.849 (0.329 - 1.725)	0.846 (0.330 - 1.713)	0.846 (0.328 - 1.706)

	Prevalence 1990	Prevalence 2005	Prevalence 2015	Incidence 1990	Incidence 2005	Incidence 2015	YLD rate 1990	YLD rate 2005	YLD rate 2015
70+ years (rate per capita)	0.105 (0.089 - 0.124)	0.105 (0.089 - 0.124)	0.104 (0.089 - 0.123)	0.007 (0.005 - 0.009)	0.007 (0.005 - 0.009)	0.006 (0.005 - 0.008)	0.661 (0.264 - 1.328)	0.658 (0.258 - 1.350)	0.650 (0.255 - 1.325)
All ages (rate per capita)	0.033 (0.029 - 0.038)	0.040 (0.034 - 0.046)	0.047 (0.040 - 0.055)	0.003 (0.003 - 0.004)	0.004 (0.003 - 0.005)	0.004 (0.004 - 0.005)	0.217 (0.085 - 0.453)	0.264 (0.101 - 0.540)	0.309 (0.118 - 0.648)
Age-standardized (rate per capita)	0.054 (0.047 - 0.063)	0.054 (0.047 - 0.063)	0.054 (0.047 - 0.062)	0.005 (0.004 - 0.005)	0.005 (0.004 - 0.005)	0.005 (0.004 - 0.005)	0.356 (0.139 - 0.745)	0.355 (0.138 - 0.735)	0.355 (0.136 - 0.734)
Total number of cases (thousands)	72 (63 - 84)	101 (87 - 118)	138 (119 - 161)	7 (6 - 8)	10 (9 - 11)	13 (11 - 15)	0 (0 - 1)	1 (0 - 1)	1 (0 - 2)
**Tajikistan**									
15 - 49 years (rate per capita)	0.037 (0.031 - 0.045)	0.041 (0.034 - 0.049)	0.042 (0.035 - 0.050)	0.005 (0.004 - 0.005)	0.005 (0.004 - 0.006)	0.005 (0.004 - 0.006)	0.250 (0.095 - 0.522)	0.276 (0.104 - 0.583)	0.283 (0.107 - 0.590)
50 - 69 years (rate per capita)	0.130 (0.108 - 0.154)	0.129 (0.108 - 0.154)	0.130 (0.108 - 0.157)	0.009 (0.006 - 0.011)	0.009 (0.006 - 0.011)	0.009 (0.007 - 0.011)	0.844 (0.323 - 1.716)	0.841 (0.325 - 1.718)	0.849 (0.329 - 1.731)
70+ years (rate per capita)	0.101 (0.086 - 0.120)	0.105 (0.088 - 0.124)	0.104 (0.088 - 0.123)	0.006 (0.005 - 0.008)	0.006 (0.005 - 0.009)	0.006 (0.005 - 0.008)	0.633 (0.252 - 1.278)	0.656 (0.263 - 1.331)	0.652 (0.258 - 1.327)
All ages (rate per capita)	0.032 (0.028 - 0.037)	0.033 (0.029 - 0.038)	0.038 (0.033 - 0.044)	0.003 (0.003 - 0.003)	0.003 (0.003 - 0.004)	0.004 (0.003 - 0.004)	0.208 (0.081 - 0.433)	0.218 (0.084 - 0.455)	0.250 (0.097 - 0.517)
Age-standardized (rate per capita)	0.054 (0.047 - 0.063)	0.054 (0.047 - 0.063)	0.054 (0.047 - 0.063)	0.005 (0.004 - 0.005)	0.005 (0.004 - 0.005)	0.005 (0.004 - 0.005)	0.354 (0.138 - 0.736)	0.355 (0.138 - 0.740)	0.355 (0.138 - 0.736)
Total number of cases (thousands)	167 (145 - 193)	227 (196 - 263)	322 (278 - 375)	16 (14 - 18)	22 (19 - 26)	31 (27 - 36)	1 (0 - 2)	1 (1 - 3)	2 (1 - 4)
**Turkmenistan**									
15 - 49 years (rate per capita)	0.039 (0.032 - 0.046)	0.044 (0.037 - 0.053)	0.046 (0.039 - 0.055)	0.005 (0.004 - 0.006)	0.005 (0.004 - 0.006)	0.005 (0.004 - 0.006)	0.258 (0.099 - 0.539)	0.297 (0.112 - 0.618)	0.308 (0.116 - 0.646)
50 - 69 years (rate per capita)	0.130 (0.108 - 0.154)	0.129 (0.107 - 0.154)	0.129 (0.108 - 0.154)	0.009 (0.006 - 0.011)	0.009 (0.006 - 0.011)	0.009 (0.007 - 0.011)	0.845 (0.328 - 1.699)	0.840 (0.326 - 1.700)	0.845 (0.328 - 1.704)
70+ years (rate per capita)	0.101 (0.085 - 0.120)	0.103 (0.087 - 0.122)	0.102 (0.086 - 0.120)	0.006 (0.005 - 0.008)	0.006 (0.005 - 0.008)	0.006 (0.005 - 0.008)	0.637 (0.253 - 1.303)	0.648 (0.256 - 1.289)	0.637 (0.248 - 1.271)
All ages (rate per capita)	0.033 (0.029 - 0.038)	0.039 (0.034 - 0.046)	0.046 (0.039 - 0.053)	0.003 (0.003 - 0.004)	0.004 (0.003 - 0.004)	0.004 (0.004 - 0.005)	0.220 (0.086 - 0.458)	0.260 (0.100 - 0.540)	0.301 (0.116 - 0.624)
Age-standardized (rate per capita)	0.054 (0.047 - 0.062)	0.054 (0.047 - 0.063)	0.054 (0.047 - 0.062)	0.005 (0.004 - 0.005)	0.005 (0.004 - 0.005)	0.005 (0.004 - 0.005)	0.355 (0.138 - 0.731)	0.354 (0.136 - 0.729)	0.354 (0.137 - 0.727)
Total number of cases (thousands)	122 (105 - 141)	187 (161 - 218)	246 (213 - 286)	12 (10 - 14)	18 (15 - 21)	23 (20 - 26)	1 (0 - 2)	1 (0 - 3)	2 (1 - 3)
**Uzbekistan**									
15 - 49 years (rate per capita)	0.038 (0.032 - 0.046)	0.044 (0.036 - 0.052)	0.045 (0.038 - 0.054)	0.005 (0.004 - 0.006)	0.005 (0.004 - 0.006)	0.005 (0.004 - 0.006)	0.256 (0.098 - 0.540)	0.292 (0.109 - 0.620)	0.303 (0.113 - 0.633)
50 - 69 years (rate per capita)	0.130 (0.108 - 0.154)	0.129 (0.108 - 0.153)	0.129 (0.108 - 0.155)	0.009 (0.006 - 0.011)	0.009 (0.007 - 0.011)	0.009 (0.006 - 0.011)	0.844 (0.330 - 1.712)	0.838 (0.326 - 1.694)	0.845 (0.326 - 1.721)
70+ years (rate per capita)	0.100 (0.085 - 0.118)	0.103 (0.087 - 0.121)	0.102 (0.086 - 0.120)	0.006 (0.005 - 0.008)	0.006 (0.005 - 0.008)	0.006 (0.005 - 0.008)	0.626 (0.249 - 1.252)	0.645 (0.253 - 1.302)	0.635 (0.249 - 1.281)
All ages (rate per capita)	0.033 (0.029 - 0.038)	0.039 (0.034 - 0.046)	0.045 (0.039 - 0.053)	0.003 (0.003 - 0.004)	0.004 (0.003 - 0.004)	0.004 (0.004 - 0.005)	0.219 (0.085 - 0.455)	0.260 (0.101 - 0.541)	0.299 (0.114 - 0.622)
Age-standardized (rate per capita)	0.054 (0.047 - 0.062)	0.054 (0.047 - 0.062)	0.054 (0.047 - 0.063)	0.005 (0.004 - 0.005)	0.005 (0.004 - 0.005)	0.005 (0.004 - 0.005)	0.354 (0.138 - 0.734)	0.354 (0.137 - 0.725)	0.354 (0.137 - 0.732)
Total number of cases (thousands)	684 (590 - 789)	1,028 (888 - 1,192)	1,358 (1,177 - 1,579)	65 (57 - 75)	97 (84 - 112)	125 (108 - 144)	5 (2 - 9)	7 (3 - 14)	9 (3 - 19)
**Latin America and Caribbean**									
15 - 49 years (rate per capita)	0.075 (0.063 - 0.088)	0.083 (0.070 - 0.098)	0.086 (0.072 - 0.102)	0.008 (0.007 - 0.010)	0.009 (0.007 - 0.010)	0.009 (0.008 - 0.011)	0.500 (0.191 - 1.056)	0.553 (0.211 - 1.155)	0.573 (0.218 - 1.201)
50 - 69 years (rate per capita)	0.219 (0.186 - 0.258)	0.215 (0.182 - 0.253)	0.217 (0.184 - 0.255)	0.014 (0.010 - 0.017)	0.013 (0.010 - 0.016)	0.013 (0.010 - 0.017)	1.429 (0.556 - 2.883)	1.400 (0.544 - 2.832)	1.414 (0.550 - 2.852)
70+ years (rate per capita)	0.160 (0.139 - 0.185)	0.156 (0.134 - 0.180)	0.157 (0.135 - 0.181)	0.009 (0.006 - 0.011)	0.009 (0.006 - 0.011)	0.008 (0.006 - 0.011)	1.002 (0.403 - 2.061)	0.971 (0.390 - 1.993)	0.977 (0.394 - 2.004)
All ages (rate per capita)	0.064 (0.056 - 0.073)	0.077 (0.067 - 0.088)	0.087 (0.076 - 0.101)	0.006 (0.005 - 0.007)	0.007 (0.006 - 0.008)	0.007 (0.006 - 0.008)	0.418 (0.161 - 0.867)	0.504 (0.194 - 1.046)	0.574 (0.222 - 1.203)
Age-standardized (rate per capita)	0.093 (0.081 - 0.106)	0.092 (0.081 - 0.106)	0.092 (0.080 - 0.106)	0.007 (0.007 - 0.008)	0.007 (0.006 - 0.008)	0.007 (0.006 - 0.008)	0.608 (0.237 - 1.269)	0.602 (0.234 - 1.256)	0.601 (0.234 - 1.256)
Total number of cases (thousands)	25,305 (22,210 - 29,026)	38,705 (33,840 - 44,692)	49,817 (43,472 - 57,605)	2,284 (2,002 - 2,620)	3,344 (2,928 - 3,847)	4,153 (3,638 - 4,759)	167 (64 - 345)	255 (98 - 529)	327 (127 - 686)
**Central Latin America**									
15 - 49 years (rate per capita)	0.068 (0.057 - 0.080)	0.076 (0.064 - 0.089)	0.080 (0.067 - 0.094)	0.008 (0.006 - 0.009)	0.008 (0.007 - 0.010)	0.008 (0.007 - 0.010)	0.452 (0.171 - 0.942)	0.507 (0.193 - 1.054)	0.531 (0.202 - 1.109)
50 - 69 years (rate per capita)	0.209 (0.176 - 0.245)	0.209 (0.176 - 0.247)	0.208 (0.175 - 0.245)	0.013 (0.009 - 0.016)	0.013 (0.009 - 0.016)	0.013 (0.009 - 0.016)	1.360 (0.527 - 2.745)	1.363 (0.529 - 2.760)	1.357 (0.527 - 2.743)
70+ years (rate per capita)	0.161 (0.139 - 0.185)	0.160 (0.138 - 0.184)	0.158 (0.137 - 0.182)	0.008 (0.006 - 0.011)	0.008 (0.006 - 0.011)	0.008 (0.006 - 0.010)	1.003 (0.404 - 2.066)	0.998 (0.400 - 2.042)	0.987 (0.396 - 2.027)
All ages (rate per capita)	0.056 (0.049 - 0.065)	0.069 (0.060 - 0.080)	0.079 (0.069 - 0.092)	0.005 (0.004 - 0.006)	0.006 (0.005 - 0.007)	0.007 (0.006 - 0.008)	0.371 (0.143 - 0.770)	0.454 (0.175 - 0.941)	0.521 (0.202 - 1.087)
Age-standardized (rate per capita)	0.088 (0.077 - 0.101)	0.088 (0.077 - 0.102)	0.088 (0.077 - 0.101)	0.007 (0.006 - 0.008)	0.007 (0.006 - 0.008)	0.007 (0.006 - 0.008)	0.578 (0.225 - 1.206)	0.579 (0.225 - 1.203)	0.577 (0.225 - 1.204)
Total number of cases (thousands)	9,500 (8,317 - 10,932)	15,079 (13,132 - 17,429)	19,925 (17,378 - 23,056)	863 (754 - 990)	1,313 (1,147 - 1,512)	1,671 (1,460 - 1,921)	63 (24 - 130)	99 (38 - 206)	131 (51 - 274)
**Colombia**									
15 - 49 years (rate per capita)	0.069 (0.058 - 0.081)	0.078 (0.066 - 0.093)	0.081 (0.068 - 0.096)	0.008 (0.006 - 0.009)	0.008 (0.007 - 0.010)	0.008 (0.007 - 0.010)	0.459 (0.175 - 0.962)	0.522 (0.200 - 1.076)	0.542 (0.205 - 1.126)
50 - 69 years (rate per capita)	0.203 (0.170 - 0.238)	0.203 (0.169 - 0.240)	0.203 (0.170 - 0.238)	0.012 (0.009 - 0.015)	0.012 (0.009 - 0.016)	0.012 (0.009 - 0.015)	1.324 (0.514 - 2.674)	1.326 (0.511 - 2.702)	1.324 (0.512 - 2.699)
70+ years (rate per capita)	0.155 (0.133 - 0.180)	0.153 (0.132 - 0.178)	0.153 (0.131 - 0.177)	0.008 (0.006 - 0.011)	0.008 (0.006 - 0.010)	0.008 (0.006 - 0.010)	0.971 (0.392 - 1.995)	0.959 (0.383 - 1.947)	0.954 (0.377 - 1.953)
All ages (rate per capita)	0.058 (0.050 - 0.067)	0.074 (0.064 - 0.085)	0.085 (0.074 - 0.098)	0.005 (0.005 - 0.006)	0.006 (0.005 - 0.007)	0.007 (0.006 - 0.008)	0.381 (0.148 - 0.791)	0.485 (0.186 - 0.999)	0.561 (0.218 - 1.169)
Age-standardized (rate per capita)	0.086 (0.075 - 0.099)	0.086 (0.075 - 0.099)	0.086 (0.075 - 0.098)	0.007 (0.006 - 0.008)	0.007 (0.006 - 0.008)	0.007 (0.006 - 0.008)	0.564 (0.219 - 1.170)	0.565 (0.218 - 1.165)	0.563 (0.220 - 1.167)
Total number of cases (thousands)	1,982 (1,732 - 2,287)	3,184 (2,760 - 3,679)	4,112 (3,575 - 4,751)	180 (156 - 208)	272 (236 - 315)	336 (291 - 386)	13 (5 - 27)	21 (8 - 43)	27 (11 - 56)
**Costa Rica**									
15 - 49 years (rate per capita)	0.073 (0.062 - 0.087)	0.079 (0.067 - 0.094)	0.081 (0.068 - 0.096)	0.008 (0.007 - 0.009)	0.008 (0.007 - 0.010)	0.008 (0.007 - 0.010)	0.490 (0.185 - 1.035)	0.531 (0.203 - 1.115)	0.541 (0.207 - 1.138)
50 - 69 years (rate per capita)	0.203 (0.171 - 0.240)	0.203 (0.172 - 0.238)	0.203 (0.170 - 0.239)	0.012 (0.009 - 0.015)	0.012 (0.009 - 0.015)	0.012 (0.009 - 0.015)	1.333 (0.518 - 2.700)	1.330 (0.516 - 2.718)	1.326 (0.512 - 2.693)
70+ years (rate per capita)	0.155 (0.133 - 0.179)	0.153 (0.132 - 0.177)	0.152 (0.131 - 0.176)	0.008 (0.006 - 0.011)	0.008 (0.006 - 0.010)	0.008 (0.006 - 0.010)	0.971 (0.390 - 1.971)	0.960 (0.383 - 1.976)	0.954 (0.384 - 1.946)
All ages (rate per capita)	0.064 (0.056 - 0.074)	0.078 (0.068 - 0.090)	0.089 (0.077 - 0.103)	0.006 (0.005 - 0.006)	0.007 (0.006 - 0.007)	0.007 (0.006 - 0.008)	0.421 (0.163 - 0.875)	0.514 (0.196 - 1.068)	0.588 (0.227 - 1.231)
Age-standardized (rate per capita)	0.086 (0.075 - 0.099)	0.086 (0.075 - 0.099)	0.086 (0.075 - 0.099)	0.007 (0.006 - 0.008)	0.007 (0.006 - 0.008)	0.007 (0.006 - 0.008)	0.566 (0.219 - 1.179)	0.566 (0.218 - 1.175)	0.564 (0.218 - 1.182)
Total number of cases (thousands)	197 (172 - 228)	331 (287 - 382)	430 (372 - 496)	17 (15 - 20)	28 (24 - 32)	34 (30 - 39)	1 (1 - 3)	2 (1 - 5)	3 (1 - 6)
**El Salvador**									
15 - 49 years (rate per capita)	0.068 (0.058 - 0.080)	0.073 (0.062 - 0.086)	0.073 (0.061 - 0.087)	0.007 (0.006 - 0.009)	0.008 (0.007 - 0.009)	0.008 (0.007 - 0.009)	0.454 (0.172 - 0.942)	0.485 (0.183 - 1.019)	0.488 (0.185 - 1.020)
50 - 69 years (rate per capita)	0.203 (0.171 - 0.240)	0.201 (0.171 - 0.238)	0.201 (0.168 - 0.238)	0.012 (0.009 - 0.015)	0.012 (0.009 - 0.015)	0.012 (0.009 - 0.015)	1.323 (0.513 - 2.664)	1.309 (0.512 - 2.645)	1.305 (0.506 - 2.632)
70+ years (rate per capita)	0.156 (0.134 - 0.181)	0.155 (0.133 - 0.180)	0.152 (0.130 - 0.176)	0.008 (0.006 - 0.011)	0.008 (0.006 - 0.010)	0.008 (0.006 - 0.010)	0.975 (0.384 - 1.988)	0.966 (0.383 - 1.989)	0.947 (0.377 - 1.941)
All ages (rate per capita)	0.057 (0.050 - 0.065)	0.067 (0.059 - 0.077)	0.076 (0.067 - 0.088)	0.005 (0.004 - 0.006)	0.006 (0.005 - 0.006)	0.006 (0.006 - 0.007)	0.373 (0.144 - 0.770)	0.441 (0.170 - 0.915)	0.498 (0.193 - 1.030)
Age-standardized (rate per capita)	0.086 (0.075 - 0.098)	0.086 (0.075 - 0.098)	0.085 (0.074 - 0.099)	0.007 (0.006 - 0.008)	0.007 (0.006 - 0.008)	0.007 (0.006 - 0.008)	0.560 (0.219 - 1.163)	0.559 (0.217 - 1.162)	0.557 (0.215 - 1.152)
Total number of cases (thousands)	300 (262 - 346)	401 (351 - 461)	467 (408 - 541)	26 (23 - 30)	34 (30 - 39)	39 (34 - 45)	2 (1 - 4)	3 (1 - 5)	3 (1 - 6)
**Guatemala**									
15 - 49 years (rate per capita)	0.066 (0.056 - 0.078)	0.065 (0.055 - 0.077)	0.068 (0.058 - 0.081)	0.007 (0.006 - 0.009)	0.007 (0.006 - 0.009)	0.008 (0.006 - 0.009)	0.437 (0.165 - 0.902)	0.432 (0.165 - 0.901)	0.453 (0.170 - 0.949)
50 - 69 years (rate per capita)	0.202 (0.170 - 0.239)	0.201 (0.170 - 0.238)	0.200 (0.168 - 0.235)	0.012 (0.009 - 0.015)	0.012 (0.009 - 0.015)	0.012 (0.009 - 0.015)	1.313 (0.510 - 2.650)	1.301 (0.509 - 2.634)	1.294 (0.500 - 2.618)
70+ years (rate per capita)	0.155 (0.134 - 0.180)	0.153 (0.132 - 0.177)	0.150 (0.129 - 0.174)	0.008 (0.006 - 0.011)	0.008 (0.006 - 0.011)	0.008 (0.006 - 0.010)	0.965 (0.385 - 1.967)	0.950 (0.376 - 1.941)	0.930 (0.365 - 1.895)
All ages (rate per capita)	0.049 (0.043 - 0.057)	0.052 (0.046 - 0.060)	0.058 (0.051 - 0.067)	0.004 (0.004 - 0.005)	0.005 (0.004 - 0.005)	0.005 (0.005 - 0.006)	0.323 (0.124 - 0.671)	0.343 (0.133 - 0.705)	0.381 (0.148 - 0.783)
Age-standardized (rate per capita)	0.085 (0.075 - 0.098)	0.085 (0.075 - 0.098)	0.085 (0.074 - 0.098)	0.007 (0.006 - 0.008)	0.007 (0.006 - 0.008)	0.007 (0.006 - 0.008)	0.557 (0.217 - 1.158)	0.555 (0.217 - 1.147)	0.554 (0.214 - 1.146)
Total number of cases (thousands)	453 (395 - 524)	691 (605 - 796)	949 (831 - 1,097)	41 (36 - 47)	62 (55 - 71)	85 (74 - 98)	3 (1 - 6)	5 (2 - 9)	6 (2 - 13)
**Honduras**									
15 - 49 years (rate per capita)	0.063 (0.053 - 0.075)	0.067 (0.056 - 0.079)	0.070 (0.059 - 0.082)	0.007 (0.006 - 0.009)	0.007 (0.006 - 0.009)	0.008 (0.006 - 0.009)	0.423 (0.160 - 0.891)	0.446 (0.170 - 0.933)	0.465 (0.178 - 0.973)
50 - 69 years (rate per capita)	0.204 (0.172 - 0.240)	0.204 (0.173 - 0.240)	0.203 (0.171 - 0.241)	0.012 (0.009 - 0.015)	0.012 (0.009 - 0.016)	0.012 (0.009 - 0.015)	1.333 (0.520 - 2.719)	1.330 (0.515 - 2.683)	1.328 (0.513 - 2.700)
70+ years (rate per capita)	0.156 (0.134 - 0.181)	0.155 (0.132 - 0.179)	0.153 (0.132 - 0.177)	0.008 (0.006 - 0.011)	0.008 (0.006 - 0.010)	0.008 (0.006 - 0.010)	0.977 (0.392 - 2.031)	0.967 (0.388 - 1.968)	0.953 (0.377 - 1.967)
All ages (rate per capita)	0.047 (0.041 - 0.054)	0.054 (0.047 - 0.063)	0.064 (0.056 - 0.074)	0.004 (0.004 - 0.005)	0.005 (0.004 - 0.006)	0.006 (0.005 - 0.007)	0.311 (0.120 - 0.646)	0.357 (0.138 - 0.739)	0.423 (0.163 - 0.874)
Age-standardized (rate per capita)	0.086 (0.075 - 0.099)	0.086 (0.076 - 0.099)	0.086 (0.075 - 0.099)	0.007 (0.006 - 0.008)	0.007 (0.006 - 0.008)	0.007 (0.006 - 0.008)	0.565 (0.220 - 1.182)	0.565 (0.219 - 1.179)	0.564 (0.219 - 1.173)
Total number of cases (thousands)	232 (201 - 267)	373 (327 - 431)	519 (451 - 598)	21 (19 - 24)	34 (29 - 39)	46 (40 - 53)	2 (1 - 3)	2 (1 - 5)	3 (1 - 7)
**Mexico**									

	Prevalence 1990	Prevalence 2005	Prevalence 2015	Incidence 1990	Incidence 2005	Incidence 2015	YLD rate 1990	YLD rate 2005	YLD rate 2015
15 - 49 years (rate per capita)	0.067 (0.057 - 0.079)	0.077 (0.064 - 0.091)	0.082 (0.068 - 0.097)	0.008 (0.007 - 0.009)	0.008 (0.007 - 0.010)	0.009 (0.007 - 0.010)	0.449 (0.171 - 0.938)	0.513 (0.195 - 1.081)	0.545 (0.208 - 1.150)
50 - 69 years (rate per capita)	0.214 (0.181 - 0.251)	0.215 (0.182 - 0.254)	0.214 (0.180 - 0.251)	0.013 (0.010 - 0.016)	0.013 (0.010 - 0.016)	0.013 (0.010 - 0.016)	1.393 (0.542 - 2.817)	1.402 (0.546 - 2.851)	1.393 (0.542 - 2.819)
70+ years (rate per capita)	0.165 (0.143 - 0.189)	0.165 (0.142 - 0.190)	0.163 (0.141 - 0.188)	0.008 (0.006 - 0.011)	0.008 (0.006 - 0.011)	0.008 (0.006 - 0.011)	1.028 (0.412 - 2.120)	1.031 (0.412 - 2.123)	1.018 (0.408 - 2.094)
All ages (rate per capita)	0.057 (0.050 - 0.065)	0.070 (0.061 - 0.081)	0.081 (0.071 - 0.094)	0.005 (0.005 - 0.006)	0.006 (0.005 - 0.007)	0.007 (0.006 - 0.008)	0.374 (0.144 - 0.776)	0.462 (0.178 - 0.960)	0.532 (0.206 - 1.111)
Age-standardized (rate per capita)	0.090 (0.079 - 0.104)	0.090 (0.079 - 0.104)	0.090 (0.079 - 0.104)	0.007 (0.006 - 0.008)	0.007 (0.006 - 0.008)	0.007 (0.006 - 0.008)	0.591 (0.230 - 1.238)	0.593 (0.230 - 1.237)	0.591 (0.230 - 1.235)
Total number of cases (thousands)	4,857 (4,257 - 5,583)	7,695 (6,704 - 8,886)	10,271 (8,967 - 11,914)	443 (388 - 508)	674 (589 - 776)	866 (755 - 997)	32 (12 - 66)	51 (20 - 105)	68 (26 - 141)
**Nicaragua**									
15 - 49 years (rate per capita)	0.064 (0.054 - 0.075)	0.068 (0.058 - 0.080)	0.073 (0.062 - 0.087)	0.007 (0.006 - 0.009)	0.007 (0.006 - 0.009)	0.008 (0.007 - 0.009)	0.425 (0.162 - 0.890)	0.454 (0.175 - 0.947)	0.489 (0.187 - 1.017)
50 - 69 years (rate per capita)	0.202 (0.171 - 0.238)	0.201 (0.170 - 0.238)	0.202 (0.170 - 0.239)	0.012 (0.009 - 0.015)	0.012 (0.009 - 0.015)	0.012 (0.009 - 0.015)	1.321 (0.512 - 2.697)	1.316 (0.513 - 2.675)	1.319 (0.512 - 2.670)
70+ years (rate per capita)	0.155 (0.132 - 0.180)	0.153 (0.130 - 0.176)	0.151 (0.130 - 0.175)	0.008 (0.006 - 0.011)	0.008 (0.006 - 0.010)	0.008 (0.006 - 0.010)	0.975 (0.395 - 1.959)	0.958 (0.378 - 1.969)	0.946 (0.376 - 1.928)
All ages (rate per capita)	0.047 (0.040 - 0.054)	0.057 (0.050 - 0.066)	0.069 (0.060 - 0.080)	0.004 (0.004 - 0.005)	0.005 (0.005 - 0.006)	0.006 (0.005 - 0.007)	0.309 (0.119 - 0.642)	0.380 (0.147 - 0.782)	0.455 (0.176 - 0.952)
Age-standardized (rate per capita)	0.086 (0.075 - 0.098)	0.086 (0.075 - 0.099)	0.085 (0.075 - 0.098)	0.007 (0.006 - 0.008)	0.007 (0.006 - 0.008)	0.007 (0.006 - 0.008)	0.562 (0.219 - 1.169)	0.562 (0.218 - 1.172)	0.561 (0.219 - 1.177)
Total number of cases (thousands)	194 (168 - 223)	310 (270 - 358)	420 (367 - 485)	18 (16 - 21)	28 (25 - 32)	37 (32 - 42)	1 (0 - 3)	2 (1 - 4)	3 (1 - 6)
**Panama**									
15 - 49 years (rate per capita)	0.069 (0.058 - 0.082)	0.077 (0.065 - 0.092)	0.081 (0.068 - 0.096)	0.008 (0.006 - 0.009)	0.008 (0.007 - 0.010)	0.008 (0.007 - 0.010)	0.460 (0.176 - 0.967)	0.516 (0.197 - 1.074)	0.540 (0.204 - 1.116)
50 - 69 years (rate per capita)	0.203 (0.170 - 0.239)	0.203 (0.171 - 0.240)	0.203 (0.171 - 0.238)	0.012 (0.009 - 0.016)	0.012 (0.009 - 0.015)	0.012 (0.009 - 0.015)	1.328 (0.514 - 2.688)	1.326 (0.510 - 2.715)	1.321 (0.514 - 2.682)
70+ years (rate per capita)	0.155 (0.133 - 0.180)	0.153 (0.131 - 0.178)	0.152 (0.131 - 0.176)	0.008 (0.006 - 0.010)	0.008 (0.006 - 0.010)	0.008 (0.006 - 0.010)	0.971 (0.387 - 1.955)	0.958 (0.385 - 1.945)	0.946 (0.380 - 1.934)
All ages (rate per capita)	0.060 (0.052 - 0.069)	0.072 (0.063 - 0.084)	0.081 (0.070 - 0.094)	0.005 (0.005 - 0.006)	0.006 (0.005 - 0.007)	0.007 (0.006 - 0.008)	0.396 (0.153 - 0.815)	0.478 (0.183 - 0.999)	0.533 (0.207 - 1.101)
Age-standardized (rate per capita)	0.086 (0.075 - 0.099)	0.086 (0.075 - 0.099)	0.086 (0.075 - 0.099)	0.007 (0.006 - 0.008)	0.007 (0.006 - 0.008)	0.007 (0.006 - 0.008)	0.564 (0.221 - 1.182)	0.565 (0.219 - 1.182)	0.563 (0.219 - 1.160)
Total number of cases (thousands)	148 (129 - 171)	241 (209 - 279)	319 (276 - 368)	13 (12 - 15)	20 (18 - 24)	26 (23 - 30)	1 (0 - 2)	2 (1 - 3)	2 (1 - 4)
**Venezuela**									
15 - 49 years (rate per capita)	0.069 (0.058 - 0.082)	0.076 (0.064 - 0.090)	0.079 (0.066 - 0.093)	0.008 (0.006 - 0.009)	0.008 (0.007 - 0.010)	0.008 (0.007 - 0.010)	0.462 (0.174 - 0.971)	0.510 (0.194 - 1.069)	0.526 (0.200 - 1.110)
50 - 69 years (rate per capita)	0.204 (0.172 - 0.241)	0.204 (0.171 - 0.242)	0.204 (0.171 - 0.241)	0.012 (0.009 - 0.015)	0.012 (0.009 - 0.015)	0.012 (0.009 - 0.015)	1.337 (0.522 - 2.703)	1.333 (0.518 - 2.730)	1.330 (0.511 - 2.697)
70+ years (rate per capita)	0.157 (0.135 - 0.183)	0.154 (0.133 - 0.180)	0.154 (0.132 - 0.178)	0.008 (0.006 - 0.011)	0.008 (0.006 - 0.010)	0.008 (0.006 - 0.010)	0.983 (0.396 - 2.026)	0.967 (0.387 - 1.954)	0.962 (0.384 - 1.927)
All ages (rate per capita)	0.057 (0.050 - 0.066)	0.069 (0.060 - 0.080)	0.078 (0.068 - 0.090)	0.005 (0.004 - 0.006)	0.006 (0.005 - 0.007)	0.006 (0.006 - 0.007)	0.379 (0.146 - 0.789)	0.457 (0.176 - 0.951)	0.516 (0.198 - 1.085)
Age-standardized (rate per capita)	0.086 (0.075 - 0.099)	0.086 (0.075 - 0.100)	0.086 (0.075 - 0.099)	0.007 (0.006 - 0.008)	0.007 (0.006 - 0.008)	0.007 (0.006 - 0.008)	0.568 (0.222 - 1.183)	0.567 (0.220 - 1.174)	0.566 (0.219 - 1.196)
Total number of cases (thousands)	1,137 (989 - 1,311)	1,854 (1,606 - 2,152)	2,438 (2,115 - 2,812)	103 (89 - 118)	160 (139 - 186)	202 (176 - 232)	8 (3 - 16)	12 (5 - 25)	16 (6 - 34)
**Andean Latin America**									
15 - 49 years (rate per capita)	0.070 (0.059 - 0.083)	0.075 (0.064 - 0.089)	0.079 (0.066 - 0.094)	0.008 (0.007 - 0.009)	0.008 (0.007 - 0.010)	0.008 (0.007 - 0.010)	0.467 (0.179 - 0.981)	0.503 (0.191 - 1.051)	0.530 (0.201 - 1.105)
50 - 69 years (rate per capita)	0.208 (0.175 - 0.246)	0.208 (0.175 - 0.245)	0.208 (0.176 - 0.244)	0.013 (0.009 - 0.016)	0.013 (0.009 - 0.016)	0.013 (0.009 - 0.016)	1.357 (0.523 - 2.740)	1.356 (0.528 - 2.753)	1.354 (0.529 - 2.745)
70+ years (rate per capita)	0.157 (0.135 - 0.182)	0.155 (0.133 - 0.179)	0.154 (0.132 - 0.177)	0.008 (0.006 - 0.011)	0.008 (0.006 - 0.011)	0.008 (0.006 - 0.010)	0.982 (0.394 - 2.016)	0.968 (0.389 - 1.977)	0.959 (0.386 - 1.951)
All ages (rate per capita)	0.058 (0.051 - 0.067)	0.068 (0.060 - 0.079)	0.077 (0.067 - 0.089)	0.005 (0.005 - 0.006)	0.006 (0.005 - 0.007)	0.007 (0.006 - 0.007)	0.382 (0.148 - 0.790)	0.450 (0.175 - 0.934)	0.507 (0.195 - 1.050)
Age-standardized (rate per capita)	0.088 (0.077 - 0.102)	0.088 (0.077 - 0.102)	0.088 (0.077 - 0.102)	0.007 (0.006 - 0.008)	0.007 (0.006 - 0.008)	0.007 (0.006 - 0.008)	0.578 (0.225 - 1.207)	0.578 (0.226 - 1.205)	0.578 (0.224 - 1.197)
Total number of cases (thousands)	2,258 (1,970 - 2,607)	3,458 (3,026 - 3,984)	4,496 (3,921 - 5,200)	204 (178 - 235)	302 (264 - 346)	380 (333 - 436)	15 (6 - 31)	23 (9 - 47)	30 (11 - 61)
**Bolivia**									
15 - 49 years (rate per capita)	0.071 (0.060 - 0.084)	0.072 (0.061 - 0.086)	0.075 (0.063 - 0.089)	0.008 (0.007 - 0.009)	0.008 (0.007 - 0.009)	0.008 (0.007 - 0.010)	0.472 (0.180 - 0.984)	0.481 (0.184 - 1.007)	0.501 (0.189 - 1.035)
50 - 69 years (rate per capita)	0.209 (0.177 - 0.247)	0.209 (0.177 - 0.247)	0.208 (0.176 - 0.245)	0.013 (0.009 - 0.016)	0.013 (0.009 - 0.016)	0.013 (0.010 - 0.016)	1.361 (0.531 - 2.798)	1.365 (0.537 - 2.760)	1.358 (0.525 - 2.737)
70+ years (rate per capita)	0.159 (0.136 - 0.186)	0.156 (0.134 - 0.182)	0.154 (0.132 - 0.179)	0.009 (0.006 - 0.011)	0.008 (0.006 - 0.011)	0.008 (0.006 - 0.010)	0.993 (0.394 - 2.030)	0.972 (0.390 - 1.964)	0.956 (0.378 - 1.946)
All ages (rate per capita)	0.057 (0.049 - 0.066)	0.063 (0.055 - 0.073)	0.070 (0.061 - 0.080)	0.005 (0.004 - 0.006)	0.006 (0.005 - 0.006)	0.006 (0.005 - 0.007)	0.373 (0.145 - 0.774)	0.413 (0.161 - 0.859)	0.457 (0.178 - 0.938)
Age-standardized (rate per capita)	0.089 (0.078 - 0.102)	0.089 (0.077 - 0.102)	0.088 (0.077 - 0.102)	0.007 (0.006 - 0.008)	0.007 (0.006 - 0.008)	0.007 (0.006 - 0.008)	0.580 (0.226 - 1.207)	0.581 (0.227 - 1.211)	0.579 (0.225 - 1.193)
Total number of cases (thousands)	391 (341 - 452)	576 (503 - 665)	750 (658 - 863)	35 (30 - 40)	51 (44 - 58)	65 (56 - 74)	3 (1 - 5)	4 (1 - 8)	5 (2 - 10)
**Ecuador**									
15 - 49 years (rate per capita)	0.070 (0.059 - 0.082)	0.076 (0.064 - 0.090)	0.079 (0.066 - 0.094)	0.008 (0.007 - 0.009)	0.008 (0.007 - 0.010)	0.008 (0.007 - 0.010)	0.466 (0.179 - 0.967)	0.509 (0.195 - 1.060)	0.529 (0.200 - 1.095)
50 - 69 years (rate per capita)	0.208 (0.175 - 0.245)	0.207 (0.175 - 0.245)	0.208 (0.175 - 0.245)	0.013 (0.009 - 0.016)	0.013 (0.009 - 0.016)	0.013 (0.010 - 0.016)	1.352 (0.524 - 2.745)	1.352 (0.524 - 2.752)	1.353 (0.525 - 2.752)
70+ years (rate per capita)	0.156 (0.134 - 0.180)	0.154 (0.132 - 0.177)	0.154 (0.133 - 0.178)	0.008 (0.006 - 0.011)	0.008 (0.006 - 0.010)	0.008 (0.006 - 0.010)	0.971 (0.383 - 1.944)	0.956 (0.386 - 1.952)	0.956 (0.381 - 1.944)
All ages (rate per capita)	0.058 (0.050 - 0.067)	0.069 (0.060 - 0.080)	0.078 (0.067 - 0.090)	0.005 (0.005 - 0.006)	0.006 (0.005 - 0.007)	0.007 (0.006 - 0.008)	0.382 (0.148 - 0.792)	0.454 (0.177 - 0.943)	0.509 (0.196 - 1.058)
Age-standardized (rate per capita)	0.088 (0.077 - 0.101)	0.088 (0.077 - 0.102)	0.088 (0.077 - 0.102)	0.007 (0.006 - 0.008)	0.007 (0.006 - 0.008)	0.007 (0.006 - 0.008)	0.576 (0.223 - 1.193)	0.577 (0.225 - 1.205)	0.576 (0.223 - 1.197)
Total number of cases (thousands)	594 (517 - 684)	950 (830 - 1,098)	1,252 (1,090 - 1,450)	54 (47 - 62)	83 (72 - 95)	106 (92 - 122)	4 (2 - 8)	6 (2 - 13)	8 (3 - 17)
**Peru**									
15 - 49 years (rate per capita)	0.070 (0.059 - 0.083)	0.076 (0.064 - 0.090)	0.081 (0.067 - 0.096)	0.008 (0.007 - 0.009)	0.008 (0.007 - 0.010)	0.008 (0.007 - 0.010)	0.466 (0.177 - 0.987)	0.507 (0.191 - 1.066)	0.540 (0.203 - 1.122)
50 - 69 years (rate per capita)	0.208 (0.175 - 0.246)	0.208 (0.174 - 0.245)	0.207 (0.175 - 0.244)	0.013 (0.009 - 0.016)	0.013 (0.009 - 0.016)	0.013 (0.009 - 0.016)	1.359 (0.524 - 2.743)	1.356 (0.524 - 2.758)	1.354 (0.529 - 2.758)
70+ years (rate per capita)	0.157 (0.134 - 0.183)	0.155 (0.133 - 0.180)	0.154 (0.132 - 0.178)	0.008 (0.006 - 0.011)	0.008 (0.006 - 0.011)	0.008 (0.006 - 0.010)	0.985 (0.392 - 2.016)	0.972 (0.390 - 1.972)	0.962 (0.386 - 1.959)
All ages (rate per capita)	0.058 (0.051 - 0.067)	0.070 (0.061 - 0.081)	0.079 (0.069 - 0.092)	0.005 (0.005 - 0.006)	0.006 (0.005 - 0.007)	0.007 (0.006 - 0.008)	0.384 (0.149 - 0.798)	0.461 (0.179 - 0.958)	0.524 (0.201 - 1.083)
Age-standardized (rate per capita)	0.088 (0.077 - 0.102)	0.088 (0.077 - 0.101)	0.088 (0.077 - 0.102)	0.007 (0.006 - 0.008)	0.007 (0.006 - 0.008)	0.007 (0.006 - 0.008)	0.578 (0.225 - 1.212)	0.578 (0.224 - 1.201)	0.578 (0.223 - 1.196)
Total number of cases (thousands)	1,272 (1,106 - 1,474)	1,933 (1,683 - 2,227)	2,494 (2,166 - 2,892)	115 (100 - 133)	169 (147 - 194)	210 (184 - 242)	8 (3 - 17)	13 (5 - 26)	16 (6 - 34)
**Caribbean**									
15 - 49 years (rate per capita)	0.071 (0.060 - 0.084)	0.079 (0.067 - 0.094)	0.081 (0.068 - 0.096)	0.008 (0.007 - 0.009)	0.008 (0.007 - 0.010)	0.008 (0.007 - 0.010)	0.471 (0.180 - 0.979)	0.529 (0.203 - 1.106)	0.540 (0.206 - 1.130)
50 - 69 years (rate per capita)	0.208 (0.175 - 0.245)	0.207 (0.176 - 0.244)	0.207 (0.175 - 0.244)	0.012 (0.009 - 0.015)	0.012 (0.009 - 0.015)	0.012 (0.009 - 0.015)	1.353 (0.523 - 2.746)	1.349 (0.523 - 2.735)	1.348 (0.522 - 2.735)
70+ years (rate per capita)	0.163 (0.141 - 0.189)	0.162 (0.140 - 0.186)	0.161 (0.138 - 0.186)	0.008 (0.006 - 0.010)	0.008 (0.006 - 0.010)	0.008 (0.006 - 0.010)	1.024 (0.410 - 2.098)	1.010 (0.406 - 2.079)	1.003 (0.403 - 2.058)
All ages (rate per capita)	0.068 (0.059 - 0.078)	0.079 (0.069 - 0.091)	0.088 (0.077 - 0.101)	0.006 (0.005 - 0.007)	0.006 (0.006 - 0.007)	0.007 (0.006 - 0.008)	0.445 (0.173 - 0.921)	0.519 (0.201 - 1.084)	0.573 (0.222 - 1.195)
Age-standardized (rate per capita)	0.088 (0.077 - 0.101)	0.088 (0.077 - 0.101)	0.087 (0.076 - 0.101)	0.007 (0.006 - 0.008)	0.007 (0.006 - 0.008)	0.007 (0.006 - 0.008)	0.573 (0.224 - 1.190)	0.573 (0.223 - 1.198)	0.572 (0.223 - 1.188)
Total number of cases (thousands)	2,425 (2,122 - 2,795)	3,308 (2,885 - 3,822)	3,963 (3,465 - 4,580)	203 (179 - 233)	266 (233 - 306)	308 (270 - 353)	16 (6 - 33)	22 (8 - 45)	26 (10 - 54)
**Antigua and Barbuda**									
15 - 49 years (rate per capita)	0.071 (0.060 - 0.085)	0.084 (0.071 - 0.101)	0.086 (0.073 - 0.102)	0.008 (0.007 - 0.009)	0.008 (0.007 - 0.010)	0.008 (0.007 - 0.010)	0.476 (0.182 - 0.986)	0.563 (0.213 - 1.182)	0.573 (0.218 - 1.215)
50 - 69 years (rate per capita)	0.206 (0.176 - 0.242)	0.206 (0.174 - 0.243)	0.207 (0.174 - 0.246)	0.012 (0.009 - 0.015)	0.012 (0.009 - 0.015)	0.012 (0.009 - 0.015)	1.341 (0.518 - 2.728)	1.340 (0.522 - 2.711)	1.348 (0.523 - 2.730)
70+ years (rate per capita)	0.163 (0.140 - 0.188)	0.158 (0.137 - 0.183)	0.160 (0.138 - 0.185)	0.008 (0.006 - 0.010)	0.008 (0.006 - 0.010)	0.008 (0.006 - 0.010)	1.022 (0.401 - 2.092)	0.987 (0.400 - 2.022)	1.000 (0.404 - 2.050)
All ages (rate per capita)	0.071 (0.063 - 0.082)	0.079 (0.069 - 0.091)	0.090 (0.078 - 0.104)	0.006 (0.005 - 0.007)	0.006 (0.006 - 0.007)	0.007 (0.006 - 0.008)	0.468 (0.182 - 0.970)	0.519 (0.200 - 1.078)	0.589 (0.227 - 1.247)
Age-standardized (rate per capita)	0.087 (0.077 - 0.100)	0.087 (0.076 - 0.100)	0.087 (0.076 - 0.100)	0.007 (0.006 - 0.008)	0.007 (0.006 - 0.008)	0.007 (0.006 - 0.008)	0.572 (0.222 - 1.187)	0.570 (0.221 - 1.187)	0.570 (0.223 - 1.189)
Total number of cases (thousands)	4 (4 - 5)	7 (6 - 8)	8 (7 - 10)	0 (0 - 0)	1 (0 - 1)	1 (1 - 1)	0 (0 - 0)	0 (0 - 0)	0 (0 - 0)
**The Bahamas**									
15 - 49 years (rate per capita)	0.070 (0.059 - 0.084)	0.082 (0.069 - 0.098)	0.083 (0.069 - 0.099)	0.008 (0.006 - 0.009)	0.008 (0.007 - 0.010)	0.008 (0.007 - 0.010)	0.468 (0.179 - 0.974)	0.547 (0.208 - 1.149)	0.552 (0.210 - 1.146)
50 - 69 years (rate per capita)	0.206 (0.174 - 0.244)	0.207 (0.174 - 0.244)	0.206 (0.174 - 0.243)	0.012 (0.009 - 0.015)	0.012 (0.009 - 0.015)	0.012 (0.009 - 0.015)	1.340 (0.519 - 2.711)	1.344 (0.514 - 2.738)	1.342 (0.519 - 2.711)
70+ years (rate per capita)	0.163 (0.141 - 0.189)	0.162 (0.139 - 0.188)	0.162 (0.140 - 0.188)	0.008 (0.006 - 0.010)	0.008 (0.006 - 0.010)	0.008 (0.006 - 0.010)	1.020 (0.410 - 2.074)	1.011 (0.402 - 2.072)	1.007 (0.405 - 2.062)
All ages (rate per capita)	0.064 (0.056 - 0.074)	0.084 (0.072 - 0.097)	0.094 (0.082 - 0.109)	0.006 (0.005 - 0.006)	0.007 (0.006 - 0.008)	0.007 (0.006 - 0.008)	0.419 (0.162 - 0.863)	0.548 (0.210 - 1.145)	0.616 (0.238 - 1.286)

	Prevalence 1990	Prevalence 2005	Prevalence 2015	Incidence 1990	Incidence 2005	Incidence 2015	YLD rate 1990	YLD rate 2005	YLD rate 2015
**Age-standardized (rate per capita)**	0.087 (0.076 - 0.100)	0.087 (0.076 - 0.101)	0.087 (0.076 - 0.100)	0.007 (0.006 - 0.008)	0.007 (0.006 - 0.008)	0.007 (0.006 - 0.008)	0.568 (0.220 - 1.192)	0.570 (0.220 - 1.187)	0.569 (0.221 - 1.183)
**Total number of cases (thousands)**	16 (14 - 19)	27 (24 - 32)	36 (32 - 42)	1 (1 - 2)	2 (2 - 3)	3 (2 - 3)	0 (0 - 0)	0 (0 - 0)	0 (0 - 0)
**Barbados**									
**15 - 49 years (rate per capita)**	0.073 (0.061 - 0.086)	0.089 (0.075 - 0.107)	0.089 (0.076 - 0.106)	0.008 (0.007 - 0.009)	0.009 (0.007 - 0.011)	0.009 (0.007 - 0.011)	0.485 (0.185 - 1.008)	0.595 (0.224 - 1.241)	0.592 (0.222 - 1.243)
**50 - 69 years (rate per capita)**	0.205 (0.173 - 0.243)	0.206 (0.174 - 0.244)	0.206 (0.174 - 0.243)	0.012 (0.009 - 0.015)	0.012 (0.009 - 0.015)	0.012 (0.009 - 0.015)	1.332 (0.520 - 2.724)	1.337 (0.516 - 2.715)	1.335 (0.517 - 2.702)
**70+ years (rate per capita)**	0.161 (0.138 - 0.186)	0.159 (0.136 - 0.183)	0.159 (0.137 - 0.185)	0.008 (0.006 - 0.010)	0.008 (0.006 - 0.010)	0.008 (0.006 - 0.010)	1.003 (0.400 - 2.060)	0.988 (0.398 - 2.035)	0.988 (0.395 - 2.026)
**All ages (rate per capita)**	0.079 (0.069 - 0.090)	0.098 (0.086 - 0.114)	0.107 (0.093 - 0.124)	0.006 (0.006 - 0.007)	0.007 (0.006 - 0.009)	0.008 (0.007 - 0.009)	0.515 (0.200 - 1.075)	0.643 (0.249 - 1.338)	0.698 (0.271 - 1.447)
**Age-standardized (rate per capita)**	0.087 (0.076 - 0.100)	0.087 (0.076 - 0.100)	0.087 (0.076 - 0.100)	0.007 (0.006 - 0.008)	0.007 (0.006 - 0.008)	0.007 (0.006 - 0.008)	0.568 (0.220 - 1.195)	0.569 (0.221 - 1.180)	0.568 (0.219 - 1.175)
**Total number of cases (thousands)**	21 (18 - 24)	27 (24 - 31)	30 (27 - 35)	2 (1 - 2)	2 (2 - 2)	2 (2 - 3)	0 (0 - 0)	0 (0 - 0)	0 (0 - 0)
**Belize**									
**15 - 49 years (rate per capita)**	0.061 (0.052 - 0.073)	0.070 (0.058 - 0.083)	0.073 (0.061 - 0.086)	0.007 (0.006 - 0.008)	0.008 (0.006 - 0.009)	0.008 (0.007 - 0.009)	0.409 (0.156 - 0.862)	0.467 (0.176 - 0.959)	0.486 (0.183 - 1.015)
**50 - 69 years (rate per capita)**	0.208 (0.176 - 0.246)	0.207 (0.175 - 0.245)	0.208 (0.175 - 0.245)	0.012 (0.009 - 0.015)	0.012 (0.009 - 0.015)	0.012 (0.009 - 0.015)	1.358 (0.531 - 2.771)	1.347 (0.516 - 2.747)	1.353 (0.523 - 2.744)
**70+ years (rate per capita)**	0.163 (0.140 - 0.187)	0.165 (0.142 - 0.192)	0.165 (0.141 - 0.191)	0.008 (0.006 - 0.010)	0.008 (0.006 - 0.011)	0.008 (0.006 - 0.010)	1.022 (0.407 - 2.111)	1.035 (0.411 - 2.114)	1.031 (0.415 - 2.116)
**All ages (rate per capita)**	0.049 (0.043 - 0.056)	0.055 (0.047 - 0.063)	0.066 (0.057 - 0.077)	0.004 (0.004 - 0.005)	0.005 (0.004 - 0.006)	0.006 (0.005 - 0.007)	0.321 (0.125 - 0.676)	0.360 (0.138 - 0.739)	0.435 (0.168 - 0.912)
**Age-standardized (rate per capita)**	0.088 (0.077 - 0.101)	0.088 (0.077 - 0.101)	0.088 (0.077 - 0.101)	0.007 (0.006 - 0.008)	0.007 (0.006 - 0.008)	0.007 (0.006 - 0.008)	0.574 (0.224 - 1.202)	0.573 (0.222 - 1.185)	0.572 (0.223 - 1.193)
**Total number of cases (thousands)**	9 (8 - 11)	15 (13 - 18)	24 (21 - 27)	1 (1 - 1)	1 (1 - 2)	2 (2 - 2)	0 (0 - 0)	0 (0 - 0)	0 (0 - 0)
**Bermuda**									
**15 - 49 years (rate per capita)**	0.071 (0.060 - 0.084)	0.079 (0.067 - 0.094)	0.081 (0.068 - 0.096)	0.008 (0.006 - 0.009)	0.008 (0.007 - 0.010)	0.008 (0.007 - 0.010)	0.471 (0.180 - 0.997)	0.529 (0.201 - 1.114)	0.541 (0.203 - 1.147)
**50 - 69 years (rate per capita)**	0.207 (0.175 - 0.244)	0.207 (0.175 - 0.244)	0.207 (0.174 - 0.244)	0.012 (0.009 - 0.015)	0.012 (0.009 - 0.015)	0.012 (0.009 - 0.015)	1.351 (0.525 - 2.738)	1.351 (0.520 - 2.754)	1.351 (0.527 - 2.736)
**70+ years (rate per capita)**	0.163 (0.141 - 0.189)	0.162 (0.139 - 0.186)	0.161 (0.138 - 0.186)	0.008 (0.006 - 0.010)	0.008 (0.006 - 0.010)	0.008 (0.006 - 0.010)	1.024 (0.418 - 2.112)	1.011 (0.405 - 2.064)	1.007 (0.409 - 2.062)
**All ages (rate per capita)**	0.071 (0.062 - 0.081)	0.081 (0.071 - 0.094)	0.090 (0.078 - 0.104)	0.006 (0.005 - 0.007)	0.007 (0.006 - 0.008)	0.007 (0.006 - 0.008)	0.465 (0.181 - 0.969)	0.535 (0.206 - 1.116)	0.589 (0.230 - 1.220)
**Age-standardized (rate per capita)**	0.087 (0.077 - 0.101)	0.088 (0.076 - 0.101)	0.087 (0.076 - 0.101)	0.007 (0.006 - 0.008)	0.007 (0.006 - 0.008)	0.007 (0.006 - 0.008)	0.573 (0.223 - 1.193)	0.574 (0.221 - 1.199)	0.573 (0.224 - 1.182)
**Total number of cases (thousands)**	4 (3 - 4)	5 (4 - 6)	6 (5 - 7)	0 (0 - 0)	0 (0 - 0)	0 (0 - 1)	0 (0 - 0)	0 (0 - 0)	0 (0 - 0)
**Cuba**									
**15 - 49 years (rate per capita)**	0.073 (0.061 - 0.086)	0.090 (0.076 - 0.108)	0.095 (0.080 - 0.114)	0.008 (0.007 - 0.009)	0.009 (0.007 - 0.011)	0.009 (0.007 - 0.011)	0.484 (0.183 - 1.017)	0.603 (0.229 - 1.270)	0.636 (0.239 - 1.334)
**50 - 69 years (rate per capita)**	0.208 (0.176 - 0.245)	0.208 (0.175 - 0.245)	0.208 (0.174 - 0.245)	0.012 (0.009 - 0.015)	0.012 (0.009 - 0.015)	0.012 (0.009 - 0.015)	1.360 (0.523 - 2.753)	1.355 (0.523 - 2.765)	1.355 (0.525 - 2.745)
**70+ years (rate per capita)**	0.163 (0.139 - 0.189)	0.161 (0.139 - 0.186)	0.161 (0.139 - 0.186)	0.008 (0.006 - 0.010)	0.008 (0.006 - 0.010)	0.008 (0.006 - 0.010)	1.022 (0.415 - 2.097)	1.010 (0.407 - 2.112)	1.006 (0.405 - 2.061)
**All ages (rate per capita)**	0.081 (0.071 - 0.093)	0.100 (0.087 - 0.115)	0.114 (0.099 - 0.131)	0.007 (0.006 - 0.008)	0.008 (0.007 - 0.009)	0.008 (0.007 - 0.010)	0.531 (0.206 - 1.109)	0.655 (0.255 - 1.366)	0.744 (0.286 - 1.541)
**Age-standardized (rate per capita)**	0.088 (0.077 - 0.101)	0.088 (0.077 - 0.102)	0.088 (0.077 - 0.101)	0.007 (0.006 - 0.008)	0.007 (0.006 - 0.008)	0.007 (0.006 - 0.008)	0.577 (0.225 - 1.197)	0.576 (0.224 - 1.203)	0.576 (0.223 - 1.182)
**Total number of cases (thousands)**	854 (750 - 980)	1,124 (981 - 1,301)	1,295 (1,125 - 1,497)	70 (61 - 80)	86 (75 - 100)	94 (81 - 109)	6 (2 - 12)	7 (3 - 15)	8 (3 - 18)
**Dominica**									
**15 - 49 years (rate per capita)**	0.071 (0.060 - 0.084)	0.079 (0.067 - 0.095)	0.081 (0.068 - 0.096)	0.008 (0.006 - 0.009)	0.008 (0.007 - 0.010)	0.008 (0.007 - 0.010)	0.470 (0.178 - 0.986)	0.528 (0.199 - 1.098)	0.538 (0.202 - 1.127)
**50 - 69 years (rate per capita)**	0.207 (0.175 - 0.244)	0.207 (0.175 - 0.244)	0.207 (0.174 - 0.243)	0.012 (0.009 - 0.015)	0.012 (0.009 - 0.015)	0.012 (0.009 - 0.015)	1.346 (0.523 - 2.723)	1.344 (0.524 - 2.708)	1.339 (0.522 - 2.725)
**70+ years (rate per capita)**	0.163 (0.141 - 0.188)	0.162 (0.139 - 0.188)	0.161 (0.138 - 0.186)	0.008 (0.006 - 0.010)	0.008 (0.006 - 0.010)	0.008 (0.006 - 0.010)	1.019 (0.409 - 2.090)	1.006 (0.397 - 2.049)	0.997 (0.402 - 2.029)
**All ages (rate per capita)**	0.068 (0.059 - 0.078)	0.080 (0.070 - 0.092)	0.088 (0.076 - 0.101)	0.006 (0.005 - 0.006)	0.006 (0.006 - 0.007)	0.007 (0.006 - 0.008)	0.443 (0.172 - 0.921)	0.523 (0.203 - 1.095)	0.572 (0.220 - 1.192)
**Age-standardized (rate per capita)**	0.087 (0.077 - 0.101)	0.087 (0.077 - 0.101)	0.087 (0.076 - 0.100)	0.007 (0.006 - 0.008)	0.007 (0.006 - 0.008)	0.007 (0.006 - 0.008)	0.571 (0.223 - 1.201)	0.571 (0.222 - 1.196)	0.569 (0.219 - 1.186)
**Total number of cases (thousands)**	5 (4 - 5)	6 (5 - 6)	6 (5 - 7)	0 (0 - 0)	0 (0 - 1)	0 (0 - 1)	0 (0 - 0)	0 (0 - 0)	0 (0 - 0)
**Dominican Republic**									
**15 - 49 years (rate per capita)**	0.067 (0.056 - 0.080)	0.074 (0.062 - 0.088)	0.077 (0.065 - 0.091)	0.007 (0.006 - 0.009)	0.008 (0.007 - 0.009)	0.008 (0.007 - 0.010)	0.449 (0.172 - 0.928)	0.495 (0.188 - 1.029)	0.512 (0.193 - 1.075)
**50 - 69 years (rate per capita)**	0.210 (0.176 - 0.247)	0.208 (0.176 - 0.245)	0.208 (0.175 - 0.244)	0.012 (0.009 - 0.015)	0.012 (0.009 - 0.015)	0.012 (0.009 - 0.015)	1.371 (0.532 - 2.792)	1.357 (0.525 - 2.771)	1.356 (0.521 - 2.754)
**70+ years (rate per capita)**	0.167 (0.144 - 0.194)	0.163 (0.142 - 0.189)	0.161 (0.139 - 0.185)	0.008 (0.006 - 0.011)	0.008 (0.006 - 0.010)	0.008 (0.006 - 0.010)	1.054 (0.414 - 2.128)	1.025 (0.401 - 2.098)	1.008 (0.404 - 2.071)
**All ages (rate per capita)**	0.057 (0.050 - 0.066)	0.068 (0.059 - 0.078)	0.076 (0.066 - 0.088)	0.005 (0.004 - 0.006)	0.006 (0.005 - 0.007)	0.006 (0.005 - 0.007)	0.378 (0.147 - 0.783)	0.446 (0.171 - 0.927)	0.498 (0.192 - 1.037)
**Age-standardized (rate per capita)**	0.088 (0.077 - 0.102)	0.088 (0.077 - 0.101)	0.088 (0.076 - 0.101)	0.007 (0.006 - 0.008)	0.007 (0.006 - 0.008)	0.007 (0.006 - 0.008)	0.579 (0.224 - 1.198)	0.576 (0.222 - 1.195)	0.574 (0.223 - 1.200)
**Total number of cases (thousands)**	412 (359 - 476)	626 (545 - 722)	797 (696 - 922)	36 (32 - 42)	53 (46 - 61)	65 (57 - 75)	3 (1 - 6)	4 (2 - 9)	5 (2 - 11)
**Grenada**									
**15 - 49 years (rate per capita)**	0.066 (0.055 - 0.078)	0.069 (0.058 - 0.082)	0.072 (0.061 - 0.086)	0.007 (0.006 - 0.009)	0.007 (0.006 - 0.009)	0.008 (0.007 - 0.009)	0.438 (0.165 - 0.900)	0.458 (0.173 - 0.957)	0.481 (0.186 - 1.002)
**50 - 69 years (rate per capita)**	0.206 (0.174 - 0.242)	0.206 (0.174 - 0.243)	0.207 (0.174 - 0.246)	0.012 (0.009 - 0.015)	0.012 (0.009 - 0.015)	0.012 (0.009 - 0.015)	1.334 (0.522 - 2.713)	1.339 (0.520 - 2.726)	1.343 (0.526 - 2.753)
**70+ years (rate per capita)**	0.164 (0.141 - 0.189)	0.160 (0.137 - 0.184)	0.159 (0.137 - 0.184)	0.008 (0.006 - 0.011)	0.008 (0.006 - 0.010)	0.008 (0.006 - 0.010)	1.028 (0.403 - 2.109)	0.993 (0.395 - 2.024)	0.985 (0.395 - 2.006)
**All ages (rate per capita)**	0.062 (0.055 - 0.072)	0.068 (0.059 - 0.078)	0.077 (0.067 - 0.089)	0.005 (0.004 - 0.006)	0.006 (0.005 - 0.007)	0.006 (0.006 - 0.007)	0.407 (0.158 - 0.841)	0.443 (0.173 - 0.927)	0.503 (0.195 - 1.034)
**Age-standardized (rate per capita)**	0.087 (0.076 - 0.100)	0.087 (0.076 - 0.101)	0.087 (0.076 - 0.101)	0.007 (0.006 - 0.008)	0.007 (0.006 - 0.008)	0.007 (0.006 - 0.008)	0.568 (0.220 - 1.168)	0.571 (0.223 - 1.200)	0.570 (0.222 - 1.182)
**Total number of cases (thousands)**	6 (5 - 7)	7 (6 - 8)	8 (7 - 9)	0 (0 - 1)	1 (1 - 1)	1 (1 - 1)	0 (0 - 0)	0 (0 - 0)	0 (0 - 0)
**Guyana**									
**15 - 49 years (rate per capita)**	0.068 (0.057 - 0.080)	0.084 (0.070 - 0.099)	0.077 (0.065 - 0.091)	0.007 (0.006 - 0.009)	0.008 (0.007 - 0.010)	0.008 (0.007 - 0.009)	0.450 (0.173 - 0.942)	0.558 (0.212 - 1.177)	0.508 (0.194 - 1.064)
**50 - 69 years (rate per capita)**	0.207 (0.175 - 0.244)	0.207 (0.174 - 0.245)	0.208 (0.174 - 0.245)	0.012 (0.009 - 0.015)	0.012 (0.009 - 0.015)	0.012 (0.009 - 0.016)	1.343 (0.522 - 2.716)	1.343 (0.520 - 2.726)	1.346 (0.520 - 2.724)
**70+ years (rate per capita)**	0.162 (0.139 - 0.187)	0.162 (0.140 - 0.188)	0.166 (0.142 - 0.193)	0.008 (0.006 - 0.010)	0.008 (0.006 - 0.010)	0.008 (0.006 - 0.011)	1.008 (0.405 - 2.080)	1.011 (0.402 - 2.075)	1.036 (0.406 - 2.152)
**All ages (rate per capita)**	0.056 (0.049 - 0.065)	0.068 (0.059 - 0.080)	0.079 (0.069 - 0.092)	0.005 (0.004 - 0.006)	0.006 (0.005 - 0.007)	0.006 (0.005 - 0.007)	0.370 (0.142 - 0.772)	0.449 (0.175 - 0.939)	0.516 (0.196 - 1.065)
**Age-standardized (rate per capita)**	0.087 (0.077 - 0.100)	0.088 (0.077 - 0.101)	0.087 (0.077 - 0.101)	0.007 (0.006 - 0.008)	0.007 (0.006 - 0.008)	0.007 (0.006 - 0.008)	0.570 (0.221 - 1.185)	0.571 (0.223 - 1.192)	0.570 (0.222 - 1.184)
**Total number of cases (thousands)**	42 (37 - 48)	51 (44 - 59)	61 (53 - 71)	4 (3 - 4)	4 (4 - 5)	5 (4 - 6)	0 (0 - 1)	0 (0 - 1)	0 (0 - 1)
**Haiti**									
**15 - 49 years (rate per capita)**	0.068 (0.058 - 0.081)	0.067 (0.057 - 0.080)	0.070 (0.059 - 0.083)	0.007 (0.006 - 0.009)	0.007 (0.006 - 0.009)	0.008 (0.006 - 0.009)	0.452 (0.173 - 0.936)	0.447 (0.171 - 0.925)	0.465 (0.179 - 0.981)
**50 - 69 years (rate per capita)**	0.207 (0.174 - 0.245)	0.207 (0.176 - 0.244)	0.207 (0.174 - 0.245)	0.012 (0.009 - 0.015)	0.012 (0.009 - 0.015)	0.012 (0.009 - 0.015)	1.335 (0.518 - 2.690)	1.340 (0.522 - 2.708)	1.332 (0.510 - 2.696)
**70+ years (rate per capita)**	0.167 (0.143 - 0.194)	0.166 (0.141 - 0.193)	0.165 (0.141 - 0.192)	0.008 (0.006 - 0.011)	0.008 (0.006 - 0.011)	0.008 (0.006 - 0.010)	1.037 (0.407 - 2.099)	1.032 (0.411 - 2.108)	1.013 (0.401 - 2.067)
**All ages (rate per capita)**	0.054 (0.047 - 0.063)	0.058 (0.050 - 0.067)	0.064 (0.056 - 0.074)	0.005 (0.004 - 0.005)	0.005 (0.004 - 0.006)	0.006 (0.005 - 0.006)	0.355 (0.138 - 0.733)	0.377 (0.146 - 0.784)	0.418 (0.161 - 0.868)
**Age-standardized (rate per capita)**	0.087 (0.076 - 0.100)	0.087 (0.076 - 0.101)	0.087 (0.076 - 0.101)	0.007 (0.006 - 0.008)	0.007 (0.006 - 0.008)	0.007 (0.006 - 0.008)	0.566 (0.219 - 1.183)	0.569 (0.221 - 1.197)	0.565 (0.219 - 1.170)
**Total number of cases (thousands)**	386 (337 - 447)	534 (464 - 618)	687 (599 - 794)	33 (29 - 38)	47 (41 - 54)	59 (52 - 69)	3 (1 - 5)	3 (1 - 7)	4 (2 - 9)
**Jamaica**									
**15 - 49 years (rate per capita)**	0.066 (0.056 - 0.078)	0.078 (0.065 - 0.092)	0.076 (0.065 - 0.091)	0.007 (0.006 - 0.009)	0.008 (0.007 - 0.010)	0.008 (0.007 - 0.010)	0.439 (0.168 - 0.925)	0.517 (0.196 - 1.078)	0.509 (0.192 - 1.075)
**50 - 69 years (rate per capita)**	0.207 (0.175 - 0.243)	0.208 (0.176 - 0.244)	0.208 (0.175 - 0.244)	0.012 (0.009 - 0.015)	0.012 (0.009 - 0.015)	0.012 (0.009 - 0.015)	1.349 (0.523 - 2.730)	1.351 (0.526 - 2.743)	1.347 (0.526 - 2.722)
**70+ years (rate per capita)**	0.161 (0.139 - 0.186)	0.160 (0.138 - 0.184)	0.159 (0.137 - 0.186)	0.008 (0.006 - 0.010)	0.008 (0.006 - 0.010)	0.008 (0.006 - 0.010)	1.010 (0.403 - 2.089)	1.002 (0.399 - 2.062)	0.991 (0.397 - 2.001)
**All ages (rate per capita)**	0.062 (0.055 - 0.071)	0.075 (0.066 - 0.086)	0.084 (0.074 - 0.097)	0.005 (0.005 - 0.006)	0.006 (0.005 - 0.007)	0.007 (0.006 - 0.008)	0.409 (0.157 - 0.851)	0.490 (0.192 - 1.020)	0.549 (0.214 - 1.137)
**Age-standardized (rate per capita)**	0.087 (0.076 - 0.100)	0.088 (0.077 - 0.101)	0.087 (0.077 - 0.101)	0.007 (0.006 - 0.008)	0.007 (0.006 - 0.008)	0.007 (0.006 - 0.008)	0.573 (0.221 - 1.193)	0.573 (0.223 - 1.195)	0.571 (0.223 - 1.185)
**Total number of cases (thousands)**	149 (131 - 171)	201 (176 - 230)	238 (209 - 275)	13 (11 - 14)	16 (14 - 19)	19 (16 - 22)	1 (0 - 2)	1 (1 - 3)	2 (1 - 3)
**Puerto Rico**									
**15 - 49 years (rate per capita)**	0.078 (0.066 - 0.093)	0.083 (0.069 - 0.099)	0.084 (0.071 - 0.101)	0.008 (0.007 - 0.010)	0.008 (0.007 - 0.010)	0.008 (0.007 - 0.010)	0.521 (0.198 - 1.071)	0.549 (0.209 - 1.151)	0.562 (0.214 - 1.177)
**50 - 69 years (rate per capita)**	0.206 (0.174 - 0.242)	0.206 (0.174 - 0.242)	0.206 (0.175 - 0.241)	0.012 (0.009 - 0.015)	0.012 (0.009 - 0.015)	0.012 (0.009 - 0.015)	1.339 (0.518 - 2.729)	1.336 (0.520 - 2.727)	1.334 (0.519 - 2.702)

	Prevalence 1990	Prevalence 2005	Prevalence 2015	Incidence 1990	Incidence 2005	Incidence 2015	YLD rate 1990	YLD rate 2005	YLD rate 2015
70+ years (rate per capita)	0.162 (0.139 - 0.187)	0.159 (0.137 - 0.183)	0.158 (0.136 - 0.183)	0.008 (0.006 - 0.010)	0.008 (0.006 - 0.010)	0.008 (0.006 - 0.010)	1.011 (0.403 - 2.073)	0.989 (0.396 - 2.019)	0.983 (0.400 - 1.978)
All ages (rate per capita)	0.082 (0.072 - 0.094)	0.095 (0.083 - 0.109)	0.102 (0.089 - 0.118)	0.006 (0.006 - 0.007)	0.007 (0.006 - 0.008)	0.008 (0.007 - 0.009)	0.535 (0.210 - 1.104)	0.619 (0.241 - 1.288)	0.665 (0.261 - 1.391)
Age-standardized (rate per capita)	0.087 (0.076 - 0.100)	0.087 (0.076 - 0.100)	0.087 (0.076 - 0.101)	0.007 (0.006 - 0.008)	0.007 (0.006 - 0.008)	0.007 (0.006 - 0.008)	0.569 (0.224 - 1.176)	0.569 (0.220 - 1.184)	0.569 (0.221 - 1.190)
Total number of cases (thousands)	289 (253 - 333)	357 (312 - 412)	376 (329 - 434)	23 (20 - 26)	27 (24 - 31)	28 (24 - 32)	2 (1 - 4)	2 (1 - 5)	2 (1 - 5)
**Saint Lucia**									
15 - 49 years (rate per capita)	0.066 (0.056 - 0.078)	0.076 (0.064 - 0.090)	0.082 (0.069 - 0.097)	0.007 (0.006 - 0.009)	0.008 (0.007 - 0.009)	0.008 (0.007 - 0.010)	0.436 (0.167 - 0.911)	0.504 (0.190 - 1.067)	0.545 (0.205 - 1.140)
50 - 69 years (rate per capita)	0.208 (0.175 - 0.245)	0.207 (0.175 - 0.244)	0.207 (0.174 - 0.245)	0.012 (0.009 - 0.015)	0.012 (0.009 - 0.015)	0.012 (0.009 - 0.015)	1.341 (0.524 - 2.713)	1.345 (0.524 - 2.726)	1.344 (0.521 - 2.750)
70+ years (rate per capita)	0.166 (0.142 - 0.193)	0.159 (0.137 - 0.184)	0.159 (0.138 - 0.184)	0.008 (0.006 - 0.011)	0.008 (0.006 - 0.010)	0.008 (0.006 - 0.010)	1.031 (0.411 - 2.091)	0.986 (0.401 - 2.007)	0.992 (0.402 - 2.002)
All ages (rate per capita)	0.062 (0.054 - 0.071)	0.074 (0.064 - 0.084)	0.089 (0.078 - 0.103)	0.005 (0.005 - 0.006)	0.006 (0.005 - 0.007)	0.007 (0.006 - 0.008)	0.403 (0.157 - 0.838)	0.482 (0.187 - 0.997)	0.585 (0.225 - 1.211)
Age-standardized (rate per capita)	0.087 (0.076 - 0.101)	0.087 (0.076 - 0.101)	0.087 (0.076 - 0.100)	0.007 (0.006 - 0.008)	0.007 (0.006 - 0.008)	0.007 (0.006 - 0.008)	0.569 (0.221 - 1.198)	0.571 (0.222 - 1.197)	0.570 (0.220 - 1.181)
Total number of cases (thousands)	9 (8 - 10)	12 (11 - 14)	17 (14 - 19)	1 (1 - 1)	1 (1 - 1)	1 (1 - 1)	0 (0 - 0)	0 (0 - 0)	0 (0 - 0)
**Saint Vincent and the Grenadines**									
15 - 49 years (rate per capita)	0.063 (0.053 - 0.074)	0.078 (0.066 - 0.093)	0.080 (0.067 - 0.094)	0.007 (0.006 - 0.008)	0.008 (0.007 - 0.010)	0.008 (0.007 - 0.010)	0.417 (0.158 - 0.871)	0.520 (0.199 - 1.086)	0.530 (0.202 - 1.108)
50 - 69 years (rate per capita)	0.206 (0.173 - 0.243)	0.207 (0.175 - 0.244)	0.208 (0.174 - 0.248)	0.012 (0.009 - 0.015)	0.012 (0.009 - 0.015)	0.012 (0.009 - 0.015)	1.340 (0.525 - 2.719)	1.345 (0.522 - 2.735)	1.350 (0.516 - 2.734)
70+ years (rate per capita)	0.163 (0.140 - 0.189)	0.162 (0.139 - 0.187)	0.161 (0.139 - 0.186)	0.008 (0.006 - 0.010)	0.008 (0.006 - 0.010)	0.008 (0.006 - 0.010)	1.020 (0.410 - 2.090)	1.008 (0.406 - 2.055)	0.999 (0.402 - 2.049)
All ages (rate per capita)	0.058 (0.050 - 0.066)	0.075 (0.065 - 0.086)	0.086 (0.075 - 0.100)	0.005 (0.004 - 0.006)	0.006 (0.005 - 0.007)	0.007 (0.006 - 0.008)	0.377 (0.146 - 0.786)	0.492 (0.189 - 1.015)	0.566 (0.218 - 1.179)
Age-standardized (rate per capita)	0.087 (0.076 - 0.101)	0.088 (0.077 - 0.101)	0.088 (0.077 - 0.101)	0.007 (0.006 - 0.008)	0.007 (0.006 - 0.008)	0.007 (0.006 - 0.008)	0.570 (0.221 - 1.191)	0.572 (0.222 - 1.196)	0.571 (0.221 - 1.184)
Total number of cases (thousands)	6 (5 - 7)	8 (7 - 9)	9 (8 - 11)	1 (0 - 1)	1 (1 - 1)	1 (1 - 1)	0 (0 - 0)	0 (0 - 0)	0 (0 - 0)
**Suriname**									
15 - 49 years (rate per capita)	0.068 (0.058 - 0.081)	0.080 (0.067 - 0.095)	0.082 (0.069 - 0.096)	0.008 (0.006 - 0.009)	0.008 (0.007 - 0.010)	0.008 (0.007 - 0.010)	0.455 (0.175 - 0.947)	0.530 (0.200 - 1.113)	0.543 (0.205 - 1.145)
50 - 69 years (rate per capita)	0.208 (0.175 - 0.245)	0.207 (0.176 - 0.243)	0.207 (0.175 - 0.244)	0.012 (0.009 - 0.015)	0.012 (0.009 - 0.015)	0.012 (0.009 - 0.015)	1.356 (0.524 - 2.747)	1.350 (0.523 - 2.745)	1.349 (0.522 - 2.725)
70+ years (rate per capita)	0.163 (0.140 - 0.188)	0.165 (0.141 - 0.190)	0.162 (0.139 - 0.187)	0.008 (0.006 - 0.010)	0.008 (0.006 - 0.010)	0.008 (0.006 - 0.010)	1.023 (0.411 - 2.094)	1.032 (0.405 - 2.109)	1.013 (0.406 - 2.068)
All ages (rate per capita)	0.064 (0.056 - 0.073)	0.075 (0.066 - 0.087)	0.084 (0.073 - 0.097)	0.006 (0.005 - 0.006)	0.006 (0.005 - 0.007)	0.007 (0.006 - 0.008)	0.420 (0.162 - 0.867)	0.496 (0.192 - 1.035)	0.552 (0.215 - 1.160)
Age-standardized (rate per capita)	0.088 (0.076 - 0.100)	0.088 (0.077 - 0.100)	0.087 (0.076 - 0.100)	0.007 (0.006 - 0.008)	0.007 (0.006 - 0.008)	0.007 (0.006 - 0.008)	0.573 (0.223 - 1.183)	0.574 (0.223 - 1.198)	0.572 (0.224 - 1.197)
Total number of cases (thousands)	26 (23 - 30)	37 (32 - 43)	46 (40 - 53)	2 (2 - 3)	3 (3 - 4)	4 (3 - 4)	0 (0 - 0)	0 (0 - 1)	0 (0 - 1)
**Trinidad and Tobago**									
15 - 49 years (rate per capita)	0.074 (0.062 - 0.088)	0.080 (0.067 - 0.094)	0.086 (0.072 - 0.102)	0.008 (0.007 - 0.009)	0.008 (0.007 - 0.010)	0.009 (0.007 - 0.010)	0.494 (0.187 - 1.024)	0.532 (0.202 - 1.105)	0.571 (0.217 - 1.199)
50 - 69 years (rate per capita)	0.207 (0.175 - 0.245)	0.208 (0.175 - 0.246)	0.208 (0.175 - 0.245)	0.012 (0.009 - 0.015)	0.012 (0.009 - 0.015)	0.012 (0.009 - 0.015)	1.344 (0.520 - 2.698)	1.348 (0.525 - 2.721)	1.344 (0.523 - 2.738)
70+ years (rate per capita)	0.164 (0.142 - 0.191)	0.162 (0.139 - 0.188)	0.163 (0.140 - 0.189)	0.008 (0.006 - 0.010)	0.008 (0.006 - 0.010)	0.008 (0.006 - 0.010)	1.025 (0.413 - 2.114)	1.010 (0.400 - 2.076)	1.016 (0.404 - 2.104)
All ages (rate per capita)	0.067 (0.058 - 0.078)	0.087 (0.076 - 0.101)	0.099 (0.086 - 0.114)	0.006 (0.005 - 0.007)	0.007 (0.006 - 0.008)	0.008 (0.007 - 0.009)	0.441 (0.170 - 0.910)	0.571 (0.219 - 1.187)	0.644 (0.251 - 1.341)
Age-standardized (rate per capita)	0.088 (0.076 - 0.101)	0.088 (0.076 - 0.101)	0.088 (0.076 - 0.101)	0.007 (0.006 - 0.008)	0.007 (0.006 - 0.008)	0.007 (0.006 - 0.008)	0.571 (0.221 - 1.194)	0.572 (0.221 - 1.181)	0.571 (0.223 - 1.187)
Total number of cases (thousands)	82 (72 - 95)	113 (98 - 131)	134 (117 - 155)	7 (6 - 8)	9 (8 - 11)	10 (9 - 12)	1 (0 - 1)	1 (0 - 2)	1 (0 - 2)
**Virgin Islands, U.S.**									
15 - 49 years (rate per capita)	0.084 (0.070 - 0.099)	0.089 (0.074 - 0.106)	0.088 (0.074 - 0.105)	0.008 (0.007 - 0.010)	0.009 (0.007 - 0.011)	0.009 (0.007 - 0.010)	0.557 (0.211 - 1.168)	0.592 (0.224 - 1.242)	0.585 (0.220 - 1.229)
50 - 69 years (rate per capita)	0.207 (0.175 - 0.244)	0.207 (0.174 - 0.245)	0.207 (0.175 - 0.242)	0.012 (0.009 - 0.015)	0.012 (0.009 - 0.015)	0.012 (0.009 - 0.015)	1.349 (0.522 - 2.759)	1.348 (0.522 - 2.748)	1.339 (0.522 - 2.709)
70+ years (rate per capita)	0.167 (0.142 - 0.193)	0.162 (0.140 - 0.188)	0.164 (0.141 - 0.190)	0.008 (0.006 - 0.011)	0.008 (0.006 - 0.010)	0.008 (0.006 - 0.011)	1.045 (0.414 - 2.115)	1.011 (0.401 - 2.085)	1.020 (0.401 - 2.078)
All ages (rate per capita)	0.079 (0.069 - 0.091)	0.102 (0.089 - 0.118)	0.110 (0.096 - 0.128)	0.006 (0.006 - 0.007)	0.007 (0.006 - 0.009)	0.008 (0.007 - 0.009)	0.519 (0.200 - 1.075)	0.666 (0.259 - 1.373)	0.718 (0.283 - 1.484)
Age-standardized (rate per capita)	0.087 (0.076 - 0.100)	0.087 (0.076 - 0.100)	0.087 (0.076 - 0.100)	0.007 (0.006 - 0.008)	0.007 (0.006 - 0.008)	0.007 (0.006 - 0.008)	0.572 (0.222 - 1.176)	0.570 (0.221 - 1.182)	0.570 (0.222 - 1.181)
Total number of cases (thousands)	8 (7 - 10)	11 (10 - 13)	12 (10 - 14)	1 (1 - 1)	1 (1 - 1)	1 (1 - 1)	0 (0 - 0)	0 (0 - 0)	0 (0 - 0)
**Tropical Latin America**									
15 - 49 years (rate per capita)	0.085 (0.072 - 0.099)	0.093 (0.079 - 0.110)	0.096 (0.081 - 0.114)	0.009 (0.008 - 0.011)	0.010 (0.008 - 0.011)	0.010 (0.008 - 0.012)	0.564 (0.217 - 1.199)	0.620 (0.237 - 1.301)	0.640 (0.242 - 1.352)
50 - 69 years (rate per capita)	0.235 (0.201 - 0.274)	0.224 (0.191 - 0.263)	0.230 (0.196 - 0.270)	0.015 (0.011 - 0.018)	0.014 (0.010 - 0.018)	0.015 (0.011 - 0.018)	1.530 (0.598 - 3.102)	1.457 (0.568 - 2.948)	1.496 (0.584 - 3.015)
70+ years (rate per capita)	0.159 (0.138 - 0.183)	0.150 (0.129 - 0.172)	0.155 (0.134 - 0.178)	0.009 (0.007 - 0.012)	0.009 (0.007 - 0.012)	0.009 (0.007 - 0.012)	0.995 (0.399 - 2.037)	0.932 (0.374 - 1.889)	0.964 (0.389 - 1.965)
All ages (rate per capita)	0.072 (0.063 - 0.082)	0.087 (0.076 - 0.100)	0.100 (0.087 - 0.116)	0.007 (0.006 - 0.008)	0.008 (0.007 - 0.009)	0.008 (0.007 - 0.010)	0.474 (0.183 - 0.983)	0.571 (0.220 - 1.186)	0.656 (0.253 - 1.369)
Age-standardized (rate per capita)	0.099 (0.088 - 0.113)	0.097 (0.085 - 0.112)	0.098 (0.086 - 0.112)	0.008 (0.007 - 0.009)	0.008 (0.007 - 0.009)	0.008 (0.007 - 0.009)	0.652 (0.254 - 1.359)	0.636 (0.246 - 1.332)	0.639 (0.248 - 1.336)
Total number of cases (thousands)	11,122 (9,769 - 12,721)	16,859 (14,742 - 19,404)	21,434 (18,767 - 24,779)	1,014 (891 - 1,162)	1,463 (1,280 - 1,686)	1,794 (1,573 - 2,051)	73 (28 - 152)	111 (43 - 230)	141 (54 - 294)
**Brazil**									
15 - 49 years (rate per capita)	0.085 (0.072 - 0.100)	0.094 (0.079 - 0.110)	0.097 (0.081 - 0.114)	0.009 (0.008 - 0.011)	0.010 (0.008 - 0.011)	0.010 (0.008 - 0.012)	0.566 (0.218 - 1.201)	0.623 (0.238 - 1.307)	0.643 (0.243 - 1.359)
50 - 69 years (rate per capita)	0.235 (0.202 - 0.274)	0.224 (0.190 - 0.262)	0.230 (0.196 - 0.270)	0.015 (0.011 - 0.018)	0.014 (0.010 - 0.018)	0.015 (0.011 - 0.018)	1.531 (0.598 - 3.103)	1.456 (0.567 - 2.945)	1.496 (0.584 - 3.015)
70+ years (rate per capita)	0.159 (0.137 - 0.183)	0.149 (0.128 - 0.172)	0.154 (0.134 - 0.178)	0.009 (0.007 - 0.012)	0.009 (0.007 - 0.012)	0.009 (0.007 - 0.012)	0.991 (0.398 - 2.028)	0.927 (0.373 - 1.877)	0.961 (0.387 - 1.957)
All ages (rate per capita)	0.072 (0.063 - 0.083)	0.087 (0.076 - 0.101)	0.101 (0.088 - 0.116)	0.007 (0.006 - 0.008)	0.008 (0.007 - 0.009)	0.008 (0.007 - 0.010)	0.476 (0.184 - 0.987)	0.575 (0.221 - 1.194)	0.660 (0.255 - 1.378)
Age-standardized (rate per capita)	0.100 (0.088 - 0.114)	0.097 (0.085 - 0.112)	0.098 (0.086 - 0.112)	0.008 (0.007 - 0.009)	0.008 (0.007 - 0.009)	0.008 (0.007 - 0.009)	0.652 (0.255 - 1.359)	0.636 (0.246 - 1.332)	0.639 (0.248 - 1.336)
Total number of cases (thousands)	10,864 (9,545 - 12,427)	16,450 (14,383 - 18,933)	20,911 (18,299 - 24,167)	992 (871 - 1,137)	1,429 (1,251 - 1,647)	1,751 (1,534 - 2,001)	72 (28 - 149)	108 (42 - 225)	137 (53 - 286)
**Paraguay**									
15 - 49 years (rate per capita)	0.077 (0.064 - 0.091)	0.079 (0.067 - 0.093)	0.080 (0.067 - 0.094)	0.008 (0.007 - 0.010)	0.008 (0.007 - 0.010)	0.009 (0.007 - 0.010)	0.513 (0.194 - 1.080)	0.527 (0.202 - 1.111)	0.533 (0.204 - 1.123)
50 - 69 years (rate per capita)	0.229 (0.194 - 0.270)	0.231 (0.194 - 0.273)	0.230 (0.195 - 0.272)	0.013 (0.010 - 0.016)	0.013 (0.010 - 0.017)	0.013 (0.010 - 0.016)	1.492 (0.583 - 3.038)	1.501 (0.586 - 3.058)	1.494 (0.584 - 3.042)
70+ years (rate per capita)	0.180 (0.155 - 0.207)	0.177 (0.153 - 0.204)	0.178 (0.154 - 0.205)	0.008 (0.006 - 0.011)	0.008 (0.006 - 0.011)	0.008 (0.006 - 0.011)	1.126 (0.445 - 2.298)	1.105 (0.437 - 2.250)	1.102 (0.442 - 2.228)
All ages (rate per capita)	0.061 (0.053 - 0.070)	0.070 (0.061 - 0.081)	0.079 (0.069 - 0.091)	0.005 (0.005 - 0.006)	0.006 (0.005 - 0.007)	0.006 (0.006 - 0.007)	0.403 (0.156 - 0.837)	0.463 (0.179 - 0.969)	0.516 (0.200 - 1.086)
Age-standardized (rate per capita)	0.097 (0.085 - 0.112)	0.097 (0.085 - 0.113)	0.097 (0.085 - 0.112)	0.007 (0.006 - 0.008)	0.007 (0.006 - 0.008)	0.007 (0.006 - 0.008)	0.635 (0.248 - 1.326)	0.637 (0.247 - 1.337)	0.634 (0.246 - 1.329)
Total number of cases (thousands)	258 (226 - 298)	409 (357 - 472)	523 (457 - 604)	22 (20 - 26)	34 (30 - 40)	43 (38 - 49)	2 (1 - 4)	3 (1 - 6)	3 (1 - 7)
**Southeast Asia, East Asia, and Oceania**									
15 - 49 years (rate per capita)	0.043 (0.036 - 0.052)	0.049 (0.041 - 0.059)	0.055 (0.046 - 0.066)	0.005 (0.004 - 0.006)	0.005 (0.004 - 0.006)	0.006 (0.005 - 0.007)	0.288 (0.109 - 0.611)	0.330 (0.125 - 0.700)	0.368 (0.139 - 0.775)
50 - 69 years (rate per capita)	0.162 (0.136 - 0.192)	0.157 (0.131 - 0.187)	0.163 (0.136 - 0.193)	0.010 (0.007 - 0.012)	0.009 (0.007 - 0.012)	0.010 (0.007 - 0.012)	1.060 (0.413 - 2.155)	1.028 (0.400 - 2.079)	1.065 (0.416 - 2.179)
70+ years (rate per capita)	0.160 (0.137 - 0.186)	0.156 (0.133 - 0.181)	0.157 (0.135 - 0.182)	0.006 (0.004 - 0.008)	0.006 (0.004 - 0.008)	0.006 (0.004 - 0.008)	1.013 (0.401 - 2.064)	0.984 (0.389 - 1.999)	0.995 (0.395 - 2.025)
All ages (rate per capita)	0.048 (0.041 - 0.055)	0.059 (0.051 - 0.069)	0.071 (0.062 - 0.083)	0.004 (0.003 - 0.005)	0.005 (0.004 - 0.005)	0.005 (0.005 - 0.006)	0.313 (0.122 - 0.654)	0.390 (0.150 - 0.806)	0.468 (0.180 - 0.960)
Age-standardized (rate per capita)	0.064 (0.056 - 0.074)	0.062 (0.054 - 0.072)	0.064 (0.056 - 0.074)	0.005 (0.004 - 0.005)	0.005 (0.004 - 0.005)	0.005 (0.004 - 0.005)	0.419 (0.164 - 0.873)	0.409 (0.159 - 0.850)	0.419 (0.163 - 0.869)
Total number of cases (thousands)	79,104 (68,885 - 91,675)	115,384 (100,139 - 134,656)	148,955 (128,849 - 173,395)	6,625 (5,756 - 7,668)	9,194 (7,942 - 10,670)	11,237 (9,661 - 13,036)	521 (202 - 1,088)	760 (292 - 1,571)	979 (377 - 2,011)
**East Asia**									
15 - 49 years (rate per capita)	0.044 (0.037 - 0.053)	0.051 (0.043 - 0.061)	0.059 (0.049 - 0.070)	0.005 (0.004 - 0.006)	0.005 (0.005 - 0.007)	0.006 (0.005 - 0.007)	0.295 (0.112 - 0.630)	0.343 (0.130 - 0.725)	0.392 (0.147 - 0.829)
50 - 69 years (rate per capita)	0.164 (0.138 - 0.194)	0.158 (0.133 - 0.188)	0.166 (0.140 - 0.196)	0.010 (0.007 - 0.012)	0.009 (0.007 - 0.012)	0.010 (0.007 - 0.012)	1.075 (0.418 - 2.175)	1.038 (0.404 - 2.097)	1.091 (0.425 - 2.216)
70+ years (rate per capita)	0.163 (0.140 - 0.190)	0.157 (0.135 - 0.182)	0.160 (0.138 - 0.186)	0.006 (0.004 - 0.008)	0.006 (0.004 - 0.008)	0.006 (0.004 - 0.008)	1.037 (0.410 - 2.124)	0.999 (0.396 - 2.042)	1.017 (0.404 - 2.084)
All ages (rate per capita)	0.051 (0.044 - 0.059)	0.064 (0.056 - 0.075)	0.079 (0.068 - 0.091)	0.004 (0.004 - 0.005)	0.005 (0.004 - 0.006)	0.006 (0.005 - 0.007)	0.333 (0.129 - 0.696)	0.423 (0.165 - 0.880)	0.518 (0.200 - 1.070)
Age-standardized (rate per capita)	0.065 (0.057 - 0.075)	0.063 (0.055 - 0.073)	0.065 (0.057 - 0.075)	0.005 (0.004 - 0.006)	0.005 (0.004 - 0.005)	0.005 (0.004 - 0.006)	0.426 (0.167 - 0.889)	0.414 (0.162 - 0.866)	0.428 (0.167 - 0.893)
Total number of cases (thousands)	60,425 (52,681 - 69,916)	87,326 (75,953 - 101,586)	112,700 (97,784 - 131,090)	5,033 (4,370 - 5,808)	6,864 (5,942 - 7,925)	8,343 (7,215 - 9,638)	398 (154 - 831)	576 (224 - 1,196)	742 (287 - 1,533)
**China**									

	Prevalence 1990	Prevalence 2005	Prevalence 2015	Incidence 1990	Incidence 2005	Incidence 2015	YLD rate 1990	YLD rate 2005	YLD rate 2015
15 - 49 years (rate per capita)	0.044 (0.037 - 0.053)	0.051 (0.043 - 0.061)	0.059 (0.049 - 0.071)	0.005 (0.004 - 0.006)	0.005 (0.005 - 0.007)	0.006 (0.005 - 0.008)	0.297 (0.112 - 0.635)	0.344 (0.131 - 0.728)	0.395 (0.149 - 0.835)
50 - 69 years (rate per capita)	0.166 (0.139 - 0.196)	0.159 (0.133 - 0.189)	0.168 (0.141 - 0.198)	0.010 (0.007 - 0.012)	0.009 (0.007 - 0.012)	0.010 (0.007 - 0.012)	1.083 (0.421 - 2.193)	1.044 (0.406 - 2.109)	1.099 (0.428 - 2.233)
70+ years (rate per capita)	0.164 (0.141 - 0.191)	0.158 (0.136 - 0.183)	0.162 (0.139 - 0.187)	0.006 (0.005 - 0.008)	0.006 (0.005 - 0.008)	0.006 (0.004 - 0.008)	1.044 (0.412 - 2.138)	1.006 (0.399 - 2.055)	1.026 (0.408 - 2.103)
All ages (rate per capita)	0.051 (0.044 - 0.059)	0.065 (0.056 - 0.075)	0.079 (0.069 - 0.092)	0.004 (0.004 - 0.005)	0.005 (0.004 - 0.006)	0.006 (0.005 - 0.007)	0.335 (0.130 - 0.699)	0.426 (0.165 - 0.884)	0.522 (0.202 - 1.078)
Age-standardized (rate per capita)	0.065 (0.057 - 0.075)	0.063 (0.055 - 0.073)	0.066 (0.057 - 0.076)	0.005 (0.004 - 0.006)	0.005 (0.004 - 0.005)	0.005 (0.004 - 0.006)	0.429 (0.168 - 0.896)	0.417 (0.163 - 0.872)	0.432 (0.169 - 0.899)
Total number of cases (thousands)	58,692 (51,189 - 67,900)	84,769 (73,711 - 98,640)	109,632 (95,112 - 127,527)	4,887 (4,244 - 5,640)	6,664 (5,768 - 7,693)	8,112 (7,014 - 9,373)	387 (150 - 808)	559 (217 - 1,161)	722 (279 - 1,491)
**North Korea**									
15 - 49 years (rate per capita)	0.039 (0.032 - 0.046)	0.047 (0.039 - 0.056)	0.048 (0.040 - 0.058)	0.004 (0.004 - 0.005)	0.005 (0.004 - 0.006)	0.005 (0.004 - 0.006)	0.257 (0.097 - 0.532)	0.312 (0.117 - 0.658)	0.321 (0.120 - 0.677)
50 - 69 years (rate per capita)	0.135 (0.112 - 0.161)	0.142 (0.118 - 0.168)	0.138 (0.115 - 0.164)	0.008 (0.006 - 0.011)	0.008 (0.006 - 0.010)	0.008 (0.006 - 0.011)	0.883 (0.345 - 1.819)	0.925 (0.361 - 1.876)	0.903 (0.352 - 1.841)
70+ years (rate per capita)	0.137 (0.117 - 0.160)	0.137 (0.117 - 0.160)	0.138 (0.117 - 0.161)	0.006 (0.004 - 0.007)	0.006 (0.004 - 0.007)	0.006 (0.004 - 0.007)	0.867 (0.345 - 1.764)	0.866 (0.345 - 1.781)	0.874 (0.344 - 1.783)
All ages (rate per capita)	0.044 (0.038 - 0.051)	0.054 (0.047 - 0.063)	0.060 (0.052 - 0.070)	0.004 (0.003 - 0.004)	0.004 (0.004 - 0.005)	0.005 (0.004 - 0.005)	0.290 (0.112 - 0.605)	0.357 (0.138 - 0.740)	0.396 (0.155 - 0.818)
Age-standardized (rate per capita)	0.054 (0.047 - 0.063)	0.055 (0.048 - 0.064)	0.055 (0.048 - 0.063)	0.004 (0.004 - 0.005)	0.004 (0.004 - 0.005)	0.004 (0.004 - 0.005)	0.357 (0.140 - 0.734)	0.361 (0.141 - 0.747)	0.359 (0.140 - 0.743)
Total number of cases (thousands)	888 (767 - 1,034)	1,290 (1,121 - 1,494)	1,517 (1,312 - 1,769)	75 (65 - 86)	102 (88 - 117)	116 (99 - 135)	6 (2 - 12)	8 (3 - 18)	10 (4 - 21)
**Taiwan**									
15 - 49 years (rate per capita)	0.035 (0.029 - 0.042)	0.044 (0.037 - 0.053)	0.044 (0.036 - 0.052)	0.004 (0.003 - 0.005)	0.005 (0.004 - 0.006)	0.005 (0.004 - 0.006)	0.236 (0.089 - 0.492)	0.295 (0.111 - 0.616)	0.292 (0.109 - 0.611)
50 - 69 years (rate per capita)	0.127 (0.106 - 0.151)	0.126 (0.105 - 0.150)	0.125 (0.103 - 0.149)	0.008 (0.006 - 0.010)	0.008 (0.006 - 0.010)	0.008 (0.006 - 0.010)	0.827 (0.323 - 1.680)	0.822 (0.322 - 1.674)	0.813 (0.316 - 1.664)
70+ years (rate per capita)	0.129 (0.110 - 0.151)	0.127 (0.108 - 0.148)	0.122 (0.105 - 0.143)	0.005 (0.004 - 0.007)	0.005 (0.004 - 0.007)	0.005 (0.004 - 0.007)	0.813 (0.323 - 1.655)	0.800 (0.319 - 1.620)	0.769 (0.306 - 1.566)
All ages (rate per capita)	0.042 (0.036 - 0.048)	0.056 (0.048 - 0.065)	0.064 (0.056 - 0.074)	0.003 (0.003 - 0.004)	0.004 (0.004 - 0.005)	0.005 (0.004 - 0.005)	0.274 (0.106 - 0.562)	0.366 (0.142 - 0.762)	0.418 (0.162 - 0.868)
Age-standardized (rate per capita)	0.050 (0.043 - 0.058)	0.051 (0.044 - 0.059)	0.050 (0.043 - 0.058)	0.004 (0.003 - 0.004)	0.004 (0.003 - 0.004)	0.004 (0.003 - 0.004)	0.326 (0.127 - 0.672)	0.333 (0.130 - 0.692)	0.325 (0.126 - 0.675)
Total number of cases (thousands)	844 (736 - 978)	1,268 (1,099 - 1,470)	1,550 (1,346 - 1,798)	70 (61 - 81)	99 (86 - 115)	115 (99 - 133)	6 (2 - 11)	8 (3 - 17)	10 (4 - 21)
**Southeast Asia**									
15 - 49 years (rate per capita)	0.040 (0.034 - 0.049)	0.045 (0.037 - 0.055)	0.048 (0.039 - 0.059)	0.005 (0.004 - 0.006)	0.005 (0.004 - 0.006)	0.005 (0.004 - 0.006)	0.268 (0.101 - 0.558)	0.300 (0.113 - 0.635)	0.319 (0.119 - 0.663)
50 - 69 years (rate per capita)	0.157 (0.129 - 0.188)	0.154 (0.127 - 0.186)	0.153 (0.125 - 0.184)	0.009 (0.007 - 0.012)	0.009 (0.007 - 0.012)	0.009 (0.007 - 0.012)	1.019 (0.396 - 2.086)	1.000 (0.387 - 2.049)	0.992 (0.386 - 2.046)
70+ years (rate per capita)	0.150 (0.125 - 0.177)	0.150 (0.125 - 0.177)	0.147 (0.123 - 0.174)	0.006 (0.004 - 0.008)	0.006 (0.004 - 0.008)	0.006 (0.004 - 0.008)	0.936 (0.371 - 1.916)	0.935 (0.370 - 1.907)	0.921 (0.369 - 1.887)
All ages (rate per capita)	0.040 (0.035 - 0.047)	0.048 (0.041 - 0.057)	0.055 (0.047 - 0.066)	0.003 (0.003 - 0.004)	0.004 (0.003 - 0.005)	0.004 (0.004 - 0.005)	0.264 (0.103 - 0.545)	0.315 (0.122 - 0.640)	0.362 (0.139 - 0.736)
Age-standardized (rate per capita)	0.062 (0.053 - 0.072)	0.061 (0.052 - 0.071)	0.060 (0.052 - 0.071)	0.005 (0.004 - 0.005)	0.005 (0.004 - 0.005)	0.004 (0.004 - 0.005)	0.401 (0.156 - 0.824)	0.396 (0.154 - 0.814)	0.394 (0.153 - 0.807)
Total number of cases (thousands)	18,554 (15,928 - 21,723)	27,864 (23,786 - 32,946)	35,997 (30,725 - 42,672)	1,581 (1,374 - 1,841)	2,312 (1,983 - 2,717)	2,871 (2,444 - 3,376)	122 (47 - 252)	183 (71 - 372)	236 (91 - 479)
**Cambodia**									
15 - 49 years (rate per capita)	0.030 (0.025 - 0.035)	0.029 (0.024 - 0.034)	0.031 (0.026 - 0.037)	0.003 (0.003 - 0.004)	0.003 (0.003 - 0.004)	0.004 (0.003 - 0.004)	0.197 (0.076 - 0.415)	0.190 (0.072 - 0.399)	0.210 (0.080 - 0.439)
50 - 69 years (rate per capita)	0.116 (0.096 - 0.138)	0.113 (0.094 - 0.134)	0.114 (0.094 - 0.137)	0.007 (0.006 - 0.009)	0.008 (0.006 - 0.009)	0.007 (0.006 - 0.009)	0.748 (0.289 - 1.519)	0.734 (0.283 - 1.503)	0.740 (0.288 - 1.494)
70+ years (rate per capita)	0.116 (0.098 - 0.138)	0.119 (0.100 - 0.141)	0.116 (0.097 - 0.137)	0.005 (0.004 - 0.007)	0.005 (0.004 - 0.007)	0.005 (0.004 - 0.007)	0.725 (0.286 - 1.496)	0.743 (0.292 - 1.541)	0.724 (0.288 - 1.479)
All ages (rate per capita)	0.025 (0.022 - 0.029)	0.028 (0.024 - 0.032)	0.033 (0.029 - 0.039)	0.002 (0.002 - 0.003)	0.003 (0.002 - 0.003)	0.003 (0.003 - 0.003)	0.165 (0.064 - 0.340)	0.181 (0.070 - 0.373)	0.219 (0.084 - 0.454)
Age-standardized (rate per capita)	0.046 (0.040 - 0.053)	0.045 (0.039 - 0.052)	0.046 (0.040 - 0.053)	0.004 (0.003 - 0.004)	0.004 (0.003 - 0.004)	0.004 (0.003 - 0.004)	0.296 (0.116 - 0.604)	0.291 (0.114 - 0.595)	0.297 (0.116 - 0.617)
Total number of cases (thousands)	228 (198 - 264)	369 (321 - 426)	519 (447 - 606)	21 (18 - 24)	34 (29 - 39)	46 (40 - 53)	1 (1 - 3)	2 (1 - 5)	3 (1 - 7)
**Indonesia**									
15 - 49 years (rate per capita)	0.053 (0.044 - 0.063)	0.058 (0.048 - 0.070)	0.063 (0.052 - 0.076)	0.006 (0.005 - 0.007)	0.006 (0.005 - 0.008)	0.007 (0.005 - 0.008)	0.350 (0.134 - 0.727)	0.386 (0.146 - 0.811)	0.421 (0.160 - 0.864)
50 - 69 years (rate per capita)	0.196 (0.162 - 0.232)	0.195 (0.162 - 0.231)	0.195 (0.163 - 0.233)	0.011 (0.008 - 0.014)	0.011 (0.008 - 0.014)	0.011 (0.008 - 0.015)	1.272 (0.492 - 2.574)	1.266 (0.490 - 2.567)	1.269 (0.489 - 2.590)
70+ years (rate per capita)	0.186 (0.156 - 0.220)	0.185 (0.155 - 0.219)	0.185 (0.155 - 0.219)	0.006 (0.004 - 0.009)	0.006 (0.004 - 0.009)	0.006 (0.004 - 0.009)	1.164 (0.459 - 2.373)	1.155 (0.458 - 2.336)	1.158 (0.462 - 2.338)
All ages (rate per capita)	0.052 (0.045 - 0.059)	0.060 (0.052 - 0.070)	0.069 (0.060 - 0.081)	0.004 (0.004 - 0.005)	0.005 (0.004 - 0.006)	0.005 (0.005 - 0.006)	0.338 (0.132 - 0.702)	0.397 (0.154 - 0.833)	0.453 (0.176 - 0.929)
Age-standardized (rate per capita)	0.078 (0.068 - 0.090)	0.077 (0.067 - 0.089)	0.077 (0.068 - 0.090)	0.006 (0.005 - 0.006)	0.006 (0.005 - 0.006)	0.006 (0.005 - 0.006)	0.508 (0.201 - 1.046)	0.502 (0.197 - 1.048)	0.505 (0.198 - 1.022)
Total number of cases (thousands)	9,335 (8,142 - 10,735)	13,686 (11,767 - 15,950)	17,784 (15,400 - 20,841)	784 (685 - 893)	1,127 (977 - 1,303)	1,412 (1,223 - 1,648)	61 (24 - 127)	90 (35 - 189)	117 (45 - 239)
**Laos**									
15 - 49 years (rate per capita)	0.024 (0.020 - 0.029)	0.025 (0.021 - 0.030)	0.025 (0.021 - 0.030)	0.003 (0.002 - 0.003)	0.003 (0.002 - 0.003)	0.003 (0.002 - 0.004)	0.162 (0.061 - 0.337)	0.165 (0.063 - 0.347)	0.168 (0.064 - 0.349)
50 - 69 years (rate per capita)	0.101 (0.085 - 0.120)	0.101 (0.084 - 0.120)	0.101 (0.084 - 0.120)	0.007 (0.005 - 0.008)	0.007 (0.005 - 0.008)	0.007 (0.005 - 0.008)	0.656 (0.256 - 1.334)	0.653 (0.256 - 1.339)	0.654 (0.252 - 1.342)
70+ years (rate per capita)	0.108 (0.092 - 0.126)	0.108 (0.091 - 0.126)	0.107 (0.091 - 0.126)	0.005 (0.004 - 0.006)	0.005 (0.004 - 0.006)	0.005 (0.004 - 0.006)	0.674 (0.265 - 1.382)	0.672 (0.265 - 1.358)	0.668 (0.259 - 1.361)
All ages (rate per capita)	0.022 (0.019 - 0.026)	0.023 (0.020 - 0.027)	0.026 (0.022 - 0.030)	0.002 (0.002 - 0.002)	0.002 (0.002 - 0.002)	0.002 (0.002 - 0.003)	0.145 (0.056 - 0.302)	0.151 (0.059 - 0.312)	0.170 (0.065 - 0.349)
Age-standardized (rate per capita)	0.040 (0.035 - 0.046)	0.040 (0.035 - 0.046)	0.040 (0.035 - 0.046)	0.003 (0.003 - 0.004)	0.003 (0.003 - 0.004)	0.003 (0.003 - 0.004)	0.261 (0.102 - 0.542)	0.261 (0.103 - 0.537)	0.261 (0.102 - 0.539)
Total number of cases (thousands)	94 (82 - 108)	133 (115 - 154)	176 (152 - 204)	8 (7 - 10)	12 (10 - 14)	16 (14 - 18)	1 (0 - 1)	1 (0 - 2)	1 (0 - 2)
**Malaysia**									
15 - 49 years (rate per capita)	0.037 (0.027 - 0.052)	0.040 (0.029 - 0.058)	0.040 (0.029 - 0.059)	0.004 (0.003 - 0.006)	0.004 (0.003 - 0.006)	0.005 (0.003 - 0.007)	0.245 (0.087 - 0.542)	0.266 (0.094 - 0.566)	0.270 (0.096 - 0.583)
50 - 69 years (rate per capita)	0.143 (0.098 - 0.192)	0.141 (0.097 - 0.191)	0.141 (0.097 - 0.190)	0.009 (0.005 - 0.013)	0.009 (0.006 - 0.013)	0.009 (0.005 - 0.013)	0.931 (0.348 - 2.100)	0.921 (0.345 - 2.071)	0.921 (0.343 - 2.089)
70+ years (rate per capita)	0.144 (0.104 - 0.188)	0.144 (0.104 - 0.189)	0.143 (0.103 - 0.190)	0.006 (0.004 - 0.011)	0.006 (0.004 - 0.011)	0.006 (0.004 - 0.011)	0.902 (0.339 - 1.900)	0.900 (0.330 - 1.888)	0.895 (0.338 - 1.888)
All ages (rate per capita)	0.035 (0.027 - 0.047)	0.043 (0.032 - 0.057)	0.050 (0.037 - 0.066)	0.003 (0.002 - 0.004)	0.004 (0.003 - 0.005)	0.004 (0.003 - 0.005)	0.233 (0.086 - 0.507)	0.283 (0.104 - 0.619)	0.325 (0.120 - 0.709)
Age-standardized (rate per capita)	0.056 (0.042 - 0.074)	0.056 (0.042 - 0.073)	0.056 (0.042 - 0.073)	0.004 (0.003 - 0.006)	0.004 (0.003 - 0.006)	0.004 (0.003 - 0.006)	0.366 (0.136 - 0.796)	0.364 (0.136 - 0.787)	0.362 (0.136 - 0.794)
Total number of cases (thousands)	644 (489 - 852)	1,108 (832 - 1,471)	1,503 (1,126 - 1,998)	58 (44 - 76)	97 (71 - 126)	127 (93 - 166)	4 (2 - 9)	7 (3 - 16)	10 (4 - 21)
**Maldives**									
15 - 49 years (rate per capita)	0.033 (0.024 - 0.047)	0.034 (0.025 - 0.049)	0.038 (0.028 - 0.055)	0.004 (0.003 - 0.006)	0.004 (0.003 - 0.006)	0.004 (0.003 - 0.006)	0.219 (0.078 - 0.472)	0.229 (0.082 - 0.512)	0.258 (0.091 - 0.553)
50 - 69 years (rate per capita)	0.146 (0.101 - 0.200)	0.147 (0.102 - 0.196)	0.142 (0.097 - 0.195)	0.009 (0.005 - 0.013)	0.009 (0.005 - 0.013)	0.009 (0.006 - 0.013)	0.952 (0.351 - 2.156)	0.956 (0.358 - 2.153)	0.930 (0.346 - 2.125)
70+ years (rate per capita)	0.156 (0.113 - 0.208)	0.152 (0.109 - 0.203)	0.148 (0.106 - 0.194)	0.007 (0.004 - 0.012)	0.007 (0.004 - 0.011)	0.006 (0.004 - 0.010)	0.977 (0.369 - 2.093)	0.957 (0.353 - 2.084)	0.929 (0.344 - 1.983)
All ages (rate per capita)	0.030 (0.023 - 0.040)	0.035 (0.027 - 0.047)	0.043 (0.033 - 0.058)	0.003 (0.002 - 0.003)	0.003 (0.002 - 0.004)	0.004 (0.003 - 0.005)	0.196 (0.072 - 0.424)	0.233 (0.087 - 0.501)	0.284 (0.104 - 0.621)
Age-standardized (rate per capita)	0.058 (0.043 - 0.076)	0.057 (0.042 - 0.074)	0.056 (0.042 - 0.075)	0.004 (0.003 - 0.006)	0.004 (0.003 - 0.006)	0.004 (0.003 - 0.006)	0.374 (0.140 - 0.808)	0.371 (0.139 - 0.810)	0.369 (0.137 - 0.808)
Total number of cases (thousands)	7 (5 - 9)	11 (8 - 14)	16 (12 - 21)	1 (0 - 1)	1 (1 - 1)	1 (1 - 2)	0 (0 - 0)	0 (0 - 0)	0 (0 - 0)
**Mauritius**									
15 - 49 years (rate per capita)	0.038 (0.028 - 0.054)	0.045 (0.033 - 0.066)	0.045 (0.032 - 0.065)	0.004 (0.003 - 0.006)	0.005 (0.003 - 0.007)	0.005 (0.003 - 0.007)	0.253 (0.090 - 0.559)	0.303 (0.105 - 0.672)	0.299 (0.104 - 0.647)
50 - 69 years (rate per capita)	0.140 (0.096 - 0.188)	0.137 (0.094 - 0.186)	0.140 (0.096 - 0.187)	0.009 (0.005 - 0.013)	0.009 (0.005 - 0.013)	0.009 (0.005 - 0.013)	0.911 (0.337 - 2.081)	0.895 (0.332 - 1.974)	0.906 (0.338 - 2.039)
70+ years (rate per capita)	0.134 (0.097 - 0.178)	0.132 (0.095 - 0.174)	0.132 (0.095 - 0.173)	0.007 (0.004 - 0.011)	0.006 (0.004 - 0.011)	0.006 (0.004 - 0.010)	0.845 (0.310 - 1.763)	0.830 (0.306 - 1.727)	0.826 (0.310 - 1.754)
All ages (rate per capita)	0.041 (0.031 - 0.054)	0.053 (0.039 - 0.070)	0.063 (0.046 - 0.082)	0.004 (0.003 - 0.005)	0.004 (0.003 - 0.006)	0.005 (0.004 - 0.006)	0.270 (0.099 - 0.582)	0.345 (0.127 - 0.758)	0.409 (0.152 - 0.898)
Age-standardized (rate per capita)	0.055 (0.041 - 0.072)	0.054 (0.041 - 0.071)	0.054 (0.041 - 0.071)	0.004 (0.003 - 0.006)	0.004 (0.003 - 0.006)	0.004 (0.003 - 0.006)	0.357 (0.132 - 0.775)	0.355 (0.131 - 0.769)	0.355 (0.133 - 0.777)
Total number of cases (thousands)	44 (33 - 57)	64 (48 - 85)	80 (59 - 105)	4 (3 - 5)	5 (4 - 7)	6 (5 - 8)	0 (0 - 1)	0 (0 - 1)	1 (0 - 1)
**Myanmar**									
15 - 49 years (rate per capita)	0.035 (0.026 - 0.051)	0.040 (0.028 - 0.058)	0.042 (0.030 - 0.062)	0.004 (0.003 - 0.006)	0.004 (0.003 - 0.006)	0.005 (0.003 - 0.007)	0.233 (0.082 - 0.505)	0.265 (0.093 - 0.591)	0.283 (0.097 - 0.639)
50 - 69 years (rate per capita)	0.150 (0.103 - 0.201)	0.148 (0.101 - 0.199)	0.147 (0.102 - 0.198)	0.009 (0.005 - 0.013)	0.009 (0.005 - 0.013)	0.009 (0.005 - 0.013)	0.972 (0.365 - 2.168)	0.960 (0.355 - 2.130)	0.955 (0.355 - 2.164)
70+ years (rate per capita)	0.160 (0.116 - 0.212)	0.159 (0.114 - 0.209)	0.157 (0.114 - 0.209)	0.006 (0.004 - 0.011)	0.006 (0.004 - 0.010)	0.006 (0.004 - 0.011)	0.993 (0.365 - 2.100)	0.983 (0.362 - 2.094)	0.977 (0.361 - 2.062)
All ages (rate per capita)	0.037 (0.028 - 0.049)	0.044 (0.033 - 0.059)	0.050 (0.038 - 0.068)	0.003 (0.002 - 0.004)	0.004 (0.003 - 0.005)	0.004 (0.003 - 0.005)	0.239 (0.088 - 0.521)	0.285 (0.105 - 0.618)	0.329 (0.119 - 0.710)

	Prevalence 1990	Prevalence 2005	Prevalence 2015	Incidence 1990	Incidence 2005	Incidence 2015	YLD rate 1990	YLD rate 2005	YLD rate 2015
**Age-standardized (rate per capita)**	0.058 (0.044 - 0.077)	0.058 (0.044 - 0.076)	0.058 (0.043 - 0.076)	0.004 (0.003 - 0.006)	0.004 (0.003 - 0.006)	0.004 (0.003 - 0.006)	0.378 (0.141 - 0.825)	0.378 (0.138 - 0.828)	0.375 (0.136 - 0.812)
**Total number of cases (thousands)**	1,540 (1,162 - 2,075)	2,182 (1,627 - 2,926)	2,716 (2,038 - 3,664)	131 (99 - 172)	184 (135 - 240)	220 (162 - 288)	10 (4 - 22)	14 (5 - 31)	18 (6 - 38)
**Philippines**									
**15 - 49 years (rate per capita)**	0.018 (0.015 - 0.021)	0.019 (0.016 - 0.023)	0.020 (0.017 - 0.024)	0.002 (0.002 - 0.003)	0.002 (0.002 - 0.003)	0.002 (0.002 - 0.003)	0.118 (0.044 - 0.247)	0.126 (0.048 - 0.265)	0.131 (0.050 - 0.277)
**50 - 69 years (rate per capita)**	0.067 (0.056 - 0.080)	0.067 (0.055 - 0.080)	0.067 (0.056 - 0.081)	0.005 (0.004 - 0.006)	0.005 (0.004 - 0.006)	0.005 (0.004 - 0.006)	0.434 (0.169 - 0.897)	0.430 (0.167 - 0.886)	0.434 (0.170 - 0.894)
**70+ years (rate per capita)**	0.071 (0.060 - 0.083)	0.070 (0.060 - 0.083)	0.070 (0.060 - 0.083)	0.004 (0.003 - 0.005)	0.004 (0.003 - 0.005)	0.004 (0.003 - 0.005)	0.439 (0.173 - 0.889)	0.436 (0.173 - 0.873)	0.436 (0.172 - 0.882)
**All ages (rate per capita)**	0.015 (0.013 - 0.018)	0.018 (0.015 - 0.021)	0.021 (0.018 - 0.024)	0.001 (0.001 - 0.002)	0.002 (0.001 - 0.002)	0.002 (0.002 - 0.002)	0.101 (0.039 - 0.212)	0.115 (0.045 - 0.241)	0.136 (0.052 - 0.285)
**Age-standardized (rate per capita)**	0.027 (0.024 - 0.032)	0.027 (0.024 - 0.031)	0.027 (0.024 - 0.032)	0.002 (0.002 - 0.003)	0.002 (0.002 - 0.003)	0.002 (0.002 - 0.003)	0.176 (0.069 - 0.367)	0.176 (0.069 - 0.364)	0.176 (0.069 - 0.368)
**Total number of cases (thousands)**	951 (822 - 1,110)	1,521 (1,314 - 1,779)	2,098 (1,811 - 2,452)	92 (79 - 107)	143 (124 - 166)	191 (165 - 221)	6 (2 - 13)	10 (4 - 21)	14 (5 - 29)
**Sri Lanka**									
**15 - 49 years (rate per capita)**	0.038 (0.028 - 0.054)	0.043 (0.031 - 0.061)	0.045 (0.032 - 0.065)	0.004 (0.003 - 0.006)	0.005 (0.003 - 0.007)	0.005 (0.003 - 0.007)	0.255 (0.090 - 0.559)	0.284 (0.099 - 0.631)	0.300 (0.104 - 0.659)
**50 - 69 years (rate per capita)**	0.148 (0.102 - 0.198)	0.146 (0.100 - 0.195)	0.147 (0.102 - 0.198)	0.009 (0.005 - 0.013)	0.009 (0.005 - 0.013)	0.009 (0.005 - 0.013)	0.965 (0.360 - 2.183)	0.948 (0.353 - 2.087)	0.955 (0.352 - 2.123)
**70+ years (rate per capita)**	0.153 (0.111 - 0.200)	0.150 (0.106 - 0.200)	0.150 (0.106 - 0.194)	0.006 (0.004 - 0.010)	0.006 (0.004 - 0.011)	0.006 (0.004 - 0.010)	0.962 (0.361 - 2.013)	0.939 (0.350 - 1.982)	0.936 (0.354 - 1.981)
**All ages (rate per capita)**	0.043 (0.032 - 0.056)	0.053 (0.040 - 0.069)	0.060 (0.045 - 0.078)	0.004 (0.003 - 0.005)	0.005 (0.003 - 0.005)	0.005 (0.003 - 0.006)	0.281 (0.104 - 0.619)	0.347 (0.129 - 0.756)	0.392 (0.144 - 0.855)
**Age-standardized (rate per capita)**	0.058 (0.043 - 0.075)	0.057 (0.043 - 0.074)	0.057 (0.043 - 0.074)	0.004 (0.003 - 0.006)	0.004 (0.003 - 0.006)	0.004 (0.003 - 0.006)	0.376 (0.139 - 0.819)	0.371 (0.138 - 0.812)	0.371 (0.136 - 0.804)
**Total number of cases (thousands)**	743 (562 - 976)	1,035 (776 - 1,350)	1,245 (930 - 1,624)	62 (46 - 80)	83 (60 - 107)	95 (68 - 123)	5 (2 - 11)	7 (3 - 15)	8 (3 - 18)
**Seychelles**									
**15 - 49 years (rate per capita)**	0.033 (0.024 - 0.047)	0.044 (0.031 - 0.062)	0.047 (0.034 - 0.068)	0.004 (0.003 - 0.006)	0.005 (0.003 - 0.007)	0.005 (0.003 - 0.007)	0.220 (0.080 - 0.478)	0.291 (0.101 - 0.643)	0.316 (0.110 - 0.689)
**50 - 69 years (rate per capita)**	0.146 (0.101 - 0.197)	0.143 (0.099 - 0.195)	0.143 (0.097 - 0.192)	0.009 (0.005 - 0.013)	0.009 (0.005 - 0.013)	0.009 (0.005 - 0.013)	0.951 (0.352 - 2.146)	0.935 (0.348 - 2.127)	0.935 (0.347 - 2.103)
**70+ years (rate per capita)**	0.142 (0.101 - 0.189)	0.143 (0.102 - 0.188)	0.141 (0.100 - 0.184)	0.006 (0.004 - 0.010)	0.006 (0.004 - 0.010)	0.006 (0.004 - 0.010)	0.893 (0.332 - 1.943)	0.894 (0.337 - 1.925)	0.880 (0.326 - 1.898)
**All ages (rate per capita)**	0.039 (0.029 - 0.050)	0.049 (0.037 - 0.065)	0.058 (0.043 - 0.077)	0.003 (0.002 - 0.004)	0.004 (0.003 - 0.006)	0.005 (0.003 - 0.006)	0.254 (0.094 - 0.551)	0.322 (0.118 - 0.704)	0.382 (0.141 - 0.841)
**Age-standardized (rate per capita)**	0.056 (0.042 - 0.074)	0.056 (0.042 - 0.075)	0.056 (0.042 - 0.074)	0.004 (0.003 - 0.006)	0.004 (0.003 - 0.006)	0.004 (0.003 - 0.006)	0.369 (0.137 - 0.808)	0.369 (0.137 - 0.804)	0.369 (0.137 - 0.806)
**Total number of cases (thousands)**	3 (2 - 4)	4 (3 - 6)	6 (4 - 7)	0 (0 - 0)	0 (0 - 0)	0 (0 - 1)	0 (0 - 0)	0 (0 - 0)	0 (0 - 0)
**Thailand**									
**15 - 49 years (rate per capita)**	0.046 (0.038 - 0.055)	0.057 (0.047 - 0.068)	0.060 (0.050 - 0.072)	0.005 (0.004 - 0.006)	0.006 (0.005 - 0.007)	0.006 (0.005 - 0.008)	0.305 (0.116 - 0.630)	0.380 (0.143 - 0.802)	0.402 (0.150 - 0.843)
**50 - 69 years (rate per capita)**	0.166 (0.138 - 0.198)	0.166 (0.139 - 0.198)	0.166 (0.137 - 0.197)	0.010 (0.007 - 0.013)	0.010 (0.007 - 0.013)	0.010 (0.007 - 0.013)	1.081 (0.421 - 2.208)	1.083 (0.422 - 2.219)	1.083 (0.424 - 2.204)
**70+ years (rate per capita)**	0.157 (0.133 - 0.186)	0.158 (0.134 - 0.187)	0.155 (0.130 - 0.183)	0.006 (0.004 - 0.008)	0.006 (0.004 - 0.008)	0.006 (0.004 - 0.008)	0.991 (0.395 - 2.019)	0.997 (0.398 - 2.028)	0.974 (0.381 - 1.997)
**All ages (rate per capita)**	0.049 (0.043 - 0.057)	0.068 (0.059 - 0.078)	0.081 (0.070 - 0.094)	0.004 (0.004 - 0.005)	0.005 (0.005 - 0.006)	0.006 (0.005 - 0.007)	0.324 (0.127 - 0.672)	0.444 (0.174 - 0.911)	0.529 (0.204 - 1.073)
**Age-standardized (rate per capita)**	0.066 (0.058 - 0.076)	0.066 (0.058 - 0.076)	0.065 (0.057 - 0.076)	0.005 (0.004 - 0.006)	0.005 (0.004 - 0.006)	0.005 (0.004 - 0.006)	0.430 (0.169 - 0.892)	0.433 (0.171 - 0.879)	0.428 (0.167 - 0.872)
**Total number of cases (thousands)**	2,791 (2,434 - 3,217)	4,441 (3,846 - 5,139)	5,485 (4,749 - 6,381)	237 (206 - 273)	347 (301 - 399)	402 (345 - 465)	18 (7 - 38)	29 (11 - 60)	36 (14 - 73)
**Timor-Leste**									
**15 - 49 years (rate per capita)**	0.035 (0.026 - 0.050)	0.035 (0.025 - 0.051)	0.035 (0.025 - 0.050)	0.004 (0.003 - 0.006)	0.004 (0.003 - 0.006)	0.004 (0.003 - 0.006)	0.234 (0.083 - 0.510)	0.233 (0.082 - 0.509)	0.232 (0.082 - 0.504)
**50 - 69 years (rate per capita)**	0.142 (0.097 - 0.193)	0.144 (0.097 - 0.195)	0.146 (0.103 - 0.196)	0.009 (0.005 - 0.013)	0.009 (0.005 - 0.013)	0.009 (0.005 - 0.013)	0.929 (0.345 - 2.105)	0.941 (0.351 - 2.111)	0.951 (0.358 - 2.152)
**70+ years (rate per capita)**	0.151 (0.106 - 0.204)	0.150 (0.105 - 0.197)	0.149 (0.109 - 0.200)	0.007 (0.004 - 0.012)	0.007 (0.004 - 0.011)	0.007 (0.004 - 0.011)	0.946 (0.351 - 2.052)	0.939 (0.346 - 2.017)	0.939 (0.345 - 2.034)
**All ages (rate per capita)**	0.029 (0.022 - 0.040)	0.029 (0.021 - 0.039)	0.034 (0.026 - 0.045)	0.003 (0.002 - 0.004)	0.003 (0.002 - 0.003)	0.003 (0.002 - 0.004)	0.194 (0.071 - 0.420)	0.189 (0.070 - 0.412)	0.222 (0.082 - 0.484)
**Age-standardized (rate per capita)**	0.057 (0.042 - 0.074)	0.057 (0.042 - 0.075)	0.056 (0.042 - 0.074)	0.004 (0.003 - 0.006)	0.004 (0.003 - 0.006)	0.004 (0.003 - 0.006)	0.369 (0.137 - 0.807)	0.369 (0.137 - 0.813)	0.368 (0.137 - 0.802)
**Total number of cases (thousands)**	22 (17 - 30)	29 (21 - 39)	40 (31 - 54)	2 (2 - 3)	3 (2 - 3)	3 (3 - 4)	0 (0 - 0)	0 (0 - 0)	0 (0 - 1)
**Vietnam**									
**15 - 49 years (rate per capita)**	0.029 (0.024 - 0.035)	0.035 (0.029 - 0.041)	0.037 (0.031 - 0.044)	0.003 (0.003 - 0.004)	0.004 (0.003 - 0.005)	0.004 (0.003 - 0.005)	0.193 (0.074 - 0.404)	0.230 (0.088 - 0.480)	0.247 (0.094 - 0.518)
**50 - 69 years (rate per capita)**	0.122 (0.102 - 0.145)	0.119 (0.100 - 0.142)	0.119 (0.098 - 0.143)	0.008 (0.006 - 0.009)	0.008 (0.006 - 0.009)	0.008 (0.006 - 0.009)	0.792 (0.309 - 1.606)	0.775 (0.299 - 1.560)	0.777 (0.303 - 1.579)
**70+ years (rate per capita)**	0.123 (0.105 - 0.144)	0.121 (0.104 - 0.141)	0.118 (0.101 - 0.137)	0.005 (0.004 - 0.007)	0.005 (0.004 - 0.006)	0.005 (0.004 - 0.006)	0.768 (0.303 - 1.540)	0.756 (0.300 - 1.526)	0.736 (0.292 - 1.487)
**All ages (rate per capita)**	0.031 (0.027 - 0.036)	0.038 (0.034 - 0.045)	0.046 (0.039 - 0.053)	0.003 (0.002 - 0.003)	0.003 (0.003 - 0.004)	0.004 (0.003 - 0.004)	0.204 (0.079 - 0.422)	0.252 (0.098 - 0.523)	0.300 (0.117 - 0.625)
**Age-standardized (rate per capita)**	0.048 (0.042 - 0.055)	0.048 (0.041 - 0.055)	0.047 (0.041 - 0.055)	0.004 (0.003 - 0.004)	0.004 (0.003 - 0.004)	0.004 (0.003 - 0.004)	0.311 (0.121 - 0.646)	0.311 (0.121 - 0.643)	0.310 (0.121 - 0.649)
**Total number of cases (thousands)**	2,127 (1,851 - 2,439)	3,238 (2,831 - 3,757)	4,270 (3,690 - 4,964)	179 (156 - 205)	273 (238 - 315)	346 (299 - 401)	14 (5 - 29)	21 (8 - 44)	28 (11 - 58)
**Oceania**									
**15 - 49 years (rate per capita)**	0.022 (0.018 - 0.026)	0.023 (0.020 - 0.028)	0.024 (0.020 - 0.029)	0.003 (0.002 - 0.003)	0.003 (0.002 - 0.003)	0.003 (0.002 - 0.003)	0.143 (0.054 - 0.299)	0.154 (0.059 - 0.322)	0.158 (0.060 - 0.332)
**50 - 69 years (rate per capita)**	0.085 (0.070 - 0.102)	0.084 (0.070 - 0.101)	0.084 (0.070 - 0.101)	0.006 (0.004 - 0.007)	0.006 (0.004 - 0.007)	0.006 (0.004 - 0.007)	0.546 (0.212 - 1.109)	0.542 (0.213 - 1.101)	0.542 (0.211 - 1.097)
**70+ years (rate per capita)**	0.091 (0.077 - 0.107)	0.090 (0.077 - 0.107)	0.090 (0.076 - 0.106)	0.004 (0.003 - 0.006)	0.004 (0.003 - 0.006)	0.004 (0.003 - 0.006)	0.562 (0.220 - 1.142)	0.558 (0.218 - 1.121)	0.554 (0.219 - 1.125)
**All ages (rate per capita)**	0.019 (0.016 - 0.022)	0.021 (0.019 - 0.025)	0.024 (0.020 - 0.027)	0.002 (0.002 - 0.002)	0.002 (0.002 - 0.002)	0.002 (0.002 - 0.002)	0.123 (0.048 - 0.255)	0.139 (0.054 - 0.287)	0.153 (0.060 - 0.318)
**Age-standardized (rate per capita)**	0.034 (0.030 - 0.039)	0.034 (0.030 - 0.039)	0.034 (0.030 - 0.039)	0.003 (0.002 - 0.003)	0.003 (0.002 - 0.003)	0.003 (0.002 - 0.003)	0.219 (0.087 - 0.457)	0.219 (0.085 - 0.452)	0.219 (0.086 - 0.451)
**Total number of cases (thousands)**	125 (108 - 146)	195 (169 - 227)	258 (224 - 301)	12 (10 - 13)	18 (15 - 21)	23 (20 - 27)	1 (0 - 2)	1 (0 - 3)	2 (1 - 3)
**American Samoa**									
**15 - 49 years (rate per capita)**	0.022 (0.019 - 0.026)	0.026 (0.022 - 0.031)	0.029 (0.024 - 0.035)	0.003 (0.002 - 0.003)	0.003 (0.002 - 0.004)	0.003 (0.003 - 0.004)	0.148 (0.057 - 0.309)	0.174 (0.066 - 0.364)	0.192 (0.073 - 0.403)
**50 - 69 years (rate per capita)**	0.085 (0.071 - 0.103)	0.084 (0.070 - 0.102)	0.084 (0.070 - 0.101)	0.006 (0.004 - 0.007)	0.006 (0.004 - 0.007)	0.006 (0.004 - 0.007)	0.551 (0.214 - 1.120)	0.545 (0.212 - 1.108)	0.543 (0.211 - 1.101)
**70+ years (rate per capita)**	0.090 (0.077 - 0.107)	0.089 (0.076 - 0.105)	0.088 (0.075 - 0.104)	0.004 (0.003 - 0.005)	0.004 (0.003 - 0.006)	0.004 (0.003 - 0.006)	0.559 (0.223 - 1.133)	0.552 (0.218 - 1.117)	0.549 (0.218 - 1.119)
**All ages (rate per capita)**	0.020 (0.018 - 0.024)	0.023 (0.020 - 0.027)	0.024 (0.020 - 0.028)	0.002 (0.002 - 0.002)	0.002 (0.002 - 0.002)	0.002 (0.002 - 0.002)	0.133 (0.052 - 0.272)	0.149 (0.058 - 0.309)	0.156 (0.061 - 0.331)
**Age-standardized (rate per capita)**	0.034 (0.030 - 0.040)	0.034 (0.029 - 0.040)	0.034 (0.029 - 0.039)	0.003 (0.002 - 0.003)	0.003 (0.002 - 0.003)	0.003 (0.002 - 0.003)	0.222 (0.087 - 0.454)	0.220 (0.086 - 0.453)	0.219 (0.086 - 0.456)
**Total number of cases (thousands)**	1 (1 - 1)	1 (1 - 2)	2 (2 - 2)	0 (0 - 0)	0 (0 - 0)	0 (0 - 0)	0 (0 - 0)	0 (0 - 0)	0 (0 - 0)
**Federated States of Micronesia**									
**15 - 49 years (rate per capita)**	0.020 (0.017 - 0.024)	0.021 (0.018 - 0.025)	0.019 (0.016 - 0.023)	0.002 (0.002 - 0.003)	0.002 (0.002 - 0.003)	0.002 (0.002 - 0.003)	0.136 (0.052 - 0.280)	0.140 (0.053 - 0.294)	0.127 (0.049 - 0.265)
**50 - 69 years (rate per capita)**	0.086 (0.071 - 0.102)	0.083 (0.069 - 0.100)	0.085 (0.071 - 0.102)	0.006 (0.004 - 0.007)	0.006 (0.004 - 0.007)	0.006 (0.004 - 0.007)	0.555 (0.217 - 1.121)	0.536 (0.206 - 1.092)	0.548 (0.213 - 1.121)
**70+ years (rate per capita)**	0.091 (0.078 - 0.108)	0.088 (0.076 - 0.104)	0.088 (0.075 - 0.104)	0.004 (0.003 - 0.006)	0.004 (0.003 - 0.005)	0.004 (0.003 - 0.005)	0.569 (0.222 - 1.142)	0.547 (0.215 - 1.113)	0.544 (0.215 - 1.109)
**All ages (rate per capita)**	0.018 (0.015 - 0.020)	0.020 (0.018 - 0.024)	0.022 (0.019 - 0.026)	0.002 (0.001 - 0.002)	0.002 (0.002 - 0.002)	0.002 (0.002 - 0.002)	0.116 (0.045 - 0.236)	0.132 (0.051 - 0.272)	0.145 (0.056 - 0.298)
**Age-standardized (rate per capita)**	0.034 (0.030 - 0.039)	0.034 (0.029 - 0.039)	0.034 (0.029 - 0.039)	0.003 (0.002 - 0.003)	0.003 (0.002 - 0.003)	0.003 (0.002 - 0.003)	0.221 (0.086 - 0.455)	0.219 (0.085 - 0.445)	0.218 (0.085 - 0.449)
**Total number of cases (thousands)**	2 (1 - 2)	2 (2 - 3)	2 (2 - 3)	0 (0 - 0)	0 (0 - 0)	0 (0 - 0)	0 (0 - 0)	0 (0 - 0)	0 (0 - 0)
**Fiji**									
**15 - 49 years (rate per capita)**	0.022 (0.018 - 0.026)	0.026 (0.021 - 0.031)	0.025 (0.021 - 0.030)	0.003 (0.002 - 0.003)	0.003 (0.002 - 0.003)	0.003 (0.002 - 0.003)	0.144 (0.055 - 0.296)	0.170 (0.064 - 0.366)	0.168 (0.064 - 0.352)
**50 - 69 years (rate per capita)**	0.082 (0.068 - 0.098)	0.084 (0.070 - 0.101)	0.084 (0.070 - 0.101)	0.006 (0.004 - 0.007)	0.006 (0.004 - 0.007)	0.006 (0.004 - 0.007)	0.527 (0.206 - 1.064)	0.539 (0.210 - 1.105)	0.539 (0.210 - 1.096)
**70+ years (rate per capita)**	0.090 (0.076 - 0.106)	0.090 (0.077 - 0.107)	0.090 (0.077 - 0.107)	0.004 (0.003 - 0.006)	0.004 (0.003 - 0.006)	0.004 (0.003 - 0.006)	0.555 (0.217 - 1.118)	0.559 (0.218 - 1.118)	0.557 (0.220 - 1.130)
**All ages (rate per capita)**	0.020 (0.017 - 0.023)	0.027 (0.023 - 0.031)	0.030 (0.026 - 0.035)	0.002 (0.002 - 0.002)	0.002 (0.002 - 0.003)	0.003 (0.002 - 0.003)	0.128 (0.050 - 0.268)	0.173 (0.067 - 0.359)	0.193 (0.075 - 0.397)
**Age-standardized (rate per capita)**	0.033 (0.029 - 0.038)	0.034 (0.029 - 0.039)	0.034 (0.029 - 0.039)	0.003 (0.002 - 0.003)	0.003 (0.002 - 0.003)	0.003 (0.002 - 0.003)	0.213 (0.083 - 0.441)	0.218 (0.085 - 0.452)	0.217 (0.085 - 0.449)
**Total number of cases (thousands)**	14 (13 - 17)	22 (19 - 26)	27 (23 - 31)	1 (1 - 2)	2 (2 - 2)	2 (2 - 3)	0 (0 - 0)	0 (0 - 0)	0 (0 - 0)
**Guam**									
**15 - 49 years (rate per capita)**	0.024 (0.020 - 0.028)	0.027 (0.023 - 0.033)	0.026 (0.022 - 0.032)	0.003 (0.002 - 0.003)	0.003 (0.002 - 0.004)	0.003 (0.002 - 0.004)	0.158 (0.060 - 0.334)	0.181 (0.068 - 0.384)	0.175 (0.067 - 0.370)
**50 - 69 years (rate per capita)**	0.086 (0.071 - 0.102)	0.084 (0.070 - 0.101)	0.085 (0.070 - 0.101)	0.006 (0.004 - 0.007)	0.006 (0.004 - 0.007)	0.006 (0.004 - 0.007)	0.557 (0.218 - 1.118)	0.547 (0.213 - 1.105)	0.549 (0.212 - 1.122)

	Prevalence 1990	Prevalence 2005	Prevalence 2015	Incidence 1990	Incidence 2005	Incidence 2015	YLD rate 1990	YLD rate 2005	YLD rate 2015
70+ years (rate per capita)	0.090 (0.077 - 0.107)	0.089 (0.076 - 0.105)	0.088 (0.075 - 0.103)	0.004 (0.003 - 0.006)	0.004 (0.003 - 0.005)	0.004 (0.003 - 0.005)	0.565 (0.222 - 1.150)	0.557 (0.219 - 1.125)	0.548 (0.215 - 1.108)
All ages (rate per capita)	0.025 (0.022 - 0.029)	0.030 (0.026 - 0.035)	0.034 (0.030 - 0.040)	0.002 (0.002 - 0.003)	0.003 (0.002 - 0.003)	0.003 (0.002 - 0.003)	0.163 (0.063 - 0.340)	0.197 (0.077 - 0.405)	0.222 (0.086 - 0.465)
Age-standardized (rate per capita)	0.034 (0.030 - 0.039)	0.034 (0.029 - 0.039)	0.034 (0.029 - 0.039)	0.003 (0.002 - 0.003)	0.003 (0.002 - 0.003)	0.003 (0.002 - 0.003)	0.223 (0.087 - 0.461)	0.220 (0.086 - 0.448)	0.220 (0.086 - 0.456)
Total number of cases (thousands)	3 (3 - 4)	5 (4 - 6)	6 (5 - 7)	0 (0 - 0)	0 (0 - 0)	0 (0 - 1)	0 (0 - 0)	0 (0 - 0)	0 (0 - 0)
**Kiribati**									
15 - 49 years (rate per capita)	0.022 (0.019 - 0.027)	0.023 (0.019 - 0.028)	0.023 (0.019 - 0.028)	0.003 (0.002 - 0.003)	0.003 (0.002 - 0.003)	0.003 (0.002 - 0.003)	0.149 (0.057 - 0.310)	0.154 (0.059 - 0.323)	0.154 (0.059 - 0.320)
50 - 69 years (rate per capita)	0.086 (0.071 - 0.103)	0.085 (0.070 - 0.101)	0.084 (0.070 - 0.101)	0.006 (0.004 - 0.007)	0.006 (0.004 - 0.007)	0.006 (0.004 - 0.007)	0.549 (0.215 - 1.107)	0.542 (0.211 - 1.101)	0.539 (0.211 - 1.085)
70+ years (rate per capita)	0.091 (0.077 - 0.108)	0.091 (0.077 - 0.107)	0.090 (0.077 - 0.107)	0.004 (0.003 - 0.006)	0.004 (0.003 - 0.006)	0.004 (0.003 - 0.006)	0.564 (0.220 - 1.154)	0.561 (0.223 - 1.147)	0.556 (0.219 - 1.119)
All ages (rate per capita)	0.021 (0.018 - 0.024)	0.022 (0.019 - 0.025)	0.024 (0.020 - 0.028)	0.002 (0.002 - 0.002)	0.002 (0.002 - 0.002)	0.002 (0.002 - 0.002)	0.133 (0.052 - 0.275)	0.140 (0.054 - 0.287)	0.153 (0.059 - 0.319)
Age-standardized (rate per capita)	0.034 (0.030 - 0.040)	0.034 (0.030 - 0.039)	0.034 (0.029 - 0.039)	0.003 (0.002 - 0.003)	0.003 (0.002 - 0.003)	0.003 (0.002 - 0.003)	0.221 (0.086 - 0.459)	0.219 (0.085 - 0.452)	0.219 (0.085 - 0.451)
Total number of cases (thousands)	1 (1 - 2)	2 (2 - 2)	3 (2 - 3)	0 (0 - 0)	0 (0 - 0)	0 (0 - 0)	0 (0 - 0)	0 (0 - 0)	0 (0 - 0)
**Marshall Islands**									
15 - 49 years (rate per capita)	0.022 (0.018 - 0.026)	0.023 (0.019 - 0.028)	0.024 (0.020 - 0.028)	0.003 (0.002 - 0.003)	0.003 (0.002 - 0.003)	0.003 (0.002 - 0.003)	0.143 (0.055 - 0.299)	0.152 (0.058 - 0.322)	0.156 (0.060 - 0.329)
50 - 69 years (rate per capita)	0.085 (0.070 - 0.102)	0.084 (0.070 - 0.100)	0.084 (0.069 - 0.100)	0.006 (0.004 - 0.007)	0.006 (0.004 - 0.007)	0.006 (0.004 - 0.007)	0.543 (0.212 - 1.106)	0.535 (0.209 - 1.085)	0.535 (0.211 - 1.102)
70+ years (rate per capita)	0.091 (0.077 - 0.108)	0.090 (0.077 - 0.105)	0.089 (0.076 - 0.105)	0.004 (0.003 - 0.006)	0.004 (0.003 - 0.006)	0.004 (0.003 - 0.005)	0.560 (0.219 - 1.138)	0.551 (0.219 - 1.109)	0.548 (0.216 - 1.101)
All ages (rate per capita)	0.018 (0.016 - 0.021)	0.021 (0.018 - 0.024)	0.023 (0.020 - 0.027)	0.002 (0.001 - 0.002)	0.002 (0.002 - 0.002)	0.002 (0.002 - 0.002)	0.119 (0.046 - 0.242)	0.135 (0.053 - 0.277)	0.150 (0.058 - 0.312)
Age-standardized (rate per capita)	0.034 (0.030 - 0.040)	0.034 (0.029 - 0.039)	0.034 (0.029 - 0.039)	0.003 (0.002 - 0.003)	0.003 (0.002 - 0.003)	0.003 (0.002 - 0.003)	0.219 (0.086 - 0.449)	0.217 (0.085 - 0.444)	0.216 (0.086 - 0.448)
Total number of cases (thousands)	1 (1 - 1)	1 (1 - 1)	2 (1 - 2)	0 (0 - 0)	0 (0 - 0)	0 (0 - 0)	0 (0 - 0)	0 (0 - 0)	0 (0 - 0)
**Northern Mariana Islands**									
15 - 49 years (rate per capita)	0.025 (0.020 - 0.030)	0.026 (0.022 - 0.031)	0.027 (0.022 - 0.032)	0.003 (0.002 - 0.003)	0.003 (0.002 - 0.004)	0.003 (0.002 - 0.004)	0.165 (0.063 - 0.347)	0.174 (0.066 - 0.368)	0.177 (0.067 - 0.368)
50 - 69 years (rate per capita)	0.085 (0.070 - 0.102)	0.083 (0.069 - 0.102)	0.083 (0.068 - 0.100)	0.006 (0.004 - 0.007)	0.006 (0.004 - 0.007)	0.006 (0.004 - 0.007)	0.551 (0.213 - 1.107)	0.541 (0.208 - 1.098)	0.538 (0.208 - 1.083)
70+ years (rate per capita)	0.089 (0.076 - 0.106)	0.089 (0.076 - 0.105)	0.088 (0.076 - 0.104)	0.004 (0.003 - 0.005)	0.004 (0.003 - 0.005)	0.004 (0.003 - 0.006)	0.558 (0.219 - 1.134)	0.555 (0.218 - 1.124)	0.552 (0.216 - 1.108)
All ages (rate per capita)	0.024 (0.020 - 0.028)	0.025 (0.021 - 0.029)	0.026 (0.022 - 0.030)	0.002 (0.002 - 0.003)	0.002 (0.002 - 0.003)	0.003 (0.002 - 0.003)	0.156 (0.060 - 0.324)	0.164 (0.062 - 0.349)	0.170 (0.065 - 0.354)
Age-standardized (rate per capita)	0.034 (0.030 - 0.040)	0.034 (0.030 - 0.039)	0.034 (0.030 - 0.039)	0.003 (0.002 - 0.003)	0.003 (0.002 - 0.003)	0.003 (0.002 - 0.003)	0.224 (0.088 - 0.463)	0.222 (0.087 - 0.460)	0.222 (0.087 - 0.462)
Total number of cases (thousands)	1 (1 - 1)	2 (2 - 2)	3 (3 - 4)	0 (0 - 0)	0 (0 - 0)	0 (0 - 0)	0 (0 - 0)	0 (0 - 0)	0 (0 - 0)
**Papua New Guinea**									
15 - 49 years (rate per capita)	0.021 (0.018 - 0.025)	0.023 (0.019 - 0.027)	0.024 (0.019 - 0.028)	0.003 (0.002 - 0.003)	0.003 (0.002 - 0.003)	0.003 (0.002 - 0.003)	0.142 (0.054 - 0.297)	0.149 (0.057 - 0.312)	0.156 (0.060 - 0.327)
50 - 69 years (rate per capita)	0.085 (0.070 - 0.102)	0.084 (0.070 - 0.101)	0.084 (0.070 - 0.101)	0.006 (0.004 - 0.007)	0.006 (0.004 - 0.007)	0.006 (0.004 - 0.007)	0.546 (0.212 - 1.114)	0.542 (0.213 - 1.095)	0.541 (0.210 - 1.096)
70+ years (rate per capita)	0.091 (0.077 - 0.108)	0.091 (0.077 - 0.107)	0.090 (0.076 - 0.107)	0.004 (0.003 - 0.006)	0.004 (0.003 - 0.006)	0.004 (0.003 - 0.006)	0.562 (0.221 - 1.145)	0.559 (0.217 - 1.121)	0.556 (0.221 - 1.118)
All ages (rate per capita)	0.018 (0.016 - 0.021)	0.020 (0.017 - 0.023)	0.022 (0.019 - 0.025)	0.002 (0.001 - 0.002)	0.002 (0.002 - 0.002)	0.002 (0.002 - 0.002)	0.118 (0.046 - 0.245)	0.129 (0.050 - 0.265)	0.141 (0.055 - 0.293)
Age-standardized (rate per capita)	0.034 (0.030 - 0.040)	0.034 (0.030 - 0.039)	0.034 (0.030 - 0.039)	0.003 (0.002 - 0.003)	0.003 (0.002 - 0.003)	0.003 (0.002 - 0.003)	0.220 (0.086 - 0.459)	0.219 (0.085 - 0.451)	0.218 (0.085 - 0.450)
Total number of cases (thousands)	76 (65 - 88)	121 (104 - 140)	165 (143 - 194)	7 (6 - 8)	11 (10 - 13)	15 (13 - 18)	0 (0 - 1)	1 (0 - 2)	1 (0 - 2)
**Samoa**									
15 - 49 years (rate per capita)	0.020 (0.017 - 0.024)	0.024 (0.020 - 0.029)	0.024 (0.020 - 0.028)	0.002 (0.002 - 0.003)	0.003 (0.002 - 0.003)	0.003 (0.002 - 0.003)	0.132 (0.050 - 0.278)	0.159 (0.060 - 0.337)	0.157 (0.059 - 0.330)
50 - 69 years (rate per capita)	0.086 (0.072 - 0.103)	0.085 (0.070 - 0.101)	0.085 (0.070 - 0.101)	0.006 (0.004 - 0.007)	0.006 (0.004 - 0.007)	0.006 (0.004 - 0.007)	0.555 (0.218 - 1.123)	0.549 (0.212 - 1.122)	0.546 (0.212 - 1.109)
70+ years (rate per capita)	0.091 (0.078 - 0.107)	0.089 (0.076 - 0.105)	0.088 (0.075 - 0.103)	0.004 (0.003 - 0.006)	0.004 (0.003 - 0.005)	0.004 (0.003 - 0.005)	0.566 (0.221 - 1.145)	0.554 (0.216 - 1.112)	0.543 (0.214 - 1.102)
All ages (rate per capita)	0.019 (0.017 - 0.022)	0.022 (0.020 - 0.026)	0.024 (0.021 - 0.028)	0.002 (0.001 - 0.002)	0.002 (0.002 - 0.002)	0.002 (0.002 - 0.002)	0.124 (0.049 - 0.255)	0.147 (0.057 - 0.308)	0.159 (0.062 - 0.332)
Age-standardized (rate per capita)	0.034 (0.030 - 0.040)	0.034 (0.030 - 0.039)	0.034 (0.030 - 0.039)	0.003 (0.002 - 0.003)	0.003 (0.002 - 0.003)	0.003 (0.002 - 0.003)	0.221 (0.087 - 0.454)	0.220 (0.086 - 0.459)	0.220 (0.086 - 0.462)
Total number of cases (thousands)	3 (3 - 4)	4 (4 - 5)	5 (4 - 6)	0 (0 - 0)	0 (0 - 0)	0 (0 - 0)	0 (0 - 0)	0 (0 - 0)	0 (0 - 0)
**Solomon Islands**									
15 - 49 years (rate per capita)	0.021 (0.018 - 0.025)	0.022 (0.018 - 0.026)	0.023 (0.019 - 0.027)	0.002 (0.002 - 0.003)	0.003 (0.002 - 0.003)	0.003 (0.002 - 0.003)	0.140 (0.053 - 0.294)	0.144 (0.055 - 0.300)	0.152 (0.058 - 0.312)
50 - 69 years (rate per capita)	0.085 (0.071 - 0.102)	0.085 (0.070 - 0.102)	0.084 (0.070 - 0.102)	0.006 (0.004 - 0.007)	0.006 (0.004 - 0.007)	0.006 (0.004 - 0.007)	0.552 (0.212 - 1.133)	0.547 (0.215 - 1.118)	0.545 (0.212 - 1.098)
70+ years (rate per capita)	0.092 (0.078 - 0.108)	0.091 (0.077 - 0.108)	0.090 (0.077 - 0.108)	0.004 (0.003 - 0.006)	0.004 (0.003 - 0.006)	0.004 (0.003 - 0.006)	0.574 (0.225 - 1.152)	0.566 (0.221 - 1.160)	0.562 (0.218 - 1.144)
All ages (rate per capita)	0.017 (0.015 - 0.020)	0.019 (0.016 - 0.022)	0.021 (0.018 - 0.024)	0.002 (0.001 - 0.002)	0.002 (0.002 - 0.002)	0.002 (0.002 - 0.002)	0.113 (0.044 - 0.235)	0.124 (0.048 - 0.255)	0.134 (0.051 - 0.279)
Age-standardized (rate per capita)	0.034 (0.030 - 0.040)	0.034 (0.029 - 0.039)	0.034 (0.029 - 0.039)	0.003 (0.002 - 0.003)	0.003 (0.002 - 0.003)	0.003 (0.002 - 0.003)	0.222 (0.086 - 0.459)	0.220 (0.086 - 0.458)	0.220 (0.085 - 0.450)
Total number of cases (thousands)	5 (5 - 6)	9 (8 - 11)	12 (10 - 14)	1 (0 - 1)	1 (1 - 1)	1 (1 - 1)	0 (0 - 0)	0 (0 - 0)	0 (0 - 0)
**Tonga**									
15 - 49 years (rate per capita)	0.020 (0.016 - 0.024)	0.023 (0.019 - 0.027)	0.023 (0.019 - 0.028)	0.002 (0.002 - 0.003)	0.003 (0.002 - 0.003)	0.003 (0.002 - 0.003)	0.131 (0.050 - 0.278)	0.153 (0.058 - 0.321)	0.153 (0.058 - 0.320)
50 - 69 years (rate per capita)	0.086 (0.071 - 0.102)	0.085 (0.071 - 0.102)	0.084 (0.070 - 0.101)	0.006 (0.004 - 0.007)	0.006 (0.004 - 0.007)	0.006 (0.004 - 0.007)	0.551 (0.217 - 1.111)	0.550 (0.215 - 1.121)	0.543 (0.211 - 1.102)
70+ years (rate per capita)	0.090 (0.077 - 0.106)	0.089 (0.076 - 0.105)	0.087 (0.075 - 0.102)	0.004 (0.003 - 0.006)	0.004 (0.003 - 0.005)	0.004 (0.003 - 0.005)	0.557 (0.219 - 1.109)	0.549 (0.217 - 1.113)	0.537 (0.213 - 1.088)
All ages (rate per capita)	0.020 (0.018 - 0.023)	0.023 (0.020 - 0.027)	0.024 (0.021 - 0.028)	0.002 (0.002 - 0.002)	0.002 (0.002 - 0.002)	0.002 (0.002 - 0.002)	0.132 (0.051 - 0.271)	0.150 (0.058 - 0.313)	0.156 (0.061 - 0.324)
Age-standardized (rate per capita)	0.034 (0.029 - 0.039)	0.034 (0.029 - 0.039)	0.034 (0.029 - 0.039)	0.003 (0.002 - 0.003)	0.003 (0.002 - 0.003)	0.003 (0.002 - 0.003)	0.220 (0.086 - 0.449)	0.219 (0.085 - 0.458)	0.218 (0.085 - 0.453)
Total number of cases (thousands)	2 (2 - 2)	2 (2 - 3)	3 (2 - 3)	0 (0 - 0)	0 (0 - 0)	0 (0 - 0)	0 (0 - 0)	0 (0 - 0)	0 (0 - 0)
**Vanuatu**									
15 - 49 years (rate per capita)	0.022 (0.018 - 0.026)	0.022 (0.019 - 0.027)	0.023 (0.019 - 0.028)	0.003 (0.002 - 0.003)	0.003 (0.002 - 0.003)	0.003 (0.002 - 0.003)	0.147 (0.055 - 0.307)	0.148 (0.056 - 0.307)	0.154 (0.059 - 0.323)
50 - 69 years (rate per capita)	0.085 (0.071 - 0.102)	0.084 (0.070 - 0.101)	0.084 (0.069 - 0.101)	0.006 (0.004 - 0.007)	0.006 (0.004 - 0.007)	0.006 (0.004 - 0.007)	0.553 (0.214 - 1.119)	0.546 (0.211 - 1.112)	0.541 (0.209 - 1.111)
70+ years (rate per capita)	0.091 (0.077 - 0.107)	0.090 (0.077 - 0.106)	0.091 (0.077 - 0.107)	0.004 (0.003 - 0.006)	0.004 (0.003 - 0.005)	0.004 (0.003 - 0.006)	0.564 (0.222 - 1.147)	0.561 (0.221 - 1.134)	0.566 (0.221 - 1.156)
All ages (rate per capita)	0.019 (0.016 - 0.022)	0.020 (0.018 - 0.024)	0.023 (0.020 - 0.027)	0.002 (0.001 - 0.002)	0.002 (0.002 - 0.002)	0.002 (0.002 - 0.002)	0.123 (0.047 - 0.255)	0.133 (0.051 - 0.277)	0.151 (0.059 - 0.312)
Age-standardized (rate per capita)	0.034 (0.030 - 0.039)	0.034 (0.029 - 0.039)	0.034 (0.029 - 0.039)	0.003 (0.002 - 0.003)	0.003 (0.002 - 0.003)	0.003 (0.002 - 0.003)	0.221 (0.086 - 0.460)	0.220 (0.086 - 0.456)	0.219 (0.085 - 0.456)
Total number of cases (thousands)	3 (2 - 3)	4 (4 - 5)	6 (5 - 7)	0 (0 - 0)	0 (0 - 0)	1 (0 - 1)	0 (0 - 0)	0 (0 - 0)	0 (0 - 0)
**North Africa and Middle East**									
15 - 49 years (rate per capita)	0.061 (0.051 - 0.074)	0.064 (0.053 - 0.077)	0.069 (0.057 - 0.084)	0.007 (0.006 - 0.008)	0.007 (0.006 - 0.009)	0.007 (0.006 - 0.009)	0.408 (0.154 - 0.846)	0.421 (0.159 - 0.889)	0.460 (0.173 - 0.965)
50 - 69 years (rate per capita)	0.196 (0.161 - 0.235)	0.193 (0.159 - 0.232)	0.194 (0.159 - 0.233)	0.012 (0.008 - 0.015)	0.012 (0.009 - 0.015)	0.012 (0.009 - 0.015)	1.264 (0.492 - 2.568)	1.249 (0.485 - 2.537)	1.251 (0.484 - 2.553)
70+ years (rate per capita)	0.158 (0.134 - 0.187)	0.160 (0.135 - 0.190)	0.158 (0.134 - 0.187)	0.007 (0.005 - 0.010)	0.007 (0.005 - 0.010)	0.007 (0.005 - 0.010)	0.980 (0.387 - 1.970)	0.992 (0.395 - 2.000)	0.977 (0.387 - 1.966)
All ages (rate per capita)	0.050 (0.043 - 0.059)	0.058 (0.050 - 0.068)	0.066 (0.056 - 0.078)	0.004 (0.004 - 0.005)	0.005 (0.004 - 0.006)	0.006 (0.005 - 0.007)	0.327 (0.128 - 0.677)	0.380 (0.147 - 0.781)	0.429 (0.166 - 0.888)
Age-standardized (rate per capita)	0.081 (0.070 - 0.095)	0.081 (0.069 - 0.095)	0.081 (0.069 - 0.095)	0.006 (0.005 - 0.007)	0.006 (0.005 - 0.007)	0.006 (0.005 - 0.007)	0.527 (0.205 - 1.085)	0.523 (0.203 - 1.074)	0.524 (0.204 - 1.079)
Total number of cases (thousands)	16,826 (14,485 - 19,757)	26,994 (23,258 - 31,685)	37,147 (31,691 - 43,952)	1,485 (1,284 - 1,734)	2,388 (2,062 - 2,797)	3,173 (2,728 - 3,743)	110 (43 - 227)	176 (68 - 363)	243 (94 - 503)
**North Africa and Middle East**									
15 - 49 years (rate per capita)	0.061 (0.051 - 0.074)	0.064 (0.053 - 0.077)	0.069 (0.057 - 0.084)	0.007 (0.006 - 0.008)	0.007 (0.006 - 0.009)	0.007 (0.006 - 0.009)	0.408 (0.154 - 0.846)	0.421 (0.159 - 0.889)	0.460 (0.173 - 0.965)
50 - 69 years (rate per capita)	0.196 (0.161 - 0.235)	0.193 (0.159 - 0.232)	0.194 (0.159 - 0.233)	0.012 (0.008 - 0.015)	0.012 (0.009 - 0.015)	0.012 (0.009 - 0.015)	1.264 (0.492 - 2.568)	1.249 (0.485 - 2.537)	1.251 (0.484 - 2.553)
70+ years (rate per capita)	0.158 (0.134 - 0.187)	0.160 (0.135 - 0.190)	0.158 (0.134 - 0.187)	0.007 (0.005 - 0.010)	0.007 (0.005 - 0.010)	0.007 (0.005 - 0.010)	0.980 (0.387 - 1.970)	0.992 (0.395 - 2.000)	0.977 (0.387 - 1.966)
All ages (rate per capita)	0.050 (0.043 - 0.059)	0.058 (0.050 - 0.068)	0.066 (0.056 - 0.078)	0.004 (0.004 - 0.005)	0.005 (0.004 - 0.006)	0.006 (0.005 - 0.007)	0.327 (0.128 - 0.677)	0.380 (0.147 - 0.781)	0.429 (0.166 - 0.888)
Age-standardized (rate per capita)	0.081 (0.070 - 0.095)	0.081 (0.069 - 0.095)	0.081 (0.069 - 0.095)	0.006 (0.005 - 0.007)	0.006 (0.005 - 0.007)	0.006 (0.005 - 0.007)	0.527 (0.205 - 1.085)	0.523 (0.203 - 1.074)	0.524 (0.204 - 1.079)
Total number of cases (thousands)	16,826 (14,485 - 19,757)	26,994 (23,258 - 31,685)	37,147 (31,691 - 43,952)	1,485 (1,284 - 1,734)	2,388 (2,062 - 2,797)	3,173 (2,728 - 3,743)	110 (43 - 227)	176 (68 - 363)	243 (94 - 503)
**Afghanistan**									

	Prevalence 1990	Prevalence 2005	Prevalence 2015	Incidence 1990	Incidence 2005	Incidence 2015	YLD rate 1990	YLD rate 2005	YLD rate 2015
**15 - 49 years (rate per capita)**	0.060 (0.049 - 0.075)	0.058 (0.047 - 0.072)	0.058 (0.047 - 0.072)	0.007 (0.005 - 0.009)	0.007 (0.005 - 0.008)	0.007 (0.005 - 0.008)	0.384 (0.144 - 0.807)	0.373 (0.139 - 0.784)	0.382 (0.143 - 0.793)
**50 - 69 years (rate per capita)**	0.195 (0.154 - 0.240)	0.195 (0.154 - 0.239)	0.195 (0.155 - 0.240)	0.012 (0.008 - 0.015)	0.012 (0.008 - 0.016)	0.012 (0.008 - 0.015)	1.237 (0.480 - 2.569)	1.216 (0.473 - 2.521)	1.191 (0.464 - 2.489)
**70+ years (rate per capita)**	0.170 (0.138 - 0.207)	0.167 (0.135 - 0.203)	0.167 (0.136 - 0.204)	0.008 (0.005 - 0.011)	0.007 (0.005 - 0.011)	0.008 (0.005 - 0.011)	1.032 (0.404 - 2.152)	1.010 (0.398 - 2.075)	1.005 (0.400 - 2.089)
**All ages (rate per capita)**	0.040 (0.034 - 0.049)	0.041 (0.035 - 0.049)	0.044 (0.037 - 0.052)	0.004 (0.003 - 0.005)	0.004 (0.003 - 0.005)	0.004 (0.003 - 0.005)	0.257 (0.100 - 0.532)	0.261 (0.100 - 0.540)	0.279 (0.108 - 0.576)
**Age-standardized (rate per capita)**	0.081 (0.068 - 0.097)	0.081 (0.068 - 0.097)	0.081 (0.068 - 0.097)	0.006 (0.005 - 0.007)	0.006 (0.005 - 0.007)	0.006 (0.005 - 0.007)	0.513 (0.198 - 1.067)	0.510 (0.199 - 1.066)	0.510 (0.198 - 1.056)
**Total number of cases (thousands)**	497 (418 - 599)	1,005 (845 - 1,208)	1,426 (1,197 - 1,706)	46 (39 - 56)	94 (79 - 113)	133 (112 - 160)	3 (1 - 7)	6 (2 - 13)	9 (4 - 19)
**Algeria**									
**15 - 49 years (rate per capita)**	0.057 (0.046 - 0.071)	0.063 (0.051 - 0.078)	0.071 (0.058 - 0.088)	0.007 (0.005 - 0.008)	0.007 (0.006 - 0.009)	0.008 (0.006 - 0.010)	0.378 (0.141 - 0.789)	0.416 (0.155 - 0.875)	0.470 (0.175 - 0.984)
**50 - 69 years (rate per capita)**	0.194 (0.155 - 0.240)	0.194 (0.154 - 0.240)	0.194 (0.155 - 0.241)	0.012 (0.008 - 0.015)	0.012 (0.008 - 0.015)	0.012 (0.008 - 0.015)	1.257 (0.486 - 2.586)	1.260 (0.484 - 2.613)	1.257 (0.481 - 2.597)
**70+ years (rate per capita)**	0.164 (0.133 - 0.201)	0.164 (0.134 - 0.199)	0.160 (0.131 - 0.195)	0.007 (0.005 - 0.011)	0.007 (0.005 - 0.010)	0.007 (0.005 - 0.010)	1.022 (0.407 - 2.129)	1.020 (0.400 - 2.092)	0.992 (0.390 - 2.053)
**All ages (rate per capita)**	0.045 (0.038 - 0.054)	0.061 (0.051 - 0.073)	0.070 (0.059 - 0.085)	0.004 (0.003 - 0.005)	0.005 (0.005 - 0.006)	0.006 (0.005 - 0.007)	0.297 (0.115 - 0.616)	0.400 (0.154 - 0.826)	0.461 (0.178 - 0.950)
**Age-standardized (rate per capita)**	0.081 (0.068 - 0.097)	0.081 (0.068 - 0.097)	0.081 (0.068 - 0.097)	0.006 (0.005 - 0.007)	0.006 (0.005 - 0.007)	0.006 (0.005 - 0.007)	0.525 (0.203 - 1.092)	0.527 (0.204 - 1.096)	0.525 (0.202 - 1.086)
**Total number of cases (thousands)**	1,176 (994 - 1,411)	2,033 (1,703 - 2,442)	2,790 (2,331 - 3,356)	107 (90 - 128)	181 (153 - 216)	234 (196 - 280)	8 (3 - 16)	13 (5 - 28)	18 (7 - 38)
**Bahrain**									
**15 - 49 years (rate per capita)**	0.070 (0.056 - 0.088)	0.077 (0.062 - 0.097)	0.079 (0.064 - 0.099)	0.008 (0.006 - 0.010)	0.008 (0.006 - 0.010)	0.008 (0.006 - 0.010)	0.463 (0.172 - 0.966)	0.513 (0.190 - 1.096)	0.525 (0.194 - 1.103)
**50 - 69 years (rate per capita)**	0.196 (0.157 - 0.243)	0.196 (0.154 - 0.245)	0.196 (0.155 - 0.246)	0.012 (0.008 - 0.016)	0.012 (0.008 - 0.016)	0.012 (0.009 - 0.017)	1.265 (0.487 - 2.614)	1.262 (0.482 - 2.607)	1.268 (0.483 - 2.649)
**70+ years (rate per capita)**	0.163 (0.132 - 0.197)	0.165 (0.135 - 0.201)	0.165 (0.133 - 0.201)	0.007 (0.005 - 0.010)	0.007 (0.005 - 0.011)	0.007 (0.005 - 0.010)	1.000 (0.397 - 2.080)	1.012 (0.400 - 2.112)	1.011 (0.398 - 2.087)
**All ages (rate per capita)**	0.056 (0.047 - 0.070)	0.067 (0.056 - 0.082)	0.077 (0.064 - 0.094)	0.005 (0.004 - 0.007)	0.006 (0.005 - 0.008)	0.007 (0.006 - 0.008)	0.371 (0.140 - 0.764)	0.442 (0.166 - 0.934)	0.506 (0.193 - 1.052)
**Age-standardized (rate per capita)**	0.082 (0.069 - 0.100)	0.083 (0.068 - 0.099)	0.083 (0.069 - 0.099)	0.006 (0.005 - 0.008)	0.006 (0.005 - 0.008)	0.006 (0.005 - 0.008)	0.535 (0.206 - 1.102)	0.535 (0.206 - 1.112)	0.536 (0.207 - 1.100)
**Total number of cases (thousands)**	28 (23 - 35)	58 (48 - 71)	105 (87 - 129)	3 (2 - 3)	5 (4 - 7)	9 (8 - 12)	0 (0 - 0)	0 (0 - 1)	1 (0 - 1)
**Egypt**									
**15 - 49 years (rate per capita)**	0.064 (0.052 - 0.079)	0.063 (0.052 - 0.079)	0.068 (0.055 - 0.084)	0.007 (0.006 - 0.009)	0.007 (0.006 - 0.009)	0.007 (0.006 - 0.009)	0.424 (0.160 - 0.876)	0.418 (0.156 - 0.888)	0.449 (0.166 - 0.940)
**50 - 69 years (rate per capita)**	0.194 (0.155 - 0.238)	0.195 (0.155 - 0.240)	0.194 (0.153 - 0.238)	0.012 (0.008 - 0.015)	0.012 (0.008 - 0.015)	0.012 (0.008 - 0.015)	1.258 (0.484 - 2.595)	1.257 (0.489 - 2.596)	1.251 (0.483 - 2.589)
**70+ years (rate per capita)**	0.164 (0.134 - 0.199)	0.164 (0.134 - 0.199)	0.162 (0.133 - 0.197)	0.007 (0.005 - 0.011)	0.007 (0.005 - 0.010)	0.007 (0.005 - 0.010)	1.019 (0.402 - 2.117)	1.015 (0.405 - 2.097)	1.000 (0.397 - 2.073)
**All ages (rate per capita)**	0.055 (0.046 - 0.066)	0.060 (0.050 - 0.072)	0.064 (0.054 - 0.077)	0.005 (0.004 - 0.006)	0.005 (0.004 - 0.006)	0.005 (0.005 - 0.006)	0.357 (0.139 - 0.735)	0.394 (0.152 - 0.814)	0.420 (0.161 - 0.855)
**Age-standardized (rate per capita)**	0.081 (0.068 - 0.097)	0.081 (0.068 - 0.097)	0.081 (0.067 - 0.097)	0.006 (0.005 - 0.007)	0.006 (0.005 - 0.007)	0.006 (0.005 - 0.007)	0.525 (0.202 - 1.080)	0.526 (0.204 - 1.082)	0.524 (0.201 - 1.067)
**Total number of cases (thousands)**	3,075 (2,583 - 3,692)	4,506 (3,768 - 5,394)	5,856 (4,883 - 7,045)	262 (220 - 313)	390 (328 - 464)	496 (415 - 590)	20 (8 - 41)	29 (11 - 61)	38 (15 - 78)
**Iran**									
**15 - 49 years (rate per capita)**	0.061 (0.052 - 0.073)	0.060 (0.052 - 0.071)	0.074 (0.063 - 0.088)	0.007 (0.006 - 0.008)	0.007 (0.006 - 0.008)	0.008 (0.006 - 0.009)	0.407 (0.154 - 0.848)	0.398 (0.150 - 0.833)	0.493 (0.186 - 1.022)
**50 - 69 years (rate per capita)**	0.207 (0.173 - 0.243)	0.202 (0.170 - 0.239)	0.205 (0.172 - 0.242)	0.011 (0.008 - 0.014)	0.012 (0.009 - 0.015)	0.012 (0.009 - 0.014)	1.339 (0.524 - 2.702)	1.311 (0.511 - 2.621)	1.330 (0.516 - 2.697)
**70+ years (rate per capita)**	0.186 (0.159 - 0.216)	0.185 (0.160 - 0.214)	0.181 (0.157 - 0.210)	0.007 (0.005 - 0.010)	0.007 (0.005 - 0.009)	0.007 (0.005 - 0.009)	1.158 (0.454 - 2.341)	1.148 (0.455 - 2.330)	1.122 (0.447 - 2.299)
**All ages (rate per capita)**	0.049 (0.043 - 0.056)	0.062 (0.055 - 0.072)	0.078 (0.068 - 0.090)	0.004 (0.004 - 0.005)	0.005 (0.005 - 0.006)	0.006 (0.006 - 0.007)	0.319 (0.123 - 0.667)	0.408 (0.160 - 0.849)	0.513 (0.198 - 1.058)
**Age-standardized (rate per capita)**	0.085 (0.075 - 0.098)	0.083 (0.074 - 0.096)	0.085 (0.075 - 0.098)	0.006 (0.005 - 0.007)	0.006 (0.005 - 0.007)	0.006 (0.005 - 0.007)	0.555 (0.216 - 1.156)	0.541 (0.213 - 1.118)	0.555 (0.215 - 1.145)
**Total number of cases (thousands)**	2,724 (2,381 - 3,141)	4,401 (3,914 - 5,065)	6,180 (5,384 - 7,135)	232 (203 - 266)	383 (338 - 438)	503 (437 - 580)	18 (7 - 37)	29 (11 - 60)	41 (16 - 84)
**Iraq**									
**15 - 49 years (rate per capita)**	0.056 (0.045 - 0.070)	0.058 (0.047 - 0.073)	0.063 (0.051 - 0.079)	0.006 (0.005 - 0.008)	0.007 (0.005 - 0.008)	0.007 (0.006 - 0.009)	0.367 (0.137 - 0.766)	0.384 (0.145 - 0.793)	0.416 (0.157 - 0.871)
**50 - 69 years (rate per capita)**	0.195 (0.155 - 0.240)	0.195 (0.154 - 0.240)	0.194 (0.154 - 0.239)	0.012 (0.008 - 0.015)	0.012 (0.008 - 0.015)	0.012 (0.008 - 0.015)	1.241 (0.481 - 2.523)	1.233 (0.478 - 2.526)	1.226 (0.479 - 2.521)
**70+ years (rate per capita)**	0.165 (0.134 - 0.200)	0.164 (0.133 - 0.198)	0.164 (0.134 - 0.200)	0.007 (0.005 - 0.010)	0.007 (0.005 - 0.010)	0.007 (0.005 - 0.011)	1.003 (0.396 - 2.065)	0.996 (0.394 - 2.063)	0.998 (0.393 - 2.070)
**All ages (rate per capita)**	0.044 (0.037 - 0.053)	0.047 (0.039 - 0.056)	0.049 (0.041 - 0.060)	0.004 (0.003 - 0.005)	0.004 (0.004 - 0.005)	0.004 (0.004 - 0.005)	0.283 (0.109 - 0.580)	0.301 (0.117 - 0.617)	0.320 (0.124 - 0.658)
**Age-standardized (rate per capita)**	0.081 (0.068 - 0.097)	0.081 (0.068 - 0.097)	0.081 (0.067 - 0.097)	0.006 (0.005 - 0.007)	0.006 (0.005 - 0.007)	0.006 (0.005 - 0.007)	0.518 (0.199 - 1.068)	0.518 (0.201 - 1.075)	0.517 (0.200 - 1.065)
**Total number of cases (thousands)**	767 (650 - 924)	1,261 (1,063 - 1,510)	1,797 (1,501 - 2,177)	69 (58 - 81)	116 (97 - 138)	164 (136 - 198)	5 (2 - 10)	8 (3 - 17)	12 (5 - 24)
**Jordan**									
**15 - 49 years (rate per capita)**	0.054 (0.044 - 0.067)	0.061 (0.049 - 0.076)	0.068 (0.055 - 0.085)	0.006 (0.005 - 0.008)	0.007 (0.006 - 0.009)	0.007 (0.006 - 0.009)	0.359 (0.132 - 0.748)	0.407 (0.151 - 0.843)	0.449 (0.167 - 0.942)
**50 - 69 years (rate per capita)**	0.195 (0.155 - 0.241)	0.196 (0.156 - 0.243)	0.194 (0.153 - 0.239)	0.012 (0.008 - 0.015)	0.012 (0.008 - 0.015)	0.012 (0.008 - 0.016)	1.255 (0.487 - 2.571)	1.260 (0.487 - 2.603)	1.255 (0.486 - 2.590)
**70+ years (rate per capita)**	0.162 (0.133 - 0.196)	0.167 (0.135 - 0.203)	0.165 (0.134 - 0.200)	0.007 (0.005 - 0.010)	0.007 (0.005 - 0.011)	0.007 (0.005 - 0.011)	0.997 (0.402 - 2.093)	1.028 (0.405 - 2.138)	1.016 (0.402 - 2.098)
**All ages (rate per capita)**	0.042 (0.036 - 0.051)	0.050 (0.043 - 0.061)	0.057 (0.048 - 0.070)	0.004 (0.003 - 0.005)	0.005 (0.004 - 0.006)	0.005 (0.004 - 0.006)	0.276 (0.107 - 0.570)	0.331 (0.128 - 0.675)	0.377 (0.145 - 0.785)
**Age-standardized (rate per capita)**	0.081 (0.068 - 0.097)	0.081 (0.068 - 0.098)	0.081 (0.068 - 0.097)	0.006 (0.005 - 0.008)	0.006 (0.005 - 0.008)	0.006 (0.005 - 0.007)	0.526 (0.203 - 1.072)	0.528 (0.204 - 1.086)	0.528 (0.204 - 1.091)
**Total number of cases (thousands)**	141 (119 - 169)	270 (228 - 327)	435 (364 - 528)	13 (11 - 15)	25 (21 - 30)	39 (32 - 47)	1 (0 - 2)	2 (1 - 4)	3 (1 - 6)
**Kuwait**									
**15 - 49 years (rate per capita)**	0.070 (0.057 - 0.087)	0.076 (0.062 - 0.096)	0.080 (0.064 - 0.101)	0.007 (0.006 - 0.009)	0.008 (0.006 - 0.010)	0.008 (0.006 - 0.011)	0.468 (0.174 - 0.977)	0.507 (0.185 - 1.064)	0.536 (0.198 - 1.128)
**50 - 69 years (rate per capita)**	0.198 (0.156 - 0.246)	0.197 (0.154 - 0.243)	0.195 (0.154 - 0.243)	0.012 (0.009 - 0.016)	0.012 (0.008 - 0.016)	0.012 (0.008 - 0.016)	1.290 (0.492 - 2.695)	1.277 (0.492 - 2.637)	1.271 (0.484 - 2.631)
**70+ years (rate per capita)**	0.163 (0.133 - 0.199)	0.170 (0.137 - 0.207)	0.166 (0.135 - 0.202)	0.007 (0.005 - 0.010)	0.008 (0.005 - 0.011)	0.008 (0.005 - 0.011)	1.013 (0.402 - 2.113)	1.057 (0.417 - 2.212)	1.031 (0.410 - 2.136)
**All ages (rate per capita)**	0.056 (0.047 - 0.069)	0.066 (0.055 - 0.081)	0.074 (0.061 - 0.091)	0.005 (0.004 - 0.006)	0.006 (0.005 - 0.008)	0.007 (0.005 - 0.008)	0.372 (0.141 - 0.762)	0.438 (0.164 - 0.913)	0.492 (0.186 - 1.035)
**Age-standardized (rate per capita)**	0.082 (0.069 - 0.099)	0.082 (0.069 - 0.099)	0.082 (0.068 - 0.098)	0.006 (0.005 - 0.008)	0.006 (0.005 - 0.008)	0.006 (0.005 - 0.008)	0.536 (0.207 - 1.113)	0.537 (0.208 - 1.109)	0.533 (0.205 - 1.105)
**Total number of cases (thousands)**	115 (96 - 141)	151 (125 - 184)	290 (239 - 354)	11 (9 - 13)	14 (12 - 17)	26 (21 - 33)	1 (0 - 2)	1 (0 - 2)	2 (1 - 4)
**Lebanon**									
**15 - 49 years (rate per capita)**	0.064 (0.052 - 0.079)	0.069 (0.057 - 0.087)	0.068 (0.055 - 0.086)	0.007 (0.006 - 0.009)	0.007 (0.006 - 0.009)	0.007 (0.006 - 0.009)	0.412 (0.155 - 0.862)	0.452 (0.170 - 0.943)	0.450 (0.167 - 0.949)
**50 - 69 years (rate per capita)**	0.197 (0.157 - 0.243)	0.197 (0.158 - 0.243)	0.197 (0.158 - 0.243)	0.012 (0.008 - 0.015)	0.012 (0.008 - 0.015)	0.012 (0.008 - 0.015)	1.248 (0.486 - 2.594)	1.245 (0.482 - 2.558)	1.241 (0.486 - 2.539)
**70+ years (rate per capita)**	0.164 (0.133 - 0.200)	0.167 (0.135 - 0.203)	0.163 (0.133 - 0.198)	0.007 (0.005 - 0.010)	0.008 (0.005 - 0.011)	0.007 (0.005 - 0.010)	0.993 (0.397 - 2.043)	1.014 (0.397 - 2.087)	0.990 (0.393 - 2.053)
**All ages (rate per capita)**	0.063 (0.053 - 0.075)	0.073 (0.062 - 0.088)	0.078 (0.066 - 0.094)	0.005 (0.004 - 0.006)	0.006 (0.005 - 0.007)	0.006 (0.005 - 0.008)	0.401 (0.156 - 0.816)	0.468 (0.182 - 0.960)	0.502 (0.194 - 1.032)
**Age-standardized (rate per capita)**	0.081 (0.068 - 0.098)	0.082 (0.069 - 0.098)	0.082 (0.069 - 0.098)	0.006 (0.005 - 0.007)	0.006 (0.005 - 0.008)	0.006 (0.005 - 0.007)	0.517 (0.202 - 1.053)	0.522 (0.202 - 1.071)	0.523 (0.203 - 1.076)
**Total number of cases (thousands)**	172 (144 - 206)	290 (244 - 348)	451 (378 - 544)	14 (12 - 17)	24 (20 - 28)	37 (31 - 43)	1 (0 - 2)	2 (1 - 4)	3 (1 - 6)
**Libya**									
**15 - 49 years (rate per capita)**	0.059 (0.048 - 0.074)	0.064 (0.051 - 0.080)	0.075 (0.060 - 0.094)	0.007 (0.005 - 0.008)	0.007 (0.006 - 0.009)	0.008 (0.006 - 0.010)	0.393 (0.147 - 0.821)	0.423 (0.155 - 0.875)	0.498 (0.185 - 1.049)
**50 - 69 years (rate per capita)**	0.195 (0.155 - 0.239)	0.195 (0.154 - 0.241)	0.194 (0.155 - 0.239)	0.012 (0.008 - 0.015)	0.012 (0.008 - 0.015)	0.012 (0.008 - 0.015)	1.267 (0.486 - 2.620)	1.262 (0.482 - 2.596)	1.253 (0.483 - 2.588)
**70+ years (rate per capita)**	0.166 (0.135 - 0.201)	0.165 (0.135 - 0.201)	0.163 (0.134 - 0.197)	0.008 (0.005 - 0.011)	0.007 (0.005 - 0.011)	0.007 (0.005 - 0.010)	1.034 (0.413 - 2.149)	1.024 (0.405 - 2.112)	1.007 (0.395 - 2.072)
**All ages (rate per capita)**	0.048 (0.040 - 0.058)	0.059 (0.050 - 0.071)	0.069 (0.058 - 0.084)	0.004 (0.004 - 0.005)	0.005 (0.005 - 0.006)	0.006 (0.005 - 0.007)	0.315 (0.122 - 0.646)	0.391 (0.149 - 0.796)	0.453 (0.174 - 0.952)
**Age-standardized (rate per capita)**	0.081 (0.068 - 0.098)	0.081 (0.068 - 0.097)	0.081 (0.068 - 0.096)	0.006 (0.005 - 0.008)	0.006 (0.005 - 0.008)	0.006 (0.005 - 0.007)	0.531 (0.204 - 1.089)	0.528 (0.204 - 1.077)	0.525 (0.204 - 1.087)
**Total number of cases (thousands)**	210 (177 - 253)	346 (289 - 415)	435 (363 - 526)	19 (16 - 23)	31 (26 - 38)	37 (31 - 45)	1 (1 - 3)	2 (1 - 5)	3 (1 - 6)
**Morocco**									
**15 - 49 years (rate per capita)**	0.089 (0.075 - 0.104)	0.096 (0.081 - 0.114)	0.100 (0.084 - 0.119)	0.010 (0.008 - 0.011)	0.010 (0.009 - 0.012)	0.010 (0.009 - 0.012)	0.589 (0.229 - 1.221)	0.637 (0.242 - 1.323)	0.663 (0.254 - 1.380)
**50 - 69 years (rate per capita)**	0.270 (0.230 - 0.312)	0.265 (0.225 - 0.308)	0.266 (0.225 - 0.310)	0.014 (0.010 - 0.018)	0.014 (0.011 - 0.018)	0.014 (0.011 - 0.018)	1.733 (0.680 - 3.469)	1.703 (0.679 - 3.407)	1.711 (0.664 - 3.447)
**70+ years (rate per capita)**	0.212 (0.182 - 0.245)	0.210 (0.180 - 0.241)	0.206 (0.180 - 0.237)	0.008 (0.006 - 0.011)	0.008 (0.005 - 0.011)	0.008 (0.006 - 0.011)	1.309 (0.536 - 2.664)	1.294 (0.516 - 2.633)	1.265 (0.511 - 2.557)
**All ages (rate per capita)**	0.075 (0.066 - 0.085)	0.090 (0.079 - 0.104)	0.103 (0.090 - 0.119)	0.006 (0.006 - 0.007)	0.007 (0.006 - 0.008)	0.008 (0.007 - 0.009)	0.488 (0.194 - 1.001)	0.590 (0.229 - 1.222)	0.670 (0.260 - 1.398)

	Prevalence 1990	Prevalence 2005	Prevalence 2015	Incidence 1990	Incidence 2005	Incidence 2015	YLD rate 1990	YLD rate 2005	YLD rate 2015
Age-standardized (rate per capita)	0.115 (0.102 - 0.131)	0.112 (0.098 - 0.128)	0.111 (0.098 - 0.128)	0.008 (0.007 - 0.009)	0.008 (0.007 - 0.009)	0.008 (0.007 - 0.009)	0.748 (0.295 - 1.540)	0.723 (0.283 - 1.505)	0.722 (0.280 - 1.503)
Total number of cases (thousands)	1,871 (1,645 - 2,126)	2,751 (2,414 - 3,173)	3,536 (3,090 - 4,101)	156 (138 - 177)	225 (197 - 257)	275 (241 - 314)	12 (5 - 25)	18 (7 - 37)	23 (9 - 48)
**Palestine**									
15 - 49 years (rate per capita)	0.057 (0.046 - 0.071)	0.059 (0.048 - 0.073)	0.060 (0.049 - 0.074)	0.007 (0.005 - 0.008)	0.007 (0.005 - 0.009)	0.007 (0.006 - 0.008)	0.375 (0.140 - 0.786)	0.387 (0.144 - 0.810)	0.397 (0.146 - 0.827)
50 - 69 years (rate per capita)	0.194 (0.155 - 0.239)	0.195 (0.156 - 0.241)	0.193 (0.153 - 0.239)	0.012 (0.008 - 0.016)	0.012 (0.008 - 0.015)	0.012 (0.008 - 0.015)	1.252 (0.483 - 2.569)	1.254 (0.487 - 2.610)	1.239 (0.480 - 2.550)
70+ years (rate per capita)	0.165 (0.134 - 0.201)	0.166 (0.134 - 0.203)	0.165 (0.135 - 0.201)	0.007 (0.005 - 0.010)	0.007 (0.005 - 0.010)	0.007 (0.005 - 0.011)	1.022 (0.408 - 2.127)	1.032 (0.408 - 2.142)	1.022 (0.409 - 2.121)
All ages (rate per capita)	0.039 (0.033 - 0.047)	0.043 (0.036 - 0.052)	0.048 (0.040 - 0.058)	0.004 (0.003 - 0.004)	0.004 (0.003 - 0.005)	0.004 (0.004 - 0.005)	0.256 (0.099 - 0.532)	0.278 (0.107 - 0.566)	0.313 (0.120 - 0.647)
Age-standardized (rate per capita)	0.081 (0.068 - 0.097)	0.081 (0.068 - 0.098)	0.081 (0.068 - 0.097)	0.006 (0.005 - 0.007)	0.006 (0.005 - 0.007)	0.006 (0.005 - 0.007)	0.522 (0.203 - 1.085)	0.524 (0.205 - 1.095)	0.523 (0.202 - 1.071)
Total number of cases (thousands)	83 (70 - 100)	153 (129 - 186)	224 (188 - 270)	8 (7 - 9)	14 (12 - 17)	21 (17 - 25)	1 (0 - 1)	1 (0 - 2)	1 (1 - 3)
**Oman**									
15 - 49 years (rate per capita)	0.069 (0.056 - 0.086)	0.065 (0.053 - 0.082)	0.074 (0.059 - 0.093)	0.007 (0.006 - 0.009)	0.007 (0.006 - 0.009)	0.008 (0.006 - 0.010)	0.461 (0.171 - 0.961)	0.436 (0.162 - 0.908)	0.492 (0.181 - 1.040)
50 - 69 years (rate per capita)	0.193 (0.152 - 0.240)	0.197 (0.155 - 0.243)	0.198 (0.158 - 0.247)	0.012 (0.008 - 0.016)	0.012 (0.008 - 0.016)	0.012 (0.008 - 0.016)	1.248 (0.483 - 2.561)	1.271 (0.488 - 2.615)	1.279 (0.492 - 2.668)
70+ years (rate per capita)	0.164 (0.133 - 0.201)	0.163 (0.132 - 0.198)	0.165 (0.135 - 0.202)	0.007 (0.005 - 0.011)	0.007 (0.005 - 0.010)	0.007 (0.005 - 0.010)	1.013 (0.407 - 2.116)	0.997 (0.398 - 2.092)	1.012 (0.405 - 2.079)
All ages (rate per capita)	0.047 (0.039 - 0.057)	0.055 (0.046 - 0.066)	0.071 (0.059 - 0.087)	0.004 (0.004 - 0.005)	0.005 (0.004 - 0.006)	0.007 (0.005 - 0.008)	0.310 (0.118 - 0.641)	0.359 (0.138 - 0.751)	0.467 (0.178 - 0.967)
Age-standardized (rate per capita)	0.082 (0.068 - 0.098)	0.082 (0.069 - 0.099)	0.083 (0.070 - 0.100)	0.006 (0.005 - 0.008)	0.006 (0.005 - 0.008)	0.006 (0.005 - 0.008)	0.530 (0.203 - 1.093)	0.535 (0.208 - 1.110)	0.540 (0.209 - 1.110)
Total number of cases (thousands)	86 (72 - 104)	137 (114 - 166)	317 (264 - 390)	8 (7 - 10)	13 (11 - 16)	30 (24 - 37)	1 (0 - 1)	1 (0 - 2)	2 (1 - 4)
**Qatar**									
15 - 49 years (rate per capita)	0.081 (0.065 - 0.101)	0.085 (0.068 - 0.108)	0.079 (0.063 - 0.099)	0.008 (0.007 - 0.011)	0.009 (0.007 - 0.011)	0.008 (0.007 - 0.011)	0.538 (0.201 - 1.133)	0.565 (0.210 - 1.182)	0.527 (0.196 - 1.096)
50 - 69 years (rate per capita)	0.200 (0.158 - 0.249)	0.202 (0.157 - 0.254)	0.200 (0.156 - 0.252)	0.012 (0.009 - 0.017)	0.013 (0.009 - 0.017)	0.012 (0.008 - 0.017)	1.292 (0.489 - 2.651)	1.304 (0.500 - 2.740)	1.295 (0.492 - 2.703)
70+ years (rate per capita)	0.162 (0.132 - 0.195)	0.166 (0.134 - 0.203)	0.169 (0.137 - 0.206)	0.007 (0.005 - 0.010)	0.007 (0.005 - 0.010)	0.008 (0.005 - 0.011)	0.996 (0.395 - 2.072)	1.019 (0.405 - 2.112)	1.040 (0.415 - 2.158)
All ages (rate per capita)	0.066 (0.055 - 0.082)	0.078 (0.064 - 0.096)	0.077 (0.063 - 0.094)	0.006 (0.005 - 0.008)	0.007 (0.006 - 0.009)	0.007 (0.006 - 0.009)	0.439 (0.166 - 0.914)	0.513 (0.193 - 1.093)	0.509 (0.193 - 1.055)
Age-standardized (rate per capita)	0.084 (0.071 - 0.101)	0.084 (0.070 - 0.102)	0.084 (0.070 - 0.101)	0.007 (0.006 - 0.008)	0.007 (0.005 - 0.008)	0.007 (0.005 - 0.008)	0.544 (0.210 - 1.120)	0.546 (0.210 - 1.127)	0.545 (0.211 - 1.126)
Total number of cases (thousands)	32 (26 - 39)	64 (53 - 80)	171 (140 - 208)	3 (2 - 4)	6 (5 - 7)	16 (13 - 20)	0 (0 - 0)	0 (0 - 1)	1 (0 - 2)
**Saudi Arabia**									
15 - 49 years (rate per capita)	0.051 (0.043 - 0.062)	0.057 (0.048 - 0.069)	0.064 (0.053 - 0.078)	0.006 (0.005 - 0.007)	0.006 (0.005 - 0.008)	0.007 (0.006 - 0.008)	0.341 (0.131 - 0.711)	0.381 (0.145 - 0.806)	0.430 (0.162 - 0.906)
50 - 69 years (rate per capita)	0.159 (0.133 - 0.190)	0.161 (0.133 - 0.194)	0.163 (0.134 - 0.197)	0.010 (0.007 - 0.012)	0.010 (0.008 - 0.013)	0.011 (0.008 - 0.013)	1.032 (0.402 - 2.102)	1.045 (0.404 - 2.136)	1.060 (0.410 - 2.165)
70+ years (rate per capita)	0.138 (0.118 - 0.162)	0.140 (0.118 - 0.165)	0.139 (0.118 - 0.163)	0.007 (0.005 - 0.009)	0.007 (0.005 - 0.009)	0.007 (0.005 - 0.009)	0.863 (0.337 - 1.762)	0.871 (0.341 - 1.770)	0.867 (0.339 - 1.767)
All ages (rate per capita)	0.038 (0.033 - 0.045)	0.047 (0.040 - 0.056)	0.059 (0.050 - 0.069)	0.004 (0.003 - 0.004)	0.004 (0.004 - 0.005)	0.005 (0.004 - 0.006)	0.251 (0.097 - 0.522)	0.312 (0.120 - 0.641)	0.386 (0.148 - 0.799)
Age-standardized (rate per capita)	0.067 (0.058 - 0.078)	0.068 (0.058 - 0.079)	0.068 (0.059 - 0.080)	0.005 (0.005 - 0.006)	0.005 (0.005 - 0.006)	0.005 (0.005 - 0.006)	0.437 (0.169 - 0.904)	0.442 (0.172 - 0.914)	0.444 (0.172 - 0.916)
Total number of cases (thousands)	622 (533 - 729)	1,168 (1,000 - 1,383)	1,840 (1,569 - 2,182)	60 (52 - 70)	111 (95 - 131)	165 (140 - 196)	4 (2 - 9)	8 (3 - 16)	12 (5 - 25)
**Sudan**									
15 - 49 years (rate per capita)	0.059 (0.048 - 0.073)	0.060 (0.049 - 0.076)	0.062 (0.050 - 0.078)	0.007 (0.005 - 0.008)	0.007 (0.006 - 0.009)	0.007 (0.006 - 0.009)	0.387 (0.145 - 0.804)	0.398 (0.147 - 0.816)	0.411 (0.154 - 0.859)
50 - 69 years (rate per capita)	0.194 (0.154 - 0.239)	0.195 (0.154 - 0.240)	0.193 (0.153 - 0.239)	0.012 (0.008 - 0.015)	0.012 (0.008 - 0.015)	0.012 (0.008 - 0.015)	1.250 (0.482 - 2.564)	1.250 (0.477 - 2.550)	1.243 (0.487 - 2.541)
70+ years (rate per capita)	0.166 (0.135 - 0.201)	0.166 (0.136 - 0.201)	0.164 (0.134 - 0.199)	0.007 (0.005 - 0.011)	0.007 (0.005 - 0.011)	0.007 (0.005 - 0.010)	1.019 (0.408 - 2.088)	1.017 (0.406 - 2.071)	1.009 (0.394 - 2.093)
All ages (rate per capita)	0.044 (0.037 - 0.053)	0.047 (0.039 - 0.056)	0.050 (0.042 - 0.060)	0.004 (0.003 - 0.005)	0.004 (0.004 - 0.005)	0.005 (0.004 - 0.005)	0.288 (0.112 - 0.589)	0.303 (0.117 - 0.621)	0.327 (0.126 - 0.665)
Age-standardized (rate per capita)	0.081 (0.068 - 0.097)	0.081 (0.068 - 0.097)	0.081 (0.067 - 0.097)	0.006 (0.005 - 0.007)	0.006 (0.005 - 0.007)	0.006 (0.005 - 0.007)	0.522 (0.201 - 1.069)	0.522 (0.202 - 1.065)	0.521 (0.202 - 1.059)
Total number of cases (thousands)	884 (746 - 1,065)	1,495 (1,248 - 1,796)	2,022 (1,684 - 2,442)	81 (68 - 97)	136 (115 - 163)	182 (152 - 218)	6 (2 - 12)	10 (4 - 20)	13 (5 - 27)
**Syria**									
15 - 49 years (rate per capita)	0.055 (0.045 - 0.070)	0.060 (0.048 - 0.074)	0.063 (0.051 - 0.080)	0.006 (0.005 - 0.008)	0.007 (0.005 - 0.008)	0.007 (0.006 - 0.009)	0.369 (0.138 - 0.763)	0.398 (0.147 - 0.826)	0.419 (0.157 - 0.883)
50 - 69 years (rate per capita)	0.196 (0.155 - 0.242)	0.195 (0.153 - 0.242)	0.194 (0.155 - 0.239)	0.012 (0.008 - 0.015)	0.012 (0.008 - 0.015)	0.012 (0.008 - 0.015)	1.275 (0.493 - 2.627)	1.270 (0.492 - 2.633)	1.258 (0.483 - 2.601)
70+ years (rate per capita)	0.166 (0.133 - 0.201)	0.166 (0.135 - 0.202)	0.163 (0.132 - 0.197)	0.007 (0.005 - 0.011)	0.007 (0.005 - 0.010)	0.007 (0.005 - 0.010)	1.035 (0.410 - 2.159)	1.037 (0.407 - 2.165)	1.011 (0.400 - 2.096)
All ages (rate per capita)	0.041 (0.035 - 0.049)	0.049 (0.041 - 0.059)	0.056 (0.047 - 0.067)	0.004 (0.003 - 0.005)	0.005 (0.004 - 0.005)	0.005 (0.004 - 0.006)	0.273 (0.104 - 0.564)	0.325 (0.125 - 0.667)	0.366 (0.141 - 0.772)
Age-standardized (rate per capita)	0.081 (0.068 - 0.098)	0.081 (0.068 - 0.098)	0.081 (0.068 - 0.097)	0.006 (0.005 - 0.007)	0.006 (0.005 - 0.008)	0.006 (0.005 - 0.007)	0.531 (0.204 - 1.099)	0.532 (0.205 - 1.098)	0.526 (0.204 - 1.100)
Total number of cases (thousands)	516 (435 - 616)	895 (749 - 1,071)	1,040 (874 - 1,255)	48 (40 - 57)	82 (69 - 99)	91 (76 - 108)	3 (1 - 7)	6 (2 - 12)	7 (3 - 14)
**Tunisia**									
15 - 49 years (rate per capita)	0.060 (0.049 - 0.075)	0.068 (0.056 - 0.085)	0.074 (0.060 - 0.093)	0.007 (0.006 - 0.009)	0.007 (0.006 - 0.009)	0.008 (0.006 - 0.010)	0.400 (0.149 - 0.826)	0.454 (0.169 - 0.961)	0.494 (0.184 - 1.047)
50 - 69 years (rate per capita)	0.196 (0.156 - 0.241)	0.195 (0.155 - 0.239)	0.195 (0.155 - 0.241)	0.012 (0.008 - 0.015)	0.012 (0.008 - 0.015)	0.012 (0.008 - 0.015)	1.267 (0.490 - 2.598)	1.265 (0.489 - 2.628)	1.266 (0.488 - 2.601)
70+ years (rate per capita)	0.166 (0.137 - 0.199)	0.165 (0.135 - 0.199)	0.161 (0.134 - 0.193)	0.007 (0.005 - 0.011)	0.007 (0.005 - 0.010)	0.007 (0.005 - 0.010)	1.032 (0.406 - 2.140)	1.028 (0.406 - 2.128)	0.997 (0.392 - 2.047)
All ages (rate per capita)	0.057 (0.047 - 0.068)	0.072 (0.061 - 0.087)	0.083 (0.069 - 0.100)	0.005 (0.004 - 0.006)	0.006 (0.005 - 0.007)	0.007 (0.006 - 0.008)	0.371 (0.144 - 0.757)	0.473 (0.182 - 0.980)	0.541 (0.209 - 1.119)
Age-standardized (rate per capita)	0.081 (0.068 - 0.097)	0.081 (0.068 - 0.097)	0.081 (0.068 - 0.097)	0.006 (0.005 - 0.007)	0.006 (0.005 - 0.007)	0.006 (0.005 - 0.007)	0.527 (0.203 - 1.073)	0.529 (0.205 - 1.104)	0.527 (0.204 - 1.087)
Total number of cases (thousands)	467 (390 - 559)	731 (613 - 882)	930 (780 - 1,121)	40 (34 - 47)	61 (51 - 73)	74 (62 - 88)	3 (1 - 6)	5 (2 - 10)	6 (2 - 13)
**Turkey**									
15 - 49 years (rate per capita)	0.059 (0.050 - 0.070)	0.064 (0.054 - 0.076)	0.067 (0.057 - 0.080)	0.007 (0.006 - 0.008)	0.007 (0.006 - 0.008)	0.007 (0.006 - 0.009)	0.390 (0.148 - 0.812)	0.427 (0.165 - 0.898)	0.450 (0.173 - 0.937)
50 - 69 years (rate per capita)	0.170 (0.142 - 0.200)	0.168 (0.141 - 0.198)	0.168 (0.141 - 0.200)	0.011 (0.008 - 0.014)	0.011 (0.008 - 0.014)	0.011 (0.008 - 0.014)	1.101 (0.428 - 2.238)	1.089 (0.420 - 2.205)	1.091 (0.422 - 2.210)
70+ years (rate per capita)	0.124 (0.106 - 0.144)	0.124 (0.105 - 0.144)	0.124 (0.107 - 0.144)	0.007 (0.005 - 0.009)	0.007 (0.005 - 0.009)	0.007 (0.005 - 0.009)	0.764 (0.296 - 1.546)	0.767 (0.306 - 1.554)	0.769 (0.310 - 1.563)
All ages (rate per capita)	0.053 (0.046 - 0.061)	0.062 (0.054 - 0.072)	0.070 (0.061 - 0.081)	0.005 (0.004 - 0.005)	0.006 (0.005 - 0.006)	0.006 (0.005 - 0.007)	0.344 (0.134 - 0.710)	0.408 (0.157 - 0.840)	0.457 (0.177 - 0.955)
Age-standardized (rate per capita)	0.072 (0.063 - 0.083)	0.071 (0.062 - 0.082)	0.071 (0.062 - 0.082)	0.006 (0.005 - 0.007)	0.006 (0.005 - 0.007)	0.006 (0.005 - 0.007)	0.466 (0.180 - 0.965)	0.465 (0.180 - 0.963)	0.464 (0.181 - 0.975)
Total number of cases (thousands)	2,876 (2,510 - 3,334)	4,247 (3,707 - 4,916)	5,462 (4,784 - 6,341)	260 (227 - 298)	378 (330 - 434)	470 (411 - 543)	19 (7 - 39)	28 (11 - 57)	36 (14 - 75)
**United Arab Emirates**									
15 - 49 years (rate per capita)	0.078 (0.063 - 0.100)	0.077 (0.062 - 0.097)	0.088 (0.070 - 0.111)	0.008 (0.006 - 0.011)	0.008 (0.006 - 0.010)	0.009 (0.007 - 0.011)	0.519 (0.191 - 1.092)	0.513 (0.189 - 1.075)	0.585 (0.214 - 1.236)
50 - 69 years (rate per capita)	0.203 (0.161 - 0.251)	0.204 (0.159 - 0.257)	0.205 (0.160 - 0.258)	0.012 (0.008 - 0.017)	0.012 (0.009 - 0.017)	0.013 (0.009 - 0.017)	1.309 (0.496 - 2.721)	1.322 (0.499 - 2.794)	1.329 (0.503 - 2.789)
70+ years (rate per capita)	0.174 (0.143 - 0.209)	0.175 (0.144 - 0.210)	0.176 (0.145 - 0.212)	0.007 (0.005 - 0.010)	0.007 (0.005 - 0.011)	0.008 (0.005 - 0.011)	1.071 (0.425 - 2.211)	1.079 (0.426 - 2.248)	1.087 (0.426 - 2.256)
All ages (rate per capita)	0.062 (0.051 - 0.077)	0.071 (0.058 - 0.087)	0.088 (0.072 - 0.109)	0.006 (0.005 - 0.007)	0.007 (0.006 - 0.009)	0.008 (0.006 - 0.010)	0.406 (0.152 - 0.856)	0.469 (0.176 - 0.982)	0.579 (0.218 - 1.226)
Age-standardized (rate per capita)	0.085 (0.071 - 0.103)	0.086 (0.072 - 0.103)	0.086 (0.072 - 0.103)	0.006 (0.005 - 0.008)	0.007 (0.005 - 0.008)	0.007 (0.005 - 0.008)	0.552 (0.214 - 1.153)	0.556 (0.215 - 1.171)	0.559 (0.216 - 1.170)
Total number of cases (thousands)	111 (91 - 138)	318 (261 - 392)	802 (655 - 994)	11 (9 - 13)	31 (25 - 39)	72 (58 - 91)	1 (0 - 2)	2 (1 - 4)	5 (2 - 11)
**Yemen**									
15 - 49 years (rate per capita)	0.046 (0.039 - 0.054)	0.044 (0.037 - 0.052)	0.045 (0.038 - 0.054)	0.005 (0.005 - 0.006)	0.005 (0.004 - 0.006)	0.005 (0.004 - 0.006)	0.304 (0.117 - 0.639)	0.293 (0.111 - 0.605)	0.301 (0.115 - 0.630)
50 - 69 years (rate per capita)	0.153 (0.128 - 0.182)	0.154 (0.128 - 0.184)	0.154 (0.128 - 0.182)	0.010 (0.007 - 0.012)	0.010 (0.007 - 0.012)	0.010 (0.007 - 0.012)	0.988 (0.389 - 2.004)	0.993 (0.387 - 2.018)	0.989 (0.387 - 2.008)
70+ years (rate per capita)	0.133 (0.112 - 0.157)	0.133 (0.112 - 0.157)	0.134 (0.112 - 0.159)	0.007 (0.005 - 0.009)	0.007 (0.005 - 0.009)	0.007 (0.005 - 0.009)	0.823 (0.334 - 1.674)	0.818 (0.326 - 1.665)	0.826 (0.328 - 1.701)
All ages (rate per capita)	0.030 (0.026 - 0.035)	0.034 (0.029 - 0.039)	0.037 (0.032 - 0.043)	0.003 (0.002 - 0.003)	0.003 (0.003 - 0.004)	0.004 (0.003 - 0.004)	0.198 (0.077 - 0.412)	0.219 (0.085 - 0.455)	0.242 (0.094 - 0.497)
Age-standardized (rate per capita)	0.064 (0.056 - 0.074)	0.064 (0.056 - 0.074)	0.064 (0.056 - 0.074)	0.005 (0.005 - 0.006)	0.005 (0.004 - 0.006)	0.005 (0.004 - 0.006)	0.416 (0.164 - 0.861)	0.416 (0.162 - 0.858)	0.416 (0.163 - 0.845)
Total number of cases (thousands)	363 (315 - 419)	689 (599 - 801)	994 (864 - 1,150)	34 (30 - 39)	65 (57 - 75)	94 (83 - 109)	2 (1 - 5)	5 (2 - 9)	7 (3 - 13)
**South Asia**									
15 - 49 years (rate per capita)	0.071 (0.060 - 0.084)	0.073 (0.061 - 0.086)	0.075 (0.063 - 0.089)	0.008 (0.007 - 0.009)	0.008 (0.007 - 0.009)	0.008 (0.007 - 0.010)	0.470 (0.179 - 0.993)	0.481 (0.184 - 1.011)	0.499 (0.191 - 1.064)
50 - 69 years (rate per capita)	0.221 (0.187 - 0.261)	0.219 (0.185 - 0.258)	0.218 (0.184 - 0.257)	0.012 (0.009 - 0.015)	0.012 (0.009 - 0.015)	0.012 (0.009 - 0.015)	1.416 (0.555 - 2.858)	1.405 (0.550 - 2.833)	1.406 (0.549 - 2.834)

	Prevalence 1990	Prevalence 2005	Prevalence 2015	Incidence 1990	Incidence 2005	Incidence 2015	YLD rate 1990	YLD rate 2005	YLD rate 2015
70+ years (rate per capita)	0.195 (0.168 - 0.226)	0.193 (0.167 - 0.223)	0.191 (0.165 - 0.221)	0.008 (0.005 - 0.010)	0.008 (0.005 - 0.010)	0.008 (0.006 - 0.010)	1.201 (0.478 - 2.457)	1.190 (0.473 - 2.429)	1.183 (0.469 - 2.425)
All ages (rate per capita)	0.061 (0.053 - 0.071)	0.068 (0.059 - 0.079)	0.076 (0.066 - 0.088)	0.005 (0.005 - 0.006)	0.006 (0.005 - 0.007)	0.006 (0.005 - 0.007)	0.400 (0.154 - 0.830)	0.444 (0.172 - 0.917)	0.497 (0.192 - 1.034)
Age-standardized (rate per capita)	0.092 (0.081 - 0.106)	0.091 (0.080 - 0.105)	0.091 (0.079 - 0.105)	0.007 (0.006 - 0.008)	0.007 (0.006 - 0.008)	0.007 (0.006 - 0.008)	0.597 (0.232 - 1.252)	0.589 (0.231 - 1.233)	0.589 (0.229 - 1.234)
Total number of cases (thousands)	67,786 (59,045 - 78,312)	100,030 (86,971 - 115,634)	128,658 (111,956 - 148,703)	5,759 (5,019 - 6,626)	8,355 (7,270 - 9,598)	10,458 (9,093 - 12,035)	441 (170 - 916)	652 (252 - 1,345)	840 (325 - 1,748)
**South Asia**									
15 - 49 years (rate per capita)	0.071 (0.060 - 0.084)	0.073 (0.061 - 0.086)	0.075 (0.063 - 0.089)	0.008 (0.007 - 0.009)	0.008 (0.007 - 0.009)	0.008 (0.007 - 0.010)	0.470 (0.179 - 0.993)	0.481 (0.184 - 1.011)	0.499 (0.191 - 1.064)
50 - 69 years (rate per capita)	0.221 (0.187 - 0.261)	0.219 (0.185 - 0.258)	0.218 (0.184 - 0.257)	0.012 (0.009 - 0.015)	0.012 (0.009 - 0.015)	0.012 (0.009 - 0.015)	1.416 (0.555 - 2.858)	1.405 (0.550 - 2.833)	1.406 (0.549 - 2.834)
70+ years (rate per capita)	0.195 (0.168 - 0.226)	0.193 (0.167 - 0.223)	0.191 (0.165 - 0.221)	0.008 (0.005 - 0.010)	0.008 (0.005 - 0.010)	0.008 (0.006 - 0.010)	1.201 (0.478 - 2.457)	1.190 (0.473 - 2.429)	1.183 (0.469 - 2.425)
All ages (rate per capita)	0.061 (0.053 - 0.071)	0.068 (0.059 - 0.079)	0.076 (0.066 - 0.088)	0.005 (0.005 - 0.006)	0.006 (0.005 - 0.007)	0.006 (0.005 - 0.007)	0.400 (0.154 - 0.830)	0.444 (0.172 - 0.917)	0.497 (0.192 - 1.034)
Age-standardized (rate per capita)	0.092 (0.081 - 0.106)	0.091 (0.080 - 0.105)	0.091 (0.079 - 0.105)	0.007 (0.006 - 0.008)	0.007 (0.006 - 0.008)	0.007 (0.006 - 0.008)	0.597 (0.232 - 1.252)	0.589 (0.231 - 1.233)	0.589 (0.229 - 1.234)
Total number of cases (thousands)	67,786 (59,045 - 78,312)	100,030 (86,971 - 115,634)	128,658 (111,956 - 148,703)	5,759 (5,019 - 6,626)	8,355 (7,270 - 9,598)	10,458 (9,093 - 12,035)	441 (170 - 916)	652 (252 - 1,345)	840 (325 - 1,748)
**Bangladesh**									
15 - 49 years (rate per capita)	0.059 (0.049 - 0.070)	0.064 (0.054 - 0.076)	0.069 (0.058 - 0.082)	0.007 (0.006 - 0.008)	0.007 (0.006 - 0.008)	0.007 (0.006 - 0.009)	0.389 (0.148 - 0.808)	0.425 (0.161 - 0.881)	0.459 (0.176 - 0.964)
50 - 69 years (rate per capita)	0.209 (0.175 - 0.247)	0.210 (0.176 - 0.246)	0.207 (0.175 - 0.246)	0.011 (0.008 - 0.014)	0.011 (0.008 - 0.013)	0.011 (0.008 - 0.014)	1.340 (0.525 - 2.729)	1.353 (0.528 - 2.695)	1.342 (0.520 - 2.726)
70+ years (rate per capita)	0.205 (0.175 - 0.237)	0.199 (0.171 - 0.230)	0.195 (0.169 - 0.225)	0.007 (0.005 - 0.009)	0.007 (0.005 - 0.009)	0.007 (0.005 - 0.009)	1.264 (0.500 - 2.609)	1.232 (0.490 - 2.542)	1.209 (0.483 - 2.489)
All ages (rate per capita)	0.050 (0.044 - 0.058)	0.059 (0.051 - 0.068)	0.069 (0.059 - 0.079)	0.004 (0.004 - 0.005)	0.005 (0.004 - 0.006)	0.006 (0.005 - 0.006)	0.326 (0.126 - 0.681)	0.387 (0.151 - 0.794)	0.449 (0.173 - 0.953)
Age-standardized (rate per capita)	0.086 (0.075 - 0.099)	0.087 (0.076 - 0.099)	0.086 (0.075 - 0.100)	0.006 (0.005 - 0.007)	0.006 (0.005 - 0.007)	0.006 (0.005 - 0.007)	0.557 (0.218 - 1.160)	0.560 (0.218 - 1.163)	0.561 (0.219 - 1.177)
Total number of cases (thousands)	5,322 (4,614 - 6,141)	8,471 (7,337 - 9,726)	11,047 (9,563 - 12,778)	446 (387 - 512)	704 (614 - 812)	891 (776 - 1,028)	35 (13 - 72)	55 (21 - 113)	72 (28 - 153)
**Bhutan**									
15 - 49 years (rate per capita)	0.065 (0.054 - 0.076)	0.060 (0.051 - 0.071)	0.068 (0.057 - 0.081)	0.007 (0.006 - 0.008)	0.007 (0.006 - 0.008)	0.007 (0.006 - 0.009)	0.430 (0.164 - 0.905)	0.401 (0.154 - 0.835)	0.456 (0.174 - 0.942)
50 - 69 years (rate per capita)	0.203 (0.170 - 0.242)	0.205 (0.172 - 0.243)	0.204 (0.171 - 0.242)	0.011 (0.008 - 0.014)	0.011 (0.008 - 0.014)	0.011 (0.008 - 0.014)	1.318 (0.512 - 2.695)	1.328 (0.518 - 2.713)	1.322 (0.511 - 2.660)
70+ years (rate per capita)	0.190 (0.161 - 0.222)	0.188 (0.161 - 0.220)	0.184 (0.158 - 0.214)	0.007 (0.005 - 0.009)	0.007 (0.005 - 0.009)	0.007 (0.005 - 0.009)	1.183 (0.463 - 2.445)	1.172 (0.470 - 2.414)	1.143 (0.452 - 2.361)
All ages (rate per capita)	0.051 (0.045 - 0.060)	0.056 (0.049 - 0.065)	0.069 (0.060 - 0.080)	0.004 (0.004 - 0.005)	0.005 (0.004 - 0.006)	0.006 (0.005 - 0.007)	0.338 (0.131 - 0.699)	0.370 (0.142 - 0.765)	0.452 (0.175 - 0.933)
Age-standardized (rate per capita)	0.085 (0.074 - 0.098)	0.085 (0.074 - 0.098)	0.085 (0.074 - 0.098)	0.006 (0.005 - 0.007)	0.006 (0.005 - 0.007)	0.006 (0.005 - 0.007)	0.552 (0.215 - 1.133)	0.554 (0.217 - 1.141)	0.553 (0.216 - 1.133)
Total number of cases (thousands)	28 (24 - 32)	37 (32 - 42)	53 (46 - 62)	2 (2 - 3)	3 (3 - 4)	4 (4 - 5)	0 (0 - 0)	0 (0 - 0)	0 (0 - 1)
**India**									
15 - 49 years (rate per capita)	0.074 (0.062 - 0.088)	0.075 (0.063 - 0.090)	0.078 (0.065 - 0.093)	0.008 (0.007 - 0.010)	0.008 (0.007 - 0.010)	0.008 (0.007 - 0.010)	0.488 (0.186 - 1.032)	0.498 (0.190 - 1.051)	0.517 (0.196 - 1.103)
50 - 69 years (rate per capita)	0.224 (0.190 - 0.265)	0.222 (0.188 - 0.262)	0.221 (0.187 - 0.261)	0.012 (0.009 - 0.015)	0.012 (0.009 - 0.015)	0.012 (0.009 - 0.015)	1.440 (0.565 - 2.904)	1.424 (0.558 - 2.879)	1.427 (0.559 - 2.879)
70+ years (rate per capita)	0.196 (0.169 - 0.227)	0.194 (0.168 - 0.224)	0.192 (0.166 - 0.222)	0.008 (0.006 - 0.010)	0.008 (0.006 - 0.010)	0.008 (0.006 - 0.010)	1.204 (0.479 - 2.454)	1.194 (0.475 - 2.434)	1.187 (0.471 - 2.426)
All ages (rate per capita)	0.064 (0.056 - 0.074)	0.071 (0.062 - 0.083)	0.080 (0.070 - 0.092)	0.005 (0.005 - 0.006)	0.006 (0.005 - 0.007)	0.006 (0.006 - 0.007)	0.419 (0.161 - 0.867)	0.465 (0.180 - 0.963)	0.521 (0.202 - 1.082)
Age-standardized (rate per capita)	0.094 (0.083 - 0.109)	0.093 (0.081 - 0.107)	0.092 (0.081 - 0.106)	0.007 (0.006 - 0.008)	0.007 (0.006 - 0.008)	0.007 (0.006 - 0.008)	0.609 (0.237 - 1.279)	0.599 (0.234 - 1.254)	0.599 (0.233 - 1.256)
Total number of cases (thousands)	56,025 (48,704 - 64,720)	81,769 (71,224 - 94,694)	104,680 (91,256 - 121,170)	4,775 (4,160 - 5,496)	6,829 (5,943 - 7,846)	8,489 (7,406 - 9,744)	365 (141 - 755)	532 (206 - 1,102)	683 (265 - 1,420)
**Nepal**									
15 - 49 years (rate per capita)	0.058 (0.048 - 0.068)	0.062 (0.053 - 0.074)	0.059 (0.049 - 0.070)	0.006 (0.005 - 0.008)	0.007 (0.006 - 0.008)	0.006 (0.005 - 0.008)	0.382 (0.145 - 0.799)	0.414 (0.158 - 0.874)	0.390 (0.148 - 0.810)
50 - 69 years (rate per capita)	0.191 (0.161 - 0.226)	0.199 (0.169 - 0.232)	0.192 (0.161 - 0.228)	0.010 (0.008 - 0.013)	0.010 (0.008 - 0.013)	0.010 (0.008 - 0.013)	1.235 (0.476 - 2.486)	1.286 (0.507 - 2.617)	1.243 (0.486 - 2.523)
70+ years (rate per capita)	0.184 (0.157 - 0.213)	0.184 (0.159 - 0.212)	0.182 (0.157 - 0.211)	0.007 (0.005 - 0.009)	0.007 (0.005 - 0.009)	0.007 (0.005 - 0.009)	1.143 (0.452 - 2.338)	1.151 (0.458 - 2.328)	1.137 (0.453 - 2.316)
All ages (rate per capita)	0.048 (0.042 - 0.056)	0.055 (0.048 - 0.063)	0.060 (0.052 - 0.069)	0.004 (0.004 - 0.005)	0.004 (0.004 - 0.005)	0.005 (0.004 - 0.005)	0.316 (0.123 - 0.657)	0.359 (0.142 - 0.744)	0.393 (0.152 - 0.806)
Age-standardized (rate per capita)	0.079 (0.069 - 0.091)	0.083 (0.073 - 0.095)	0.079 (0.069 - 0.091)	0.006 (0.005 - 0.007)	0.006 (0.005 - 0.007)	0.006 (0.005 - 0.007)	0.514 (0.201 - 1.068)	0.538 (0.212 - 1.111)	0.515 (0.199 - 1.055)
Total number of cases (thousands)	907 (784 - 1,048)	1,402 (1,222 - 1,612)	1,713 (1,490 - 1,983)	76 (66 - 87)	114 (100 - 129)	136 (119 - 157)	6 (2 - 12)	9 (4 - 19)	11 (4 - 23)
**Pakistan**									
15 - 49 years (rate per capita)	0.061 (0.052 - 0.072)	0.062 (0.052 - 0.073)	0.064 (0.054 - 0.076)	0.007 (0.006 - 0.008)	0.007 (0.006 - 0.008)	0.007 (0.006 - 0.008)	0.408 (0.156 - 0.851)	0.409 (0.156 - 0.849)	0.423 (0.161 - 0.883)
50 - 69 years (rate per capita)	0.201 (0.169 - 0.239)	0.201 (0.169 - 0.240)	0.200 (0.169 - 0.236)	0.011 (0.008 - 0.014)	0.011 (0.008 - 0.013)	0.011 (0.008 - 0.014)	1.305 (0.506 - 2.642)	1.301 (0.510 - 2.645)	1.295 (0.503 - 2.597)
70+ years (rate per capita)	0.183 (0.158 - 0.213)	0.182 (0.156 - 0.211)	0.182 (0.156 - 0.209)	0.007 (0.005 - 0.009)	0.007 (0.005 - 0.009)	0.007 (0.005 - 0.009)	1.139 (0.450 - 2.303)	1.128 (0.447 - 2.277)	1.126 (0.444 - 2.329)
All ages (rate per capita)	0.051 (0.044 - 0.059)	0.054 (0.047 - 0.063)	0.059 (0.051 - 0.068)	0.004 (0.004 - 0.005)	0.005 (0.004 - 0.005)	0.005 (0.004 - 0.006)	0.334 (0.129 - 0.695)	0.355 (0.137 - 0.730)	0.386 (0.150 - 0.794)
Age-standardized (rate per capita)	0.084 (0.073 - 0.096)	0.084 (0.073 - 0.097)	0.084 (0.073 - 0.096)	0.006 (0.005 - 0.007)	0.006 (0.005 - 0.007)	0.006 (0.005 - 0.007)	0.545 (0.211 - 1.130)	0.544 (0.211 - 1.128)	0.543 (0.211 - 1.121)
Total number of cases (thousands)	5,504 (4,800 - 6,321)	8,352 (7,249 - 9,667)	11,164 (9,687 - 12,897)	460 (402 - 526)	705 (612 - 816)	937 (814 - 1,075)	36 (14 - 75)	55 (21 - 112)	73 (28 - 150)
**Sub-Saharan Africa**									
15 - 49 years (rate per capita)	0.063 (0.048 - 0.086)	0.062 (0.048 - 0.085)	0.064 (0.049 - 0.088)	0.007 (0.005 - 0.010)	0.007 (0.005 - 0.010)	0.007 (0.005 - 0.010)	0.414 (0.150 - 0.892)	0.409 (0.148 - 0.890)	0.422 (0.152 - 0.911)
50 - 69 years (rate per capita)	0.222 (0.168 - 0.278)	0.222 (0.169 - 0.278)	0.223 (0.170 - 0.278)	0.012 (0.008 - 0.016)	0.012 (0.008 - 0.016)	0.012 (0.008 - 0.016)	1.427 (0.543 - 3.077)	1.429 (0.542 - 3.106)	1.439 (0.552 - 3.100)
70+ years (rate per capita)	0.228 (0.178 - 0.277)	0.228 (0.178 - 0.279)	0.229 (0.178 - 0.278)	0.009 (0.005 - 0.013)	0.009 (0.005 - 0.013)	0.009 (0.006 - 0.013)	1.405 (0.538 - 2.980)	1.411 (0.538 - 2.981)	1.416 (0.540 - 2.994)
All ages (rate per capita)	0.050 (0.040 - 0.065)	0.050 (0.040 - 0.065)	0.052 (0.041 - 0.067)	0.004 (0.003 - 0.005)	0.004 (0.004 - 0.006)	0.004 (0.004 - 0.006)	0.326 (0.123 - 0.701)	0.328 (0.123 - 0.702)	0.340 (0.129 - 0.730)
Age-standardized (rate per capita)	0.092 (0.074 - 0.115)	0.092 (0.074 - 0.115)	0.093 (0.074 - 0.116)	0.007 (0.005 - 0.008)	0.007 (0.005 - 0.008)	0.007 (0.005 - 0.008)	0.590 (0.225 - 1.274)	0.594 (0.225 - 1.288)	0.601 (0.227 - 1.303)
Total number of cases (thousands)	24,590 (19,471 - 31,806)	36,816 (29,334 - 47,491)	49,927 (39,706 - 64,697)	2,096 (1,689 - 2,641)	3,183 (2,575 - 4,051)	4,319 (3,487 - 5,542)	160 (61 - 344)	240 (90 - 514)	326 (124 - 701)
**Southern Sub-Saharan Africa**									
15 - 49 years (rate per capita)	0.031 (0.026 - 0.036)	0.031 (0.026 - 0.037)	0.032 (0.027 - 0.038)	0.004 (0.003 - 0.004)	0.004 (0.003 - 0.004)	0.004 (0.003 - 0.004)	0.204 (0.077 - 0.430)	0.205 (0.078 - 0.430)	0.211 (0.081 - 0.441)
50 - 69 years (rate per capita)	0.101 (0.084 - 0.120)	0.099 (0.083 - 0.118)	0.100 (0.083 - 0.119)	0.007 (0.005 - 0.008)	0.007 (0.005 - 0.008)	0.007 (0.005 - 0.008)	0.649 (0.254 - 1.313)	0.636 (0.249 - 1.287)	0.638 (0.250 - 1.289)
70+ years (rate per capita)	0.100 (0.085 - 0.117)	0.099 (0.085 - 0.116)	0.099 (0.085 - 0.116)	0.005 (0.004 - 0.007)	0.005 (0.004 - 0.007)	0.005 (0.004 - 0.006)	0.617 (0.243 - 1.257)	0.611 (0.241 - 1.241)	0.610 (0.241 - 1.227)
All ages (rate per capita)	0.026 (0.023 - 0.030)	0.029 (0.026 - 0.034)	0.032 (0.028 - 0.037)	0.002 (0.002 - 0.003)	0.003 (0.002 - 0.003)	0.003 (0.003 - 0.003)	0.170 (0.066 - 0.350)	0.190 (0.074 - 0.393)	0.207 (0.080 - 0.427)
Age-standardized (rate per capita)	0.042 (0.037 - 0.049)	0.042 (0.037 - 0.048)	0.042 (0.037 - 0.049)	0.004 (0.003 - 0.004)	0.004 (0.003 - 0.004)	0.004 (0.003 - 0.004)	0.275 (0.106 - 0.572)	0.271 (0.105 - 0.564)	0.272 (0.106 - 0.566)
Total number of cases (thousands)	1,390 (1,215 - 1,604)	2,005 (1,746 - 2,317)	2,470 (2,158 - 2,870)	132 (115 - 153)	189 (164 - 218)	230 (199 - 266)	9 (4 - 19)	13 (5 - 27)	16 (6 - 33)
**Botswana**									
15 - 49 years (rate per capita)	0.029 (0.024 - 0.035)	0.029 (0.024 - 0.034)	0.032 (0.027 - 0.038)	0.003 (0.003 - 0.004)	0.003 (0.003 - 0.004)	0.004 (0.003 - 0.004)	0.194 (0.074 - 0.406)	0.189 (0.073 - 0.397)	0.210 (0.080 - 0.446)
50 - 69 years (rate per capita)	0.096 (0.080 - 0.116)	0.095 (0.079 - 0.113)	0.096 (0.080 - 0.116)	0.007 (0.005 - 0.008)	0.007 (0.005 - 0.008)	0.007 (0.005 - 0.008)	0.621 (0.244 - 1.251)	0.608 (0.238 - 1.243)	0.614 (0.236 - 1.239)
70+ years (rate per capita)	0.096 (0.081 - 0.115)	0.095 (0.080 - 0.114)	0.096 (0.080 - 0.115)	0.005 (0.004 - 0.007)	0.005 (0.004 - 0.006)	0.005 (0.004 - 0.006)	0.596 (0.236 - 1.206)	0.589 (0.233 - 1.195)	0.591 (0.233 - 1.203)
All ages (rate per capita)	0.022 (0.019 - 0.026)	0.025 (0.022 - 0.030)	0.030 (0.026 - 0.034)	0.002 (0.002 - 0.003)	0.002 (0.002 - 0.003)	0.003 (0.002 - 0.003)	0.144 (0.055 - 0.291)	0.165 (0.064 - 0.336)	0.191 (0.074 - 0.392)
Age-standardized (rate per capita)	0.041 (0.036 - 0.048)	0.041 (0.035 - 0.047)	0.041 (0.035 - 0.047)	0.003 (0.003 - 0.004)	0.003 (0.003 - 0.004)	0.003 (0.003 - 0.004)	0.266 (0.104 - 0.535)	0.261 (0.102 - 0.528)	0.262 (0.102 - 0.535)
Total number of cases (thousands)	30 (26 - 35)	47 (41 - 55)	67 (58 - 78)	3 (3 - 3)	5 (4 - 5)	6 (6 - 7)	0 (0 - 0)	0 (0 - 1)	0 (0 - 1)
**Lesotho**									
15 - 49 years (rate per capita)	0.028 (0.023 - 0.033)	0.027 (0.023 - 0.032)	0.027 (0.023 - 0.032)	0.003 (0.003 - 0.004)	0.003 (0.003 - 0.004)	0.003 (0.003 - 0.004)	0.185 (0.071 - 0.388)	0.178 (0.068 - 0.373)	0.180 (0.069 - 0.374)
50 - 69 years (rate per capita)	0.097 (0.080 - 0.115)	0.095 (0.079 - 0.113)	0.096 (0.080 - 0.115)	0.007 (0.005 - 0.008)	0.006 (0.005 - 0.008)	0.007 (0.005 - 0.008)	0.624 (0.245 - 1.261)	0.606 (0.239 - 1.236)	0.613 (0.240 - 1.239)
70+ years (rate per capita)	0.096 (0.081 - 0.113)	0.095 (0.080 - 0.112)	0.094 (0.081 - 0.112)	0.005 (0.004 - 0.007)	0.005 (0.004 - 0.006)	0.005 (0.004 - 0.006)	0.597 (0.236 - 1.215)	0.583 (0.231 - 1.185)	0.580 (0.230 - 1.187)
All ages (rate per capita)	0.024 (0.021 - 0.028)	0.024 (0.021 - 0.028)	0.025 (0.022 - 0.029)	0.002 (0.002 - 0.003)	0.002 (0.002 - 0.003)	0.002 (0.002 - 0.003)	0.156 (0.061 - 0.323)	0.158 (0.062 - 0.324)	0.161 (0.063 - 0.331)
Age-standardized (rate per capita)	0.041 (0.036 - 0.047)	0.040 (0.035 - 0.046)	0.041 (0.035 - 0.047)	0.003 (0.003 - 0.004)	0.003 (0.003 - 0.004)	0.003 (0.003 - 0.004)	0.265 (0.104 - 0.545)	0.260 (0.101 - 0.532)	0.261 (0.102 - 0.531)
Total number of cases (thousands)	38 (33 - 44)	47 (41 - 54)	53 (46 - 61)	4 (3 - 4)	4 (4 - 5)	5 (5 - 6)	0 (0 - 1)	0 (0 - 1)	0 (0 - 1)
**Namibia**									

	Prevalence 1990	Prevalence 2005	Prevalence 2015	Incidence 1990	Incidence 2005	Incidence 2015	YLD rate 1990	YLD rate 2005	YLD rate 2015
15 - 49 years (rate per capita)	0.028 (0.023 - 0.033)	0.029 (0.024 - 0.034)	0.029 (0.024 - 0.035)	0.003 (0.003 - 0.004)	0.003 (0.003 - 0.004)	0.004 (0.003 - 0.004)	0.183 (0.070 - 0.376)	0.189 (0.073 - 0.395)	0.194 (0.075 - 0.402)
50 - 69 years (rate per capita)	0.091 (0.076 - 0.109)	0.089 (0.074 - 0.107)	0.090 (0.075 - 0.108)	0.007 (0.005 - 0.008)	0.007 (0.005 - 0.008)	0.007 (0.005 - 0.008)	0.587 (0.228 - 1.191)	0.574 (0.223 - 1.165)	0.578 (0.223 - 1.163)
70+ years (rate per capita)	0.086 (0.073 - 0.102)	0.086 (0.073 - 0.101)	0.086 (0.073 - 0.101)	0.005 (0.004 - 0.007)	0.005 (0.004 - 0.006)	0.005 (0.004 - 0.006)	0.536 (0.210 - 1.097)	0.530 (0.207 - 1.093)	0.532 (0.208 - 1.081)
All ages (rate per capita)	0.022 (0.019 - 0.025)	0.023 (0.020 - 0.027)	0.025 (0.022 - 0.030)	0.002 (0.002 - 0.003)	0.002 (0.002 - 0.003)	0.003 (0.002 - 0.003)	0.142 (0.054 - 0.293)	0.151 (0.058 - 0.310)	0.166 (0.065 - 0.341)
Age-standardized (rate per capita)	0.039 (0.034 - 0.045)	0.038 (0.033 - 0.044)	0.038 (0.034 - 0.045)	0.003 (0.003 - 0.004)	0.003 (0.003 - 0.004)	0.003 (0.003 - 0.004)	0.252 (0.098 - 0.516)	0.247 (0.097 - 0.513)	0.249 (0.097 - 0.511)
Total number of cases (thousands)	31 (27 - 36)	47 (41 - 55)	62 (54 - 73)	3 (3 - 4)	5 (4 - 6)	6 (5 - 7)	0 (0 - 0)	0 (0 - 1)	0 (0 - 1)
**South Africa**									
15 - 49 years (rate per capita)	0.032 (0.027 - 0.038)	0.033 (0.027 - 0.039)	0.033 (0.028 - 0.040)	0.004 (0.003 - 0.005)	0.004 (0.003 - 0.005)	0.004 (0.003 - 0.005)	0.212 (0.080 - 0.448)	0.216 (0.082 - 0.453)	0.220 (0.084 - 0.464)
50 - 69 years (rate per capita)	0.102 (0.085 - 0.121)	0.100 (0.084 - 0.119)	0.100 (0.084 - 0.120)	0.007 (0.005 - 0.008)	0.007 (0.005 - 0.008)	0.007 (0.005 - 0.009)	0.656 (0.255 - 1.332)	0.641 (0.250 - 1.300)	0.643 (0.251 - 1.298)
70+ years (rate per capita)	0.100 (0.086 - 0.117)	0.100 (0.086 - 0.116)	0.100 (0.086 - 0.116)	0.005 (0.004 - 0.007)	0.005 (0.004 - 0.007)	0.005 (0.004 - 0.007)	0.619 (0.243 - 1.259)	0.614 (0.243 - 1.246)	0.613 (0.243 - 1.227)
All ages (rate per capita)	0.028 (0.024 - 0.032)	0.032 (0.028 - 0.037)	0.036 (0.031 - 0.041)	0.003 (0.002 - 0.003)	0.003 (0.003 - 0.003)	0.003 (0.003 - 0.004)	0.182 (0.071 - 0.375)	0.209 (0.081 - 0.432)	0.230 (0.089 - 0.478)
Age-standardized (rate per capita)	0.043 (0.037 - 0.049)	0.042 (0.037 - 0.049)	0.043 (0.037 - 0.049)	0.004 (0.003 - 0.004)	0.004 (0.003 - 0.004)	0.004 (0.003 - 0.004)	0.278 (0.108 - 0.579)	0.273 (0.106 - 0.568)	0.274 (0.106 - 0.572)
Total number of cases (thousands)	1,050 (917 - 1,211)	1,563 (1,358 - 1,809)	1,912 (1,665 - 2,221)	99 (86 - 115)	145 (126 - 168)	175 (151 - 202)	7 (3 - 14)	10 (4 - 21)	12 (5 - 26)
**Swaziland**									
15 - 49 years (rate per capita)	0.028 (0.024 - 0.034)	0.027 (0.023 - 0.032)	0.027 (0.023 - 0.032)	0.003 (0.003 - 0.004)	0.003 (0.003 - 0.004)	0.003 (0.003 - 0.004)	0.188 (0.072 - 0.396)	0.177 (0.067 - 0.366)	0.177 (0.068 - 0.373)
50 - 69 years (rate per capita)	0.098 (0.081 - 0.117)	0.097 (0.081 - 0.116)	0.098 (0.082 - 0.116)	0.007 (0.005 - 0.008)	0.007 (0.005 - 0.008)	0.007 (0.005 - 0.008)	0.630 (0.245 - 1.271)	0.615 (0.241 - 1.241)	0.619 (0.244 - 1.265)
70+ years (rate per capita)	0.100 (0.085 - 0.118)	0.099 (0.084 - 0.117)	0.099 (0.084 - 0.117)	0.005 (0.004 - 0.007)	0.005 (0.004 - 0.006)	0.005 (0.004 - 0.006)	0.619 (0.244 - 1.245)	0.606 (0.241 - 1.220)	0.607 (0.239 - 1.224)
All ages (rate per capita)	0.021 (0.018 - 0.024)	0.023 (0.020 - 0.026)	0.024 (0.021 - 0.028)	0.002 (0.002 - 0.002)	0.002 (0.002 - 0.003)	0.002 (0.002 - 0.003)	0.137 (0.052 - 0.283)	0.146 (0.057 - 0.298)	0.157 (0.061 - 0.326)
Age-standardized (rate per capita)	0.041 (0.036 - 0.048)	0.041 (0.036 - 0.047)	0.041 (0.036 - 0.048)	0.003 (0.003 - 0.004)	0.003 (0.003 - 0.004)	0.003 (0.003 - 0.004)	0.268 (0.104 - 0.554)	0.262 (0.102 - 0.540)	0.263 (0.102 - 0.543)
Total number of cases (thousands)	18 (16 - 21)	25 (22 - 29)	31 (27 - 37)	2 (2 - 2)	2 (2 - 3)	3 (3 - 4)	0 (0 - 0)	0 (0 - 0)	0 (0 - 0)
**Zimbabwe**									
15 - 49 years (rate per capita)	0.028 (0.023 - 0.032)	0.026 (0.022 - 0.031)	0.028 (0.023 - 0.033)	0.003 (0.003 - 0.004)	0.003 (0.003 - 0.004)	0.003 (0.003 - 0.004)	0.183 (0.069 - 0.384)	0.170 (0.065 - 0.357)	0.186 (0.071 - 0.394)
50 - 69 years (rate per capita)	0.098 (0.082 - 0.118)	0.098 (0.082 - 0.117)	0.099 (0.082 - 0.118)	0.007 (0.005 - 0.008)	0.007 (0.005 - 0.008)	0.007 (0.005 - 0.008)	0.637 (0.249 - 1.284)	0.630 (0.248 - 1.274)	0.634 (0.248 - 1.296)
70+ years (rate per capita)	0.100 (0.086 - 0.119)	0.100 (0.085 - 0.117)	0.100 (0.086 - 0.117)	0.005 (0.004 - 0.007)	0.005 (0.004 - 0.006)	0.005 (0.004 - 0.006)	0.626 (0.246 - 1.271)	0.616 (0.245 - 1.246)	0.618 (0.246 - 1.251)
All ages (rate per capita)	0.021 (0.018 - 0.024)	0.021 (0.018 - 0.025)	0.022 (0.019 - 0.026)	0.002 (0.002 - 0.002)	0.002 (0.002 - 0.002)	0.002 (0.002 - 0.003)	0.138 (0.053 - 0.287)	0.137 (0.053 - 0.288)	0.144 (0.056 - 0.299)
Age-standardized (rate per capita)	0.041 (0.036 - 0.048)	0.041 (0.036 - 0.048)	0.042 (0.036 - 0.048)	0.003 (0.003 - 0.004)	0.003 (0.003 - 0.004)	0.003 (0.003 - 0.004)	0.268 (0.105 - 0.560)	0.267 (0.104 - 0.554)	0.269 (0.104 - 0.560)
Total number of cases (thousands)	222 (194 - 256)	276 (240 - 320)	345 (299 - 400)	21 (19 - 25)	27 (24 - 32)	34 (30 - 40)	1 (1 - 3)	2 (1 - 4)	2 (1 - 5)
**Western Sub-Saharan Africa**									
15 - 49 years (rate per capita)	0.073 (0.057 - 0.096)	0.073 (0.057 - 0.097)	0.075 (0.059 - 0.099)	0.008 (0.006 - 0.011)	0.008 (0.006 - 0.011)	0.008 (0.006 - 0.011)	0.482 (0.175 - 1.027)	0.485 (0.176 - 1.038)	0.499 (0.181 - 1.063)
50 - 69 years (rate per capita)	0.244 (0.189 - 0.299)	0.248 (0.192 - 0.304)	0.249 (0.194 - 0.307)	0.013 (0.009 - 0.017)	0.013 (0.009 - 0.017)	0.013 (0.009 - 0.017)	1.563 (0.612 - 3.327)	1.595 (0.624 - 3.359)	1.606 (0.629 - 3.398)
70+ years (rate per capita)	0.256 (0.207 - 0.308)	0.260 (0.210 - 0.313)	0.258 (0.210 - 0.310)	0.009 (0.006 - 0.014)	0.009 (0.006 - 0.014)	0.009 (0.006 - 0.014)	1.575 (0.607 - 3.257)	1.602 (0.616 - 3.295)	1.597 (0.618 - 3.284)
All ages (rate per capita)	0.057 (0.047 - 0.071)	0.058 (0.047 - 0.072)	0.059 (0.048 - 0.074)	0.005 (0.004 - 0.006)	0.005 (0.004 - 0.006)	0.005 (0.004 - 0.006)	0.373 (0.143 - 0.799)	0.378 (0.145 - 0.816)	0.386 (0.146 - 0.830)
Age-standardized (rate per capita)	0.103 (0.084 - 0.125)	0.105 (0.086 - 0.128)	0.105 (0.086 - 0.128)	0.007 (0.006 - 0.009)	0.007 (0.006 - 0.009)	0.007 (0.006 - 0.009)	0.661 (0.253 - 1.418)	0.677 (0.260 - 1.450)	0.682 (0.260 - 1.469)
Total number of cases (thousands)	11,416 (9,293 - 14,106)	17,235 (14,068 - 21,505)	23,055 (18,799 - 28,924)	950 (779 - 1,174)	1,461 (1,202 - 1,832)	1,959 (1,606 - 2,448)	74 (28 - 159)	112 (43 - 242)	151 (57 - 325)
**Benin**									
15 - 49 years (rate per capita)	0.096 (0.070 - 0.136)	0.097 (0.070 - 0.139)	0.099 (0.071 - 0.142)	0.010 (0.008 - 0.015)	0.011 (0.008 - 0.015)	0.011 (0.008 - 0.015)	0.630 (0.224 - 1.373)	0.640 (0.230 - 1.417)	0.655 (0.230 - 1.431)
50 - 69 years (rate per capita)	0.321 (0.227 - 0.407)	0.321 (0.226 - 0.410)	0.322 (0.231 - 0.408)	0.016 (0.009 - 0.022)	0.016 (0.009 - 0.022)	0.016 (0.010 - 0.022)	2.048 (0.773 - 4.509)	2.060 (0.791 - 4.447)	2.077 (0.792 - 4.483)
70+ years (rate per capita)	0.318 (0.240 - 0.399)	0.316 (0.238 - 0.394)	0.319 (0.242 - 0.397)	0.011 (0.006 - 0.018)	0.011 (0.006 - 0.018)	0.011 (0.006 - 0.018)	1.950 (0.745 - 4.067)	1.950 (0.741 - 4.046)	1.974 (0.749 - 4.118)
All ages (rate per capita)	0.074 (0.057 - 0.098)	0.074 (0.057 - 0.100)	0.079 (0.059 - 0.105)	0.006 (0.005 - 0.008)	0.006 (0.005 - 0.008)	0.007 (0.005 - 0.009)	0.481 (0.181 - 1.033)	0.486 (0.181 - 1.052)	0.516 (0.192 - 1.121)
Age-standardized (rate per capita)	0.135 (0.105 - 0.173)	0.135 (0.104 - 0.174)	0.136 (0.104 - 0.174)	0.009 (0.007 - 0.012)	0.009 (0.007 - 0.012)	0.009 (0.007 - 0.012)	0.866 (0.329 - 1.911)	0.872 (0.330 - 1.894)	0.880 (0.331 - 1.903)
Total number of cases (thousands)	371 (286 - 491)	610 (463 - 815)	861 (649 - 1,146)	31 (24 - 40)	52 (40 - 69)	72 (56 - 95)	2 (1 - 5)	4 (1 - 9)	6 (2 - 12)
**Burkina Faso**									
15 - 49 years (rate per capita)	0.093 (0.067 - 0.132)	0.092 (0.066 - 0.130)	0.095 (0.068 - 0.137)	0.010 (0.008 - 0.014)	0.010 (0.007 - 0.015)	0.010 (0.008 - 0.015)	0.615 (0.224 - 1.347)	0.611 (0.221 - 1.328)	0.634 (0.226 - 1.424)
50 - 69 years (rate per capita)	0.327 (0.233 - 0.417)	0.325 (0.231 - 0.410)	0.325 (0.233 - 0.414)	0.016 (0.009 - 0.022)	0.016 (0.010 - 0.022)	0.016 (0.010 - 0.022)	2.092 (0.805 - 4.579)	2.091 (0.791 - 4.530)	2.106 (0.797 - 4.639)
70+ years (rate per capita)	0.329 (0.248 - 0.407)	0.325 (0.247 - 0.405)	0.327 (0.251 - 0.407)	0.011 (0.006 - 0.018)	0.011 (0.006 - 0.018)	0.011 (0.006 - 0.018)	2.020 (0.760 - 4.279)	2.010 (0.743 - 4.195)	2.038 (0.763 - 4.324)
All ages (rate per capita)	0.072 (0.055 - 0.095)	0.068 (0.052 - 0.091)	0.071 (0.053 - 0.094)	0.006 (0.005 - 0.008)	0.006 (0.005 - 0.008)	0.006 (0.005 - 0.008)	0.467 (0.175 - 1.004)	0.448 (0.166 - 0.974)	0.466 (0.172 - 1.024)
Age-standardized (rate per capita)	0.137 (0.106 - 0.178)	0.136 (0.105 - 0.175)	0.137 (0.106 - 0.176)	0.009 (0.007 - 0.012)	0.009 (0.007 - 0.012)	0.009 (0.007 - 0.012)	0.881 (0.333 - 1.900)	0.882 (0.330 - 1.917)	0.891 (0.338 - 1.919)
Total number of cases (thousands)	634 (487 - 838)	921 (699 - 1,222)	1,282 (966 - 1,707)	52 (40 - 67)	79 (61 - 105)	110 (85 - 146)	4 (2 - 9)	6 (2 - 13)	8 (3 - 19)
**Cameroon**									
15 - 49 years (rate per capita)	0.097 (0.070 - 0.140)	0.092 (0.066 - 0.132)	0.095 (0.069 - 0.138)	0.011 (0.008 - 0.015)	0.010 (0.007 - 0.015)	0.010 (0.008 - 0.015)	0.639 (0.226 - 1.381)	0.607 (0.217 - 1.349)	0.632 (0.224 - 1.387)
50 - 69 years (rate per capita)	0.323 (0.231 - 0.412)	0.322 (0.231 - 0.409)	0.323 (0.231 - 0.409)	0.016 (0.010 - 0.022)	0.016 (0.010 - 0.022)	0.016 (0.010 - 0.022)	2.060 (0.783 - 4.538)	2.057 (0.794 - 4.470)	2.072 (0.797 - 4.554)
70+ years (rate per capita)	0.316 (0.241 - 0.391)	0.315 (0.237 - 0.388)	0.315 (0.239 - 0.390)	0.011 (0.006 - 0.018)	0.011 (0.006 - 0.018)	0.011 (0.006 - 0.018)	1.928 (0.741 - 4.036)	1.922 (0.735 - 4.094)	1.928 (0.722 - 4.039)
All ages (rate per capita)	0.076 (0.058 - 0.102)	0.073 (0.056 - 0.097)	0.076 (0.058 - 0.102)	0.006 (0.005 - 0.008)	0.006 (0.005 - 0.008)	0.006 (0.005 - 0.009)	0.493 (0.182 - 1.065)	0.476 (0.175 - 1.033)	0.496 (0.184 - 1.070)
Age-standardized (rate per capita)	0.136 (0.104 - 0.175)	0.135 (0.105 - 0.174)	0.136 (0.106 - 0.176)	0.009 (0.007 - 0.012)	0.009 (0.007 - 0.012)	0.009 (0.007 - 0.012)	0.870 (0.327 - 1.909)	0.868 (0.325 - 1.898)	0.877 (0.332 - 1.926)
Total number of cases (thousands)	921 (701 - 1,228)	1,332 (1,021 - 1,769)	1,778 (1,367 - 2,377)	74 (57 - 96)	112 (86 - 149)	151 (117 - 201)	6 (2 - 13)	9 (3 - 19)	12 (4 - 25)
**Cape Verde**									
15 - 49 years (rate per capita)	0.081 (0.059 - 0.115)	0.094 (0.068 - 0.135)	0.102 (0.073 - 0.146)	0.010 (0.007 - 0.014)	0.010 (0.008 - 0.015)	0.011 (0.008 - 0.016)	0.543 (0.198 - 1.188)	0.625 (0.223 - 1.366)	0.678 (0.240 - 1.496)
50 - 69 years (rate per capita)	0.321 (0.228 - 0.407)	0.321 (0.231 - 0.404)	0.316 (0.224 - 0.405)	0.016 (0.010 - 0.022)	0.016 (0.009 - 0.022)	0.016 (0.010 - 0.023)	2.077 (0.792 - 4.595)	2.072 (0.794 - 4.565)	2.054 (0.773 - 4.529)
70+ years (rate per capita)	0.311 (0.232 - 0.384)	0.318 (0.240 - 0.395)	0.302 (0.227 - 0.374)	0.011 (0.006 - 0.017)	0.011 (0.006 - 0.018)	0.011 (0.006 - 0.016)	1.924 (0.731 - 4.020)	1.973 (0.743 - 4.138)	1.869 (0.712 - 3.925)
All ages (rate per capita)	0.073 (0.058 - 0.094)	0.084 (0.065 - 0.111)	0.101 (0.077 - 0.135)	0.006 (0.005 - 0.007)	0.007 (0.005 - 0.009)	0.008 (0.006 - 0.011)	0.477 (0.179 - 1.033)	0.548 (0.204 - 1.175)	0.663 (0.246 - 1.452)
Age-standardized (rate per capita)	0.134 (0.105 - 0.172)	0.135 (0.104 - 0.174)	0.135 (0.105 - 0.176)	0.009 (0.007 - 0.012)	0.009 (0.007 - 0.012)	0.009 (0.007 - 0.012)	0.872 (0.330 - 1.917)	0.875 (0.330 - 1.914)	0.881 (0.331 - 1.925)
Total number of cases (thousands)	25 (20 - 32)	40 (31 - 53)	53 (40 - 70)	2 (2 - 3)	3 (3 - 4)	4 (3 - 6)	0 (0 - 0)	0 (0 - 1)	0 (0 - 1)
**Chad**									
15 - 49 years (rate per capita)	0.096 (0.070 - 0.137)	0.091 (0.066 - 0.130)	0.090 (0.065 - 0.128)	0.011 (0.008 - 0.015)	0.010 (0.008 - 0.014)	0.010 (0.008 - 0.015)	0.632 (0.225 - 1.367)	0.603 (0.219 - 1.338)	0.597 (0.213 - 1.296)
50 - 69 years (rate per capita)	0.320 (0.228 - 0.409)	0.318 (0.225 - 0.407)	0.320 (0.227 - 0.405)	0.016 (0.010 - 0.022)	0.016 (0.010 - 0.022)	0.016 (0.010 - 0.022)	2.049 (0.774 - 4.510)	2.034 (0.784 - 4.399)	2.048 (0.773 - 4.418)
70+ years (rate per capita)	0.313 (0.240 - 0.387)	0.311 (0.235 - 0.387)	0.312 (0.236 - 0.386)	0.011 (0.006 - 0.018)	0.011 (0.006 - 0.018)	0.011 (0.006 - 0.018)	1.920 (0.741 - 4.020)	1.906 (0.730 - 3.976)	1.910 (0.729 - 3.995)
All ages (rate per capita)	0.073 (0.055 - 0.097)	0.066 (0.050 - 0.088)	0.065 (0.050 - 0.087)	0.006 (0.005 - 0.008)	0.006 (0.004 - 0.007)	0.006 (0.004 - 0.008)	0.472 (0.176 - 1.018)	0.427 (0.159 - 0.918)	0.426 (0.159 - 0.922)
Age-standardized (rate per capita)	0.134 (0.104 - 0.174)	0.134 (0.103 - 0.174)	0.135 (0.104 - 0.174)	0.009 (0.007 - 0.012)	0.009 (0.007 - 0.012)	0.009 (0.007 - 0.012)	0.865 (0.325 - 1.896)	0.863 (0.326 - 1.859)	0.868 (0.324 - 1.869)
Total number of cases (thousands)	432 (330 - 578)	661 (505 - 881)	919 (707 - 1,228)	36 (27 - 47)	57 (44 - 75)	81 (63 - 107)	3 (1 - 6)	4 (2 - 9)	6 (2 - 13)
**Cote d'Ivoire**									
15 - 49 years (rate per capita)	0.101 (0.074 - 0.147)	0.097 (0.070 - 0.141)	0.096 (0.070 - 0.139)	0.011 (0.008 - 0.016)	0.011 (0.008 - 0.015)	0.011 (0.008 - 0.015)	0.668 (0.238 - 1.445)	0.642 (0.227 - 1.413)	0.637 (0.226 - 1.393)
50 - 69 years (rate per capita)	0.316 (0.225 - 0.400)	0.318 (0.228 - 0.404)	0.318 (0.225 - 0.401)	0.017 (0.010 - 0.023)	0.017 (0.010 - 0.023)	0.017 (0.010 - 0.022)	2.017 (0.767 - 4.444)	2.041 (0.782 - 4.419)	2.043 (0.787 - 4.471)
70+ years (rate per capita)	0.307 (0.231 - 0.383)	0.312 (0.240 - 0.386)	0.311 (0.241 - 0.384)	0.011 (0.006 - 0.019)	0.011 (0.006 - 0.019)	0.011 (0.006 - 0.019)	1.861 (0.703 - 3.904)	1.914 (0.709 - 4.014)	1.913 (0.709 - 3.996)
All ages (rate per capita)	0.078 (0.059 - 0.104)	0.077 (0.059 - 0.103)	0.078 (0.059 - 0.103)	0.007 (0.005 - 0.009)	0.006 (0.005 - 0.009)	0.007 (0.005 - 0.009)	0.504 (0.186 - 1.087)	0.500 (0.183 - 1.089)	0.507 (0.188 - 1.083)

	Prevalence 1990	Prevalence 2005	Prevalence 2015	Incidence 1990	Incidence 2005	Incidence 2015	YLD rate 1990	YLD rate 2005	YLD rate 2015
**Age-standardized (rate per capita)**	0.134 (0.104 - 0.174)	0.134 (0.104 - 0.174)	0.134 (0.104 - 0.172)	0.010 (0.007 - 0.012)	0.010 (0.007 - 0.012)	0.009 (0.007 - 0.012)	0.857 (0.320 - 1.851)	0.861 (0.321 - 1.857)	0.864 (0.325 - 1.882)
**Total number of cases (thousands)**	944 (717 - 1,263)	1,390 (1,070 - 1,862)	1,762 (1,343 - 2,347)	79 (62 - 104)	118 (91 - 155)	150 (116 - 198)	6 (2 - 13)	9 (3 - 20)	12 (4 - 25)
**The Gambia**									
**15 - 49 years (rate per capita)**	0.135 (0.114 - 0.158)	0.136 (0.115 - 0.159)	0.138 (0.117 - 0.162)	0.014 (0.012 - 0.016)	0.014 (0.012 - 0.016)	0.014 (0.012 - 0.016)	0.893 (0.341 - 1.833)	0.899 (0.346 - 1.865)	0.916 (0.350 - 1.880)
**50 - 69 years (rate per capita)**	0.413 (0.363 - 0.468)	0.411 (0.362 - 0.467)	0.409 (0.359 - 0.467)	0.017 (0.012 - 0.020)	0.017 (0.012 - 0.020)	0.017 (0.012 - 0.021)	2.667 (1.046 - 5.489)	2.657 (1.040 - 5.432)	2.649 (1.042 - 5.409)
**70+ years (rate per capita)**	0.392 (0.344 - 0.444)	0.387 (0.340 - 0.437)	0.388 (0.342 - 0.439)	0.009 (0.006 - 0.013)	0.009 (0.006 - 0.013)	0.009 (0.006 - 0.013)	2.441 (0.986 - 4.895)	2.404 (0.967 - 4.859)	2.414 (0.973 - 4.878)
**All ages (rate per capita)**	0.094 (0.083 - 0.107)	0.094 (0.083 - 0.107)	0.095 (0.084 - 0.109)	0.008 (0.007 - 0.009)	0.007 (0.007 - 0.009)	0.008 (0.007 - 0.009)	0.617 (0.244 - 1.261)	0.615 (0.239 - 1.262)	0.627 (0.245 - 1.310)
**Age-standardized (rate per capita)**	0.180 (0.161 - 0.202)	0.179 (0.161 - 0.202)	0.180 (0.161 - 0.202)	0.011 (0.010 - 0.012)	0.011 (0.010 - 0.012)	0.011 (0.010 - 0.012)	1.171 (0.461 - 2.408)	1.166 (0.458 - 2.403)	1.168 (0.457 - 2.411)
**Total number of cases (thousands)**	87 (76 - 98)	136 (120 - 155)	191 (168 - 217)	7 (6 - 8)	11 (10 - 12)	15 (13 - 17)	1 (0 - 1)	1 (0 - 2)	1 (0 - 3)
**Ghana**									
**15 - 49 years (rate per capita)**	0.097 (0.070 - 0.138)	0.099 (0.072 - 0.142)	0.103 (0.075 - 0.148)	0.011 (0.008 - 0.015)	0.011 (0.008 - 0.015)	0.011 (0.008 - 0.016)	0.640 (0.229 - 1.414)	0.655 (0.231 - 1.436)	0.685 (0.240 - 1.492)
**50 - 69 years (rate per capita)**	0.327 (0.231 - 0.414)	0.328 (0.231 - 0.417)	0.327 (0.235 - 0.417)	0.016 (0.010 - 0.022)	0.016 (0.010 - 0.022)	0.016 (0.010 - 0.022)	2.096 (0.800 - 4.569)	2.113 (0.808 - 4.630)	2.113 (0.801 - 4.668)
**70+ years (rate per capita)**	0.330 (0.250 - 0.408)	0.326 (0.246 - 0.406)	0.323 (0.245 - 0.398)	0.011 (0.006 - 0.018)	0.011 (0.006 - 0.018)	0.011 (0.006 - 0.018)	2.028 (0.770 - 4.274)	2.019 (0.754 - 4.245)	1.999 (0.777 - 4.196)
**All ages (rate per capita)**	0.077 (0.058 - 0.101)	0.083 (0.063 - 0.111)	0.088 (0.067 - 0.118)	0.006 (0.005 - 0.008)	0.007 (0.005 - 0.009)	0.007 (0.006 - 0.010)	0.499 (0.187 - 1.075)	0.543 (0.203 - 1.180)	0.575 (0.213 - 1.246)
**Age-standardized (rate per capita)**	0.138 (0.106 - 0.176)	0.138 (0.106 - 0.180)	0.137 (0.107 - 0.179)	0.009 (0.007 - 0.012)	0.009 (0.007 - 0.012)	0.009 (0.007 - 0.012)	0.886 (0.335 - 1.951)	0.890 (0.334 - 1.919)	0.892 (0.333 - 1.924)
**Total number of cases (thousands)**	1,122 (851 - 1,481)	1,778 (1,342 - 2,366)	2,408 (1,839 - 3,246)	93 (72 - 123)	146 (112 - 193)	197 (151 - 261)	7 (3 - 16)	12 (4 - 25)	16 (6 - 34)
**Guinea**									
**15 - 49 years (rate per capita)**	0.101 (0.073 - 0.146)	0.098 (0.072 - 0.141)	0.097 (0.071 - 0.139)	0.011 (0.008 - 0.016)	0.011 (0.008 - 0.015)	0.011 (0.008 - 0.015)	0.665 (0.237 - 1.444)	0.649 (0.232 - 1.440)	0.644 (0.227 - 1.403)
**50 - 69 years (rate per capita)**	0.323 (0.230 - 0.414)	0.321 (0.227 - 0.410)	0.324 (0.230 - 0.411)	0.016 (0.010 - 0.022)	0.016 (0.010 - 0.022)	0.016 (0.010 - 0.022)	2.074 (0.788 - 4.590)	2.069 (0.796 - 4.470)	2.090 (0.791 - 4.513)
**70+ years (rate per capita)**	0.322 (0.248 - 0.400)	0.320 (0.240 - 0.398)	0.320 (0.246 - 0.398)	0.011 (0.006 - 0.019)	0.011 (0.006 - 0.018)	0.011 (0.006 - 0.019)	1.992 (0.748 - 4.187)	1.979 (0.754 - 4.110)	1.988 (0.754 - 4.200)
**All ages (rate per capita)**	0.082 (0.062 - 0.109)	0.078 (0.059 - 0.104)	0.080 (0.061 - 0.105)	0.007 (0.005 - 0.009)	0.006 (0.005 - 0.008)	0.007 (0.005 - 0.009)	0.532 (0.200 - 1.155)	0.510 (0.191 - 1.100)	0.523 (0.196 - 1.129)
**Age-standardized (rate per capita)**	0.135 (0.105 - 0.176)	0.135 (0.104 - 0.175)	0.136 (0.105 - 0.176)	0.009 (0.007 - 0.012)	0.009 (0.007 - 0.012)	0.009 (0.007 - 0.012)	0.874 (0.329 - 1.919)	0.876 (0.332 - 1.901)	0.882 (0.332 - 1.911)
**Total number of cases (thousands)**	493 (374 - 656)	758 (574 - 1,009)	1,006 (768 - 1,325)	40 (30 - 52)	62 (48 - 81)	83 (65 - 109)	3 (1 - 7)	5 (2 - 11)	7 (2 - 14)
**Guinea-Bissau**									
**15 - 49 years (rate per capita)**	0.101 (0.073 - 0.145)	0.096 (0.069 - 0.139)	0.098 (0.071 - 0.142)	0.011 (0.008 - 0.015)	0.010 (0.008 - 0.015)	0.011 (0.008 - 0.015)	0.669 (0.240 - 1.439)	0.635 (0.225 - 1.398)	0.651 (0.232 - 1.431)
**50 - 69 years (rate per capita)**	0.323 (0.230 - 0.410)	0.322 (0.228 - 0.412)	0.323 (0.230 - 0.408)	0.016 (0.010 - 0.022)	0.017 (0.010 - 0.022)	0.016 (0.010 - 0.022)	2.075 (0.788 - 4.534)	2.071 (0.787 - 4.557)	2.079 (0.806 - 4.556)
**70+ years (rate per capita)**	0.321 (0.245 - 0.403)	0.318 (0.243 - 0.396)	0.320 (0.245 - 0.396)	0.011 (0.006 - 0.018)	0.011 (0.006 - 0.018)	0.011 (0.006 - 0.019)	1.980 (0.748 - 4.203)	1.960 (0.738 - 4.088)	1.970 (0.737 - 4.129)
**All ages (rate per capita)**	0.081 (0.062 - 0.107)	0.079 (0.061 - 0.105)	0.082 (0.063 - 0.110)	0.006 (0.005 - 0.008)	0.007 (0.005 - 0.009)	0.007 (0.005 - 0.009)	0.525 (0.196 - 1.136)	0.515 (0.189 - 1.118)	0.536 (0.200 - 1.151)
**Age-standardized (rate per capita)**	0.135 (0.104 - 0.176)	0.135 (0.105 - 0.177)	0.136 (0.105 - 0.176)	0.009 (0.007 - 0.012)	0.009 (0.007 - 0.012)	0.009 (0.007 - 0.012)	0.874 (0.328 - 1.884)	0.876 (0.328 - 1.912)	0.877 (0.333 - 1.928)
**Total number of cases (thousands)**	85 (65 - 113)	116 (89 - 155)	152 (116 - 203)	7 (5 - 9)	10 (7 - 13)	13 (10 - 17)	1 (0 - 1)	1 (0 - 2)	1 (0 - 2)
**Liberia**									
**15 - 49 years (rate per capita)**	0.097 (0.071 - 0.139)	0.097 (0.069 - 0.140)	0.100 (0.073 - 0.145)	0.011 (0.008 - 0.015)	0.011 (0.008 - 0.015)	0.011 (0.008 - 0.015)	0.637 (0.230 - 1.359)	0.634 (0.225 - 1.398)	0.658 (0.233 - 1.441)
**50 - 69 years (rate per capita)**	0.321 (0.231 - 0.408)	0.323 (0.231 - 0.412)	0.322 (0.229 - 0.409)	0.016 (0.010 - 0.022)	0.016 (0.010 - 0.022)	0.016 (0.010 - 0.022)	2.039 (0.780 - 4.468)	2.048 (0.784 - 4.465)	2.052 (0.790 - 4.488)
**70+ years (rate per capita)**	0.323 (0.249 - 0.402)	0.321 (0.247 - 0.401)	0.321 (0.246 - 0.397)	0.011 (0.006 - 0.019)	0.011 (0.006 - 0.019)	0.011 (0.006 - 0.019)	1.952 (0.738 - 4.115)	1.936 (0.729 - 4.013)	1.951 (0.725 - 4.055)
**All ages (rate per capita)**	0.075 (0.058 - 0.099)	0.077 (0.059 - 0.103)	0.080 (0.061 - 0.108)	0.006 (0.005 - 0.008)	0.006 (0.005 - 0.009)	0.007 (0.005 - 0.009)	0.485 (0.181 - 1.049)	0.497 (0.183 - 1.079)	0.519 (0.193 - 1.120)
**Age-standardized (rate per capita)**	0.135 (0.105 - 0.174)	0.135 (0.105 - 0.177)	0.136 (0.105 - 0.176)	0.009 (0.007 - 0.012)	0.009 (0.007 - 0.012)	0.009 (0.007 - 0.012)	0.864 (0.325 - 1.868)	0.863 (0.322 - 1.868)	0.870 (0.329 - 1.901)
**Total number of cases (thousands)**	161 (124 - 212)	252 (194 - 338)	361 (275 - 486)	13 (10 - 17)	21 (16 - 28)	30 (23 - 40)	1 (0 - 2)	2 (1 - 4)	2 (1 - 5)
**Mali**									
**15 - 49 years (rate per capita)**	0.094 (0.070 - 0.135)	0.091 (0.065 - 0.130)	0.095 (0.069 - 0.139)	0.010 (0.008 - 0.015)	0.010 (0.007 - 0.015)	0.010 (0.008 - 0.015)	0.620 (0.222 - 1.359)	0.602 (0.216 - 1.351)	0.634 (0.223 - 1.399)
**50 - 69 years (rate per capita)**	0.325 (0.232 - 0.413)	0.324 (0.231 - 0.410)	0.325 (0.230 - 0.411)	0.016 (0.010 - 0.022)	0.016 (0.010 - 0.022)	0.016 (0.010 - 0.022)	2.074 (0.781 - 4.579)	2.072 (0.801 - 4.477)	2.093 (0.806 - 4.596)
**70+ years (rate per capita)**	0.328 (0.247 - 0.409)	0.323 (0.244 - 0.398)	0.323 (0.247 - 0.400)	0.011 (0.006 - 0.019)	0.011 (0.006 - 0.018)	0.011 (0.006 - 0.018)	2.005 (0.759 - 4.224)	1.978 (0.762 - 4.181)	1.993 (0.736 - 4.190)
**All ages (rate per capita)**	0.075 (0.058 - 0.099)	0.069 (0.053 - 0.092)	0.069 (0.053 - 0.092)	0.006 (0.005 - 0.008)	0.006 (0.005 - 0.008)	0.006 (0.005 - 0.008)	0.486 (0.182 - 1.039)	0.450 (0.165 - 0.979)	0.450 (0.166 - 0.966)
**Age-standardized (rate per capita)**	0.136 (0.106 - 0.177)	0.136 (0.105 - 0.175)	0.137 (0.106 - 0.177)	0.009 (0.007 - 0.012)	0.009 (0.007 - 0.012)	0.009 (0.007 - 0.012)	0.872 (0.325 - 1.885)	0.875 (0.328 - 1.898)	0.884 (0.332 - 1.933)
**Total number of cases (thousands)**	639 (493 - 840)	893 (684 - 1,190)	1,206 (924 - 1,617)	51 (40 - 66)	76 (58 - 102)	103 (80 - 138)	4 (2 - 9)	6 (2 - 13)	8 (3 - 17)
**Mauritania**									
**15 - 49 years (rate per capita)**	0.093 (0.068 - 0.133)	0.098 (0.071 - 0.141)	0.101 (0.073 - 0.146)	0.010 (0.008 - 0.015)	0.011 (0.008 - 0.015)	0.011 (0.008 - 0.015)	0.615 (0.220 - 1.364)	0.651 (0.228 - 1.436)	0.673 (0.236 - 1.497)
**50 - 69 years (rate per capita)**	0.318 (0.227 - 0.403)	0.317 (0.226 - 0.401)	0.317 (0.228 - 0.404)	0.016 (0.010 - 0.022)	0.016 (0.010 - 0.022)	0.016 (0.010 - 0.022)	2.038 (0.776 - 4.472)	2.039 (0.772 - 4.472)	2.044 (0.776 - 4.497)
**70+ years (rate per capita)**	0.314 (0.239 - 0.391)	0.313 (0.235 - 0.386)	0.311 (0.238 - 0.386)	0.011 (0.006 - 0.018)	0.011 (0.006 - 0.019)	0.011 (0.006 - 0.018)	1.924 (0.723 - 4.036)	1.923 (0.730 - 4.056)	1.918 (0.715 - 4.075)
**All ages (rate per capita)**	0.074 (0.057 - 0.098)	0.078 (0.060 - 0.105)	0.084 (0.063 - 0.112)	0.006 (0.005 - 0.008)	0.007 (0.005 - 0.009)	0.007 (0.005 - 0.009)	0.480 (0.180 - 1.031)	0.512 (0.189 - 1.109)	0.549 (0.203 - 1.207)
**Age-standardized (rate per capita)**	0.134 (0.103 - 0.173)	0.134 (0.103 - 0.172)	0.134 (0.103 - 0.172)	0.009 (0.007 - 0.012)	0.009 (0.007 - 0.012)	0.009 (0.007 - 0.012)	0.862 (0.324 - 1.855)	0.866 (0.325 - 1.886)	0.868 (0.330 - 1.861)
**Total number of cases (thousands)**	150 (115 - 198)	248 (190 - 332)	343 (259 - 456)	13 (10 - 16)	21 (16 - 28)	29 (22 - 37)	1 (0 - 2)	2 (1 - 4)	2 (1 - 5)
**Niger**									
**15 - 49 years (rate per capita)**	0.068 (0.057 - 0.080)	0.069 (0.058 - 0.081)	0.064 (0.055 - 0.076)	0.007 (0.006 - 0.009)	0.007 (0.006 - 0.009)	0.007 (0.006 - 0.008)	0.449 (0.169 - 0.946)	0.456 (0.173 - 0.954)	0.428 (0.163 - 0.902)
**50 - 69 years (rate per capita)**	0.219 (0.184 - 0.257)	0.219 (0.184 - 0.259)	0.221 (0.185 - 0.260)	0.012 (0.009 - 0.015)	0.012 (0.009 - 0.015)	0.012 (0.009 - 0.015)	1.412 (0.552 - 2.840)	1.413 (0.548 - 2.869)	1.430 (0.552 - 2.907)
**70+ years (rate per capita)**	0.225 (0.193 - 0.262)	0.223 (0.191 - 0.260)	0.223 (0.191 - 0.258)	0.008 (0.006 - 0.011)	0.008 (0.006 - 0.011)	0.008 (0.006 - 0.011)	1.392 (0.547 - 2.839)	1.378 (0.546 - 2.791)	1.382 (0.549 - 2.831)
**All ages (rate per capita)**	0.049 (0.042 - 0.056)	0.049 (0.043 - 0.057)	0.047 (0.040 - 0.054)	0.004 (0.004 - 0.005)	0.004 (0.004 - 0.005)	0.004 (0.003 - 0.004)	0.319 (0.124 - 0.659)	0.320 (0.124 - 0.663)	0.306 (0.118 - 0.633)
**Age-standardized (rate per capita)**	0.092 (0.081 - 0.106)	0.092 (0.081 - 0.105)	0.093 (0.081 - 0.106)	0.007 (0.006 - 0.007)	0.007 (0.006 - 0.007)	0.007 (0.006 - 0.007)	0.596 (0.231 - 1.223)	0.595 (0.234 - 1.232)	0.600 (0.234 - 1.244)
**Total number of cases (thousands)**	387 (336 - 446)	659 (573 - 760)	928 (804 - 1,071)	33 (29 - 38)	55 (48 - 63)	78 (68 - 89)	3 (1 - 5)	4 (2 - 9)	6 (2 - 13)
**Nigeria**									
**15 - 49 years (rate per capita)**	0.050 (0.042 - 0.059)	0.052 (0.043 - 0.061)	0.053 (0.044 - 0.062)	0.005 (0.005 - 0.006)	0.006 (0.005 - 0.007)	0.006 (0.005 - 0.007)	0.329 (0.126 - 0.690)	0.340 (0.129 - 0.718)	0.348 (0.133 - 0.727)
**50 - 69 years (rate per capita)**	0.169 (0.142 - 0.200)	0.179 (0.150 - 0.212)	0.178 (0.150 - 0.211)	0.010 (0.008 - 0.012)	0.010 (0.008 - 0.012)	0.010 (0.008 - 0.012)	1.085 (0.427 - 2.189)	1.149 (0.445 - 2.326)	1.148 (0.448 - 2.326)
**70+ years (rate per capita)**	0.183 (0.157 - 0.214)	0.190 (0.163 - 0.221)	0.190 (0.162 - 0.222)	0.007 (0.005 - 0.010)	0.007 (0.005 - 0.010)	0.008 (0.005 - 0.010)	1.129 (0.444 - 2.303)	1.169 (0.454 - 2.373)	1.173 (0.457 - 2.391)
**All ages (rate per capita)**	0.040 (0.035 - 0.046)	0.042 (0.036 - 0.048)	0.042 (0.036 - 0.048)	0.003 (0.003 - 0.004)	0.004 (0.003 - 0.004)	0.004 (0.003 - 0.004)	0.259 (0.100 - 0.532)	0.272 (0.105 - 0.564)	0.272 (0.105 - 0.562)
**Age-standardized (rate per capita)**	0.071 (0.062 - 0.081)	0.075 (0.065 - 0.086)	0.074 (0.065 - 0.086)	0.005 (0.004 - 0.006)	0.005 (0.005 - 0.006)	0.005 (0.005 - 0.006)	0.455 (0.176 - 0.931)	0.480 (0.187 - 0.996)	0.481 (0.188 - 0.993)
**Total number of cases (thousands)**	3,818 (3,324 - 4,402)	5,825 (5,049 - 6,731)	7,601 (6,633 - 8,793)	325 (282 - 374)	500 (435 - 578)	653 (569 - 755)	25 (10 - 51)	38 (15 - 79)	50 (19 - 102)
**Sao Tome and Principe**									
**15 - 49 years (rate per capita)**	0.083 (0.060 - 0.119)	0.091 (0.065 - 0.131)	0.099 (0.071 - 0.143)	0.010 (0.007 - 0.014)	0.010 (0.007 - 0.015)	0.011 (0.008 - 0.015)	0.556 (0.199 - 1.239)	0.604 (0.215 - 1.335)	0.658 (0.232 - 1.456)
**50 - 69 years (rate per capita)**	0.324 (0.229 - 0.415)	0.324 (0.233 - 0.411)	0.319 (0.226 - 0.407)	0.016 (0.010 - 0.022)	0.016 (0.009 - 0.022)	0.016 (0.010 - 0.023)	2.093 (0.799 - 4.635)	2.090 (0.783 - 4.582)	2.067 (0.794 - 4.525)
**70+ years (rate per capita)**	0.310 (0.236 - 0.386)	0.312 (0.234 - 0.387)	0.304 (0.228 - 0.377)	0.011 (0.006 - 0.017)	0.011 (0.006 - 0.018)	0.011 (0.006 - 0.016)	1.924 (0.731 - 4.031)	1.934 (0.740 - 4.037)	1.878 (0.709 - 3.920)
**All ages (rate per capita)**	0.073 (0.057 - 0.094)	0.072 (0.055 - 0.096)	0.077 (0.058 - 0.103)	0.006 (0.004 - 0.007)	0.006 (0.005 - 0.008)	0.007 (0.005 - 0.009)	0.479 (0.182 - 1.026)	0.470 (0.173 - 1.011)	0.502 (0.186 - 1.087)
**Age-standardized (rate per capita)**	0.135 (0.104 - 0.175)	0.135 (0.104 - 0.178)	0.136 (0.105 - 0.176)	0.009 (0.007 - 0.012)	0.009 (0.007 - 0.012)	0.009 (0.007 - 0.012)	0.879 (0.332 - 1.923)	0.880 (0.326 - 1.925)	0.882 (0.334 - 1.941)
**Total number of cases (thousands)**	8 (6 - 11)	11 (8 - 15)	15 (11 - 20)	1 (1 - 1)	1 (1 - 1)	1 (1 - 2)	0 (0 - 0)	0 (0 - 0)	0 (0 - 0)
**Senegal**									
**15 - 49 years (rate per capita)**	0.093 (0.068 - 0.134)	0.091 (0.066 - 0.130)	0.096 (0.070 - 0.137)	0.010 (0.008 - 0.015)	0.010 (0.007 - 0.015)	0.011 (0.008 - 0.015)	0.617 (0.220 - 1.360)	0.603 (0.213 - 1.322)	0.638 (0.227 - 1.388)
**50 - 69 years (rate per capita)**	0.318 (0.226 - 0.408)	0.319 (0.224 - 0.405)	0.318 (0.228 - 0.405)	0.016 (0.010 - 0.022)	0.016 (0.010 - 0.022)	0.016 (0.010 - 0.022)	2.065 (0.788 - 4.562)	2.068 (0.786 - 4.539)	2.062 (0.784 - 4.567)

	Prevalence 1990	Prevalence 2005	Prevalence 2015	Incidence 1990	Incidence 2005	Incidence 2015	YLD rate 1990	YLD rate 2005	YLD rate 2015
**70+ years (rate per capita)**	0.315 (0.242 - 0.392)	0.314 (0.237 - 0.390)	0.312 (0.237 - 0.386)	0.011 (0.006 - 0.019)	0.011 (0.006 - 0.019)	0.011 (0.006 - 0.018)	1.971 (0.741 - 4.217)	1.963 (0.736 - 4.128)	1.945 (0.758 - 4.105)
**All ages (rate per capita)**	0.072 (0.055 - 0.095)	0.072 (0.055 - 0.095)	0.074 (0.057 - 0.100)	0.006 (0.005 - 0.008)	0.006 (0.005 - 0.008)	0.006 (0.005 - 0.009)	0.471 (0.177 - 1.021)	0.471 (0.176 - 1.023)	0.487 (0.180 - 1.064)
**Age-standardized (rate per capita)**	0.134 (0.103 - 0.173)	0.134 (0.103 - 0.175)	0.134 (0.104 - 0.175)	0.009 (0.007 - 0.012)	0.009 (0.007 - 0.012)	0.009 (0.007 - 0.012)	0.872 (0.328 - 1.888)	0.872 (0.326 - 1.883)	0.873 (0.326 - 1.886)
**Total number of cases (thousands)**	540 (413 - 717)	810 (618 - 1,073)	1,119 (857 - 1,510)	45 (35 - 59)	70 (54 - 92)	97 (74 - 130)	4 (1 - 8)	5 (2 - 12)	7 (3 - 16)
**Sierra Leone**									
**15 - 49 years (rate per capita)**	0.102 (0.073 - 0.148)	0.095 (0.069 - 0.136)	0.098 (0.070 - 0.141)	0.011 (0.008 - 0.015)	0.010 (0.008 - 0.015)	0.011 (0.008 - 0.015)	0.671 (0.235 - 1.452)	0.625 (0.222 - 1.372)	0.647 (0.228 - 1.418)
**50 - 69 years (rate per capita)**	0.322 (0.231 - 0.411)	0.323 (0.228 - 0.409)	0.324 (0.234 - 0.410)	0.016 (0.010 - 0.022)	0.016 (0.010 - 0.022)	0.016 (0.010 - 0.022)	2.062 (0.785 - 4.445)	2.061 (0.795 - 4.405)	2.083 (0.789 - 4.489)
**70+ years (rate per capita)**	0.326 (0.247 - 0.403)	0.327 (0.248 - 0.406)	0.326 (0.252 - 0.404)	0.011 (0.006 - 0.020)	0.011 (0.006 - 0.019)	0.011 (0.006 - 0.019)	2.003 (0.758 - 4.242)	2.003 (0.763 - 4.196)	2.008 (0.770 - 4.249)
**All ages (rate per capita)**	0.082 (0.062 - 0.109)	0.076 (0.058 - 0.101)	0.077 (0.058 - 0.104)	0.007 (0.005 - 0.009)	0.006 (0.005 - 0.008)	0.007 (0.005 - 0.009)	0.531 (0.196 - 1.150)	0.492 (0.184 - 1.059)	0.507 (0.187 - 1.109)
**Age-standardized (rate per capita)**	0.135 (0.105 - 0.174)	0.136 (0.105 - 0.177)	0.136 (0.105 - 0.176)	0.009 (0.007 - 0.012)	0.009 (0.007 - 0.012)	0.009 (0.007 - 0.012)	0.871 (0.327 - 1.892)	0.870 (0.330 - 1.890)	0.880 (0.327 - 1.913)
**Total number of cases (thousands)**	320 (242 - 427)	382 (292 - 509)	500 (377 - 672)	26 (20 - 34)	32 (25 - 43)	43 (33 - 56)	2 (1 - 5)	2 (1 - 5)	3 (1 - 7)
**Togo**									
**15 - 49 years (rate per capita)**	0.095 (0.070 - 0.138)	0.093 (0.067 - 0.134)	0.099 (0.072 - 0.144)	0.010 (0.008 - 0.015)	0.010 (0.008 - 0.015)	0.011 (0.008 - 0.015)	0.632 (0.224 - 1.376)	0.615 (0.218 - 1.350)	0.658 (0.232 - 1.456)
**50 - 69 years (rate per capita)**	0.321 (0.229 - 0.412)	0.322 (0.227 - 0.413)	0.322 (0.233 - 0.409)	0.016 (0.010 - 0.022)	0.016 (0.010 - 0.022)	0.016 (0.010 - 0.022)	2.069 (0.786 - 4.551)	2.076 (0.785 - 4.533)	2.085 (0.807 - 4.576)
**70+ years (rate per capita)**	0.319 (0.245 - 0.398)	0.320 (0.241 - 0.399)	0.321 (0.245 - 0.399)	0.011 (0.006 - 0.018)	0.011 (0.006 - 0.019)	0.011 (0.006 - 0.019)	1.976 (0.746 - 4.171)	1.980 (0.754 - 4.165)	1.992 (0.747 - 4.173)
**All ages (rate per capita)**	0.073 (0.056 - 0.098)	0.074 (0.056 - 0.099)	0.078 (0.060 - 0.105)	0.006 (0.005 - 0.008)	0.006 (0.005 - 0.008)	0.007 (0.005 - 0.009)	0.478 (0.178 - 1.029)	0.483 (0.179 - 1.051)	0.513 (0.190 - 1.121)
**Age-standardized (rate per capita)**	0.135 (0.104 - 0.175)	0.135 (0.104 - 0.178)	0.136 (0.106 - 0.177)	0.009 (0.007 - 0.012)	0.009 (0.007 - 0.012)	0.009 (0.007 - 0.012)	0.876 (0.330 - 1.911)	0.876 (0.324 - 1.893)	0.882 (0.328 - 1.914)
**Total number of cases (thousands)**	277 (211 - 371)	412 (313 - 555)	570 (436 - 765)	23 (18 - 30)	35 (27 - 47)	49 (38 - 65)	2 (1 - 4)	3 (1 - 6)	4 (1 - 8)
**Eastern Sub-Saharan Africa**									
**15 - 49 years (rate per capita)**	0.063 (0.044 - 0.097)	0.060 (0.042 - 0.091)	0.061 (0.043 - 0.094)	0.007 (0.005 - 0.011)	0.007 (0.005 - 0.010)	0.007 (0.005 - 0.011)	0.418 (0.142 - 0.969)	0.396 (0.136 - 0.925)	0.408 (0.142 - 0.943)
**50 - 69 years (rate per capita)**	0.240 (0.164 - 0.326)	0.238 (0.163 - 0.322)	0.239 (0.164 - 0.322)	0.013 (0.007 - 0.018)	0.013 (0.007 - 0.018)	0.013 (0.007 - 0.018)	1.550 (0.558 - 3.493)	1.537 (0.560 - 3.453)	1.544 (0.556 - 3.481)
**70+ years (rate per capita)**	0.261 (0.178 - 0.334)	0.258 (0.177 - 0.328)	0.255 (0.170 - 0.326)	0.010 (0.005 - 0.016)	0.010 (0.005 - 0.016)	0.010 (0.005 - 0.016)	1.625 (0.591 - 3.590)	1.605 (0.593 - 3.490)	1.587 (0.584 - 3.465)
**All ages (rate per capita)**	0.050 (0.036 - 0.070)	0.049 (0.036 - 0.068)	0.051 (0.037 - 0.071)	0.004 (0.003 - 0.006)	0.004 (0.003 - 0.006)	0.004 (0.003 - 0.006)	0.328 (0.117 - 0.733)	0.321 (0.115 - 0.711)	0.336 (0.121 - 0.741)
**Age-standardized (rate per capita)**	0.098 (0.072 - 0.133)	0.097 (0.072 - 0.130)	0.097 (0.072 - 0.132)	0.007 (0.005 - 0.009)	0.007 (0.005 - 0.009)	0.007 (0.005 - 0.009)	0.637 (0.230 - 1.378)	0.629 (0.226 - 1.355)	0.631 (0.229 - 1.355)
**Total number of cases (thousands)**	9,349 (6,785 - 13,090)	13,881 (10,145 - 19,357)	19,338 (14,001 - 26,888)	795 (582 - 1,099)	1,198 (892 - 1,659)	1,668 (1,233 - 2,343)	61 (22 - 136)	91 (33 - 201)	127 (46 - 279)
**Burundi**									
**15 - 49 years (rate per capita)**	0.066 (0.039 - 0.111)	0.061 (0.037 - 0.103)	0.063 (0.038 - 0.106)	0.008 (0.004 - 0.013)	0.007 (0.004 - 0.012)	0.007 (0.004 - 0.013)	0.435 (0.139 - 1.048)	0.406 (0.130 - 0.988)	0.421 (0.136 - 1.032)
**50 - 69 years (rate per capita)**	0.265 (0.160 - 0.381)	0.261 (0.158 - 0.372)	0.264 (0.157 - 0.378)	0.015 (0.007 - 0.021)	0.015 (0.007 - 0.022)	0.015 (0.007 - 0.022)	1.710 (0.594 - 3.909)	1.688 (0.582 - 3.858)	1.721 (0.592 - 3.911)
**70+ years (rate per capita)**	0.289 (0.164 - 0.385)	0.287 (0.172 - 0.383)	0.288 (0.170 - 0.383)	0.011 (0.005 - 0.020)	0.011 (0.005 - 0.020)	0.011 (0.005 - 0.020)	1.798 (0.620 - 4.018)	1.792 (0.626 - 3.948)	1.810 (0.647 - 3.996)
**All ages (rate per capita)**	0.053 (0.034 - 0.080)	0.051 (0.033 - 0.077)	0.053 (0.034 - 0.081)	0.005 (0.003 - 0.007)	0.004 (0.003 - 0.007)	0.005 (0.003 - 0.007)	0.343 (0.118 - 0.779)	0.333 (0.114 - 0.773)	0.351 (0.120 - 0.808)
**Age-standardized (rate per capita)**	0.106 (0.070 - 0.154)	0.105 (0.070 - 0.152)	0.106 (0.069 - 0.155)	0.008 (0.005 - 0.011)	0.008 (0.005 - 0.011)	0.008 (0.005 - 0.011)	0.686 (0.239 - 1.563)	0.683 (0.238 - 1.561)	0.693 (0.241 - 1.567)
**Total number of cases (thousands)**	296 (189 - 453)	406 (261 - 613)	600 (385 - 914)	26 (17 - 40)	36 (24 - 53)	53 (35 - 81)	2 (1 - 4)	3 (1 - 6)	4 (1 - 9)
**Comoros**									
**15 - 49 years (rate per capita)**	0.063 (0.037 - 0.109)	0.065 (0.039 - 0.111)	0.068 (0.041 - 0.118)	0.007 (0.004 - 0.013)	0.008 (0.004 - 0.013)	0.008 (0.005 - 0.013)	0.422 (0.133 - 1.045)	0.432 (0.139 - 1.064)	0.452 (0.144 - 1.114)
**50 - 69 years (rate per capita)**	0.262 (0.158 - 0.379)	0.262 (0.161 - 0.375)	0.262 (0.161 - 0.379)	0.015 (0.007 - 0.022)	0.015 (0.007 - 0.022)	0.015 (0.007 - 0.022)	1.693 (0.585 - 3.858)	1.697 (0.579 - 3.832)	1.700 (0.590 - 3.857)
**70+ years (rate per capita)**	0.285 (0.173 - 0.385)	0.285 (0.171 - 0.381)	0.286 (0.173 - 0.380)	0.011 (0.005 - 0.021)	0.011 (0.005 - 0.020)	0.011 (0.005 - 0.020)	1.768 (0.626 - 3.900)	1.773 (0.625 - 3.922)	1.780 (0.632 - 3.931)
**All ages (rate per capita)**	0.053 (0.035 - 0.082)	0.056 (0.036 - 0.085)	0.060 (0.039 - 0.094)	0.005 (0.003 - 0.007)	0.005 (0.003 - 0.008)	0.005 (0.004 - 0.008)	0.349 (0.120 - 0.792)	0.365 (0.124 - 0.851)	0.397 (0.134 - 0.921)
**Age-standardized (rate per capita)**	0.105 (0.070 - 0.153)	0.105 (0.072 - 0.153)	0.106 (0.071 - 0.156)	0.008 (0.005 - 0.011)	0.008 (0.005 - 0.011)	0.008 (0.005 - 0.011)	0.682 (0.235 - 1.549)	0.685 (0.235 - 1.548)	0.689 (0.237 - 1.544)
**Total number of cases (thousands)**	22 (14 - 34)	35 (23 - 53)	48 (31 - 74)	2 (1 - 3)	3 (2 - 5)	4 (3 - 6)	0 (0 - 0)	0 (0 - 1)	0 (0 - 1)
**Djibouti**									
**15 - 49 years (rate per capita)**	0.065 (0.038 - 0.112)	0.067 (0.040 - 0.115)	0.072 (0.043 - 0.125)	0.008 (0.004 - 0.013)	0.008 (0.005 - 0.013)	0.008 (0.005 - 0.014)	0.432 (0.136 - 1.069)	0.446 (0.142 - 1.096)	0.477 (0.152 - 1.184)
**50 - 69 years (rate per capita)**	0.263 (0.160 - 0.381)	0.265 (0.164 - 0.379)	0.264 (0.163 - 0.380)	0.015 (0.007 - 0.022)	0.015 (0.007 - 0.022)	0.015 (0.007 - 0.021)	1.706 (0.587 - 3.882)	1.716 (0.584 - 3.869)	1.709 (0.588 - 3.885)
**70+ years (rate per capita)**	0.289 (0.175 - 0.389)	0.289 (0.173 - 0.385)	0.289 (0.174 - 0.388)	0.011 (0.005 - 0.021)	0.011 (0.005 - 0.020)	0.011 (0.005 - 0.020)	1.801 (0.638 - 4.003)	1.798 (0.632 - 3.927)	1.793 (0.632 - 3.968)
**All ages (rate per capita)**	0.055 (0.036 - 0.086)	0.063 (0.042 - 0.097)	0.074 (0.047 - 0.114)	0.005 (0.003 - 0.007)	0.005 (0.004 - 0.008)	0.006 (0.004 - 0.009)	0.363 (0.125 - 0.827)	0.413 (0.140 - 0.962)	0.484 (0.164 - 1.115)
**Age-standardized (rate per capita)**	0.106 (0.071 - 0.154)	0.106 (0.072 - 0.154)	0.107 (0.071 - 0.159)	0.008 (0.005 - 0.011)	0.008 (0.005 - 0.011)	0.008 (0.005 - 0.011)	0.688 (0.237 - 1.563)	0.689 (0.237 - 1.558)	0.691 (0.238 - 1.586)
**Total number of cases (thousands)**	32 (21 - 49)	49 (32 - 76)	66 (42 - 101)	3 (2 - 4)	4 (3 - 6)	6 (4 - 8)	0 (0 - 0)	0 (0 - 1)	0 (0 - 1)
**Eritrea**									
**15 - 49 years (rate per capita)**	0.066 (0.039 - 0.111)	0.057 (0.034 - 0.098)	0.064 (0.038 - 0.109)	0.008 (0.004 - 0.013)	0.007 (0.004 - 0.012)	0.008 (0.004 - 0.013)	0.438 (0.140 - 1.053)	0.377 (0.120 - 0.926)	0.424 (0.136 - 1.035)
**50 - 69 years (rate per capita)**	0.261 (0.157 - 0.379)	0.263 (0.161 - 0.381)	0.265 (0.159 - 0.378)	0.015 (0.007 - 0.022)	0.015 (0.007 - 0.022)	0.015 (0.007 - 0.021)	1.685 (0.582 - 3.892)	1.693 (0.578 - 3.841)	1.703 (0.581 - 3.868)
**70+ years (rate per capita)**	0.288 (0.165 - 0.387)	0.287 (0.175 - 0.385)	0.289 (0.171 - 0.384)	0.011 (0.005 - 0.021)	0.011 (0.005 - 0.021)	0.011 (0.005 - 0.021)	1.792 (0.614 - 3.967)	1.780 (0.620 - 3.946)	1.794 (0.640 - 3.952)
**All ages (rate per capita)**	0.050 (0.031 - 0.077)	0.051 (0.032 - 0.079)	0.054 (0.035 - 0.083)	0.005 (0.003 - 0.007)	0.005 (0.003 - 0.007)	0.005 (0.003 - 0.008)	0.325 (0.110 - 0.755)	0.330 (0.112 - 0.760)	0.353 (0.121 - 0.804)
**Age-standardized (rate per capita)**	0.106 (0.070 - 0.154)	0.105 (0.070 - 0.153)	0.106 (0.069 - 0.155)	0.008 (0.005 - 0.011)	0.008 (0.005 - 0.011)	0.008 (0.005 - 0.011)	0.685 (0.239 - 1.551)	0.679 (0.235 - 1.531)	0.681 (0.236 - 1.534)
**Total number of cases (thousands)**	156 (98 - 243)	213 (135 - 331)	284 (181 - 438)	14 (9 - 22)	19 (13 - 29)	26 (17 - 40)	1 (0 - 2)	1 (0 - 3)	2 (1 - 4)
**Ethiopia**									
**15 - 49 years (rate per capita)**	0.068 (0.040 - 0.114)	0.063 (0.037 - 0.108)	0.063 (0.038 - 0.106)	0.008 (0.004 - 0.013)	0.007 (0.004 - 0.013)	0.007 (0.004 - 0.013)	0.450 (0.145 - 1.075)	0.419 (0.133 - 1.037)	0.420 (0.134 - 1.019)
**50 - 69 years (rate per capita)**	0.267 (0.162 - 0.384)	0.266 (0.163 - 0.386)	0.267 (0.160 - 0.380)	0.015 (0.007 - 0.021)	0.015 (0.007 - 0.022)	0.015 (0.007 - 0.021)	1.728 (0.597 - 4.003)	1.727 (0.585 - 3.915)	1.731 (0.595 - 3.904)
**70+ years (rate per capita)**	0.296 (0.170 - 0.394)	0.292 (0.178 - 0.388)	0.293 (0.173 - 0.387)	0.011 (0.005 - 0.020)	0.011 (0.005 - 0.020)	0.011 (0.005 - 0.020)	1.843 (0.650 - 4.115)	1.821 (0.644 - 4.010)	1.828 (0.652 - 3.996)
**All ages (rate per capita)**	0.057 (0.037 - 0.087)	0.054 (0.035 - 0.083)	0.058 (0.038 - 0.088)	0.005 (0.003 - 0.007)	0.005 (0.003 - 0.007)	0.005 (0.003 - 0.007)	0.374 (0.128 - 0.850)	0.355 (0.120 - 0.814)	0.380 (0.131 - 0.868)
**Age-standardized (rate per capita)**	0.107 (0.071 - 0.156)	0.106 (0.071 - 0.154)	0.107 (0.070 - 0.156)	0.008 (0.005 - 0.011)	0.008 (0.005 - 0.011)	0.008 (0.005 - 0.011)	0.694 (0.241 - 1.572)	0.691 (0.239 - 1.574)	0.694 (0.240 - 1.559)
**Total number of cases (thousands)**	2,742 (1,765 - 4,176)	4,144 (2,681 - 6,337)	5,760 (3,729 - 8,756)	230 (151 - 344)	353 (238 - 528)	494 (329 - 745)	18 (6 - 41)	27 (9 - 62)	38 (13 - 86)
**Kenya**									
**15 - 49 years (rate per capita)**	0.109 (0.093 - 0.128)	0.101 (0.085 - 0.119)	0.109 (0.092 - 0.128)	0.012 (0.010 - 0.014)	0.011 (0.009 - 0.013)	0.011 (0.010 - 0.013)	0.727 (0.277 - 1.521)	0.669 (0.254 - 1.393)	0.723 (0.278 - 1.517)
**50 - 69 years (rate per capita)**	0.351 (0.301 - 0.405)	0.336 (0.288 - 0.391)	0.338 (0.289 - 0.393)	0.015 (0.011 - 0.018)	0.015 (0.011 - 0.019)	0.015 (0.011 - 0.019)	2.284 (0.895 - 4.619)	2.185 (0.853 - 4.427)	2.201 (0.856 - 4.474)
**70+ years (rate per capita)**	0.330 (0.287 - 0.377)	0.328 (0.284 - 0.376)	0.323 (0.280 - 0.368)	0.008 (0.006 - 0.011)	0.008 (0.006 - 0.011)	0.008 (0.006 - 0.011)	2.079 (0.829 - 4.237)	2.066 (0.828 - 4.199)	2.031 (0.810 - 4.131)
**All ages (rate per capita)**	0.075 (0.066 - 0.086)	0.077 (0.068 - 0.089)	0.084 (0.074 - 0.096)	0.006 (0.005 - 0.007)	0.006 (0.006 - 0.007)	0.007 (0.006 - 0.008)	0.495 (0.193 - 1.021)	0.508 (0.198 - 1.061)	0.553 (0.217 - 1.147)
**Age-standardized (rate per capita)**	0.153 (0.135 - 0.174)	0.145 (0.128 - 0.165)	0.145 (0.128 - 0.166)	0.010 (0.008 - 0.011)	0.009 (0.008 - 0.010)	0.009 (0.008 - 0.011)	1.003 (0.391 - 2.097)	0.946 (0.369 - 1.973)	0.949 (0.369 - 1.978)
**Total number of cases (thousands)**	1,763 (1,549 - 2,006)	2,738 (2,397 - 3,138)	3,878 (3,399 - 4,438)	143 (125 - 163)	228 (200 - 261)	315 (275 - 361)	12 (5 - 24)	18 (7 - 38)	26 (10 - 53)
**Madagascar**									
**15 - 49 years (rate per capita)**	0.034 (0.029 - 0.040)	0.037 (0.031 - 0.044)	0.037 (0.031 - 0.044)	0.004 (0.003 - 0.005)	0.004 (0.003 - 0.005)	0.004 (0.003 - 0.005)	0.224 (0.085 - 0.471)	0.244 (0.094 - 0.510)	0.243 (0.093 - 0.506)
**50 - 69 years (rate per capita)**	0.126 (0.105 - 0.149)	0.125 (0.104 - 0.149)	0.124 (0.104 - 0.149)	0.008 (0.006 - 0.010)	0.008 (0.006 - 0.009)	0.008 (0.006 - 0.009)	0.808 (0.319 - 1.646)	0.806 (0.315 - 1.630)	0.806 (0.312 - 1.637)
**70+ years (rate per capita)**	0.138 (0.118 - 0.162)	0.139 (0.119 - 0.164)	0.136 (0.116 - 0.160)	0.006 (0.004 - 0.008)	0.006 (0.004 - 0.008)	0.006 (0.004 - 0.008)	0.855 (0.337 - 1.734)	0.862 (0.343 - 1.753)	0.848 (0.337 - 1.706)
**All ages (rate per capita)**	0.028 (0.024 - 0.032)	0.029 (0.025 - 0.033)	0.030 (0.026 - 0.035)	0.002 (0.002 - 0.003)	0.003 (0.002 - 0.003)	0.003 (0.002 - 0.003)	0.181 (0.071 - 0.374)	0.187 (0.073 - 0.382)	0.199 (0.077 - 0.411)
**Age-standardized (rate per capita)**	0.052 (0.046 - 0.060)	0.053 (0.046 - 0.061)	0.053 (0.046 - 0.061)	0.004 (0.003 - 0.005)	0.004 (0.003 - 0.005)	0.004 (0.003 - 0.005)	0.338 (0.133 - 0.692)	0.343 (0.135 - 0.709)	0.342 (0.134 - 0.709)
**Total number of cases (thousands)**	322 (282 - 371)	525 (458 - 608)	735 (637 - 857)	28 (25 - 33)	47 (41 - 54)	65 (56 - 75)	2 (1 - 4)	3 (1 - 7)	5 (2 - 10)
**Malawi**									

	Prevalence 1990	Prevalence 2005	Prevalence 2015	Incidence 1990	Incidence 2005	Incidence 2015	YLD rate 1990	YLD rate 2005	YLD rate 2015
15 - 49 years (rate per capita)	0.066 (0.039 - 0.114)	0.058 (0.034 - 0.098)	0.059 (0.035 - 0.101)	0.008 (0.004 - 0.013)	0.007 (0.004 - 0.012)	0.007 (0.004 - 0.012)	0.434 (0.139 - 1.054)	0.381 (0.121 - 0.949)	0.395 (0.127 - 0.964)
50 - 69 years (rate per capita)	0.261 (0.162 - 0.377)	0.263 (0.163 - 0.376)	0.263 (0.163 - 0.375)	0.015 (0.007 - 0.022)	0.015 (0.007 - 0.021)	0.015 (0.007 - 0.021)	1.682 (0.589 - 3.784)	1.692 (0.592 - 3.844)	1.703 (0.593 - 3.866)
70+ years (rate per capita)	0.291 (0.176 - 0.391)	0.287 (0.172 - 0.383)	0.286 (0.172 - 0.387)	0.011 (0.005 - 0.021)	0.011 (0.005 - 0.020)	0.011 (0.005 - 0.020)	1.807 (0.638 - 4.037)	1.778 (0.636 - 3.870)	1.790 (0.636 - 3.914)
All ages (rate per capita)	0.053 (0.034 - 0.082)	0.050 (0.033 - 0.075)	0.050 (0.032 - 0.076)	0.005 (0.003 - 0.007)	0.004 (0.003 - 0.007)	0.004 (0.003 - 0.007)	0.345 (0.118 - 0.780)	0.325 (0.110 - 0.732)	0.331 (0.113 - 0.767)
Age-standardized (rate per capita)	0.105 (0.072 - 0.153)	0.105 (0.071 - 0.153)	0.104 (0.069 - 0.151)	0.008 (0.005 - 0.011)	0.008 (0.005 - 0.011)	0.008 (0.005 - 0.011)	0.679 (0.233 - 1.544)	0.678 (0.232 - 1.518)	0.678 (0.234 - 1.547)
Total number of cases (thousands)	496 (321 - 773)	640 (423 - 963)	869 (559 - 1,305)	44 (29 - 67)	56 (38 - 85)	77 (52 - 119)	3 (1 - 7)	4 (1 - 9)	6 (2 - 13)
**Mozambique**									
15 - 49 years (rate per capita)	0.064 (0.037 - 0.111)	0.063 (0.038 - 0.107)	0.062 (0.037 - 0.108)	0.007 (0.004 - 0.012)	0.007 (0.004 - 0.012)	0.007 (0.004 - 0.012)	0.422 (0.134 - 1.045)	0.415 (0.133 - 1.029)	0.412 (0.133 - 1.014)
50 - 69 years (rate per capita)	0.262 (0.159 - 0.380)	0.262 (0.161 - 0.378)	0.264 (0.164 - 0.382)	0.015 (0.007 - 0.022)	0.015 (0.007 - 0.021)	0.015 (0.007 - 0.021)	1.679 (0.579 - 3.800)	1.674 (0.583 - 3.752)	1.689 (0.578 - 3.851)
70+ years (rate per capita)	0.288 (0.175 - 0.389)	0.289 (0.168 - 0.389)	0.288 (0.173 - 0.388)	0.011 (0.005 - 0.021)	0.011 (0.005 - 0.020)	0.011 (0.005 - 0.020)	1.770 (0.626 - 3.906)	1.766 (0.622 - 3.922)	1.762 (0.624 - 3.894)
All ages (rate per capita)	0.055 (0.036 - 0.083)	0.055 (0.036 - 0.083)	0.054 (0.035 - 0.083)	0.005 (0.003 - 0.007)	0.005 (0.003 - 0.007)	0.005 (0.003 - 0.007)	0.353 (0.121 - 0.802)	0.354 (0.123 - 0.789)	0.352 (0.121 - 0.812)
Age-standardized (rate per capita)	0.105 (0.070 - 0.153)	0.105 (0.071 - 0.152)	0.106 (0.071 - 0.158)	0.008 (0.005 - 0.011)	0.008 (0.005 - 0.011)	0.008 (0.005 - 0.011)	0.674 (0.233 - 1.526)	0.671 (0.233 - 1.518)	0.679 (0.234 - 1.545)
Total number of cases (thousands)	734 (479 - 1,116)	1,158 (763 - 1,758)	1,523 (985 - 2,314)	61 (41 - 90)	99 (67 - 146)	130 (88 - 196)	5 (2 - 11)	7 (3 - 17)	10 (3 - 23)
**Rwanda**									
15 - 49 years (rate per capita)	0.062 (0.037 - 0.108)	0.060 (0.036 - 0.103)	0.064 (0.039 - 0.112)	0.007 (0.004 - 0.013)	0.007 (0.004 - 0.012)	0.008 (0.004 - 0.013)	0.414 (0.130 - 1.017)	0.394 (0.126 - 0.972)	0.423 (0.134 - 1.048)
50 - 69 years (rate per capita)	0.265 (0.161 - 0.383)	0.258 (0.159 - 0.374)	0.260 (0.159 - 0.379)	0.015 (0.007 - 0.022)	0.015 (0.007 - 0.022)	0.015 (0.007 - 0.021)	1.721 (0.596 - 3.896)	1.670 (0.563 - 3.822)	1.678 (0.582 - 3.804)
70+ years (rate per capita)	0.285 (0.172 - 0.384)	0.283 (0.167 - 0.376)	0.283 (0.170 - 0.377)	0.011 (0.005 - 0.020)	0.011 (0.005 - 0.019)	0.011 (0.005 - 0.019)	1.788 (0.633 - 3.979)	1.764 (0.621 - 3.855)	1.771 (0.631 - 3.911)
All ages (rate per capita)	0.048 (0.031 - 0.074)	0.052 (0.034 - 0.080)	0.057 (0.037 - 0.089)	0.004 (0.003 - 0.006)	0.005 (0.003 - 0.007)	0.005 (0.003 - 0.008)	0.312 (0.108 - 0.711)	0.337 (0.115 - 0.780)	0.375 (0.127 - 0.872)
Age-standardized (rate per capita)	0.106 (0.071 - 0.154)	0.105 (0.071 - 0.153)	0.105 (0.070 - 0.155)	0.008 (0.005 - 0.011)	0.008 (0.005 - 0.011)	0.008 (0.005 - 0.011)	0.687 (0.236 - 1.560)	0.678 (0.233 - 1.546)	0.682 (0.235 - 1.531)
Total number of cases (thousands)	345 (223 - 535)	469 (308 - 722)	668 (434 - 1,035)	31 (21 - 47)	42 (29 - 64)	59 (39 - 91)	2 (1 - 5)	3 (1 - 7)	4 (1 - 10)
**Somalia**									
15 - 49 years (rate per capita)	0.067 (0.040 - 0.115)	0.065 (0.039 - 0.111)	0.062 (0.038 - 0.107)	0.008 (0.005 - 0.013)	0.008 (0.004 - 0.013)	0.007 (0.004 - 0.012)	0.444 (0.142 - 1.091)	0.433 (0.139 - 1.042)	0.413 (0.131 - 0.989)
50 - 69 years (rate per capita)	0.264 (0.163 - 0.379)	0.263 (0.164 - 0.377)	0.263 (0.163 - 0.379)	0.015 (0.007 - 0.022)	0.015 (0.007 - 0.021)	0.015 (0.007 - 0.022)	1.701 (0.597 - 3.809)	1.693 (0.591 - 3.828)	1.690 (0.584 - 3.892)
70+ years (rate per capita)	0.290 (0.175 - 0.390)	0.289 (0.175 - 0.386)	0.289 (0.174 - 0.387)	0.011 (0.005 - 0.021)	0.011 (0.005 - 0.020)	0.011 (0.005 - 0.020)	1.804 (0.640 - 4.007)	1.790 (0.643 - 3.930)	1.789 (0.636 - 3.929)
All ages (rate per capita)	0.057 (0.037 - 0.087)	0.053 (0.035 - 0.081)	0.052 (0.033 - 0.079)	0.005 (0.003 - 0.007)	0.005 (0.003 - 0.007)	0.004 (0.003 - 0.007)	0.371 (0.127 - 0.844)	0.344 (0.118 - 0.783)	0.336 (0.115 - 0.768)
Age-standardized (rate per capita)	0.106 (0.071 - 0.154)	0.106 (0.072 - 0.154)	0.105 (0.069 - 0.154)	0.008 (0.005 - 0.011)	0.008 (0.005 - 0.011)	0.008 (0.005 - 0.011)	0.686 (0.239 - 1.547)	0.682 (0.236 - 1.539)	0.680 (0.235 - 1.548)
Total number of cases (thousands)	365 (239 - 560)	450 (296 - 685)	560 (362 - 857)	31 (21 - 46)	39 (26 - 58)	48 (32 - 73)	2 (1 - 5)	3 (1 - 7)	4 (1 - 8)
**South Sudan**									
15 - 49 years (rate per capita)	0.068 (0.041 - 0.116)	0.066 (0.040 - 0.112)	0.065 (0.039 - 0.111)	0.008 (0.005 - 0.013)	0.008 (0.004 - 0.013)	0.007 (0.004 - 0.013)	0.446 (0.142 - 1.082)	0.434 (0.140 - 1.044)	0.430 (0.137 - 1.030)
50 - 69 years (rate per capita)	0.263 (0.162 - 0.379)	0.266 (0.167 - 0.379)	0.263 (0.163 - 0.379)	0.015 (0.007 - 0.022)	0.015 (0.007 - 0.021)	0.015 (0.007 - 0.021)	1.675 (0.586 - 3.775)	1.692 (0.593 - 3.824)	1.680 (0.579 - 3.852)
70+ years (rate per capita)	0.293 (0.177 - 0.394)	0.291 (0.177 - 0.389)	0.290 (0.175 - 0.389)	0.011 (0.005 - 0.021)	0.011 (0.005 - 0.021)	0.011 (0.005 - 0.021)	1.769 (0.627 - 3.897)	1.765 (0.636 - 3.840)	1.766 (0.627 - 3.859)
All ages (rate per capita)	0.057 (0.037 - 0.089)	0.057 (0.038 - 0.087)	0.059 (0.038 - 0.090)	0.005 (0.003 - 0.007)	0.005 (0.003 - 0.007)	0.005 (0.003 - 0.007)	0.372 (0.128 - 0.850)	0.370 (0.127 - 0.847)	0.381 (0.130 - 0.870)
Age-standardized (rate per capita)	0.106 (0.071 - 0.154)	0.106 (0.072 - 0.154)	0.106 (0.069 - 0.155)	0.008 (0.005 - 0.011)	0.008 (0.005 - 0.011)	0.008 (0.005 - 0.011)	0.678 (0.236 - 1.530)	0.677 (0.234 - 1.522)	0.676 (0.234 - 1.534)
Total number of cases (thousands)	335 (217 - 516)	464 (305 - 702)	722 (467 - 1,103)	29 (19 - 42)	40 (27 - 60)	62 (41 - 92)	2 (1 - 5)	3 (1 - 7)	5 (2 - 11)
**Tanzania**									
15 - 49 years (rate per capita)	0.023 (0.019 - 0.027)	0.023 (0.020 - 0.027)	0.024 (0.020 - 0.028)	0.003 (0.002 - 0.003)	0.003 (0.002 - 0.003)	0.003 (0.002 - 0.003)	0.150 (0.057 - 0.310)	0.152 (0.058 - 0.314)	0.157 (0.059 - 0.332)
50 - 69 years (rate per capita)	0.079 (0.066 - 0.094)	0.082 (0.068 - 0.097)	0.082 (0.068 - 0.098)	0.005 (0.004 - 0.006)	0.005 (0.004 - 0.007)	0.005 (0.004 - 0.007)	0.514 (0.200 - 1.042)	0.530 (0.204 - 1.081)	0.533 (0.203 - 1.095)
70+ years (rate per capita)	0.092 (0.079 - 0.109)	0.095 (0.081 - 0.111)	0.093 (0.080 - 0.110)	0.005 (0.004 - 0.006)	0.005 (0.004 - 0.006)	0.005 (0.004 - 0.006)	0.574 (0.225 - 1.173)	0.591 (0.236 - 1.199)	0.585 (0.230 - 1.171)
All ages (rate per capita)	0.018 (0.015 - 0.020)	0.018 (0.016 - 0.021)	0.019 (0.016 - 0.021)	0.002 (0.001 - 0.002)	0.002 (0.001 - 0.002)	0.002 (0.001 - 0.002)	0.115 (0.045 - 0.236)	0.119 (0.047 - 0.244)	0.122 (0.047 - 0.254)
Age-standardized (rate per capita)	0.034 (0.029 - 0.039)	0.035 (0.030 - 0.040)	0.035 (0.030 - 0.040)	0.003 (0.002 - 0.003)	0.003 (0.002 - 0.003)	0.003 (0.002 - 0.003)	0.219 (0.086 - 0.452)	0.226 (0.088 - 0.466)	0.226 (0.088 - 0.469)
Total number of cases (thousands)	448 (390 - 519)	713 (621 - 823)	991 (863 - 1,145)	41 (36 - 48)	65 (57 - 75)	90 (78 - 104)	3 (1 - 6)	5 (2 - 10)	7 (3 - 14)
**Uganda**									
15 - 49 years (rate per capita)	0.063 (0.038 - 0.109)	0.058 (0.034 - 0.099)	0.060 (0.037 - 0.104)	0.007 (0.004 - 0.013)	0.007 (0.004 - 0.012)	0.007 (0.004 - 0.012)	0.414 (0.132 - 1.014)	0.384 (0.122 - 0.955)	0.402 (0.128 - 0.986)
50 - 69 years (rate per capita)	0.264 (0.164 - 0.379)	0.262 (0.163 - 0.376)	0.264 (0.163 - 0.378)	0.015 (0.007 - 0.022)	0.015 (0.007 - 0.022)	0.015 (0.007 - 0.021)	1.705 (0.597 - 3.852)	1.693 (0.592 - 3.813)	1.706 (0.594 - 3.890)
70+ years (rate per capita)	0.290 (0.174 - 0.390)	0.289 (0.174 - 0.385)	0.286 (0.171 - 0.382)	0.011 (0.005 - 0.021)	0.011 (0.005 - 0.020)	0.011 (0.005 - 0.020)	1.807 (0.639 - 4.008)	1.799 (0.646 - 3.960)	1.788 (0.640 - 3.990)
All ages (rate per capita)	0.050 (0.033 - 0.078)	0.046 (0.030 - 0.069)	0.046 (0.030 - 0.071)	0.004 (0.003 - 0.007)	0.004 (0.003 - 0.006)	0.004 (0.003 - 0.007)	0.328 (0.112 - 0.742)	0.298 (0.101 - 0.675)	0.302 (0.104 - 0.698)
Age-standardized (rate per capita)	0.106 (0.072 - 0.154)	0.105 (0.071 - 0.154)	0.106 (0.070 - 0.155)	0.008 (0.005 - 0.011)	0.008 (0.005 - 0.011)	0.008 (0.005 - 0.011)	0.683 (0.235 - 1.550)	0.682 (0.234 - 1.523)	0.686 (0.238 - 1.548)
Total number of cases (thousands)	877 (573 - 1,357)	1,281 (846 - 1,944)	1,805 (1,171 - 2,783)	76 (51 - 116)	115 (77 - 176)	164 (110 - 257)	6 (2 - 13)	8 (3 - 19)	12 (4 - 27)
**Zambia**									
15 - 49 years (rate per capita)	0.062 (0.038 - 0.108)	0.058 (0.034 - 0.097)	0.063 (0.038 - 0.110)	0.007 (0.004 - 0.012)	0.007 (0.004 - 0.012)	0.007 (0.004 - 0.013)	0.414 (0.133 - 1.003)	0.382 (0.120 - 0.959)	0.417 (0.133 - 1.036)
50 - 69 years (rate per capita)	0.263 (0.163 - 0.379)	0.266 (0.164 - 0.379)	0.266 (0.164 - 0.382)	0.015 (0.007 - 0.022)	0.015 (0.007 - 0.021)	0.015 (0.006 - 0.021)	1.704 (0.593 - 3.846)	1.713 (0.591 - 3.860)	1.716 (0.600 - 3.827)
70+ years (rate per capita)	0.291 (0.174 - 0.389)	0.290 (0.177 - 0.387)	0.289 (0.174 - 0.384)	0.011 (0.005 - 0.020)	0.011 (0.005 - 0.020)	0.011 (0.005 - 0.020)	1.816 (0.643 - 4.066)	1.800 (0.647 - 3.985)	1.794 (0.638 - 3.962)
All ages (rate per capita)	0.051 (0.033 - 0.079)	0.049 (0.032 - 0.073)	0.050 (0.033 - 0.078)	0.004 (0.003 - 0.007)	0.004 (0.003 - 0.007)	0.005 (0.003 - 0.007)	0.333 (0.115 - 0.756)	0.317 (0.107 - 0.720)	0.328 (0.111 - 0.759)
Age-standardized (rate per capita)	0.106 (0.072 - 0.154)	0.106 (0.071 - 0.154)	0.107 (0.071 - 0.157)	0.008 (0.005 - 0.011)	0.008 (0.005 - 0.011)	0.008 (0.005 - 0.011)	0.687 (0.238 - 1.564)	0.685 (0.235 - 1.537)	0.689 (0.237 - 1.534)
Total number of cases (thousands)	414 (270 - 639)	587 (386 - 881)	815 (529 - 1,264)	36 (24 - 54)	52 (35 - 80)	73 (49 - 115)	3 (1 - 6)	4 (1 - 9)	5 (2 - 12)
**Central Sub-Saharan Africa**									
15 - 49 years (rate per capita)	0.058 (0.049 - 0.069)	0.057 (0.048 - 0.067)	0.056 (0.047 - 0.066)	0.007 (0.006 - 0.008)	0.007 (0.006 - 0.008)	0.007 (0.006 - 0.008)	0.381 (0.147 - 0.799)	0.372 (0.143 - 0.777)	0.370 (0.141 - 0.773)
50 - 69 years (rate per capita)	0.205 (0.172 - 0.242)	0.204 (0.172 - 0.241)	0.204 (0.172 - 0.240)	0.012 (0.009 - 0.015)	0.012 (0.009 - 0.015)	0.013 (0.009 - 0.015)	1.299 (0.511 - 2.633)	1.298 (0.514 - 2.634)	1.304 (0.511 - 2.630)
70+ years (rate per capita)	0.206 (0.178 - 0.239)	0.206 (0.177 - 0.238)	0.206 (0.177 - 0.239)	0.008 (0.006 - 0.011)	0.009 (0.006 - 0.011)	0.008 (0.006 - 0.011)	1.247 (0.490 - 2.548)	1.250 (0.496 - 2.533)	1.254 (0.499 - 2.519)
All ages (rate per capita)	0.046 (0.040 - 0.053)	0.044 (0.038 - 0.051)	0.044 (0.038 - 0.051)	0.004 (0.004 - 0.005)	0.004 (0.003 - 0.005)	0.004 (0.003 - 0.005)	0.295 (0.114 - 0.610)	0.284 (0.111 - 0.588)	0.286 (0.111 - 0.595)
Age-standardized (rate per capita)	0.084 (0.074 - 0.097)	0.084 (0.074 - 0.097)	0.084 (0.074 - 0.096)	0.006 (0.006 - 0.007)	0.006 (0.006 - 0.007)	0.006 (0.006 - 0.007)	0.538 (0.210 - 1.113)	0.537 (0.211 - 1.114)	0.539 (0.210 - 1.125)
Total number of cases (thousands)	2,434 (2,126 - 2,827)	3,695 (3,218 - 4,272)	5,064 (4,417 - 5,838)	219 (190 - 251)	336 (293 - 386)	462 (401 - 530)	16 (6 - 32)	24 (9 - 49)	33 (13 - 68)
**Angola**									
15 - 49 years (rate per capita)	0.056 (0.047 - 0.066)	0.056 (0.047 - 0.066)	0.055 (0.047 - 0.066)	0.007 (0.006 - 0.008)	0.007 (0.006 - 0.008)	0.006 (0.005 - 0.008)	0.368 (0.141 - 0.763)	0.366 (0.141 - 0.756)	0.364 (0.139 - 0.763)
50 - 69 years (rate per capita)	0.205 (0.173 - 0.242)	0.203 (0.171 - 0.241)	0.204 (0.171 - 0.241)	0.012 (0.009 - 0.015)	0.012 (0.009 - 0.015)	0.013 (0.009 - 0.015)	1.304 (0.515 - 2.642)	1.298 (0.509 - 2.626)	1.303 (0.510 - 2.631)
70+ years (rate per capita)	0.207 (0.179 - 0.242)	0.207 (0.178 - 0.241)	0.206 (0.176 - 0.240)	0.009 (0.006 - 0.011)	0.009 (0.006 - 0.011)	0.009 (0.006 - 0.011)	1.268 (0.505 - 2.585)	1.267 (0.499 - 2.587)	1.265 (0.508 - 2.592)
All ages (rate per capita)	0.042 (0.036 - 0.048)	0.041 (0.035 - 0.047)	0.041 (0.036 - 0.047)	0.004 (0.003 - 0.004)	0.004 (0.003 - 0.004)	0.004 (0.003 - 0.004)	0.271 (0.104 - 0.558)	0.264 (0.102 - 0.543)	0.265 (0.103 - 0.551)
Age-standardized (rate per capita)	0.084 (0.074 - 0.097)	0.084 (0.073 - 0.097)	0.084 (0.073 - 0.096)	0.006 (0.006 - 0.007)	0.006 (0.006 - 0.007)	0.006 (0.006 - 0.007)	0.539 (0.211 - 1.115)	0.538 (0.213 - 1.106)	0.540 (0.213 - 1.116)
Total number of cases (thousands)	472 (411 - 546)	734 (640 - 848)	1,030 (900 - 1,193)	43 (38 - 50)	68 (60 - 79)	96 (84 - 110)	3 (1 - 6)	5 (2 - 10)	7 (3 - 14)
**Central African Republic**									
15 - 49 years (rate per capita)	0.059 (0.050 - 0.070)	0.056 (0.048 - 0.067)	0.057 (0.048 - 0.067)	0.007 (0.006 - 0.008)	0.007 (0.006 - 0.008)	0.007 (0.006 - 0.008)	0.388 (0.149 - 0.806)	0.371 (0.142 - 0.778)	0.375 (0.142 - 0.788)
50 - 69 years (rate per capita)	0.206 (0.173 - 0.243)	0.205 (0.172 - 0.241)	0.205 (0.172 - 0.242)	0.012 (0.009 - 0.015)	0.012 (0.009 - 0.015)	0.012 (0.009 - 0.015)	1.308 (0.512 - 2.655)	1.302 (0.511 - 2.632)	1.306 (0.511 - 2.620)
70+ years (rate per capita)	0.205 (0.176 - 0.238)	0.205 (0.176 - 0.239)	0.205 (0.176 - 0.238)	0.008 (0.006 - 0.011)	0.009 (0.006 - 0.011)	0.008 (0.006 - 0.011)	1.246 (0.492 - 2.572)	1.247 (0.501 - 2.529)	1.247 (0.492 - 2.547)
All ages (rate per capita)	0.052 (0.045 - 0.059)	0.050 (0.043 - 0.057)	0.051 (0.045 - 0.059)	0.004 (0.004 - 0.005)	0.004 (0.004 - 0.005)	0.005 (0.004 - 0.005)	0.332 (0.129 - 0.685)	0.320 (0.124 - 0.668)	0.332 (0.129 - 0.693)

	Prevalence 1990	Prevalence 2005	Prevalence 2015	Incidence 1990	Incidence 2005	Incidence 2015	YLD rate 1990	YLD rate 2005	YLD rate 2015
**Age-standardized (rate per capita)**	0.084 (0.074 - 0.097)	0.084 (0.074 - 0.097)	0.084 (0.074 - 0.097)	0.006 (0.006 - 0.007)	0.006 (0.006 - 0.007)	0.006 (0.006 - 0.007)	0.539 (0.211 - 1.110)	0.537 (0.210 - 1.123)	0.539 (0.209 - 1.110)
**Total number of cases (thousands)**	151 (132 - 174)	202 (176 - 232)	252 (219 - 291)	13 (11 - 15)	18 (16 - 21)	23 (20 - 26)	1 (0 - 2)	1 (1 - 3)	2 (1 - 3)
**Congo**									
**15 - 49 years (rate per capita)**	0.057 (0.048 - 0.067)	0.057 (0.048 - 0.068)	0.060 (0.050 - 0.070)	0.007 (0.006 - 0.008)	0.007 (0.006 - 0.008)	0.007 (0.006 - 0.008)	0.374 (0.143 - 0.782)	0.376 (0.144 - 0.786)	0.394 (0.151 - 0.831)
**50 - 69 years (rate per capita)**	0.205 (0.173 - 0.241)	0.205 (0.172 - 0.242)	0.205 (0.172 - 0.240)	0.012 (0.009 - 0.015)	0.012 (0.009 - 0.015)	0.013 (0.009 - 0.016)	1.308 (0.508 - 2.641)	1.309 (0.511 - 2.658)	1.311 (0.512 - 2.669)
**70+ years (rate per capita)**	0.205 (0.175 - 0.238)	0.205 (0.177 - 0.238)	0.205 (0.175 - 0.238)	0.008 (0.006 - 0.011)	0.008 (0.006 - 0.011)	0.008 (0.006 - 0.011)	1.248 (0.497 - 2.520)	1.251 (0.499 - 2.515)	1.252 (0.503 - 2.543)
**All ages (rate per capita)**	0.048 (0.042 - 0.055)	0.049 (0.042 - 0.056)	0.050 (0.043 - 0.057)	0.004 (0.004 - 0.005)	0.004 (0.004 - 0.005)	0.004 (0.004 - 0.005)	0.311 (0.120 - 0.645)	0.314 (0.121 - 0.653)	0.322 (0.125 - 0.670)
**Age-standardized (rate per capita)**	0.084 (0.074 - 0.097)	0.084 (0.074 - 0.097)	0.084 (0.074 - 0.096)	0.006 (0.006 - 0.007)	0.006 (0.006 - 0.007)	0.006 (0.006 - 0.007)	0.541 (0.210 - 1.119)	0.540 (0.210 - 1.119)	0.541 (0.212 - 1.134)
**Total number of cases (thousands)**	115 (101 - 133)	171 (149 - 197)	230 (200 - 264)	10 (9 - 12)	15 (13 - 18)	20 (18 - 24)	1 (0 - 2)	1 (0 - 2)	1 (1 - 3)
**Democratic Republic of the Congo**									
**15 - 49 years (rate per capita)**	0.059 (0.049 - 0.070)	0.057 (0.048 - 0.068)	0.056 (0.047 - 0.066)	0.007 (0.006 - 0.008)	0.007 (0.006 - 0.008)	0.007 (0.005 - 0.008)	0.384 (0.148 - 0.794)	0.372 (0.143 - 0.781)	0.370 (0.141 - 0.769)
**50 - 69 years (rate per capita)**	0.205 (0.172 - 0.243)	0.204 (0.172 - 0.241)	0.204 (0.172 - 0.240)	0.012 (0.009 - 0.016)	0.012 (0.009 - 0.015)	0.013 (0.009 - 0.015)	1.296 (0.509 - 2.631)	1.297 (0.515 - 2.627)	1.304 (0.512 - 2.642)
**70+ years (rate per capita)**	0.206 (0.178 - 0.240)	0.206 (0.177 - 0.239)	0.206 (0.176 - 0.238)	0.008 (0.006 - 0.011)	0.009 (0.006 - 0.011)	0.008 (0.006 - 0.011)	1.242 (0.486 - 2.538)	1.246 (0.494 - 2.523)	1.254 (0.498 - 2.504)
**All ages (rate per capita)**	0.046 (0.040 - 0.054)	0.044 (0.038 - 0.051)	0.044 (0.038 - 0.050)	0.004 (0.004 - 0.005)	0.004 (0.003 - 0.005)	0.004 (0.003 - 0.005)	0.296 (0.114 - 0.613)	0.284 (0.111 - 0.588)	0.285 (0.111 - 0.593)
**Age-standardized (rate per capita)**	0.084 (0.074 - 0.097)	0.084 (0.073 - 0.097)	0.084 (0.073 - 0.096)	0.006 (0.006 - 0.007)	0.006 (0.006 - 0.007)	0.006 (0.006 - 0.007)	0.537 (0.209 - 1.110)	0.536 (0.211 - 1.114)	0.539 (0.210 - 1.127)
**Total number of cases (thousands)**	1,620 (1,412 - 1,886)	2,479 (2,162 - 2,882)	3,407 (2,962 - 3,909)	146 (127 - 168)	225 (196 - 259)	311 (269 - 357)	10 (4 - 22)	16 (6 - 33)	22 (9 - 46)
**Equatorial Guinea**									
**15 - 49 years (rate per capita)**	0.056 (0.047 - 0.066)	0.069 (0.057 - 0.082)	0.061 (0.051 - 0.073)	0.007 (0.006 - 0.008)	0.007 (0.006 - 0.009)	0.007 (0.006 - 0.008)	0.369 (0.143 - 0.764)	0.453 (0.171 - 0.946)	0.405 (0.154 - 0.864)
**50 - 69 years (rate per capita)**	0.207 (0.175 - 0.244)	0.203 (0.171 - 0.240)	0.202 (0.169 - 0.240)	0.012 (0.009 - 0.015)	0.013 (0.009 - 0.016)	0.013 (0.010 - 0.016)	1.321 (0.516 - 2.686)	1.300 (0.505 - 2.615)	1.299 (0.507 - 2.620)
**70+ years (rate per capita)**	0.205 (0.177 - 0.239)	0.204 (0.175 - 0.237)	0.202 (0.175 - 0.235)	0.008 (0.006 - 0.011)	0.008 (0.006 - 0.011)	0.008 (0.006 - 0.011)	1.252 (0.496 - 2.571)	1.243 (0.497 - 2.515)	1.235 (0.491 - 2.539)
**All ages (rate per capita)**	0.053 (0.046 - 0.060)	0.054 (0.047 - 0.063)	0.056 (0.049 - 0.065)	0.005 (0.004 - 0.005)	0.005 (0.004 - 0.006)	0.005 (0.004 - 0.006)	0.341 (0.133 - 0.703)	0.350 (0.135 - 0.721)	0.365 (0.140 - 0.761)
**Age-standardized (rate per capita)**	0.085 (0.074 - 0.097)	0.084 (0.074 - 0.097)	0.084 (0.074 - 0.097)	0.007 (0.006 - 0.007)	0.007 (0.006 - 0.007)	0.007 (0.006 - 0.007)	0.543 (0.211 - 1.114)	0.543 (0.212 - 1.131)	0.542 (0.211 - 1.139)
**Total number of cases (thousands)**	20 (17 - 23)	34 (29 - 39)	48 (41 - 55)	2 (2 - 2)	3 (3 - 4)	4 (4 - 5)	0 (0 - 0)	0 (0 - 0)	0 (0 - 1)
**Gabon**									
**15 - 49 years (rate per capita)**	0.060 (0.051 - 0.072)	0.058 (0.048 - 0.068)	0.060 (0.050 - 0.071)	0.007 (0.006 - 0.008)	0.007 (0.006 - 0.008)	0.007 (0.006 - 0.008)	0.397 (0.152 - 0.828)	0.378 (0.145 - 0.794)	0.394 (0.151 - 0.822)
**50 - 69 years (rate per capita)**	0.206 (0.174 - 0.243)	0.206 (0.172 - 0.242)	0.205 (0.173 - 0.242)	0.012 (0.009 - 0.015)	0.012 (0.009 - 0.015)	0.012 (0.009 - 0.015)	1.312 (0.517 - 2.663)	1.309 (0.518 - 2.647)	1.306 (0.512 - 2.671)
**70+ years (rate per capita)**	0.204 (0.176 - 0.237)	0.203 (0.175 - 0.235)	0.202 (0.174 - 0.234)	0.008 (0.006 - 0.011)	0.008 (0.006 - 0.011)	0.008 (0.006 - 0.011)	1.242 (0.490 - 2.506)	1.235 (0.497 - 2.541)	1.226 (0.488 - 2.540)
**All ages (rate per capita)**	0.059 (0.051 - 0.068)	0.055 (0.048 - 0.063)	0.057 (0.050 - 0.065)	0.005 (0.004 - 0.005)	0.005 (0.004 - 0.005)	0.005 (0.004 - 0.006)	0.377 (0.147 - 0.778)	0.353 (0.137 - 0.730)	0.364 (0.141 - 0.754)
**Age-standardized (rate per capita)**	0.084 (0.074 - 0.097)	0.084 (0.073 - 0.097)	0.084 (0.074 - 0.097)	0.006 (0.006 - 0.007)	0.006 (0.006 - 0.007)	0.006 (0.006 - 0.007)	0.540 (0.212 - 1.113)	0.538 (0.211 - 1.117)	0.539 (0.209 - 1.109)
**Total number of cases (thousands)**	56 (49 - 64)	76 (66 - 87)	98 (86 - 113)	5 (4 - 5)	6 (6 - 7)	8 (7 - 10)	0 (0 - 1)	0 (0 - 1)	1 (0 - 1)

Appendix 2, Table 5. Prevalence, incidence, and YLDs per 1,000 population (with 95% uncertainty intervals) for edentulism and severe tooth loss, 1990 and 2015, by country and by age

	Prevalence 1990	Prevalence 2005	Prevalence 2015	Incidence 1990	Incidence 2005	Incidence 2015	YLD rate 1990	YLD rate 2005	YLD rate 2015
**Global**									
**15 - 49 years (rate per capita)**	0.013 (0.012 - 0.014)	0.014 (0.013 - 0.015)	0.014 (0.013 - 0.015)	0.001 (0.001 - 0.001)	0.001 (0.001 - 0.001)	0.001 (0.001 - 0.001)	0.368 (0.243 - 0.517)	0.392 (0.260 - 0.550)	0.412 (0.274 - 0.579)
**50 - 69 years (rate per capita)**	0.107 (0.101 - 0.114)	0.102 (0.096 - 0.108)	0.101 (0.095 - 0.107)	0.011 (0.010 - 0.011)	0.010 (0.009 - 0.011)	0.010 (0.009 - 0.011)	2.976 (1.967 - 4.151)	2.827 (1.870 - 3.947)	2.814 (1.859 - 3.928)
**70+ years (rate per capita)**	0.248 (0.234 - 0.263)	0.240 (0.227 - 0.255)	0.237 (0.224 - 0.251)	0.010 (0.009 - 0.012)	0.010 (0.009 - 0.012)	0.010 (0.009 - 0.011)	6.644 (4.458 - 9.147)	6.451 (4.337 - 8.876)	6.349 (4.264 - 8.754)
**All ages (rate per capita)**	0.030 (0.028 - 0.031)	0.033 (0.032 - 0.035)	0.037 (0.036 - 0.039)	0.002 (0.002 - 0.002)	0.002 (0.002 - 0.003)	0.003 (0.003 - 0.003)	0.818 (0.546 - 1.128)	0.918 (0.614 - 1.269)	1.034 (0.690 - 1.430)
**Age-standardized (rate per capita)**	0.043 (0.041 - 0.045)	0.041 (0.040 - 0.043)	0.041 (0.039 - 0.043)	0.003 (0.003 - 0.003)	0.003 (0.003 - 0.003)	0.003 (0.003 - 0.003)	1.173 (0.786 - 1.621)	1.143 (0.765 - 1.581)	1.134 (0.759 - 1.567)
**Total number of cases (thousands)**	156,786 (150,462 - 163,829)	216,473 (207,563 - 226,331)	275,619 (264,201 - 288,252)	11,870 (11,246 - 12,548)	16,088 (15,249 - 16,954)	20,560 (19,500 - 21,707)	4,340 (2,897 - 5,984)	5,989 (4,004 - 8,277)	7,625 (5,088 - 10,540)
**High-income**									
**15 - 49 years (rate per capita)**	0.020 (0.019 - 0.021)	0.021 (0.020 - 0.022)	0.021 (0.020 - 0.023)	0.001 (0.001 - 0.002)	0.001 (0.001 - 0.002)	0.002 (0.001 - 0.002)	0.568 (0.377 - 0.795)	0.605 (0.401 - 0.845)	0.616 (0.409 - 0.861)
**50 - 69 years (rate per capita)**	0.139 (0.132 - 0.147)	0.133 (0.126 - 0.140)	0.137 (0.130 - 0.144)	0.014 (0.013 - 0.015)	0.014 (0.013 - 0.015)	0.014 (0.013 - 0.015)	3.895 (2.600 - 5.410)	3.725 (2.477 - 5.180)	3.829 (2.550 - 5.316)
**70+ years (rate per capita)**	0.297 (0.282 - 0.312)	0.289 (0.275 - 0.305)	0.286 (0.272 - 0.302)	0.010 (0.009 - 0.012)	0.010 (0.009 - 0.012)	0.010 (0.009 - 0.011)	7.987 (5.399 - 10.985)	7.786 (5.267 - 10.702)	7.690 (5.213 - 10.561)
**All ages (rate per capita)**	0.062 (0.060 - 0.065)	0.071 (0.068 - 0.074)	0.079 (0.076 - 0.082)	0.004 (0.004 - 0.005)	0.005 (0.005 - 0.005)	0.005 (0.005 - 0.006)	1.721 (1.162 - 2.376)	1.951 (1.317 - 2.696)	2.176 (1.470 - 3.001)
**Age-standardized (rate per capita)**	0.053 (0.051 - 0.055)	0.052 (0.050 - 0.054)	0.052 (0.050 - 0.055)	0.004 (0.004 - 0.004)	0.004 (0.004 - 0.004)	0.004 (0.004 - 0.004)	1.467 (0.989 - 2.026)	1.448 (0.975 - 2.000)	1.453 (0.979 - 2.008)
**Total number of cases (thousands)**	55,858 (53,850 - 58,132)	70,332 (67,787 - 73,298)	84,410 (81,306 - 88,043)	3,936 (3,737 - 4,139)	4,826 (4,590 - 5,070)	5,785 (5,493 - 6,088)	1,545 (1,042 - 2,132)	1,943 (1,312 - 2,685)	2,329 (1,573 - 3,212)
**High-income North America**									
**15 - 49 years (rate per capita)**	0.021 (0.020 - 0.023)	0.022 (0.021 - 0.024)	0.022 (0.021 - 0.023)	0.002 (0.001 - 0.002)	0.002 (0.001 - 0.002)	0.002 (0.001 - 0.002)	0.616 (0.409 - 0.861)	0.640 (0.425 - 0.891)	0.626 (0.416 - 0.876)
**50 - 69 years (rate per capita)**	0.145 (0.139 - 0.152)	0.123 (0.118 - 0.129)	0.129 (0.124 - 0.135)	0.014 (0.013 - 0.015)	0.012 (0.011 - 0.012)	0.012 (0.011 - 0.013)	4.021 (2.710 - 5.540)	3.433 (2.312 - 4.728)	3.590 (2.403 - 4.953)
**70+ years (rate per capita)**	0.273 (0.259 - 0.287)	0.252 (0.239 - 0.265)	0.251 (0.239 - 0.264)	0.008 (0.007 - 0.009)	0.007 (0.006 - 0.008)	0.007 (0.006 - 0.008)	7.270 (4.936 - 9.974)	6.683 (4.538 - 9.164)	6.642 (4.519 - 9.109)
**All ages (rate per capita)**	0.059 (0.057 - 0.061)	0.059 (0.057 - 0.062)	0.067 (0.064 - 0.069)	0.004 (0.004 - 0.004)	0.004 (0.004 - 0.004)	0.005 (0.004 - 0.005)	1.618 (1.093 - 2.223)	1.627 (1.098 - 2.229)	1.829 (1.238 - 2.516)
**Age-standardized (rate per capita)**	0.053 (0.051 - 0.055)	0.050 (0.048 - 0.052)	0.050 (0.048 - 0.052)	0.004 (0.003 - 0.004)	0.003 (0.003 - 0.004)	0.003 (0.003 - 0.004)	1.449 (0.978 - 1.991)	1.367 (0.922 - 1.873)	1.377 (0.931 - 1.893)
**Total number of cases (thousands)**	16,341 (15,784 - 16,965)	19,425 (18,757 - 20,187)	23,994 (23,176 - 24,943)	1,066 (1,014 - 1,117)	1,269 (1,212 - 1,328)	1,639 (1,556 - 1,722)	449 (304 - 617)	534 (360 - 731)	658 (445 - 905)
**Canada**									
**15 - 49 years (rate per capita)**	0.022 (0.020 - 0.023)	0.024 (0.022 - 0.026)	0.023 (0.021 - 0.025)	0.002 (0.001 - 0.002)	0.002 (0.002 - 0.002)	0.002 (0.002 - 0.002)	0.618 (0.408 - 0.866)	0.678 (0.450 - 0.945)	0.665 (0.439 - 0.934)
**50 - 69 years (rate per capita)**	0.140 (0.131 - 0.150)	0.129 (0.121 - 0.139)	0.133 (0.124 - 0.143)	0.013 (0.012 - 0.015)	0.012 (0.011 - 0.014)	0.013 (0.012 - 0.014)	3.905 (2.595 - 5.469)	3.614 (2.406 - 5.022)	3.728 (2.477 - 5.192)
**70+ years (rate per capita)**	0.271 (0.251 - 0.292)	0.265 (0.245 - 0.286)	0.260 (0.241 - 0.281)	0.008 (0.007 - 0.010)	0.008 (0.006 - 0.009)	0.007 (0.006 - 0.009)	7.254 (4.860 - 10.008)	7.085 (4.795 - 9.749)	6.950 (4.667 - 9.577)
**All ages (rate per capita)**	0.056 (0.053 - 0.059)	0.065 (0.062 - 0.069)	0.073 (0.069 - 0.078)	0.004 (0.003 - 0.004)	0.004 (0.004 - 0.005)	0.005 (0.005 - 0.005)	1.551 (1.036 - 2.142)	1.804 (1.205 - 2.485)	2.020 (1.350 - 2.786)
**Age-standardized (rate per capita)**	0.052 (0.049 - 0.055)	0.052 (0.049 - 0.055)	0.052 (0.049 - 0.055)	0.004 (0.003 - 0.004)	0.004 (0.003 - 0.004)	0.004 (0.003 - 0.004)	1.433 (0.957 - 1.979)	1.432 (0.961 - 1.976)	1.432 (0.956 - 1.976)
**Total number of cases (thousands)**	1,547 (1,469 - 1,638)	2,103 (1,995 - 2,232)	2,643 (2,506 - 2,805)	103 (97 - 111)	138 (129 - 148)	179 (167 - 193)	43 (29 - 59)	58 (39 - 80)	73 (49 - 101)
**Greenland**									
**15 - 49 years (rate per capita)**	0.020 (0.019 - 0.022)	0.023 (0.022 - 0.026)	0.022 (0.021 - 0.025)	0.002 (0.001 - 0.002)	0.002 (0.001 - 0.002)	0.002 (0.002 - 0.002)	0.583 (0.384 - 0.817)	0.668 (0.438 - 0.939)	0.643 (0.423 - 0.905)
**50 - 69 years (rate per capita)**	0.121 (0.112 - 0.130)	0.122 (0.113 - 0.131)	0.119 (0.110 - 0.127)	0.012 (0.011 - 0.013)	0.012 (0.011 - 0.013)	0.011 (0.010 - 0.013)	3.354 (2.205 - 4.656)	3.393 (2.233 - 4.751)	3.311 (2.188 - 4.640)
**70+ years (rate per capita)**	0.283 (0.263 - 0.307)	0.286 (0.265 - 0.309)	0.285 (0.265 - 0.308)	0.010 (0.008 - 0.012)	0.011 (0.009 - 0.013)	0.011 (0.009 - 0.014)	7.535 (5.088 - 10.415)	7.620 (5.139 - 10.468)	7.629 (5.181 - 10.630)
**All ages (rate per capita)**	0.029 (0.027 - 0.031)	0.038 (0.036 - 0.040)	0.047 (0.044 - 0.049)	0.002 (0.002 - 0.002)	0.003 (0.003 - 0.003)	0.004 (0.003 - 0.004)	0.807 (0.536 - 1.112)	1.062 (0.706 - 1.465)	1.299 (0.866 - 1.803)
**Age-standardized (rate per capita)**	0.051 (0.048 - 0.054)	0.051 (0.048 - 0.054)	0.051 (0.048 - 0.054)	0.003 (0.003 - 0.004)	0.003 (0.003 - 0.004)	0.003 (0.003 - 0.004)	1.401 (0.937 - 1.927)	1.399 (0.936 - 1.933)	1.398 (0.935 - 1.935)
**Total number of cases (thousands)**	2 (2 - 2)	2 (2 - 2)	3 (2 - 3)	0 (0 - 0)	0 (0 - 0)	0 (0 - 0)	0 (0 - 0)	0 (0 - 0)	0 (0 - 0)
**United States**									
**15 - 49 years (rate per capita)**	0.021 (0.020 - 0.023)	0.022 (0.021 - 0.023)	0.022 (0.021 - 0.023)	0.002 (0.001 - 0.002)	0.002 (0.001 - 0.002)	0.002 (0.001 - 0.002)	0.615 (0.409 - 0.861)	0.635 (0.422 - 0.887)	0.622 (0.413 - 0.871)
**50 - 69 years (rate per capita)**	0.146 (0.140 - 0.152)	0.123 (0.118 - 0.128)	0.129 (0.124 - 0.135)	0.014 (0.013 - 0.015)	0.012 (0.011 - 0.012)	0.012 (0.011 - 0.013)	4.035 (2.723 - 5.552)	3.412 (2.298 - 4.702)	3.574 (2.394 - 4.927)
**70+ years (rate per capita)**	0.273 (0.260 - 0.287)	0.250 (0.239 - 0.263)	0.250 (0.238 - 0.263)	0.008 (0.007 - 0.009)	0.007 (0.007 - 0.008)	0.007 (0.007 - 0.008)	7.271 (4.946 - 9.951)	6.637 (4.515 - 9.083)	6.605 (4.508 - 9.055)
**All ages (rate per capita)**	0.059 (0.057 - 0.061)	0.059 (0.057 - 0.061)	0.066 (0.064 - 0.068)	0.004 (0.004 - 0.004)	0.004 (0.004 - 0.004)	0.005 (0.004 - 0.005)	1.626 (1.098 - 2.232)	1.608 (1.085 - 2.206)	1.808 (1.223 - 2.487)
**Age-standardized (rate per capita)**	0.053 (0.051 - 0.055)	0.049 (0.048 - 0.051)	0.050 (0.048 - 0.052)	0.004 (0.003 - 0.004)	0.003 (0.003 - 0.003)	0.003 (0.003 - 0.004)	1.451 (0.979 - 1.993)	1.360 (0.917 - 1.866)	1.370 (0.925 - 1.883)
**Total number of cases (thousands)**	14,787 (14,298 - 15,332)	17,313 (16,735 - 17,946)	21,340 (20,625 - 22,149)	962 (917 - 1,006)	1,130 (1,082 - 1,180)	1,460 (1,391 - 1,529)	406 (275 - 558)	476 (321 - 652)	585 (396 - 804)
**Australasia**									
**15 - 49 years (rate per capita)**	0.027 (0.025 - 0.028)	0.028 (0.027 - 0.030)	0.028 (0.027 - 0.030)	0.002 (0.002 - 0.002)	0.002 (0.002 - 0.002)	0.002 (0.002 - 0.002)	0.762 (0.507 - 1.070)	0.811 (0.535 - 1.139)	0.805 (0.533 - 1.130)
**50 - 69 years (rate per capita)**	0.179 (0.168 - 0.189)	0.161 (0.153 - 0.171)	0.172 (0.162 - 0.182)	0.018 (0.016 - 0.019)	0.016 (0.015 - 0.017)	0.017 (0.016 - 0.019)	4.995 (3.331 - 6.976)	4.521 (3.017 - 6.283)	4.799 (3.222 - 6.672)
**70+ years (rate per capita)**	0.375 (0.355 - 0.396)	0.360 (0.341 - 0.379)	0.357 (0.339 - 0.378)	0.015 (0.013 - 0.018)	0.014 (0.012 - 0.016)	0.013 (0.011 - 0.015)	10.127 (6.840 - 13.963)	9.695 (6.578 - 13.382)	9.604 (6.500 - 13.117)
**All ages (rate per capita)**	0.072 (0.069 - 0.075)	0.080 (0.077 - 0.084)	0.087 (0.084 - 0.091)	0.005 (0.005 - 0.005)	0.006 (0.005 - 0.006)	0.006 (0.006 - 0.007)	2.001 (1.342 - 2.767)	2.227 (1.495 - 3.061)	2.410 (1.620 - 3.319)
**Age-standardized (rate per capita)**	0.068 (0.065 - 0.071)	0.066 (0.064 - 0.069)	0.067 (0.064 - 0.070)	0.005 (0.005 - 0.005)	0.005 (0.005 - 0.005)	0.005 (0.005 - 0.005)	1.885 (1.262 - 2.609)	1.830 (1.228 - 2.520)	1.859 (1.248 - 2.560)
**Total number of cases (thousands)**	1,472 (1,414 - 1,532)	1,972 (1,900 - 2,052)	2,516 (2,418 - 2,625)	105 (99 - 110)	137 (130 - 144)	178 (169 - 188)	41 (27 - 56)	55 (37 - 75)	70 (47 - 96)
**Australia**									
**15 - 49 years (rate per capita)**	0.026 (0.025 - 0.027)	0.028 (0.026 - 0.029)	0.027 (0.026 - 0.029)	0.002 (0.002 - 0.002)	0.002 (0.002 - 0.002)	0.002 (0.002 - 0.002)	0.745 (0.495 - 1.043)	0.790 (0.521 - 1.111)	0.783 (0.517 - 1.098)
**50 - 69 years (rate per capita)**	0.175 (0.165 - 0.185)	0.157 (0.149 - 0.167)	0.168 (0.158 - 0.178)	0.017 (0.016 - 0.019)	0.016 (0.014 - 0.017)	0.017 (0.015 - 0.018)	4.888 (3.256 - 6.830)	4.409 (2.937 - 6.106)	4.696 (3.152 - 6.507)
**70+ years (rate per capita)**	0.367 (0.348 - 0.388)	0.352 (0.333 - 0.370)	0.350 (0.332 - 0.371)	0.015 (0.012 - 0.017)	0.013 (0.011 - 0.016)	0.013 (0.011 - 0.015)	9.901 (6.683 - 13.619)	9.477 (6.421 - 13.037)	9.402 (6.353 - 12.882)
**All ages (rate per capita)**	0.071 (0.068 - 0.074)	0.079 (0.076 - 0.083)	0.085 (0.082 - 0.089)	0.005 (0.005 - 0.005)	0.005 (0.005 - 0.006)	0.006 (0.006 - 0.006)	1.961 (1.314 - 2.710)	2.194 (1.474 - 3.015)	2.357 (1.584 - 3.246)
**Age-standardized (rate per capita)**	0.066 (0.064 - 0.069)	0.064 (0.062 - 0.067)	0.066 (0.063 - 0.068)	0.005 (0.005 - 0.005)	0.005 (0.004 - 0.005)	0.005 (0.004 - 0.005)	1.842 (1.233 - 2.546)	1.786 (1.198 - 2.461)	1.819 (1.222 - 2.500)
**Total number of cases (thousands)**	1,204 (1,155 - 1,253)	1,615 (1,554 - 1,682)	2,072 (1,990 - 2,163)	86 (81 - 90)	112 (106 - 118)	146 (139 - 155)	33 (22 - 46)	45 (30 - 61)	57 (39 - 79)
**New Zealand**									
**15 - 49 years (rate per capita)**	0.030 (0.028 - 0.031)	0.032 (0.030 - 0.034)	0.032 (0.030 - 0.034)	0.002 (0.002 - 0.002)	0.002 (0.002 - 0.002)	0.002 (0.002 - 0.003)	0.848 (0.556 - 1.189)	0.913 (0.603 - 1.274)	0.923 (0.611 - 1.296)
**50 - 69 years (rate per capita)**	0.198 (0.186 - 0.211)	0.182 (0.171 - 0.194)	0.190 (0.179 - 0.203)	0.020 (0.018 - 0.022)	0.018 (0.017 - 0.020)	0.019 (0.017 - 0.021)	5.548 (3.683 - 7.767)	5.103 (3.395 - 7.151)	5.332 (3.534 - 7.519)
**70+ years (rate per capita)**	0.416 (0.392 - 0.440)	0.403 (0.382 - 0.427)	0.398 (0.376 - 0.420)	0.016 (0.014 - 0.019)	0.015 (0.013 - 0.018)	0.015 (0.012 - 0.017)	11.250 (7.601 - 15.501)	10.864 (7.325 - 15.036)	10.710 (7.247 - 14.766)
**All ages (rate per capita)**	0.079 (0.076 - 0.083)	0.086 (0.083 - 0.090)	0.097 (0.093 - 0.102)	0.006 (0.005 - 0.006)	0.006 (0.006 - 0.006)	0.007 (0.007 - 0.007)	2.205 (1.478 - 3.052)	2.391 (1.609 - 3.286)	2.694 (1.808 - 3.744)
**Age-standardized (rate per capita)**	0.076 (0.073 - 0.079)	0.074 (0.071 - 0.077)	0.075 (0.072 - 0.078)	0.005 (0.005 - 0.006)	0.005 (0.005 - 0.006)	0.005 (0.005 - 0.006)	2.105 (1.410 - 2.911)	2.058 (1.383 - 2.829)	2.074 (1.392 - 2.882)
**Total number of cases (thousands)**	268 (257 - 280)	357 (343 - 372)	444 (426 - 464)	19 (18 - 20)	25 (24 - 26)	32 (30 - 34)	7 (5 - 10)	10 (7 - 14)	12 (8 - 17)
**High-income Asia Pacific**									
**15 - 49 years (rate per capita)**	0.020 (0.019 - 0.022)	0.022 (0.021 - 0.023)	0.023 (0.022 - 0.025)	0.001 (0.001 - 0.002)	0.002 (0.001 - 0.002)	0.002 (0.002 - 0.002)	0.589 (0.389 - 0.832)	0.630 (0.417 - 0.881)	0.668 (0.440 - 0.939)
**50 - 69 years (rate per capita)**	0.137 (0.131 - 0.143)	0.143 (0.137 - 0.150)	0.152 (0.145 - 0.159)	0.014 (0.013 - 0.015)	0.015 (0.014 - 0.016)	0.016 (0.015 - 0.017)	3.848 (2.577 - 5.326)	4.051 (2.706 - 5.623)	4.295 (2.869 - 5.945)
**70+ years (rate per capita)**	0.303 (0.289 - 0.318)	0.303 (0.289 - 0.318)	0.297 (0.283 - 0.312)	0.012 (0.011 - 0.013)	0.011 (0.010 - 0.013)	0.010 (0.009 - 0.012)	8.234 (5.549 - 11.331)	8.250 (5.559 - 11.365)	8.056 (5.456 - 11.063)
**All ages (rate per capita)**	0.058 (0.056 - 0.060)	0.082 (0.080 - 0.086)	0.098 (0.094 - 0.101)	0.004 (0.004 - 0.005)	0.006 (0.006 - 0.006)	0.007 (0.006 - 0.007)	1.614 (1.085 - 2.233)	2.297 (1.547 - 3.182)	2.711 (1.828 - 3.757)
**Age-standardized (rate per capita)**	0.054 (0.052 - 0.056)	0.055 (0.053 - 0.057)	0.054 (0.053 - 0.057)	0.004 (0.004 - 0.004)	0.004 (0.004 - 0.004)	0.004 (0.004 - 0.004)	1.508 (1.014 - 2.086)	1.531 (1.028 - 2.117)	1.526 (1.025 - 2.111)
**Total number of cases (thousands)**	9,747 (9,435 - 10,110)	14,612 (14,108 - 15,172)	17,841 (17,180 - 18,562)	736 (703 - 770)	1,031 (981 - 1,079)	1,201 (1,139 - 1,262)	273 (183 - 377)	407 (274 - 564)	496 (334 - 687)
**Brunei**									
**15 - 49 years (rate per capita)**	0.018 (0.017 - 0.019)	0.019 (0.018 - 0.021)	0.021 (0.019 - 0.022)	0.001 (0.001 - 0.001)	0.001 (0.001 - 0.002)	0.001 (0.001 - 0.002)	0.518 (0.344 - 0.735)	0.563 (0.373 - 0.787)	0.596 (0.392 - 0.835)
**50 - 69 years (rate per capita)**	0.126 (0.119 - 0.135)	0.120 (0.113 - 0.128)	0.124 (0.116 - 0.132)	0.013 (0.012 - 0.014)	0.012 (0.011 - 0.014)	0.013 (0.012 - 0.014)	3.547 (2.338 - 4.927)	3.380 (2.231 - 4.697)	3.476 (2.305 - 4.873)

	Prevalence 1990	Prevalence 2005	Prevalence 2015	Incidence 1990	Incidence 2005	Incidence 2015	YLD rate 1990	YLD rate 2005	YLD rate 2015
**70+ years (rate per capita)**	0.304 (0.282 - 0.326)	0.305 (0.283 - 0.327)	0.305 (0.283 - 0.327)	0.012 (0.010 - 0.014)	0.012 (0.011 - 0.015)	0.013 (0.011 - 0.015)	8.180 (5.468 - 11.306)	8.226 (5.507 - 11.374)	8.233 (5.520 - 11.373)
**All ages (rate per capita)**	0.024 (0.023 - 0.025)	0.029 (0.028 - 0.031)	0.039 (0.037 - 0.041)	0.002 (0.002 - 0.002)	0.002 (0.002 - 0.002)	0.003 (0.003 - 0.003)	0.665 (0.440 - 0.924)	0.823 (0.547 - 1.137)	1.096 (0.726 - 1.524)
**Age-standardized (rate per capita)**	0.054 (0.052 - 0.057)	0.054 (0.051 - 0.057)	0.054 (0.051 - 0.057)	0.004 (0.004 - 0.004)	0.004 (0.004 - 0.004)	0.004 (0.004 - 0.004)	1.504 (1.000 - 2.093)	1.499 (0.998 - 2.080)	1.498 (0.999 - 2.082)
**Total number of cases (thousands)**	6 (6 - 6)	11 (10 - 11)	16 (16 - 17)	0 (0 - 0)	1 (1 - 1)	1 (1 - 1)	0 (0 - 0)	0 (0 - 0)	0 (0 - 1)
**Japan**									
**15 - 49 years (rate per capita)**	0.021 (0.020 - 0.023)	0.022 (0.021 - 0.023)	0.023 (0.022 - 0.025)	0.002 (0.001 - 0.002)	0.002 (0.001 - 0.002)	0.002 (0.002 - 0.002)	0.618 (0.410 - 0.870)	0.633 (0.420 - 0.889)	0.678 (0.449 - 0.952)
**50 - 69 years (rate per capita)**	0.138 (0.133 - 0.144)	0.147 (0.141 - 0.153)	0.160 (0.153 - 0.167)	0.014 (0.013 - 0.015)	0.015 (0.014 - 0.016)	0.017 (0.015 - 0.018)	3.905 (2.617 - 5.393)	4.146 (2.780 - 5.749)	4.526 (3.042 - 6.260)
**70+ years (rate per capita)**	0.303 (0.289 - 0.317)	0.304 (0.290 - 0.318)	0.296 (0.283 - 0.310)	0.012 (0.010 - 0.013)	0.011 (0.010 - 0.013)	0.010 (0.009 - 0.011)	8.237 (5.556 - 11.336)	8.259 (5.572 - 11.403)	8.052 (5.460 - 11.063)
**All ages (rate per capita)**	0.066 (0.064 - 0.068)	0.094 (0.090 - 0.097)	0.109 (0.105 - 0.113)	0.005 (0.005 - 0.005)	0.006 (0.006 - 0.007)	0.007 (0.007 - 0.007)	1.838 (1.234 - 2.535)	2.604 (1.751 - 3.604)	3.023 (2.039 - 4.199)
**Age-standardized (rate per capita)**	0.054 (0.053 - 0.056)	0.055 (0.053 - 0.057)	0.055 (0.053 - 0.057)	0.004 (0.004 - 0.004)	0.004 (0.004 - 0.004)	0.004 (0.004 - 0.004)	1.513 (1.015 - 2.086)	1.539 (1.032 - 2.131)	1.534 (1.027 - 2.122)
**Total number of cases (thousands)**	8,076 (7,830 - 8,365)	11,789 (11,393 - 12,236)	13,969 (13,482 - 14,516)	603 (578 - 629)	816 (778 - 853)	913 (869 - 958)	226 (152 - 311)	328 (221 - 454)	388 (262 - 539)
**Singapore**									
**15 - 49 years (rate per capita)**	0.019 (0.018 - 0.021)	0.023 (0.021 - 0.025)	0.022 (0.021 - 0.024)	0.001 (0.001 - 0.001)	0.002 (0.001 - 0.002)	0.002 (0.001 - 0.002)	0.563 (0.370 - 0.792)	0.665 (0.438 - 0.933)	0.647 (0.426 - 0.908)
**50 - 69 years (rate per capita)**	0.133 (0.125 - 0.142)	0.126 (0.118 - 0.134)	0.127 (0.120 - 0.136)	0.014 (0.012 - 0.015)	0.013 (0.012 - 0.014)	0.013 (0.012 - 0.015)	3.756 (2.473 - 5.267)	3.570 (2.351 - 5.023)	3.625 (2.388 - 5.117)
**70+ years (rate per capita)**	0.304 (0.283 - 0.327)	0.297 (0.276 - 0.320)	0.290 (0.270 - 0.312)	0.012 (0.011 - 0.014)	0.011 (0.010 - 0.013)	0.011 (0.009 - 0.012)	8.299 (5.583 - 11.511)	8.127 (5.467 - 11.342)	7.934 (5.309 - 11.085)
**All ages (rate per capita)**	0.041 (0.039 - 0.043)	0.054 (0.051 - 0.057)	0.065 (0.062 - 0.069)	0.003 (0.003 - 0.003)	0.004 (0.004 - 0.004)	0.005 (0.005 - 0.005)	1.151 (0.762 - 1.604)	1.521 (1.012 - 2.106)	1.844 (1.228 - 2.574)
**Age-standardized (rate per capita)**	0.054 (0.051 - 0.057)	0.054 (0.051 - 0.057)	0.054 (0.051 - 0.057)	0.004 (0.004 - 0.004)	0.004 (0.004 - 0.004)	0.004 (0.004 - 0.004)	1.509 (1.007 - 2.107)	1.514 (1.012 - 2.108)	1.516 (1.010 - 2.121)
**Total number of cases (thousands)**	112 (107 - 118)	192 (183 - 202)	257 (245 - 270)	8 (8 - 9)	14 (14 - 15)	19 (18 - 21)	3 (2 - 4)	5 (4 - 7)	7 (5 - 10)
**South Korea**									
**15 - 49 years (rate per capita)**	0.018 (0.017 - 0.019)	0.021 (0.020 - 0.023)	0.023 (0.021 - 0.024)	0.001 (0.001 - 0.001)	0.002 (0.001 - 0.002)	0.002 (0.001 - 0.002)	0.519 (0.340 - 0.731)	0.621 (0.407 - 0.871)	0.651 (0.426 - 0.914)
**50 - 69 years (rate per capita)**	0.128 (0.120 - 0.136)	0.132 (0.124 - 0.141)	0.131 (0.123 - 0.140)	0.013 (0.012 - 0.014)	0.014 (0.013 - 0.015)	0.014 (0.012 - 0.015)	3.583 (2.375 - 4.968)	3.726 (2.482 - 5.200)	3.707 (2.464 - 5.180)
**70+ years (rate per capita)**	0.304 (0.283 - 0.327)	0.303 (0.283 - 0.326)	0.299 (0.279 - 0.321)	0.013 (0.011 - 0.015)	0.013 (0.011 - 0.015)	0.012 (0.010 - 0.014)	8.206 (5.505 - 11.339)	8.198 (5.495 - 11.288)	8.088 (5.429 - 11.179)
**All ages (rate per capita)**	0.036 (0.034 - 0.038)	0.055 (0.053 - 0.058)	0.072 (0.068 - 0.075)	0.003 (0.003 - 0.003)	0.004 (0.004 - 0.004)	0.005 (0.005 - 0.006)	1.012 (0.673 - 1.400)	1.547 (1.039 - 2.132)	1.996 (1.336 - 2.758)
**Age-standardized (rate per capita)**	0.053 (0.051 - 0.056)	0.054 (0.051 - 0.056)	0.054 (0.051 - 0.056)	0.004 (0.004 - 0.004)	0.004 (0.004 - 0.004)	0.004 (0.004 - 0.004)	1.481 (0.991 - 2.045)	1.491 (1.003 - 2.057)	1.497 (1.002 - 2.068)
**Total number of cases (thousands)**	1,553 (1,483 - 1,632)	2,620 (2,497 - 2,750)	3,599 (3,430 - 3,784)	124 (117 - 131)	200 (188 - 213)	267 (251 - 284)	44 (29 - 60)	73 (49 - 101)	100 (67 - 139)
**Western Europe**									
**15 - 49 years (rate per capita)**	0.018 (0.017 - 0.020)	0.020 (0.019 - 0.022)	0.021 (0.020 - 0.022)	0.001 (0.001 - 0.001)	0.001 (0.001 - 0.002)	0.001 (0.001 - 0.002)	0.529 (0.351 - 0.740)	0.579 (0.383 - 0.811)	0.599 (0.397 - 0.839)
**50 - 69 years (rate per capita)**	0.138 (0.130 - 0.147)	0.136 (0.128 - 0.144)	0.137 (0.128 - 0.145)	0.015 (0.014 - 0.016)	0.015 (0.014 - 0.016)	0.015 (0.014 - 0.016)	3.853 (2.556 - 5.339)	3.795 (2.521 - 5.301)	3.827 (2.537 - 5.325)
**70+ years (rate per capita)**	0.310 (0.294 - 0.327)	0.306 (0.289 - 0.322)	0.301 (0.284 - 0.317)	0.011 (0.010 - 0.013)	0.011 (0.010 - 0.013)	0.011 (0.010 - 0.013)	8.356 (5.649 - 11.503)	8.234 (5.568 - 11.320)	8.097 (5.487 - 11.131)
**All ages (rate per capita)**	0.069 (0.066 - 0.072)	0.078 (0.075 - 0.082)	0.085 (0.082 - 0.089)	0.005 (0.005 - 0.005)	0.005 (0.005 - 0.006)	0.006 (0.006 - 0.006)	1.911 (1.288 - 2.643)	2.153 (1.450 - 2.975)	2.351 (1.587 - 3.251)
**Age-standardized (rate per capita)**	0.053 (0.051 - 0.055)	0.053 (0.050 - 0.055)	0.053 (0.051 - 0.055)	0.004 (0.004 - 0.004)	0.004 (0.004 - 0.004)	0.004 (0.004 - 0.004)	1.461 (0.982 - 2.019)	1.458 (0.979 - 2.014)	1.465 (0.984 - 2.024)
**Total number of cases (thousands)**	26,393 (25,310 - 27,610)	31,788 (30,500 - 33,297)	36,972 (35,463 - 38,737)	1,887 (1,785 - 1,992)	2,207 (2,087 - 2,328)	2,546 (2,409 - 2,696)	729 (491 - 1,008)	877 (591 - 1,212)	1,019 (688 - 1,410)
**Andorra**									
**15 - 49 years (rate per capita)**	0.023 (0.019 - 0.027)	0.025 (0.021 - 0.029)	0.026 (0.022 - 0.031)	0.002 (0.001 - 0.002)	0.002 (0.001 - 0.002)	0.002 (0.002 - 0.003)	0.651 (0.416 - 0.948)	0.713 (0.458 - 1.027)	0.744 (0.479 - 1.079)
**50 - 69 years (rate per capita)**	0.181 (0.150 - 0.214)	0.178 (0.147 - 0.210)	0.177 (0.146 - 0.209)	0.019 (0.015 - 0.024)	0.019 (0.015 - 0.023)	0.019 (0.015 - 0.023)	5.079 (3.196 - 7.386)	4.986 (3.119 - 7.297)	4.958 (3.109 - 7.251)
**70+ years (rate per capita)**	0.383 (0.329 - 0.444)	0.379 (0.326 - 0.440)	0.372 (0.320 - 0.432)	0.013 (0.010 - 0.018)	0.014 (0.010 - 0.018)	0.013 (0.009 - 0.017)	10.336 (6.669 - 14.446)	10.245 (6.620 - 14.492)	10.028 (6.472 - 14.135)
**All ages (rate per capita)**	0.088 (0.077 - 0.100)	0.099 (0.087 - 0.112)	0.110 (0.096 - 0.126)	0.006 (0.005 - 0.007)	0.007 (0.006 - 0.008)	0.007 (0.006 - 0.009)	2.429 (1.561 - 3.418)	2.736 (1.753 - 3.870)	3.043 (1.956 - 4.302)
**Age-standardized (rate per capita)**	0.067 (0.059 - 0.076)	0.067 (0.058 - 0.076)	0.067 (0.058 - 0.075)	0.005 (0.004 - 0.006)	0.005 (0.004 - 0.006)	0.005 (0.004 - 0.006)	1.857 (1.195 - 2.620)	1.852 (1.189 - 2.614)	1.849 (1.189 - 2.616)
**Total number of cases (thousands)**	4 (4 - 5)	7 (6 - 8)	9 (8 - 10)	0 (0 - 0)	0 (0 - 1)	1 (1 - 1)	0 (0 - 0)	0 (0 - 0)	0 (0 - 0)
**Austria**									
**15 - 49 years (rate per capita)**	0.027 (0.025 - 0.029)	0.029 (0.027 - 0.032)	0.030 (0.028 - 0.032)	0.002 (0.002 - 0.002)	0.002 (0.002 - 0.002)	0.002 (0.002 - 0.002)	0.766 (0.507 - 1.069)	0.847 (0.560 - 1.191)	0.859 (0.568 - 1.209)
**50 - 69 years (rate per capita)**	0.199 (0.184 - 0.215)	0.193 (0.178 - 0.208)	0.186 (0.172 - 0.202)	0.022 (0.019 - 0.024)	0.021 (0.019 - 0.023)	0.020 (0.018 - 0.022)	5.554 (3.680 - 7.775)	5.390 (3.555 - 7.558)	5.208 (3.423 - 7.302)
**70+ years (rate per capita)**	0.420 (0.393 - 0.447)	0.414 (0.389 - 0.441)	0.410 (0.383 - 0.435)	0.014 (0.012 - 0.017)	0.014 (0.012 - 0.017)	0.015 (0.013 - 0.018)	11.313 (7.613 - 15.604)	11.161 (7.571 - 15.452)	11.039 (7.413 - 15.201)
**All ages (rate per capita)**	0.097 (0.092 - 0.103)	0.106 (0.100 - 0.112)	0.118 (0.111 - 0.125)	0.007 (0.006 - 0.007)	0.007 (0.007 - 0.008)	0.008 (0.008 - 0.009)	2.685 (1.795 - 3.719)	2.915 (1.961 - 4.063)	3.244 (2.179 - 4.511)
**Age-standardized (rate per capita)**	0.073 (0.070 - 0.078)	0.073 (0.070 - 0.078)	0.073 (0.069 - 0.077)	0.005 (0.005 - 0.006)	0.005 (0.005 - 0.006)	0.005 (0.005 - 0.006)	2.037 (1.362 - 2.822)	2.035 (1.363 - 2.836)	2.030 (1.356 - 2.808)
**Total number of cases (thousands)**	745 (707 - 789)	868 (825 - 921)	1,020 (967 - 1,080)	53 (49 - 57)	60 (56 - 65)	71 (66 - 76)	21 (14 - 29)	24 (16 - 33)	28 (19 - 39)
**Belgium**									
**15 - 49 years (rate per capita)**	0.025 (0.023 - 0.027)	0.027 (0.026 - 0.030)	0.027 (0.025 - 0.029)	0.002 (0.002 - 0.002)	0.002 (0.002 - 0.002)	0.002 (0.002 - 0.002)	0.713 (0.469 - 0.996)	0.785 (0.524 - 1.104)	0.772 (0.510 - 1.080)
**50 - 69 years (rate per capita)**	0.187 (0.174 - 0.202)	0.176 (0.164 - 0.190)	0.173 (0.160 - 0.187)	0.020 (0.018 - 0.022)	0.019 (0.017 - 0.021)	0.019 (0.017 - 0.021)	5.211 (3.446 - 7.323)	4.921 (3.266 - 6.899)	4.837 (3.206 - 6.814)
**70+ years (rate per capita)**	0.401 (0.377 - 0.426)	0.399 (0.376 - 0.424)	0.392 (0.368 - 0.416)	0.014 (0.012 - 0.017)	0.015 (0.012 - 0.017)	0.013 (0.011 - 0.016)	10.752 (7.264 - 14.730)	10.694 (7.241 - 14.704)	10.479 (7.115 - 14.388)
**All ages (rate per capita)**	0.093 (0.088 - 0.098)	0.103 (0.098 - 0.108)	0.107 (0.102 - 0.113)	0.007 (0.006 - 0.007)	0.007 (0.007 - 0.008)	0.007 (0.007 - 0.008)	2.562 (1.727 - 3.554)	2.825 (1.908 - 3.936)	2.944 (1.990 - 4.094)
**Age-standardized (rate per capita)**	0.069 (0.066 - 0.073)	0.069 (0.066 - 0.073)	0.069 (0.066 - 0.073)	0.005 (0.005 - 0.006)	0.005 (0.005 - 0.005)	0.005 (0.005 - 0.006)	1.918 (1.294 - 2.653)	1.919 (1.289 - 2.671)	1.912 (1.288 - 2.653)
**Total number of cases (thousands)**	926 (878 - 976)	1,079 (1,026 - 1,137)	1,216 (1,155 - 1,282)	67 (63 - 71)	74 (69 - 79)	83 (78 - 89)	25 (17 - 35)	30 (20 - 41)	33 (23 - 46)
**Cyprus**									
**15 - 49 years (rate per capita)**	0.021 (0.018 - 0.026)	0.023 (0.019 - 0.027)	0.023 (0.019 - 0.027)	0.002 (0.001 - 0.002)	0.002 (0.001 - 0.002)	0.002 (0.001 - 0.002)	0.617 (0.398 - 0.900)	0.657 (0.424 - 0.960)	0.651 (0.420 - 0.946)
**50 - 69 years (rate per capita)**	0.165 (0.137 - 0.196)	0.167 (0.139 - 0.199)	0.168 (0.140 - 0.200)	0.018 (0.014 - 0.022)	0.018 (0.014 - 0.023)	0.018 (0.014 - 0.023)	4.623 (2.877 - 6.732)	4.679 (2.923 - 6.869)	4.716 (2.947 - 6.922)
**70+ years (rate per capita)**	0.369 (0.316 - 0.433)	0.370 (0.317 - 0.433)	0.368 (0.315 - 0.431)	0.014 (0.010 - 0.019)	0.014 (0.010 - 0.018)	0.014 (0.010 - 0.018)	9.947 (6.387 - 14.136)	9.943 (6.381 - 14.152)	9.881 (6.352 - 14.116)
**All ages (rate per capita)**	0.064 (0.056 - 0.073)	0.078 (0.068 - 0.089)	0.085 (0.075 - 0.097)	0.005 (0.004 - 0.006)	0.006 (0.005 - 0.007)	0.006 (0.005 - 0.007)	1.768 (1.132 - 2.525)	2.148 (1.379 - 3.049)	2.356 (1.502 - 3.373)
**Age-standardized (rate per capita)**	0.065 (0.057 - 0.074)	0.065 (0.057 - 0.074)	0.065 (0.057 - 0.074)	0.005 (0.004 - 0.006)	0.005 (0.004 - 0.006)	0.005 (0.004 - 0.006)	1.805 (1.155 - 2.581)	1.804 (1.161 - 2.562)	1.804 (1.153 - 2.574)
**Total number of cases (thousands)**	43 (37 - 49)	59 (51 - 67)	76 (66 - 87)	3 (3 - 4)	4 (4 - 5)	6 (5 - 6)	1 (1 - 2)	2 (1 - 2)	2 (1 - 3)
**Denmark**									
**15 - 49 years (rate per capita)**	0.018 (0.017 - 0.019)	0.019 (0.018 - 0.021)	0.019 (0.018 - 0.020)	0.001 (0.001 - 0.001)	0.001 (0.001 - 0.001)	0.001 (0.001 - 0.002)	0.524 (0.348 - 0.738)	0.553 (0.368 - 0.777)	0.547 (0.363 - 0.767)
**50 - 69 years (rate per capita)**	0.132 (0.124 - 0.141)	0.125 (0.117 - 0.133)	0.138 (0.128 - 0.148)	0.014 (0.013 - 0.015)	0.013 (0.012 - 0.015)	0.015 (0.013 - 0.016)	3.691 (2.453 - 5.167)	3.488 (2.318 - 4.883)	3.847 (2.555 - 5.346)
**70+ years (rate per capita)**	0.300 (0.283 - 0.317)	0.291 (0.275 - 0.308)	0.296 (0.279 - 0.314)	0.011 (0.010 - 0.013)	0.011 (0.009 - 0.012)	0.012 (0.010 - 0.013)	8.065 (5.435 - 11.040)	7.835 (5.298 - 10.772)	7.982 (5.359 - 10.984)
**All ages (rate per capita)**	0.069 (0.066 - 0.072)	0.070 (0.067 - 0.074)	0.079 (0.075 - 0.083)	0.005 (0.004 - 0.005)	0.005 (0.005 - 0.005)	0.006 (0.005 - 0.006)	1.890 (1.268 - 2.599)	1.934 (1.296 - 2.665)	2.174 (1.457 - 3.012)
**Age-standardized (rate per capita)**	0.050 (0.048 - 0.053)	0.050 (0.048 - 0.052)	0.050 (0.048 - 0.053)	0.004 (0.004 - 0.004)	0.004 (0.003 - 0.004)	0.004 (0.004 - 0.004)	1.396 (0.934 - 1.920)	1.382 (0.925 - 1.904)	1.396 (0.935 - 1.940)
**Total number of cases (thousands)**	352 (337 - 369)	380 (363 - 398)	450 (427 - 475)	24 (23 - 26)	27 (26 - 29)	33 (31 - 35)	10 (7 - 13)	10 (7 - 14)	12 (8 - 17)
**Finland**									
**15 - 49 years (rate per capita)**	0.025 (0.024 - 0.027)	0.026 (0.024 - 0.027)	0.025 (0.024 - 0.027)	0.002 (0.002 - 0.002)	0.002 (0.002 - 0.002)	0.002 (0.002 - 0.002)	0.730 (0.482 - 1.024)	0.740 (0.494 - 1.032)	0.725 (0.482 - 1.024)
**50 - 69 years (rate per capita)**	0.181 (0.170 - 0.192)	0.166 (0.156 - 0.177)	0.180 (0.169 - 0.192)	0.020 (0.018 - 0.021)	0.018 (0.017 - 0.020)	0.020 (0.018 - 0.022)	5.060 (3.383 - 7.047)	4.643 (3.082 - 6.429)	5.036 (3.344 - 7.012)
**70+ years (rate per capita)**	0.406 (0.386 - 0.427)	0.390 (0.371 - 0.409)	0.385 (0.365 - 0.405)	0.015 (0.013 - 0.018)	0.014 (0.012 - 0.016)	0.014 (0.012 - 0.016)	10.942 (7.384 - 14.973)	10.449 (7.113 - 14.243)	10.314 (6.965 - 14.094)
**All ages (rate per capita)**	0.086 (0.082 - 0.090)	0.098 (0.094 - 0.102)	0.112 (0.107 - 0.116)	0.006 (0.006 - 0.007)	0.007 (0.007 - 0.007)	0.008 (0.008 - 0.009)	2.374 (1.601 - 3.271)	2.704 (1.817 - 3.724)	3.071 (2.062 - 4.217)
**Age-standardized (rate per capita)**	0.069 (0.066 - 0.072)	0.067 (0.065 - 0.070)	0.068 (0.065 - 0.070)	0.005 (0.005 - 0.005)	0.005 (0.005 - 0.005)	0.005 (0.005 - 0.005)	1.905 (1.284 - 2.629)	1.860 (1.251 - 2.565)	1.873 (1.254 - 2.578)
**Total number of cases (thousands)**	429 (411 - 447)	515 (494 - 538)	619 (593 - 646)	31 (29 - 33)	37 (35 - 39)	45 (42 - 48)	12 (8 - 16)	14 (10 - 20)	17 (11 - 23)
**France**									

	Prevalence 1990	Prevalence 2005	Prevalence 2015	Incidence 1990	Incidence 2005	Incidence 2015	YLD rate 1990	YLD rate 2005	YLD rate 2015
15 - 49 years (rate per capita)	0.018 (0.017 - 0.020)	0.020 (0.019 - 0.022)	0.020 (0.019 - 0.022)	0.001 (0.001 - 0.001)	0.001 (0.001 - 0.002)	0.001 (0.001 - 0.002)	0.531 (0.347 - 0.745)	0.578 (0.384 - 0.816)	0.584 (0.385 - 0.816)
50 - 69 years (rate per capita)	0.146 (0.136 - 0.156)	0.135 (0.126 - 0.145)	0.141 (0.132 - 0.153)	0.016 (0.014 - 0.017)	0.015 (0.013 - 0.016)	0.016 (0.014 - 0.017)	4.077 (2.692 - 5.763)	3.786 (2.514 - 5.296)	3.963 (2.629 - 5.582)
70+ years (rate per capita)	0.320 (0.300 - 0.341)	0.323 (0.303 - 0.346)	0.313 (0.293 - 0.333)	0.011 (0.009 - 0.013)	0.012 (0.010 - 0.013)	0.010 (0.009 - 0.012)	8.616 (5.812 - 11.835)	8.707 (5.881 - 11.983)	8.408 (5.699 - 11.562)
All ages (rate per capita)	0.069 (0.065 - 0.072)	0.079 (0.075 - 0.083)	0.085 (0.081 - 0.091)	0.005 (0.005 - 0.005)	0.005 (0.005 - 0.006)	0.006 (0.005 - 0.006)	1.893 (1.269 - 2.610)	2.178 (1.472 - 3.003)	2.349 (1.579 - 3.274)
Age-standardized (rate per capita)	0.055 (0.052 - 0.057)	0.055 (0.052 - 0.058)	0.055 (0.052 - 0.058)	0.004 (0.004 - 0.004)	0.004 (0.004 - 0.004)	0.004 (0.004 - 0.004)	1.513 (1.012 - 2.085)	1.527 (1.028 - 2.114)	1.521 (1.017 - 2.121)
Total number of cases (thousands)	3,886 (3,699 - 4,093)	4,835 (4,593 - 5,096)	5,567 (5,279 - 5,906)	274 (257 - 293)	327 (306 - 349)	381 (355 - 410)	107 (72 - 148)	133 (90 - 184)	153 (103 - 214)
**Germany**									
15 - 49 years (rate per capita)	0.019 (0.018 - 0.021)	0.021 (0.020 - 0.022)	0.022 (0.021 - 0.023)	0.001 (0.001 - 0.001)	0.001 (0.001 - 0.002)	0.002 (0.001 - 0.002)	0.554 (0.371 - 0.775)	0.598 (0.396 - 0.842)	0.629 (0.418 - 0.879)
50 - 69 years (rate per capita)	0.136 (0.127 - 0.146)	0.144 (0.134 - 0.153)	0.132 (0.123 - 0.141)	0.015 (0.013 - 0.016)	0.015 (0.014 - 0.017)	0.014 (0.013 - 0.016)	3.800 (2.511 - 5.264)	4.013 (2.659 - 5.636)	3.687 (2.432 - 5.084)
70+ years (rate per capita)	0.320 (0.302 - 0.339)	0.314 (0.298 - 0.333)	0.313 (0.295 - 0.331)	0.011 (0.009 - 0.012)	0.012 (0.010 - 0.014)	0.013 (0.011 - 0.014)	8.597 (5.827 - 11.830)	8.486 (5.752 - 11.614)	8.445 (5.709 - 11.609)
All ages (rate per capita)	0.074 (0.071 - 0.078)	0.085 (0.081 - 0.089)	0.098 (0.093 - 0.103)	0.005 (0.005 - 0.006)	0.006 (0.006 - 0.006)	0.007 (0.006 - 0.007)	2.047 (1.373 - 2.813)	2.346 (1.575 - 3.242)	2.695 (1.809 - 3.729)
Age-standardized (rate per capita)	0.054 (0.052 - 0.057)	0.053 (0.051 - 0.056)	0.053 (0.051 - 0.056)	0.004 (0.004 - 0.004)	0.004 (0.004 - 0.004)	0.004 (0.004 - 0.004)	1.497 (1.002 - 2.057)	1.478 (0.990 - 2.040)	1.482 (0.995 - 2.046)
Total number of cases (thousands)	5,877 (5,613 - 6,166)	7,003 (6,686 - 7,333)	8,186 (7,809 - 8,593)	417 (394 - 441)	495 (467 - 525)	556 (523 - 589)	162 (109 - 223)	193 (130 - 267)	225 (151 - 312)
**Greece**									
15 - 49 years (rate per capita)	0.023 (0.022 - 0.025)	0.025 (0.023 - 0.027)	0.026 (0.025 - 0.028)	0.002 (0.001 - 0.002)	0.002 (0.002 - 0.002)	0.002 (0.002 - 0.002)	0.659 (0.438 - 0.927)	0.710 (0.471 - 0.995)	0.759 (0.504 - 1.063)
50 - 69 years (rate per capita)	0.171 (0.160 - 0.184)	0.178 (0.166 - 0.190)	0.176 (0.165 - 0.188)	0.019 (0.017 - 0.020)	0.019 (0.018 - 0.021)	0.019 (0.017 - 0.021)	4.799 (3.169 - 6.697)	4.993 (3.297 - 6.968)	4.942 (3.269 - 6.890)
70+ years (rate per capita)	0.384 (0.362 - 0.407)	0.389 (0.366 - 0.411)	0.374 (0.352 - 0.396)	0.014 (0.012 - 0.017)	0.015 (0.013 - 0.018)	0.012 (0.011 - 0.015)	10.364 (7.000 - 14.429)	10.528 (7.059 - 14.517)	10.085 (6.793 - 13.885)
All ages (rate per capita)	0.087 (0.083 - 0.092)	0.103 (0.098 - 0.108)	0.114 (0.109 - 0.119)	0.006 (0.006 - 0.007)	0.007 (0.007 - 0.008)	0.007 (0.007 - 0.008)	2.410 (1.616 - 3.339)	2.844 (1.907 - 3.927)	3.140 (2.112 - 4.337)
Age-standardized (rate per capita)	0.066 (0.063 - 0.070)	0.066 (0.063 - 0.070)	0.066 (0.063 - 0.069)	0.005 (0.005 - 0.005)	0.005 (0.005 - 0.005)	0.005 (0.005 - 0.005)	1.839 (1.234 - 2.553)	1.843 (1.232 - 2.543)	1.832 (1.226 - 2.528)
Total number of cases (thousands)	882 (841 - 928)	1,139 (1,088 - 1,195)	1,243 (1,185 - 1,304)	66 (62 - 70)	79 (74 - 84)	82 (77 - 87)	24 (16 - 34)	31 (21 - 43)	34 (23 - 47)
**Iceland**									
15 - 49 years (rate per capita)	0.021 (0.018 - 0.025)	0.023 (0.020 - 0.028)	0.023 (0.019 - 0.027)	0.002 (0.001 - 0.002)	0.002 (0.001 - 0.002)	0.002 (0.001 - 0.002)	0.610 (0.395 - 0.873)	0.674 (0.439 - 0.976)	0.652 (0.425 - 0.949)
50 - 69 years (rate per capita)	0.177 (0.147 - 0.209)	0.161 (0.134 - 0.189)	0.169 (0.139 - 0.198)	0.019 (0.015 - 0.023)	0.017 (0.013 - 0.021)	0.018 (0.014 - 0.022)	4.968 (3.125 - 7.192)	4.525 (2.836 - 6.585)	4.737 (2.959 - 6.884)
70+ years (rate per capita)	0.372 (0.318 - 0.433)	0.371 (0.317 - 0.432)	0.364 (0.311 - 0.423)	0.014 (0.010 - 0.019)	0.014 (0.010 - 0.018)	0.012 (0.009 - 0.017)	10.038 (6.431 - 14.219)	10.004 (6.412 - 14.149)	9.784 (6.262 - 13.774)
All ages (rate per capita)	0.066 (0.057 - 0.075)	0.074 (0.065 - 0.084)	0.083 (0.072 - 0.094)	0.005 (0.004 - 0.006)	0.005 (0.005 - 0.006)	0.006 (0.005 - 0.007)	1.818 (1.165 - 2.540)	2.052 (1.321 - 2.882)	2.287 (1.464 - 3.204)
Age-standardized (rate per capita)	0.065 (0.057 - 0.074)	0.065 (0.057 - 0.074)	0.065 (0.057 - 0.074)	0.005 (0.004 - 0.006)	0.005 (0.004 - 0.006)	0.005 (0.004 - 0.006)	1.814 (1.162 - 2.536)	1.813 (1.163 - 2.546)	1.812 (1.161 - 2.550)
Total number of cases (thousands)	17 (15 - 19)	22 (19 - 25)	27 (23 - 31)	1 (1 - 1)	2 (1 - 2)	2 (2 - 2)	0 (0 - 1)	1 (0 - 1)	1 (0 - 1)
**Ireland**									
15 - 49 years (rate per capita)	0.026 (0.025 - 0.028)	0.029 (0.027 - 0.031)	0.031 (0.029 - 0.033)	0.002 (0.002 - 0.002)	0.002 (0.002 - 0.002)	0.002 (0.002 - 0.002)	0.755 (0.495 - 1.055)	0.821 (0.543 - 1.150)	0.889 (0.592 - 1.249)
50 - 69 years (rate per capita)	0.208 (0.193 - 0.224)	0.193 (0.179 - 0.208)	0.198 (0.184 - 0.213)	0.022 (0.020 - 0.025)	0.021 (0.019 - 0.023)	0.022 (0.020 - 0.024)	5.818 (3.858 - 8.222)	5.415 (3.603 - 7.587)	5.565 (3.695 - 7.839)
70+ years (rate per capita)	0.446 (0.419 - 0.473)	0.439 (0.414 - 0.465)	0.434 (0.409 - 0.463)	0.017 (0.014 - 0.021)	0.016 (0.013 - 0.019)	0.016 (0.013 - 0.019)	12.055 (8.089 - 16.617)	11.853 (7.972 - 16.325)	11.713 (7.875 - 16.095)
All ages (rate per capita)	0.080 (0.077 - 0.085)	0.085 (0.081 - 0.089)	0.091 (0.087 - 0.095)	0.006 (0.005 - 0.006)	0.006 (0.006 - 0.007)	0.007 (0.006 - 0.007)	2.227 (1.490 - 3.092)	2.360 (1.591 - 3.259)	2.520 (1.682 - 3.504)
Age-standardized (rate per capita)	0.078 (0.074 - 0.082)	0.078 (0.074 - 0.082)	0.077 (0.074 - 0.081)	0.006 (0.005 - 0.006)	0.006 (0.005 - 0.006)	0.006 (0.005 - 0.006)	2.156 (1.442 - 2.993)	2.154 (1.452 - 2.979)	2.150 (1.436 - 2.985)
Total number of cases (thousands)	284 (270 - 298)	355 (339 - 373)	435 (414 - 457)	20 (19 - 22)	26 (24 - 28)	32 (30 - 34)	8 (5 - 11)	10 (7 - 14)	12 (8 - 17)
**Israel**									
15 - 49 years (rate per capita)	0.021 (0.019 - 0.022)	0.022 (0.020 - 0.024)	0.022 (0.021 - 0.024)	0.001 (0.001 - 0.002)	0.002 (0.001 - 0.002)	0.002 (0.001 - 0.002)	0.595 (0.390 - 0.835)	0.626 (0.417 - 0.876)	0.642 (0.425 - 0.907)
50 - 69 years (rate per capita)	0.171 (0.158 - 0.184)	0.156 (0.145 - 0.168)	0.172 (0.159 - 0.185)	0.019 (0.017 - 0.020)	0.017 (0.015 - 0.019)	0.019 (0.017 - 0.020)	4.788 (3.166 - 6.699)	4.367 (2.870 - 6.164)	4.796 (3.167 - 6.725)
70+ years (rate per capita)	0.380 (0.355 - 0.406)	0.375 (0.350 - 0.401)	0.368 (0.344 - 0.393)	0.015 (0.012 - 0.017)	0.014 (0.012 - 0.016)	0.013 (0.011 - 0.015)	10.263 (6.820 - 14.167)	10.090 (6.844 - 13.946)	9.873 (6.638 - 13.559)
All ages (rate per capita)	0.056 (0.053 - 0.059)	0.063 (0.060 - 0.067)	0.068 (0.065 - 0.072)	0.004 (0.004 - 0.004)	0.005 (0.004 - 0.005)	0.005 (0.005 - 0.005)	1.551 (1.032 - 2.154)	1.748 (1.174 - 2.424)	1.886 (1.262 - 2.609)
Age-standardized (rate per capita)	0.065 (0.062 - 0.069)	0.065 (0.062 - 0.069)	0.065 (0.062 - 0.069)	0.005 (0.005 - 0.005)	0.005 (0.005 - 0.005)	0.005 (0.005 - 0.005)	1.801 (1.198 - 2.505)	1.801 (1.207 - 2.501)	1.797 (1.201 - 2.482)
Total number of cases (thousands)	252 (239 - 265)	418 (396 - 441)	550 (522 - 581)	18 (17 - 20)	30 (28 - 32)	40 (38 - 43)	7 (5 - 10)	12 (8 - 16)	15 (10 - 21)
**Italy**									
15 - 49 years (rate per capita)	0.010 (0.010 - 0.011)	0.012 (0.011 - 0.013)	0.013 (0.012 - 0.014)	0.001 (0.001 - 0.001)	0.001 (0.001 - 0.001)	0.001 (0.001 - 0.001)	0.290 (0.192 - 0.405)	0.349 (0.231 - 0.487)	0.368 (0.243 - 0.519)
50 - 69 years (rate per capita)	0.083 (0.078 - 0.088)	0.085 (0.079 - 0.090)	0.082 (0.077 - 0.088)	0.009 (0.008 - 0.010)	0.009 (0.008 - 0.010)	0.009 (0.008 - 0.010)	2.309 (1.530 - 3.187)	2.362 (1.566 - 3.304)	2.303 (1.531 - 3.226)
70+ years (rate per capita)	0.197 (0.186 - 0.209)	0.196 (0.185 - 0.208)	0.190 (0.179 - 0.202)	0.007 (0.007 - 0.008)	0.008 (0.007 - 0.009)	0.007 (0.006 - 0.008)	5.279 (3.578 - 7.240)	5.269 (3.558 - 7.281)	5.100 (3.439 - 7.039)
All ages (rate per capita)	0.043 (0.041 - 0.046)	0.054 (0.051 - 0.056)	0.057 (0.054 - 0.060)	0.003 (0.003 - 0.003)	0.004 (0.003 - 0.004)	0.004 (0.004 - 0.004)	1.196 (0.803 - 1.642)	1.472 (0.986 - 2.033)	1.567 (1.053 - 2.160)
Age-standardized (rate per capita)	0.032 (0.030 - 0.033)	0.033 (0.031 - 0.034)	0.032 (0.031 - 0.034)	0.002 (0.002 - 0.003)	0.002 (0.002 - 0.003)	0.002 (0.002 - 0.003)	0.879 (0.591 - 1.207)	0.899 (0.601 - 1.239)	0.892 (0.596 - 1.238)
Total number of cases (thousands)	2,474 (2,361 - 2,602)	3,143 (2,990 - 3,302)	3,590 (3,416 - 3,796)	182 (172 - 193)	214 (201 - 227)	237 (223 - 252)	68 (46 - 93)	86 (58 - 119)	98 (66 - 136)
**Luxembourg**									
15 - 49 years (rate per capita)	0.027 (0.025 - 0.030)	0.029 (0.027 - 0.032)	0.029 (0.027 - 0.032)	0.002 (0.002 - 0.002)	0.002 (0.002 - 0.002)	0.002 (0.002 - 0.002)	0.771 (0.513 - 1.087)	0.845 (0.563 - 1.189)	0.828 (0.546 - 1.163)
50 - 69 years (rate per capita)	0.188 (0.174 - 0.203)	0.180 (0.166 - 0.193)	0.176 (0.162 - 0.190)	0.020 (0.018 - 0.023)	0.019 (0.018 - 0.021)	0.019 (0.017 - 0.021)	5.226 (3.475 - 7.358)	5.024 (3.331 - 7.012)	4.914 (3.266 - 6.912)
70+ years (rate per capita)	0.418 (0.390 - 0.447)	0.413 (0.386 - 0.440)	0.405 (0.378 - 0.434)	0.015 (0.013 - 0.018)	0.015 (0.013 - 0.018)	0.014 (0.012 - 0.017)	11.173 (7.539 - 15.346)	11.098 (7.507 - 15.243)	10.857 (7.334 - 14.996)
All ages (rate per capita)	0.092 (0.088 - 0.098)	0.095 (0.090 - 0.100)	0.095 (0.090 - 0.101)	0.007 (0.006 - 0.007)	0.007 (0.006 - 0.007)	0.007 (0.006 - 0.007)	2.544 (1.708 - 3.516)	2.612 (1.758 - 3.632)	2.628 (1.760 - 3.643)
Age-standardized (rate per capita)	0.072 (0.069 - 0.076)	0.072 (0.068 - 0.076)	0.072 (0.068 - 0.076)	0.005 (0.005 - 0.006)	0.005 (0.005 - 0.006)	0.005 (0.005 - 0.006)	1.993 (1.338 - 2.757)	1.989 (1.333 - 2.770)	1.989 (1.332 - 2.758)
Total number of cases (thousands)	35 (33 - 37)	43 (41 - 46)	53 (50 - 56)	3 (2 - 3)	3 (3 - 3)	4 (3 - 4)	1 (1 - 1)	1 (1 - 2)	1 (1 - 2)
**Malta**									
15 - 49 years (rate per capita)	0.022 (0.019 - 0.027)	0.024 (0.020 - 0.028)	0.024 (0.020 - 0.028)	0.002 (0.001 - 0.002)	0.002 (0.001 - 0.002)	0.002 (0.001 - 0.002)	0.640 (0.414 - 0.920)	0.683 (0.443 - 0.986)	0.677 (0.440 - 0.982)
50 - 69 years (rate per capita)	0.169 (0.140 - 0.199)	0.170 (0.141 - 0.199)	0.182 (0.150 - 0.215)	0.018 (0.014 - 0.023)	0.018 (0.014 - 0.023)	0.019 (0.015 - 0.024)	4.732 (2.964 - 6.958)	4.752 (2.990 - 6.969)	5.085 (3.200 - 7.486)
70+ years (rate per capita)	0.377 (0.322 - 0.442)	0.377 (0.322 - 0.442)	0.378 (0.323 - 0.443)	0.015 (0.011 - 0.020)	0.014 (0.011 - 0.019)	0.015 (0.011 - 0.020)	10.166 (6.474 - 14.418)	10.177 (6.512 - 14.393)	10.203 (6.530 - 14.434)
All ages (rate per capita)	0.066 (0.058 - 0.075)	0.087 (0.076 - 0.099)	0.106 (0.092 - 0.123)	0.005 (0.004 - 0.006)	0.006 (0.005 - 0.008)	0.008 (0.007 - 0.009)	1.824 (1.164 - 2.583)	2.398 (1.531 - 3.380)	2.935 (1.875 - 4.172)
Age-standardized (rate per capita)	0.065 (0.057 - 0.074)	0.065 (0.057 - 0.075)	0.065 (0.057 - 0.074)	0.005 (0.004 - 0.006)	0.005 (0.004 - 0.006)	0.005 (0.004 - 0.006)	1.808 (1.153 - 2.554)	1.816 (1.159 - 2.558)	1.807 (1.158 - 2.555)
Total number of cases (thousands)	23 (20 - 27)	34 (30 - 39)	44 (39 - 51)	2 (1 - 2)	3 (2 - 3)	3 (3 - 4)	1 (0 - 1)	1 (1 - 1)	1 (1 - 2)
**Netherlands**									
15 - 49 years (rate per capita)	0.031 (0.030 - 0.033)	0.034 (0.033 - 0.037)	0.034 (0.032 - 0.036)	0.002 (0.002 - 0.002)	0.002 (0.002 - 0.003)	0.002 (0.002 - 0.003)	0.902 (0.600 - 1.262)	0.986 (0.657 - 1.379)	0.980 (0.653 - 1.373)
50 - 69 years (rate per capita)	0.227 (0.214 - 0.241)	0.215 (0.203 - 0.228)	0.226 (0.213 - 0.240)	0.025 (0.023 - 0.027)	0.024 (0.022 - 0.026)	0.025 (0.023 - 0.027)	6.346 (4.216 - 8.797)	6.026 (4.028 - 8.366)	6.314 (4.195 - 8.815)
70+ years (rate per capita)	0.481 (0.459 - 0.504)	0.476 (0.454 - 0.499)	0.472 (0.450 - 0.495)	0.017 (0.014 - 0.020)	0.017 (0.014 - 0.020)	0.016 (0.014 - 0.019)	12.936 (8.770 - 17.756)	12.800 (8.638 - 17.487)	12.690 (8.663 - 17.433)
All ages (rate per capita)	0.101 (0.098 - 0.106)	0.114 (0.110 - 0.118)	0.129 (0.124 - 0.134)	0.007 (0.007 - 0.008)	0.008 (0.008 - 0.009)	0.010 (0.009 - 0.010)	2.805 (1.889 - 3.864)	3.147 (2.118 - 4.329)	3.554 (2.394 - 4.908)
Age-standardized (rate per capita)	0.085 (0.082 - 0.089)	0.085 (0.082 - 0.089)	0.085 (0.082 - 0.088)	0.006 (0.006 - 0.007)	0.006 (0.006 - 0.007)	0.006 (0.006 - 0.007)	2.365 (1.591 - 3.255)	2.364 (1.593 - 3.255)	2.352 (1.585 - 3.246)
Total number of cases (thousands)	1,516 (1,460 - 1,576)	1,857 (1,788 - 1,930)	2,213 (2,130 - 2,302)	109 (103 - 114)	134 (127 - 141)	165 (156 - 173)	42 (28 - 58)	51 (35 - 71)	61 (41 - 84)
**Norway**									
15 - 49 years (rate per capita)	0.023 (0.022 - 0.025)	0.025 (0.024 - 0.027)	0.025 (0.023 - 0.026)	0.002 (0.001 - 0.002)	0.002 (0.002 - 0.002)	0.002 (0.002 - 0.002)	0.661 (0.443 - 0.932)	0.716 (0.470 - 0.999)	0.709 (0.472 - 0.990)
50 - 69 years (rate per capita)	0.183 (0.172 - 0.195)	0.162 (0.153 - 0.172)	0.175 (0.164 - 0.186)	0.020 (0.018 - 0.021)	0.018 (0.016 - 0.019)	0.019 (0.018 - 0.021)	5.101 (3.403 - 7.055)	4.537 (3.017 - 6.282)	4.890 (3.259 - 6.795)
70+ years (rate per capita)	0.386 (0.367 - 0.406)	0.376 (0.358 - 0.395)	0.375 (0.357 - 0.395)	0.015 (0.013 - 0.017)	0.013 (0.011 - 0.015)	0.013 (0.011 - 0.015)	10.406 (7.062 - 14.233)	10.092 (6.839 - 13.796)	10.074 (6.839 - 13.787)
All ages (rate per capita)	0.090 (0.086 - 0.094)	0.088 (0.085 - 0.092)	0.093 (0.089 - 0.097)	0.006 (0.006 - 0.007)	0.006 (0.006 - 0.006)	0.007 (0.006 - 0.007)	2.469 (1.674 - 3.387)	2.434 (1.640 - 3.358)	2.555 (1.726 - 3.526)

	Prevalence 1990	Prevalence 2005	Prevalence 2015	Incidence 1990	Incidence 2005	Incidence 2015	YLD rate 1990	YLD rate 2005	YLD rate 2015
**Age-standardized (rate per capita)**	0.066 (0.063 - 0.069)	0.066 (0.063 - 0.069)	0.066 (0.064 - 0.069)	0.005 (0.005 - 0.005)	0.005 (0.005 - 0.005)	0.005 (0.005 - 0.005)	1.825 (1.230 - 2.502)	1.826 (1.231 - 2.523)	1.836 (1.239 - 2.535)
**Total number of cases (thousands)**	380 (364 - 397)	409 (393 - 426)	478 (458 - 499)	26 (25 - 28)	28 (27 - 29)	35 (33 - 37)	10 (7 - 14)	11 (8 - 16)	13 (9 - 18)
**Portugal**									
**15 - 49 years (rate per capita)**	0.020 (0.018 - 0.021)	0.022 (0.020 - 0.023)	0.023 (0.022 - 0.025)	0.001 (0.001 - 0.002)	0.001 (0.001 - 0.002)	0.002 (0.001 - 0.002)	0.562 (0.371 - 0.791)	0.624 (0.415 - 0.875)	0.665 (0.438 - 0.935)
**50 - 69 years (rate per capita)**	0.154 (0.142 - 0.166)	0.152 (0.142 - 0.164)	0.150 (0.139 - 0.162)	0.017 (0.015 - 0.018)	0.017 (0.015 - 0.018)	0.016 (0.015 - 0.018)	4.277 (2.843 - 5.960)	4.249 (2.819 - 5.979)	4.179 (2.786 - 5.900)
**70+ years (rate per capita)**	0.349 (0.325 - 0.371)	0.348 (0.325 - 0.372)	0.343 (0.320 - 0.366)	0.014 (0.011 - 0.016)	0.013 (0.011 - 0.016)	0.013 (0.011 - 0.015)	9.341 (6.348 - 12.893)	9.338 (6.319 - 12.925)	9.215 (6.239 - 12.658)
**All ages (rate per capita)**	0.073 (0.070 - 0.078)	0.087 (0.083 - 0.092)	0.096 (0.091 - 0.101)	0.005 (0.005 - 0.006)	0.006 (0.006 - 0.007)	0.006 (0.006 - 0.007)	2.020 (1.359 - 2.800)	2.396 (1.616 - 3.308)	2.631 (1.775 - 3.650)
**Age-standardized (rate per capita)**	0.059 (0.056 - 0.062)	0.059 (0.056 - 0.062)	0.059 (0.056 - 0.062)	0.004 (0.004 - 0.005)	0.004 (0.004 - 0.005)	0.004 (0.004 - 0.005)	1.619 (1.088 - 2.245)	1.622 (1.090 - 2.247)	1.621 (1.089 - 2.248)
**Total number of cases (thousands)**	731 (692 - 773)	920 (873 - 972)	1,035 (979 - 1,093)	54 (50 - 58)	65 (60 - 69)	70 (65 - 75)	20 (14 - 28)	25 (17 - 35)	28 (19 - 39)
**Spain**									
**15 - 49 years (rate per capita)**	0.018 (0.016 - 0.019)	0.020 (0.019 - 0.021)	0.022 (0.020 - 0.023)	0.001 (0.001 - 0.001)	0.001 (0.001 - 0.001)	0.001 (0.001 - 0.002)	0.504 (0.335 - 0.712)	0.572 (0.375 - 0.800)	0.624 (0.414 - 0.880)
**50 - 69 years (rate per capita)**	0.142 (0.133 - 0.151)	0.134 (0.126 - 0.143)	0.134 (0.125 - 0.143)	0.015 (0.014 - 0.017)	0.014 (0.013 - 0.016)	0.014 (0.013 - 0.016)	3.976 (2.648 - 5.521)	3.769 (2.506 - 5.261)	3.765 (2.486 - 5.267)
**70+ years (rate per capita)**	0.316 (0.299 - 0.334)	0.309 (0.292 - 0.327)	0.303 (0.286 - 0.320)	0.012 (0.010 - 0.014)	0.012 (0.010 - 0.014)	0.010 (0.009 - 0.012)	8.506 (5.747 - 11.659)	8.330 (5.644 - 11.407)	8.167 (5.512 - 11.154)
**All ages (rate per capita)**	0.067 (0.064 - 0.070)	0.077 (0.074 - 0.080)	0.080 (0.077 - 0.084)	0.005 (0.005 - 0.005)	0.005 (0.005 - 0.005)	0.005 (0.005 - 0.006)	1.849 (1.247 - 2.553)	2.119 (1.425 - 2.928)	2.220 (1.494 - 3.058)
**Age-standardized (rate per capita)**	0.054 (0.051 - 0.056)	0.053 (0.051 - 0.055)	0.053 (0.051 - 0.055)	0.004 (0.004 - 0.004)	0.004 (0.004 - 0.004)	0.004 (0.004 - 0.004)	1.483 (0.998 - 2.043)	1.462 (0.980 - 2.021)	1.475 (0.988 - 2.038)
**Total number of cases (thousands)**	2,608 (2,494 - 2,736)	3,328 (3,189 - 3,479)	3,912 (3,745 - 4,097)	190 (180 - 201)	224 (212 - 236)	259 (245 - 273)	72 (49 - 99)	92 (62 - 127)	108 (73 - 149)
**Sweden**									
**15 - 49 years (rate per capita)**	0.015 (0.014 - 0.017)	0.016 (0.014 - 0.017)	0.016 (0.015 - 0.017)	0.001 (0.001 - 0.001)	0.001 (0.001 - 0.001)	0.001 (0.001 - 0.001)	0.443 (0.288 - 0.624)	0.450 (0.296 - 0.636)	0.458 (0.302 - 0.645)
**50 - 69 years (rate per capita)**	0.118 (0.106 - 0.130)	0.109 (0.098 - 0.122)	0.121 (0.110 - 0.135)	0.012 (0.010 - 0.014)	0.012 (0.010 - 0.013)	0.012 (0.011 - 0.014)	3.290 (2.139 - 4.677)	3.059 (2.007 - 4.355)	3.395 (2.208 - 4.792)
**70+ years (rate per capita)**	0.242 (0.220 - 0.266)	0.232 (0.212 - 0.254)	0.233 (0.213 - 0.257)	0.009 (0.007 - 0.012)	0.008 (0.006 - 0.011)	0.009 (0.007 - 0.011)	6.534 (4.389 - 9.131)	6.230 (4.130 - 8.709)	6.279 (4.185 - 8.769)
**All ages (rate per capita)**	0.062 (0.058 - 0.067)	0.063 (0.059 - 0.068)	0.067 (0.062 - 0.073)	0.004 (0.004 - 0.005)	0.004 (0.004 - 0.005)	0.005 (0.004 - 0.005)	1.711 (1.129 - 2.389)	1.739 (1.148 - 2.439)	1.854 (1.228 - 2.600)
**Age-standardized (rate per capita)**	0.042 (0.039 - 0.045)	0.042 (0.039 - 0.045)	0.042 (0.039 - 0.045)	0.003 (0.003 - 0.003)	0.003 (0.003 - 0.003)	0.003 (0.003 - 0.003)	1.171 (0.769 - 1.640)	1.158 (0.763 - 1.634)	1.169 (0.772 - 1.643)
**Total number of cases (thousands)**	530 (493 - 572)	569 (529 - 611)	659 (613 - 712)	36 (33 - 40)	39 (36 - 43)	47 (42 - 51)	15 (10 - 20)	16 (10 - 22)	18 (12 - 25)
**Switzerland**									
**15 - 49 years (rate per capita)**	0.027 (0.025 - 0.029)	0.028 (0.026 - 0.031)	0.029 (0.027 - 0.031)	0.002 (0.002 - 0.002)	0.002 (0.002 - 0.002)	0.002 (0.002 - 0.002)	0.768 (0.508 - 1.079)	0.813 (0.537 - 1.144)	0.827 (0.545 - 1.161)
**50 - 69 years (rate per capita)**	0.188 (0.174 - 0.203)	0.185 (0.171 - 0.201)	0.188 (0.175 - 0.204)	0.020 (0.018 - 0.022)	0.020 (0.018 - 0.022)	0.020 (0.018 - 0.023)	5.263 (3.483 - 7.381)	5.170 (3.412 - 7.234)	5.277 (3.496 - 7.349)
**70+ years (rate per capita)**	0.409 (0.381 - 0.437)	0.409 (0.383 - 0.439)	0.405 (0.378 - 0.434)	0.014 (0.012 - 0.017)	0.014 (0.012 - 0.017)	0.014 (0.012 - 0.016)	10.963 (7.357 - 15.122)	11.012 (7.384 - 15.171)	10.906 (7.276 - 15.083)
**All ages (rate per capita)**	0.094 (0.089 - 0.100)	0.103 (0.098 - 0.110)	0.111 (0.104 - 0.118)	0.007 (0.006 - 0.007)	0.007 (0.007 - 0.008)	0.008 (0.007 - 0.008)	2.596 (1.735 - 3.567)	2.852 (1.905 - 3.952)	3.057 (2.043 - 4.255)
**Age-standardized (rate per capita)**	0.072 (0.068 - 0.076)	0.072 (0.068 - 0.076)	0.072 (0.068 - 0.076)	0.005 (0.005 - 0.006)	0.005 (0.005 - 0.006)	0.005 (0.005 - 0.006)	1.985 (1.327 - 2.732)	2.001 (1.331 - 2.771)	2.001 (1.333 - 2.779)
**Total number of cases (thousands)**	632 (600 - 669)	768 (725 - 816)	915 (864 - 974)	44 (41 - 48)	54 (50 - 58)	64 (60 - 70)	17 (12 - 24)	21 (14 - 29)	25 (17 - 35)
**United Kingdom**									
**15 - 49 years (rate per capita)**	0.017 (0.016 - 0.018)	0.017 (0.017 - 0.018)	0.018 (0.017 - 0.019)	0.001 (0.001 - 0.001)	0.001 (0.001 - 0.001)	0.001 (0.001 - 0.001)	0.484 (0.321 - 0.678)	0.500 (0.332 - 0.698)	0.523 (0.348 - 0.731)
**50 - 69 years (rate per capita)**	0.128 (0.123 - 0.134)	0.118 (0.113 - 0.123)	0.125 (0.119 - 0.130)	0.014 (0.013 - 0.015)	0.013 (0.012 - 0.014)	0.014 (0.013 - 0.014)	3.593 (2.411 - 4.984)	3.303 (2.214 - 4.567)	3.510 (2.352 - 4.871)
**70+ years (rate per capita)**	0.286 (0.273 - 0.300)	0.278 (0.265 - 0.292)	0.277 (0.265 - 0.291)	0.011 (0.010 - 0.012)	0.011 (0.010 - 0.012)	0.010 (0.009 - 0.012)	7.766 (5.265 - 10.659)	7.530 (5.101 - 10.346)	7.517 (5.098 - 10.300)
**All ages (rate per capita)**	0.065 (0.063 - 0.068)	0.067 (0.064 - 0.069)	0.072 (0.070 - 0.075)	0.005 (0.004 - 0.005)	0.005 (0.004 - 0.005)	0.005 (0.005 - 0.005)	1.812 (1.221 - 2.505)	1.842 (1.245 - 2.555)	2.001 (1.349 - 2.759)
**Age-standardized (rate per capita)**	0.048 (0.047 - 0.050)	0.047 (0.045 - 0.049)	0.048 (0.046 - 0.050)	0.004 (0.003 - 0.004)	0.004 (0.003 - 0.004)	0.004 (0.003 - 0.004)	1.339 (0.901 - 1.848)	1.306 (0.880 - 1.808)	1.334 (0.897 - 1.842)
**Total number of cases (thousands)**	3,742 (3,612 - 3,887)	4,008 (3,871 - 4,163)	4,639 (4,480 - 4,821)	263 (251 - 276)	280 (267 - 293)	328 (313 - 344)	104 (70 - 143)	111 (75 - 154)	129 (87 - 177)
**England**									
**15 - 49 years (rate per capita)**	0.017 (0.016 - 0.018)	0.017 (0.016 - 0.018)	0.018 (0.017 - 0.019)	0.001 (0.001 - 0.001)	0.001 (0.001 - 0.001)	0.001 (0.001 - 0.001)	0.485 (0.321 - 0.681)	0.500 (0.332 - 0.698)	0.523 (0.348 - 0.732)
**50 - 69 years (rate per capita)**	0.128 (0.123 - 0.134)	0.118 (0.113 - 0.123)	0.125 (0.120 - 0.131)	0.014 (0.013 - 0.015)	0.013 (0.012 - 0.014)	0.014 (0.013 - 0.014)	3.599 (2.414 - 4.995)	3.306 (2.212 - 4.569)	3.521 (2.359 - 4.887)
**70+ years (rate per capita)**	0.286 (0.273 - 0.300)	0.278 (0.264 - 0.291)	0.277 (0.264 - 0.291)	0.011 (0.010 - 0.012)	0.011 (0.010 - 0.012)	0.010 (0.009 - 0.012)	7.771 (5.267 - 10.658)	7.534 (5.099 - 10.361)	7.519 (5.098 - 10.300)
**All ages (rate per capita)**	0.066 (0.063 - 0.068)	0.066 (0.064 - 0.069)	0.072 (0.069 - 0.075)	0.005 (0.004 - 0.005)	0.005 (0.004 - 0.005)	0.005 (0.005 - 0.005)	1.821 (1.226 - 2.514)	1.838 (1.241 - 2.552)	1.990 (1.341 - 2.740)
**Age-standardized (rate per capita)**	0.048 (0.047 - 0.050)	0.047 (0.045 - 0.049)	0.048 (0.046 - 0.050)	0.004 (0.003 - 0.004)	0.004 (0.003 - 0.004)	0.004 (0.003 - 0.004)	1.340 (0.902 - 1.848)	1.308 (0.881 - 1.813)	1.336 (0.898 - 1.841)
**Total number of cases (thousands)**	3,131 (3,022 - 3,256)	3,347 (3,233 - 3,477)	3,874 (3,741 - 4,024)	220 (209 - 230)	233 (222 - 244)	273 (261 - 287)	87 (58 - 120)	93 (63 - 129)	107 (72 - 148)
**Northern Ireland**									
**15 - 49 years (rate per capita)**	0.016 (0.015 - 0.017)	0.017 (0.016 - 0.018)	0.018 (0.017 - 0.019)	0.001 (0.001 - 0.001)	0.001 (0.001 - 0.001)	0.001 (0.001 - 0.001)	0.459 (0.303 - 0.642)	0.482 (0.318 - 0.674)	0.518 (0.340 - 0.729)
**50 - 69 years (rate per capita)**	0.126 (0.120 - 0.133)	0.117 (0.111 - 0.123)	0.121 (0.115 - 0.127)	0.014 (0.013 - 0.015)	0.013 (0.012 - 0.014)	0.013 (0.012 - 0.014)	3.548 (2.354 - 4.935)	3.278 (2.204 - 4.533)	3.405 (2.267 - 4.764)
**70+ years (rate per capita)**	0.286 (0.271 - 0.302)	0.279 (0.265 - 0.295)	0.279 (0.264 - 0.294)	0.011 (0.010 - 0.013)	0.011 (0.010 - 0.012)	0.011 (0.010 - 0.012)	7.773 (5.224 - 10.712)	7.565 (5.130 - 10.426)	7.546 (5.098 - 10.420)
**All ages (rate per capita)**	0.055 (0.053 - 0.057)	0.059 (0.057 - 0.061)	0.067 (0.064 - 0.069)	0.004 (0.004 - 0.004)	0.004 (0.004 - 0.004)	0.005 (0.005 - 0.005)	1.534 (1.032 - 2.137)	1.633 (1.102 - 2.250)	1.846 (1.242 - 2.573)
**Age-standardized (rate per capita)**	0.048 (0.046 - 0.050)	0.047 (0.045 - 0.049)	0.048 (0.046 - 0.050)	0.004 (0.003 - 0.004)	0.004 (0.003 - 0.004)	0.004 (0.003 - 0.004)	1.337 (0.899 - 1.868)	1.302 (0.878 - 1.796)	1.329 (0.893 - 1.854)
**Total number of cases (thousands)**	88 (85 - 92)	102 (98 - 106)	122 (117 - 127)	6 (6 - 7)	7 (7 - 8)	9 (8 - 9)	2 (2 - 3)	3 (2 - 4)	3 (2 - 5)
**Scotland**									
**15 - 49 years (rate per capita)**	0.017 (0.016 - 0.018)	0.018 (0.017 - 0.019)	0.018 (0.017 - 0.019)	0.001 (0.001 - 0.001)	0.001 (0.001 - 0.001)	0.001 (0.001 - 0.001)	0.477 (0.313 - 0.665)	0.507 (0.335 - 0.709)	0.526 (0.347 - 0.741)
**50 - 69 years (rate per capita)**	0.126 (0.120 - 0.132)	0.117 (0.112 - 0.123)	0.122 (0.116 - 0.128)	0.014 (0.013 - 0.015)	0.013 (0.012 - 0.014)	0.013 (0.012 - 0.014)	3.527 (2.354 - 4.873)	3.275 (2.205 - 4.542)	3.411 (2.281 - 4.734)
**70+ years (rate per capita)**	0.286 (0.271 - 0.301)	0.279 (0.264 - 0.295)	0.279 (0.265 - 0.294)	0.011 (0.010 - 0.013)	0.011 (0.010 - 0.012)	0.011 (0.010 - 0.012)	7.733 (5.232 - 10.629)	7.519 (5.117 - 10.294)	7.511 (5.096 - 10.336)
**All ages (rate per capita)**	0.064 (0.061 - 0.066)	0.068 (0.066 - 0.071)	0.075 (0.072 - 0.078)	0.005 (0.004 - 0.005)	0.005 (0.005 - 0.005)	0.005 (0.005 - 0.006)	1.765 (1.185 - 2.434)	1.883 (1.266 - 2.608)	2.073 (1.397 - 2.854)
**Age-standardized (rate per capita)**	0.048 (0.046 - 0.050)	0.047 (0.045 - 0.049)	0.048 (0.046 - 0.049)	0.004 (0.003 - 0.004)	0.004 (0.003 - 0.004)	0.004 (0.003 - 0.004)	1.329 (0.891 - 1.833)	1.295 (0.871 - 1.798)	1.321 (0.889 - 1.820)
**Total number of cases (thousands)**	325 (312 - 337)	347 (334 - 362)	400 (385 - 417)	23 (22 - 24)	25 (23 - 26)	29 (27 - 30)	9 (6 - 12)	10 (6 - 13)	11 (7 - 15)
**Wales**									
**15 - 49 years (rate per capita)**	0.017 (0.016 - 0.018)	0.017 (0.016 - 0.018)	0.018 (0.017 - 0.019)	0.001 (0.001 - 0.001)	0.001 (0.001 - 0.001)	0.001 (0.001 - 0.001)	0.488 (0.323 - 0.691)	0.495 (0.325 - 0.694)	0.513 (0.338 - 0.725)
**50 - 69 years (rate per capita)**	0.130 (0.124 - 0.136)	0.118 (0.113 - 0.124)	0.127 (0.121 - 0.133)	0.014 (0.013 - 0.015)	0.013 (0.012 - 0.014)	0.014 (0.013 - 0.015)	3.634 (2.432 - 5.031)	3.315 (2.206 - 4.597)	3.561 (2.373 - 4.930)
**70+ years (rate per capita)**	0.286 (0.271 - 0.303)	0.277 (0.263 - 0.293)	0.277 (0.263 - 0.293)	0.011 (0.010 - 0.013)	0.011 (0.010 - 0.012)	0.011 (0.009 - 0.012)	7.734 (5.236 - 10.664)	7.474 (5.053 - 10.245)	7.472 (5.059 - 10.330)
**All ages (rate per capita)**	0.069 (0.067 - 0.072)	0.071 (0.069 - 0.075)	0.079 (0.076 - 0.082)	0.005 (0.005 - 0.005)	0.005 (0.005 - 0.005)	0.006 (0.005 - 0.006)	1.913 (1.293 - 2.661)	1.970 (1.322 - 2.696)	2.171 (1.466 - 3.023)
**Age-standardized (rate per capita)**	0.048 (0.046 - 0.050)	0.047 (0.045 - 0.049)	0.048 (0.046 - 0.050)	0.004 (0.003 - 0.004)	0.004 (0.003 - 0.004)	0.004 (0.003 - 0.004)	1.332 (0.900 - 1.856)	1.298 (0.873 - 1.784)	1.325 (0.891 - 1.841)
**Total number of cases (thousands)**	198 (191 - 206)	212 (204 - 221)	242 (233 - 252)	14 (13 - 15)	15 (14 - 16)	17 (16 - 18)	5 (4 - 8)	6 (4 - 8)	7 (5 - 9)
**Southern Latin America**									
**15 - 49 years (rate per capita)**	0.015 (0.014 - 0.016)	0.015 (0.014 - 0.016)	0.016 (0.015 - 0.017)	0.001 (0.001 - 0.001)	0.001 (0.001 - 0.001)	0.001 (0.001 - 0.001)	0.421 (0.279 - 0.590)	0.436 (0.287 - 0.616)	0.449 (0.296 - 0.630)
**50 - 69 years (rate per capita)**	0.116 (0.110 - 0.123)	0.113 (0.107 - 0.120)	0.115 (0.108 - 0.122)	0.012 (0.011 - 0.013)	0.012 (0.011 - 0.013)	0.012 (0.011 - 0.013)	3.265 (2.168 - 4.544)	3.174 (2.098 - 4.393)	3.224 (2.131 - 4.466)
**70+ years (rate per capita)**	0.253 (0.236 - 0.270)	0.250 (0.233 - 0.267)	0.246 (0.229 - 0.263)	0.010 (0.008 - 0.011)	0.009 (0.008 - 0.011)	0.009 (0.007 - 0.010)	6.873 (4.624 - 9.571)	6.775 (4.561 - 9.424)	6.673 (4.495 - 9.267)
**All ages (rate per capita)**	0.039 (0.037 - 0.041)	0.043 (0.041 - 0.045)	0.048 (0.045 - 0.050)	0.003 (0.003 - 0.003)	0.003 (0.003 - 0.003)	0.003 (0.003 - 0.004)	1.085 (0.724 - 1.501)	1.206 (0.805 - 1.669)	1.326 (0.884 - 1.830)
**Age-standardized (rate per capita)**	0.044 (0.042 - 0.046)	0.044 (0.042 - 0.046)	0.044 (0.042 - 0.046)	0.003 (0.003 - 0.003)	0.003 (0.003 - 0.003)	0.003 (0.003 - 0.003)	1.235 (0.824 - 1.707)	1.235 (0.824 - 1.711)	1.236 (0.823 - 1.705)
**Total number of cases (thousands)**	1,906 (1,820 - 1,996)	2,535 (2,420 - 2,657)	3,087 (2,944 - 3,236)	142 (134 - 151)	182 (171 - 193)	221 (208 - 235)	53 (35 - 74)	71 (47 - 98)	86 (57 - 119)
**Argentina**									
**15 - 49 years (rate per capita)**	0.015 (0.014 - 0.016)	0.015 (0.014 - 0.016)	0.015 (0.014 - 0.016)	0.001 (0.001 - 0.001)	0.001 (0.001 - 0.001)	0.001 (0.001 - 0.001)	0.426 (0.282 - 0.598)	0.426 (0.279 - 0.603)	0.440 (0.290 - 0.619)
**50 - 69 years (rate per capita)**	0.117 (0.110 - 0.124)	0.114 (0.107 - 0.121)	0.116 (0.109 - 0.123)	0.012 (0.011 - 0.013)	0.012 (0.011 - 0.013)	0.012 (0.011 - 0.013)	3.288 (2.187 - 4.591)	3.195 (2.112 - 4.433)	3.261 (2.151 - 4.532)

	Prevalence 1990	Prevalence 2005	Prevalence 2015	Incidence 1990	Incidence 2005	Incidence 2015	YLD rate 1990	YLD rate 2005	YLD rate 2015
70+ years (rate per capita)	0.254 (0.236 - 0.272)	0.250 (0.232 - 0.268)	0.246 (0.229 - 0.264)	0.010 (0.008 - 0.011)	0.009 (0.008 - 0.011)	0.009 (0.007 - 0.010)	6.904 (4.652 - 9.605)	6.787 (4.576 - 9.460)	6.691 (4.504 - 9.279)
All ages (rate per capita)	0.041 (0.039 - 0.043)	0.043 (0.041 - 0.046)	0.046 (0.044 - 0.048)	0.003 (0.003 - 0.003)	0.003 (0.003 - 0.003)	0.003 (0.003 - 0.003)	1.131 (0.753 - 1.567)	1.209 (0.808 - 1.681)	1.285 (0.857 - 1.784)
Age-standardized (rate per capita)	0.044 (0.042 - 0.047)	0.044 (0.042 - 0.047)	0.044 (0.042 - 0.047)	0.003 (0.003 - 0.003)	0.003 (0.003 - 0.003)	0.003 (0.003 - 0.003)	1.235 (0.823 - 1.712)	1.235 (0.825 - 1.716)	1.235 (0.824 - 1.715)
Total number of cases (thousands)	1,327 (1,264 - 1,393)	1,699 (1,618 - 1,784)	2,004 (1,907 - 2,104)	99 (93 - 106)	121 (114 - 129)	143 (134 - 152)	37 (25 - 51)	47 (32 - 66)	56 (37 - 77)
**Chile**									
15 - 49 years (rate per capita)	0.014 (0.013 - 0.015)	0.016 (0.015 - 0.017)	0.016 (0.015 - 0.018)	0.001 (0.001 - 0.001)	0.001 (0.001 - 0.001)	0.001 (0.001 - 0.001)	0.408 (0.269 - 0.576)	0.456 (0.301 - 0.644)	0.470 (0.310 - 0.661)
50 - 69 years (rate per capita)	0.113 (0.106 - 0.121)	0.110 (0.103 - 0.117)	0.112 (0.105 - 0.119)	0.012 (0.011 - 0.013)	0.011 (0.010 - 0.012)	0.011 (0.010 - 0.013)	3.175 (2.099 - 4.406)	3.097 (2.048 - 4.289)	3.142 (2.085 - 4.377)
70+ years (rate per capita)	0.251 (0.232 - 0.270)	0.249 (0.230 - 0.268)	0.246 (0.227 - 0.264)	0.009 (0.008 - 0.011)	0.009 (0.008 - 0.011)	0.009 (0.007 - 0.010)	6.800 (4.513 - 9.444)	6.751 (4.489 - 9.364)	6.663 (4.423 - 9.223)
All ages (rate per capita)	0.032 (0.031 - 0.034)	0.041 (0.039 - 0.043)	0.050 (0.047 - 0.052)	0.002 (0.002 - 0.003)	0.003 (0.003 - 0.003)	0.004 (0.003 - 0.004)	0.907 (0.607 - 1.257)	1.144 (0.762 - 1.586)	1.384 (0.922 - 1.918)
Age-standardized (rate per capita)	0.044 (0.042 - 0.047)	0.044 (0.042 - 0.047)	0.044 (0.042 - 0.047)	0.003 (0.003 - 0.003)	0.003 (0.003 - 0.003)	0.003 (0.003 - 0.003)	1.235 (0.827 - 1.710)	1.236 (0.824 - 1.718)	1.237 (0.823 - 1.718)
Total number of cases (thousands)	427 (407 - 449)	660 (629 - 695)	891 (847 - 938)	32 (30 - 34)	49 (46 - 52)	65 (61 - 70)	12 (8 - 17)	18 (12 - 26)	25 (17 - 34)
**Uruguay**									
15 - 49 years (rate per capita)	0.015 (0.014 - 0.016)	0.015 (0.015 - 0.016)	0.016 (0.015 - 0.016)	0.001 (0.001 - 0.001)	0.001 (0.001 - 0.001)	0.001 (0.001 - 0.001)	0.430 (0.284 - 0.607)	0.446 (0.295 - 0.627)	0.449 (0.295 - 0.633)
50 - 69 years (rate per capita)	0.118 (0.112 - 0.125)	0.117 (0.111 - 0.123)	0.116 (0.110 - 0.122)	0.012 (0.011 - 0.013)	0.012 (0.011 - 0.013)	0.012 (0.011 - 0.013)	3.318 (2.198 - 4.575)	3.282 (2.189 - 4.532)	3.250 (2.162 - 4.501)
70+ years (rate per capita)	0.251 (0.239 - 0.264)	0.249 (0.237 - 0.261)	0.242 (0.231 - 0.255)	0.009 (0.008 - 0.011)	0.009 (0.008 - 0.010)	0.008 (0.007 - 0.009)	6.798 (4.568 - 9.379)	6.736 (4.525 - 9.269)	6.553 (4.419 - 9.030)
All ages (rate per capita)	0.049 (0.047 - 0.051)	0.053 (0.051 - 0.055)	0.056 (0.054 - 0.058)	0.004 (0.003 - 0.004)	0.004 (0.003 - 0.004)	0.004 (0.004 - 0.004)	1.359 (0.909 - 1.878)	1.467 (0.984 - 2.020)	1.548 (1.040 - 2.152)
Age-standardized (rate per capita)	0.044 (0.043 - 0.046)	0.044 (0.043 - 0.046)	0.044 (0.043 - 0.046)	0.003 (0.003 - 0.003)	0.003 (0.003 - 0.003)	0.003 (0.003 - 0.003)	1.229 (0.822 - 1.700)	1.230 (0.826 - 1.697)	1.230 (0.825 - 1.712)
Total number of cases (thousands)	152 (146 - 158)	176 (170 - 183)	192 (184 - 199)	11 (11 - 12)	12 (12 - 13)	13 (12 - 14)	4 (3 - 6)	5 (3 - 7)	5 (4 - 7)
**Central Europe, Eastern Europe, and Central Asia**									
15 - 49 years (rate per capita)	0.020 (0.019 - 0.022)	0.021 (0.020 - 0.023)	0.022 (0.021 - 0.024)	0.002 (0.001 - 0.002)	0.002 (0.002 - 0.002)	0.002 (0.002 - 0.002)	0.578 (0.381 - 0.813)	0.617 (0.405 - 0.868)	0.638 (0.421 - 0.906)
50 - 69 years (rate per capita)	0.150 (0.139 - 0.162)	0.150 (0.139 - 0.162)	0.144 (0.133 - 0.156)	0.015 (0.014 - 0.017)	0.015 (0.014 - 0.017)	0.015 (0.013 - 0.017)	4.161 (2.743 - 5.873)	4.172 (2.750 - 5.828)	4.001 (2.639 - 5.622)
70+ years (rate per capita)	0.330 (0.307 - 0.354)	0.332 (0.308 - 0.359)	0.324 (0.302 - 0.349)	0.014 (0.011 - 0.016)	0.014 (0.012 - 0.017)	0.014 (0.012 - 0.016)	8.826 (5.902 - 12.157)	8.886 (5.946 - 12.261)	8.674 (5.791 - 11.907)
All ages (rate per capita)	0.060 (0.057 - 0.064)	0.069 (0.065 - 0.073)	0.077 (0.073 - 0.082)	0.005 (0.004 - 0.005)	0.005 (0.005 - 0.005)	0.006 (0.005 - 0.006)	1.652 (1.099 - 2.287)	1.900 (1.263 - 2.636)	2.118 (1.411 - 2.941)
Age-standardized (rate per capita)	0.058 (0.055 - 0.062)	0.058 (0.055 - 0.062)	0.059 (0.055 - 0.062)	0.004 (0.004 - 0.005)	0.004 (0.004 - 0.005)	0.004 (0.004 - 0.005)	1.605 (1.069 - 2.221)	1.607 (1.067 - 2.230)	1.615 (1.076 - 2.238)
Total number of cases (thousands)	24,757 (23,404 - 26,310)	28,055 (26,430 - 29,747)	32,251 (30,431 - 34,299)	1,924 (1,790 - 2,069)	2,088 (1,936 - 2,233)	2,415 (2,244 - 2,598)	683 (454 - 945)	772 (513 - 1,071)	887 (591 - 1,231)
**Eastern Europe**									
15 - 49 years (rate per capita)	0.020 (0.019 - 0.022)	0.021 (0.020 - 0.023)	0.022 (0.021 - 0.023)	0.001 (0.001 - 0.002)	0.002 (0.001 - 0.002)	0.002 (0.002 - 0.002)	0.578 (0.380 - 0.812)	0.613 (0.402 - 0.858)	0.627 (0.416 - 0.888)
50 - 69 years (rate per capita)	0.143 (0.134 - 0.153)	0.147 (0.138 - 0.156)	0.134 (0.125 - 0.144)	0.015 (0.014 - 0.016)	0.015 (0.014 - 0.016)	0.014 (0.013 - 0.016)	3.968 (2.633 - 5.536)	4.060 (2.713 - 5.642)	3.716 (2.456 - 5.189)
70+ years (rate per capita)	0.326 (0.306 - 0.346)	0.329 (0.308 - 0.349)	0.319 (0.299 - 0.339)	0.013 (0.011 - 0.015)	0.014 (0.012 - 0.017)	0.014 (0.012 - 0.016)	8.705 (5.892 - 12.008)	8.770 (5.932 - 12.098)	8.528 (5.732 - 11.802)
All ages (rate per capita)	0.064 (0.061 - 0.067)	0.073 (0.070 - 0.077)	0.082 (0.078 - 0.086)	0.005 (0.005 - 0.005)	0.005 (0.005 - 0.006)	0.006 (0.006 - 0.006)	1.752 (1.172 - 2.409)	2.005 (1.348 - 2.774)	2.246 (1.508 - 3.094)
Age-standardized (rate per capita)	0.057 (0.054 - 0.060)	0.057 (0.054 - 0.059)	0.057 (0.054 - 0.060)	0.004 (0.004 - 0.005)	0.004 (0.004 - 0.005)	0.004 (0.004 - 0.005)	1.560 (1.046 - 2.150)	1.557 (1.048 - 2.160)	1.561 (1.050 - 2.152)
Total number of cases (thousands)	14,098 (13,446 - 14,805)	15,425 (14,710 - 16,183)	17,614 (16,799 - 18,485)	1,093 (1,029 - 1,162)	1,144 (1,071 - 1,212)	1,311 (1,234 - 1,396)	388 (259 - 533)	423 (284 - 585)	483 (324 - 665)
**Belarus**									
15 - 49 years (rate per capita)	0.019 (0.017 - 0.021)	0.021 (0.019 - 0.023)	0.021 (0.019 - 0.023)	0.001 (0.001 - 0.002)	0.002 (0.001 - 0.002)	0.002 (0.001 - 0.002)	0.544 (0.356 - 0.773)	0.595 (0.393 - 0.836)	0.600 (0.396 - 0.848)
50 - 69 years (rate per capita)	0.141 (0.127 - 0.155)	0.143 (0.129 - 0.156)	0.127 (0.115 - 0.140)	0.015 (0.013 - 0.017)	0.014 (0.013 - 0.016)	0.013 (0.012 - 0.015)	3.921 (2.586 - 5.540)	3.958 (2.608 - 5.565)	3.542 (2.351 - 4.993)
70+ years (rate per capita)	0.311 (0.284 - 0.340)	0.316 (0.288 - 0.346)	0.308 (0.280 - 0.337)	0.012 (0.010 - 0.015)	0.014 (0.011 - 0.016)	0.013 (0.011 - 0.015)	8.330 (5.593 - 11.531)	8.471 (5.666 - 11.775)	8.234 (5.479 - 11.417)
All ages (rate per capita)	0.061 (0.057 - 0.066)	0.072 (0.066 - 0.077)	0.078 (0.072 - 0.084)	0.005 (0.004 - 0.005)	0.005 (0.005 - 0.006)	0.006 (0.005 - 0.006)	1.696 (1.134 - 2.360)	1.972 (1.315 - 2.725)	2.143 (1.426 - 2.987)
Age-standardized (rate per capita)	0.055 (0.051 - 0.059)	0.055 (0.051 - 0.059)	0.055 (0.051 - 0.059)	0.004 (0.004 - 0.005)	0.004 (0.004 - 0.004)	0.004 (0.004 - 0.004)	1.511 (1.013 - 2.102)	1.508 (1.006 - 2.094)	1.508 (1.002 - 2.103)
Total number of cases (thousands)	623 (576 - 669)	694 (642 - 745)	750 (693 - 804)	48 (44 - 53)	51 (46 - 55)	55 (50 - 60)	17 (12 - 24)	19 (13 - 26)	21 (14 - 29)
**Estonia**									
15 - 49 years (rate per capita)	0.021 (0.019 - 0.022)	0.021 (0.020 - 0.022)	0.022 (0.020 - 0.024)	0.002 (0.001 - 0.002)	0.002 (0.001 - 0.002)	0.002 (0.002 - 0.002)	0.593 (0.393 - 0.835)	0.601 (0.394 - 0.846)	0.631 (0.415 - 0.886)
50 - 69 years (rate per capita)	0.145 (0.135 - 0.156)	0.149 (0.139 - 0.159)	0.143 (0.133 - 0.153)	0.015 (0.014 - 0.017)	0.015 (0.014 - 0.017)	0.015 (0.013 - 0.016)	4.031 (2.680 - 5.664)	4.137 (2.748 - 5.785)	3.981 (2.654 - 5.586)
70+ years (rate per capita)	0.319 (0.297 - 0.343)	0.320 (0.299 - 0.342)	0.314 (0.292 - 0.337)	0.013 (0.011 - 0.015)	0.014 (0.012 - 0.016)	0.013 (0.011 - 0.015)	8.528 (5.724 - 11.848)	8.591 (5.760 - 11.902)	8.428 (5.654 - 11.717)
All ages (rate per capita)	0.066 (0.063 - 0.070)	0.081 (0.077 - 0.085)	0.088 (0.084 - 0.094)	0.005 (0.005 - 0.005)	0.006 (0.005 - 0.006)	0.006 (0.006 - 0.007)	1.818 (1.219 - 2.538)	2.226 (1.481 - 3.069)	2.430 (1.633 - 3.392)
Age-standardized (rate per capita)	0.056 (0.054 - 0.059)	0.056 (0.053 - 0.059)	0.056 (0.053 - 0.059)	0.004 (0.004 - 0.005)	0.004 (0.004 - 0.004)	0.004 (0.004 - 0.005)	1.552 (1.041 - 2.170)	1.550 (1.030 - 2.140)	1.557 (1.047 - 2.171)
Total number of cases (thousands)	103 (98 - 109)	109 (104 - 115)	120 (114 - 127)	8 (7 - 8)	8 (7 - 8)	8 (8 - 9)	3 (2 - 4)	3 (2 - 4)	3 (2 - 5)
**Latvia**									
15 - 49 years (rate per capita)	0.018 (0.017 - 0.020)	0.019 (0.017 - 0.020)	0.019 (0.018 - 0.021)	0.001 (0.001 - 0.002)	0.001 (0.001 - 0.002)	0.001 (0.001 - 0.002)	0.525 (0.347 - 0.732)	0.531 (0.350 - 0.743)	0.555 (0.361 - 0.776)
50 - 69 years (rate per capita)	0.128 (0.120 - 0.137)	0.135 (0.126 - 0.145)	0.125 (0.117 - 0.133)	0.013 (0.012 - 0.015)	0.014 (0.012 - 0.015)	0.013 (0.012 - 0.014)	3.546 (2.366 - 4.943)	3.744 (2.493 - 5.229)	3.467 (2.306 - 4.799)
70+ years (rate per capita)	0.287 (0.268 - 0.306)	0.290 (0.270 - 0.310)	0.284 (0.265 - 0.303)	0.012 (0.010 - 0.013)	0.012 (0.011 - 0.014)	0.012 (0.010 - 0.014)	7.641 (5.142 - 10.510)	7.740 (5.223 - 10.672)	7.585 (5.109 - 10.496)
All ages (rate per capita)	0.060 (0.058 - 0.063)	0.073 (0.069 - 0.077)	0.079 (0.076 - 0.083)	0.005 (0.004 - 0.005)	0.005 (0.005 - 0.006)	0.006 (0.005 - 0.006)	1.656 (1.114 - 2.294)	2.000 (1.343 - 2.765)	2.175 (1.462 - 3.006)
Age-standardized (rate per capita)	0.050 (0.048 - 0.053)	0.050 (0.048 - 0.053)	0.050 (0.048 - 0.053)	0.004 (0.004 - 0.004)	0.004 (0.004 - 0.004)	0.004 (0.004 - 0.004)	1.383 (0.931 - 1.916)	1.379 (0.925 - 1.905)	1.384 (0.927 - 1.916)
Total number of cases (thousands)	160 (152 - 168)	168 (160 - 178)	176 (167 - 185)	12 (11 - 13)	12 (11 - 13)	12 (12 - 13)	4 (3 - 6)	5 (3 - 6)	5 (3 - 7)
**Lithuania**									
15 - 49 years (rate per capita)	0.019 (0.018 - 0.021)	0.021 (0.019 - 0.023)	0.021 (0.019 - 0.023)	0.001 (0.001 - 0.002)	0.002 (0.001 - 0.002)	0.002 (0.001 - 0.002)	0.553 (0.361 - 0.779)	0.591 (0.385 - 0.836)	0.604 (0.396 - 0.857)
50 - 69 years (rate per capita)	0.142 (0.129 - 0.156)	0.147 (0.133 - 0.161)	0.137 (0.124 - 0.150)	0.015 (0.013 - 0.017)	0.015 (0.013 - 0.017)	0.014 (0.012 - 0.016)	3.941 (2.600 - 5.577)	4.065 (2.653 - 5.724)	3.808 (2.491 - 5.370)
70+ years (rate per capita)	0.309 (0.283 - 0.338)	0.315 (0.286 - 0.344)	0.309 (0.282 - 0.337)	0.012 (0.010 - 0.015)	0.014 (0.011 - 0.016)	0.013 (0.010 - 0.015)	8.223 (5.536 - 11.397)	8.390 (5.575 - 11.549)	8.238 (5.523 - 11.357)
All ages (rate per capita)	0.061 (0.056 - 0.065)	0.074 (0.069 - 0.080)	0.085 (0.079 - 0.091)	0.005 (0.004 - 0.005)	0.005 (0.005 - 0.006)	0.006 (0.005 - 0.006)	1.668 (1.116 - 2.312)	2.038 (1.367 - 2.813)	2.313 (1.552 - 3.195)
Age-standardized (rate per capita)	0.055 (0.051 - 0.059)	0.055 (0.051 - 0.059)	0.055 (0.051 - 0.059)	0.004 (0.004 - 0.004)	0.004 (0.004 - 0.004)	0.004 (0.004 - 0.004)	1.509 (1.009 - 2.087)	1.509 (1.009 - 2.090)	1.511 (1.011 - 2.106)
Total number of cases (thousands)	224 (208 - 240)	254 (236 - 273)	267 (248 - 286)	17 (16 - 19)	18 (17 - 20)	19 (17 - 20)	6 (4 - 9)	7 (5 - 10)	7 (5 - 10)
**Moldova**									
15 - 49 years (rate per capita)	0.019 (0.017 - 0.021)	0.019 (0.017 - 0.021)	0.020 (0.018 - 0.022)	0.001 (0.001 - 0.002)	0.002 (0.001 - 0.002)	0.002 (0.001 - 0.002)	0.538 (0.352 - 0.762)	0.545 (0.361 - 0.771)	0.565 (0.371 - 0.808)
50 - 69 years (rate per capita)	0.141 (0.127 - 0.155)	0.134 (0.121 - 0.147)	0.132 (0.119 - 0.145)	0.015 (0.013 - 0.017)	0.014 (0.012 - 0.016)	0.014 (0.012 - 0.016)	3.904 (2.577 - 5.506)	3.713 (2.436 - 5.229)	3.687 (2.401 - 5.196)
70+ years (rate per capita)	0.315 (0.287 - 0.346)	0.315 (0.287 - 0.346)	0.310 (0.283 - 0.341)	0.014 (0.011 - 0.017)	0.014 (0.012 - 0.017)	0.013 (0.011 - 0.016)	8.417 (5.602 - 11.703)	8.449 (5.629 - 11.737)	8.320 (5.523 - 11.618)
All ages (rate per capita)	0.050 (0.046 - 0.054)	0.056 (0.052 - 0.060)	0.064 (0.059 - 0.069)	0.004 (0.004 - 0.004)	0.004 (0.004 - 0.005)	0.005 (0.005 - 0.006)	1.375 (0.915 - 1.900)	1.537 (1.021 - 2.131)	1.767 (1.173 - 2.447)
Age-standardized (rate per capita)	0.055 (0.051 - 0.059)	0.055 (0.051 - 0.059)	0.055 (0.051 - 0.059)	0.004 (0.004 - 0.005)	0.004 (0.004 - 0.005)	0.004 (0.004 - 0.005)	1.500 (0.997 - 2.074)	1.503 (0.999 - 2.094)	1.505 (1.002 - 2.088)
Total number of cases (thousands)	218 (202 - 234)	232 (216 - 249)	260 (242 - 280)	17 (16 - 19)	18 (16 - 20)	21 (19 - 23)	6 (4 - 8)	6 (4 - 9)	7 (5 - 10)
**Russia**									
15 - 49 years (rate per capita)	0.021 (0.019 - 0.022)	0.022 (0.021 - 0.024)	0.022 (0.021 - 0.024)	0.002 (0.001 - 0.002)	0.002 (0.002 - 0.002)	0.002 (0.002 - 0.002)	0.592 (0.389 - 0.832)	0.631 (0.413 - 0.883)	0.644 (0.427 - 0.913)
50 - 69 years (rate per capita)	0.146 (0.137 - 0.156)	0.149 (0.140 - 0.158)	0.136 (0.127 - 0.146)	0.015 (0.014 - 0.017)	0.015 (0.014 - 0.016)	0.015 (0.013 - 0.016)	4.041 (2.681 - 5.642)	4.102 (2.753 - 5.703)	3.772 (2.497 - 5.288)
70+ years (rate per capita)	0.336 (0.315 - 0.355)	0.338 (0.317 - 0.359)	0.328 (0.308 - 0.347)	0.014 (0.012 - 0.016)	0.015 (0.012 - 0.017)	0.014 (0.012 - 0.017)	8.947 (6.074 - 12.283)	8.999 (6.065 - 12.402)	8.746 (5.900 - 12.066)
All ages (rate per capita)	0.064 (0.061 - 0.067)	0.073 (0.070 - 0.077)	0.082 (0.079 - 0.086)	0.005 (0.005 - 0.005)	0.005 (0.005 - 0.006)	0.006 (0.006 - 0.007)	1.749 (1.169 - 2.412)	2.002 (1.350 - 2.773)	2.256 (1.516 - 3.105)
Age-standardized (rate per capita)	0.058 (0.056 - 0.061)	0.058 (0.056 - 0.061)	0.058 (0.056 - 0.061)	0.004 (0.004 - 0.005)	0.004 (0.004 - 0.005)	0.004 (0.004 - 0.005)	1.601 (1.073 - 2.211)	1.596 (1.081 - 2.208)	1.599 (1.076 - 2.210)
Total number of cases (thousands)	9,388 (8,972 - 9,844)	10,459 (9,996 - 10,977)	12,192 (11,647 - 12,783)	732 (690 - 778)	778 (732 - 824)	914 (864 - 972)	258 (172 - 356)	287 (193 - 397)	334 (224 - 460)
**Ukraine**									

	Prevalence 1990	Prevalence 2005	Prevalence 2015	Incidence 1990	Incidence 2005	Incidence 2015	YLD rate 1990	YLD rate 2005	YLD rate 2015
15 - 49 years (rate per capita)	0.019 (0.018 - 0.021)	0.020 (0.019 - 0.021)	0.021 (0.019 - 0.022)	0.001 (0.001 - 0.002)	0.002 (0.001 - 0.002)	0.002 (0.001 - 0.002)	0.549 (0.358 - 0.765)	0.574 (0.378 - 0.809)	0.590 (0.390 - 0.830)
50 - 69 years (rate per capita)	0.137 (0.128 - 0.148)	0.144 (0.135 - 0.155)	0.128 (0.120 - 0.138)	0.014 (0.013 - 0.016)	0.015 (0.013 - 0.016)	0.014 (0.012 - 0.015)	3.810 (2.539 - 5.322)	3.998 (2.654 - 5.552)	3.573 (2.372 - 4.974)
70+ years (rate per capita)	0.310 (0.289 - 0.331)	0.311 (0.290 - 0.332)	0.301 (0.280 - 0.322)	0.013 (0.011 - 0.015)	0.013 (0.011 - 0.015)	0.014 (0.012 - 0.016)	8.296 (5.575 - 11.491)	8.334 (5.632 - 11.562)	8.078 (5.429 - 11.184)
All ages (rate per capita)	0.066 (0.063 - 0.069)	0.075 (0.071 - 0.079)	0.083 (0.078 - 0.087)	0.005 (0.005 - 0.005)	0.006 (0.005 - 0.006)	0.006 (0.006 - 0.006)	1.813 (1.204 - 2.493)	2.051 (1.376 - 2.827)	2.273 (1.513 - 3.141)
Age-standardized (rate per capita)	0.054 (0.051 - 0.057)	0.054 (0.051 - 0.056)	0.054 (0.051 - 0.056)	0.004 (0.004 - 0.004)	0.004 (0.004 - 0.004)	0.004 (0.004 - 0.004)	1.480 (0.988 - 2.038)	1.475 (0.991 - 2.037)	1.478 (0.987 - 2.041)
Total number of cases (thousands)	3,382 (3,214 - 3,564)	3,508 (3,335 - 3,697)	3,851 (3,651 - 4,060)	259 (241 - 276)	258 (242 - 275)	281 (262 - 301)	93 (62 - 128)	96 (65 - 133)	106 (70 - 146)
**Central Europe**									
15 - 49 years (rate per capita)	0.020 (0.017 - 0.022)	0.020 (0.018 - 0.023)	0.021 (0.019 - 0.024)	0.002 (0.001 - 0.002)	0.002 (0.001 - 0.002)	0.002 (0.001 - 0.002)	0.564 (0.369 - 0.804)	0.588 (0.384 - 0.836)	0.615 (0.401 - 0.877)
50 - 69 years (rate per capita)	0.154 (0.136 - 0.174)	0.147 (0.130 - 0.165)	0.152 (0.134 - 0.171)	0.015 (0.013 - 0.018)	0.015 (0.012 - 0.017)	0.015 (0.013 - 0.018)	4.279 (2.746 - 6.068)	4.089 (2.621 - 5.782)	4.242 (2.727 - 6.014)
70+ years (rate per capita)	0.319 (0.286 - 0.358)	0.321 (0.287 - 0.360)	0.316 (0.283 - 0.354)	0.013 (0.011 - 0.017)	0.014 (0.011 - 0.017)	0.013 (0.010 - 0.016)	8.538 (5.633 - 11.859)	8.591 (5.649 - 11.898)	8.442 (5.565 - 11.741)
All ages (rate per capita)	0.061 (0.056 - 0.067)	0.075 (0.069 - 0.083)	0.086 (0.078 - 0.094)	0.005 (0.004 - 0.005)	0.006 (0.005 - 0.006)	0.006 (0.006 - 0.007)	1.695 (1.112 - 2.372)	2.067 (1.360 - 2.878)	2.355 (1.543 - 3.286)
Age-standardized (rate per capita)	0.057 (0.052 - 0.062)	0.057 (0.052 - 0.062)	0.057 (0.052 - 0.062)	0.004 (0.004 - 0.005)	0.004 (0.004 - 0.005)	0.004 (0.004 - 0.005)	1.569 (1.029 - 2.192)	1.570 (1.032 - 2.189)	1.570 (1.029 - 2.188)
Total number of cases (thousands)	7,568 (6,905 - 8,300)	8,877 (8,107 - 9,754)	9,995 (9,111 - 10,960)	581 (521 - 650)	655 (588 - 728)	731 (653 - 818)	209 (137 - 292)	244 (161 - 340)	275 (180 - 383)
**Albania**									
15 - 49 years (rate per capita)	0.017 (0.015 - 0.020)	0.019 (0.017 - 0.023)	0.019 (0.016 - 0.022)	0.001 (0.001 - 0.002)	0.002 (0.001 - 0.002)	0.002 (0.001 - 0.002)	0.489 (0.316 - 0.702)	0.560 (0.362 - 0.804)	0.541 (0.348 - 0.771)
50 - 69 years (rate per capita)	0.146 (0.125 - 0.169)	0.150 (0.128 - 0.173)	0.150 (0.128 - 0.173)	0.014 (0.012 - 0.018)	0.015 (0.012 - 0.018)	0.015 (0.012 - 0.018)	4.082 (2.590 - 5.923)	4.187 (2.672 - 6.029)	4.193 (2.651 - 6.063)
70+ years (rate per capita)	0.323 (0.280 - 0.372)	0.322 (0.280 - 0.372)	0.321 (0.279 - 0.369)	0.014 (0.011 - 0.019)	0.015 (0.011 - 0.019)	0.014 (0.011 - 0.018)	8.680 (5.607 - 12.273)	8.688 (5.624 - 12.268)	8.639 (5.645 - 12.209)
All ages (rate per capita)	0.038 (0.034 - 0.042)	0.053 (0.047 - 0.059)	0.071 (0.063 - 0.080)	0.003 (0.003 - 0.003)	0.004 (0.004 - 0.005)	0.005 (0.005 - 0.006)	1.052 (0.687 - 1.483)	1.461 (0.953 - 2.063)	1.959 (1.269 - 2.773)
Age-standardized (rate per capita)	0.058 (0.051 - 0.065)	0.057 (0.051 - 0.065)	0.058 (0.051 - 0.065)	0.004 (0.004 - 0.005)	0.004 (0.004 - 0.005)	0.004 (0.004 - 0.005)	1.591 (1.041 - 2.242)	1.591 (1.037 - 2.238)	1.595 (1.035 - 2.258)
Total number of cases (thousands)	124 (111 - 139)	162 (145 - 182)	205 (182 - 231)	10 (9 - 11)	13 (11 - 15)	16 (14 - 18)	3 (2 - 5)	5 (3 - 6)	6 (4 - 8)
**Bosnia and Herzegovina**									
15 - 49 years (rate per capita)	0.021 (0.020 - 0.023)	0.025 (0.023 - 0.026)	0.025 (0.023 - 0.027)	0.002 (0.002 - 0.002)	0.002 (0.002 - 0.002)	0.002 (0.002 - 0.002)	0.608 (0.402 - 0.854)	0.701 (0.465 - 0.983)	0.714 (0.468 - 1.013)
50 - 69 years (rate per capita)	0.163 (0.151 - 0.175)	0.170 (0.158 - 0.182)	0.171 (0.159 - 0.185)	0.017 (0.015 - 0.018)	0.017 (0.015 - 0.018)	0.017 (0.015 - 0.019)	4.546 (3.003 - 6.325)	4.713 (3.138 - 6.573)	4.727 (3.123 - 6.629)
70+ years (rate per capita)	0.367 (0.346 - 0.391)	0.371 (0.348 - 0.396)	0.360 (0.338 - 0.383)	0.015 (0.013 - 0.017)	0.017 (0.014 - 0.020)	0.014 (0.012 - 0.017)	9.884 (6.680 - 13.676)	9.961 (6.727 - 13.671)	9.592 (6.481 - 13.112)
All ages (rate per capita)	0.055 (0.052 - 0.058)	0.082 (0.078 - 0.086)	0.096 (0.091 - 0.101)	0.004 (0.004 - 0.005)	0.006 (0.006 - 0.007)	0.007 (0.007 - 0.008)	1.529 (1.023 - 2.121)	2.254 (1.511 - 3.121)	2.627 (1.758 - 3.623)
Age-standardized (rate per capita)	0.065 (0.062 - 0.069)	0.065 (0.062 - 0.069)	0.064 (0.061 - 0.068)	0.005 (0.005 - 0.005)	0.005 (0.005 - 0.005)	0.005 (0.005 - 0.005)	1.813 (1.213 - 2.507)	1.802 (1.209 - 2.501)	1.772 (1.186 - 2.441)
Total number of cases (thousands)	248 (236 - 262)	314 (299 - 330)	365 (347 - 385)	20 (19 - 22)	24 (22 - 25)	27 (25 - 29)	7 (5 - 10)	9 (6 - 12)	10 (7 - 14)
**Bulgaria**									
15 - 49 years (rate per capita)	0.021 (0.018 - 0.024)	0.021 (0.018 - 0.025)	0.023 (0.019 - 0.026)	0.002 (0.001 - 0.002)	0.002 (0.001 - 0.002)	0.002 (0.002 - 0.002)	0.596 (0.388 - 0.857)	0.611 (0.394 - 0.884)	0.652 (0.421 - 0.949)
50 - 69 years (rate per capita)	0.160 (0.136 - 0.186)	0.153 (0.130 - 0.178)	0.161 (0.137 - 0.187)	0.016 (0.013 - 0.019)	0.015 (0.012 - 0.019)	0.016 (0.013 - 0.020)	4.444 (2.832 - 6.476)	4.271 (2.711 - 6.165)	4.485 (2.841 - 6.520)
70+ years (rate per capita)	0.322 (0.280 - 0.371)	0.322 (0.280 - 0.372)	0.319 (0.277 - 0.368)	0.014 (0.010 - 0.018)	0.014 (0.011 - 0.018)	0.013 (0.010 - 0.017)	8.609 (5.605 - 12.032)	8.631 (5.615 - 12.104)	8.538 (5.563 - 11.920)
All ages (rate per capita)	0.073 (0.064 - 0.083)	0.089 (0.078 - 0.100)	0.098 (0.086 - 0.111)	0.006 (0.005 - 0.006)	0.006 (0.006 - 0.007)	0.007 (0.006 - 0.008)	2.012 (1.308 - 2.828)	2.434 (1.584 - 3.412)	2.700 (1.761 - 3.814)
Age-standardized (rate per capita)	0.057 (0.051 - 0.064)	0.057 (0.051 - 0.065)	0.057 (0.051 - 0.065)	0.004 (0.004 - 0.005)	0.004 (0.004 - 0.005)	0.004 (0.004 - 0.005)	1.580 (1.031 - 2.222)	1.583 (1.034 - 2.227)	1.584 (1.034 - 2.239)
Total number of cases (thousands)	644 (568 - 729)	680 (601 - 769)	714 (627 - 808)	49 (43 - 57)	49 (43 - 57)	51 (44 - 59)	18 (12 - 25)	19 (12 - 26)	20 (13 - 28)
**Croatia**									
15 - 49 years (rate per capita)	0.020 (0.019 - 0.022)	0.022 (0.020 - 0.023)	0.022 (0.020 - 0.024)	0.002 (0.001 - 0.002)	0.002 (0.002 - 0.002)	0.002 (0.002 - 0.002)	0.580 (0.382 - 0.821)	0.625 (0.410 - 0.873)	0.630 (0.413 - 0.894)
50 - 69 years (rate per capita)	0.150 (0.140 - 0.162)	0.152 (0.141 - 0.163)	0.155 (0.143 - 0.167)	0.015 (0.014 - 0.017)	0.015 (0.014 - 0.016)	0.015 (0.014 - 0.017)	4.173 (2.773 - 5.833)	4.211 (2.805 - 5.888)	4.298 (2.851 - 6.020)
70+ years (rate per capita)	0.317 (0.297 - 0.340)	0.320 (0.299 - 0.343)	0.314 (0.294 - 0.336)	0.013 (0.011 - 0.015)	0.014 (0.012 - 0.017)	0.012 (0.011 - 0.014)	8.464 (5.732 - 11.750)	8.587 (5.796 - 11.795)	8.386 (5.671 - 11.655)
All ages (rate per capita)	0.068 (0.065 - 0.072)	0.084 (0.080 - 0.089)	0.093 (0.088 - 0.099)	0.005 (0.005 - 0.006)	0.006 (0.006 - 0.007)	0.007 (0.006 - 0.007)	1.875 (1.255 - 2.601)	2.311 (1.550 - 3.204)	2.554 (1.714 - 3.550)
Age-standardized (rate per capita)	0.057 (0.054 - 0.060)	0.057 (0.054 - 0.060)	0.057 (0.054 - 0.060)	0.004 (0.004 - 0.005)	0.004 (0.004 - 0.005)	0.004 (0.004 - 0.005)	1.561 (1.048 - 2.161)	1.563 (1.048 - 2.168)	1.563 (1.049 - 2.172)
Total number of cases (thousands)	325 (308 - 345)	369 (350 - 390)	395 (375 - 419)	25 (23 - 27)	27 (25 - 29)	28 (26 - 30)	9 (6 - 12)	10 (7 - 14)	11 (7 - 15)
**Czech Republic**									
15 - 49 years (rate per capita)	0.021 (0.020 - 0.023)	0.021 (0.020 - 0.023)	0.024 (0.022 - 0.025)	0.002 (0.002 - 0.002)	0.002 (0.002 - 0.002)	0.002 (0.002 - 0.002)	0.616 (0.404 - 0.867)	0.613 (0.402 - 0.859)	0.679 (0.444 - 0.958)
50 - 69 years (rate per capita)	0.164 (0.153 - 0.177)	0.148 (0.138 - 0.160)	0.168 (0.156 - 0.181)	0.016 (0.015 - 0.018)	0.015 (0.014 - 0.016)	0.017 (0.015 - 0.018)	4.568 (3.034 - 6.391)	4.139 (2.735 - 5.769)	4.665 (3.113 - 6.529)
70+ years (rate per capita)	0.329 (0.309 - 0.352)	0.328 (0.305 - 0.351)	0.321 (0.301 - 0.343)	0.013 (0.011 - 0.015)	0.014 (0.012 - 0.016)	0.013 (0.011 - 0.015)	8.792 (5.960 - 12.147)	8.781 (5.925 - 12.220)	8.572 (5.812 - 11.805)
All ages (rate per capita)	0.069 (0.066 - 0.073)	0.080 (0.076 - 0.085)	0.091 (0.086 - 0.096)	0.005 (0.005 - 0.005)	0.006 (0.006 - 0.006)	0.007 (0.006 - 0.007)	1.912 (1.283 - 2.664)	2.215 (1.483 - 3.066)	2.501 (1.678 - 3.477)
Age-standardized (rate per capita)	0.059 (0.056 - 0.062)	0.058 (0.055 - 0.062)	0.058 (0.056 - 0.062)	0.004 (0.004 - 0.005)	0.004 (0.004 - 0.005)	0.004 (0.004 - 0.005)	1.614 (1.084 - 2.248)	1.612 (1.078 - 2.233)	1.611 (1.078 - 2.238)
Total number of cases (thousands)	715 (679 - 757)	826 (783 - 873)	972 (924 - 1,030)	53 (49 - 57)	61 (57 - 65)	71 (66 - 77)	20 (13 - 27)	23 (15 - 32)	27 (18 - 37)
**Hungary**									
15 - 49 years (rate per capita)	0.020 (0.018 - 0.021)	0.020 (0.018 - 0.021)	0.020 (0.019 - 0.021)	0.002 (0.001 - 0.002)	0.002 (0.001 - 0.002)	0.002 (0.001 - 0.002)	0.562 (0.371 - 0.785)	0.563 (0.373 - 0.788)	0.573 (0.378 - 0.808)
50 - 69 years (rate per capita)	0.147 (0.138 - 0.158)	0.139 (0.130 - 0.148)	0.145 (0.136 - 0.155)	0.015 (0.013 - 0.016)	0.014 (0.013 - 0.015)	0.014 (0.013 - 0.016)	4.082 (2.714 - 5.626)	3.851 (2.555 - 5.360)	4.012 (2.662 - 5.544)
70+ years (rate per capita)	0.304 (0.285 - 0.324)	0.303 (0.285 - 0.320)	0.299 (0.281 - 0.318)	0.013 (0.011 - 0.015)	0.013 (0.011 - 0.015)	0.012 (0.010 - 0.014)	8.092 (5.470 - 11.241)	8.068 (5.433 - 11.125)	7.962 (5.373 - 11.017)
All ages (rate per capita)	0.068 (0.064 - 0.071)	0.077 (0.074 - 0.081)	0.083 (0.080 - 0.088)	0.005 (0.005 - 0.005)	0.006 (0.005 - 0.006)	0.006 (0.006 - 0.006)	1.854 (1.241 - 2.565)	2.110 (1.412 - 2.903)	2.284 (1.530 - 3.169)
Age-standardized (rate per capita)	0.054 (0.051 - 0.056)	0.054 (0.051 - 0.056)	0.054 (0.051 - 0.056)	0.004 (0.004 - 0.004)	0.004 (0.004 - 0.004)	0.004 (0.004 - 0.004)	1.472 (0.985 - 2.035)	1.472 (0.985 - 2.028)	1.475 (0.988 - 2.044)
Total number of cases (thousands)	702 (670 - 738)	777 (742 - 813)	848 (809 - 891)	52 (49 - 56)	56 (53 - 60)	61 (57 - 65)	19 (13 - 27)	21 (14 - 29)	23 (16 - 32)
**Macedonia**									
15 - 49 years (rate per capita)	0.019 (0.016 - 0.022)	0.020 (0.018 - 0.024)	0.021 (0.018 - 0.025)	0.002 (0.001 - 0.002)	0.002 (0.001 - 0.002)	0.002 (0.001 - 0.002)	0.540 (0.348 - 0.782)	0.590 (0.382 - 0.849)	0.614 (0.399 - 0.879)
50 - 69 years (rate per capita)	0.149 (0.127 - 0.172)	0.148 (0.127 - 0.170)	0.152 (0.129 - 0.175)	0.015 (0.012 - 0.018)	0.015 (0.012 - 0.018)	0.015 (0.012 - 0.019)	4.157 (2.631 - 5.983)	4.121 (2.640 - 5.878)	4.228 (2.668 - 6.108)
70+ years (rate per capita)	0.321 (0.278 - 0.371)	0.324 (0.279 - 0.375)	0.321 (0.277 - 0.370)	0.014 (0.011 - 0.018)	0.015 (0.011 - 0.019)	0.014 (0.011 - 0.018)	8.610 (5.579 - 12.021)	8.690 (5.593 - 12.160)	8.592 (5.524 - 12.070)
All ages (rate per capita)	0.049 (0.043 - 0.054)	0.062 (0.055 - 0.069)	0.073 (0.065 - 0.081)	0.004 (0.003 - 0.004)	0.005 (0.004 - 0.005)	0.006 (0.005 - 0.006)	1.352 (0.879 - 1.903)	1.714 (1.115 - 2.402)	2.011 (1.302 - 2.845)
Age-standardized (rate per capita)	0.057 (0.051 - 0.064)	0.057 (0.051 - 0.064)	0.057 (0.051 - 0.064)	0.004 (0.004 - 0.005)	0.004 (0.004 - 0.005)	0.004 (0.004 - 0.005)	1.578 (1.031 - 2.218)	1.582 (1.031 - 2.210)	1.581 (1.030 - 2.232)
Total number of cases (thousands)	98 (87 - 109)	126 (112 - 141)	151 (134 - 169)	8 (7 - 9)	10 (9 - 11)	12 (10 - 13)	3 (2 - 4)	3 (2 - 5)	4 (3 - 6)
**Montenegro**									
15 - 49 years (rate per capita)	0.018 (0.016 - 0.022)	0.020 (0.018 - 0.024)	0.021 (0.018 - 0.024)	0.002 (0.001 - 0.002)	0.002 (0.001 - 0.002)	0.002 (0.001 - 0.002)	0.532 (0.348 - 0.771)	0.588 (0.383 - 0.855)	0.598 (0.386 - 0.870)
50 - 69 years (rate per capita)	0.146 (0.124 - 0.168)	0.150 (0.127 - 0.173)	0.153 (0.130 - 0.178)	0.015 (0.012 - 0.018)	0.015 (0.012 - 0.018)	0.015 (0.012 - 0.019)	4.073 (2.605 - 5.882)	4.174 (2.672 - 6.026)	4.276 (2.732 - 6.152)
70+ years (rate per capita)	0.319 (0.277 - 0.366)	0.324 (0.282 - 0.374)	0.322 (0.280 - 0.369)	0.013 (0.010 - 0.016)	0.015 (0.011 - 0.019)	0.014 (0.010 - 0.017)	8.568 (5.605 - 11.991)	8.726 (5.694 - 12.219)	8.631 (5.639 - 12.022)
All ages (rate per capita)	0.053 (0.047 - 0.059)	0.070 (0.062 - 0.078)	0.077 (0.068 - 0.086)	0.004 (0.004 - 0.005)	0.005 (0.005 - 0.006)	0.006 (0.005 - 0.007)	1.474 (0.963 - 2.075)	1.922 (1.255 - 2.690)	2.123 (1.386 - 2.991)
Age-standardized (rate per capita)	0.057 (0.051 - 0.064)	0.057 (0.051 - 0.064)	0.057 (0.051 - 0.064)	0.004 (0.004 - 0.005)	0.004 (0.004 - 0.005)	0.004 (0.004 - 0.005)	1.591 (1.043 - 2.242)	1.589 (1.039 - 2.228)	1.588 (1.035 - 2.229)
Total number of cases (thousands)	33 (29 - 36)	43 (38 - 48)	48 (43 - 54)	3 (2 - 3)	3 (3 - 4)	4 (3 - 4)	1 (1 - 1)	1 (1 - 2)	1 (1 - 2)
**Poland**									
15 - 49 years (rate per capita)	0.020 (0.017 - 0.023)	0.021 (0.018 - 0.024)	0.020 (0.017 - 0.023)	0.002 (0.001 - 0.002)	0.002 (0.001 - 0.002)	0.002 (0.001 - 0.002)	0.567 (0.365 - 0.823)	0.590 (0.382 - 0.851)	0.579 (0.376 - 0.832)
50 - 69 years (rate per capita)	0.156 (0.132 - 0.179)	0.144 (0.123 - 0.166)	0.145 (0.123 - 0.167)	0.015 (0.012 - 0.019)	0.014 (0.012 - 0.017)	0.015 (0.012 - 0.018)	4.311 (2.741 - 6.243)	4.009 (2.539 - 5.778)	4.026 (2.576 - 5.766)
70+ years (rate per capita)	0.321 (0.280 - 0.371)	0.322 (0.280 - 0.373)	0.317 (0.276 - 0.365)	0.013 (0.010 - 0.017)	0.014 (0.010 - 0.018)	0.013 (0.010 - 0.016)	8.562 (5.583 - 11.989)	8.610 (5.602 - 12.049)	8.447 (5.525 - 11.776)
All ages (rate per capita)	0.059 (0.053 - 0.066)	0.072 (0.064 - 0.081)	0.083 (0.074 - 0.093)	0.005 (0.004 - 0.005)	0.005 (0.005 - 0.006)	0.006 (0.005 - 0.007)	1.630 (1.060 - 2.294)	1.977 (1.294 - 2.779)	2.278 (1.491 - 3.197)

	Prevalence 1990	Prevalence 2005	Prevalence 2015	Incidence 1990	Incidence 2005	Incidence 2015	YLD rate 1990	YLD rate 2005	YLD rate 2015
Age-standardized (rate per capita)	0.057 (0.051 - 0.065)	0.057 (0.051 - 0.064)	0.057 (0.051 - 0.064)	0.004 (0.004 - 0.005)	0.004 (0.004 - 0.005)	0.004 (0.004 - 0.005)	1.579 (1.030 - 2.222)	1.580 (1.032 - 2.213)	1.581 (1.034 - 2.211)
Total number of cases (thousands)	2,263 (2,010 - 2,541)	2,747 (2,449 - 3,090)	3,226 (2,879 - 3,626)	172 (150 - 197)	203 (178 - 232)	239 (206 - 278)	62 (41 - 88)	76 (49 - 106)	89 (58 - 124)
**Romania**									
15 - 49 years (rate per capita)	0.019 (0.016 - 0.022)	0.020 (0.017 - 0.024)	0.024 (0.021 - 0.028)	0.002 (0.001 - 0.002)	0.002 (0.001 - 0.002)	0.002 (0.002 - 0.002)	0.542 (0.352 - 0.782)	0.577 (0.377 - 0.836)	0.678 (0.438 - 0.977)
50 - 69 years (rate per capita)	0.153 (0.130 - 0.177)	0.152 (0.130 - 0.176)	0.160 (0.136 - 0.186)	0.015 (0.012 - 0.019)	0.015 (0.012 - 0.018)	0.016 (0.013 - 0.020)	4.248 (2.701 - 6.132)	4.242 (2.702 - 6.153)	4.471 (2.843 - 6.414)
70+ years (rate per capita)	0.324 (0.280 - 0.374)	0.323 (0.280 - 0.375)	0.319 (0.276 - 0.368)	0.014 (0.010 - 0.018)	0.014 (0.011 - 0.019)	0.013 (0.010 - 0.017)	8.685 (5.607 - 12.102)	8.696 (5.611 - 12.188)	8.568 (5.525 - 11.901)
All ages (rate per capita)	0.062 (0.055 - 0.070)	0.077 (0.069 - 0.087)	0.089 (0.079 - 0.101)	0.005 (0.004 - 0.006)	0.006 (0.005 - 0.007)	0.006 (0.006 - 0.007)	1.720 (1.102 - 2.401)	2.134 (1.381 - 2.995)	2.465 (1.584 - 3.443)
Age-standardized (rate per capita)	0.057 (0.051 - 0.064)	0.057 (0.051 - 0.064)	0.057 (0.051 - 0.064)	0.004 (0.004 - 0.005)	0.004 (0.004 - 0.005)	0.004 (0.004 - 0.005)	1.581 (1.020 - 2.215)	1.584 (1.020 - 2.217)	1.583 (1.021 - 2.224)
Total number of cases (thousands)	1,457 (1,294 - 1,634)	1,656 (1,473 - 1,861)	1,748 (1,548 - 1,968)	114 (98 - 131)	122 (106 - 139)	126 (109 - 145)	40 (26 - 56)	46 (30 - 64)	48 (31 - 67)
**Serbia**									
15 - 49 years (rate per capita)	0.020 (0.017 - 0.023)	0.020 (0.018 - 0.024)	0.021 (0.019 - 0.025)	0.002 (0.001 - 0.002)	0.002 (0.001 - 0.002)	0.002 (0.001 - 0.002)	0.568 (0.369 - 0.817)	0.584 (0.377 - 0.838)	0.618 (0.394 - 0.887)
50 - 69 years (rate per capita)	0.154 (0.131 - 0.179)	0.148 (0.127 - 0.171)	0.163 (0.138 - 0.190)	0.015 (0.012 - 0.019)	0.015 (0.012 - 0.018)	0.016 (0.013 - 0.020)	4.288 (2.720 - 6.206)	4.118 (2.620 - 5.962)	4.518 (2.869 - 6.484)
70+ years (rate per capita)	0.320 (0.278 - 0.368)	0.325 (0.282 - 0.375)	0.319 (0.277 - 0.367)	0.013 (0.010 - 0.017)	0.015 (0.011 - 0.019)	0.013 (0.010 - 0.017)	8.559 (5.576 - 11.938)	8.696 (5.658 - 12.143)	8.510 (5.513 - 11.893)
All ages (rate per capita)	0.061 (0.054 - 0.068)	0.077 (0.068 - 0.086)	0.087 (0.077 - 0.098)	0.005 (0.004 - 0.006)	0.006 (0.005 - 0.007)	0.006 (0.006 - 0.007)	1.675 (1.089 - 2.375)	2.109 (1.378 - 2.961)	2.395 (1.564 - 3.353)
Age-standardized (rate per capita)	0.057 (0.051 - 0.064)	0.057 (0.051 - 0.064)	0.057 (0.051 - 0.064)	0.004 (0.004 - 0.005)	0.004 (0.004 - 0.005)	0.004 (0.004 - 0.005)	1.582 (1.036 - 2.222)	1.579 (1.032 - 2.222)	1.579 (1.033 - 2.219)
Total number of cases (thousands)	576 (512 - 648)	706 (628 - 794)	772 (684 - 870)	46 (40 - 53)	52 (46 - 60)	57 (49 - 65)	16 (10 - 23)	19 (13 - 27)	21 (14 - 30)
**Slovakia**									
15 - 49 years (rate per capita)	0.015 (0.014 - 0.016)	0.015 (0.014 - 0.017)	0.016 (0.015 - 0.017)	0.001 (0.001 - 0.001)	0.001 (0.001 - 0.001)	0.001 (0.001 - 0.001)	0.427 (0.282 - 0.598)	0.444 (0.293 - 0.623)	0.466 (0.307 - 0.656)
50 - 69 years (rate per capita)	0.123 (0.114 - 0.133)	0.111 (0.103 - 0.120)	0.115 (0.107 - 0.125)	0.012 (0.011 - 0.013)	0.011 (0.010 - 0.012)	0.012 (0.011 - 0.013)	3.418 (2.267 - 4.789)	3.093 (2.042 - 4.356)	3.217 (2.124 - 4.514)
70+ years (rate per capita)	0.254 (0.236 - 0.274)	0.253 (0.234 - 0.272)	0.249 (0.232 - 0.269)	0.011 (0.009 - 0.013)	0.011 (0.010 - 0.013)	0.011 (0.009 - 0.012)	6.800 (4.600 - 9.346)	6.776 (4.572 - 9.322)	6.671 (4.475 - 9.197)
All ages (rate per capita)	0.045 (0.043 - 0.048)	0.052 (0.049 - 0.056)	0.060 (0.056 - 0.063)	0.003 (0.003 - 0.004)	0.004 (0.004 - 0.004)	0.005 (0.004 - 0.005)	1.243 (0.833 - 1.717)	1.442 (0.957 - 2.003)	1.646 (1.102 - 2.294)
Age-standardized (rate per capita)	0.045 (0.042 - 0.047)	0.044 (0.042 - 0.047)	0.045 (0.042 - 0.047)	0.003 (0.003 - 0.004)	0.003 (0.003 - 0.004)	0.003 (0.003 - 0.004)	1.229 (0.824 - 1.700)	1.226 (0.815 - 1.705)	1.231 (0.825 - 1.711)
Total number of cases (thousands)	237 (225 - 253)	282 (266 - 300)	331 (313 - 353)	18 (17 - 20)	21 (20 - 23)	26 (24 - 27)	7 (4 - 9)	8 (5 - 11)	9 (6 - 13)
**Slovenia**									
15 - 49 years (rate per capita)	0.024 (0.022 - 0.026)	0.026 (0.024 - 0.028)	0.027 (0.025 - 0.029)	0.002 (0.002 - 0.002)	0.002 (0.002 - 0.002)	0.002 (0.002 - 0.002)	0.684 (0.452 - 0.968)	0.745 (0.491 - 1.053)	0.784 (0.518 - 1.102)
50 - 69 years (rate per capita)	0.177 (0.165 - 0.190)	0.173 (0.161 - 0.186)	0.175 (0.164 - 0.188)	0.018 (0.016 - 0.019)	0.017 (0.016 - 0.019)	0.017 (0.016 - 0.019)	4.917 (3.250 - 6.853)	4.808 (3.197 - 6.743)	4.896 (3.236 - 6.831)
70+ years (rate per capita)	0.369 (0.346 - 0.391)	0.369 (0.346 - 0.394)	0.360 (0.338 - 0.382)	0.014 (0.012 - 0.016)	0.015 (0.013 - 0.018)	0.014 (0.012 - 0.016)	9.856 (6.656 - 13.574)	9.895 (6.660 - 13.698)	9.638 (6.536 - 13.311)
All ages (rate per capita)	0.073 (0.070 - 0.077)	0.094 (0.089 - 0.099)	0.107 (0.102 - 0.112)	0.006 (0.005 - 0.006)	0.007 (0.006 - 0.007)	0.007 (0.007 - 0.008)	2.025 (1.358 - 2.793)	2.591 (1.733 - 3.587)	2.933 (1.962 - 4.044)
Age-standardized (rate per capita)	0.066 (0.063 - 0.070)	0.066 (0.063 - 0.070)	0.066 (0.063 - 0.069)	0.005 (0.005 - 0.005)	0.005 (0.005 - 0.005)	0.005 (0.005 - 0.005)	1.829 (1.227 - 2.516)	1.831 (1.228 - 2.537)	1.821 (1.224 - 2.515)
Total number of cases (thousands)	147 (140 - 155)	188 (179 - 198)	220 (210 - 232)	11 (10 - 12)	13 (13 - 14)	15 (14 - 16)	4 (3 - 6)	5 (3 - 7)	6 (4 - 8)
**Central Asia**									
15 - 49 years (rate per capita)	0.021 (0.019 - 0.023)	0.023 (0.021 - 0.025)	0.024 (0.022 - 0.026)	0.002 (0.002 - 0.002)	0.002 (0.002 - 0.002)	0.002 (0.002 - 0.002)	0.603 (0.395 - 0.852)	0.668 (0.440 - 0.938)	0.691 (0.453 - 0.975)
50 - 69 years (rate per capita)	0.176 (0.162 - 0.189)	0.180 (0.166 - 0.194)	0.165 (0.152 - 0.178)	0.018 (0.016 - 0.020)	0.018 (0.016 - 0.020)	0.017 (0.015 - 0.019)	4.904 (3.266 - 6.870)	5.020 (3.325 - 7.077)	4.608 (3.057 - 6.453)
70+ years (rate per capita)	0.398 (0.370 - 0.428)	0.399 (0.370 - 0.430)	0.397 (0.369 - 0.427)	0.016 (0.014 - 0.019)	0.018 (0.015 - 0.021)	0.016 (0.014 - 0.019)	10.728 (7.188 - 14.832)	10.761 (7.220 - 14.936)	10.661 (7.148 - 14.776)
All ages (rate per capita)	0.045 (0.043 - 0.047)	0.049 (0.046 - 0.052)	0.053 (0.050 - 0.056)	0.004 (0.003 - 0.004)	0.004 (0.004 - 0.004)	0.004 (0.004 - 0.005)	1.252 (0.837 - 1.740)	1.358 (0.909 - 1.889)	1.486 (0.995 - 2.066)
Age-standardized (rate per capita)	0.071 (0.067 - 0.075)	0.071 (0.067 - 0.075)	0.071 (0.067 - 0.075)	0.005 (0.005 - 0.006)	0.005 (0.005 - 0.006)	0.005 (0.005 - 0.006)	1.970 (1.318 - 2.735)	1.961 (1.310 - 2.727)	1.960 (1.311 - 2.718)
Total number of cases (thousands)	3,091 (2,930 - 3,267)	3,754 (3,561 - 3,971)	4,641 (4,390 - 4,914)	250 (233 - 268)	289 (270 - 309)	373 (347 - 400)	86 (58 - 120)	105 (70 - 145)	129 (87 - 180)
**Armenia**									
15 - 49 years (rate per capita)	0.022 (0.020 - 0.025)	0.025 (0.023 - 0.028)	0.025 (0.023 - 0.028)	0.002 (0.002 - 0.002)	0.002 (0.002 - 0.002)	0.002 (0.002 - 0.002)	0.638 (0.421 - 0.900)	0.718 (0.466 - 1.016)	0.722 (0.470 - 1.019)
50 - 69 years (rate per capita)	0.174 (0.159 - 0.191)	0.188 (0.172 - 0.205)	0.172 (0.156 - 0.188)	0.018 (0.016 - 0.020)	0.018 (0.016 - 0.021)	0.018 (0.016 - 0.020)	4.847 (3.212 - 6.857)	5.202 (3.467 - 7.316)	4.774 (3.173 - 6.745)
70+ years (rate per capita)	0.393 (0.360 - 0.426)	0.396 (0.363 - 0.431)	0.392 (0.360 - 0.426)	0.016 (0.013 - 0.019)	0.017 (0.014 - 0.021)	0.014 (0.012 - 0.017)	10.445 (6.968 - 14.553)	10.602 (7.075 - 14.717)	10.433 (6.960 - 14.549)
All ages (rate per capita)	0.053 (0.050 - 0.057)	0.073 (0.068 - 0.078)	0.084 (0.078 - 0.090)	0.004 (0.004 - 0.005)	0.005 (0.005 - 0.006)	0.006 (0.006 - 0.007)	1.480 (0.988 - 2.067)	2.001 (1.337 - 2.776)	2.302 (1.537 - 3.196)
Age-standardized (rate per capita)	0.071 (0.066 - 0.076)	0.070 (0.066 - 0.075)	0.071 (0.066 - 0.075)	0.005 (0.005 - 0.006)	0.005 (0.005 - 0.006)	0.005 (0.005 - 0.006)	1.941 (1.292 - 2.693)	1.936 (1.295 - 2.692)	1.941 (1.293 - 2.692)
Total number of cases (thousands)	189 (176 - 202)	220 (205 - 235)	252 (235 - 270)	16 (14 - 17)	16 (15 - 17)	19 (18 - 21)	5 (3 - 7)	6 (4 - 8)	7 (5 - 10)
**Azerbaijan**									
15 - 49 years (rate per capita)	0.021 (0.019 - 0.023)	0.024 (0.022 - 0.027)	0.025 (0.023 - 0.027)	0.002 (0.002 - 0.002)	0.002 (0.002 - 0.002)	0.002 (0.002 - 0.002)	0.593 (0.389 - 0.838)	0.704 (0.467 - 0.996)	0.716 (0.472 - 1.015)
50 - 69 years (rate per capita)	0.169 (0.153 - 0.185)	0.179 (0.163 - 0.195)	0.155 (0.141 - 0.171)	0.018 (0.015 - 0.020)	0.018 (0.015 - 0.020)	0.016 (0.014 - 0.019)	4.721 (3.110 - 6.684)	4.983 (3.286 - 7.053)	4.351 (2.858 - 6.126)
70+ years (rate per capita)	0.403 (0.368 - 0.440)	0.397 (0.362 - 0.434)	0.394 (0.361 - 0.430)	0.018 (0.015 - 0.021)	0.019 (0.016 - 0.023)	0.016 (0.014 - 0.019)	10.872 (7.259 - 15.203)	10.721 (7.131 - 15.039)	10.587 (7.073 - 14.785)
All ages (rate per capita)	0.043 (0.040 - 0.046)	0.049 (0.046 - 0.053)	0.060 (0.056 - 0.064)	0.004 (0.003 - 0.004)	0.004 (0.004 - 0.004)	0.005 (0.004 - 0.005)	1.200 (0.801 - 1.675)	1.380 (0.917 - 1.922)	1.665 (1.111 - 2.322)
Age-standardized (rate per capita)	0.071 (0.066 - 0.076)	0.070 (0.065 - 0.075)	0.070 (0.065 - 0.075)	0.005 (0.005 - 0.006)	0.005 (0.005 - 0.006)	0.005 (0.005 - 0.006)	1.953 (1.308 - 2.719)	1.942 (1.294 - 2.710)	1.940 (1.298 - 2.696)
Total number of cases (thousands)	307 (286 - 329)	429 (400 - 459)	585 (546 - 627)	26 (24 - 28)	33 (31 - 36)	48 (44 - 52)	9 (6 - 12)	12 (8 - 17)	16 (11 - 23)
**Georgia**									
15 - 49 years (rate per capita)	0.022 (0.021 - 0.024)	0.024 (0.022 - 0.026)	0.025 (0.023 - 0.026)	0.002 (0.002 - 0.002)	0.002 (0.002 - 0.002)	0.002 (0.002 - 0.002)	0.639 (0.418 - 0.898)	0.696 (0.456 - 0.975)	0.709 (0.466 - 0.998)
50 - 69 years (rate per capita)	0.167 (0.156 - 0.179)	0.179 (0.168 - 0.191)	0.163 (0.153 - 0.175)	0.017 (0.016 - 0.019)	0.018 (0.016 - 0.019)	0.017 (0.015 - 0.018)	4.686 (3.090 - 6.534)	4.990 (3.307 - 6.918)	4.565 (3.018 - 6.377)
70+ years (rate per capita)	0.368 (0.345 - 0.391)	0.371 (0.348 - 0.395)	0.363 (0.341 - 0.386)	0.015 (0.013 - 0.018)	0.016 (0.014 - 0.019)	0.014 (0.012 - 0.017)	9.956 (6.730 - 13.785)	10.044 (6.780 - 13.937)	9.760 (6.590 - 13.450)
All ages (rate per capita)	0.068 (0.064 - 0.071)	0.082 (0.078 - 0.087)	0.087 (0.083 - 0.091)	0.005 (0.005 - 0.006)	0.006 (0.006 - 0.006)	0.006 (0.006 - 0.007)	1.883 (1.249 - 2.617)	2.273 (1.524 - 3.149)	2.398 (1.609 - 3.313)
Age-standardized (rate per capita)	0.065 (0.062 - 0.069)	0.065 (0.062 - 0.069)	0.065 (0.062 - 0.069)	0.005 (0.005 - 0.005)	0.005 (0.005 - 0.005)	0.005 (0.005 - 0.005)	1.820 (1.214 - 2.523)	1.818 (1.221 - 2.516)	1.808 (1.214 - 2.500)
Total number of cases (thousands)	369 (351 - 389)	370 (353 - 390)	348 (331 - 366)	29 (27 - 31)	27 (25 - 29)	25 (23 - 26)	10 (7 - 14)	10 (7 - 14)	10 (6 - 13)
**Kazakhstan**									
15 - 49 years (rate per capita)	0.024 (0.022 - 0.026)	0.026 (0.025 - 0.028)	0.027 (0.025 - 0.029)	0.002 (0.002 - 0.002)	0.002 (0.002 - 0.002)	0.002 (0.002 - 0.002)	0.690 (0.455 - 0.980)	0.754 (0.495 - 1.055)	0.779 (0.515 - 1.091)
50 - 69 years (rate per capita)	0.184 (0.172 - 0.197)	0.191 (0.179 - 0.204)	0.177 (0.165 - 0.189)	0.019 (0.017 - 0.021)	0.019 (0.017 - 0.021)	0.019 (0.017 - 0.020)	5.144 (3.438 - 7.161)	5.320 (3.507 - 7.393)	4.950 (3.290 - 6.881)
70+ years (rate per capita)	0.425 (0.399 - 0.450)	0.426 (0.402 - 0.453)	0.428 (0.402 - 0.454)	0.017 (0.014 - 0.020)	0.018 (0.015 - 0.022)	0.018 (0.015 - 0.021)	11.458 (7.769 - 15.856)	11.480 (7.792 - 15.878)	11.515 (7.819 - 15.893)
All ages (rate per capita)	0.055 (0.052 - 0.057)	0.062 (0.059 - 0.065)	0.065 (0.062 - 0.068)	0.004 (0.004 - 0.005)	0.005 (0.005 - 0.005)	0.005 (0.005 - 0.005)	1.522 (1.021 - 2.094)	1.710 (1.145 - 2.365)	1.798 (1.204 - 2.480)
Age-standardized (rate per capita)	0.076 (0.072 - 0.079)	0.075 (0.072 - 0.079)	0.075 (0.072 - 0.079)	0.006 (0.005 - 0.006)	0.006 (0.005 - 0.006)	0.006 (0.005 - 0.006)	2.093 (1.408 - 2.879)	2.085 (1.395 - 2.879)	2.080 (1.398 - 2.860)
Total number of cases (thousands)	907 (864 - 950)	952 (911 - 999)	1,134 (1,081 - 1,189)	72 (68 - 77)	74 (70 - 78)	90 (85 - 96)	25 (17 - 35)	26 (18 - 37)	32 (21 - 43)
**Kyrgyzstan**									
15 - 49 years (rate per capita)	0.020 (0.018 - 0.022)	0.022 (0.020 - 0.025)	0.023 (0.021 - 0.025)	0.002 (0.001 - 0.002)	0.002 (0.002 - 0.002)	0.002 (0.002 - 0.002)	0.575 (0.373 - 0.811)	0.643 (0.417 - 0.905)	0.654 (0.429 - 0.926)
50 - 69 years (rate per capita)	0.177 (0.161 - 0.193)	0.173 (0.157 - 0.188)	0.160 (0.146 - 0.175)	0.018 (0.016 - 0.021)	0.017 (0.015 - 0.019)	0.017 (0.015 - 0.019)	4.937 (3.259 - 6.983)	4.821 (3.185 - 6.776)	4.489 (2.952 - 6.316)
70+ years (rate per capita)	0.393 (0.361 - 0.427)	0.395 (0.363 - 0.432)	0.391 (0.359 - 0.425)	0.016 (0.013 - 0.019)	0.017 (0.014 - 0.020)	0.016 (0.013 - 0.019)	10.573 (7.110 - 14.698)	10.663 (7.172 - 14.823)	10.520 (7.035 - 14.697)
All ages (rate per capita)	0.044 (0.041 - 0.046)	0.043 (0.040 - 0.046)	0.045 (0.042 - 0.048)	0.004 (0.003 - 0.004)	0.003 (0.003 - 0.004)	0.004 (0.003 - 0.004)	1.209 (0.804 - 1.687)	1.201 (0.801 - 1.663)	1.250 (0.833 - 1.735)
Age-standardized (rate per capita)	0.070 (0.066 - 0.075)	0.070 (0.065 - 0.075)	0.070 (0.065 - 0.075)	0.005 (0.005 - 0.006)	0.005 (0.005 - 0.006)	0.005 (0.005 - 0.006)	1.946 (1.298 - 2.710)	1.939 (1.286 - 2.682)	1.941 (1.293 - 2.690)
Total number of cases (thousands)	191 (179 - 205)	222 (208 - 236)	264 (247 - 281)	15 (14 - 17)	17 (16 - 18)	22 (20 - 24)	5 (4 - 7)	6 (4 - 9)	7 (5 - 10)
**Mongolia**									
15 - 49 years (rate per capita)	0.019 (0.017 - 0.021)	0.022 (0.020 - 0.024)	0.025 (0.022 - 0.027)	0.002 (0.001 - 0.002)	0.002 (0.002 - 0.002)	0.002 (0.002 - 0.002)	0.550 (0.362 - 0.778)	0.623 (0.409 - 0.879)	0.708 (0.466 - 1.002)
50 - 69 years (rate per capita)	0.176 (0.161 - 0.192)	0.171 (0.157 - 0.187)	0.158 (0.144 - 0.172)	0.018 (0.016 - 0.020)	0.017 (0.015 - 0.020)	0.016 (0.014 - 0.018)	4.932 (3.254 - 6.953)	4.800 (3.178 - 6.788)	4.421 (2.919 - 6.218)

	Prevalence 1990	Prevalence 2005	Prevalence 2015	Incidence 1990	Incidence 2005	Incidence 2015	YLD rate 1990	YLD rate 2005	YLD rate 2015
70+ years (rate per capita)	0.392 (0.358 - 0.428)	0.394 (0.360 - 0.431)	0.391 (0.357 - 0.426)	0.018 (0.015 - 0.022)	0.018 (0.015 - 0.022)	0.017 (0.014 - 0.021)	10.604 (7.076 - 14.888)	10.664 (7.091 - 15.011)	10.533 (7.009 - 14.813)
All ages (rate per capita)	0.035 (0.033 - 0.038)	0.037 (0.035 - 0.039)	0.044 (0.041 - 0.047)	0.003 (0.003 - 0.003)	0.003 (0.003 - 0.003)	0.004 (0.003 - 0.004)	0.986 (0.653 - 1.368)	1.034 (0.687 - 1.431)	1.235 (0.822 - 1.716)
Age-standardized (rate per capita)	0.070 (0.065 - 0.075)	0.070 (0.065 - 0.075)	0.070 (0.065 - 0.075)	0.005 (0.005 - 0.006)	0.005 (0.005 - 0.006)	0.005 (0.005 - 0.006)	1.934 (1.287 - 2.698)	1.933 (1.288 - 2.688)	1.932 (1.284 - 2.700)
Total number of cases (thousands)	77 (72 - 82)	93 (87 - 100)	130 (122 - 139)	6 (6 - 7)	8 (7 - 8)	11 (10 - 12)	2 (1 - 3)	3 (2 - 4)	4 (2 - 5)
**Tajikistan**									
15 - 49 years (rate per capita)	0.019 (0.017 - 0.021)	0.020 (0.019 - 0.023)	0.021 (0.019 - 0.024)	0.002 (0.001 - 0.002)	0.002 (0.002 - 0.002)	0.002 (0.002 - 0.002)	0.537 (0.353 - 0.761)	0.590 (0.388 - 0.835)	0.615 (0.405 - 0.873)
50 - 69 years (rate per capita)	0.174 (0.159 - 0.191)	0.175 (0.160 - 0.190)	0.155 (0.141 - 0.169)	0.018 (0.016 - 0.020)	0.017 (0.015 - 0.020)	0.016 (0.014 - 0.018)	4.871 (3.205 - 6.866)	4.876 (3.234 - 6.896)	4.338 (2.859 - 6.106)
70+ years (rate per capita)	0.389 (0.357 - 0.426)	0.393 (0.360 - 0.429)	0.387 (0.356 - 0.423)	0.016 (0.013 - 0.019)	0.018 (0.015 - 0.021)	0.016 (0.014 - 0.019)	10.479 (7.021 - 14.653)	10.599 (7.050 - 14.762)	10.427 (6.944 - 14.470)
All ages (rate per capita)	0.035 (0.032 - 0.037)	0.032 (0.030 - 0.034)	0.035 (0.033 - 0.038)	0.003 (0.003 - 0.003)	0.003 (0.002 - 0.003)	0.003 (0.003 - 0.003)	0.964 (0.641 - 1.334)	0.893 (0.596 - 1.232)	0.987 (0.657 - 1.367)
Age-standardized (rate per capita)	0.070 (0.065 - 0.075)	0.070 (0.065 - 0.075)	0.070 (0.065 - 0.075)	0.005 (0.005 - 0.006)	0.005 (0.005 - 0.006)	0.005 (0.005 - 0.006)	1.944 (1.300 - 2.709)	1.933 (1.290 - 2.691)	1.932 (1.284 - 2.681)
Total number of cases (thousands)	183 (171 - 196)	219 (206 - 234)	299 (281 - 320)	15 (14 - 16)	17 (16 - 19)	25 (23 - 27)	5 (3 - 7)	6 (4 - 8)	8 (6 - 12)
**Turkmenistan**									
15 - 49 years (rate per capita)	0.019 (0.017 - 0.021)	0.022 (0.020 - 0.024)	0.023 (0.021 - 0.026)	0.002 (0.001 - 0.002)	0.002 (0.002 - 0.002)	0.002 (0.002 - 0.002)	0.553 (0.360 - 0.777)	0.637 (0.415 - 0.898)	0.668 (0.432 - 0.939)
50 - 69 years (rate per capita)	0.174 (0.158 - 0.190)	0.171 (0.156 - 0.186)	0.162 (0.147 - 0.177)	0.018 (0.016 - 0.020)	0.017 (0.015 - 0.019)	0.017 (0.015 - 0.019)	4.868 (3.212 - 6.894)	4.780 (3.158 - 6.729)	4.541 (3.001 - 6.394)
70+ years (rate per capita)	0.395 (0.362 - 0.430)	0.396 (0.363 - 0.431)	0.393 (0.361 - 0.427)	0.017 (0.014 - 0.020)	0.018 (0.015 - 0.021)	0.016 (0.014 - 0.019)	10.679 (7.139 - 14.778)	10.723 (7.100 - 14.931)	10.582 (7.058 - 14.753)
All ages (rate per capita)	0.035 (0.033 - 0.038)	0.039 (0.037 - 0.042)	0.045 (0.042 - 0.048)	0.003 (0.003 - 0.003)	0.003 (0.003 - 0.003)	0.004 (0.003 - 0.004)	0.987 (0.657 - 1.372)	1.092 (0.724 - 1.517)	1.252 (0.832 - 1.741)
Age-standardized (rate per capita)	0.070 (0.065 - 0.075)	0.070 (0.065 - 0.075)	0.070 (0.065 - 0.075)	0.005 (0.005 - 0.006)	0.005 (0.005 - 0.006)	0.005 (0.005 - 0.006)	1.950 (1.297 - 2.690)	1.941 (1.287 - 2.673)	1.940 (1.294 - 2.690)
Total number of cases (thousands)	129 (121 - 138)	185 (173 - 197)	241 (225 - 256)	11 (10 - 11)	15 (13 - 16)	20 (18 - 22)	4 (2 - 5)	5 (3 - 7)	7 (4 - 9)
**Uzbekistan**									
15 - 49 years (rate per capita)	0.019 (0.017 - 0.021)	0.022 (0.020 - 0.024)	0.023 (0.021 - 0.025)	0.002 (0.001 - 0.002)	0.002 (0.002 - 0.002)	0.002 (0.002 - 0.002)	0.551 (0.361 - 0.782)	0.627 (0.417 - 0.883)	0.659 (0.430 - 0.928)
50 - 69 years (rate per capita)	0.173 (0.158 - 0.189)	0.174 (0.159 - 0.190)	0.163 (0.148 - 0.178)	0.018 (0.016 - 0.020)	0.017 (0.015 - 0.020)	0.017 (0.015 - 0.019)	4.842 (3.189 - 6.858)	4.863 (3.216 - 6.871)	4.560 (3.035 - 6.406)
70+ years (rate per capita)	0.389 (0.356 - 0.424)	0.394 (0.360 - 0.430)	0.390 (0.357 - 0.424)	0.016 (0.013 - 0.018)	0.017 (0.014 - 0.020)	0.016 (0.013 - 0.018)	10.480 (6.996 - 14.636)	10.650 (7.118 - 14.808)	10.492 (7.024 - 14.672)
All ages (rate per capita)	0.036 (0.034 - 0.038)	0.041 (0.038 - 0.044)	0.046 (0.044 - 0.050)	0.003 (0.003 - 0.003)	0.003 (0.003 - 0.003)	0.004 (0.003 - 0.004)	1.004 (0.670 - 1.401)	1.141 (0.761 - 1.583)	1.296 (0.864 - 1.800)
Age-standardized (rate per capita)	0.070 (0.066 - 0.075)	0.070 (0.066 - 0.075)	0.070 (0.066 - 0.075)	0.005 (0.005 - 0.006)	0.005 (0.005 - 0.006)	0.005 (0.005 - 0.006)	1.947 (1.295 - 2.722)	1.941 (1.296 - 2.704)	1.940 (1.297 - 2.701)
Total number of cases (thousands)	738 (693 - 790)	1,064 (1,001 - 1,138)	1,388 (1,303 - 1,483)	60 (55 - 65)	82 (77 - 89)	113 (104 - 122)	21 (14 - 29)	30 (20 - 41)	39 (26 - 54)
**Latin America and Caribbean**									
15 - 49 years (rate per capita)	0.014 (0.014 - 0.015)	0.016 (0.015 - 0.017)	0.017 (0.016 - 0.018)	0.001 (0.001 - 0.002)	0.002 (0.001 - 0.002)	0.002 (0.002 - 0.002)	0.416 (0.275 - 0.583)	0.465 (0.310 - 0.649)	0.489 (0.327 - 0.681)
50 - 69 years (rate per capita)	0.173 (0.165 - 0.183)	0.173 (0.165 - 0.182)	0.173 (0.164 - 0.182)	0.018 (0.017 - 0.019)	0.018 (0.017 - 0.019)	0.018 (0.017 - 0.019)	4.841 (3.233 - 6.704)	4.825 (3.235 - 6.675)	4.821 (3.219 - 6.669)
70+ years (rate per capita)	0.379 (0.359 - 0.399)	0.385 (0.365 - 0.406)	0.381 (0.362 - 0.401)	0.017 (0.015 - 0.019)	0.016 (0.014 - 0.018)	0.016 (0.014 - 0.018)	10.203 (6.896 - 14.039)	10.354 (7.011 - 14.255)	10.229 (6.922 - 14.037)
All ages (rate per capita)	0.034 (0.033 - 0.036)	0.044 (0.042 - 0.046)	0.054 (0.052 - 0.056)	0.003 (0.003 - 0.003)	0.004 (0.003 - 0.004)	0.004 (0.004 - 0.005)	0.950 (0.638 - 1.309)	1.218 (0.820 - 1.677)	1.494 (1.004 - 2.058)
Age-standardized (rate per capita)	0.065 (0.063 - 0.068)	0.066 (0.063 - 0.069)	0.066 (0.063 - 0.068)	0.005 (0.005 - 0.005)	0.005 (0.005 - 0.005)	0.005 (0.005 - 0.005)	1.794 (1.207 - 2.469)	1.821 (1.227 - 2.507)	1.811 (1.220 - 2.498)
Total number of cases (thousands)	13,620 (13,122 - 14,167)	22,193 (21,402 - 23,078)	30,744 (29,584 - 31,991)	1,152 (1,101 - 1,204)	1,847 (1,765 - 1,932)	2,528 (2,413 - 2,641)	379 (254 - 522)	616 (414 - 847)	851 (572 - 1,173)
**Central Latin America**									
15 - 49 years (rate per capita)	0.014 (0.013 - 0.015)	0.016 (0.015 - 0.017)	0.017 (0.016 - 0.018)	0.001 (0.001 - 0.001)	0.001 (0.001 - 0.002)	0.002 (0.001 - 0.002)	0.403 (0.268 - 0.566)	0.461 (0.307 - 0.645)	0.487 (0.324 - 0.681)
50 - 69 years (rate per capita)	0.159 (0.151 - 0.167)	0.153 (0.145 - 0.161)	0.155 (0.147 - 0.164)	0.016 (0.015 - 0.017)	0.015 (0.014 - 0.016)	0.015 (0.014 - 0.017)	4.434 (2.967 - 6.130)	4.270 (2.853 - 5.892)	4.338 (2.908 - 6.007)
70+ years (rate per capita)	0.348 (0.329 - 0.367)	0.346 (0.327 - 0.365)	0.344 (0.325 - 0.363)	0.015 (0.013 - 0.017)	0.015 (0.013 - 0.017)	0.014 (0.013 - 0.016)	9.358 (6.345 - 12.910)	9.300 (6.293 - 12.804)	9.229 (6.256 - 12.729)
All ages (rate per capita)	0.030 (0.029 - 0.031)	0.037 (0.036 - 0.039)	0.046 (0.044 - 0.048)	0.002 (0.002 - 0.003)	0.003 (0.003 - 0.003)	0.004 (0.003 - 0.004)	0.831 (0.557 - 1.144)	1.036 (0.696 - 1.424)	1.268 (0.851 - 1.745)
Age-standardized (rate per capita)	0.060 (0.058 - 0.063)	0.060 (0.058 - 0.063)	0.060 (0.058 - 0.063)	0.005 (0.004 - 0.005)	0.005 (0.004 - 0.005)	0.005 (0.004 - 0.005)	1.664 (1.119 - 2.289)	1.659 (1.117 - 2.282)	1.664 (1.119 - 2.289)
Total number of cases (thousands)	5,047 (4,856 - 5,257)	8,152 (7,842 - 8,487)	11,505 (11,057 - 11,997)	411 (393 - 431)	654 (624 - 686)	924 (881 - 969)	140 (94 - 193)	227 (152 - 312)	319 (214 - 440)
**Colombia**									
15 - 49 years (rate per capita)	0.015 (0.014 - 0.016)	0.017 (0.016 - 0.019)	0.018 (0.017 - 0.020)	0.001 (0.001 - 0.002)	0.002 (0.001 - 0.002)	0.002 (0.002 - 0.002)	0.426 (0.281 - 0.599)	0.491 (0.324 - 0.697)	0.523 (0.345 - 0.738)
50 - 69 years (rate per capita)	0.163 (0.153 - 0.175)	0.155 (0.144 - 0.166)	0.159 (0.149 - 0.171)	0.016 (0.015 - 0.018)	0.015 (0.014 - 0.017)	0.016 (0.014 - 0.017)	4.567 (3.012 - 6.349)	4.336 (2.855 - 6.036)	4.461 (2.950 - 6.184)
70+ years (rate per capita)	0.358 (0.333 - 0.383)	0.356 (0.331 - 0.380)	0.354 (0.330 - 0.379)	0.016 (0.013 - 0.019)	0.015 (0.013 - 0.018)	0.015 (0.013 - 0.018)	9.651 (6.521 - 13.364)	9.588 (6.490 - 13.247)	9.529 (6.455 - 13.175)
All ages (rate per capita)	0.031 (0.030 - 0.033)	0.041 (0.038 - 0.043)	0.052 (0.050 - 0.055)	0.003 (0.002 - 0.003)	0.003 (0.003 - 0.004)	0.004 (0.004 - 0.005)	0.872 (0.581 - 1.207)	1.130 (0.752 - 1.566)	1.456 (0.971 - 2.006)
Age-standardized (rate per capita)	0.062 (0.059 - 0.065)	0.062 (0.059 - 0.065)	0.062 (0.059 - 0.065)	0.005 (0.004 - 0.005)	0.005 (0.004 - 0.005)	0.005 (0.004 - 0.005)	1.709 (1.137 - 2.362)	1.711 (1.141 - 2.370)	1.709 (1.143 - 2.357)
Total number of cases (thousands)	1,074 (1,020 - 1,131)	1,754 (1,663 - 1,848)	2,525 (2,396 - 2,664)	87 (82 - 93)	144 (135 - 152)	207 (194 - 220)	30 (20 - 41)	49 (33 - 68)	70 (47 - 97)
**Costa Rica**									
15 - 49 years (rate per capita)	0.016 (0.015 - 0.017)	0.018 (0.016 - 0.019)	0.018 (0.017 - 0.020)	0.001 (0.001 - 0.002)	0.002 (0.002 - 0.002)	0.002 (0.002 - 0.002)	0.461 (0.302 - 0.651)	0.505 (0.334 - 0.712)	0.522 (0.346 - 0.737)
50 - 69 years (rate per capita)	0.161 (0.150 - 0.173)	0.160 (0.149 - 0.172)	0.161 (0.151 - 0.173)	0.016 (0.014 - 0.018)	0.016 (0.014 - 0.017)	0.016 (0.014 - 0.017)	4.532 (2.991 - 6.286)	4.500 (2.973 - 6.265)	4.530 (2.986 - 6.298)
70+ years (rate per capita)	0.357 (0.333 - 0.382)	0.355 (0.332 - 0.380)	0.353 (0.330 - 0.378)	0.015 (0.013 - 0.018)	0.015 (0.013 - 0.017)	0.015 (0.013 - 0.017)	9.666 (6.544 - 13.394)	9.596 (6.473 - 13.304)	9.510 (6.424 - 13.180)
All ages (rate per capita)	0.036 (0.034 - 0.038)	0.047 (0.044 - 0.049)	0.059 (0.057 - 0.063)	0.003 (0.003 - 0.003)	0.004 (0.004 - 0.004)	0.005 (0.004 - 0.005)	1.000 (0.665 - 1.384)	1.301 (0.868 - 1.794)	1.654 (1.100 - 2.283)
Age-standardized (rate per capita)	0.062 (0.059 - 0.065)	0.062 (0.059 - 0.065)	0.062 (0.059 - 0.065)	0.005 (0.004 - 0.005)	0.005 (0.004 - 0.005)	0.005 (0.004 - 0.005)	1.718 (1.147 - 2.369)	1.717 (1.147 - 2.366)	1.715 (1.145 - 2.366)
Total number of cases (thousands)	111 (105 - 116)	198 (189 - 209)	286 (272 - 301)	9 (8 - 10)	16 (15 - 17)	23 (21 - 24)	3 (2 - 4)	6 (4 - 8)	8 (5 - 11)
**El Salvador**									
15 - 49 years (rate per capita)	0.015 (0.014 - 0.016)	0.016 (0.015 - 0.018)	0.017 (0.015 - 0.018)	0.001 (0.001 - 0.002)	0.002 (0.001 - 0.002)	0.002 (0.001 - 0.002)	0.429 (0.286 - 0.606)	0.464 (0.307 - 0.653)	0.473 (0.314 - 0.667)
50 - 69 years (rate per capita)	0.159 (0.147 - 0.171)	0.166 (0.154 - 0.178)	0.166 (0.154 - 0.178)	0.016 (0.014 - 0.017)	0.016 (0.015 - 0.018)	0.016 (0.015 - 0.018)	4.433 (2.969 - 6.177)	4.620 (3.085 - 6.387)	4.617 (3.088 - 6.408)
70+ years (rate per capita)	0.359 (0.333 - 0.385)	0.356 (0.331 - 0.381)	0.354 (0.329 - 0.380)	0.016 (0.014 - 0.019)	0.016 (0.013 - 0.018)	0.015 (0.013 - 0.017)	9.648 (6.468 - 13.371)	9.543 (6.442 - 13.227)	9.476 (6.417 - 13.109)
All ages (rate per capita)	0.033 (0.031 - 0.034)	0.043 (0.041 - 0.045)	0.052 (0.050 - 0.055)	0.003 (0.003 - 0.003)	0.003 (0.003 - 0.004)	0.004 (0.004 - 0.004)	0.906 (0.608 - 1.256)	1.188 (0.797 - 1.639)	1.441 (0.965 - 1.996)
Age-standardized (rate per capita)	0.062 (0.058 - 0.065)	0.062 (0.059 - 0.065)	0.062 (0.059 - 0.066)	0.005 (0.004 - 0.005)	0.005 (0.004 - 0.005)	0.005 (0.004 - 0.005)	1.696 (1.138 - 2.345)	1.704 (1.147 - 2.355)	1.713 (1.146 - 2.370)
Total number of cases (thousands)	173 (164 - 182)	256 (243 - 269)	321 (305 - 338)	14 (13 - 15)	20 (19 - 22)	25 (23 - 26)	5 (3 - 7)	7 (5 - 10)	9 (6 - 12)
**Guatemala**									
15 - 49 years (rate per capita)	0.015 (0.014 - 0.016)	0.015 (0.014 - 0.016)	0.015 (0.015 - 0.016)	0.001 (0.001 - 0.001)	0.001 (0.001 - 0.002)	0.001 (0.001 - 0.002)	0.423 (0.281 - 0.587)	0.427 (0.286 - 0.599)	0.443 (0.294 - 0.617)
50 - 69 years (rate per capita)	0.164 (0.155 - 0.174)	0.166 (0.156 - 0.175)	0.171 (0.161 - 0.180)	0.016 (0.015 - 0.018)	0.016 (0.015 - 0.018)	0.017 (0.016 - 0.018)	4.568 (3.063 - 6.304)	4.604 (3.098 - 6.407)	4.728 (3.191 - 6.538)
70+ years (rate per capita)	0.364 (0.346 - 0.384)	0.366 (0.347 - 0.384)	0.363 (0.345 - 0.382)	0.017 (0.014 - 0.019)	0.016 (0.014 - 0.018)	0.015 (0.013 - 0.017)	9.764 (6.606 - 13.379)	9.771 (6.637 - 13.377)	9.664 (6.546 - 13.295)
All ages (rate per capita)	0.027 (0.026 - 0.029)	0.032 (0.030 - 0.033)	0.035 (0.034 - 0.037)	0.002 (0.002 - 0.002)	0.003 (0.002 - 0.003)	0.003 (0.003 - 0.003)	0.763 (0.514 - 1.047)	0.873 (0.592 - 1.201)	0.971 (0.653 - 1.337)
Age-standardized (rate per capita)	0.063 (0.061 - 0.066)	0.064 (0.061 - 0.066)	0.064 (0.061 - 0.066)	0.005 (0.005 - 0.005)	0.005 (0.005 - 0.005)	0.005 (0.005 - 0.005)	1.733 (1.171 - 2.377)	1.749 (1.188 - 2.401)	1.751 (1.182 - 2.413)
Total number of cases (thousands)	253 (243 - 263)	417 (401 - 434)	576 (554 - 598)	21 (20 - 23)	34 (32 - 35)	46 (44 - 48)	7 (5 - 10)	12 (8 - 16)	16 (11 - 22)
**Honduras**									
15 - 49 years (rate per capita)	0.013 (0.013 - 0.015)	0.014 (0.013 - 0.016)	0.015 (0.014 - 0.016)	0.001 (0.001 - 0.001)	0.001 (0.001 - 0.001)	0.001 (0.001 - 0.002)	0.387 (0.258 - 0.544)	0.413 (0.275 - 0.573)	0.435 (0.287 - 0.612)
50 - 69 years (rate per capita)	0.160 (0.149 - 0.172)	0.155 (0.145 - 0.167)	0.156 (0.145 - 0.168)	0.016 (0.014 - 0.017)	0.016 (0.014 - 0.017)	0.016 (0.014 - 0.017)	4.479 (2.980 - 6.253)	4.344 (2.882 - 6.073)	4.365 (2.896 - 6.090)
70+ years (rate per capita)	0.355 (0.330 - 0.379)	0.351 (0.327 - 0.375)	0.348 (0.324 - 0.371)	0.016 (0.013 - 0.018)	0.015 (0.013 - 0.018)	0.014 (0.012 - 0.017)	9.583 (6.449 - 13.256)	9.455 (6.375 - 13.088)	9.344 (6.319 - 12.929)
All ages (rate per capita)	0.025 (0.024 - 0.027)	0.030 (0.028 - 0.031)	0.036 (0.034 - 0.037)	0.002 (0.002 - 0.002)	0.002 (0.002 - 0.003)	0.003 (0.003 - 0.003)	0.709 (0.476 - 0.981)	0.824 (0.553 - 1.143)	0.992 (0.662 - 1.379)
Age-standardized (rate per capita)	0.061 (0.058 - 0.064)	0.061 (0.058 - 0.064)	0.061 (0.058 - 0.064)	0.005 (0.004 - 0.005)	0.005 (0.004 - 0.005)	0.005 (0.004 - 0.005)	1.686 (1.128 - 2.334)	1.682 (1.127 - 2.339)	1.683 (1.121 - 2.327)
Total number of cases (thousands)	125 (119 - 131)	204 (194 - 214)	289 (274 - 303)	10 (10 - 11)	16 (15 - 17)	23 (22 - 25)	3 (2 - 5)	6 (4 - 8)	8 (5 - 11)
**Mexico**									

	Prevalence 1990	Prevalence 2005	Prevalence 2015	Incidence 1990	Incidence 2005	Incidence 2015	YLD rate 1990	YLD rate 2005	YLD rate 2015
15 - 49 years (rate per capita)	0.013 (0.013 - 0.014)	0.016 (0.015 - 0.017)	0.017 (0.016 - 0.018)	0.001 (0.001 - 0.001)	0.001 (0.001 - 0.002)	0.002 (0.001 - 0.002)	0.383 (0.255 - 0.537)	0.451 (0.300 - 0.632)	0.478 (0.318 - 0.671)
50 - 69 years (rate per capita)	0.156 (0.150 - 0.163)	0.148 (0.141 - 0.154)	0.151 (0.145 - 0.158)	0.016 (0.015 - 0.016)	0.015 (0.014 - 0.016)	0.015 (0.014 - 0.016)	4.352 (2.926 - 6.012)	4.120 (2.764 - 5.690)	4.207 (2.825 - 5.813)
70+ years (rate per capita)	0.339 (0.324 - 0.355)	0.337 (0.321 - 0.352)	0.334 (0.319 - 0.349)	0.015 (0.013 - 0.016)	0.015 (0.013 - 0.016)	0.014 (0.013 - 0.015)	9.104 (6.154 - 12.487)	9.048 (6.118 - 12.431)	8.955 (6.056 - 12.241)
All ages (rate per capita)	0.030 (0.029 - 0.031)	0.036 (0.035 - 0.038)	0.044 (0.043 - 0.046)	0.002 (0.002 - 0.002)	0.003 (0.003 - 0.003)	0.004 (0.003 - 0.004)	0.820 (0.553 - 1.131)	1.012 (0.682 - 1.394)	1.226 (0.827 - 1.690)
Age-standardized (rate per capita)	0.059 (0.057 - 0.061)	0.058 (0.056 - 0.060)	0.059 (0.057 - 0.061)	0.004 (0.004 - 0.005)	0.004 (0.004 - 0.005)	0.004 (0.004 - 0.005)	1.623 (1.097 - 2.241)	1.609 (1.088 - 2.221)	1.618 (1.094 - 2.231)
Total number of cases (thousands)	2,530 (2,452 - 2,617)	3,997 (3,874 - 4,135)	5,616 (5,441 - 5,812)	204 (196 - 212)	316 (304 - 328)	448 (430 - 466)	70 (47 - 97)	111 (75 - 153)	156 (105 - 215)
**Nicaragua**									
15 - 49 years (rate per capita)	0.014 (0.013 - 0.015)	0.015 (0.014 - 0.016)	0.016 (0.015 - 0.017)	0.001 (0.001 - 0.001)	0.001 (0.001 - 0.002)	0.001 (0.001 - 0.002)	0.393 (0.261 - 0.552)	0.429 (0.285 - 0.603)	0.465 (0.309 - 0.655)
50 - 69 years (rate per capita)	0.164 (0.153 - 0.176)	0.160 (0.149 - 0.172)	0.158 (0.148 - 0.170)	0.016 (0.015 - 0.018)	0.016 (0.014 - 0.017)	0.016 (0.014 - 0.017)	4.590 (3.047 - 6.410)	4.472 (2.972 - 6.229)	4.432 (2.949 - 6.175)
70+ years (rate per capita)	0.363 (0.339 - 0.388)	0.358 (0.334 - 0.382)	0.354 (0.331 - 0.378)	0.016 (0.014 - 0.019)	0.015 (0.013 - 0.018)	0.015 (0.012 - 0.017)	9.823 (6.616 - 13.652)	9.640 (6.498 - 13.311)	9.512 (6.428 - 13.205)
All ages (rate per capita)	0.025 (0.024 - 0.027)	0.032 (0.030 - 0.033)	0.040 (0.038 - 0.042)	0.002 (0.002 - 0.002)	0.003 (0.002 - 0.003)	0.003 (0.003 - 0.003)	0.710 (0.476 - 0.983)	0.883 (0.592 - 1.221)	1.106 (0.740 - 1.533)
Age-standardized (rate per capita)	0.062 (0.059 - 0.066)	0.062 (0.059 - 0.066)	0.062 (0.059 - 0.066)	0.005 (0.004 - 0.005)	0.005 (0.004 - 0.005)	0.005 (0.004 - 0.005)	1.723 (1.157 - 2.393)	1.721 (1.155 - 2.387)	1.723 (1.152 - 2.384)
Total number of cases (thousands)	106 (100 - 111)	171 (162 - 180)	242 (230 - 255)	9 (8 - 9)	14 (13 - 15)	19 (18 - 21)	3 (2 - 4)	5 (3 - 7)	7 (5 - 9)
**Panama**									
15 - 49 years (rate per capita)	0.015 (0.014 - 0.016)	0.017 (0.016 - 0.018)	0.018 (0.017 - 0.019)	0.001 (0.001 - 0.002)	0.002 (0.001 - 0.002)	0.002 (0.002 - 0.002)	0.430 (0.285 - 0.604)	0.488 (0.320 - 0.688)	0.516 (0.345 - 0.723)
50 - 69 years (rate per capita)	0.161 (0.150 - 0.172)	0.161 (0.150 - 0.172)	0.160 (0.150 - 0.172)	0.016 (0.014 - 0.017)	0.016 (0.014 - 0.017)	0.016 (0.014 - 0.017)	4.510 (2.991 - 6.334)	4.495 (2.987 - 6.310)	4.483 (2.973 - 6.270)
70+ years (rate per capita)	0.354 (0.330 - 0.379)	0.351 (0.327 - 0.376)	0.351 (0.327 - 0.376)	0.015 (0.013 - 0.018)	0.015 (0.013 - 0.017)	0.014 (0.012 - 0.017)	9.559 (6.424 - 13.257)	9.431 (6.312 - 13.020)	9.389 (6.306 - 12.999)
All ages (rate per capita)	0.034 (0.033 - 0.036)	0.043 (0.041 - 0.045)	0.052 (0.049 - 0.054)	0.003 (0.003 - 0.003)	0.003 (0.003 - 0.004)	0.004 (0.004 - 0.004)	0.955 (0.634 - 1.327)	1.193 (0.795 - 1.654)	1.433 (0.956 - 1.981)
Age-standardized (rate per capita)	0.061 (0.058 - 0.065)	0.062 (0.058 - 0.065)	0.062 (0.059 - 0.065)	0.005 (0.004 - 0.005)	0.005 (0.004 - 0.005)	0.005 (0.004 - 0.005)	1.705 (1.135 - 2.378)	1.704 (1.138 - 2.364)	1.704 (1.136 - 2.359)
Total number of cases (thousands)	85 (80 - 89)	143 (136 - 150)	203 (193 - 214)	7 (6 - 7)	11 (11 - 12)	16 (15 - 17)	2 (2 - 3)	4 (3 - 5)	6 (4 - 8)
**Venezuela**									
15 - 49 years (rate per capita)	0.015 (0.014 - 0.016)	0.017 (0.015 - 0.018)	0.017 (0.016 - 0.019)	0.001 (0.001 - 0.002)	0.002 (0.001 - 0.002)	0.002 (0.001 - 0.002)	0.429 (0.280 - 0.603)	0.477 (0.317 - 0.670)	0.500 (0.330 - 0.704)
50 - 69 years (rate per capita)	0.158 (0.147 - 0.169)	0.157 (0.147 - 0.169)	0.156 (0.145 - 0.167)	0.016 (0.014 - 0.017)	0.016 (0.014 - 0.017)	0.016 (0.014 - 0.017)	4.435 (2.917 - 6.163)	4.410 (2.894 - 6.109)	4.359 (2.874 - 6.094)
70+ years (rate per capita)	0.355 (0.331 - 0.382)	0.354 (0.330 - 0.381)	0.354 (0.330 - 0.381)	0.016 (0.014 - 0.018)	0.015 (0.013 - 0.018)	0.015 (0.013 - 0.018)	9.608 (6.491 - 13.338)	9.548 (6.460 - 13.166)	9.528 (6.429 - 13.147)
All ages (rate per capita)	0.030 (0.028 - 0.031)	0.038 (0.036 - 0.040)	0.047 (0.044 - 0.049)	0.002 (0.002 - 0.003)	0.003 (0.003 - 0.003)	0.004 (0.004 - 0.004)	0.835 (0.553 - 1.150)	1.055 (0.704 - 1.456)	1.295 (0.860 - 1.783)
Age-standardized (rate per capita)	0.061 (0.058 - 0.065)	0.061 (0.058 - 0.065)	0.061 (0.058 - 0.065)	0.005 (0.004 - 0.005)	0.005 (0.004 - 0.005)	0.005 (0.004 - 0.005)	1.701 (1.127 - 2.340)	1.701 (1.130 - 2.345)	1.700 (1.126 - 2.343)
Total number of cases (thousands)	592 (563 - 624)	1,012 (963 - 1,068)	1,448 (1,377 - 1,529)	49 (47 - 53)	83 (79 - 89)	118 (111 - 126)	17 (11 - 23)	28 (19 - 39)	40 (27 - 55)
**Andean Latin America**									
15 - 49 years (rate per capita)	0.014 (0.013 - 0.015)	0.016 (0.015 - 0.017)	0.017 (0.016 - 0.018)	0.001 (0.001 - 0.001)	0.001 (0.001 - 0.002)	0.002 (0.001 - 0.002)	0.414 (0.275 - 0.581)	0.450 (0.299 - 0.632)	0.478 (0.316 - 0.666)
50 - 69 years (rate per capita)	0.166 (0.155 - 0.178)	0.165 (0.154 - 0.176)	0.166 (0.155 - 0.177)	0.017 (0.016 - 0.019)	0.017 (0.015 - 0.018)	0.017 (0.016 - 0.019)	4.637 (3.088 - 6.460)	4.619 (3.091 - 6.401)	4.632 (3.074 - 6.432)
70+ years (rate per capita)	0.371 (0.348 - 0.395)	0.367 (0.344 - 0.391)	0.364 (0.342 - 0.387)	0.017 (0.014 - 0.019)	0.016 (0.013 - 0.018)	0.015 (0.013 - 0.018)	9.968 (6.764 - 13.757)	9.865 (6.655 - 13.660)	9.767 (6.602 - 13.501)
All ages (rate per capita)	0.032 (0.031 - 0.034)	0.040 (0.038 - 0.042)	0.048 (0.045 - 0.050)	0.003 (0.003 - 0.003)	0.003 (0.003 - 0.003)	0.004 (0.004 - 0.004)	0.892 (0.597 - 1.230)	1.114 (0.747 - 1.536)	1.320 (0.884 - 1.823)
Age-standardized (rate per capita)	0.063 (0.060 - 0.067)	0.063 (0.060 - 0.066)	0.063 (0.060 - 0.067)	0.005 (0.005 - 0.005)	0.005 (0.005 - 0.005)	0.005 (0.005 - 0.005)	1.743 (1.171 - 2.410)	1.744 (1.170 - 2.407)	1.745 (1.171 - 2.406)
Total number of cases (thousands)	1,249 (1,192 - 1,313)	2,028 (1,934 - 2,128)	2,775 (2,643 - 2,918)	105 (99 - 111)	164 (155 - 174)	223 (210 - 237)	35 (23 - 48)	56 (38 - 78)	77 (52 - 106)
**Bolivia**									
15 - 49 years (rate per capita)	0.015 (0.014 - 0.016)	0.015 (0.014 - 0.016)	0.016 (0.014 - 0.017)	0.001 (0.001 - 0.002)	0.001 (0.001 - 0.002)	0.001 (0.001 - 0.002)	0.417 (0.275 - 0.588)	0.427 (0.281 - 0.599)	0.447 (0.293 - 0.625)
50 - 69 years (rate per capita)	0.170 (0.157 - 0.184)	0.166 (0.153 - 0.179)	0.167 (0.155 - 0.181)	0.017 (0.016 - 0.019)	0.017 (0.015 - 0.019)	0.017 (0.015 - 0.019)	4.738 (3.144 - 6.621)	4.623 (3.081 - 6.460)	4.668 (3.080 - 6.519)
70+ years (rate per capita)	0.372 (0.345 - 0.399)	0.368 (0.341 - 0.393)	0.362 (0.336 - 0.388)	0.017 (0.014 - 0.020)	0.016 (0.013 - 0.019)	0.015 (0.013 - 0.018)	9.978 (6.719 - 13.742)	9.841 (6.655 - 13.533)	9.668 (6.552 - 13.309)
All ages (rate per capita)	0.033 (0.031 - 0.034)	0.038 (0.036 - 0.040)	0.043 (0.041 - 0.046)	0.003 (0.003 - 0.003)	0.003 (0.003 - 0.003)	0.003 (0.003 - 0.004)	0.902 (0.602 - 1.254)	1.040 (0.695 - 1.444)	1.198 (0.797 - 1.661)
Age-standardized (rate per capita)	0.063 (0.059 - 0.067)	0.063 (0.059 - 0.067)	0.063 (0.059 - 0.067)	0.005 (0.005 - 0.005)	0.005 (0.005 - 0.005)	0.005 (0.005 - 0.005)	1.729 (1.159 - 2.400)	1.733 (1.155 - 2.406)	1.732 (1.151 - 2.407)
Total number of cases (thousands)	224 (212 - 237)	344 (326 - 364)	467 (442 - 494)	19 (18 - 20)	28 (26 - 30)	37 (34 - 39)	6 (4 - 9)	10 (6 - 13)	13 (9 - 18)
**Ecuador**									
15 - 49 years (rate per capita)	0.014 (0.014 - 0.015)	0.016 (0.015 - 0.017)	0.016 (0.016 - 0.017)	0.001 (0.001 - 0.001)	0.001 (0.001 - 0.002)	0.002 (0.001 - 0.002)	0.411 (0.274 - 0.577)	0.454 (0.301 - 0.635)	0.474 (0.315 - 0.662)
50 - 69 years (rate per capita)	0.165 (0.156 - 0.176)	0.163 (0.154 - 0.172)	0.165 (0.155 - 0.175)	0.017 (0.016 - 0.018)	0.017 (0.015 - 0.018)	0.017 (0.016 - 0.018)	4.612 (3.068 - 6.454)	4.543 (3.054 - 6.298)	4.595 (3.039 - 6.417)
70+ years (rate per capita)	0.368 (0.350 - 0.387)	0.362 (0.343 - 0.383)	0.364 (0.345 - 0.383)	0.016 (0.014 - 0.018)	0.015 (0.013 - 0.017)	0.015 (0.013 - 0.018)	9.879 (6.681 - 13.480)	9.702 (6.587 - 13.341)	9.717 (6.567 - 13.405)
All ages (rate per capita)	0.032 (0.031 - 0.033)	0.040 (0.038 - 0.042)	0.048 (0.046 - 0.050)	0.003 (0.003 - 0.003)	0.003 (0.003 - 0.003)	0.004 (0.004 - 0.004)	0.891 (0.599 - 1.237)	1.108 (0.746 - 1.527)	1.324 (0.888 - 1.830)
Age-standardized (rate per capita)	0.063 (0.060 - 0.066)	0.063 (0.060 - 0.065)	0.063 (0.060 - 0.066)	0.005 (0.005 - 0.005)	0.005 (0.005 - 0.005)	0.005 (0.005 - 0.005)	1.737 (1.170 - 2.400)	1.734 (1.169 - 2.389)	1.734 (1.166 - 2.393)
Total number of cases (thousands)	329 (316 - 343)	550 (529 - 571)	774 (743 - 808)	27 (26 - 28)	44 (42 - 46)	62 (59 - 66)	9 (6 - 13)	15 (10 - 21)	21 (14 - 30)
**Peru**									
15 - 49 years (rate per capita)	0.014 (0.013 - 0.016)	0.016 (0.015 - 0.017)	0.017 (0.016 - 0.018)	0.001 (0.001 - 0.001)	0.001 (0.001 - 0.002)	0.002 (0.001 - 0.002)	0.415 (0.275 - 0.583)	0.456 (0.300 - 0.645)	0.490 (0.324 - 0.686)
50 - 69 years (rate per capita)	0.165 (0.153 - 0.178)	0.166 (0.154 - 0.179)	0.166 (0.153 - 0.179)	0.017 (0.015 - 0.019)	0.017 (0.015 - 0.019)	0.017 (0.015 - 0.019)	4.616 (3.078 - 6.468)	4.655 (3.104 - 6.473)	4.641 (3.080 - 6.476)
70+ years (rate per capita)	0.372 (0.346 - 0.398)	0.370 (0.345 - 0.395)	0.365 (0.341 - 0.390)	0.017 (0.014 - 0.020)	0.016 (0.014 - 0.019)	0.015 (0.013 - 0.018)	10.012 (6.765 - 13.869)	9.953 (6.705 - 13.791)	9.825 (6.620 - 13.580)
All ages (rate per capita)	0.032 (0.030 - 0.034)	0.041 (0.039 - 0.043)	0.049 (0.046 - 0.052)	0.003 (0.003 - 0.003)	0.003 (0.003 - 0.004)	0.004 (0.004 - 0.004)	0.889 (0.593 - 1.231)	1.141 (0.764 - 1.577)	1.359 (0.909 - 1.882)
Age-standardized (rate per capita)	0.063 (0.060 - 0.067)	0.063 (0.060 - 0.067)	0.063 (0.060 - 0.067)	0.005 (0.005 - 0.005)	0.005 (0.005 - 0.005)	0.005 (0.005 - 0.005)	1.751 (1.175 - 2.433)	1.753 (1.173 - 2.419)	1.754 (1.176 - 2.424)
Total number of cases (thousands)	697 (661 - 736)	1,134 (1,076 - 1,196)	1,534 (1,455 - 1,623)	59 (55 - 63)	92 (86 - 98)	124 (116 - 132)	19 (13 - 27)	32 (21 - 44)	43 (29 - 59)
**Caribbean**									
15 - 49 years (rate per capita)	0.012 (0.011 - 0.013)	0.013 (0.012 - 0.014)	0.014 (0.013 - 0.015)	0.001 (0.001 - 0.001)	0.001 (0.001 - 0.001)	0.001 (0.001 - 0.002)	0.338 (0.225 - 0.473)	0.383 (0.255 - 0.534)	0.400 (0.267 - 0.561)
50 - 69 years (rate per capita)	0.141 (0.132 - 0.152)	0.140 (0.132 - 0.151)	0.138 (0.129 - 0.148)	0.014 (0.013 - 0.016)	0.014 (0.013 - 0.016)	0.014 (0.013 - 0.015)	3.944 (2.605 - 5.530)	3.918 (2.611 - 5.460)	3.843 (2.546 - 5.379)
70+ years (rate per capita)	0.311 (0.291 - 0.332)	0.310 (0.290 - 0.331)	0.309 (0.289 - 0.330)	0.013 (0.011 - 0.016)	0.013 (0.011 - 0.015)	0.013 (0.011 - 0.015)	8.391 (5.674 - 11.588)	8.330 (5.622 - 11.519)	8.276 (5.609 - 11.418)
All ages (rate per capita)	0.036 (0.034 - 0.038)	0.043 (0.041 - 0.045)	0.050 (0.048 - 0.053)	0.003 (0.003 - 0.003)	0.003 (0.003 - 0.004)	0.004 (0.004 - 0.004)	1.002 (0.668 - 1.381)	1.183 (0.791 - 1.636)	1.387 (0.927 - 1.919)
Age-standardized (rate per capita)	0.053 (0.050 - 0.056)	0.053 (0.050 - 0.056)	0.053 (0.050 - 0.056)	0.004 (0.004 - 0.004)	0.004 (0.004 - 0.004)	0.004 (0.004 - 0.004)	1.454 (0.970 - 2.008)	1.454 (0.971 - 2.012)	1.450 (0.969 - 2.008)
Total number of cases (thousands)	1,294 (1,233 - 1,363)	1,790 (1,707 - 1,885)	2,278 (2,173 - 2,401)	103 (97 - 109)	141 (133 - 150)	180 (169 - 190)	36 (24 - 49)	49 (33 - 68)	63 (42 - 87)
**Antigua and Barbuda**									
15 - 49 years (rate per capita)	0.012 (0.011 - 0.013)	0.014 (0.013 - 0.016)	0.016 (0.014 - 0.017)	0.001 (0.001 - 0.001)	0.001 (0.001 - 0.002)	0.002 (0.001 - 0.002)	0.341 (0.223 - 0.480)	0.413 (0.271 - 0.580)	0.444 (0.293 - 0.626)
50 - 69 years (rate per capita)	0.154 (0.143 - 0.166)	0.140 (0.130 - 0.151)	0.130 (0.120 - 0.140)	0.015 (0.014 - 0.017)	0.014 (0.013 - 0.016)	0.013 (0.012 - 0.015)	4.286 (2.853 - 5.985)	3.906 (2.592 - 5.462)	3.616 (2.406 - 5.025)
70+ years (rate per capita)	0.315 (0.293 - 0.339)	0.306 (0.285 - 0.330)	0.312 (0.290 - 0.337)	0.014 (0.012 - 0.016)	0.012 (0.010 - 0.014)	0.013 (0.011 - 0.015)	8.506 (5.709 - 11.772)	8.218 (5.543 - 11.343)	8.373 (5.597 - 11.525)
All ages (rate per capita)	0.042 (0.040 - 0.044)	0.040 (0.038 - 0.042)	0.046 (0.044 - 0.049)	0.003 (0.003 - 0.003)	0.003 (0.003 - 0.003)	0.004 (0.004 - 0.004)	1.155 (0.770 - 1.605)	1.109 (0.740 - 1.538)	1.280 (0.854 - 1.771)
Age-standardized (rate per capita)	0.053 (0.050 - 0.056)	0.053 (0.050 - 0.056)	0.053 (0.050 - 0.056)	0.004 (0.004 - 0.004)	0.004 (0.004 - 0.004)	0.004 (0.004 - 0.004)	1.464 (0.974 - 2.030)	1.453 (0.968 - 2.017)	1.456 (0.973 - 2.015)
Total number of cases (thousands)	3 (2 - 3)	3 (3 - 4)	4 (4 - 4)	0 (0 - 0)	0 (0 - 0)	0 (0 - 0)	0 (0 - 0)	0 (0 - 0)	0 (0 - 0)
**The Bahamas**									
15 - 49 years (rate per capita)	0.012 (0.011 - 0.013)	0.014 (0.013 - 0.015)	0.014 (0.013 - 0.016)	0.001 (0.001 - 0.001)	0.001 (0.001 - 0.002)	0.001 (0.001 - 0.002)	0.336 (0.220 - 0.475)	0.404 (0.266 - 0.567)	0.411 (0.273 - 0.578)
50 - 69 years (rate per capita)	0.135 (0.125 - 0.146)	0.135 (0.125 - 0.146)	0.136 (0.126 - 0.147)	0.014 (0.013 - 0.015)	0.014 (0.013 - 0.015)	0.014 (0.013 - 0.015)	3.746 (2.487 - 5.270)	3.742 (2.495 - 5.249)	3.774 (2.506 - 5.281)
70+ years (rate per capita)	0.316 (0.294 - 0.341)	0.314 (0.291 - 0.338)	0.313 (0.291 - 0.337)	0.014 (0.012 - 0.016)	0.014 (0.012 - 0.016)	0.013 (0.011 - 0.016)	8.503 (5.727 - 11.767)	8.404 (5.689 - 11.608)	8.369 (5.642 - 11.567)
All ages (rate per capita)	0.029 (0.027 - 0.031)	0.041 (0.038 - 0.043)	0.051 (0.048 - 0.054)	0.002 (0.002 - 0.003)	0.003 (0.003 - 0.004)	0.004 (0.004 - 0.005)	0.797 (0.533 - 1.114)	1.121 (0.750 - 1.563)	1.400 (0.939 - 1.940)

	Prevalence 1990	Prevalence 2005	Prevalence 2015	Incidence 1990	Incidence 2005	Incidence 2015	YLD rate 1990	YLD rate 2005	YLD rate 2015
Age-standardized (rate per capita)	0.053 (0.050 - 0.056)	0.053 (0.050 - 0.056)	0.053 (0.050 - 0.056)	0.004 (0.004 - 0.004)	0.004 (0.004 - 0.004)	0.004 (0.004 - 0.004)	1.453 (0.973 - 2.032)	1.451 (0.969 - 2.015)	1.448 (0.972 - 2.005)
Total number of cases (thousands)	7 (7 - 8)	13 (13 - 14)	20 (19 - 21)	1 (1 - 1)	1 (1 - 1)	2 (2 - 2)	0 (0 - 0)	0 (0 - 1)	1 (0 - 1)
**Barbados**									
15 - 49 years (rate per capita)	0.012 (0.011 - 0.013)	0.016 (0.015 - 0.017)	0.016 (0.015 - 0.017)	0.001 (0.001 - 0.001)	0.002 (0.001 - 0.002)	0.002 (0.001 - 0.002)	0.346 (0.228 - 0.486)	0.450 (0.299 - 0.631)	0.449 (0.300 - 0.631)
50 - 69 years (rate per capita)	0.152 (0.141 - 0.164)	0.139 (0.129 - 0.150)	0.144 (0.134 - 0.155)	0.015 (0.014 - 0.017)	0.014 (0.013 - 0.015)	0.015 (0.013 - 0.016)	4.218 (2.805 - 5.888)	3.851 (2.560 - 5.357)	3.990 (2.638 - 5.541)
70+ years (rate per capita)	0.314 (0.294 - 0.338)	0.310 (0.289 - 0.332)	0.309 (0.289 - 0.332)	0.013 (0.011 - 0.015)	0.012 (0.011 - 0.014)	0.013 (0.011 - 0.014)	8.446 (5.728 - 11.648)	8.290 (5.645 - 11.395)	8.254 (5.580 - 11.343)
All ages (rate per capita)	0.049 (0.047 - 0.052)	0.060 (0.057 - 0.064)	0.072 (0.068 - 0.076)	0.004 (0.003 - 0.004)	0.005 (0.004 - 0.005)	0.006 (0.005 - 0.006)	1.351 (0.905 - 1.865)	1.648 (1.104 - 2.271)	1.972 (1.315 - 2.716)
Age-standardized (rate per capita)	0.053 (0.051 - 0.056)	0.053 (0.050 - 0.056)	0.053 (0.050 - 0.056)	0.004 (0.004 - 0.004)	0.004 (0.004 - 0.004)	0.004 (0.004 - 0.004)	1.467 (0.980 - 2.024)	1.455 (0.975 - 2.007)	1.452 (0.968 - 2.001)
Total number of cases (thousands)	13 (12 - 14)	16 (16 - 17)	20 (19 - 22)	1 (1 - 1)	1 (1 - 1)	2 (1 - 2)	0 (0 - 0)	0 (0 - 1)	1 (0 - 1)
**Belize**									
15 - 49 years (rate per capita)	0.010 (0.009 - 0.011)	0.011 (0.011 - 0.012)	0.012 (0.011 - 0.013)	0.001 (0.001 - 0.001)	0.001 (0.001 - 0.001)	0.001 (0.001 - 0.001)	0.284 (0.189 - 0.400)	0.327 (0.215 - 0.461)	0.352 (0.230 - 0.495)
50 - 69 years (rate per capita)	0.140 (0.129 - 0.151)	0.135 (0.125 - 0.146)	0.129 (0.119 - 0.139)	0.014 (0.013 - 0.016)	0.014 (0.013 - 0.015)	0.013 (0.012 - 0.015)	3.910 (2.568 - 5.471)	3.768 (2.484 - 5.273)	3.591 (2.376 - 5.026)
70+ years (rate per capita)	0.309 (0.286 - 0.331)	0.314 (0.291 - 0.336)	0.310 (0.288 - 0.332)	0.013 (0.011 - 0.015)	0.014 (0.012 - 0.017)	0.014 (0.012 - 0.016)	8.340 (5.570 - 11.589)	8.441 (5.676 - 11.678)	8.330 (5.628 - 11.579)
All ages (rate per capita)	0.024 (0.023 - 0.025)	0.022 (0.021 - 0.023)	0.028 (0.026 - 0.029)	0.002 (0.002 - 0.002)	0.002 (0.002 - 0.002)	0.002 (0.002 - 0.003)	0.664 (0.442 - 0.928)	0.613 (0.407 - 0.852)	0.772 (0.511 - 1.072)
Age-standardized (rate per capita)	0.052 (0.049 - 0.055)	0.052 (0.049 - 0.055)	0.052 (0.049 - 0.055)	0.004 (0.004 - 0.004)	0.004 (0.004 - 0.004)	0.004 (0.004 - 0.004)	1.445 (0.963 - 2.008)	1.442 (0.961 - 2.001)	1.437 (0.959 - 1.990)
Total number of cases (thousands)	5 (4 - 5)	6 (6 - 7)	10 (9 - 11)	0 (0 - 0)	1 (0 - 1)	1 (1 - 1)	0 (0 - 0)	0 (0 - 0)	0 (0 - 0)
**Bermuda**									
15 - 49 years (rate per capita)	0.012 (0.011 - 0.013)	0.013 (0.012 - 0.014)	0.014 (0.013 - 0.015)	0.001 (0.001 - 0.001)	0.001 (0.001 - 0.001)	0.001 (0.001 - 0.002)	0.339 (0.224 - 0.478)	0.384 (0.252 - 0.535)	0.403 (0.265 - 0.563)
50 - 69 years (rate per capita)	0.141 (0.131 - 0.152)	0.140 (0.130 - 0.151)	0.138 (0.128 - 0.148)	0.014 (0.013 - 0.016)	0.014 (0.013 - 0.016)	0.014 (0.013 - 0.015)	3.941 (2.603 - 5.494)	3.916 (2.587 - 5.472)	3.846 (2.541 - 5.383)
70+ years (rate per capita)	0.311 (0.290 - 0.334)	0.310 (0.289 - 0.332)	0.309 (0.288 - 0.331)	0.013 (0.011 - 0.016)	0.013 (0.011 - 0.015)	0.013 (0.011 - 0.015)	8.407 (5.654 - 11.635)	8.339 (5.626 - 11.507)	8.316 (5.579 - 11.475)
All ages (rate per capita)	0.038 (0.036 - 0.040)	0.044 (0.041 - 0.046)	0.051 (0.048 - 0.054)	0.003 (0.003 - 0.003)	0.003 (0.003 - 0.004)	0.004 (0.004 - 0.004)	1.046 (0.700 - 1.447)	1.214 (0.813 - 1.677)	1.419 (0.947 - 1.963)
Age-standardized (rate per capita)	0.053 (0.050 - 0.056)	0.053 (0.050 - 0.056)	0.053 (0.050 - 0.056)	0.004 (0.004 - 0.004)	0.004 (0.004 - 0.004)	0.004 (0.004 - 0.004)	1.455 (0.973 - 2.011)	1.455 (0.975 - 2.008)	1.455 (0.972 - 2.012)
Total number of cases (thousands)	2 (2 - 2)	3 (3 - 3)	3 (3 - 4)	0 (0 - 0)	0 (0 - 0)	0 (0 - 0)	0 (0 - 0)	0 (0 - 0)	0 (0 - 0)
**Cuba**									
15 - 49 years (rate per capita)	0.012 (0.011 - 0.013)	0.015 (0.014 - 0.016)	0.017 (0.016 - 0.019)	0.001 (0.001 - 0.001)	0.001 (0.001 - 0.002)	0.002 (0.002 - 0.002)	0.349 (0.230 - 0.492)	0.437 (0.291 - 0.619)	0.489 (0.326 - 0.689)
50 - 69 years (rate per capita)	0.142 (0.132 - 0.153)	0.143 (0.132 - 0.154)	0.137 (0.127 - 0.148)	0.014 (0.013 - 0.016)	0.014 (0.013 - 0.016)	0.014 (0.013 - 0.015)	3.983 (2.620 - 5.595)	3.991 (2.632 - 5.592)	3.843 (2.544 - 5.367)
70+ years (rate per capita)	0.310 (0.289 - 0.334)	0.309 (0.287 - 0.332)	0.308 (0.287 - 0.331)	0.013 (0.011 - 0.015)	0.013 (0.011 - 0.015)	0.013 (0.011 - 0.015)	8.385 (5.632 - 11.606)	8.327 (5.575 - 11.470)	8.283 (5.581 - 11.403)
All ages (rate per capita)	0.046 (0.044 - 0.049)	0.057 (0.054 - 0.061)	0.072 (0.068 - 0.076)	0.004 (0.003 - 0.004)	0.004 (0.004 - 0.005)	0.006 (0.005 - 0.006)	1.282 (0.852 - 1.775)	1.592 (1.060 - 2.211)	1.990 (1.325 - 2.757)
Age-standardized (rate per capita)	0.053 (0.050 - 0.056)	0.053 (0.050 - 0.056)	0.053 (0.050 - 0.056)	0.004 (0.004 - 0.004)	0.004 (0.004 - 0.004)	0.004 (0.004 - 0.004)	1.461 (0.970 - 2.026)	1.459 (0.972 - 2.026)	1.458 (0.970 - 2.020)
Total number of cases (thousands)	489 (463 - 517)	648 (614 - 686)	821 (776 - 871)	38 (35 - 40)	50 (47 - 53)	63 (59 - 67)	14 (9 - 19)	18 (12 - 25)	23 (15 - 31)
**Dominica**									
15 - 49 years (rate per capita)	0.012 (0.011 - 0.013)	0.013 (0.012 - 0.015)	0.014 (0.013 - 0.015)	0.001 (0.001 - 0.001)	0.001 (0.001 - 0.001)	0.001 (0.001 - 0.002)	0.338 (0.223 - 0.476)	0.383 (0.248 - 0.538)	0.401 (0.265 - 0.560)
50 - 69 years (rate per capita)	0.141 (0.131 - 0.152)	0.140 (0.130 - 0.151)	0.138 (0.128 - 0.149)	0.014 (0.013 - 0.016)	0.014 (0.013 - 0.016)	0.014 (0.013 - 0.015)	3.929 (2.594 - 5.516)	3.896 (2.576 - 5.476)	3.819 (2.530 - 5.355)
70+ years (rate per capita)	0.311 (0.289 - 0.334)	0.309 (0.288 - 0.332)	0.309 (0.287 - 0.331)	0.013 (0.011 - 0.016)	0.013 (0.011 - 0.015)	0.013 (0.011 - 0.015)	8.358 (5.674 - 11.539)	8.287 (5.614 - 11.434)	8.238 (5.554 - 11.392)
All ages (rate per capita)	0.036 (0.034 - 0.038)	0.043 (0.041 - 0.046)	0.050 (0.048 - 0.053)	0.003 (0.003 - 0.003)	0.003 (0.003 - 0.004)	0.004 (0.004 - 0.004)	0.996 (0.667 - 1.374)	1.187 (0.795 - 1.646)	1.377 (0.922 - 1.894)
Age-standardized (rate per capita)	0.053 (0.050 - 0.056)	0.053 (0.050 - 0.056)	0.053 (0.050 - 0.056)	0.004 (0.004 - 0.004)	0.004 (0.004 - 0.004)	0.004 (0.004 - 0.004)	1.449 (0.970 - 1.996)	1.447 (0.965 - 2.003)	1.444 (0.968 - 1.980)
Total number of cases (thousands)	2 (2 - 3)	3 (3 - 3)	4 (3 - 4)	0 (0 - 0)	0 (0 - 0)	0 (0 - 0)	0 (0 - 0)	0 (0 - 0)	0 (0 - 0)
**Dominican Republic**									
15 - 49 years (rate per capita)	0.011 (0.010 - 0.011)	0.012 (0.012 - 0.013)	0.013 (0.012 - 0.014)	0.001 (0.001 - 0.001)	0.001 (0.001 - 0.001)	0.001 (0.001 - 0.001)	0.312 (0.206 - 0.436)	0.353 (0.235 - 0.495)	0.369 (0.246 - 0.517)
50 - 69 years (rate per capita)	0.137 (0.129 - 0.145)	0.138 (0.131 - 0.147)	0.137 (0.129 - 0.145)	0.014 (0.013 - 0.015)	0.014 (0.013 - 0.015)	0.014 (0.013 - 0.015)	3.834 (2.534 - 5.315)	3.872 (2.592 - 5.367)	3.830 (2.537 - 5.328)
70+ years (rate per capita)	0.312 (0.295 - 0.329)	0.310 (0.293 - 0.327)	0.308 (0.291 - 0.324)	0.014 (0.012 - 0.016)	0.013 (0.011 - 0.015)	0.013 (0.011 - 0.014)	8.464 (5.694 - 11.689)	8.364 (5.649 - 11.466)	8.280 (5.599 - 11.417)
All ages (rate per capita)	0.026 (0.025 - 0.027)	0.033 (0.032 - 0.035)	0.039 (0.038 - 0.041)	0.002 (0.002 - 0.002)	0.003 (0.003 - 0.003)	0.003 (0.003 - 0.003)	0.721 (0.483 - 0.995)	0.923 (0.620 - 1.270)	1.091 (0.734 - 1.498)
Age-standardized (rate per capita)	0.052 (0.050 - 0.054)	0.053 (0.051 - 0.055)	0.053 (0.050 - 0.055)	0.004 (0.004 - 0.004)	0.004 (0.004 - 0.004)	0.004 (0.004 - 0.004)	1.450 (0.970 - 2.001)	1.456 (0.979 - 2.006)	1.455 (0.979 - 1.998)
Total number of cases (thousands)	186 (178 - 193)	307 (296 - 320)	414 (398 - 431)	16 (15 - 17)	25 (24 - 26)	33 (32 - 35)	5 (3 - 7)	9 (6 - 12)	11 (8 - 16)
**Grenada**									
15 - 49 years (rate per capita)	0.011 (0.010 - 0.012)	0.011 (0.011 - 0.012)	0.012 (0.011 - 0.013)	0.001 (0.001 - 0.001)	0.001 (0.001 - 0.001)	0.001 (0.001 - 0.001)	0.307 (0.201 - 0.432)	0.328 (0.219 - 0.460)	0.344 (0.227 - 0.483)
50 - 69 years (rate per capita)	0.151 (0.140 - 0.163)	0.143 (0.133 - 0.155)	0.132 (0.123 - 0.143)	0.015 (0.014 - 0.017)	0.014 (0.013 - 0.016)	0.014 (0.012 - 0.015)	4.185 (2.772 - 5.823)	3.970 (2.634 - 5.522)	3.658 (2.424 - 5.098)
70+ years (rate per capita)	0.315 (0.293 - 0.336)	0.313 (0.291 - 0.334)	0.310 (0.288 - 0.331)	0.014 (0.012 - 0.017)	0.013 (0.011 - 0.015)	0.012 (0.011 - 0.015)	8.485 (5.736 - 11.661)	8.387 (5.684 - 11.540)	8.275 (5.618 - 11.437)
All ages (rate per capita)	0.039 (0.037 - 0.042)	0.038 (0.036 - 0.040)	0.041 (0.039 - 0.043)	0.003 (0.003 - 0.003)	0.003 (0.003 - 0.003)	0.003 (0.003 - 0.003)	1.079 (0.723 - 1.495)	1.049 (0.700 - 1.451)	1.129 (0.755 - 1.560)
Age-standardized (rate per capita)	0.053 (0.050 - 0.056)	0.053 (0.050 - 0.056)	0.052 (0.050 - 0.056)	0.004 (0.004 - 0.004)	0.004 (0.004 - 0.004)	0.004 (0.004 - 0.004)	1.452 (0.970 - 2.016)	1.446 (0.966 - 2.006)	1.440 (0.963 - 1.995)
Total number of cases (thousands)	4 (4 - 4)	4 (4 - 4)	4 (4 - 5)	0 (0 - 0)	0 (0 - 0)	0 (0 - 0)	0 (0 - 0)	0 (0 - 0)	0 (0 - 0)
**Guyana**									
15 - 49 years (rate per capita)	0.011 (0.010 - 0.012)	0.014 (0.013 - 0.015)	0.013 (0.012 - 0.014)	0.001 (0.001 - 0.001)	0.001 (0.001 - 0.002)	0.001 (0.001 - 0.002)	0.319 (0.212 - 0.449)	0.406 (0.271 - 0.569)	0.376 (0.249 - 0.533)
50 - 69 years (rate per capita)	0.140 (0.131 - 0.151)	0.131 (0.122 - 0.141)	0.128 (0.120 - 0.138)	0.014 (0.013 - 0.016)	0.014 (0.012 - 0.015)	0.013 (0.012 - 0.015)	3.898 (2.609 - 5.437)	3.634 (2.429 - 5.036)	3.561 (2.362 - 4.985)
70+ years (rate per capita)	0.301 (0.279 - 0.323)	0.311 (0.289 - 0.333)	0.316 (0.293 - 0.339)	0.013 (0.011 - 0.015)	0.013 (0.011 - 0.015)	0.015 (0.012 - 0.017)	8.060 (5.464 - 11.198)	8.320 (5.624 - 11.574)	8.472 (5.774 - 11.740)
All ages (rate per capita)	0.025 (0.023 - 0.026)	0.029 (0.028 - 0.031)	0.037 (0.035 - 0.039)	0.002 (0.002 - 0.002)	0.002 (0.002 - 0.003)	0.003 (0.003 - 0.004)	0.680 (0.455 - 0.940)	0.805 (0.540 - 1.105)	1.027 (0.690 - 1.414)
Age-standardized (rate per capita)	0.052 (0.049 - 0.055)	0.052 (0.050 - 0.055)	0.052 (0.050 - 0.055)	0.004 (0.004 - 0.004)	0.004 (0.004 - 0.004)	0.004 (0.004 - 0.004)	1.434 (0.959 - 1.972)	1.433 (0.962 - 1.977)	1.432 (0.963 - 1.976)
Total number of cases (thousands)	18 (17 - 19)	22 (20 - 23)	29 (27 - 30)	2 (1 - 2)	2 (2 - 2)	3 (2 - 3)	1 (0 - 1)	1 (0 - 1)	1 (1 - 1)
**Haiti**									
15 - 49 years (rate per capita)	0.011 (0.011 - 0.012)	0.011 (0.010 - 0.012)	0.012 (0.011 - 0.013)	0.001 (0.001 - 0.001)	0.001 (0.001 - 0.001)	0.001 (0.001 - 0.001)	0.325 (0.217 - 0.454)	0.318 (0.212 - 0.443)	0.333 (0.219 - 0.468)
50 - 69 years (rate per capita)	0.136 (0.126 - 0.147)	0.138 (0.128 - 0.149)	0.134 (0.125 - 0.145)	0.014 (0.013 - 0.015)	0.014 (0.013 - 0.015)	0.014 (0.013 - 0.015)	3.763 (2.501 - 5.270)	3.825 (2.547 - 5.367)	3.699 (2.457 - 5.169)
70+ years (rate per capita)	0.314 (0.291 - 0.336)	0.313 (0.291 - 0.335)	0.313 (0.291 - 0.335)	0.015 (0.012 - 0.017)	0.014 (0.012 - 0.017)	0.014 (0.012 - 0.017)	8.382 (5.581 - 11.538)	8.382 (5.613 - 11.603)	8.275 (5.560 - 11.487)
All ages (rate per capita)	0.026 (0.024 - 0.027)	0.027 (0.025 - 0.028)	0.030 (0.028 - 0.032)	0.002 (0.002 - 0.002)	0.002 (0.002 - 0.002)	0.003 (0.002 - 0.003)	0.705 (0.470 - 0.977)	0.737 (0.491 - 1.021)	0.818 (0.546 - 1.129)
Age-standardized (rate per capita)	0.052 (0.049 - 0.055)	0.052 (0.049 - 0.055)	0.052 (0.049 - 0.055)	0.004 (0.004 - 0.004)	0.004 (0.004 - 0.004)	0.004 (0.004 - 0.004)	1.428 (0.955 - 1.975)	1.434 (0.958 - 1.988)	1.421 (0.949 - 1.968)
Total number of cases (thousands)	182 (172 - 193)	248 (234 - 262)	321 (303 - 339)	16 (15 - 17)	21 (20 - 23)	27 (25 - 29)	5 (3 - 7)	7 (5 - 9)	9 (6 - 12)
**Jamaica**									
15 - 49 years (rate per capita)	0.011 (0.010 - 0.012)	0.013 (0.012 - 0.014)	0.013 (0.012 - 0.014)	0.001 (0.001 - 0.001)	0.001 (0.001 - 0.001)	0.001 (0.001 - 0.001)	0.312 (0.206 - 0.443)	0.376 (0.247 - 0.529)	0.374 (0.251 - 0.526)
50 - 69 years (rate per capita)	0.148 (0.137 - 0.159)	0.139 (0.129 - 0.149)	0.138 (0.128 - 0.149)	0.015 (0.013 - 0.016)	0.014 (0.013 - 0.016)	0.014 (0.013 - 0.016)	4.121 (2.731 - 5.752)	3.853 (2.548 - 5.370)	3.838 (2.546 - 5.351)
70+ years (rate per capita)	0.309 (0.288 - 0.331)	0.308 (0.286 - 0.330)	0.305 (0.284 - 0.328)	0.013 (0.011 - 0.015)	0.013 (0.011 - 0.014)	0.012 (0.011 - 0.014)	8.354 (5.631 - 11.582)	8.273 (5.572 - 11.435)	8.176 (5.495 - 11.282)
All ages (rate per capita)	0.037 (0.035 - 0.039)	0.042 (0.039 - 0.044)	0.048 (0.046 - 0.051)	0.003 (0.003 - 0.003)	0.003 (0.003 - 0.003)	0.004 (0.004 - 0.004)	1.017 (0.679 - 1.408)	1.146 (0.766 - 1.580)	1.329 (0.891 - 1.843)
Age-standardized (rate per capita)	0.053 (0.050 - 0.056)	0.053 (0.050 - 0.056)	0.053 (0.050 - 0.056)	0.004 (0.004 - 0.004)	0.004 (0.004 - 0.004)	0.004 (0.004 - 0.004)	1.459 (0.977 - 2.024)	1.450 (0.968 - 2.004)	1.444 (0.968 - 2.002)
Total number of cases (thousands)	88 (84 - 93)	112 (106 - 118)	137 (130 - 145)	7 (6 - 7)	8 (8 - 9)	11 (10 - 11)	2 (2 - 3)	3 (2 - 4)	4 (3 - 5)
**Puerto Rico**									
15 - 49 years (rate per capita)	0.014 (0.013 - 0.015)	0.014 (0.013 - 0.016)	0.015 (0.014 - 0.016)	0.001 (0.001 - 0.002)	0.001 (0.001 - 0.002)	0.001 (0.001 - 0.002)	0.388 (0.257 - 0.544)	0.412 (0.274 - 0.576)	0.422 (0.281 - 0.590)
50 - 69 years (rate per capita)	0.147 (0.137 - 0.159)	0.145 (0.135 - 0.157)	0.149 (0.139 - 0.161)	0.015 (0.013 - 0.016)	0.015 (0.013 - 0.016)	0.015 (0.014 - 0.016)	4.102 (2.721 - 5.724)	4.039 (2.677 - 5.638)	4.144 (2.733 - 5.749)

	Prevalence 1990	Prevalence 2005	Prevalence 2015	Incidence 1990	Incidence 2005	Incidence 2015	YLD rate 1990	YLD rate 2005	YLD rate 2015
70+ years (rate per capita)	0.311 (0.290 - 0.332)	0.309 (0.288 - 0.330)	0.309 (0.288 - 0.330)	0.013 (0.011 - 0.015)	0.012 (0.011 - 0.014)	0.012 (0.011 - 0.014)	8.372 (5.618 - 11.533)	8.282 (5.574 - 11.463)	8.255 (5.581 - 11.381)
All ages (rate per capita)	0.050 (0.047 - 0.052)	0.062 (0.058 - 0.065)	0.070 (0.066 - 0.074)	0.004 (0.004 - 0.004)	0.005 (0.004 - 0.005)	0.005 (0.005 - 0.006)	1.369 (0.909 - 1.879)	1.694 (1.130 - 2.330)	1.921 (1.274 - 2.655)
Age-standardized (rate per capita)	0.053 (0.050 - 0.056)	0.053 (0.051 - 0.056)	0.053 (0.051 - 0.056)	0.004 (0.004 - 0.004)	0.004 (0.004 - 0.004)	0.004 (0.004 - 0.004)	1.466 (0.973 - 2.013)	1.466 (0.978 - 2.019)	1.465 (0.970 - 2.027)
Total number of cases (thousands)	175 (166 - 185)	232 (220 - 245)	258 (244 - 273)	13 (13 - 14)	17 (16 - 19)	19 (18 - 20)	5 (3 - 7)	6 (4 - 9)	7 (5 - 10)
**Saint Lucia**									
15 - 49 years (rate per capita)	0.011 (0.010 - 0.012)	0.013 (0.012 - 0.014)	0.014 (0.013 - 0.015)	0.001 (0.001 - 0.001)	0.001 (0.001 - 0.001)	0.001 (0.001 - 0.002)	0.310 (0.206 - 0.434)	0.365 (0.240 - 0.512)	0.407 (0.272 - 0.573)
50 - 69 years (rate per capita)	0.147 (0.136 - 0.159)	0.139 (0.129 - 0.151)	0.135 (0.125 - 0.146)	0.015 (0.013 - 0.016)	0.014 (0.013 - 0.016)	0.014 (0.013 - 0.015)	4.061 (2.701 - 5.677)	3.874 (2.567 - 5.413)	3.754 (2.489 - 5.251)
70+ years (rate per capita)	0.315 (0.292 - 0.340)	0.302 (0.280 - 0.325)	0.307 (0.284 - 0.330)	0.014 (0.012 - 0.017)	0.012 (0.010 - 0.014)	0.012 (0.011 - 0.015)	8.456 (5.676 - 11.721)	8.082 (5.466 - 11.232)	8.209 (5.554 - 11.365)
All ages (rate per capita)	0.037 (0.035 - 0.039)	0.038 (0.036 - 0.041)	0.050 (0.047 - 0.053)	0.003 (0.003 - 0.003)	0.003 (0.003 - 0.003)	0.004 (0.004 - 0.004)	1.013 (0.677 - 1.396)	1.058 (0.709 - 1.460)	1.372 (0.919 - 1.904)
Age-standardized (rate per capita)	0.053 (0.049 - 0.056)	0.053 (0.050 - 0.056)	0.053 (0.049 - 0.056)	0.004 (0.004 - 0.004)	0.004 (0.004 - 0.004)	0.004 (0.004 - 0.004)	1.440 (0.963 - 1.991)	1.446 (0.966 - 2.004)	1.446 (0.966 - 2.007)
Total number of cases (thousands)	5 (5 - 5)	6 (6 - 7)	9 (9 - 10)	0 (0 - 0)	0 (0 - 1)	1 (1 - 1)	0 (0 - 0)	0 (0 - 0)	0 (0 - 0)
**Saint Vincent and the Grenadines**									
15 - 49 years (rate per capita)	0.010 (0.009 - 0.011)	0.013 (0.012 - 0.014)	0.014 (0.013 - 0.015)	0.001 (0.001 - 0.001)	0.001 (0.001 - 0.001)	0.001 (0.001 - 0.001)	0.290 (0.191 - 0.405)	0.375 (0.249 - 0.531)	0.387 (0.258 - 0.544)
50 - 69 years (rate per capita)	0.145 (0.134 - 0.156)	0.139 (0.129 - 0.150)	0.130 (0.121 - 0.140)	0.015 (0.013 - 0.016)	0.014 (0.013 - 0.015)	0.014 (0.012 - 0.015)	4.023 (2.677 - 5.668)	3.853 (2.568 - 5.390)	3.610 (2.399 - 5.050)
70+ years (rate per capita)	0.312 (0.289 - 0.335)	0.310 (0.288 - 0.333)	0.310 (0.287 - 0.333)	0.014 (0.011 - 0.016)	0.013 (0.011 - 0.015)	0.013 (0.011 - 0.015)	8.401 (5.681 - 11.640)	8.308 (5.626 - 11.497)	8.282 (5.578 - 11.394)
All ages (rate per capita)	0.032 (0.031 - 0.034)	0.038 (0.036 - 0.040)	0.045 (0.042 - 0.048)	0.003 (0.002 - 0.003)	0.003 (0.003 - 0.003)	0.004 (0.003 - 0.004)	0.891 (0.599 - 1.229)	1.055 (0.711 - 1.455)	1.238 (0.831 - 1.710)
Age-standardized (rate per capita)	0.053 (0.050 - 0.056)	0.052 (0.049 - 0.055)	0.052 (0.049 - 0.055)	0.004 (0.004 - 0.004)	0.004 (0.004 - 0.004)	0.004 (0.004 - 0.004)	1.449 (0.974 - 1.994)	1.436 (0.966 - 1.982)	1.432 (0.964 - 1.980)
Total number of cases (thousands)	3 (3 - 4)	4 (4 - 4)	5 (5 - 5)	0 (0 - 0)	0 (0 - 0)	0 (0 - 0)	0 (0 - 0)	0 (0 - 0)	0 (0 - 0)
**Suriname**									
15 - 49 years (rate per capita)	0.011 (0.011 - 0.012)	0.013 (0.012 - 0.014)	0.014 (0.013 - 0.015)	0.001 (0.001 - 0.001)	0.001 (0.001 - 0.001)	0.001 (0.001 - 0.002)	0.325 (0.215 - 0.456)	0.382 (0.252 - 0.539)	0.400 (0.266 - 0.563)
50 - 69 years (rate per capita)	0.137 (0.128 - 0.149)	0.140 (0.130 - 0.151)	0.131 (0.122 - 0.142)	0.014 (0.013 - 0.016)	0.014 (0.013 - 0.016)	0.014 (0.012 - 0.015)	3.837 (2.544 - 5.360)	3.894 (2.584 - 5.430)	3.669 (2.443 - 5.126)
70+ years (rate per capita)	0.310 (0.290 - 0.333)	0.315 (0.294 - 0.338)	0.313 (0.292 - 0.336)	0.013 (0.011 - 0.016)	0.014 (0.012 - 0.017)	0.014 (0.012 - 0.016)	8.373 (5.703 - 11.600)	8.502 (5.715 - 11.800)	8.438 (5.717 - 11.732)
All ages (rate per capita)	0.030 (0.029 - 0.032)	0.037 (0.035 - 0.039)	0.043 (0.041 - 0.046)	0.003 (0.002 - 0.003)	0.003 (0.003 - 0.003)	0.004 (0.003 - 0.004)	0.843 (0.565 - 1.170)	1.024 (0.683 - 1.424)	1.193 (0.796 - 1.657)
Age-standardized (rate per capita)	0.052 (0.050 - 0.056)	0.053 (0.050 - 0.056)	0.053 (0.050 - 0.056)	0.004 (0.004 - 0.004)	0.004 (0.004 - 0.004)	0.004 (0.004 - 0.004)	1.450 (0.976 - 2.015)	1.453 (0.969 - 2.020)	1.452 (0.973 - 2.025)
Total number of cases (thousands)	12 (12 - 13)	18 (17 - 19)	23 (22 - 25)	1 (1 - 1)	1 (1 - 2)	2 (2 - 2)	0 (0 - 0)	1 (0 - 1)	1 (0 - 1)
**Trinidad and Tobago**									
15 - 49 years (rate per capita)	0.012 (0.011 - 0.013)	0.014 (0.013 - 0.015)	0.015 (0.014 - 0.016)	0.001 (0.001 - 0.001)	0.001 (0.001 - 0.002)	0.001 (0.001 - 0.002)	0.354 (0.234 - 0.496)	0.391 (0.261 - 0.548)	0.420 (0.276 - 0.587)
50 - 69 years (rate per capita)	0.140 (0.130 - 0.151)	0.135 (0.125 - 0.146)	0.137 (0.127 - 0.148)	0.014 (0.013 - 0.016)	0.014 (0.013 - 0.015)	0.014 (0.013 - 0.015)	3.896 (2.568 - 5.432)	3.741 (2.475 - 5.237)	3.788 (2.502 - 5.303)
70+ years (rate per capita)	0.314 (0.291 - 0.337)	0.312 (0.290 - 0.336)	0.313 (0.290 - 0.336)	0.014 (0.012 - 0.016)	0.013 (0.011 - 0.016)	0.014 (0.012 - 0.016)	8.437 (5.667 - 11.567)	8.373 (5.619 - 11.575)	8.372 (5.658 - 11.513)
All ages (rate per capita)	0.033 (0.032 - 0.035)	0.044 (0.042 - 0.047)	0.055 (0.052 - 0.059)	0.003 (0.003 - 0.003)	0.004 (0.003 - 0.004)	0.005 (0.004 - 0.005)	0.924 (0.617 - 1.277)	1.222 (0.816 - 1.694)	1.517 (1.011 - 2.109)
Age-standardized (rate per capita)	0.052 (0.050 - 0.056)	0.052 (0.050 - 0.056)	0.052 (0.050 - 0.056)	0.004 (0.004 - 0.004)	0.004 (0.004 - 0.004)	0.004 (0.004 - 0.004)	1.444 (0.965 - 1.999)	1.441 (0.964 - 1.999)	1.439 (0.960 - 1.994)
Total number of cases (thousands)	41 (39 - 43)	58 (55 - 61)	75 (71 - 80)	3 (3 - 4)	5 (4 - 5)	6 (6 - 7)	1 (1 - 2)	2 (1 - 2)	2 (1 - 3)
**Virgin Islands, U.S.**									
15 - 49 years (rate per capita)	0.015 (0.013 - 0.016)	0.016 (0.015 - 0.017)	0.016 (0.015 - 0.017)	0.001 (0.001 - 0.002)	0.002 (0.001 - 0.002)	0.002 (0.001 - 0.002)	0.417 (0.274 - 0.585)	0.452 (0.299 - 0.640)	0.451 (0.299 - 0.638)
50 - 69 years (rate per capita)	0.135 (0.125 - 0.144)	0.142 (0.131 - 0.152)	0.152 (0.141 - 0.163)	0.014 (0.013 - 0.015)	0.014 (0.013 - 0.016)	0.015 (0.014 - 0.017)	3.755 (2.481 - 5.263)	3.942 (2.612 - 5.494)	4.222 (2.818 - 5.907)
70+ years (rate per capita)	0.318 (0.297 - 0.341)	0.310 (0.289 - 0.333)	0.312 (0.291 - 0.334)	0.015 (0.012 - 0.017)	0.013 (0.011 - 0.016)	0.014 (0.012 - 0.016)	8.588 (5.767 - 11.938)	8.323 (5.629 - 11.576)	8.352 (5.642 - 11.542)
All ages (rate per capita)	0.038 (0.036 - 0.041)	0.061 (0.058 - 0.065)	0.082 (0.078 - 0.087)	0.003 (0.003 - 0.004)	0.005 (0.005 - 0.005)	0.006 (0.006 - 0.007)	1.066 (0.706 - 1.479)	1.698 (1.130 - 2.352)	2.257 (1.504 - 3.128)
Age-standardized (rate per capita)	0.053 (0.050 - 0.056)	0.053 (0.050 - 0.056)	0.053 (0.050 - 0.056)	0.004 (0.004 - 0.004)	0.004 (0.004 - 0.004)	0.004 (0.004 - 0.004)	1.456 (0.969 - 2.023)	1.452 (0.970 - 2.008)	1.452 (0.969 - 2.008)
Total number of cases (thousands)	4 (4 - 4)	7 (6 - 7)	9 (8 - 9)	0 (0 - 0)	1 (1 - 1)	1 (1 - 1)	0 (0 - 0)	0 (0 - 0)	0 (0 - 0)
**Tropical Latin America**									
15 - 49 years (rate per capita)	0.016 (0.015 - 0.016)	0.017 (0.016 - 0.018)	0.018 (0.017 - 0.019)	0.002 (0.001 - 0.002)	0.002 (0.002 - 0.002)	0.002 (0.002 - 0.002)	0.447 (0.298 - 0.625)	0.489 (0.326 - 0.684)	0.513 (0.342 - 0.716)
50 - 69 years (rate per capita)	0.198 (0.189 - 0.207)	0.201 (0.193 - 0.211)	0.199 (0.190 - 0.208)	0.021 (0.020 - 0.022)	0.022 (0.020 - 0.023)	0.021 (0.020 - 0.023)	5.509 (3.692 - 7.613)	5.600 (3.769 - 7.773)	5.534 (3.722 - 7.673)
70+ years (rate per capita)	0.447 (0.426 - 0.467)	0.455 (0.434 - 0.476)	0.442 (0.422 - 0.462)	0.020 (0.017 - 0.022)	0.019 (0.017 - 0.021)	0.018 (0.016 - 0.020)	12.029 (8.115 - 16.610)	12.225 (8.273 - 16.726)	11.858 (8.024 - 16.239)
All ages (rate per capita)	0.039 (0.038 - 0.040)	0.053 (0.051 - 0.054)	0.066 (0.064 - 0.068)	0.003 (0.003 - 0.004)	0.005 (0.004 - 0.005)	0.006 (0.005 - 0.006)	1.083 (0.730 - 1.492)	1.458 (0.983 - 2.015)	1.828 (1.233 - 2.535)
Age-standardized (rate per capita)	0.074 (0.072 - 0.077)	0.076 (0.073 - 0.079)	0.074 (0.072 - 0.077)	0.006 (0.006 - 0.006)	0.006 (0.006 - 0.006)	0.006 (0.006 - 0.006)	2.051 (1.385 - 2.829)	2.091 (1.417 - 2.885)	2.050 (1.386 - 2.844)
Total number of cases (thousands)	6,029 (5,826 - 6,240)	10,222 (9,891 - 10,581)	14,186 (13,713 - 14,687)	533 (512 - 555)	887 (851 - 924)	1,201 (1,151 - 1,253)	168 (113 - 231)	283 (191 - 391)	392 (265 - 544)
**Brazil**									
15 - 49 years (rate per capita)	0.016 (0.015 - 0.017)	0.017 (0.016 - 0.018)	0.018 (0.017 - 0.019)	0.002 (0.001 - 0.002)	0.002 (0.002 - 0.002)	0.002 (0.002 - 0.002)	0.451 (0.300 - 0.631)	0.494 (0.330 - 0.691)	0.519 (0.346 - 0.725)
50 - 69 years (rate per capita)	0.199 (0.190 - 0.208)	0.203 (0.194 - 0.213)	0.200 (0.192 - 0.209)	0.021 (0.020 - 0.023)	0.022 (0.021 - 0.023)	0.021 (0.020 - 0.023)	5.549 (3.720 - 7.670)	5.648 (3.801 - 7.839)	5.574 (3.750 - 7.728)
70+ years (rate per capita)	0.451 (0.430 - 0.471)	0.459 (0.438 - 0.480)	0.445 (0.425 - 0.466)	0.020 (0.017 - 0.022)	0.019 (0.017 - 0.021)	0.018 (0.016 - 0.020)	12.138 (8.192 - 16.758)	12.329 (8.346 - 16.869)	11.948 (8.085 - 16.360)
All ages (rate per capita)	0.039 (0.038 - 0.041)	0.053 (0.052 - 0.055)	0.067 (0.065 - 0.070)	0.003 (0.003 - 0.004)	0.005 (0.004 - 0.005)	0.006 (0.005 - 0.006)	1.094 (0.737 - 1.508)	1.478 (0.996 - 2.044)	1.856 (1.252 - 2.573)
Age-standardized (rate per capita)	0.075 (0.073 - 0.078)	0.077 (0.074 - 0.079)	0.075 (0.072 - 0.078)	0.006 (0.006 - 0.006)	0.006 (0.006 - 0.006)	0.006 (0.006 - 0.006)	2.068 (1.397 - 2.853)	2.109 (1.428 - 2.910)	2.066 (1.396 - 2.866)
Total number of cases (thousands)	5,925 (5,725 - 6,131)	10,055 (9,731 - 10,406)	13,955 (13,489 - 14,448)	524 (503 - 546)	873 (837 - 910)	1,182 (1,132 - 1,233)	165 (111 - 227)	278 (188 - 385)	386 (260 - 535)
**Paraguay**									
15 - 49 years (rate per capita)	0.010 (0.010 - 0.011)	0.011 (0.010 - 0.011)	0.011 (0.010 - 0.011)	0.001 (0.001 - 0.001)	0.001 (0.001 - 0.001)	0.001 (0.001 - 0.001)	0.289 (0.193 - 0.403)	0.303 (0.201 - 0.427)	0.306 (0.202 - 0.426)
50 - 69 years (rate per capita)	0.137 (0.129 - 0.146)	0.131 (0.124 - 0.139)	0.136 (0.128 - 0.145)	0.014 (0.013 - 0.015)	0.014 (0.013 - 0.015)	0.014 (0.013 - 0.015)	3.827 (2.571 - 5.342)	3.646 (2.440 - 5.079)	3.777 (2.533 - 5.289)
70+ years (rate per capita)	0.312 (0.295 - 0.329)	0.307 (0.291 - 0.323)	0.307 (0.290 - 0.323)	0.014 (0.012 - 0.016)	0.013 (0.011 - 0.015)	0.013 (0.011 - 0.015)	8.409 (5.699 - 11.559)	8.229 (5.569 - 11.229)	8.198 (5.564 - 11.309)
All ages (rate per capita)	0.025 (0.024 - 0.026)	0.029 (0.028 - 0.030)	0.035 (0.033 - 0.036)	0.002 (0.002 - 0.002)	0.002 (0.002 - 0.003)	0.003 (0.003 - 0.003)	0.686 (0.460 - 0.950)	0.799 (0.539 - 1.101)	0.958 (0.642 - 1.329)
Age-standardized (rate per capita)	0.051 (0.049 - 0.054)	0.051 (0.049 - 0.053)	0.051 (0.049 - 0.053)	0.004 (0.004 - 0.004)	0.004 (0.004 - 0.004)	0.004 (0.004 - 0.004)	1.410 (0.949 - 1.942)	1.397 (0.938 - 1.925)	1.397 (0.938 - 1.939)
Total number of cases (thousands)	105 (100 - 109)	168 (161 - 175)	231 (222 - 241)	9 (8 - 9)	14 (13 - 15)	19 (18 - 20)	3 (2 - 4)	5 (3 - 6)	6 (4 - 9)
**Southeast Asia, East Asia, and Oceania**									
15 - 49 years (rate per capita)	0.006 (0.006 - 0.007)	0.008 (0.007 - 0.008)	0.009 (0.008 - 0.009)	0.001 (0.001 - 0.001)	0.001 (0.001 - 0.001)	0.001 (0.001 - 0.001)	0.183 (0.121 - 0.258)	0.218 (0.144 - 0.307)	0.246 (0.162 - 0.345)
50 - 69 years (rate per capita)	0.061 (0.057 - 0.065)	0.058 (0.055 - 0.063)	0.060 (0.056 - 0.065)	0.006 (0.005 - 0.007)	0.006 (0.005 - 0.006)	0.006 (0.005 - 0.006)	1.702 (1.116 - 2.394)	1.639 (1.074 - 2.297)	1.691 (1.109 - 2.381)
70+ years (rate per capita)	0.145 (0.136 - 0.156)	0.143 (0.135 - 0.154)	0.144 (0.135 - 0.155)	0.008 (0.007 - 0.010)	0.008 (0.007 - 0.009)	0.008 (0.007 - 0.009)	3.942 (2.625 - 5.457)	3.884 (2.592 - 5.384)	3.911 (2.602 - 5.447)
All ages (rate per capita)	0.015 (0.014 - 0.016)	0.019 (0.019 - 0.021)	0.024 (0.023 - 0.026)	0.001 (0.001 - 0.001)	0.002 (0.001 - 0.002)	0.002 (0.002 - 0.002)	0.421 (0.279 - 0.584)	0.544 (0.360 - 0.754)	0.679 (0.451 - 0.943)
Age-standardized (rate per capita)	0.024 (0.023 - 0.026)	0.024 (0.023 - 0.026)	0.024 (0.023 - 0.026)	0.002 (0.002 - 0.002)	0.002 (0.002 - 0.002)	0.002 (0.002 - 0.002)	0.675 (0.448 - 0.937)	0.671 (0.445 - 0.933)	0.676 (0.449 - 0.936)
Total number of cases (thousands)	25,071 (23,854 - 26,427)	37,977 (36,128 - 40,068)	51,021 (48,487 - 53,888)	2,082 (1,966 - 2,218)	3,091 (2,920 - 3,298)	4,188 (3,953 - 4,473)	700 (463 - 971)	1,060 (701 - 1,470)	1,423 (944 - 1,976)
**East Asia**									
15 - 49 years (rate per capita)	0.006 (0.005 - 0.006)	0.007 (0.007 - 0.007)	0.008 (0.008 - 0.008)	0.000 (0.000 - 0.001)	0.001 (0.001 - 0.001)	0.001 (0.001 - 0.001)	0.167 (0.111 - 0.234)	0.199 (0.133 - 0.278)	0.229 (0.153 - 0.320)
50 - 69 years (rate per capita)	0.058 (0.055 - 0.060)	0.055 (0.052 - 0.057)	0.057 (0.055 - 0.060)	0.006 (0.005 - 0.006)	0.005 (0.005 - 0.006)	0.006 (0.005 - 0.006)	1.618 (1.083 - 2.238)	1.537 (1.032 - 2.130)	1.609 (1.077 - 2.229)
70+ years (rate per capita)	0.141 (0.133 - 0.147)	0.138 (0.131 - 0.145)	0.138 (0.131 - 0.145)	0.008 (0.007 - 0.009)	0.008 (0.007 - 0.009)	0.008 (0.007 - 0.008)	3.828 (2.570 - 5.276)	3.752 (2.521 - 5.149)	3.763 (2.545 - 5.158)
All ages (rate per capita)	0.015 (0.015 - 0.016)	0.020 (0.019 - 0.021)	0.025 (0.024 - 0.026)	0.001 (0.001 - 0.001)	0.002 (0.002 - 0.002)	0.002 (0.002 - 0.002)	0.420 (0.282 - 0.581)	0.554 (0.373 - 0.766)	0.707 (0.476 - 0.978)
Age-standardized (rate per capita)	0.023 (0.022 - 0.024)	0.023 (0.022 - 0.024)	0.023 (0.022 - 0.024)	0.002 (0.002 - 0.002)	0.002 (0.002 - 0.002)	0.002 (0.002 - 0.002)	0.640 (0.432 - 0.884)	0.632 (0.427 - 0.872)	0.636 (0.429 - 0.879)
Total number of cases (thousands)	17,933 (17,329 - 18,589)	26,958 (26,058 - 27,963)	36,236 (35,022 - 37,613)	1,504 (1,444 - 1,565)	2,210 (2,120 - 2,303)	3,004 (2,882 - 3,128)	502 (337 - 694)	754 (507 - 1,041)	1,013 (682 - 1,401)
**China**									

	Prevalence 1990	Prevalence 2005	Prevalence 2015	Incidence 1990	Incidence 2005	Incidence 2015	YLD rate 1990	YLD rate 2005	YLD rate 2015
15 - 49 years (rate per capita)	0.006 (0.005 - 0.006)	0.007 (0.007 - 0.007)	0.008 (0.008 - 0.008)	0.000 (0.000 - 0.001)	0.001 (0.001 - 0.001)	0.001 (0.001 - 0.001)	0.166 (0.111 - 0.234)	0.199 (0.132 - 0.277)	0.229 (0.153 - 0.320)
50 - 69 years (rate per capita)	0.058 (0.055 - 0.060)	0.055 (0.052 - 0.057)	0.057 (0.055 - 0.060)	0.006 (0.005 - 0.006)	0.005 (0.005 - 0.006)	0.006 (0.005 - 0.006)	1.620 (1.084 - 2.241)	1.533 (1.030 - 2.126)	1.611 (1.079 - 2.233)
70+ years (rate per capita)	0.141 (0.134 - 0.147)	0.138 (0.131 - 0.145)	0.138 (0.131 - 0.145)	0.008 (0.007 - 0.009)	0.008 (0.007 - 0.009)	0.008 (0.007 - 0.008)	3.830 (2.572 - 5.278)	3.751 (2.519 - 5.146)	3.764 (2.544 - 5.156)
All ages (rate per capita)	0.015 (0.014 - 0.016)	0.020 (0.019 - 0.020)	0.025 (0.024 - 0.026)	0.001 (0.001 - 0.001)	0.002 (0.002 - 0.002)	0.002 (0.002 - 0.002)	0.419 (0.282 - 0.580)	0.553 (0.372 - 0.763)	0.706 (0.476 - 0.977)
Age-standardized (rate per capita)	0.023 (0.022 - 0.024)	0.023 (0.022 - 0.024)	0.023 (0.022 - 0.024)	0.002 (0.002 - 0.002)	0.002 (0.002 - 0.002)	0.002 (0.002 - 0.002)	0.640 (0.432 - 0.884)	0.632 (0.427 - 0.872)	0.636 (0.429 - 0.879)
Total number of cases (thousands)	17,310 (16,733 - 17,943)	25,960 (25,100 - 26,907)	34,951 (33,798 - 36,258)	1,451 (1,394 - 1,510)	2,129 (2,043 - 2,218)	2,901 (2,782 - 3,020)	484 (326 - 670)	726 (489 - 1,002)	977 (658 - 1,352)
**North Korea**									
15 - 49 years (rate per capita)	0.006 (0.006 - 0.007)	0.007 (0.007 - 0.008)	0.008 (0.007 - 0.008)	0.001 (0.001 - 0.001)	0.001 (0.001 - 0.001)	0.001 (0.001 - 0.001)	0.179 (0.118 - 0.250)	0.210 (0.138 - 0.295)	0.222 (0.145 - 0.313)
50 - 69 years (rate per capita)	0.053 (0.050 - 0.057)	0.063 (0.058 - 0.067)	0.056 (0.052 - 0.060)	0.005 (0.005 - 0.006)	0.006 (0.006 - 0.007)	0.006 (0.005 - 0.006)	1.491 (0.992 - 2.087)	1.750 (1.168 - 2.434)	1.577 (1.051 - 2.196)
70+ years (rate per capita)	0.139 (0.129 - 0.150)	0.140 (0.129 - 0.150)	0.140 (0.130 - 0.150)	0.008 (0.007 - 0.009)	0.008 (0.007 - 0.009)	0.008 (0.007 - 0.009)	3.793 (2.534 - 5.263)	3.794 (2.540 - 5.249)	3.793 (2.521 - 5.260)
All ages (rate per capita)	0.014 (0.014 - 0.015)	0.020 (0.019 - 0.021)	0.023 (0.022 - 0.025)	0.001 (0.001 - 0.001)	0.002 (0.002 - 0.002)	0.002 (0.002 - 0.002)	0.403 (0.269 - 0.554)	0.551 (0.369 - 0.762)	0.646 (0.431 - 0.892)
Age-standardized (rate per capita)	0.023 (0.022 - 0.024)	0.023 (0.022 - 0.024)	0.023 (0.022 - 0.024)	0.002 (0.002 - 0.002)	0.002 (0.002 - 0.002)	0.002 (0.002 - 0.002)	0.639 (0.428 - 0.882)	0.637 (0.427 - 0.878)	0.635 (0.425 - 0.879)
Total number of cases (thousands)	290 (275 - 306)	469 (446 - 496)	583 (554 - 617)	25 (24 - 27)	39 (37 - 42)	47 (44 - 50)	8 (5 - 11)	13 (9 - 18)	16 (11 - 22)
**Taiwan**									
15 - 49 years (rate per capita)	0.006 (0.006 - 0.007)	0.008 (0.007 - 0.008)	0.008 (0.007 - 0.008)	0.001 (0.000 - 0.001)	0.001 (0.001 - 0.001)	0.001 (0.001 - 0.001)	0.178 (0.117 - 0.254)	0.218 (0.144 - 0.305)	0.225 (0.147 - 0.317)
50 - 69 years (rate per capita)	0.059 (0.055 - 0.063)	0.056 (0.052 - 0.060)	0.055 (0.051 - 0.058)	0.006 (0.005 - 0.006)	0.005 (0.005 - 0.006)	0.005 (0.005 - 0.006)	1.644 (1.084 - 2.291)	1.557 (1.031 - 2.157)	1.530 (1.008 - 2.140)
70+ years (rate per capita)	0.139 (0.129 - 0.148)	0.139 (0.130 - 0.148)	0.137 (0.128 - 0.146)	0.008 (0.007 - 0.009)	0.008 (0.007 - 0.009)	0.007 (0.006 - 0.008)	3.760 (2.522 - 5.182)	3.748 (2.523 - 5.180)	3.701 (2.485 - 5.082)
All ages (rate per capita)	0.016 (0.016 - 0.017)	0.023 (0.022 - 0.024)	0.029 (0.028 - 0.031)	0.001 (0.001 - 0.001)	0.002 (0.002 - 0.002)	0.002 (0.002 - 0.002)	0.458 (0.306 - 0.632)	0.645 (0.430 - 0.888)	0.802 (0.535 - 1.103)
Age-standardized (rate per capita)	0.023 (0.022 - 0.024)	0.023 (0.022 - 0.024)	0.023 (0.022 - 0.024)	0.002 (0.002 - 0.002)	0.002 (0.002 - 0.002)	0.002 (0.002 - 0.002)	0.632 (0.422 - 0.869)	0.636 (0.425 - 0.876)	0.637 (0.426 - 0.877)
Total number of cases (thousands)	333 (317 - 351)	529 (503 - 556)	702 (667 - 739)	28 (26 - 29)	42 (39 - 44)	55 (52 - 58)	9 (6 - 13)	15 (10 - 20)	19 (13 - 27)
**Southeast Asia**									
15 - 49 years (rate per capita)	0.008 (0.007 - 0.010)	0.009 (0.008 - 0.011)	0.010 (0.008 - 0.012)	0.001 (0.001 - 0.001)	0.001 (0.001 - 0.001)	0.001 (0.001 - 0.001)	0.230 (0.146 - 0.332)	0.264 (0.168 - 0.386)	0.283 (0.180 - 0.416)
50 - 69 years (rate per capita)	0.071 (0.059 - 0.084)	0.071 (0.059 - 0.084)	0.070 (0.059 - 0.083)	0.006 (0.005 - 0.008)	0.006 (0.005 - 0.008)	0.006 (0.005 - 0.008)	1.967 (1.218 - 2.836)	1.975 (1.226 - 2.841)	1.949 (1.211 - 2.813)
70+ years (rate per capita)	0.161 (0.140 - 0.191)	0.162 (0.139 - 0.193)	0.165 (0.142 - 0.197)	0.009 (0.007 - 0.012)	0.009 (0.007 - 0.012)	0.009 (0.007 - 0.012)	4.327 (2.771 - 6.120)	4.338 (2.769 - 6.124)	4.429 (2.832 - 6.260)
All ages (rate per capita)	0.015 (0.013 - 0.017)	0.019 (0.016 - 0.021)	0.022 (0.020 - 0.025)	0.001 (0.001 - 0.001)	0.002 (0.001 - 0.002)	0.002 (0.002 - 0.002)	0.426 (0.273 - 0.601)	0.522 (0.335 - 0.739)	0.624 (0.399 - 0.883)
Age-standardized (rate per capita)	0.028 (0.025 - 0.032)	0.029 (0.025 - 0.033)	0.029 (0.025 - 0.033)	0.002 (0.002 - 0.003)	0.002 (0.002 - 0.003)	0.002 (0.002 - 0.003)	0.784 (0.498 - 1.100)	0.792 (0.504 - 1.113)	0.796 (0.506 - 1.119)
Total number of cases (thousands)	7,069 (6,146 - 7,989)	10,910 (9,486 - 12,317)	14,634 (12,714 - 16,549)	573 (497 - 662)	872 (759 - 1,015)	1,171 (1,014 - 1,368)	196 (126 - 277)	303 (194 - 429)	406 (260 - 575)
**Cambodia**									
15 - 49 years (rate per capita)	0.008 (0.006 - 0.012)	0.009 (0.006 - 0.012)	0.009 (0.007 - 0.012)	0.001 (0.001 - 0.001)	0.001 (0.001 - 0.001)	0.001 (0.001 - 0.001)	0.240 (0.142 - 0.365)	0.243 (0.145 - 0.372)	0.259 (0.154 - 0.391)
50 - 69 years (rate per capita)	0.076 (0.056 - 0.099)	0.077 (0.056 - 0.100)	0.077 (0.056 - 0.100)	0.007 (0.005 - 0.010)	0.007 (0.004 - 0.010)	0.007 (0.004 - 0.010)	2.114 (1.223 - 3.184)	2.127 (1.228 - 3.208)	2.136 (1.233 - 3.233)
70+ years (rate per capita)	0.182 (0.139 - 0.239)	0.180 (0.139 - 0.235)	0.181 (0.140 - 0.236)	0.011 (0.007 - 0.017)	0.011 (0.007 - 0.017)	0.011 (0.007 - 0.016)	4.848 (2.901 - 7.272)	4.797 (2.829 - 7.102)	4.861 (2.902 - 7.226)
All ages (rate per capita)	0.013 (0.010 - 0.016)	0.015 (0.012 - 0.018)	0.018 (0.014 - 0.022)	0.001 (0.001 - 0.001)	0.001 (0.001 - 0.002)	0.002 (0.001 - 0.002)	0.366 (0.222 - 0.534)	0.416 (0.255 - 0.611)	0.510 (0.311 - 0.747)
Age-standardized (rate per capita)	0.032 (0.025 - 0.038)	0.032 (0.025 - 0.039)	0.032 (0.025 - 0.039)	0.002 (0.002 - 0.003)	0.002 (0.002 - 0.003)	0.002 (0.002 - 0.003)	0.863 (0.524 - 1.254)	0.868 (0.527 - 1.264)	0.872 (0.528 - 1.276)
Total number of cases (thousands)	119 (93 - 144)	201 (156 - 242)	285 (222 - 345)	10 (8 - 12)	17 (13 - 21)	24 (18 - 30)	3 (2 - 5)	6 (3 - 8)	8 (5 - 12)
**Indonesia**									
15 - 49 years (rate per capita)	0.008 (0.006 - 0.011)	0.010 (0.007 - 0.013)	0.010 (0.008 - 0.014)	0.001 (0.001 - 0.001)	0.001 (0.001 - 0.001)	0.001 (0.001 - 0.001)	0.239 (0.144 - 0.362)	0.274 (0.164 - 0.414)	0.301 (0.178 - 0.461)
50 - 69 years (rate per capita)	0.076 (0.056 - 0.098)	0.077 (0.057 - 0.099)	0.074 (0.055 - 0.095)	0.007 (0.005 - 0.010)	0.007 (0.005 - 0.010)	0.007 (0.004 - 0.010)	2.115 (1.221 - 3.178)	2.141 (1.222 - 3.228)	2.066 (1.187 - 3.134)
70+ years (rate per capita)	0.180 (0.140 - 0.237)	0.180 (0.140 - 0.236)	0.181 (0.141 - 0.237)	0.011 (0.007 - 0.016)	0.011 (0.007 - 0.016)	0.011 (0.007 - 0.016)	4.822 (2.847 - 7.258)	4.822 (2.830 - 7.227)	4.838 (2.849 - 7.226)
All ages (rate per capita)	0.016 (0.013 - 0.020)	0.019 (0.015 - 0.023)	0.022 (0.017 - 0.027)	0.001 (0.001 - 0.002)	0.002 (0.001 - 0.002)	0.002 (0.001 - 0.002)	0.451 (0.275 - 0.662)	0.541 (0.332 - 0.796)	0.620 (0.377 - 0.909)
Age-standardized (rate per capita)	0.031 (0.025 - 0.038)	0.031 (0.025 - 0.038)	0.031 (0.025 - 0.038)	0.002 (0.002 - 0.003)	0.002 (0.002 - 0.003)	0.002 (0.002 - 0.003)	0.860 (0.522 - 1.260)	0.860 (0.523 - 1.257)	0.859 (0.520 - 1.252)
Total number of cases (thousands)	2,941 (2,304 - 3,548)	4,411 (3,472 - 5,302)	5,739 (4,502 - 6,928)	244 (194 - 306)	360 (286 - 450)	469 (369 - 594)	82 (50 - 120)	123 (75 - 180)	160 (97 - 234)
**Laos**									
15 - 49 years (rate per capita)	0.006 (0.005 - 0.006)	0.006 (0.005 - 0.006)	0.006 (0.006 - 0.007)	0.000 (0.000 - 0.001)	0.000 (0.000 - 0.001)	0.001 (0.000 - 0.001)	0.164 (0.108 - 0.230)	0.166 (0.110 - 0.235)	0.173 (0.115 - 0.241)
50 - 69 years (rate per capita)	0.055 (0.051 - 0.059)	0.053 (0.050 - 0.057)	0.053 (0.049 - 0.057)	0.005 (0.005 - 0.006)	0.005 (0.005 - 0.006)	0.005 (0.005 - 0.006)	1.515 (1.001 - 2.130)	1.478 (0.981 - 2.063)	1.473 (0.968 - 2.067)
70+ years (rate per capita)	0.128 (0.119 - 0.137)	0.128 (0.118 - 0.137)	0.128 (0.119 - 0.137)	0.008 (0.007 - 0.009)	0.007 (0.006 - 0.009)	0.007 (0.006 - 0.008)	3.439 (2.313 - 4.728)	3.414 (2.286 - 4.699)	3.434 (2.314 - 4.762)
All ages (rate per capita)	0.010 (0.010 - 0.011)	0.010 (0.010 - 0.011)	0.011 (0.011 - 0.012)	0.001 (0.001 - 0.001)	0.001 (0.001 - 0.001)	0.001 (0.001 - 0.001)	0.279 (0.186 - 0.387)	0.283 (0.190 - 0.394)	0.317 (0.211 - 0.437)
Age-standardized (rate per capita)	0.022 (0.021 - 0.023)	0.022 (0.021 - 0.023)	0.022 (0.021 - 0.023)	0.002 (0.002 - 0.002)	0.002 (0.002 - 0.002)	0.002 (0.002 - 0.002)	0.601 (0.401 - 0.832)	0.597 (0.402 - 0.831)	0.603 (0.402 - 0.833)
Total number of cases (thousands)	42 (40 - 45)	59 (56 - 62)	77 (73 - 82)	4 (3 - 4)	5 (5 - 5)	6 (6 - 7)	1 (1 - 2)	2 (1 - 2)	2 (1 - 3)
**Malaysia**									
15 - 49 years (rate per capita)	0.011 (0.010 - 0.011)	0.012 (0.011 - 0.012)	0.012 (0.011 - 0.013)	0.001 (0.001 - 0.001)	0.001 (0.001 - 0.001)	0.001 (0.001 - 0.001)	0.304 (0.200 - 0.428)	0.335 (0.220 - 0.469)	0.339 (0.225 - 0.474)
50 - 69 years (rate per capita)	0.085 (0.080 - 0.090)	0.085 (0.080 - 0.091)	0.087 (0.082 - 0.091)	0.008 (0.007 - 0.009)	0.008 (0.007 - 0.009)	0.008 (0.007 - 0.009)	2.373 (1.588 - 3.272)	2.380 (1.578 - 3.307)	2.414 (1.628 - 3.338)
70+ years (rate per capita)	0.201 (0.189 - 0.213)	0.201 (0.189 - 0.213)	0.200 (0.188 - 0.210)	0.011 (0.009 - 0.012)	0.011 (0.009 - 0.012)	0.011 (0.009 - 0.012)	5.393 (3.614 - 7.392)	5.396 (3.634 - 7.441)	5.355 (3.636 - 7.337)
All ages (rate per capita)	0.018 (0.017 - 0.019)	0.022 (0.021 - 0.023)	0.027 (0.026 - 0.028)	0.001 (0.001 - 0.001)	0.002 (0.002 - 0.002)	0.002 (0.002 - 0.002)	0.497 (0.334 - 0.682)	0.618 (0.414 - 0.851)	0.745 (0.503 - 1.024)
Age-standardized (rate per capita)	0.035 (0.034 - 0.037)	0.035 (0.034 - 0.037)	0.035 (0.034 - 0.036)	0.003 (0.002 - 0.003)	0.003 (0.002 - 0.003)	0.003 (0.002 - 0.003)	0.970 (0.653 - 1.332)	0.978 (0.659 - 1.345)	0.969 (0.654 - 1.333)
Total number of cases (thousands)	324 (311 - 338)	570 (547 - 594)	811 (779 - 844)	26 (24 - 27)	46 (43 - 48)	65 (62 - 68)	9 (6 - 12)	16 (11 - 22)	23 (15 - 31)
**Maldives**									
15 - 49 years (rate per capita)	0.008 (0.006 - 0.010)	0.008 (0.006 - 0.011)	0.009 (0.007 - 0.013)	0.001 (0.001 - 0.001)	0.001 (0.001 - 0.001)	0.001 (0.001 - 0.001)	0.221 (0.133 - 0.341)	0.234 (0.138 - 0.361)	0.270 (0.160 - 0.419)
50 - 69 years (rate per capita)	0.072 (0.054 - 0.093)	0.082 (0.061 - 0.108)	0.073 (0.053 - 0.094)	0.007 (0.004 - 0.010)	0.007 (0.005 - 0.010)	0.007 (0.004 - 0.010)	2.009 (1.172 - 3.006)	2.290 (1.323 - 3.496)	2.041 (1.174 - 3.120)
70+ years (rate per capita)	0.170 (0.134 - 0.219)	0.171 (0.135 - 0.219)	0.177 (0.139 - 0.228)	0.010 (0.007 - 0.015)	0.010 (0.007 - 0.015)	0.010 (0.007 - 0.015)	4.562 (2.715 - 6.731)	4.622 (2.775 - 6.806)	4.772 (2.855 - 7.014)
All ages (rate per capita)	0.013 (0.010 - 0.015)	0.016 (0.012 - 0.019)	0.019 (0.015 - 0.023)	0.001 (0.001 - 0.001)	0.001 (0.001 - 0.002)	0.002 (0.001 - 0.002)	0.350 (0.214 - 0.514)	0.442 (0.271 - 0.645)	0.543 (0.330 - 0.799)
Age-standardized (rate per capita)	0.030 (0.024 - 0.036)	0.031 (0.024 - 0.037)	0.031 (0.024 - 0.038)	0.002 (0.002 - 0.003)	0.002 (0.002 - 0.003)	0.002 (0.002 - 0.003)	0.829 (0.507 - 1.207)	0.849 (0.517 - 1.236)	0.859 (0.523 - 1.253)
Total number of cases (thousands)	3 (2 - 3)	5 (4 - 6)	7 (6 - 8)	0 (0 - 0)	0 (0 - 0)	1 (0 - 1)	0 (0 - 0)	0 (0 - 0)	0 (0 - 0)
**Mauritius**									
15 - 49 years (rate per capita)	0.014 (0.013 - 0.015)	0.017 (0.016 - 0.019)	0.017 (0.016 - 0.018)	0.001 (0.001 - 0.001)	0.001 (0.001 - 0.002)	0.001 (0.001 - 0.001)	0.411 (0.270 - 0.577)	0.500 (0.332 - 0.703)	0.482 (0.316 - 0.680)
50 - 69 years (rate per capita)	0.108 (0.101 - 0.116)	0.104 (0.097 - 0.112)	0.108 (0.100 - 0.116)	0.010 (0.009 - 0.011)	0.009 (0.008 - 0.011)	0.010 (0.009 - 0.011)	3.017 (2.003 - 4.213)	2.911 (1.916 - 4.056)	2.993 (1.981 - 4.200)
70+ years (rate per capita)	0.252 (0.234 - 0.270)	0.248 (0.231 - 0.265)	0.244 (0.228 - 0.261)	0.013 (0.011 - 0.015)	0.012 (0.010 - 0.014)	0.012 (0.010 - 0.013)	6.821 (4.614 - 9.399)	6.675 (4.472 - 9.266)	6.570 (4.412 - 9.055)
All ages (rate per capita)	0.027 (0.026 - 0.029)	0.037 (0.035 - 0.039)	0.047 (0.045 - 0.050)	0.002 (0.002 - 0.002)	0.003 (0.003 - 0.003)	0.004 (0.003 - 0.004)	0.760 (0.508 - 1.049)	1.025 (0.683 - 1.416)	1.304 (0.873 - 1.804)
Age-standardized (rate per capita)	0.044 (0.042 - 0.046)	0.044 (0.042 - 0.047)	0.044 (0.041 - 0.046)	0.003 (0.003 - 0.003)	0.003 (0.003 - 0.003)	0.003 (0.003 - 0.003)	1.221 (0.817 - 1.681)	1.227 (0.818 - 1.703)	1.204 (0.805 - 1.666)
Total number of cases (thousands)	29 (27 - 30)	45 (43 - 47)	60 (57 - 63)	2 (2 - 2)	3 (3 - 4)	5 (4 - 5)	1 (1 - 1)	1 (1 - 2)	2 (1 - 2)
**Myanmar**									
15 - 49 years (rate per capita)	0.005 (0.005 - 0.006)	0.006 (0.005 - 0.006)	0.006 (0.006 - 0.007)	0.000 (0.000 - 0.000)	0.000 (0.000 - 0.001)	0.001 (0.000 - 0.001)	0.146 (0.096 - 0.205)	0.163 (0.108 - 0.231)	0.177 (0.116 - 0.251)
50 - 69 years (rate per capita)	0.049 (0.045 - 0.053)	0.046 (0.043 - 0.050)	0.047 (0.043 - 0.051)	0.005 (0.004 - 0.005)	0.004 (0.004 - 0.005)	0.005 (0.004 - 0.005)	1.359 (0.898 - 1.883)	1.279 (0.846 - 1.785)	1.301 (0.858 - 1.826)
70+ years (rate per capita)	0.116 (0.107 - 0.125)	0.115 (0.106 - 0.124)	0.116 (0.107 - 0.125)	0.007 (0.006 - 0.008)	0.007 (0.006 - 0.008)	0.007 (0.006 - 0.008)	3.097 (2.099 - 4.253)	3.055 (2.061 - 4.214)	3.086 (2.090 - 4.228)
All ages (rate per capita)	0.010 (0.010 - 0.011)	0.012 (0.011 - 0.013)	0.014 (0.013 - 0.015)	0.001 (0.001 - 0.001)	0.001 (0.001 - 0.001)	0.001 (0.001 - 0.001)	0.286 (0.192 - 0.397)	0.329 (0.220 - 0.455)	0.392 (0.262 - 0.544)

	Prevalence 1990	Prevalence 2005	Prevalence 2015	Incidence 1990	Incidence 2005	Incidence 2015	YLD rate 1990	YLD rate 2005	YLD rate 2015
Age-standardized (rate per capita)	0.020 (0.018 - 0.021)	0.019 (0.018 - 0.021)	0.020 (0.018 - 0.021)	0.002 (0.001 - 0.002)	0.002 (0.001 - 0.002)	0.002 (0.001 - 0.002)	0.534 (0.357 - 0.740)	0.528 (0.354 - 0.729)	0.534 (0.359 - 0.741)
Total number of cases (thousands)	436 (410 - 463)	596 (561 - 633)	765 (721 - 813)	36 (34 - 39)	49 (46 - 53)	64 (60 - 69)	12 (8 - 17)	16 (11 - 23)	21 (14 - 29)
**Philippines**									
15 - 49 years (rate per capita)	0.010 (0.010 - 0.011)	0.012 (0.011 - 0.013)	0.012 (0.011 - 0.013)	0.001 (0.001 - 0.001)	0.001 (0.001 - 0.001)	0.001 (0.001 - 0.001)	0.299 (0.200 - 0.421)	0.333 (0.220 - 0.468)	0.336 (0.222 - 0.475)
50 - 69 years (rate per capita)	0.086 (0.080 - 0.093)	0.085 (0.079 - 0.091)	0.086 (0.080 - 0.093)	0.008 (0.007 - 0.009)	0.008 (0.007 - 0.008)	0.008 (0.007 - 0.009)	2.391 (1.579 - 3.339)	2.338 (1.545 - 3.269)	2.370 (1.575 - 3.282)
70+ years (rate per capita)	0.201 (0.186 - 0.214)	0.200 (0.186 - 0.214)	0.201 (0.187 - 0.214)	0.011 (0.009 - 0.012)	0.011 (0.009 - 0.012)	0.010 (0.009 - 0.012)	5.356 (3.605 - 7.376)	5.331 (3.592 - 7.401)	5.350 (3.611 - 7.386)
All ages (rate per capita)	0.016 (0.015 - 0.017)	0.018 (0.017 - 0.019)	0.022 (0.021 - 0.024)	0.001 (0.001 - 0.001)	0.001 (0.001 - 0.002)	0.002 (0.002 - 0.002)	0.436 (0.291 - 0.600)	0.506 (0.338 - 0.701)	0.619 (0.415 - 0.855)
Age-standardized (rate per capita)	0.035 (0.034 - 0.037)	0.036 (0.034 - 0.038)	0.035 (0.034 - 0.037)	0.003 (0.002 - 0.003)	0.003 (0.002 - 0.003)	0.003 (0.002 - 0.003)	0.972 (0.648 - 1.339)	0.980 (0.657 - 1.358)	0.971 (0.650 - 1.340)
Total number of cases (thousands)	972 (925 - 1,024)	1,570 (1,492 - 1,658)	2,255 (2,144 - 2,377)	76 (72 - 81)	124 (117 - 131)	178 (168 - 190)	27 (18 - 37)	44 (29 - 60)	62 (42 - 86)
**Sri Lanka**									
15 - 49 years (rate per capita)	0.008 (0.007 - 0.008)	0.009 (0.008 - 0.009)	0.009 (0.009 - 0.010)	0.001 (0.001 - 0.001)	0.001 (0.001 - 0.001)	0.001 (0.001 - 0.001)	0.223 (0.148 - 0.312)	0.250 (0.167 - 0.354)	0.262 (0.173 - 0.368)
50 - 69 years (rate per capita)	0.064 (0.060 - 0.069)	0.063 (0.059 - 0.067)	0.066 (0.062 - 0.070)	0.006 (0.005 - 0.006)	0.006 (0.005 - 0.006)	0.006 (0.006 - 0.007)	1.790 (1.186 - 2.501)	1.742 (1.159 - 2.432)	1.827 (1.216 - 2.548)
70+ years (rate per capita)	0.147 (0.137 - 0.156)	0.147 (0.138 - 0.156)	0.147 (0.137 - 0.156)	0.008 (0.007 - 0.009)	0.008 (0.007 - 0.009)	0.008 (0.007 - 0.009)	3.950 (2.684 - 5.475)	3.937 (2.677 - 5.436)	3.931 (2.651 - 5.469)
All ages (rate per capita)	0.017 (0.016 - 0.017)	0.021 (0.020 - 0.022)	0.026 (0.024 - 0.027)	0.001 (0.001 - 0.001)	0.002 (0.002 - 0.002)	0.002 (0.002 - 0.002)	0.461 (0.310 - 0.633)	0.586 (0.394 - 0.813)	0.709 (0.477 - 0.982)
Age-standardized (rate per capita)	0.025 (0.024 - 0.027)	0.026 (0.024 - 0.027)	0.026 (0.025 - 0.027)	0.002 (0.002 - 0.002)	0.002 (0.002 - 0.002)	0.002 (0.002 - 0.002)	0.703 (0.474 - 0.968)	0.705 (0.474 - 0.975)	0.707 (0.474 - 0.978)
Total number of cases (thousands)	287 (275 - 300)	413 (395 - 433)	532 (508 - 557)	23 (22 - 24)	32 (31 - 34)	42 (39 - 44)	8 (5 - 11)	11 (8 - 16)	15 (10 - 20)
**Seychelles**									
15 - 49 years (rate per capita)	0.008 (0.006 - 0.011)	0.010 (0.008 - 0.014)	0.011 (0.008 - 0.015)	0.001 (0.001 - 0.001)	0.001 (0.001 - 0.001)	0.001 (0.001 - 0.002)	0.220 (0.134 - 0.334)	0.301 (0.179 - 0.466)	0.324 (0.194 - 0.498)
50 - 69 years (rate per capita)	0.082 (0.060 - 0.107)	0.075 (0.056 - 0.097)	0.072 (0.053 - 0.094)	0.007 (0.005 - 0.011)	0.007 (0.005 - 0.010)	0.007 (0.004 - 0.010)	2.294 (1.313 - 3.476)	2.097 (1.206 - 3.155)	2.033 (1.172 - 3.111)
70+ years (rate per capita)	0.184 (0.143 - 0.239)	0.184 (0.143 - 0.239)	0.184 (0.143 - 0.238)	0.010 (0.007 - 0.015)	0.010 (0.007 - 0.015)	0.010 (0.007 - 0.014)	4.978 (2.945 - 7.402)	4.958 (2.944 - 7.359)	4.946 (2.944 - 7.274)
All ages (rate per capita)	0.021 (0.016 - 0.026)	0.024 (0.019 - 0.029)	0.028 (0.022 - 0.034)	0.002 (0.001 - 0.002)	0.002 (0.001 - 0.002)	0.002 (0.002 - 0.003)	0.586 (0.356 - 0.856)	0.663 (0.410 - 0.959)	0.787 (0.481 - 1.155)
Age-standardized (rate per capita)	0.031 (0.024 - 0.038)	0.031 (0.024 - 0.038)	0.031 (0.024 - 0.038)	0.002 (0.002 - 0.003)	0.002 (0.002 - 0.003)	0.002 (0.002 - 0.003)	0.874 (0.529 - 1.276)	0.870 (0.530 - 1.272)	0.865 (0.529 - 1.264)
Total number of cases (thousands)	1 (1 - 2)	2 (2 - 3)	3 (2 - 3)	0 (0 - 0)	0 (0 - 0)	0 (0 - 0)	0 (0 - 0)	0 (0 - 0)	0 (0 - 0)
**Thailand**									
15 - 49 years (rate per capita)	0.009 (0.006 - 0.012)	0.011 (0.008 - 0.015)	0.012 (0.009 - 0.017)	0.001 (0.001 - 0.001)	0.001 (0.001 - 0.002)	0.001 (0.001 - 0.002)	0.256 (0.151 - 0.394)	0.323 (0.190 - 0.499)	0.352 (0.206 - 0.545)
50 - 69 years (rate per capita)	0.076 (0.056 - 0.098)	0.076 (0.057 - 0.099)	0.077 (0.057 - 0.099)	0.007 (0.004 - 0.010)	0.007 (0.004 - 0.010)	0.007 (0.004 - 0.010)	2.123 (1.231 - 3.211)	2.134 (1.237 - 3.262)	2.154 (1.252 - 3.244)
70+ years (rate per capita)	0.180 (0.141 - 0.232)	0.179 (0.141 - 0.232)	0.180 (0.142 - 0.234)	0.010 (0.007 - 0.016)	0.010 (0.007 - 0.016)	0.010 (0.007 - 0.015)	4.847 (2.895 - 7.230)	4.833 (2.884 - 7.181)	4.844 (2.907 - 7.235)
All ages (rate per capita)	0.019 (0.015 - 0.023)	0.028 (0.022 - 0.033)	0.037 (0.029 - 0.044)	0.002 (0.001 - 0.002)	0.002 (0.002 - 0.003)	0.003 (0.002 - 0.004)	0.526 (0.319 - 0.777)	0.772 (0.470 - 1.143)	1.015 (0.612 - 1.509)
Age-standardized (rate per capita)	0.031 (0.025 - 0.038)	0.031 (0.025 - 0.038)	0.031 (0.025 - 0.038)	0.002 (0.002 - 0.003)	0.002 (0.002 - 0.003)	0.002 (0.002 - 0.003)	0.864 (0.520 - 1.264)	0.863 (0.519 - 1.266)	0.862 (0.522 - 1.271)
Total number of cases (thousands)	1,067 (842 - 1,286)	1,822 (1,435 - 2,200)	2,483 (1,944 - 3,021)	86 (68 - 108)	144 (114 - 181)	192 (151 - 245)	30 (18 - 44)	51 (31 - 75)	69 (42 - 102)
**Timor-Leste**									
15 - 49 years (rate per capita)	0.008 (0.006 - 0.011)	0.008 (0.006 - 0.011)	0.008 (0.006 - 0.011)	0.001 (0.001 - 0.001)	0.001 (0.001 - 0.001)	0.001 (0.001 - 0.001)	0.236 (0.140 - 0.363)	0.234 (0.139 - 0.359)	0.236 (0.140 - 0.364)
50 - 69 years (rate per capita)	0.072 (0.053 - 0.093)	0.075 (0.056 - 0.097)	0.084 (0.063 - 0.109)	0.006 (0.004 - 0.010)	0.007 (0.005 - 0.010)	0.007 (0.005 - 0.010)	2.025 (1.160 - 3.092)	2.109 (1.211 - 3.179)	2.337 (1.341 - 3.570)
70+ years (rate per capita)	0.178 (0.138 - 0.235)	0.180 (0.140 - 0.237)	0.178 (0.139 - 0.234)	0.011 (0.007 - 0.017)	0.011 (0.007 - 0.017)	0.011 (0.007 - 0.017)	4.793 (2.813 - 7.171)	4.840 (2.857 - 7.301)	4.778 (2.809 - 7.120)
All ages (rate per capita)	0.011 (0.009 - 0.013)	0.012 (0.010 - 0.015)	0.017 (0.013 - 0.020)	0.001 (0.001 - 0.001)	0.001 (0.001 - 0.001)	0.001 (0.001 - 0.002)	0.306 (0.184 - 0.452)	0.341 (0.206 - 0.501)	0.462 (0.281 - 0.683)
Age-standardized (rate per capita)	0.031 (0.025 - 0.038)	0.031 (0.025 - 0.038)	0.031 (0.025 - 0.038)	0.002 (0.002 - 0.003)	0.002 (0.002 - 0.003)	0.002 (0.002 - 0.003)	0.862 (0.522 - 1.264)	0.861 (0.520 - 1.259)	0.858 (0.520 - 1.263)
Total number of cases (thousands)	8 (6 - 10)	12 (10 - 15)	20 (16 - 24)	1 (1 - 1)	1 (1 - 1)	2 (1 - 2)	0 (0 - 0)	0 (0 - 0)	1 (0 - 1)
**Vietnam**									
15 - 49 years (rate per capita)	0.005 (0.005 - 0.006)	0.006 (0.006 - 0.007)	0.007 (0.006 - 0.007)	0.000 (0.000 - 0.000)	0.001 (0.000 - 0.001)	0.001 (0.001 - 0.001)	0.148 (0.097 - 0.207)	0.176 (0.116 - 0.249)	0.192 (0.127 - 0.272)
50 - 69 years (rate per capita)	0.052 (0.048 - 0.055)	0.047 (0.044 - 0.051)	0.047 (0.043 - 0.050)	0.005 (0.004 - 0.005)	0.004 (0.004 - 0.005)	0.004 (0.004 - 0.005)	1.434 (0.955 - 1.988)	1.325 (0.875 - 1.839)	1.305 (0.858 - 1.815)
70+ years (rate per capita)	0.117 (0.109 - 0.125)	0.116 (0.108 - 0.123)	0.116 (0.108 - 0.124)	0.006 (0.006 - 0.007)	0.006 (0.005 - 0.007)	0.006 (0.005 - 0.007)	3.136 (2.111 - 4.318)	3.092 (2.094 - 4.257)	3.103 (2.085 - 4.251)
All ages (rate per capita)	0.012 (0.012 - 0.013)	0.014 (0.013 - 0.015)	0.017 (0.016 - 0.018)	0.001 (0.001 - 0.001)	0.001 (0.001 - 0.001)	0.001 (0.001 - 0.001)	0.335 (0.224 - 0.463)	0.390 (0.260 - 0.535)	0.467 (0.310 - 0.644)
Age-standardized (rate per capita)	0.020 (0.019 - 0.021)	0.020 (0.019 - 0.021)	0.020 (0.019 - 0.021)	0.002 (0.001 - 0.002)	0.002 (0.001 - 0.002)	0.002 (0.001 - 0.002)	0.550 (0.369 - 0.760)	0.547 (0.365 - 0.752)	0.553 (0.366 - 0.765)
Total number of cases (thousands)	828 (790 - 873)	1,187 (1,136 - 1,245)	1,570 (1,498 - 1,652)	64 (60 - 67)	90 (85 - 95)	122 (115 - 129)	23 (15 - 32)	33 (22 - 45)	44 (29 - 60)
**Oceania**									
15 - 49 years (rate per capita)	0.007 (0.006 - 0.007)	0.007 (0.007 - 0.008)	0.007 (0.007 - 0.008)	0.001 (0.001 - 0.001)	0.001 (0.001 - 0.001)	0.001 (0.001 - 0.001)	0.189 (0.125 - 0.265)	0.204 (0.134 - 0.285)	0.209 (0.137 - 0.294)
50 - 69 years (rate per capita)	0.060 (0.056 - 0.065)	0.060 (0.055 - 0.065)	0.061 (0.056 - 0.066)	0.006 (0.005 - 0.006)	0.006 (0.005 - 0.006)	0.006 (0.005 - 0.006)	1.662 (1.101 - 2.308)	1.647 (1.093 - 2.312)	1.667 (1.108 - 2.327)
70+ years (rate per capita)	0.148 (0.138 - 0.159)	0.148 (0.137 - 0.159)	0.148 (0.137 - 0.159)	0.008 (0.007 - 0.010)	0.008 (0.007 - 0.010)	0.008 (0.007 - 0.009)	3.930 (2.671 - 5.429)	3.912 (2.661 - 5.380)	3.912 (2.638 - 5.396)
All ages (rate per capita)	0.010 (0.010 - 0.011)	0.012 (0.011 - 0.013)	0.014 (0.013 - 0.015)	0.001 (0.001 - 0.001)	0.001 (0.001 - 0.001)	0.001 (0.001 - 0.001)	0.288 (0.193 - 0.398)	0.330 (0.220 - 0.457)	0.377 (0.252 - 0.521)
Age-standardized (rate per capita)	0.025 (0.024 - 0.027)	0.025 (0.024 - 0.027)	0.025 (0.024 - 0.027)	0.002 (0.002 - 0.002)	0.002 (0.002 - 0.002)	0.002 (0.002 - 0.002)	0.685 (0.460 - 0.944)	0.685 (0.459 - 0.945)	0.685 (0.460 - 0.946)
Total number of cases (thousands)	69 (65 - 73)	109 (103 - 116)	150 (142 - 160)	6 (5 - 6)	9 (9 - 10)	13 (12 - 13)	2 (1 - 3)	3 (2 - 4)	4 (3 - 6)
**American Samoa**									
15 - 49 years (rate per capita)	0.007 (0.006 - 0.008)	0.008 (0.007 - 0.009)	0.009 (0.008 - 0.010)	0.001 (0.001 - 0.001)	0.001 (0.001 - 0.001)	0.001 (0.001 - 0.001)	0.196 (0.131 - 0.275)	0.232 (0.151 - 0.324)	0.258 (0.169 - 0.361)
50 - 69 years (rate per capita)	0.061 (0.057 - 0.066)	0.060 (0.055 - 0.064)	0.058 (0.054 - 0.063)	0.006 (0.005 - 0.006)	0.006 (0.005 - 0.006)	0.006 (0.005 - 0.006)	1.696 (1.131 - 2.376)	1.641 (1.098 - 2.286)	1.610 (1.073 - 2.258)
70+ years (rate per capita)	0.146 (0.136 - 0.158)	0.147 (0.137 - 0.159)	0.148 (0.137 - 0.160)	0.008 (0.007 - 0.009)	0.008 (0.007 - 0.009)	0.008 (0.007 - 0.010)	3.899 (2.619 - 5.378)	3.920 (2.648 - 5.447)	3.937 (2.646 - 5.407)
All ages (rate per capita)	0.012 (0.011 - 0.012)	0.013 (0.012 - 0.013)	0.013 (0.012 - 0.014)	0.001 (0.001 - 0.001)	0.001 (0.001 - 0.001)	0.001 (0.001 - 0.001)	0.323 (0.218 - 0.445)	0.349 (0.232 - 0.485)	0.359 (0.242 - 0.497)
Age-standardized (rate per capita)	0.025 (0.024 - 0.027)	0.025 (0.024 - 0.027)	0.025 (0.024 - 0.027)	0.002 (0.002 - 0.002)	0.002 (0.002 - 0.002)	0.002 (0.002 - 0.002)	0.689 (0.461 - 0.949)	0.690 (0.464 - 0.949)	0.691 (0.464 - 0.953)
Total number of cases (thousands)	1 (1 - 1)	1 (1 - 1)	1 (1 - 1)	0 (0 - 0)	0 (0 - 0)	0 (0 - 0)	0 (0 - 0)	0 (0 - 0)	0 (0 - 0)
**Federated States of Micronesia**									
15 - 49 years (rate per capita)	0.006 (0.006 - 0.007)	0.007 (0.006 - 0.007)	0.006 (0.006 - 0.007)	0.001 (0.000 - 0.001)	0.001 (0.001 - 0.001)	0.001 (0.000 - 0.001)	0.179 (0.118 - 0.255)	0.188 (0.124 - 0.265)	0.172 (0.113 - 0.242)
50 - 69 years (rate per capita)	0.065 (0.060 - 0.071)	0.058 (0.053 - 0.062)	0.064 (0.058 - 0.069)	0.006 (0.006 - 0.007)	0.006 (0.005 - 0.006)	0.006 (0.005 - 0.007)	1.801 (1.198 - 2.527)	1.591 (1.051 - 2.222)	1.753 (1.161 - 2.460)
70+ years (rate per capita)	0.148 (0.137 - 0.160)	0.146 (0.135 - 0.158)	0.147 (0.137 - 0.159)	0.008 (0.007 - 0.010)	0.008 (0.007 - 0.009)	0.008 (0.007 - 0.009)	3.957 (2.663 - 5.467)	3.885 (2.614 - 5.339)	3.912 (2.629 - 5.431)
All ages (rate per capita)	0.011 (0.010 - 0.012)	0.012 (0.012 - 0.013)	0.014 (0.014 - 0.015)	0.001 (0.001 - 0.001)	0.001 (0.001 - 0.001)	0.001 (0.001 - 0.001)	0.300 (0.200 - 0.416)	0.343 (0.229 - 0.475)	0.396 (0.263 - 0.550)
Age-standardized (rate per capita)	0.025 (0.024 - 0.027)	0.025 (0.024 - 0.027)	0.025 (0.024 - 0.027)	0.002 (0.002 - 0.002)	0.002 (0.002 - 0.002)	0.002 (0.002 - 0.002)	0.691 (0.462 - 0.957)	0.691 (0.463 - 0.955)	0.690 (0.461 - 0.953)
Total number of cases (thousands)	1 (1 - 1)	1 (1 - 1)	2 (1 - 2)	0 (0 - 0)	0 (0 - 0)	0 (0 - 0)	0 (0 - 0)	0 (0 - 0)	0 (0 - 0)
**Fiji**									
15 - 49 years (rate per capita)	0.007 (0.006 - 0.008)	0.008 (0.007 - 0.009)	0.008 (0.007 - 0.009)	0.001 (0.001 - 0.001)	0.001 (0.001 - 0.001)	0.001 (0.001 - 0.001)	0.200 (0.130 - 0.279)	0.227 (0.150 - 0.318)	0.225 (0.148 - 0.316)
50 - 69 years (rate per capita)	0.060 (0.055 - 0.064)	0.061 (0.056 - 0.065)	0.061 (0.056 - 0.066)	0.006 (0.005 - 0.006)	0.006 (0.005 - 0.006)	0.006 (0.005 - 0.006)	1.635 (1.082 - 2.280)	1.659 (1.087 - 2.324)	1.677 (1.110 - 2.336)
70+ years (rate per capita)	0.148 (0.136 - 0.159)	0.148 (0.137 - 0.159)	0.148 (0.137 - 0.160)	0.008 (0.007 - 0.010)	0.008 (0.007 - 0.010)	0.008 (0.007 - 0.010)	3.914 (2.625 - 5.415)	3.909 (2.626 - 5.430)	3.918 (2.623 - 5.430)
All ages (rate per capita)	0.011 (0.011 - 0.012)	0.015 (0.014 - 0.016)	0.019 (0.018 - 0.020)	0.001 (0.001 - 0.001)	0.001 (0.001 - 0.001)	0.002 (0.001 - 0.002)	0.311 (0.206 - 0.430)	0.421 (0.279 - 0.583)	0.519 (0.344 - 0.712)
Age-standardized (rate per capita)	0.025 (0.024 - 0.027)	0.025 (0.024 - 0.027)	0.025 (0.024 - 0.027)	0.002 (0.002 - 0.002)	0.002 (0.002 - 0.002)	0.002 (0.002 - 0.002)	0.684 (0.456 - 0.944)	0.684 (0.455 - 0.943)	0.684 (0.456 - 0.941)
Total number of cases (thousands)	8 (8 - 9)	13 (12 - 13)	17 (16 - 18)	1 (1 - 1)	1 (1 - 1)	1 (1 - 2)	0 (0 - 0)	0 (0 - 0)	0 (0 - 1)
**Guam**									
15 - 49 years (rate per capita)	0.007 (0.007 - 0.008)	0.008 (0.008 - 0.009)	0.008 (0.008 - 0.009)	0.001 (0.001 - 0.001)	0.001 (0.001 - 0.001)	0.001 (0.001 - 0.001)	0.208 (0.138 - 0.294)	0.242 (0.159 - 0.338)	0.236 (0.155 - 0.334)
50 - 69 years (rate per capita)	0.063 (0.058 - 0.068)	0.062 (0.057 - 0.067)	0.064 (0.059 - 0.069)	0.006 (0.005 - 0.007)	0.006 (0.005 - 0.007)	0.006 (0.005 - 0.007)	1.758 (1.148 - 2.440)	1.721 (1.136 - 2.408)	1.766 (1.171 - 2.462)

	Prevalence 1990	Prevalence 2005	Prevalence 2015	Incidence 1990	Incidence 2005	Incidence 2015	YLD rate 1990	YLD rate 2005	YLD rate 2015
70+ years (rate per capita)	0.149<br>(0.138 - 0.160)	0.148<br>(0.137 - 0.159)	0.148<br>(0.138 - 0.159)	0.008<br>(0.007 - 0.010)	0.008<br>(0.007 - 0.009)	0.008<br>(0.007 - 0.009)	4.008<br>(2.712 - 5.615)	3.972<br>(2.712 - 5.491)	3.968<br>(2.686 - 5.456)
All ages (rate per capita)	0.014<br>(0.013 - 0.015)	0.019<br>(0.018 - 0.021)	0.024<br>(0.023 - 0.026)	0.001<br>(0.001 - 0.001)	0.002<br>(0.001 - 0.002)	0.002<br>(0.002 - 0.002)	0.399<br>(0.265 - 0.554)	0.537<br>(0.360 - 0.746)	0.670<br>(0.448 - 0.930)
Age-standardized (rate per capita)	0.025<br>(0.024 - 0.027)	0.025<br>(0.024 - 0.027)	0.025<br>(0.024 - 0.027)	0.002<br>(0.002 - 0.002)	0.002<br>(0.002 - 0.002)	0.002<br>(0.002 - 0.002)	0.698<br>(0.466 - 0.971)	0.699<br>(0.470 - 0.968)	0.697<br>(0.466 - 0.968)
Total number of cases (thousands)	2<br>(2 - 2)	3<br>(3 - 3)	4<br>(4 - 4)	0<br>(0 - 0)	0<br>(0 - 0)	0<br>(0 - 0)	0<br>(0 - 0)	0<br>(0 - 0)	0<br>(0 - 0)
**Kiribati**									
15 - 49 years (rate per capita)	0.007<br>(0.006 - 0.008)	0.007<br>(0.007 - 0.008)	0.007<br>(0.007 - 0.008)	0.001<br>(0.001 - 0.001)	0.001<br>(0.001 - 0.001)	0.001<br>(0.001 - 0.001)	0.198<br>(0.132 - 0.279)	0.204<br>(0.133 - 0.286)	0.207<br>(0.134 - 0.293)
50 - 69 years (rate per capita)	0.062<br>(0.057 - 0.067)	0.060<br>(0.056 - 0.065)	0.059<br>(0.054 - 0.064)	0.006<br>(0.005 - 0.007)	0.006<br>(0.005 - 0.006)	0.006<br>(0.005 - 0.006)	1.701<br>(1.140 - 2.377)	1.653<br>(1.099 - 2.293)	1.621<br>(1.072 - 2.277)
70+ years (rate per capita)	0.149<br>(0.138 - 0.159)	0.149<br>(0.138 - 0.159)	0.149<br>(0.138 - 0.160)	0.008<br>(0.007 - 0.010)	0.008<br>(0.007 - 0.010)	0.008<br>(0.007 - 0.010)	3.946<br>(2.687 - 5.423)	3.932<br>(2.648 - 5.375)	3.951<br>(2.675 - 5.411)
All ages (rate per capita)	0.012<br>(0.011 - 0.013)	0.012<br>(0.012 - 0.013)	0.014<br>(0.013 - 0.015)	0.001<br>(0.001 - 0.001)	0.001<br>(0.001 - 0.001)	0.001<br>(0.001 - 0.001)	0.330<br>(0.221 - 0.455)	0.339<br>(0.226 - 0.467)	0.379<br>(0.253 - 0.527)
Age-standardized (rate per capita)	0.025<br>(0.024 - 0.027)	0.025<br>(0.024 - 0.027)	0.025<br>(0.024 - 0.027)	0.002<br>(0.002 - 0.002)	0.002<br>(0.002 - 0.002)	0.002<br>(0.002 - 0.002)	0.688<br>(0.461 - 0.948)	0.687<br>(0.462 - 0.944)	0.688<br>(0.458 - 0.951)
Total number of cases (thousands)	1<br>(1 - 1)	1<br>(1 - 1)	2<br>(1 - 2)	0<br>(0 - 0)	0<br>(0 - 0)	0<br>(0 - 0)	0<br>(0 - 0)	0<br>(0 - 0)	0<br>(0 - 0)
**Marshall Islands**									
15 - 49 years (rate per capita)	0.007<br>(0.006 - 0.007)	0.007<br>(0.007 - 0.008)	0.007<br>(0.007 - 0.008)	0.001<br>(0.001 - 0.001)	0.001<br>(0.001 - 0.001)	0.001<br>(0.001 - 0.001)	0.189<br>(0.125 - 0.264)	0.203<br>(0.133 - 0.287)	0.208<br>(0.136 - 0.291)
50 - 69 years (rate per capita)	0.060<br>(0.056 - 0.065)	0.060<br>(0.055 - 0.065)	0.061<br>(0.056 - 0.066)	0.006<br>(0.005 - 0.006)	0.006<br>(0.005 - 0.006)	0.006<br>(0.005 - 0.006)	1.652<br>(1.089 - 2.311)	1.640<br>(1.088 - 2.302)	1.654<br>(1.111 - 2.318)
70+ years (rate per capita)	0.148<br>(0.137 - 0.160)	0.148<br>(0.137 - 0.159)	0.148<br>(0.137 - 0.159)	0.008<br>(0.007 - 0.010)	0.008<br>(0.007 - 0.010)	0.008<br>(0.007 - 0.010)	3.911<br>(2.648 - 5.389)	3.889<br>(2.646 - 5.340)	3.883<br>(2.634 - 5.337)
All ages (rate per capita)	0.010<br>(0.009 - 0.011)	0.012<br>(0.011 - 0.012)	0.013<br>(0.013 - 0.014)	0.001<br>(0.001 - 0.001)	0.001<br>(0.001 - 0.001)	0.001<br>(0.001 - 0.001)	0.276<br>(0.185 - 0.380)	0.321<br>(0.215 - 0.445)	0.369<br>(0.247 - 0.509)
Age-standardized (rate per capita)	0.025<br>(0.024 - 0.027)	0.025<br>(0.024 - 0.027)	0.025<br>(0.024 - 0.027)	0.002<br>(0.002 - 0.002)	0.002<br>(0.002 - 0.002)	0.002<br>(0.002 - 0.002)	0.682<br>(0.457 - 0.942)	0.681<br>(0.457 - 0.938)	0.680<br>(0.457 - 0.937)
Total number of cases (thousands)	0<br>(0 - 1)	1<br>(1 - 1)	1<br>(1 - 1)	0<br>(0 - 0)	0<br>(0 - 0)	0<br>(0 - 0)	0<br>(0 - 0)	0<br>(0 - 0)	0<br>(0 - 0)
**Northern Mariana Islands**									
15 - 49 years (rate per capita)	0.007<br>(0.007 - 0.008)	0.008<br>(0.008 - 0.009)	0.008<br>(0.008 - 0.009)	0.001<br>(0.001 - 0.001)	0.001<br>(0.001 - 0.001)	0.001<br>(0.001 - 0.001)	0.214<br>(0.140 - 0.305)	0.236<br>(0.155 - 0.331)	0.243<br>(0.159 - 0.344)
50 - 69 years (rate per capita)	0.056<br>(0.052 - 0.061)	0.054<br>(0.049 - 0.058)	0.052<br>(0.048 - 0.056)	0.005<br>(0.005 - 0.006)	0.005<br>(0.005 - 0.006)	0.005<br>(0.005 - 0.006)	1.556<br>(1.026 - 2.184)	1.495<br>(0.969 - 2.094)	1.448<br>(0.952 - 2.030)
70+ years (rate per capita)	0.149<br>(0.138 - 0.160)	0.146<br>(0.135 - 0.157)	0.145<br>(0.134 - 0.156)	0.008<br>(0.007 - 0.009)	0.008<br>(0.007 - 0.009)	0.008<br>(0.007 - 0.009)	4.003<br>(2.687 - 5.498)	3.921<br>(2.634 - 5.367)	3.887<br>(2.606 - 5.314)
All ages (rate per capita)	0.010<br>(0.010 - 0.011)	0.011<br>(0.010 - 0.011)	0.011<br>(0.010 - 0.012)	0.001<br>(0.001 - 0.001)	0.001<br>(0.001 - 0.001)	0.001<br>(0.001 - 0.001)	0.289<br>(0.192 - 0.401)	0.300<br>(0.199 - 0.416)	0.309<br>(0.206 - 0.428)
Age-standardized (rate per capita)	0.025<br>(0.024 - 0.027)	0.025<br>(0.024 - 0.027)	0.025<br>(0.024 - 0.027)	0.002<br>(0.002 - 0.002)	0.002<br>(0.002 - 0.002)	0.002<br>(0.002 - 0.002)	0.690<br>(0.460 - 0.949)	0.692<br>(0.461 - 0.956)	0.692<br>(0.462 - 0.957)
Total number of cases (thousands)	0<br>(0 - 0)	1<br>(1 - 1)	1<br>(1 - 1)	0<br>(0 - 0)	0<br>(0 - 0)	0<br>(0 - 0)	0<br>(0 - 0)	0<br>(0 - 0)	0<br>(0 - 0)
**Papua New Guinea**									
15 - 49 years (rate per capita)	0.006<br>(0.006 - 0.007)	0.007<br>(0.006 - 0.008)	0.007<br>(0.007 - 0.008)	0.001<br>(0.000 - 0.001)	0.001<br>(0.001 - 0.001)	0.001<br>(0.001 - 0.001)	0.185<br>(0.122 - 0.259)	0.198<br>(0.130 - 0.275)	0.205<br>(0.134 - 0.287)
50 - 69 years (rate per capita)	0.060<br>(0.055 - 0.065)	0.059<br>(0.055 - 0.064)	0.060<br>(0.055 - 0.065)	0.006<br>(0.005 - 0.006)	0.006<br>(0.005 - 0.006)	0.006<br>(0.005 - 0.006)	1.640<br>(1.089 - 2.287)	1.630<br>(1.082 - 2.296)	1.651<br>(1.097 - 2.299)
70+ years (rate per capita)	0.148<br>(0.137 - 0.160)	0.148<br>(0.137 - 0.160)	0.148<br>(0.137 - 0.160)	0.008<br>(0.007 - 0.010)	0.008<br>(0.007 - 0.010)	0.008<br>(0.007 - 0.010)	3.929<br>(2.665 - 5.415)	3.911<br>(2.662 - 5.389)	3.914<br>(2.644 - 5.382)
All ages (rate per capita)	0.010<br>(0.009 - 0.010)	0.011<br>(0.010 - 0.011)	0.012<br>(0.011 - 0.013)	0.001<br>(0.001 - 0.001)	0.001<br>(0.001 - 0.001)	0.001<br>(0.001 - 0.001)	0.266<br>(0.178 - 0.369)	0.294<br>(0.196 - 0.406)	0.328<br>(0.219 - 0.453)
Age-standardized (rate per capita)	0.025<br>(0.024 - 0.027)	0.025<br>(0.024 - 0.027)	0.025<br>(0.024 - 0.027)	0.002<br>(0.002 - 0.002)	0.002<br>(0.002 - 0.002)	0.002<br>(0.002 - 0.002)	0.684<br>(0.461 - 0.944)	0.683<br>(0.459 - 0.943)	0.684<br>(0.459 - 0.946)
Total number of cases (thousands)	40<br>(38 - 43)	65<br>(61 - 69)	91<br>(86 - 97)	3<br>(3 - 4)	5<br>(5 - 6)	8<br>(7 - 8)	1<br>(1 - 2)	2<br>(1 - 2)	3<br>(2 - 3)
**Samoa**									
15 - 49 years (rate per capita)	0.006<br>(0.006 - 0.007)	0.007<br>(0.007 - 0.008)	0.007<br>(0.007 - 0.008)	0.001<br>(0.000 - 0.001)	0.001<br>(0.001 - 0.001)	0.001<br>(0.001 - 0.001)	0.175<br>(0.115 - 0.247)	0.210<br>(0.138 - 0.296)	0.207<br>(0.138 - 0.290)
50 - 69 years (rate per capita)	0.064<br>(0.059 - 0.070)	0.063<br>(0.057 - 0.068)	0.061<br>(0.056 - 0.066)	0.006<br>(0.005 - 0.007)	0.006<br>(0.005 - 0.007)	0.006<br>(0.005 - 0.006)	1.782<br>(1.190 - 2.492)	1.730<br>(1.153 - 2.412)	1.683<br>(1.118 - 2.342)
70+ years (rate per capita)	0.148<br>(0.137 - 0.160)	0.148<br>(0.138 - 0.159)	0.147<br>(0.137 - 0.158)	0.008<br>(0.007 - 0.009)	0.008<br>(0.007 - 0.009)	0.008<br>(0.007 - 0.009)	3.961<br>(2.642 - 5.481)	3.952<br>(2.656 - 5.426)	3.918<br>(2.652 - 5.364)
All ages (rate per capita)	0.012<br>(0.011 - 0.012)	0.014<br>(0.013 - 0.015)	0.016<br>(0.015 - 0.017)	0.001<br>(0.001 - 0.001)	0.001<br>(0.001 - 0.001)	0.001<br>(0.001 - 0.001)	0.326<br>(0.216 - 0.452)	0.391<br>(0.260 - 0.540)	0.438<br>(0.292 - 0.605)
Age-standardized (rate per capita)	0.025<br>(0.024 - 0.027)	0.025<br>(0.024 - 0.027)	0.025<br>(0.024 - 0.027)	0.002<br>(0.002 - 0.002)	0.002<br>(0.002 - 0.002)	0.002<br>(0.002 - 0.002)	0.693<br>(0.462 - 0.960)	0.691<br>(0.458 - 0.955)	0.691<br>(0.462 - 0.954)
Total number of cases (thousands)	2<br>(2 - 2)	3<br>(2 - 3)	3<br>(3 - 3)	0<br>(0 - 0)	0<br>(0 - 0)	0<br>(0 - 0)	0<br>(0 - 0)	0<br>(0 - 0)	0<br>(0 - 0)
**Solomon Islands**									
15 - 49 years (rate per capita)	0.007<br>(0.006 - 0.007)	0.007<br>(0.006 - 0.007)	0.007<br>(0.006 - 0.008)	0.001<br>(0.001 - 0.001)	0.001<br>(0.001 - 0.001)	0.001<br>(0.001 - 0.001)	0.186<br>(0.123 - 0.261)	0.192<br>(0.127 - 0.272)	0.202<br>(0.133 - 0.287)
50 - 69 years (rate per capita)	0.061<br>(0.056 - 0.066)	0.061<br>(0.056 - 0.066)	0.061<br>(0.056 - 0.066)	0.006<br>(0.005 - 0.007)	0.006<br>(0.005 - 0.007)	0.006<br>(0.005 - 0.006)	1.686<br>(1.103 - 2.351)	1.684<br>(1.111 - 2.356)	1.675<br>(1.101 - 2.340)
70+ years (rate per capita)	0.147<br>(0.136 - 0.157)	0.147<br>(0.136 - 0.158)	0.147<br>(0.136 - 0.157)	0.008<br>(0.007 - 0.010)	0.008<br>(0.007 - 0.010)	0.008<br>(0.007 - 0.010)	3.917<br>(2.621 - 5.370)	3.922<br>(2.627 - 5.405)	3.909<br>(2.626 - 5.377)
All ages (rate per capita)	0.010<br>(0.009 - 0.010)	0.011<br>(0.010 - 0.011)	0.012<br>(0.011 - 0.012)	0.001<br>(0.001 - 0.001)	0.001<br>(0.001 - 0.001)	0.001<br>(0.001 - 0.001)	0.269<br>(0.179 - 0.372)	0.299<br>(0.199 - 0.414)	0.324<br>(0.217 - 0.451)
Age-standardized (rate per capita)	0.025<br>(0.024 - 0.027)	0.025<br>(0.024 - 0.027)	0.025<br>(0.024 - 0.027)	0.002<br>(0.002 - 0.002)	0.002<br>(0.002 - 0.002)	0.002<br>(0.002 - 0.002)	0.686<br>(0.455 - 0.950)	0.688<br>(0.458 - 0.950)	0.687<br>(0.459 - 0.954)
Total number of cases (thousands)	3<br>(3 - 3)	5<br>(5 - 5)	7<br>(6 - 7)	0<br>(0 - 0)	0<br>(0 - 0)	1<br>(1 - 1)	0<br>(0 - 0)	0<br>(0 - 0)	0<br>(0 - 0)
**Tonga**									
15 - 49 years (rate per capita)	0.006<br>(0.006 - 0.007)	0.007<br>(0.007 - 0.008)	0.007<br>(0.007 - 0.008)	0.001<br>(0.000 - 0.001)	0.001<br>(0.001 - 0.001)	0.001<br>(0.001 - 0.001)	0.176<br>(0.118 - 0.246)	0.206<br>(0.137 - 0.292)	0.205<br>(0.136 - 0.288)
50 - 69 years (rate per capita)	0.065<br>(0.060 - 0.071)	0.066<br>(0.061 - 0.071)	0.062<br>(0.057 - 0.068)	0.006<br>(0.006 - 0.007)	0.006<br>(0.006 - 0.007)	0.006<br>(0.005 - 0.007)	1.788<br>(1.188 - 2.488)	1.812<br>(1.200 - 2.520)	1.716<br>(1.134 - 2.386)
70+ years (rate per capita)	0.147<br>(0.137 - 0.159)	0.148<br>(0.138 - 0.160)	0.147<br>(0.137 - 0.158)	0.008<br>(0.007 - 0.009)	0.008<br>(0.007 - 0.009)	0.008<br>(0.007 - 0.009)	3.911<br>(2.651 - 5.369)	3.935<br>(2.655 - 5.399)	3.893<br>(2.618 - 5.359)
All ages (rate per capita)	0.013<br>(0.013 - 0.014)	0.016<br>(0.015 - 0.017)	0.016<br>(0.015 - 0.017)	0.001<br>(0.001 - 0.001)	0.001<br>(0.001 - 0.001)	0.001<br>(0.001 - 0.001)	0.364<br>(0.244 - 0.497)	0.434<br>(0.291 - 0.597)	0.449<br>(0.300 - 0.617)
Age-standardized (rate per capita)	0.025<br>(0.024 - 0.027)	0.025<br>(0.024 - 0.027)	0.025<br>(0.024 - 0.027)	0.002<br>(0.002 - 0.002)	0.002<br>(0.002 - 0.002)	0.002<br>(0.002 - 0.002)	0.691<br>(0.465 - 0.943)	0.692<br>(0.463 - 0.952)	0.692<br>(0.463 - 0.952)
Total number of cases (thousands)	1<br>(1 - 1)	2<br>(2 - 2)	2<br>(2 - 2)	0<br>(0 - 0)	0<br>(0 - 0)	0<br>(0 - 0)	0<br>(0 - 0)	0<br>(0 - 0)	0<br>(0 - 0)
**Vanuatu**									
15 - 49 years (rate per capita)	0.007<br>(0.006 - 0.007)	0.007<br>(0.006 - 0.008)	0.007<br>(0.007 - 0.008)	0.001<br>(0.001 - 0.001)	0.001<br>(0.001 - 0.001)	0.001<br>(0.001 - 0.001)	0.196<br>(0.128 - 0.276)	0.199<br>(0.131 - 0.280)	0.208<br>(0.138 - 0.291)
50 - 69 years (rate per capita)	0.062<br>(0.057 - 0.067)	0.061<br>(0.056 - 0.066)	0.060<br>(0.056 - 0.066)	0.006<br>(0.005 - 0.007)	0.006<br>(0.005 - 0.006)	0.006<br>(0.005 - 0.006)	1.713<br>(1.131 - 2.402)	1.682<br>(1.113 - 2.354)	1.668<br>(1.108 - 2.338)
70+ years (rate per capita)	0.147<br>(0.136 - 0.157)	0.147<br>(0.136 - 0.157)	0.148<br>(0.137 - 0.159)	0.008<br>(0.007 - 0.009)	0.008<br>(0.007 - 0.009)	0.008<br>(0.007 - 0.010)	3.917<br>(2.627 - 5.376)	3.916<br>(2.611 - 5.388)	3.949<br>(2.649 - 5.531)
All ages (rate per capita)	0.011<br>(0.011 - 0.012)	0.012<br>(0.011 - 0.012)	0.014<br>(0.013 - 0.015)	0.001<br>(0.001 - 0.001)	0.001<br>(0.001 - 0.001)	0.001<br>(0.001 - 0.001)	0.309<br>(0.208 - 0.429)	0.323<br>(0.216 - 0.448)	0.388<br>(0.262 - 0.535)
Age-standardized (rate per capita)	0.025<br>(0.024 - 0.027)	0.025<br>(0.024 - 0.027)	0.025<br>(0.024 - 0.027)	0.002<br>(0.002 - 0.002)	0.002<br>(0.002 - 0.002)	0.002<br>(0.002 - 0.002)	0.686<br>(0.460 - 0.948)	0.688<br>(0.458 - 0.948)	0.689<br>(0.464 - 0.948)
Total number of cases (thousands)	2<br>(2 - 2)	2<br>(2 - 3)	4<br>(3 - 4)	0<br>(0 - 0)	0<br>(0 - 0)	0<br>(0 - 0)	0<br>(0 - 0)	0<br>(0 - 0)	0<br>(0 - 0)
**North Africa and Middle East**									
15 - 49 years (rate per capita)	0.018<br>(0.017 - 0.021)	0.019<br>(0.017 - 0.022)	0.021<br>(0.019 - 0.024)	0.002<br>(0.001 - 0.002)	0.002<br>(0.002 - 0.002)	0.002<br>(0.002 - 0.002)	0.525<br>(0.345 - 0.736)	0.551<br>(0.363 - 0.778)	0.604<br>(0.398 - 0.850)
50 - 69 years (rate per capita)	0.140<br>(0.126 - 0.155)	0.138<br>(0.124 - 0.153)	0.135<br>(0.122 - 0.150)	0.012<br>(0.010 - 0.014)	0.012<br>(0.010 - 0.014)	0.011<br>(0.010 - 0.013)	3.866<br>(2.511 - 5.430)	3.815<br>(2.478 - 5.316)	3.744<br>(2.427 - 5.258)
70+ years (rate per capita)	0.297<br>(0.272 - 0.326)	0.294<br>(0.271 - 0.323)	0.290<br>(0.265 - 0.318)	0.014<br>(0.011 - 0.017)	0.014<br>(0.011 - 0.017)	0.013<br>(0.011 - 0.016)	7.873<br>(5.204 - 10.873)	7.818<br>(5.210 - 10.701)	7.674<br>(5.112 - 10.605)
All ages (rate per capita)	0.028<br>(0.026 - 0.030)	0.033<br>(0.030 - 0.035)	0.037<br>(0.034 - 0.040)	0.002<br>(0.002 - 0.002)	0.002<br>(0.002 - 0.003)	0.003<br>(0.003 - 0.003)	0.779<br>(0.511 - 1.073)	0.905<br>(0.594 - 1.254)	1.012<br>(0.664 - 1.399)
Age-standardized (rate per capita)	0.056<br>(0.052 - 0.061)	0.056<br>(0.052 - 0.060)	0.055<br>(0.051 - 0.060)	0.004<br>(0.004 - 0.004)	0.004<br>(0.004 - 0.004)	0.004<br>(0.004 - 0.004)	1.537<br>(1.009 - 2.128)	1.533<br>(1.004 - 2.126)	1.516<br>(0.992 - 2.104)
Total number of cases (thousands)	9,465<br>(8,783 - 10,211)	15,209<br>(14,131 - 16,394)	20,715<br>(19,182 - 22,383)	723<br>(669 - 789)	1,152<br>(1,067 - 1,251)	1,563<br>(1,441 - 1,705)	262<br>(172 - 361)	421<br>(276 - 583)	573<br>(376 - 792)
**North Africa and Middle East**									
15 - 49 years (rate per capita)	0.018<br>(0.017 - 0.021)	0.019<br>(0.017 - 0.022)	0.021<br>(0.019 - 0.024)	0.002<br>(0.001 - 0.002)	0.002<br>(0.002 - 0.002)	0.002<br>(0.002 - 0.002)	0.525<br>(0.345 - 0.736)	0.551<br>(0.363 - 0.778)	0.604<br>(0.398 - 0.850)
50 - 69 years (rate per capita)	0.140<br>(0.126 - 0.155)	0.138<br>(0.124 - 0.153)	0.135<br>(0.122 - 0.150)	0.012<br>(0.010 - 0.014)	0.012<br>(0.010 - 0.014)	0.011<br>(0.010 - 0.013)	3.866<br>(2.511 - 5.430)	3.815<br>(2.478 - 5.316)	3.744<br>(2.427 - 5.258)
70+ years (rate per capita)	0.297<br>(0.272 - 0.326)	0.294<br>(0.271 - 0.323)	0.290<br>(0.265 - 0.318)	0.014<br>(0.011 - 0.017)	0.014<br>(0.011 - 0.017)	0.013<br>(0.011 - 0.016)	7.873<br>(5.204 - 10.873)	7.818<br>(5.210 - 10.701)	7.674<br>(5.112 - 10.605)
All ages (rate per capita)	0.028<br>(0.026 - 0.030)	0.033<br>(0.030 - 0.035)	0.037<br>(0.034 - 0.040)	0.002<br>(0.002 - 0.002)	0.002<br>(0.002 - 0.003)	0.003<br>(0.003 - 0.003)	0.779<br>(0.511 - 1.073)	0.905<br>(0.594 - 1.254)	1.012<br>(0.664 - 1.399)
Age-standardized (rate per capita)	0.056<br>(0.052 - 0.061)	0.056<br>(0.052 - 0.060)	0.055<br>(0.051 - 0.060)	0.004<br>(0.004 - 0.004)	0.004<br>(0.004 - 0.004)	0.004<br>(0.004 - 0.004)	1.537<br>(1.009 - 2.128)	1.533<br>(1.004 - 2.126)	1.516<br>(0.992 - 2.104)
Total number of cases (thousands)	9,465<br>(8,783 - 10,211)	15,209<br>(14,131 - 16,394)	20,715<br>(19,182 - 22,383)	723<br>(669 - 789)	1,152<br>(1,067 - 1,251)	1,563<br>(1,441 - 1,705)	262<br>(172 - 361)	421<br>(276 - 583)	573<br>(376 - 792)
**Afghanistan**									

	Prevalence 1990	Prevalence 2005	Prevalence 2015	Incidence 1990	Incidence 2005	Incidence 2015	YLD rate 1990	YLD rate 2005	YLD rate 2015
15 - 49 years (rate per capita)	0.017 (0.014 - 0.021)	0.017 (0.014 - 0.020)	0.017 (0.014 - 0.020)	0.002 (0.001 - 0.002)	0.002 (0.001 - 0.002)	0.002 (0.001 - 0.002)	0.475 (0.308 - 0.685)	0.454 (0.294 - 0.659)	0.465 (0.303 - 0.682)
50 - 69 years (rate per capita)	0.131 (0.109 - 0.155)	0.128 (0.107 - 0.152)	0.131 (0.109 - 0.155)	0.011 (0.008 - 0.014)	0.011 (0.008 - 0.014)	0.011 (0.008 - 0.014)	3.561 (2.244 - 5.151)	3.435 (2.174 - 4.979)	3.434 (2.174 - 4.932)
70+ years (rate per capita)	0.275 (0.233 - 0.321)	0.274 (0.233 - 0.320)	0.274 (0.232 - 0.320)	0.014 (0.010 - 0.020)	0.014 (0.010 - 0.019)	0.014 (0.010 - 0.019)	7.163 (4.604 - 10.132)	7.121 (4.594 - 10.038)	7.075 (4.561 - 9.969)
All ages (rate per capita)	0.019 (0.017 - 0.022)	0.019 (0.017 - 0.022)	0.021 (0.018 - 0.024)	0.002 (0.001 - 0.002)	0.002 (0.001 - 0.002)	0.002 (0.001 - 0.002)	0.513 (0.334 - 0.723)	0.523 (0.341 - 0.738)	0.559 (0.365 - 0.796)
Age-standardized (rate per capita)	0.053 (0.046 - 0.060)	0.053 (0.046 - 0.060)	0.053 (0.046 - 0.060)	0.004 (0.003 - 0.004)	0.004 (0.003 - 0.004)	0.004 (0.003 - 0.004)	1.414 (0.916 - 1.994)	1.404 (0.910 - 1.987)	1.397 (0.904 - 1.961)
Total number of cases (thousands)	233 (204 - 265)	475 (416 - 539)	679 (595 - 769)	19 (17 - 22)	39 (34 - 44)	55 (48 - 63)	6 (4 - 9)	13 (8 - 18)	18 (12 - 26)
**Algeria**									
15 - 49 years (rate per capita)	0.016 (0.013 - 0.020)	0.018 (0.015 - 0.022)	0.021 (0.018 - 0.026)	0.001 (0.001 - 0.002)	0.002 (0.001 - 0.002)	0.002 (0.002 - 0.002)	0.465 (0.297 - 0.679)	0.526 (0.336 - 0.775)	0.610 (0.391 - 0.885)
50 - 69 years (rate per capita)	0.135 (0.112 - 0.160)	0.134 (0.112 - 0.159)	0.134 (0.111 - 0.158)	0.011 (0.009 - 0.014)	0.011 (0.009 - 0.014)	0.011 (0.009 - 0.014)	3.748 (2.342 - 5.495)	3.724 (2.360 - 5.477)	3.711 (2.311 - 5.472)
70+ years (rate per capita)	0.275 (0.234 - 0.322)	0.275 (0.235 - 0.322)	0.273 (0.234 - 0.319)	0.014 (0.010 - 0.018)	0.013 (0.010 - 0.018)	0.013 (0.009 - 0.017)	7.342 (4.726 - 10.292)	7.345 (4.739 - 10.299)	7.266 (4.663 - 10.236)
All ages (rate per capita)	0.024 (0.021 - 0.027)	0.033 (0.029 - 0.037)	0.040 (0.035 - 0.045)	0.002 (0.002 - 0.002)	0.003 (0.002 - 0.003)	0.003 (0.003 - 0.003)	0.667 (0.430 - 0.953)	0.913 (0.589 - 1.295)	1.108 (0.711 - 1.584)
Age-standardized (rate per capita)	0.053 (0.047 - 0.061)	0.053 (0.047 - 0.061)	0.053 (0.047 - 0.061)	0.004 (0.003 - 0.004)	0.004 (0.003 - 0.004)	0.004 (0.003 - 0.004)	1.470 (0.937 - 2.073)	1.473 (0.941 - 2.092)	1.473 (0.936 - 2.096)
Total number of cases (thousands)	623 (548 - 706)	1,097 (967 - 1,243)	1,585 (1,393 - 1,794)	49 (43 - 56)	84 (74 - 96)	118 (103 - 135)	17 (11 - 25)	30 (20 - 43)	44 (28 - 63)
**Bahrain**									
15 - 49 years (rate per capita)	0.020 (0.016 - 0.024)	0.023 (0.019 - 0.027)	0.023 (0.020 - 0.028)	0.002 (0.001 - 0.002)	0.002 (0.002 - 0.002)	0.002 (0.002 - 0.002)	0.564 (0.364 - 0.818)	0.646 (0.413 - 0.939)	0.664 (0.422 - 0.963)
50 - 69 years (rate per capita)	0.130 (0.109 - 0.154)	0.123 (0.103 - 0.146)	0.119 (0.100 - 0.140)	0.011 (0.009 - 0.014)	0.010 (0.008 - 0.013)	0.010 (0.008 - 0.013)	3.587 (2.280 - 5.210)	3.395 (2.173 - 4.942)	3.289 (2.083 - 4.770)
70+ years (rate per capita)	0.274 (0.237 - 0.319)	0.277 (0.239 - 0.323)	0.276 (0.238 - 0.323)	0.013 (0.010 - 0.017)	0.013 (0.010 - 0.018)	0.013 (0.010 - 0.018)	7.235 (4.701 - 10.248)	7.303 (4.754 - 10.324)	7.283 (4.709 - 10.305)
All ages (rate per capita)	0.024 (0.021 - 0.027)	0.028 (0.024 - 0.032)	0.033 (0.029 - 0.037)	0.002 (0.002 - 0.002)	0.002 (0.002 - 0.003)	0.003 (0.002 - 0.003)	0.667 (0.433 - 0.957)	0.776 (0.500 - 1.114)	0.917 (0.591 - 1.307)
Age-standardized (rate per capita)	0.054 (0.047 - 0.061)	0.054 (0.047 - 0.061)	0.054 (0.047 - 0.061)	0.004 (0.003 - 0.004)	0.004 (0.003 - 0.004)	0.004 (0.003 - 0.004)	1.466 (0.942 - 2.070)	1.460 (0.944 - 2.058)	1.464 (0.942 - 2.063)
Total number of cases (thousands)	12 (10 - 14)	24 (21 - 27)	45 (39 - 51)	1 (1 - 1)	2 (2 - 2)	4 (3 - 4)	0 (0 - 0)	1 (0 - 1)	1 (1 - 2)
**Egypt**									
15 - 49 years (rate per capita)	0.019 (0.016 - 0.023)	0.019 (0.015 - 0.022)	0.020 (0.017 - 0.024)	0.002 (0.001 - 0.002)	0.002 (0.001 - 0.002)	0.002 (0.001 - 0.002)	0.539 (0.344 - 0.786)	0.530 (0.338 - 0.775)	0.574 (0.369 - 0.837)
50 - 69 years (rate per capita)	0.135 (0.114 - 0.160)	0.132 (0.110 - 0.155)	0.133 (0.112 - 0.157)	0.011 (0.009 - 0.014)	0.011 (0.009 - 0.014)	0.011 (0.009 - 0.014)	3.754 (2.366 - 5.467)	3.642 (2.298 - 5.284)	3.682 (2.317 - 5.347)
70+ years (rate per capita)	0.271 (0.232 - 0.320)	0.272 (0.232 - 0.320)	0.271 (0.232 - 0.318)	0.013 (0.010 - 0.018)	0.013 (0.010 - 0.018)	0.013 (0.010 - 0.017)	7.234 (4.638 - 10.187)	7.216 (4.630 - 10.152)	7.161 (4.614 - 10.073)
All ages (rate per capita)	0.031 (0.027 - 0.035)	0.033 (0.029 - 0.038)	0.035 (0.031 - 0.040)	0.002 (0.002 - 0.003)	0.003 (0.002 - 0.003)	0.003 (0.002 - 0.003)	0.852 (0.549 - 1.205)	0.918 (0.589 - 1.307)	0.979 (0.632 - 1.396)
Age-standardized (rate per capita)	0.053 (0.046 - 0.060)	0.053 (0.046 - 0.060)	0.053 (0.046 - 0.060)	0.004 (0.003 - 0.004)	0.004 (0.003 - 0.004)	0.004 (0.003 - 0.004)	1.453 (0.934 - 2.047)	1.451 (0.932 - 2.049)	1.448 (0.931 - 2.035)
Total number of cases (thousands)	1,731 (1,515 - 1,968)	2,484 (2,181 - 2,823)	3,229 (2,829 - 3,663)	133 (115 - 153)	189 (165 - 219)	247 (216 - 286)	48 (31 - 68)	69 (44 - 98)	89 (58 - 127)
**Iran**									
15 - 49 years (rate per capita)	0.011 (0.011 - 0.012)	0.012 (0.011 - 0.012)	0.014 (0.013 - 0.015)	0.001 (0.001 - 0.001)	0.001 (0.001 - 0.001)	0.001 (0.001 - 0.001)	0.325 (0.215 - 0.456)	0.336 (0.223 - 0.474)	0.407 (0.268 - 0.569)
50 - 69 years (rate per capita)	0.088 (0.083 - 0.093)	0.083 (0.079 - 0.088)	0.082 (0.078 - 0.087)	0.007 (0.007 - 0.008)	0.007 (0.006 - 0.008)	0.007 (0.006 - 0.008)	2.440 (1.629 - 3.384)	2.313 (1.530 - 3.206)	2.280 (1.519 - 3.152)
70+ years (rate per capita)	0.184 (0.173 - 0.194)	0.181 (0.171 - 0.193)	0.180 (0.170 - 0.190)	0.009 (0.008 - 0.011)	0.009 (0.008 - 0.010)	0.009 (0.008 - 0.010)	4.888 (3.294 - 6.689)	4.824 (3.252 - 6.624)	4.770 (3.227 - 6.537)
All ages (rate per capita)	0.016 (0.015 - 0.017)	0.021 (0.020 - 0.022)	0.026 (0.025 - 0.027)	0.001 (0.001 - 0.001)	0.002 (0.002 - 0.002)	0.002 (0.002 - 0.002)	0.445 (0.299 - 0.615)	0.587 (0.390 - 0.810)	0.709 (0.474 - 0.977)
Age-standardized (rate per capita)	0.035 (0.033 - 0.036)	0.035 (0.033 - 0.036)	0.034 (0.033 - 0.036)	0.002 (0.002 - 0.003)	0.002 (0.002 - 0.003)	0.002 (0.002 - 0.003)	0.958 (0.646 - 1.320)	0.948 (0.636 - 1.306)	0.944 (0.635 - 1.297)
Total number of cases (thousands)	898 (861 - 936)	1,495 (1,437 - 1,558)	2,020 (1,938 - 2,106)	71 (67 - 74)	114 (108 - 119)	152 (145 - 159)	25 (17 - 34)	41 (28 - 57)	56 (37 - 77)
**Iraq**									
15 - 49 years (rate per capita)	0.016 (0.013 - 0.019)	0.017 (0.014 - 0.020)	0.019 (0.015 - 0.022)	0.001 (0.001 - 0.002)	0.002 (0.001 - 0.002)	0.002 (0.001 - 0.002)	0.448 (0.292 - 0.658)	0.470 (0.301 - 0.691)	0.523 (0.337 - 0.766)
50 - 69 years (rate per capita)	0.134 (0.112 - 0.159)	0.131 (0.110 - 0.155)	0.131 (0.110 - 0.156)	0.011 (0.009 - 0.014)	0.011 (0.009 - 0.014)	0.011 (0.009 - 0.014)	3.639 (2.311 - 5.292)	3.553 (2.248 - 5.148)	3.538 (2.236 - 5.133)
70+ years (rate per capita)	0.273 (0.235 - 0.318)	0.272 (0.233 - 0.317)	0.272 (0.234 - 0.318)	0.013 (0.010 - 0.018)	0.013 (0.010 - 0.017)	0.013 (0.010 - 0.018)	7.145 (4.608 - 10.193)	7.084 (4.562 - 9.979)	7.097 (4.554 - 10.049)
All ages (rate per capita)	0.024 (0.021 - 0.027)	0.024 (0.021 - 0.027)	0.025 (0.022 - 0.028)	0.002 (0.002 - 0.002)	0.002 (0.002 - 0.002)	0.002 (0.002 - 0.002)	0.657 (0.426 - 0.928)	0.648 (0.423 - 0.914)	0.670 (0.432 - 0.952)
Age-standardized (rate per capita)	0.053 (0.046 - 0.060)	0.053 (0.046 - 0.060)	0.053 (0.046 - 0.060)	0.004 (0.003 - 0.004)	0.004 (0.003 - 0.004)	0.004 (0.003 - 0.004)	1.428 (0.921 - 2.010)	1.423 (0.917 - 2.015)	1.421 (0.910 - 2.026)
Total number of cases (thousands)	422 (370 - 478)	644 (565 - 728)	894 (785 - 1,009)	32 (28 - 37)	50 (44 - 57)	70 (61 - 80)	11 (7 - 16)	18 (11 - 25)	24 (16 - 35)
**Jordan**									
15 - 49 years (rate per capita)	0.015 (0.013 - 0.018)	0.017 (0.015 - 0.021)	0.020 (0.017 - 0.024)	0.001 (0.001 - 0.002)	0.002 (0.001 - 0.002)	0.002 (0.001 - 0.002)	0.432 (0.280 - 0.634)	0.501 (0.321 - 0.735)	0.572 (0.369 - 0.830)
50 - 69 years (rate per capita)	0.133 (0.111 - 0.160)	0.139 (0.116 - 0.167)	0.129 (0.108 - 0.155)	0.011 (0.009 - 0.014)	0.012 (0.009 - 0.015)	0.011 (0.008 - 0.014)	3.670 (2.334 - 5.349)	3.831 (2.438 - 5.563)	3.567 (2.252 - 5.215)
70+ years (rate per capita)	0.275 (0.236 - 0.320)	0.276 (0.236 - 0.324)	0.276 (0.236 - 0.323)	0.013 (0.009 - 0.017)	0.014 (0.010 - 0.019)	0.014 (0.010 - 0.018)	7.257 (4.697 - 10.252)	7.301 (4.688 - 10.386)	7.298 (4.689 - 10.366)
All ages (rate per capita)	0.023 (0.020 - 0.026)	0.025 (0.022 - 0.029)	0.029 (0.025 - 0.033)	0.002 (0.002 - 0.002)	0.002 (0.002 - 0.002)	0.002 (0.002 - 0.003)	0.624 (0.401 - 0.885)	0.701 (0.454 - 0.996)	0.803 (0.521 - 1.141)
Age-standardized (rate per capita)	0.053 (0.047 - 0.061)	0.053 (0.047 - 0.061)	0.053 (0.047 - 0.061)	0.004 (0.003 - 0.004)	0.004 (0.003 - 0.004)	0.004 (0.003 - 0.004)	1.461 (0.939 - 2.068)	1.461 (0.944 - 2.072)	1.462 (0.947 - 2.064)
Total number of cases (thousands)	75 (66 - 85)	135 (119 - 154)	219 (193 - 248)	6 (5 - 7)	11 (9 - 12)	17 (15 - 19)	2 (1 - 3)	4 (2 - 5)	6 (4 - 9)
**Kuwait**									
15 - 49 years (rate per capita)	0.021 (0.018 - 0.025)	0.022 (0.019 - 0.027)	0.024 (0.020 - 0.029)	0.002 (0.001 - 0.002)	0.002 (0.002 - 0.002)	0.002 (0.002 - 0.003)	0.602 (0.390 - 0.871)	0.644 (0.419 - 0.941)	0.697 (0.451 - 1.017)
50 - 69 years (rate per capita)	0.124 (0.105 - 0.145)	0.127 (0.108 - 0.149)	0.122 (0.103 - 0.143)	0.010 (0.008 - 0.013)	0.011 (0.008 - 0.013)	0.010 (0.008 - 0.013)	3.451 (2.198 - 4.978)	3.531 (2.239 - 5.130)	3.398 (2.155 - 4.914)
70+ years (rate per capita)	0.274 (0.236 - 0.320)	0.282 (0.243 - 0.331)	0.281 (0.243 - 0.329)	0.013 (0.010 - 0.017)	0.015 (0.011 - 0.020)	0.014 (0.010 - 0.018)	7.303 (4.703 - 10.385)	7.525 (4.775 - 10.624)	7.479 (4.808 - 10.561)
All ages (rate per capita)	0.023 (0.020 - 0.027)	0.028 (0.024 - 0.032)	0.031 (0.027 - 0.035)	0.002 (0.002 - 0.002)	0.002 (0.002 - 0.003)	0.002 (0.002 - 0.003)	0.656 (0.423 - 0.932)	0.783 (0.503 - 1.112)	0.872 (0.564 - 1.252)
Age-standardized (rate per capita)	0.054 (0.048 - 0.061)	0.054 (0.048 - 0.062)	0.054 (0.048 - 0.061)	0.004 (0.003 - 0.004)	0.004 (0.003 - 0.004)	0.004 (0.003 - 0.004)	1.493 (0.957 - 2.101)	1.491 (0.960 - 2.097)	1.489 (0.958 - 2.104)
Total number of cases (thousands)	48 (42 - 55)	63 (56 - 72)	121 (105 - 138)	4 (3 - 5)	5 (5 - 6)	10 (8 - 11)	1 (1 - 2)	2 (1 - 3)	3 (2 - 5)
**Lebanon**									
15 - 49 years (rate per capita)	0.019 (0.016 - 0.023)	0.021 (0.017 - 0.024)	0.020 (0.017 - 0.024)	0.002 (0.001 - 0.002)	0.002 (0.001 - 0.002)	0.002 (0.001 - 0.002)	0.520 (0.335 - 0.757)	0.571 (0.370 - 0.835)	0.574 (0.368 - 0.840)
50 - 69 years (rate per capita)	0.138 (0.115 - 0.164)	0.140 (0.117 - 0.166)	0.135 (0.113 - 0.159)	0.012 (0.009 - 0.015)	0.012 (0.009 - 0.015)	0.011 (0.009 - 0.014)	3.737 (2.367 - 5.444)	3.784 (2.406 - 5.512)	3.638 (2.308 - 5.291)
70+ years (rate per capita)	0.274 (0.235 - 0.322)	0.274 (0.235 - 0.322)	0.274 (0.235 - 0.322)	0.013 (0.010 - 0.017)	0.013 (0.010 - 0.018)	0.013 (0.010 - 0.017)	7.134 (4.625 - 10.070)	7.176 (4.626 - 10.130)	7.154 (4.581 - 10.115)
All ages (rate per capita)	0.037 (0.032 - 0.042)	0.044 (0.038 - 0.050)	0.048 (0.042 - 0.054)	0.003 (0.002 - 0.003)	0.003 (0.003 - 0.004)	0.003 (0.003 - 0.004)	0.997 (0.646 - 1.404)	1.179 (0.767 - 1.666)	1.287 (0.836 - 1.831)
Age-standardized (rate per capita)	0.053 (0.047 - 0.061)	0.053 (0.047 - 0.061)	0.053 (0.047 - 0.061)	0.004 (0.003 - 0.004)	0.004 (0.003 - 0.004)	0.004 (0.003 - 0.004)	1.431 (0.927 - 2.034)	1.436 (0.933 - 2.037)	1.439 (0.935 - 2.047)
Total number of cases (thousands)	101 (88 - 115)	173 (151 - 196)	275 (242 - 312)	8 (7 - 9)	13 (11 - 15)	20 (18 - 23)	3 (2 - 4)	5 (3 - 7)	7 (5 - 11)
**Libya**									
15 - 49 years (rate per capita)	0.017 (0.014 - 0.020)	0.019 (0.015 - 0.022)	0.023 (0.019 - 0.027)	0.002 (0.001 - 0.002)	0.002 (0.001 - 0.002)	0.002 (0.002 - 0.002)	0.484 (0.315 - 0.710)	0.530 (0.339 - 0.778)	0.655 (0.418 - 0.980)
50 - 69 years (rate per capita)	0.134 (0.112 - 0.159)	0.134 (0.112 - 0.159)	0.130 (0.108 - 0.154)	0.011 (0.009 - 0.014)	0.011 (0.009 - 0.014)	0.011 (0.008 - 0.014)	3.738 (2.343 - 5.442)	3.704 (2.309 - 5.438)	3.594 (2.250 - 5.218)
70+ years (rate per capita)	0.277 (0.235 - 0.326)	0.277 (0.235 - 0.325)	0.275 (0.233 - 0.323)	0.014 (0.010 - 0.019)	0.014 (0.010 - 0.018)	0.013 (0.010 - 0.018)	7.414 (4.754 - 10.521)	7.369 (4.734 - 10.449)	7.298 (4.654 - 10.313)
All ages (rate per capita)	0.024 (0.021 - 0.028)	0.030 (0.026 - 0.034)	0.036 (0.031 - 0.041)	0.002 (0.002 - 0.002)	0.002 (0.002 - 0.003)	0.003 (0.002 - 0.003)	0.678 (0.433 - 0.961)	0.835 (0.533 - 1.188)	0.997 (0.634 - 1.417)
Age-standardized (rate per capita)	0.054 (0.047 - 0.061)	0.053 (0.047 - 0.061)	0.053 (0.047 - 0.061)	0.004 (0.003 - 0.004)	0.004 (0.003 - 0.004)	0.004 (0.003 - 0.004)	1.476 (0.945 - 2.097)	1.471 (0.940 - 2.102)	1.467 (0.938 - 2.096)
Total number of cases (thousands)	107 (93 - 121)	174 (153 - 198)	226 (198 - 258)	8 (7 - 10)	14 (12 - 16)	17 (15 - 20)	3 (2 - 4)	5 (3 - 7)	6 (4 - 9)
**Morocco**									
15 - 49 years (rate per capita)	0.018 (0.017 - 0.019)	0.022 (0.020 - 0.023)	0.023 (0.021 - 0.024)	0.002 (0.001 - 0.002)	0.002 (0.002 - 0.002)	0.002 (0.002 - 0.002)	0.507 (0.334 - 0.709)	0.624 (0.411 - 0.873)	0.648 (0.431 - 0.905)
50 - 69 years (rate per capita)	0.141 (0.131 - 0.151)	0.139 (0.129 - 0.149)	0.133 (0.124 - 0.143)	0.012 (0.011 - 0.013)	0.012 (0.011 - 0.013)	0.011 (0.010 - 0.013)	3.872 (2.578 - 5.415)	3.815 (2.528 - 5.296)	3.676 (2.448 - 5.119)
70+ years (rate per capita)	0.288 (0.268 - 0.310)	0.291 (0.270 - 0.313)	0.287 (0.266 - 0.308)	0.014 (0.012 - 0.017)	0.014 (0.012 - 0.016)	0.013 (0.011 - 0.015)	7.626 (5.154 - 10.570)	7.679 (5.171 - 10.638)	7.545 (5.100 - 10.411)
All ages (rate per capita)	0.029 (0.028 - 0.031)	0.038 (0.036 - 0.040)	0.045 (0.042 - 0.047)	0.002 (0.002 - 0.002)	0.003 (0.003 - 0.003)	0.003 (0.003 - 0.004)	0.802 (0.536 - 1.107)	1.056 (0.707 - 1.454)	1.225 (0.818 - 1.692)

	Prevalence 1990	Prevalence 2005	Prevalence 2015	Incidence 1990	Incidence 2005	Incidence 2015	YLD rate 1990	YLD rate 2005	YLD rate 2015
**Age-standardized (rate per capita)**	0.055 (0.053 - 0.059)	0.056 (0.053 - 0.059)	0.056 (0.053 - 0.059)	0.004 (0.004 - 0.004)	0.004 (0.004 - 0.004)	0.004 (0.004 - 0.004)	1.516 (1.015 - 2.097)	1.526 (1.024 - 2.097)	1.516 (1.015 - 2.108)
**Total number of cases (thousands)**	727 (690 - 768)	1,168 (1,111 - 1,232)	1,531 (1,454 - 1,616)	57 (54 - 61)	87 (82 - 92)	114 (107 - 121)	20 (13 - 28)	32 (22 - 44)	42 (28 - 58)
**Palestine**									
**15 - 49 years (rate per capita)**	0.016 (0.014 - 0.020)	0.017 (0.014 - 0.020)	0.017 (0.014 - 0.021)	0.002 (0.001 - 0.002)	0.002 (0.001 - 0.002)	0.002 (0.001 - 0.002)	0.462 (0.299 - 0.672)	0.477 (0.308 - 0.701)	0.495 (0.315 - 0.722)
**50 - 69 years (rate per capita)**	0.129 (0.108 - 0.152)	0.133 (0.112 - 0.157)	0.130 (0.110 - 0.154)	0.011 (0.008 - 0.014)	0.011 (0.009 - 0.014)	0.011 (0.008 - 0.014)	3.555 (2.236 - 5.137)	3.658 (2.309 - 5.328)	3.582 (2.272 - 5.169)
**70+ years (rate per capita)**	0.273 (0.234 - 0.320)	0.274 (0.234 - 0.321)	0.275 (0.235 - 0.322)	0.014 (0.010 - 0.018)	0.014 (0.010 - 0.018)	0.014 (0.010 - 0.018)	7.280 (4.670 - 10.346)	7.297 (4.670 - 10.381)	7.298 (4.668 - 10.328)
**All ages (rate per capita)**	0.019 (0.016 - 0.021)	0.021 (0.018 - 0.023)	0.024 (0.021 - 0.027)	0.002 (0.001 - 0.002)	0.002 (0.001 - 0.002)	0.002 (0.002 - 0.002)	0.516 (0.333 - 0.736)	0.571 (0.364 - 0.812)	0.653 (0.417 - 0.929)
**Age-standardized (rate per capita)**	0.053 (0.047 - 0.060)	0.053 (0.047 - 0.060)	0.053 (0.047 - 0.060)	0.004 (0.003 - 0.004)	0.004 (0.003 - 0.004)	0.004 (0.003 - 0.004)	1.453 (0.927 - 2.060)	1.454 (0.927 - 2.058)	1.454 (0.932 - 2.069)
**Total number of cases (thousands)**	39 (34 - 45)	74 (65 - 84)	110 (96 - 124)	3 (3 - 4)	6 (5 - 7)	9 (8 - 10)	1 (1 - 2)	2 (1 - 3)	3 (2 - 4)
**Oman**									
**15 - 49 years (rate per capita)**	0.020 (0.017 - 0.024)	0.019 (0.016 - 0.023)	0.021 (0.018 - 0.025)	0.002 (0.001 - 0.002)	0.002 (0.001 - 0.002)	0.002 (0.002 - 0.002)	0.573 (0.366 - 0.840)	0.539 (0.346 - 0.788)	0.599 (0.386 - 0.880)
**50 - 69 years (rate per capita)**	0.128 (0.107 - 0.152)	0.129 (0.109 - 0.153)	0.129 (0.109 - 0.152)	0.011 (0.008 - 0.013)	0.011 (0.009 - 0.013)	0.011 (0.009 - 0.014)	3.530 (2.240 - 5.146)	3.566 (2.264 - 5.172)	3.558 (2.274 - 5.184)
**70+ years (rate per capita)**	0.275 (0.235 - 0.323)	0.274 (0.235 - 0.320)	0.276 (0.238 - 0.322)	0.014 (0.010 - 0.019)	0.013 (0.010 - 0.017)	0.014 (0.010 - 0.018)	7.278 (4.688 - 10.257)	7.215 (4.635 - 10.182)	7.253 (4.677 - 10.190)
**All ages (rate per capita)**	0.021 (0.019 - 0.024)	0.025 (0.022 - 0.028)	0.030 (0.026 - 0.034)	0.002 (0.001 - 0.002)	0.002 (0.002 - 0.002)	0.002 (0.002 - 0.003)	0.596 (0.382 - 0.850)	0.686 (0.441 - 0.978)	0.833 (0.540 - 1.194)
**Age-standardized (rate per capita)**	0.053 (0.047 - 0.061)	0.054 (0.047 - 0.061)	0.054 (0.048 - 0.061)	0.004 (0.003 - 0.004)	0.004 (0.003 - 0.004)	0.004 (0.003 - 0.004)	1.458 (0.935 - 2.062)	1.466 (0.946 - 2.080)	1.468 (0.951 - 2.062)
**Total number of cases (thousands)**	39 (34 - 44)	62 (54 - 70)	133 (118 - 151)	3 (3 - 4)	5 (4 - 6)	11 (10 - 12)	1 (1 - 2)	2 (1 - 2)	4 (2 - 5)
**Qatar**									
**15 - 49 years (rate per capita)**	0.023 (0.020 - 0.028)	0.025 (0.021 - 0.030)	0.023 (0.019 - 0.027)	0.002 (0.002 - 0.002)	0.002 (0.002 - 0.003)	0.002 (0.002 - 0.002)	0.665 (0.428 - 0.964)	0.715 (0.462 - 1.049)	0.647 (0.419 - 0.939)
**50 - 69 years (rate per capita)**	0.119 (0.102 - 0.139)	0.115 (0.098 - 0.133)	0.117 (0.099 - 0.136)	0.010 (0.008 - 0.013)	0.010 (0.008 - 0.012)	0.010 (0.008 - 0.013)	3.290 (2.083 - 4.758)	3.174 (2.012 - 4.586)	3.234 (2.058 - 4.656)
**70+ years (rate per capita)**	0.274 (0.237 - 0.321)	0.277 (0.239 - 0.324)	0.287 (0.246 - 0.336)	0.013 (0.010 - 0.017)	0.014 (0.010 - 0.018)	0.014 (0.011 - 0.019)	7.231 (4.683 - 10.135)	7.303 (4.710 - 10.218)	7.564 (4.907 - 10.598)
**All ages (rate per capita)**	0.025 (0.022 - 0.028)	0.030 (0.026 - 0.034)	0.028 (0.025 - 0.032)	0.002 (0.002 - 0.002)	0.002 (0.002 - 0.003)	0.002 (0.002 - 0.003)	0.698 (0.453 - 1.000)	0.843 (0.542 - 1.207)	0.796 (0.516 - 1.135)
**Age-standardized (rate per capita)**	0.054 (0.048 - 0.061)	0.054 (0.048 - 0.061)	0.054 (0.048 - 0.061)	0.004 (0.003 - 0.004)	0.004 (0.003 - 0.004)	0.004 (0.003 - 0.004)	1.478 (0.964 - 2.091)	1.479 (0.961 - 2.078)	1.483 (0.963 - 2.094)
**Total number of cases (thousands)**	12 (10 - 14)	25 (22 - 28)	63 (55 - 72)	1 (1 - 1)	2 (2 - 2)	5 (5 - 6)	0 (0 - 0)	1 (0 - 1)	2 (1 - 3)
**Saudi Arabia**									
**15 - 49 years (rate per capita)**	0.018 (0.016 - 0.020)	0.021 (0.019 - 0.023)	0.024 (0.022 - 0.026)	0.002 (0.001 - 0.002)	0.002 (0.002 - 0.002)	0.002 (0.002 - 0.002)	0.509 (0.334 - 0.715)	0.590 (0.389 - 0.831)	0.686 (0.447 - 0.960)
**50 - 69 years (rate per capita)**	0.141 (0.127 - 0.153)	0.129 (0.118 - 0.142)	0.127 (0.115 - 0.138)	0.012 (0.010 - 0.013)	0.011 (0.009 - 0.012)	0.011 (0.009 - 0.012)	3.907 (2.573 - 5.493)	3.608 (2.369 - 5.061)	3.539 (2.312 - 4.974)
**70+ years (rate per capita)**	0.277 (0.252 - 0.303)	0.277 (0.253 - 0.303)	0.276 (0.251 - 0.301)	0.014 (0.011 - 0.017)	0.013 (0.011 - 0.016)	0.013 (0.011 - 0.016)	7.414 (4.944 - 10.395)	7.411 (4.945 - 10.388)	7.368 (4.929 - 10.342)
**All ages (rate per capita)**	0.022 (0.021 - 0.024)	0.026 (0.025 - 0.028)	0.033 (0.031 - 0.036)	0.002 (0.002 - 0.002)	0.002 (0.002 - 0.002)	0.003 (0.002 - 0.003)	0.625 (0.415 - 0.867)	0.737 (0.489 - 1.024)	0.929 (0.614 - 1.291)
**Age-standardized (rate per capita)**	0.054 (0.050 - 0.058)	0.054 (0.050 - 0.058)	0.054 (0.051 - 0.058)	0.004 (0.004 - 0.004)	0.004 (0.004 - 0.004)	0.004 (0.004 - 0.004)	1.493 (0.990 - 2.062)	1.495 (0.991 - 2.070)	1.498 (0.992 - 2.072)
**Total number of cases (thousands)**	365 (341 - 391)	651 (608 - 700)	1,038 (967 - 1,120)	29 (27 - 32)	51 (47 - 55)	82 (76 - 89)	10 (7 - 14)	18 (12 - 25)	29 (19 - 41)
**Sudan**									
**15 - 49 years (rate per capita)**	0.017 (0.014 - 0.020)	0.017 (0.015 - 0.021)	0.018 (0.015 - 0.022)	0.002 (0.001 - 0.002)	0.002 (0.001 - 0.002)	0.002 (0.001 - 0.002)	0.480 (0.311 - 0.708)	0.495 (0.321 - 0.728)	0.515 (0.335 - 0.756)
**50 - 69 years (rate per capita)**	0.132 (0.111 - 0.155)	0.131 (0.110 - 0.155)	0.131 (0.110 - 0.155)	0.011 (0.009 - 0.014)	0.011 (0.009 - 0.014)	0.011 (0.009 - 0.014)	3.632 (2.311 - 5.280)	3.604 (2.280 - 5.246)	3.605 (2.287 - 5.226)
**70+ years (rate per capita)**	0.273 (0.233 - 0.320)	0.273 (0.233 - 0.320)	0.273 (0.233 - 0.319)	0.014 (0.010 - 0.018)	0.013 (0.010 - 0.018)	0.013 (0.010 - 0.018)	7.222 (4.632 - 10.189)	7.190 (4.640 - 10.158)	7.180 (4.635 - 10.113)
**All ages (rate per capita)**	0.022 (0.020 - 0.025)	0.023 (0.020 - 0.026)	0.025 (0.022 - 0.029)	0.002 (0.002 - 0.002)	0.002 (0.002 - 0.002)	0.002 (0.002 - 0.002)	0.618 (0.401 - 0.874)	0.644 (0.418 - 0.911)	0.701 (0.452 - 0.995)
**Age-standardized (rate per capita)**	0.053 (0.046 - 0.060)	0.053 (0.046 - 0.060)	0.053 (0.046 - 0.060)	0.004 (0.003 - 0.004)	0.004 (0.003 - 0.004)	0.004 (0.003 - 0.004)	1.444 (0.931 - 2.047)	1.440 (0.932 - 2.025)	1.442 (0.927 - 2.038)
**Total number of cases (thousands)**	448 (392 - 507)	749 (657 - 847)	1,026 (898 - 1,161)	35 (31 - 41)	59 (52 - 68)	80 (70 - 92)	12 (8 - 17)	21 (13 - 29)	28 (18 - 40)
**Syria**									
**15 - 49 years (rate per capita)**	0.016 (0.013 - 0.019)	0.017 (0.014 - 0.021)	0.019 (0.016 - 0.023)	0.001 (0.001 - 0.002)	0.002 (0.001 - 0.002)	0.002 (0.001 - 0.002)	0.446 (0.286 - 0.656)	0.493 (0.315 - 0.725)	0.529 (0.338 - 0.770)
**50 - 69 years (rate per capita)**	0.138 (0.115 - 0.164)	0.132 (0.110 - 0.156)	0.131 (0.109 - 0.156)	0.012 (0.009 - 0.015)	0.011 (0.009 - 0.014)	0.011 (0.009 - 0.014)	3.835 (2.436 - 5.613)	3.674 (2.340 - 5.354)	3.638 (2.288 - 5.310)
**70+ years (rate per capita)**	0.276 (0.237 - 0.324)	0.277 (0.239 - 0.325)	0.273 (0.235 - 0.320)	0.014 (0.010 - 0.019)	0.014 (0.010 - 0.019)	0.013 (0.010 - 0.018)	7.398 (4.769 - 10.533)	7.444 (4.767 - 10.567)	7.293 (4.674 - 10.341)
**All ages (rate per capita)**	0.022 (0.019 - 0.025)	0.025 (0.022 - 0.028)	0.030 (0.026 - 0.034)	0.002 (0.001 - 0.002)	0.002 (0.002 - 0.002)	0.002 (0.002 - 0.003)	0.601 (0.386 - 0.859)	0.697 (0.449 - 0.997)	0.832 (0.535 - 1.183)
**Age-standardized (rate per capita)**	0.053 (0.047 - 0.061)	0.053 (0.047 - 0.061)	0.053 (0.047 - 0.061)	0.004 (0.003 - 0.004)	0.004 (0.003 - 0.004)	0.004 (0.003 - 0.004)	1.476 (0.951 - 2.093)	1.480 (0.946 - 2.111)	1.468 (0.943 - 2.087)
**Total number of cases (thousands)**	268 (235 - 306)	453 (399 - 514)	558 (491 - 636)	21 (19 - 24)	35 (31 - 41)	43 (37 - 49)	7 (5 - 11)	13 (8 - 18)	15 (10 - 22)
**Tunisia**									
**15 - 49 years (rate per capita)**	0.018 (0.016 - 0.019)	0.020 (0.019 - 0.022)	0.023 (0.021 - 0.024)	0.002 (0.001 - 0.002)	0.002 (0.002 - 0.002)	0.002 (0.002 - 0.002)	0.502 (0.332 - 0.704)	0.585 (0.387 - 0.817)	0.647 (0.426 - 0.910)
**50 - 69 years (rate per capita)**	0.132 (0.124 - 0.142)	0.131 (0.123 - 0.139)	0.128 (0.119 - 0.137)	0.011 (0.010 - 0.012)	0.011 (0.010 - 0.012)	0.011 (0.010 - 0.012)	3.675 (2.437 - 5.087)	3.635 (2.408 - 5.050)	3.551 (2.369 - 4.922)
**70+ years (rate per capita)**	0.275 (0.256 - 0.293)	0.273 (0.255 - 0.291)	0.267 (0.249 - 0.285)	0.013 (0.011 - 0.015)	0.013 (0.011 - 0.015)	0.012 (0.010 - 0.014)	7.339 (4.939 - 10.096)	7.274 (4.947 - 10.108)	7.107 (4.805 - 9.792)
**All ages (rate per capita)**	0.032 (0.030 - 0.033)	0.042 (0.040 - 0.044)	0.048 (0.046 - 0.051)	0.002 (0.002 - 0.003)	0.003 (0.003 - 0.003)	0.004 (0.003 - 0.004)	0.879 (0.588 - 1.214)	1.160 (0.780 - 1.606)	1.336 (0.893 - 1.847)
**Age-standardized (rate per capita)**	0.052 (0.050 - 0.055)	0.052 (0.050 - 0.055)	0.052 (0.050 - 0.055)	0.004 (0.003 - 0.004)	0.004 (0.003 - 0.004)	0.004 (0.003 - 0.004)	1.442 (0.967 - 1.989)	1.443 (0.968 - 1.994)	1.436 (0.962 - 1.975)
**Total number of cases (thousands)**	261 (249 - 274)	424 (405 - 445)	543 (518 - 570)	20 (19 - 21)	31 (29 - 33)	40 (37 - 42)	7 (5 - 10)	12 (8 - 16)	15 (10 - 21)
**Turkey**									
**15 - 49 years (rate per capita)**	0.028 (0.027 - 0.030)	0.031 (0.030 - 0.033)	0.034 (0.032 - 0.036)	0.002 (0.002 - 0.003)	0.003 (0.002 - 0.003)	0.003 (0.003 - 0.003)	0.814 (0.537 - 1.129)	0.902 (0.601 - 1.271)	0.971 (0.641 - 1.359)
**50 - 69 years (rate per capita)**	0.196 (0.185 - 0.208)	0.202 (0.192 - 0.214)	0.197 (0.186 - 0.209)	0.017 (0.015 - 0.018)	0.018 (0.016 - 0.019)	0.017 (0.015 - 0.018)	5.420 (3.630 - 7.488)	5.632 (3.776 - 7.761)	5.489 (3.674 - 7.593)
**70+ years (rate per capita)**	0.402 (0.381 - 0.423)	0.417 (0.395 - 0.439)	0.398 (0.378 - 0.419)	0.016 (0.014 - 0.019)	0.018 (0.015 - 0.021)	0.017 (0.014 - 0.020)	10.652 (7.158 - 14.635)	11.139 (7.543 - 15.281)	10.611 (7.152 - 14.620)
**All ages (rate per capita)**	0.051 (0.049 - 0.053)	0.063 (0.061 - 0.065)	0.071 (0.068 - 0.073)	0.004 (0.004 - 0.004)	0.005 (0.004 - 0.005)	0.005 (0.005 - 0.005)	1.409 (0.948 - 1.933)	1.746 (1.176 - 2.400)	1.955 (1.318 - 2.693)
**Age-standardized (rate per capita)**	0.079 (0.076 - 0.082)	0.080 (0.078 - 0.083)	0.079 (0.076 - 0.082)	0.006 (0.005 - 0.006)	0.006 (0.005 - 0.006)	0.006 (0.005 - 0.006)	2.162 (1.455 - 2.969)	2.219 (1.493 - 3.047)	2.174 (1.466 - 2.994)
**Total number of cases (thousands)**	2,789 (2,689 - 2,892)	4,304 (4,148 - 4,461)	5,532 (5,334 - 5,740)	202 (193 - 210)	313 (299 - 327)	398 (380 - 416)	77 (52 - 106)	119 (80 - 164)	153 (103 - 211)
**United Arab Emirates**									
**15 - 49 years (rate per capita)**	0.020 (0.018 - 0.021)	0.019 (0.018 - 0.021)	0.023 (0.021 - 0.024)	0.002 (0.001 - 0.002)	0.002 (0.001 - 0.002)	0.002 (0.002 - 0.002)	0.561 (0.368 - 0.784)	0.552 (0.364 - 0.775)	0.647 (0.422 - 0.908)
**50 - 69 years (rate per capita)**	0.101 (0.094 - 0.109)	0.099 (0.091 - 0.107)	0.100 (0.092 - 0.108)	0.009 (0.008 - 0.010)	0.009 (0.008 - 0.010)	0.009 (0.008 - 0.010)	2.805 (1.861 - 3.885)	2.740 (1.819 - 3.795)	2.773 (1.850 - 3.853)
**70+ years (rate per capita)**	0.244 (0.226 - 0.263)	0.239 (0.222 - 0.258)	0.247 (0.229 - 0.267)	0.012 (0.010 - 0.013)	0.012 (0.010 - 0.014)	0.012 (0.010 - 0.015)	6.451 (4.360 - 8.904)	6.329 (4.253 - 8.691)	6.542 (4.429 - 9.029)
**All ages (rate per capita)**	0.019 (0.018 - 0.021)	0.021 (0.020 - 0.023)	0.028 (0.026 - 0.030)	0.002 (0.001 - 0.002)	0.002 (0.002 - 0.002)	0.002 (0.002 - 0.002)	0.546 (0.361 - 0.758)	0.608 (0.404 - 0.844)	0.794 (0.525 - 1.103)
**Age-standardized (rate per capita)**	0.046 (0.044 - 0.049)	0.047 (0.044 - 0.050)	0.048 (0.045 - 0.051)	0.003 (0.003 - 0.004)	0.003 (0.003 - 0.004)	0.003 (0.003 - 0.004)	1.264 (0.847 - 1.742)	1.281 (0.855 - 1.768)	1.305 (0.872 - 1.803)
**Total number of cases (thousands)**	35 (33 - 37)	96 (91 - 103)	257 (241 - 274)	3 (3 - 3)	8 (7 - 9)	21 (19 - 22)	1 (1 - 1)	3 (2 - 4)	7 (5 - 10)
**Yemen**									
**15 - 49 years (rate per capita)**	0.016 (0.014 - 0.020)	0.016 (0.013 - 0.019)	0.016 (0.013 - 0.020)	0.001 (0.001 - 0.002)	0.001 (0.001 - 0.002)	0.001 (0.001 - 0.002)	0.466 (0.300 - 0.684)	0.451 (0.291 - 0.660)	0.458 (0.298 - 0.673)
**50 - 69 years (rate per capita)**	0.131 (0.109 - 0.155)	0.126 (0.106 - 0.148)	0.133 (0.111 - 0.157)	0.011 (0.008 - 0.014)	0.011 (0.008 - 0.014)	0.011 (0.009 - 0.014)	3.619 (2.273 - 5.352)	3.468 (2.201 - 5.029)	3.667 (2.315 - 5.389)
**70+ years (rate per capita)**	0.272 (0.232 - 0.324)	0.274 (0.236 - 0.323)	0.273 (0.233 - 0.323)	0.014 (0.010 - 0.018)	0.013 (0.010 - 0.018)	0.014 (0.010 - 0.019)	7.227 (4.608 - 10.308)	7.237 (4.646 - 10.270)	7.226 (4.638 - 10.204)
**All ages (rate per capita)**	0.019 (0.017 - 0.021)	0.021 (0.018 - 0.024)	0.023 (0.020 - 0.026)	0.002 (0.001 - 0.002)	0.002 (0.001 - 0.002)	0.002 (0.002 - 0.002)	0.525 (0.338 - 0.745)	0.574 (0.371 - 0.814)	0.629 (0.405 - 0.889)
**Age-standardized (rate per capita)**	0.053 (0.046 - 0.060)	0.053 (0.046 - 0.060)	0.053 (0.046 - 0.060)	0.004 (0.003 - 0.004)	0.004 (0.003 - 0.004)	0.004 (0.003 - 0.004)	1.440 (0.920 - 2.040)	1.446 (0.931 - 2.046)	1.445 (0.928 - 2.055)
**Total number of cases (thousands)**	227 (198 - 257)	428 (373 - 483)	611 (533 - 690)	18 (16 - 21)	34 (30 - 39)	50 (43 - 57)	6 (4 - 9)	12 (8 - 17)	17 (11 - 24)
**South Asia**									
**15 - 49 years (rate per capita)**	0.011 (0.010 - 0.012)	0.011 (0.011 - 0.012)	0.012 (0.011 - 0.012)	0.001 (0.001 - 0.001)	0.001 (0.001 - 0.001)	0.001 (0.001 - 0.001)	0.308 (0.204 - 0.427)	0.326 (0.216 - 0.455)	0.335 (0.223 - 0.466)
**50 - 69 years (rate per capita)**	0.090 (0.085 - 0.095)	0.091 (0.086 - 0.096)	0.091 (0.085 - 0.096)	0.009 (0.008 - 0.009)	0.009 (0.008 - 0.009)	0.009 (0.008 - 0.009)	2.467 (1.651 - 3.411)	2.492 (1.664 - 3.454)	2.497 (1.670 - 3.464)

	Prevalence 1990	Prevalence 2005	Prevalence 2015	Incidence 1990	Incidence 2005	Incidence 2015	YLD rate 1990	YLD rate 2005	YLD rate 2015
**70+ years (rate per capita)**	0.195<br>(0.183 - 0.207)	0.193<br>(0.181 - 0.204)	0.191<br>(0.179 - 0.203)	0.008<br>(0.007 - 0.009)	0.008<br>(0.007 - 0.009)	0.008<br>(0.007 - 0.009)	5.133<br>(3.492 - 7.086)	5.092<br>(3.450 - 7.047)	5.068<br>(3.448 - 6.991)
**All ages (rate per capita)**	0.019<br>(0.018 - 0.019)	0.022<br>(0.021 - 0.023)	0.025<br>(0.024 - 0.026)	0.001<br>(0.001 - 0.002)	0.002<br>(0.002 - 0.002)	0.002<br>(0.002 - 0.002)	0.513<br>(0.344 - 0.707)	0.595<br>(0.399 - 0.819)	0.685<br>(0.460 - 0.945)
**Age-standardized (rate per capita)**	0.036<br>(0.034 - 0.037)	0.036<br>(0.034 - 0.037)	0.035<br>(0.034 - 0.037)	0.003<br>(0.002 - 0.003)	0.003<br>(0.002 - 0.003)	0.003<br>(0.002 - 0.003)	0.966<br>(0.652 - 1.324)	0.969<br>(0.653 - 1.331)	0.970<br>(0.653 - 1.334)
**Total number of cases (thousands)**	20,622<br>(19,789 - 21,492)	31,750<br>(30,412 - 33,113)	42,031<br>(40,339 - 43,845)	1,639<br>(1,560 - 1,724)	2,467<br>(2,347 - 2,596)	3,268<br>(3,107 - 3,436)	566<br>(380 - 780)	873<br>(586 - 1,202)	1,158<br>(777 - 1,598)
**South Asia**									
**15 - 49 years (rate per capita)**	0.011<br>(0.010 - 0.012)	0.011<br>(0.011 - 0.012)	0.012<br>(0.011 - 0.012)	0.001<br>(0.001 - 0.001)	0.001<br>(0.001 - 0.001)	0.001<br>(0.001 - 0.001)	0.308<br>(0.204 - 0.427)	0.326<br>(0.216 - 0.455)	0.335<br>(0.223 - 0.466)
**50 - 69 years (rate per capita)**	0.090<br>(0.085 - 0.095)	0.091<br>(0.086 - 0.096)	0.091<br>(0.085 - 0.096)	0.009<br>(0.008 - 0.009)	0.009<br>(0.008 - 0.009)	0.009<br>(0.008 - 0.009)	2.467<br>(1.651 - 3.411)	2.492<br>(1.664 - 3.454)	2.497<br>(1.670 - 3.464)
**70+ years (rate per capita)**	0.195<br>(0.183 - 0.207)	0.193<br>(0.181 - 0.204)	0.191<br>(0.179 - 0.203)	0.008<br>(0.007 - 0.009)	0.008<br>(0.007 - 0.009)	0.008<br>(0.007 - 0.009)	5.133<br>(3.492 - 7.086)	5.092<br>(3.450 - 7.047)	5.068<br>(3.448 - 6.991)
**All ages (rate per capita)**	0.019<br>(0.018 - 0.019)	0.022<br>(0.021 - 0.023)	0.025<br>(0.024 - 0.026)	0.001<br>(0.001 - 0.002)	0.002<br>(0.002 - 0.002)	0.002<br>(0.002 - 0.002)	0.513<br>(0.344 - 0.707)	0.595<br>(0.399 - 0.819)	0.685<br>(0.460 - 0.945)
**Age-standardized (rate per capita)**	0.036<br>(0.034 - 0.037)	0.036<br>(0.034 - 0.037)	0.035<br>(0.034 - 0.037)	0.003<br>(0.002 - 0.003)	0.003<br>(0.002 - 0.003)	0.003<br>(0.002 - 0.003)	0.966<br>(0.652 - 1.324)	0.969<br>(0.653 - 1.331)	0.970<br>(0.653 - 1.334)
**Total number of cases (thousands)**	20,622<br>(19,789 - 21,492)	31,750<br>(30,412 - 33,113)	42,031<br>(40,339 - 43,845)	1,639<br>(1,560 - 1,724)	2,467<br>(2,347 - 2,596)	3,268<br>(3,107 - 3,436)	566<br>(380 - 780)	873<br>(586 - 1,202)	1,158<br>(777 - 1,598)
**Bangladesh**									
**15 - 49 years (rate per capita)**	0.005<br>(0.004 - 0.005)	0.005<br>(0.004 - 0.005)	0.005<br>(0.005 - 0.006)	0.000<br>(0.000 - 0.000)	0.000<br>(0.000 - 0.000)	0.000<br>(0.000 - 0.000)	0.129<br>(0.085 - 0.182)	0.137<br>(0.090 - 0.194)	0.152<br>(0.101 - 0.215)
**50 - 69 years (rate per capita)**	0.040<br>(0.037 - 0.043)	0.041<br>(0.038 - 0.045)	0.038<br>(0.036 - 0.041)	0.004<br>(0.003 - 0.004)	0.004<br>(0.003 - 0.004)	0.003<br>(0.003 - 0.004)	1.094<br>(0.726 - 1.523)	1.140<br>(0.757 - 1.576)	1.064<br>(0.702 - 1.476)
**70+ years (rate per capita)**	0.090<br>(0.083 - 0.097)	0.088<br>(0.081 - 0.095)	0.088<br>(0.081 - 0.095)	0.005<br>(0.004 - 0.006)	0.005<br>(0.004 - 0.005)	0.005<br>(0.004 - 0.005)	2.367<br>(1.606 - 3.283)	2.332<br>(1.577 - 3.215)	2.331<br>(1.582 - 3.226)
**All ages (rate per capita)**	0.007<br>(0.007 - 0.008)	0.009<br>(0.008 - 0.009)	0.010<br>(0.010 - 0.011)	0.001<br>(0.001 - 0.001)	0.001<br>(0.001 - 0.001)	0.001<br>(0.001 - 0.001)	0.200<br>(0.134 - 0.278)	0.241<br>(0.161 - 0.331)	0.281<br>(0.188 - 0.391)
**Age-standardized (rate per capita)**	0.016<br>(0.015 - 0.017)	0.016<br>(0.015 - 0.017)	0.016<br>(0.015 - 0.017)	0.001<br>(0.001 - 0.001)	0.001<br>(0.001 - 0.001)	0.001<br>(0.001 - 0.001)	0.436<br>(0.293 - 0.599)	0.433<br>(0.292 - 0.596)	0.438<br>(0.294 - 0.606)
**Total number of cases (thousands)**	771<br>(728 - 818)	1,247<br>(1,172 - 1,319)	1,640<br>(1,553 - 1,738)	64<br>(60 - 68)	98<br>(92 - 105)	128<br>(120 - 136)	21<br>(14 - 29)	34<br>(23 - 47)	45<br>(30 - 63)
**Bhutan**									
**15 - 49 years (rate per capita)**	0.008<br>(0.006 - 0.010)	0.007<br>(0.006 - 0.010)	0.009<br>(0.007 - 0.011)	0.001<br>(0.001 - 0.001)	0.001<br>(0.001 - 0.001)	0.001<br>(0.001 - 0.001)	0.232<br>(0.144 - 0.344)	0.214<br>(0.134 - 0.319)	0.244<br>(0.152 - 0.368)
**50 - 69 years (rate per capita)**	0.065<br>(0.051 - 0.083)	0.068<br>(0.053 - 0.086)	0.067<br>(0.052 - 0.085)	0.006<br>(0.004 - 0.008)	0.006<br>(0.004 - 0.008)	0.006<br>(0.004 - 0.008)	1.806<br>(1.101 - 2.728)	1.879<br>(1.137 - 2.827)	1.852<br>(1.120 - 2.784)
**70+ years (rate per capita)**	0.143<br>(0.114 - 0.179)	0.144<br>(0.115 - 0.179)	0.144<br>(0.116 - 0.178)	0.008<br>(0.005 - 0.011)	0.007<br>(0.005 - 0.010)	0.007<br>(0.005 - 0.010)	3.821<br>(2.340 - 5.550)	3.832<br>(2.365 - 5.553)	3.813<br>(2.365 - 5.496)
**All ages (rate per capita)**	0.012<br>(0.010 - 0.014)	0.014<br>(0.012 - 0.016)	0.017<br>(0.014 - 0.020)	0.001<br>(0.001 - 0.001)	0.001<br>(0.001 - 0.001)	0.001<br>(0.001 - 0.002)	0.336<br>(0.211 - 0.491)	0.385<br>(0.240 - 0.559)	0.473<br>(0.295 - 0.687)
**Age-standardized (rate per capita)**	0.026<br>(0.022 - 0.031)	0.026<br>(0.022 - 0.031)	0.026<br>(0.022 - 0.031)	0.002<br>(0.002 - 0.002)	0.002<br>(0.002 - 0.002)	0.002<br>(0.002 - 0.002)	0.723<br>(0.450 - 1.044)	0.726<br>(0.449 - 1.043)	0.725<br>(0.449 - 1.043)
**Total number of cases (thousands)**	6<br>(5 - 8)	9<br>(8 - 11)	13<br>(11 - 16)	1<br>(0 - 1)	1<br>(1 - 1)	1<br>(1 - 1)	0<br>(0 - 0)	0<br>(0 - 0)	0<br>(0 - 1)
**India**									
**15 - 49 years (rate per capita)**	0.012<br>(0.011 - 0.013)	0.013<br>(0.012 - 0.013)	0.013<br>(0.012 - 0.014)	0.001<br>(0.001 - 0.001)	0.001<br>(0.001 - 0.001)	0.001<br>(0.001 - 0.001)	0.336<br>(0.223 - 0.467)	0.361<br>(0.239 - 0.504)	0.371<br>(0.246 - 0.515)
**50 - 69 years (rate per capita)**	0.097<br>(0.092 - 0.103)	0.098<br>(0.092 - 0.103)	0.098<br>(0.093 - 0.104)	0.009<br>(0.009 - 0.010)	0.009<br>(0.009 - 0.010)	0.009<br>(0.009 - 0.010)	2.666<br>(1.784 - 3.696)	2.677<br>(1.792 - 3.704)	2.699<br>(1.804 - 3.755)
**70+ years (rate per capita)**	0.209<br>(0.197 - 0.222)	0.209<br>(0.197 - 0.221)	0.207<br>(0.195 - 0.220)	0.009<br>(0.007 - 0.010)	0.008<br>(0.007 - 0.009)	0.008<br>(0.007 - 0.009)	5.514<br>(3.747 - 7.578)	5.520<br>(3.737 - 7.600)	5.501<br>(3.739 - 7.556)
**All ages (rate per capita)**	0.021<br>(0.020 - 0.022)	0.024<br>(0.023 - 0.025)	0.028<br>(0.027 - 0.029)	0.002<br>(0.002 - 0.002)	0.002<br>(0.002 - 0.002)	0.002<br>(0.002 - 0.002)	0.567<br>(0.381 - 0.781)	0.665<br>(0.446 - 0.916)	0.770<br>(0.517 - 1.062)
**Age-standardized (rate per capita)**	0.038<br>(0.037 - 0.040)	0.039<br>(0.037 - 0.040)	0.038<br>(0.037 - 0.040)	0.003<br>(0.003 - 0.003)	0.003<br>(0.003 - 0.003)	0.003<br>(0.003 - 0.003)	1.043<br>(0.704 - 1.428)	1.052<br>(0.709 - 1.445)	1.050<br>(0.708 - 1.444)
**Total number of cases (thousands)**	18,001<br>(17,288 - 18,734)	27,697<br>(26,545 - 28,861)	36,682<br>(35,236 - 38,205)	1,430<br>(1,361 - 1,500)	2,150<br>(2,051 - 2,260)	2,852<br>(2,712 - 2,992)	494<br>(332 - 680)	761<br>(511 - 1,049)	1,010<br>(678 - 1,393)
**Nepal**									
**15 - 49 years (rate per capita)**	0.006<br>(0.006 - 0.007)	0.006<br>(0.006 - 0.007)	0.006<br>(0.006 - 0.007)	0.001<br>(0.000 - 0.001)	0.001<br>(0.000 - 0.001)	0.001<br>(0.000 - 0.001)	0.177<br>(0.120 - 0.250)	0.181<br>(0.120 - 0.254)	0.183<br>(0.123 - 0.257)
**50 - 69 years (rate per capita)**	0.052<br>(0.047 - 0.056)	0.052<br>(0.048 - 0.057)	0.054<br>(0.049 - 0.058)	0.005<br>(0.004 - 0.005)	0.005<br>(0.004 - 0.005)	0.005<br>(0.004 - 0.005)	1.426<br>(0.948 - 1.989)	1.445<br>(0.960 - 2.024)	1.483<br>(0.975 - 2.077)
**70+ years (rate per capita)**	0.111<br>(0.102 - 0.120)	0.111<br>(0.102 - 0.120)	0.112<br>(0.103 - 0.121)	0.006<br>(0.005 - 0.007)	0.006<br>(0.005 - 0.007)	0.006<br>(0.005 - 0.007)	2.963<br>(1.981 - 4.107)	2.958<br>(1.999 - 4.090)	2.999<br>(2.012 - 4.166)
**All ages (rate per capita)**	0.010<br>(0.009 - 0.011)	0.011<br>(0.011 - 0.012)	0.014<br>(0.013 - 0.014)	0.001<br>(0.001 - 0.001)	0.001<br>(0.001 - 0.001)	0.001<br>(0.001 - 0.001)	0.275<br>(0.185 - 0.381)	0.311<br>(0.209 - 0.428)	0.377<br>(0.252 - 0.522)
**Age-standardized (rate per capita)**	0.020<br>(0.019 - 0.022)	0.020<br>(0.019 - 0.022)	0.020<br>(0.019 - 0.022)	0.001<br>(0.001 - 0.002)	0.001<br>(0.001 - 0.002)	0.001<br>(0.001 - 0.002)	0.557<br>(0.375 - 0.774)	0.557<br>(0.374 - 0.769)	0.564<br>(0.377 - 0.781)
**Total number of cases (thousands)**	186<br>(176 - 198)	287<br>(270 - 305)	388<br>(366 - 413)	15<br>(14 - 16)	22<br>(21 - 24)	30<br>(28 - 32)	5<br>(3 - 7)	8<br>(5 - 11)	11<br>(7 - 15)
**Pakistan**									
**15 - 49 years (rate per capita)**	0.009<br>(0.008 - 0.010)	0.009<br>(0.008 - 0.010)	0.009<br>(0.009 - 0.010)	0.001<br>(0.001 - 0.001)	0.001<br>(0.001 - 0.001)	0.001<br>(0.001 - 0.001)	0.257<br>(0.170 - 0.363)	0.257<br>(0.168 - 0.363)	0.268<br>(0.178 - 0.377)
**50 - 69 years (rate per capita)**	0.078<br>(0.072 - 0.085)	0.080<br>(0.074 - 0.086)	0.076<br>(0.070 - 0.083)	0.007<br>(0.007 - 0.008)	0.007<br>(0.007 - 0.008)	0.007<br>(0.006 - 0.008)	2.166<br>(1.438 - 3.058)	2.204<br>(1.453 - 3.082)	2.099<br>(1.394 - 2.933)
**70+ years (rate per capita)**	0.169<br>(0.156 - 0.183)	0.169<br>(0.156 - 0.183)	0.168<br>(0.155 - 0.182)	0.008<br>(0.006 - 0.009)	0.008<br>(0.006 - 0.009)	0.008<br>(0.006 - 0.009)	4.504<br>(3.038 - 6.250)	4.484<br>(2.995 - 6.175)	4.451<br>(2.985 - 6.132)
**All ages (rate per capita)**	0.015<br>(0.015 - 0.016)	0.016<br>(0.015 - 0.017)	0.017<br>(0.017 - 0.019)	0.001<br>(0.001 - 0.001)	0.001<br>(0.001 - 0.001)	0.001<br>(0.001 - 0.001)	0.425<br>(0.284 - 0.589)	0.450<br>(0.300 - 0.621)	0.483<br>(0.323 - 0.666)
**Age-standardized (rate per capita)**	0.031<br>(0.029 - 0.033)	0.031<br>(0.029 - 0.032)	0.031<br>(0.029 - 0.032)	0.002<br>(0.002 - 0.002)	0.002<br>(0.002 - 0.002)	0.002<br>(0.002 - 0.002)	0.841<br>(0.564 - 1.162)	0.839<br>(0.561 - 1.155)	0.835<br>(0.560 - 1.152)
**Total number of cases (thousands)**	1,657<br>(1,566 - 1,757)	2,510<br>(2,372 - 2,652)	3,308<br>(3,131 - 3,502)	131<br>(122 - 140)	195<br>(183 - 208)	257<br>(241 - 274)	46<br>(31 - 64)	69<br>(46 - 96)	91<br>(61 - 126)
**Sub-Saharan Africa**									
**15 - 49 years (rate per capita)**	0.018<br>(0.016 - 0.019)	0.017<br>(0.016 - 0.019)	0.017<br>(0.016 - 0.019)	0.001<br>(0.001 - 0.001)	0.001<br>(0.001 - 0.001)	0.001<br>(0.001 - 0.001)	0.505<br>(0.336 - 0.702)	0.488<br>(0.324 - 0.680)	0.482<br>(0.320 - 0.672)
**50 - 69 years (rate per capita)**	0.064<br>(0.059 - 0.069)	0.064<br>(0.059 - 0.069)	0.063<br>(0.059 - 0.069)	0.004<br>(0.003 - 0.004)	0.004<br>(0.003 - 0.004)	0.004<br>(0.003 - 0.004)	1.748<br>(1.148 - 2.436)	1.752<br>(1.153 - 2.437)	1.748<br>(1.149 - 2.426)
**70+ years (rate per capita)**	0.113<br>(0.105 - 0.123)	0.111<br>(0.103 - 0.121)	0.110<br>(0.102 - 0.119)	0.005<br>(0.004 - 0.006)	0.005<br>(0.004 - 0.006)	0.005<br>(0.004 - 0.006)	2.980<br>(1.995 - 4.091)	2.942<br>(1.970 - 4.058)	2.906<br>(1.946 - 4.006)
**All ages (rate per capita)**	0.015<br>(0.014 - 0.016)	0.015<br>(0.014 - 0.016)	0.015<br>(0.014 - 0.016)	0.001<br>(0.001 - 0.001)	0.001<br>(0.001 - 0.001)	0.001<br>(0.001 - 0.001)	0.420<br>(0.279 - 0.579)	0.417<br>(0.276 - 0.572)	0.420<br>(0.278 - 0.580)
**Age-standardized (rate per capita)**	0.029<br>(0.028 - 0.031)	0.029<br>(0.027 - 0.031)	0.029<br>(0.027 - 0.030)	0.002<br>(0.001 - 0.002)	0.002<br>(0.001 - 0.002)	0.002<br>(0.001 - 0.002)	0.805<br>(0.534 - 1.110)	0.798<br>(0.528 - 1.097)	0.786<br>(0.521 - 1.083)
**Total number of cases (thousands)**	7,394<br>(6,966 - 7,886)	10,957<br>(10,317 - 11,677)	14,448<br>(13,589 - 15,420)	414<br>(388 - 443)	616<br>(577 - 660)	813<br>(762 - 871)	206<br>(137 - 284)	305<br>(202 - 419)	403<br>(267 - 557)
**Southern Sub-Saharan Africa**									
**15 - 49 years (rate per capita)**	0.052<br>(0.049 - 0.055)	0.052<br>(0.049 - 0.055)	0.053<br>(0.051 - 0.057)	0.002<br>(0.002 - 0.003)	0.002<br>(0.002 - 0.003)	0.002<br>(0.002 - 0.003)	1.483<br>(0.990 - 2.067)	1.481<br>(0.993 - 2.059)	1.522<br>(1.022 - 2.114)
**50 - 69 years (rate per capita)**	0.154<br>(0.146 - 0.162)	0.151<br>(0.143 - 0.158)	0.150<br>(0.143 - 0.158)	0.009<br>(0.009 - 0.010)	0.009<br>(0.008 - 0.010)	0.009<br>(0.008 - 0.010)	4.259<br>(2.861 - 5.875)	4.145<br>(2.779 - 5.687)	4.121<br>(2.768 - 5.668)
**70+ years (rate per capita)**	0.236<br>(0.223 - 0.250)	0.232<br>(0.220 - 0.246)	0.233<br>(0.220 - 0.246)	0.008<br>(0.007 - 0.009)	0.008<br>(0.007 - 0.009)	0.008<br>(0.007 - 0.009)	6.264<br>(4.252 - 8.572)	6.134<br>(4.161 - 8.415)	6.133<br>(4.180 - 8.421)
**All ages (rate per capita)**	0.044<br>(0.042 - 0.046)	0.050<br>(0.047 - 0.052)	0.054<br>(0.051 - 0.056)	0.002<br>(0.002 - 0.002)	0.002<br>(0.002 - 0.003)	0.003<br>(0.002 - 0.003)	1.241<br>(0.834 - 1.715)	1.380<br>(0.928 - 1.902)	1.493<br>(1.010 - 2.059)
**Age-standardized (rate per capita)**	0.071<br>(0.069 - 0.074)	0.071<br>(0.068 - 0.074)	0.071<br>(0.068 - 0.074)	0.003<br>(0.003 - 0.004)	0.003<br>(0.003 - 0.004)	0.003<br>(0.003 - 0.004)	1.983<br>(1.333 - 2.735)	1.966<br>(1.321 - 2.710)	1.963<br>(1.326 - 2.697)
**Total number of cases (thousands)**	2,358<br>(2,264 - 2,469)	3,388<br>(3,249 - 3,543)	4,149<br>(3,978 - 4,345)	115<br>(110 - 120)	164<br>(157 - 172)	202<br>(193 - 211)	66<br>(45 - 92)	94<br>(64 - 130)	116<br>(78 - 159)
**Botswana**									
**15 - 49 years (rate per capita)**	0.044<br>(0.037 - 0.052)	0.045<br>(0.037 - 0.052)	0.049<br>(0.041 - 0.057)	0.002<br>(0.002 - 0.003)	0.002<br>(0.002 - 0.003)	0.002<br>(0.002 - 0.003)	1.268<br>(0.830 - 1.815)	1.264<br>(0.830 - 1.809)	1.384<br>(0.913 - 1.971)
**50 - 69 years (rate per capita)**	0.144<br>(0.126 - 0.163)	0.144<br>(0.125 - 0.163)	0.145<br>(0.126 - 0.164)	0.009<br>(0.007 - 0.011)	0.009<br>(0.007 - 0.011)	0.009<br>(0.007 - 0.011)	3.993<br>(2.597 - 5.671)	3.951<br>(2.562 - 5.609)	3.951<br>(2.569 - 5.621)
**70+ years (rate per capita)**	0.233<br>(0.203 - 0.270)	0.233<br>(0.203 - 0.270)	0.234<br>(0.203 - 0.271)	0.009<br>(0.006 - 0.012)	0.009<br>(0.006 - 0.012)	0.009<br>(0.006 - 0.012)	6.218<br>(4.022 - 8.718)	6.171<br>(4.028 - 8.602)	6.184<br>(4.028 - 8.633)
**All ages (rate per capita)**	0.034<br>(0.030 - 0.039)	0.041<br>(0.035 - 0.046)	0.047<br>(0.041 - 0.053)	0.002<br>(0.002 - 0.002)	0.002<br>(0.002 - 0.002)	0.002<br>(0.002 - 0.003)	0.967<br>(0.645 - 1.354)	1.131<br>(0.754 - 1.583)	1.301<br>(0.866 - 1.813)
**Age-standardized (rate per capita)**	0.067<br>(0.059 - 0.074)	0.067<br>(0.059 - 0.075)	0.067<br>(0.059 - 0.075)	0.003<br>(0.003 - 0.004)	0.003<br>(0.003 - 0.004)	0.003<br>(0.003 - 0.004)	1.860<br>(1.215 - 2.566)	1.845<br>(1.210 - 2.561)	1.842<br>(1.212 - 2.550)
**Total number of cases (thousands)**	48<br>(41 - 54)	76<br>(66 - 85)	106<br>(92 - 119)	3<br>(2 - 3)	4<br>(4 - 5)	5<br>(5 - 6)	1<br>(1 - 2)	2<br>(1 - 3)	3<br>(2 - 4)
**Lesotho**									
**15 - 49 years (rate per capita)**	0.043<br>(0.036 - 0.050)	0.042<br>(0.035 - 0.049)	0.043<br>(0.036 - 0.051)	0.002<br>(0.002 - 0.003)	0.002<br>(0.002 - 0.003)	0.002<br>(0.002 - 0.003)	1.227<br>(0.806 - 1.742)	1.200<br>(0.795 - 1.689)	1.232<br>(0.808 - 1.744)
**50 - 69 years (rate per capita)**	0.148<br>(0.129 - 0.168)	0.146<br>(0.128 - 0.166)	0.150<br>(0.130 - 0.171)	0.009<br>(0.007 - 0.012)	0.009<br>(0.007 - 0.011)	0.009<br>(0.007 - 0.012)	4.101<br>(2.664 - 5.855)	4.017<br>(2.624 - 5.681)	4.097<br>(2.666 - 5.799)
**70+ years (rate per capita)**	0.234<br>(0.203 - 0.270)	0.231<br>(0.201 - 0.267)	0.230<br>(0.200 - 0.265)	0.009<br>(0.007 - 0.012)	0.009<br>(0.006 - 0.011)	0.008<br>(0.006 - 0.011)	6.253<br>(4.085 - 8.753)	6.122<br>(4.010 - 8.613)	6.055<br>(3.964 - 8.504)
**All ages (rate per capita)**	0.039<br>(0.034 - 0.043)	0.040<br>(0.036 - 0.045)	0.041<br>(0.036 - 0.046)	0.002<br>(0.002 - 0.002)	0.002<br>(0.002 - 0.002)	0.002<br>(0.002 - 0.002)	1.088<br>(0.713 - 1.521)	1.122<br>(0.735 - 1.558)	1.148<br>(0.758 - 1.610)
**Age-standardized (rate per capita)**	0.067<br>(0.060 - 0.075)	0.067<br>(0.060 - 0.075)	0.067<br>(0.059 - 0.075)	0.003<br>(0.003 - 0.004)	0.003<br>(0.003 - 0.004)	0.003<br>(0.003 - 0.004)	1.872<br>(1.218 - 2.592)	1.847<br>(1.205 - 2.545)	1.832<br>(1.200 - 2.521)
**Total number of cases (thousands)**	62<br>(55 - 70)	78<br>(69 - 87)	88<br>(77 - 99)	3<br>(3 - 4)	4<br>(4 - 5)	5<br>(4 - 5)	2<br>(1 - 2)	2<br>(1 - 3)	2<br>(2 - 3)
**Namibia**									

	Prevalence 1990	Prevalence 2005	Prevalence 2015	Incidence 1990	Incidence 2005	Incidence 2015	YLD rate 1990	YLD rate 2005	YLD rate 2015
15 - 49 years (rate per capita)	0.067 (0.063 - 0.072)	0.071 (0.067 - 0.076)	0.071 (0.067 - 0.076)	0.003 (0.003 - 0.003)	0.003 (0.003 - 0.003)	0.003 (0.003 - 0.003)	1.927 (1.289 - 2.711)	2.035 (1.355 - 2.843)	2.031 (1.365 - 2.844)
50 - 69 years (rate per capita)	0.199 (0.188 - 0.212)	0.196 (0.185 - 0.208)	0.194 (0.183 - 0.205)	0.013 (0.011 - 0.015)	0.012 (0.011 - 0.014)	0.012 (0.011 - 0.014)	5.529 (3.678 - 7.738)	5.420 (3.620 - 7.514)	5.347 (3.575 - 7.442)
70+ years (rate per capita)	0.313 (0.292 - 0.337)	0.311 (0.290 - 0.332)	0.311 (0.290 - 0.334)	0.010 (0.008 - 0.012)	0.010 (0.008 - 0.012)	0.010 (0.008 - 0.012)	8.373 (5.641 - 11.596)	8.273 (5.613 - 11.447)	8.265 (5.582 - 11.406)
All ages (rate per capita)	0.053 (0.051 - 0.056)	0.058 (0.055 - 0.060)	0.061 (0.059 - 0.065)	0.003 (0.002 - 0.003)	0.003 (0.003 - 0.003)	0.003 (0.003 - 0.003)	1.501 (1.009 - 2.093)	1.621 (1.086 - 2.246)	1.726 (1.163 - 2.399)
Age-standardized (rate per capita)	0.095 (0.091 - 0.099)	0.095 (0.091 - 0.100)	0.094 (0.090 - 0.099)	0.004 (0.004 - 0.005)	0.004 (0.004 - 0.005)	0.004 (0.004 - 0.005)	2.637 (1.773 - 3.657)	2.647 (1.777 - 3.672)	2.620 (1.771 - 3.624)
Total number of cases (thousands)	76 (72 - 79)	117 (112 - 123)	151 (144 - 158)	4 (3 - 4)	5 (5 - 6)	7 (7 - 8)	2 (1 - 3)	3 (2 - 5)	4 (3 - 6)
**South Africa**									
15 - 49 years (rate per capita)	0.055 (0.052 - 0.058)	0.056 (0.053 - 0.059)	0.057 (0.054 - 0.060)	0.002 (0.002 - 0.003)	0.002 (0.002 - 0.003)	0.003 (0.002 - 0.003)	1.577 (1.053 - 2.199)	1.582 (1.060 - 2.197)	1.616 (1.083 - 2.246)
50 - 69 years (rate per capita)	0.160 (0.153 - 0.168)	0.154 (0.147 - 0.162)	0.153 (0.146 - 0.160)	0.010 (0.009 - 0.011)	0.009 (0.009 - 0.010)	0.009 (0.009 - 0.010)	4.431 (2.987 - 6.099)	4.241 (2.858 - 5.839)	4.194 (2.827 - 5.765)
70+ years (rate per capita)	0.242 (0.229 - 0.255)	0.237 (0.225 - 0.249)	0.237 (0.225 - 0.250)	0.008 (0.007 - 0.009)	0.008 (0.007 - 0.009)	0.008 (0.007 - 0.009)	6.401 (4.368 - 8.775)	6.253 (4.258 - 8.564)	6.240 (4.247 - 8.564)
All ages (rate per capita)	0.049 (0.047 - 0.051)	0.055 (0.053 - 0.058)	0.061 (0.058 - 0.063)	0.002 (0.002 - 0.002)	0.003 (0.003 - 0.003)	0.003 (0.003 - 0.003)	1.367 (0.921 - 1.896)	1.540 (1.038 - 2.117)	1.687 (1.142 - 2.326)
Age-standardized (rate per capita)	0.074 (0.071 - 0.077)	0.073 (0.070 - 0.076)	0.073 (0.070 - 0.076)	0.003 (0.003 - 0.004)	0.003 (0.003 - 0.004)	0.003 (0.003 - 0.004)	2.053 (1.384 - 2.828)	2.020 (1.364 - 2.785)	2.016 (1.367 - 2.765)
Total number of cases (thousands)	1,832 (1,760 - 1,918)	2,679 (2,574 - 2,800)	3,257 (3,133 - 3,397)	88 (85 - 92)	128 (123 - 134)	158 (152 - 166)	51 (35 - 71)	75 (50 - 103)	91 (61 - 125)
**Swaziland**									
15 - 49 years (rate per capita)	0.041 (0.038 - 0.045)	0.040 (0.037 - 0.043)	0.042 (0.039 - 0.045)	0.002 (0.002 - 0.002)	0.002 (0.002 - 0.002)	0.002 (0.002 - 0.002)	1.184 (0.785 - 1.669)	1.125 (0.748 - 1.572)	1.175 (0.790 - 1.644)
50 - 69 years (rate per capita)	0.128 (0.120 - 0.137)	0.129 (0.120 - 0.138)	0.131 (0.122 - 0.139)	0.008 (0.007 - 0.009)	0.008 (0.007 - 0.009)	0.008 (0.007 - 0.009)	3.537 (2.363 - 4.934)	3.519 (2.345 - 4.866)	3.538 (2.377 - 4.881)
70+ years (rate per capita)	0.206 (0.191 - 0.222)	0.204 (0.189 - 0.220)	0.206 (0.191 - 0.222)	0.008 (0.007 - 0.009)	0.008 (0.007 - 0.009)	0.008 (0.007 - 0.009)	5.480 (3.694 - 7.544)	5.355 (3.623 - 7.315)	5.396 (3.655 - 7.395)
All ages (rate per capita)	0.030 (0.029 - 0.032)	0.033 (0.032 - 0.035)	0.037 (0.035 - 0.039)	0.002 (0.001 - 0.002)	0.002 (0.002 - 0.002)	0.002 (0.002 - 0.002)	0.855 (0.572 - 1.189)	0.923 (0.617 - 1.273)	1.021 (0.688 - 1.411)
Age-standardized (rate per capita)	0.061 (0.058 - 0.065)	0.061 (0.058 - 0.064)	0.061 (0.058 - 0.064)	0.003 (0.003 - 0.003)	0.003 (0.003 - 0.003)	0.003 (0.003 - 0.003)	1.693 (1.132 - 2.346)	1.669 (1.116 - 2.283)	1.658 (1.117 - 2.281)
Total number of cases (thousands)	26 (25 - 28)	37 (35 - 39)	48 (45 - 50)	1 (1 - 1)	2 (2 - 2)	2 (2 - 3)	1 (0 - 1)	1 (1 - 1)	1 (1 - 2)
**Zimbabwe**									
15 - 49 years (rate per capita)	0.040 (0.037 - 0.043)	0.038 (0.035 - 0.041)	0.041 (0.038 - 0.044)	0.002 (0.002 - 0.002)	0.002 (0.002 - 0.002)	0.002 (0.002 - 0.002)	1.135 (0.752 - 1.587)	1.081 (0.724 - 1.506)	1.174 (0.785 - 1.634)
50 - 69 years (rate per capita)	0.123 (0.114 - 0.132)	0.124 (0.116 - 0.133)	0.123 (0.115 - 0.132)	0.007 (0.006 - 0.008)	0.007 (0.006 - 0.008)	0.007 (0.007 - 0.008)	3.412 (2.275 - 4.715)	3.411 (2.273 - 4.704)	3.398 (2.269 - 4.697)
70+ years (rate per capita)	0.196 (0.181 - 0.211)	0.194 (0.180 - 0.210)	0.194 (0.180 - 0.209)	0.008 (0.006 - 0.009)	0.007 (0.006 - 0.009)	0.007 (0.006 - 0.008)	5.247 (3.524 - 7.228)	5.150 (3.483 - 7.066)	5.167 (3.492 - 7.120)
All ages (rate per capita)	0.030 (0.028 - 0.032)	0.031 (0.029 - 0.032)	0.032 (0.030 - 0.034)	0.001 (0.001 - 0.002)	0.002 (0.002 - 0.002)	0.002 (0.001 - 0.002)	0.844 (0.563 - 1.167)	0.860 (0.576 - 1.188)	0.901 (0.604 - 1.245)
Age-standardized (rate per capita)	0.058 (0.055 - 0.061)	0.058 (0.055 - 0.061)	0.058 (0.055 - 0.061)	0.003 (0.003 - 0.003)	0.003 (0.003 - 0.003)	0.003 (0.003 - 0.003)	1.618 (1.082 - 2.228)	1.607 (1.083 - 2.218)	1.612 (1.078 - 2.214)
Total number of cases (thousands)	315 (298 - 333)	401 (379 - 423)	500 (472 - 529)	16 (15 - 17)	21 (20 - 22)	24 (23 - 26)	9 (6 - 12)	11 (7 - 15)	14 (9 - 19)
**Western Sub-Saharan Africa**									
15 - 49 years (rate per capita)	0.013 (0.012 - 0.014)	0.013 (0.012 - 0.014)	0.013 (0.012 - 0.014)	0.001 (0.001 - 0.001)	0.001 (0.001 - 0.001)	0.001 (0.001 - 0.001)	0.364 (0.243 - 0.509)	0.363 (0.242 - 0.506)	0.370 (0.246 - 0.518)
50 - 69 years (rate per capita)	0.051 (0.046 - 0.055)	0.051 (0.046 - 0.055)	0.051 (0.046 - 0.055)	0.003 (0.003 - 0.004)	0.003 (0.003 - 0.004)	0.003 (0.003 - 0.004)	1.387 (0.906 - 1.925)	1.392 (0.911 - 1.941)	1.397 (0.914 - 1.935)
70+ years (rate per capita)	0.088 (0.080 - 0.098)	0.089 (0.081 - 0.098)	0.089 (0.081 - 0.099)	0.005 (0.004 - 0.006)	0.005 (0.004 - 0.006)	0.005 (0.004 - 0.006)	2.320 (1.535 - 3.190)	2.336 (1.547 - 3.220)	2.356 (1.555 - 3.245)
All ages (rate per capita)	0.011 (0.011 - 0.012)	0.011 (0.011 - 0.012)	0.011 (0.011 - 0.012)	0.001 (0.001 - 0.001)	0.001 (0.001 - 0.001)	0.001 (0.001 - 0.001)	0.318 (0.211 - 0.441)	0.317 (0.209 - 0.439)	0.321 (0.213 - 0.444)
Age-standardized (rate per capita)	0.022 (0.021 - 0.024)	0.022 (0.021 - 0.024)	0.023 (0.021 - 0.024)	0.001 (0.001 - 0.001)	0.001 (0.001 - 0.001)	0.001 (0.001 - 0.001)	0.614 (0.406 - 0.847)	0.618 (0.408 - 0.847)	0.622 (0.412 - 0.859)
Total number of cases (thousands)	2,275 (2,111 - 2,453)	3,376 (3,124 - 3,642)	4,483 (4,158 - 4,840)	136 (126 - 147)	202 (187 - 218)	267 (248 - 289)	63 (42 - 88)	94 (62 - 130)	125 (83 - 174)
**Benin**									
15 - 49 years (rate per capita)	0.015 (0.012 - 0.018)	0.015 (0.012 - 0.018)	0.015 (0.012 - 0.018)	0.001 (0.001 - 0.001)	0.001 (0.001 - 0.001)	0.001 (0.001 - 0.001)	0.412 (0.261 - 0.611)	0.417 (0.267 - 0.616)	0.425 (0.273 - 0.624)
50 - 69 years (rate per capita)	0.062 (0.051 - 0.076)	0.062 (0.050 - 0.075)	0.061 (0.050 - 0.075)	0.004 (0.003 - 0.005)	0.004 (0.003 - 0.005)	0.004 (0.003 - 0.005)	1.700 (1.060 - 2.496)	1.707 (1.061 - 2.492)	1.696 (1.054 - 2.500)
70+ years (rate per capita)	0.106 (0.087 - 0.130)	0.106 (0.087 - 0.130)	0.106 (0.087 - 0.130)	0.006 (0.004 - 0.008)	0.005 (0.004 - 0.008)	0.006 (0.004 - 0.008)	2.772 (1.732 - 3.991)	2.787 (1.727 - 4.027)	2.799 (1.744 - 4.017)
All ages (rate per capita)	0.013 (0.011 - 0.016)	0.013 (0.011 - 0.015)	0.014 (0.012 - 0.016)	0.001 (0.001 - 0.001)	0.001 (0.001 - 0.001)	0.001 (0.001 - 0.001)	0.373 (0.241 - 0.531)	0.368 (0.237 - 0.528)	0.388 (0.250 - 0.557)
Age-standardized (rate per capita)	0.027 (0.023 - 0.031)	0.027 (0.023 - 0.031)	0.027 (0.023 - 0.031)	0.002 (0.001 - 0.002)	0.002 (0.001 - 0.002)	0.002 (0.001 - 0.002)	0.732 (0.469 - 1.053)	0.737 (0.473 - 1.051)	0.740 (0.474 - 1.075)
Total number of cases (thousands)	68 (57 - 78)	108 (91 - 126)	152 (127 - 177)	4 (4 - 5)	7 (6 - 8)	9 (8 - 11)	2 (1 - 3)	3 (2 - 4)	4 (3 - 6)
**Burkina Faso**									
15 - 49 years (rate per capita)	0.012 (0.011 - 0.013)	0.011 (0.011 - 0.012)	0.012 (0.011 - 0.013)	0.001 (0.001 - 0.001)	0.001 (0.001 - 0.001)	0.001 (0.001 - 0.001)	0.328 (0.219 - 0.458)	0.323 (0.217 - 0.452)	0.338 (0.225 - 0.476)
50 - 69 years (rate per capita)	0.047 (0.044 - 0.051)	0.046 (0.043 - 0.050)	0.046 (0.043 - 0.050)	0.003 (0.003 - 0.003)	0.003 (0.003 - 0.003)	0.003 (0.003 - 0.003)	1.301 (0.865 - 1.817)	1.281 (0.852 - 1.778)	1.284 (0.849 - 1.788)
70+ years (rate per capita)	0.082 (0.075 - 0.088)	0.081 (0.074 - 0.088)	0.081 (0.074 - 0.087)	0.005 (0.004 - 0.005)	0.005 (0.004 - 0.005)	0.005 (0.004 - 0.005)	2.140 (1.444 - 2.920)	2.134 (1.431 - 2.937)	2.159 (1.443 - 2.968)
All ages (rate per capita)	0.010 (0.010 - 0.011)	0.009 (0.009 - 0.010)	0.010 (0.009 - 0.010)	0.001 (0.001 - 0.001)	0.001 (0.001 - 0.001)	0.001 (0.001 - 0.001)	0.283 (0.189 - 0.389)	0.264 (0.177 - 0.367)	0.274 (0.183 - 0.380)
Age-standardized (rate per capita)	0.021 (0.020 - 0.022)	0.021 (0.019 - 0.022)	0.021 (0.020 - 0.022)	0.001 (0.001 - 0.001)	0.001 (0.001 - 0.001)	0.001 (0.001 - 0.001)	0.569 (0.380 - 0.777)	0.566 (0.380 - 0.778)	0.576 (0.384 - 0.792)
Total number of cases (thousands)	90 (85 - 96)	127 (120 - 136)	176 (166 - 188)	5 (5 - 6)	8 (7 - 8)	11 (10 - 11)	2 (2 - 3)	4 (2 - 5)	5 (3 - 7)
**Cameroon**									
15 - 49 years (rate per capita)	0.015 (0.012 - 0.018)	0.014 (0.011 - 0.018)	0.015 (0.012 - 0.018)	0.001 (0.001 - 0.001)	0.001 (0.001 - 0.001)	0.001 (0.001 - 0.001)	0.415 (0.265 - 0.606)	0.397 (0.254 - 0.591)	0.414 (0.265 - 0.611)
50 - 69 years (rate per capita)	0.062 (0.051 - 0.075)	0.062 (0.051 - 0.075)	0.062 (0.051 - 0.075)	0.004 (0.003 - 0.005)	0.004 (0.003 - 0.005)	0.004 (0.003 - 0.005)	1.695 (1.063 - 2.478)	1.700 (1.074 - 2.481)	1.700 (1.065 - 2.492)
70+ years (rate per capita)	0.105 (0.087 - 0.128)	0.105 (0.087 - 0.128)	0.105 (0.087 - 0.128)	0.005 (0.004 - 0.008)	0.005 (0.004 - 0.008)	0.005 (0.004 - 0.008)	2.753 (1.727 - 3.936)	2.753 (1.745 - 3.913)	2.765 (1.731 - 3.968)
All ages (rate per capita)	0.014 (0.012 - 0.016)	0.013 (0.011 - 0.016)	0.014 (0.012 - 0.016)	0.001 (0.001 - 0.001)	0.001 (0.001 - 0.001)	0.001 (0.001 - 0.001)	0.381 (0.246 - 0.541)	0.368 (0.239 - 0.524)	0.379 (0.245 - 0.543)
Age-standardized (rate per capita)	0.027 (0.023 - 0.031)	0.027 (0.023 - 0.031)	0.027 (0.023 - 0.031)	0.002 (0.001 - 0.002)	0.002 (0.001 - 0.002)	0.002 (0.001 - 0.002)	0.731 (0.472 - 1.036)	0.732 (0.472 - 1.040)	0.736 (0.475 - 1.051)
Total number of cases (thousands)	167 (141 - 195)	242 (205 - 283)	319 (269 - 374)	10 (9 - 12)	15 (13 - 18)	20 (17 - 23)	5 (3 - 7)	7 (4 - 10)	9 (6 - 13)
**Cape Verde**									
15 - 49 years (rate per capita)	0.013 (0.010 - 0.016)	0.014 (0.011 - 0.018)	0.016 (0.012 - 0.019)	0.001 (0.001 - 0.001)	0.001 (0.001 - 0.001)	0.001 (0.001 - 0.001)	0.374 (0.238 - 0.549)	0.406 (0.260 - 0.598)	0.447 (0.287 - 0.662)
50 - 69 years (rate per capita)	0.063 (0.051 - 0.074)	0.065 (0.053 - 0.078)	0.059 (0.048 - 0.070)	0.004 (0.003 - 0.005)	0.004 (0.003 - 0.005)	0.004 (0.002 - 0.005)	1.733 (1.085 - 2.551)	1.785 (1.113 - 2.592)	1.640 (1.033 - 2.405)
70+ years (rate per capita)	0.106 (0.088 - 0.129)	0.107 (0.087 - 0.130)	0.106 (0.088 - 0.128)	0.005 (0.004 - 0.007)	0.006 (0.004 - 0.008)	0.005 (0.004 - 0.007)	2.810 (1.759 - 4.046)	2.835 (1.755 - 4.081)	2.798 (1.758 - 4.000)
All ages (rate per capita)	0.014 (0.012 - 0.017)	0.016 (0.014 - 0.019)	0.019 (0.016 - 0.022)	0.001 (0.001 - 0.001)	0.001 (0.001 - 0.001)	0.001 (0.001 - 0.001)	0.401 (0.258 - 0.571)	0.446 (0.288 - 0.636)	0.523 (0.338 - 0.750)
Age-standardized (rate per capita)	0.027 (0.023 - 0.031)	0.027 (0.023 - 0.031)	0.027 (0.023 - 0.031)	0.002 (0.001 - 0.002)	0.002 (0.001 - 0.002)	0.002 (0.001 - 0.002)	0.747 (0.481 - 1.065)	0.745 (0.479 - 1.068)	0.745 (0.480 - 1.068)
Total number of cases (thousands)	5 (4 - 6)	8 (6 - 9)	10 (8 - 11)	0 (0 - 0)	0 (0 - 1)	1 (0 - 1)	0 (0 - 0)	0 (0 - 0)	0 (0 - 0)
**Chad**									
15 - 49 years (rate per capita)	0.019 (0.018 - 0.021)	0.019 (0.017 - 0.020)	0.018 (0.017 - 0.020)	0.001 (0.001 - 0.001)	0.001 (0.001 - 0.001)	0.001 (0.001 - 0.001)	0.550 (0.360 - 0.771)	0.535 (0.352 - 0.748)	0.524 (0.345 - 0.734)
50 - 69 years (rate per capita)	0.071 (0.066 - 0.076)	0.071 (0.066 - 0.075)	0.070 (0.065 - 0.075)	0.004 (0.004 - 0.005)	0.004 (0.004 - 0.005)	0.004 (0.004 - 0.005)	1.943 (1.296 - 2.702)	1.935 (1.291 - 2.696)	1.925 (1.291 - 2.674)
70+ years (rate per capita)	0.118 (0.109 - 0.127)	0.118 (0.109 - 0.127)	0.118 (0.109 - 0.127)	0.006 (0.005 - 0.007)	0.006 (0.005 - 0.007)	0.006 (0.005 - 0.007)	3.097 (2.107 - 4.235)	3.100 (2.088 - 4.258)	3.086 (2.095 - 4.247)
All ages (rate per capita)	0.016 (0.015 - 0.017)	0.015 (0.014 - 0.016)	0.014 (0.014 - 0.015)	0.001 (0.001 - 0.001)	0.001 (0.001 - 0.001)	0.001 (0.001 - 0.001)	0.450 (0.299 - 0.622)	0.411 (0.272 - 0.567)	0.403 (0.267 - 0.555)
Age-standardized (rate per capita)	0.032 (0.030 - 0.033)	0.032 (0.030 - 0.034)	0.032 (0.030 - 0.033)	0.002 (0.002 - 0.002)	0.002 (0.002 - 0.002)	0.002 (0.002 - 0.002)	0.870 (0.580 - 1.200)	0.875 (0.583 - 1.206)	0.869 (0.580 - 1.191)
Total number of cases (thousands)	96 (91 - 102)	148 (140 - 157)	203 (192 - 215)	5 (5 - 6)	8 (8 - 9)	12 (11 - 12)	3 (2 - 4)	4 (3 - 6)	6 (4 - 8)
**Cote d'Ivoire**									
15 - 49 years (rate per capita)	0.024 (0.023 - 0.026)	0.024 (0.022 - 0.026)	0.023 (0.022 - 0.025)	0.001 (0.001 - 0.001)	0.001 (0.001 - 0.001)	0.001 (0.001 - 0.001)	0.681 (0.454 - 0.951)	0.684 (0.456 - 0.953)	0.665 (0.442 - 0.930)
50 - 69 years (rate per capita)	0.082 (0.076 - 0.088)	0.084 (0.078 - 0.089)	0.083 (0.078 - 0.089)	0.005 (0.004 - 0.006)	0.005 (0.004 - 0.006)	0.005 (0.004 - 0.006)	2.249 (1.501 - 3.118)	2.296 (1.520 - 3.170)	2.299 (1.540 - 3.181)
70+ years (rate per capita)	0.139 (0.129 - 0.150)	0.140 (0.129 - 0.150)	0.140 (0.130 - 0.151)	0.007 (0.006 - 0.008)	0.007 (0.006 - 0.008)	0.007 (0.006 - 0.008)	3.617 (2.436 - 4.968)	3.679 (2.483 - 5.036)	3.707 (2.499 - 5.071)
All ages (rate per capita)	0.020 (0.019 - 0.021)	0.020 (0.019 - 0.022)	0.020 (0.019 - 0.022)	0.001 (0.001 - 0.001)	0.001 (0.001 - 0.001)	0.001 (0.001 - 0.001)	0.556 (0.372 - 0.767)	0.568 (0.379 - 0.785)	0.572 (0.381 - 0.785)

	Prevalence 1990	Prevalence 2005	Prevalence 2015	Incidence 1990	Incidence 2005	Incidence 2015	YLD rate 1990	YLD rate 2005	YLD rate 2015
**Age-standardized (rate per capita)**	0.038 (0.036 - 0.040)	0.038 (0.036 - 0.040)	0.038 (0.036 - 0.040)	0.002 (0.002 - 0.002)	0.002 (0.002 - 0.002)	0.002 (0.002 - 0.002)	1.026 (0.687 - 1.412)	1.042 (0.697 - 1.436)	1.039 (0.696 - 1.424)
**Total number of cases (thousands)**	244 (231 - 257)	369 (349 - 390)	464 (440 - 489)	14 (13 - 14)	20 (19 - 22)	26 (25 - 27)	7 (5 - 9)	10 (7 - 14)	13 (9 - 18)
**The Gambia**									
**15 - 49 years (rate per capita)**	0.014 (0.011 - 0.018)	0.014 (0.011 - 0.018)	0.015 (0.012 - 0.018)	0.001 (0.001 - 0.001)	0.001 (0.001 - 0.001)	0.001 (0.001 - 0.001)	0.408 (0.257 - 0.607)	0.410 (0.258 - 0.608)	0.416 (0.263 - 0.619)
**50 - 69 years (rate per capita)**	0.064 (0.051 - 0.077)	0.062 (0.051 - 0.076)	0.061 (0.050 - 0.074)	0.004 (0.003 - 0.005)	0.004 (0.003 - 0.005)	0.004 (0.003 - 0.005)	1.758 (1.087 - 2.591)	1.727 (1.079 - 2.545)	1.694 (1.059 - 2.509)
**70+ years (rate per capita)**	0.108 (0.089 - 0.133)	0.108 (0.089 - 0.133)	0.107 (0.089 - 0.132)	0.006 (0.004 - 0.008)	0.006 (0.004 - 0.008)	0.006 (0.004 - 0.008)	2.865 (1.776 - 4.135)	2.864 (1.772 - 4.111)	2.855 (1.771 - 4.088)
**All ages (rate per capita)**	0.012 (0.010 - 0.014)	0.012 (0.010 - 0.014)	0.012 (0.010 - 0.014)	0.001 (0.001 - 0.001)	0.001 (0.001 - 0.001)	0.001 (0.001 - 0.001)	0.343 (0.222 - 0.498)	0.339 (0.220 - 0.490)	0.342 (0.221 - 0.498)
**Age-standardized (rate per capita)**	0.027 (0.023 - 0.032)	0.027 (0.023 - 0.031)	0.027 (0.023 - 0.031)	0.002 (0.001 - 0.002)	0.002 (0.001 - 0.002)	0.002 (0.001 - 0.002)	0.750 (0.481 - 1.087)	0.750 (0.480 - 1.083)	0.749 (0.479 - 1.082)
**Total number of cases (thousands)**	11 (9 - 13)	18 (15 - 20)	24 (21 - 28)	1 (1 - 1)	1 (1 - 1)	2 (1 - 2)	0 (0 - 0)	0 (0 - 1)	1 (0 - 1)
**Ghana**									
**15 - 49 years (rate per capita)**	0.012 (0.011 - 0.013)	0.012 (0.011 - 0.013)	0.013 (0.012 - 0.014)	0.001 (0.001 - 0.001)	0.001 (0.001 - 0.001)	0.001 (0.001 - 0.001)	0.338 (0.225 - 0.474)	0.341 (0.227 - 0.478)	0.362 (0.239 - 0.507)
**50 - 69 years (rate per capita)**	0.047 (0.044 - 0.051)	0.047 (0.043 - 0.050)	0.047 (0.043 - 0.050)	0.003 (0.003 - 0.003)	0.003 (0.003 - 0.003)	0.003 (0.003 - 0.003)	1.290 (0.859 - 1.793)	1.286 (0.851 - 1.787)	1.292 (0.854 - 1.800)
**70+ years (rate per capita)**	0.082 (0.075 - 0.089)	0.081 (0.075 - 0.088)	0.081 (0.075 - 0.088)	0.005 (0.004 - 0.005)	0.005 (0.004 - 0.005)	0.004 (0.004 - 0.005)	2.161 (1.459 - 2.959)	2.153 (1.434 - 2.952)	2.155 (1.455 - 2.946)
**All ages (rate per capita)**	0.011 (0.010 - 0.011)	0.011 (0.011 - 0.012)	0.012 (0.011 - 0.013)	0.001 (0.001 - 0.001)	0.001 (0.001 - 0.001)	0.001 (0.001 - 0.001)	0.294 (0.197 - 0.407)	0.321 (0.213 - 0.443)	0.342 (0.227 - 0.473)
**Age-standardized (rate per capita)**	0.021 (0.020 - 0.022)	0.021 (0.019 - 0.022)	0.021 (0.020 - 0.022)	0.001 (0.001 - 0.001)	0.001 (0.001 - 0.001)	0.001 (0.001 - 0.001)	0.573 (0.384 - 0.788)	0.569 (0.379 - 0.784)	0.576 (0.386 - 0.795)
**Total number of cases (thousands)**	155 (146 - 165)	246 (232 - 262)	335 (315 - 356)	9 (9 - 10)	15 (14 - 16)	20 (18 - 21)	4 (3 - 6)	7 (5 - 9)	9 (6 - 13)
**Guinea**									
**15 - 49 years (rate per capita)**	0.015 (0.012 - 0.019)	0.015 (0.012 - 0.018)	0.015 (0.012 - 0.018)	0.001 (0.001 - 0.001)	0.001 (0.001 - 0.001)	0.001 (0.001 - 0.001)	0.434 (0.275 - 0.641)	0.422 (0.266 - 0.623)	0.419 (0.266 - 0.618)
**50 - 69 years (rate per capita)**	0.062 (0.050 - 0.075)	0.062 (0.050 - 0.074)	0.062 (0.050 - 0.074)	0.004 (0.003 - 0.005)	0.004 (0.003 - 0.005)	0.004 (0.003 - 0.005)	1.716 (1.078 - 2.536)	1.701 (1.066 - 2.510)	1.709 (1.053 - 2.513)
**70+ years (rate per capita)**	0.106 (0.087 - 0.130)	0.106 (0.088 - 0.130)	0.106 (0.088 - 0.130)	0.006 (0.004 - 0.008)	0.006 (0.004 - 0.008)	0.006 (0.004 - 0.008)	2.807 (1.740 - 4.042)	2.815 (1.753 - 4.045)	2.820 (1.743 - 4.043)
**All ages (rate per capita)**	0.015 (0.012 - 0.017)	0.014 (0.012 - 0.016)	0.014 (0.012 - 0.016)	0.001 (0.001 - 0.001)	0.001 (0.001 - 0.001)	0.001 (0.001 - 0.001)	0.410 (0.263 - 0.591)	0.390 (0.251 - 0.560)	0.398 (0.256 - 0.573)
**Age-standardized (rate per capita)**	0.027 (0.023 - 0.031)	0.027 (0.023 - 0.031)	0.027 (0.023 - 0.031)	0.002 (0.001 - 0.002)	0.002 (0.001 - 0.002)	0.002 (0.001 - 0.002)	0.738 (0.477 - 1.064)	0.741 (0.473 - 1.069)	0.742 (0.476 - 1.075)
**Total number of cases (thousands)**	89 (75 - 102)	136 (114 - 157)	179 (151 - 207)	5 (5 - 6)	8 (7 - 10)	11 (9 - 13)	2 (2 - 4)	4 (2 - 5)	5 (3 - 7)
**Guinea-Bissau**									
**15 - 49 years (rate per capita)**	0.015 (0.012 - 0.019)	0.015 (0.012 - 0.018)	0.015 (0.012 - 0.019)	0.001 (0.001 - 0.001)	0.001 (0.001 - 0.001)	0.001 (0.001 - 0.001)	0.435 (0.274 - 0.641)	0.415 (0.263 - 0.605)	0.426 (0.272 - 0.629)
**50 - 69 years (rate per capita)**	0.063 (0.051 - 0.077)	0.061 (0.050 - 0.074)	0.062 (0.051 - 0.075)	0.004 (0.003 - 0.005)	0.004 (0.003 - 0.005)	0.004 (0.003 - 0.005)	1.743 (1.091 - 2.578)	1.690 (1.057 - 2.500)	1.713 (1.072 - 2.525)
**70+ years (rate per capita)**	0.106 (0.088 - 0.129)	0.106 (0.088 - 0.129)	0.106 (0.088 - 0.129)	0.006 (0.004 - 0.008)	0.006 (0.004 - 0.008)	0.006 (0.004 - 0.008)	2.806 (1.752 - 4.046)	2.803 (1.755 - 4.040)	2.799 (1.752 - 4.039)
**All ages (rate per capita)**	0.015 (0.012 - 0.017)	0.014 (0.012 - 0.016)	0.015 (0.012 - 0.017)	0.001 (0.001 - 0.001)	0.001 (0.001 - 0.001)	0.001 (0.001 - 0.001)	0.409 (0.265 - 0.584)	0.394 (0.254 - 0.567)	0.409 (0.263 - 0.586)
**Age-standardized (rate per capita)**	0.027 (0.023 - 0.031)	0.027 (0.023 - 0.031)	0.027 (0.023 - 0.031)	0.002 (0.001 - 0.002)	0.002 (0.001 - 0.002)	0.002 (0.001 - 0.002)	0.739 (0.474 - 1.058)	0.740 (0.474 - 1.059)	0.740 (0.474 - 1.067)
**Total number of cases (thousands)**	16 (13 - 18)	21 (17 - 24)	27 (23 - 32)	1 (1 - 1)	1 (1 - 2)	2 (1 - 2)	0 (0 - 1)	1 (0 - 1)	1 (0 - 1)
**Liberia**									
**15 - 49 years (rate per capita)**	0.015 (0.012 - 0.019)	0.015 (0.012 - 0.019)	0.015 (0.012 - 0.019)	0.001 (0.001 - 0.001)	0.001 (0.001 - 0.001)	0.001 (0.001 - 0.001)	0.415 (0.267 - 0.610)	0.415 (0.266 - 0.612)	0.427 (0.273 - 0.631)
**50 - 69 years (rate per capita)**	0.062 (0.050 - 0.075)	0.062 (0.051 - 0.075)	0.061 (0.050 - 0.074)	0.004 (0.003 - 0.005)	0.004 (0.003 - 0.005)	0.004 (0.003 - 0.005)	1.678 (1.053 - 2.467)	1.688 (1.059 - 2.473)	1.672 (1.052 - 2.447)
**70+ years (rate per capita)**	0.107 (0.088 - 0.130)	0.106 (0.087 - 0.130)	0.106 (0.088 - 0.130)	0.006 (0.004 - 0.008)	0.006 (0.004 - 0.008)	0.006 (0.004 - 0.008)	2.755 (1.712 - 3.965)	2.736 (1.698 - 3.963)	2.765 (1.713 - 3.974)
**All ages (rate per capita)**	0.013 (0.011 - 0.016)	0.014 (0.012 - 0.016)	0.014 (0.012 - 0.017)	0.001 (0.001 - 0.001)	0.001 (0.001 - 0.001)	0.001 (0.001 - 0.001)	0.369 (0.240 - 0.530)	0.378 (0.246 - 0.541)	0.392 (0.254 - 0.562)
**Age-standardized (rate per capita)**	0.027 (0.023 - 0.031)	0.027 (0.023 - 0.031)	0.027 (0.023 - 0.031)	0.002 (0.001 - 0.002)	0.002 (0.001 - 0.002)	0.002 (0.001 - 0.002)	0.730 (0.473 - 1.041)	0.727 (0.471 - 1.038)	0.731 (0.471 - 1.050)
**Total number of cases (thousands)**	29 (24 - 33)	45 (38 - 52)	64 (54 - 74)	2 (1 - 2)	3 (2 - 3)	4 (3 - 5)	1 (1 - 1)	1 (1 - 2)	2 (1 - 3)
**Mali**									
**15 - 49 years (rate per capita)**	0.014 (0.013 - 0.015)	0.014 (0.013 - 0.015)	0.014 (0.013 - 0.015)	0.001 (0.001 - 0.001)	0.001 (0.001 - 0.001)	0.001 (0.001 - 0.001)	0.398 (0.263 - 0.557)	0.390 (0.257 - 0.545)	0.405 (0.269 - 0.575)
**50 - 69 years (rate per capita)**	0.056 (0.052 - 0.061)	0.055 (0.051 - 0.059)	0.055 (0.051 - 0.059)	0.003 (0.003 - 0.004)	0.003 (0.003 - 0.004)	0.003 (0.003 - 0.004)	1.530 (1.023 - 2.116)	1.496 (0.995 - 2.064)	1.509 (1.003 - 2.093)
**70+ years (rate per capita)**	0.094 (0.087 - 0.102)	0.093 (0.086 - 0.101)	0.094 (0.086 - 0.102)	0.005 (0.004 - 0.006)	0.005 (0.004 - 0.006)	0.005 (0.004 - 0.006)	2.473 (1.670 - 3.394)	2.451 (1.639 - 3.373)	2.475 (1.668 - 3.400)
**All ages (rate per capita)**	0.013 (0.012 - 0.014)	0.012 (0.011 - 0.012)	0.011 (0.011 - 0.012)	0.001 (0.001 - 0.001)	0.001 (0.001 - 0.001)	0.001 (0.001 - 0.001)	0.354 (0.236 - 0.491)	0.323 (0.214 - 0.447)	0.316 (0.210 - 0.441)
**Age-standardized (rate per capita)**	0.024 (0.023 - 0.026)	0.024 (0.023 - 0.026)	0.024 (0.023 - 0.026)	0.001 (0.001 - 0.001)	0.001 (0.001 - 0.001)	0.001 (0.001 - 0.001)	0.668 (0.449 - 0.921)	0.665 (0.441 - 0.916)	0.673 (0.448 - 0.934)
**Total number of cases (thousands)**	109 (103 - 116)	150 (142 - 159)	199 (187 - 212)	6 (6 - 7)	9 (8 - 9)	12 (11 - 12)	3 (2 - 4)	4 (3 - 6)	6 (4 - 8)
**Mauritania**									
**15 - 49 years (rate per capita)**	0.020 (0.018 - 0.021)	0.021 (0.019 - 0.022)	0.021 (0.020 - 0.023)	0.001 (0.001 - 0.001)	0.001 (0.001 - 0.001)	0.001 (0.001 - 0.001)	0.559 (0.368 - 0.781)	0.589 (0.391 - 0.820)	0.600 (0.395 - 0.841)
**50 - 69 years (rate per capita)**	0.074 (0.069 - 0.080)	0.074 (0.069 - 0.080)	0.073 (0.068 - 0.078)	0.004 (0.004 - 0.005)	0.004 (0.004 - 0.005)	0.004 (0.004 - 0.005)	2.037 (1.363 - 2.832)	2.042 (1.365 - 2.821)	2.016 (1.346 - 2.794)
**70+ years (rate per capita)**	0.124 (0.114 - 0.133)	0.124 (0.115 - 0.134)	0.123 (0.113 - 0.132)	0.006 (0.005 - 0.007)	0.006 (0.005 - 0.007)	0.006 (0.005 - 0.007)	3.246 (2.209 - 4.471)	3.268 (2.198 - 4.498)	3.245 (2.187 - 4.476)
**All ages (rate per capita)**	0.017 (0.016 - 0.018)	0.018 (0.017 - 0.019)	0.019 (0.018 - 0.020)	0.001 (0.001 - 0.001)	0.001 (0.001 - 0.001)	0.001 (0.001 - 0.001)	0.477 (0.315 - 0.658)	0.507 (0.339 - 0.698)	0.535 (0.355 - 0.742)
**Age-standardized (rate per capita)**	0.033 (0.031 - 0.035)	0.033 (0.031 - 0.035)	0.033 (0.031 - 0.035)	0.002 (0.002 - 0.002)	0.002 (0.002 - 0.002)	0.002 (0.002 - 0.002)	0.906 (0.603 - 1.248)	0.914 (0.612 - 1.259)	0.911 (0.610 - 1.257)
**Total number of cases (thousands)**	35 (33 - 37)	57 (54 - 61)	78 (74 - 83)	2 (2 - 2)	3 (3 - 3)	4 (4 - 5)	1 (1 - 1)	2 (1 - 2)	2 (1 - 3)
**Niger**									
**15 - 49 years (rate per capita)**	0.015 (0.012 - 0.019)	0.015 (0.012 - 0.019)	0.014 (0.011 - 0.018)	0.001 (0.001 - 0.001)	0.001 (0.001 - 0.001)	0.001 (0.001 - 0.001)	0.433 (0.275 - 0.644)	0.440 (0.280 - 0.654)	0.411 (0.257 - 0.605)
**50 - 69 years (rate per capita)**	0.061 (0.050 - 0.074)	0.061 (0.050 - 0.074)	0.061 (0.050 - 0.074)	0.004 (0.003 - 0.005)	0.004 (0.003 - 0.005)	0.004 (0.003 - 0.005)	1.693 (1.068 - 2.472)	1.694 (1.054 - 2.494)	1.704 (1.058 - 2.512)
**70+ years (rate per capita)**	0.106 (0.087 - 0.129)	0.106 (0.088 - 0.129)	0.106 (0.087 - 0.129)	0.006 (0.004 - 0.008)	0.006 (0.004 - 0.008)	0.006 (0.004 - 0.008)	2.798 (1.739 - 4.033)	2.811 (1.747 - 4.012)	2.804 (1.734 - 4.019)
**All ages (rate per capita)**	0.013 (0.010 - 0.015)	0.013 (0.011 - 0.015)	0.012 (0.010 - 0.014)	0.001 (0.001 - 0.001)	0.001 (0.001 - 0.001)	0.001 (0.001 - 0.001)	0.350 (0.226 - 0.509)	0.355 (0.228 - 0.513)	0.341 (0.220 - 0.492)
**Age-standardized (rate per capita)**	0.027 (0.023 - 0.031)	0.027 (0.023 - 0.031)	0.027 (0.023 - 0.031)	0.002 (0.001 - 0.002)	0.002 (0.001 - 0.002)	0.002 (0.001 - 0.002)	0.739 (0.475 - 1.057)	0.743 (0.476 - 1.063)	0.745 (0.477 - 1.070)
**Total number of cases (thousands)**	99 (83 - 116)	171 (144 - 198)	242 (203 - 281)	6 (5 - 7)	10 (9 - 12)	15 (13 - 18)	3 (2 - 4)	5 (3 - 7)	7 (4 - 10)
**Nigeria**									
**15 - 49 years (rate per capita)**	0.010 (0.009 - 0.010)	0.009 (0.009 - 0.010)	0.010 (0.009 - 0.010)	0.001 (0.001 - 0.001)	0.001 (0.001 - 0.001)	0.001 (0.001 - 0.001)	0.274 (0.183 - 0.384)	0.270 (0.180 - 0.376)	0.276 (0.184 - 0.386)
**50 - 69 years (rate per capita)**	0.038 (0.036 - 0.041)	0.038 (0.036 - 0.041)	0.038 (0.036 - 0.041)	0.002 (0.002 - 0.003)	0.002 (0.002 - 0.003)	0.002 (0.002 - 0.003)	1.056 (0.701 - 1.475)	1.054 (0.709 - 1.474)	1.061 (0.710 - 1.471)
**70+ years (rate per capita)**	0.067 (0.062 - 0.073)	0.067 (0.062 - 0.072)	0.068 (0.062 - 0.073)	0.004 (0.003 - 0.005)	0.004 (0.003 - 0.005)	0.004 (0.003 - 0.005)	1.777 (1.202 - 2.457)	1.766 (1.184 - 2.457)	1.791 (1.196 - 2.461)
**All ages (rate per capita)**	0.009 (0.008 - 0.009)	0.009 (0.008 - 0.009)	0.009 (0.008 - 0.009)	0.001 (0.000 - 0.001)	0.001 (0.000 - 0.001)	0.001 (0.000 - 0.001)	0.241 (0.162 - 0.335)	0.239 (0.159 - 0.331)	0.240 (0.162 - 0.332)
**Age-standardized (rate per capita)**	0.017 (0.016 - 0.018)	0.017 (0.016 - 0.018)	0.017 (0.016 - 0.018)	0.001 (0.001 - 0.001)	0.001 (0.001 - 0.001)	0.001 (0.001 - 0.001)	0.465 (0.311 - 0.641)	0.463 (0.309 - 0.641)	0.467 (0.313 - 0.645)
**Total number of cases (thousands)**	831 (786 - 882)	1,198 (1,133 - 1,275)	1,566 (1,479 - 1,660)	50 (47 - 54)	73 (69 - 78)	95 (89 - 101)	23 (16 - 32)	33 (22 - 46)	44 (29 - 61)
**Sao Tome and Principe**									
**15 - 49 years (rate per capita)**	0.013 (0.010 - 0.016)	0.014 (0.011 - 0.018)	0.015 (0.012 - 0.019)	0.001 (0.001 - 0.001)	0.001 (0.001 - 0.001)	0.001 (0.001 - 0.001)	0.371 (0.235 - 0.545)	0.399 (0.254 - 0.598)	0.430 (0.275 - 0.635)
**50 - 69 years (rate per capita)**	0.064 (0.052 - 0.077)	0.065 (0.053 - 0.077)	0.060 (0.049 - 0.072)	0.004 (0.003 - 0.005)	0.004 (0.003 - 0.005)	0.004 (0.003 - 0.005)	1.768 (1.092 - 2.601)	1.786 (1.124 - 2.614)	1.659 (1.042 - 2.442)
**70+ years (rate per capita)**	0.106 (0.087 - 0.128)	0.106 (0.088 - 0.129)	0.106 (0.088 - 0.128)	0.005 (0.004 - 0.007)	0.005 (0.004 - 0.007)	0.005 (0.004 - 0.007)	2.803 (1.759 - 4.035)	2.811 (1.748 - 4.023)	2.802 (1.769 - 4.020)
**All ages (rate per capita)**	0.014 (0.012 - 0.017)	0.014 (0.012 - 0.016)	0.014 (0.012 - 0.016)	0.001 (0.001 - 0.001)	0.001 (0.001 - 0.001)	0.001 (0.001 - 0.001)	0.398 (0.257 - 0.571)	0.379 (0.244 - 0.547)	0.385 (0.250 - 0.554)
**Age-standardized (rate per capita)**	0.027 (0.023 - 0.031)	0.027 (0.023 - 0.031)	0.027 (0.023 - 0.031)	0.002 (0.001 - 0.002)	0.002 (0.001 - 0.002)	0.002 (0.001 - 0.002)	0.747 (0.478 - 1.084)	0.745 (0.479 - 1.070)	0.744 (0.476 - 1.070)
**Total number of cases (thousands)**	2 (1 - 2)	2 (2 - 2)	3 (2 - 3)	0 (0 - 0)	0 (0 - 0)	0 (0 - 0)	0 (0 - 0)	0 (0 - 0)	0 (0 - 0)
**Senegal**									
**15 - 49 years (rate per capita)**	0.019 (0.018 - 0.021)	0.020 (0.018 - 0.021)	0.020 (0.019 - 0.022)	0.001 (0.001 - 0.001)	0.001 (0.001 - 0.001)	0.001 (0.001 - 0.001)	0.552 (0.367 - 0.776)	0.561 (0.371 - 0.785)	0.577 (0.385 - 0.807)
**50 - 69 years (rate per capita)**	0.072 (0.067 - 0.077)	0.073 (0.068 - 0.078)	0.071 (0.066 - 0.077)	0.004 (0.004 - 0.005)	0.004 (0.004 - 0.005)	0.004 (0.004 - 0.005)	1.998 (1.335 - 2.779)	2.025 (1.351 - 2.815)	1.985 (1.322 - 2.756)

	Prevalence 1990	Prevalence 2005	Prevalence 2015	Incidence 1990	Incidence 2005	Incidence 2015	YLD rate 1990	YLD rate 2005	YLD rate 2015
70+ years (rate per capita)	0.121 (0.111 - 0.132)	0.121 (0.112 - 0.131)	0.121 (0.111 - 0.131)	0.006 (0.005 - 0.007)	0.006 (0.005 - 0.007)	0.006 (0.005 - 0.007)	3.254 (2.185 - 4.470)	3.238 (2.169 - 4.468)	3.229 (2.167 - 4.448)
All ages (rate per capita)	0.016 (0.015 - 0.017)	0.017 (0.016 - 0.018)	0.017 (0.016 - 0.018)	0.001 (0.001 - 0.001)	0.001 (0.001 - 0.001)	0.001 (0.001 - 0.001)	0.458 (0.306 - 0.637)	0.471 (0.314 - 0.650)	0.476 (0.319 - 0.658)
Age-standardized (rate per capita)	0.032 (0.031 - 0.034)	0.033 (0.031 - 0.035)	0.032 (0.031 - 0.034)	0.002 (0.002 - 0.002)	0.002 (0.002 - 0.002)	0.002 (0.002 - 0.002)	0.901 (0.605 - 1.245)	0.908 (0.608 - 1.250)	0.901 (0.608 - 1.246)
Total number of cases (thousands)	122 (116 - 130)	189 (179 - 201)	255 (241 - 271)	7 (7 - 7)	11 (10 - 11)	14 (13 - 15)	3 (2 - 5)	5 (4 - 7)	7 (5 - 10)
**Sierra Leone**									
15 - 49 years (rate per capita)	0.015 (0.012 - 0.019)	0.014 (0.011 - 0.018)	0.015 (0.012 - 0.018)	0.001 (0.001 - 0.001)	0.001 (0.001 - 0.001)	0.001 (0.001 - 0.001)	0.435 (0.275 - 0.635)	0.408 (0.260 - 0.599)	0.422 (0.268 - 0.621)
50 - 69 years (rate per capita)	0.062 (0.050 - 0.076)	0.062 (0.050 - 0.075)	0.062 (0.050 - 0.075)	0.004 (0.003 - 0.005)	0.004 (0.003 - 0.005)	0.004 (0.003 - 0.005)	1.700 (1.051 - 2.478)	1.691 (1.058 - 2.479)	1.705 (1.065 - 2.510)
70+ years (rate per capita)	0.107 (0.088 - 0.132)	0.107 (0.088 - 0.131)	0.107 (0.088 - 0.131)	0.006 (0.004 - 0.008)	0.006 (0.004 - 0.008)	0.006 (0.004 - 0.008)	2.816 (1.741 - 4.079)	2.789 (1.725 - 4.021)	2.817 (1.747 - 4.080)
All ages (rate per capita)	0.015 (0.012 - 0.017)	0.013 (0.011 - 0.016)	0.014 (0.011 - 0.016)	0.001 (0.001 - 0.001)	0.001 (0.001 - 0.001)	0.001 (0.001 - 0.001)	0.402 (0.260 - 0.573)	0.368 (0.239 - 0.529)	0.379 (0.245 - 0.544)
Age-standardized (rate per capita)	0.027 (0.023 - 0.031)	0.027 (0.023 - 0.031)	0.027 (0.023 - 0.031)	0.002 (0.001 - 0.002)	0.002 (0.001 - 0.002)	0.002 (0.001 - 0.002)	0.739 (0.476 - 1.051)	0.734 (0.472 - 1.048)	0.741 (0.470 - 1.060)
Total number of cases (thousands)	57 (48 - 66)	67 (56 - 78)	88 (73 - 103)	3 (3 - 4)	4 (4 - 5)	5 (5 - 6)	2 (1 - 2)	2 (1 - 3)	2 (2 - 4)
**Togo**									
15 - 49 years (rate per capita)	0.014 (0.011 - 0.018)	0.014 (0.011 - 0.018)	0.015 (0.012 - 0.019)	0.001 (0.001 - 0.001)	0.001 (0.001 - 0.001)	0.001 (0.001 - 0.001)	0.412 (0.264 - 0.606)	0.406 (0.258 - 0.598)	0.432 (0.272 - 0.630)
50 - 69 years (rate per capita)	0.062 (0.050 - 0.075)	0.062 (0.050 - 0.075)	0.062 (0.050 - 0.074)	0.004 (0.003 - 0.005)	0.004 (0.003 - 0.005)	0.004 (0.003 - 0.005)	1.710 (1.068 - 2.500)	1.704 (1.062 - 2.470)	1.704 (1.073 - 2.502)
70+ years (rate per capita)	0.106 (0.088 - 0.129)	0.106 (0.088 - 0.130)	0.106 (0.088 - 0.130)	0.006 (0.004 - 0.008)	0.006 (0.004 - 0.008)	0.006 (0.004 - 0.008)	2.806 (1.738 - 4.015)	2.806 (1.749 - 4.012)	2.816 (1.757 - 4.034)
All ages (rate per capita)	0.013 (0.011 - 0.015)	0.013 (0.011 - 0.015)	0.014 (0.011 - 0.016)	0.001 (0.001 - 0.001)	0.001 (0.001 - 0.001)	0.001 (0.001 - 0.001)	0.366 (0.236 - 0.529)	0.367 (0.237 - 0.525)	0.386 (0.249 - 0.556)
Age-standardized (rate per capita)	0.027 (0.023 - 0.031)	0.027 (0.023 - 0.031)	0.027 (0.023 - 0.031)	0.002 (0.001 - 0.002)	0.002 (0.001 - 0.002)	0.002 (0.001 - 0.002)	0.740 (0.474 - 1.066)	0.740 (0.478 - 1.053)	0.743 (0.475 - 1.070)
Total number of cases (thousands)	50 (42 - 58)	73 (61 - 85)	100 (84 - 117)	3 (3 - 4)	5 (4 - 5)	6 (5 - 7)	1 (1 - 2)	2 (1 - 3)	3 (2 - 4)
**Eastern Sub-Saharan Africa**									
15 - 49 years (rate per capita)	0.009 (0.008 - 0.011)	0.009 (0.008 - 0.010)	0.009 (0.008 - 0.010)	0.001 (0.001 - 0.001)	0.001 (0.000 - 0.001)	0.001 (0.001 - 0.001)	0.263 (0.174 - 0.373)	0.253 (0.168 - 0.359)	0.260 (0.172 - 0.373)
50 - 69 years (rate per capita)	0.039 (0.035 - 0.044)	0.039 (0.035 - 0.044)	0.039 (0.035 - 0.044)	0.002 (0.002 - 0.003)	0.002 (0.002 - 0.003)	0.002 (0.002 - 0.003)	1.089 (0.701 - 1.547)	1.083 (0.700 - 1.531)	1.089 (0.702 - 1.543)
70+ years (rate per capita)	0.068 (0.061 - 0.077)	0.068 (0.061 - 0.077)	0.069 (0.061 - 0.078)	0.004 (0.003 - 0.005)	0.004 (0.003 - 0.005)	0.004 (0.003 - 0.005)	1.820 (1.196 - 2.533)	1.820 (1.193 - 2.527)	1.839 (1.207 - 2.561)
All ages (rate per capita)	0.008 (0.007 - 0.009)	0.008 (0.007 - 0.009)	0.008 (0.008 - 0.009)	0.001 (0.000 - 0.001)	0.001 (0.000 - 0.001)	0.001 (0.000 - 0.001)	0.228 (0.149 - 0.319)	0.226 (0.148 - 0.316)	0.237 (0.155 - 0.332)
Age-standardized (rate per capita)	0.017 (0.016 - 0.019)	0.017 (0.016 - 0.019)	0.017 (0.016 - 0.019)	0.001 (0.001 - 0.001)	0.001 (0.001 - 0.001)	0.001 (0.001 - 0.001)	0.474 (0.310 - 0.662)	0.471 (0.308 - 0.658)	0.473 (0.308 - 0.663)
Total number of cases (thousands)	1,519 (1,372 - 1,672)	2,288 (2,070 - 2,515)	3,190 (2,884 - 3,515)	94 (85 - 104)	142 (130 - 157)	197 (179 - 217)	42 (28 - 59)	64 (42 - 89)	89 (58 - 125)
**Burundi**									
15 - 49 years (rate per capita)	0.010 (0.008 - 0.012)	0.009 (0.008 - 0.011)	0.010 (0.008 - 0.012)	0.001 (0.001 - 0.001)	0.001 (0.001 - 0.001)	0.001 (0.001 - 0.001)	0.286 (0.182 - 0.419)	0.264 (0.170 - 0.387)	0.281 (0.180 - 0.411)
50 - 69 years (rate per capita)	0.043 (0.036 - 0.052)	0.042 (0.034 - 0.051)	0.042 (0.034 - 0.051)	0.003 (0.002 - 0.004)	0.003 (0.002 - 0.003)	0.003 (0.002 - 0.003)	1.198 (0.757 - 1.755)	1.166 (0.735 - 1.716)	1.177 (0.744 - 1.731)
70+ years (rate per capita)	0.075 (0.063 - 0.091)	0.075 (0.063 - 0.091)	0.075 (0.063 - 0.091)	0.004 (0.003 - 0.005)	0.004 (0.003 - 0.005)	0.004 (0.003 - 0.005)	2.016 (1.269 - 2.894)	2.017 (1.274 - 2.909)	2.025 (1.280 - 2.895)
All ages (rate per capita)	0.009 (0.007 - 0.010)	0.008 (0.007 - 0.010)	0.009 (0.007 - 0.010)	0.001 (0.000 - 0.001)	0.001 (0.000 - 0.001)	0.001 (0.000 - 0.001)	0.245 (0.159 - 0.351)	0.236 (0.153 - 0.340)	0.249 (0.161 - 0.358)
Age-standardized (rate per capita)	0.019 (0.016 - 0.021)	0.019 (0.016 - 0.021)	0.019 (0.016 - 0.021)	0.001 (0.001 - 0.001)	0.001 (0.001 - 0.001)	0.001 (0.001 - 0.001)	0.515 (0.331 - 0.737)	0.515 (0.333 - 0.734)	0.518 (0.334 - 0.744)
Total number of cases (thousands)	49 (42 - 57)	67 (57 - 78)	99 (84 - 115)	3 (3 - 4)	4 (4 - 5)	6 (5 - 7)	1 (1 - 2)	2 (1 - 3)	3 (2 - 4)
**Comoros**									
15 - 49 years (rate per capita)	0.013 (0.012 - 0.014)	0.013 (0.012 - 0.014)	0.013 (0.012 - 0.014)	0.001 (0.001 - 0.001)	0.001 (0.001 - 0.001)	0.001 (0.001 - 0.001)	0.361 (0.238 - 0.505)	0.366 (0.243 - 0.518)	0.380 (0.251 - 0.534)
50 - 69 years (rate per capita)	0.050 (0.046 - 0.053)	0.049 (0.046 - 0.053)	0.048 (0.045 - 0.052)	0.003 (0.003 - 0.003)	0.003 (0.003 - 0.003)	0.003 (0.003 - 0.003)	1.379 (0.915 - 1.932)	1.366 (0.903 - 1.901)	1.351 (0.890 - 1.881)
70+ years (rate per capita)	0.086 (0.080 - 0.093)	0.086 (0.080 - 0.092)	0.086 (0.080 - 0.092)	0.005 (0.004 - 0.005)	0.005 (0.004 - 0.005)	0.005 (0.004 - 0.005)	2.297 (1.535 - 3.180)	2.295 (1.531 - 3.168)	2.293 (1.523 - 3.188)
All ages (rate per capita)	0.011 (0.010 - 0.012)	0.011 (0.011 - 0.012)	0.012 (0.011 - 0.013)	0.001 (0.001 - 0.001)	0.001 (0.001 - 0.001)	0.001 (0.001 - 0.001)	0.307 (0.204 - 0.424)	0.317 (0.212 - 0.440)	0.340 (0.226 - 0.472)
Age-standardized (rate per capita)	0.022 (0.021 - 0.023)	0.022 (0.021 - 0.023)	0.022 (0.021 - 0.023)	0.001 (0.001 - 0.001)	0.001 (0.001 - 0.001)	0.001 (0.001 - 0.001)	0.612 (0.407 - 0.844)	0.613 (0.409 - 0.851)	0.613 (0.408 - 0.847)
Total number of cases (thousands)	5 (4 - 5)	7 (7 - 7)	10 (9 - 10)	0 (0 - 0)	0 (0 - 0)	1 (1 - 1)	0 (0 - 0)	0 (0 - 0)	0 (0 - 0)
**Djibouti**									
15 - 49 years (rate per capita)	0.010 (0.008 - 0.012)	0.010 (0.008 - 0.012)	0.011 (0.009 - 0.013)	0.001 (0.001 - 0.001)	0.001 (0.001 - 0.001)	0.001 (0.001 - 0.001)	0.280 (0.179 - 0.411)	0.285 (0.183 - 0.414)	0.306 (0.196 - 0.445)
50 - 69 years (rate per capita)	0.042 (0.035 - 0.051)	0.043 (0.035 - 0.052)	0.042 (0.035 - 0.051)	0.003 (0.002 - 0.003)	0.003 (0.002 - 0.004)	0.003 (0.002 - 0.003)	1.182 (0.745 - 1.737)	1.194 (0.750 - 1.745)	1.173 (0.738 - 1.717)
70+ years (rate per capita)	0.075 (0.063 - 0.091)	0.075 (0.063 - 0.091)	0.075 (0.063 - 0.091)	0.004 (0.003 - 0.006)	0.004 (0.003 - 0.005)	0.004 (0.003 - 0.005)	2.017 (1.271 - 2.900)	2.005 (1.259 - 2.911)	1.998 (1.256 - 2.848)
All ages (rate per capita)	0.009 (0.008 - 0.011)	0.010 (0.009 - 0.012)	0.012 (0.010 - 0.014)	0.001 (0.000 - 0.001)	0.001 (0.001 - 0.001)	0.001 (0.001 - 0.001)	0.254 (0.164 - 0.364)	0.289 (0.185 - 0.412)	0.339 (0.220 - 0.485)
Age-standardized (rate per capita)	0.019 (0.016 - 0.022)	0.019 (0.016 - 0.022)	0.019 (0.016 - 0.022)	0.001 (0.001 - 0.001)	0.001 (0.001 - 0.001)	0.001 (0.001 - 0.001)	0.516 (0.333 - 0.738)	0.514 (0.331 - 0.730)	0.513 (0.331 - 0.728)
Total number of cases (thousands)	5 (4 - 6)	8 (7 - 9)	11 (9 - 13)	0 (0 - 0)	0 (0 - 1)	1 (1 - 1)	0 (0 - 0)	0 (0 - 0)	0 (0 - 0)
**Eritrea**									
15 - 49 years (rate per capita)	0.010 (0.008 - 0.012)	0.009 (0.007 - 0.011)	0.010 (0.008 - 0.012)	0.001 (0.001 - 0.001)	0.001 (0.001 - 0.001)	0.001 (0.001 - 0.001)	0.280 (0.183 - 0.412)	0.251 (0.162 - 0.367)	0.282 (0.182 - 0.414)
50 - 69 years (rate per capita)	0.041 (0.034 - 0.050)	0.042 (0.035 - 0.051)	0.043 (0.035 - 0.052)	0.003 (0.002 - 0.003)	0.003 (0.002 - 0.003)	0.003 (0.002 - 0.004)	1.150 (0.729 - 1.682)	1.168 (0.735 - 1.715)	1.190 (0.751 - 1.750)
70+ years (rate per capita)	0.076 (0.063 - 0.091)	0.076 (0.063 - 0.091)	0.075 (0.063 - 0.090)	0.004 (0.003 - 0.006)	0.004 (0.003 - 0.006)	0.004 (0.003 - 0.006)	2.020 (1.266 - 2.906)	2.013 (1.254 - 2.869)	2.011 (1.266 - 2.880)
All ages (rate per capita)	0.008 (0.007 - 0.009)	0.008 (0.007 - 0.010)	0.009 (0.008 - 0.010)	0.000 (0.000 - 0.001)	0.001 (0.000 - 0.001)	0.001 (0.000 - 0.001)	0.219 (0.144 - 0.314)	0.232 (0.152 - 0.332)	0.250 (0.162 - 0.359)
Age-standardized (rate per capita)	0.019 (0.016 - 0.021)	0.019 (0.016 - 0.021)	0.019 (0.016 - 0.021)	0.001 (0.001 - 0.001)	0.001 (0.001 - 0.001)	0.001 (0.001 - 0.001)	0.514 (0.332 - 0.737)	0.512 (0.330 - 0.727)	0.512 (0.329 - 0.736)
Total number of cases (thousands)	25 (21 - 29)	35 (30 - 41)	47 (40 - 55)	2 (1 - 2)	2 (2 - 3)	3 (3 - 3)	1 (0 - 1)	1 (1 - 1)	1 (1 - 2)
**Ethiopia**									
15 - 49 years (rate per capita)	0.009 (0.008 - 0.010)	0.009 (0.008 - 0.009)	0.009 (0.008 - 0.009)	0.001 (0.001 - 0.001)	0.001 (0.000 - 0.001)	0.001 (0.000 - 0.001)	0.258 (0.171 - 0.358)	0.246 (0.161 - 0.344)	0.248 (0.163 - 0.350)
50 - 69 years (rate per capita)	0.036 (0.034 - 0.039)	0.036 (0.034 - 0.039)	0.037 (0.035 - 0.040)	0.002 (0.002 - 0.003)	0.002 (0.002 - 0.003)	0.002 (0.002 - 0.003)	1.013 (0.674 - 1.406)	1.011 (0.668 - 1.399)	1.028 (0.674 - 1.420)
70+ years (rate per capita)	0.064 (0.060 - 0.069)	0.064 (0.059 - 0.068)	0.064 (0.060 - 0.069)	0.004 (0.003 - 0.004)	0.004 (0.003 - 0.004)	0.004 (0.003 - 0.004)	1.716 (1.150 - 2.370)	1.706 (1.149 - 2.359)	1.717 (1.143 - 2.367)
All ages (rate per capita)	0.008 (0.008 - 0.009)	0.008 (0.007 - 0.008)	0.008 (0.008 - 0.009)	0.000 (0.000 - 0.001)	0.000 (0.000 - 0.001)	0.001 (0.000 - 0.001)	0.228 (0.151 - 0.313)	0.220 (0.146 - 0.304)	0.238 (0.157 - 0.330)
Age-standardized (rate per capita)	0.016 (0.015 - 0.017)	0.016 (0.015 - 0.017)	0.016 (0.015 - 0.017)	0.001 (0.001 - 0.001)	0.001 (0.001 - 0.001)	0.001 (0.001 - 0.001)	0.445 (0.296 - 0.610)	0.443 (0.295 - 0.613)	0.447 (0.297 - 0.619)
Total number of cases (thousands)	391 (370 - 413)	599 (568 - 634)	841 (798 - 888)	24 (23 - 25)	37 (35 - 39)	52 (49 - 55)	11 (7 - 15)	17 (11 - 23)	24 (16 - 33)
**Kenya**									
15 - 49 years (rate per capita)	0.006 (0.006 - 0.006)	0.006 (0.006 - 0.006)	0.006 (0.006 - 0.007)	0.000 (0.000 - 0.000)	0.000 (0.000 - 0.000)	0.000 (0.000 - 0.000)	0.172 (0.115 - 0.242)	0.172 (0.114 - 0.240)	0.185 (0.124 - 0.260)
50 - 69 years (rate per capita)	0.025 (0.024 - 0.026)	0.025 (0.024 - 0.026)	0.025 (0.024 - 0.027)	0.002 (0.001 - 0.002)	0.002 (0.001 - 0.002)	0.002 (0.001 - 0.002)	0.700 (0.469 - 0.966)	0.691 (0.465 - 0.956)	0.711 (0.476 - 0.979)
70+ years (rate per capita)	0.044 (0.042 - 0.046)	0.045 (0.043 - 0.047)	0.045 (0.043 - 0.048)	0.002 (0.002 - 0.003)	0.003 (0.002 - 0.003)	0.002 (0.002 - 0.003)	1.186 (0.801 - 1.629)	1.208 (0.819 - 1.657)	1.223 (0.829 - 1.683)
All ages (rate per capita)	0.005 (0.005 - 0.005)	0.005 (0.005 - 0.006)	0.006 (0.006 - 0.006)	0.000 (0.000 - 0.000)	0.000 (0.000 - 0.000)	0.000 (0.000 - 0.000)	0.139 (0.093 - 0.191)	0.151 (0.101 - 0.208)	0.164 (0.110 - 0.227)
Age-standardized (rate per capita)	0.011 (0.011 - 0.012)	0.011 (0.011 - 0.012)	0.011 (0.011 - 0.012)	0.001 (0.001 - 0.001)	0.001 (0.001 - 0.001)	0.001 (0.001 - 0.001)	0.312 (0.210 - 0.429)	0.313 (0.211 - 0.430)	0.315 (0.212 - 0.434)
Total number of cases (thousands)	116 (111 - 121)	190 (183 - 198)	269 (259 - 281)	7 (7 - 7)	11 (11 - 12)	16 (15 - 16)	3 (2 - 4)	5 (4 - 7)	8 (5 - 10)
**Madagascar**									
15 - 49 years (rate per capita)	0.010 (0.008 - 0.012)	0.010 (0.008 - 0.012)	0.010 (0.008 - 0.012)	0.001 (0.001 - 0.001)	0.001 (0.001 - 0.001)	0.001 (0.001 - 0.001)	0.270 (0.173 - 0.392)	0.283 (0.181 - 0.413)	0.284 (0.182 - 0.414)
50 - 69 years (rate per capita)	0.043 (0.035 - 0.052)	0.042 (0.035 - 0.051)	0.042 (0.035 - 0.051)	0.003 (0.002 - 0.004)	0.003 (0.002 - 0.003)	0.003 (0.002 - 0.003)	1.180 (0.737 - 1.722)	1.170 (0.739 - 1.708)	1.173 (0.733 - 1.713)
70+ years (rate per capita)	0.075 (0.062 - 0.090)	0.075 (0.063 - 0.090)	0.075 (0.063 - 0.091)	0.004 (0.003 - 0.005)	0.004 (0.003 - 0.006)	0.004 (0.003 - 0.005)	1.975 (1.249 - 2.829)	1.994 (1.251 - 2.864)	2.008 (1.276 - 2.911)
All ages (rate per capita)	0.009 (0.008 - 0.010)	0.009 (0.008 - 0.010)	0.010 (0.008 - 0.011)	0.001 (0.000 - 0.001)	0.001 (0.000 - 0.001)	0.001 (0.001 - 0.001)	0.251 (0.163 - 0.358)	0.252 (0.163 - 0.362)	0.269 (0.173 - 0.385)
Age-standardized (rate per capita)	0.019 (0.016 - 0.021)	0.019 (0.016 - 0.021)	0.019 (0.016 - 0.021)	0.001 (0.001 - 0.001)	0.001 (0.001 - 0.001)	0.001 (0.001 - 0.001)	0.509 (0.327 - 0.733)	0.511 (0.329 - 0.735)	0.514 (0.331 - 0.742)
Total number of cases (thousands)	104 (89 - 120)	165 (141 - 191)	232 (198 - 268)	7 (6 - 8)	10 (9 - 12)	14 (12 - 17)	3 (2 - 4)	5 (3 - 7)	7 (4 - 9)
**Malawi**									

	Prevalence 1990	Prevalence 2005	Prevalence 2015	Incidence 1990	Incidence 2005	Incidence 2015	YLD rate 1990	YLD rate 2005	YLD rate 2015
15 - 49 years (rate per capita)	0.012 (0.011 - 0.013)	0.011 (0.010 - 0.012)	0.011 (0.011 - 0.012)	0.001 (0.001 - 0.001)	0.001 (0.001 - 0.001)	0.001 (0.001 - 0.001)	0.339 (0.225 - 0.477)	0.312 (0.206 - 0.437)	0.323 (0.212 - 0.457)
50 - 69 years (rate per capita)	0.047 (0.043 - 0.050)	0.047 (0.044 - 0.051)	0.048 (0.045 - 0.052)	0.003 (0.002 - 0.003)	0.003 (0.003 - 0.003)	0.003 (0.003 - 0.003)	1.289 (0.858 - 1.780)	1.306 (0.873 - 1.826)	1.340 (0.879 - 1.869)
70+ years (rate per capita)	0.080 (0.075 - 0.086)	0.080 (0.074 - 0.086)	0.080 (0.074 - 0.085)	0.005 (0.004 - 0.005)	0.004 (0.004 - 0.005)	0.004 (0.004 - 0.005)	2.142 (1.447 - 2.960)	2.125 (1.420 - 2.944)	2.141 (1.441 - 2.948)
All ages (rate per capita)	0.010 (0.009 - 0.011)	0.010 (0.009 - 0.010)	0.010 (0.009 - 0.011)	0.001 (0.001 - 0.001)	0.001 (0.001 - 0.001)	0.001 (0.001 - 0.001)	0.279 (0.185 - 0.386)	0.272 (0.181 - 0.376)	0.281 (0.187 - 0.390)
Age-standardized (rate per capita)	0.021 (0.020 - 0.022)	0.021 (0.020 - 0.022)	0.021 (0.020 - 0.022)	0.001 (0.001 - 0.001)	0.001 (0.001 - 0.001)	0.001 (0.001 - 0.001)	0.570 (0.380 - 0.786)	0.571 (0.383 - 0.787)	0.573 (0.383 - 0.793)
Total number of cases (thousands)	94 (89 - 99)	125 (119 - 132)	172 (163 - 182)	6 (5 - 6)	8 (7 - 8)	10 (10 - 11)	3 (2 - 4)	3 (2 - 5)	5 (3 - 7)
**Mozambique**									
15 - 49 years (rate per capita)	0.010 (0.008 - 0.012)	0.010 (0.008 - 0.012)	0.010 (0.008 - 0.012)	0.001 (0.001 - 0.001)	0.001 (0.001 - 0.001)	0.001 (0.001 - 0.001)	0.277 (0.179 - 0.402)	0.278 (0.180 - 0.401)	0.270 (0.173 - 0.395)
50 - 69 years (rate per capita)	0.043 (0.036 - 0.052)	0.043 (0.036 - 0.052)	0.043 (0.036 - 0.053)	0.003 (0.002 - 0.004)	0.003 (0.002 - 0.004)	0.003 (0.002 - 0.004)	1.188 (0.751 - 1.740)	1.183 (0.744 - 1.725)	1.192 (0.757 - 1.762)
70+ years (rate per capita)	0.075 (0.063 - 0.091)	0.075 (0.063 - 0.091)	0.075 (0.063 - 0.090)	0.004 (0.003 - 0.006)	0.004 (0.003 - 0.006)	0.004 (0.003 - 0.005)	1.985 (1.262 - 2.861)	1.981 (1.260 - 2.828)	1.982 (1.253 - 2.826)
All ages (rate per capita)	0.009 (0.008 - 0.011)	0.009 (0.008 - 0.011)	0.009 (0.008 - 0.011)	0.001 (0.000 - 0.001)	0.001 (0.000 - 0.001)	0.001 (0.000 - 0.001)	0.253 (0.163 - 0.364)	0.257 (0.166 - 0.368)	0.254 (0.165 - 0.363)
Age-standardized (rate per capita)	0.019 (0.016 - 0.022)	0.019 (0.016 - 0.022)	0.019 (0.016 - 0.022)	0.001 (0.001 - 0.001)	0.001 (0.001 - 0.001)	0.001 (0.001 - 0.001)	0.513 (0.330 - 0.740)	0.511 (0.328 - 0.735)	0.511 (0.331 - 0.739)
Total number of cases (thousands)	123 (106 - 143)	197 (168 - 229)	257 (220 - 300)	8 (7 - 9)	12 (11 - 14)	16 (14 - 19)	3 (2 - 5)	5 (4 - 8)	7 (5 - 10)
**Rwanda**									
15 - 49 years (rate per capita)	0.010 (0.008 - 0.012)	0.009 (0.008 - 0.011)	0.010 (0.008 - 0.012)	0.001 (0.001 - 0.001)	0.001 (0.001 - 0.001)	0.001 (0.001 - 0.001)	0.273 (0.175 - 0.402)	0.264 (0.168 - 0.384)	0.286 (0.182 - 0.423)
50 - 69 years (rate per capita)	0.043 (0.035 - 0.052)	0.042 (0.035 - 0.051)	0.042 (0.035 - 0.051)	0.003 (0.002 - 0.004)	0.003 (0.002 - 0.003)	0.003 (0.002 - 0.003)	1.203 (0.751 - 1.773)	1.164 (0.733 - 1.690)	1.178 (0.749 - 1.731)
70+ years (rate per capita)	0.075 (0.063 - 0.090)	0.074 (0.062 - 0.089)	0.075 (0.063 - 0.090)	0.004 (0.003 - 0.005)	0.004 (0.003 - 0.005)	0.004 (0.003 - 0.005)	2.031 (1.265 - 2.935)	1.981 (1.260 - 2.870)	2.009 (1.269 - 2.899)
All ages (rate per capita)	0.008 (0.007 - 0.009)	0.009 (0.008 - 0.010)	0.010 (0.008 - 0.011)	0.000 (0.000 - 0.001)	0.001 (0.000 - 0.001)	0.001 (0.001 - 0.001)	0.220 (0.142 - 0.315)	0.244 (0.157 - 0.348)	0.270 (0.174 - 0.388)
Age-standardized (rate per capita)	0.019 (0.016 - 0.022)	0.019 (0.016 - 0.022)	0.019 (0.016 - 0.022)	0.001 (0.001 - 0.001)	0.001 (0.001 - 0.001)	0.001 (0.001 - 0.001)	0.518 (0.333 - 0.750)	0.518 (0.332 - 0.738)	0.521 (0.336 - 0.742)
Total number of cases (thousands)	57 (48 - 66)	79 (68 - 92)	112 (96 - 130)	4 (3 - 4)	5 (4 - 6)	7 (6 - 8)	2 (1 - 2)	2 (1 - 3)	3 (2 - 5)
**Somalia**									
15 - 49 years (rate per capita)	0.010 (0.008 - 0.012)	0.010 (0.008 - 0.012)	0.009 (0.008 - 0.012)	0.001 (0.001 - 0.001)	0.001 (0.001 - 0.001)	0.001 (0.001 - 0.001)	0.285 (0.184 - 0.422)	0.280 (0.180 - 0.410)	0.270 (0.174 - 0.394)
50 - 69 years (rate per capita)	0.043 (0.035 - 0.052)	0.043 (0.035 - 0.052)	0.043 (0.035 - 0.052)	0.003 (0.002 - 0.004)	0.003 (0.002 - 0.004)	0.003 (0.002 - 0.004)	1.185 (0.748 - 1.739)	1.181 (0.745 - 1.732)	1.184 (0.747 - 1.729)
70+ years (rate per capita)	0.075 (0.063 - 0.091)	0.075 (0.063 - 0.091)	0.076 (0.063 - 0.091)	0.004 (0.003 - 0.006)	0.004 (0.003 - 0.006)	0.004 (0.003 - 0.006)	2.010 (1.254 - 2.890)	2.003 (1.266 - 2.842)	2.006 (1.266 - 2.860)
All ages (rate per capita)	0.009 (0.008 - 0.011)	0.009 (0.007 - 0.010)	0.009 (0.007 - 0.010)	0.001 (0.001 - 0.001)	0.001 (0.000 - 0.001)	0.001 (0.000 - 0.001)	0.262 (0.170 - 0.374)	0.243 (0.158 - 0.350)	0.239 (0.155 - 0.339)
Age-standardized (rate per capita)	0.019 (0.016 - 0.021)	0.019 (0.016 - 0.021)	0.019 (0.016 - 0.021)	0.001 (0.001 - 0.001)	0.001 (0.001 - 0.001)	0.001 (0.001 - 0.001)	0.514 (0.328 - 0.739)	0.513 (0.330 - 0.744)	0.513 (0.330 - 0.735)
Total number of cases (thousands)	60 (51 - 69)	74 (63 - 85)	93 (79 - 107)	4 (3 - 4)	5 (4 - 5)	6 (5 - 7)	2 (1 - 2)	2 (1 - 3)	3 (2 - 4)
**South Sudan**									
15 - 49 years (rate per capita)	0.010 (0.008 - 0.012)	0.010 (0.008 - 0.012)	0.010 (0.008 - 0.012)	0.001 (0.001 - 0.001)	0.001 (0.001 - 0.001)	0.001 (0.001 - 0.001)	0.284 (0.182 - 0.414)	0.279 (0.181 - 0.408)	0.279 (0.180 - 0.409)
50 - 69 years (rate per capita)	0.042 (0.035 - 0.051)	0.043 (0.035 - 0.052)	0.043 (0.035 - 0.052)	0.003 (0.002 - 0.003)	0.003 (0.002 - 0.004)	0.003 (0.002 - 0.003)	1.155 (0.733 - 1.668)	1.184 (0.751 - 1.720)	1.173 (0.743 - 1.719)
70+ years (rate per capita)	0.075 (0.062 - 0.091)	0.075 (0.062 - 0.091)	0.075 (0.063 - 0.091)	0.004 (0.003 - 0.006)	0.004 (0.003 - 0.006)	0.004 (0.003 - 0.006)	1.941 (1.224 - 2.773)	1.950 (1.228 - 2.761)	1.960 (1.244 - 2.802)
All ages (rate per capita)	0.009 (0.008 - 0.011)	0.009 (0.008 - 0.011)	0.010 (0.008 - 0.011)	0.001 (0.000 - 0.001)	0.001 (0.001 - 0.001)	0.001 (0.001 - 0.001)	0.256 (0.166 - 0.364)	0.261 (0.169 - 0.371)	0.271 (0.176 - 0.388)
Age-standardized (rate per capita)	0.019 (0.016 - 0.021)	0.019 (0.016 - 0.021)	0.019 (0.016 - 0.021)	0.001 (0.001 - 0.001)	0.001 (0.001 - 0.001)	0.001 (0.001 - 0.001)	0.504 (0.327 - 0.715)	0.504 (0.328 - 0.717)	0.507 (0.328 - 0.727)
Total number of cases (thousands)	54 (46 - 62)	77 (65 - 88)	120 (102 - 139)	3 (3 - 4)	5 (4 - 6)	8 (6 - 9)	1 (1 - 2)	2 (1 - 3)	3 (2 - 5)
**Tanzania**									
15 - 49 years (rate per capita)	0.010 (0.008 - 0.012)	0.010 (0.008 - 0.012)	0.010 (0.008 - 0.012)	0.001 (0.001 - 0.001)	0.001 (0.001 - 0.001)	0.001 (0.001 - 0.001)	0.277 (0.180 - 0.401)	0.272 (0.176 - 0.397)	0.282 (0.181 - 0.414)
50 - 69 years (rate per capita)	0.043 (0.035 - 0.051)	0.043 (0.035 - 0.051)	0.043 (0.035 - 0.051)	0.003 (0.002 - 0.004)	0.003 (0.002 - 0.004)	0.003 (0.002 - 0.004)	1.185 (0.747 - 1.737)	1.195 (0.757 - 1.736)	1.196 (0.751 - 1.745)
70+ years (rate per capita)	0.075 (0.063 - 0.090)	0.075 (0.062 - 0.090)	0.075 (0.063 - 0.090)	0.004 (0.003 - 0.006)	0.004 (0.003 - 0.006)	0.004 (0.003 - 0.005)	2.010 (1.259 - 2.845)	1.997 (1.250 - 2.858)	2.015 (1.271 - 2.871)
All ages (rate per capita)	0.009 (0.007 - 0.010)	0.009 (0.008 - 0.010)	0.009 (0.008 - 0.010)	0.001 (0.000 - 0.001)	0.001 (0.000 - 0.001)	0.001 (0.000 - 0.001)	0.245 (0.158 - 0.349)	0.248 (0.163 - 0.357)	0.256 (0.165 - 0.366)
Age-standardized (rate per capita)	0.019 (0.016 - 0.021)	0.019 (0.016 - 0.021)	0.019 (0.016 - 0.021)	0.001 (0.001 - 0.001)	0.001 (0.001 - 0.001)	0.001 (0.001 - 0.001)	0.514 (0.331 - 0.735)	0.513 (0.331 - 0.729)	0.516 (0.333 - 0.736)
Total number of cases (thousands)	222 (190 - 256)	347 (297 - 399)	485 (416 - 558)	14 (12 - 16)	22 (19 - 25)	30 (26 - 35)	6 (4 - 9)	10 (6 - 14)	14 (9 - 20)
**Uganda**									
15 - 49 years (rate per capita)	0.010 (0.008 - 0.012)	0.009 (0.007 - 0.011)	0.009 (0.007 - 0.011)	0.001 (0.001 - 0.001)	0.001 (0.000 - 0.001)	0.001 (0.001 - 0.001)	0.270 (0.171 - 0.394)	0.254 (0.162 - 0.373)	0.264 (0.168 - 0.386)
50 - 69 years (rate per capita)	0.043 (0.036 - 0.052)	0.043 (0.035 - 0.052)	0.043 (0.036 - 0.052)	0.003 (0.002 - 0.004)	0.003 (0.002 - 0.004)	0.003 (0.002 - 0.004)	1.197 (0.758 - 1.760)	1.190 (0.750 - 1.730)	1.204 (0.759 - 1.763)
70+ years (rate per capita)	0.075 (0.063 - 0.091)	0.075 (0.063 - 0.090)	0.075 (0.063 - 0.090)	0.004 (0.003 - 0.006)	0.004 (0.003 - 0.006)	0.004 (0.003 - 0.005)	2.004 (1.256 - 2.886)	2.014 (1.270 - 2.910)	2.011 (1.268 - 2.924)
All ages (rate per capita)	0.008 (0.007 - 0.010)	0.008 (0.007 - 0.009)	0.008 (0.007 - 0.009)	0.001 (0.000 - 0.001)	0.000 (0.000 - 0.001)	0.000 (0.000 - 0.001)	0.232 (0.151 - 0.333)	0.215 (0.139 - 0.305)	0.216 (0.140 - 0.309)
Age-standardized (rate per capita)	0.019 (0.016 - 0.021)	0.019 (0.016 - 0.021)	0.019 (0.016 - 0.021)	0.001 (0.001 - 0.001)	0.001 (0.001 - 0.001)	0.001 (0.001 - 0.001)	0.513 (0.331 - 0.734)	0.514 (0.331 - 0.735)	0.516 (0.332 - 0.737)
Total number of cases (thousands)	145 (124 - 168)	216 (185 - 249)	301 (257 - 349)	9 (8 - 11)	14 (12 - 16)	19 (16 - 22)	4 (3 - 6)	6 (4 - 9)	8 (5 - 12)
**Zambia**									
15 - 49 years (rate per capita)	0.010 (0.009 - 0.011)	0.010 (0.009 - 0.010)	0.010 (0.009 - 0.011)	0.001 (0.001 - 0.001)	0.001 (0.001 - 0.001)	0.001 (0.001 - 0.001)	0.287 (0.187 - 0.401)	0.274 (0.181 - 0.385)	0.288 (0.191 - 0.404)
50 - 69 years (rate per capita)	0.041 (0.039 - 0.044)	0.042 (0.039 - 0.045)	0.042 (0.040 - 0.045)	0.003 (0.002 - 0.003)	0.003 (0.002 - 0.003)	0.003 (0.002 - 0.003)	1.151 (0.764 - 1.598)	1.168 (0.783 - 1.607)	1.169 (0.778 - 1.621)
70+ years (rate per capita)	0.072 (0.067 - 0.078)	0.072 (0.067 - 0.078)	0.072 (0.067 - 0.078)	0.004 (0.003 - 0.005)	0.004 (0.003 - 0.005)	0.004 (0.003 - 0.005)	1.943 (1.306 - 2.683)	1.930 (1.291 - 2.684)	1.935 (1.298 - 2.678)
All ages (rate per capita)	0.009 (0.008 - 0.009)	0.008 (0.008 - 0.009)	0.009 (0.008 - 0.009)	0.001 (0.000 - 0.001)	0.001 (0.000 - 0.001)	0.001 (0.000 - 0.001)	0.239 (0.158 - 0.330)	0.234 (0.157 - 0.324)	0.238 (0.158 - 0.328)
Age-standardized (rate per capita)	0.018 (0.018 - 0.019)	0.018 (0.017 - 0.019)	0.018 (0.018 - 0.019)	0.001 (0.001 - 0.001)	0.001 (0.001 - 0.001)	0.001 (0.001 - 0.001)	0.511 (0.338 - 0.702)	0.509 (0.343 - 0.698)	0.508 (0.339 - 0.700)
Total number of cases (thousands)	69 (66 - 73)	101 (96 - 107)	138 (131 - 146)	4 (4 - 4)	6 (6 - 7)	8 (8 - 9)	2 (1 - 3)	3 (2 - 4)	4 (3 - 5)
**Central Sub-Saharan Africa**									
15 - 49 years (rate per capita)	0.028 (0.026 - 0.030)	0.028 (0.026 - 0.030)	0.028 (0.026 - 0.030)	0.002 (0.001 - 0.002)	0.002 (0.001 - 0.002)	0.002 (0.001 - 0.002)	0.796 (0.523 - 1.110)	0.785 (0.518 - 1.109)	0.788 (0.520 - 1.102)
50 - 69 years (rate per capita)	0.100 (0.093 - 0.107)	0.101 (0.094 - 0.108)	0.101 (0.094 - 0.108)	0.006 (0.006 - 0.007)	0.006 (0.006 - 0.007)	0.006 (0.006 - 0.007)	2.711 (1.806 - 3.733)	2.741 (1.825 - 3.783)	2.755 (1.816 - 3.786)
70+ years (rate per capita)	0.165 (0.154 - 0.178)	0.166 (0.155 - 0.179)	0.167 (0.155 - 0.179)	0.008 (0.006 - 0.009)	0.008 (0.006 - 0.009)	0.007 (0.006 - 0.009)	4.277 (2.879 - 5.858)	4.310 (2.910 - 5.891)	4.347 (2.921 - 5.944)
All ages (rate per capita)	0.023 (0.022 - 0.025)	0.023 (0.022 - 0.024)	0.023 (0.022 - 0.024)	0.001 (0.001 - 0.001)	0.001 (0.001 - 0.001)	0.001 (0.001 - 0.001)	0.645 (0.426 - 0.886)	0.628 (0.416 - 0.866)	0.635 (0.419 - 0.878)
Age-standardized (rate per capita)	0.045 (0.043 - 0.048)	0.045 (0.043 - 0.048)	0.045 (0.043 - 0.048)	0.002 (0.002 - 0.003)	0.002 (0.002 - 0.003)	0.002 (0.002 - 0.003)	1.223 (0.814 - 1.678)	1.232 (0.821 - 1.691)	1.241 (0.826 - 1.707)
Total number of cases (thousands)	1,241 (1,177 - 1,313)	1,905 (1,807 - 2,015)	2,626 (2,491 - 2,778)	70 (66 - 74)	107 (101 - 114)	148 (139 - 157)	34 (23 - 47)	53 (35 - 73)	73 (48 - 101)
**Angola**									
15 - 49 years (rate per capita)	0.028 (0.026 - 0.030)	0.027 (0.026 - 0.030)	0.027 (0.026 - 0.029)	0.001 (0.001 - 0.002)	0.002 (0.001 - 0.002)	0.001 (0.001 - 0.002)	0.779 (0.522 - 1.093)	0.779 (0.515 - 1.095)	0.778 (0.516 - 1.084)
50 - 69 years (rate per capita)	0.100 (0.093 - 0.108)	0.100 (0.093 - 0.107)	0.100 (0.093 - 0.107)	0.006 (0.006 - 0.007)	0.006 (0.006 - 0.007)	0.006 (0.006 - 0.007)	2.739 (1.823 - 3.799)	2.722 (1.810 - 3.770)	2.728 (1.806 - 3.788)
70+ years (rate per capita)	0.167 (0.155 - 0.180)	0.167 (0.155 - 0.180)	0.167 (0.155 - 0.180)	0.008 (0.006 - 0.009)	0.008 (0.006 - 0.009)	0.008 (0.006 - 0.009)	4.366 (2.953 - 5.987)	4.384 (2.953 - 6.008)	4.387 (2.948 - 6.003)
All ages (rate per capita)	0.021 (0.020 - 0.023)	0.021 (0.020 - 0.022)	0.021 (0.020 - 0.022)	0.001 (0.001 - 0.001)	0.001 (0.001 - 0.001)	0.001 (0.001 - 0.001)	0.594 (0.395 - 0.819)	0.581 (0.387 - 0.806)	0.584 (0.388 - 0.809)
Age-standardized (rate per capita)	0.045 (0.043 - 0.048)	0.045 (0.043 - 0.048)	0.045 (0.043 - 0.048)	0.002 (0.002 - 0.003)	0.002 (0.002 - 0.003)	0.002 (0.002 - 0.003)	1.234 (0.822 - 1.702)	1.240 (0.825 - 1.707)	1.244 (0.824 - 1.713)
Total number of cases (thousands)	241 (228 - 256)	377 (356 - 400)	529 (500 - 562)	14 (13 - 14)	21 (20 - 23)	30 (28 - 32)	7 (4 - 9)	10 (7 - 15)	15 (10 - 20)
**Central African Republic**									
15 - 49 years (rate per capita)	0.029 (0.027 - 0.031)	0.028 (0.026 - 0.030)	0.028 (0.026 - 0.031)	0.002 (0.001 - 0.002)	0.002 (0.001 - 0.002)	0.002 (0.001 - 0.002)	0.806 (0.536 - 1.133)	0.785 (0.524 - 1.102)	0.804 (0.536 - 1.125)
50 - 69 years (rate per capita)	0.102 (0.094 - 0.109)	0.102 (0.094 - 0.109)	0.101 (0.094 - 0.109)	0.006 (0.006 - 0.007)	0.006 (0.006 - 0.007)	0.006 (0.006 - 0.007)	2.763 (1.842 - 3.819)	2.764 (1.838 - 3.820)	2.758 (1.830 - 3.838)
70+ years (rate per capita)	0.167 (0.156 - 0.180)	0.166 (0.155 - 0.179)	0.166 (0.155 - 0.178)	0.007 (0.006 - 0.009)	0.007 (0.006 - 0.009)	0.007 (0.006 - 0.009)	4.342 (2.926 - 5.926)	4.324 (2.936 - 5.919)	4.320 (2.911 - 5.913)
All ages (rate per capita)	0.027 (0.025 - 0.028)	0.026 (0.025 - 0.028)	0.027 (0.026 - 0.029)	0.001 (0.001 - 0.002)	0.001 (0.001 - 0.002)	0.001 (0.001 - 0.002)	0.735 (0.489 - 1.010)	0.716 (0.476 - 0.987)	0.746 (0.498 - 1.026)

	Prevalence 1990	Prevalence 2005	Prevalence 2015	Incidence 1990	Incidence 2005	Incidence 2015	YLD rate 1990	YLD rate 2005	YLD rate 2015
Age-standardized (rate per capita)	0.045 (0.043 - 0.048)	0.045 (0.043 - 0.048)	0.045 (0.043 - 0.048)	0.002 (0.002 - 0.003)	0.002 (0.002 - 0.003)	0.002 (0.002 - 0.003)	1.233 (0.823 - 1.692)	1.231 (0.823 - 1.688)	1.235 (0.827 - 1.696)
Total number of cases (thousands)	78 (74 - 83)	106 (100 - 112)	132 (125 - 140)	4 (4 - 5)	6 (6 - 6)	7 (7 - 8)	2 (1 - 3)	3 (2 - 4)	4 (2 - 5)
**Congo**									
15 - 49 years (rate per capita)	0.028 (0.026 - 0.029)	0.028 (0.027 - 0.030)	0.029 (0.028 - 0.031)	0.001 (0.001 - 0.002)	0.002 (0.001 - 0.002)	0.002 (0.001 - 0.002)	0.782 (0.519 - 1.089)	0.804 (0.534 - 1.120)	0.825 (0.547 - 1.147)
50 - 69 years (rate per capita)	0.101 (0.096 - 0.107)	0.101 (0.096 - 0.107)	0.101 (0.096 - 0.107)	0.006 (0.006 - 0.007)	0.006 (0.006 - 0.007)	0.006 (0.006 - 0.007)	2.763 (1.850 - 3.799)	2.762 (1.859 - 3.812)	2.772 (1.854 - 3.813)
70+ years (rate per capita)	0.167 (0.157 - 0.177)	0.166 (0.156 - 0.176)	0.166 (0.156 - 0.176)	0.007 (0.006 - 0.009)	0.007 (0.006 - 0.009)	0.007 (0.006 - 0.008)	4.354 (2.957 - 5.925)	4.344 (2.930 - 5.926)	4.358 (2.954 - 5.940)
All ages (rate per capita)	0.025 (0.024 - 0.026)	0.025 (0.024 - 0.027)	0.026 (0.025 - 0.027)	0.001 (0.001 - 0.001)	0.001 (0.001 - 0.001)	0.001 (0.001 - 0.001)	0.691 (0.464 - 0.947)	0.705 (0.474 - 0.970)	0.715 (0.481 - 0.984)
Age-standardized (rate per capita)	0.045 (0.043 - 0.047)	0.045 (0.044 - 0.047)	0.045 (0.043 - 0.047)	0.002 (0.002 - 0.003)	0.002 (0.002 - 0.003)	0.002 (0.002 - 0.003)	1.235 (0.831 - 1.689)	1.238 (0.833 - 1.694)	1.243 (0.840 - 1.704)
Total number of cases (thousands)	60 (58 - 62)	89 (86 - 93)	119 (114 - 124)	3 (3 - 4)	5 (5 - 5)	7 (6 - 7)	2 (1 - 2)	2 (2 - 3)	3 (2 - 5)
**Democratic Republic of the Congo**									
15 - 49 years (rate per capita)	0.028 (0.027 - 0.031)	0.028 (0.026 - 0.030)	0.028 (0.026 - 0.030)	0.002 (0.001 - 0.002)	0.002 (0.001 - 0.002)	0.002 (0.001 - 0.002)	0.800 (0.527 - 1.114)	0.784 (0.517 - 1.102)	0.786 (0.519 - 1.101)
50 - 69 years (rate per capita)	0.099 (0.092 - 0.107)	0.101 (0.094 - 0.108)	0.101 (0.094 - 0.109)	0.006 (0.006 - 0.007)	0.006 (0.006 - 0.007)	0.006 (0.006 - 0.007)	2.688 (1.792 - 3.710)	2.739 (1.830 - 3.789)	2.760 (1.817 - 3.807)
70+ years (rate per capita)	0.164 (0.153 - 0.178)	0.166 (0.154 - 0.179)	0.167 (0.155 - 0.180)	0.007 (0.006 - 0.009)	0.008 (0.006 - 0.009)	0.008 (0.006 - 0.009)	4.233 (2.849 - 5.754)	4.286 (2.890 - 5.863)	4.339 (2.924 - 5.957)
All ages (rate per capita)	0.023 (0.022 - 0.025)	0.023 (0.021 - 0.024)	0.023 (0.022 - 0.024)	0.001 (0.001 - 0.001)	0.001 (0.001 - 0.001)	0.001 (0.001 - 0.001)	0.643 (0.425 - 0.883)	0.625 (0.416 - 0.863)	0.634 (0.421 - 0.876)
Age-standardized (rate per capita)	0.045 (0.043 - 0.047)	0.045 (0.043 - 0.048)	0.045 (0.043 - 0.048)	0.002 (0.002 - 0.003)	0.002 (0.002 - 0.003)	0.002 (0.002 - 0.003)	1.217 (0.810 - 1.672)	1.229 (0.819 - 1.688)	1.240 (0.825 - 1.703)
Total number of cases (thousands)	821 (778 - 870)	1,275 (1,208 - 1,352)	1,769 (1,676 - 1,875)	46 (43 - 49)	72 (68 - 76)	100 (94 - 106)	23 (15 - 31)	35 (23 - 49)	49 (33 - 68)
**Equatorial Guinea**									
15 - 49 years (rate per capita)	0.029 (0.027 - 0.032)	0.032 (0.029 - 0.034)	0.030 (0.028 - 0.032)	0.002 (0.001 - 0.002)	0.002 (0.001 - 0.002)	0.002 (0.001 - 0.002)	0.835 (0.558 - 1.172)	0.898 (0.595 - 1.259)	0.850 (0.559 - 1.196)
50 - 69 years (rate per capita)	0.105 (0.098 - 0.113)	0.101 (0.094 - 0.109)	0.099 (0.092 - 0.106)	0.007 (0.006 - 0.007)	0.006 (0.006 - 0.007)	0.006 (0.005 - 0.007)	2.871 (1.915 - 3.998)	2.775 (1.841 - 3.847)	2.722 (1.797 - 3.767)
70+ years (rate per capita)	0.167 (0.155 - 0.181)	0.169 (0.157 - 0.182)	0.171 (0.159 - 0.184)	0.008 (0.006 - 0.009)	0.007 (0.006 - 0.009)	0.007 (0.006 - 0.009)	4.372 (2.939 - 6.044)	4.402 (2.965 - 6.040)	4.462 (3.026 - 6.061)
All ages (rate per capita)	0.029 (0.027 - 0.031)	0.027 (0.025 - 0.029)	0.029 (0.027 - 0.030)	0.002 (0.002 - 0.002)	0.001 (0.001 - 0.002)	0.002 (0.002 - 0.002)	0.801 (0.534 - 1.101)	0.751 (0.496 - 1.038)	0.799 (0.525 - 1.102)
Age-standardized (rate per capita)	0.046 (0.043 - 0.048)	0.046 (0.044 - 0.049)	0.047 (0.044 - 0.049)	0.002 (0.002 - 0.003)	0.002 (0.002 - 0.003)	0.002 (0.002 - 0.003)	1.252 (0.838 - 1.726)	1.269 (0.845 - 1.752)	1.277 (0.848 - 1.758)
Total number of cases (thousands)	11 (10 - 12)	17 (16 - 18)	24 (23 - 26)	1 (1 - 1)	1 (1 - 1)	1 (1 - 1)	0 (0 - 0)	0 (0 - 1)	1 (0 - 1)
**Gabon**									
15 - 49 years (rate per capita)	0.029 (0.027 - 0.031)	0.028 (0.027 - 0.031)	0.030 (0.028 - 0.032)	0.002 (0.001 - 0.002)	0.002 (0.001 - 0.002)	0.002 (0.001 - 0.002)	0.818 (0.547 - 1.148)	0.804 (0.536 - 1.128)	0.839 (0.560 - 1.178)
50 - 69 years (rate per capita)	0.102 (0.095 - 0.110)	0.104 (0.097 - 0.112)	0.104 (0.096 - 0.111)	0.006 (0.006 - 0.007)	0.007 (0.006 - 0.007)	0.007 (0.006 - 0.007)	2.788 (1.857 - 3.884)	2.838 (1.880 - 3.931)	2.823 (1.892 - 3.932)
70+ years (rate per capita)	0.166 (0.154 - 0.178)	0.165 (0.153 - 0.177)	0.165 (0.153 - 0.177)	0.007 (0.006 - 0.009)	0.007 (0.006 - 0.008)	0.007 (0.006 - 0.008)	4.325 (2.943 - 5.958)	4.288 (2.910 - 5.854)	4.291 (2.915 - 5.905)
All ages (rate per capita)	0.031 (0.029 - 0.033)	0.030 (0.028 - 0.031)	0.030 (0.029 - 0.032)	0.002 (0.002 - 0.002)	0.002 (0.002 - 0.002)	0.002 (0.002 - 0.002)	0.851 (0.567 - 1.178)	0.816 (0.544 - 1.121)	0.836 (0.562 - 1.155)
Age-standardized (rate per capita)	0.045 (0.043 - 0.048)	0.045 (0.043 - 0.048)	0.046 (0.043 - 0.048)	0.002 (0.002 - 0.003)	0.002 (0.002 - 0.003)	0.002 (0.002 - 0.003)	1.233 (0.824 - 1.698)	1.241 (0.829 - 1.707)	1.251 (0.839 - 1.724)
Total number of cases (thousands)	30 (28 - 31)	41 (39 - 43)	52 (50 - 55)	2 (2 - 2)	2 (2 - 2)	3 (3 - 3)	1 (1 - 1)	1 (1 - 2)	1 (1 - 2)
